# Diel movement of brown trout, *Salmo trutta*, is reduced in dense populations with high site fidelity

**DOI:** 10.1002/ece3.3981

**Published:** 2018-04-06

**Authors:** Ondřej Slavík, Pavel Horký, Matúš Maciak, Petra Horká, Iva Langrová

**Affiliations:** ^1^ Department of Zoology and Fisheries Faculty of Agrobiology, Food and Natural Resources Czech University of Life Sciences Prague Prague 6 Czech Republic; ^2^ Department of Probability and Mathematical Statistics Faculty of Mathematics and Physics Charles University Prague 8 Czech Republic; ^3^ Institute for Environmental Studies Faculty of Science Charles University Prague 2 Czech Republic

**Keywords:** density‐dependent process, diel cycle, movement, radio‐telemetry, subpopulation site fidelity

## Abstract

The movement of individuals within preferred areas is reduced by a high availability of food and information about its distribution, while high number of competitors promotes increased movement. Experienced animals use information about social and physical environment to improve resources exploitation, tended to maintain positions within the preferred areas and reuse the environment that is often referred to as site fidelity. In this study, radio‐telemetry was used to observe the movements of 98 adult brown trout, *Salmo trutta*, in oligotrophic streams with different population densities; to determine subpopulation site fidelity, 5,195 conspecifics from 14 subpopulations were individually tagged during spring and autumn. During a 7‐year‐long field study, we tested the hypothesis that brown trout individuals from subpopulations with high site fidelity would display lower movement. The hypothesis was supported, and reduced movement was further related to high subpopulation density in association with high slope indicating the physical environment‐influenced movement. The probability of contact between individuals increased with subpopulation site fidelity and subpopulation density. No influence of food abundance on brown trout movement was found. Furthermore, increased body size predicted higher movement (and vice versa). The least movement occurred during the day and during the full moons. Our study tended to show that individuals reused preferred areas and needed less movement to exploit available resources.

## 1 INTRODUCTION

In response to the presence of conspecifics and the spatial distribution of resources, animals tend to avoid areas with a high number of competitors (Kuefler, Avgar, & Fryxell, 2013; Leibold, 1995) and spend more time in patches with high‐quality forage (Benhamou, 2007; Fretwell & Lucas, 1970) with the aim of increasing energy intake. Poor competitors either emigrate from highly contested areas to areas with a lower abundance of conspecifics (Elliott, 1994; Grant & Kramer, 1990; Matthysen, 2005) and/or move around the peripheries of contested areas (Hansen & Closs, 2005; Nakano, 1995) and more often change their positions (Beisner & Isbell, 2009). The decision of animals to maintain positions within preferred areas results in less movement, while low food availability induces faster movement (Klaassen, Nolet, & Bankert, 2006). The movement of individuals is further affected by the available information about variably dispersed resources (Dall, Giraldeau, Olsson, McNamara, & Stephens, 2005; Harwood, Griffiths, Metcalfe, & Armstrong, 2003; Klaassen, Nolet, & van Leeuwen, 2007; Morales, Moorcroft, Matthiopoulos, Merrill, & Haydon, 2010). Experienced individuals ignore areas with low food availability and move toward food‐rich areas, and less‐experienced individuals are limited by the continuous search for resources. Hence, the information available about resources can be a predictor of movement distances (Dias, Granadeiro, & Palmeirim, 2009; Klaassen, Nolet, van Gils, & Bauer, 2006). Moreover, information about resources motivates individuals to return and reuse preferred areas, which is often referred to as site fidelity (Switzer, 1993, 1997; Ward, James, Wilson, & Webster, 2013). Site fidelity corresponds to improved resource exploitation and greater fitness as reported, for example, in amphibians (Bucciarelli, Green, Shaffer, Bradley, & Kats, 2016), birds (Lourenco et al., 2016; Piotr, 2016), and mammals (Forrester, Cassady, & Wittmer, 2015; Geinapp & Merilä, 2011; McIntire, Bester, Bornemann, Tosh, & Nico de Bruyn, 2017). However, high site fidelity is also apparently associated with the occurrence of animals at preferred, food‐rich areas that are host dense populations (Edwards, Nagy, & Derocher, 2009). Taken together, these trends appeared to impact the behavior of animals in the preferred areas, where their movement is reduced by the high availability of food and information about its dispersal, while, in contrast, the high number of competitors promotes increased movement.

Density‐dependent changes in behavioral patterns are well documented from experimental observations of salmonids. Juvenile Atlantic salmon (*Salmo salar* Linnaeus, 1758) were found to emigrate from familiar environments with high population densities to new environments with lower densities of conspecifics (Steingrímsson & Grant, 2003), but the effect of competition on movement and position maintenance was inconclusive compared to the effects of environmental parameters. Similarly, juvenile Arctic charr (*Salvelinus alpinus* Linnaeus, 1758) showed increased diel activity with increasing abundance as a result of competition for shelters (Larranaga & Steingrímsson, 2015) and food (Fingerle, Larranga, & Steingrímsson, 2016). Furthermore, high population density and decreased food availability promoted increased feeding activity, which was followed by lower growth in Arctic charr (Guénard et al., 2012). The opposite response of faster growth was found in dense brown trout (*Salmo trutta* Linnaeus, 1758) subpopulations with high subpopulation site fidelity (Závorka, Horký, Höjesjö, & Slavík, 2016), but because the available data were collected by different methods and from different species, it is difficult to conclude whether site fidelity in salmonids is associated with behavioral changes, for example, with changes in local movements at preferred areas. Based on the assumption that preferred areas are associated with high food availability and population density, we tested the hypothesis that brown trout, *S. trutta*, individuals would move less in areas with high subpopulation site fidelity.

To test this hypothesis, we observed the diel movements of brown trout in variably abundant subpopulations in the Šumava Mountains (Czech Republic, Central Europe) by radio‐telemetry and determined the spatial distribution of the observed individuals. We performed observations throughout the diel cycle as the movement of brown trout varies over 24 hr, and movement peaks have been reported during the day (Höjesjö, Økland, Sundström, Pettersson, & Johnsson, 2007) or night (Young, 1999). We tagged individuals from several size classes in each studied subpopulation, because the size of the areas used by brown trout has been shown to increase with body size (Gunnarsson & Steingrímsson, 2011; Nicola, Ayllón, Elvira, & Almódovar, 2016). Abundance of individuals and subpopulation site fidelity were monitored over a 7‐year period. Subpopulation site fidelity was defined as the return and long‐term persistence of individuals at home sites (Bond et al., 2012; Knope, Tice, & Rypkema, 2017; Marnane, 2000; Steingrímsson & Grant, 2003; White & Garrott, 1990), and it has previously been reported in brown trout (Bridcut & Giller, 1993). In our study, the movement, density, and subpopulation site fidelity of brown trout were described in environments that were characterized in terms of food abundance and by typical parameters such as temperature, water velocity, height of the water column, flow, substrata size, and river slope (e.g., Heggenes, Krog, Lindås, Dokk, & Bremnes, 1993; Höjesjö, Johnsson, & Bohlin, 2004; Larranaga & Steingrímsson, 2015; Steingrímsson & Grant, 2003). In further, we observed light intensity and lunar phase during the study as the movement patterns are being affected by light intensity (Imre & Boisclair, 2005; Metcalfe, Valdimarsson, & Fraser, 1997). In our study, data about brown trout movement in subpopulations with variable density and site fidelity were obtained and further related to food abundancy and physical parameters of environment.

## 2 MATERIALS AND METHODS

### 2.1 Study area

**Figure 1:**
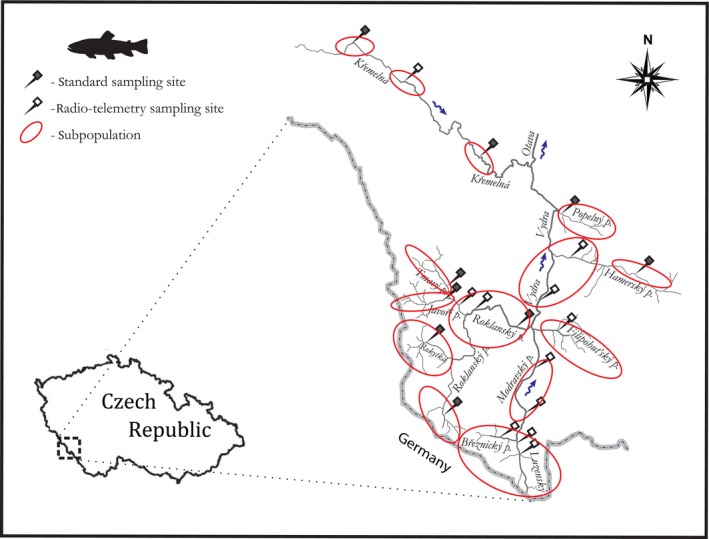
Map of sampling sites in the headwaters of the Otava River, located in the Šumava National Park, Czech Republic. Subpopulations are outlined according to Závorka et al. (2013)

The study was carried out in the headwaters of the Otava River, located in Šumava National Park, Czech Republic. The overall catchment area of the studied streams, including the two main tributaries of the Otava River, Vydra and Křemelná, is approximately 224 square km (Figure 1). The Otava River has an average discharge of 8 m^3^/s (range during the study period 1.69–104 m^3^/s), and its headwaters consist of mountainous oligotrophic streams with pristine morphologies. Twenty representative sampling sites distributed across the entire studied catchment area were selected based on maps, field visits, and knowledge/restrictions of the national park management to maximize the representativeness of the stream characteristics; however, final site selection was also influenced by the access granted by the national park. All sampling sites were wadable with substrate dominated by pebble and gravel; the average conductivity was 20.36 μS/cm (range 8.5–40 μS/cm), and the average flow varied from 0.01 to 2 m^3^/s along the longitudinal gradient (Figure 1; see Závorka, Horký, & Slavík, 2013 for detailed descriptions). Sampling sites were pooled into fourteen synchronized population units for further analyses according to a previous study by Závorka et al. (2013) that analyzed the synchrony in population abundance within the studied catchment area through the correlation of seasonal growth rates and abundance between pairs of sampling sites. These synchronized units were labeled “subpopulations” for this study. Overall, fisheries management, including stocking and fishing activities, is restricted in the studied streams, so the local populations consist of only autochthonous brown trout and bullhead (*Cottus gobio* Linnaeus, 1758).

### 2.2 Brown trout population data

All twenty sites were sampled twice a year (May and October) over the 7‐year period from October 2005 to October 2011 (Závorka et al., 2016). Every specimen caught via electrofishing (FEG 1500; EFKO‐Elektrofischfanggeräte GmbH, Germany) was measured (standard length *L*_S_, mm), weighed (body size, g), and individually tagged on the lower left jaw using VIA tags (Visible Implant Alphanumeric tags; Northwest Marine Technology, USA). Specimens of insufficient size for individual tagging were marked using VIE tags (Visible Implant Elastomer tags; Northwest Marine Technology). Throughout the study, 5,195 brown trout were tagged (5,013 using VIA and 182 using VIE tags, allowing for individual site identification) and released at the site of their capture. Regarding the welfare of the study animals, a single‐pass electrofishing method was used, which is considered sufficient for determining the abundance of brown trout in mountain headwater streams (Kruse, Hubert, & Rahel, 1998). The location and size of the sampling sites, as well as fishing effort, were constant throughout the study. The pooling of the sampling sites resulted in an average subpopulation unit area of 546 m^2^ (Závorka et al., 2016). The detection of previously tagged fishes was recorded as a recapture (overall recapture rate was 9%; Závorka et al., 2013). No statistically significant differences were detected between recapture rate and environmental (e.g., slope, stream width) as well as sampling (e.g., sampling area) characteristics (Závorka et al., 2016).

### 2.3 Brown trout behavioral data

The fish used for radio‐telemetry tag implantation were caught via electrofishing (650 V, 4 A, pulsed D.C.) from eleven sampling sites that were identical to those from which the population data were obtained (Figure 1). Altogether, 130 individual brown trout were radio‐tagged from 2006 to 2011 (Table 1). The fish were anaesthetized with 2‐phenoxy‐ethanol (0.2 ml/L), measured (*L*_S_, mm), and weighed (g). The types of transmitters available from the manufacturer (Lotek Engineering Inc., Canada) differed over the duration of our study. Thus, six types of coded radio transmitters (NTQ‐1, NTQ‐2, NTC‐3‐2, NTC‐M‐2, NTC‐M‐3, and MCFT‐3GM; all at a frequency of 138.300 MHz), with mean operational life of 33 days (range 21–43 days), an average weight of 0.74 g in the air (range of 0.26–1.8 g), an average width of 6.36 mm (range of 5–8.2 mm), and an average length of 14.5 mm (range of 10–19 mm), were used throughout the study. Radio transmitters were implanted into the body cavities of brown trout through a midventral incision that was closed with three separate stitches using sterile, braided, absorbable sutures (Ethicon Coated Vicryl). The mass of the transmitter never exceeded 2% of the body mass of the fish (Winter, 1983). The fish were released at or near the point of capture after they recovered and exhibited spontaneous swimming activity (ca. 5 min after surgery).

**Table 1:** Number (number of tagged specimens used in the analysis in parentheses) and characteristics of tagged specimens (standard length and weight ranges; means in parentheses) and study periods

Stream name	Number of tagged specimens	*L*_S_ (mm)	Weight (g)	Study period
Březnický Brook	3	113–188 (145)	19–88 (47)	2008
Filipohuťský Brook	10	99–192 (148)	13–101 (52)	2010
Hamerský Brook	15 (8)	165–218 (182)	62–136 (80)	2006
Javoří Brook	20 (15)	98–198 (129)	14–88 (33)	2009
Křemelná	15 (7)	138–195 (163)	47–123 (77)	2011
Luzenský Brook 1	2	140–203 (172)	38–115 (77)	2008
Luzenský Brook 2	10 (6)	108–276 (144)	20–254 (62)	2008
Modravský Brook 1	12 (11)	132–217 (166)	30–119 (60)	2011
Modravský Brook 2	15	96–232 (152)	13–171 (58)	2010
Roklanský Brook	15 (10)	181–260 (221)	83–240 (168)	2007
Vydra	13 (11)	117–238 (178)	21–199 (90)	2011

At all study sites, tracking series were carried out weekly until the end of transmitter battery life. Tracking predominantly occurred in June (87% of data) with some observations made in late May. Once all the fish in a particular tracking series were positioned, most (depending on the tracking conditions) were observed over two subsequent 24‐hr tracking cycles, resulting in 5,520 individual fish positions. In a 24‐hr cycle, fish positions were determined once during each 3‐hr period (06:00–08:59, 09:00–11:59, 12:00–14:59, 15:00–17:59, 18:00–20:59, 21:00–23:59, 24:00–02:59, 03:00–05:59 hr). The time between measurements varied slightly depending on the tracking conditions (3 hr ± 30 min). The fish were positioned with the help of a GPS unit (GPS map 76S; Garmin Ltd., USA) using a radio receiver (Lotek SRX_400 receiver firmware version W31) and a three‐element Yagi antenna equipped with a compass. Compass bearings were taken on the transmitter direction from locations positioned with the help of a GPS unit. A computer program was developed to obtain fish position coordinates and plot them on a map using the biangulation method proposed by White and Garrott (1990). The accuracy of fish positioning across sampling sites was estimated to be ±1 m based on calibrations using a tag located at the river bottom, the position of which an observer did not know.

### 2.4 Habitat measurements

Habitat variables were measured using the following devices: water temperature (°C) and conductivity (μS/cm; WTW, pH/Cond 340i SET); light intensity (Ev; SECONIC Super Zoom Master L—68 SECONIC, Tokyo, Japan); and lunar phase (BAR 928 H Remote Weather Station, Huger Electronics, Germany). Throughout the study, all the above‐mentioned variables were measured once every 3 hr on the days when fish were tracked. River slopes (%) were measured using a Pulse Total Station (Topcon GPT 2000; Itabashi, Tokyo, Japan) at each sampling site (the average sampling site length was 266 m; see Závorka et al., 2016 for details). The river slope was calculated as the difference between the water levels in two adjacent stream cross sections (Boiten, 2000). The river substratum was determined by assessing the proportions of sand, gravel, pebbles, and boulders according to Wolman (1954).

### 2.5 Food availability

To assess food availability, aquatic invertebrates were quantitatively collected from the sampling sites during autumn 2006 before the fish sampling occurred. Four replicate Surber samples were taken to estimate the densities of benthic invertebrates (350‐μm mesh size; 33 × 33‐cm quadrat size; Surber, 1936). The river bottom was sampled randomly with a focus on habitats with high expected invertebrate abundance, such as pools and flows (Resh, 1979). Samples were preserved immediately after collection and stored in 80% ethanol, and the collected invertebrates were identified to genus or species and enumerated. Finally, the overall abundance and the abundance of particular orders were determined.

### 2.6 Data analyses

The number of trout from the actual year (spring and autumn samples were counted together), when the tracking series were conducted, and the area used for calculating each “subpopulation density” were obtained by pooling all the trout and the areas, respectively, of the sampling sites integrated at a given spatial scale (Imre, Grant, & Cunjak, 2005). Accordingly, river “slope” was calculated as an average value for the sampling sites integrated at the subpopulation scale. According to White and Garrott (1990), site fidelity was defined as the tendency to remain in an area over an extended period or to return to a previously occupied area. In our study, the area for which site fidelity was assessed included all areas of the sampling sites integrated at the subpopulation scale, and “subpopulation site fidelity” was calculated as the percentage of recaptured individuals (i.e., those displaying site fidelity) relative to the total number of tagged individuals within a subpopulation during the entire study. In other words, subpopulation site fidelity is an assemblage measure based on 7 years of data and represents the proportion of all individuals recaptured within a subpopulation, suggesting that these individuals either remained in an area over an extended period or returned to it (White & Garrott, 1990); individuals who disappeared via emigration or mortality were not distinguished (Steingrímsson & Grant, 2003). The “subpopulation site fidelity ratio” was defined according to Závorka et al. (2016) using the PROC Rank procedure to split the subpopulation site fidelity values into two distinct groups. Thus, subpopulations with site fidelity values lower than 7% were classified as “low site fidelity” sites, and subpopulations with values higher than 7% were classified as “high site fidelity” sites. Radio‐telemetry data from 98 brown trout were included in our statistical analyses. Otters caught fourteen individuals, and eighteen individuals moved to inaccessible locations outside of our study sites. These fish were excluded from further analyses. The distance (m) between fish positions at two subsequent 3‐hr intervals during a 24‐hr cycle is henceforth referred to as “movement.” Three “light intervals” (day, twilight, and night) were used to describe diel activity patterns. These intervals were determined based on illumination (Ev), according to Slavík, Horký, Bartoš, Kolářová, and Randák (2007), that is, twilight ranged from between 2 and 6 Ev; day was defined as higher than 6 Ev; and night was defined as lower than 2 Ev. Based on the fish positions, the “distance” between two tagged individuals was obtained. One‐to‐one analyses were performed for all combinations of tracked individuals, thus, obtaining pairs of individuals over particular 24‐hr tracking cycles. The “probability of contact” between two individuals was determined assuming a fish position accuracy of ±1 m. When the distance was less than 2 m, the probability of contact was considered to be “1”; otherwise, the probability of contact was considered to be “0.” For every pair of individuals, “weight difference” and “length difference” values were calculated to assess their “size similarity.” Data analysed during the study are included in the Tables S1 ‐ S4.

### 2.7 Statistical analyses

Statistical analyses of movement as the dependent variable were performed using a linear mixed model (LMM) with random factors (PROC MIXED) in the SAS software package (SAS Institute Inc., version 9.4, http://www.sas.com). The data (movement, river slope, subpopulation site fidelity, and subpopulation density) were log_10_ transformed prior to the LMM analyses when necessary to improve their fit to the normal distribution of the model (Thode, 2002). The random factors (intercept with fish ID as a subject) were used to account for the repeated measures collected for the same subjects (individual fish) over the duration of the experiment assuming complete independence across subjects. The significance of each explanatory variable (subpopulation density, body size, light interval, moon phase, slope, subpopulation site fidelity, subpopulation site fidelity ratio, water temperature, conductivity, substratum) was assessed using *F* tests to sequentially drop the least significant effect, beginning with the full model containing all variables and their possible two‐way interactions (backward selection procedure). Fixed effects and their interactions that were not statistically significant are not discussed further. The differences between classification variables defined in the CLASS statement were tested with t tests (posthoc analysis following a significant main effect), and a Tukey–Kramer adjustment was used for multiple comparisons. The degrees of freedom were calculated using the Kenward–Roger method (Kenward & Roger, 1997). The relative importance of variables in the final model was assessed according to an Akaike's Information Criterion (AIC) value (Burnham & Anderson, 1998). Separate models without a variable were fitted, considering the ∆ AIC comparing their fit with the final model as a sign of the variable importance, that is, ∆ AIC showed how the exclusion of a variable influenced the model fit. Variable with the highest ∆ AIC was suggested as the most important as its exclusion reduced the fit of the model to the greatest extent.

A GENMOD procedure with binomial distributions was designed to estimate the probability of contact between two tracked brown trout conspecifics (i.e., probability of contact equal to 1) in relation to length difference, subpopulation site fidelity, light interval, moon phase, weight difference, slope, subpopulation density, subpopulation site fidelity ratio, water temperature, conductivity, and their possible two‐way interactions. We applied an analysis of repeated measurements based on the generalized estimating equation (GEE) approach (Liang & Zeger, 1986), which is an extension of a generalized linear model and provides a semi‐parametric approach to longitudinal data analysis. To account for the repeated measures collected for the same experimental units (fish pair ID) throughout the duration of the experiment, we used a REPEATED statement with fish pair ID defined as a subject.

Statistical analyses of subpopulation site fidelity ratio as the dependent variable were also subjected to a chi‐squared test using the GEE approach (Liang & Zeger, 1986) and the GENMOD procedure with binomial distributions. In this context, the GENMOD procedure was applied to predict the subpopulation site fidelity ratio in response to the aquatic invertebrate and habitat structure variables.

## 3 RESULTS

Brown trout behavior was influenced by subpopulation site fidelity as well as by the physical environment and individual characteristics. The movement of brown trout across all localities varied from 0 to 949 m (mean 25 m), while distances varied from 0 to 1,509 m (mean 221 m). Brown trout displayed contact in 4% of all pairwise observations. Among the aquatic invertebrates used for the food availability analyses, individuals belonging to the orders *Diptera*,* Plecoptera*, and *Trichoptera* were found most frequently with an overall abundance from 15.25 to 139.75 ind/m^2^ (mean 53.15 ind/m^2^; Table 2).

**Table 2:** Aquatic invertebrate assemblage abundance values

Variable	Mean (ind/m^2^)	Range (ind/m^2^)	χ^2^	*df*	*p*
Overall abundance	53.15	15.25–139.75	0.86	1	<.35
*Diptera*	13.23	0.25–5.75	0.13	1	<.72
*Plecoptera*	12.5	1.25–43.5	0.95	1	<.33
*Trichoptera*	10.66	3–34.25	0.01	1	<.92
*Ephemeroptera*	7.54	0–35.25	0.22	1	<.64
*Crustacea*	4.89	0–45.25	0.4	1	<.52
*Coleoptera*	4.33	0–22	3.35	1	<.07

The results from the GENMOD procedure, which was applied to predict the subpopulation site fidelity ratio in response to the aquatic invertebrate abundance, are given as chi‐squared with corresponding *p*‐values.

**Figure 2:**
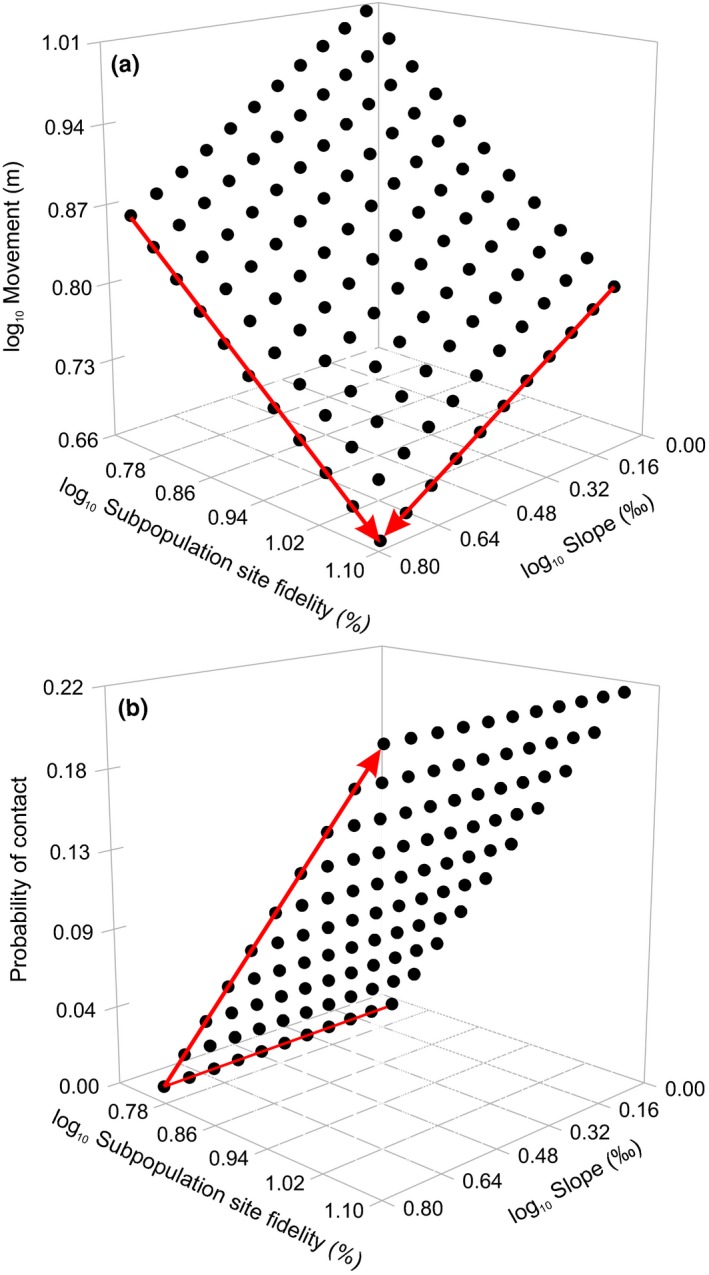
The relationship between brown trout movement (a) and probability of contact (b), plotted against the subpopulation site fidelity and slope. Predicted values of brown trout movement (a) are from log_10_‐transformed data

**Figure 3:**
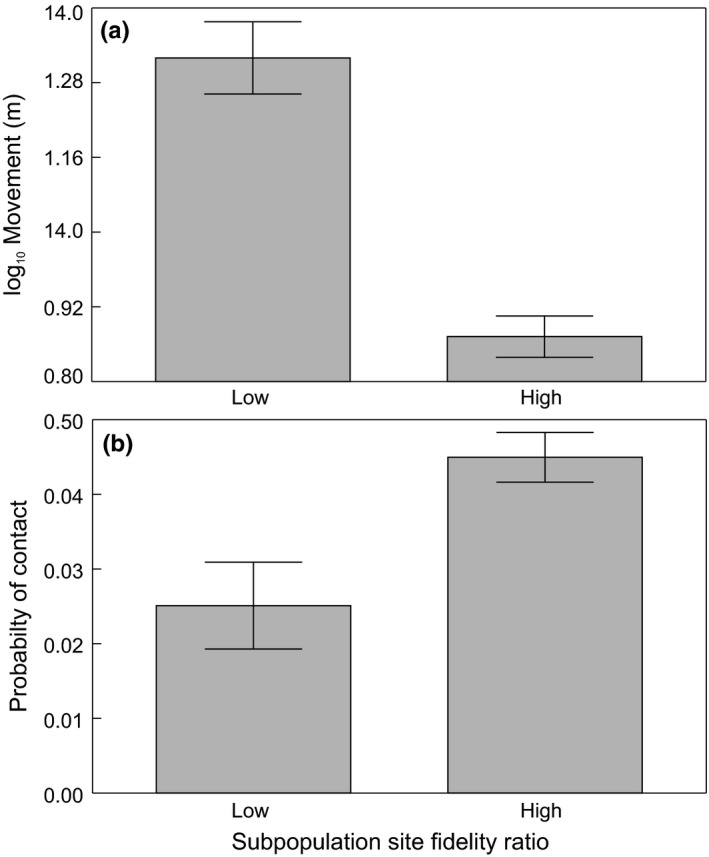
Brown trout movement (a) and probability of contact (b) across different subpopulation site fidelity ratios. Adjusted means ± *SE* of brown trout movement (A) are from log_10_‐transformed data

**Figure 4:**
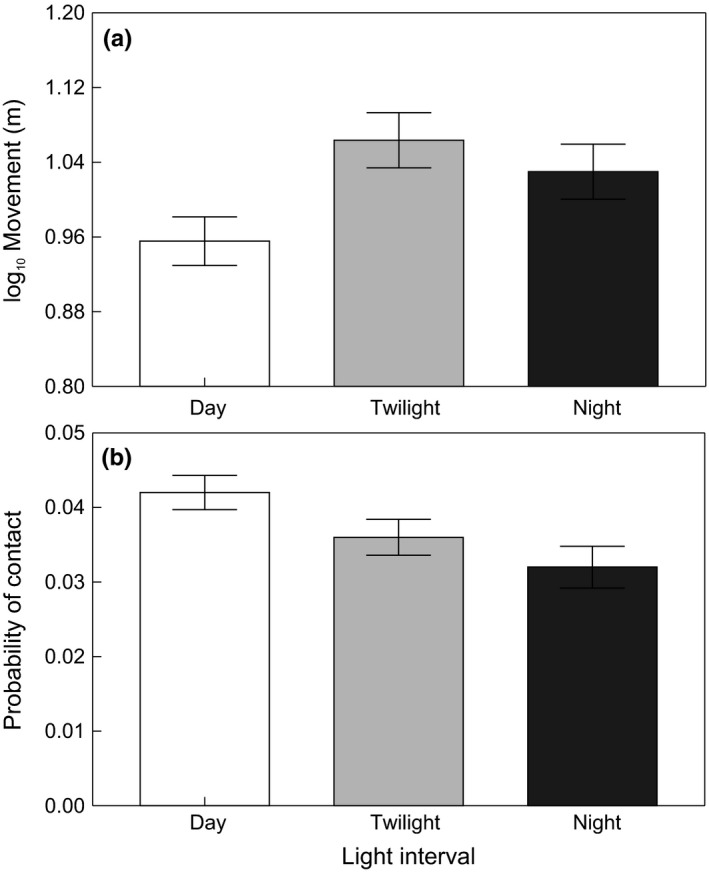
Brown trout movement (a) and probability of contact (b) across different light intervals (day, night twilight). Adjusted means ± *SE* of brown trout movement (a) are from log_10_‐transformed data

**Figure 5:**
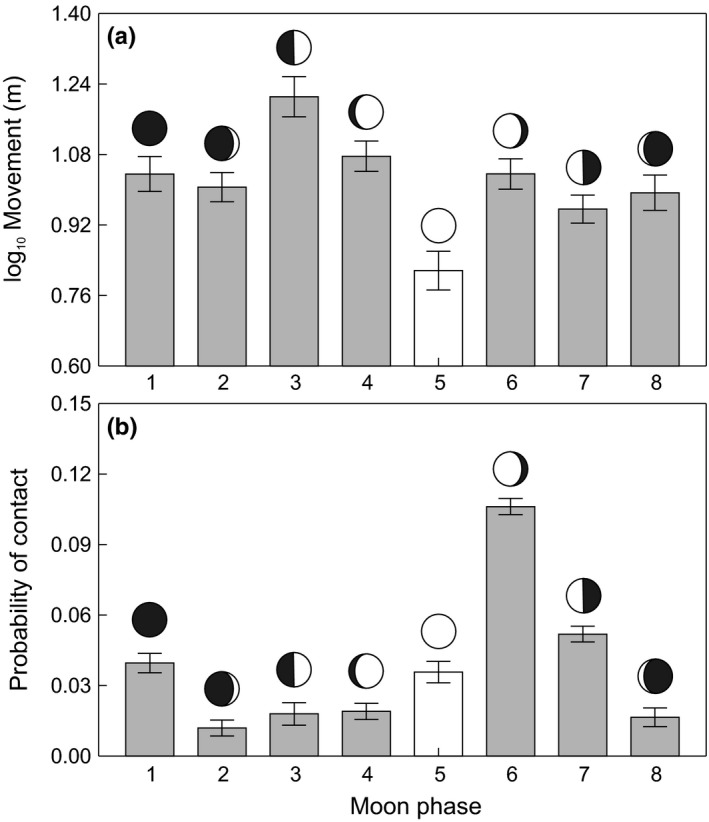
Movement (a) and probability of contact (b) across eight different moon phases. Number five represents the full moon. Adjusted means ± *SE* of brown trout movement (a) are from log_10_‐transformed data

Brown trout movement decreased with increasing subpopulation density (*F*_1,117_ = 31.88, *p* < .0001). There was also an interaction between subpopulation site fidelity and river slope (*F*_1,170_ = 44.47, *p* < .0001), indicating brown trout movement decreased when slope steepened and site fidelity increased (Figure 2a). Accordingly, brown trout moved shorter distances in subpopulations with a high site fidelity ratio (Figure 3a; *F*_1,140_ = 31.88, *p* < .0001). All variables in the final model improved its fit (range of the ∆ AIC from 19.5 for the model with excluded light interval to 52.9 for the model with excluded moon phase). Models with excluded variables describing site fidelity had the second and third highest ∆ AIC values (∆ AIC 40 for the model with excluded interaction of river slope and subpopulation site fidelity and ∆ AIC 27.7 for the model with excluded subpopulation site fidelity ratio), suggesting the overall importance of site fidelity in brown trout movement explanation. Nevertheless, the probability that a subpopulation had a high site fidelity ratio was not influenced by the aquatic insect assemblage (Table 2) or habitat structure (Table 3). Brown trout were less active when light levels were high; their movements were lowest during the day (Figure 4a; *F*_2,5425_ = 11.77, *p* < .0001; Adj. *p *<* *.05) and during the full moon (Figure 5a; *F*_7,5419_ = 9.70, *p* < .0001; Adj. *p *<* *.05). Movement increased with fish body size (*F*_1,175_ = 22.82, *p* < .0001).

**Table 3:** Slope and substrate values

Variable	Mean (%)	Range (%)	χ^2^	*df*	*p*
Slope	4.42	0.68–35	0.93	1	<.34
Sand	2.11	0–7	0.16	1	<.69
Gravel	39.77	5–83	0.6	1	<.44
Pebble	42.7	11–64	0.41	1	<.52
Boulder	15.38	1–51	0.15	1	<.70

The results from the GENMOD procedure, which was applied to predict the subpopulation site fidelity ratio in response to the habitat variables, are given as chi‐squared with corresponding *p*‐values.

The probability of contact increased as the size between two individuals became more similar (χ^2^ = 12.39, *df* = 1; *p* < .0004). The probability of contact further increased with increasing subpopulation density (χ^2^ = 24.8, *df* = 1; *p* < .0001) and site fidelity (χ^2^ = 25.34, *df* = 1; *p* < .0001). The interaction between subpopulation site fidelity and river slope (χ^2^ = 17.56, *df* = 1; *p* < .0001) confirmed that the probability of contact increased with subpopulation site fidelity, while the influence of river slope was not possible to determine (Figure 2b). The probability of contact also increased in subpopulations with a high site fidelity ratio (Figure 3b; χ^2^ = 29.36, *df* = 1; *p* < .0001). The probability of contact was the highest during the day (Figure 4b; χ^2^ = 12.55, *df* = 2; *p* < .0019) and during the waning moon, that is, the first moon phase after the full moon (Figure 5b; χ^2^ = 70.54, *df* = 7; *p* < .0001).

## 4 DISCUSSION

### 4.1 Movement and site fidelity and slope

This field study revealed reduced movement in brown trout subpopulations showing high site fidelity. The findings can be interpreted as the capability of the brown trout to effectively use available resources within the preferred, reused areas.

It is generally known that animals use information to decide whether to return to, reuse or avoid specific areas (Morales et al., 2010), and the available information about resources corresponds to not only the local movement of individuals within an area but also site fidelity (McIntire et al., 2017; Switzer, 1997; Wittmer, McLellan, & Hovey, 2006). Comparable data about brown trout movement and subpopulation site fidelity are not available, but our findings are indirectly supported by some previous studies. For example, newcomer coral fish (*Stegastes diencaeus* Jordan and Rutter, 1897) showed higher movement and lower food intake compared with residents, and they experienced more attacks, indicating that relocating to a new territory induces higher energy costs, including those associated with stress, as well as decreased energy intake (McDougall & Kramer, 2007). Similarly, prior residency reduced the movement of catfish (*Silurus glanis* Linnaeus, 1758) during competition for shelter; that is, to acquire available resources, intruders increased their movement more than residents (Slavík, Horký, Maciak, & Wackermannova, 2016). Specifically, individuals familiar with an area tend to be more successful in designating that area as a territory, so the specific behavior patterns of intruders, for example, increased movement, may be motivated by an effort to obtain more information about the local environment (Bruinzeel & van de Pol, 2004). Spatial information about a physical habitat, including the available resources, is considered crucial for achieving site familiarity (e.g., Piper, 2011). Although no relationship between movement and side fidelity was found in juvenile salmon, higher emigration rates of conspecifics from a high‐density area have been reported (Steingrímsson & Grant, 2003).

A similar relationship that shown between brown trout movement and subpopulation site fidelity occurred in the interaction with slope, that is, the reduced movement of brown trout from subpopulations with high site fidelity was observed in the stretches with higher slopes. Water velocities affect the spatial and temporal distribution of salmonids (see Klemetsen et al., 2003 for review). For example, juvenile salmon preferred stretches with high velocities in allopatry with older conspecifics (Höjesjö, Kaspersson, & Armstrong, 2016), and the effectiveness of nocturnal feeding appeared to be a combination of the intensity of lunar illumination and water velocity (Metcalfe et al., 1997). A negative correlation between the intensity of brown trout migrations and river slope was reported by Slavík, Horký, Randák, Balvín, and Bílý (2012), and wider spatial distributions of juvenile salmon in stretches with lower velocities were found by Steingrímsson and Grant (2003). Our results tended to show that brown trout from stretches with higher slopes remained within a single habitat, for example, pools, to reduce their daily energy costs. An inverse relationship between feeding mobility and water velocity in salmonids including brown trout was reported by an early study (Tunney & Steingrímsson, 2012). Similarly, brown trout prefer to occupy positions in a stream that favor the intake of food that is drifting with the flow (Bachman, 1984; Fausch, 1984), so fish at positions with higher slope‐related velocities may show reduced movement. Further, substrate size increases with increasing river slope (Chow, 1959), and large boulders increase visual isolation and reduce the size of brown trout territories (Höjesjö et al., 2004; Imre, Grant, & Keeley, 2002) that can be defended, reducing the required amount of movement. It can be assumed that the data presented here for brown trout movement represent the different energy costs related to resources exploitation in different habitats. The energy costs of movement in slope‐related velocities can be compensate by benefits of the environment represented, for example, by facilitated intake of drifting prey and/or reduced agonistic behavior.

### 4.2 Movement and population density

Brown trout displayed reduced movement in high‐density subpopulations, which agrees with former studies that found reduced activity to be a consequence of competitive pressure related to resource allocation in Atlantic salmon (Armstrong & Griffiths, 2001) and bullhead (Davey, Doncaster, & Jones, 2009). It is generally agreed that individuals adjust their spatial distribution to avoid contact and competition with conspecifics (Kuefler et al., 2013; Leibold, 1995), which is consistent with our results. With increasing population density, an increase in competitive pressure can be expected (Harrison, Blount, Inger, Norris, & Bearhop, 2011; Rose, Cowan, Winemiller, Myers, & Hilborn, 2001), which often appears as spatial and temporal changes in a distribution (Einum, Sundt‐Hansen, & Nislow, 2006; Kronfeld‐Schor & Dayan, 2003), such as shown for Arctic charr (Fingerle et al., 2016; Guénard et al., 2012; Larranaga & Steingrímsson, 2015). Furthermore, higher aggressiveness and stress accompanying an increase in abundance have been recorded in juvenile brown trout (Kaspersson, Höjesjö, & Pedersen, 2010). Although these parameters were not observed during our study, we can assume that lower movement is as an adaptation to avoid pressure from conspecifics in high‐density populations.

### 4.3 Body size‐related movement

In riverine environments, body size determines most of the variability related to the spatial needs of juvenile salmonids (Elliott, 1990; Grant & Kramer, 1990; Grant, Noakes, & Jonas, 1989; Keeley & Grant, 1995). In our study, the largest individuals moved the most, corresponding with earlier studies that reported higher mobility by larger conspecifics in salmonids (Armstrong, Braithwaite, & Huntingford, 1997; Parra, Almódovar, Ayllón, Nicola, & Elvira, 2011), other fish species (Kobler, Klefoth, Wolter, Fredrich, & Arlinghaus, 2008; Landsman et al., 2015), and amphibians (Marzeole, 2001). Accordingly, the metabolic hypothesis (i.e., see Brown & Braithwaite, 2004; Krause, Loader, McDermott, & Ruxton, 1998) predicts that changes in movement are related to physiological status (e.g., hunger level, size of energy reserves) rather than body size itself. For example, smaller individual zebrafish (*Danio rerio* Hamilton, 1822) were more active in areas with a potentially higher predation risk (Polverino, Bierbach, Killen, Uusi‐Heikkilä, & Arlinghaus, 2016). Similarly, smaller catfish (*S. glanis*) showed more motivation to search for shelter than larger conspecifics (Slavík et al., 2016). However, the results from the field study presented here tended to show a positive relationship between body size and movement at the subpopulation level. Similar questions have been raised about the relationship between body size and territory or the size of feeding areas in brown trout and other salmonids, with studies generally reporting a positive correlation but also high intra‐ and interspecies variability, the interpretations of which are not clear (see Nicola et al., 2016).

### 4.4 Movement during 24‐hr and lunar cycles

The results presented here agree with former studies observing brown trout feeding activity over a 24‐hr cycle (Elliott, 1973; Kalleberg, 1958), with peaks occurring during twilight and at night (Young, 1999). The key factors responsible for the nocturnal activity of salmonids are reported to be lower predation risk (Metcalfe, Fraser, & Burns, 1999) and reduced aggressiveness (Valdimarsson & Metcalfe, 2001), which both facilitate food intake. Furthermore, the lowest movement in our study occurred during the full moon, supporting the assumption that prey changes their spatial distribution to avoid visual predators benefitting from lunar illumination (Longland & Price, 1991). For example, to avoid predation risks during their return to refuges (Riou & Hamer, 2008), prey species stayed hidden (Kotler, Brown, & Hasson, 1991; Price, Waser, & Bass, 1984) or showed lower movement activity (Morrison, 1978), which was followed by reduced activity of aquatic (Horký, Slavík, Bartoš, Kolářová, & Randák, 2006) or terrestrial predators (Sábato, de Melo, Magni, Young, & Coelho, 2006). However, increases in the activity of predators have rarely been observed within the lunar cycle. For example, Eurasian eagle–owls (*Bubo bubo* Linnaeus, 1758) nurturing offspring displayed increased activity during a full moon, but solitary individuals did not (Penteriani, Kuparinen, Delgado, Lourenco, & Campioni, 2011). Juvenile salmon were more effective at feeding during bright nights under a full moon when they fed in stretches with high water velocities and on dark nights when they shifted to habitats with lower velocities (Metcalfe et al., 1997). The lowest intensity of brown trout spawning migrations was observed during a full moon in an earlier study performed within the same catchment area (Slavík et al., 2012). However, based on the available data, it is not possible to conclude whether reduced brown trout movement corresponded with antipredation behaviors or feeding strategy.

### 4.5 Probability of contact between tagged individuals

Presented results indicate that changes in distance between radio‐tagged individuals were not random showing a higher probability of contact between individuals in subpopulations with high site fidelity and density. The long‐term relation to preferred areas, that is, site fidelity forms a social population structure displayed by lower spatial requirements and reduced agonistic interactions (Stamps, 1991; Wolf & Trillmich, 2007). The influence of social familiarity on effective food intake, higher fitness, better protection from predators and less aggressive interactions were reported for brown trout (Griffiths, Brockmark, Höjesjö, & Johnsson, 2004; Höjesjö, Johnsson, Petersson, & Järvi, 1998). High site fidelity increases social familiarity, and more frequent contact between individuals does not necessarily result in aggressive behavior associated with stress; hence, brown trout could display faster growth than growth observed in subpopulations with low density and site fidelity as shown by Závorka et al. (2016). Furthermore, the results presented here show a high probability of contact between two similarly sized individuals in a subpopulation, while the probability that large individuals meet smaller conspecifics decreases with increasing differences in their sizes. Correspondingly, different size groups of salmonids occupy different environments, for example, differentiated by velocities (Armstrong, Kemp, Kennedy, Ladle, & Milner, 2003; Höjesjö et al., 2016). Additionally, the higher probability of contact between individuals was recorded during daylight, when fish also showed lower movement, suggesting the higher usage of preferred areas with concealments. Salmonids are able to share concealments as reported by earlier studies (Griffiths & Armstrong, 2002; Valdimarsson, Metcalfe, Thorpe, & Huntingford, 1997). The lower probability of contact occurred during twilight and dark, which can be associated with movement among feeding areas (Metcalfe et al., 1999; Steingrímsson & Grant, 2008). Although the full moon significantly reduced the movement of brown trout, a similar relationship was not shown for the probability of contact. The high probability of contact occurred just after the full moon, which again, indicates the usage of concealments in preferred areas; however, further observations are required for a more satisfactory interpretation.

## 5 CONCLUSIONS

The findings presented here illustrate the ability of animals to alter movement based on social and physical conditions and the tendency to reuse preferred areas. Brown trout showed reduced movement in subpopulations with high site fidelity and density and within the high‐slope stretches. The results tended to show the influence of site fidelity on the social structure of the brown trout subpopulation, and the spatial requirements and energy costs of their movement supported the reuse of preferred areas. This study also revealed the reduced movement of brown trout in the wild was correlated with the full moon and daylight indicating the influence of physical conditions on the spatial distribution of individuals. Behavioral ecology research is mostly performed under experimental conditions in laboratories, but there are examples that show considerable differences in activity patterns of the same species or even the same individual between the laboratory and the natural environment (Calisi & Bentley, 2009; Hut, Kronfeld‐Schor, van der Vinne, & de la Iglesia, 2012). Thus, observations of animals in the wild appear to be necessary for verifying the reported effects of population and environmental parameters.

## CONFLICT OF INTEREST

None declared.

## AUTHOR CONTRIBUTIONS

Ondřej Slavík conceived and designed the experiments, performed the experiments, wrote the paper, and reviewed drafts of the paper. Pavel Horký conceived and designed the experiments, performed the experiments, analyzed the data, prepared figures or tables, contributed materials/analysis tools, and reviewed drafts of the paper. Matúš Maciak analyzed the data, developed the statistical model, contributed materials/analysis tools, and prepared figures. Petra Horká analyzed the data and reviewed drafts of the paper. Iva Langrová analyzed the data and reviewed drafts of the paper.
