# Supplementary material for: Diel movement of brown trout, Salmo trutta, is reduced in dense populations with high site fidelity
Source: Ecol Evol. 2018 Apr 6;8(9):4495–507. doi: 10.1002/ece3.3981 (PMC5938464; doi:10.1002/ece3.3981)
Supplement: Supplementary file 1 [file ECE3-8-4495-s001.pdf]

code	date	light_interval	moon_phase	body_size	movement_log
13	16MAY2011	day	5	48	1.00004
13	16MAY2011	day	5	48	0.30125
13	16MAY2011	day	5	48	0.47727
13	16MAY2011	twilight	5	48	1.14616
13	16MAY2011	night	5	48	1.11398
13	16MAY2011	night	5	48	1.25530
13	17MAY2011	twilight	5	48	1.04143
13	17MAY2011	day	5	48	0.00043
13	17MAY2011	day	5	48	1.11398
13	17MAY2011	day	5	48	0.60217
13	17MAY2011	day	5	48	0.30125
13	17MAY2011	twilight	5	48	1.04143
13	17MAY2011	night	5	48	1.11398
13	17MAY2011	night	5	48	1.17612
13	18MAY2011	twilight	5	48	1.17612
13	18MAY2011	day	5	48	-3.00000
13	25MAY2011	day	7	48	0.69906
13	25MAY2011	day	7	48	1.47714
13	25MAY2011	day	7	48	1.41499
13	25MAY2011	twilight	7	48	0.77822
13	25MAY2011	night	7	48	0.95429
13	25MAY2011	night	7	48	0.84516
13	26MAY2011	twilight	7	48	1.00004
13	26MAY2011	day	7	48	0.47727
13	26MAY2011	day	7	48	-3.00000
13	26MAY2011	day	7	48	0.84516
13	26MAY2011	day	7	48	1.79240
13	26MAY2011	twilight	7	48	1.79935
13	26MAY2011	night	8	48	0.60217
13	27MAY2011	twilight	8	48	1.07922
13	27MAY2011	day	8	48	0.69906
13	15JUN2011	day	5	123	1.27878
13	15JUN2011	day	5	123	1.25530
13	15JUN2011	day	5	123	2.49416
13	15JUN2011	twilight	5	123	2.49693
13	15JUN2011	night	5	123	2.49831
13	15JUN2011	night	5	123	1.38023
13	15JUN2011	twilight	5	123	1.43138
13	16JUN2011	day	5	123	-3.00000
13	16JUN2011	day	5	123	1.30105
13	16JUN2011	day	5	123	1.25530
13	16JUN2011	day	5	123	1.30105
13	16JUN2011	day	5	123	1.30105
13	16JUN2011	twilight	5	123	1.25530
13	16JUN2011	night	5	123	1.76344
13	16JUN2011	twilight	5	123	1.74037
13	17JUN2011	day	5	123	1.25530
13	22JUN2011	day	7	123	1.27878
13	22JUN2011	day	7	123	0.90314
13	22JUN2011	day	7	123	1.17612
13	22JUN2011	twilight	7	123	0.95429
13	22JUN2011	night	7	123	1.23047
13	22JUN2011	night	7	123	1.51853
13	23JUN2011	twilight	7	123	1.47714
13	23JUN2011	day	7	123	1.17612

13	23JUN2011	day	7	123	0.95429
13	23JUN2011	day	7	123	0.84516
13	23JUN2011	day	7	123	1.20415
13	23JUN2011	twilight	7	123	1.34244
13	23JUN2011	night	7	123	1.11398
13	23JUN2011	night	7	123	0.95429
13	24JUN2011	twilight	7	123	0.90314
13	24JUN2011	day	7	123	1.25530
13	27JUN2011	day	8	123	0.60217
13	27JUN2011	day	8	123	0.60217
13	27JUN2011	day	8	123	0.69906
13	27JUN2011	twilight	8	123	1.20415
13	27JUN2011	night	8	123	1.78534
13	27JUN2011	night	8	123	1.54408
13	28JUN2011	twilight	8	123	1.30105
13	28JUN2011	day	8	123	1.11398
13	28JUN2011	day	8	123	0.60217
13	28JUN2011	day	8	123	0.77822
13	28JUN2011	day	8	123	1.14616
13	28JUN2011	twilight	8	123	1.20415
13	28JUN2011	night	1	123	1.70758
13	28JUN2011	night	1	123	1.60207
13	29JUN2011	twilight	1	123	0.69906
13	29JUN2011	day	1	123	1.23047
14	16MAY2011	day	5	87	1.20415
14	16MAY2011	day	5	87	-3.00000
14	16MAY2011	day	5	87	-3.00000
14	16MAY2011	twilight	5	87	1.30105
14	16MAY2011	night	5	87	1.60207
14	16MAY2011	night	5	87	0.77822
14	17MAY2011	twilight	5	87	1.23047
14	17MAY2011	day	5	87	-3.00000
14	17MAY2011	day	6	87	1.79240
14	17MAY2011	day	6	87	1.14616
14	17MAY2011	day	6	87	1.00004
14	17MAY2011	twilight	6	87	0.95429
14	17MAY2011	night	6	87	0.77822
14	17MAY2011	night	6	87	1.11398
14	18MAY2011	twilight	6	87	1.20415
14	18MAY2011	day	6	87	1.38023
14	25MAY2011	day	7	87	1.50516
14	25MAY2011	day	7	87	1.43138
14	25MAY2011	day	7	87	0.69906
14	25MAY2011	twilight	7	87	1.34244
14	25MAY2011	night	7	87	0.00043
14	26MAY2011	twilight	7	87	1.62326
14	26MAY2011	day	7	87	0.95429
14	26MAY2011	day	7	87	1.43138
14	26MAY2011	day	7	87	1.14616
14	26MAY2011	day	7	87	1.81292
14	26MAY2011	twilight	7	87	1.77816
14	26MAY2011	night	7	87	1.34244
14	26MAY2011	night	7	87	1.20415
14	27MAY2011	twilight	8	87	0.95429
14	27MAY2011	day	8	87	1.04143
14	01JUN2011	day	1	87	1.17612

14	01JUN2011	day	1	87	0.30125
14	01JUN2011	day	1	87	1.43138
14	01JUN2011	twilight	1	87	1.14616
14	01JUN2011	night	1	87	0.69906
14	01JUN2011	night	1	87	1.17612
14	02JUN2011	twilight	1	87	1.53149
14	02JUN2011	day	1	87	1.25530
14	02JUN2011	day	1	87	1.04143
14	02JUN2011	day	1	87	1.36175
14	02JUN2011	day	1	87	0.69906
14	02JUN2011	twilight	1	87	1.54408
14	02JUN2011	night	1	87	1.11398
14	02JUN2011	night	2	87	1.50516
14	03JUN2011	twilight	2	87	0.60217
14	03JUN2011	day	2	87	0.60217
14	06JUN2011	day	2	87	0.69906
14	06JUN2011	day	2	87	1.00004
14	06JUN2011	day	2	87	1.43138
14	06JUN2011	twilight	2	87	0.77822
14	06JUN2011	night	3	87	1.62326
14	06JUN2011	night	3	87	1.17612
14	07JUN2011	twilight	3	87	1.30105
14	07JUN2011	day	3	87	1.39796
15	16MAY2011	day	5	59	1.20415
15	16MAY2011	day	5	59	-3.00000
15	16MAY2011	day	5	59	0.30125
15	16MAY2011	twilight	5	59	1.51853
15	16MAY2011	night	5	59	1.79935
15	16MAY2011	night	5	59	1.79935
15	17MAY2011	twilight	5	59	1.39796
15	17MAY2011	day	5	59	1.32224
15	17MAY2011	day	6	59	1.64346
15	17MAY2011	day	6	59	1.39796
15	17MAY2011	day	6	59	1.72428
15	17MAY2011	twilight	6	59	1.79935
15	17MAY2011	night	6	59	0.90314
15	17MAY2011	night	6	59	1.73240
15	18MAY2011	twilight	6	59	1.90310
15	18MAY2011	day	6	59	1.59108
15	25MAY2011	day	7	59	1.41499
15	25MAY2011	day	7	59	1.43138
15	25MAY2011	day	7	59	1.36175
15	25MAY2011	day	7	59	1.62326
15	25MAY2011	twilight	7	59	1.81292
15	25MAY2011	night	7	59	1.07922
15	26MAY2011	twilight	7	59	1.00004
15	26MAY2011	day	7	59	1.91908
15	26MAY2011	day	7	59	1.83886
15	26MAY2011	twilight	7	59	1.49138
15	26MAY2011	night	7	59	1.79935
15	26MAY2011	night	7	59	0.77822
15	27MAY2011	twilight	8	59	1.77816
15	27MAY2011	day	8	59	0.90314
15	01JUN2011	day	1	59	1.38023
15	01JUN2011	day	1	59	0.30125
15	01JUN2011	twilight	1	59	1.34244

15	01JUN2011	night	1	59	0.95429
15	01JUN2011	night	1	59	0.69906
15	02JUN2011	twilight	1	59	0.30125
15	02JUN2011	day	1	59	1.63348
15	02JUN2011	day	1	59	1.14616
15	02JUN2011	day	1	59	1.07922
15	02JUN2011	day	1	59	1.36175
15	02JUN2011	twilight	1	59	0.84516
15	02JUN2011	night	2	59	0.69906
15	03JUN2011	twilight	2	59	0.77822
15	03JUN2011	day	2	59	0.95429
15	06JUN2011	day	2	59	0.60217
15	06JUN2011	day	2	59	0.47727
15	06JUN2011	day	2	59	0.95429
15	06JUN2011	twilight	2	59	0.77822
15	06JUN2011	night	3	59	0.84516
15	06JUN2011	night	3	59	1.17612
15	07JUN2011	twilight	3	59	1.63348
15	07JUN2011	day	3	59	0.95429
16	16MAY2011	day	5	67	0.90314
16	16MAY2011	day	5	67	0.30125
16	16MAY2011	day	5	67	-3.00000
16	16MAY2011	twilight	5	67	0.95429
16	16MAY2011	night	5	67	0.30125
16	16MAY2011	night	5	67	1.57980
16	17MAY2011	twilight	5	67	1.41499
16	17MAY2011	day	5	67	1.17612
16	17MAY2011	day	6	67	0.90314
16	17MAY2011	day	6	67	1.07922
16	17MAY2011	day	6	67	1.39796
16	17MAY2011	twilight	6	67	-3.00000
16	17MAY2011	night	6	67	0.60217
16	17MAY2011	night	6	67	1.00004
16	18MAY2011	twilight	6	67	1.61279
16	18MAY2011	day	6	67	1.59108
16	25MAY2011	day	7	67	0.60217
16	25MAY2011	day	7	67	1.07922
16	25MAY2011	day	7	67	1.69898
16	25MAY2011	day	7	67	0.90314
16	25MAY2011	night	7	67	1.80619
16	25MAY2011	night	7	67	1.56821
16	26MAY2011	twilight	7	67	1.51853
16	26MAY2011	day	7	67	1.34244
16	26MAY2011	day	7	67	1.71601
16	26MAY2011	twilight	7	67	1.11398
16	26MAY2011	night	7	67	1.85126
16	26MAY2011	night	7	67	0.95429
16	27MAY2011	twilight	8	67	1.30105
16	27MAY2011	day	8	67	1.07922
16	01JUN2011	day	1	67	0.47727
16	01JUN2011	day	1	67	1.59108
16	01JUN2011	day	1	67	1.59108
16	01JUN2011	twilight	1	67	1.34244
16	01JUN2011	night	1	67	1.07922
16	01JUN2011	night	1	67	1.43138
16	02JUN2011	twilight	1	67	1.77816

16	02JUN2011	day	1	67	0.77822
16	02JUN2011	day	1	67	1.79935
16	02JUN2011	day	1	67	1.65322
16	02JUN2011	day	1	67	1.34244
16	02JUN2011	twilight	1	67	1.23047
16	02JUN2011	night	1	67	0.84516
16	02JUN2011	night	2	67	0.84516
16	03JUN2011	twilight	2	67	1.07922
16	03JUN2011	day	2	67	0.00043
16	06JUN2011	day	2	67	0.69906
16	06JUN2011	day	2	67	1.11398
16	06JUN2011	day	2	67	1.30105
16	06JUN2011	twilight	2	67	0.90314
16	06JUN2011	night	3	67	0.30125
16	06JUN2011	night	3	67	0.60217
16	07JUN2011	twilight	3	67	1.75588
16	07JUN2011	day	3	67	1.49138
17	16MAY2011	day	5	34	1.20415
17	16MAY2011	day	5	34	0.00043
17	16MAY2011	day	5	34	0.60217
17	16MAY2011	twilight	5	34	1.23047
17	16MAY2011	night	5	34	1.04143
17	16MAY2011	night	5	34	1.20415
17	17MAY2011	twilight	5	34	1.11398
17	17MAY2011	day	5	34	1.23047
17	17MAY2011	day	6	34	1.41499
17	17MAY2011	day	6	34	0.95429
17	17MAY2011	day	6	34	0.95429
17	17MAY2011	twilight	6	34	0.47727
17	17MAY2011	night	6	34	-3.00000
17	17MAY2011	night	6	34	1.14616
17	18MAY2011	twilight	6	34	1.61279
17	18MAY2011	day	6	34	1.59108
17	25MAY2011	day	7	34	1.07922
17	25MAY2011	day	7	34	1.00004
17	25MAY2011	day	7	34	0.77822
17	25MAY2011	day	7	34	1.14616
17	25MAY2011	twilight	7	34	0.60217
17	25MAY2011	night	7	34	0.95429
17	26MAY2011	twilight	7	34	0.60217
17	26MAY2011	day	7	34	0.60217
17	26MAY2011	day	7	34	1.04143
17	26MAY2011	day	7	34	0.60217
17	26MAY2011	day	7	34	1.30105
17	26MAY2011	twilight	7	34	1.04143
17	26MAY2011	night	7	34	1.39796
17	26MAY2011	night	7	34	0.77822
17	27MAY2011	twilight	8	34	0.90314
17	27MAY2011	day	8	34	0.90314
19	16MAY2011	day	5	58	0.69906
19	16MAY2011	day	5	58	0.60217
19	16MAY2011	day	5	58	0.60217
19	16MAY2011	twilight	5	58	1.43138
19	16MAY2011	night	5	58	1.47714
19	16MAY2011	night	5	58	1.04143
19	17MAY2011	twilight	5	58	1.17612

19	17MAY2011	day	5	58	0.30125
19	17MAY2011	day	5	58	0.30125
19	17MAY2011	day	5	58	-3.00000
19	17MAY2011	day	5	58	0.69906
19	17MAY2011	twilight	5	58	0.77822
19	17MAY2011	night	5	58	1.30105
19	17MAY2011	night	5	58	1.00004
19	18MAY2011	twilight	5	58	-3.00000
19	18MAY2011	day	5	58	0.47727
19	25MAY2011	day	7	58	0.77822
19	25MAY2011	day	7	58	0.69906
19	25MAY2011	day	7	58	0.77822
19	25MAY2011	twilight	7	58	0.47727
19	25MAY2011	night	7	58	0.47727
19	25MAY2011	night	7	58	0.30125
19	26MAY2011	twilight	7	58	1.04143
19	26MAY2011	day	7	58	1.07922
19	26MAY2011	day	7	58	1.20415
19	26MAY2011	day	7	58	1.32224
19	26MAY2011	day	7	58	1.34244
19	26MAY2011	twilight	7	58	0.60217
19	27MAY2011	twilight	8	58	0.77822
19	27MAY2011	day	8	58	0.90314
19	01JUN2011	day	1	58	1.11398
19	01JUN2011	day	1	58	0.47727
19	01JUN2011	day	1	58	1.30105
19	01JUN2011	twilight	1	58	0.60217
19	01JUN2011	night	1	58	1.17612
19	01JUN2011	night	1	58	1.23047
19	02JUN2011	twilight	1	58	0.47727
19	02JUN2011	day	1	58	1.04143
19	02JUN2011	day	1	58	1.25530
19	02JUN2011	day	1	58	1.23047
19	02JUN2011	day	1	58	1.39796
19	02JUN2011	twilight	1	58	1.62326
19	02JUN2011	night	1	58	1.34244
19	02JUN2011	night	2	58	0.84516
19	03JUN2011	twilight	2	58	1.04143
19	03JUN2011	day	2	58	0.69906
19	06JUN2011	day	2	58	0.47727
19	06JUN2011	day	2	58	0.30125
19	06JUN2011	day	2	58	1.14616
19	06JUN2011	twilight	2	58	0.47727
19	06JUN2011	night	3	58	1.04143
19	06JUN2011	night	3	58	0.47727
19	07JUN2011	twilight	3	58	0.84516
19	07JUN2011	day	3	58	0.30125
20	15JUN2011	day	5	65	0.90314
20	15JUN2011	day	5	65	-3.00000
20	15JUN2011	day	5	65	0.90314
20	15JUN2011	twilight	5	65	0.90314
20	15JUN2011	night	5	65	1.34244
20	15JUN2011	night	5	65	1.14616
20	15JUN2011	twilight	5	65	2.25042
20	16JUN2011	day	5	65	1.36175
20	16JUN2011	day	5	65	0.84516

20	16JUN2011	day	5	65	0.77822
20	16JUN2011	twilight	5	65	1.11398
20	16JUN2011	night	5	65	0.84516
20	16JUN2011	twilight	5	65	1.49138
20	17JUN2011	day	5	65	1.17612
20	22JUN2011	day	7	65	0.60217
20	22JUN2011	day	7	65	0.60217
20	22JUN2011	day	7	65	1.51853
20	22JUN2011	twilight	7	65	0.69906
20	22JUN2011	night	7	65	1.81955
20	22JUN2011	night	7	65	1.77086
20	23JUN2011	twilight	7	65	1.17612
20	23JUN2011	day	7	65	1.07922
20	23JUN2011	day	7	65	0.47727
20	23JUN2011	day	7	65	0.60217
20	23JUN2011	day	7	65	1.47714
20	23JUN2011	twilight	7	65	1.00004
20	23JUN2011	night	7	65	1.46241
20	23JUN2011	night	7	65	0.47727
20	24JUN2011	twilight	7	65	1.34244
20	24JUN2011	day	7	65	0.47727
20	27JUN2011	day	8	65	0.60217
20	27JUN2011	day	8	65	-3.00000
20	27JUN2011	day	8	65	0.47727
20	27JUN2011	twilight	8	65	1.49138
20	27JUN2011	night	8	65	1.50516
20	27JUN2011	night	8	65	0.84516
20	28JUN2011	twilight	8	65	0.77822
20	28JUN2011	day	8	65	0.60217
20	28JUN2011	day	8	65	1.38023
20	28JUN2011	day	8	65	0.69906
20	28JUN2011	day	8	65	0.69906
20	28JUN2011	twilight	8	65	0.69906
20	28JUN2011	night	1	65	-3.00000
20	28JUN2011	night	1	65	0.47727
20	29JUN2011	twilight	1	65	0.47727
20	29JUN2011	day	1	65	0.77822
22	16MAY2011	day	5	24	1.20415
22	16MAY2011	day	5	24	0.30125
22	16MAY2011	day	5	24	-3.00000
22	16MAY2011	twilight	5	24	1.17612
22	16MAY2011	night	5	24	1.61279
22	16MAY2011	night	5	24	1.30105
22	17MAY2011	twilight	5	24	1.82608
22	17MAY2011	day	5	24	1.77086
22	17MAY2011	day	6	24	1.77816
22	17MAY2011	day	6	24	0.84516
22	17MAY2011	day	6	24	1.88650
22	17MAY2011	twilight	6	24	1.82608
22	17MAY2011	night	6	24	0.77822
22	17MAY2011	night	6	24	1.23047
22	18MAY2011	twilight	6	24	1.61279
22	18MAY2011	day	6	24	1.65322
22	25MAY2011	day	7	24	1.14616
22	25MAY2011	day	7	24	1.07922
22	25MAY2011	day	7	24	0.90314

22	25MAY2011	twilight	7	24	1.27878
22	25MAY2011	night	7	24	0.95429
22	26MAY2011	twilight	7	24	1.32224
22	26MAY2011	day	7	24	0.30125
22	26MAY2011	day	7	24	1.07922
22	26MAY2011	day	7	24	1.47714
22	26MAY2011	twilight	7	24	1.14616
22	26MAY2011	night	7	24	0.90314
22	26MAY2011	night	7	24	1.30105
22	27MAY2011	twilight	8	24	1.04143
22	27MAY2011	day	8	24	1.47714
22	01JUN2011	day	1	24	1.20415
22	01JUN2011	day	1	24	1.04143
22	01JUN2011	day	1	24	1.65322
22	02JUN2011	twilight	1	24	1.74820
22	02JUN2011	day	1	24	0.60217
22	02JUN2011	day	1	24	1.51853
22	02JUN2011	day	1	24	1.43138
22	02JUN2011	day	1	24	1.38023
22	02JUN2011	twilight	1	24	1.59108
22	02JUN2011	night	1	24	1.11398
22	02JUN2011	night	2	24	1.79935
22	03JUN2011	twilight	2	24	0.60217
22	03JUN2011	day	2	24	0.60217
22	06JUN2011	day	2	24	0.95429
22	06JUN2011	day	2	24	1.04143
22	06JUN2011	day	2	24	1.59108
22	06JUN2011	twilight	2	24	1.41499
22	06JUN2011	night	3	24	1.55631
22	06JUN2011	night	3	24	0.95429
22	07JUN2011	twilight	3	24	1.68125
22	07JUN2011	day	3	24	1.07922
23	16MAY2011	day	5	21	0.95429
23	16MAY2011	day	5	21	0.30125
23	16MAY2011	twilight	5	21	1.25530
23	16MAY2011	night	5	21	1.66277
23	16MAY2011	night	5	21	0.77822
23	17MAY2011	twilight	5	21	0.60217
23	17MAY2011	day	5	21	1.00004
23	17MAY2011	day	6	21	1.11398
23	17MAY2011	day	6	21	1.89763
23	17MAY2011	twilight	6	21	1.92942
23	17MAY2011	night	6	21	1.25530
23	17MAY2011	night	6	21	0.95429
23	18MAY2011	twilight	6	21	1.64346
23	18MAY2011	day	6	21	1.59108
23	25MAY2011	day	7	21	1.50516
23	25MAY2011	day	7	21	1.11398
23	25MAY2011	day	7	21	1.07922
23	25MAY2011	day	7	21	0.00043
23	25MAY2011	twilight	7	21	1.53149
23	25MAY2011	night	7	21	1.43138
23	26MAY2011	twilight	7	21	1.62326
23	26MAY2011	day	7	21	1.07922
23	26MAY2011	day	7	21	1.88650
23	26MAY2011	day	7	21	0.77822

23	26MAY2011	day	7	21	1.04143
23	26MAY2011	twilight	7	21	1.71601
23	26MAY2011	night	7	21	0.60217
23	26MAY2011	night	7	21	1.17612
23	27MAY2011	twilight	8	21	1.04143
23	27MAY2011	day	8	21	1.04143
23	01JUN2011	day	1	21	1.34244
23	01JUN2011	day	1	21	0.69906
23	01JUN2011	day	1	21	0.60217
23	01JUN2011	twilight	1	21	0.90314
23	01JUN2011	night	1	21	1.51853
23	01JUN2011	night	1	21	1.27878
23	02JUN2011	twilight	1	21	1.39796
23	02JUN2011	day	1	21	1.04143
23	02JUN2011	day	1	21	1.34244
23	02JUN2011	day	1	21	1.78534
23	02JUN2011	twilight	1	21	1.00004
23	02JUN2011	night	1	21	0.69906
23	02JUN2011	night	2	21	1.76344
23	03JUN2011	twilight	2	21	0.60217
23	03JUN2011	day	2	21	0.69906
23	06JUN2011	day	2	21	0.60217
23	06JUN2011	day	2	21	0.77822
23	06JUN2011	day	2	21	0.69906
23	06JUN2011	twilight	2	21	0.90314
23	06JUN2011	night	3	21	1.68125
23	06JUN2011	night	3	21	0.90314
23	07JUN2011	twilight	3	21	1.78534
23	07JUN2011	day	3	21	0.77822
24	16MAY2011	day	5	56	0.90314
24	16MAY2011	day	5	56	1.17612
24	16MAY2011	day	5	56	0.30125
24	16MAY2011	twilight	5	56	0.60217
24	16MAY2011	night	5	56	0.69906
24	16MAY2011	night	5	56	0.69906
24	17MAY2011	twilight	5	56	0.30125
24	17MAY2011	day	5	56	0.30125
24	17MAY2011	day	5	56	1.17612
24	17MAY2011	day	5	56	0.47727
24	17MAY2011	day	5	56	0.30125
24	17MAY2011	twilight	5	56	1.30105
24	17MAY2011	night	5	56	0.90314
24	17MAY2011	night	5	56	1.17612
24	18MAY2011	twilight	5	56	1.30105
24	18MAY2011	day	5	56	-3.00000
24	25MAY2011	day	7	56	0.77822
24	25MAY2011	day	7	56	0.60217
24	25MAY2011	day	7	56	0.60217
24	25MAY2011	twilight	7	56	1.07922
24	25MAY2011	night	7	56	1.36175
24	25MAY2011	night	7	56	0.84516
24	26MAY2011	twilight	7	56	1.00004
24	26MAY2011	day	7	56	0.60217
24	26MAY2011	day	7	56	0.00043
24	26MAY2011	day	7	56	0.69906
24	26MAY2011	day	7	56	0.60217

24	26MAY2011	twilight	7	56	0.60217
24	27MAY2011	twilight	8	56	0.84516
24	27MAY2011	day	8	56	0.90314
24	01JUN2011	day	1	56	0.84516
24	01JUN2011	day	1	56	1.20415
24	01JUN2011	twilight	1	56	1.20415
24	01JUN2011	night	1	56	1.11398
24	01JUN2011	night	1	56	0.77822
24	02JUN2011	twilight	1	56	-3.00000
24	02JUN2011	day	1	56	0.69906
24	02JUN2011	day	1	56	1.11398
24	02JUN2011	day	1	56	1.34244
24	02JUN2011	day	1	56	0.00043
24	02JUN2011	twilight	1	56	0.60217
24	02JUN2011	night	2	56	0.84516
24	03JUN2011	twilight	2	56	0.84516
24	03JUN2011	day	2	56	0.77822
24	06JUN2011	day	2	56	0.47727
24	06JUN2011	day	2	56	0.60217
24	06JUN2011	day	2	56	0.77822
24	06JUN2011	twilight	2	56	0.60217
24	06JUN2011	night	3	56	1.14616
24	06JUN2011	night	3	56	0.77822
24	07JUN2011	twilight	3	56	0.69906
24	07JUN2011	day	3	56	0.77822
24	07JUN2011	day	3	56	0.60217
27	16MAY2011	day	5	46	1.04143
27	16MAY2011	day	5	46	0.60217
27	16MAY2011	day	5	46	0.60217
27	16MAY2011	twilight	5	46	0.30125
27	16MAY2011	night	5	46	1.25530
27	16MAY2011	night	5	46	1.25530
27	17MAY2011	twilight	5	46	0.60217
27	17MAY2011	day	5	46	-3.00000
27	17MAY2011	day	5	46	0.77822
27	17MAY2011	day	5	46	1.07922
27	17MAY2011	twilight	5	46	0.84516
27	17MAY2011	night	5	46	1.11398
27	17MAY2011	night	5	46	1.46241
27	18MAY2011	twilight	5	46	1.17612
27	18MAY2011	day	5	46	0.47727
27	25MAY2011	day	7	46	1.17612
27	25MAY2011	day	7	46	0.95429
27	25MAY2011	day	7	46	0.60217
27	25MAY2011	twilight	7	46	0.84516
27	25MAY2011	night	7	46	0.69906
27	25MAY2011	night	7	46	1.07922
27	26MAY2011	twilight	7	46	1.07922
27	26MAY2011	day	7	46	0.30125
27	26MAY2011	day	7	46	0.30125
27	26MAY2011	day	7	46	0.69906
27	26MAY2011	day	7	46	0.60217
27	26MAY2011	twilight	7	46	1.04143
27	26MAY2011	night	8	46	0.30125
27	27MAY2011	twilight	8	46	0.90314
27	27MAY2011	day	8	46	0.95429

27	01JUN2011	day	1	46	-3.00000
27	01JUN2011	day	1	46	0.90314
27	01JUN2011	day	1	46	0.77822
27	01JUN2011	twilight	1	46	1.23047
27	01JUN2011	night	1	46	1.38023
27	01JUN2011	night	1	46	1.11398
27	02JUN2011	twilight	1	46	0.30125
27	02JUN2011	day	1	46	-3.00000
27	02JUN2011	day	1	46	0.84516
27	02JUN2011	day	1	46	0.69906
27	02JUN2011	day	1	46	0.30125
27	02JUN2011	twilight	1	46	0.47727
27	02JUN2011	night	2	46	0.84516
27	03JUN2011	twilight	2	46	0.84516
27	03JUN2011	day	2	46	0.69906
27	06JUN2011	day	2	46	0.47727
27	06JUN2011	day	2	46	0.90314
27	06JUN2011	day	2	46	1.00004
27	06JUN2011	twilight	2	46	0.69906
27	06JUN2011	night	3	46	1.04143
27	06JUN2011	night	3	46	0.84516
27	07JUN2011	twilight	3	46	0.90314
27	07JUN2011	day	3	46	0.77822
28	15JUN2011	day	5	53	0.30125
28	15JUN2011	day	5	53	0.00043
28	15JUN2011	day	5	53	0.60217
28	15JUN2011	twilight	5	53	0.60217
28	15JUN2011	night	5	53	0.60217
28	15JUN2011	night	5	53	0.60217
28	15JUN2011	twilight	5	53	0.69906
28	16JUN2011	day	5	53	0.60217
28	16JUN2011	day	5	53	0.84516
28	16JUN2011	day	5	53	0.95429
28	16JUN2011	day	5	53	0.84516
28	16JUN2011	day	5	53	0.84516
28	16JUN2011	twilight	5	53	0.84516
28	16JUN2011	night	5	53	1.04143
28	16JUN2011	twilight	5	53	1.11398
28	17JUN2011	day	5	53	0.69906
28	22JUN2011	day	7	53	0.95429
28	22JUN2011	day	7	53	0.95429
28	22JUN2011	day	7	53	0.95429
28	22JUN2011	twilight	7	53	0.69906
28	22JUN2011	night	7	53	0.69906
28	22JUN2011	night	7	53	1.17612
28	23JUN2011	twilight	7	53	1.14616
28	23JUN2011	day	7	53	0.69906
28	23JUN2011	day	7	53	0.95429
28	23JUN2011	day	7	53	0.95429
28	23JUN2011	day	7	53	1.11398
28	23JUN2011	twilight	7	53	0.95429
28	23JUN2011	night	7	53	0.60217
28	23JUN2011	night	7	53	0.60217
28	24JUN2011	twilight	7	53	-3.00000
28	24JUN2011	day	7	53	0.69906
28	27JUN2011	day	8	53	0.60217

28	27JUN2011	day	8	53	0.69906
28	27JUN2011	day	8	53	0.69906
28	27JUN2011	twilight	8	53	0.47727
28	27JUN2011	night	8	53	0.30125
28	27JUN2011	night	8	53	0.30125
28	28JUN2011	twilight	8	53	0.84516
28	28JUN2011	day	8	53	0.60217
28	28JUN2011	day	8	53	0.30125
28	28JUN2011	day	8	53	-3.00000
28	28JUN2011	day	8	53	0.84516
28	28JUN2011	twilight	8	53	0.90314
28	28JUN2011	night	1	53	0.90314
28	28JUN2011	night	1	53	1.23047
28	29JUN2011	twilight	1	53	1.14616
28	29JUN2011	day	1	53	0.84516
29	30MAY2007	day	4	240	1.46241
29	30MAY2007	day	4	240	0.48586
29	30MAY2007	day	4	240	0.93151
29	30MAY2007	twilight	4	240	1.08994
29	30MAY2007	night	4	240	0.62024
29	31MAY2007	night	4	240	1.76194
29	31MAY2007	twilight	4	240	1.27878
29	31MAY2007	day	4	240	1.61279
29	06JUN2007	day	6	240	1.70330
29	06JUN2007	day	6	240	1.44092
29	06JUN2007	twilight	6	240	1.44250
29	06JUN2007	night	6	240	1.03346
29	07JUN2007	night	6	240	1.08640
29	07JUN2007	twilight	6	240	1.19315
29	07JUN2007	day	6	240	1.08994
29	07JUN2007	day	6	240	0.90693
29	11JUN2007	day	8	240	1.23807
29	11JUN2007	day	8	240	1.24057
29	11JUN2007	day	8	240	1.22274
29	11JUN2007	twilight	8	240	1.11398
29	11JUN2007	night	8	240	1.75359
29	12JUN2007	night	8	240	1.73800
29	12JUN2007	twilight	8	240	1.20415
29	12JUN2007	day	8	240	1.49556
29	12JUN2007	day	8	240	0.95670
29	12JUN2007	day	8	240	1.13675
29	12JUN2007	day	8	240	1.16140
29	12JUN2007	twilight	8	240	1.28106
29	12JUN2007	night	8	240	0.87570
29	13JUN2007	night	8	240	0.61920
29	13JUN2007	twilight	8	240	0.99699
29	13JUN2007	day	8	240	0.60325
29	18JUN2007	day	2	240	1.78248
29	18JUN2007	day	2	240	1.71685
29	18JUN2007	day	2	240	0.80625
29	18JUN2007	twilight	2	240	0.65619
29	18JUN2007	night	2	240	1.69811
29	19JUN2007	night	2	240	1.09694
29	19JUN2007	twilight	2	240	1.02535
29	19JUN2007	day	2	240	1.44406
29	19JUN2007	day	2	240	1.90037

29	19JUN2007	day	2	240	1.02123
29	19JUN2007	day	2	240	1.19593
29	19JUN2007	twilight	2	240	1.73240
29	19JUN2007	night	2	240	1.29228
29	20JUN2007	night	2	240	1.02942
29	20JUN2007	twilight	2	240	0.96759
29	20JUN2007	day	2	240	1.22791
29	25JUN2007	day	4	240	1.56821
29	25JUN2007	day	4	240	1.39095
29	25JUN2007	day	4	240	0.94748
29	25JUN2007	twilight	4	240	0.94156
29	25JUN2007	night	4	240	0.85679
29	26JUN2007	night	4	240	1.51853
29	26JUN2007	twilight	4	240	1.06450
29	26JUN2007	day	4	240	0.82679
29	26JUN2007	day	4	240	0.18498
29	26JUN2007	day	4	240	0.82027
29	26JUN2007	day	4	240	0.12743
29	26JUN2007	twilight	4	240	0.94206
29	26JUN2007	night	4	240	1.49694
29	27JUN2007	night	4	240	1.19315
29	27JUN2007	twilight	4	240	0.28126
29	27JUN2007	day	4	240	0.62439
30	30MAY2007	day	4	122	1.94102
30	30MAY2007	day	4	122	0.90854
30	30MAY2007	day	4	122	0.77166
30	30MAY2007	twilight	4	122	0.62951
30	30MAY2007	night	4	122	1.22274
30	31MAY2007	night	4	122	1.00864
30	31MAY2007	twilight	4	122	1.38204
30	31MAY2007	day	4	122	2.01704
30	06JUN2007	day	6	122	0.63458
30	06JUN2007	day	6	122	0.70079
30	06JUN2007	twilight	6	122	0.49982
30	06JUN2007	night	6	122	1.26954
30	07JUN2007	night	6	122	1.72428
30	07JUN2007	twilight	6	122	1.73240
30	07JUN2007	day	6	122	2.04140
30	07JUN2007	day	6	122	1.33648
30	11JUN2007	day	8	122	1.19315
30	11JUN2007	day	8	122	1.19315
30	11JUN2007	day	8	122	0.98412
30	11JUN2007	twilight	8	122	0.75823
30	11JUN2007	night	8	122	0.60649
30	12JUN2007	night	8	122	0.76275
30	12JUN2007	twilight	8	122	0.92793
30	12JUN2007	day	8	122	0.92018
30	12JUN2007	day	8	122	0.41514
30	12JUN2007	day	8	122	1.07922
30	12JUN2007	day	8	122	0.75519
30	12JUN2007	twilight	8	122	1.28332
30	12JUN2007	night	8	122	1.08282
30	13JUN2007	night	8	122	0.98502
30	13JUN2007	twilight	8	122	0.66661
30	13JUN2007	day	8	122	1.08640
30	18JUN2007	day	2	122	0.99260

30	18JUN2007	day	2	122	0.54419
30	18JUN2007	day	2	122	1.29669
30	18JUN2007	twilight	2	122	1.04536
30	18JUN2007	night	2	122	0.98637
30	19JUN2007	night	2	122	0.88144
30	19JUN2007	twilight	2	122	1.83823
30	19JUN2007	day	2	122	1.81091
30	19JUN2007	day	2	122	0.56478
30	19JUN2007	day	2	122	1.22274
30	19JUN2007	day	2	122	1.12388
30	19JUN2007	twilight	2	122	0.98277
30	19JUN2007	night	2	122	0.23578
30	20JUN2007	night	2	122	1.05312
30	20JUN2007	twilight	2	122	0.69992
30	20JUN2007	day	2	122	1.43138
30	25JUN2007	day	4	122	0.76425
30	25JUN2007	day	4	122	1.15232
30	25JUN2007	day	4	122	1.00864
30	25JUN2007	twilight	4	122	0.61500
30	25JUN2007	night	4	122	0.61500
30	26JUN2007	night	4	122	0.68494
30	26JUN2007	twilight	4	122	0.74593
30	26JUN2007	day	4	122	1.01708
30	26JUN2007	day	4	122	0.61395
30	26JUN2007	day	4	122	0.96759
30	26JUN2007	day	4	122	0.82743
30	26JUN2007	twilight	4	122	0.36754
30	26JUN2007	night	4	122	0.31408
30	27JUN2007	night	4	122	1.10384
30	27JUN2007	twilight	4	122	0.36754
30	27JUN2007	day	4	122	-3.00000
30	16MAY2011	day	5	42	0.77822
30	16MAY2011	day	5	42	0.30125
30	16MAY2011	day	5	42	0.47727
30	16MAY2011	twilight	5	42	1.14616
30	16MAY2011	night	5	42	1.11398
30	16MAY2011	night	5	42	1.17612
30	17MAY2011	twilight	5	42	1.00004
30	17MAY2011	day	5	42	0.00043
30	17MAY2011	day	5	42	0.84516
30	17MAY2011	day	5	42	-3.00000
30	17MAY2011	day	5	42	1.23047
30	17MAY2011	twilight	5	42	1.04143
30	17MAY2011	night	5	42	1.11398
30	17MAY2011	night	5	42	1.17612
30	18MAY2011	twilight	5	42	0.47727
30	18MAY2011	day	5	42	0.47727
30	25MAY2011	day	7	42	0.69906
30	25MAY2011	day	7	42	0.47727
30	25MAY2011	day	7	42	0.77822
30	25MAY2011	twilight	7	42	0.84516
30	25MAY2011	night	7	42	0.77822
30	25MAY2011	night	7	42	0.84516
30	26MAY2011	twilight	7	42	1.07922
30	26MAY2011	day	7	42	-3.00000
30	26MAY2011	day	7	42	0.47727

30	26MAY2011	day	7	42	-3.00000
30	26MAY2011	day	7	42	0.47727
30	26MAY2011	twilight	7	42	0.90314
30	26MAY2011	night	8	42	0.95429
30	27MAY2011	twilight	8	42	1.79935
30	27MAY2011	day	8	42	1.74037
30	01JUN2011	day	1	42	1.11398
30	01JUN2011	day	1	42	0.84516
30	01JUN2011	day	1	42	1.47714
30	01JUN2011	twilight	1	42	1.51853
30	01JUN2011	night	1	42	1.41499
30	01JUN2011	night	1	42	1.25530
30	02JUN2011	twilight	1	42	0.77822
30	02JUN2011	day	1	42	1.17612
30	02JUN2011	day	1	42	0.77822
30	02JUN2011	day	1	42	1.23047
30	02JUN2011	day	1	42	1.23047
30	02JUN2011	twilight	1	42	0.60217
30	02JUN2011	night	2	42	0.69906
30	03JUN2011	twilight	2	42	0.30125
30	03JUN2011	day	2	42	0.77822
30	06JUN2011	day	2	42	0.90314
30	06JUN2011	day	2	42	0.69906
30	06JUN2011	day	2	42	1.23047
30	06JUN2011	twilight	2	42	0.60217
30	06JUN2011	night	3	42	0.95429
30	06JUN2011	night	3	42	1.25530
30	07JUN2011	twilight	3	42	1.25530
30	07JUN2011	day	3	42	0.69906
31	30MAY2007	day	4	191	1.68665
31	30MAY2007	day	4	191	1.50380
31	30MAY2007	day	4	191	1.31178
31	30MAY2007	twilight	4	191	1.33648
31	30MAY2007	night	4	191	1.71685
31	31MAY2007	night	4	191	1.89928
31	31MAY2007	twilight	4	191	0.70766
31	31MAY2007	day	4	191	1.48431
31	06JUN2007	day	6	191	1.69724
31	06JUN2007	day	6	191	1.47714
31	06JUN2007	twilight	6	191	1.10384
31	06JUN2007	night	6	191	1.49277
31	07JUN2007	night	6	191	1.30322
31	07JUN2007	twilight	6	191	1.59989
31	07JUN2007	day	6	191	1.62942
31	07JUN2007	day	6	191	1.26010
31	11JUN2007	day	8	191	1.20954
31	11JUN2007	day	8	191	1.79240
31	11JUN2007	day	8	191	1.87853
31	11JUN2007	twilight	8	191	1.62119
31	11JUN2007	night	8	191	1.75740
31	12JUN2007	night	8	191	1.35985
31	12JUN2007	twilight	8	191	1.14616
31	12JUN2007	day	8	191	1.31808
31	12JUN2007	day	8	191	1.23555
31	12JUN2007	day	8	191	1.23302
31	12JUN2007	day	8	191	1.19036

31	12JUN2007	twilight	8	191	2.81225
31	12JUN2007	night	8	191	1.40826
31	13JUN2007	night	8	191	2.81757
31	13JUN2007	twilight	8	191	1.26484
31	13JUN2007	day	8	191	1.66840
31	18JUN2007	day	2	191	1.13675
31	18JUN2007	day	2	191	1.89321
31	18JUN2007	day	2	191	1.76418
31	18JUN2007	twilight	2	191	2.04922
31	18JUN2007	night	2	191	1.02123
31	19JUN2007	night	2	191	1.02123
31	19JUN2007	twilight	2	191	1.39969
31	19JUN2007	day	2	191	0.49568
31	19JUN2007	day	2	191	1.74430
31	19JUN2007	day	2	191	1.62429
31	19JUN2007	day	2	191	2.00000
31	19JUN2007	twilight	2	191	2.78104
31	19JUN2007	night	2	191	2.90795
31	20JUN2007	night	2	191	0.96004
31	20JUN2007	twilight	2	191	2.90417
31	20JUN2007	day	2	191	2.84073
31	25JUN2007	day	4	191	0.73488
31	25JUN2007	day	4	191	1.16735
31	25JUN2007	day	4	191	1.20685
31	25JUN2007	twilight	4	191	0.68494
31	25JUN2007	night	4	191	0.51601
31	26JUN2007	night	4	191	0.54419
31	26JUN2007	twilight	4	191	0.58331
31	26JUN2007	day	4	191	0.57299
31	26JUN2007	day	4	191	0.80079
31	26JUN2007	day	4	191	1.09694
31	26JUN2007	day	4	191	0.89159
31	26JUN2007	twilight	4	191	0.89823
31	26JUN2007	night	4	191	0.87743
31	27JUN2007	night	4	191	0.45194
31	27JUN2007	twilight	4	191	0.14953
31	27JUN2007	day	4	191	0.27898
32	30MAY2007	day	4	194	1.12061
32	30MAY2007	day	4	194	1.04926
32	30MAY2007	day	4	194	1.15839
32	30MAY2007	twilight	4	194	0.65906
32	30MAY2007	night	4	194	1.57635
32	31MAY2007	night	4	194	1.72836
32	31MAY2007	twilight	4	194	2.31597
32	31MAY2007	day	4	194	2.39445
32	06JUN2007	day	6	194	1.40656
32	06JUN2007	day	6	194	0.93455
32	06JUN2007	twilight	6	194	0.94944
32	06JUN2007	night	6	194	0.82223
32	07JUN2007	night	6	194	0.78326
32	07JUN2007	twilight	6	194	2.31807
32	07JUN2007	day	6	194	2.32015
32	07JUN2007	day	6	194	1.27648
32	11JUN2007	day	8	194	1.43138
32	11JUN2007	day	8	194	1.41997
32	11JUN2007	day	8	194	1.68754

32	11JUN2007	twilight	8	194	1.48431
32	11JUN2007	night	8	194	1.24800
32	12JUN2007	night	8	194	0.50529
32	12JUN2007	twilight	8	194	1.35604
32	12JUN2007	day	8	194	1.34244
32	12JUN2007	day	8	194	1.43935
32	12JUN2007	day	8	194	1.21487
32	12JUN2007	day	8	194	1.21487
32	12JUN2007	twilight	8	194	1.68754
32	12JUN2007	night	8	194	1.48431
32	13JUN2007	night	8	194	1.24800
32	13JUN2007	twilight	8	194	0.50529
32	13JUN2007	day	8	194	0.93252
32	18JUN2007	day	2	194	0.70079
32	18JUN2007	day	2	194	0.65619
32	18JUN2007	day	2	194	1.42977
32	18JUN2007	twilight	2	194	1.43299
32	18JUN2007	night	2	194	1.47858
32	19JUN2007	night	2	194	1.05312
32	19JUN2007	twilight	2	194	1.32224
32	19JUN2007	day	2	194	1.04926
32	19JUN2007	day	2	194	0.78111
32	19JUN2007	day	2	194	1.37477
32	19JUN2007	day	2	194	1.43458
32	19JUN2007	twilight	2	194	1.48002
32	19JUN2007	night	2	194	2.16137
32	20JUN2007	night	2	194	2.04922
32	20JUN2007	twilight	2	194	1.97727
32	20JUN2007	day	2	194	1.97819
32	25JUN2007	day	4	194	1.57520
32	25JUN2007	day	4	194	1.35795
32	25JUN2007	day	4	194	1.10041
32	25JUN2007	twilight	4	194	0.98994
32	25JUN2007	night	4	194	0.63256
32	26JUN2007	night	4	194	1.27186
32	26JUN2007	twilight	4	194	0.87163
32	26JUN2007	day	4	194	1.08640
32	26JUN2007	day	4	194	0.26505
32	26JUN2007	day	4	194	0.20710
32	26JUN2007	day	4	194	1.05694
32	26JUN2007	twilight	4	194	1.00004
32	26JUN2007	night	4	194	1.35413
32	27JUN2007	night	4	194	1.52764
32	27JUN2007	twilight	4	194	0.84763
32	27JUN2007	day	4	194	1.36175
33	30MAY2007	day	4	162	1.69637
33	30MAY2007	day	4	162	1.77012
33	30MAY2007	day	4	162	1.00864
33	30MAY2007	twilight	4	162	1.18868
33	30MAY2007	night	4	162	1.29669
33	31MAY2007	night	4	162	1.24306
33	31MAY2007	twilight	4	162	0.91174
33	31MAY2007	day	4	162	0.45194
33	06JUN2007	day	6	162	1.71518
33	06JUN2007	day	6	162	1.75512
33	06JUN2007	twilight	6	162	0.89326

33	06JUN2007	night	6	162	1.68932
33	07JUN2007	night	6	162	1.26247
33	07JUN2007	twilight	6	162	0.65234
33	07JUN2007	day	6	162	1.24800
33	07JUN2007	day	6	162	1.10041
33	11JUN2007	day	8	162	1.87507
33	11JUN2007	day	8	162	1.88082
33	11JUN2007	day	8	162	1.33043
33	11JUN2007	twilight	8	162	0.54790
33	11JUN2007	night	8	162	1.46091
33	12JUN2007	night	8	162	1.40142
33	12JUN2007	twilight	8	162	1.32636
33	12JUN2007	day	8	162	1.22013
33	12JUN2007	day	8	162	1.22013
33	12JUN2007	day	8	162	0.60541
33	12JUN2007	day	8	162	1.06074
33	12JUN2007	twilight	8	162	0.48302
33	12JUN2007	night	8	162	0.42991
33	13JUN2007	night	8	162	1.23807
33	13JUN2007	twilight	8	162	1.27186
33	13JUN2007	day	8	162	0.98726
33	18JUN2007	day	2	162	1.12388
33	18JUN2007	day	2	162	0.75289
33	18JUN2007	day	2	162	1.06450
33	18JUN2007	twilight	2	162	1.91276
33	18JUN2007	night	2	162	1.94201
33	19JUN2007	night	2	162	0.91913
33	19JUN2007	twilight	2	162	1.22534
33	19JUN2007	day	2	162	1.41832
33	19JUN2007	day	2	162	0.70851
33	19JUN2007	day	2	162	0.98277
33	19JUN2007	day	2	162	1.67395
33	19JUN2007	twilight	2	162	0.85315
33	19JUN2007	night	2	162	1.56111
33	20JUN2007	night	2	162	1.55390
33	20JUN2007	twilight	2	162	2.02531
33	20JUN2007	day	2	162	2.00433
33	25JUN2007	day	4	162	0.98457
33	25JUN2007	day	4	162	0.63558
33	25JUN2007	day	4	162	0.95381
33	25JUN2007	twilight	4	162	0.95670
33	25JUN2007	night	4	162	0.72024
33	26JUN2007	night	4	162	0.99436
33	26JUN2007	twilight	4	162	1.10041
33	26JUN2007	day	4	162	1.24057
33	26JUN2007	day	4	162	0.63458
33	26JUN2007	day	4	162	0.76425
33	26JUN2007	day	4	162	0.34064
33	26JUN2007	twilight	4	162	0.76125
33	26JUN2007	night	4	162	0.59890
33	27JUN2007	night	4	162	0.34459
33	27JUN2007	twilight	4	162	0.34459
33	27JUN2007	day	4	162	0.88144
33	16MAY2011	day	5	30	1.00004
33	16MAY2011	day	5	30	0.30125
33	16MAY2011	day	5	30	0.47727

33	16MAY2011	twilight	5	30	1.00004
33	16MAY2011	night	5	30	1.04143
33	16MAY2011	night	5	30	1.00004
33	17MAY2011	twilight	5	30	0.60217
33	17MAY2011	day	5	30	1.04143
33	17MAY2011	day	5	30	1.54408
33	17MAY2011	day	5	30	0.77822
33	17MAY2011	day	5	30	1.44717
33	17MAY2011	twilight	5	30	0.30125
33	17MAY2011	night	5	30	0.95429
33	17MAY2011	night	5	30	1.04143
33	18MAY2011	twilight	5	30	1.17612
33	18MAY2011	day	5	30	-3.00000
33	25MAY2011	day	7	30	-3.00000
33	25MAY2011	day	7	30	0.30125
33	25MAY2011	day	7	30	0.47727
33	25MAY2011	twilight	7	30	0.84516
33	25MAY2011	night	7	30	0.77822
33	25MAY2011	night	7	30	0.77822
33	26MAY2011	twilight	7	30	1.04143
33	26MAY2011	day	7	30	-3.00000
33	26MAY2011	day	7	30	-3.00000
33	26MAY2011	day	7	30	0.69906
33	26MAY2011	day	7	30	0.60217
33	26MAY2011	twilight	7	30	1.04143
33	26MAY2011	night	8	30	0.30125
33	27MAY2011	twilight	8	30	0.69906
33	27MAY2011	day	8	30	1.07922
33	01JUN2011	day	1	30	0.84516
33	01JUN2011	day	1	30	0.84516
33	01JUN2011	day	1	30	1.39796
33	01JUN2011	twilight	1	30	1.34244
33	01JUN2011	night	1	30	0.95429
33	01JUN2011	night	1	30	1.04143
33	02JUN2011	twilight	1	30	0.30125
33	02JUN2011	day	1	30	0.00043
33	02JUN2011	day	1	30	0.84516
33	02JUN2011	day	1	30	1.46241
33	02JUN2011	day	1	30	1.11398
33	02JUN2011	twilight	1	30	1.41499
33	02JUN2011	night	2	30	1.47714
33	03JUN2011	twilight	2	30	1.00004
33	03JUN2011	day	2	30	1.17612
33	06JUN2011	day	2	30	0.84516
33	06JUN2011	day	2	30	0.47727
33	06JUN2011	day	2	30	1.14616
33	06JUN2011	twilight	2	30	0.95429
33	06JUN2011	night	3	30	0.60217
33	06JUN2011	night	3	30	0.84516
33	07JUN2011	twilight	3	30	1.00004
33	07JUN2011	day	3	30	0.30125
34	30MAY2007	day	4	173	1.38562
34	30MAY2007	day	4	173	0.92122
34	30MAY2007	day	4	173	1.25044
34	30MAY2007	twilight	4	173	1.56230
34	30MAY2007	night	4	173	2.04140

34	31MAY2007	night	4	173	1.02535
34	31MAY2007	twilight	4	173	1.66087
34	31MAY2007	day	4	173	1.48289
34	06JUN2007	day	6	173	1.76118
34	06JUN2007	day	6	173	0.92588
34	06JUN2007	twilight	6	173	1.26010
34	06JUN2007	night	6	173	1.26247
34	07JUN2007	night	6	173	1.64148
34	07JUN2007	twilight	6	173	1.24800
34	07JUN2007	day	6	173	1.06074
34	07JUN2007	day	6	173	0.72599
34	11JUN2007	day	8	173	1.07922
34	11JUN2007	day	8	173	1.06822
34	11JUN2007	day	8	173	0.46553
34	11JUN2007	twilight	8	173	1.35220
34	11JUN2007	night	8	173	0.84948
34	12JUN2007	night	8	173	1.33447
34	12JUN2007	twilight	8	173	1.01288
34	12JUN2007	day	8	173	1.26954
34	12JUN2007	day	8	173	1.23555
34	12JUN2007	day	8	173	1.66653
34	12JUN2007	day	8	173	1.03346
34	12JUN2007	twilight	8	173	1.90849
34	12JUN2007	night	8	173	0.85193
34	13JUN2007	night	8	173	1.27418
34	13JUN2007	twilight	8	173	1.72836
34	13JUN2007	day	8	173	0.84578
34	18JUN2007	day	2	173	1.27878
34	18JUN2007	day	2	173	1.18755
34	18JUN2007	day	2	173	1.17029
34	18JUN2007	twilight	2	173	0.90260
34	18JUN2007	night	2	173	1.02123
34	19JUN2007	night	2	173	1.30105
34	19JUN2007	twilight	2	173	1.51589
34	19JUN2007	day	2	173	1.00004
34	19JUN2007	day	2	173	0.68494
34	19JUN2007	day	2	173	1.85126
34	19JUN2007	day	2	173	0.83891
34	19JUN2007	twilight	2	173	0.90531
34	19JUN2007	night	2	173	1.96942
34	20JUN2007	night	2	173	2.05691
34	20JUN2007	twilight	2	173	2.16436
34	20JUN2007	day	2	173	2.16436
34	25JUN2007	day	4	173	1.21751
34	25JUN2007	day	4	173	1.26247
34	25JUN2007	day	4	173	1.15232
34	25JUN2007	twilight	4	173	0.70935
34	25JUN2007	night	4	173	0.81164
34	26JUN2007	night	4	173	0.76574
34	26JUN2007	twilight	4	173	0.72354
34	26JUN2007	day	4	173	0.73488
34	26JUN2007	day	4	173	1.00436
34	26JUN2007	day	4	173	0.66001
34	26JUN2007	day	4	173	0.63357
34	26JUN2007	twilight	4	173	0.30125
34	26JUN2007	night	4	173	0.56714

34	27JUN2007	night	4	173	0.49150
34	27JUN2007	twilight	4	173	0.35813
34	27JUN2007	day	4	173	0.85132
34	15JUN2011	day	5	47	0.84516
34	15JUN2011	day	5	47	0.60217
34	15JUN2011	day	5	47	1.00004
34	15JUN2011	twilight	5	47	-3.00000
34	15JUN2011	night	5	47	1.20415
34	15JUN2011	night	5	47	1.32224
34	16JUN2011	twilight	5	47	0.60217
34	16JUN2011	day	5	47	0.84516
34	16JUN2011	day	5	47	0.00043
34	16JUN2011	day	5	47	0.47727
34	16JUN2011	day	5	47	-3.00000
34	16JUN2011	day	5	47	0.47727
34	16JUN2011	twilight	5	47	0.84516
34	16JUN2011	night	5	47	0.00043
34	17JUN2011	twilight	5	47	1.07922
34	17JUN2011	day	5	47	1.11398
34	22JUN2011	day	7	47	0.77822
34	22JUN2011	day	7	47	-3.00000
34	22JUN2011	day	7	47	1.17612
34	22JUN2011	twilight	7	47	1.14616
34	22JUN2011	night	7	47	1.27878
34	22JUN2011	night	7	47	1.39796
34	23JUN2011	twilight	7	47	0.95429
34	23JUN2011	day	7	47	-3.00000
34	23JUN2011	day	7	47	0.30125
34	23JUN2011	day	7	47	0.69906
34	23JUN2011	day	7	47	1.30105
34	23JUN2011	twilight	7	47	1.23047
34	23JUN2011	night	7	47	1.51853
34	23JUN2011	night	7	47	1.59108
34	24JUN2011	twilight	7	47	1.25530
34	24JUN2011	day	7	47	1.07922
34	27JUN2011	day	8	47	-3.00000
34	27JUN2011	day	8	47	-3.00000
34	27JUN2011	day	8	47	0.30125
34	27JUN2011	twilight	8	47	1.04143
34	27JUN2011	night	8	47	1.14616
34	27JUN2011	night	8	47	1.23047
34	28JUN2011	twilight	8	47	1.00004
34	28JUN2011	day	8	47	0.69906
34	28JUN2011	day	8	47	0.00043
34	28JUN2011	day	8	47	-3.00000
34	28JUN2011	day	8	47	1.20415
34	28JUN2011	twilight	8	47	0.30125
34	28JUN2011	night	1	47	-3.00000
34	28JUN2011	night	1	47	0.30125
34	29JUN2011	twilight	1	47	1.04143
34	29JUN2011	day	1	47	1.04143
35	15JUN2011	day	5	50	0.30125
35	15JUN2011	day	5	50	0.47727
35	15JUN2011	day	5	50	0.00043
35	15JUN2011	twilight	5	50	0.69906
35	15JUN2011	night	5	50	0.77822

35	15JUN2011	night	5	50	0.69906
35	15JUN2011	twilight	5	50	0.47727
35	16JUN2011	day	5	50	0.47727
35	16JUN2011	day	5	50	0.69906
35	16JUN2011	day	5	50	0.84516
35	16JUN2011	day	5	50	0.84516
35	16JUN2011	twilight	5	50	0.69906
35	16JUN2011	night	5	50	0.77822
35	16JUN2011	twilight	5	50	0.47727
35	17JUN2011	day	5	50	1.11398
35	22JUN2011	day	7	50	0.95429
35	22JUN2011	day	7	50	0.60217
35	22JUN2011	day	7	50	0.69906
35	22JUN2011	twilight	7	50	0.84516
35	22JUN2011	night	7	50	0.60217
35	22JUN2011	night	7	50	0.60217
35	23JUN2011	twilight	7	50	1.11398
35	23JUN2011	day	7	50	-3.00000
35	23JUN2011	day	7	50	0.60217
35	23JUN2011	day	7	50	0.69906
35	23JUN2011	day	7	50	1.11398
35	23JUN2011	twilight	7	50	0.95429
35	23JUN2011	night	7	50	0.30125
35	23JUN2011	night	7	50	0.60217
35	24JUN2011	twilight	7	50	-3.00000
35	24JUN2011	day	7	50	0.77822
35	27JUN2011	day	8	50	0.60217
35	27JUN2011	day	8	50	0.69906
35	27JUN2011	day	8	50	0.95429
35	27JUN2011	twilight	8	50	0.77822
35	27JUN2011	night	8	50	0.77822
35	27JUN2011	night	8	50	0.60217
35	28JUN2011	twilight	8	50	0.77822
35	28JUN2011	day	8	50	0.69906
35	28JUN2011	day	8	50	0.77822
35	28JUN2011	day	8	50	0.84516
35	28JUN2011	day	8	50	0.69906
35	28JUN2011	twilight	8	50	0.47727
35	28JUN2011	night	1	50	0.84516
35	28JUN2011	night	1	50	0.47727
35	29JUN2011	twilight	1	50	1.00004
35	29JUN2011	day	1	50	0.95429
36	30MAY2007	day	4	128	1.92942
36	30MAY2007	day	4	128	1.06822
36	30MAY2007	day	4	128	1.56230
36	30MAY2007	twilight	4	128	0.98096
36	30MAY2007	night	4	128	0.90639
36	31MAY2007	night	4	128	0.77459
36	31MAY2007	twilight	4	128	1.00864
36	31MAY2007	day	4	128	0.10755
36	06JUN2007	day	6	128	1.34832
36	06JUN2007	day	6	128	0.98637
36	06JUN2007	twilight	6	128	0.09377
36	06JUN2007	night	6	128	1.32224
36	07JUN2007	night	6	128	0.68494
36	07JUN2007	twilight	6	128	0.64058

36	07JUN2007	day	6	128	1.39095
36	07JUN2007	day	6	128	0.83512
36	11JUN2007	day	8	128	1.87041
36	11JUN2007	day	8	128	1.88593
36	11JUN2007	day	8	128	1.88762
36	11JUN2007	twilight	8	128	1.12061
36	11JUN2007	night	8	128	1.80004
36	12JUN2007	night	8	128	1.74273
36	12JUN2007	twilight	8	128	1.23047
36	12JUN2007	day	8	128	1.41832
36	12JUN2007	day	8	128	1.10384
36	12JUN2007	day	8	128	1.52635
36	12JUN2007	day	8	128	1.89321
36	12JUN2007	twilight	8	128	1.29449
36	12JUN2007	night	8	128	1.88819
36	13JUN2007	night	8	128	0.80079
36	13JUN2007	twilight	8	128	0.88372
36	13JUN2007	day	8	128	1.13037
36	18JUN2007	day	2	128	0.79106
36	18JUN2007	day	2	128	0.72024
36	18JUN2007	day	2	128	1.55752
36	18JUN2007	twilight	2	128	1.82413
36	18JUN2007	night	2	128	1.28332
36	19JUN2007	night	2	128	1.08994
36	19JUN2007	twilight	2	128	0.87163
36	19JUN2007	day	2	128	0.98050
36	19JUN2007	day	2	128	0.89548
36	19JUN2007	day	2	128	0.92896
36	19JUN2007	day	2	128	1.91646
36	19JUN2007	twilight	2	128	1.92117
36	19JUN2007	night	2	128	2.83569
36	20JUN2007	night	2	128	2.22011
36	20JUN2007	twilight	2	128	2.69108
36	20JUN2007	day	2	128	1.86333
36	25JUN2007	day	4	128	0.60217
36	25JUN2007	day	4	128	0.69205
36	25JUN2007	day	4	128	0.50934
36	25JUN2007	twilight	4	128	0.58894
36	25JUN2007	night	4	128	1.12714
36	26JUN2007	night	4	128	1.06074
36	26JUN2007	twilight	4	128	1.36737
36	26JUN2007	day	4	128	0.33264
36	26JUN2007	day	4	128	0.23070
36	26JUN2007	day	4	128	1.38918
36	26JUN2007	day	4	128	1.09346
36	26JUN2007	twilight	4	128	-0.24718
36	26JUN2007	night	4	128	0.71609
36	27JUN2007	night	4	128	0.82289
36	27JUN2007	twilight	4	128	0.68314
36	27JUN2007	day	4	128	0.67770
37	30MAY2007	day	4	217	1.53404
37	30MAY2007	day	4	217	1.34637
37	30MAY2007	day	4	217	0.63659
37	30MAY2007	twilight	4	217	1.02535
37	30MAY2007	night	4	217	1.43618
37	31MAY2007	night	4	217	2.66181

37	31MAY2007	twilight	4	217	2.16137
37	31MAY2007	day	4	217	1.77452
37	06JUN2007	day	6	217	1.74742
37	06JUN2007	day	6	217	0.09026
37	06JUN2007	twilight	6	217	1.74273
37	06JUN2007	night	6	217	1.72099
37	07JUN2007	night	6	217	0.96946
37	07JUN2007	twilight	6	217	1.13675
37	07JUN2007	day	6	217	1.26484
37	07JUN2007	day	6	217	0.82679
37	11JUN2007	day	8	217	1.13037
37	11JUN2007	day	8	217	1.93044
37	11JUN2007	day	8	217	1.86806
37	11JUN2007	twilight	8	217	2.08279
37	11JUN2007	night	8	217	2.10721
37	12JUN2007	night	8	217	1.02942
37	12JUN2007	twilight	8	217	1.49970
37	12JUN2007	day	8	217	1.66087
37	12JUN2007	day	8	217	0.69028
37	12JUN2007	day	8	217	1.03346
37	12JUN2007	day	8	217	1.04536
37	12JUN2007	twilight	8	217	1.24306
37	12JUN2007	night	8	217	1.22013
37	13JUN2007	night	8	217	0.79176
37	13JUN2007	twilight	8	217	1.42815
37	13JUN2007	day	8	217	1.42815
37	18JUN2007	day	2	217	1.60207
37	18JUN2007	day	2	217	1.42653
37	18JUN2007	day	2	217	1.24554
37	18JUN2007	twilight	2	217	1.29228
37	18JUN2007	night	2	217	1.33447
37	19JUN2007	night	2	217	1.52635
37	19JUN2007	twilight	2	217	1.43299
37	19JUN2007	day	2	217	1.14925
37	19JUN2007	day	2	217	1.34832
37	19JUN2007	day	2	217	1.49694
37	19JUN2007	day	2	217	1.50107
37	19JUN2007	twilight	2	217	1.94939
37	19JUN2007	night	2	217	1.80889
37	20JUN2007	night	2	217	0.50393
37	20JUN2007	twilight	2	217	0.89271
37	20JUN2007	day	2	217	0.45194
37	25JUN2007	day	4	217	1.07922
37	25JUN2007	day	4	217	1.20142
37	25JUN2007	day	4	217	1.48856
37	25JUN2007	twilight	4	217	1.26954
37	25JUN2007	night	4	217	0.96099
37	26JUN2007	night	4	217	1.01288
37	26JUN2007	twilight	4	217	1.17322
37	26JUN2007	day	4	217	0.75059
37	26JUN2007	day	4	217	0.54913
37	26JUN2007	day	4	217	0.67034
37	26JUN2007	day	4	217	0.58218
37	26JUN2007	twilight	4	217	0.72681
37	26JUN2007	night	4	217	0.97777
37	27JUN2007	night	4	217	1.15839

37	27JUN2007	twilight	4	217	1.28558
37	27JUN2007	day	4	217	0.88144
38	30MAY2007	day	4	166	1.41332
38	30MAY2007	day	4	166	1.57404
38	30MAY2007	day	4	166	1.69898
38	30MAY2007	twilight	4	166	0.80353
38	30MAY2007	night	4	166	1.78320
38	31MAY2007	night	4	166	1.51190
38	31MAY2007	twilight	4	166	1.31808
38	31MAY2007	day	4	166	1.65897
38	06JUN2007	day	6	166	1.75740
38	06JUN2007	day	6	166	1.29669
38	06JUN2007	twilight	6	166	1.59880
38	06JUN2007	night	6	166	1.61910
38	07JUN2007	night	6	166	1.54408
38	07JUN2007	twilight	6	166	1.41997
38	07JUN2007	day	6	166	1.23807
38	07JUN2007	day	6	166	1.16735
38	11JUN2007	day	8	166	0.96619
38	11JUN2007	day	8	166	2.30750
38	11JUN2007	day	8	166	2.45939
38	11JUN2007	twilight	8	166	1.48997
38	11JUN2007	night	8	166	1.37660
38	12JUN2007	night	8	166	1.24306
38	12JUN2007	twilight	8	166	1.77816
38	12JUN2007	day	8	166	1.84510
38	12JUN2007	day	8	166	1.23047
38	12JUN2007	day	8	166	0.88655
38	12JUN2007	day	8	166	0.99348
38	12JUN2007	twilight	8	166	-0.04335
38	12JUN2007	night	8	166	1.50107
38	13JUN2007	night	8	166	1.44250
38	13JUN2007	twilight	8	166	1.60639
38	13JUN2007	day	8	166	1.03346
38	18JUN2007	day	2	166	1.16438
38	18JUN2007	day	2	166	1.59771
38	18JUN2007	day	2	166	1.61173
38	18JUN2007	twilight	2	166	1.06822
38	18JUN2007	night	2	166	1.06074
38	19JUN2007	night	2	166	1.11398
38	19JUN2007	twilight	2	166	1.08994
38	19JUN2007	day	2	166	1.25770
38	19JUN2007	day	2	166	0.78183
38	19JUN2007	day	2	166	1.61385
38	19JUN2007	day	2	166	2.65321
38	19JUN2007	twilight	2	166	2.38561
38	19JUN2007	night	2	166	2.75435
38	20JUN2007	night	2	166	0.78469
38	20JUN2007	twilight	2	166	2.74194
38	20JUN2007	day	2	166	1.07922
38	25JUN2007	day	4	166	0.15259
38	25JUN2007	day	4	166	1.40656
38	25JUN2007	day	4	166	1.35604
38	25JUN2007	twilight	4	166	1.22274
38	25JUN2007	night	4	166	0.99699
38	26JUN2007	night	4	166	0.83257

38	26JUN2007	twilight	4	166	0.80489
38	26JUN2007	day	4	166	0.53161
38	26JUN2007	day	4	166	0.45954
38	26JUN2007	day	4	166	0.79316
38	26JUN2007	day	4	166	0.89271
38	26JUN2007	twilight	4	166	0.97777
38	26JUN2007	night	4	166	-0.00043
38	27JUN2007	night	4	166	0.72272
38	27JUN2007	twilight	4	166	0.74044
38	27JUN2007	day	4	166	0.26741
39	30MAY2007	day	4	83	0.74123
39	30MAY2007	day	4	83	1.22534
39	30MAY2007	day	4	83	2.06819
39	30MAY2007	twilight	4	83	1.76716
39	30MAY2007	night	4	83	1.76716
39	31MAY2007	night	4	83	0.99304
39	31MAY2007	twilight	4	83	1.84510
39	31MAY2007	day	4	83	1.55631
39	06JUN2007	day	6	83	1.09694
39	06JUN2007	day	6	83	1.00004
39	06JUN2007	twilight	6	83	1.68575
39	06JUN2007	night	6	83	1.47714
39	07JUN2007	night	6	83	1.69985
39	07JUN2007	twilight	6	83	1.44562
39	07JUN2007	day	6	83	1.79029
39	07JUN2007	day	6	83	1.11398
39	11JUN2007	day	8	83	1.30105
39	11JUN2007	day	8	83	1.28106
39	11JUN2007	day	8	83	1.71013
39	11JUN2007	twilight	8	83	1.13675
39	11JUN2007	night	8	83	0.77967
39	12JUN2007	night	8	83	-3.00000
39	12JUN2007	twilight	8	83	-0.14752
39	12JUN2007	day	8	83	0.94846
39	12JUN2007	day	8	83	0.91387
39	12JUN2007	day	8	83	1.59661
39	12JUN2007	day	8	83	1.36551
39	12JUN2007	twilight	8	83	1.56468
39	12JUN2007	night	8	83	1.94399
39	13JUN2007	night	8	83	0.20439
39	13JUN2007	twilight	8	83	0.80625
39	13JUN2007	day	8	83	2.00433
39	18JUN2007	day	2	83	0.94846
39	18JUN2007	day	2	83	0.56241
39	18JUN2007	day	2	83	1.48997
39	18JUN2007	twilight	2	83	2.20412
39	18JUN2007	night	2	83	2.13354
39	19JUN2007	night	2	83	1.26954
39	19JUN2007	twilight	2	83	0.97777
39	19JUN2007	day	2	83	1.08282
39	19JUN2007	day	2	83	0.32243
39	19JUN2007	day	2	83	1.37477
39	19JUN2007	day	2	83	1.21751
39	19JUN2007	twilight	2	83	1.16140
39	19JUN2007	night	2	83	1.36551
39	20JUN2007	night	2	83	0.32654

39	20JUN2007	twilight	2	83	0.40841
39	20JUN2007	day	2	83	1.57055
39	25JUN2007	day	4	83	1.55268
39	25JUN2007	day	4	83	1.38741
39	25JUN2007	day	4	83	0.99568
39	25JUN2007	twilight	4	83	0.83512
39	25JUN2007	night	4	83	0.49429
39	26JUN2007	night	4	83	1.20415
39	26JUN2007	twilight	4	83	0.97823
39	26JUN2007	day	4	83	0.25551
39	26JUN2007	day	4	83	0.51601
39	26JUN2007	day	4	83	1.06822
39	26JUN2007	day	4	83	0.84080
39	26JUN2007	twilight	4	83	0.99568
39	26JUN2007	night	4	83	0.95284
39	27JUN2007	night	4	83	0.40329
39	27JUN2007	twilight	4	83	0.68224
39	27JUN2007	day	4	83	0.75823
46	15JUN2011	day	5	118	-3.00000
46	15JUN2011	day	5	118	-3.00000
46	15JUN2011	day	5	118	1.50516
46	15JUN2011	twilight	5	118	1.50516
46	15JUN2011	night	5	118	2.11059
46	15JUN2011	night	5	118	1.43138
46	15JUN2011	twilight	5	118	1.92428
46	16JUN2011	day	5	118	1.25530
46	16JUN2011	day	5	118	1.00004
46	16JUN2011	day	5	118	-3.00000
46	16JUN2011	day	5	118	0.84516
46	16JUN2011	day	5	118	0.47727
46	16JUN2011	day	5	118	1.92428
46	16JUN2011	night	5	118	1.11398
46	16JUN2011	twilight	5	118	1.77086
46	17JUN2011	day	5	118	1.20415
46	22JUN2011	day	7	118	-3.00000
46	22JUN2011	day	7	118	1.75588
46	22JUN2011	day	7	118	1.75588
46	22JUN2011	twilight	7	118	1.07922
46	22JUN2011	night	7	118	1.53149
46	22JUN2011	night	7	118	1.55631
46	23JUN2011	twilight	7	118	1.54408
46	23JUN2011	day	7	118	1.54408
46	23JUN2011	day	7	118	0.69906
46	23JUN2011	day	7	118	0.69906
46	23JUN2011	day	7	118	1.14616
46	23JUN2011	twilight	7	118	1.14616
46	23JUN2011	night	7	118	0.47727
46	23JUN2011	night	7	118	0.69906
46	24JUN2011	twilight	7	118	1.00004
46	24JUN2011	day	7	118	0.47727
46	27JUN2011	day	8	118	0.90314
46	27JUN2011	day	8	118	-3.00000
46	27JUN2011	day	8	118	0.77822
46	27JUN2011	twilight	8	118	0.69906
46	27JUN2011	night	8	118	1.98228
46	27JUN2011	night	8	118	1.30105

46	28JUN2011	twilight	8	118	1.75588
46	28JUN2011	day	8	118	1.27878
46	28JUN2011	day	8	118	0.77822
46	28JUN2011	day	8	118	0.47727
46	28JUN2011	day	8	118	0.84516
46	28JUN2011	twilight	8	118	0.69906
46	28JUN2011	night	1	118	1.46241
46	28JUN2011	night	1	118	1.51853
46	29JUN2011	twilight	1	118	1.34244
46	29JUN2011	day	1	118	1.25530
48	15JUN2011	day	5	81	0.77822
48	15JUN2011	day	5	81	-3.00000
48	15JUN2011	day	5	81	0.60217
48	15JUN2011	twilight	5	81	1.04143
48	15JUN2011	night	5	81	1.04143
48	15JUN2011	night	5	81	-3.00000
48	15JUN2011	twilight	5	81	1.27878
48	16JUN2011	day	5	81	0.00043
48	16JUN2011	day	5	81	1.25530
48	16JUN2011	day	5	81	0.30125
48	16JUN2011	day	5	81	0.69906
48	16JUN2011	twilight	5	81	0.47727
48	16JUN2011	night	5	81	1.04143
48	16JUN2011	twilight	5	81	0.90314
48	17JUN2011	day	5	81	0.60217
48	22JUN2011	day	7	81	1.30105
48	22JUN2011	day	7	81	0.60217
48	22JUN2011	day	7	81	0.69906
48	22JUN2011	twilight	7	81	0.47727
48	22JUN2011	night	7	81	1.55631
48	22JUN2011	night	7	81	1.55631
48	23JUN2011	twilight	7	81	0.69906
48	23JUN2011	day	7	81	0.30125
48	23JUN2011	day	7	81	0.84516
48	23JUN2011	day	7	81	0.60217
48	23JUN2011	day	7	81	1.41499
48	23JUN2011	twilight	7	81	1.43138
48	23JUN2011	night	7	81	1.11398
48	23JUN2011	night	7	81	1.11398
48	24JUN2011	twilight	7	81	0.47727
48	24JUN2011	day	7	81	0.60217
48	27JUN2011	day	8	81	1.00004
48	27JUN2011	day	8	81	-3.00000
48	27JUN2011	day	8	81	0.60217
48	27JUN2011	twilight	8	81	0.84516
48	27JUN2011	night	8	81	0.30125
48	27JUN2011	night	8	81	0.60217
48	28JUN2011	twilight	8	81	0.77822
48	28JUN2011	day	8	81	0.69906
48	28JUN2011	day	8	81	-3.00000
48	28JUN2011	day	8	81	0.00043
48	28JUN2011	day	8	81	1.34244
48	28JUN2011	twilight	8	81	0.69906
48	28JUN2011	night	1	81	-3.00000
48	28JUN2011	night	1	81	1.38023
48	29JUN2011	twilight	1	81	1.34244

48	29JUN2011	day	1	81	0.60217
50	04JUN2008	day	1	20	0.39811
50	04JUN2008	day	1	20	1.23555
50	04JUN2008	day	1	20	1.16438
50	04JUN2008	twilight	1	20	0.88315
50	04JUN2008	night	1	20	0.64943
50	05JUN2008	night	1	20	0.81431
50	05JUN2008	twilight	1	20	0.90423
50	05JUN2008	day	1	20	1.37477
50	05JUN2008	day	1	20	1.07922
50	05JUN2008	day	1	20	1.11062
50	05JUN2008	day	1	20	1.32017
50	05JUN2008	twilight	1	20	1.02942
50	05JUN2008	night	1	20	1.28558
50	06JUN2008	night	2	20	1.46836
50	06JUN2008	twilight	2	20	1.17901
50	06JUN2008	day	2	20	0.93707
50	11JUN2008	day	3	20	1.18755
50	11JUN2008	day	3	20	2.21749
50	11JUN2008	day	3	20	2.19590
50	11JUN2008	twilight	3	20	2.08279
50	11JUN2008	night	3	20	1.99826
50	12JUN2008	night	3	20	2.05691
50	12JUN2008	twilight	3	20	2.08636
50	12JUN2008	day	3	20	2.17898
50	12JUN2008	day	3	20	1.85004
50	12JUN2008	day	3	20	1.37293
50	12JUN2008	twilight	3	20	1.39622
50	12JUN2008	night	3	20	1.30965
50	12JUN2008	night	3	20	1.98945
50	13JUN2008	twilight	3	20	1.20415
50	13JUN2008	day	3	20	1.02942
50	13JUN2008	day	3	20	1.41332
50	18JUN2008	day	5	20	1.24554
50	18JUN2008	day	5	20	0.79246
50	18JUN2008	day	5	20	0.79941
50	18JUN2008	twilight	5	20	1.05694
50	18JUN2008	night	5	20	0.77822
50	19JUN2008	night	6	20	0.45652
50	19JUN2008	twilight	6	20	1.06822
50	19JUN2008	day	6	20	1.13991
50	19JUN2008	day	6	20	0.84948
50	19JUN2008	day	6	20	0.83321
50	19JUN2008	day	6	20	0.26505
50	19JUN2008	twilight	6	20	0.74515
50	19JUN2008	night	6	20	1.04926
50	20JUN2008	night	6	20	0.48444
50	20JUN2008	twilight	6	20	0.99392
50	20JUN2008	day	6	20	0.30125
50	25JUN2008	day	7	20	0.48444
50	25JUN2008	day	7	20	0.58218
50	25JUN2008	day	7	20	0.95381
50	25JUN2008	twilight	7	20	0.55400
50	25JUN2008	night	7	20	0.89326
50	26JUN2008	night	7	20	0.53161
50	26JUN2008	twilight	7	20	0.53288

50	26JUN2008	day	7	20	0.34850
50	26JUN2008	day	7	20	0.75366
50	26JUN2008	day	7	20	-3.00000
50	26JUN2008	day	7	20	0.73568
50	26JUN2008	day	7	20	0.47727
50	26JUN2008	night	7	20	0.38757
50	27JUN2008	night	7	20	-0.14206
50	27JUN2008	day	7	20	-0.15428
50	27JUN2008	day	7	20	0.67587
51	04JUN2008	day	1	22	0.44264
51	04JUN2008	day	1	22	1.04143
51	04JUN2008	day	1	22	0.85739
51	04JUN2008	twilight	1	22	1.51721
51	04JUN2008	night	1	22	1.73800
51	05JUN2008	night	1	22	1.06450
51	05JUN2008	twilight	1	22	0.63357
51	05JUN2008	day	1	22	1.78959
51	05JUN2008	day	1	22	0.66001
51	05JUN2008	day	1	22	1.69637
51	05JUN2008	day	1	22	0.94354
51	05JUN2008	twilight	1	22	0.89548
51	05JUN2008	night	1	22	1.26954
51	06JUN2008	night	2	22	1.40656
51	06JUN2008	twilight	2	22	1.17901
51	06JUN2008	day	2	22	1.17901
51	11JUN2008	day	3	22	1.55146
51	11JUN2008	day	3	22	1.74820
51	11JUN2008	day	3	22	1.54034
51	11JUN2008	twilight	3	22	1.62222
51	11JUN2008	night	3	22	1.83759
51	12JUN2008	night	3	22	1.84386
51	12JUN2008	twilight	3	22	1.92221
51	12JUN2008	day	3	22	1.75664
51	12JUN2008	day	3	22	1.35795
51	12JUN2008	day	3	22	0.46553
51	12JUN2008	twilight	3	22	0.74749
51	12JUN2008	night	3	22	0.40500
51	12JUN2008	night	3	22	1.83443
51	13JUN2008	twilight	3	22	1.23047
51	13JUN2008	day	3	22	0.99699
51	13JUN2008	day	3	22	1.56230
51	18JUN2008	day	5	22	1.37109
51	18JUN2008	day	5	22	-0.19518
51	18JUN2008	day	5	22	0.80489
51	18JUN2008	twilight	5	22	0.00903
51	18JUN2008	night	5	22	1.23807
51	19JUN2008	night	6	22	0.93202
51	19JUN2008	twilight	6	22	0.75823
51	19JUN2008	day	6	22	1.04536
51	19JUN2008	day	6	22	0.73167
51	19JUN2008	day	6	22	0.58104
51	19JUN2008	day	6	22	0.66661
51	19JUN2008	twilight	6	22	0.67219
51	19JUN2008	night	6	22	1.13357
51	20JUN2008	night	6	22	-0.22330
51	20JUN2008	twilight	6	22	1.11730

51	20JUN2008	day	6	22	0.41847
51	25JUN2008	day	7	22	0.92433
51	25JUN2008	day	7	22	0.99304
51	25JUN2008	day	7	22	0.31408
51	25JUN2008	twilight	7	22	0.10415
51	25JUN2008	night	7	22	1.33648
51	26JUN2008	night	7	22	0.84516
51	26JUN2008	twilight	7	22	0.86338
51	26JUN2008	day	7	22	0.70766
51	26JUN2008	day	7	22	0.40329
51	26JUN2008	day	7	22	0.75366
51	26JUN2008	day	7	22	0.96194
51	26JUN2008	twilight	7	22	0.31408
51	26JUN2008	night	7	22	-3.00000
51	27JUN2008	night	7	22	-3.00000
51	27JUN2008	twilight	7	22	-3.00000
51	27JUN2008	day	7	22	0.16167
51	16MAY2011	day	5	141	1.11398
51	16MAY2011	day	5	141	-3.00000
51	16MAY2011	day	5	141	1.46241
51	16MAY2011	twilight	5	141	1.77086
51	16MAY2011	night	5	141	0.60217
51	16MAY2011	night	5	141	1.07922
51	17MAY2011	twilight	5	141	1.11398
51	17MAY2011	day	5	141	0.47727
51	17MAY2011	day	6	141	1.76344
51	17MAY2011	day	6	141	1.04143
51	17MAY2011	day	6	141	1.60207
51	17MAY2011	twilight	6	141	1.73240
51	17MAY2011	night	6	141	0.77822
51	17MAY2011	night	6	141	0.69906
51	18MAY2011	twilight	6	141	1.71601
51	18MAY2011	day	6	141	1.76344
51	25MAY2011	day	7	141	1.44717
51	25MAY2011	day	7	141	1.38023
51	25MAY2011	day	7	141	0.95429
51	25MAY2011	day	7	141	1.17612
51	25MAY2011	twilight	7	141	1.56821
51	25MAY2011	night	7	141	1.32224
51	26MAY2011	twilight	7	141	1.86924
51	26MAY2011	day	7	141	0.90314
51	26MAY2011	day	7	141	1.76344
51	26MAY2011	day	7	141	1.25530
51	26MAY2011	day	7	141	0.84516
51	26MAY2011	twilight	7	141	1.11398
51	26MAY2011	night	7	141	1.23047
51	27MAY2011	twilight	8	141	1.38023
51	27MAY2011	day	8	141	0.47727
51	01JUN2011	day	1	141	1.25530
51	01JUN2011	day	1	141	0.95429
51	01JUN2011	day	1	141	1.11398
51	01JUN2011	twilight	1	141	1.85734
51	01JUN2011	night	1	141	2.00000
51	01JUN2011	night	1	141	1.57980
51	02JUN2011	twilight	1	141	1.39796
51	02JUN2011	day	1	141	1.32224

51	02JUN2011	day	1	141	1.69020
51	02JUN2011	day	1	141	1.82608
51	02JUN2011	day	1	141	1.25530
51	02JUN2011	twilight	1	141	1.07922
51	02JUN2011	night	1	141	0.90314
51	02JUN2011	night	2	141	0.77822
51	03JUN2011	twilight	2	141	1.14616
51	03JUN2011	day	2	141	0.47727
51	06JUN2011	day	2	141	0.47727
51	06JUN2011	day	2	141	1.23047
51	06JUN2011	day	2	141	1.87507
51	06JUN2011	twilight	2	141	1.54408
51	06JUN2011	night	3	141	1.91382
51	06JUN2011	night	3	141	1.11398
51	07JUN2011	twilight	3	141	1.17612
51	07JUN2011	day	3	141	1.34244
52	16MAY2011	day	5	147	0.95429
52	16MAY2011	day	5	147	1.20415
52	16MAY2011	day	5	147	0.47727
52	16MAY2011	twilight	5	147	1.04143
52	16MAY2011	night	5	147	1.23047
52	16MAY2011	night	5	147	0.60217
52	17MAY2011	twilight	5	147	-3.00000
52	17MAY2011	day	5	147	1.14616
52	17MAY2011	day	6	147	1.00004
52	17MAY2011	day	6	147	1.11398
52	17MAY2011	day	6	147	0.84516
52	17MAY2011	twilight	6	147	-3.00000
52	17MAY2011	night	6	147	1.38023
52	17MAY2011	night	6	147	1.89763
52	18MAY2011	twilight	6	147	1.32224
52	18MAY2011	day	6	147	1.83252
52	25MAY2011	day	7	147	0.30125
52	25MAY2011	day	7	147	1.38023
52	25MAY2011	day	7	147	1.41499
52	25MAY2011	day	7	147	0.69906
52	25MAY2011	twilight	7	147	1.34244
52	25MAY2011	night	7	147	0.90314
52	26MAY2011	twilight	7	147	1.30105
52	26MAY2011	day	7	147	0.30125
52	26MAY2011	day	7	147	1.17612
52	26MAY2011	day	7	147	1.98228
52	26MAY2011	day	7	147	1.99123
52	26MAY2011	twilight	7	147	0.84516
52	26MAY2011	night	7	147	1.14616
52	26MAY2011	night	7	147	1.30105
52	27MAY2011	twilight	8	147	1.39796
52	27MAY2011	day	8	147	0.90314
52	01JUN2011	day	1	147	0.95429
52	01JUN2011	day	1	147	0.77822
52	01JUN2011	twilight	1	147	1.74820
52	01JUN2011	night	1	147	1.79240
52	01JUN2011	night	1	147	1.30105
52	02JUN2011	twilight	1	147	1.87507
52	02JUN2011	day	1	147	1.62326
52	02JUN2011	day	1	147	1.77086

52	02JUN2011	twilight	1	147	1.69898
52	02JUN2011	night	1	147	1.77086
52	02JUN2011	night	2	147	1.49138
52	03JUN2011	twilight	2	147	0.95429
52	03JUN2011	day	2	147	1.11398
52	06JUN2011	day	2	147	1.39796
52	06JUN2011	day	2	147	0.95429
52	06JUN2011	day	2	147	1.00004
52	06JUN2011	twilight	2	147	1.20415
52	06JUN2011	night	3	147	1.17612
52	06JUN2011	night	3	147	0.69906
52	07JUN2011	twilight	3	147	0.69906
52	07JUN2011	day	3	147	0.84516
53	16MAY2011	day	5	152	0.60217
53	16MAY2011	day	5	152	0.30125
53	16MAY2011	day	5	152	-3.00000
53	16MAY2011	twilight	5	152	1.51853
53	16MAY2011	night	5	152	1.00004
53	16MAY2011	night	5	152	0.95429
53	17MAY2011	twilight	5	152	1.70758
53	17MAY2011	day	5	152	0.77822
53	17MAY2011	day	6	152	1.72428
53	17MAY2011	day	6	152	1.32224
53	17MAY2011	day	6	152	1.93450
53	17MAY2011	twilight	6	152	1.80619
53	17MAY2011	night	6	152	1.27878
53	17MAY2011	night	6	152	0.30125
53	18MAY2011	twilight	6	152	1.41499
53	18MAY2011	day	6	152	1.32224
53	25MAY2011	day	7	152	1.77086
53	25MAY2011	day	7	152	0.95429
53	25MAY2011	day	7	152	-3.00000
53	25MAY2011	twilight	7	152	1.41499
53	25MAY2011	night	7	152	1.25530
53	26MAY2011	twilight	7	152	1.27878
53	26MAY2011	day	7	152	0.95429
53	26MAY2011	day	7	152	1.23047
53	26MAY2011	day	7	152	1.30105
53	26MAY2011	day	7	152	1.04143
53	26MAY2011	twilight	7	152	1.27878
53	26MAY2011	night	7	152	1.27878
53	26MAY2011	night	7	152	0.60217
53	27MAY2011	twilight	8	152	0.84516
53	27MAY2011	day	8	152	1.00004
53	01JUN2011	day	1	152	1.83886
53	01JUN2011	day	1	152	-3.00000
53	01JUN2011	day	1	152	1.00004
53	01JUN2011	twilight	1	152	0.47727
53	01JUN2011	night	1	152	0.47727
53	01JUN2011	night	1	152	0.69906
53	02JUN2011	twilight	1	152	1.30105
53	02JUN2011	day	1	152	0.60217
53	02JUN2011	day	1	152	1.27878
53	02JUN2011	day	1	152	0.95429
53	02JUN2011	twilight	1	152	1.07922
53	02JUN2011	night	1	152	1.27878

53	02JUN2011	night	2	152	-3.00000
53	03JUN2011	twilight	2	152	1.04143
53	03JUN2011	day	2	152	1.00004
53	06JUN2011	day	2	152	1.30105
53	06JUN2011	day	2	152	1.65322
53	06JUN2011	day	2	152	0.47727
53	06JUN2011	twilight	2	152	1.00004
53	06JUN2011	night	3	152	1.69898
53	06JUN2011	night	3	152	-3.00000
53	07JUN2011	twilight	3	152	1.61279
53	07JUN2011	day	3	152	1.00004
54	16MAY2011	day	5	199	1.20415
54	16MAY2011	day	5	199	0.00043
54	16MAY2011	day	5	199	1.32224
54	16MAY2011	twilight	5	199	0.90314
54	16MAY2011	night	5	199	1.69020
54	16MAY2011	night	5	199	1.23047
54	17MAY2011	twilight	5	199	0.90314
54	17MAY2011	day	5	199	0.30125
54	17MAY2011	day	6	199	1.56821
54	17MAY2011	day	6	199	1.07922
54	17MAY2011	day	6	199	1.60207
54	17MAY2011	twilight	6	199	0.95429
54	17MAY2011	night	6	199	1.67211
54	17MAY2011	night	6	199	1.54408
54	18MAY2011	twilight	6	199	1.92428
54	18MAY2011	day	6	199	1.73240
55	04JUN2008	day	1	88	0.16465
55	04JUN2008	day	1	88	0.60433
55	04JUN2008	day	1	88	0.45803
55	04JUN2008	twilight	1	88	0.71609
55	04JUN2008	night	1	88	0.50120
55	05JUN2008	night	1	88	0.79316
55	05JUN2008	twilight	1	88	1.00864
55	05JUN2008	day	1	88	0.60756
55	05JUN2008	day	1	88	0.63155
55	05JUN2008	day	1	88	-0.19382
55	05JUN2008	day	1	88	0.08672
55	05JUN2008	twilight	1	88	0.37310
55	05JUN2008	night	1	88	0.33465
55	06JUN2008	night	2	88	0.33264
55	06JUN2008	twilight	2	88	0.44886
55	06JUN2008	day	2	88	0.14644
55	11JUN2008	day	3	88	0.32654
55	11JUN2008	day	3	88	0.26269
55	11JUN2008	day	3	88	1.39095
55	11JUN2008	twilight	3	88	1.46540
55	11JUN2008	night	3	88	0.97914
55	12JUN2008	night	3	88	0.92330
55	12JUN2008	twilight	3	88	-0.17457
55	12JUN2008	day	3	88	0.10755
55	12JUN2008	day	3	88	0.10755
55	12JUN2008	day	3	88	-3.00000
55	12JUN2008	twilight	3	88	1.71518
55	12JUN2008	night	3	88	0.79525
55	12JUN2008	night	4	88	1.77379

55	13JUN2008	twilight	4	88	1.11398
55	13JUN2008	day	4	88	0.88767
55	13JUN2008	day	3	88	1.69811
55	18JUN2008	day	5	88	0.34262
55	18JUN2008	day	5	88	0.10415
55	18JUN2008	day	5	88	0.55642
55	18JUN2008	twilight	5	88	0.42991
55	18JUN2008	night	5	88	0.25310
55	19JUN2008	night	6	88	0.75366
55	19JUN2008	twilight	6	88	0.33264
55	19JUN2008	day	6	88	0.33264
55	19JUN2008	day	6	88	0.16167
55	19JUN2008	day	6	88	0.73648
55	19JUN2008	day	6	88	0.62849
55	19JUN2008	twilight	6	88	0.59999
55	19JUN2008	night	6	88	1.02942
55	20JUN2008	night	6	88	0.16167
55	20JUN2008	twilight	6	88	0.52517
55	20JUN2008	day	6	88	0.53415
55	25JUN2008	day	7	88	0.64454
55	25JUN2008	day	7	88	0.61606
55	25JUN2008	day	7	88	0.64552
55	25JUN2008	twilight	7	88	0.82937
55	25JUN2008	night	7	88	0.91174
55	26JUN2008	night	7	88	0.17638
55	26JUN2008	twilight	7	88	0.19061
55	26JUN2008	day	7	88	0.41514
55	26JUN2008	day	7	88	0.64157
55	26JUN2008	day	7	88	0.23325
55	26JUN2008	day	7	88	0.59006
55	26JUN2008	twilight	7	88	0.67403
55	26JUN2008	night	7	88	0.00043
55	27JUN2008	night	7	88	0.00043
55	27JUN2008	twilight	7	88	-0.17980
55	27JUN2008	day	7	88	0.68494
55	16MAY2011	day	5	126	1.27878
55	16MAY2011	day	5	126	0.30125
55	16MAY2011	day	5	126	0.30125
55	16MAY2011	twilight	5	126	1.74037
55	16MAY2011	night	5	126	1.46241
55	16MAY2011	night	5	126	1.44717
55	17MAY2011	twilight	5	126	0.77822
55	17MAY2011	day	5	126	-3.00000
55	17MAY2011	day	6	126	1.91382
55	17MAY2011	day	6	126	1.43138
55	17MAY2011	day	6	126	1.88650
55	17MAY2011	twilight	6	126	0.69906
55	17MAY2011	night	6	126	0.69906
55	17MAY2011	night	6	126	1.76344
55	18MAY2011	twilight	6	126	1.34244
55	18MAY2011	day	6	126	1.14616
55	25MAY2011	day	7	126	1.86333
55	25MAY2011	day	7	126	1.30105
55	25MAY2011	day	7	126	1.07922
55	25MAY2011	day	7	126	1.11398
55	25MAY2011	twilight	7	126	1.56821

55	25MAY2011	night	7	126	1.50516
55	26MAY2011	twilight	7	126	1.27878
55	26MAY2011	day	7	126	0.69906
55	26MAY2011	day	7	126	1.20415
55	26MAY2011	day	7	126	0.90314
55	26MAY2011	day	7	126	0.84516
55	26MAY2011	twilight	7	126	1.17612
55	26MAY2011	night	7	126	1.14616
55	26MAY2011	night	7	126	0.84516
55	27MAY2011	twilight	8	126	1.00004
55	27MAY2011	day	8	126	1.00004
56	04JUN2008	day	1	254	1.29887
56	04JUN2008	day	1	254	1.09694
56	04JUN2008	day	1	254	2.02531
56	04JUN2008	twilight	1	254	1.19593
56	04JUN2008	night	1	254	1.91961
56	05JUN2008	night	1	254	0.81298
56	05JUN2008	twilight	1	254	2.07918
56	05JUN2008	day	1	254	0.61815
56	05JUN2008	day	1	254	0.30341
56	05JUN2008	day	1	254	1.74586
56	05JUN2008	day	1	254	1.33043
56	05JUN2008	twilight	1	254	1.24800
56	05JUN2008	night	1	254	1.44092
56	06JUN2008	night	2	254	0.60217
56	06JUN2008	twilight	2	254	1.97727
56	06JUN2008	day	2	254	1.00864
56	11JUN2008	day	3	254	0.87338
56	11JUN2008	day	3	254	2.13354
56	11JUN2008	day	3	254	2.12058
56	11JUN2008	twilight	3	254	0.62439
56	11JUN2008	night	3	254	0.63357
56	12JUN2008	night	3	254	2.22789
56	12JUN2008	twilight	3	254	2.21219
56	12JUN2008	day	3	254	2.78533
56	12JUN2008	day	3	254	1.80822
56	12JUN2008	day	3	254	2.73239
56	12JUN2008	twilight	3	254	1.23302
56	12JUN2008	night	3	254	0.93455
56	12JUN2008	night	3	254	2.34045
56	13JUN2008	twilight	3	254	0.62849
56	13JUN2008	day	3	254	0.71609
56	13JUN2008	day	3	254	1.50380
56	18JUN2008	day	5	254	0.95139
56	18JUN2008	day	5	254	0.84516
56	18JUN2008	day	5	254	2.06819
56	18JUN2008	twilight	5	254	1.56230
56	18JUN2008	night	5	254	1.36551
56	19JUN2008	night	6	254	1.39969
56	19JUN2008	twilight	6	254	2.24304
56	19JUN2008	day	6	254	1.39969
56	19JUN2008	day	6	254	0.45194
56	19JUN2008	day	6	254	0.35044
56	19JUN2008	day	6	254	2.17609
56	19JUN2008	twilight	6	254	0.88315
56	19JUN2008	night	6	254	2.19590

56	20JUN2008	night	6	254	0.88201
56	20JUN2008	twilight	6	254	1.49694
56	20JUN2008	day	6	254	0.30341
56	25JUN2008	day	7	254	0.53415
56	25JUN2008	day	7	254	0.56714
56	25JUN2008	day	7	254	2.17898
56	25JUN2008	twilight	7	254	-0.34775
56	25JUN2008	night	7	254	0.49150
56	26JUN2008	night	7	254	0.47727
56	26JUN2008	twilight	7	254	1.85004
56	26JUN2008	day	7	254	1.79029
56	26JUN2008	day	7	254	1.71182
56	26JUN2008	day	7	254	1.75282
56	26JUN2008	day	7	254	1.34244
56	26JUN2008	day	7	254	0.50664
56	26JUN2008	night	7	254	1.81292
56	27JUN2008	night	7	254	1.81292
56	27JUN2008	day	7	254	1.78534
56	27JUN2008	day	7	254	0.94895
56	16MAY2011	day	5	67	1.11398
56	16MAY2011	day	5	67	1.66277
56	16MAY2011	day	5	67	1.20415
56	16MAY2011	twilight	5	67	1.07922
56	16MAY2011	night	5	67	1.32224
56	16MAY2011	night	5	67	1.39796
56	17MAY2011	twilight	5	67	1.41499
56	17MAY2011	day	5	67	1.49138
56	17MAY2011	day	5	67	1.69020
56	17MAY2011	day	5	67	1.25530
56	17MAY2011	day	5	67	1.66277
56	17MAY2011	twilight	5	67	1.30105
56	17MAY2011	night	5	67	1.41499
56	17MAY2011	night	5	67	1.17612
56	18MAY2011	twilight	5	67	0.69906
56	18MAY2011	day	5	67	0.47727
56	25MAY2011	day	7	67	-3.00000
56	25MAY2011	day	7	67	0.60217
56	25MAY2011	day	7	67	2.08991
56	25MAY2011	twilight	7	67	2.10037
56	25MAY2011	night	7	67	0.60217
56	25MAY2011	night	7	67	1.04143
56	26MAY2011	twilight	7	67	1.25530
56	26MAY2011	day	7	67	0.69906
56	26MAY2011	day	7	67	0.95429
56	26MAY2011	day	7	67	1.69898
56	26MAY2011	day	7	67	1.56821
56	26MAY2011	twilight	7	67	1.00004
56	27MAY2011	night	8	67	1.04143
56	27MAY2011	day	8	67	0.69906
56	01JUN2011	day	1	67	0.84516
56	01JUN2011	day	1	67	1.14616
56	01JUN2011	day	1	67	1.07922
56	01JUN2011	twilight	1	67	0.60217
56	01JUN2011	night	1	67	1.11398
56	01JUN2011	night	1	67	0.77822
56	02JUN2011	twilight	1	67	1.43138

56	02JUN2011	day	1	67	1.38023
56	02JUN2011	day	1	67	1.56821
56	02JUN2011	day	1	67	1.07922
56	02JUN2011	day	1	67	1.30105
56	02JUN2011	twilight	1	67	1.00004
56	02JUN2011	night	1	67	1.46241
56	02JUN2011	night	2	67	1.41499
56	03JUN2011	twilight	2	67	1.77086
56	03JUN2011	day	2	67	0.84516
56	06JUN2011	day	2	67	0.00043
56	06JUN2011	day	2	67	0.30125
56	06JUN2011	day	2	67	0.69906
56	06JUN2011	twilight	2	67	0.90314
56	06JUN2011	night	3	67	1.04143
56	06JUN2011	night	3	67	0.30125
56	07JUN2011	twilight	3	67	0.84516
56	07JUN2011	day	3	67	0.60217
57	04JUN2008	day	1	27	1.46984
57	04JUN2008	day	1	27	1.19593
57	04JUN2008	day	1	27	0.85376
57	04JUN2008	twilight	1	27	1.26954
57	04JUN2008	night	1	27	0.82354
57	05JUN2008	night	1	27	1.32636
57	05JUN2008	twilight	1	27	0.03383
57	05JUN2008	day	1	27	1.45026
57	05JUN2008	day	1	27	1.28106
57	05JUN2008	day	1	27	0.90800
57	05JUN2008	day	1	27	1.07922
57	05JUN2008	twilight	1	27	1.25044
57	05JUN2008	night	1	27	1.22013
57	06JUN2008	night	2	27	1.38023
57	06JUN2008	twilight	2	27	1.31389
57	06JUN2008	day	2	27	1.18755
57	11JUN2008	day	3	27	1.36363
57	11JUN2008	day	3	27	1.17612
57	11JUN2008	day	3	27	1.72836
57	11JUN2008	twilight	3	27	1.72917
57	11JUN2008	night	3	27	1.77233
57	12JUN2008	night	3	27	1.77086
57	12JUN2008	twilight	3	27	1.68843
57	12JUN2008	day	3	27	1.71768
57	12JUN2008	day	3	27	1.78534
57	12JUN2008	day	3	27	0.97731
57	12JUN2008	twilight	3	27	1.60854
57	12JUN2008	night	3	27	1.38204
57	12JUN2008	night	3	27	1.88480
57	13JUN2008	twilight	3	27	1.51456
57	13JUN2008	day	3	27	1.41997
57	13JUN2008	day	3	27	1.66465
57	18JUN2008	day	5	27	0.66001
57	18JUN2008	day	5	27	0.98860
57	18JUN2008	day	5	27	0.83065
57	18JUN2008	twilight	5	27	0.95766
57	18JUN2008	night	5	27	0.74359
57	19JUN2008	night	6	27	0.25792
57	19JUN2008	twilight	6	27	0.61500

57	19JUN2008	day	6	27	0.83257
57	19JUN2008	day	6	27	0.35430
57	19JUN2008	day	6	27	0.35430
57	19JUN2008	day	6	27	0.83954
57	19JUN2008	twilight	6	27	0.43473
57	19JUN2008	night	6	27	1.02123
57	20JUN2008	night	6	27	0.62747
57	20JUN2008	twilight	6	27	0.41514
57	20JUN2008	day	6	27	0.55883
57	25JUN2008	day	7	27	0.68224
57	25JUN2008	day	7	27	0.60970
57	25JUN2008	day	7	27	0.81631
57	25JUN2008	twilight	7	27	0.97364
57	25JUN2008	night	7	27	0.79036
57	26JUN2008	night	7	27	0.36192
57	26JUN2008	twilight	7	27	0.36192
57	26JUN2008	day	7	27	0.25551
57	26JUN2008	day	7	27	0.44420
57	26JUN2008	day	7	27	-0.04672
57	26JUN2008	day	7	27	0.94748
57	26JUN2008	twilight	7	27	1.05312
57	26JUN2008	night	7	27	-0.46725
57	27JUN2008	night	7	27	-0.46725
57	27JUN2008	twilight	7	27	-0.53611
57	27JUN2008	day	7	27	0.62747
57	16MAY2011	day	5	72	1.00004
57	16MAY2011	day	5	72	0.30125
57	16MAY2011	day	5	72	0.47727
57	16MAY2011	twilight	5	72	1.30105
57	16MAY2011	night	5	72	1.41499
57	16MAY2011	night	5	72	1.00004
57	17MAY2011	twilight	5	72	1.38023
57	17MAY2011	day	5	72	1.67211
57	17MAY2011	day	5	72	1.82608
57	17MAY2011	day	5	72	1.63348
57	17MAY2011	day	5	72	1.04143
57	17MAY2011	twilight	5	72	0.84516
57	17MAY2011	night	5	72	1.11398
57	17MAY2011	night	5	72	1.17612
57	18MAY2011	twilight	5	72	0.30125
57	18MAY2011	day	5	72	0.47727
57	25MAY2011	day	7	72	0.69906
57	25MAY2011	day	7	72	0.60217
57	25MAY2011	day	7	72	0.77822
57	25MAY2011	twilight	7	72	0.77822
57	25MAY2011	night	7	72	0.95429
57	25MAY2011	night	7	72	1.11398
57	26MAY2011	twilight	7	72	1.07922
57	26MAY2011	day	7	72	-3.00000
57	26MAY2011	day	7	72	0.90314
57	26MAY2011	day	7	72	0.47727
57	26MAY2011	day	7	72	0.30125
57	26MAY2011	twilight	7	72	0.69906
57	26MAY2011	night	8	72	0.69906
57	27MAY2011	twilight	8	72	1.14616
57	27MAY2011	day	8	72	1.07922

57	01JUN2011	day	1	72	0.84516
57	01JUN2011	day	1	72	1.00004
57	01JUN2011	day	1	72	1.04143
57	01JUN2011	twilight	1	72	0.60217
57	01JUN2011	night	1	72	1.30105
57	01JUN2011	night	1	72	1.23047
57	02JUN2011	twilight	1	72	1.91908
57	02JUN2011	day	1	72	1.89210
57	02JUN2011	day	1	72	0.60217
57	02JUN2011	day	1	72	0.84516
57	02JUN2011	twilight	1	72	0.60217
57	02JUN2011	night	1	72	1.27878
57	03JUN2011	night	2	72	1.00004
57	03JUN2011	twilight	2	72	0.60217
57	06JUN2011	day	2	72	0.60217
57	06JUN2011	day	2	72	0.47727
57	06JUN2011	twilight	2	72	1.04143
57	06JUN2011	night	3	72	0.47727
57	06JUN2011	night	3	72	-3.00000
57	07JUN2011	twilight	3	72	1.68125
57	07JUN2011	day	3	72	0.84516
58	16MAY2011	day	5	71	1.88650
58	16MAY2011	day	5	71	1.47714
58	16MAY2011	day	5	71	0.90314
58	16MAY2011	twilight	5	71	1.27878
58	16MAY2011	night	5	71	1.00004
58	16MAY2011	night	5	71	1.39796
58	17MAY2011	twilight	5	71	1.46241
58	17MAY2011	day	5	71	0.69906
58	17MAY2011	day	5	71	1.30105
58	17MAY2011	day	5	71	1.30105
58	17MAY2011	day	5	71	0.77822
58	17MAY2011	twilight	5	71	1.86924
58	17MAY2011	night	5	71	1.53149
58	17MAY2011	night	5	71	1.04143
58	18MAY2011	twilight	5	71	1.71601
58	18MAY2011	day	5	71	1.44717
58	25MAY2011	day	7	71	2.19313
58	25MAY2011	day	7	71	2.13354
58	25MAY2011	day	7	71	1.00004
58	25MAY2011	twilight	7	71	1.62326
58	25MAY2011	night	7	71	1.62326
58	25MAY2011	night	7	71	1.11398
58	26MAY2011	twilight	7	71	1.61279
58	26MAY2011	day	7	71	0.47727
58	26MAY2011	day	7	71	1.04143
58	26MAY2011	day	7	71	1.61279
58	26MAY2011	day	7	71	1.20415
58	26MAY2011	twilight	7	71	0.30125
58	26MAY2011	night	7	71	0.84516
58	26MAY2011	night	8	71	1.47714
58	27MAY2011	twilight	8	71	1.23047
58	27MAY2011	day	8	71	0.30125
58	01JUN2011	day	1	71	1.66277
58	01JUN2011	day	1	71	1.17612
58	01JUN2011	day	1	71	1.50516

58	01JUN2011	twilight	1	71	1.47714
58	01JUN2011	night	1	71	1.53149
58	01JUN2011	night	1	71	1.62326
58	02JUN2011	twilight	1	71	1.74820
58	02JUN2011	day	1	71	1.78534
58	02JUN2011	day	1	71	1.81292
58	02JUN2011	day	1	71	1.41499
58	02JUN2011	day	1	71	1.47714
58	02JUN2011	twilight	1	71	-3.00000
58	02JUN2011	night	2	71	0.60217
58	03JUN2011	twilight	2	71	1.04143
58	03JUN2011	day	2	71	0.60217
58	06JUN2011	day	2	71	0.77822
58	06JUN2011	day	2	71	0.69906
58	06JUN2011	day	2	71	1.11398
58	06JUN2011	twilight	2	71	1.00004
58	06JUN2011	night	3	71	0.30125
58	06JUN2011	night	3	71	0.95429
58	07JUN2011	twilight	3	71	0.60217
58	07JUN2011	day	3	71	1.46241
59	16MAY2011	day	5	89	1.46241
59	16MAY2011	day	5	89	-3.00000
59	16MAY2011	day	5	89	0.60217
59	16MAY2011	twilight	5	89	1.04143
59	16MAY2011	night	5	89	1.79935
59	16MAY2011	night	5	89	1.86333
59	17MAY2011	twilight	5	89	1.86333
59	17MAY2011	day	5	89	1.20415
59	17MAY2011	day	5	89	1.62326
59	17MAY2011	day	5	89	1.44717
59	17MAY2011	day	5	89	1.41499
59	17MAY2011	twilight	5	89	0.90314
59	17MAY2011	night	5	89	1.14616
59	17MAY2011	night	5	89	1.11398
59	18MAY2011	twilight	5	89	0.60217
59	18MAY2011	day	5	89	0.60217
60	16MAY2011	day	5	119	1.00004
60	16MAY2011	day	5	119	-3.00000
60	16MAY2011	day	5	119	0.30125
60	16MAY2011	twilight	5	119	0.60217
60	16MAY2011	night	5	119	1.39796
60	16MAY2011	night	5	119	1.55631
60	17MAY2011	twilight	5	119	1.61279
60	17MAY2011	day	5	119	1.23047
60	17MAY2011	day	5	119	1.82608
60	17MAY2011	day	5	119	1.71601
60	17MAY2011	day	5	119	0.90314
60	17MAY2011	twilight	5	119	0.84516
60	17MAY2011	night	5	119	0.84516
60	17MAY2011	night	5	119	0.95429
60	18MAY2011	twilight	5	119	0.47727
60	18MAY2011	day	5	119	0.47727
60	25MAY2011	day	7	119	0.60217
60	25MAY2011	day	7	119	0.60217
60	25MAY2011	day	7	119	0.77822
60	25MAY2011	twilight	7	119	0.47727

60	25MAY2011	night	7	119	0.95429
60	25MAY2011	night	7	119	0.84516
60	26MAY2011	twilight	7	119	1.25530
60	26MAY2011	day	7	119	1.14616
60	26MAY2011	day	7	119	0.95429
60	26MAY2011	day	7	119	0.47727
60	26MAY2011	day	7	119	0.30125
60	26MAY2011	twilight	7	119	0.69906
60	26MAY2011	night	7	119	0.69906
60	26MAY2011	night	8	119	-3.00000
60	27MAY2011	twilight	8	119	0.77822
60	27MAY2011	day	8	119	1.07922
63	04JUN2008	day	1	115	1.26954
63	04JUN2008	day	1	115	1.68754
63	04JUN2008	day	1	115	1.59330
63	04JUN2008	twilight	1	115	1.03747
63	04JUN2008	night	1	115	1.83379
63	05JUN2008	night	1	115	0.23070
63	05JUN2008	twilight	1	115	1.73401
63	05JUN2008	day	1	115	0.70079
63	05JUN2008	day	1	115	0.86279
63	05JUN2008	day	1	115	1.27878
63	05JUN2008	day	1	115	1.33043
63	05JUN2008	twilight	1	115	1.15232
63	05JUN2008	night	1	115	1.70158
63	06JUN2008	night	2	115	1.54284
63	06JUN2008	twilight	2	115	1.47858
63	06JUN2008	day	2	115	0.94552
63	11JUN2008	day	3	115	2.29886
63	11JUN2008	day	3	115	0.31408
63	11JUN2008	day	3	115	1.64934
63	11JUN2008	twilight	3	115	2.18185
63	11JUN2008	night	3	115	1.56704
63	12JUN2008	night	3	115	0.46553
63	12JUN2008	twilight	3	115	1.74897
63	12JUN2008	day	3	115	0.71775
63	12JUN2008	day	3	115	0.42991
63	12JUN2008	day	3	115	2.21219
63	12JUN2008	twilight	3	115	0.80625
63	12JUN2008	night	3	115	1.48856
63	12JUN2008	night	3	115	1.66746
63	13JUN2008	twilight	3	115	0.92947
63	13JUN2008	day	3	115	1.47714
63	13JUN2008	day	3	115	1.38918
63	18JUN2008	day	5	115	0.68940
63	18JUN2008	day	5	115	0.77166
63	18JUN2008	day	5	115	0.88711
63	18JUN2008	twilight	5	115	0.03383
63	18JUN2008	night	5	115	1.43777
63	19JUN2008	night	6	115	1.48997
63	19JUN2008	twilight	6	115	0.07591
63	19JUN2008	day	6	115	0.26505
63	19JUN2008	day	6	115	0.53415
63	19JUN2008	day	6	115	0.20710
63	19JUN2008	day	6	115	0.74437
63	19JUN2008	twilight	6	115	0.70680

63	19JUN2008	night	6	115	1.40656
63	20JUN2008	night	6	115	1.44717
63	20JUN2008	twilight	6	115	1.73481
63	20JUN2008	day	6	115	0.26741
63	25JUN2008	day	7	115	1.96521
63	25JUN2008	day	7	115	1.93044
63	25JUN2008	day	7	115	1.49417
63	25JUN2008	twilight	7	115	0.55642
63	25JUN2008	night	7	115	2.22531
63	26JUN2008	night	7	115	1.34441
63	26JUN2008	twilight	7	115	2.21485
63	26JUN2008	day	7	115	0.91703
63	26JUN2008	day	7	115	1.24306
63	26JUN2008	day	7	115	0.46553
63	26JUN2008	day	7	115	0.55763
63	26JUN2008	twilight	7	115	2.04922
63	26JUN2008	night	7	115	1.36175
63	27JUN2008	night	7	115	1.36175
63	27JUN2008	twilight	7	115	1.35795
63	27JUN2008	day	7	115	0.61606
64	04JUN2008	day	1	38	1.95231
64	04JUN2008	day	1	38	1.67118
64	04JUN2008	day	1	38	1.70244
64	04JUN2008	twilight	1	38	0.92588
64	04JUN2008	night	1	38	1.84943
64	05JUN2008	night	1	38	1.31599
64	05JUN2008	twilight	1	38	1.89488
64	05JUN2008	day	1	38	1.17901
64	05JUN2008	day	1	38	1.86118
64	05JUN2008	day	1	38	1.45180
64	05JUN2008	day	1	38	1.45196
64	05JUN2008	twilight	1	38	1.69197
64	05JUN2008	night	1	38	2.10381
64	06JUN2008	night	2	38	0.92742
64	06JUN2008	twilight	2	38	1.50380
64	06JUN2008	day	2	38	1.22791
64	11JUN2008	day	3	38	0.61500
64	11JUN2008	day	3	38	1.05694
64	11JUN2008	day	3	38	1.12388
64	11JUN2008	twilight	3	38	2.42325
64	11JUN2008	night	3	38	2.44871
64	12JUN2008	night	3	38	1.98182
64	12JUN2008	twilight	3	38	1.98498
64	12JUN2008	day	3	38	1.00864
64	12JUN2008	day	3	38	0.87163
64	12JUN2008	day	3	38	1.31389
64	12JUN2008	twilight	3	38	1.38023
64	12JUN2008	night	3	38	0.92896
64	12JUN2008	night	3	38	1.28558
64	13JUN2008	twilight	3	38	2.27876
64	13JUN2008	day	3	38	2.19033
64	13JUN2008	day	3	38	2.11727
64	18JUN2008	day	5	38	0.93505
64	18JUN2008	day	5	38	1.04143
64	18JUN2008	day	5	38	0.88542
64	18JUN2008	twilight	5	38	0.62747

64	18JUN2008	night	5	38	0.97868
64	19JUN2008	night	6	38	1.04536
64	19JUN2008	twilight	6	38	0.60325
64	19JUN2008	day	6	38	1.00436
64	19JUN2008	day	6	38	0.91121
64	19JUN2008	day	6	38	0.25066
64	19JUN2008	day	6	38	1.35604
64	19JUN2008	twilight	6	38	1.35027
64	19JUN2008	night	6	38	2.29004
64	20JUN2008	night	6	38	1.35413
64	20JUN2008	twilight	6	38	1.65322
64	20JUN2008	day	6	38	0.70079
64	25JUN2008	day	7	38	-0.20273
64	25JUN2008	day	7	38	0.58218
64	25JUN2008	day	7	38	1.44717
64	25JUN2008	twilight	7	38	1.08640
64	25JUN2008	night	7	38	0.96099
64	26JUN2008	night	7	38	0.91068
64	26JUN2008	twilight	7	38	1.03346
64	26JUN2008	day	7	38	0.70766
64	26JUN2008	day	7	38	0.64748
64	26JUN2008	day	7	38	0.65619
64	26JUN2008	day	7	38	0.62849
64	26JUN2008	twilight	7	38	0.76500
64	26JUN2008	night	7	38	0.36192
64	27JUN2008	night	7	38	0.35813
64	27JUN2008	twilight	7	38	0.36192
64	27JUN2008	day	7	38	0.82679
65	04JUN2008	day	1	24	1.17322
65	04JUN2008	day	1	24	0.96619
65	04JUN2008	day	1	24	0.51996
65	04JUN2008	twilight	1	24	1.26720
65	04JUN2008	night	1	24	1.22534
65	05JUN2008	night	1	24	1.36737
65	05JUN2008	twilight	1	24	1.61067
65	05JUN2008	day	1	24	1.35795
65	05JUN2008	day	1	24	0.64355
65	05JUN2008	day	1	24	0.66001
65	05JUN2008	day	1	24	0.64650
65	05JUN2008	twilight	1	24	0.77822
65	05JUN2008	night	1	24	-0.07160
65	06JUN2008	night	2	24	1.52115
65	06JUN2008	twilight	2	24	1.29006
65	06JUN2008	day	2	24	1.05312
65	11JUN2008	day	3	24	2.17026
65	11JUN2008	day	3	24	2.15837
65	11JUN2008	day	3	24	1.30537
65	11JUN2008	twilight	3	24	1.77159
65	11JUN2008	night	3	24	1.77086
65	12JUN2008	night	3	24	1.67579
65	12JUN2008	twilight	3	24	1.70071
65	12JUN2008	day	3	24	1.87853
65	12JUN2008	day	3	24	1.88987
65	12JUN2008	day	3	24	-0.00043
65	12JUN2008	twilight	3	24	0.90639
65	12JUN2008	night	3	24	0.85497

65	12JUN2008	night	3	24	0.32654
65	13JUN2008	twilight	3	24	0.67311
65	13JUN2008	day	3	24	1.38741
65	13JUN2008	day	3	24	1.28106
65	18JUN2008	day	5	24	0.72681
65	18JUN2008	day	5	24	0.80216
65	18JUN2008	day	5	24	0.62335
65	18JUN2008	twilight	5	24	0.88655
65	18JUN2008	night	5	24	0.54419
65	19JUN2008	night	6	24	-3.00000
65	19JUN2008	twilight	6	24	0.80216
65	19JUN2008	day	6	24	0.97271
65	19JUN2008	day	6	24	-3.00000
65	19JUN2008	day	6	24	0.80216
65	19JUN2008	day	6	24	0.62335
65	19JUN2008	twilight	6	24	0.88655
65	19JUN2008	night	6	24	0.54419
65	20JUN2008	night	6	24	-3.00000
65	20JUN2008	twilight	6	24	0.60217
65	20JUN2008	day	6	24	0.58331
65	25JUN2008	day	7	24	0.91174
65	25JUN2008	day	7	24	0.81164
65	25JUN2008	day	7	24	1.50380
65	25JUN2008	twilight	7	24	0.91174
65	25JUN2008	night	7	24	1.28106
65	26JUN2008	night	7	24	0.24329
65	26JUN2008	twilight	7	24	0.25310
65	26JUN2008	day	7	24	0.70851
65	26JUN2008	day	7	24	0.75289
65	26JUN2008	day	7	24	0.54419
65	26JUN2008	day	7	24	0.62542
65	26JUN2008	twilight	7	24	0.84516
65	26JUN2008	night	7	24	0.60217
65	27JUN2008	night	7	24	0.60217
65	27JUN2008	twilight	7	24	0.61289
65	27JUN2008	day	7	24	0.33264
69	04JUN2008	day	1	31	0.79456
69	04JUN2008	day	1	31	0.87800
69	04JUN2008	day	1	31	0.49982
69	04JUN2008	twilight	1	31	0.38399
69	04JUN2008	night	1	31	0.62747
69	05JUN2008	night	1	31	0.79664
69	05JUN2008	twilight	1	31	0.88315
69	05JUN2008	day	1	31	0.60649
69	05JUN2008	day	1	31	0.56714
69	05JUN2008	day	1	31	-0.00043
69	05JUN2008	day	1	31	0.62335
69	05JUN2008	twilight	1	31	0.44731
69	05JUN2008	night	1	31	0.47727
69	06JUN2008	night	2	31	0.38757
69	06JUN2008	twilight	2	31	0.47290
69	06JUN2008	day	2	31	0.18213
69	11JUN2008	day	3	31	0.20439
69	11JUN2008	day	3	31	0.98547
69	11JUN2008	day	3	31	1.50380
69	11JUN2008	twilight	3	31	1.63247

69	11JUN2008	night	3	31	1.65032
69	12JUN2008	night	3	31	1.70502
69	12JUN2008	twilight	3	31	1.93400
69	12JUN2008	day	3	31	1.95569
69	12JUN2008	day	3	31	0.69381
69	12JUN2008	day	3	31	1.43138
69	12JUN2008	twilight	3	31	1.62532
69	12JUN2008	night	3	31	-3.00000
69	12JUN2008	night	4	31	0.35813
69	13JUN2008	twilight	4	31	0.34459
69	13JUN2008	day	4	31	0.43473
69	13JUN2008	day	3	31	1.24800
69	18JUN2008	day	5	31	0.54913
69	18JUN2008	day	5	31	-0.14146
69	18JUN2008	day	5	31	0.18213
69	18JUN2008	twilight	5	31	-0.19382
69	18JUN2008	night	5	31	0.50120
69	19JUN2008	night	6	31	0.42830
69	19JUN2008	twilight	6	31	0.62747
69	19JUN2008	day	6	31	0.71273
69	19JUN2008	day	6	31	1.02123
69	19JUN2008	day	6	31	0.74359
69	19JUN2008	day	6	31	0.61500
69	19JUN2008	twilight	6	31	0.66941
69	19JUN2008	night	6	31	0.94305
69	20JUN2008	night	6	31	0.38220
69	20JUN2008	twilight	6	31	0.67034
69	20JUN2008	day	6	31	0.58218
70	04JUN2008	day	1	25	1.65322
70	04JUN2008	day	1	25	1.36175
70	04JUN2008	day	1	25	2.14613
70	04JUN2008	twilight	1	25	0.92896
70	04JUN2008	night	1	25	1.08282
70	05JUN2008	night	1	25	1.13675
70	05JUN2008	twilight	1	25	1.78462
70	05JUN2008	day	1	25	1.88367
70	05JUN2008	day	1	25	1.91488
70	05JUN2008	day	1	25	1.83696
70	05JUN2008	day	1	25	1.00436
70	05JUN2008	twilight	1	25	1.11730
70	05JUN2008	night	1	25	1.57750
70	06JUN2008	night	2	25	1.07192
70	06JUN2008	twilight	2	25	2.14302
70	06JUN2008	day	2	25	1.42653
70	11JUN2008	day	3	25	-0.70553
70	11JUN2008	day	3	25	1.81625
70	11JUN2008	day	3	25	1.73561
70	11JUN2008	twilight	3	25	1.70071
70	11JUN2008	night	3	25	1.79100
70	12JUN2008	night	3	25	1.66371
70	12JUN2008	twilight	3	25	1.64346
70	12JUN2008	day	3	25	2.06446
70	12JUN2008	day	3	25	1.53149
70	12JUN2008	day	3	25	1.77233
70	12JUN2008	twilight	3	25	1.93095
70	12JUN2008	night	3	25	0.67127

70	12JUN2008	night	3	25	1.82673
70	13JUN2008	twilight	3	25	0.98005
70	13JUN2008	day	3	25	0.92896
70	13JUN2008	day	3	25	1.73160
70	18JUN2008	day	5	25	0.99699
70	18JUN2008	day	5	25	0.91440
70	18JUN2008	day	5	25	1.41332
70	18JUN2008	twilight	5	25	0.53033
70	18JUN2008	night	5	25	1.10384
70	19JUN2008	night	6	25	0.20978
70	19JUN2008	twilight	6	25	2.22531
70	19JUN2008	day	6	25	0.49707
70	19JUN2008	day	6	25	2.25042
70	19JUN2008	day	6	25	0.79664
70	19JUN2008	day	6	25	1.10041
70	19JUN2008	twilight	6	25	0.60433
70	19JUN2008	night	6	25	1.31808
70	20JUN2008	night	6	25	-0.30016
70	20JUN2008	twilight	6	25	2.08279
70	20JUN2008	day	6	25	1.26247
70	25JUN2008	day	7	25	0.95477
70	25JUN2008	day	7	25	1.30752
70	25JUN2008	day	7	25	1.29669
70	25JUN2008	twilight	7	25	1.13357
70	25JUN2008	night	7	25	0.70766
70	26JUN2008	night	7	25	0.77822
70	26JUN2008	twilight	7	25	1.24057
70	26JUN2008	day	7	25	0.71609
70	26JUN2008	day	7	25	1.11062
70	26JUN2008	day	7	25	-0.04915
70	26JUN2008	day	7	25	1.48997
70	26JUN2008	twilight	7	25	0.71858
70	26JUN2008	night	7	25	0.30125
70	27JUN2008	night	7	25	0.30125
70	27JUN2008	twilight	7	25	0.17348
70	27JUN2008	day	7	25	0.31197
71	04JUN2008	day	1	19	0.95041
71	04JUN2008	day	1	19	1.06450
71	04JUN2008	day	1	19	1.02942
71	04JUN2008	twilight	1	19	1.16140
71	04JUN2008	night	1	19	1.88025
71	05JUN2008	night	1	19	0.24329
71	05JUN2008	twilight	1	19	1.89928
71	05JUN2008	day	1	19	0.33465
71	05JUN2008	day	1	19	0.77240
71	05JUN2008	day	1	19	0.75823
71	05JUN2008	day	1	19	1.82867
71	05JUN2008	twilight	1	19	0.61920
71	05JUN2008	night	1	19	1.77306
71	06JUN2008	night	2	19	0.65715
71	06JUN2008	twilight	2	19	0.27898
71	06JUN2008	day	2	19	0.00043
71	11JUN2008	day	3	19	1.37842
71	11JUN2008	day	3	19	2.21749
71	11JUN2008	day	3	19	2.23805
71	11JUN2008	twilight	3	19	1.64837

71	11JUN2008	night	3	19	1.76043
71	12JUN2008	night	3	19	2.18185
71	12JUN2008	twilight	3	19	2.17898
71	12JUN2008	day	3	19	2.33646
71	12JUN2008	day	3	19	2.06446
71	12JUN2008	day	3	19	1.73720
71	12JUN2008	twilight	3	19	1.85613
71	12JUN2008	night	3	19	1.38023
71	12JUN2008	night	4	19	1.86214
71	13JUN2008	twilight	4	19	1.41997
71	13JUN2008	day	4	19	1.22013
71	13JUN2008	day	3	19	0.96478
71	18JUN2008	day	5	19	0.73328
71	18JUN2008	day	5	19	0.39811
71	18JUN2008	day	5	19	0.80147
71	18JUN2008	twilight	5	19	0.85497
71	18JUN2008	night	5	19	0.31408
71	19JUN2008	night	6	19	-3.00000
71	19JUN2008	twilight	6	19	1.48856
71	19JUN2008	day	6	19	1.54034
71	19JUN2008	day	6	19	0.84640
71	19JUN2008	day	6	19	0.48302
71	19JUN2008	day	6	19	-3.00000
71	19JUN2008	twilight	6	19	0.58104
71	19JUN2008	night	6	19	0.48302
71	20JUN2008	night	6	19	0.52517
71	20JUN2008	twilight	6	19	0.72518
71	20JUN2008	day	6	19	0.15259
71	25JUN2008	day	7	19	0.74044
71	25JUN2008	day	7	19	0.71609
71	25JUN2008	day	7	19	0.82158
71	25JUN2008	twilight	7	19	0.41514
71	25JUN2008	night	7	19	0.39811
71	26JUN2008	night	7	19	0.40671
71	26JUN2008	twilight	7	19	0.41347
71	26JUN2008	day	7	19	0.66285
71	26JUN2008	day	7	19	0.26505
71	26JUN2008	day	7	19	0.26505
71	26JUN2008	day	7	19	0.47727
71	26JUN2008	day	7	19	0.18213
71	26JUN2008	night	7	19	0.06108
71	27JUN2008	night	7	19	0.06108
71	27JUN2008	day	7	19	0.04961
71	27JUN2008	day	7	19	0.35813
106	03JUN2009	night	4	25	1.23047
106	04JUN2009	night	4	25	1.38918
106	04JUN2009	twilight	4	25	1.33447
106	04JUN2009	day	4	25	1.56704
106	04JUN2009	day	4	25	1.21751
106	04JUN2009	day	4	25	2.17609
106	04JUN2009	day	4	25	2.23045
106	04JUN2009	twilight	4	25	0.48444
106	04JUN2009	night	4	25	1.54408
106	05JUN2009	night	4	25	1.50652
106	05JUN2009	twilight	4	25	1.50922
106	05JUN2009	day	4	25	1.06822

106	10JUN2009	day	6	25	1.35985
106	10JUN2009	day	6	25	1.55752
106	10JUN2009	day	6	25	0.73328
106	10JUN2009	twilight	6	25	0.78540
106	10JUN2009	night	6	25	0.92018
106	11JUN2009	night	6	25	1.29887
106	11JUN2009	twilight	6	25	1.45790
106	11JUN2009	day	6	25	1.08640
106	11JUN2009	day	6	25	0.99216
106	11JUN2009	day	6	25	0.92896
106	11JUN2009	day	6	25	0.99830
106	11JUN2009	twilight	6	25	0.87512
106	11JUN2009	night	6	25	0.85920
106	12JUN2009	night	6	25	1.53909
106	12JUN2009	twilight	6	25	1.49138
106	12JUN2009	day	6	25	1.65129
106	25JUN2009	day	8	25	1.55872
106	25JUN2009	day	8	25	1.94597
106	25JUN2009	twilight	8	25	1.64346
106	25JUN2009	night	8	25	0.12743
106	26JUN2009	night	8	25	0.74280
106	26JUN2009	twilight	8	25	1.72099
106	26JUN2009	day	8	25	2.17319
112	03JUN2009	day	4	17	0.78183
112	03JUN2009	day	4	17	1.16140
112	03JUN2009	day	4	17	0.89326
112	03JUN2009	twilight	4	17	1.42490
112	03JUN2009	night	4	17	1.50516
112	04JUN2009	night	4	17	1.14925
112	04JUN2009	twilight	4	17	0.75595
112	04JUN2009	day	4	17	0.63256
112	04JUN2009	day	4	17	1.74273
112	04JUN2009	day	4	17	1.92583
112	04JUN2009	day	4	17	1.19868
112	04JUN2009	twilight	4	17	1.95857
112	04JUN2009	night	4	17	1.63348
112	05JUN2009	night	4	17	1.74037
112	05JUN2009	twilight	4	17	1.70843
112	05JUN2009	day	4	17	1.76418
112	10JUN2009	day	6	17	1.36923
112	10JUN2009	day	6	17	0.15866
112	10JUN2009	day	6	17	0.58557
112	10JUN2009	twilight	6	17	1.81691
112	10JUN2009	night	6	17	1.36551
112	11JUN2009	night	6	17	0.26741
112	11JUN2009	twilight	6	17	1.81492
112	11JUN2009	day	6	17	1.56821
112	11JUN2009	day	6	17	1.36551
112	11JUN2009	day	6	17	1.32224
112	11JUN2009	day	6	17	1.31599
112	11JUN2009	twilight	6	17	1.57404
112	11JUN2009	night	6	17	1.69020
112	12JUN2009	night	6	17	1.06074
112	12JUN2009	twilight	6	17	1.90092
112	12JUN2009	day	6	17	0.85071
112	17JUN2009	day	8	17	0.00475

112	17JUN2009	day	8	17	0.61077
112	17JUN2009	day	8	17	1.32840
112	17JUN2009	twilight	8	17	0.35813
112	17JUN2009	night	8	17	1.35027
112	18JUN2009	night	8	17	1.76118
112	18JUN2009	twilight	8	17	1.62222
112	18JUN2009	day	8	17	0.84516
112	18JUN2009	day	8	17	1.42490
112	18JUN2009	day	8	17	0.45500
112	18JUN2009	day	8	17	1.74352
112	18JUN2009	twilight	8	17	0.70252
112	18JUN2009	night	8	17	0.16167
112	19JUN2009	night	8	17	1.75968
112	19JUN2009	twilight	8	17	1.59330
112	19JUN2009	day	8	17	1.51190
112	24JUN2009	day	1	17	0.65619
112	24JUN2009	day	1	17	0.41514
112	24JUN2009	day	1	17	0.44107
112	24JUN2009	twilight	1	17	1.03346
112	24JUN2009	night	1	17	1.13357
112	25JUN2009	night	2	17	1.23047
112	25JUN2009	twilight	2	17	1.78391
112	25JUN2009	day	2	17	1.76043
112	25JUN2009	day	2	17	0.43313
112	25JUN2009	day	2	17	1.14616
112	25JUN2009	day	2	17	1.35795
112	25JUN2009	twilight	2	17	1.29887
112	25JUN2009	night	2	17	1.02942
112	26JUN2009	night	2	17	0.57990
112	26JUN2009	twilight	2	17	1.36737
112	26JUN2009	day	2	17	0.95332
113	03JUN2009	day	4	19	1.86214
113	03JUN2009	day	4	19	1.02123
113	03JUN2009	day	4	19	1.60639
113	03JUN2009	twilight	4	19	1.30322
113	03JUN2009	night	4	19	1.46241
113	04JUN2009	night	4	19	1.67487
113	04JUN2009	twilight	4	19	0.94305
113	04JUN2009	day	4	19	1.04536
113	04JUN2009	day	4	19	1.37477
113	04JUN2009	day	4	19	1.02123
113	04JUN2009	day	4	19	1.75359
113	04JUN2009	twilight	4	19	1.68396
113	04JUN2009	night	4	19	0.80216
113	05JUN2009	night	4	19	1.51323
113	05JUN2009	twilight	4	19	1.83379
113	05JUN2009	day	4	19	1.77159
113	10JUN2009	day	6	19	1.78888
113	10JUN2009	day	6	19	1.08994
113	10JUN2009	day	6	19	1.86747
113	10JUN2009	twilight	6	19	0.79386
113	10JUN2009	night	6	19	1.01708
113	11JUN2009	night	6	19	1.78391
113	11JUN2009	twilight	6	19	1.20954
113	11JUN2009	day	6	19	1.80004
113	11JUN2009	day	6	19	1.82413

113	11JUN2009	day	6	19	1.85431
113	11JUN2009	day	6	19	1.21751
113	11JUN2009	twilight	6	19	1.76568
113	11JUN2009	night	6	19	1.72755
113	12JUN2009	night	6	19	0.88315
113	12JUN2009	twilight	6	19	1.82086
113	12JUN2009	day	6	19	1.77525
113	17JUN2009	day	8	19	1.68215
113	17JUN2009	day	8	19	1.76568
113	17JUN2009	day	8	19	1.80004
113	17JUN2009	twilight	8	19	1.05694
113	17JUN2009	night	8	19	1.87853
113	18JUN2009	night	8	19	0.98905
113	18JUN2009	twilight	8	19	1.86982
113	18JUN2009	day	8	19	1.85794
113	18JUN2009	day	8	19	1.80686
113	18JUN2009	day	8	19	0.95574
113	18JUN2009	day	8	19	1.84634
113	18JUN2009	twilight	8	19	1.87041
113	18JUN2009	night	8	19	1.87967
113	19JUN2009	night	8	19	1.82152
113	19JUN2009	twilight	8	19	1.67487
113	19JUN2009	day	8	19	1.40995
113	24JUN2009	day	1	19	0.60970
113	24JUN2009	day	1	19	0.35430
113	24JUN2009	day	1	19	0.08672
113	24JUN2009	twilight	1	19	0.40329
113	24JUN2009	night	1	19	0.82419
113	25JUN2009	night	2	19	0.70079
113	25JUN2009	twilight	2	19	0.58670
113	25JUN2009	day	2	19	1.27878
113	25JUN2009	day	2	19	1.24306
113	25JUN2009	day	2	19	0.90800
113	25JUN2009	day	2	19	1.04458
113	25JUN2009	twilight	2	19	1.39447
113	25JUN2009	night	2	19	1.22013
113	26JUN2009	night	2	19	1.40656
113	26JUN2009	twilight	2	19	1.38383
113	26JUN2009	day	2	19	1.00864
114	03JUN2009	day	4	17	1.11062
114	03JUN2009	day	4	17	0.90639
114	03JUN2009	day	4	17	0.98322
114	03JUN2009	twilight	4	17	2.13988
114	03JUN2009	night	4	17	1.62119
114	04JUN2009	night	4	17	1.34832
114	04JUN2009	twilight	4	17	1.27878
114	04JUN2009	day	4	17	0.52517
114	04JUN2009	day	4	17	1.83948
114	04JUN2009	day	4	17	2.42325
114	04JUN2009	day	4	17	2.13988
114	04JUN2009	twilight	4	17	1.04143
114	04JUN2009	night	4	17	1.48715
114	05JUN2009	night	4	17	1.38383
114	05JUN2009	twilight	4	17	2.40140
114	05JUN2009	day	4	17	2.41996
114	17JUN2009	day	6	17	2.56467

114	17JUN2009	day	6	17	2.20140
114	17JUN2009	day	6	17	1.81492
114	17JUN2009	twilight	6	17	1.53531
114	17JUN2009	night	6	17	1.20954
114	18JUN2009	night	6	17	0.74827
114	18JUN2009	twilight	6	17	2.16436
114	18JUN2009	day	6	17	1.54284
114	18JUN2009	day	6	17	1.39796
114	18JUN2009	day	6	17	1.52893
114	18JUN2009	day	6	17	1.79727
114	18JUN2009	twilight	6	17	1.86688
114	18JUN2009	night	6	17	1.45026
114	19JUN2009	night	6	17	1.65897
114	19JUN2009	twilight	6	17	1.50516
114	19JUN2009	day	6	17	2.08279
115	03JUN2009	day	4	14	0.25551
115	03JUN2009	day	4	14	0.62747
115	03JUN2009	day	4	14	0.31408
115	03JUN2009	twilight	4	14	0.79246
115	03JUN2009	night	4	14	-0.15428
115	04JUN2009	night	4	14	-0.14206
115	04JUN2009	twilight	4	14	0.63458
115	04JUN2009	day	4	14	0.89713
115	04JUN2009	day	4	14	1.78605
115	04JUN2009	day	4	14	0.54913
115	04JUN2009	day	4	14	0.60433
115	04JUN2009	twilight	4	14	1.67762
115	04JUN2009	day	4	14	0.51865
115	05JUN2009	night	4	14	1.39796
115	05JUN2009	twilight	4	14	1.42977
115	05JUN2009	day	4	14	1.39095
115	10JUN2009	day	6	14	1.27648
115	10JUN2009	day	6	14	1.26484
115	10JUN2009	day	6	14	1.57865
115	10JUN2009	twilight	6	14	0.72272
115	10JUN2009	night	6	14	1.60532
115	11JUN2009	night	6	14	1.54159
115	11JUN2009	twilight	6	14	1.12388
115	11JUN2009	day	6	14	0.54790
115	11JUN2009	day	6	14	0.98322
115	11JUN2009	day	6	14	1.53909
115	11JUN2009	day	6	14	1.49832
115	11JUN2009	twilight	6	14	0.97317
115	11JUN2009	night	6	14	0.70079
115	12JUN2009	night	6	14	1.19315
115	12JUN2009	twilight	6	14	1.41332
115	12JUN2009	day	6	14	1.70673
115	17JUN2009	day	8	14	0.39811
115	17JUN2009	day	8	14	0.39811
115	17JUN2009	day	8	14	0.53161
115	17JUN2009	twilight	8	14	0.92639
115	17JUN2009	night	8	14	1.19036
115	18JUN2009	night	8	14	0.98682
115	18JUN2009	twilight	8	14	1.02123
115	18JUN2009	day	8	14	0.69469
115	18JUN2009	day	8	14	1.04143

115	18JUN2009	day	8	14	0.64552
115	18JUN2009	day	8	14	0.98816
115	18JUN2009	twilight	8	14	0.04571
115	18JUN2009	night	8	14	0.01326
115	19JUN2009	night	8	14	0.96572
115	19JUN2009	twilight	8	14	1.02942
115	19JUN2009	day	8	14	0.38399
115	24JUN2009	day	1	14	0.89878
115	24JUN2009	day	1	14	0.30125
115	24JUN2009	day	1	14	0.62335
115	24JUN2009	twilight	1	14	1.03747
115	24JUN2009	night	1	14	1.24306
115	25JUN2009	night	2	14	1.02535
115	25JUN2009	twilight	2	14	1.49556
115	25JUN2009	day	2	14	0.71692
115	25JUN2009	day	2	14	1.42815
115	25JUN2009	day	2	14	1.15232
115	25JUN2009	day	2	14	0.91174
115	25JUN2009	twilight	2	14	0.91068
115	25JUN2009	night	2	14	0.47727
115	26JUN2009	night	2	14	0.78398
115	26JUN2009	twilight	2	14	1.11730
115	26JUN2009	day	2	14	0.55157
116	03JUN2009	day	4	60	1.26720
116	03JUN2009	day	4	60	1.22534
116	03JUN2009	day	4	60	1.24306
116	03JUN2009	twilight	4	60	0.61077
116	03JUN2009	night	4	60	1.00436
116	04JUN2009	night	4	60	1.91010
116	04JUN2009	twilight	4	60	1.62326
116	04JUN2009	day	4	60	1.75436
116	04JUN2009	day	4	60	1.92065
116	04JUN2009	day	4	60	1.23807
116	04JUN2009	day	4	60	0.89933
116	04JUN2009	twilight	4	60	0.12743
116	04JUN2009	night	4	60	0.68224
116	05JUN2009	night	4	60	0.54295
116	05JUN2009	twilight	4	60	0.88824
116	05JUN2009	day	4	60	0.61500
116	10JUN2009	day	6	60	0.84948
116	10JUN2009	day	6	60	1.19868
116	10JUN2009	day	6	60	1.52376
116	10JUN2009	twilight	6	60	1.38741
116	10JUN2009	night	6	60	0.00903
116	11JUN2009	night	6	60	0.32243
116	11JUN2009	twilight	6	60	1.17901
116	11JUN2009	day	6	60	0.68494
116	11JUN2009	day	6	60	1.16438
116	11JUN2009	day	6	60	0.87628
116	11JUN2009	day	6	60	1.21751
116	11JUN2009	twilight	6	60	0.95670
116	11JUN2009	night	6	60	0.56478
116	12JUN2009	night	6	60	1.36923
116	12JUN2009	twilight	6	60	0.62335
116	12JUN2009	day	6	60	0.45194
116	17JUN2009	day	8	60	2.48430

116	17JUN2009	day	8	60	2.48430
116	17JUN2009	day	8	60	0.92742
117	03JUN2009	day	4	29	1.08282
117	03JUN2009	day	4	29	0.95429
117	03JUN2009	day	4	29	1.28558
117	03JUN2009	twilight	4	29	0.63458
117	03JUN2009	night	4	29	0.92070
117	04JUN2009	night	4	29	0.96478
117	04JUN2009	twilight	4	29	0.84392
117	04JUN2009	day	4	29	0.89215
117	04JUN2009	day	4	29	0.95766
117	04JUN2009	day	4	29	1.12714
117	04JUN2009	day	4	29	0.92070
117	04JUN2009	twilight	4	29	0.48586
117	04JUN2009	night	4	29	0.68762
117	05JUN2009	night	4	29	0.45954
117	05JUN2009	twilight	4	29	0.69906
117	05JUN2009	day	4	29	-0.09583
117	10JUN2009	day	6	29	0.64058
117	10JUN2009	day	6	29	1.08640
117	10JUN2009	day	6	29	1.27418
117	10JUN2009	twilight	6	29	1.33246
117	10JUN2009	night	6	29	0.76125
117	11JUN2009	night	6	29	0.12743
117	11JUN2009	twilight	6	29	0.74904
117	11JUN2009	day	6	29	0.70766
117	11JUN2009	day	6	29	1.17612
117	11JUN2009	day	6	29	0.99874
117	11JUN2009	day	6	29	1.45180
117	11JUN2009	twilight	6	29	0.17638
117	11JUN2009	night	6	29	-0.02411
117	12JUN2009	night	6	29	1.07558
117	12JUN2009	twilight	6	29	0.95477
117	12JUN2009	day	6	29	0.76125
117	17JUN2009	day	8	29	1.48715
117	17JUN2009	day	8	29	0.48586
117	17JUN2009	day	8	29	0.70338
117	17JUN2009	twilight	8	29	0.65427
117	17JUN2009	night	8	29	1.01708
117	18JUN2009	night	8	29	0.66474
117	18JUN2009	twilight	8	29	0.96478
117	18JUN2009	day	8	29	0.94453
117	18JUN2009	day	8	29	0.60756
117	18JUN2009	day	8	29	1.00436
117	18JUN2009	day	8	29	0.32858
117	18JUN2009	twilight	8	29	1.11062
117	18JUN2009	night	8	29	1.16438
117	19JUN2009	night	8	29	0.59561
117	19JUN2009	twilight	8	29	1.07192
117	19JUN2009	day	8	29	1.25770
117	24JUN2009	day	1	29	1.47131
117	24JUN2009	day	1	29	1.00004
117	24JUN2009	day	1	29	0.96900
117	24JUN2009	twilight	1	29	1.15232
117	24JUN2009	night	1	29	1.10384
117	25JUN2009	night	2	29	0.33465

117	25JUN2009	twilight	2	29	1.88082
117	25JUN2009	day	2	29	1.49138
117	25JUN2009	day	2	29	1.48574
117	25JUN2009	day	2	29	0.83891
117	25JUN2009	day	2	29	0.66474
117	25JUN2009	twilight	2	29	0.80625
117	25JUN2009	night	2	29	0.74515
117	26JUN2009	night	2	29	1.06450
117	26JUN2009	twilight	2	29	0.65619
117	26JUN2009	day	2	29	1.11062
118	03JUN2009	night	4	41	1.83823
118	04JUN2009	night	4	41	1.44562
118	04JUN2009	twilight	4	41	0.34850
118	04JUN2009	day	4	41	0.81431
118	04JUN2009	day	4	41	2.39094
118	04JUN2009	day	4	41	1.92065
118	04JUN2009	day	4	41	1.40485
118	04JUN2009	twilight	4	41	2.14613
118	04JUN2009	night	4	41	2.27416
118	05JUN2009	night	4	41	2.26245
118	05JUN2009	twilight	4	41	1.97313
118	05JUN2009	day	4	41	2.11059
118	10JUN2009	day	6	41	2.77306
118	10JUN2009	day	6	41	2.73719
118	10JUN2009	day	6	41	0.74280
118	10JUN2009	twilight	6	41	1.78104
118	10JUN2009	night	6	41	1.02123
118	11JUN2009	night	6	41	2.19866
118	11JUN2009	twilight	6	41	2.17898
118	11JUN2009	day	6	41	2.19866
118	11JUN2009	day	6	41	1.88196
118	11JUN2009	day	6	41	2.13034
118	11JUN2009	day	6	41	2.18185
118	11JUN2009	twilight	6	41	1.18472
118	11JUN2009	night	6	41	1.19315
118	12JUN2009	night	6	41	1.91699
118	12JUN2009	twilight	6	41	0.94748
118	12JUN2009	day	6	41	2.13672
118	17JUN2009	day	8	41	1.35985
118	17JUN2009	day	8	41	1.91751
118	17JUN2009	day	8	41	1.51984
118	17JUN2009	twilight	8	41	2.13672
118	17JUN2009	night	8	41	2.31176
118	18JUN2009	night	8	41	2.36549
118	18JUN2009	twilight	8	41	1.06450
118	18JUN2009	day	8	41	2.22789
118	18JUN2009	day	8	41	2.26717
118	18JUN2009	day	8	41	1.07558
118	18JUN2009	day	8	41	2.13034
118	18JUN2009	twilight	8	41	2.04922
118	18JUN2009	night	8	41	1.40656
118	19JUN2009	night	8	41	1.49556
118	19JUN2009	twilight	8	41	1.59551
118	19JUN2009	day	8	41	1.58884
118	24JUN2009	day	1	41	2.09691
118	24JUN2009	day	1	41	0.85800

118	24JUN2009	day	1	41	2.37475
118	24JUN2009	twilight	1	41	1.49277
118	24JUN2009	night	1	41	1.30322
118	25JUN2009	night	2	41	1.46091
118	25JUN2009	twilight	2	41	1.53149
118	25JUN2009	day	2	41	1.48997
118	25JUN2009	day	2	41	1.28106
118	25JUN2009	day	2	41	1.49277
118	25JUN2009	day	2	41	2.33244
118	25JUN2009	twilight	2	41	1.32224
118	25JUN2009	night	2	41	2.19033
118	26JUN2009	night	2	41	2.18752
118	26JUN2009	twilight	2	41	2.31387
118	26JUN2009	day	2	41	1.39796
119	08JUN2006	day	4	94	1.46836
119	08JUN2006	day	4	94	1.95037
119	08JUN2006	day	4	94	1.95761
119	08JUN2006	twilight	4	94	1.78534
119	08JUN2006	night	4	94	1.85854
119	09JUN2006	night	4	94	1.56939
119	09JUN2006	twilight	4	94	1.25770
119	09JUN2006	day	4	94	0.99127
119	09JUN2006	day	4	94	0.87512
119	09JUN2006	day	4	94	0.90314
119	09JUN2006	day	4	94	0.93202
119	09JUN2006	twilight	4	94	1.13675
119	09JUN2006	night	4	94	1.65129
119	10JUN2006	night	4	94	1.65032
119	10JUN2006	twilight	4	94	1.36923
119	12JUN2006	day	6	94	0.99699
119	12JUN2006	day	6	94	0.97594
119	12JUN2006	twilight	6	94	1.42977
119	12JUN2006	night	6	94	0.20439
119	13JUN2006	night	6	94	1.51056
119	13JUN2006	twilight	6	94	1.15537
119	13JUN2006	day	6	94	1.43299
119	13JUN2006	day	6	94	1.01708
119	13JUN2006	day	6	94	0.19340
119	13JUN2006	day	6	94	1.32224
119	13JUN2006	twilight	6	94	1.04926
119	13JUN2006	night	6	94	1.13675
119	14JUN2006	night	6	94	1.67762
119	14JUN2006	twilight	6	94	0.30125
119	19JUN2006	day	7	94	1.24800
119	19JUN2006	day	7	94	1.51056
119	19JUN2006	day	7	94	1.53657
119	19JUN2006	twilight	7	94	2.07189
119	19JUN2006	night	7	94	2.07189
119	20JUN2006	night	7	94	1.26484
119	20JUN2006	twilight	7	94	1.26720
119	20JUN2006	day	7	94	1.94052
119	20JUN2006	day	7	94	2.00433
119	20JUN2006	day	7	94	1.25044
119	20JUN2006	day	7	94	1.46091
119	20JUN2006	day	7	94	1.56230
119	20JUN2006	twilight	7	94	2.08636

119	20JUN2006	night	7	94	2.12711
119	21JUN2006	night	7	94	1.48289
119	21JUN2006	twilight	7	94	0.98005
119	03JUN2009	day	4	88	1.74195
119	03JUN2009	day	4	88	1.33043
119	03JUN2009	day	4	88	1.16438
119	03JUN2009	twilight	4	88	0.68224
119	03JUN2009	night	4	88	0.74123
119	04JUN2009	night	4	88	1.13037
119	04JUN2009	twilight	4	88	1.06450
119	04JUN2009	day	4	88	1.36363
119	04JUN2009	day	4	88	2.01284
119	04JUN2009	day	4	88	1.76194
119	04JUN2009	day	4	88	1.41164
119	04JUN2009	twilight	4	88	1.43777
119	04JUN2009	night	4	88	0.73167
119	05JUN2009	night	4	88	1.44406
119	05JUN2009	twilight	4	88	1.49694
119	05JUN2009	day	4	88	1.63145
119	10JUN2009	day	6	88	0.82354
119	10JUN2009	day	6	88	1.77012
119	10JUN2009	day	6	88	1.85492
119	10JUN2009	twilight	6	88	0.45194
119	10JUN2009	night	6	88	1.41666
119	11JUN2009	night	6	88	1.61279
119	11JUN2009	twilight	6	88	0.22814
119	11JUN2009	day	6	88	1.58434
119	11JUN2009	day	6	88	1.03346
119	11JUN2009	day	6	88	1.41164
119	11JUN2009	day	6	88	1.37293
119	11JUN2009	twilight	6	88	1.62326
119	11JUN2009	night	6	88	0.62024
119	12JUN2009	night	6	88	1.56468
119	12JUN2009	twilight	6	88	1.55992
119	12JUN2009	day	6	88	1.08640
119	17JUN2009	day	8	88	1.58996
119	17JUN2009	day	8	88	1.22274
119	17JUN2009	day	8	88	1.65226
119	17JUN2009	twilight	8	88	1.75968
119	17JUN2009	night	8	88	1.12061
119	18JUN2009	night	8	88	1.30752
119	18JUN2009	twilight	8	88	1.35795
119	18JUN2009	day	8	88	1.62222
119	18JUN2009	day	8	88	1.37842
119	18JUN2009	day	8	88	1.36737
119	18JUN2009	day	8	88	1.57172
119	18JUN2009	twilight	8	88	0.91598
119	18JUN2009	night	8	88	1.15537
119	19JUN2009	night	8	88	1.20142
119	19JUN2009	twilight	8	88	1.21221
119	19JUN2009	day	8	88	1.48431
119	24JUN2009	day	1	88	0.40671
119	24JUN2009	day	1	88	0.06108
119	24JUN2009	day	1	88	0.38757
119	24JUN2009	twilight	1	88	1.00436
119	24JUN2009	night	1	88	0.87512

119	25JUN2009	night	2	88	0.35044
119	25JUN2009	twilight	2	88	1.31599
119	25JUN2009	day	2	88	1.30105
119	25JUN2009	day	2	88	1.43138
119	25JUN2009	day	2	88	2.13354
119	25JUN2009	day	2	88	2.26007
119	25JUN2009	twilight	2	88	1.52246
119	25JUN2009	night	2	88	1.87853
119	26JUN2009	night	2	88	1.29669
119	26JUN2009	twilight	2	88	1.32224
119	26JUN2009	day	2	88	1.65226
120	08JUN2006	day	4	82	1.62635
120	08JUN2006	day	4	82	0.99568
120	08JUN2006	day	4	82	1.29669
120	08JUN2006	twilight	4	82	0.76500
120	08JUN2006	night	4	82	1.20685
120	09JUN2006	night	4	82	1.45790
120	09JUN2006	twilight	4	82	1.53657
120	09JUN2006	day	4	82	1.58094
120	09JUN2006	day	4	82	1.56111
120	09JUN2006	day	4	82	1.10384
120	09JUN2006	twilight	4	82	1.22791
120	09JUN2006	night	4	82	2.21801
120	10JUN2006	night	4	82	1.90526
120	10JUN2006	twilight	4	82	.
120	03JUN2009	day	4	24	1.77888
120	03JUN2009	day	4	24	1.86392
120	03JUN2009	day	4	24	1.63247
120	03JUN2009	twilight	4	24	1.58207
120	03JUN2009	night	4	24	1.81889
120	04JUN2009	night	4	24	1.90201
120	04JUN2009	twilight	4	24	1.15537
120	04JUN2009	day	4	24	0.03383
120	04JUN2009	day	4	24	0.82937
120	04JUN2009	day	4	24	0.59229
120	04JUN2009	day	4	24	-0.04721
120	04JUN2009	twilight	4	24	1.17029
120	04JUN2009	night	4	24	1.24057
120	05JUN2009	night	4	24	1.91908
120	05JUN2009	twilight	4	24	1.87853
120	05JUN2009	day	4	24	0.83512
120	10JUN2009	day	6	24	1.32636
120	10JUN2009	day	6	24	1.24057
120	10JUN2009	day	6	24	0.71273
120	10JUN2009	twilight	6	24	0.70766
120	10JUN2009	night	6	24	1.08994
120	11JUN2009	night	6	24	0.40329
120	11JUN2009	twilight	6	24	0.78540
120	11JUN2009	day	6	24	0.85739
120	11JUN2009	day	6	24	1.36923
120	11JUN2009	day	6	24	1.42490
120	11JUN2009	day	6	24	0.31408
120	11JUN2009	twilight	6	24	0.91068
120	11JUN2009	night	6	24	0.58894
120	12JUN2009	night	6	24	1.16438
120	12JUN2009	twilight	6	24	1.29228

120	12JUN2009	day	6	24	0.96993
120	17JUN2009	day	8	24	1.45180
120	17JUN2009	day	8	24	1.24057
120	17JUN2009	day	8	24	1.02535
120	17JUN2009	twilight	8	24	1.29669
120	17JUN2009	night	8	24	1.34046
120	18JUN2009	night	8	24	0.26505
120	18JUN2009	twilight	8	24	0.96900
120	18JUN2009	day	8	24	0.73568
120	18JUN2009	day	8	24	0.41681
120	18JUN2009	day	8	24	0.72518
120	18JUN2009	day	8	24	0.96806
120	18JUN2009	twilight	8	24	1.01708
120	18JUN2009	night	8	24	0.31408
120	19JUN2009	night	8	24	0.77822
120	19JUN2009	twilight	8	24	0.81298
120	19JUN2009	day	8	24	0.97410
121	08JUN2006	day	4	68	1.63247
121	08JUN2006	day	4	68	1.25288
121	08JUN2006	day	4	68	1.39447
121	08JUN2006	twilight	4	68	1.23302
121	08JUN2006	night	4	68	0.82484
121	09JUN2006	night	4	68	0.82743
121	09JUN2006	twilight	4	68	1.45790
121	09JUN2006	day	4	68	1.26010
121	09JUN2006	day	4	68	1.21487
121	09JUN2006	day	4	68	1.09346
121	09JUN2006	twilight	4	68	1.27186
121	09JUN2006	night	4	68	1.47858
121	10JUN2006	night	4	68	1.38918
121	10JUN2006	twilight	4	68	1.44562
121	12JUN2006	day	6	68	1.79866
121	12JUN2006	twilight	6	68	1.52246
121	12JUN2006	night	6	68	1.37660
121	13JUN2006	night	6	68	1.38204
121	13JUN2006	twilight	6	68	1.33447
121	13JUN2006	day	6	68	1.25770
121	13JUN2006	day	6	68	0.98726
121	13JUN2006	day	6	68	1.11398
121	13JUN2006	day	6	68	0.60217
121	13JUN2006	twilight	6	68	0.76125
121	13JUN2006	night	6	68	0.95718
121	14JUN2006	night	6	68	1.46688
121	14JUN2006	twilight	6	68	1.55146
121	19JUN2006	day	7	68	0.98502
121	19JUN2006	day	7	68	1.44562
121	19JUN2006	day	7	68	1.53149
121	19JUN2006	twilight	7	68	2.10037
121	19JUN2006	night	7	68	2.11059
121	20JUN2006	night	7	68	1.01708
121	20JUN2006	twilight	7	68	1.16438
121	20JUN2006	day	7	68	1.96237
121	20JUN2006	day	7	68	2.00433
121	20JUN2006	day	7	68	1.35413
121	20JUN2006	day	7	68	1.51984
121	20JUN2006	day	7	68	1.26720

121	20JUN2006	twilight	7	68	2.03743
121	20JUN2006	night	7	68	2.08991
121	21JUN2006	night	7	68	1.36551
121	21JUN2006	twilight	7	68	1.37109
121	26JUN2006	day	2	68	0.49982
121	26JUN2006	day	2	68	1.33648
121	26JUN2006	day	2	68	1.49277
121	26JUN2006	twilight	2	68	1.82543
121	26JUN2006	night	2	68	2.04533
121	27JUN2006	night	2	68	1.80619
121	27JUN2006	twilight	2	68	1.06450
121	27JUN2006	day	2	68	1.05312
121	27JUN2006	day	2	68	1.22534
121	27JUN2006	day	2	68	1.21221
121	27JUN2006	day	2	68	1.20685
121	27JUN2006	twilight	2	68	1.76418
121	27JUN2006	night	2	68	1.38383
121	28JUN2006	night	2	68	1.59551
121	28JUN2006	twilight	2	68	0.68404
121	28JUN2006	day	2	68	0.88824
121	03JUN2009	day	4	40	1.35985
121	03JUN2009	day	4	40	0.44107
121	03JUN2009	day	4	40	1.04536
121	03JUN2009	twilight	4	40	1.07922
121	03JUN2009	night	4	40	1.70502
121	04JUN2009	night	4	40	1.14616
121	04JUN2009	twilight	4	40	1.13991
121	04JUN2009	day	4	40	1.96332
121	04JUN2009	day	4	40	1.89928
121	04JUN2009	day	4	40	0.93907
121	04JUN2009	day	4	40	0.56714
121	04JUN2009	day	4	40	-0.04721
121	04JUN2009	twilight	4	40	0.71525
121	05JUN2009	night	4	40	0.92536
121	05JUN2009	twilight	4	40	-0.14448
121	05JUN2009	day	4	40	0.68762
121	10JUN2009	day	6	40	2.10721
121	10JUN2009	day	6	40	0.64748
121	10JUN2009	day	6	40	1.15839
121	10JUN2009	twilight	6	40	1.26484
121	10JUN2009	night	6	40	1.47131
121	11JUN2009	night	6	40	1.11062
121	11JUN2009	twilight	6	40	1.13991
121	11JUN2009	day	6	40	0.68404
121	11JUN2009	day	6	40	0.66755
121	11JUN2009	day	6	40	1.04926
121	11JUN2009	day	6	40	1.14305
121	11JUN2009	twilight	6	40	1.27878
121	11JUN2009	night	6	40	0.72024
121	12JUN2009	night	6	40	1.09346
121	12JUN2009	twilight	6	40	0.94699
121	12JUN2009	day	6	40	0.81431
121	17JUN2009	day	8	40	1.44717
121	17JUN2009	day	8	40	0.73648
121	17JUN2009	day	8	40	1.35413
121	17JUN2009	twilight	8	40	0.57183

121	17JUN2009	night	8	40	1.18755
121	18JUN2009	night	8	40	1.06450
121	18JUN2009	twilight	8	40	0.86576
121	18JUN2009	day	8	40	1.08282
121	18JUN2009	day	8	40	1.26484
121	18JUN2009	day	8	40	0.63458
121	18JUN2009	day	8	40	1.03346
121	18JUN2009	twilight	8	40	1.07192
121	18JUN2009	night	8	40	0.71609
121	19JUN2009	night	8	40	0.87800
121	19JUN2009	twilight	8	40	0.71525
121	19JUN2009	day	8	40	1.32224
122	08JUN2006	day	4	62	0.83321
122	08JUN2006	day	4	62	-0.08302
122	08JUN2006	day	4	62	0.89878
122	08JUN2006	twilight	4	62	1.36551
122	08JUN2006	night	4	62	1.10384
122	09JUN2006	night	4	62	0.96004
122	09JUN2006	twilight	4	62	1.07558
122	09JUN2006	day	4	62	1.26010
122	09JUN2006	day	4	62	0.84267
122	09JUN2006	day	4	62	1.05312
122	09JUN2006	twilight	4	62	1.27648
122	09JUN2006	night	4	62	1.31178
122	10JUN2006	night	4	62	1.37293
122	10JUN2006	twilight	4	62	1.18755
122	12JUN2006	day	6	62	1.35413
122	12JUN2006	day	6	62	1.25770
122	12JUN2006	twilight	6	62	1.83633
122	12JUN2006	night	6	62	1.62326
122	13JUN2006	night	6	62	1.07558
122	13JUN2006	twilight	6	62	1.26010
122	13JUN2006	day	6	62	1.65032
122	13JUN2006	day	6	62	1.53277
122	13JUN2006	day	6	62	1.26010
122	13JUN2006	day	6	62	0.50934
122	13JUN2006	twilight	6	62	0.55157
122	13JUN2006	night	6	62	1.23047
122	14JUN2006	night	6	62	1.04536
122	14JUN2006	twilight	6	62	1.54902
122	19JUN2006	day	7	62	0.93151
122	19JUN2006	day	7	62	1.04926
122	19JUN2006	day	7	62	1.08282
122	19JUN2006	twilight	7	62	1.22013
122	19JUN2006	night	7	62	0.87105
122	20JUN2006	night	7	62	1.39271
122	20JUN2006	twilight	7	62	1.34244
122	20JUN2006	day	7	62	1.66371
122	20JUN2006	day	7	62	1.74116
122	20JUN2006	day	7	62	0.78039
122	20JUN2006	day	7	62	1.41666
122	20JUN2006	day	7	62	1.27186
122	20JUN2006	twilight	7	62	1.47569
122	20JUN2006	night	7	62	1.55511
122	21JUN2006	night	7	62	0.75136
122	21JUN2006	twilight	7	62	1.04926

122	26JUN2006	day	2	62	0.71775
122	26JUN2006	day	2	62	0.84640
122	26JUN2006	day	2	62	1.29669
122	26JUN2006	twilight	2	62	1.59108
122	26JUN2006	night	2	62	1.65032
122	27JUN2006	night	2	62	1.70673
122	27JUN2006	twilight	2	62	1.35795
122	27JUN2006	day	2	62	0.96478
122	27JUN2006	day	2	62	1.09694
122	27JUN2006	day	2	62	0.49982
122	27JUN2006	day	2	62	1.00004
122	27JUN2006	twilight	2	62	1.95280
122	27JUN2006	night	2	62	0.75136
122	28JUN2006	night	2	62	1.92428
122	28JUN2006	twilight	2	62	1.51190
122	28JUN2006	day	2	62	1.62738
122	03JUN2009	day	4	34	1.12714
122	03JUN2009	day	4	34	0.69028
122	03JUN2009	day	4	34	1.51190
122	03JUN2009	twilight	4	34	1.34637
122	03JUN2009	night	4	34	1.06074
122	04JUN2009	night	4	34	1.22013
122	04JUN2009	twilight	4	34	1.09346
122	04JUN2009	day	4	34	0.87046
122	04JUN2009	day	4	34	1.26247
122	04JUN2009	day	4	34	0.96289
122	04JUN2009	day	4	34	0.77822
122	04JUN2009	twilight	4	34	1.74664
122	04JUN2009	night	4	34	1.71097
122	05JUN2009	night	4	34	0.68762
122	05JUN2009	twilight	4	34	0.47727
122	05JUN2009	day	4	34	1.18755
122	10JUN2009	day	6	34	1.21487
122	10JUN2009	day	6	34	1.23555
122	10JUN2009	twilight	6	34	0.59450
122	10JUN2009	night	6	34	0.86219
122	11JUN2009	night	6	34	0.93757
122	11JUN2009	twilight	6	34	0.47144
122	11JUN2009	day	6	34	0.67495
122	11JUN2009	day	6	34	1.35220
122	11JUN2009	day	6	34	1.14616
122	11JUN2009	day	6	34	0.90314
122	11JUN2009	twilight	6	34	0.26741
122	11JUN2009	night	6	34	0.41514
122	12JUN2009	night	6	34	0.36940
122	12JUN2009	twilight	6	34	0.41514
122	12JUN2009	day	6	34	0.77822
122	17JUN2009	day	8	34	0.85800
122	17JUN2009	day	8	34	1.16140
122	17JUN2009	day	8	34	0.79386
122	17JUN2009	twilight	8	34	0.93151
122	17JUN2009	night	8	34	0.15259
122	18JUN2009	night	8	34	0.86753
122	18JUN2009	twilight	8	34	0.77895
122	18JUN2009	day	8	34	1.32017
122	18JUN2009	day	8	34	1.45026

122	18JUN2009	day	8	34	0.87280
122	18JUN2009	day	8	34	0.69992
122	18JUN2009	twilight	8	34	0.80284
122	18JUN2009	night	8	34	1.38023
122	19JUN2009	night	8	34	1.16140
122	19JUN2009	twilight	8	34	0.42667
122	19JUN2009	day	8	34	0.87105
122	24JUN2009	day	1	34	0.32858
122	24JUN2009	day	1	34	0.77459
122	24JUN2009	day	1	34	0.87163
122	24JUN2009	twilight	1	34	0.79106
122	24JUN2009	night	1	34	0.98905
122	25JUN2009	night	2	34	1.09346
122	25JUN2009	twilight	2	34	1.00004
122	25JUN2009	day	2	34	1.11062
122	25JUN2009	day	2	34	0.69205
122	25JUN2009	day	2	34	1.20954
122	25JUN2009	day	2	34	1.32224
122	25JUN2009	twilight	2	34	1.19868
122	25JUN2009	night	2	34	1.06822
122	26JUN2009	night	2	34	0.10072
122	26JUN2009	twilight	2	34	1.04536
122	26JUN2009	day	2	34	0.72107
123	08JUN2006	day	4	68	0.72599
123	08JUN2006	day	4	68	1.60424
123	08JUN2006	day	4	68	1.40485
123	08JUN2006	twilight	4	68	1.17901
123	08JUN2006	night	4	68	1.31599
123	09JUN2006	night	4	68	1.30965
123	09JUN2006	twilight	4	68	1.65897
123	09JUN2006	day	4	68	1.96143
123	09JUN2006	day	4	68	1.10384
123	09JUN2006	day	4	68	0.58670
123	09JUN2006	twilight	4	68	1.84943
123	09JUN2006	night	4	68	1.98767
123	10JUN2006	night	4	68	2.08279
123	10JUN2006	twilight	4	68	1.60532
123	12JUN2006	day	6	68	1.22013
123	12JUN2006	day	6	68	1.35985
123	12JUN2006	twilight	6	68	1.40314
123	12JUN2006	night	6	68	1.07558
123	13JUN2006	night	6	68	1.69374
123	13JUN2006	twilight	6	68	1.81492
123	13JUN2006	day	6	68	1.23302
123	13JUN2006	day	6	68	0.51733
123	13JUN2006	day	6	68	1.06450
123	13JUN2006	day	6	68	0.68133
123	13JUN2006	twilight	6	68	1.03346
123	13JUN2006	night	6	68	1.09346
123	14JUN2006	night	6	68	1.02535
123	14JUN2006	twilight	6	68	1.78534
123	19JUN2006	day	7	68	1.22013
123	19JUN2006	day	7	68	1.13037
123	19JUN2006	day	7	68	1.13357
123	19JUN2006	twilight	7	68	1.28782
123	19JUN2006	night	7	68	0.26505

123	20JUN2006	night	7	68	1.14305
123	20JUN2006	twilight	7	68	1.09346
123	20JUN2006	day	7	68	1.50787
123	20JUN2006	day	7	68	1.46391
123	20JUN2006	day	7	68	0.98322
123	20JUN2006	day	7	68	0.93100
123	20JUN2006	day	7	68	0.66848
123	20JUN2006	twilight	7	68	1.89873
123	20JUN2006	night	7	68	1.92169
123	21JUN2006	night	7	68	1.39447
123	21JUN2006	twilight	7	68	1.68665
123	26JUN2006	day	2	68	0.32243
123	26JUN2006	day	2	68	1.13037
123	26JUN2006	day	2	68	1.07922
123	26JUN2006	twilight	2	68	1.79935
123	26JUN2006	night	2	68	1.70071
123	27JUN2006	night	2	68	1.90146
123	27JUN2006	twilight	2	68	1.48715
123	27JUN2006	day	2	68	1.59661
123	27JUN2006	day	2	68	1.60424
123	27JUN2006	day	2	68	0.44731
123	27JUN2006	day	2	68	0.84516
123	27JUN2006	twilight	2	68	1.99652
123	27JUN2006	night	2	68	0.96004
123	28JUN2006	night	2	68	1.99695
123	28JUN2006	twilight	2	68	1.75816
123	28JUN2006	day	2	68	1.73879
123	03JUN2009	day	4	33	0.68494
123	03JUN2009	day	4	33	1.82152
123	03JUN2009	day	4	33	0.71189
123	03JUN2009	twilight	4	33	2.11727
123	03JUN2009	night	4	33	2.12058
123	04JUN2009	night	4	33	1.55992
123	04JUN2009	twilight	4	33	1.92325
123	04JUN2009	day	4	33	1.98678
123	04JUN2009	day	4	33	0.97502
123	04JUN2009	day	4	33	0.84948
123	04JUN2009	day	4	33	1.32636
123	04JUN2009	twilight	4	33	0.88144
123	04JUN2009	night	4	33	1.58996
123	05JUN2009	night	4	33	1.67118
123	05JUN2009	twilight	4	33	1.54656
123	05JUN2009	day	4	33	0.61606
123	10JUN2009	day	6	33	0.73965
123	10JUN2009	day	6	33	1.26010
123	10JUN2009	day	6	33	1.79866
123	10JUN2009	twilight	6	33	0.86159
123	10JUN2009	night	6	33	1.28332
123	11JUN2009	night	6	33	1.62119
123	11JUN2009	twilight	6	33	1.67118
123	11JUN2009	day	6	33	0.99260
123	11JUN2009	day	6	33	1.56821
123	11JUN2009	day	6	33	1.90795
123	11JUN2009	day	6	33	0.74202
123	11JUN2009	twilight	6	33	1.39796
123	11JUN2009	night	6	33	1.50380

123	12JUN2009	night	6	33	1.26010
123	12JUN2009	twilight	6	33	2.20952
123	12JUN2009	day	6	33	2.22272
123	17JUN2009	day	8	33	0.66941
123	17JUN2009	day	8	33	0.19340
123	17JUN2009	day	8	33	0.91861
123	17JUN2009	twilight	8	33	2.95617
123	17JUN2009	night	8	33	2.97727
123	18JUN2009	night	8	33	-3.00000
123	18JUN2009	twilight	8	33	-3.00000
123	18JUN2009	day	8	33	1.84696
123	18JUN2009	day	8	33	0.73167
123	18JUN2009	day	8	33	0.69205
123	18JUN2009	day	8	33	1.32636
123	18JUN2009	twilight	8	33	0.57530
123	18JUN2009	night	8	33	0.98277
123	19JUN2009	night	8	33	1.02123
123	19JUN2009	twilight	8	33	1.82931
123	19JUN2009	day	8	33	0.90423
123	24JUN2009	day	1	33	0.14953
123	24JUN2009	day	1	33	0.67219
123	24JUN2009	day	1	33	0.64650
123	24JUN2009	twilight	1	33	1.04536
123	24JUN2009	night	1	33	0.48444
123	25JUN2009	night	2	33	0.98096
123	25JUN2009	twilight	2	33	1.07192
123	25JUN2009	day	2	33	0.46997
123	25JUN2009	day	2	33	1.21751
123	25JUN2009	day	2	33	0.72024
123	25JUN2009	day	2	33	1.61596
123	25JUN2009	twilight	2	33	1.59551
123	25JUN2009	night	2	33	0.77093
123	26JUN2009	night	2	33	0.77822
123	26JUN2009	twilight	2	33	0.12743
123	26JUN2009	day	2	33	0.42667
124	03JUN2009	day	4	26	0.76350
124	03JUN2009	day	4	26	0.81431
124	03JUN2009	day	4	26	0.71775
124	03JUN2009	twilight	4	26	0.80147
124	03JUN2009	night	4	26	0.80693
124	04JUN2009	night	4	26	0.89159
124	04JUN2009	twilight	4	26	1.15537
124	04JUN2009	day	4	26	0.51996
124	04JUN2009	day	4	26	1.14305
124	04JUN2009	day	4	26	0.93404
124	04JUN2009	day	4	26	0.71105
124	04JUN2009	twilight	4	26	0.08672
124	04JUN2009	night	4	26	0.75519
124	05JUN2009	night	4	26	1.02535
124	05JUN2009	twilight	4	26	1.28332
124	05JUN2009	day	4	26	1.26010
124	10JUN2009	twilight	6	26	0.89104
124	10JUN2009	night	6	26	0.93757
124	11JUN2009	night	6	26	0.84763
124	11JUN2009	twilight	6	26	0.47871
124	11JUN2009	day	6	26	1.33447

124	11JUN2009	day	6	26	1.41332
124	11JUN2009	day	6	26	1.21487
124	11JUN2009	day	6	26	1.09694
124	11JUN2009	twilight	6	26	0.53161
124	11JUN2009	night	6	26	0.38934
124	12JUN2009	night	6	26	0.25310
124	12JUN2009	twilight	6	26	0.36754
124	12JUN2009	day	6	26	0.05346
124	17JUN2009	day	8	26	1.43458
124	17JUN2009	day	8	26	0.48728
124	17JUN2009	day	8	26	0.21775
124	17JUN2009	twilight	8	26	1.14305
124	17JUN2009	night	8	26	1.09346
124	18JUN2009	night	8	26	1.21751
124	18JUN2009	twilight	8	26	1.30105
124	18JUN2009	day	8	26	1.04536
124	18JUN2009	day	8	26	0.79456
124	18JUN2009	day	8	26	0.85132
124	18JUN2009	day	8	26	0.27898
124	18JUN2009	twilight	8	26	0.90639
124	18JUN2009	night	8	26	0.93049
124	19JUN2009	night	8	26	0.87221
124	19JUN2009	twilight	8	26	0.09726
124	19JUN2009	day	8	26	0.10072
124	24JUN2009	day	1	26	0.40329
124	24JUN2009	day	1	26	0.84080
124	24JUN2009	day	1	26	0.74827
124	24JUN2009	twilight	1	26	1.19315
124	24JUN2009	night	1	26	1.15537
124	25JUN2009	night	2	26	1.07922
124	25JUN2009	twilight	2	26	0.36940
124	25JUN2009	day	2	26	0.62849
124	25JUN2009	day	2	26	0.76125
124	25JUN2009	day	2	26	0.78183
124	25JUN2009	day	2	26	0.57990
124	25JUN2009	twilight	2	26	0.90314
124	25JUN2009	night	2	26	0.56478
124	26JUN2009	night	2	26	0.79316
124	26JUN2009	twilight	2	26	0.55400
124	26JUN2009	day	2	26	0.50934
125	08JUN2006	day	4	64	1.74664
125	08JUN2006	day	4	64	1.48289
125	08JUN2006	day	4	64	1.46241
125	08JUN2006	twilight	4	64	1.96849
125	08JUN2006	night	4	64	1.92428
125	09JUN2006	night	4	64	1.54656
125	09JUN2006	twilight	4	64	1.64049
125	09JUN2006	day	4	64	1.02123
125	09JUN2006	day	4	64	1.36363
125	09JUN2006	day	4	64	1.01708
125	09JUN2006	twilight	4	64	1.31808
125	09JUN2006	night	4	64	2.15534
125	10JUN2006	night	4	64	2.00860
125	10JUN2006	twilight	4	64	1.83886
125	12JUN2006	day	6	64	1.43458
125	12JUN2006	day	6	64	1.34637

125	12JUN2006	twilight	6	64	1.54159
125	12JUN2006	night	6	64	1.58434
125	13JUN2006	night	6	64	1.60854
125	13JUN2006	twilight	6	64	0.82743
125	13JUN2006	day	6	64	0.90369
125	13JUN2006	day	6	64	1.02535
125	13JUN2006	day	6	64	0.26505
125	13JUN2006	day	6	64	0.38399
125	13JUN2006	twilight	6	64	0.99830
125	13JUN2006	night	6	64	1.18755
125	14JUN2006	night	6	64	1.04926
125	14JUN2006	twilight	6	64	0.96993
125	19JUN2006	day	7	64	0.46404
125	19JUN2006	day	7	64	1.17322
125	19JUN2006	day	7	64	1.47277
125	19JUN2006	twilight	7	64	1.10384
125	19JUN2006	night	7	64	1.57520
125	20JUN2006	night	7	64	1.59219
125	20JUN2006	twilight	7	64	1.02942
125	20JUN2006	day	7	64	1.71768
125	20JUN2006	day	7	64	1.68125
125	20JUN2006	day	7	64	0.67587
125	20JUN2006	day	7	64	1.10724
125	20JUN2006	day	7	64	0.39811
125	20JUN2006	twilight	7	64	0.42504
125	20JUN2006	night	7	64	1.48574
125	21JUN2006	night	7	64	1.26484
125	21JUN2006	twilight	7	64	1.30322
125	26JUN2006	day	2	64	1.02942
125	26JUN2006	day	2	64	1.06074
125	26JUN2006	day	2	64	1.30537
125	26JUN2006	twilight	2	64	1.65611
125	26JUN2006	night	2	64	1.96095
125	27JUN2006	night	2	64	1.86154
125	27JUN2006	twilight	2	64	1.35795
125	27JUN2006	day	2	64	0.82743
125	27JUN2006	day	2	64	1.04143
125	27JUN2006	day	2	64	1.13357
125	27JUN2006	day	2	64	0.98050
125	27JUN2006	twilight	2	64	0.96478
125	27JUN2006	night	2	64	0.94156
125	28JUN2006	night	2	64	0.69205
125	28JUN2006	twilight	2	64	1.56586
125	28JUN2006	day	2	64	1.71350
125	03JUN2009	day	4	25	1.29006
125	03JUN2009	day	4	25	0.47727
125	03JUN2009	day	4	25	1.00864
125	03JUN2009	twilight	4	25	1.55390
125	03JUN2009	night	4	25	1.68396
125	04JUN2009	night	4	25	1.19036
125	04JUN2009	twilight	4	25	1.33447
125	04JUN2009	day	4	25	1.43777
125	04JUN2009	day	4	25	1.38741
125	04JUN2009	day	4	25	0.95766
125	04JUN2009	day	4	25	0.51202
125	04JUN2009	twilight	4	25	1.31808

125	04JUN2009	night	4	25	1.69286
125	05JUN2009	night	4	25	1.28106
125	05JUN2009	twilight	4	25	1.32430
125	05JUN2009	day	4	25	1.28558
125	10JUN2009	day	6	25	1.03346
125	10JUN2009	day	6	25	1.03346
125	10JUN2009	day	6	25	1.07922
125	10JUN2009	twilight	6	25	0.87685
125	10JUN2009	night	6	25	1.01288
125	11JUN2009	night	6	25	1.11730
125	11JUN2009	twilight	6	25	0.33666
125	11JUN2009	day	6	25	1.28106
125	11JUN2009	day	6	25	0.92793
125	11JUN2009	day	6	25	0.98322
125	11JUN2009	day	6	25	0.69381
125	11JUN2009	twilight	6	25	0.67587
125	11JUN2009	night	6	25	1.65419
125	12JUN2009	night	6	25	1.69286
125	12JUN2009	twilight	6	25	0.74202
125	12JUN2009	day	6	25	1.73800
125	17JUN2009	day	8	25	0.94993
125	17JUN2009	day	8	25	0.53288
125	17JUN2009	day	8	25	1.36551
125	17JUN2009	twilight	8	25	1.18755
125	17JUN2009	night	8	25	1.32017
125	18JUN2009	night	8	25	1.39095
125	18JUN2009	twilight	8	25	1.10041
125	18JUN2009	day	8	25	0.38039
125	18JUN2009	day	8	25	0.38220
125	18JUN2009	day	8	25	0.42830
125	18JUN2009	day	8	25	0.70680
125	18JUN2009	twilight	8	25	0.99127
125	18JUN2009	night	8	25	0.96946
125	19JUN2009	night	8	25	0.90585
125	19JUN2009	twilight	8	25	0.34459
125	19JUN2009	day	8	25	0.56714
125	24JUN2009	day	1	25	0.57530
125	24JUN2009	day	1	25	0.00043
125	24JUN2009	day	1	25	0.58104
125	24JUN2009	twilight	1	25	1.25770
125	24JUN2009	night	1	25	1.25044
125	25JUN2009	night	2	25	0.41681
125	25JUN2009	twilight	2	25	0.59890
125	25JUN2009	day	2	25	1.72674
125	25JUN2009	day	2	25	1.76418
125	25JUN2009	day	2	25	0.70338
125	25JUN2009	day	2	25	1.33848
125	25JUN2009	twilight	2	25	1.18187
125	25JUN2009	night	2	25	0.50799
125	26JUN2009	night	2	25	1.35604
125	26JUN2009	twilight	2	25	1.54779
125	26JUN2009	day	2	25	1.28106
127	08JUN2006	day	4	136	1.59219
127	08JUN2006	day	4	136	1.35413
127	08JUN2006	day	4	136	1.30752
127	08JUN2006	twilight	4	136	1.48574

127	08JUN2006	night	4	136	0.97410
127	09JUN2006	night	4	136	1.57172
127	09JUN2006	twilight	4	136	1.60532
127	09JUN2006	day	4	136	1.91856
127	09JUN2006	day	4	136	1.94052
127	09JUN2006	day	4	136	1.16438
127	09JUN2006	twilight	4	136	1.19593
127	09JUN2006	night	4	136	1.68306
127	10JUN2006	night	4	136	1.61701
127	10JUN2006	twilight	4	136	1.29887
127	12JUN2006	day	6	136	1.26720
127	12JUN2006	day	6	136	1.36551
127	12JUN2006	twilight	6	136	1.54159
127	12JUN2006	night	6	136	1.58884
127	13JUN2006	night	6	136	1.02123
127	13JUN2006	twilight	6	136	1.20142
127	13JUN2006	day	6	136	1.16735
127	13JUN2006	day	6	136	1.44562
127	13JUN2006	day	6	136	1.51984
127	13JUN2006	day	6	136	1.11730
127	13JUN2006	twilight	6	136	1.60207
127	13JUN2006	night	6	136	1.40142
127	14JUN2006	night	6	136	0.20710
127	14JUN2006	twilight	6	136	0.93404
127	19JUN2006	day	7	136	0.73568
127	19JUN2006	day	7	136	0.96384
127	19JUN2006	day	7	136	1.06450
127	19JUN2006	twilight	7	136	1.15537
127	19JUN2006	night	7	136	1.23047
127	20JUN2006	night	7	136	1.23982
127	20JUN2006	twilight	7	136	1.34244
127	20JUN2006	day	7	136	1.35027
127	20JUN2006	day	7	136	1.41499
127	20JUN2006	day	7	136	1.41666
127	20JUN2006	day	7	136	1.15537
127	20JUN2006	day	7	136	1.23807
127	20JUN2006	twilight	7	136	1.49277
127	20JUN2006	night	7	136	1.69898
127	21JUN2006	night	7	136	1.46984
127	21JUN2006	twilight	7	136	1.55024
127	26JUN2006	day	2	136	1.01288
127	26JUN2006	day	2	136	0.23578
127	26JUN2006	day	2	136	1.14925
127	26JUN2006	twilight	2	136	1.80754
127	26JUN2006	night	2	136	1.60961
127	27JUN2006	night	2	136	1.63549
127	27JUN2006	twilight	2	136	1.19315
127	27JUN2006	day	2	136	0.64058
127	27JUN2006	day	2	136	0.74827
127	27JUN2006	day	2	136	1.06074
127	27JUN2006	day	2	136	0.91334
127	27JUN2006	twilight	2	136	0.43152
127	27JUN2006	night	2	136	1.06074
127	28JUN2006	night	2	136	2.08991
127	28JUN2006	twilight	2	136	1.83570
127	28JUN2006	day	2	136	1.74352

131	08JUN2006	day	4	74	1.26954
131	08JUN2006	day	4	74	0.99392
131	08JUN2006	day	4	74	1.13357
131	08JUN2006	twilight	4	74	1.12061
131	08JUN2006	night	4	74	1.26954
131	09JUN2006	night	4	74	0.12743
131	09JUN2006	twilight	4	74	1.17322
131	09JUN2006	day	4	74	0.64650
131	09JUN2006	day	4	74	0.36754
131	09JUN2006	day	4	74	1.02535
131	09JUN2006	twilight	4	74	0.69381
131	09JUN2006	night	4	74	1.71768
131	10JUN2006	night	4	74	1.61491
131	10JUN2006	twilight	4	74	0.90314
131	12JUN2006	day	6	74	1.38741
131	12JUN2006	day	6	74	1.28782
131	12JUN2006	twilight	6	74	0.68133
131	12JUN2006	night	6	74	1.17612
131	13JUN2006	night	6	74	1.17029
131	13JUN2006	twilight	6	74	0.97271
131	13JUN2006	day	6	74	1.01288
131	13JUN2006	day	6	74	1.29669
131	13JUN2006	day	6	74	0.90747
131	13JUN2006	day	6	74	0.95429
131	13JUN2006	twilight	6	74	1.01708
131	13JUN2006	night	6	74	1.01708
131	14JUN2006	night	6	74	1.15232
131	14JUN2006	twilight	6	74	1.21487
131	19JUN2006	day	7	74	0.98994
131	19JUN2006	day	7	74	0.46702
131	19JUN2006	day	7	74	1.12388
131	19JUN2006	twilight	7	74	0.86219
131	19JUN2006	night	7	74	1.30537
131	20JUN2006	night	7	74	1.13357
131	20JUN2006	twilight	7	74	0.86988
131	20JUN2006	day	7	74	1.60532
131	20JUN2006	day	7	74	1.64543
131	20JUN2006	day	7	74	1.02942
131	20JUN2006	day	7	74	1.17322
131	20JUN2006	day	7	74	0.88429
131	20JUN2006	twilight	7	74	0.65234
131	20JUN2006	night	7	74	0.93606
131	21JUN2006	night	7	74	0.94156
131	21JUN2006	twilight	7	74	1.36175
131	26JUN2006	day	2	74	0.69906
131	26JUN2006	day	2	74	0.70165
131	26JUN2006	day	2	74	1.30105
131	26JUN2006	twilight	2	74	1.41666
131	26JUN2006	night	2	74	1.79029
131	27JUN2006	night	2	74	1.73879
131	27JUN2006	twilight	2	74	1.19868
131	27JUN2006	day	2	74	1.27418
131	27JUN2006	day	2	74	1.41164
131	27JUN2006	day	2	74	0.69205
131	27JUN2006	day	2	74	1.10724
131	27JUN2006	twilight	2	74	1.92221

131	27JUN2006	night	2	74	-3.00000
131	28JUN2006	night	2	74	1.92993
131	28JUN2006	twilight	2	74	0.91068
131	28JUN2006	day	2	74	1.07558
170	02JUN2010	day	6	9	0.69906
170	02JUN2010	day	6	9	1.14616
170	02JUN2010	day	6	9	0.69906
170	02JUN2010	twilight	6	9	0.95429
170	02JUN2010	night	7	9	0.90314
170	02JUN2010	night	7	9	1.17612
170	02JUN2010	twilight	7	9	1.23047
170	03JUN2010	day	7	9	1.25530
170	03JUN2010	day	7	9	1.63348
170	03JUN2010	day	7	9	1.74037
170	03JUN2010	day	7	9	1.76344
170	03JUN2010	day	7	9	1.14616
170	03JUN2010	night	7	9	0.60217
170	03JUN2010	night	7	9	1.23047
170	03JUN2010	twilight	7	9	1.38023
170	04JUN2010	day	7	9	1.30105
170	09JUN2010	day	6	9	0.47727
170	09JUN2010	day	6	9	1.38023
170	09JUN2010	day	6	9	0.30125
170	09JUN2010	twilight	6	9	0.30125
170	09JUN2010	night	7	9	1.38023
170	09JUN2010	night	7	9	0.95429
170	09JUN2010	twilight	7	9	1.07922
170	10JUN2010	day	7	9	1.25530
170	10JUN2010	day	7	9	1.50516
170	10JUN2010	day	7	9	0.90314
170	10JUN2010	day	7	9	0.77822
170	10JUN2010	twilight	7	9	0.77822
170	10JUN2010	night	7	9	0.69906
170	10JUN2010	night	7	9	1.25530
170	10JUN2010	twilight	7	9	1.11398
170	11JUN2010	day	7	9	1.25530
170	14JUN2010	day	2	9	1.38023
170	14JUN2010	day	2	9	0.90314
170	14JUN2010	day	2	9	1.04143
170	14JUN2010	twilight	2	9	1.32224
170	14JUN2010	night	2	9	1.17612
170	14JUN2010	night	2	9	0.84516
170	14JUN2010	twilight	2	9	0.30125
170	15JUN2010	day	2	9	1.27878
170	15JUN2010	day	2	9	1.36175
170	15JUN2010	day	2	9	0.60217
170	15JUN2010	day	2	9	0.95429
170	15JUN2010	twilight	2	9	1.36175
170	15JUN2010	night	2	9	1.04143
170	15JUN2010	night	2	9	0.95429
170	15JUN2010	twilight	2	9	0.30125
170	16JUN2010	day	2	9	1.17612
170	21JUN2010	day	3	9	-3.00000
170	21JUN2010	day	3	9	0.00043
170	21JUN2010	day	3	9	0.47727
170	21JUN2010	twilight	3	9	1.49138

170	21JUN2010	night	3	9	1.32224
170	21JUN2010	night	3	9	0.69906
170	21JUN2010	twilight	4	9	1.27878
170	22JUN2010	day	4	9	0.77822
170	22JUN2010	day	4	9	0.90314
170	22JUN2010	day	4	9	1.32224
170	22JUN2010	day	4	9	1.20415
170	22JUN2010	twilight	4	9	1.25530
170	22JUN2010	night	4	9	1.43138
170	22JUN2010	night	4	9	1.43138
170	22JUN2010	twilight	4	9	1.34244
170	23JUN2010	twilight	4	9	1.07922
171	02JUN2010	day	6	13	-3.00000
171	02JUN2010	day	6	13	1.00004
171	02JUN2010	day	6	13	0.69906
171	02JUN2010	twilight	6	13	0.95429
171	02JUN2010	night	6	13	0.77822
171	02JUN2010	night	6	13	0.77822
171	02JUN2010	twilight	7	13	0.47727
171	03JUN2010	day	7	13	-3.00000
171	03JUN2010	day	7	13	1.67211
171	03JUN2010	day	7	13	0.69906
171	03JUN2010	day	7	13	1.47714
171	03JUN2010	twilight	7	13	0.00043
171	03JUN2010	night	7	13	1.04143
171	03JUN2010	night	7	13	0.84516
171	03JUN2010	twilight	7	13	2.02119
171	04JUN2010	day	7	13	0.60217
171	09JUN2010	day	8	13	0.95429
171	09JUN2010	day	8	13	0.47727
171	09JUN2010	day	8	13	-3.00000
171	09JUN2010	twilight	8	13	0.30125
171	09JUN2010	night	1	13	0.95429
171	09JUN2010	night	1	13	0.69906
171	09JUN2010	twilight	1	13	0.95429
171	10JUN2010	day	1	13	0.84516
171	10JUN2010	day	1	13	0.69906
171	10JUN2010	day	1	13	0.00043
171	10JUN2010	day	1	13	0.60217
171	10JUN2010	twilight	1	13	0.77822
171	10JUN2010	night	1	13	0.69906
171	10JUN2010	night	1	13	0.69906
171	10JUN2010	twilight	1	13	0.84516
171	11JUN2010	day	1	13	0.47727
171	14JUN2010	day	2	13	0.60217
171	14JUN2010	day	2	13	0.00043
171	14JUN2010	day	2	13	1.30105
171	14JUN2010	twilight	2	13	1.34244
171	14JUN2010	night	2	13	0.47727
171	14JUN2010	night	2	13	0.69906
171	14JUN2010	twilight	2	13	0.47727
171	15JUN2010	day	2	13	0.47727
171	15JUN2010	day	2	13	0.77822
171	15JUN2010	day	2	13	0.30125
171	15JUN2010	day	2	13	0.47727
171	15JUN2010	twilight	2	13	0.30125

171	15JUN2010	night	2	13	0.69906
171	15JUN2010	night	2	13	0.30125
171	15JUN2010	twilight	2	13	0.00043
171	16JUN2010	day	2	13	0.00043
171	21JUN2010	day	3	13	0.30125
171	21JUN2010	day	3	13	0.84516
171	21JUN2010	day	3	13	0.60217
171	21JUN2010	twilight	3	13	0.47727
171	21JUN2010	night	4	13	0.84516
171	21JUN2010	night	4	13	0.30125
171	21JUN2010	twilight	4	13	0.84516
171	22JUN2010	day	4	13	0.60217
171	22JUN2010	day	4	13	0.60217
171	22JUN2010	day	4	13	0.84516
171	22JUN2010	day	4	13	0.60217
171	22JUN2010	twilight	4	13	0.30125
171	22JUN2010	night	4	13	0.60217
171	22JUN2010	night	4	13	0.00043
171	22JUN2010	twilight	4	13	0.84516
171	23JUN2010	day	4	13	2.12058
172	02JUN2010	day	6	19	1.17612
172	02JUN2010	day	6	19	1.04143
172	02JUN2010	day	6	19	1.34244
172	02JUN2010	twilight	6	19	1.34244
172	02JUN2010	night	7	19	1.87507
172	02JUN2010	night	7	19	1.41499
172	02JUN2010	twilight	7	19	0.60217
172	03JUN2010	day	7	19	1.07922
172	03JUN2010	day	7	19	1.69898
172	03JUN2010	day	7	19	1.38023
172	03JUN2010	day	7	19	0.60217
172	03JUN2010	twilight	7	19	1.25530
172	03JUN2010	night	7	19	0.77822
172	03JUN2010	night	7	19	0.47727
172	03JUN2010	twilight	7	19	0.84516
172	04JUN2010	day	7	19	1.07922
172	09JUN2010	day	6	19	0.60217
172	09JUN2010	day	6	19	1.17612
172	09JUN2010	day	6	19	1.07922
172	09JUN2010	twilight	6	19	1.17612
172	09JUN2010	night	7	19	1.55631
172	09JUN2010	night	7	19	0.30125
172	09JUN2010	twilight	7	19	1.65322
172	10JUN2010	day	7	19	1.07922
172	10JUN2010	day	7	19	1.81955
172	10JUN2010	day	7	19	1.95905
172	10JUN2010	day	7	19	1.49138
172	10JUN2010	twilight	7	19	1.25530
172	10JUN2010	night	7	19	1.36175
172	10JUN2010	night	7	19	1.36175
172	10JUN2010	twilight	7	19	1.07922
172	11JUN2010	day	7	19	1.07922
172	14JUN2010	day	2	19	1.11398
172	14JUN2010	day	2	19	1.11398
172	14JUN2010	day	2	19	0.69906
172	14JUN2010	twilight	2	19	1.39796

172	14JUN2010	night	2	19	1.39796
172	14JUN2010	night	2	19	1.41499
172	14JUN2010	twilight	2	19	1.23047
172	15JUN2010	day	2	19	1.07922
172	15JUN2010	day	2	19	1.36175
172	15JUN2010	day	2	19	1.36175
172	15JUN2010	day	2	19	1.27878
172	15JUN2010	twilight	2	19	0.84516
172	15JUN2010	night	2	19	0.30125
172	15JUN2010	night	2	19	0.47727
172	15JUN2010	twilight	2	19	0.60217
172	16JUN2010	day	2	19	1.27878
172	21JUN2010	day	3	19	1.27878
172	21JUN2010	day	3	19	0.90314
172	21JUN2010	day	3	19	1.17612
172	21JUN2010	twilight	3	19	1.56821
172	21JUN2010	night	3	19	1.07922
172	21JUN2010	night	3	19	0.77822
172	21JUN2010	twilight	4	19	0.90314
172	22JUN2010	day	4	19	0.47727
172	22JUN2010	day	4	19	0.60217
172	22JUN2010	day	4	19	1.41499
172	22JUN2010	day	4	19	1.46241
172	22JUN2010	twilight	4	19	1.41499
172	22JUN2010	night	4	19	1.53149
172	22JUN2010	night	4	19	0.95429
172	22JUN2010	twilight	4	19	1.00004
172	23JUN2010	twilight	4	19	1.39796
173	02JUN2010	day	6	16	-3.00000
173	02JUN2010	day	6	16	0.77822
173	02JUN2010	day	6	16	0.95429
173	02JUN2010	twilight	6	16	0.60217
173	02JUN2010	night	7	16	1.69898
173	02JUN2010	night	7	16	1.62326
173	02JUN2010	twilight	7	16	1.56821
173	03JUN2010	day	7	16	1.11398
173	03JUN2010	day	7	16	0.95429
173	03JUN2010	day	7	16	0.84516
173	03JUN2010	day	7	16	1.04143
173	03JUN2010	twilight	7	16	0.95429
173	03JUN2010	night	7	16	1.07922
173	03JUN2010	night	7	16	1.20415
173	03JUN2010	twilight	7	16	0.77822
173	04JUN2010	day	7	16	0.60217
173	09JUN2010	day	6	16	1.50516
173	09JUN2010	day	6	16	0.69906
173	09JUN2010	day	6	16	1.04143
173	09JUN2010	twilight	6	16	1.17612
173	09JUN2010	night	7	16	0.90314
173	09JUN2010	night	7	16	0.47727
173	09JUN2010	twilight	7	16	1.25530
173	10JUN2010	day	7	16	0.30125
173	10JUN2010	day	7	16	0.84516
173	10JUN2010	day	7	16	1.04143
173	10JUN2010	day	7	16	1.23047
173	10JUN2010	twilight	7	16	0.90314

173	10JUN2010	night	7	16	1.00004
173	10JUN2010	night	7	16	1.14616
173	10JUN2010	twilight	7	16	1.11398
173	11JUN2010	day	7	16	0.47727
173	14JUN2010	day	2	16	1.50516
173	14JUN2010	day	2	16	1.00004
173	14JUN2010	day	2	16	1.17612
173	14JUN2010	twilight	2	16	1.27878
173	14JUN2010	night	2	16	1.20415
173	14JUN2010	night	2	16	1.39796
173	14JUN2010	twilight	2	16	1.49138
173	15JUN2010	day	2	16	1.34244
173	15JUN2010	day	2	16	0.30125
173	15JUN2010	day	2	16	0.30125
173	15JUN2010	day	2	16	1.04143
173	15JUN2010	twilight	2	16	1.38023
173	15JUN2010	night	2	16	0.77822
173	15JUN2010	night	2	16	1.38023
173	15JUN2010	twilight	2	16	1.11398
173	16JUN2010	day	2	16	1.32224
173	21JUN2010	day	3	16	0.77822
173	21JUN2010	day	3	16	1.00004
173	21JUN2010	day	3	16	0.69906
173	21JUN2010	twilight	3	16	1.27878
173	21JUN2010	night	3	16	1.25530
173	21JUN2010	night	3	16	1.34244
173	21JUN2010	twilight	4	16	0.77822
173	22JUN2010	day	4	16	1.00004
173	22JUN2010	day	4	16	1.77086
173	22JUN2010	day	4	16	1.66277
173	22JUN2010	day	4	16	1.56821
173	22JUN2010	twilight	4	16	1.57980
173	22JUN2010	night	4	16	1.65322
173	22JUN2010	night	4	16	1.66277
173	22JUN2010	twilight	4	16	0.60217
173	23JUN2010	twilight	4	16	1.41499
174	02JUN2010	day	6	125	1.36175
174	02JUN2010	day	6	125	2.35794
174	02JUN2010	day	6	125	2.37291
174	02JUN2010	twilight	6	125	1.63348
174	02JUN2010	night	7	125	1.27878
174	02JUN2010	night	7	125	0.95429
174	02JUN2010	twilight	7	125	1.25530
174	03JUN2010	day	7	125	-3.00000
174	03JUN2010	day	7	125	1.49138
174	03JUN2010	day	7	125	1.34244
174	03JUN2010	day	7	125	1.17612
174	03JUN2010	twilight	7	125	0.77822
174	03JUN2010	night	7	125	1.07922
174	03JUN2010	night	7	125	0.00043
174	03JUN2010	twilight	7	125	1.20415
174	04JUN2010	day	7	125	1.30105
174	09JUN2010	day	6	125	1.44717
174	09JUN2010	day	6	125	1.14616
174	09JUN2010	day	6	125	2.32428
174	09JUN2010	twilight	6	125	2.31387

174	09JUN2010	night	7	125	-3.00000
174	09JUN2010	night	7	125	0.47727
174	09JUN2010	twilight	7	125	1.43138
174	10JUN2010	day	7	125	0.90314
174	10JUN2010	day	7	125	1.07922
174	10JUN2010	day	7	125	1.20415
174	10JUN2010	day	7	125	1.83886
174	10JUN2010	twilight	7	125	1.69898
174	10JUN2010	night	7	125	1.27878
174	10JUN2010	night	7	125	1.04143
174	10JUN2010	twilight	7	125	1.04143
174	11JUN2010	day	7	125	1.60207
174	14JUN2010	day	2	125	1.69020
174	14JUN2010	day	2	125	1.11398
174	14JUN2010	day	2	125	0.47727
174	14JUN2010	twilight	2	125	1.54408
174	14JUN2010	night	2	125	2.37291
174	14JUN2010	night	2	125	2.34635
174	14JUN2010	twilight	2	125	0.47727
174	15JUN2010	day	2	125	1.00004
174	15JUN2010	day	2	125	1.17612
174	15JUN2010	day	2	125	1.25530
174	15JUN2010	day	2	125	1.25530
174	15JUN2010	twilight	2	125	1.30105
174	15JUN2010	night	2	125	1.47714
174	15JUN2010	night	2	125	1.44717
174	15JUN2010	twilight	2	125	1.27878
174	16JUN2010	day	2	125	1.23047
174	21JUN2010	day	3	125	1.32224
174	21JUN2010	day	3	125	1.34244
174	21JUN2010	day	3	125	1.30105
174	21JUN2010	twilight	3	125	0.30125
174	21JUN2010	night	3	125	1.53149
174	21JUN2010	night	3	125	1.57980
174	21JUN2010	twilight	4	125	1.00004
174	22JUN2010	day	4	125	1.04143
174	22JUN2010	day	4	125	2.31807
174	22JUN2010	day	4	125	2.31807
174	22JUN2010	day	4	125	2.33042
174	22JUN2010	twilight	4	125	2.35218
174	22JUN2010	night	4	125	2.35218
174	22JUN2010	night	4	125	2.31807
174	22JUN2010	twilight	4	125	1.80619
174	23JUN2010	twilight	4	125	1.84510
175	02JUN2010	day	6	52	1.14616
175	02JUN2010	day	6	52	0.60217
175	02JUN2010	day	6	52	0.84516
175	02JUN2010	twilight	6	52	1.53149
175	02JUN2010	night	7	52	1.57980
175	02JUN2010	night	7	52	1.47714
175	02JUN2010	twilight	7	52	1.41499
175	03JUN2010	day	7	52	1.23047
175	03JUN2010	day	7	52	1.11398
175	03JUN2010	day	7	52	0.47727
175	03JUN2010	day	7	52	0.47727
175	03JUN2010	twilight	7	52	0.60217

175	03JUN2010	night	7	52	1.27878
175	03JUN2010	night	7	52	1.44717
175	03JUN2010	twilight	7	52	1.34244
175	04JUN2010	day	7	52	1.23047
175	09JUN2010	day	6	52	0.95429
175	09JUN2010	day	6	52	1.00004
175	09JUN2010	day	6	52	1.04143
175	09JUN2010	twilight	6	52	1.20415
175	09JUN2010	night	7	52	0.69906
175	09JUN2010	night	7	52	0.77822
175	09JUN2010	twilight	7	52	1.04143
175	10JUN2010	day	7	52	0.77822
175	10JUN2010	day	7	52	0.77822
175	10JUN2010	day	7	52	0.95429
175	10JUN2010	day	7	52	1.34244
175	10JUN2010	twilight	7	52	0.84516
175	10JUN2010	night	7	52	0.69906
175	10JUN2010	night	7	52	1.14616
175	10JUN2010	twilight	7	52	1.23047
175	11JUN2010	day	7	52	1.17612
175	14JUN2010	day	2	52	1.69020
175	14JUN2010	day	2	52	0.95429
175	14JUN2010	day	2	52	0.77822
175	14JUN2010	twilight	2	52	0.95429
175	14JUN2010	night	2	52	1.20415
175	14JUN2010	night	2	52	1.72428
175	14JUN2010	twilight	2	52	1.61279
175	15JUN2010	day	2	52	0.90314
175	15JUN2010	day	2	52	0.95429
175	15JUN2010	day	2	52	1.00004
175	15JUN2010	day	2	52	0.95429
175	15JUN2010	twilight	2	52	0.77822
175	15JUN2010	night	2	52	1.11398
175	15JUN2010	night	2	52	1.04143
175	15JUN2010	twilight	2	52	0.60217
175	16JUN2010	day	2	52	0.47727
175	21JUN2010	day	3	52	1.11398
175	21JUN2010	day	3	52	0.00043
175	21JUN2010	day	3	52	0.95429
175	21JUN2010	twilight	3	52	1.27878
175	21JUN2010	night	3	52	0.95429
175	21JUN2010	night	3	52	1.61279
175	21JUN2010	twilight	4	52	1.59108
175	22JUN2010	day	4	52	0.95429
175	22JUN2010	day	4	52	0.90314
175	22JUN2010	day	4	52	1.30105
175	22JUN2010	day	4	52	1.34244
175	22JUN2010	twilight	4	52	1.36175
175	22JUN2010	night	4	52	1.43138
175	22JUN2010	night	4	52	1.32224
175	22JUN2010	twilight	4	52	1.38023
175	23JUN2010	twilight	4	52	1.27878
176	02JUN2010	day	6	37	0.47727
176	02JUN2010	day	6	37	1.27878
176	02JUN2010	day	6	37	0.69906
176	02JUN2010	twilight	6	37	1.14616

176	02JUN2010	night	7	37	0.90314
176	02JUN2010	night	7	37	1.04143
176	02JUN2010	twilight	7	37	1.17612
176	03JUN2010	day	7	37	1.07922
176	03JUN2010	day	7	37	1.20415
176	03JUN2010	day	7	37	0.77822
176	03JUN2010	day	7	37	0.47727
176	03JUN2010	twilight	7	37	0.77822
176	03JUN2010	night	7	37	1.46241
176	03JUN2010	night	7	37	1.56821
176	03JUN2010	twilight	7	37	1.41499
176	04JUN2010	day	7	37	1.36175
176	09JUN2010	day	6	37	-3.00000
176	09JUN2010	day	6	37	0.47727
176	09JUN2010	day	6	37	1.41499
176	09JUN2010	twilight	6	37	1.50516
176	09JUN2010	night	7	37	1.55631
176	09JUN2010	night	7	37	-3.00000
176	09JUN2010	twilight	7	37	1.55631
176	10JUN2010	day	7	37	0.95429
176	10JUN2010	day	7	37	1.50516
176	10JUN2010	day	7	37	1.60207
176	10JUN2010	day	7	37	0.84516
176	10JUN2010	twilight	7	37	0.84516
176	10JUN2010	night	7	37	0.47727
176	10JUN2010	night	7	37	0.60217
176	10JUN2010	twilight	7	37	1.00004
176	11JUN2010	day	7	37	1.14616
176	14JUN2010	day	2	37	0.90314
176	14JUN2010	day	2	37	1.00004
176	14JUN2010	day	2	37	1.57980
176	14JUN2010	twilight	2	37	0.95429
176	14JUN2010	night	2	37	1.20415
176	14JUN2010	night	2	37	1.72428
176	14JUN2010	twilight	2	37	1.61279
176	15JUN2010	day	2	37	0.90314
176	15JUN2010	day	2	37	0.95429
176	15JUN2010	day	2	37	1.65322
176	15JUN2010	day	2	37	0.90314
176	15JUN2010	twilight	2	37	0.95429
176	15JUN2010	night	2	37	1.66277
176	15JUN2010	night	2	37	1.04143
176	15JUN2010	twilight	2	37	0.60217
176	16JUN2010	day	2	37	0.47727
176	21JUN2010	day	3	37	0.30125
176	21JUN2010	day	3	37	0.90314
176	21JUN2010	day	3	37	1.04143
176	21JUN2010	twilight	3	37	1.32224
176	21JUN2010	night	3	37	1.32224
176	21JUN2010	night	3	37	0.47727
176	21JUN2010	twilight	4	37	0.47727
176	22JUN2010	day	4	37	0.90314
176	22JUN2010	day	4	37	0.47727
176	22JUN2010	day	4	37	1.38023
176	22JUN2010	day	4	37	1.32224
176	22JUN2010	twilight	4	37	1.14616

176	22JUN2010	night	4	37	1.36175
176	22JUN2010	night	4	37	1.41499
176	22JUN2010	twilight	4	37	1.38023
176	23JUN2010	twilight	4	37	1.14616
177	02JUN2010	day	6	13	1.77086
177	02JUN2010	day	6	13	1.14616
177	02JUN2010	day	6	13	1.14616
177	02JUN2010	twilight	6	13	0.60217
177	02JUN2010	night	7	13	0.90314
177	02JUN2010	night	7	13	0.90314
177	02JUN2010	twilight	7	13	0.77822
177	03JUN2010	day	7	13	1.92942
177	03JUN2010	day	7	13	2.02119
177	03JUN2010	day	7	13	1.07922
177	03JUN2010	day	7	13	1.04143
177	03JUN2010	twilight	7	13	1.14616
177	03JUN2010	night	7	13	1.86924
177	03JUN2010	night	7	13	1.85126
177	03JUN2010	twilight	7	13	1.07922
177	04JUN2010	day	7	13	1.07922
177	09JUN2010	day	6	13	0.30125
177	09JUN2010	day	6	13	1.07922
177	09JUN2010	day	6	13	1.54408
177	09JUN2010	twilight	6	13	1.56821
177	09JUN2010	night	7	13	0.95429
177	09JUN2010	night	7	13	1.27878
177	09JUN2010	twilight	7	13	1.47714
177	10JUN2010	day	7	13	0.77822
177	10JUN2010	day	7	13	0.77822
177	10JUN2010	day	7	13	0.60217
177	10JUN2010	day	7	13	0.84516
177	10JUN2010	twilight	7	13	0.95429
177	10JUN2010	night	7	13	0.77822
177	10JUN2010	night	7	13	0.47727
177	10JUN2010	twilight	7	13	0.60217
177	11JUN2010	day	7	13	0.90314
177	14JUN2010	day	2	13	0.60217
177	14JUN2010	day	2	13	0.30125
177	14JUN2010	day	2	13	1.04143
177	14JUN2010	twilight	2	13	1.25530
177	14JUN2010	night	2	13	1.44717
177	14JUN2010	night	2	13	1.20415
177	14JUN2010	twilight	2	13	0.84516
177	15JUN2010	day	2	13	0.47727
177	15JUN2010	day	2	13	0.84516
177	15JUN2010	day	2	13	0.47727
177	15JUN2010	day	2	13	0.60217
177	15JUN2010	twilight	2	13	0.60217
177	15JUN2010	night	2	13	1.07922
177	15JUN2010	night	2	13	0.77822
177	15JUN2010	twilight	2	13	0.90314
177	16JUN2010	day	2	13	0.60217
177	21JUN2010	day	3	13	0.30125
177	21JUN2010	day	3	13	0.60217
177	21JUN2010	day	3	13	0.95429
177	21JUN2010	twilight	3	13	1.32224

177	21JUN2010	night	3	13	0.90314
177	21JUN2010	night	3	13	0.69906
177	21JUN2010	twilight	4	13	0.77822
177	22JUN2010	day	4	13	0.77822
177	22JUN2010	day	4	13	0.90314
177	22JUN2010	day	4	13	1.38023
177	22JUN2010	day	4	13	1.36175
177	22JUN2010	twilight	4	13	1.27878
177	22JUN2010	night	4	13	1.38023
177	22JUN2010	night	4	13	1.25530
177	22JUN2010	twilight	4	13	1.23047
177	23JUN2010	twilight	4	13	1.23047
178	02JUN2010	day	6	26	1.00004
178	02JUN2010	day	6	26	1.07922
178	02JUN2010	day	6	26	0.30125
178	02JUN2010	twilight	6	26	1.14616
178	02JUN2010	night	7	26	1.00004
178	02JUN2010	night	7	26	1.11398
178	02JUN2010	twilight	7	26	1.07922
178	03JUN2010	day	7	26	0.84516
178	03JUN2010	day	7	26	0.90314
178	03JUN2010	day	7	26	1.34244
178	03JUN2010	day	7	26	1.43138
178	03JUN2010	twilight	7	26	1.14616
178	03JUN2010	night	7	26	0.69906
178	03JUN2010	night	7	26	0.95429
178	03JUN2010	twilight	7	26	1.34244
178	04JUN2010	day	7	26	1.00004
178	09JUN2010	day	6	26	1.60207
178	09JUN2010	day	6	26	1.34244
178	09JUN2010	day	6	26	0.69906
178	09JUN2010	twilight	6	26	1.17612
178	09JUN2010	night	7	26	0.90314
178	09JUN2010	night	7	26	0.90314
178	09JUN2010	twilight	7	26	1.00004
178	10JUN2010	day	7	26	1.17612
178	10JUN2010	day	7	26	0.69906
178	10JUN2010	day	7	26	1.87507
178	10JUN2010	day	7	26	1.71601
178	10JUN2010	twilight	7	26	1.47714
178	10JUN2010	night	7	26	1.27878
178	10JUN2010	night	7	26	1.11398
178	10JUN2010	twilight	7	26	1.11398
178	11JUN2010	day	7	26	1.07922
178	14JUN2010	day	2	26	1.30105
178	14JUN2010	day	2	26	1.07922
178	14JUN2010	day	2	26	0.90314
178	14JUN2010	twilight	2	26	1.00004
178	14JUN2010	night	2	26	1.04143
178	14JUN2010	night	2	26	1.11398
178	14JUN2010	twilight	2	26	1.25530
178	15JUN2010	day	2	26	0.84516
178	15JUN2010	day	2	26	0.69906
178	15JUN2010	day	2	26	0.84516
178	15JUN2010	day	2	26	0.77822
178	15JUN2010	twilight	2	26	0.95429

178	15JUN2010	night	2	26	1.17612
178	15JUN2010	night	2	26	0.77822
178	15JUN2010	twilight	2	26	-3.00000
178	16JUN2010	day	2	26	0.00043
178	21JUN2010	day	3	26	0.77822
178	21JUN2010	day	3	26	0.47727
178	21JUN2010	day	3	26	1.17612
178	21JUN2010	twilight	3	26	1.47714
178	21JUN2010	night	3	26	1.30105
178	21JUN2010	night	3	26	0.77822
178	21JUN2010	twilight	4	26	0.60217
178	22JUN2010	day	4	26	0.69906
178	22JUN2010	day	4	26	0.95429
178	22JUN2010	day	4	26	1.46241
178	22JUN2010	day	4	26	1.43138
178	22JUN2010	twilight	4	26	1.38023
178	22JUN2010	night	4	26	1.44717
178	22JUN2010	night	4	26	1.34244
178	22JUN2010	twilight	4	26	1.46241
178	23JUN2010	twilight	4	26	1.43138
179	02JUN2010	day	6	54	0.47727
179	02JUN2010	day	6	54	1.25530
179	02JUN2010	day	6	54	1.32224
179	02JUN2010	twilight	6	54	0.60217
179	02JUN2010	night	7	54	1.73240
179	02JUN2010	night	7	54	1.07922
179	02JUN2010	twilight	7	54	0.47727
179	03JUN2010	day	7	54	0.69906
179	03JUN2010	day	7	54	1.17612
179	03JUN2010	day	7	54	1.17612
179	03JUN2010	day	7	54	0.95429
179	03JUN2010	twilight	7	54	0.95429
179	03JUN2010	night	7	54	1.17612
179	03JUN2010	night	7	54	1.00004
179	03JUN2010	twilight	7	54	0.90314
179	04JUN2010	day	7	54	0.95429
179	09JUN2010	day	6	54	0.60217
179	09JUN2010	day	6	54	1.23047
179	09JUN2010	day	6	54	1.30105
179	09JUN2010	twilight	6	54	1.25530
179	09JUN2010	night	7	54	1.34244
179	09JUN2010	night	7	54	0.60217
179	09JUN2010	twilight	7	54	1.25530
179	10JUN2010	day	7	54	1.20415
179	10JUN2010	day	7	54	1.51853
179	10JUN2010	day	7	54	1.00004
179	10JUN2010	day	7	54	1.32224
179	10JUN2010	twilight	7	54	1.17612
179	10JUN2010	night	7	54	0.47727
179	10JUN2010	night	7	54	0.30125
179	10JUN2010	twilight	7	54	1.25530
179	11JUN2010	day	7	54	1.30105
179	14JUN2010	day	2	54	1.04143
179	14JUN2010	day	2	54	1.51853
179	14JUN2010	day	2	54	1.56821
179	14JUN2010	twilight	2	54	1.00004

179	14JUN2010	night	2	54	1.04143
179	14JUN2010	night	2	54	1.11398
179	14JUN2010	twilight	2	54	1.36175
179	15JUN2010	day	2	54	1.27878
179	15JUN2010	day	2	54	0.90314
179	15JUN2010	day	2	54	1.34244
179	15JUN2010	day	2	54	1.07922
179	15JUN2010	twilight	2	54	0.60217
179	15JUN2010	night	2	54	1.43138
179	15JUN2010	night	2	54	0.00043
179	15JUN2010	twilight	2	54	0.69906
179	16JUN2010	day	2	54	1.11398
179	21JUN2010	day	3	54	1.00004
179	21JUN2010	day	3	54	1.04143
179	21JUN2010	day	3	54	0.95429
179	21JUN2010	twilight	3	54	1.55631
179	21JUN2010	night	3	54	1.55631
179	21JUN2010	night	3	54	0.47727
179	21JUN2010	twilight	4	54	0.69906
179	22JUN2010	day	4	54	1.55631
179	22JUN2010	day	4	54	1.57980
179	22JUN2010	day	4	54	1.60207
179	22JUN2010	day	4	54	1.64346
179	22JUN2010	twilight	4	54	1.69020
179	22JUN2010	night	4	54	1.70758
179	22JUN2010	night	4	54	1.62326
179	22JUN2010	twilight	4	54	1.57980
179	23JUN2010	twilight	4	54	1.77816
180	02JUN2010	day	6	67	1.32224
180	02JUN2010	day	6	67	1.14616
180	02JUN2010	day	6	67	0.90314
180	02JUN2010	twilight	6	67	1.20415
180	02JUN2010	night	7	67	1.78534
180	02JUN2010	night	7	67	1.34244
180	02JUN2010	twilight	7	67	0.47727
180	03JUN2010	day	7	67	1.47714
180	03JUN2010	day	7	67	1.20415
180	03JUN2010	day	7	67	0.84516
180	03JUN2010	day	7	67	1.07922
180	03JUN2010	twilight	7	67	1.34244
180	03JUN2010	night	7	67	0.95429
180	03JUN2010	night	7	67	0.77822
180	03JUN2010	twilight	7	67	1.43138
180	04JUN2010	day	7	67	0.84516
180	09JUN2010	day	6	67	1.47714
180	09JUN2010	day	6	67	0.90314
180	09JUN2010	day	6	67	1.17612
180	09JUN2010	twilight	6	67	1.32224
180	09JUN2010	night	7	67	2.08636
180	09JUN2010	night	7	67	2.03343
180	09JUN2010	twilight	7	67	1.17612
180	10JUN2010	day	7	67	0.69906
180	10JUN2010	day	7	67	1.27878
180	10JUN2010	day	7	67	1.56821
180	10JUN2010	day	7	67	1.23047
180	10JUN2010	twilight	7	67	1.27878

180	10JUN2010	night	7	67	1.14616
180	10JUN2010	night	7	67	1.07922
180	10JUN2010	twilight	7	67	0.90314
180	11JUN2010	day	7	67	1.00004
180	14JUN2010	day	2	67	1.39796
180	14JUN2010	day	2	67	1.53149
180	14JUN2010	day	2	67	0.60217
180	14JUN2010	twilight	2	67	1.04143
180	14JUN2010	night	2	67	0.30125
180	14JUN2010	night	2	67	1.20415
180	14JUN2010	twilight	2	67	1.07922
180	15JUN2010	day	2	67	1.07922
180	15JUN2010	day	2	67	0.69906
180	15JUN2010	day	2	67	1.17612
180	15JUN2010	day	2	67	1.17612
180	15JUN2010	twilight	2	67	0.95429
180	15JUN2010	night	2	67	0.30125
180	15JUN2010	night	2	67	1.25530
180	15JUN2010	twilight	2	67	0.00043
180	16JUN2010	day	2	67	1.00004
180	21JUN2010	day	3	67	1.50516
180	21JUN2010	day	3	67	1.34244
180	21JUN2010	day	3	67	1.00004
180	21JUN2010	twilight	3	67	1.11398
180	21JUN2010	night	3	67	1.20415
180	21JUN2010	night	3	67	0.90314
180	21JUN2010	twilight	4	67	1.30105
180	22JUN2010	day	4	67	0.90314
180	22JUN2010	day	4	67	1.32224
180	22JUN2010	day	4	67	1.32224
180	22JUN2010	day	4	67	1.49138
180	22JUN2010	twilight	4	67	1.20415
180	22JUN2010	night	4	67	1.11398
180	22JUN2010	night	4	67	1.17612
180	22JUN2010	twilight	4	67	1.00004
180	23JUN2010	twilight	4	67	1.14616
181	02JUN2010	day	6	60	1.76344
181	02JUN2010	day	6	60	0.95429
181	02JUN2010	day	6	60	1.66277
181	02JUN2010	twilight	6	60	0.47727
181	02JUN2010	night	7	60	1.49138
181	02JUN2010	night	7	60	0.95429
181	02JUN2010	twilight	7	60	1.20415
181	03JUN2010	day	7	60	1.38023
181	03JUN2010	day	7	60	1.41499
181	03JUN2010	day	7	60	1.47714
181	03JUN2010	day	7	60	1.30105
181	03JUN2010	twilight	7	60	1.04143
181	03JUN2010	night	7	60	0.77822
181	03JUN2010	night	7	60	1.04143
181	03JUN2010	twilight	7	60	0.69906
181	04JUN2010	day	7	60	1.30105
181	09JUN2010	day	6	60	0.69906
181	09JUN2010	day	6	60	0.77822
181	09JUN2010	day	6	60	1.32224
181	09JUN2010	twilight	6	60	1.49138

181	09JUN2010	night	7	60	0.69906
181	09JUN2010	night	7	60	0.60217
181	09JUN2010	twilight	7	60	0.84516
181	10JUN2010	day	7	60	0.95429
181	10JUN2010	day	7	60	1.20415
181	10JUN2010	day	7	60	1.25530
181	10JUN2010	day	7	60	0.00043
181	10JUN2010	twilight	7	60	0.47727
181	10JUN2010	night	7	60	1.11398
181	10JUN2010	night	7	60	1.00004
181	10JUN2010	twilight	7	60	0.84516
181	11JUN2010	day	7	60	0.60217
181	14JUN2010	day	2	60	0.00043
181	14JUN2010	day	2	60	1.11398
181	14JUN2010	day	2	60	1.27878
181	14JUN2010	twilight	2	60	1.27878
181	14JUN2010	night	2	60	0.84516
181	14JUN2010	night	2	60	0.84516
181	14JUN2010	twilight	2	60	1.34244
181	15JUN2010	day	2	60	1.17612
181	15JUN2010	day	2	60	1.14616
181	15JUN2010	day	2	60	1.00004
181	15JUN2010	day	2	60	1.38023
181	15JUN2010	twilight	2	60	0.77822
181	15JUN2010	night	2	60	0.95429
181	15JUN2010	night	2	60	0.00043
181	15JUN2010	twilight	2	60	0.60217
181	16JUN2010	day	2	60	1.04143
181	21JUN2010	day	3	60	1.25530
181	21JUN2010	day	3	60	0.90314
181	21JUN2010	day	3	60	0.77822
181	21JUN2010	twilight	3	60	1.23047
181	21JUN2010	night	3	60	1.17612
181	21JUN2010	night	3	60	0.69906
181	21JUN2010	twilight	4	60	0.77822
181	22JUN2010	day	4	60	0.30125
181	22JUN2010	day	4	60	1.34244
181	22JUN2010	day	4	60	1.51853
181	22JUN2010	day	4	60	1.43138
181	22JUN2010	twilight	4	60	1.17612
181	22JUN2010	night	4	60	1.47714
181	22JUN2010	night	4	60	1.46241
181	22JUN2010	twilight	4	60	0.95429
181	23JUN2010	twilight	4	60	1.27878
182	02JUN2010	day	6	13	0.95429
182	02JUN2010	day	6	13	0.77822
182	02JUN2010	day	6	13	0.95429
182	02JUN2010	twilight	6	13	0.90314
182	02JUN2010	night	7	13	0.84516
182	02JUN2010	night	7	13	0.47727
182	02JUN2010	twilight	7	13	1.30105
182	03JUN2010	day	7	13	1.00004
182	03JUN2010	day	7	13	1.04143
182	03JUN2010	day	7	13	1.17612
182	03JUN2010	day	7	13	1.39796
182	03JUN2010	twilight	7	13	1.53149

182	03JUN2010	night	7	13	1.51853
182	03JUN2010	night	7	13	1.32224
182	03JUN2010	twilight	7	13	1.20415
182	04JUN2010	day	7	13	0.30125
182	09JUN2010	day	6	13	0.95429
182	09JUN2010	day	6	13	1.57980
182	09JUN2010	day	6	13	1.69898
182	09JUN2010	twilight	6	13	1.73240
182	09JUN2010	night	7	13	0.90314
182	09JUN2010	night	7	13	0.77822
182	09JUN2010	twilight	7	13	1.60207
182	10JUN2010	day	7	13	1.07922
182	10JUN2010	day	7	13	1.25530
182	10JUN2010	day	7	13	1.32224
182	10JUN2010	day	7	13	0.30125
182	10JUN2010	twilight	7	13	0.47727
182	10JUN2010	night	7	13	1.04143
182	10JUN2010	night	7	13	0.95429
182	10JUN2010	twilight	7	13	0.77822
182	11JUN2010	day	7	13	0.30125
182	14JUN2010	day	2	13	0.77822
182	14JUN2010	day	2	13	0.47727
182	14JUN2010	day	2	13	1.04143
182	14JUN2010	twilight	2	13	1.00004
182	14JUN2010	night	2	13	0.95429
182	14JUN2010	night	2	13	1.07922
182	14JUN2010	twilight	2	13	0.69906
182	15JUN2010	day	2	13	1.32224
182	15JUN2010	day	2	13	1.27878
182	15JUN2010	day	2	13	0.90314
182	15JUN2010	day	2	13	0.69906
182	15JUN2010	twilight	2	13	0.90314
182	15JUN2010	night	2	13	1.00004
182	15JUN2010	night	2	13	0.30125
182	15JUN2010	twilight	2	13	0.60217
182	16JUN2010	day	2	13	0.69906
182	21JUN2010	twilight	3	13	1.55631
182	21JUN2010	night	3	13	1.51853
182	21JUN2010	night	3	13	1.50516
182	21JUN2010	twilight	4	13	0.84516
182	22JUN2010	day	4	13	1.81292
182	22JUN2010	day	4	13	1.95425
182	22JUN2010	day	4	13	1.41499
182	22JUN2010	day	4	13	1.36175
182	22JUN2010	twilight	4	13	1.53149
182	22JUN2010	night	4	13	0.95429
182	22JUN2010	night	4	13	0.77822
182	22JUN2010	twilight	4	13	0.60217
182	23JUN2010	twilight	4	13	0.90314
183	02JUN2010	day	6	47	0.69906
183	02JUN2010	day	6	47	0.77822
183	02JUN2010	day	6	47	1.11398
183	02JUN2010	twilight	6	47	1.17612
183	02JUN2010	night	6	47	1.11398
183	02JUN2010	night	6	47	1.73240
183	02JUN2010	twilight	7	47	1.60207

183	03JUN2010	day	7	47	0.77822
183	03JUN2010	day	7	47	1.14616
183	03JUN2010	day	7	47	0.77822
183	03JUN2010	day	7	47	1.11398
183	03JUN2010	twilight	7	47	0.60217
183	03JUN2010	night	7	47	0.77822
183	03JUN2010	night	7	47	0.30125
183	03JUN2010	twilight	7	47	0.69906
183	04JUN2010	day	7	47	0.77822
183	09JUN2010	day	8	47	0.90314
183	09JUN2010	day	8	47	0.69906
183	09JUN2010	day	8	47	1.00004
183	09JUN2010	twilight	8	47	0.47727
183	09JUN2010	night	1	47	1.20415
183	09JUN2010	night	1	47	0.69906
183	09JUN2010	twilight	1	47	0.47727
183	10JUN2010	day	1	47	1.07922
183	10JUN2010	day	1	47	0.47727
183	10JUN2010	day	1	47	0.30125
183	10JUN2010	day	1	47	0.60217
183	10JUN2010	twilight	1	47	0.47727
183	10JUN2010	night	1	47	0.30125
183	10JUN2010	night	1	47	0.47727
183	10JUN2010	twilight	1	47	0.90314
183	11JUN2010	day	1	47	1.32224
183	14JUN2010	day	2	47	0.90314
183	14JUN2010	day	2	47	0.77822
183	14JUN2010	day	2	47	1.11398
183	14JUN2010	twilight	2	47	0.47727
183	14JUN2010	night	2	47	-3.00000
183	14JUN2010	night	2	47	2.06819
183	14JUN2010	twilight	2	47	2.11727
183	15JUN2010	day	2	47	0.84516
183	15JUN2010	day	2	47	1.04143
183	15JUN2010	day	2	47	-3.00000
183	15JUN2010	day	2	47	0.00043
183	15JUN2010	twilight	2	47	1.11398
183	15JUN2010	night	2	47	0.47727
183	15JUN2010	night	2	47	0.30125
183	15JUN2010	twilight	2	47	1.27878
183	16JUN2010	day	2	47	1.04143
183	21JUN2010	day	3	47	0.69906
183	21JUN2010	day	3	47	1.14616
183	21JUN2010	day	3	47	2.09691
183	21JUN2010	twilight	3	47	0.69906
183	21JUN2010	night	4	47	2.10721
183	21JUN2010	night	4	47	2.09691
183	21JUN2010	twilight	4	47	2.08636
183	22JUN2010	day	4	47	0.84516
183	22JUN2010	day	4	47	2.08991
183	22JUN2010	day	4	47	0.30125
183	22JUN2010	day	4	47	0.47727
183	22JUN2010	twilight	4	47	0.30125
183	22JUN2010	night	4	47	0.60217
183	22JUN2010	night	4	47	0.00043
183	22JUN2010	twilight	4	47	0.47727

183	23JUN2010	day	4	47	0.47727
184	02JUN2010	day	6	34	0.47727
184	02JUN2010	day	6	34	1.14616
184	02JUN2010	day	6	34	0.47727
184	02JUN2010	twilight	6	34	1.34244
184	02JUN2010	night	6	34	1.14616
184	02JUN2010	night	6	34	1.30105
184	02JUN2010	twilight	7	34	1.34244
184	03JUN2010	day	7	34	1.39796
184	03JUN2010	day	7	34	0.95429
184	03JUN2010	day	7	34	0.60217
184	03JUN2010	day	7	34	1.04143
184	03JUN2010	twilight	7	34	1.04143
184	03JUN2010	night	7	34	0.95429
184	03JUN2010	night	7	34	0.60217
184	03JUN2010	twilight	7	34	0.47727
184	04JUN2010	day	7	34	0.47727
184	09JUN2010	day	8	34	0.77822
184	09JUN2010	day	8	34	1.27878
184	09JUN2010	day	8	34	1.27878
184	09JUN2010	twilight	8	34	1.07922
184	09JUN2010	night	1	34	0.69906
184	09JUN2010	night	1	34	-3.00000
184	09JUN2010	twilight	1	34	0.69906
184	10JUN2010	day	1	34	0.90314
184	10JUN2010	day	1	34	0.77822
184	10JUN2010	day	1	34	0.84516
184	10JUN2010	day	1	34	0.47727
184	10JUN2010	twilight	1	34	0.69906
184	10JUN2010	night	1	34	0.60217
184	10JUN2010	night	1	34	1.11398
184	10JUN2010	twilight	1	34	0.84516
184	11JUN2010	day	1	34	0.30125
184	14JUN2010	day	2	34	0.77822
184	14JUN2010	day	2	34	1.07922
184	14JUN2010	day	2	34	0.60217
184	14JUN2010	twilight	2	34	0.47727
184	14JUN2010	night	2	34	0.60217
184	14JUN2010	night	2	34	0.47727
184	14JUN2010	twilight	2	34	0.77822
184	15JUN2010	day	2	34	0.30125
184	15JUN2010	day	2	34	0.69906
184	15JUN2010	day	2	34	0.30125
184	15JUN2010	day	2	34	0.69906
184	15JUN2010	twilight	2	34	0.00043
184	15JUN2010	night	2	34	0.69906
184	15JUN2010	night	2	34	0.30125
184	15JUN2010	twilight	2	34	0.60217
184	16JUN2010	day	2	34	0.90314
184	21JUN2010	day	3	34	0.60217
184	21JUN2010	day	3	34	0.30125
184	21JUN2010	day	3	34	1.86924
184	21JUN2010	twilight	3	34	0.00043
184	21JUN2010	night	4	34	1.84510
184	21JUN2010	night	4	34	-3.00000
184	21JUN2010	twilight	4	34	0.69906

184	22JUN2010	day	4	34	0.60217
184	22JUN2010	day	4	34	1.86333
184	22JUN2010	day	4	34	0.60217
184	22JUN2010	day	4	34	0.69906
184	22JUN2010	twilight	4	34	0.47727
184	22JUN2010	night	4	34	0.84516
184	22JUN2010	night	4	34	0.47727
184	22JUN2010	twilight	4	34	0.60217
184	23JUN2010	day	4	34	-3.00000
185	02JUN2010	day	6	88	0.95429
185	02JUN2010	day	6	88	0.47727
185	02JUN2010	day	6	88	2.51983
185	02JUN2010	twilight	6	88	2.49831
185	02JUN2010	night	6	88	1.11398
185	02JUN2010	night	6	88	0.69906
185	02JUN2010	twilight	7	88	0.77822
185	03JUN2010	day	7	88	0.60217
185	03JUN2010	day	7	88	1.14616
185	03JUN2010	day	7	88	0.90314
185	03JUN2010	day	7	88	0.84516
185	03JUN2010	twilight	7	88	1.00004
185	03JUN2010	night	7	88	0.47727
185	03JUN2010	night	7	88	0.90314
185	03JUN2010	twilight	7	88	0.47727
185	04JUN2010	day	7	88	0.30125
185	09JUN2010	day	8	88	1.14616
185	09JUN2010	day	8	88	0.30125
185	09JUN2010	day	8	88	0.47727
185	09JUN2010	twilight	8	88	0.47727
185	09JUN2010	night	1	88	0.84516
185	09JUN2010	night	1	88	0.84516
185	09JUN2010	twilight	1	88	0.60217
185	10JUN2010	day	1	88	0.47727
185	10JUN2010	day	1	88	0.77822
185	10JUN2010	day	1	88	-3.00000
185	10JUN2010	day	1	88	0.47727
185	10JUN2010	twilight	1	88	0.00043
185	10JUN2010	night	1	88	0.69906
185	10JUN2010	night	1	88	0.30125
185	10JUN2010	twilight	1	88	0.30125
185	11JUN2010	day	1	88	0.00043
185	14JUN2010	day	2	88	0.69906
185	14JUN2010	day	2	88	0.30125
185	14JUN2010	day	2	88	0.60217
185	14JUN2010	twilight	2	88	0.30125
185	14JUN2010	night	2	88	0.47727
185	14JUN2010	night	2	88	0.47727
185	14JUN2010	twilight	2	88	0.47727
185	15JUN2010	day	2	88	0.47727
185	15JUN2010	day	2	88	1.07922
185	15JUN2010	day	2	88	0.90314
185	15JUN2010	day	2	88	0.60217
185	15JUN2010	twilight	2	88	0.00043
185	15JUN2010	night	2	88	0.60217
185	15JUN2010	night	2	88	-3.00000
185	15JUN2010	twilight	2	88	0.47727

185	16JUN2010	day	2	88	1.20415
185	21JUN2010	day	3	88	-3.00000
185	21JUN2010	day	3	88	0.00043
185	21JUN2010	day	3	88	0.77822
185	21JUN2010	twilight	3	88	2.08991
185	21JUN2010	night	4	88	2.09691
185	21JUN2010	night	4	88	0.47727
185	21JUN2010	twilight	4	88	0.60217
185	22JUN2010	day	4	88	0.77822
185	22JUN2010	day	4	88	0.60217
185	22JUN2010	day	4	88	0.00043
185	22JUN2010	day	4	88	0.90314
185	22JUN2010	twilight	4	88	2.12711
185	22JUN2010	night	4	88	2.11727
185	22JUN2010	night	4	88	2.09691
185	22JUN2010	twilight	4	88	0.47727
185	23JUN2010	day	4	88	2.10381
186	02JUN2010	day	6	45	1.56821
186	02JUN2010	day	6	45	1.92942
186	02JUN2010	day	6	45	1.88082
186	02JUN2010	day	6	45	1.04143
186	02JUN2010	night	6	45	0.69906
186	02JUN2010	night	6	45	1.14616
186	02JUN2010	day	7	45	0.95429
186	03JUN2010	day	7	45	0.60217
186	03JUN2010	day	7	45	1.00004
186	03JUN2010	day	7	45	0.69906
186	03JUN2010	day	7	45	0.69906
186	03JUN2010	twilight	7	45	0.00043
186	03JUN2010	night	7	45	-3.00000
186	03JUN2010	night	7	45	0.00043
186	03JUN2010	twilight	7	45	0.00043
186	04JUN2010	day	7	45	1.04143
186	09JUN2010	day	8	45	0.95429
186	09JUN2010	day	8	45	1.20415
186	09JUN2010	day	8	45	1.51853
186	09JUN2010	twilight	8	45	0.47727
186	09JUN2010	night	1	45	1.53149
186	09JUN2010	night	1	45	0.77822
186	09JUN2010	twilight	1	45	1.11398
186	10JUN2010	day	1	45	0.69906
186	10JUN2010	day	1	45	1.20415
186	10JUN2010	day	1	45	1.44717
186	10JUN2010	day	1	45	1.41499
186	10JUN2010	twilight	1	45	0.47727
186	10JUN2010	night	1	45	0.90314
186	10JUN2010	night	1	45	0.47727
186	10JUN2010	twilight	1	45	0.47727
186	11JUN2010	day	1	45	0.47727
186	14JUN2010	day	2	45	1.68125
186	14JUN2010	day	2	45	0.77822
186	14JUN2010	day	2	45	0.95429
186	14JUN2010	twilight	2	45	1.20415
186	14JUN2010	night	2	45	0.77822
186	14JUN2010	night	2	45	0.95429
186	14JUN2010	twilight	2	45	0.90314

186	15JUN2010	day	2	45	1.07922
186	15JUN2010	day	2	45	0.84516
186	15JUN2010	day	2	45	1.17612
186	15JUN2010	day	2	45	1.17612
186	15JUN2010	twilight	2	45	0.47727
186	15JUN2010	night	2	45	0.69906
186	15JUN2010	night	2	45	0.30125
186	15JUN2010	twilight	2	45	0.84516
186	16JUN2010	day	2	45	0.00043
186	21JUN2010	day	3	45	0.90314
186	21JUN2010	day	3	45	1.00004
186	21JUN2010	day	3	45	2.59988
186	21JUN2010	twilight	3	45	1.74820
186	21JUN2010	night	4	45	2.53276
186	21JUN2010	night	4	45	0.69906
186	21JUN2010	twilight	4	45	1.07922
186	22JUN2010	day	4	45	0.77822
186	22JUN2010	day	4	45	2.40140
186	22JUN2010	day	4	45	2.33646
186	22JUN2010	day	4	45	2.15229
186	22JUN2010	twilight	4	45	2.36922
186	22JUN2010	night	4	45	2.50786
186	22JUN2010	night	4	45	2.05308
186	22JUN2010	twilight	4	45	1.73240
186	23JUN2010	day	4	45	2.37107
187	02JUN2010	day	6	75	1.00004
187	02JUN2010	day	6	75	1.25530
187	02JUN2010	day	6	75	0.90314
187	02JUN2010	twilight	6	75	0.95429
187	02JUN2010	night	6	75	0.84516
187	02JUN2010	night	6	75	0.60217
187	02JUN2010	twilight	7	75	0.60217
187	03JUN2010	day	7	75	0.00043
187	03JUN2010	day	7	75	1.54408
187	03JUN2010	day	7	75	1.63348
187	03JUN2010	day	7	75	0.47727
187	03JUN2010	twilight	7	75	1.49138
187	03JUN2010	night	7	75	1.68125
187	03JUN2010	night	7	75	0.47727
187	03JUN2010	twilight	7	75	1.92428
187	04JUN2010	day	7	75	1.14616
187	09JUN2010	day	8	75	0.95429
187	09JUN2010	day	8	75	0.95429
187	09JUN2010	day	8	75	1.44717
187	09JUN2010	twilight	8	75	1.23047
187	09JUN2010	night	1	75	0.00043
187	09JUN2010	night	1	75	0.30125
187	09JUN2010	twilight	1	75	0.30125
187	10JUN2010	day	1	75	0.60217
187	10JUN2010	day	1	75	1.07922
187	10JUN2010	day	1	75	0.77822
187	10JUN2010	day	1	75	0.30125
187	10JUN2010	twilight	1	75	0.00043
187	10JUN2010	night	1	75	1.25530
187	10JUN2010	night	1	75	0.47727
187	10JUN2010	twilight	1	75	1.11398

187	11JUN2010	day	1	75	0.69906
187	14JUN2010	day	2	75	1.04143
187	14JUN2010	day	2	75	1.76344
187	14JUN2010	day	2	75	1.83252
187	14JUN2010	twilight	2	75	1.20415
187	14JUN2010	night	2	75	0.77822
187	14JUN2010	night	2	75	1.07922
187	14JUN2010	twilight	2	75	0.47727
187	15JUN2010	day	2	75	0.30125
187	15JUN2010	day	2	75	1.20415
187	15JUN2010	day	2	75	1.17612
187	15JUN2010	day	2	75	0.47727
187	15JUN2010	twilight	2	75	0.00043
187	15JUN2010	night	2	75	1.07922
187	15JUN2010	night	2	75	0.90314
187	15JUN2010	twilight	2	75	0.47727
187	16JUN2010	day	2	75	0.30125
187	21JUN2010	day	3	75	0.47727
187	21JUN2010	day	3	75	-3.00000
187	21JUN2010	day	3	75	1.88650
187	21JUN2010	twilight	3	75	0.47727
187	21JUN2010	night	4	75	1.86924
187	21JUN2010	night	4	75	-3.00000
187	21JUN2010	twilight	4	75	0.47727
187	22JUN2010	day	4	75	0.47727
187	22JUN2010	day	4	75	1.72428
187	22JUN2010	day	4	75	0.90314
187	22JUN2010	day	4	75	1.25530
187	22JUN2010	twilight	4	75	-3.00000
187	22JUN2010	night	4	75	0.47727
187	22JUN2010	night	4	75	0.30125
187	22JUN2010	twilight	4	75	0.00043
187	23JUN2010	day	4	75	0.30125
188	02JUN2010	day	6	49	1.61279
188	02JUN2010	day	6	49	1.49138
188	02JUN2010	day	6	49	0.69906
188	02JUN2010	day	6	49	1.04143
188	02JUN2010	night	6	49	1.20415
188	02JUN2010	night	6	49	1.20415
188	02JUN2010	day	7	49	1.66277
188	03JUN2010	day	7	49	1.81292
188	03JUN2010	day	7	49	1.39796
188	03JUN2010	day	7	49	1.11398
188	03JUN2010	day	7	49	1.55631
188	03JUN2010	twilight	7	49	1.04143
188	03JUN2010	night	7	49	0.77822
188	03JUN2010	night	7	49	1.25530
188	03JUN2010	twilight	7	49	1.23047
188	04JUN2010	day	7	49	1.14616
188	09JUN2010	day	8	49	0.69906
188	09JUN2010	day	8	49	1.11398
188	09JUN2010	day	8	49	0.69906
188	09JUN2010	twilight	8	49	0.69906
188	09JUN2010	night	1	49	0.95429
188	09JUN2010	night	1	49	0.69906
188	09JUN2010	twilight	1	49	1.00004

188	10JUN2010	day	1	49	0.60217
188	10JUN2010	day	1	49	0.69906
188	10JUN2010	day	1	49	1.39796
188	10JUN2010	day	1	49	1.39796
188	10JUN2010	twilight	1	49	0.30125
188	10JUN2010	night	1	49	1.00004
188	10JUN2010	night	1	49	0.84516
188	10JUN2010	twilight	1	49	1.25530
188	11JUN2010	day	1	49	1.17612
188	14JUN2010	day	2	49	0.95429
188	14JUN2010	day	2	49	0.90314
188	14JUN2010	day	2	49	1.00004
188	14JUN2010	twilight	2	49	0.47727
188	14JUN2010	night	2	49	-3.00000
188	14JUN2010	night	2	49	0.90314
188	14JUN2010	twilight	2	49	1.11398
188	15JUN2010	day	2	49	0.84516
188	15JUN2010	day	2	49	1.00004
188	15JUN2010	day	2	49	0.90314
188	15JUN2010	day	2	49	1.17612
188	15JUN2010	twilight	2	49	0.47727
188	15JUN2010	night	2	49	0.90314
188	15JUN2010	night	2	49	0.47727
188	15JUN2010	twilight	2	49	0.84516
188	16JUN2010	day	2	49	0.47727
188	21JUN2010	day	3	49	1.07922
188	21JUN2010	day	3	49	1.07922
188	21JUN2010	day	3	49	1.89763
188	21JUN2010	twilight	3	49	0.90314
188	21JUN2010	night	4	49	1.85126
188	21JUN2010	night	4	49	0.00043
188	21JUN2010	twilight	4	49	0.77822
188	22JUN2010	day	4	49	0.30125
188	22JUN2010	day	4	49	1.89763
188	22JUN2010	day	4	49	0.47727
188	22JUN2010	day	4	49	0.60217
188	22JUN2010	twilight	4	49	0.60217
188	22JUN2010	night	4	49	0.77822
188	22JUN2010	night	4	49	0.60217
188	22JUN2010	twilight	4	49	0.69906
188	23JUN2010	day	4	49	0.30125
189	02JUN2010	day	6	39	0.95429
189	02JUN2010	day	6	39	1.07922
189	02JUN2010	day	6	39	1.17612
189	02JUN2010	twilight	6	39	0.30125
189	02JUN2010	night	6	39	0.95429
189	02JUN2010	night	6	39	0.90314
189	02JUN2010	twilight	7	39	0.30125
189	03JUN2010	day	7	39	0.47727
189	03JUN2010	day	7	39	0.84516
189	03JUN2010	day	7	39	0.84516
189	03JUN2010	day	7	39	0.00043
189	03JUN2010	twilight	7	39	0.60217
189	03JUN2010	night	7	39	0.90314
189	03JUN2010	night	7	39	0.30125
189	03JUN2010	twilight	7	39	1.43138

189	04JUN2010	day	7	39	1.47714
189	09JUN2010	day	8	39	0.69906
189	09JUN2010	day	8	39	0.30125
189	09JUN2010	day	8	39	1.65322
189	09JUN2010	twilight	8	39	0.30125
189	09JUN2010	night	1	39	0.69906
189	09JUN2010	night	1	39	0.30125
189	09JUN2010	twilight	1	39	1.00004
189	10JUN2010	day	1	39	1.00004
189	10JUN2010	day	1	39	0.77822
189	10JUN2010	day	1	39	0.95429
189	10JUN2010	day	1	39	0.77822
189	10JUN2010	twilight	1	39	0.90314
189	10JUN2010	night	1	39	0.69906
189	10JUN2010	night	1	39	1.49138
189	10JUN2010	twilight	1	39	1.51853
189	11JUN2010	day	1	39	1.51853
189	14JUN2010	day	2	39	1.00004
189	14JUN2010	day	2	39	0.30125
189	14JUN2010	day	2	39	1.50516
189	14JUN2010	twilight	2	39	0.47727
189	14JUN2010	night	2	39	1.04143
189	14JUN2010	night	2	39	0.77822
189	14JUN2010	twilight	2	39	0.60217
189	15JUN2010	day	2	39	0.30125
189	15JUN2010	day	2	39	1.62326
189	15JUN2010	day	2	39	0.69906
189	15JUN2010	day	2	39	-3.00000
189	15JUN2010	twilight	2	39	1.53149
189	15JUN2010	night	2	39	0.69906
189	15JUN2010	night	2	39	0.30125
189	15JUN2010	twilight	2	39	0.30125
189	16JUN2010	day	2	39	1.11398
189	21JUN2010	day	3	39	0.30125
189	21JUN2010	day	3	39	-3.00000
189	21JUN2010	day	3	39	1.83886
189	21JUN2010	twilight	3	39	0.90314
189	21JUN2010	night	4	39	1.88082
189	21JUN2010	night	4	39	0.30125
189	21JUN2010	twilight	4	39	0.77822
189	22JUN2010	day	4	39	0.00043
189	22JUN2010	day	4	39	1.89763
189	22JUN2010	day	4	39	0.00043
189	22JUN2010	day	4	39	0.60217
189	22JUN2010	twilight	4	39	0.47727
189	22JUN2010	night	4	39	0.47727
189	22JUN2010	night	4	39	0.60217
189	22JUN2010	twilight	4	39	0.30125
189	23JUN2010	day	4	39	0.30125
190	02JUN2010	day	6	25	0.47727
190	02JUN2010	day	6	25	0.30125
190	02JUN2010	day	6	25	1.00004
190	02JUN2010	twilight	6	25	0.60217
190	02JUN2010	night	6	25	1.20415
190	02JUN2010	night	6	25	0.90314
190	02JUN2010	twilight	7	25	0.30125

190	03JUN2010	day	7	25	0.47727
190	03JUN2010	day	7	25	1.04143
190	03JUN2010	day	7	25	0.47727
190	03JUN2010	day	7	25	0.30125
190	03JUN2010	twilight	7	25	0.47727
190	03JUN2010	night	7	25	0.69906
190	03JUN2010	night	7	25	0.30125
190	03JUN2010	twilight	7	25	0.60217
190	04JUN2010	day	7	25	0.30125
190	09JUN2010	day	8	25	0.69906
190	09JUN2010	day	8	25	0.30125
190	09JUN2010	day	8	25	1.39796
190	09JUN2010	twilight	8	25	0.00043
190	09JUN2010	night	1	25	1.14616
190	09JUN2010	night	1	25	1.20415
190	09JUN2010	twilight	1	25	1.38023
190	10JUN2010	day	1	25	1.07922
190	10JUN2010	day	1	25	0.95429
190	10JUN2010	day	1	25	0.77822
190	10JUN2010	day	1	25	1.11398
190	10JUN2010	twilight	1	25	0.47727
190	10JUN2010	night	1	25	0.77822
190	10JUN2010	night	1	25	0.77822
190	10JUN2010	twilight	1	25	1.00004
190	11JUN2010	day	1	25	0.00043
190	14JUN2010	day	2	25	1.34244
190	14JUN2010	day	2	25	0.60217
190	14JUN2010	day	2	25	1.39796
190	14JUN2010	twilight	2	25	1.14616
190	14JUN2010	night	2	25	1.23047
190	14JUN2010	night	2	25	0.47727
190	14JUN2010	twilight	2	25	0.77822
190	15JUN2010	day	2	25	1.07922
190	15JUN2010	day	2	25	0.47727
190	15JUN2010	day	2	25	0.47727
190	15JUN2010	day	2	25	0.00043
190	15JUN2010	twilight	2	25	0.47727
190	15JUN2010	night	2	25	1.07922
190	15JUN2010	night	2	25	0.00043
190	15JUN2010	twilight	2	25	0.69906
190	16JUN2010	day	2	25	0.69906
190	21JUN2010	day	3	25	0.00043
190	21JUN2010	day	3	25	1.74820
190	21JUN2010	day	3	25	0.30125
190	21JUN2010	twilight	3	25	1.77816
190	21JUN2010	night	4	25	0.77822
190	21JUN2010	night	4	25	0.69906
190	21JUN2010	twilight	4	25	0.00043
190	22JUN2010	day	4	25	0.00043
190	22JUN2010	day	4	25	1.80619
190	22JUN2010	day	4	25	0.60217
190	22JUN2010	day	4	25	1.17612
190	22JUN2010	twilight	4	25	0.30125
190	22JUN2010	night	4	25	0.84516
190	22JUN2010	night	4	25	-3.00000
190	22JUN2010	twilight	4	25	0.30125

190	23JUN2010	day	4	25	0.00043
191	02JUN2010	day	6	128	0.47727
191	02JUN2010	day	6	128	0.69906
191	02JUN2010	day	6	128	0.90314
191	02JUN2010	twilight	6	128	0.47727
191	02JUN2010	night	7	128	0.95429
191	02JUN2010	night	7	128	0.69906
191	02JUN2010	twilight	7	128	0.69906
191	03JUN2010	day	7	128	0.47727
191	03JUN2010	day	7	128	0.77822
191	03JUN2010	day	7	128	1.41499
191	03JUN2010	day	7	128	0.84516
191	03JUN2010	twilight	7	128	1.32224
191	03JUN2010	night	7	128	1.20415
191	03JUN2010	night	7	128	0.69906
191	03JUN2010	twilight	7	128	0.77822
191	04JUN2010	day	7	128	0.69906
191	09JUN2010	day	6	128	0.84516
191	09JUN2010	day	6	128	1.04143
191	09JUN2010	day	6	128	1.00004
191	09JUN2010	twilight	6	128	1.14616
191	09JUN2010	night	7	128	0.95429
191	09JUN2010	night	7	128	0.30125
191	09JUN2010	twilight	7	128	0.69906
191	10JUN2010	day	7	128	1.20415
191	10JUN2010	day	7	128	1.20415
191	10JUN2010	day	7	128	0.00043
191	10JUN2010	day	7	128	1.32224
191	10JUN2010	twilight	7	128	0.77822
191	10JUN2010	night	7	128	0.60217
191	10JUN2010	night	7	128	1.20415
191	10JUN2010	twilight	7	128	0.84516
191	11JUN2010	day	7	128	0.60217
191	14JUN2010	day	2	128	0.84516
191	14JUN2010	day	2	128	0.30125
191	14JUN2010	day	2	128	1.44717
191	14JUN2010	twilight	2	128	0.77822
191	14JUN2010	night	2	128	1.23047
191	14JUN2010	night	2	128	1.54408
191	14JUN2010	twilight	2	128	1.07922
191	15JUN2010	day	2	128	0.84516
191	15JUN2010	day	2	128	1.23047
191	15JUN2010	day	2	128	0.90314
191	15JUN2010	day	2	128	1.07922
191	15JUN2010	twilight	2	128	0.77822
191	15JUN2010	night	2	128	2.20412
191	15JUN2010	night	2	128	2.20412
191	15JUN2010	twilight	2	128	0.60217
191	16JUN2010	day	2	128	0.47727
191	21JUN2010	day	3	128	0.00043
191	21JUN2010	day	3	128	0.84516
191	21JUN2010	day	3	128	0.90314
191	21JUN2010	twilight	3	128	1.11398
191	21JUN2010	night	3	128	0.95429
191	21JUN2010	night	3	128	1.00004
191	21JUN2010	twilight	4	128	0.47727

191	22JUN2010	day	4	128	0.95429
191	22JUN2010	day	4	128	1.11398
191	22JUN2010	day	4	128	1.27878
191	22JUN2010	day	4	128	1.23047
191	22JUN2010	twilight	4	128	1.17612
191	22JUN2010	night	4	128	1.50516
191	22JUN2010	night	4	128	1.54408
191	22JUN2010	twilight	4	128	1.50516
191	23JUN2010	twilight	4	128	1.50516
192	02JUN2010	day	6	101	1.11398
192	02JUN2010	day	6	101	1.27878
192	02JUN2010	day	6	101	0.95429
192	02JUN2010	twilight	6	101	1.17612
192	02JUN2010	night	6	101	1.00004
192	02JUN2010	night	6	101	0.60217
192	02JUN2010	twilight	7	101	1.25530
192	03JUN2010	day	7	101	1.11398
192	03JUN2010	day	7	101	1.27878
192	03JUN2010	day	7	101	0.69906
192	03JUN2010	day	7	101	0.95429
192	03JUN2010	twilight	7	101	0.30125
192	03JUN2010	night	7	101	0.95429
192	03JUN2010	night	7	101	0.69906
192	03JUN2010	twilight	7	101	0.47727
192	04JUN2010	day	7	101	-3.00000
192	09JUN2010	day	8	101	0.84516
192	09JUN2010	day	8	101	0.69906
192	09JUN2010	day	8	101	1.57980
192	09JUN2010	twilight	8	101	0.47727
192	09JUN2010	night	1	101	1.47714
192	09JUN2010	night	1	101	0.60217
192	09JUN2010	twilight	1	101	0.69906
192	10JUN2010	day	1	101	1.17612
192	10JUN2010	day	1	101	0.77822
192	10JUN2010	day	1	101	-3.00000
192	10JUN2010	day	1	101	0.30125
192	10JUN2010	twilight	1	101	0.60217
192	10JUN2010	night	1	101	0.84516
192	10JUN2010	night	1	101	0.30125
192	10JUN2010	twilight	1	101	0.00043
192	11JUN2010	day	1	101	0.30125
192	14JUN2010	day	2	101	0.60217
192	14JUN2010	day	2	101	0.30125
192	14JUN2010	day	2	101	1.23047
192	14JUN2010	twilight	2	101	0.47727
192	14JUN2010	night	2	101	0.30125
192	14JUN2010	night	2	101	0.47727
192	14JUN2010	twilight	2	101	0.60217
192	15JUN2010	day	2	101	0.69906
192	15JUN2010	day	2	101	0.84516
192	15JUN2010	day	2	101	1.23047
192	15JUN2010	day	2	101	1.07922
192	15JUN2010	twilight	2	101	1.39796
192	15JUN2010	night	2	101	0.60217
192	15JUN2010	night	2	101	0.00043
192	15JUN2010	twilight	2	101	0.00043

192	16JUN2010	day	2	101	0.30125
192	21JUN2010	day	3	101	0.30125
192	21JUN2010	day	3	101	0.84516
192	21JUN2010	day	3	101	2.12385
192	21JUN2010	twilight	3	101	2.10381
192	21JUN2010	night	4	101	0.60217
192	21JUN2010	night	4	101	0.60217
192	21JUN2010	twilight	4	101	0.30125
192	22JUN2010	day	4	101	0.00043
192	22JUN2010	day	4	101	2.16137
192	22JUN2010	day	4	101	1.20415
192	22JUN2010	day	4	101	1.57980
192	22JUN2010	twilight	4	101	2.07918
192	22JUN2010	night	4	101	2.10721
192	22JUN2010	night	4	101	2.11395
192	22JUN2010	twilight	4	101	0.84516
192	23JUN2010	day	4	101	0.00043
193	02JUN2010	day	6	171	1.47714
193	02JUN2010	day	6	171	0.69906
193	02JUN2010	day	6	171	0.90314
193	02JUN2010	twilight	6	171	1.00004
193	02JUN2010	night	7	171	1.07922
193	02JUN2010	night	7	171	1.76344
193	02JUN2010	twilight	7	171	1.38023
193	03JUN2010	day	7	171	0.47727
193	03JUN2010	day	7	171	-3.00000
193	03JUN2010	day	7	171	1.00004
193	03JUN2010	day	7	171	1.32224
193	03JUN2010	twilight	7	171	1.07922
193	03JUN2010	night	7	171	1.17612
193	03JUN2010	night	7	171	1.17612
193	03JUN2010	twilight	7	171	0.60217
193	04JUN2010	day	7	171	0.30125
193	09JUN2010	day	6	171	0.90314
193	09JUN2010	day	6	171	1.14616
193	09JUN2010	day	6	171	0.47727
193	09JUN2010	twilight	6	171	1.11398
193	09JUN2010	night	7	171	1.27878
193	09JUN2010	night	7	171	0.60217
193	09JUN2010	twilight	7	171	1.27878
193	10JUN2010	day	7	171	0.90314
193	10JUN2010	day	7	171	0.90314
193	10JUN2010	day	7	171	0.95429
193	10JUN2010	day	7	171	1.00004
193	10JUN2010	twilight	7	171	0.30125
193	10JUN2010	night	7	171	0.90314
193	10JUN2010	night	7	171	0.77822
193	10JUN2010	twilight	7	171	0.95429
193	11JUN2010	day	7	171	1.00004
193	14JUN2010	day	2	171	1.43138
193	14JUN2010	day	2	171	0.95429
193	14JUN2010	day	2	171	1.32224
193	14JUN2010	twilight	2	171	1.36175
193	14JUN2010	night	2	171	0.60217
193	14JUN2010	night	2	171	0.47727
193	14JUN2010	twilight	2	171	0.30125

193	15JUN2010	day	2	171	1.11398
193	15JUN2010	day	2	171	1.59108
193	15JUN2010	day	2	171	1.23047
193	15JUN2010	day	2	171	1.25530
193	15JUN2010	twilight	2	171	0.95429
193	15JUN2010	night	2	171	0.30125
193	15JUN2010	night	2	171	0.30125
193	15JUN2010	twilight	2	171	0.30125
193	16JUN2010	day	2	171	-3.00000
193	21JUN2010	day	3	171	1.23047
193	21JUN2010	day	3	171	0.60217
193	21JUN2010	day	3	171	0.84516
193	21JUN2010	twilight	3	171	1.14616
193	21JUN2010	night	3	171	1.07922
193	21JUN2010	night	3	171	0.69906
193	21JUN2010	twilight	4	171	0.90314
193	22JUN2010	day	4	171	0.90314
193	22JUN2010	day	4	171	0.84516
193	22JUN2010	day	4	171	0.69906
193	22JUN2010	day	4	171	1.38023
193	22JUN2010	twilight	4	171	1.50516
193	22JUN2010	night	4	171	1.32224
193	22JUN2010	night	4	171	1.36175
193	22JUN2010	twilight	4	171	1.47714
193	23JUN2010	twilight	4	171	1.20415
194	02JUN2010	day	6	81	1.46241
194	02JUN2010	day	6	81	1.11398
194	02JUN2010	day	6	81	0.47727
194	02JUN2010	twilight	6	81	0.95429
194	02JUN2010	night	7	81	0.90314
194	02JUN2010	night	7	81	1.11398
194	02JUN2010	twilight	7	81	1.34244
194	03JUN2010	day	7	81	1.20415
194	03JUN2010	day	7	81	0.95429
194	03JUN2010	day	7	81	1.04143
194	03JUN2010	day	7	81	1.17612
194	03JUN2010	twilight	7	81	1.34244
194	03JUN2010	night	7	81	1.51853
194	03JUN2010	night	7	81	1.65322
194	03JUN2010	twilight	7	81	1.53149
194	04JUN2010	day	7	81	1.36175
194	09JUN2010	day	6	81	0.69906
194	09JUN2010	day	6	81	1.00004
194	09JUN2010	day	6	81	1.00004
194	09JUN2010	twilight	6	81	0.95429
194	09JUN2010	night	7	81	1.11398
194	09JUN2010	night	7	81	1.20415
194	09JUN2010	twilight	7	81	1.04143
194	10JUN2010	day	7	81	1.14616
194	10JUN2010	day	7	81	1.11398
194	10JUN2010	day	7	81	1.04143
194	10JUN2010	day	7	81	0.77822
194	10JUN2010	twilight	7	81	1.20415
194	10JUN2010	night	7	81	1.20415
194	10JUN2010	night	7	81	1.04143
194	10JUN2010	twilight	7	81	1.17612

194	11JUN2010	day	7	81	1.53149
194	14JUN2010	day	2	81	1.14616
194	14JUN2010	day	2	81	0.30125
194	14JUN2010	day	2	81	1.38023
194	14JUN2010	twilight	2	81	2.02939
194	14JUN2010	night	2	81	1.23047
194	14JUN2010	night	2	81	1.93952
194	14JUN2010	twilight	2	81	1.20415
194	15JUN2010	day	2	81	1.11398
194	15JUN2010	day	2	81	1.46241
194	15JUN2010	day	2	81	1.14616
194	15JUN2010	day	2	81	-3.00000
194	15JUN2010	twilight	2	81	-3.00000
194	15JUN2010	night	2	81	0.69906
194	15JUN2010	night	2	81	0.90314
194	15JUN2010	twilight	2	81	0.30125
194	16JUN2010	day	2	81	0.90314
194	21JUN2010	day	3	81	1.17612
194	21JUN2010	day	3	81	1.23047
194	21JUN2010	day	3	81	1.20415
194	21JUN2010	twilight	3	81	2.34635
194	21JUN2010	night	3	81	2.31597
194	21JUN2010	night	3	81	1.20415
194	21JUN2010	twilight	4	81	1.14616
194	22JUN2010	day	4	81	0.95429
194	22JUN2010	day	4	81	1.00004
194	22JUN2010	day	4	81	2.34242
194	22JUN2010	day	4	81	2.36922
194	22JUN2010	twilight	4	81	2.38739
194	22JUN2010	night	4	81	2.38561
194	22JUN2010	night	4	81	2.35411
194	22JUN2010	twilight	4	81	2.34635
194	23JUN2010	twilight	4	81	2.36361

slope_log	subpopulation_density_log	subpopulation_site_fidelity_log	site_fidelity_ratio
0.41597	-1.50040	1.09133	1
0.41597	-1.50040	1.09133	1
0.41597	-1.50040	1.09133	1
0.41597	-1.50040	1.09133	1
0.41597	-1.50040	1.09133	1
0.41597	-1.50040	1.09133	1
0.41597	-1.50040	1.09133	1
0.41597	-1.50040	1.09133	1
0.41597	-1.50040	1.09133	1
0.41597	-1.50040	1.09133	1
0.41597	-1.50040	1.09133	1
0.41597	-1.50040	1.09133	1
0.41597	-1.50040	1.09133	1
0.41597	-1.50040	1.09133	1
0.41597	-1.50040	1.09133	1
0.41597	-1.50040	1.09133	1
0.41597	-1.50040	1.09133	1
0.41597	-1.50040	1.09133	1
0.41597	-1.50040	1.09133	1
0.41597	-1.50040	1.09133	1
0.41597	-1.50040	1.09133	1
0.41597	-1.50040	1.09133	1
0.41597	-1.50040	1.09133	1
0.41597	-1.50040	1.09133	1
0.41597	-1.50040	1.09133	1
0.41597	-1.50040	1.09133	1
0.41597	-1.50040	1.09133	1
0.41597	-1.50040	1.09133	1
0.41597	-1.50040	1.09133	1
0.41597	-1.50040	1.09133	1
0.41597	-1.50040	1.09133	1
0.09377	-1.19145	0.79922	0
0.09377	-1.19145	0.79922	0
0.09377	-1.19145	0.79922	0
0.09377	-1.19145	0.79922	0
0.09377	-1.19145	0.79922	0
0.09377	-1.19145	0.79922	0
0.09377	-1.19145	0.79922	0
0.09377	-1.19145	0.79922	0
0.09377	-1.19145	0.79922	0
0.09377	-1.19145	0.79922	0
0.09377	-1.19145	0.79922	0
0.09377	-1.19145	0.79922	0
0.09377	-1.19145	0.79922	0
0.09377	-1.19145	0.79922	0
0.09377	-1.19145	0.79922	0
0.09377	-1.19145	0.79922	0
0.09377	-1.19145	0.79922	0
0.09377	-1.19145	0.79922	0
0.09377	-1.19145	0.79922	0
0.09377	-1.19145	0.79922	0
0.09377	-1.19145	0.79922	0
0.09377	-1.19145	0.79922	0
0.09377	-1.19145	0.79922	0
0.09377	-1.19145	0.79922	0

0.09377	-1.19145	0.79922	0
0.09377	-1.19145	0.79922	0
0.09377	-1.19145	0.79922	0
0.09377	-1.19145	0.79922	0
0.09377	-1.19145	0.79922	0
0.09377	-1.19145	0.79922	0
0.09377	-1.19145	0.79922	0
0.09377	-1.19145	0.79922	0
0.09377	-1.19145	0.79922	0
0.09377	-1.19145	0.79922	0
0.09377	-1.19145	0.79922	0
0.09377	-1.19145	0.79922	0
0.09377	-1.19145	0.79922	0
0.09377	-1.19145	0.79922	0
0.09377	-1.19145	0.79922	0
0.09377	-1.19145	0.79922	0
0.09377	-1.19145	0.79922	0
0.09377	-1.19145	0.79922	0
0.09377	-1.19145	0.79922	0
0.09377	-1.19145	0.79922	0
0.09377	-1.19145	0.79922	0
0.09377	-1.19145	0.79922	0
0.09377	-1.19145	0.79922	0
0.09377	-1.19145	0.79922	0
0.77859	-1.35208	0.99459	1
0.77859	-1.35208	0.99459	1
0.77859	-1.35208	0.99459	1
0.77859	-1.35208	0.99459	1
0.77859	-1.35208	0.99459	1
0.77859	-1.35208	0.99459	1
0.77859	-1.35208	0.99459	1
0.77859	-1.35208	0.99459	1
0.77859	-1.35208	0.99459	1
0.77859	-1.35208	0.99459	1
0.77859	-1.35208	0.99459	1
0.77859	-1.35208	0.99459	1
0.77859	-1.35208	0.99459	1
0.77859	-1.35208	0.99459	1
0.77859	-1.35208	0.99459	1
0.77859	-1.35208	0.99459	1
0.77859	-1.35208	0.99459	1
0.77859	-1.35208	0.99459	1
0.77859	-1.35208	0.99459	1
0.77859	-1.35208	0.99459	1
0.77859	-1.35208	0.99459	1
0.77859	-1.35208	0.99459	1
0.77859	-1.35208	0.99459	1
0.77859	-1.35208	0.99459	1
0.77859	-1.35208	0.99459	1
0.77859	-1.35208	0.99459	1
0.77859	-1.35208	0.99459	1
0.77859	-1.35208	0.99459	1
0.77859	-1.35208	0.99459	1
0.77859	-1.35208	0.99459	1
0.77859	-1.35208	0.99459	1
0.77859	-1.35208	0.99459	1

0.77859	-1.35208	0.99459	1
0.77859	-1.35208	0.99459	1
0.77859	-1.35208	0.99459	1
0.77859	-1.35208	0.99459	1
0.77859	-1.35208	0.99459	1
0.77859	-1.35208	0.99459	1
0.77859	-1.35208	0.99459	1
0.77859	-1.35208	0.99459	1
0.77859	-1.35208	0.99459	1
0.77859	-1.35208	0.99459	1
0.77859	-1.35208	0.99459	1
0.77859	-1.35208	0.99459	1
0.77859	-1.35208	0.99459	1
0.77859	-1.35208	0.99459	1
0.77859	-1.35208	0.99459	1
0.77859	-1.35208	0.99459	1
0.77859	-1.35208	0.99459	1
0.77859	-1.35208	0.99459	1
0.77859	-1.35208	0.99459	1
0.77859	-1.35208	0.99459	1
0.77859	-1.35208	0.99459	1
0.77859	-1.35208	0.99459	1
0.77859	-1.35208	0.99459	1
0.77859	-1.35208	0.99459	1
0.77859	-1.35208	0.99459	1
0.77859	-1.35208	0.99459	1
0.77859	-1.35208	0.99459	1
0.77859	-1.35208	0.99459	1
0.77859	-1.35208	0.99459	1
0.77859	-1.35208	0.99459	1
0.77859	-1.35208	0.99459	1
0.77859	-1.35208	0.99459	1
0.77859	-1.35208	0.99459	1
0.77859	-1.35208	0.99459	1
0.77859	-1.35208	0.99459	1
0.77859	-1.35208	0.99459	1
0.77859	-1.35208	0.99459	1
0.77859	-1.35208	0.99459	1
0.77859	-1.35208	0.99459	1
0.77859	-1.35208	0.99459	1
0.77859	-1.35208	0.99459	1
0.77859	-1.35208	0.99459	1
0.77859	-1.35208	0.99459	1
0.77859	-1.35208	0.99459	1
0.77859	-1.35208	0.99459	1
0.77859	-1.35208	0.99459	1
0.77859	-1.35208	0.99459	1
0.77859	-1.35208	0.99459	1
0.77859	-1.35208	0.99459	1
0.77859	-1.35208	0.99459	1
0.77859	-1.35208	0.99459	1
0.77859	-1.35208	0.99459	1
0.77859	-1.35208	0.99459	1
0.77859	-1.35208	0.99459	1
0.77859	-1.35208	0.99459	1
0.77859	-1.35208	0.99459	1

0.77859	-1.35208	0.99459	1
0.77859	-1.35208	0.99459	1
0.77859	-1.35208	0.99459	1
0.77859	-1.35208	0.99459	1
0.77859	-1.35208	0.99459	1
0.77859	-1.35208	0.99459	1
0.77859	-1.35208	0.99459	1
0.77859	-1.35208	0.99459	1
0.77859	-1.35208	0.99459	1
0.77859	-1.35208	0.99459	1
0.77859	-1.35208	0.99459	1
0.77859	-1.35208	0.99459	1
0.77859	-1.35208	0.99459	1
0.77859	-1.35208	0.99459	1
0.77859	-1.35208	0.99459	1
0.77859	-1.35208	0.99459	1
0.77859	-1.35208	0.99459	1
0.77859	-1.35208	0.99459	1
0.77859	-1.35208	0.99459	1
0.77859	-1.35208	0.99459	1
0.77859	-1.35208	0.99459	1
0.77859	-1.35208	0.99459	1
0.77859	-1.35208	0.99459	1
0.77859	-1.35208	0.99459	1
0.77859	-1.35208	0.99459	1
0.77859	-1.35208	0.99459	1
0.77859	-1.35208	0.99459	1
0.77859	-1.35208	0.99459	1
0.77859	-1.35208	0.99459	1
0.77859	-1.35208	0.99459	1
0.77859	-1.35208	0.99459	1
0.77859	-1.35208	0.99459	1
0.77859	-1.35208	0.99459	1
0.77859	-1.35208	0.99459	1
0.77859	-1.35208	0.99459	1
0.77859	-1.35208	0.99459	1
0.77859	-1.35208	0.99459	1
0.77859	-1.35208	0.99459	1
0.77859	-1.35208	0.99459	1
0.77859	-1.35208	0.99459	1
0.77859	-1.35208	0.99459	1
0.77859	-1.35208	0.99459	1
0.77859	-1.35208	0.99459	1
0.77859	-1.35208	0.99459	1
0.77859	-1.35208	0.99459	1
0.77859	-1.35208	0.99459	1
0.77859	-1.35208	0.99459	1
0.77859	-1.35208	0.99459	1
0.77859	-1.35208	0.99459	1
0.77859	-1.35208	0.99459	1
0.77859	-1.35208	0.99459	1
0.77859	-1.35208	0.99459	1
0.77859	-1.35208	0.99459	1
0.77859	-1.35208	0.99459	1
0.77859	-1.35208	0.99459	1
0.77859	-1.35208	0.99459	1

0.77859	-1.35208	0.99459	1
0.77859	-1.35208	0.99459	1
0.77859	-1.35208	0.99459	1
0.77859	-1.35208	0.99459	1
0.77859	-1.35208	0.99459	1
0.77859	-1.35208	0.99459	1
0.77859	-1.35208	0.99459	1
0.77859	-1.35208	0.99459	1
0.77859	-1.35208	0.99459	1
0.77859	-1.35208	0.99459	1
0.77859	-1.35208	0.99459	1
0.77859	-1.35208	0.99459	1
0.77859	-1.35208	0.99459	1
0.77859	-1.35208	0.99459	1
0.77859	-1.35208	0.99459	1
0.77859	-1.35208	0.99459	1
0.77859	-1.35208	0.99459	1
0.77859	-1.35208	0.99459	1
0.77859	-1.35208	0.99459	1
0.77859	-1.35208	0.99459	1
0.77859	-1.35208	0.99459	1
0.77859	-1.35208	0.99459	1
0.77859	-1.35208	0.99459	1
0.77859	-1.35208	0.99459	1
0.77859	-1.35208	0.99459	1
0.77859	-1.35208	0.99459	1
0.77859	-1.35208	0.99459	1
0.77859	-1.35208	0.99459	1
0.77859	-1.35208	0.99459	1
0.77859	-1.35208	0.99459	1
0.77859	-1.35208	0.99459	1
0.77859	-1.35208	0.99459	1
0.77859	-1.35208	0.99459	1
0.77859	-1.35208	0.99459	1
0.77859	-1.35208	0.99459	1
0.77859	-1.35208	0.99459	1
0.77859	-1.35208	0.99459	1
0.77859	-1.35208	0.99459	1
0.77859	-1.35208	0.99459	1
0.77859	-1.35208	0.99459	1
0.77859	-1.35208	0.99459	1
0.77859	-1.35208	0.99459	1
0.77859	-1.35208	0.99459	1
0.77859	-1.35208	0.99459	1
0.77859	-1.35208	0.99459	1
0.77859	-1.35208	0.99459	1
0.77859	-1.35208	0.99459	1
0.77859	-1.35208	0.99459	1
0.77859	-1.35208	0.99459	1
0.41597	-1.50040	1.09133	1
0.41597	-1.50040	1.09133	1
0.41597	-1.50040	1.09133	1
0.41597	-1.50040	1.09133	1
0.41597	-1.50040	1.09133	1
0.41597	-1.50040	1.09133	1
0.41597	-1.50040	1.09133	1

0.41597	-1.50040	1.09133	1
0.41597	-1.50040	1.09133	1
0.41597	-1.50040	1.09133	1
0.41597	-1.50040	1.09133	1
0.41597	-1.50040	1.09133	1
0.41597	-1.50040	1.09133	1
0.41597	-1.50040	1.09133	1
0.41597	-1.50040	1.09133	1
0.41597	-1.50040	1.09133	1
0.41597	-1.50040	1.09133	1
0.41597	-1.50040	1.09133	1
0.41597	-1.50040	1.09133	1
0.41597	-1.50040	1.09133	1
0.41597	-1.50040	1.09133	1
0.41597	-1.50040	1.09133	1
0.41597	-1.50040	1.09133	1
0.41597	-1.50040	1.09133	1
0.41597	-1.50040	1.09133	1
0.41597	-1.50040	1.09133	1
0.41597	-1.50040	1.09133	1
0.41597	-1.50040	1.09133	1
0.41597	-1.50040	1.09133	1
0.41597	-1.50040	1.09133	1
0.41597	-1.50040	1.09133	1
0.41597	-1.50040	1.09133	1
0.41597	-1.50040	1.09133	1
0.41597	-1.50040	1.09133	1
0.41597	-1.50040	1.09133	1
0.41597	-1.50040	1.09133	1
0.41597	-1.50040	1.09133	1
0.41597	-1.50040	1.09133	1
0.41597	-1.50040	1.09133	1
0.41597	-1.50040	1.09133	1
0.41597	-1.50040	1.09133	1
0.41597	-1.50040	1.09133	1
0.41597	-1.50040	1.09133	1
0.41597	-1.50040	1.09133	1
0.41597	-1.50040	1.09133	1
0.41597	-1.50040	1.09133	1
0.41597	-1.50040	1.09133	1
0.41597	-1.50040	1.09133	1
0.41597	-1.50040	1.09133	1
0.41597	-1.50040	1.09133	1
0.41597	-1.50040	1.09133	1
0.41597	-1.50040	1.09133	1
0.41597	-1.50040	1.09133	1
0.41597	-1.50040	1.09133	1
0.09377	-1.19145	0.79922	0
0.09377	-1.19145	0.79922	0
0.09377	-1.19145	0.79922	0
0.09377	-1.19145	0.79922	0
0.09377	-1.19145	0.79922	0
0.09377	-1.19145	0.79922	0
0.09377	-1.19145	0.79922	0
0.09377	-1.19145	0.79922	0
0.09377	-1.19145	0.79922	0

0.09377	-1.19145	0.79922	0
0.09377	-1.19145	0.79922	0
0.09377	-1.19145	0.79922	0
0.09377	-1.19145	0.79922	0
0.09377	-1.19145	0.79922	0
0.09377	-1.19145	0.79922	0
0.09377	-1.19145	0.79922	0
0.09377	-1.19145	0.79922	0
0.09377	-1.19145	0.79922	0
0.09377	-1.19145	0.79922	0
0.09377	-1.19145	0.79922	0
0.09377	-1.19145	0.79922	0
0.09377	-1.19145	0.79922	0
0.09377	-1.19145	0.79922	0
0.09377	-1.19145	0.79922	0
0.09377	-1.19145	0.79922	0
0.09377	-1.19145	0.79922	0
0.09377	-1.19145	0.79922	0
0.09377	-1.19145	0.79922	0
0.09377	-1.19145	0.79922	0
0.09377	-1.19145	0.79922	0
0.09377	-1.19145	0.79922	0
0.09377	-1.19145	0.79922	0
0.09377	-1.19145	0.79922	0
0.09377	-1.19145	0.79922	0
0.09377	-1.19145	0.79922	0
0.09377	-1.19145	0.79922	0
0.09377	-1.19145	0.79922	0
0.09377	-1.19145	0.79922	0
0.09377	-1.19145	0.79922	0
0.09377	-1.19145	0.79922	0
0.09377	-1.19145	0.79922	0
0.09377	-1.19145	0.79922	0
0.09377	-1.19145	0.79922	0
0.09377	-1.19145	0.79922	0
0.09377	-1.19145	0.79922	0
0.09377	-1.19145	0.79922	0
0.77859	-1.35208	0.99459	1
0.77859	-1.35208	0.99459	1
0.77859	-1.35208	0.99459	1
0.77859	-1.35208	0.99459	1
0.77859	-1.35208	0.99459	1
0.77859	-1.35208	0.99459	1
0.77859	-1.35208	0.99459	1
0.77859	-1.35208	0.99459	1
0.77859	-1.35208	0.99459	1
0.77859	-1.35208	0.99459	1
0.77859	-1.35208	0.99459	1
0.77859	-1.35208	0.99459	1
0.77859	-1.35208	0.99459	1
0.77859	-1.35208	0.99459	1
0.77859	-1.35208	0.99459	1
0.77859	-1.35208	0.99459	1
0.77859	-1.35208	0.99459	1
0.77859	-1.35208	0.99459	1
0.77859	-1.35208	0.99459	1

0.77859	-1.35208	0.99459	1
0.77859	-1.35208	0.99459	1
0.77859	-1.35208	0.99459	1
0.77859	-1.35208	0.99459	1
0.77859	-1.35208	0.99459	1
0.77859	-1.35208	0.99459	1
0.77859	-1.35208	0.99459	1
0.77859	-1.35208	0.99459	1
0.77859	-1.35208	0.99459	1
0.77859	-1.35208	0.99459	1
0.77859	-1.35208	0.99459	1
0.77859	-1.35208	0.99459	1
0.77859	-1.35208	0.99459	1
0.77859	-1.35208	0.99459	1
0.77859	-1.35208	0.99459	1
0.77859	-1.35208	0.99459	1
0.77859	-1.35208	0.99459	1
0.77859	-1.35208	0.99459	1
0.77859	-1.35208	0.99459	1
0.77859	-1.35208	0.99459	1
0.77859	-1.35208	0.99459	1
0.77859	-1.35208	0.99459	1
0.77859	-1.35208	0.99459	1
0.77859	-1.35208	0.99459	1
0.77859	-1.35208	0.99459	1
0.77859	-1.35208	0.99459	1
0.77859	-1.35208	0.99459	1
0.77859	-1.35208	0.99459	1
0.77859	-1.35208	0.99459	1
0.77859	-1.35208	0.99459	1
0.77859	-1.35208	0.99459	1
0.77859	-1.35208	0.99459	1
0.77859	-1.35208	0.99459	1
0.77859	-1.35208	0.99459	1
0.77859	-1.35208	0.99459	1
0.77859	-1.35208	0.99459	1
0.77859	-1.35208	0.99459	1
0.77859	-1.35208	0.99459	1
0.77859	-1.35208	0.99459	1
0.77859	-1.35208	0.99459	1
0.77859	-1.35208	0.99459	1
0.77859	-1.35208	0.99459	1
0.77859	-1.35208	0.99459	1
0.77859	-1.35208	0.99459	1
0.77859	-1.35208	0.99459	1
0.77859	-1.35208	0.99459	1
0.77859	-1.35208	0.99459	1
0.77859	-1.35208	0.99459	1
0.77859	-1.35208	0.99459	1
0.77859	-1.35208	0.99459	1
0.77859	-1.35208	0.99459	1
0.77859	-1.35208	0.99459	1
0.77859	-1.35208	0.99459	1
0.77859	-1.35208	0.99459	1
0.77859	-1.35208	0.99459	1
0.77859	-1.35208	0.99459	1

0.77859	-1.35208	0.99459	1
0.77859	-1.35208	0.99459	1
0.77859	-1.35208	0.99459	1
0.77859	-1.35208	0.99459	1
0.77859	-1.35208	0.99459	1
0.77859	-1.35208	0.99459	1
0.77859	-1.35208	0.99459	1
0.77859	-1.35208	0.99459	1
0.77859	-1.35208	0.99459	1
0.77859	-1.35208	0.99459	1
0.77859	-1.35208	0.99459	1
0.77859	-1.35208	0.99459	1
0.77859	-1.35208	0.99459	1
0.77859	-1.35208	0.99459	1
0.77859	-1.35208	0.99459	1
0.77859	-1.35208	0.99459	1
0.77859	-1.35208	0.99459	1
0.77859	-1.35208	0.99459	1
0.77859	-1.35208	0.99459	1
0.77859	-1.35208	0.99459	1
0.77859	-1.35208	0.99459	1
0.77859	-1.35208	0.99459	1
0.77859	-1.35208	0.99459	1
0.77859	-1.35208	0.99459	1
0.77859	-1.35208	0.99459	1
0.77859	-1.35208	0.99459	1
0.77859	-1.35208	0.99459	1
0.77859	-1.35208	0.99459	1
0.77859	-1.35208	0.99459	1
0.41597	-1.50040	1.09133	1
0.41597	-1.50040	1.09133	1
0.41597	-1.50040	1.09133	1
0.41597	-1.50040	1.09133	1
0.41597	-1.50040	1.09133	1
0.41597	-1.50040	1.09133	1
0.41597	-1.50040	1.09133	1
0.41597	-1.50040	1.09133	1
0.41597	-1.50040	1.09133	1
0.41597	-1.50040	1.09133	1
0.41597	-1.50040	1.09133	1
0.41597	-1.50040	1.09133	1
0.41597	-1.50040	1.09133	1
0.41597	-1.50040	1.09133	1
0.41597	-1.50040	1.09133	1
0.41597	-1.50040	1.09133	1
0.41597	-1.50040	1.09133	1
0.41597	-1.50040	1.09133	1
0.41597	-1.50040	1.09133	1
0.41597	-1.50040	1.09133	1
0.41597	-1.50040	1.09133	1
0.41597	-1.50040	1.09133	1
0.41597	-1.50040	1.09133	1
0.41597	-1.50040	1.09133	1
0.41597	-1.50040	1.09133	1
0.41597	-1.50040	1.09133	1
0.41597	-1.50040	1.09133	1

0.41597	-1.50040	1.09133	1
0.41597	-1.50040	1.09133	1
0.41597	-1.50040	1.09133	1
0.41597	-1.50040	1.09133	1
0.41597	-1.50040	1.09133	1
0.41597	-1.50040	1.09133	1
0.41597	-1.50040	1.09133	1
0.41597	-1.50040	1.09133	1
0.41597	-1.50040	1.09133	1
0.41597	-1.50040	1.09133	1
0.41597	-1.50040	1.09133	1
0.41597	-1.50040	1.09133	1
0.41597	-1.50040	1.09133	1
0.41597	-1.50040	1.09133	1
0.41597	-1.50040	1.09133	1
0.41597	-1.50040	1.09133	1
0.41597	-1.50040	1.09133	1
0.41597	-1.50040	1.09133	1
0.41597	-1.50040	1.09133	1
0.41597	-1.50040	1.09133	1
0.41597	-1.50040	1.09133	1
0.41597	-1.50040	1.09133	1
0.41597	-1.50040	1.09133	1
0.41597	-1.50040	1.09133	1
0.41597	-1.50040	1.09133	1
0.41597	-1.50040	1.09133	1
0.41597	-1.50040	1.09133	1
0.41597	-1.50040	1.09133	1
0.41597	-1.50040	1.09133	1
0.41597	-1.50040	1.09133	1
0.41597	-1.50040	1.09133	1
0.41597	-1.50040	1.09133	1
0.41597	-1.50040	1.09133	1
0.41597	-1.50040	1.09133	1
0.41597	-1.50040	1.09133	1
0.41597	-1.50040	1.09133	1
0.41597	-1.50040	1.09133	1
0.41597	-1.50040	1.09133	1
0.41597	-1.50040	1.09133	1
0.41597	-1.50040	1.09133	1
0.41597	-1.50040	1.09133	1
0.41597	-1.50040	1.09133	1
0.41597	-1.50040	1.09133	1
0.41597	-1.50040	1.09133	1
0.41597	-1.50040	1.09133	1
0.41597	-1.50040	1.09133	1
0.41597	-1.50040	1.09133	1
0.41597	-1.50040	1.09133	1
0.41597	-1.50040	1.09133	1
0.41597	-1.50040	1.09133	1
0.41597	-1.50040	1.09133	1
0.41597	-1.50040	1.09133	1
0.41597	-1.50040	1.09133	1
0.41597	-1.50040	1.09133	1
0.41597	-1.50040	1.09133	1
0.41597	-1.50040	1.09133	1

0.41597	-1.50040	1.09133	1
0.41597	-1.50040	1.09133	1
0.41597	-1.50040	1.09133	1
0.41597	-1.50040	1.09133	1
0.41597	-1.50040	1.09133	1
0.41597	-1.50040	1.09133	1
0.41597	-1.50040	1.09133	1
0.41597	-1.50040	1.09133	1
0.41597	-1.50040	1.09133	1
0.41597	-1.50040	1.09133	1
0.41597	-1.50040	1.09133	1
0.41597	-1.50040	1.09133	1
0.41597	-1.50040	1.09133	1
0.41597	-1.50040	1.09133	1
0.41597	-1.50040	1.09133	1
0.41597	-1.50040	1.09133	1
0.41597	-1.50040	1.09133	1
0.41597	-1.50040	1.09133	1
0.41597	-1.50040	1.09133	1
0.41597	-1.50040	1.09133	1
0.41597	-1.50040	1.09133	1
0.41597	-1.50040	1.09133	1
0.41597	-1.50040	1.09133	1
0.09377	-1.19145	0.79922	0
0.09377	-1.19145	0.79922	0
0.09377	-1.19145	0.79922	0
0.09377	-1.19145	0.79922	0
0.09377	-1.19145	0.79922	0
0.09377	-1.19145	0.79922	0
0.09377	-1.19145	0.79922	0
0.09377	-1.19145	0.79922	0
0.09377	-1.19145	0.79922	0
0.09377	-1.19145	0.79922	0
0.09377	-1.19145	0.79922	0
0.09377	-1.19145	0.79922	0
0.09377	-1.19145	0.79922	0
0.09377	-1.19145	0.79922	0
0.09377	-1.19145	0.79922	0
0.09377	-1.19145	0.79922	0
0.09377	-1.19145	0.79922	0
0.09377	-1.19145	0.79922	0
0.09377	-1.19145	0.79922	0
0.09377	-1.19145	0.79922	0
0.09377	-1.19145	0.79922	0
0.09377	-1.19145	0.79922	0
0.09377	-1.19145	0.79922	0
0.09377	-1.19145	0.79922	0
0.09377	-1.19145	0.79922	0
0.09377	-1.19145	0.79922	0
0.09377	-1.19145	0.79922	0
0.09377	-1.19145	0.79922	0
0.09377	-1.19145	0.79922	0
0.09377	-1.19145	0.79922	0
0.09377	-1.19145	0.79922	0
0.09377	-1.19145	0.79922	0
0.09377	-1.19145	0.79922	0

0.09377	-1.19145	0.79922	0
0.09377	-1.19145	0.79922	0
0.09377	-1.19145	0.79922	0
0.09377	-1.19145	0.79922	0
0.09377	-1.19145	0.79922	0
0.09377	-1.19145	0.79922	0
0.09377	-1.19145	0.79922	0
0.09377	-1.19145	0.79922	0
0.09377	-1.19145	0.79922	0
0.09377	-1.19145	0.79922	0
0.09377	-1.19145	0.79922	0
0.09377	-1.19145	0.79922	0
0.09377	-1.19145	0.79922	0
0.09377	-1.19145	0.79922	0
0.09377	-1.19145	0.79922	0
0.25066	-1.40783	0.72694	0
0.25066	-1.40783	0.72694	0
0.25066	-1.40783	0.72694	0
0.25066	-1.40783	0.72694	0
0.25066	-1.40783	0.72694	0
0.25066	-1.40783	0.72694	0
0.25066	-1.40783	0.72694	0
0.25066	-1.40783	0.72694	0
0.25066	-1.40783	0.72694	0
0.25066	-1.40783	0.72694	0
0.25066	-1.40783	0.72694	0
0.25066	-1.40783	0.72694	0
0.25066	-1.40783	0.72694	0
0.25066	-1.40783	0.72694	0
0.25066	-1.40783	0.72694	0
0.25066	-1.40783	0.72694	0
0.25066	-1.40783	0.72694	0
0.25066	-1.40783	0.72694	0
0.25066	-1.40783	0.72694	0
0.25066	-1.40783	0.72694	0
0.25066	-1.40783	0.72694	0
0.25066	-1.40783	0.72694	0
0.25066	-1.40783	0.72694	0
0.25066	-1.40783	0.72694	0
0.25066	-1.40783	0.72694	0
0.25066	-1.40783	0.72694	0
0.25066	-1.40783	0.72694	0
0.25066	-1.40783	0.72694	0
0.25066	-1.40783	0.72694	0
0.25066	-1.40783	0.72694	0
0.25066	-1.40783	0.72694	0
0.25066	-1.40783	0.72694	0
0.25066	-1.40783	0.72694	0
0.25066	-1.40783	0.72694	0
0.25066	-1.40783	0.72694	0
0.25066	-1.40783	0.72694	0
0.25066	-1.40783	0.72694	0
0.25066	-1.40783	0.72694	0
0.25066	-1.40783	0.72694	0
0.25066	-1.40783	0.72694	0
0.25066	-1.40783	0.72694	0

0.25066	-1.40783	0.72694	0
0.25066	-1.40783	0.72694	0
0.25066	-1.40783	0.72694	0
0.25066	-1.40783	0.72694	0
0.25066	-1.40783	0.72694	0
0.25066	-1.40783	0.72694	0
0.25066	-1.40783	0.72694	0
0.25066	-1.40783	0.72694	0
0.25066	-1.40783	0.72694	0
0.25066	-1.40783	0.72694	0
0.25066	-1.40783	0.72694	0
0.25066	-1.40783	0.72694	0
0.25066	-1.40783	0.72694	0
0.25066	-1.40783	0.72694	0
0.25066	-1.40783	0.72694	0
0.25066	-1.40783	0.72694	0
0.25066	-1.40783	0.72694	0
0.25066	-1.40783	0.72694	0
0.25066	-1.40783	0.72694	0
0.25066	-1.40783	0.72694	0
0.25066	-1.40783	0.72694	0
0.25066	-1.40783	0.72694	0
0.25066	-1.40783	0.72694	0
0.25066	-1.40783	0.72694	0
0.25066	-1.40783	0.72694	0
0.25066	-1.40783	0.72694	0
0.25066	-1.40783	0.72694	0
0.25066	-1.40783	0.72694	0
0.25066	-1.40783	0.72694	0
0.25066	-1.40783	0.72694	0
0.25066	-1.40783	0.72694	0
0.25066	-1.40783	0.72694	0
0.25066	-1.40783	0.72694	0
0.25066	-1.40783	0.72694	0
0.25066	-1.40783	0.72694	0
0.25066	-1.40783	0.72694	0
0.25066	-1.40783	0.72694	0
0.25066	-1.40783	0.72694	0
0.25066	-1.40783	0.72694	0
0.25066	-1.40783	0.72694	0
0.25066	-1.40783	0.72694	0
0.25066	-1.40783	0.72694	0
0.25066	-1.40783	0.72694	0
0.25066	-1.40783	0.72694	0
0.25066	-1.40783	0.72694	0
0.25066	-1.40783	0.72694	0
0.25066	-1.40783	0.72694	0
0.25066	-1.40783	0.72694	0
0.25066	-1.40783	0.72694	0
0.25066	-1.40783	0.72694	0
0.25066	-1.40783	0.72694	0
0.25066	-1.40783	0.72694	0
0.25066	-1.40783	0.72694	0
0.25066	-1.40783	0.72694	0
0.25066	-1.40783	0.72694	0
0.25066	-1.40783	0.72694	0

0.25066	-1.40783	0.72694	0
0.25066	-1.40783	0.72694	0
0.25066	-1.40783	0.72694	0
0.25066	-1.40783	0.72694	0
0.25066	-1.40783	0.72694	0
0.25066	-1.40783	0.72694	0
0.25066	-1.40783	0.72694	0
0.25066	-1.40783	0.72694	0
0.25066	-1.40783	0.72694	0
0.25066	-1.40783	0.72694	0
0.25066	-1.40783	0.72694	0
0.25066	-1.40783	0.72694	0
0.25066	-1.40783	0.72694	0
0.25066	-1.40783	0.72694	0
0.25066	-1.40783	0.72694	0
0.25066	-1.40783	0.72694	0
0.25066	-1.40783	0.72694	0
0.25066	-1.40783	0.72694	0
0.25066	-1.40783	0.72694	0
0.25066	-1.40783	0.72694	0
0.25066	-1.40783	0.72694	0
0.25066	-1.40783	0.72694	0
0.25066	-1.40783	0.72694	0
0.25066	-1.40783	0.72694	0
0.25066	-1.40783	0.72694	0
0.25066	-1.40783	0.72694	0
0.25066	-1.40783	0.72694	0
0.25066	-1.40783	0.72694	0
0.25066	-1.40783	0.72694	0
0.25066	-1.40783	0.72694	0
0.25066	-1.40783	0.72694	0
0.41597	-1.50040	1.09133	1
0.41597	-1.50040	1.09133	1
0.41597	-1.50040	1.09133	1
0.41597	-1.50040	1.09133	1
0.41597	-1.50040	1.09133	1
0.41597	-1.50040	1.09133	1
0.41597	-1.50040	1.09133	1
0.41597	-1.50040	1.09133	1
0.41597	-1.50040	1.09133	1
0.41597	-1.50040	1.09133	1
0.41597	-1.50040	1.09133	1
0.41597	-1.50040	1.09133	1
0.41597	-1.50040	1.09133	1
0.41597	-1.50040	1.09133	1
0.41597	-1.50040	1.09133	1
0.41597	-1.50040	1.09133	1
0.41597	-1.50040	1.09133	1
0.41597	-1.50040	1.09133	1
0.41597	-1.50040	1.09133	1
0.41597	-1.50040	1.09133	1
0.41597	-1.50040	1.09133	1
0.41597	-1.50040	1.09133	1
0.41597	-1.50040	1.09133	1
0.41597	-1.50040	1.09133	1
0.41597	-1.50040	1.09133	1

0.41597	-1.50040	1.09133	1
0.41597	-1.50040	1.09133	1
0.41597	-1.50040	1.09133	1
0.41597	-1.50040	1.09133	1
0.41597	-1.50040	1.09133	1
0.41597	-1.50040	1.09133	1
0.41597	-1.50040	1.09133	1
0.41597	-1.50040	1.09133	1
0.41597	-1.50040	1.09133	1
0.41597	-1.50040	1.09133	1
0.41597	-1.50040	1.09133	1
0.41597	-1.50040	1.09133	1
0.41597	-1.50040	1.09133	1
0.41597	-1.50040	1.09133	1
0.41597	-1.50040	1.09133	1
0.41597	-1.50040	1.09133	1
0.41597	-1.50040	1.09133	1
0.41597	-1.50040	1.09133	1
0.41597	-1.50040	1.09133	1
0.41597	-1.50040	1.09133	1
0.41597	-1.50040	1.09133	1
0.41597	-1.50040	1.09133	1
0.41597	-1.50040	1.09133	1
0.41597	-1.50040	1.09133	1
0.41597	-1.50040	1.09133	1
0.41597	-1.50040	1.09133	1
0.41597	-1.50040	1.09133	1
0.41597	-1.50040	1.09133	1
0.41597	-1.50040	1.09133	1
0.25066	-1.40783	0.72694	0
0.25066	-1.40783	0.72694	0
0.25066	-1.40783	0.72694	0
0.25066	-1.40783	0.72694	0
0.25066	-1.40783	0.72694	0
0.25066	-1.40783	0.72694	0
0.25066	-1.40783	0.72694	0
0.25066	-1.40783	0.72694	0
0.25066	-1.40783	0.72694	0
0.25066	-1.40783	0.72694	0
0.25066	-1.40783	0.72694	0
0.25066	-1.40783	0.72694	0
0.25066	-1.40783	0.72694	0
0.25066	-1.40783	0.72694	0
0.25066	-1.40783	0.72694	0
0.25066	-1.40783	0.72694	0
0.25066	-1.40783	0.72694	0
0.25066	-1.40783	0.72694	0
0.25066	-1.40783	0.72694	0
0.25066	-1.40783	0.72694	0
0.25066	-1.40783	0.72694	0
0.25066	-1.40783	0.72694	0
0.25066	-1.40783	0.72694	0
0.25066	-1.40783	0.72694	0
0.25066	-1.40783	0.72694	0
0.25066	-1.40783	0.72694	0
0.25066	-1.40783	0.72694	0

0.25066	-1.40783	0.72694	0
0.25066	-1.40783	0.72694	0
0.25066	-1.40783	0.72694	0
0.25066	-1.40783	0.72694	0
0.25066	-1.40783	0.72694	0
0.25066	-1.40783	0.72694	0
0.25066	-1.40783	0.72694	0
0.25066	-1.40783	0.72694	0
0.25066	-1.40783	0.72694	0
0.25066	-1.40783	0.72694	0
0.25066	-1.40783	0.72694	0
0.25066	-1.40783	0.72694	0
0.25066	-1.40783	0.72694	0
0.25066	-1.40783	0.72694	0
0.25066	-1.40783	0.72694	0
0.25066	-1.40783	0.72694	0
0.25066	-1.40783	0.72694	0
0.25066	-1.40783	0.72694	0
0.25066	-1.40783	0.72694	0
0.25066	-1.40783	0.72694	0
0.25066	-1.40783	0.72694	0
0.25066	-1.40783	0.72694	0
0.25066	-1.40783	0.72694	0
0.25066	-1.40783	0.72694	0
0.25066	-1.40783	0.72694	0
0.25066	-1.40783	0.72694	0
0.25066	-1.40783	0.72694	0
0.25066	-1.40783	0.72694	0
0.25066	-1.40783	0.72694	0
0.25066	-1.40783	0.72694	0
0.25066	-1.40783	0.72694	0
0.25066	-1.40783	0.72694	0
0.25066	-1.40783	0.72694	0
0.25066	-1.40783	0.72694	0
0.25066	-1.40783	0.72694	0
0.25066	-1.40783	0.72694	0
0.25066	-1.40783	0.72694	0
0.25066	-1.40783	0.72694	0
0.25066	-1.40783	0.72694	0
0.25066	-1.40783	0.72694	0
0.25066	-1.40783	0.72694	0
0.25066	-1.40783	0.72694	0
0.25066	-1.40783	0.72694	0
0.25066	-1.40783	0.72694	0
0.25066	-1.40783	0.72694	0
0.25066	-1.40783	0.72694	0
0.25066	-1.40783	0.72694	0
0.25066	-1.40783	0.72694	0
0.25066	-1.40783	0.72694	0
0.25066	-1.40783	0.72694	0
0.25066	-1.40783	0.72694	0
0.25066	-1.40783	0.72694	0
0.25066	-1.40783	0.72694	0
0.25066	-1.40783	0.72694	0
0.25066	-1.40783	0.72694	0
0.25066	-1.40783	0.72694	0

0.25066	-1.40783	0.72694	0
0.25066	-1.40783	0.72694	0
0.25066	-1.40783	0.72694	0
0.25066	-1.40783	0.72694	0
0.25066	-1.40783	0.72694	0
0.25066	-1.40783	0.72694	0
0.25066	-1.40783	0.72694	0
0.25066	-1.40783	0.72694	0
0.25066	-1.40783	0.72694	0
0.25066	-1.40783	0.72694	0
0.25066	-1.40783	0.72694	0
0.25066	-1.40783	0.72694	0
0.25066	-1.40783	0.72694	0
0.25066	-1.40783	0.72694	0
0.25066	-1.40783	0.72694	0
0.25066	-1.40783	0.72694	0
0.25066	-1.40783	0.72694	0
0.25066	-1.40783	0.72694	0
0.25066	-1.40783	0.72694	0
0.25066	-1.40783	0.72694	0
0.25066	-1.40783	0.72694	0
0.25066	-1.40783	0.72694	0
0.25066	-1.40783	0.72694	0
0.25066	-1.40783	0.72694	0
0.25066	-1.40783	0.72694	0
0.25066	-1.40783	0.72694	0
0.25066	-1.40783	0.72694	0
0.25066	-1.40783	0.72694	0
0.25066	-1.40783	0.72694	0
0.25066	-1.40783	0.72694	0
0.25066	-1.40783	0.72694	0
0.25066	-1.40783	0.72694	0
0.25066	-1.40783	0.72694	0
0.25066	-1.40783	0.72694	0
0.25066	-1.40783	0.72694	0
0.25066	-1.40783	0.72694	0
0.25066	-1.40783	0.72694	0
0.25066	-1.40783	0.72694	0
0.25066	-1.40783	0.72694	0
0.25066	-1.40783	0.72694	0
0.25066	-1.40783	0.72694	0
0.25066	-1.40783	0.72694	0
0.25066	-1.40783	0.72694	0
0.25066	-1.40783	0.72694	0
0.25066	-1.40783	0.72694	0
0.25066	-1.40783	0.72694	0
0.25066	-1.40783	0.72694	0
0.25066	-1.40783	0.72694	0
0.25066	-1.40783	0.72694	0
0.25066	-1.40783	0.72694	0
0.25066	-1.40783	0.72694	0
0.25066	-1.40783	0.72694	0
0.25066	-1.40783	0.72694	0
0.25066	-1.40783	0.72694	0
0.25066	-1.40783	0.72694	0
0.25066	-1.40783	0.72694	0

0.25066	-1.40783	0.72694	0
0.25066	-1.40783	0.72694	0
0.25066	-1.40783	0.72694	0
0.25066	-1.40783	0.72694	0
0.25066	-1.40783	0.72694	0
0.25066	-1.40783	0.72694	0
0.25066	-1.40783	0.72694	0
0.25066	-1.40783	0.72694	0
0.25066	-1.40783	0.72694	0
0.25066	-1.40783	0.72694	0
0.25066	-1.40783	0.72694	0
0.25066	-1.40783	0.72694	0
0.25066	-1.40783	0.72694	0
0.25066	-1.40783	0.72694	0
0.25066	-1.40783	0.72694	0
0.25066	-1.40783	0.72694	0
0.25066	-1.40783	0.72694	0
0.25066	-1.40783	0.72694	0
0.25066	-1.40783	0.72694	0
0.25066	-1.40783	0.72694	0
0.25066	-1.40783	0.72694	0
0.25066	-1.40783	0.72694	0
0.25066	-1.40783	0.72694	0
0.25066	-1.40783	0.72694	0
0.25066	-1.40783	0.72694	0
0.25066	-1.40783	0.72694	0
0.25066	-1.40783	0.72694	0
0.25066	-1.40783	0.72694	0
0.25066	-1.40783	0.72694	0
0.25066	-1.40783	0.72694	0
0.25066	-1.40783	0.72694	0
0.25066	-1.40783	0.72694	0
0.25066	-1.40783	0.72694	0
0.25066	-1.40783	0.72694	0
0.25066	-1.40783	0.72694	0
0.25066	-1.40783	0.72694	0
0.25066	-1.40783	0.72694	0
0.25066	-1.40783	0.72694	0
0.25066	-1.40783	0.72694	0
0.25066	-1.40783	0.72694	0
0.25066	-1.40783	0.72694	0
0.25066	-1.40783	0.72694	0
0.25066	-1.40783	0.72694	0
0.25066	-1.40783	0.72694	0
0.25066	-1.40783	0.72694	0
0.25066	-1.40783	0.72694	0
0.25066	-1.40783	0.72694	0
0.25066	-1.40783	0.72694	0
0.25066	-1.40783	0.72694	0
0.25066	-1.40783	0.72694	0
0.25066	-1.40783	0.72694	0
0.25066	-1.40783	0.72694	0
0.25066	-1.40783	0.72694	0
0.41597	-1.50040	1.09133	1
0.41597	-1.50040	1.09133	1
0.41597	-1.50040	1.09133	1

0.41597	-1.50040	1.09133	1
0.41597	-1.50040	1.09133	1
0.41597	-1.50040	1.09133	1
0.41597	-1.50040	1.09133	1
0.41597	-1.50040	1.09133	1
0.41597	-1.50040	1.09133	1
0.41597	-1.50040	1.09133	1
0.41597	-1.50040	1.09133	1
0.41597	-1.50040	1.09133	1
0.41597	-1.50040	1.09133	1
0.41597	-1.50040	1.09133	1
0.41597	-1.50040	1.09133	1
0.41597	-1.50040	1.09133	1
0.41597	-1.50040	1.09133	1
0.41597	-1.50040	1.09133	1
0.41597	-1.50040	1.09133	1
0.41597	-1.50040	1.09133	1
0.41597	-1.50040	1.09133	1
0.41597	-1.50040	1.09133	1
0.41597	-1.50040	1.09133	1
0.41597	-1.50040	1.09133	1
0.41597	-1.50040	1.09133	1
0.41597	-1.50040	1.09133	1
0.41597	-1.50040	1.09133	1
0.41597	-1.50040	1.09133	1
0.41597	-1.50040	1.09133	1
0.41597	-1.50040	1.09133	1
0.41597	-1.50040	1.09133	1
0.41597	-1.50040	1.09133	1
0.41597	-1.50040	1.09133	1
0.41597	-1.50040	1.09133	1
0.41597	-1.50040	1.09133	1
0.41597	-1.50040	1.09133	1
0.41597	-1.50040	1.09133	1
0.41597	-1.50040	1.09133	1
0.41597	-1.50040	1.09133	1
0.41597	-1.50040	1.09133	1
0.41597	-1.50040	1.09133	1
0.41597	-1.50040	1.09133	1
0.41597	-1.50040	1.09133	1
0.41597	-1.50040	1.09133	1
0.41597	-1.50040	1.09133	1
0.41597	-1.50040	1.09133	1
0.41597	-1.50040	1.09133	1
0.41597	-1.50040	1.09133	1
0.41597	-1.50040	1.09133	1
0.41597	-1.50040	1.09133	1
0.41597	-1.50040	1.09133	1
0.41597	-1.50040	1.09133	1
0.41597	-1.50040	1.09133	1
0.41597	-1.50040	1.09133	1
0.25066	-1.40783	0.72694	0
0.25066	-1.40783	0.72694	0
0.25066	-1.40783	0.72694	0
0.25066	-1.40783	0.72694	0
0.25066	-1.40783	0.72694	0

0.25066	-1.40783	0.72694	0
0.25066	-1.40783	0.72694	0
0.25066	-1.40783	0.72694	0
0.25066	-1.40783	0.72694	0
0.25066	-1.40783	0.72694	0
0.25066	-1.40783	0.72694	0
0.25066	-1.40783	0.72694	0
0.25066	-1.40783	0.72694	0
0.25066	-1.40783	0.72694	0
0.25066	-1.40783	0.72694	0
0.25066	-1.40783	0.72694	0
0.25066	-1.40783	0.72694	0
0.25066	-1.40783	0.72694	0
0.25066	-1.40783	0.72694	0
0.25066	-1.40783	0.72694	0
0.25066	-1.40783	0.72694	0
0.25066	-1.40783	0.72694	0
0.25066	-1.40783	0.72694	0
0.25066	-1.40783	0.72694	0
0.25066	-1.40783	0.72694	0
0.25066	-1.40783	0.72694	0
0.25066	-1.40783	0.72694	0
0.25066	-1.40783	0.72694	0
0.25066	-1.40783	0.72694	0
0.25066	-1.40783	0.72694	0
0.25066	-1.40783	0.72694	0
0.25066	-1.40783	0.72694	0
0.25066	-1.40783	0.72694	0
0.25066	-1.40783	0.72694	0
0.25066	-1.40783	0.72694	0
0.25066	-1.40783	0.72694	0
0.25066	-1.40783	0.72694	0
0.25066	-1.40783	0.72694	0
0.25066	-1.40783	0.72694	0
0.25066	-1.40783	0.72694	0
0.25066	-1.40783	0.72694	0
0.25066	-1.40783	0.72694	0
0.25066	-1.40783	0.72694	0
0.25066	-1.40783	0.72694	0
0.25066	-1.40783	0.72694	0
0.25066	-1.40783	0.72694	0
0.25066	-1.40783	0.72694	0
0.25066	-1.40783	0.72694	0
0.25066	-1.40783	0.72694	0
0.25066	-1.40783	0.72694	0
0.25066	-1.40783	0.72694	0
0.25066	-1.40783	0.72694	0
0.25066	-1.40783	0.72694	0
0.25066	-1.40783	0.72694	0
0.25066	-1.40783	0.72694	0
0.25066	-1.40783	0.72694	0
0.25066	-1.40783	0.72694	0
0.25066	-1.40783	0.72694	0
0.25066	-1.40783	0.72694	0
0.25066	-1.40783	0.72694	0
0.25066	-1.40783	0.72694	0

0.25066	-1.40783	0.72694	0
0.25066	-1.40783	0.72694	0
0.25066	-1.40783	0.72694	0
0.09377	-1.19145	0.79922	0
0.09377	-1.19145	0.79922	0
0.09377	-1.19145	0.79922	0
0.09377	-1.19145	0.79922	0
0.09377	-1.19145	0.79922	0
0.09377	-1.19145	0.79922	0
0.09377	-1.19145	0.79922	0
0.09377	-1.19145	0.79922	0
0.09377	-1.19145	0.79922	0
0.09377	-1.19145	0.79922	0
0.09377	-1.19145	0.79922	0
0.09377	-1.19145	0.79922	0
0.09377	-1.19145	0.79922	0
0.09377	-1.19145	0.79922	0
0.09377	-1.19145	0.79922	0
0.09377	-1.19145	0.79922	0
0.09377	-1.19145	0.79922	0
0.09377	-1.19145	0.79922	0
0.09377	-1.19145	0.79922	0
0.09377	-1.19145	0.79922	0
0.09377	-1.19145	0.79922	0
0.09377	-1.19145	0.79922	0
0.09377	-1.19145	0.79922	0
0.09377	-1.19145	0.79922	0
0.09377	-1.19145	0.79922	0
0.09377	-1.19145	0.79922	0
0.09377	-1.19145	0.79922	0
0.09377	-1.19145	0.79922	0
0.09377	-1.19145	0.79922	0
0.09377	-1.19145	0.79922	0
0.09377	-1.19145	0.79922	0
0.09377	-1.19145	0.79922	0
0.09377	-1.19145	0.79922	0
0.09377	-1.19145	0.79922	0
0.09377	-1.19145	0.79922	0
0.09377	-1.19145	0.79922	0
0.09377	-1.19145	0.79922	0
0.09377	-1.19145	0.79922	0
0.09377	-1.19145	0.79922	0
0.09377	-1.19145	0.79922	0
0.09377	-1.19145	0.79922	0
0.09377	-1.19145	0.79922	0
0.09377	-1.19145	0.79922	0
0.09377	-1.19145	0.79922	0
0.09377	-1.19145	0.79922	0
0.09377	-1.19145	0.79922	0
0.09377	-1.19145	0.79922	0
0.09377	-1.19145	0.79922	0
0.09377	-1.19145	0.79922	0
0.09377	-1.19145	0.79922	0
0.09377	-1.19145	0.79922	0
0.09377	-1.19145	0.79922	0
0.09377	-1.19145	0.79922	0

0.09377	-1.19145	0.79922	0
0.09377	-1.19145	0.79922	0
0.09377	-1.19145	0.79922	0
0.09377	-1.19145	0.79922	0
0.09377	-1.19145	0.79922	0
0.09377	-1.19145	0.79922	0
0.09377	-1.19145	0.79922	0
0.09377	-1.19145	0.79922	0
0.09377	-1.19145	0.79922	0
0.09377	-1.19145	0.79922	0
0.09377	-1.19145	0.79922	0
0.09377	-1.19145	0.79922	0
0.09377	-1.19145	0.79922	0
0.09377	-1.19145	0.79922	0
0.09377	-1.19145	0.79922	0
0.09377	-1.19145	0.79922	0
0.09377	-1.19145	0.79922	0
0.09377	-1.19145	0.79922	0
0.09377	-1.19145	0.79922	0
0.09377	-1.19145	0.79922	0
0.09377	-1.19145	0.79922	0
0.09377	-1.19145	0.79922	0
0.09377	-1.19145	0.79922	0
0.09377	-1.19145	0.79922	0
0.09377	-1.19145	0.79922	0
0.09377	-1.19145	0.79922	0
0.09377	-1.19145	0.79922	0
0.09377	-1.19145	0.79922	0
0.09377	-1.19145	0.79922	0
0.09377	-1.19145	0.79922	0
0.09377	-1.19145	0.79922	0
0.09377	-1.19145	0.79922	0
0.09377	-1.19145	0.79922	0
0.09377	-1.19145	0.79922	0
0.09377	-1.19145	0.79922	0
0.09377	-1.19145	0.79922	0
0.09377	-1.19145	0.79922	0
0.09377	-1.19145	0.79922	0
0.09377	-1.19145	0.79922	0
0.09377	-1.19145	0.79922	0
0.09377	-1.19145	0.79922	0
0.09377	-1.19145	0.79922	0
0.25066	-1.40783	0.72694	0
0.25066	-1.40783	0.72694	0
0.25066	-1.40783	0.72694	0
0.25066	-1.40783	0.72694	0
0.25066	-1.40783	0.72694	0
0.25066	-1.40783	0.72694	0
0.25066	-1.40783	0.72694	0
0.25066	-1.40783	0.72694	0
0.25066	-1.40783	0.72694	0
0.25066	-1.40783	0.72694	0
0.25066	-1.40783	0.72694	0
0.25066	-1.40783	0.72694	0
0.25066	-1.40783	0.72694	0
0.25066	-1.40783	0.72694	0

0.25066	-1.40783	0.72694	0
0.25066	-1.40783	0.72694	0
0.25066	-1.40783	0.72694	0
0.25066	-1.40783	0.72694	0
0.25066	-1.40783	0.72694	0
0.25066	-1.40783	0.72694	0
0.25066	-1.40783	0.72694	0
0.25066	-1.40783	0.72694	0
0.25066	-1.40783	0.72694	0
0.25066	-1.40783	0.72694	0
0.25066	-1.40783	0.72694	0
0.25066	-1.40783	0.72694	0
0.25066	-1.40783	0.72694	0
0.25066	-1.40783	0.72694	0
0.25066	-1.40783	0.72694	0
0.25066	-1.40783	0.72694	0
0.25066	-1.40783	0.72694	0
0.25066	-1.40783	0.72694	0
0.25066	-1.40783	0.72694	0
0.25066	-1.40783	0.72694	0
0.25066	-1.40783	0.72694	0
0.25066	-1.40783	0.72694	0
0.25066	-1.40783	0.72694	0
0.25066	-1.40783	0.72694	0
0.25066	-1.40783	0.72694	0
0.25066	-1.40783	0.72694	0
0.25066	-1.40783	0.72694	0
0.25066	-1.40783	0.72694	0
0.25066	-1.40783	0.72694	0
0.25066	-1.40783	0.72694	0
0.25066	-1.40783	0.72694	0
0.25066	-1.40783	0.72694	0
0.25066	-1.40783	0.72694	0
0.25066	-1.40783	0.72694	0
0.25066	-1.40783	0.72694	0
0.25066	-1.40783	0.72694	0
0.25066	-1.40783	0.72694	0
0.25066	-1.40783	0.72694	0
0.25066	-1.40783	0.72694	0
0.25066	-1.40783	0.72694	0
0.25066	-1.40783	0.72694	0
0.25066	-1.40783	0.72694	0
0.25066	-1.40783	0.72694	0
0.25066	-1.40783	0.72694	0
0.25066	-1.40783	0.72694	0
0.25066	-1.40783	0.72694	0
0.25066	-1.40783	0.72694	0
0.25066	-1.40783	0.72694	0
0.25066	-1.40783	0.72694	0
0.25066	-1.40783	0.72694	0
0.25066	-1.40783	0.72694	0
0.25066	-1.40783	0.72694	0
0.25066	-1.40783	0.72694	0
0.25066	-1.40783	0.72694	0
0.25066	-1.40783	0.72694	0
0.25066	-1.40783	0.72694	0

0.25066	-1.40783	0.72694	0
0.25066	-1.40783	0.72694	0
0.25066	-1.40783	0.72694	0
0.25066	-1.40783	0.72694	0
0.25066	-1.40783	0.72694	0
0.25066	-1.40783	0.72694	0
0.25066	-1.40783	0.72694	0
0.25066	-1.40783	0.72694	0
0.25066	-1.40783	0.72694	0
0.25066	-1.40783	0.72694	0
0.25066	-1.40783	0.72694	0
0.25066	-1.40783	0.72694	0
0.25066	-1.40783	0.72694	0
0.25066	-1.40783	0.72694	0
0.25066	-1.40783	0.72694	0
0.25066	-1.40783	0.72694	0
0.25066	-1.40783	0.72694	0
0.25066	-1.40783	0.72694	0
0.25066	-1.40783	0.72694	0
0.25066	-1.40783	0.72694	0
0.25066	-1.40783	0.72694	0
0.25066	-1.40783	0.72694	0
0.25066	-1.40783	0.72694	0
0.25066	-1.40783	0.72694	0
0.25066	-1.40783	0.72694	0
0.25066	-1.40783	0.72694	0
0.25066	-1.40783	0.72694	0
0.25066	-1.40783	0.72694	0
0.25066	-1.40783	0.72694	0
0.25066	-1.40783	0.72694	0
0.25066	-1.40783	0.72694	0
0.25066	-1.40783	0.72694	0
0.25066	-1.40783	0.72694	0
0.25066	-1.40783	0.72694	0
0.25066	-1.40783	0.72694	0
0.25066	-1.40783	0.72694	0
0.25066	-1.40783	0.72694	0
0.25066	-1.40783	0.72694	0
0.25066	-1.40783	0.72694	0
0.25066	-1.40783	0.72694	0
0.25066	-1.40783	0.72694	0
0.25066	-1.40783	0.72694	0
0.25066	-1.40783	0.72694	0
0.25066	-1.40783	0.72694	0
0.25066	-1.40783	0.72694	0
0.25066	-1.40783	0.72694	0
0.25066	-1.40783	0.72694	0
0.25066	-1.40783	0.72694	0
0.25066	-1.40783	0.72694	0
0.25066	-1.40783	0.72694	0
0.25066	-1.40783	0.72694	0
0.25066	-1.40783	0.72694	0
0.25066	-1.40783	0.72694	0
0.25066	-1.40783	0.72694	0
0.25066	-1.40783	0.72694	0
0.25066	-1.40783	0.72694	0

0.25066	-1.40783	0.72694	0
0.25066	-1.40783	0.72694	0
0.25066	-1.40783	0.72694	0
0.25066	-1.40783	0.72694	0
0.25066	-1.40783	0.72694	0
0.25066	-1.40783	0.72694	0
0.25066	-1.40783	0.72694	0
0.25066	-1.40783	0.72694	0
0.25066	-1.40783	0.72694	0
0.25066	-1.40783	0.72694	0
0.25066	-1.40783	0.72694	0
0.25066	-1.40783	0.72694	0
0.25066	-1.40783	0.72694	0
0.25066	-1.40783	0.72694	0
0.25066	-1.40783	0.72694	0
0.25066	-1.40783	0.72694	0
0.25066	-1.40783	0.72694	0
0.25066	-1.40783	0.72694	0
0.25066	-1.40783	0.72694	0
0.25066	-1.40783	0.72694	0
0.25066	-1.40783	0.72694	0
0.25066	-1.40783	0.72694	0
0.25066	-1.40783	0.72694	0
0.25066	-1.40783	0.72694	0
0.25066	-1.40783	0.72694	0
0.25066	-1.40783	0.72694	0
0.25066	-1.40783	0.72694	0
0.25066	-1.40783	0.72694	0
0.25066	-1.40783	0.72694	0
0.25066	-1.40783	0.72694	0
0.25066	-1.40783	0.72694	0
0.25066	-1.40783	0.72694	0
0.25066	-1.40783	0.72694	0
0.25066	-1.40783	0.72694	0
0.25066	-1.40783	0.72694	0
0.25066	-1.40783	0.72694	0
0.25066	-1.40783	0.72694	0
0.25066	-1.40783	0.72694	0
0.25066	-1.40783	0.72694	0
0.25066	-1.40783	0.72694	0
0.25066	-1.40783	0.72694	0
0.25066	-1.40783	0.72694	0
0.25066	-1.40783	0.72694	0
0.25066	-1.40783	0.72694	0
0.25066	-1.40783	0.72694	0
0.25066	-1.40783	0.72694	0
0.25066	-1.40783	0.72694	0
0.25066	-1.40783	0.72694	0
0.25066	-1.40783	0.72694	0
0.25066	-1.40783	0.72694	0
0.25066	-1.40783	0.72694	0
0.25066	-1.40783	0.72694	0
0.25066	-1.40783	0.72694	0
0.25066	-1.40783	0.72694	0
0.25066	-1.40783	0.72694	0
0.25066	-1.40783	0.72694	0

0.25066	-1.40783	0.72694	0
0.25066	-1.40783	0.72694	0
0.25066	-1.40783	0.72694	0
0.25066	-1.40783	0.72694	0
0.25066	-1.40783	0.72694	0
0.25066	-1.40783	0.72694	0
0.25066	-1.40783	0.72694	0
0.25066	-1.40783	0.72694	0
0.25066	-1.40783	0.72694	0
0.25066	-1.40783	0.72694	0
0.25066	-1.40783	0.72694	0
0.25066	-1.40783	0.72694	0
0.25066	-1.40783	0.72694	0
0.25066	-1.40783	0.72694	0
0.25066	-1.40783	0.72694	0
0.25066	-1.40783	0.72694	0
0.25066	-1.40783	0.72694	0
0.25066	-1.40783	0.72694	0
0.25066	-1.40783	0.72694	0
0.25066	-1.40783	0.72694	0
0.25066	-1.40783	0.72694	0
0.25066	-1.40783	0.72694	0
0.25066	-1.40783	0.72694	0
0.25066	-1.40783	0.72694	0
0.25066	-1.40783	0.72694	0
0.25066	-1.40783	0.72694	0
0.25066	-1.40783	0.72694	0
0.25066	-1.40783	0.72694	0
0.25066	-1.40783	0.72694	0
0.25066	-1.40783	0.72694	0
0.25066	-1.40783	0.72694	0
0.25066	-1.40783	0.72694	0
0.25066	-1.40783	0.72694	0
0.25066	-1.40783	0.72694	0
0.25066	-1.40783	0.72694	0
0.25066	-1.40783	0.72694	0
0.25066	-1.40783	0.72694	0
0.25066	-1.40783	0.72694	0
0.25066	-1.40783	0.72694	0
0.25066	-1.40783	0.72694	0
0.25066	-1.40783	0.72694	0
0.25066	-1.40783	0.72694	0
0.25066	-1.40783	0.72694	0
0.25066	-1.40783	0.72694	0
0.25066	-1.40783	0.72694	0
0.25066	-1.40783	0.72694	0
0.25066	-1.40783	0.72694	0
0.25066	-1.40783	0.72694	0
0.25066	-1.40783	0.72694	0
0.25066	-1.40783	0.72694	0
0.25066	-1.40783	0.72694	0
0.25066	-1.40783	0.72694	0
0.25066	-1.40783	0.72694	0
0.25066	-1.40783	0.72694	0
0.25066	-1.40783	0.72694	0
0.25066	-1.40783	0.72694	0

0.25066	-1.40783	0.72694	0
0.25066	-1.40783	0.72694	0
0.25066	-1.40783	0.72694	0
0.25066	-1.40783	0.72694	0
0.25066	-1.40783	0.72694	0
0.25066	-1.40783	0.72694	0
0.25066	-1.40783	0.72694	0
0.25066	-1.40783	0.72694	0
0.25066	-1.40783	0.72694	0
0.25066	-1.40783	0.72694	0
0.25066	-1.40783	0.72694	0
0.25066	-1.40783	0.72694	0
0.25066	-1.40783	0.72694	0
0.25066	-1.40783	0.72694	0
0.25066	-1.40783	0.72694	0
0.25066	-1.40783	0.72694	0
0.25066	-1.40783	0.72694	0
0.25066	-1.40783	0.72694	0
0.09377	-1.19145	0.79922	0
0.09377	-1.19145	0.79922	0
0.09377	-1.19145	0.79922	0
0.09377	-1.19145	0.79922	0
0.09377	-1.19145	0.79922	0
0.09377	-1.19145	0.79922	0
0.09377	-1.19145	0.79922	0
0.09377	-1.19145	0.79922	0
0.09377	-1.19145	0.79922	0
0.09377	-1.19145	0.79922	0
0.09377	-1.19145	0.79922	0
0.09377	-1.19145	0.79922	0
0.09377	-1.19145	0.79922	0
0.09377	-1.19145	0.79922	0
0.09377	-1.19145	0.79922	0
0.09377	-1.19145	0.79922	0
0.09377	-1.19145	0.79922	0
0.09377	-1.19145	0.79922	0
0.09377	-1.19145	0.79922	0
0.09377	-1.19145	0.79922	0
0.09377	-1.19145	0.79922	0
0.09377	-1.19145	0.79922	0
0.09377	-1.19145	0.79922	0
0.09377	-1.19145	0.79922	0
0.09377	-1.19145	0.79922	0
0.09377	-1.19145	0.79922	0
0.09377	-1.19145	0.79922	0
0.09377	-1.19145	0.79922	0
0.09377	-1.19145	0.79922	0
0.09377	-1.19145	0.79922	0
0.09377	-1.19145	0.79922	0
0.09377	-1.19145	0.79922	0
0.09377	-1.19145	0.79922	0
0.09377	-1.19145	0.79922	0
0.09377	-1.19145	0.79922	0
0.09377	-1.19145	0.79922	0
0.09377	-1.19145	0.79922	0
0.09377	-1.19145	0.79922	0

0.09377	-1.19145	0.79922	0
0.09377	-1.19145	0.79922	0
0.09377	-1.19145	0.79922	0
0.09377	-1.19145	0.79922	0
0.09377	-1.19145	0.79922	0
0.09377	-1.19145	0.79922	0
0.09377	-1.19145	0.79922	0
0.09377	-1.19145	0.79922	0
0.09377	-1.19145	0.79922	0
0.09377	-1.19145	0.79922	0
0.09377	-1.19145	0.79922	0
0.09377	-1.19145	0.79922	0
0.09377	-1.19145	0.79922	0
0.09377	-1.19145	0.79922	0
0.09377	-1.19145	0.79922	0
0.09377	-1.19145	0.79922	0
0.09377	-1.19145	0.79922	0
0.09377	-1.19145	0.79922	0
0.09377	-1.19145	0.79922	0
0.09377	-1.19145	0.79922	0
0.09377	-1.19145	0.79922	0
0.09377	-1.19145	0.79922	0
0.09377	-1.19145	0.79922	0
0.09377	-1.19145	0.79922	0
0.09377	-1.19145	0.79922	0
0.09377	-1.19145	0.79922	0
0.09377	-1.19145	0.79922	0
0.09377	-1.19145	0.79922	0
0.09377	-1.19145	0.79922	0
0.09377	-1.19145	0.79922	0
0.09377	-1.19145	0.79922	0
0.09377	-1.19145	0.79922	0
0.09377	-1.19145	0.79922	0
0.09377	-1.19145	0.79922	0
0.09377	-1.19145	0.79922	0
0.09377	-1.19145	0.79922	0
0.09377	-1.19145	0.79922	0
0.09377	-1.19145	0.79922	0
0.09377	-1.19145	0.79922	0
0.09377	-1.19145	0.79922	0
0.09377	-1.19145	0.79922	0
0.09377	-1.19145	0.79922	0
0.09377	-1.19145	0.79922	0
0.09377	-1.19145	0.79922	0
0.09377	-1.19145	0.79922	0
0.09377	-1.19145	0.79922	0
0.09377	-1.19145	0.79922	0
0.09377	-1.19145	0.79922	0
0.09377	-1.19145	0.79922	0
0.09377	-1.19145	0.79922	0
0.09377	-1.19145	0.79922	0
0.09377	-1.19145	0.79922	0
0.09377	-1.19145	0.79922	0
0.09377	-1.19145	0.79922	0
0.09377	-1.19145	0.79922	0
0.09377	-1.19145	0.79922	0

0.09377	-1.19145	0.79922	0
0.45652	-1.22088	0.97886	1
0.45652	-1.22088	0.97886	1
0.45652	-1.22088	0.97886	1
0.45652	-1.22088	0.97886	1
0.45652	-1.22088	0.97886	1
0.45652	-1.22088	0.97886	1
0.45652	-1.22088	0.97886	1
0.45652	-1.22088	0.97886	1
0.45652	-1.22088	0.97886	1
0.45652	-1.22088	0.97886	1
0.45652	-1.22088	0.97886	1
0.45652	-1.22088	0.97886	1
0.45652	-1.22088	0.97886	1
0.45652	-1.22088	0.97886	1
0.45652	-1.22088	0.97886	1
0.45652	-1.22088	0.97886	1
0.45652	-1.22088	0.97886	1
0.45652	-1.22088	0.97886	1
0.45652	-1.22088	0.97886	1
0.45652	-1.22088	0.97886	1
0.45652	-1.22088	0.97886	1
0.45652	-1.22088	0.97886	1
0.45652	-1.22088	0.97886	1
0.45652	-1.22088	0.97886	1
0.45652	-1.22088	0.97886	1
0.45652	-1.22088	0.97886	1
0.45652	-1.22088	0.97886	1
0.45652	-1.22088	0.97886	1
0.45652	-1.22088	0.97886	1
0.45652	-1.22088	0.97886	1
0.45652	-1.22088	0.97886	1
0.45652	-1.22088	0.97886	1
0.45652	-1.22088	0.97886	1
0.45652	-1.22088	0.97886	1
0.45652	-1.22088	0.97886	1
0.45652	-1.22088	0.97886	1
0.45652	-1.22088	0.97886	1
0.45652	-1.22088	0.97886	1
0.45652	-1.22088	0.97886	1
0.45652	-1.22088	0.97886	1
0.45652	-1.22088	0.97886	1
0.45652	-1.22088	0.97886	1
0.45652	-1.22088	0.97886	1
0.45652	-1.22088	0.97886	1
0.45652	-1.22088	0.97886	1
0.45652	-1.22088	0.97886	1
0.45652	-1.22088	0.97886	1
0.45652	-1.22088	0.97886	1
0.45652	-1.22088	0.97886	1
0.45652	-1.22088	0.97886	1
0.45652	-1.22088	0.97886	1
0.45652	-1.22088	0.97886	1
0.45652	-1.22088	0.97886	1
0.45652	-1.22088	0.97886	1
0.45652	-1.22088	0.97886	1

0.45652	-1.22088	0.97886	1
0.45652	-1.22088	0.97886	1
0.45652	-1.22088	0.97886	1
0.45652	-1.22088	0.97886	1
0.45652	-1.22088	0.97886	1
0.45652	-1.22088	0.97886	1
0.45652	-1.22088	0.97886	1
0.45652	-1.22088	0.97886	1
0.45652	-1.22088	0.97886	1
0.45652	-1.22088	0.97886	1
0.45652	-1.22088	0.97886	1
0.45652	-1.22088	0.97886	1
0.45652	-1.22088	0.97886	1
0.45652	-1.22088	0.97886	1
0.45652	-1.22088	0.97886	1
0.45652	-1.22088	0.97886	1
0.45652	-1.22088	0.97886	1
0.45652	-1.22088	0.97886	1
0.45652	-1.22088	0.97886	1
0.45652	-1.22088	0.97886	1
0.45652	-1.22088	0.97886	1
0.45652	-1.22088	0.97886	1
0.45652	-1.22088	0.97886	1
0.45652	-1.22088	0.97886	1
0.45652	-1.22088	0.97886	1
0.45652	-1.22088	0.97886	1
0.45652	-1.22088	0.97886	1
0.45652	-1.22088	0.97886	1
0.45652	-1.22088	0.97886	1
0.45652	-1.22088	0.97886	1
0.45652	-1.22088	0.97886	1
0.45652	-1.22088	0.97886	1
0.45652	-1.22088	0.97886	1
0.45652	-1.22088	0.97886	1
0.45652	-1.22088	0.97886	1
0.45652	-1.22088	0.97886	1
0.45652	-1.22088	0.97886	1
0.45652	-1.22088	0.97886	1
0.45652	-1.22088	0.97886	1
0.45652	-1.22088	0.97886	1
0.45652	-1.22088	0.97886	1
0.45652	-1.22088	0.97886	1
0.45652	-1.22088	0.97886	1
0.45652	-1.22088	0.97886	1
0.45652	-1.22088	0.97886	1
0.45652	-1.22088	0.97886	1
0.45652	-1.22088	0.97886	1
0.45652	-1.22088	0.97886	1
0.45652	-1.22088	0.97886	1
0.45652	-1.22088	0.97886	1
0.45652	-1.22088	0.97886	1
0.45652	-1.22088	0.97886	1
0.45652	-1.22088	0.97886	1
0.45652	-1.22088	0.97886	1
0.45652	-1.22088	0.97886	1
0.45652	-1.22088	0.97886	1

0.45652	-1.22088	0.97886	1
0.45652	-1.22088	0.97886	1
0.45652	-1.22088	0.97886	1
0.45652	-1.22088	0.97886	1
0.45652	-1.22088	0.97886	1
0.45652	-1.22088	0.97886	1
0.45652	-1.22088	0.97886	1
0.45652	-1.22088	0.97886	1
0.45652	-1.22088	0.97886	1
0.45652	-1.22088	0.97886	1
0.45652	-1.22088	0.97886	1
0.45652	-1.22088	0.97886	1
0.45652	-1.22088	0.97886	1
0.45652	-1.22088	0.97886	1
0.45652	-1.22088	0.97886	1
0.45652	-1.22088	0.97886	1
0.45652	-1.22088	0.97886	1
0.77859	-1.35208	0.99459	1
0.77859	-1.35208	0.99459	1
0.77859	-1.35208	0.99459	1
0.77859	-1.35208	0.99459	1
0.77859	-1.35208	0.99459	1
0.77859	-1.35208	0.99459	1
0.77859	-1.35208	0.99459	1
0.77859	-1.35208	0.99459	1
0.77859	-1.35208	0.99459	1
0.77859	-1.35208	0.99459	1
0.77859	-1.35208	0.99459	1
0.77859	-1.35208	0.99459	1
0.77859	-1.35208	0.99459	1
0.77859	-1.35208	0.99459	1
0.77859	-1.35208	0.99459	1
0.77859	-1.35208	0.99459	1
0.77859	-1.35208	0.99459	1
0.77859	-1.35208	0.99459	1
0.77859	-1.35208	0.99459	1
0.77859	-1.35208	0.99459	1
0.77859	-1.35208	0.99459	1
0.77859	-1.35208	0.99459	1
0.77859	-1.35208	0.99459	1
0.77859	-1.35208	0.99459	1
0.77859	-1.35208	0.99459	1
0.77859	-1.35208	0.99459	1
0.77859	-1.35208	0.99459	1
0.77859	-1.35208	0.99459	1
0.77859	-1.35208	0.99459	1
0.77859	-1.35208	0.99459	1
0.77859	-1.35208	0.99459	1
0.77859	-1.35208	0.99459	1
0.77859	-1.35208	0.99459	1
0.77859	-1.35208	0.99459	1
0.77859	-1.35208	0.99459	1
0.77859	-1.35208	0.99459	1
0.77859	-1.35208	0.99459	1
0.77859	-1.35208	0.99459	1
0.77859	-1.35208	0.99459	1

0.77859	-1.35208	0.99459	1
0.77859	-1.35208	0.99459	1
0.77859	-1.35208	0.99459	1
0.77859	-1.35208	0.99459	1
0.77859	-1.35208	0.99459	1
0.77859	-1.35208	0.99459	1
0.77859	-1.35208	0.99459	1
0.77859	-1.35208	0.99459	1
0.77859	-1.35208	0.99459	1
0.77859	-1.35208	0.99459	1
0.77859	-1.35208	0.99459	1
0.77859	-1.35208	0.99459	1
0.77859	-1.35208	0.99459	1
0.77859	-1.35208	0.99459	1
0.77859	-1.35208	0.99459	1
0.77859	-1.35208	0.99459	1
0.77859	-1.35208	0.99459	1
0.77859	-1.35208	0.99459	1
0.77859	-1.35208	0.99459	1
0.77859	-1.35208	0.99459	1
0.77859	-1.35208	0.99459	1
0.77859	-1.35208	0.99459	1
0.77859	-1.35208	0.99459	1
0.77859	-1.35208	0.99459	1
0.77859	-1.35208	0.99459	1
0.77859	-1.35208	0.99459	1
0.77859	-1.35208	0.99459	1
0.77859	-1.35208	0.99459	1
0.77859	-1.35208	0.99459	1
0.77859	-1.35208	0.99459	1
0.77859	-1.35208	0.99459	1
0.77859	-1.35208	0.99459	1
0.77859	-1.35208	0.99459	1
0.77859	-1.35208	0.99459	1
0.77859	-1.35208	0.99459	1
0.77859	-1.35208	0.99459	1
0.77859	-1.35208	0.99459	1
0.77859	-1.35208	0.99459	1
0.77859	-1.35208	0.99459	1
0.77859	-1.35208	0.99459	1
0.77859	-1.35208	0.99459	1
0.77859	-1.35208	0.99459	1
0.77859	-1.35208	0.99459	1
0.77859	-1.35208	0.99459	1
0.77859	-1.35208	0.99459	1
0.77859	-1.35208	0.99459	1
0.77859	-1.35208	0.99459	1
0.77859	-1.35208	0.99459	1
0.77859	-1.35208	0.99459	1
0.77859	-1.35208	0.99459	1
0.77859	-1.35208	0.99459	1
0.77859	-1.35208	0.99459	1
0.77859	-1.35208	0.99459	1
0.77859	-1.35208	0.99459	1
0.77859	-1.35208	0.99459	1
0.77859	-1.35208	0.99459	1

0.77859	-1.35208	0.99459	1
0.77859	-1.35208	0.99459	1
0.77859	-1.35208	0.99459	1
0.77859	-1.35208	0.99459	1
0.77859	-1.35208	0.99459	1
0.77859	-1.35208	0.99459	1
0.77859	-1.35208	0.99459	1
0.77859	-1.35208	0.99459	1
0.77859	-1.35208	0.99459	1
0.77859	-1.35208	0.99459	1
0.77859	-1.35208	0.99459	1
0.77859	-1.35208	0.99459	1
0.77859	-1.35208	0.99459	1
0.77859	-1.35208	0.99459	1
0.77859	-1.35208	0.99459	1
0.77859	-1.35208	0.99459	1
0.77859	-1.35208	0.99459	1
0.77859	-1.35208	0.99459	1
0.77859	-1.35208	0.99459	1
0.77859	-1.35208	0.99459	1
0.77859	-1.35208	0.99459	1
0.77859	-1.35208	0.99459	1
0.77859	-1.35208	0.99459	1
0.77859	-1.35208	0.99459	1
0.77859	-1.35208	0.99459	1
0.77859	-1.35208	0.99459	1
0.77859	-1.35208	0.99459	1
0.77859	-1.35208	0.99459	1
0.77859	-1.35208	0.99459	1
0.77859	-1.35208	0.99459	1
0.77859	-1.35208	0.99459	1
0.77859	-1.35208	0.99459	1
0.77859	-1.35208	0.99459	1
0.77859	-1.35208	0.99459	1
0.77859	-1.35208	0.99459	1
0.77859	-1.35208	0.99459	1
0.77859	-1.35208	0.99459	1
0.77859	-1.35208	0.99459	1
0.77859	-1.35208	0.99459	1
0.77859	-1.35208	0.99459	1
0.77859	-1.35208	0.99459	1
0.77859	-1.35208	0.99459	1
0.77859	-1.35208	0.99459	1
0.77859	-1.35208	0.99459	1
0.77859	-1.35208	0.99459	1
0.77859	-1.35208	0.99459	1
0.77859	-1.35208	0.99459	1
0.77859	-1.35208	0.99459	1
0.77859	-1.35208	0.99459	1
0.77859	-1.35208	0.99459	1
0.77859	-1.35208	0.99459	1
0.77859	-1.35208	0.99459	1
0.77859	-1.35208	0.99459	1
0.77859	-1.35208	0.99459	1
0.77859	-1.35208	0.99459	1
0.77859	-1.35208	0.99459	1

0.77859	-1.35208	0.99459	1
0.77859	-1.35208	0.99459	1
0.77859	-1.35208	0.99459	1
0.77859	-1.35208	0.99459	1
0.77859	-1.35208	0.99459	1
0.77859	-1.35208	0.99459	1
0.77859	-1.35208	0.99459	1
0.77859	-1.35208	0.99459	1
0.77859	-1.35208	0.99459	1
0.77859	-1.35208	0.99459	1
0.77859	-1.35208	0.99459	1
0.77859	-1.35208	0.99459	1
0.77859	-1.35208	0.99459	1
0.77859	-1.35208	0.99459	1
0.77859	-1.35208	0.99459	1
0.77859	-1.35208	0.99459	1
0.77859	-1.35208	0.99459	1
0.77859	-1.35208	0.99459	1
0.77859	-1.35208	0.99459	1
0.77859	-1.35208	0.99459	1
0.77859	-1.35208	0.99459	1
0.77859	-1.35208	0.99459	1
0.77859	-1.35208	0.99459	1
0.77859	-1.35208	0.99459	1
0.77859	-1.35208	0.99459	1
0.77859	-1.35208	0.99459	1
0.77859	-1.35208	0.99459	1
0.45652	-1.07838	0.97886	1
0.45652	-1.07838	0.97886	1
0.45652	-1.07838	0.97886	1
0.45652	-1.07838	0.97886	1
0.45652	-1.07838	0.97886	1
0.45652	-1.07838	0.97886	1
0.45652	-1.07838	0.97886	1
0.45652	-1.07838	0.97886	1
0.45652	-1.07838	0.97886	1
0.45652	-1.07838	0.97886	1
0.45652	-1.07838	0.97886	1
0.45652	-1.07838	0.97886	1
0.45652	-1.07838	0.97886	1
0.45652	-1.07838	0.97886	1
0.45652	-1.07838	0.97886	1
0.45652	-1.07838	0.97886	1
0.45652	-1.07838	0.97886	1
0.45652	-1.07838	0.97886	1
0.45652	-1.07838	0.97886	1
0.45652	-1.07838	0.97886	1
0.45652	-1.07838	0.97886	1
0.45652	-1.07838	0.97886	1
0.45652	-1.07838	0.97886	1
0.45652	-1.07838	0.97886	1
0.45652	-1.07838	0.97886	1
0.45652	-1.07838	0.97886	1
0.45652	-1.07838	0.97886	1
0.45652	-1.07838	0.97886	1
0.45652	-1.07838	0.97886	1

0.45652	-1.07838	0.97886	1
0.45652	-1.07838	0.97886	1
0.45652	-1.07838	0.97886	1
0.45652	-1.07838	0.97886	1
0.45652	-1.07838	0.97886	1
0.45652	-1.07838	0.97886	1
0.45652	-1.07838	0.97886	1
0.45652	-1.07838	0.97886	1
0.45652	-1.07838	0.97886	1
0.45652	-1.07838	0.97886	1
0.45652	-1.07838	0.97886	1
0.45652	-1.07838	0.97886	1
0.45652	-1.07838	0.97886	1
0.45652	-1.07838	0.97886	1
0.45652	-1.07838	0.97886	1
0.45652	-1.07838	0.97886	1
0.45652	-1.07838	0.97886	1
0.45652	-1.07838	0.97886	1
0.45652	-1.07838	0.97886	1
0.45652	-1.07838	0.97886	1
0.45652	-1.07838	0.97886	1
0.45652	-1.07838	0.97886	1
0.45652	-1.07838	0.97886	1
0.45652	-1.07838	0.97886	1
0.45652	-1.07838	0.97886	1
0.45652	-1.07838	0.97886	1
0.45652	-1.07838	0.97886	1
0.45652	-1.07838	0.97886	1
0.45652	-1.07838	0.97886	1
0.45652	-1.07838	0.97886	1
0.45652	-1.07838	0.97886	1
0.45652	-1.07838	0.97886	1
0.45652	-1.07838	0.97886	1
0.45652	-1.07838	0.97886	1
0.45652	-1.07838	0.97886	1
0.77859	-1.35208	0.99459	1
0.77859	-1.35208	0.99459	1
0.77859	-1.35208	0.99459	1
0.77859	-1.35208	0.99459	1
0.77859	-1.35208	0.99459	1
0.77859	-1.35208	0.99459	1
0.77859	-1.35208	0.99459	1
0.77859	-1.35208	0.99459	1
0.77859	-1.35208	0.99459	1
0.77859	-1.35208	0.99459	1
0.77859	-1.35208	0.99459	1
0.77859	-1.35208	0.99459	1
0.77859	-1.35208	0.99459	1
0.77859	-1.35208	0.99459	1
0.77859	-1.35208	0.99459	1
0.77859	-1.35208	0.99459	1
0.77859	-1.35208	0.99459	1
0.77859	-1.35208	0.99459	1
0.77859	-1.35208	0.99459	1
0.77859	-1.35208	0.99459	1
0.77859	-1.35208	0.99459	1

0.77859	-1.35208	0.99459	1
0.77859	-1.35208	0.99459	1
0.77859	-1.35208	0.99459	1
0.77859	-1.35208	0.99459	1
0.77859	-1.35208	0.99459	1
0.77859	-1.35208	0.99459	1
0.77859	-1.35208	0.99459	1
0.77859	-1.35208	0.99459	1
0.77859	-1.35208	0.99459	1
0.77859	-1.35208	0.99459	1
0.77859	-1.35208	0.99459	1
0.45652	-1.22088	0.97886	1
0.45652	-1.22088	0.97886	1
0.45652	-1.22088	0.97886	1
0.45652	-1.22088	0.97886	1
0.45652	-1.22088	0.97886	1
0.45652	-1.22088	0.97886	1
0.45652	-1.22088	0.97886	1
0.45652	-1.22088	0.97886	1
0.45652	-1.22088	0.97886	1
0.45652	-1.22088	0.97886	1
0.45652	-1.22088	0.97886	1
0.45652	-1.22088	0.97886	1
0.45652	-1.22088	0.97886	1
0.45652	-1.22088	0.97886	1
0.45652	-1.22088	0.97886	1
0.45652	-1.22088	0.97886	1
0.45652	-1.22088	0.97886	1
0.45652	-1.22088	0.97886	1
0.45652	-1.22088	0.97886	1
0.45652	-1.22088	0.97886	1
0.45652	-1.22088	0.97886	1
0.45652	-1.22088	0.97886	1
0.45652	-1.22088	0.97886	1
0.45652	-1.22088	0.97886	1
0.45652	-1.22088	0.97886	1
0.45652	-1.22088	0.97886	1
0.45652	-1.22088	0.97886	1
0.45652	-1.22088	0.97886	1
0.45652	-1.22088	0.97886	1
0.45652	-1.22088	0.97886	1
0.45652	-1.22088	0.97886	1
0.45652	-1.22088	0.97886	1
0.45652	-1.22088	0.97886	1
0.45652	-1.22088	0.97886	1
0.45652	-1.22088	0.97886	1
0.45652	-1.22088	0.97886	1
0.45652	-1.22088	0.97886	1
0.45652	-1.22088	0.97886	1
0.45652	-1.22088	0.97886	1
0.45652	-1.22088	0.97886	1
0.45652	-1.22088	0.97886	1
0.45652	-1.22088	0.97886	1
0.45652	-1.22088	0.97886	1
0.45652	-1.22088	0.97886	1
0.45652	-1.22088	0.97886	1

0.45652	-1.22088	0.97886	1
0.45652	-1.22088	0.97886	1
0.45652	-1.22088	0.97886	1
0.45652	-1.22088	0.97886	1
0.45652	-1.22088	0.97886	1
0.45652	-1.22088	0.97886	1
0.45652	-1.22088	0.97886	1
0.45652	-1.22088	0.97886	1
0.45652	-1.22088	0.97886	1
0.45652	-1.22088	0.97886	1
0.45652	-1.22088	0.97886	1
0.45652	-1.22088	0.97886	1
0.45652	-1.22088	0.97886	1
0.45652	-1.22088	0.97886	1
0.45652	-1.22088	0.97886	1
0.45652	-1.22088	0.97886	1
0.45652	-1.22088	0.97886	1
0.45652	-1.22088	0.97886	1
0.45652	-1.22088	0.97886	1
0.41597	-1.50040	1.09133	1
0.41597	-1.50040	1.09133	1
0.41597	-1.50040	1.09133	1
0.41597	-1.50040	1.09133	1
0.41597	-1.50040	1.09133	1
0.41597	-1.50040	1.09133	1
0.41597	-1.50040	1.09133	1
0.41597	-1.50040	1.09133	1
0.41597	-1.50040	1.09133	1
0.41597	-1.50040	1.09133	1
0.41597	-1.50040	1.09133	1
0.41597	-1.50040	1.09133	1
0.41597	-1.50040	1.09133	1
0.41597	-1.50040	1.09133	1
0.41597	-1.50040	1.09133	1
0.41597	-1.50040	1.09133	1
0.41597	-1.50040	1.09133	1
0.41597	-1.50040	1.09133	1
0.41597	-1.50040	1.09133	1
0.41597	-1.50040	1.09133	1
0.41597	-1.50040	1.09133	1
0.41597	-1.50040	1.09133	1
0.41597	-1.50040	1.09133	1
0.41597	-1.50040	1.09133	1
0.41597	-1.50040	1.09133	1
0.41597	-1.50040	1.09133	1
0.41597	-1.50040	1.09133	1
0.41597	-1.50040	1.09133	1
0.41597	-1.50040	1.09133	1
0.41597	-1.50040	1.09133	1
0.41597	-1.50040	1.09133	1
0.41597	-1.50040	1.09133	1
0.41597	-1.50040	1.09133	1
0.41597	-1.50040	1.09133	1
0.41597	-1.50040	1.09133	1
0.41597	-1.50040	1.09133	1
0.41597	-1.50040	1.09133	1

0.41597	-1.50040	1.09133	1
0.41597	-1.50040	1.09133	1
0.41597	-1.50040	1.09133	1
0.41597	-1.50040	1.09133	1
0.41597	-1.50040	1.09133	1
0.41597	-1.50040	1.09133	1
0.41597	-1.50040	1.09133	1
0.41597	-1.50040	1.09133	1
0.41597	-1.50040	1.09133	1
0.41597	-1.50040	1.09133	1
0.41597	-1.50040	1.09133	1
0.41597	-1.50040	1.09133	1
0.41597	-1.50040	1.09133	1
0.41597	-1.50040	1.09133	1
0.41597	-1.50040	1.09133	1
0.41597	-1.50040	1.09133	1
0.41597	-1.50040	1.09133	1
0.45652	-1.22088	0.97886	1
0.45652	-1.22088	0.97886	1
0.45652	-1.22088	0.97886	1
0.45652	-1.22088	0.97886	1
0.45652	-1.22088	0.97886	1
0.45652	-1.22088	0.97886	1
0.45652	-1.22088	0.97886	1
0.45652	-1.22088	0.97886	1
0.45652	-1.22088	0.97886	1
0.45652	-1.22088	0.97886	1
0.45652	-1.22088	0.97886	1
0.45652	-1.22088	0.97886	1
0.45652	-1.22088	0.97886	1
0.45652	-1.22088	0.97886	1
0.45652	-1.22088	0.97886	1
0.45652	-1.22088	0.97886	1
0.45652	-1.22088	0.97886	1
0.45652	-1.22088	0.97886	1
0.45652	-1.22088	0.97886	1
0.45652	-1.22088	0.97886	1
0.45652	-1.22088	0.97886	1
0.45652	-1.22088	0.97886	1
0.45652	-1.22088	0.97886	1
0.45652	-1.22088	0.97886	1
0.45652	-1.22088	0.97886	1
0.45652	-1.22088	0.97886	1
0.45652	-1.22088	0.97886	1
0.45652	-1.22088	0.97886	1
0.45652	-1.22088	0.97886	1
0.45652	-1.22088	0.97886	1
0.45652	-1.22088	0.97886	1
0.45652	-1.22088	0.97886	1
0.45652	-1.22088	0.97886	1
0.45652	-1.22088	0.97886	1
0.45652	-1.22088	0.97886	1
0.45652	-1.22088	0.97886	1
0.45652	-1.22088	0.97886	1
0.45652	-1.22088	0.97886	1
0.45652	-1.22088	0.97886	1

0.45652	-1.22088	0.97886	1
0.45652	-1.22088	0.97886	1
0.45652	-1.22088	0.97886	1
0.45652	-1.22088	0.97886	1
0.45652	-1.22088	0.97886	1
0.45652	-1.22088	0.97886	1
0.45652	-1.22088	0.97886	1
0.45652	-1.22088	0.97886	1
0.45652	-1.22088	0.97886	1
0.45652	-1.22088	0.97886	1
0.45652	-1.22088	0.97886	1
0.45652	-1.22088	0.97886	1
0.45652	-1.22088	0.97886	1
0.45652	-1.22088	0.97886	1
0.45652	-1.22088	0.97886	1
0.45652	-1.22088	0.97886	1
0.45652	-1.22088	0.97886	1
0.45652	-1.22088	0.97886	1
0.45652	-1.22088	0.97886	1
0.45652	-1.22088	0.97886	1
0.45652	-1.22088	0.97886	1
0.45652	-1.22088	0.97886	1
0.45652	-1.22088	0.97886	1
0.45652	-1.22088	0.97886	1
0.45652	-1.22088	0.97886	1
0.41597	-1.50040	1.09133	1
0.41597	-1.50040	1.09133	1
0.41597	-1.50040	1.09133	1
0.41597	-1.50040	1.09133	1
0.41597	-1.50040	1.09133	1
0.41597	-1.50040	1.09133	1
0.41597	-1.50040	1.09133	1
0.41597	-1.50040	1.09133	1
0.41597	-1.50040	1.09133	1
0.41597	-1.50040	1.09133	1
0.41597	-1.50040	1.09133	1
0.41597	-1.50040	1.09133	1
0.41597	-1.50040	1.09133	1
0.41597	-1.50040	1.09133	1
0.41597	-1.50040	1.09133	1
0.41597	-1.50040	1.09133	1
0.41597	-1.50040	1.09133	1
0.41597	-1.50040	1.09133	1
0.41597	-1.50040	1.09133	1
0.41597	-1.50040	1.09133	1
0.41597	-1.50040	1.09133	1
0.41597	-1.50040	1.09133	1
0.41597	-1.50040	1.09133	1
0.41597	-1.50040	1.09133	1
0.41597	-1.50040	1.09133	1
0.41597	-1.50040	1.09133	1
0.41597	-1.50040	1.09133	1
0.41597	-1.50040	1.09133	1
0.41597	-1.50040	1.09133	1
0.41597	-1.50040	1.09133	1
0.41597	-1.50040	1.09133	1

0.41597	-1.50040	1.09133	1
0.41597	-1.50040	1.09133	1
0.41597	-1.50040	1.09133	1
0.41597	-1.50040	1.09133	1
0.41597	-1.50040	1.09133	1
0.41597	-1.50040	1.09133	1
0.41597	-1.50040	1.09133	1
0.41597	-1.50040	1.09133	1
0.41597	-1.50040	1.09133	1
0.41597	-1.50040	1.09133	1
0.41597	-1.50040	1.09133	1
0.41597	-1.50040	1.09133	1
0.41597	-1.50040	1.09133	1
0.41597	-1.50040	1.09133	1
0.41597	-1.50040	1.09133	1
0.41597	-1.50040	1.09133	1
0.41597	-1.50040	1.09133	1
0.41597	-1.50040	1.09133	1
0.41597	-1.50040	1.09133	1
0.41597	-1.50040	1.09133	1
0.41597	-1.50040	1.09133	1
0.41597	-1.50040	1.09133	1
0.41597	-1.50040	1.09133	1
0.41597	-1.50040	1.09133	1
0.41597	-1.50040	1.09133	1
0.41597	-1.50040	1.09133	1
0.41597	-1.50040	1.09133	1
0.41597	-1.50040	1.09133	1
0.41597	-1.50040	1.09133	1
0.41597	-1.50040	1.09133	1
0.41597	-1.50040	1.09133	1
0.41597	-1.50040	1.09133	1
0.41597	-1.50040	1.09133	1
0.41597	-1.50040	1.09133	1
0.41597	-1.50040	1.09133	1
0.41597	-1.50040	1.09133	1
0.41597	-1.50040	1.09133	1
0.41597	-1.50040	1.09133	1
0.41597	-1.50040	1.09133	1
0.41597	-1.50040	1.09133	1
0.41597	-1.50040	1.09133	1
0.41597	-1.50040	1.09133	1
0.41597	-1.50040	1.09133	1
0.41597	-1.50040	1.09133	1
0.41597	-1.50040	1.09133	1
0.41597	-1.50040	1.09133	1
0.41597	-1.50040	1.09133	1
0.41597	-1.50040	1.09133	1
0.41597	-1.50040	1.09133	1
0.41597	-1.50040	1.09133	1
0.41597	-1.50040	1.09133	1
0.41597	-1.50040	1.09133	1
0.41597	-1.50040	1.09133	1
0.41597	-1.50040	1.09133	1
0.41597	-1.50040	1.09133	1
0.41597	-1.50040	1.09133	1

0.41597	-1.50040	1.09133	1
0.41597	-1.50040	1.09133	1
0.41597	-1.50040	1.09133	1
0.41597	-1.50040	1.09133	1
0.41597	-1.50040	1.09133	1
0.41597	-1.50040	1.09133	1
0.41597	-1.50040	1.09133	1
0.41597	-1.50040	1.09133	1
0.41597	-1.50040	1.09133	1
0.41597	-1.50040	1.09133	1
0.41597	-1.50040	1.09133	1
0.41597	-1.50040	1.09133	1
0.41597	-1.50040	1.09133	1
0.41597	-1.50040	1.09133	1
0.41597	-1.50040	1.09133	1
0.41597	-1.50040	1.09133	1
0.41597	-1.50040	1.09133	1
0.41597	-1.50040	1.09133	1
0.41597	-1.50040	1.09133	1
0.41597	-1.50040	1.09133	1
0.41597	-1.50040	1.09133	1
0.41597	-1.50040	1.09133	1
0.41597	-1.50040	1.09133	1
0.41597	-1.50040	1.09133	1
0.41597	-1.50040	1.09133	1
0.41597	-1.50040	1.09133	1
0.41597	-1.50040	1.09133	1
0.41597	-1.50040	1.09133	1
0.41597	-1.50040	1.09133	1
0.41597	-1.50040	1.09133	1
0.41597	-1.50040	1.09133	1
0.41597	-1.50040	1.09133	1
0.41597	-1.50040	1.09133	1
0.41597	-1.50040	1.09133	1
0.41597	-1.50040	1.09133	1
0.41597	-1.50040	1.09133	1
0.41597	-1.50040	1.09133	1
0.41597	-1.50040	1.09133	1
0.41597	-1.50040	1.09133	1
0.41597	-1.50040	1.09133	1
0.41597	-1.50040	1.09133	1
0.41597	-1.50040	1.09133	1
0.41597	-1.50040	1.09133	1
0.41597	-1.50040	1.09133	1
0.41597	-1.50040	1.09133	1
0.41597	-1.50040	1.09133	1
0.41597	-1.50040	1.09133	1
0.41597	-1.50040	1.09133	1
0.41597	-1.50040	1.09133	1
0.41597	-1.50040	1.09133	1
0.41597	-1.50040	1.09133	1
0.41597	-1.50040	1.09133	1
0.41597	-1.50040	1.09133	1
0.41597	-1.50040	1.09133	1
0.41597	-1.50040	1.09133	1
0.41597	-1.50040	1.09133	1

0.41597	-1.50040	1.09133	1
0.41597	-1.50040	1.09133	1
0.41597	-1.50040	1.09133	1
0.41597	-1.50040	1.09133	1
0.41597	-1.50040	1.09133	1
0.41597	-1.50040	1.09133	1
0.41597	-1.50040	1.09133	1
0.41597	-1.50040	1.09133	1
0.41597	-1.50040	1.09133	1
0.41597	-1.50040	1.09133	1
0.41597	-1.50040	1.09133	1
0.41597	-1.50040	1.09133	1
0.45652	-1.59130	0.97886	1
0.45652	-1.59130	0.97886	1
0.45652	-1.59130	0.97886	1
0.45652	-1.59130	0.97886	1
0.45652	-1.59130	0.97886	1
0.45652	-1.59130	0.97886	1
0.45652	-1.59130	0.97886	1
0.45652	-1.59130	0.97886	1
0.45652	-1.59130	0.97886	1
0.45652	-1.59130	0.97886	1
0.45652	-1.59130	0.97886	1
0.45652	-1.59130	0.97886	1
0.45652	-1.59130	0.97886	1
0.45652	-1.59130	0.97886	1
0.45652	-1.59130	0.97886	1
0.45652	-1.59130	0.97886	1
0.45652	-1.59130	0.97886	1
0.45652	-1.59130	0.97886	1
0.45652	-1.59130	0.97886	1
0.45652	-1.59130	0.97886	1
0.45652	-1.59130	0.97886	1
0.45652	-1.59130	0.97886	1
0.45652	-1.59130	0.97886	1
0.45652	-1.59130	0.97886	1
0.45652	-1.59130	0.97886	1
0.45652	-1.59130	0.97886	1
0.45652	-1.59130	0.97886	1
0.45652	-1.59130	0.97886	1
0.45652	-1.59130	0.97886	1
0.45652	-1.59130	0.97886	1
0.45652	-1.59130	0.97886	1
0.45652	-1.59130	0.97886	1
0.45652	-1.59130	0.97886	1
0.45652	-1.59130	0.97886	1
0.45652	-1.59130	0.97886	1
0.45652	-1.59130	0.97886	1
0.45652	-1.59130	0.97886	1
0.45652	-1.59130	0.97886	1
0.45652	-1.59130	0.97886	1
0.45652	-1.59130	0.97886	1
0.45652	-1.59130	0.97886	1
0.45652	-1.59130	0.97886	1
0.45652	-1.59130	0.97886	1
0.45652	-1.59130	0.97886	1

0.45652	-1.59130	0.97886	1
0.45652	-1.59130	0.97886	1
0.45652	-1.59130	0.97886	1
0.45652	-1.59130	0.97886	1
0.45652	-1.59130	0.97886	1
0.45652	-1.59130	0.97886	1
0.45652	-1.59130	0.97886	1
0.45652	-1.59130	0.97886	1
0.45652	-1.59130	0.97886	1
0.45652	-1.59130	0.97886	1
0.45652	-1.59130	0.97886	1
0.45652	-1.59130	0.97886	1
0.45652	-1.59130	0.97886	1
0.45652	-1.59130	0.97886	1
0.45652	-1.59130	0.97886	1
0.45652	-1.59130	0.97886	1
0.45652	-1.59130	0.97886	1
0.45652	-1.59130	0.97886	1
0.45652	-1.59130	0.97886	1
0.45652	-1.59130	0.97886	1
0.45652	-1.59130	0.97886	1
0.45652	-1.59130	0.97886	1
0.45652	-1.59130	0.97886	1
0.45652	-1.59130	0.97886	1
0.45652	-1.59130	0.97886	1
0.45652	-1.59130	0.97886	1
0.45652	-1.59130	0.97886	1
0.45652	-1.59130	0.97886	1
0.45652	-1.59130	0.97886	1
0.45652	-1.59130	0.97886	1
0.45652	-1.59130	0.97886	1
0.45652	-1.59130	0.97886	1
0.45652	-1.59130	0.97886	1
0.45652	-1.59130	0.97886	1
0.45652	-1.59130	0.97886	1
0.45652	-1.59130	0.97886	1
0.45652	-1.59130	0.97886	1
0.45652	-1.59130	0.97886	1
0.45652	-1.59130	0.97886	1
0.45652	-1.59130	0.97886	1
0.45652	-1.59130	0.97886	1
0.45652	-1.59130	0.97886	1
0.45652	-1.59130	0.97886	1
0.45652	-1.59130	0.97886	1
0.45652	-1.59130	0.97886	1
0.45652	-1.59130	0.97886	1
0.45652	-1.59130	0.97886	1
0.45652	-1.59130	0.97886	1
0.45652	-1.59130	0.97886	1
0.45652	-1.59130	0.97886	1
0.45652	-1.59130	0.97886	1
0.45652	-1.59130	0.97886	1
0.45652	-1.59130	0.97886	1
0.45652	-1.59130	0.97886	1
0.45652	-1.59130	0.97886	1
0.45652	-1.59130	0.97886	1

0.45652	-1.59130	0.97886	1
0.45652	-1.59130	0.97886	1
0.45652	-1.59130	0.97886	1
0.45652	-1.59130	0.97886	1
0.45652	-1.59130	0.97886	1
0.45652	-1.59130	0.97886	1
0.45652	-1.59130	0.97886	1
0.45652	-1.59130	0.97886	1
0.45652	-1.59130	0.97886	1
0.45652	-1.59130	0.97886	1
0.45652	-1.59130	0.97886	1
0.45652	-1.59130	0.97886	1
0.45652	-1.59130	0.97886	1
0.45652	-1.59130	0.97886	1
0.45652	-1.59130	0.97886	1
0.45652	-1.59130	0.97886	1
0.45652	-1.59130	0.97886	1
0.45652	-1.59130	0.97886	1
0.45652	-1.59130	0.97886	1
0.45652	-1.59130	0.97886	1
0.45652	-1.59130	0.97886	1
0.45652	-1.59130	0.97886	1
0.45652	-1.59130	0.97886	1
0.45652	-1.59130	0.97886	1
0.45652	-1.59130	0.97886	1
0.45652	-1.59130	0.97886	1
0.45652	-1.59130	0.97886	1
0.45652	-1.59130	0.97886	1
0.45652	-1.22088	0.97886	1
0.45652	-1.22088	0.97886	1
0.45652	-1.22088	0.97886	1
0.45652	-1.22088	0.97886	1
0.45652	-1.22088	0.97886	1
0.45652	-1.22088	0.97886	1
0.45652	-1.22088	0.97886	1
0.45652	-1.22088	0.97886	1
0.45652	-1.22088	0.97886	1
0.45652	-1.22088	0.97886	1
0.45652	-1.22088	0.97886	1
0.45652	-1.22088	0.97886	1
0.45652	-1.22088	0.97886	1
0.45652	-1.22088	0.97886	1
0.45652	-1.22088	0.97886	1
0.45652	-1.22088	0.97886	1
0.45652	-1.22088	0.97886	1
0.45652	-1.22088	0.97886	1
0.45652	-1.22088	0.97886	1
0.45652	-1.22088	0.97886	1
0.45652	-1.22088	0.97886	1
0.45652	-1.22088	0.97886	1
0.45652	-1.22088	0.97886	1
0.45652	-1.22088	0.97886	1
0.45652	-1.22088	0.97886	1
0.45652	-1.22088	0.97886	1
0.45652	-1.22088	0.97886	1
0.45652	-1.22088	0.97886	1

0.45652	-1.22088	0.97886	1
0.45652	-1.22088	0.97886	1
0.45652	-1.22088	0.97886	1
0.45652	-1.22088	0.97886	1
0.45652	-1.22088	0.97886	1
0.45652	-1.22088	0.97886	1
0.45652	-1.22088	0.97886	1
0.45652	-1.22088	0.97886	1
0.45652	-1.22088	0.97886	1
0.45652	-1.22088	0.97886	1
0.45652	-1.22088	0.97886	1
0.45652	-1.22088	0.97886	1
0.45652	-1.22088	0.97886	1
0.45652	-1.22088	0.97886	1
0.45652	-1.22088	0.97886	1
0.45652	-1.22088	0.97886	1
0.45652	-1.22088	0.97886	1
0.45652	-1.22088	0.97886	1
0.45652	-1.22088	0.97886	1
0.45652	-1.22088	0.97886	1
0.45652	-1.22088	0.97886	1
0.45652	-1.22088	0.97886	1
0.45652	-1.22088	0.97886	1
0.45652	-1.22088	0.97886	1
0.45652	-1.22088	0.97886	1
0.45652	-1.22088	0.97886	1
0.45652	-1.22088	0.97886	1
0.45652	-1.22088	0.97886	1
0.45652	-1.22088	0.97886	1
0.45652	-1.22088	0.97886	1
0.45652	-1.22088	0.97886	1
0.45652	-1.22088	0.97886	1
0.45652	-1.22088	0.97886	1
0.45652	-1.22088	0.97886	1
0.45652	-1.22088	0.97886	1
0.45652	-1.22088	0.97886	1
0.45652	-1.07838	0.97886	1
0.45652	-1.07838	0.97886	1
0.45652	-1.07838	0.97886	1
0.45652	-1.07838	0.97886	1
0.45652	-1.07838	0.97886	1
0.45652	-1.07838	0.97886	1
0.45652	-1.07838	0.97886	1
0.45652	-1.07838	0.97886	1
0.45652	-1.07838	0.97886	1
0.45652	-1.07838	0.97886	1
0.45652	-1.07838	0.97886	1
0.45652	-1.07838	0.97886	1
0.45652	-1.07838	0.97886	1
0.45652	-1.07838	0.97886	1
0.45652	-1.07838	0.97886	1
0.45652	-1.07838	0.97886	1
0.45652	-1.07838	0.97886	1
0.45652	-1.07838	0.97886	1
0.45652	-1.07838	0.97886	1
0.45652	-1.07838	0.97886	1

0.45652	-1.07838	0.97886	1
0.45652	-1.07838	0.97886	1
0.45652	-1.07838	0.97886	1
0.45652	-1.07838	0.97886	1
0.45652	-1.07838	0.97886	1
0.45652	-1.07838	0.97886	1
0.45652	-1.07838	0.97886	1
0.45652	-1.07838	0.97886	1
0.45652	-1.07838	0.97886	1
0.45652	-1.07838	0.97886	1
0.45652	-1.07838	0.97886	1
0.45652	-1.07838	0.97886	1
0.45652	-1.07838	0.97886	1
0.45652	-1.07838	0.97886	1
0.45652	-1.07838	0.97886	1
0.45652	-1.07838	0.97886	1
0.45652	-1.07838	0.97886	1
0.45652	-1.07838	0.97886	1
0.45652	-1.07838	0.97886	1
0.45652	-1.07838	0.97886	1
0.45652	-1.07838	0.97886	1
0.45652	-1.07838	0.97886	1
0.45652	-1.07838	0.97886	1
0.45652	-1.07838	0.97886	1
0.45652	-1.07838	0.97886	1
0.45652	-1.07838	0.97886	1
0.45652	-1.07838	0.97886	1
0.45652	-1.07838	0.97886	1
0.45652	-1.22088	0.97886	1
0.45652	-1.22088	0.97886	1
0.45652	-1.22088	0.97886	1
0.45652	-1.22088	0.97886	1
0.45652	-1.22088	0.97886	1
0.45652	-1.22088	0.97886	1
0.45652	-1.22088	0.97886	1
0.45652	-1.22088	0.97886	1
0.45652	-1.22088	0.97886	1
0.45652	-1.22088	0.97886	1
0.45652	-1.22088	0.97886	1
0.45652	-1.22088	0.97886	1
0.45652	-1.22088	0.97886	1
0.45652	-1.22088	0.97886	1
0.45652	-1.22088	0.97886	1
0.45652	-1.22088	0.97886	1
0.45652	-1.22088	0.97886	1
0.45652	-1.22088	0.97886	1
0.45652	-1.22088	0.97886	1
0.45652	-1.22088	0.97886	1
0.45652	-1.22088	0.97886	1
0.45652	-1.22088	0.97886	1
0.45652	-1.22088	0.97886	1
0.45652	-1.22088	0.97886	1
0.45652	-1.22088	0.97886	1
0.45652	-1.22088	0.97886	1
0.45652	-1.22088	0.97886	1
0.45652	-1.22088	0.97886	1

0.45652	-1.22088	0.97886	1
0.45652	-1.22088	0.97886	1
0.45652	-1.22088	0.97886	1
0.45652	-1.22088	0.97886	1
0.45652	-1.22088	0.97886	1
0.45652	-1.22088	0.97886	1
0.45652	-1.22088	0.97886	1
0.45652	-1.22088	0.97886	1
0.45652	-1.22088	0.97886	1
0.45652	-1.22088	0.97886	1
0.45652	-1.22088	0.97886	1
0.45652	-1.22088	0.97886	1
0.45652	-1.22088	0.97886	1
0.45652	-1.22088	0.97886	1
0.45652	-1.22088	0.97886	1
0.45652	-1.22088	0.97886	1
0.45652	-1.22088	0.97886	1
0.45652	-1.22088	0.97886	1
0.45652	-1.22088	0.97886	1
0.45652	-1.22088	0.97886	1
0.45652	-1.22088	0.97886	1
0.45652	-1.22088	0.97886	1
0.45652	-1.22088	0.97886	1
0.45652	-1.22088	0.97886	1
0.45652	-1.22088	0.97886	1
0.45652	-1.22088	0.97886	1
0.45652	-1.22088	0.97886	1
0.45652	-1.22088	0.97886	1
0.45652	-1.22088	0.97886	1
0.45652	-1.22088	0.97886	1
0.45652	-1.22088	0.97886	1
0.45652	-1.22088	0.97886	1
0.45652	-1.22088	0.97886	1
0.45652	-1.22088	0.97886	1
0.45652	-1.22088	0.97886	1
0.45652	-1.22088	0.97886	1
0.45652	-1.07838	0.97886	1
0.45652	-1.07838	0.97886	1
0.45652	-1.07838	0.97886	1
0.45652	-1.07838	0.97886	1
0.45652	-1.07838	0.97886	1
0.45652	-1.07838	0.97886	1
0.45652	-1.07838	0.97886	1
0.45652	-1.07838	0.97886	1
0.45652	-1.07838	0.97886	1
0.45652	-1.07838	0.97886	1
0.45652	-1.07838	0.97886	1
0.45652	-1.07838	0.97886	1
0.45652	-1.07838	0.97886	1
0.45652	-1.07838	0.97886	1
0.45652	-1.07838	0.97886	1
0.45652	-1.07838	0.97886	1
0.45652	-1.07838	0.97886	1
0.45652	-1.07838	0.97886	1
0.45652	-1.07838	0.97886	1
0.45652	-1.07838	0.97886	1

0.45652	-1.07838	0.97886	1
0.45652	-1.07838	0.97886	1
0.45652	-1.07838	0.97886	1
0.45652	-1.07838	0.97886	1
0.45652	-1.07838	0.97886	1
0.45652	-1.07838	0.97886	1
0.45652	-1.07838	0.97886	1
0.45652	-1.07838	0.97886	1
0.45652	-1.07838	0.97886	1
0.45652	-1.07838	0.97886	1
0.45652	-1.07838	0.97886	1
0.45652	-1.07838	0.97886	1
0.45652	-1.07838	0.97886	1
0.45652	-1.07838	0.97886	1
0.45652	-1.07838	0.97886	1
0.45652	-1.07838	0.97886	1
0.45652	-1.07838	0.97886	1
0.45652	-1.07838	0.97886	1
0.45652	-1.07838	0.97886	1
0.45652	-1.07838	0.97886	1
0.45652	-1.07838	0.97886	1
0.45652	-1.07838	0.97886	1
0.45652	-1.07838	0.97886	1
0.45652	-1.07838	0.97886	1
0.45652	-1.07838	0.97886	1
0.45652	-1.07838	0.97886	1
0.45652	-1.07838	0.97886	1
0.45652	-1.07838	0.97886	1
0.45652	-1.07838	0.97886	1
0.45652	-1.07838	0.97886	1
0.45652	-1.07838	0.97886	1
0.45652	-1.07838	0.97886	1
0.45652	-1.07838	0.97886	1
0.45652	-1.07838	0.97886	1
0.45652	-1.07838	0.97886	1
0.45652	-1.07838	0.97886	1
0.45652	-1.07838	0.97886	1
0.45652	-1.07838	0.97886	1
0.45652	-1.07838	0.97886	1
0.45652	-1.07838	0.97886	1
0.45652	-1.07838	0.97886	1
0.45652	-1.07838	0.97886	1
0.45652	-1.07838	0.97886	1
0.45652	-1.07838	0.97886	1
0.25066	-1.60547	0.72694	0
0.25066	-1.60547	0.72694	0
0.25066	-1.60547	0.72694	0
0.25066	-1.60547	0.72694	0
0.25066	-1.60547	0.72694	0
0.25066	-1.60547	0.72694	0
0.25066	-1.60547	0.72694	0
0.25066	-1.60547	0.72694	0
0.25066	-1.60547	0.72694	0
0.25066	-1.60547	0.72694	0
0.25066	-1.60547	0.72694	0
0.25066	-1.60547	0.72694	0

0.25066	-1.60547	0.72694	0
0.25066	-1.60547	0.72694	0
0.25066	-1.60547	0.72694	0
0.25066	-1.60547	0.72694	0
0.25066	-1.60547	0.72694	0
0.25066	-1.60547	0.72694	0
0.25066	-1.60547	0.72694	0
0.25066	-1.60547	0.72694	0
0.25066	-1.60547	0.72694	0
0.25066	-1.60547	0.72694	0
0.25066	-1.60547	0.72694	0
0.25066	-1.60547	0.72694	0
0.25066	-1.60547	0.72694	0
0.25066	-1.60547	0.72694	0
0.25066	-1.60547	0.72694	0
0.25066	-1.60547	0.72694	0
0.25066	-1.60547	0.72694	0
0.25066	-1.60547	0.72694	0
0.25066	-1.60547	0.72694	0
0.25066	-1.60547	0.72694	0
0.25066	-1.60547	0.72694	0
0.25066	-1.60547	0.72694	0
0.25066	-1.60547	0.72694	0
0.25066	-1.60547	0.72694	0
0.25066	-1.60547	0.72694	0
0.25066	-1.60547	0.72694	0
0.25066	-1.60547	0.72694	0
0.25066	-1.60547	0.72694	0
0.25066	-1.60547	0.72694	0
0.25066	-1.60547	0.72694	0
0.25066	-1.60547	0.72694	0
0.25066	-1.60547	0.72694	0
0.25066	-1.60547	0.72694	0
0.25066	-1.60547	0.72694	0
0.25066	-1.60547	0.72694	0
0.25066	-1.60547	0.72694	0
0.25066	-1.60547	0.72694	0
0.25066	-1.60547	0.72694	0
0.25066	-1.60547	0.72694	0
0.25066	-1.60547	0.72694	0
0.25066	-1.60547	0.72694	0
0.25066	-1.60547	0.72694	0
0.25066	-1.60547	0.72694	0
0.25066	-1.60547	0.72694	0
0.25066	-1.60547	0.72694	0
0.25066	-1.60547	0.72694	0
0.25066	-1.60547	0.72694	0
0.25066	-1.60547	0.72694	0
0.25066	-1.60547	0.72694	0
0.25066	-1.60547	0.72694	0
0.25066	-1.60547	0.72694	0
0.25066	-1.60547	0.72694	0
0.25066	-1.60547	0.72694	0
0.25066	-1.60547	0.72694	0
0.25066	-1.60547	0.72694	0
0.25066	-1.60547	0.72694	0

0.25066	-1.60547	0.72694	0
0.25066	-1.60547	0.72694	0
0.25066	-1.60547	0.72694	0
0.25066	-1.60547	0.72694	0
0.25066	-1.60547	0.72694	0
0.25066	-1.60547	0.72694	0
0.25066	-1.60547	0.72694	0
0.25066	-1.60547	0.72694	0
0.25066	-1.60547	0.72694	0
0.25066	-1.60547	0.72694	0
0.25066	-1.60547	0.72694	0
0.25066	-1.60547	0.72694	0
0.25066	-1.60547	0.72694	0
0.25066	-1.60547	0.72694	0
0.25066	-1.60547	0.72694	0
0.25066	-1.60547	0.72694	0
0.25066	-1.60547	0.72694	0
0.25066	-1.60547	0.72694	0
0.25066	-1.60547	0.72694	0
0.25066	-1.60547	0.72694	0
0.25066	-1.60547	0.72694	0
0.25066	-1.60547	0.72694	0
0.25066	-1.60547	0.72694	0
0.25066	-1.60547	0.72694	0
0.25066	-1.60547	0.72694	0
0.25066	-1.60547	0.72694	0
0.25066	-1.60547	0.72694	0
0.25066	-1.60547	0.72694	0
0.25066	-1.60547	0.72694	0
0.25066	-1.60547	0.72694	0
0.25066	-1.60547	0.72694	0
0.25066	-1.60547	0.72694	0
0.25066	-1.60547	0.72694	0
0.25066	-1.60547	0.72694	0
0.25066	-1.60547	0.72694	0
0.25066	-1.60547	0.72694	0
0.25066	-1.60547	0.72694	0
0.25066	-1.60547	0.72694	0
0.25066	-1.60547	0.72694	0
0.25066	-1.60547	0.72694	0
0.25066	-1.60547	0.72694	0
0.25066	-1.60547	0.72694	0
0.25066	-1.60547	0.72694	0
0.25066	-1.60547	0.72694	0
0.25066	-1.60547	0.72694	0
0.25066	-1.60547	0.72694	0
0.25066	-1.60547	0.72694	0
0.25066	-1.60547	0.72694	0
0.25066	-1.60547	0.72694	0
0.25066	-1.60547	0.72694	0
0.25066	-1.60547	0.72694	0
0.25066	-1.60547	0.72694	0
0.25066	-1.60547	0.72694	0
0.25066	-1.60547	0.72694	0
0.25066	-1.60547	0.72694	0
0.25066	-1.60547	0.72694	0

0.25066	-1.60547	0.72694	0
0.25066	-1.60547	0.72694	0
0.25066	-1.60547	0.72694	0
0.25066	-1.60547	0.72694	0
0.25066	-1.60547	0.72694	0
0.25066	-1.60547	0.72694	0
0.25066	-1.60547	0.72694	0
0.25066	-1.60547	0.72694	0
0.25066	-1.60547	0.72694	0
0.25066	-1.60547	0.72694	0
0.25066	-1.60547	0.72694	0
0.25066	-1.60547	0.72694	0
0.25066	-1.60547	0.72694	0
0.25066	-1.60547	0.72694	0
0.25066	-1.60547	0.72694	0
0.25066	-1.60547	0.72694	0
0.25066	-1.60547	0.72694	0
0.25066	-1.60547	0.72694	0
0.25066	-1.60547	0.72694	0
0.25066	-1.60547	0.72694	0
0.25066	-1.60547	0.72694	0
0.25066	-1.60547	0.72694	0
0.25066	-1.60547	0.72694	0
0.25066	-1.60547	0.72694	0
0.25066	-1.60547	0.72694	0
0.25066	-1.60547	0.72694	0
0.25066	-1.60547	0.72694	0
0.25066	-1.60547	0.72694	0
0.25066	-1.60547	0.72694	0
0.25066	-1.60547	0.72694	0
0.25066	-1.60547	0.72694	0
0.25066	-1.60547	0.72694	0
0.25066	-1.60547	0.72694	0
0.25066	-1.60547	0.72694	0
0.25066	-1.60547	0.72694	0
0.25066	-1.60547	0.72694	0
0.25066	-1.60547	0.72694	0
0.25066	-1.60547	0.72694	0
0.25066	-1.60547	0.72694	0
0.25066	-1.60547	0.72694	0
0.25066	-1.60547	0.72694	0
0.25066	-1.60547	0.72694	0
0.25066	-1.60547	0.72694	0
0.25066	-1.60547	0.72694	0
0.25066	-1.60547	0.72694	0
0.25066	-1.60547	0.72694	0
0.25066	-1.60547	0.72694	0
0.25066	-1.60547	0.72694	0
0.25066	-1.60547	0.72694	0
0.25066	-1.60547	0.72694	0
0.25066	-1.60547	0.72694	0
0.25066	-1.60547	0.72694	0
0.25066	-1.60547	0.72694	0
0.25066	-1.60547	0.72694	0
0.25066	-1.60547	0.72694	0
0.25066	-1.60547	0.72694	0

0.25066	-1.60547	0.72694	0
0.25066	-1.60547	0.72694	0
0.25066	-1.60547	0.72694	0
0.25066	-1.60547	0.72694	0
0.25066	-1.60547	0.72694	0
0.25066	-1.60547	0.72694	0
0.25066	-1.60547	0.72694	0
0.25066	-1.60547	0.72694	0
0.25066	-1.60547	0.72694	0
0.25066	-1.60547	0.72694	0
0.25066	-1.60547	0.72694	0
0.25066	-1.60547	0.72694	0
0.25066	-1.60547	0.72694	0
0.25066	-1.60547	0.72694	0
0.25066	-1.60547	0.72694	0
0.25066	-1.60547	0.72694	0
0.25066	-1.60547	0.72694	0
0.25066	-1.60547	0.72694	0
0.25066	-1.60547	0.72694	0
0.25066	-1.60547	0.72694	0
0.25066	-1.60547	0.72694	0
0.25066	-1.60547	0.72694	0
0.25066	-1.60547	0.72694	0
0.25066	-1.60547	0.72694	0
0.25066	-1.60547	0.72694	0
0.25066	-1.60547	0.72694	0
0.25066	-1.60547	0.72694	0
0.25066	-1.60547	0.72694	0
0.25066	-1.60547	0.72694	0
0.25066	-1.60547	0.72694	0
0.25066	-1.60547	0.72694	0
0.25066	-1.60547	0.72694	0
0.25066	-1.60547	0.72694	0
0.25066	-1.60547	0.72694	0
0.25066	-1.60547	0.72694	0
0.25066	-1.60547	0.72694	0
0.25066	-1.60547	0.72694	0
0.25066	-1.60547	0.72694	0
0.25066	-1.60547	0.72694	0
0.25066	-1.60547	0.72694	0
0.25066	-1.60547	0.72694	0
0.25066	-1.60547	0.72694	0
0.25066	-1.60547	0.72694	0
0.25066	-1.60547	0.72694	0
0.25066	-1.60547	0.72694	0
0.25066	-1.60547	0.72694	0
0.25066	-1.60547	0.72694	0
0.25066	-1.60547	0.72694	0
0.25066	-1.60547	0.72694	0
0.25066	-1.60547	0.72694	0
0.25066	-1.60547	0.72694	0
0.25066	-1.60547	0.72694	0
0.25066	-1.60547	0.72694	0
0.25066	-1.60547	0.72694	0
0.25066	-1.60547	0.72694	0
0.25066	-1.60547	0.72694	0

0.25066	-1.60547	0.72694	0
0.25066	-1.60547	0.72694	0
0.25066	-1.60547	0.72694	0
0.25066	-1.60547	0.72694	0
0.25066	-1.60547	0.72694	0
0.25066	-1.60547	0.72694	0
0.25066	-1.60547	0.72694	0
0.25066	-1.60547	0.72694	0
0.25066	-1.60547	0.72694	0
0.25066	-1.60547	0.72694	0
0.25066	-1.60547	0.72694	0
0.25066	-1.60547	0.72694	0
0.25066	-1.60547	0.72694	0
0.25066	-1.60547	0.72694	0
0.25066	-1.60547	0.72694	0
0.25066	-1.60547	0.72694	0
0.25066	-1.60547	0.72694	0
0.25066	-1.60547	0.72694	0
0.25066	-1.60547	0.72694	0
0.25066	-1.60547	0.72694	0
0.25066	-1.60547	0.72694	0
0.25066	-1.60547	0.72694	0
0.25066	-1.60547	0.72694	0
0.25066	-1.60547	0.72694	0
0.25066	-1.60547	0.72694	0
0.25066	-1.60547	0.72694	0
0.25066	-1.60547	0.72694	0
0.25066	-1.60547	0.72694	0
0.25066	-1.60547	0.72694	0
0.25066	-1.60547	0.72694	0
0.25066	-1.60547	0.72694	0
0.25066	-1.60547	0.72694	0
0.25066	-1.60547	0.72694	0
0.25066	-1.60547	0.72694	0
0.25066	-1.60547	0.72694	0
0.25066	-1.60547	0.72694	0
0.25066	-1.60547	0.72694	0
0.25066	-1.60547	0.72694	0
0.25066	-1.60547	0.72694	0
0.25066	-1.60547	0.72694	0
0.25066	-1.60547	0.72694	0
0.25066	-1.60547	0.72694	0
0.25066	-1.60547	0.72694	0
0.25066	-1.60547	0.72694	0
0.25066	-1.60547	0.72694	0
0.25066	-1.60547	0.72694	0
0.25066	-1.60547	0.72694	0
0.25066	-1.60547	0.72694	0
0.25066	-1.60547	0.72694	0
0.25066	-1.60547	0.72694	0
0.25066	-1.60547	0.72694	0
0.25066	-1.60547	0.72694	0
0.25066	-1.60547	0.72694	0
0.25066	-1.60547	0.72694	0
0.25066	-1.60547	0.72694	0
0.25066	-1.60547	0.72694	0

0.25066	-1.60547	0.72694	0
0.25066	-1.60547	0.72694	0
0.25066	-1.60547	0.72694	0
0.25066	-1.60547	0.72694	0
0.25066	-1.60547	0.72694	0
0.25066	-1.60547	0.72694	0
0.25066	-1.60547	0.72694	0
0.25066	-1.60547	0.72694	0
0.25066	-1.60547	0.72694	0
0.25066	-1.60547	0.72694	0
0.25066	-1.60547	0.72694	0
0.25066	-1.60547	0.72694	0
0.25066	-1.60547	0.72694	0
0.25066	-1.60547	0.72694	0
0.25066	-1.60547	0.72694	0
0.25066	-1.60547	0.72694	0
0.25066	-1.60547	0.72694	0
0.25066	-1.60547	0.72694	0
0.25066	-1.60547	0.72694	0
0.25066	-1.60547	0.72694	0
0.25066	-1.60547	0.72694	0
0.25066	-1.60547	0.72694	0
0.25066	-1.60547	0.72694	0
0.25066	-1.60547	0.72694	0
0.25066	-1.60547	0.72694	0
0.25066	-1.60547	0.72694	0
0.25066	-1.60547	0.72694	0
0.25066	-1.60547	0.72694	0
0.25066	-1.60547	0.72694	0
0.25066	-1.60547	0.72694	0
0.25066	-1.60547	0.72694	0
0.25066	-1.60547	0.72694	0
0.25066	-1.60547	0.72694	0
0.25066	-1.60547	0.72694	0
0.25066	-1.60547	0.72694	0
0.25066	-1.60547	0.72694	0
0.25066	-1.60547	0.72694	0
0.25066	-1.60547	0.72694	0
0.25066	-1.60547	0.72694	0
0.25066	-1.60547	0.72694	0
0.25066	-1.60547	0.72694	0
0.25066	-1.60547	0.72694	0
0.25066	-1.60547	0.72694	0
0.25066	-1.60547	0.72694	0
0.25066	-1.60547	0.72694	0
0.25066	-1.60547	0.72694	0
0.25066	-1.60547	0.72694	0
0.25066	-1.60547	0.72694	0
0.25066	-1.60547	0.72694	0
0.25066	-1.60547	0.72694	0
0.25066	-1.60547	0.72694	0
0.25066	-1.60547	0.72694	0
0.25066	-1.60547	0.72694	0
0.25066	-1.60547	0.72694	0
0.25066	-1.60547	0.72694	0
0.25066	-1.60547	0.72694	0

0.25066	-1.60547	0.72694	0
0.25066	-1.60547	0.72694	0
0.25066	-1.60547	0.72694	0
0.25066	-1.60547	0.72694	0
0.25066	-1.60547	0.72694	0
0.25066	-1.60547	0.72694	0
0.25066	-1.60547	0.72694	0
0.25066	-1.60547	0.72694	0
0.25066	-1.60547	0.72694	0
0.25066	-1.60547	0.72694	0
0.25066	-1.60547	0.72694	0
0.25066	-1.60547	0.72694	0
0.25066	-1.60547	0.72694	0
0.25066	-1.60547	0.72694	0
0.25066	-1.60547	0.72694	0
0.25066	-1.60547	0.72694	0
0.25066	-1.60547	0.72694	0
0.25066	-1.60547	0.72694	0
0.25066	-1.60547	0.72694	0
0.25066	-1.60547	0.72694	0
0.25066	-1.60547	0.72694	0
0.25066	-1.60547	0.72694	0
0.25066	-1.60547	0.72694	0
0.25066	-1.60547	0.72694	0
0.25066	-1.60547	0.72694	0
0.25066	-1.60547	0.72694	0
0.25066	-1.60547	0.72694	0
0.25066	-1.60547	0.72694	0
0.25066	-1.60547	0.72694	0
0.25066	-1.60547	0.72694	0
0.25066	-1.60547	0.72694	0
0.25066	-1.60547	0.72694	0
0.25066	-1.60547	0.72694	0
0.25066	-1.60547	0.72694	0
0.25066	-1.60547	0.72694	0
0.25066	-1.60547	0.72694	0
0.25066	-1.60547	0.72694	0
0.25066	-1.60547	0.72694	0
0.25066	-1.60547	0.72694	0
0.25066	-1.60547	0.72694	0
0.25066	-1.60547	0.72694	0
0.25066	-1.60547	0.72694	0
0.25066	-1.60547	0.72694	0
0.25066	-1.60547	0.72694	0
0.25066	-1.60547	0.72694	0
0.25066	-1.60547	0.72694	0
0.25066	-1.60547	0.72694	0
0.25066	-1.60547	0.72694	0
0.25066	-1.60547	0.72694	0
0.25066	-1.60547	0.72694	0
0.25066	-1.60547	0.72694	0
0.25066	-1.60547	0.72694	0
0.25066	-1.60547	0.72694	0
0.25066	-1.60547	0.72694	0
0.25066	-1.60547	0.72694	0
0.25066	-1.60547	0.72694	0

0.25066	-1.60547	0.72694	0
0.25066	-1.60547	0.72694	0
0.25066	-1.60547	0.72694	0
0.25066	-1.60547	0.72694	0
0.25066	-1.60547	0.72694	0
0.25066	-1.60547	0.72694	0
0.25066	-1.60547	0.72694	0
0.25066	-1.60547	0.72694	0
0.25066	-1.60547	0.72694	0
0.25066	-1.60547	0.72694	0
0.25066	-1.60547	0.72694	0
0.25066	-1.60547	0.72694	0
0.25066	-1.60547	0.72694	0
0.25066	-1.60547	0.72694	0
0.77859	-1.39959	0.99459	1
0.77859	-1.39959	0.99459	1
0.77859	-1.39959	0.99459	1
0.77859	-1.39959	0.99459	1
0.77859	-1.39959	0.99459	1
0.77859	-1.39959	0.99459	1
0.77859	-1.39959	0.99459	1
0.77859	-1.39959	0.99459	1
0.77859	-1.39959	0.99459	1
0.77859	-1.39959	0.99459	1
0.77859	-1.39959	0.99459	1
0.77859	-1.39959	0.99459	1
0.77859	-1.39959	0.99459	1
0.77859	-1.39959	0.99459	1
0.77859	-1.39959	0.99459	1
0.77859	-1.39959	0.99459	1
0.77859	-1.39959	0.99459	1
0.77859	-1.39959	0.99459	1
0.77859	-1.39959	0.99459	1
0.77859	-1.39959	0.99459	1
0.77859	-1.39959	0.99459	1
0.77859	-1.39959	0.99459	1
0.77859	-1.39959	0.99459	1
0.77859	-1.39959	0.99459	1
0.77859	-1.39959	0.99459	1
0.77859	-1.39959	0.99459	1
0.77859	-1.39959	0.99459	1
0.77859	-1.39959	0.99459	1
0.77859	-1.39959	0.99459	1
0.77859	-1.39959	0.99459	1
0.77859	-1.39959	0.99459	1
0.77859	-1.39959	0.99459	1
0.77859	-1.39959	0.99459	1
0.77859	-1.39959	0.99459	1
0.77859	-1.39959	0.99459	1
0.77859	-1.39959	0.99459	1
0.77859	-1.39959	0.99459	1
0.77859	-1.39959	0.99459	1
0.77859	-1.39959	0.99459	1
0.77859	-1.39959	0.99459	1
0.77859	-1.39959	0.99459	1
0.77859	-1.39959	0.99459	1

0.77859	-1.39959	0.99459	1
0.77859	-1.39959	0.99459	1
0.77859	-1.39959	0.99459	1
0.25066	-1.60547	0.72694	0
0.25066	-1.60547	0.72694	0
0.25066	-1.60547	0.72694	0
0.25066	-1.60547	0.72694	0
0.25066	-1.60547	0.72694	0
0.25066	-1.60547	0.72694	0
0.25066	-1.60547	0.72694	0
0.25066	-1.60547	0.72694	0
0.25066	-1.60547	0.72694	0
0.25066	-1.60547	0.72694	0
0.25066	-1.60547	0.72694	0
0.25066	-1.60547	0.72694	0
0.25066	-1.60547	0.72694	0
0.25066	-1.60547	0.72694	0
0.25066	-1.60547	0.72694	0
0.25066	-1.60547	0.72694	0
0.25066	-1.60547	0.72694	0
0.25066	-1.60547	0.72694	0
0.25066	-1.60547	0.72694	0
0.25066	-1.60547	0.72694	0
0.25066	-1.60547	0.72694	0
0.25066	-1.60547	0.72694	0
0.25066	-1.60547	0.72694	0
0.25066	-1.60547	0.72694	0
0.25066	-1.60547	0.72694	0
0.25066	-1.60547	0.72694	0
0.25066	-1.60547	0.72694	0
0.25066	-1.60547	0.72694	0
0.25066	-1.60547	0.72694	0
0.25066	-1.60547	0.72694	0
0.25066	-1.60547	0.72694	0
0.25066	-1.60547	0.72694	0
0.25066	-1.60547	0.72694	0
0.25066	-1.60547	0.72694	0
0.25066	-1.60547	0.72694	0
0.25066	-1.60547	0.72694	0
0.25066	-1.60547	0.72694	0
0.25066	-1.60547	0.72694	0
0.25066	-1.60547	0.72694	0
0.25066	-1.60547	0.72694	0
0.25066	-1.60547	0.72694	0
0.25066	-1.60547	0.72694	0
0.25066	-1.60547	0.72694	0
0.25066	-1.60547	0.72694	0
0.25066	-1.60547	0.72694	0
0.25066	-1.60547	0.72694	0
0.25066	-1.60547	0.72694	0
0.25066	-1.60547	0.72694	0
0.25066	-1.60547	0.72694	0
0.25066	-1.60547	0.72694	0
0.25066	-1.60547	0.72694	0
0.25066	-1.60547	0.72694	0
0.25066	-1.60547	0.72694	0

0.25066	-1.60547	0.72694	0
0.25066	-1.60547	0.72694	0
0.25066	-1.60547	0.72694	0
0.25066	-1.60547	0.72694	0
0.25066	-1.60547	0.72694	0
0.25066	-1.60547	0.72694	0
0.25066	-1.60547	0.72694	0
0.25066	-1.60547	0.72694	0
0.25066	-1.60547	0.72694	0
0.25066	-1.60547	0.72694	0
0.25066	-1.60547	0.72694	0
0.77859	-1.39959	0.99459	1
0.77859	-1.39959	0.99459	1
0.77859	-1.39959	0.99459	1
0.77859	-1.39959	0.99459	1
0.77859	-1.39959	0.99459	1
0.77859	-1.39959	0.99459	1
0.77859	-1.39959	0.99459	1
0.77859	-1.39959	0.99459	1
0.77859	-1.39959	0.99459	1
0.77859	-1.39959	0.99459	1
0.77859	-1.39959	0.99459	1
0.77859	-1.39959	0.99459	1
0.77859	-1.39959	0.99459	1
0.77859	-1.39959	0.99459	1
0.25066	-1.60547	0.72694	0
0.25066	-1.60547	0.72694	0
0.25066	-1.60547	0.72694	0
0.25066	-1.60547	0.72694	0
0.25066	-1.60547	0.72694	0
0.25066	-1.60547	0.72694	0
0.25066	-1.60547	0.72694	0
0.25066	-1.60547	0.72694	0
0.25066	-1.60547	0.72694	0
0.25066	-1.60547	0.72694	0
0.25066	-1.60547	0.72694	0
0.25066	-1.60547	0.72694	0
0.25066	-1.60547	0.72694	0
0.25066	-1.60547	0.72694	0
0.25066	-1.60547	0.72694	0
0.25066	-1.60547	0.72694	0
0.25066	-1.60547	0.72694	0
0.25066	-1.60547	0.72694	0
0.25066	-1.60547	0.72694	0
0.25066	-1.60547	0.72694	0
0.25066	-1.60547	0.72694	0
0.25066	-1.60547	0.72694	0
0.25066	-1.60547	0.72694	0
0.25066	-1.60547	0.72694	0
0.25066	-1.60547	0.72694	0
0.25066	-1.60547	0.72694	0
0.25066	-1.60547	0.72694	0
0.25066	-1.60547	0.72694	0
0.25066	-1.60547	0.72694	0
0.25066	-1.60547	0.72694	0
0.25066	-1.60547	0.72694	0

0.25066	-1.60547	0.72694	0
0.25066	-1.60547	0.72694	0
0.25066	-1.60547	0.72694	0
0.25066	-1.60547	0.72694	0
0.25066	-1.60547	0.72694	0
0.25066	-1.60547	0.72694	0
0.25066	-1.60547	0.72694	0
0.25066	-1.60547	0.72694	0
0.25066	-1.60547	0.72694	0
0.25066	-1.60547	0.72694	0
0.25066	-1.60547	0.72694	0
0.25066	-1.60547	0.72694	0
0.25066	-1.60547	0.72694	0
0.25066	-1.60547	0.72694	0
0.25066	-1.60547	0.72694	0
0.25066	-1.60547	0.72694	0
0.25066	-1.60547	0.72694	0
0.77859	-1.39959	0.99459	1
0.77859	-1.39959	0.99459	1
0.77859	-1.39959	0.99459	1
0.77859	-1.39959	0.99459	1
0.77859	-1.39959	0.99459	1
0.77859	-1.39959	0.99459	1
0.77859	-1.39959	0.99459	1
0.77859	-1.39959	0.99459	1
0.77859	-1.39959	0.99459	1
0.77859	-1.39959	0.99459	1
0.77859	-1.39959	0.99459	1
0.77859	-1.39959	0.99459	1
0.77859	-1.39959	0.99459	1
0.77859	-1.39959	0.99459	1
0.77859	-1.39959	0.99459	1
0.77859	-1.39959	0.99459	1
0.77859	-1.39959	0.99459	1
0.77859	-1.39959	0.99459	1
0.77859	-1.39959	0.99459	1
0.77859	-1.39959	0.99459	1
0.77859	-1.39959	0.99459	1
0.77859	-1.39959	0.99459	1
0.77859	-1.39959	0.99459	1
0.77859	-1.39959	0.99459	1
0.77859	-1.39959	0.99459	1
0.77859	-1.39959	0.99459	1
0.77859	-1.39959	0.99459	1
0.77859	-1.39959	0.99459	1
0.77859	-1.39959	0.99459	1
0.77859	-1.39959	0.99459	1
0.77859	-1.39959	0.99459	1
0.77859	-1.39959	0.99459	1
0.77859	-1.39959	0.99459	1
0.77859	-1.39959	0.99459	1
0.77859	-1.39959	0.99459	1
0.77859	-1.39959	0.99459	1
0.77859	-1.39959	0.99459	1
0.77859	-1.39959	0.99459	1
0.77859	-1.39959	0.99459	1
0.77859	-1.39959	0.99459	1

0.77859	-1.39959	0.99459	1
0.77859	-1.39959	0.99459	1
0.77859	-1.39959	0.99459	1
0.77859	-1.39959	0.99459	1
0.77859	-1.39959	0.99459	1
0.77859	-1.39959	0.99459	1
0.77859	-1.39959	0.99459	1
0.77859	-1.39959	0.99459	1
0.77859	-1.39959	0.99459	1
0.77859	-1.39959	0.99459	1
0.77859	-1.39959	0.99459	1
0.77859	-1.39959	0.99459	1
0.77859	-1.39959	0.99459	1
0.77859	-1.39959	0.99459	1
0.77859	-1.39959	0.99459	1
0.77859	-1.39959	0.99459	1
0.77859	-1.39959	0.99459	1
0.77859	-1.39959	0.99459	1
0.77859	-1.39959	0.99459	1
0.77859	-1.39959	0.99459	1
0.25066	-1.60547	0.72694	0
0.25066	-1.60547	0.72694	0
0.25066	-1.60547	0.72694	0
0.25066	-1.60547	0.72694	0
0.25066	-1.60547	0.72694	0
0.25066	-1.60547	0.72694	0
0.25066	-1.60547	0.72694	0
0.25066	-1.60547	0.72694	0
0.25066	-1.60547	0.72694	0
0.25066	-1.60547	0.72694	0
0.25066	-1.60547	0.72694	0
0.25066	-1.60547	0.72694	0
0.25066	-1.60547	0.72694	0
0.25066	-1.60547	0.72694	0
0.25066	-1.60547	0.72694	0
0.25066	-1.60547	0.72694	0
0.25066	-1.60547	0.72694	0
0.25066	-1.60547	0.72694	0
0.25066	-1.60547	0.72694	0
0.25066	-1.60547	0.72694	0
0.25066	-1.60547	0.72694	0
0.25066	-1.60547	0.72694	0
0.25066	-1.60547	0.72694	0
0.25066	-1.60547	0.72694	0
0.25066	-1.60547	0.72694	0
0.25066	-1.60547	0.72694	0
0.25066	-1.60547	0.72694	0
0.25066	-1.60547	0.72694	0
0.25066	-1.60547	0.72694	0
0.25066	-1.60547	0.72694	0
0.25066	-1.60547	0.72694	0
0.25066	-1.60547	0.72694	0
0.25066	-1.60547	0.72694	0
0.25066	-1.60547	0.72694	0
0.25066	-1.60547	0.72694	0
0.25066	-1.60547	0.72694	0

0.25066	-1.60547	0.72694	0
0.25066	-1.60547	0.72694	0
0.25066	-1.60547	0.72694	0
0.25066	-1.60547	0.72694	0
0.25066	-1.60547	0.72694	0
0.25066	-1.60547	0.72694	0
0.25066	-1.60547	0.72694	0
0.25066	-1.60547	0.72694	0
0.25066	-1.60547	0.72694	0
0.25066	-1.60547	0.72694	0
0.25066	-1.60547	0.72694	0
0.25066	-1.60547	0.72694	0
0.77859	-1.39959	0.99459	1
0.77859	-1.39959	0.99459	1
0.77859	-1.39959	0.99459	1
0.77859	-1.39959	0.99459	1
0.77859	-1.39959	0.99459	1
0.77859	-1.39959	0.99459	1
0.77859	-1.39959	0.99459	1
0.77859	-1.39959	0.99459	1
0.77859	-1.39959	0.99459	1
0.77859	-1.39959	0.99459	1
0.77859	-1.39959	0.99459	1
0.77859	-1.39959	0.99459	1
0.77859	-1.39959	0.99459	1
0.77859	-1.39959	0.99459	1
0.77859	-1.39959	0.99459	1
0.77859	-1.39959	0.99459	1
0.77859	-1.39959	0.99459	1
0.77859	-1.39959	0.99459	1
0.77859	-1.39959	0.99459	1
0.77859	-1.39959	0.99459	1
0.77859	-1.39959	0.99459	1
0.77859	-1.39959	0.99459	1
0.77859	-1.39959	0.99459	1
0.77859	-1.39959	0.99459	1
0.77859	-1.39959	0.99459	1
0.77859	-1.39959	0.99459	1
0.77859	-1.39959	0.99459	1
0.77859	-1.39959	0.99459	1
0.77859	-1.39959	0.99459	1
0.77859	-1.39959	0.99459	1
0.77859	-1.39959	0.99459	1
0.77859	-1.39959	0.99459	1
0.77859	-1.39959	0.99459	1
0.77859	-1.39959	0.99459	1
0.77859	-1.39959	0.99459	1
0.77859	-1.39959	0.99459	1
0.77859	-1.39959	0.99459	1
0.77859	-1.39959	0.99459	1
0.77859	-1.39959	0.99459	1
0.77859	-1.39959	0.99459	1
0.77859	-1.39959	0.99459	1
0.77859	-1.39959	0.99459	1
0.77859	-1.39959	0.99459	1
0.77859	-1.39959	0.99459	1

0.77859	-1.39959	0.99459	1
0.77859	-1.39959	0.99459	1
0.77859	-1.39959	0.99459	1
0.77859	-1.39959	0.99459	1
0.77859	-1.39959	0.99459	1
0.77859	-1.39959	0.99459	1
0.77859	-1.39959	0.99459	1
0.77859	-1.39959	0.99459	1
0.77859	-1.39959	0.99459	1
0.77859	-1.39959	0.99459	1
0.77859	-1.39959	0.99459	1
0.77859	-1.39959	0.99459	1
0.77859	-1.39959	0.99459	1
0.77859	-1.39959	0.99459	1
0.77859	-1.39959	0.99459	1
0.77859	-1.39959	0.99459	1
0.25066	-1.60547	0.72694	0
0.25066	-1.60547	0.72694	0
0.25066	-1.60547	0.72694	0
0.25066	-1.60547	0.72694	0
0.25066	-1.60547	0.72694	0
0.25066	-1.60547	0.72694	0
0.25066	-1.60547	0.72694	0
0.25066	-1.60547	0.72694	0
0.25066	-1.60547	0.72694	0
0.25066	-1.60547	0.72694	0
0.25066	-1.60547	0.72694	0
0.25066	-1.60547	0.72694	0
0.25066	-1.60547	0.72694	0
0.25066	-1.60547	0.72694	0
0.25066	-1.60547	0.72694	0
0.25066	-1.60547	0.72694	0
0.25066	-1.60547	0.72694	0
0.25066	-1.60547	0.72694	0
0.25066	-1.60547	0.72694	0
0.25066	-1.60547	0.72694	0
0.25066	-1.60547	0.72694	0
0.25066	-1.60547	0.72694	0
0.25066	-1.60547	0.72694	0
0.25066	-1.60547	0.72694	0
0.25066	-1.60547	0.72694	0
0.25066	-1.60547	0.72694	0
0.25066	-1.60547	0.72694	0
0.25066	-1.60547	0.72694	0
0.25066	-1.60547	0.72694	0
0.25066	-1.60547	0.72694	0
0.25066	-1.60547	0.72694	0
0.25066	-1.60547	0.72694	0
0.25066	-1.60547	0.72694	0
0.25066	-1.60547	0.72694	0
0.25066	-1.60547	0.72694	0
0.25066	-1.60547	0.72694	0
0.25066	-1.60547	0.72694	0
0.25066	-1.60547	0.72694	0
0.25066	-1.60547	0.72694	0
0.25066	-1.60547	0.72694	0

0.25066	-1.60547	0.72694	0
0.25066	-1.60547	0.72694	0
0.25066	-1.60547	0.72694	0
0.25066	-1.60547	0.72694	0
0.25066	-1.60547	0.72694	0
0.25066	-1.60547	0.72694	0
0.25066	-1.60547	0.72694	0
0.25066	-1.60547	0.72694	0
0.25066	-1.60547	0.72694	0
0.25066	-1.60547	0.72694	0
0.25066	-1.60547	0.72694	0
0.25066	-1.60547	0.72694	0
0.25066	-1.60547	0.72694	0
0.25066	-1.60547	0.72694	0
0.25066	-1.60547	0.72694	0
0.25066	-1.60547	0.72694	0
0.25066	-1.60547	0.72694	0
0.25066	-1.60547	0.72694	0
0.25066	-1.60547	0.72694	0
0.25066	-1.60547	0.72694	0
0.25066	-1.60547	0.72694	0
0.25066	-1.60547	0.72694	0
0.25066	-1.60547	0.72694	0
0.77859	-1.39959	0.99459	1
0.77859	-1.39959	0.99459	1
0.77859	-1.39959	0.99459	1
0.77859	-1.39959	0.99459	1
0.77859	-1.39959	0.99459	1
0.77859	-1.39959	0.99459	1
0.77859	-1.39959	0.99459	1
0.77859	-1.39959	0.99459	1
0.77859	-1.39959	0.99459	1
0.77859	-1.39959	0.99459	1
0.77859	-1.39959	0.99459	1
0.77859	-1.39959	0.99459	1
0.77859	-1.39959	0.99459	1
0.77859	-1.39959	0.99459	1
0.77859	-1.39959	0.99459	1
0.77859	-1.39959	0.99459	1
0.77859	-1.39959	0.99459	1
0.77859	-1.39959	0.99459	1
0.77859	-1.39959	0.99459	1
0.77859	-1.39959	0.99459	1
0.77859	-1.39959	0.99459	1
0.77859	-1.39959	0.99459	1
0.77859	-1.39959	0.99459	1
0.77859	-1.39959	0.99459	1
0.77859	-1.39959	0.99459	1
0.77859	-1.39959	0.99459	1
0.77859	-1.39959	0.99459	1
0.77859	-1.39959	0.99459	1
0.77859	-1.39959	0.99459	1
0.77859	-1.39959	0.99459	1
0.77859	-1.39959	0.99459	1
0.77859	-1.39959	0.99459	1
0.77859	-1.39959	0.99459	1

0.77859	-1.39959	0.99459	1
0.77859	-1.39959	0.99459	1
0.77859	-1.39959	0.99459	1
0.77859	-1.39959	0.99459	1
0.77859	-1.39959	0.99459	1
0.77859	-1.39959	0.99459	1
0.77859	-1.39959	0.99459	1
0.77859	-1.39959	0.99459	1
0.77859	-1.39959	0.99459	1
0.77859	-1.39959	0.99459	1
0.77859	-1.39959	0.99459	1
0.77859	-1.39959	0.99459	1
0.77859	-1.39959	0.99459	1
0.77859	-1.39959	0.99459	1
0.77859	-1.39959	0.99459	1
0.77859	-1.39959	0.99459	1
0.77859	-1.39959	0.99459	1
0.77859	-1.39959	0.99459	1
0.77859	-1.39959	0.99459	1
0.77859	-1.39959	0.99459	1
0.77859	-1.39959	0.99459	1
0.77859	-1.39959	0.99459	1
0.77859	-1.39959	0.99459	1
0.77859	-1.39959	0.99459	1
0.77859	-1.39959	0.99459	1
0.77859	-1.39959	0.99459	1
0.77859	-1.39959	0.99459	1
0.25066	-1.60547	0.72694	0
0.25066	-1.60547	0.72694	0
0.25066	-1.60547	0.72694	0
0.25066	-1.60547	0.72694	0
0.25066	-1.60547	0.72694	0
0.25066	-1.60547	0.72694	0
0.25066	-1.60547	0.72694	0
0.25066	-1.60547	0.72694	0
0.25066	-1.60547	0.72694	0
0.25066	-1.60547	0.72694	0
0.25066	-1.60547	0.72694	0
0.25066	-1.60547	0.72694	0
0.25066	-1.60547	0.72694	0
0.25066	-1.60547	0.72694	0
0.25066	-1.60547	0.72694	0
0.25066	-1.60547	0.72694	0
0.25066	-1.60547	0.72694	0
0.25066	-1.60547	0.72694	0
0.25066	-1.60547	0.72694	0
0.25066	-1.60547	0.72694	0
0.25066	-1.60547	0.72694	0
0.25066	-1.60547	0.72694	0
0.25066	-1.60547	0.72694	0
0.25066	-1.60547	0.72694	0
0.25066	-1.60547	0.72694	0
0.25066	-1.60547	0.72694	0
0.25066	-1.60547	0.72694	0
0.25066	-1.60547	0.72694	0
0.25066	-1.60547	0.72694	0

0.25066	-1.60547	0.72694	0
0.25066	-1.60547	0.72694	0
0.25066	-1.60547	0.72694	0
0.25066	-1.60547	0.72694	0
0.25066	-1.60547	0.72694	0
0.25066	-1.60547	0.72694	0
0.25066	-1.60547	0.72694	0
0.25066	-1.60547	0.72694	0
0.25066	-1.60547	0.72694	0
0.25066	-1.60547	0.72694	0
0.25066	-1.60547	0.72694	0
0.25066	-1.60547	0.72694	0
0.25066	-1.60547	0.72694	0
0.25066	-1.60547	0.72694	0
0.25066	-1.60547	0.72694	0
0.25066	-1.60547	0.72694	0
0.25066	-1.60547	0.72694	0
0.25066	-1.60547	0.72694	0
0.25066	-1.60547	0.72694	0
0.25066	-1.60547	0.72694	0
0.25066	-1.60547	0.72694	0
0.25066	-1.60547	0.72694	0
0.25066	-1.60547	0.72694	0
0.25066	-1.60547	0.72694	0
0.25066	-1.60547	0.72694	0
0.25066	-1.60547	0.72694	0
0.25066	-1.60547	0.72694	0
0.25066	-1.60547	0.72694	0
0.25066	-1.60547	0.72694	0
0.25066	-1.60547	0.72694	0
0.25066	-1.60547	0.72694	0
0.25066	-1.60547	0.72694	0
0.25066	-1.60547	0.72694	0
0.25066	-1.60547	0.72694	0
0.25066	-1.60547	0.72694	0
0.25066	-1.60547	0.72694	0
0.25066	-1.60547	0.72694	0
0.25066	-1.60547	0.72694	0
0.25066	-1.60547	0.72694	0
0.25066	-1.60547	0.72694	0
0.25066	-1.60547	0.72694	0
0.25066	-1.60547	0.72694	0
0.25066	-1.60547	0.72694	0
0.25066	-1.60547	0.72694	0
0.25066	-1.60547	0.72694	0
0.25066	-1.60547	0.72694	0
0.25066	-1.60547	0.72694	0
0.25066	-1.60547	0.72694	0
0.25066	-1.60547	0.72694	0
0.25066	-1.60547	0.72694	0
0.25066	-1.60547	0.72694	0
0.25066	-1.60547	0.72694	0
0.25066	-1.60547	0.72694	0
0.25066	-1.60547	0.72694	0
0.25066	-1.60547	0.72694	0
0.25066	-1.60547	0.72694	0

0.25066	-1.60547	0.72694	0
0.25066	-1.60547	0.72694	0
0.25066	-1.60547	0.72694	0
0.25066	-1.60547	0.72694	0
0.25066	-1.60547	0.72694	0
0.25066	-1.60547	0.72694	0
0.25066	-1.60547	0.72694	0
0.25066	-1.60547	0.72694	0
0.25066	-1.60547	0.72694	0
0.25066	-1.60547	0.72694	0
0.25066	-1.60547	0.72694	0
0.25066	-1.60547	0.72694	0
0.25066	-1.60547	0.72694	0
0.25066	-1.60547	0.72694	0
0.25066	-1.60547	0.72694	0
0.25066	-1.60547	0.72694	0
0.25066	-1.60547	0.72694	0
0.25066	-1.60547	0.72694	0
0.25066	-1.60547	0.72694	0
0.25066	-1.60547	0.72694	0
0.25066	-1.60547	0.72694	0
0.25066	-1.60547	0.72694	0
0.25066	-1.60547	0.72694	0
0.25066	-1.60547	0.72694	0
0.25066	-1.60547	0.72694	0
0.25066	-1.60547	0.72694	0
0.25066	-1.60547	0.72694	0
0.25066	-1.60547	0.72694	0
0.25066	-1.60547	0.72694	0
0.25066	-1.60547	0.72694	0
0.25066	-1.60547	0.72694	0
0.25066	-1.60547	0.72694	0
0.25066	-1.60547	0.72694	0
0.25066	-1.60547	0.72694	0
0.25066	-1.60547	0.72694	0
0.25066	-1.60547	0.72694	0
0.25066	-1.60547	0.72694	0
0.25066	-1.60547	0.72694	0
0.25066	-1.60547	0.72694	0
0.25066	-1.60547	0.72694	0
0.77859	-1.39959	0.99459	1
0.77859	-1.39959	0.99459	1
0.77859	-1.39959	0.99459	1
0.77859	-1.39959	0.99459	1
0.77859	-1.39959	0.99459	1
0.77859	-1.39959	0.99459	1
0.77859	-1.39959	0.99459	1
0.77859	-1.39959	0.99459	1
0.77859	-1.39959	0.99459	1
0.77859	-1.39959	0.99459	1
0.77859	-1.39959	0.99459	1
0.77859	-1.39959	0.99459	1
0.77859	-1.39959	0.99459	1
0.77859	-1.39959	0.99459	1
0.77859	-1.39959	0.99459	1
0.77859	-1.39959	0.99459	1

0.77859	-1.39959	0.99459	1
0.77859	-1.39959	0.99459	1
0.77859	-1.39959	0.99459	1
0.77859	-1.39959	0.99459	1
0.77859	-1.39959	0.99459	1
0.77859	-1.39959	0.99459	1
0.77859	-1.39959	0.99459	1
0.77859	-1.39959	0.99459	1
0.77859	-1.39959	0.99459	1
0.77859	-1.39959	0.99459	1
0.77859	-1.39959	0.99459	1
0.77859	-1.39959	0.99459	1
0.77859	-1.39959	0.99459	1
0.77859	-1.39959	0.99459	1
0.77859	-1.39959	0.99459	1
0.77859	-1.39959	0.99459	1
0.77859	-1.39959	0.99459	1
0.77859	-1.39959	0.99459	1
0.77859	-1.39959	0.99459	1
0.77859	-1.39959	0.99459	1
0.77859	-1.39959	0.99459	1
0.77859	-1.39959	0.99459	1
0.77859	-1.39959	0.99459	1
0.77859	-1.39959	0.99459	1
0.77859	-1.39959	0.99459	1
0.77859	-1.39959	0.99459	1
0.77859	-1.39959	0.99459	1
0.77859	-1.39959	0.99459	1
0.77859	-1.39959	0.99459	1
0.77859	-1.39959	0.99459	1
0.77859	-1.39959	0.99459	1
0.77859	-1.39959	0.99459	1
0.77859	-1.39959	0.99459	1
0.77859	-1.39959	0.99459	1
0.77859	-1.39959	0.99459	1
0.77859	-1.39959	0.99459	1
0.77859	-1.39959	0.99459	1
0.77859	-1.39959	0.99459	1
0.77859	-1.39959	0.99459	1
0.77859	-1.39959	0.99459	1
0.77859	-1.39959	0.99459	1
0.77859	-1.39959	0.99459	1
0.77859	-1.39959	0.99459	1
0.77859	-1.39959	0.99459	1
0.25066	-1.60547	0.72694	0
0.25066	-1.60547	0.72694	0
0.25066	-1.60547	0.72694	0
0.25066	-1.60547	0.72694	0
0.25066	-1.60547	0.72694	0
0.25066	-1.60547	0.72694	0
0.25066	-1.60547	0.72694	0
0.25066	-1.60547	0.72694	0
0.25066	-1.60547	0.72694	0
0.25066	-1.60547	0.72694	0
0.25066	-1.60547	0.72694	0
0.25066	-1.60547	0.72694	0

0.25066	-1.60547	0.72694	0
0.25066	-1.60547	0.72694	0
0.25066	-1.60547	0.72694	0
0.25066	-1.60547	0.72694	0
0.25066	-1.60547	0.72694	0
0.25066	-1.60547	0.72694	0
0.25066	-1.60547	0.72694	0
0.25066	-1.60547	0.72694	0
0.25066	-1.60547	0.72694	0
0.25066	-1.60547	0.72694	0
0.25066	-1.60547	0.72694	0
0.25066	-1.60547	0.72694	0
0.25066	-1.60547	0.72694	0
0.25066	-1.60547	0.72694	0
0.25066	-1.60547	0.72694	0
0.25066	-1.60547	0.72694	0
0.25066	-1.60547	0.72694	0
0.25066	-1.60547	0.72694	0
0.25066	-1.60547	0.72694	0
0.25066	-1.60547	0.72694	0
0.25066	-1.60547	0.72694	0
0.25066	-1.60547	0.72694	0
0.25066	-1.60547	0.72694	0
0.25066	-1.60547	0.72694	0
0.25066	-1.60547	0.72694	0
0.25066	-1.60547	0.72694	0
0.25066	-1.60547	0.72694	0
0.25066	-1.60547	0.72694	0
0.25066	-1.60547	0.72694	0
0.25066	-1.60547	0.72694	0
0.25066	-1.60547	0.72694	0
0.25066	-1.60547	0.72694	0
0.25066	-1.60547	0.72694	0
0.25066	-1.60547	0.72694	0
0.25066	-1.60547	0.72694	0
0.25066	-1.60547	0.72694	0
0.25066	-1.60547	0.72694	0
0.25066	-1.60547	0.72694	0
0.25066	-1.60547	0.72694	0
0.25066	-1.60547	0.72694	0
0.25066	-1.60547	0.72694	0
0.25066	-1.60547	0.72694	0
0.25066	-1.60547	0.72694	0
0.25066	-1.60547	0.72694	0
0.25066	-1.60547	0.72694	0
0.25066	-1.60547	0.72694	0
0.25066	-1.60547	0.72694	0
0.25066	-1.60547	0.72694	0
0.25066	-1.60547	0.72694	0
0.25066	-1.60547	0.72694	0
0.25066	-1.60547	0.72694	0
0.25066	-1.60547	0.72694	0
0.77859	-1.39959	0.99459	1
0.77859	-1.39959	0.99459	1
0.77859	-1.39959	0.99459	1
0.77859	-1.39959	0.99459	1

0.77859	-1.39959	0.99459	1
0.77859	-1.39959	0.99459	1
0.77859	-1.39959	0.99459	1
0.77859	-1.39959	0.99459	1
0.77859	-1.39959	0.99459	1
0.77859	-1.39959	0.99459	1
0.77859	-1.39959	0.99459	1
0.77859	-1.39959	0.99459	1
0.77859	-1.39959	0.99459	1
0.77859	-1.39959	0.99459	1
0.77859	-1.39959	0.99459	1
0.77859	-1.39959	0.99459	1
0.77859	-1.39959	0.99459	1
0.77859	-1.39959	0.99459	1
0.77859	-1.39959	0.99459	1
0.77859	-1.39959	0.99459	1
0.77859	-1.39959	0.99459	1
0.77859	-1.39959	0.99459	1
0.77859	-1.39959	0.99459	1
0.77859	-1.39959	0.99459	1
0.77859	-1.39959	0.99459	1
0.77859	-1.39959	0.99459	1
0.77859	-1.39959	0.99459	1
0.77859	-1.39959	0.99459	1
0.77859	-1.39959	0.99459	1
0.77859	-1.39959	0.99459	1
0.77859	-1.39959	0.99459	1
0.77859	-1.39959	0.99459	1
0.77859	-1.39959	0.99459	1
0.77859	-1.39959	0.99459	1
0.77859	-1.39959	0.99459	1
0.77859	-1.39959	0.99459	1
0.77859	-1.39959	0.99459	1
0.77859	-1.39959	0.99459	1
0.77859	-1.39959	0.99459	1
0.77859	-1.39959	0.99459	1
0.77859	-1.39959	0.99459	1
0.77859	-1.39959	0.99459	1
0.77859	-1.39959	0.99459	1
0.77859	-1.39959	0.99459	1
0.77859	-1.39959	0.99459	1
0.77859	-1.39959	0.99459	1
0.77859	-1.39959	0.99459	1
0.77859	-1.39959	0.99459	1
0.77859	-1.39959	0.99459	1
0.77859	-1.39959	0.99459	1
0.77859	-1.39959	0.99459	1
0.77859	-1.39959	0.99459	1
0.77859	-1.39959	0.99459	1
0.77859	-1.39959	0.99459	1
0.77859	-1.39959	0.99459	1
0.77859	-1.39959	0.99459	1
0.77859	-1.39959	0.99459	1
0.77859	-1.39959	0.99459	1
0.77859	-1.39959	0.99459	1
0.77859	-1.39959	0.99459	1

0.77859	-1.39959	0.99459	1
0.77859	-1.39959	0.99459	1
0.77859	-1.39959	0.99459	1
0.77859	-1.39959	0.99459	1
0.77859	-1.39959	0.99459	1
0.77859	-1.39959	0.99459	1
0.77859	-1.39959	0.99459	1
0.77859	-1.39959	0.99459	1
0.77859	-1.39959	0.99459	1
0.77859	-1.39959	0.99459	1
0.77859	-1.39959	0.99459	1
0.77859	-1.39959	0.99459	1
0.77859	-1.39959	0.99459	1
0.77859	-1.39959	0.99459	1
0.77859	-1.39959	0.99459	1
0.77859	-1.39959	0.99459	1
0.77859	-1.39959	0.99459	1
0.77859	-1.39959	0.99459	1
0.77859	-1.39959	0.99459	1
0.77859	-1.39959	0.99459	1
0.77859	-1.39959	0.99459	1
0.77859	-1.39959	0.99459	1
0.77859	-1.39959	0.99459	1
0.77859	-1.39959	0.99459	1
0.77859	-1.39959	0.99459	1
0.77859	-1.39959	0.99459	1
0.77859	-1.39959	0.99459	1
0.77859	-1.39959	0.99459	1
0.77859	-1.39959	0.99459	1
0.77859	-1.39959	0.99459	1
0.77859	-1.39959	0.99459	1
0.77859	-1.39959	0.99459	1
0.77859	-1.39959	0.99459	1
0.77859	-1.39959	0.99459	1
0.77859	-1.39959	0.99459	1
0.77859	-1.39959	0.99459	1
0.77859	-1.39959	0.99459	1
0.77859	-1.39959	0.99459	1
0.77859	-1.39959	0.99459	1
0.77859	-1.39959	0.99459	1
0.77859	-1.39959	0.99459	1
0.77859	-1.39959	0.99459	1
0.77859	-1.39959	0.99459	1
0.77859	-1.39959	0.99459	1
0.77859	-1.39959	0.99459	1
0.77859	-1.39959	0.99459	1
0.77859	-1.39959	0.99459	1
0.77859	-1.39959	0.99459	1
0.77859	-1.39959	0.99459	1
0.77859	-1.39959	0.99459	1
0.77859	-1.39959	0.99459	1
0.77859	-1.39959	0.99459	1
0.77859	-1.39959	0.99459	1
0.77859	-1.39959	0.99459	1
0.77859	-1.39959	0.99459	1
0.77859	-1.39959	0.99459	1

0.77859	-1.39959	0.99459	1
0.77859	-1.39959	0.99459	1
0.77859	-1.39959	0.99459	1
0.77859	-1.39959	0.99459	1
0.41597	-1.78733	1.09133	1
0.41597	-1.78733	1.09133	1
0.41597	-1.78733	1.09133	1
0.41597	-1.78733	1.09133	1
0.41597	-1.78733	1.09133	1
0.41597	-1.78733	1.09133	1
0.41597	-1.78733	1.09133	1
0.41597	-1.78733	1.09133	1
0.41597	-1.78733	1.09133	1
0.41597	-1.78733	1.09133	1
0.41597	-1.78733	1.09133	1
0.41597	-1.78733	1.09133	1
0.41597	-1.78733	1.09133	1
0.41597	-1.78733	1.09133	1
0.41597	-1.78733	1.09133	1
0.41597	-1.78733	1.09133	1
0.41597	-1.78733	1.09133	1
0.41597	-1.78733	1.09133	1
0.41597	-1.78733	1.09133	1
0.41597	-1.78733	1.09133	1
0.41597	-1.78733	1.09133	1
0.41597	-1.78733	1.09133	1
0.41597	-1.78733	1.09133	1
0.41597	-1.78733	1.09133	1
0.41597	-1.78733	1.09133	1
0.41597	-1.78733	1.09133	1
0.41597	-1.78733	1.09133	1
0.41597	-1.78733	1.09133	1
0.41597	-1.78733	1.09133	1
0.41597	-1.78733	1.09133	1
0.41597	-1.78733	1.09133	1
0.41597	-1.78733	1.09133	1
0.41597	-1.78733	1.09133	1
0.41597	-1.78733	1.09133	1
0.41597	-1.78733	1.09133	1
0.41597	-1.78733	1.09133	1
0.41597	-1.78733	1.09133	1
0.41597	-1.78733	1.09133	1
0.41597	-1.78733	1.09133	1
0.41597	-1.78733	1.09133	1
0.41597	-1.78733	1.09133	1
0.41597	-1.78733	1.09133	1
0.41597	-1.78733	1.09133	1
0.41597	-1.78733	1.09133	1
0.41597	-1.78733	1.09133	1
0.41597	-1.78733	1.09133	1
0.41597	-1.78733	1.09133	1
0.41597	-1.78733	1.09133	1
0.41597	-1.78733	1.09133	1
0.41597	-1.78733	1.09133	1
0.41597	-1.78733	1.09133	1
0.41597	-1.78733	1.09133	1

0.41597	-1.78733	1.09133	1
0.41597	-1.78733	1.09133	1
0.41597	-1.78733	1.09133	1
0.41597	-1.78733	1.09133	1
0.41597	-1.78733	1.09133	1
0.41597	-1.78733	1.09133	1
0.41597	-1.78733	1.09133	1
0.41597	-1.78733	1.09133	1
0.41597	-1.78733	1.09133	1
0.41597	-1.78733	1.09133	1
0.41597	-1.78733	1.09133	1
0.41597	-1.78733	1.09133	1
0.55157	-1.10415	0.92087	1
0.55157	-1.10415	0.92087	1
0.55157	-1.10415	0.92087	1
0.55157	-1.10415	0.92087	1
0.55157	-1.10415	0.92087	1
0.55157	-1.10415	0.92087	1
0.55157	-1.10415	0.92087	1
0.55157	-1.10415	0.92087	1
0.55157	-1.10415	0.92087	1
0.55157	-1.10415	0.92087	1
0.55157	-1.10415	0.92087	1
0.55157	-1.10415	0.92087	1
0.55157	-1.10415	0.92087	1
0.55157	-1.10415	0.92087	1
0.55157	-1.10415	0.92087	1
0.55157	-1.10415	0.92087	1
0.55157	-1.10415	0.92087	1
0.55157	-1.10415	0.92087	1
0.55157	-1.10415	0.92087	1
0.55157	-1.10415	0.92087	1
0.55157	-1.10415	0.92087	1
0.55157	-1.10415	0.92087	1
0.55157	-1.10415	0.92087	1
0.55157	-1.10415	0.92087	1
0.55157	-1.10415	0.92087	1
0.55157	-1.10415	0.92087	1
0.55157	-1.10415	0.92087	1
0.55157	-1.10415	0.92087	1
0.55157	-1.10415	0.92087	1
0.55157	-1.10415	0.92087	1
0.55157	-1.10415	0.92087	1
0.55157	-1.10415	0.92087	1
0.55157	-1.10415	0.92087	1
0.55157	-1.10415	0.92087	1
0.55157	-1.10415	0.92087	1
0.55157	-1.10415	0.92087	1
0.55157	-1.10415	0.92087	1
0.55157	-1.10415	0.92087	1
0.55157	-1.10415	0.92087	1
0.55157	-1.10415	0.92087	1
0.55157	-1.10415	0.92087	1
0.55157	-1.10415	0.92087	1
0.55157	-1.10415	0.92087	1
0.55157	-1.10415	0.92087	1

0.55157	-1.10415	0.92087	1
0.55157	-1.10415	0.92087	1
0.55157	-1.10415	0.92087	1
0.55157	-1.10415	0.92087	1
0.55157	-1.10415	0.92087	1
0.55157	-1.10415	0.92087	1
0.55157	-1.10415	0.92087	1
0.55157	-1.10415	0.92087	1
0.55157	-1.10415	0.92087	1
0.55157	-1.10415	0.92087	1
0.55157	-1.10415	0.92087	1
0.55157	-1.10415	0.92087	1
0.55157	-1.10415	0.92087	1
0.55157	-1.10415	0.92087	1
0.55157	-1.10415	0.92087	1
0.55157	-1.10415	0.92087	1
0.55157	-1.10415	0.92087	1
0.55157	-1.10415	0.92087	1
0.55157	-1.10415	0.92087	1
0.55157	-1.10415	0.92087	1
0.41597	-1.78733	1.09133	1
0.41597	-1.78733	1.09133	1
0.41597	-1.78733	1.09133	1
0.41597	-1.78733	1.09133	1
0.41597	-1.78733	1.09133	1
0.41597	-1.78733	1.09133	1
0.41597	-1.78733	1.09133	1
0.41597	-1.78733	1.09133	1
0.41597	-1.78733	1.09133	1
0.41597	-1.78733	1.09133	1
0.41597	-1.78733	1.09133	1
0.41597	-1.78733	1.09133	1
0.41597	-1.78733	1.09133	1
0.41597	-1.78733	1.09133	1
0.41597	-1.78733	1.09133	1
0.41597	-1.78733	1.09133	1
0.41597	-1.78733	1.09133	1
0.41597	-1.78733	1.09133	1
0.41597	-1.78733	1.09133	1
0.41597	-1.78733	1.09133	1
0.41597	-1.78733	1.09133	1
0.41597	-1.78733	1.09133	1
0.41597	-1.78733	1.09133	1
0.41597	-1.78733	1.09133	1
0.41597	-1.78733	1.09133	1
0.41597	-1.78733	1.09133	1
0.41597	-1.78733	1.09133	1
0.41597	-1.78733	1.09133	1
0.41597	-1.78733	1.09133	1
0.41597	-1.78733	1.09133	1
0.41597	-1.78733	1.09133	1
0.41597	-1.78733	1.09133	1
0.41597	-1.78733	1.09133	1
0.41597	-1.78733	1.09133	1
0.41597	-1.78733	1.09133	1
0.41597	-1.78733	1.09133	1

0.41597	-1.78733	1.09133	1
0.41597	-1.78733	1.09133	1
0.41597	-1.78733	1.09133	1
0.41597	-1.78733	1.09133	1
0.41597	-1.78733	1.09133	1
0.41597	-1.78733	1.09133	1
0.41597	-1.78733	1.09133	1
0.41597	-1.78733	1.09133	1
0.41597	-1.78733	1.09133	1
0.41597	-1.78733	1.09133	1
0.41597	-1.78733	1.09133	1
0.41597	-1.78733	1.09133	1
0.41597	-1.78733	1.09133	1
0.41597	-1.78733	1.09133	1
0.41597	-1.78733	1.09133	1
0.41597	-1.78733	1.09133	1
0.41597	-1.78733	1.09133	1
0.41597	-1.78733	1.09133	1
0.41597	-1.78733	1.09133	1
0.41597	-1.78733	1.09133	1
0.41597	-1.78733	1.09133	1
0.41597	-1.78733	1.09133	1
0.41597	-1.78733	1.09133	1
0.41597	-1.78733	1.09133	1
0.41597	-1.78733	1.09133	1
0.41597	-1.78733	1.09133	1
0.41597	-1.78733	1.09133	1
0.41597	-1.78733	1.09133	1
0.41597	-1.78733	1.09133	1
0.41597	-1.78733	1.09133	1
0.41597	-1.78733	1.09133	1
0.41597	-1.78733	1.09133	1
0.41597	-1.78733	1.09133	1
0.41597	-1.78733	1.09133	1
0.41597	-1.78733	1.09133	1
0.41597	-1.78733	1.09133	1
0.41597	-1.78733	1.09133	1
0.41597	-1.78733	1.09133	1
0.41597	-1.78733	1.09133	1
0.41597	-1.78733	1.09133	1
0.41597	-1.78733	1.09133	1
0.41597	-1.78733	1.09133	1
0.41597	-1.78733	1.09133	1
0.41597	-1.78733	1.09133	1
0.41597	-1.78733	1.09133	1
0.41597	-1.78733	1.09133	1
0.41597	-1.78733	1.09133	1
0.41597	-1.78733	1.09133	1
0.41597	-1.78733	1.09133	1
0.41597	-1.78733	1.09133	1
0.41597	-1.78733	1.09133	1
0.41597	-1.78733	1.09133	1
0.41597	-1.78733	1.09133	1
0.41597	-1.78733	1.09133	1
0.41597	-1.78733	1.09133	1
0.41597	-1.78733	1.09133	1

0.41597	-1.78733	1.09133	1
0.41597	-1.78733	1.09133	1
0.41597	-1.78733	1.09133	1
0.41597	-1.78733	1.09133	1
0.41597	-1.78733	1.09133	1
0.41597	-1.78733	1.09133	1
0.41597	-1.78733	1.09133	1
0.41597	-1.78733	1.09133	1
0.41597	-1.78733	1.09133	1
0.41597	-1.78733	1.09133	1
0.41597	-1.78733	1.09133	1
0.41597	-1.78733	1.09133	1
0.41597	-1.78733	1.09133	1
0.41597	-1.78733	1.09133	1
0.41597	-1.78733	1.09133	1
0.41597	-1.78733	1.09133	1
0.41597	-1.78733	1.09133	1
0.41597	-1.78733	1.09133	1
0.41597	-1.78733	1.09133	1
0.41597	-1.78733	1.09133	1
0.41597	-1.78733	1.09133	1
0.41597	-1.78733	1.09133	1
0.41597	-1.78733	1.09133	1
0.41597	-1.78733	1.09133	1
0.41597	-1.78733	1.09133	1
0.41597	-1.78733	1.09133	1
0.41597	-1.78733	1.09133	1
0.41597	-1.78733	1.09133	1
0.41597	-1.78733	1.09133	1
0.41597	-1.78733	1.09133	1
0.41597	-1.78733	1.09133	1
0.41597	-1.78733	1.09133	1
0.41597	-1.78733	1.09133	1
0.41597	-1.78733	1.09133	1
0.41597	-1.78733	1.09133	1
0.41597	-1.78733	1.09133	1
0.41597	-1.78733	1.09133	1
0.41597	-1.78733	1.09133	1
0.41597	-1.78733	1.09133	1
0.41597	-1.78733	1.09133	1
0.41597	-1.78733	1.09133	1
0.41597	-1.78733	1.09133	1
0.41597	-1.78733	1.09133	1
0.41597	-1.78733	1.09133	1
0.41597	-1.78733	1.09133	1
0.41597	-1.78733	1.09133	1
0.41597	-1.78733	1.09133	1
0.41597	-1.78733	1.09133	1
0.41597	-1.78733	1.09133	1
0.41597	-1.78733	1.09133	1
0.41597	-1.78733	1.09133	1
0.41597	-1.78733	1.09133	1
0.41597	-1.78733	1.09133	1
0.41597	-1.78733	1.09133	1
0.41597	-1.78733	1.09133	1
0.41597	-1.78733	1.09133	1

0.41597	-1.78733	1.09133	1
0.41597	-1.78733	1.09133	1
0.41597	-1.78733	1.09133	1
0.41597	-1.78733	1.09133	1
0.41597	-1.78733	1.09133	1
0.41597	-1.78733	1.09133	1
0.41597	-1.78733	1.09133	1
0.41597	-1.78733	1.09133	1
0.41597	-1.78733	1.09133	1
0.41597	-1.78733	1.09133	1
0.41597	-1.78733	1.09133	1
0.41597	-1.78733	1.09133	1
0.41597	-1.78733	1.09133	1
0.41597	-1.78733	1.09133	1
0.41597	-1.78733	1.09133	1
0.41597	-1.78733	1.09133	1
0.41597	-1.78733	1.09133	1
0.41597	-1.78733	1.09133	1
0.41597	-1.78733	1.09133	1
0.41597	-1.78733	1.09133	1
0.41597	-1.78733	1.09133	1
0.41597	-1.78733	1.09133	1
0.41597	-1.78733	1.09133	1
0.41597	-1.78733	1.09133	1
0.41597	-1.78733	1.09133	1
0.41597	-1.78733	1.09133	1
0.41597	-1.78733	1.09133	1
0.41597	-1.78733	1.09133	1
0.41597	-1.78733	1.09133	1
0.41597	-1.78733	1.09133	1
0.41597	-1.78733	1.09133	1
0.41597	-1.78733	1.09133	1
0.41597	-1.78733	1.09133	1
0.41597	-1.78733	1.09133	1
0.41597	-1.78733	1.09133	1
0.41597	-1.78733	1.09133	1
0.41597	-1.78733	1.09133	1
0.41597	-1.78733	1.09133	1
0.41597	-1.78733	1.09133	1
0.41597	-1.78733	1.09133	1
0.41597	-1.78733	1.09133	1
0.41597	-1.78733	1.09133	1
0.41597	-1.78733	1.09133	1
0.41597	-1.78733	1.09133	1
0.41597	-1.78733	1.09133	1
0.41597	-1.78733	1.09133	1
0.41597	-1.78733	1.09133	1
0.41597	-1.78733	1.09133	1
0.41597	-1.78733	1.09133	1
0.41597	-1.78733	1.09133	1
0.41597	-1.78733	1.09133	1
0.41597	-1.78733	1.09133	1
0.41597	-1.78733	1.09133	1
0.41597	-1.78733	1.09133	1
0.41597	-1.78733	1.09133	1
0.41597	-1.78733	1.09133	1

0.41597	-1.78733	1.09133	1
0.41597	-1.78733	1.09133	1
0.41597	-1.78733	1.09133	1
0.41597	-1.78733	1.09133	1
0.41597	-1.78733	1.09133	1
0.41597	-1.78733	1.09133	1
0.41597	-1.78733	1.09133	1
0.41597	-1.78733	1.09133	1
0.41597	-1.78733	1.09133	1
0.41597	-1.78733	1.09133	1
0.41597	-1.78733	1.09133	1
0.41597	-1.78733	1.09133	1
0.41597	-1.78733	1.09133	1
0.41597	-1.78733	1.09133	1
0.41597	-1.78733	1.09133	1
0.41597	-1.78733	1.09133	1
0.41597	-1.78733	1.09133	1
0.41597	-1.78733	1.09133	1
0.41597	-1.78733	1.09133	1
0.41597	-1.78733	1.09133	1
0.41597	-1.78733	1.09133	1
0.41597	-1.78733	1.09133	1
0.41597	-1.78733	1.09133	1
0.41597	-1.78733	1.09133	1
0.41597	-1.78733	1.09133	1
0.41597	-1.78733	1.09133	1
0.41597	-1.78733	1.09133	1
0.41597	-1.78733	1.09133	1
0.41597	-1.78733	1.09133	1
0.41597	-1.78733	1.09133	1
0.41597	-1.78733	1.09133	1
0.41597	-1.78733	1.09133	1
0.41597	-1.78733	1.09133	1
0.41597	-1.78733	1.09133	1
0.41597	-1.78733	1.09133	1
0.41597	-1.78733	1.09133	1
0.41597	-1.78733	1.09133	1
0.41597	-1.78733	1.09133	1
0.41597	-1.78733	1.09133	1
0.41597	-1.78733	1.09133	1
0.41597	-1.78733	1.09133	1
0.41597	-1.78733	1.09133	1
0.41597	-1.78733	1.09133	1
0.41597	-1.78733	1.09133	1
0.41597	-1.78733	1.09133	1
0.41597	-1.78733	1.09133	1
0.41597	-1.78733	1.09133	1
0.41597	-1.78733	1.09133	1
0.41597	-1.78733	1.09133	1
0.41597	-1.78733	1.09133	1
0.41597	-1.78733	1.09133	1
0.41597	-1.78733	1.09133	1
0.41597	-1.78733	1.09133	1
0.41597	-1.78733	1.09133	1
0.41597	-1.78733	1.09133	1
0.41597	-1.78733	1.09133	1

0.41597	-1.78733	1.09133	1
0.41597	-1.78733	1.09133	1
0.41597	-1.78733	1.09133	1
0.41597	-1.78733	1.09133	1
0.41597	-1.78733	1.09133	1
0.41597	-1.78733	1.09133	1
0.41597	-1.78733	1.09133	1
0.41597	-1.78733	1.09133	1
0.41597	-1.78733	1.09133	1
0.41597	-1.78733	1.09133	1
0.41597	-1.78733	1.09133	1
0.41597	-1.78733	1.09133	1
0.41597	-1.78733	1.09133	1
0.41597	-1.78733	1.09133	1
0.41597	-1.78733	1.09133	1
0.41597	-1.78733	1.09133	1
0.41597	-1.78733	1.09133	1
0.41597	-1.78733	1.09133	1
0.41597	-1.78733	1.09133	1
0.41597	-1.78733	1.09133	1
0.41597	-1.78733	1.09133	1
0.41597	-1.78733	1.09133	1
0.41597	-1.78733	1.09133	1
0.41597	-1.78733	1.09133	1
0.41597	-1.78733	1.09133	1
0.41597	-1.78733	1.09133	1
0.41597	-1.78733	1.09133	1
0.41597	-1.78733	1.09133	1
0.41597	-1.78733	1.09133	1
0.41597	-1.78733	1.09133	1
0.41597	-1.78733	1.09133	1
0.41597	-1.78733	1.09133	1
0.41597	-1.78733	1.09133	1
0.41597	-1.78733	1.09133	1
0.41597	-1.78733	1.09133	1
0.41597	-1.78733	1.09133	1
0.41597	-1.78733	1.09133	1
0.41597	-1.78733	1.09133	1
0.41597	-1.78733	1.09133	1
0.41597	-1.78733	1.09133	1
0.41597	-1.78733	1.09133	1
0.41597	-1.78733	1.09133	1
0.41597	-1.78733	1.09133	1
0.41597	-1.78733	1.09133	1
0.41597	-1.78733	1.09133	1
0.41597	-1.78733	1.09133	1
0.41597	-1.78733	1.09133	1
0.41597	-1.78733	1.09133	1
0.41597	-1.78733	1.09133	1
0.41597	-1.78733	1.09133	1
0.41597	-1.78733	1.09133	1
0.41597	-1.78733	1.09133	1
0.41597	-1.78733	1.09133	1
0.41597	-1.78733	1.09133	1
0.41597	-1.78733	1.09133	1
0.41597	-1.78733	1.09133	1

0.41597	-1.78733	1.09133	1
0.41597	-1.78733	1.09133	1
0.41597	-1.78733	1.09133	1
0.41597	-1.78733	1.09133	1
0.41597	-1.78733	1.09133	1
0.41597	-1.78733	1.09133	1
0.41597	-1.78733	1.09133	1
0.41597	-1.78733	1.09133	1
0.41597	-1.78733	1.09133	1
0.41597	-1.78733	1.09133	1
0.41597	-1.78733	1.09133	1
0.41597	-1.78733	1.09133	1
0.41597	-1.78733	1.09133	1
0.41597	-1.78733	1.09133	1
0.41597	-1.78733	1.09133	1
0.41597	-1.78733	1.09133	1
0.41597	-1.78733	1.09133	1
0.41597	-1.78733	1.09133	1
0.41597	-1.78733	1.09133	1
0.41597	-1.78733	1.09133	1
0.41597	-1.78733	1.09133	1
0.41597	-1.78733	1.09133	1
0.41597	-1.78733	1.09133	1
0.41597	-1.78733	1.09133	1
0.41597	-1.78733	1.09133	1
0.41597	-1.78733	1.09133	1
0.41597	-1.78733	1.09133	1
0.41597	-1.78733	1.09133	1
0.41597	-1.78733	1.09133	1
0.41597	-1.78733	1.09133	1
0.41597	-1.78733	1.09133	1
0.41597	-1.78733	1.09133	1
0.41597	-1.78733	1.09133	1
0.41597	-1.78733	1.09133	1
0.41597	-1.78733	1.09133	1
0.41597	-1.78733	1.09133	1
0.41597	-1.78733	1.09133	1
0.41597	-1.78733	1.09133	1
0.41597	-1.78733	1.09133	1
0.41597	-1.78733	1.09133	1
0.41597	-1.78733	1.09133	1
0.41597	-1.78733	1.09133	1
0.41597	-1.78733	1.09133	1
0.41597	-1.78733	1.09133	1
0.41597	-1.78733	1.09133	1
0.41597	-1.78733	1.09133	1
0.41597	-1.78733	1.09133	1
0.41597	-1.78733	1.09133	1
0.41597	-1.78733	1.09133	1
0.41597	-1.78733	1.09133	1
0.41597	-1.78733	1.09133	1
0.41597	-1.78733	1.09133	1
0.41597	-1.78733	1.09133	1
0.41597	-1.78733	1.09133	1
0.41597	-1.78733	1.09133	1
0.41597	-1.78733	1.09133	1

0.41597	-1.78733	1.09133	1
0.41597	-1.78733	1.09133	1
0.41597	-1.78733	1.09133	1
0.41597	-1.78733	1.09133	1
0.41597	-1.78733	1.09133	1
0.41597	-1.78733	1.09133	1
0.41597	-1.78733	1.09133	1
0.41597	-1.78733	1.09133	1
0.41597	-1.78733	1.09133	1
0.41597	-1.78733	1.09133	1
0.41597	-1.78733	1.09133	1
0.41597	-1.78733	1.09133	1
0.41597	-1.78733	1.09133	1
0.41597	-1.78733	1.09133	1
0.41597	-1.78733	1.09133	1
0.41597	-1.78733	1.09133	1
0.41597	-1.78733	1.09133	1
0.41597	-1.78733	1.09133	1
0.41597	-1.78733	1.09133	1
0.41597	-1.78733	1.09133	1
0.41597	-1.78733	1.09133	1
0.41597	-1.78733	1.09133	1
0.41597	-1.78733	1.09133	1
0.41597	-1.78733	1.09133	1
0.41597	-1.78733	1.09133	1
0.41597	-1.78733	1.09133	1
0.41597	-1.78733	1.09133	1
0.41597	-1.78733	1.09133	1
0.41597	-1.78733	1.09133	1
0.41597	-1.78733	1.09133	1
0.41597	-1.78733	1.09133	1
0.41597	-1.78733	1.09133	1
0.41597	-1.78733	1.09133	1
0.41597	-1.78733	1.09133	1
0.41597	-1.78733	1.09133	1
0.41597	-1.78733	1.09133	1
0.41597	-1.78733	1.09133	1
0.41597	-1.78733	1.09133	1
0.41597	-1.78733	1.09133	1
0.41597	-1.78733	1.09133	1
0.41597	-1.78733	1.09133	1
0.41597	-1.78733	1.09133	1
0.41597	-1.78733	1.09133	1
0.41597	-1.78733	1.09133	1
0.41597	-1.78733	1.09133	1
0.41597	-1.78733	1.09133	1
0.41597	-1.78733	1.09133	1
0.41597	-1.78733	1.09133	1
0.41597	-1.78733	1.09133	1
0.41597	-1.78733	1.09133	1
0.41597	-1.78733	1.09133	1
0.41597	-1.78733	1.09133	1
0.41597	-1.78733	1.09133	1
0.41597	-1.78733	1.09133	1
0.41597	-1.78733	1.09133	1
0.41597	-1.78733	1.09133	1

0.41597	-1.78733	1.09133	1
0.41597	-1.78733	1.09133	1
0.41597	-1.78733	1.09133	1
0.41597	-1.78733	1.09133	1
0.41597	-1.78733	1.09133	1
0.41597	-1.78733	1.09133	1
0.41597	-1.78733	1.09133	1
0.41597	-1.78733	1.09133	1
0.41597	-1.78733	1.09133	1
0.41597	-1.78733	1.09133	1
0.41597	-1.78733	1.09133	1
0.41597	-1.78733	1.09133	1
0.41597	-1.78733	1.09133	1
0.41597	-1.78733	1.09133	1
0.41597	-1.78733	1.09133	1
0.41597	-1.78733	1.09133	1
0.41597	-1.78733	1.09133	1
0.41597	-1.78733	1.09133	1
0.41597	-1.78733	1.09133	1
0.41597	-1.78733	1.09133	1
0.41597	-1.78733	1.09133	1
0.41597	-1.78733	1.09133	1
0.41597	-1.78733	1.09133	1
0.41597	-1.78733	1.09133	1
0.41597	-1.78733	1.09133	1
0.41597	-1.78733	1.09133	1
0.41597	-1.78733	1.09133	1
0.41597	-1.78733	1.09133	1
0.41597	-1.78733	1.09133	1
0.41597	-1.78733	1.09133	1
0.41597	-1.78733	1.09133	1
0.41597	-1.78733	1.09133	1
0.41597	-1.78733	1.09133	1
0.41597	-1.78733	1.09133	1
0.41597	-1.78733	1.09133	1
0.41597	-1.78733	1.09133	1
0.41597	-1.78733	1.09133	1
0.41597	-1.78733	1.09133	1
0.41597	-1.78733	1.09133	1
0.41597	-1.78733	1.09133	1
0.41597	-1.78733	1.09133	1
0.41597	-1.78733	1.09133	1
0.41597	-1.78733	1.09133	1
0.41597	-1.78733	1.09133	1
0.41597	-1.78733	1.09133	1
0.41597	-1.78733	1.09133	1
0.41597	-1.78733	1.09133	1
0.41597	-1.78733	1.09133	1
0.41597	-1.78733	1.09133	1
0.41597	-1.78733	1.09133	1
0.41597	-1.78733	1.09133	1
0.41597	-1.78733	1.09133	1
0.41597	-1.78733	1.09133	1
0.41597	-1.78733	1.09133	1
0.41597	-1.78733	1.09133	1
0.41597	-1.78733	1.09133	1

0.41597	-1.78733	1.09133	1
0.41597	-1.78733	1.09133	1
0.41597	-1.78733	1.09133	1
0.41597	-1.78733	1.09133	1
0.41597	-1.78733	1.09133	1
0.41597	-1.78733	1.09133	1
0.41597	-1.78733	1.09133	1
0.41597	-1.78733	1.09133	1
0.41597	-1.78733	1.09133	1
0.41597	-1.78733	1.09133	1
0.41597	-1.78733	1.09133	1
0.41597	-1.78733	1.09133	1
0.41597	-1.78733	1.09133	1
0.41597	-1.78733	1.09133	1
0.41597	-1.78733	1.09133	1
0.41597	-1.78733	1.09133	1
0.41597	-1.78733	1.09133	1
0.41597	-1.78733	1.09133	1
0.41597	-1.78733	1.09133	1
0.41597	-1.78733	1.09133	1
0.41597	-1.78733	1.09133	1
0.41597	-1.78733	1.09133	1
0.41597	-1.78733	1.09133	1
0.41597	-1.78733	1.09133	1
0.41597	-1.78733	1.09133	1
0.41597	-1.78733	1.09133	1
0.41597	-1.78733	1.09133	1
0.41597	-1.78733	1.09133	1
0.41597	-1.78733	1.09133	1
0.41597	-1.78733	1.09133	1
0.41597	-1.78733	1.09133	1
0.41597	-1.78733	1.09133	1
0.41597	-1.78733	1.09133	1
0.41597	-1.78733	1.09133	1
0.41597	-1.78733	1.09133	1
0.41597	-1.78733	1.09133	1
0.41597	-1.78733	1.09133	1
0.41597	-1.78733	1.09133	1
0.41597	-1.78733	1.09133	1
0.41597	-1.78733	1.09133	1
0.41597	-1.78733	1.09133	1
0.41597	-1.78733	1.09133	1
0.41597	-1.78733	1.09133	1
0.41597	-1.78733	1.09133	1
0.41597	-1.78733	1.09133	1
0.41597	-1.78733	1.09133	1
0.41597	-1.78733	1.09133	1
0.41597	-1.78733	1.09133	1
0.41597	-1.78733	1.09133	1
0.41597	-1.78733	1.09133	1
0.41597	-1.78733	1.09133	1
0.41597	-1.78733	1.09133	1
0.41597	-1.78733	1.09133	1
0.41597	-1.78733	1.09133	1
0.41597	-1.78733	1.09133	1
0.41597	-1.78733	1.09133	1

0.41597	-1.78733	1.09133	1
0.41597	-1.78733	1.09133	1
0.41597	-1.78733	1.09133	1
0.41597	-1.78733	1.09133	1
0.41597	-1.78733	1.09133	1
0.41597	-1.78733	1.09133	1
0.41597	-1.78733	1.09133	1
0.41597	-1.78733	1.09133	1
0.41597	-1.78733	1.09133	1
0.41597	-1.78733	1.09133	1
0.41597	-1.78733	1.09133	1
0.41597	-1.78733	1.09133	1
0.41597	-1.78733	1.09133	1
0.41597	-1.78733	1.09133	1
0.41597	-1.78733	1.09133	1
0.41597	-1.78733	1.09133	1
0.41597	-1.78733	1.09133	1
0.41597	-1.78733	1.09133	1
0.41597	-1.78733	1.09133	1
0.41597	-1.78733	1.09133	1
0.41597	-1.78733	1.09133	1
0.41597	-1.78733	1.09133	1
0.41597	-1.78733	1.09133	1
0.41597	-1.78733	1.09133	1
0.41597	-1.78733	1.09133	1
0.41597	-1.78733	1.09133	1
0.41597	-1.78733	1.09133	1
0.41597	-1.78733	1.09133	1
0.41597	-1.78733	1.09133	1
0.41597	-1.78733	1.09133	1
0.41597	-1.78733	1.09133	1
0.41597	-1.78733	1.09133	1
0.41597	-1.78733	1.09133	1
0.41597	-1.78733	1.09133	1
0.41597	-1.78733	1.09133	1
0.41597	-1.78733	1.09133	1
0.41597	-1.78733	1.09133	1
0.41597	-1.78733	1.09133	1
0.41597	-1.78733	1.09133	1
0.41597	-1.78733	1.09133	1
0.41597	-1.78733	1.09133	1
0.41597	-1.78733	1.09133	1
0.41597	-1.78733	1.09133	1
0.41597	-1.78733	1.09133	1
0.41597	-1.78733	1.09133	1
0.41597	-1.78733	1.09133	1
0.41597	-1.78733	1.09133	1
0.41597	-1.78733	1.09133	1
0.41597	-1.78733	1.09133	1
0.41597	-1.78733	1.09133	1
0.41597	-1.78733	1.09133	1
0.41597	-1.78733	1.09133	1
0.41597	-1.78733	1.09133	1
0.41597	-1.78733	1.09133	1
0.41597	-1.78733	1.09133	1
0.41597	-1.78733	1.09133	1

0.41597	-1.78733	1.09133	1
0.41597	-1.78733	1.09133	1
0.41597	-1.78733	1.09133	1
0.41597	-1.78733	1.09133	1
0.41597	-1.78733	1.09133	1
0.41597	-1.78733	1.09133	1
0.41597	-1.78733	1.09133	1
0.41597	-1.78733	1.09133	1
0.41597	-1.78733	1.09133	1
0.41597	-1.78733	1.09133	1
0.41597	-1.78733	1.09133	1
0.41597	-1.78733	1.09133	1
0.41597	-1.78733	1.09133	1
0.41597	-1.78733	1.09133	1
0.41597	-1.78733	1.09133	1
0.41597	-1.78733	1.09133	1
0.41597	-1.78733	1.09133	1
0.41597	-1.78733	1.09133	1
0.41597	-1.78733	1.09133	1
0.41597	-1.78733	1.09133	1
0.41597	-1.78733	1.09133	1
0.41597	-1.78733	1.09133	1
0.41597	-1.78733	1.09133	1
0.41597	-1.78733	1.09133	1
0.41597	-1.78733	1.09133	1
0.41597	-1.78733	1.09133	1
0.41597	-1.78733	1.09133	1
0.41597	-1.78733	1.09133	1
0.41597	-1.78733	1.09133	1
0.41597	-1.78733	1.09133	1
0.41597	-1.78733	1.09133	1
0.41597	-1.78733	1.09133	1
0.41597	-1.78733	1.09133	1
0.41597	-1.78733	1.09133	1
0.41597	-1.78733	1.09133	1
0.41597	-1.78733	1.09133	1
0.41597	-1.78733	1.09133	1
0.41597	-1.78733	1.09133	1
0.41597	-1.78733	1.09133	1
0.41597	-1.78733	1.09133	1
0.41597	-1.78733	1.09133	1
0.41597	-1.78733	1.09133	1
0.41597	-1.78733	1.09133	1
0.41597	-1.78733	1.09133	1
0.41597	-1.78733	1.09133	1
0.41597	-1.78733	1.09133	1
0.41597	-1.78733	1.09133	1
0.41597	-1.78733	1.09133	1
0.41597	-1.78733	1.09133	1
0.41597	-1.78733	1.09133	1
0.41597	-1.78733	1.09133	1
0.41597	-1.78733	1.09133	1
0.41597	-1.78733	1.09133	1
0.41597	-1.78733	1.09133	1
0.41597	-1.78733	1.09133	1
0.41597	-1.78733	1.09133	1

0.41597	-1.78733	1.09133	1
0.41597	-1.78733	1.09133	1
0.41597	-1.78733	1.09133	1
0.41597	-1.78733	1.09133	1
0.41597	-1.78733	1.09133	1
0.41597	-1.78733	1.09133	1
0.41597	-1.78733	1.09133	1
0.41597	-1.78733	1.09133	1
0.41597	-1.78733	1.09133	1
0.41597	-1.78733	1.09133	1
0.41597	-1.78733	1.09133	1
0.41597	-1.78733	1.09133	1
0.41597	-1.78733	1.09133	1
0.41597	-1.78733	1.09133	1
0.41597	-1.78733	1.09133	1
0.41597	-1.78733	1.09133	1
0.41597	-1.78733	1.09133	1
0.41597	-1.78733	1.09133	1
0.41597	-1.78733	1.09133	1
0.41597	-1.78733	1.09133	1
0.41597	-1.78733	1.09133	1
0.41597	-1.78733	1.09133	1
0.41597	-1.78733	1.09133	1
0.41597	-1.78733	1.09133	1
0.41597	-1.78733	1.09133	1
0.41597	-1.78733	1.09133	1
0.41597	-1.78733	1.09133	1
0.41597	-1.78733	1.09133	1
0.41597	-1.78733	1.09133	1
0.41597	-1.78733	1.09133	1
0.41597	-1.78733	1.09133	1
0.41597	-1.78733	1.09133	1
0.41597	-1.78733	1.09133	1
0.41597	-1.78733	1.09133	1
0.41597	-1.78733	1.09133	1
0.41597	-1.78733	1.09133	1
0.41597	-1.78733	1.09133	1
0.41597	-1.78733	1.09133	1
0.41597	-1.78733	1.09133	1
0.41597	-1.78733	1.09133	1
0.41597	-1.78733	1.09133	1
0.41597	-1.78733	1.09133	1
0.41597	-1.78733	1.09133	1
0.41597	-1.78733	1.09133	1
0.41597	-1.78733	1.09133	1
0.41597	-1.78733	1.09133	1
0.41597	-1.78733	1.09133	1
0.41597	-1.78733	1.09133	1
0.41597	-1.78733	1.09133	1
0.55157	-1.10415	0.92087	1
0.55157	-1.10415	0.92087	1
0.55157	-1.10415	0.92087	1
0.55157	-1.10415	0.92087	1
0.55157	-1.10415	0.92087	1
0.55157	-1.10415	0.92087	1
0.55157	-1.10415	0.92087	1

0.55157	-1.10415	0.92087	1
0.55157	-1.10415	0.92087	1
0.55157	-1.10415	0.92087	1
0.55157	-1.10415	0.92087	1
0.55157	-1.10415	0.92087	1
0.55157	-1.10415	0.92087	1
0.55157	-1.10415	0.92087	1
0.55157	-1.10415	0.92087	1
0.55157	-1.10415	0.92087	1
0.55157	-1.10415	0.92087	1
0.55157	-1.10415	0.92087	1
0.55157	-1.10415	0.92087	1
0.55157	-1.10415	0.92087	1
0.55157	-1.10415	0.92087	1
0.55157	-1.10415	0.92087	1
0.55157	-1.10415	0.92087	1
0.55157	-1.10415	0.92087	1
0.55157	-1.10415	0.92087	1
0.55157	-1.10415	0.92087	1
0.55157	-1.10415	0.92087	1
0.55157	-1.10415	0.92087	1
0.55157	-1.10415	0.92087	1
0.55157	-1.10415	0.92087	1
0.55157	-1.10415	0.92087	1
0.55157	-1.10415	0.92087	1
0.55157	-1.10415	0.92087	1
0.55157	-1.10415	0.92087	1
0.55157	-1.10415	0.92087	1
0.55157	-1.10415	0.92087	1
0.55157	-1.10415	0.92087	1
0.55157	-1.10415	0.92087	1
0.55157	-1.10415	0.92087	1
0.55157	-1.10415	0.92087	1
0.55157	-1.10415	0.92087	1
0.55157	-1.10415	0.92087	1
0.55157	-1.10415	0.92087	1
0.55157	-1.10415	0.92087	1
0.55157	-1.10415	0.92087	1
0.55157	-1.10415	0.92087	1
0.55157	-1.10415	0.92087	1
0.55157	-1.10415	0.92087	1
0.55157	-1.10415	0.92087	1
0.55157	-1.10415	0.92087	1
0.55157	-1.10415	0.92087	1
0.55157	-1.10415	0.92087	1
0.55157	-1.10415	0.92087	1
0.55157	-1.10415	0.92087	1
0.55157	-1.10415	0.92087	1
0.55157	-1.10415	0.92087	1
0.55157	-1.10415	0.92087	1
0.55157	-1.10415	0.92087	1
0.55157	-1.10415	0.92087	1
0.55157	-1.10415	0.92087	1
0.55157	-1.10415	0.92087	1
0.55157	-1.10415	0.92087	1
0.55157	-1.10415	0.92087	1

0.55157	-1.10415	0.92087	1
0.55157	-1.10415	0.92087	1
0.55157	-1.10415	0.92087	1
0.55157	-1.10415	0.92087	1
0.55157	-1.10415	0.92087	1
0.55157	-1.10415	0.92087	1
0.55157	-1.10415	0.92087	1
0.55157	-1.10415	0.92087	1
0.55157	-1.10415	0.92087	1
0.55157	-1.10415	0.92087	1
0.55157	-1.10415	0.92087	1
0.55157	-1.10415	0.92087	1
0.55157	-1.10415	0.92087	1
0.55157	-1.10415	0.92087	1
0.55157	-1.10415	0.92087	1
0.55157	-1.10415	0.92087	1
0.55157	-1.10415	0.92087	1
0.55157	-1.10415	0.92087	1
0.55157	-1.10415	0.92087	1
0.55157	-1.10415	0.92087	1
0.55157	-1.10415	0.92087	1
0.55157	-1.10415	0.92087	1
0.55157	-1.10415	0.92087	1
0.55157	-1.10415	0.92087	1
0.55157	-1.10415	0.92087	1
0.55157	-1.10415	0.92087	1
0.55157	-1.10415	0.92087	1
0.55157	-1.10415	0.92087	1
0.55157	-1.10415	0.92087	1
0.55157	-1.10415	0.92087	1
0.55157	-1.10415	0.92087	1
0.55157	-1.10415	0.92087	1
0.55157	-1.10415	0.92087	1
0.55157	-1.10415	0.92087	1
0.55157	-1.10415	0.92087	1
0.55157	-1.10415	0.92087	1
0.55157	-1.10415	0.92087	1
0.55157	-1.10415	0.92087	1
0.55157	-1.10415	0.92087	1
0.55157	-1.10415	0.92087	1
0.55157	-1.10415	0.92087	1
0.55157	-1.10415	0.92087	1
0.55157	-1.10415	0.92087	1
0.55157	-1.10415	0.92087	1
0.55157	-1.10415	0.92087	1
0.55157	-1.10415	0.92087	1
0.55157	-1.10415	0.92087	1
0.55157	-1.10415	0.92087	1
0.55157	-1.10415	0.92087	1
0.55157	-1.10415	0.92087	1
0.55157	-1.10415	0.92087	1
0.55157	-1.10415	0.92087	1
0.55157	-1.10415	0.92087	1
0.55157	-1.10415	0.92087	1
0.55157	-1.10415	0.92087	1
0.55157	-1.10415	0.92087	1

0.55157	-1.10415	0.92087	1
0.55157	-1.10415	0.92087	1
0.55157	-1.10415	0.92087	1
0.55157	-1.10415	0.92087	1
0.55157	-1.10415	0.92087	1
0.55157	-1.10415	0.92087	1
0.55157	-1.10415	0.92087	1
0.55157	-1.10415	0.92087	1
0.55157	-1.10415	0.92087	1
0.55157	-1.10415	0.92087	1
0.55157	-1.10415	0.92087	1
0.55157	-1.10415	0.92087	1
0.55157	-1.10415	0.92087	1
0.55157	-1.10415	0.92087	1
0.55157	-1.10415	0.92087	1
0.55157	-1.10415	0.92087	1
0.55157	-1.10415	0.92087	1
0.55157	-1.10415	0.92087	1
0.55157	-1.10415	0.92087	1
0.55157	-1.10415	0.92087	1
0.55157	-1.10415	0.92087	1
0.55157	-1.10415	0.92087	1
0.55157	-1.10415	0.92087	1
0.55157	-1.10415	0.92087	1
0.55157	-1.10415	0.92087	1
0.55157	-1.10415	0.92087	1
0.55157	-1.10415	0.92087	1
0.55157	-1.10415	0.92087	1
0.55157	-1.10415	0.92087	1
0.55157	-1.10415	0.92087	1
0.55157	-1.10415	0.92087	1
0.55157	-1.10415	0.92087	1
0.55157	-1.10415	0.92087	1
0.55157	-1.10415	0.92087	1
0.55157	-1.10415	0.92087	1
0.55157	-1.10415	0.92087	1
0.55157	-1.10415	0.92087	1
0.55157	-1.10415	0.92087	1
0.55157	-1.10415	0.92087	1
0.55157	-1.10415	0.92087	1
0.55157	-1.10415	0.92087	1
0.55157	-1.10415	0.92087	1
0.55157	-1.10415	0.92087	1
0.55157	-1.10415	0.92087	1
0.55157	-1.10415	0.92087	1
0.55157	-1.10415	0.92087	1
0.55157	-1.10415	0.92087	1
0.55157	-1.10415	0.92087	1
0.55157	-1.10415	0.92087	1
0.55157	-1.10415	0.92087	1
0.55157	-1.10415	0.92087	1
0.55157	-1.10415	0.92087	1
0.55157	-1.10415	0.92087	1
0.55157	-1.10415	0.92087	1
0.55157	-1.10415	0.92087	1
0.55157	-1.10415	0.92087	1

0.55157	-1.10415	0.92087	1
0.55157	-1.10415	0.92087	1
0.55157	-1.10415	0.92087	1
0.55157	-1.10415	0.92087	1
0.55157	-1.10415	0.92087	1
0.55157	-1.10415	0.92087	1
0.55157	-1.10415	0.92087	1
0.55157	-1.10415	0.92087	1
0.55157	-1.10415	0.92087	1
0.55157	-1.10415	0.92087	1
0.55157	-1.10415	0.92087	1
0.55157	-1.10415	0.92087	1
0.55157	-1.10415	0.92087	1
0.55157	-1.10415	0.92087	1
0.55157	-1.10415	0.92087	1
0.55157	-1.10415	0.92087	1
0.55157	-1.10415	0.92087	1
0.55157	-1.10415	0.92087	1
0.55157	-1.10415	0.92087	1
0.55157	-1.10415	0.92087	1
0.55157	-1.10415	0.92087	1
0.55157	-1.10415	0.92087	1
0.55157	-1.10415	0.92087	1
0.55157	-1.10415	0.92087	1
0.55157	-1.10415	0.92087	1
0.55157	-1.10415	0.92087	1
0.55157	-1.10415	0.92087	1
0.55157	-1.10415	0.92087	1
0.55157	-1.10415	0.92087	1
0.55157	-1.10415	0.92087	1
0.55157	-1.10415	0.92087	1
0.55157	-1.10415	0.92087	1
0.55157	-1.10415	0.92087	1
0.55157	-1.10415	0.92087	1
0.55157	-1.10415	0.92087	1
0.55157	-1.10415	0.92087	1
0.55157	-1.10415	0.92087	1
0.55157	-1.10415	0.92087	1
0.55157	-1.10415	0.92087	1
0.55157	-1.10415	0.92087	1
0.55157	-1.10415	0.92087	1
0.55157	-1.10415	0.92087	1
0.55157	-1.10415	0.92087	1
0.55157	-1.10415	0.92087	1
0.55157	-1.10415	0.92087	1
0.55157	-1.10415	0.92087	1
0.55157	-1.10415	0.92087	1
0.55157	-1.10415	0.92087	1
0.55157	-1.10415	0.92087	1
0.55157	-1.10415	0.92087	1
0.55157	-1.10415	0.92087	1
0.55157	-1.10415	0.92087	1
0.55157	-1.10415	0.92087	1
0.55157	-1.10415	0.92087	1
0.55157	-1.10415	0.92087	1
0.55157	-1.10415	0.92087	1

0.55157	-1.10415	0.92087	1
0.55157	-1.10415	0.92087	1
0.55157	-1.10415	0.92087	1
0.55157	-1.10415	0.92087	1
0.55157	-1.10415	0.92087	1
0.55157	-1.10415	0.92087	1
0.55157	-1.10415	0.92087	1
0.55157	-1.10415	0.92087	1
0.55157	-1.10415	0.92087	1
0.55157	-1.10415	0.92087	1
0.55157	-1.10415	0.92087	1
0.55157	-1.10415	0.92087	1
0.55157	-1.10415	0.92087	1
0.55157	-1.10415	0.92087	1
0.55157	-1.10415	0.92087	1
0.55157	-1.10415	0.92087	1
0.55157	-1.10415	0.92087	1
0.55157	-1.10415	0.92087	1
0.55157	-1.10415	0.92087	1
0.55157	-1.10415	0.92087	1
0.55157	-1.10415	0.92087	1
0.55157	-1.10415	0.92087	1
0.55157	-1.10415	0.92087	1
0.55157	-1.10415	0.92087	1
0.55157	-1.10415	0.92087	1
0.55157	-1.10415	0.92087	1
0.55157	-1.10415	0.92087	1
0.55157	-1.10415	0.92087	1
0.55157	-1.10415	0.92087	1
0.55157	-1.10415	0.92087	1
0.55157	-1.10415	0.92087	1
0.55157	-1.10415	0.92087	1
0.55157	-1.10415	0.92087	1
0.55157	-1.10415	0.92087	1
0.55157	-1.10415	0.92087	1
0.55157	-1.10415	0.92087	1
0.55157	-1.10415	0.92087	1
0.55157	-1.10415	0.92087	1
0.55157	-1.10415	0.92087	1
0.55157	-1.10415	0.92087	1
0.55157	-1.10415	0.92087	1
0.55157	-1.10415	0.92087	1
0.55157	-1.10415	0.92087	1
0.55157	-1.10415	0.92087	1
0.55157	-1.10415	0.92087	1
0.55157	-1.10415	0.92087	1
0.55157	-1.10415	0.92087	1
0.55157	-1.10415	0.92087	1
0.55157	-1.10415	0.92087	1
0.55157	-1.10415	0.92087	1
0.55157	-1.10415	0.92087	1
0.55157	-1.10415	0.92087	1
0.55157	-1.10415	0.92087	1
0.55157	-1.10415	0.92087	1
0.55157	-1.10415	0.92087	1
0.55157	-1.10415	0.92087	1

0.55157	-1.10415	0.92087	1
0.55157	-1.10415	0.92087	1
0.55157	-1.10415	0.92087	1
0.55157	-1.10415	0.92087	1
0.55157	-1.10415	0.92087	1
0.55157	-1.10415	0.92087	1
0.55157	-1.10415	0.92087	1
0.55157	-1.10415	0.92087	1
0.55157	-1.10415	0.92087	1
0.55157	-1.10415	0.92087	1
0.55157	-1.10415	0.92087	1
0.55157	-1.10415	0.92087	1
0.55157	-1.10415	0.92087	1
0.55157	-1.10415	0.92087	1
0.55157	-1.10415	0.92087	1
0.55157	-1.10415	0.92087	1
0.55157	-1.10415	0.92087	1
0.55157	-1.10415	0.92087	1
0.55157	-1.10415	0.92087	1
0.55157	-1.10415	0.92087	1
0.55157	-1.10415	0.92087	1
0.55157	-1.10415	0.92087	1
0.55157	-1.10415	0.92087	1
0.55157	-1.10415	0.92087	1
0.55157	-1.10415	0.92087	1
0.55157	-1.10415	0.92087	1
0.55157	-1.10415	0.92087	1
0.55157	-1.10415	0.92087	1
0.55157	-1.10415	0.92087	1
0.55157	-1.10415	0.92087	1
0.55157	-1.10415	0.92087	1
0.55157	-1.10415	0.92087	1
0.55157	-1.10415	0.92087	1
0.55157	-1.10415	0.92087	1
0.55157	-1.10415	0.92087	1
0.55157	-1.10415	0.92087	1
0.55157	-1.10415	0.92087	1
0.55157	-1.10415	0.92087	1
0.55157	-1.10415	0.92087	1
0.55157	-1.10415	0.92087	1
0.55157	-1.10415	0.92087	1
0.55157	-1.10415	0.92087	1
0.55157	-1.10415	0.92087	1
0.55157	-1.10415	0.92087	1
0.55157	-1.10415	0.92087	1
0.55157	-1.10415	0.92087	1
0.55157	-1.10415	0.92087	1
0.55157	-1.10415	0.92087	1
0.55157	-1.10415	0.92087	1
0.55157	-1.10415	0.92087	1
0.55157	-1.10415	0.92087	1
0.55157	-1.10415	0.92087	1
0.55157	-1.10415	0.92087	1
0.55157	-1.10415	0.92087	1
0.55157	-1.10415	0.92087	1
0.55157	-1.10415	0.92087	1

0.55157	-1.10415	0.92087	1
0.55157	-1.10415	0.92087	1
0.55157	-1.10415	0.92087	1
0.55157	-1.10415	0.92087	1
0.55157	-1.10415	0.92087	1
0.55157	-1.10415	0.92087	1
0.55157	-1.10415	0.92087	1
0.55157	-1.10415	0.92087	1
0.55157	-1.10415	0.92087	1
0.55157	-1.10415	0.92087	1
0.55157	-1.10415	0.92087	1
0.55157	-1.10415	0.92087	1
0.55157	-1.10415	0.92087	1
0.55157	-1.10415	0.92087	1
0.55157	-1.10415	0.92087	1
0.55157	-1.10415	0.92087	1
0.55157	-1.10415	0.92087	1
0.55157	-1.10415	0.92087	1
0.55157	-1.10415	0.92087	1
0.55157	-1.10415	0.92087	1
0.55157	-1.10415	0.92087	1
0.55157	-1.10415	0.92087	1
0.55157	-1.10415	0.92087	1
0.55157	-1.10415	0.92087	1
0.55157	-1.10415	0.92087	1
0.55157	-1.10415	0.92087	1
0.55157	-1.10415	0.92087	1
0.55157	-1.10415	0.92087	1
0.55157	-1.10415	0.92087	1
0.55157	-1.10415	0.92087	1
0.55157	-1.10415	0.92087	1
0.55157	-1.10415	0.92087	1
0.55157	-1.10415	0.92087	1
0.55157	-1.10415	0.92087	1
0.55157	-1.10415	0.92087	1
0.55157	-1.10415	0.92087	1
0.55157	-1.10415	0.92087	1
0.55157	-1.10415	0.92087	1
0.55157	-1.10415	0.92087	1
0.55157	-1.10415	0.92087	1
0.55157	-1.10415	0.92087	1
0.55157	-1.10415	0.92087	1
0.55157	-1.10415	0.92087	1
0.55157	-1.10415	0.92087	1
0.55157	-1.10415	0.92087	1
0.55157	-1.10415	0.92087	1
0.55157	-1.10415	0.92087	1
0.55157	-1.10415	0.92087	1
0.55157	-1.10415	0.92087	1
0.55157	-1.10415	0.92087	1
0.55157	-1.10415	0.92087	1
0.55157	-1.10415	0.92087	1
0.55157	-1.10415	0.92087	1
0.55157	-1.10415	0.92087	1
0.55157	-1.10415	0.92087	1
0.55157	-1.10415	0.92087	1

0.55157	-1.10415	0.92087	1
0.55157	-1.10415	0.92087	1
0.55157	-1.10415	0.92087	1
0.55157	-1.10415	0.92087	1
0.55157	-1.10415	0.92087	1
0.55157	-1.10415	0.92087	1
0.55157	-1.10415	0.92087	1
0.55157	-1.10415	0.92087	1
0.55157	-1.10415	0.92087	1
0.55157	-1.10415	0.92087	1
0.55157	-1.10415	0.92087	1
0.55157	-1.10415	0.92087	1
0.55157	-1.10415	0.92087	1
0.55157	-1.10415	0.92087	1
0.55157	-1.10415	0.92087	1
0.55157	-1.10415	0.92087	1
0.55157	-1.10415	0.92087	1
0.55157	-1.10415	0.92087	1
0.55157	-1.10415	0.92087	1
0.55157	-1.10415	0.92087	1
0.55157	-1.10415	0.92087	1
0.55157	-1.10415	0.92087	1
0.55157	-1.10415	0.92087	1
0.55157	-1.10415	0.92087	1
0.55157	-1.10415	0.92087	1
0.55157	-1.10415	0.92087	1
0.55157	-1.10415	0.92087	1
0.55157	-1.10415	0.92087	1
0.55157	-1.10415	0.92087	1
0.55157	-1.10415	0.92087	1
0.55157	-1.10415	0.92087	1
0.55157	-1.10415	0.92087	1
0.55157	-1.10415	0.92087	1
0.55157	-1.10415	0.92087	1
0.55157	-1.10415	0.92087	1
0.55157	-1.10415	0.92087	1
0.55157	-1.10415	0.92087	1
0.55157	-1.10415	0.92087	1
0.55157	-1.10415	0.92087	1
0.55157	-1.10415	0.92087	1
0.55157	-1.10415	0.92087	1
0.55157	-1.10415	0.92087	1
0.55157	-1.10415	0.92087	1
0.55157	-1.10415	0.92087	1
0.55157	-1.10415	0.92087	1
0.55157	-1.10415	0.92087	1
0.55157	-1.10415	0.92087	1
0.55157	-1.10415	0.92087	1
0.55157	-1.10415	0.92087	1
0.55157	-1.10415	0.92087	1
0.55157	-1.10415	0.92087	1
0.55157	-1.10415	0.92087	1
0.55157	-1.10415	0.92087	1
0.55157	-1.10415	0.92087	1
0.55157	-1.10415	0.92087	1
0.55157	-1.10415	0.92087	1

0.55157	-1.10415	0.92087	1
0.55157	-1.10415	0.92087	1
0.55157	-1.10415	0.92087	1
0.55157	-1.10415	0.92087	1
0.55157	-1.10415	0.92087	1
0.55157	-1.10415	0.92087	1
0.55157	-1.10415	0.92087	1
0.55157	-1.10415	0.92087	1
0.55157	-1.10415	0.92087	1
0.55157	-1.10415	0.92087	1
0.55157	-1.10415	0.92087	1
0.55157	-1.10415	0.92087	1
0.55157	-1.10415	0.92087	1
0.55157	-1.10415	0.92087	1
0.55157	-1.10415	0.92087	1
0.55157	-1.10415	0.92087	1
0.55157	-1.10415	0.92087	1
0.55157	-1.10415	0.92087	1
0.55157	-1.10415	0.92087	1
0.55157	-1.10415	0.92087	1
0.55157	-1.10415	0.92087	1
0.55157	-1.10415	0.92087	1
0.55157	-1.10415	0.92087	1
0.55157	-1.10415	0.92087	1
0.55157	-1.10415	0.92087	1
0.55157	-1.10415	0.92087	1
0.55157	-1.10415	0.92087	1
0.55157	-1.10415	0.92087	1
0.55157	-1.10415	0.92087	1
0.55157	-1.10415	0.92087	1
0.55157	-1.10415	0.92087	1
0.55157	-1.10415	0.92087	1
0.55157	-1.10415	0.92087	1
0.55157	-1.10415	0.92087	1
0.55157	-1.10415	0.92087	1
0.55157	-1.10415	0.92087	1
0.55157	-1.10415	0.92087	1
0.55157	-1.10415	0.92087	1
0.55157	-1.10415	0.92087	1
0.55157	-1.10415	0.92087	1
0.55157	-1.10415	0.92087	1
0.55157	-1.10415	0.92087	1
0.55157	-1.10415	0.92087	1
0.55157	-1.10415	0.92087	1
0.55157	-1.10415	0.92087	1
0.55157	-1.10415	0.92087	1
0.55157	-1.10415	0.92087	1
0.55157	-1.10415	0.92087	1
0.55157	-1.10415	0.92087	1
0.55157	-1.10415	0.92087	1
0.55157	-1.10415	0.92087	1
0.55157	-1.10415	0.92087	1
0.55157	-1.10415	0.92087	1
0.55157	-1.10415	0.92087	1
0.55157	-1.10415	0.92087	1
0.55157	-1.10415	0.92087	1

0.55157	-1.10415	0.92087	1
0.41597	-1.78733	1.09133	1
0.41597	-1.78733	1.09133	1
0.41597	-1.78733	1.09133	1
0.41597	-1.78733	1.09133	1
0.41597	-1.78733	1.09133	1
0.41597	-1.78733	1.09133	1
0.41597	-1.78733	1.09133	1
0.41597	-1.78733	1.09133	1
0.41597	-1.78733	1.09133	1
0.41597	-1.78733	1.09133	1
0.41597	-1.78733	1.09133	1
0.41597	-1.78733	1.09133	1
0.41597	-1.78733	1.09133	1
0.41597	-1.78733	1.09133	1
0.41597	-1.78733	1.09133	1
0.41597	-1.78733	1.09133	1
0.41597	-1.78733	1.09133	1
0.41597	-1.78733	1.09133	1
0.41597	-1.78733	1.09133	1
0.41597	-1.78733	1.09133	1
0.41597	-1.78733	1.09133	1
0.41597	-1.78733	1.09133	1
0.41597	-1.78733	1.09133	1
0.41597	-1.78733	1.09133	1
0.41597	-1.78733	1.09133	1
0.41597	-1.78733	1.09133	1
0.41597	-1.78733	1.09133	1
0.41597	-1.78733	1.09133	1
0.41597	-1.78733	1.09133	1
0.41597	-1.78733	1.09133	1
0.41597	-1.78733	1.09133	1
0.41597	-1.78733	1.09133	1
0.41597	-1.78733	1.09133	1
0.41597	-1.78733	1.09133	1
0.41597	-1.78733	1.09133	1
0.41597	-1.78733	1.09133	1
0.41597	-1.78733	1.09133	1
0.41597	-1.78733	1.09133	1
0.41597	-1.78733	1.09133	1
0.41597	-1.78733	1.09133	1
0.41597	-1.78733	1.09133	1
0.41597	-1.78733	1.09133	1
0.41597	-1.78733	1.09133	1
0.41597	-1.78733	1.09133	1
0.41597	-1.78733	1.09133	1
0.41597	-1.78733	1.09133	1
0.41597	-1.78733	1.09133	1
0.41597	-1.78733	1.09133	1
0.41597	-1.78733	1.09133	1
0.41597	-1.78733	1.09133	1
0.41597	-1.78733	1.09133	1
0.41597	-1.78733	1.09133	1
0.41597	-1.78733	1.09133	1
0.41597	-1.78733	1.09133	1
0.41597	-1.78733	1.09133	1

0.41597	-1.78733	1.09133	1
0.41597	-1.78733	1.09133	1
0.41597	-1.78733	1.09133	1
0.41597	-1.78733	1.09133	1
0.41597	-1.78733	1.09133	1
0.41597	-1.78733	1.09133	1
0.41597	-1.78733	1.09133	1
0.41597	-1.78733	1.09133	1
0.41597	-1.78733	1.09133	1
0.55157	-1.10415	0.92087	1
0.55157	-1.10415	0.92087	1
0.55157	-1.10415	0.92087	1
0.55157	-1.10415	0.92087	1
0.55157	-1.10415	0.92087	1
0.55157	-1.10415	0.92087	1
0.55157	-1.10415	0.92087	1
0.55157	-1.10415	0.92087	1
0.55157	-1.10415	0.92087	1
0.55157	-1.10415	0.92087	1
0.55157	-1.10415	0.92087	1
0.55157	-1.10415	0.92087	1
0.55157	-1.10415	0.92087	1
0.55157	-1.10415	0.92087	1
0.55157	-1.10415	0.92087	1
0.55157	-1.10415	0.92087	1
0.55157	-1.10415	0.92087	1
0.55157	-1.10415	0.92087	1
0.55157	-1.10415	0.92087	1
0.55157	-1.10415	0.92087	1
0.55157	-1.10415	0.92087	1
0.55157	-1.10415	0.92087	1
0.55157	-1.10415	0.92087	1
0.55157	-1.10415	0.92087	1
0.55157	-1.10415	0.92087	1
0.55157	-1.10415	0.92087	1
0.55157	-1.10415	0.92087	1
0.55157	-1.10415	0.92087	1
0.55157	-1.10415	0.92087	1
0.55157	-1.10415	0.92087	1
0.55157	-1.10415	0.92087	1
0.55157	-1.10415	0.92087	1
0.55157	-1.10415	0.92087	1
0.55157	-1.10415	0.92087	1
0.55157	-1.10415	0.92087	1
0.55157	-1.10415	0.92087	1
0.55157	-1.10415	0.92087	1
0.55157	-1.10415	0.92087	1
0.55157	-1.10415	0.92087	1
0.55157	-1.10415	0.92087	1
0.55157	-1.10415	0.92087	1
0.55157	-1.10415	0.92087	1
0.55157	-1.10415	0.92087	1
0.55157	-1.10415	0.92087	1
0.55157	-1.10415	0.92087	1
0.55157	-1.10415	0.92087	1
0.55157	-1.10415	0.92087	1

0.55157	-1.10415	0.92087	1
0.55157	-1.10415	0.92087	1
0.55157	-1.10415	0.92087	1
0.55157	-1.10415	0.92087	1
0.55157	-1.10415	0.92087	1
0.55157	-1.10415	0.92087	1
0.55157	-1.10415	0.92087	1
0.55157	-1.10415	0.92087	1
0.55157	-1.10415	0.92087	1
0.55157	-1.10415	0.92087	1
0.55157	-1.10415	0.92087	1
0.55157	-1.10415	0.92087	1
0.55157	-1.10415	0.92087	1
0.55157	-1.10415	0.92087	1
0.55157	-1.10415	0.92087	1
0.55157	-1.10415	0.92087	1
0.55157	-1.10415	0.92087	1
0.41597	-1.78733	1.09133	1
0.41597	-1.78733	1.09133	1
0.41597	-1.78733	1.09133	1
0.41597	-1.78733	1.09133	1
0.41597	-1.78733	1.09133	1
0.41597	-1.78733	1.09133	1
0.41597	-1.78733	1.09133	1
0.41597	-1.78733	1.09133	1
0.41597	-1.78733	1.09133	1
0.41597	-1.78733	1.09133	1
0.41597	-1.78733	1.09133	1
0.41597	-1.78733	1.09133	1
0.41597	-1.78733	1.09133	1
0.41597	-1.78733	1.09133	1
0.41597	-1.78733	1.09133	1
0.41597	-1.78733	1.09133	1
0.41597	-1.78733	1.09133	1
0.41597	-1.78733	1.09133	1
0.41597	-1.78733	1.09133	1
0.41597	-1.78733	1.09133	1
0.41597	-1.78733	1.09133	1
0.41597	-1.78733	1.09133	1
0.41597	-1.78733	1.09133	1
0.41597	-1.78733	1.09133	1
0.41597	-1.78733	1.09133	1
0.41597	-1.78733	1.09133	1
0.41597	-1.78733	1.09133	1
0.41597	-1.78733	1.09133	1
0.41597	-1.78733	1.09133	1
0.41597	-1.78733	1.09133	1
0.41597	-1.78733	1.09133	1
0.41597	-1.78733	1.09133	1
0.41597	-1.78733	1.09133	1
0.41597	-1.78733	1.09133	1
0.41597	-1.78733	1.09133	1
0.41597	-1.78733	1.09133	1
0.41597	-1.78733	1.09133	1
0.41597	-1.78733	1.09133	1
0.41597	-1.78733	1.09133	1

0.41597	-1.78733	1.09133	1
0.41597	-1.78733	1.09133	1
0.41597	-1.78733	1.09133	1
0.41597	-1.78733	1.09133	1
0.41597	-1.78733	1.09133	1
0.41597	-1.78733	1.09133	1
0.41597	-1.78733	1.09133	1
0.41597	-1.78733	1.09133	1
0.41597	-1.78733	1.09133	1
0.41597	-1.78733	1.09133	1
0.41597	-1.78733	1.09133	1
0.41597	-1.78733	1.09133	1
0.41597	-1.78733	1.09133	1
0.41597	-1.78733	1.09133	1
0.41597	-1.78733	1.09133	1
0.41597	-1.78733	1.09133	1
0.41597	-1.78733	1.09133	1
0.41597	-1.78733	1.09133	1
0.41597	-1.78733	1.09133	1
0.41597	-1.78733	1.09133	1
0.41597	-1.78733	1.09133	1
0.41597	-1.78733	1.09133	1
0.41597	-1.78733	1.09133	1
0.41597	-1.78733	1.09133	1
0.41597	-1.78733	1.09133	1
0.41597	-1.78733	1.09133	1
0.41597	-1.78733	1.09133	1
0.41597	-1.78733	1.09133	1
0.41597	-1.78733	1.09133	1
0.41597	-1.78733	1.09133	1
0.41597	-1.78733	1.09133	1
0.41597	-1.78733	1.09133	1
0.41597	-1.78733	1.09133	1
0.41597	-1.78733	1.09133	1
0.41597	-1.78733	1.09133	1
0.41597	-1.78733	1.09133	1
0.41597	-1.78733	1.09133	1
0.41597	-1.78733	1.09133	1
0.41597	-1.78733	1.09133	1
0.41597	-1.78733	1.09133	1
0.41597	-1.78733	1.09133	1
0.41597	-1.78733	1.09133	1
0.41597	-1.78733	1.09133	1
0.41597	-1.78733	1.09133	1
0.41597	-1.78733	1.09133	1
0.41597	-1.78733	1.09133	1
0.41597	-1.78733	1.09133	1
0.41597	-1.78733	1.09133	1
0.41597	-1.78733	1.09133	1
0.41597	-1.78733	1.09133	1
0.41597	-1.78733	1.09133	1
0.41597	-1.78733	1.09133	1
0.41597	-1.78733	1.09133	1
0.41597	-1.78733	1.09133	1
0.41597	-1.78733	1.09133	1
0.41597	-1.78733	1.09133	1

0.41597	-1.78733	1.09133	1
0.41597	-1.78733	1.09133	1
0.41597	-1.78733	1.09133	1
0.41597	-1.78733	1.09133	1
0.41597	-1.78733	1.09133	1
0.41597	-1.78733	1.09133	1
0.41597	-1.78733	1.09133	1
0.41597	-1.78733	1.09133	1
0.41597	-1.78733	1.09133	1
0.41597	-1.78733	1.09133	1
0.41597	-1.78733	1.09133	1
0.41597	-1.78733	1.09133	1
0.41597	-1.78733	1.09133	1
0.41597	-1.78733	1.09133	1
0.41597	-1.78733	1.09133	1
0.41597	-1.78733	1.09133	1
0.41597	-1.78733	1.09133	1
0.41597	-1.78733	1.09133	1
0.41597	-1.78733	1.09133	1
0.41597	-1.78733	1.09133	1
0.41597	-1.78733	1.09133	1
0.41597	-1.78733	1.09133	1
0.41597	-1.78733	1.09133	1
0.41597	-1.78733	1.09133	1
0.41597	-1.78733	1.09133	1
0.41597	-1.78733	1.09133	1
0.41597	-1.78733	1.09133	1
0.41597	-1.78733	1.09133	1
0.41597	-1.78733	1.09133	1
0.41597	-1.78733	1.09133	1
0.41597	-1.78733	1.09133	1
0.41597	-1.78733	1.09133	1
0.41597	-1.78733	1.09133	1
