# Supplementary material for: Diel movement of brown trout, Salmo trutta, is reduced in dense populations with high site fidelity
Source: Ecol Evol. 2018 Apr 6;8(9):4495–507. doi: 10.1002/ece3.3981 (PMC5938464; doi:10.1002/ece3.3981)
Supplement: Supplementary file 2 [file ECE3-8-4495-s002.pdf]

lokalita	site_fidelity_ratio	log_Ephemeroptera	log_Plecoptera	log_Trichoptera
Roklansky_I	0	1.54716	3.54096	1.53466
Roklansky_II	0	.	0.22314	1.10551
Roklansky_III	1	0.60206	1.94591	1.02119
Javori_I	0	0.69897	2.11021	0.75967
Javori_II	0	-0.12494	0.69315	1.52179
Rokytka	0	0.51188	0.40547	0.57403
Tmavy_potok	0	1.15381	2.72458	0.79588
Modravsky_I	1	0.57403	1.25276	0.86034
Modravsky_II	1	0.24304	0.22314	0.95424
Luzensky_I	0	.	1.09861	0.47712
Luzensky_II	1	0.77815	2.01490	1.13830
Breznicky	1	0.75967	2.78809	0.79588
Hamersky_I	0	-0.12494	0.40547	0.84510
Hamersky_II	1	0.62839	2.42037	0.67669
Filipohutsky	1	-0.30103	2.46385	0.69897
Svelsky	0	1.24304	2.48491	0.69897
Vydra	0	0.39794	3.77276	0.90309
Kremelna_I	1	-0.60206	2.98315	1.13033
Kremelna_II	1	1.49831	3.51155	0.81291
Kremelna_III	1	1.13830	2.74084	1.24920

log_Diptera	log_Coleoptera	log_Crustacea	log_Invertebrates_all
1.51188	0.43933	-0.30103	2.14535
1.24304	-0.60206	.	1.50174
0.39794	1.06070	.	1.55023
0.57403	-0.60206	0.84510	1.47712
0.98900	-0.30103	.	1.66511
1.37107	-0.60206	.	1.50853
0.17609	0.00000	-0.60206	1.58546
0.75967	.	.	1.30643
0.47712	-0.60206	.	1.18327
1.47712	1.04139	.	1.67210
1.39358	0.67669	.	1.75397
1.10551	0.24304	.	1.63094
1.70544	-0.12494	.	1.78355
0.09691	0.54407	0.77815	1.49136
0.90309	0.35218	.	1.43933
-0.60206	0.94201	1.65562	1.94817
0.62839	0.47712	.	1.78711
0.81291	0.62839	1.30103	1.80787
1.13033	1.34242	1.26126	2.09778
1.11394	0.88930	-0.30103	1.83410
