# Supplementary material for: Diel movement of brown trout, Salmo trutta, is reduced in dense populations with high site fidelity
Source: Ecol Evol. 2018 Apr 6;8(9):4495–507. doi: 10.1002/ece3.3981 (PMC5938464; doi:10.1002/ece3.3981)
Supplement: Supplementary file 3 [file ECE3-8-4495-s003.pdf]

date	code_1	weight_1	length_1	code_2	weight_2	length_2	mutual_distance
16.05.2011	15	59	166	17	34	137	0,0
16.05.2011	13	48	159	27	46	152	0,0
16.05.2011	13	48	159	33	30	132	0,0
16.05.2011	27	46	152	33	30	132	0,0
16.05.2011	14	87	198	15	59	166	0,0
16.05.2011	14	87	198	16	67	169	0,0
16.05.2011	14	87	198	51	141	207	0,0
16.05.2011	14	87	198	53	152	218	0,0
16.05.2011	15	59	166	17	34	137	0,0
16.05.2011	15	59	166	22	24	124	0,0
16.05.2011	15	59	166	51	141	207	0,0
16.05.2011	16	67	169	51	141	207	0,0
16.05.2011	16	67	169	53	152	218	0,0
16.05.2011	17	34	137	22	24	124	0,0
16.05.2011	17	34	137	51	141	207	0,0
16.05.2011	51	141	207	53	152	218	0,0
16.05.2011	53	152	218	54	199	238	0,0
16.05.2011	13	48	159	24	56	163	0,0
16.05.2011	13	48	159	33	30	132	0,0
16.05.2011	24	56	163	33	30	132	0,0
16.05.2011	24	56	163	57	72	182	0,0
16.05.2011	13	48	159	59	89	191	0,0
16.05.2011	24	56	163	59	89	191	0,0
16.05.2011	33	30	132	59	89	191	0,0
16.05.2011	57	72	182	59	89	191	0,0
16.05.2011	13	48	159	60	119	217	0,0
16.05.2011	33	30	132	60	119	217	0,0
16.05.2011	59	89	191	60	119	217	0,0
16.05.2011	17	34	137	22	24	124	0,0
16.05.2011	22	24	124	23	21	117	0,0
16.05.2011	53	152	218	54	199	238	0,0
16.05.2011	13	48	159	19	58	166	0,0

16.05.2011	13	48	159	27	46	152	0,0
16.05.2011	19	58	166	27	46	152	0,0
16.05.2011	57	72	182	59	89	191	0,0
16.05.2011	14	87	198	15	59	166	0,0
16.05.2011	30	42	151	33	30	132	0,0
16.05.2011	17	34	137	54	199	238	0,0
16.05.2011	30	42	151	57	72	182	0,0
17.05.2011	51	141	207	52	147	212	0,0
17.05.2011	14	87	198	55	126	201	0,0
17.05.2011	27	46	152	30	42	151	0,0
17.05.2011	15	59	166	17	34	137	0,0
17.05.2011	17	34	137	55	126	201	0,0
17.05.2011	24	56	163	27	46	152	0,0
17.05.2011	30	42	151	33	30	132	0,0
17.05.2011	24	56	163	56	67	170	0,0
17.05.2011	57	72	182	59	89	191	0,0
17.05.2011	13	48	159	27	46	152	0,0
17.05.2011	13	48	159	30	42	151	0,0
17.05.2011	27	46	152	30	42	151	0,0
17.05.2011	17	34	137	23	21	117	0,0
17.05.2011	13	48	159	27	46	152	0,0
17.05.2011	13	48	159	30	42	151	0,0
17.05.2011	27	46	152	30	42	151	0,0
17.05.2011	13	48	159	56	67	170	0,0
17.05.2011	27	46	152	56	67	170	0,0
17.05.2011	30	42	151	56	67	170	0,0
17.05.2011	13	48	159	57	72	182	0,0
17.05.2011	27	46	152	57	72	182	0,0
17.05.2011	56	67	170	57	72	182	0,0
17.05.2011	13	48	159	59	89	191	0,0
17.05.2011	27	46	152	59	89	191	0,0
17.05.2011	56	67	170	59	89	191	0,0
17.05.2011	57	72	182	59	89	191	0,0

18.05.2011	13	48	159	30	42	151	0,0
18.05.2011	13	48	159	33	30	132	0,0
18.05.2011	30	42	151	33	30	132	0,0
18.05.2011	13	48	159	56	67	170	0,0
18.05.2011	30	42	151	56	67	170	0,0
18.05.2011	33	30	132	56	67	170	0,0
18.05.2011	13	48	159	57	72	182	0,0
18.05.2011	30	42	151	57	72	182	0,0
18.05.2011	56	67	170	57	72	182	0,0
18.05.2011	33	30	132	60	119	217	0,0
18.05.2011	27	46	152	56	67	170	0,0
18.05.2011	27	46	152	59	89	191	0,0
18.05.2011	56	67	170	59	89	191	0,0
18.05.2011	15	59	166	16	67	169	0,0
18.05.2011	15	59	166	17	34	137	0,0
18.05.2011	16	67	169	17	34	137	0,0
25.05.2011	57	72	182	60	119	217	0,0
25.05.2011	19	58	166	24	56	163	0,0
25.05.2011	13	48	159	30	42	151	0,0
25.05.2011	13	48	159	33	30	132	0,0
25.05.2011	30	42	151	33	30	132	0,0
25.05.2011	33	30	132	57	72	182	0,0
25.05.2011	56	67	170	57	72	182	0,0
25.05.2011	13	48	159	30	42	151	0,0
25.05.2011	13	48	159	33	30	132	0,0
25.05.2011	30	42	151	33	30	132	0,0
26.05.2011	15	59	166	22	24	124	0,0
26.05.2011	24	56	163	33	30	132	0,0
26.05.2011	56	67	170	57	72	182	0,0
26.05.2011	13	48	159	57	72	182	0,0
26.05.2011	13	48	159	60	119	217	0,0
26.05.2011	57	72	182	60	119	217	0,0
26.05.2011	57	72	182	60	119	217	0,0

26.05.2011	19	58	166	24	56	163	0,0
27.05.2011	13	48	159	30	42	151	0,0
27.05.2011	13	48	159	57	72	182	0,0
27.05.2011	27	46	152	33	30	132	0,0
27.05.2011	30	42	151	57	72	182	0,0
27.05.2011	19	58	166	24	56	163	0,0
27.05.2011	13	48	159	56	67	170	0,0
27.05.2011	56	67	170	57	72	182	0,0
27.05.2011	17	34	137	22	24	124	0,0
27.05.2011	24	56	163	33	30	132	0,0
01.06.2011	24	56	163	57	72	182	0,0
01.06.2011	56	67	170	57	72	182	0,0
01.06.2011	24	56	163	57	72	182	0,0
03.06.2011	27	30	132	56	67	170	0,0
06.06.2011	27	46	152	56	67	170	0,0
06.06.2011	19	58	166	27	46	152	0,0
16.06.2011	13	123	195	20	65	158	0,0
16.06.2011	13	123	195	48	81	168	0,0
27.06.2011	20	65	158	48	81	168	0,0
28.06.2011	20	65	158	48	81	168	0,0
16.05.2011	14	87	198	22	24	124	0,1
16.05.2011	14	87	198	17	34	137	0,1
16.05.2011	14	87	198	22	24	124	0,1
16.05.2011	14	87	198	54	199	238	0,1
16.05.2011	15	59	166	16	67	169	0,1
16.05.2011	15	59	166	53	152	218	0,1
16.05.2011	16	67	169	17	34	137	0,1
16.05.2011	16	67	169	22	24	124	0,1
16.05.2011	16	67	169	54	199	238	0,1
16.05.2011	17	34	137	53	152	218	0,1
16.05.2011	22	24	124	51	141	207	0,1
16.05.2011	22	24	124	53	152	218	0,1
16.05.2011	51	141	207	54	199	238	0,1

16.05.2011	13	48	159	57	72	182	0,1
16.05.2011	33	30	132	57	72	182	0,1
16.05.2011	24	56	163	60	119	217	0,1
16.05.2011	16	67	169	17	34	137	0,1
16.05.2011	17	34	137	23	21	117	0,1
16.05.2011	51	141	207	53	152	218	0,1
16.05.2011	51	141	207	54	199	238	0,1
16.05.2011	15	59	166	55	126	201	0,1
16.05.2011	51	141	207	55	126	201	0,1
16.05.2011	53	152	218	55	126	201	0,1
16.05.2011	13	48	159	30	42	151	0,1
16.05.2011	19	58	166	30	42	151	0,1
16.05.2011	27	46	152	30	42	151	0,1
16.05.2011	13	48	159	33	30	132	0,1
16.05.2011	19	58	166	33	30	132	0,1
16.05.2011	27	46	152	33	30	132	0,1
16.05.2011	30	42	151	33	30	132	0,1
16.05.2011	13	48	159	57	72	182	0,1
16.05.2011	19	58	166	57	72	182	0,1
16.05.2011	13	48	159	59	89	191	0,1
16.05.2011	19	58	166	59	89	191	0,1
16.05.2011	15	59	166	16	67	169	0,1
16.05.2011	16	67	169	17	34	137	0,1
16.05.2011	17	34	137	22	24	124	0,1
16.05.2011	22	24	124	23	21	117	0,1
16.05.2011	13	48	159	30	42	151	0,1
16.05.2011	13	48	159	33	30	132	0,1
16.05.2011	13	48	159	57	72	182	0,1
16.05.2011	30	42	151	57	72	182	0,1
16.05.2011	57	72	182	59	89	191	0,1
16.05.2011	15	59	166	53	152	218	0,1
16.05.2011	13	48	159	30	42	151	0,1
16.05.2011	13	48	159	57	72	182	0,1

17.05.2011	13	48	159	24	56	163	0,1
17.05.2011	13	48	159	33	30	132	0,1
17.05.2011	13	48	159	27	46	152	0,1
17.05.2011	16	67	169	17	34	137	0,1
17.05.2011	27	46	152	33	30	132	0,1
17.05.2011	30	42	151	33	30	132	0,1
17.05.2011	51	141	207	54	199	238	0,1
17.05.2011	22	24	124	51	141	207	0,1
17.05.2011	27	46	152	56	67	170	0,1
17.05.2011	56	67	170	57	72	182	0,1
17.05.2011	17	34	137	22	24	124	0,1
17.05.2011	24	56	163	33	30	132	0,1
17.05.2011	13	48	159	57	72	182	0,1
17.05.2011	27	46	152	57	72	182	0,1
17.05.2011	30	42	151	57	72	182	0,1
17.05.2011	13	48	159	59	89	191	0,1
17.05.2011	57	72	182	59	89	191	0,1
17.05.2011	57	72	182	60	119	217	0,1
17.05.2011	59	89	191	60	119	217	0,1
17.05.2011	24	56	163	33	30	132	0,1
17.05.2011	30	42	151	57	72	182	0,1
17.05.2011	30	42	151	59	89	191	0,1
18.05.2011	33	30	132	57	72	182	0,1
18.05.2011	13	48	159	60	119	217	0,1
18.05.2011	30	42	151	60	119	217	0,1
18.05.2011	56	67	170	60	119	217	0,1
18.05.2011	15	59	166	16	67	169	0,1
18.05.2011	16	67	169	17	34	137	0,1
18.05.2011	22	24	124	23	21	117	0,1
18.05.2011	14	87	198	55	126	201	0,1
18.05.2011	27	46	152	30	42	151	0,1
18.05.2011	30	42	151	56	67	170	0,1
18.05.2011	30	42	151	59	89	191	0,1

18.05.2011	22	24	124	55	126	201	0,1
18.05.2011	27	46	152	30	42	151	0,1
18.05.2011	13	48	159	33	30	132	0,1
25.05.2011	13	48	159	57	72	182	0,1
25.05.2011	30	42	151	57	72	182	0,1
25.05.2011	13	48	159	60	119	217	0,1
25.05.2011	30	42	151	60	119	217	0,1
25.05.2011	33	30	132	60	119	217	0,1
25.05.2011	57	72	182	60	119	217	0,1
25.05.2011	56	67	170	60	119	217	0,1
25.05.2011	57	72	182	60	119	217	0,1
25.05.2011	15	59	166	52	147	212	0,1
25.05.2011	30	42	151	57	72	182	0,1
25.05.2011	13	48	159	24	56	163	0,1
25.05.2011	24	56	163	57	72	182	0,1
25.05.2011	30	42	151	56	67	170	0,1
26.05.2011	24	56	163	27	46	152	0,1
26.05.2011	13	48	159	57	72	182	0,1
26.05.2011	30	42	151	57	72	182	0,1
26.05.2011	30	42	151	57	72	182	0,1
26.05.2011	24	56	163	27	46	152	0,1
26.05.2011	24	56	163	27	46	152	0,1
26.05.2011	13	48	159	56	67	170	0,1
27.05.2011	13	48	159	27	46	152	0,1
27.05.2011	13	48	159	33	30	132	0,1
27.05.2011	19	58	166	27	46	152	0,1
27.05.2011	24	56	163	27	46	152	0,1
27.05.2011	13	48	159	57	72	182	0,1
27.05.2011	19	58	166	24	56	163	0,1
01.06.2011	33	30	132	57	72	182	0,1
01.06.2011	27	46	152	33	30	132	0,1
03.06.2011	24	30	132	57	72	182	0,1
16.06.2011	13	123	195	48	81	168	0,1

16.06.2011	20	65	158	48	81	168	0,1
27.06.2011	20	65	158	48	81	168	0,1
16.05.2011	14	87	198	15	59	166	0,2
16.05.2011	15	59	166	22	24	124	0,2
16.05.2011	17	34	137	22	24	124	0,2
16.05.2011	13	48	159	60	119	217	0,2
16.05.2011	27	46	152	60	119	217	0,2
16.05.2011	33	30	132	60	119	217	0,2
16.05.2011	56	67	170	57	72	182	0,2
16.05.2011	15	59	166	54	199	238	0,2
16.05.2011	17	34	137	54	199	238	0,2
16.05.2011	22	24	124	54	199	238	0,2
16.05.2011	53	152	218	55	126	201	0,2
16.05.2011	54	199	238	55	126	201	0,2
16.05.2011	13	48	159	30	42	151	0,2
16.05.2011	30	42	151	33	30	132	0,2
16.05.2011	30	42	151	60	119	217	0,2
16.05.2011	57	72	182	60	119	217	0,2
16.05.2011	16	67	169	22	24	124	0,2
16.05.2011	16	67	169	23	21	117	0,2
16.05.2011	15	59	166	51	141	207	0,2
16.05.2011	15	59	166	53	152	218	0,2
16.05.2011	54	199	238	55	126	201	0,2
16.05.2011	27	46	152	57	72	182	0,2
16.05.2011	27	46	152	59	89	191	0,2
16.05.2011	57	72	182	60	119	217	0,2
16.05.2011	59	89	191	60	119	217	0,2
16.05.2011	14	87	198	16	67	169	0,2
16.05.2011	16	67	169	22	24	124	0,2
16.05.2011	17	34	137	23	21	117	0,2
16.05.2011	14	87	198	55	126	201	0,2
16.05.2011	15	59	166	55	126	201	0,2
16.05.2011	53	152	218	55	126	201	0,2

16.05.2011	19	58	166	27	46	152	0,2
16.05.2011	33	30	132	57	72	182	0,2
16.05.2011	13	48	159	59	89	191	0,2
16.05.2011	30	42	151	59	89	191	0,2
17.05.2011	17	34	137	23	21	117	0,2
17.05.2011	22	24	124	23	21	117	0,2
17.05.2011	23	21	117	55	126	201	0,2
17.05.2011	33	30	132	56	67	170	0,2
17.05.2011	24	56	163	57	72	182	0,2
17.05.2011	27	46	152	57	72	182	0,2
17.05.2011	56	67	170	59	89	191	0,2
17.05.2011	57	72	182	60	119	217	0,2
17.05.2011	59	89	191	60	119	217	0,2
17.05.2011	24	56	163	56	67	170	0,2
17.05.2011	27	46	152	59	89	191	0,2
17.05.2011	30	42	151	59	89	191	0,2
17.05.2011	13	48	159	60	119	217	0,2
17.05.2011	27	46	152	60	119	217	0,2
18.05.2011	14	87	198	23	21	117	0,2
18.05.2011	51	141	207	55	126	201	0,2
18.05.2011	19	58	166	27	46	152	0,2
18.05.2011	57	72	182	60	119	217	0,2
18.05.2011	15	59	166	17	34	137	0,2
18.05.2011	17	34	137	22	24	124	0,2
18.05.2011	13	48	159	24	56	163	0,2
18.05.2011	15	59	166	23	21	117	0,2
18.05.2011	16	67	169	23	21	117	0,2
18.05.2011	17	34	137	23	21	117	0,2
18.05.2011	22	24	124	54	199	238	0,2
18.05.2011	57	72	182	60	119	217	0,2
25.05.2011	19	58	166	24	56	163	0,2
25.05.2011	33	30	132	56	67	170	0,2
25.05.2011	13	48	159	57	72	182	0,2

25.05.2011	19	58	166	27	46	152	0,2
25.05.2011	33	30	132	57	72	182	0,2
25.05.2011	13	48	159	57	72	182	0,2
26.05.2011	13	48	159	30	42	151	0,2
26.05.2011	17	34	137	22	24	124	0,2
26.05.2011	13	48	159	30	42	151	0,2
26.05.2011	56	67	170	60	119	217	0,2
26.05.2011	57	72	182	60	119	217	0,2
26.05.2011	24	56	163	27	46	152	0,2
26.05.2011	30	42	151	57	72	182	0,2
26.05.2011	30	42	151	60	119	217	0,2
26.05.2011	14	87	198	23	21	117	0,2
26.05.2011	14	87	198	55	126	201	0,2
27.05.2011	27	46	152	30	42	151	0,2
27.05.2011	27	46	152	57	72	182	0,2
27.05.2011	30	42	151	33	30	132	0,2
27.05.2011	33	30	132	57	72	182	0,2
27.05.2011	19	58	166	60	119	217	0,2
27.05.2011	19	58	166	33	30	132	0,2
01.06.2011	24	56	163	33	30	132	0,2
02.06.2011	33	30	132	57	72	182	0,2
02.06.2011	24	56	163	27	46	152	0,2
03.06.2011	24	42	151	57	72	182	0,2
07.06.2011	19	58	166	27	46	152	0,2
16.06.2011	20	65	158	48	81	168	0,2
28.06.2011	20	65	158	48	81	168	0,2
16.05.2011	14	87	198	17	34	137	0,3
16.05.2011	14	87	198	55	126	201	0,3
16.05.2011	16	67	169	55	126	201	0,3
16.05.2011	51	141	207	55	126	201	0,3
16.05.2011	24	56	163	30	42	151	0,3
16.05.2011	30	42	151	57	72	182	0,3
16.05.2011	30	42	151	59	89	191	0,3

16.05.2011	15	59	166	54	199	238	0,3
16.05.2011	30	42	151	57	72	182	0,3
16.05.2011	33	30	132	57	72	182	0,3
16.05.2011	30	42	151	59	89	191	0,3
16.05.2011	33	30	132	59	89	191	0,3
16.05.2011	13	48	159	60	119	217	0,3
16.05.2011	19	58	166	60	119	217	0,3
16.05.2011	14	87	198	17	34	137	0,3
16.05.2011	15	59	166	17	34	137	0,3
16.05.2011	15	59	166	22	24	124	0,3
16.05.2011	16	67	169	23	21	117	0,3
16.05.2011	33	30	132	59	89	191	0,3
17.05.2011	24	56	163	33	30	132	0,3
17.05.2011	24	56	163	33	30	132	0,3
17.05.2011	19	58	166	57	72	182	0,3
17.05.2011	13	48	159	30	42	151	0,3
17.05.2011	24	56	163	59	89	191	0,3
17.05.2011	27	46	152	59	89	191	0,3
17.05.2011	56	67	170	60	119	217	0,3
17.05.2011	33	30	132	56	67	170	0,3
17.05.2011	30	42	151	60	119	217	0,3
17.05.2011	51	141	207	54	199	238	0,3
18.05.2011	15	59	166	22	24	124	0,3
18.05.2011	16	67	169	22	24	124	0,3
18.05.2011	15	59	166	51	141	207	0,3
18.05.2011	16	67	169	51	141	207	0,3
18.05.2011	24	56	163	33	30	132	0,3
18.05.2011	54	199	238	55	126	201	0,3
25.05.2011	30	42	151	60	119	217	0,3
25.05.2011	19	58	166	24	56	163	0,3
25.05.2011	57	72	182	60	119	217	0,3
25.05.2011	30	42	151	60	119	217	0,3
26.05.2011	24	56	163	33	30	132	0,3

26.05.2011	57	72	182	60	119	217	0,3
27.05.2011	14	87	198	23	21	117	0,3
27.05.2011	24	56	163	60	119	217	0,3
27.05.2011	24	56	163	27	46	152	0,3
27.05.2011	27	46	152	33	30	132	0,3
02.06.2011	27	58	166	33	71	182	0,3
16.06.2011	13	123	195	20	65	158	0,3
27.06.2011	20	65	158	48	81	168	0,3
16.05.2011	15	59	166	55	126	201	0,4
16.05.2011	17	34	137	55	126	201	0,4
16.05.2011	22	24	124	55	126	201	0,4
16.05.2011	19	58	166	27	46	152	0,4
16.05.2011	27	46	152	60	119	217	0,4
16.05.2011	30	42	151	60	119	217	0,4
16.05.2011	14	87	198	22	24	124	0,4
16.05.2011	14	87	198	53	152	218	0,4
16.05.2011	16	67	169	55	126	201	0,4
16.05.2011	24	56	163	33	30	132	0,4
16.05.2011	19	58	166	33	30	132	0,4
17.05.2011	19	58	166	56	67	170	0,4
17.05.2011	30	42	151	59	89	191	0,4
17.05.2011	23	21	117	55	126	201	0,4
17.05.2011	13	48	159	27	46	152	0,4
17.05.2011	13	48	159	30	42	151	0,4
17.05.2011	14	87	198	15	59	166	0,4
17.05.2011	14	87	198	17	34	137	0,4
17.05.2011	17	34	137	22	24	124	0,4
17.05.2011	22	24	124	55	126	201	0,4
17.05.2011	13	48	159	33	30	132	0,4
17.05.2011	24	56	163	60	119	217	0,4
17.05.2011	27	46	152	60	119	217	0,4
17.05.2011	15	59	166	22	24	124	0,4
18.05.2011	15	59	166	23	21	117	0,4

18.05.2011	16	67	169	23	21	117	0,4
18.05.2011	17	34	137	23	21	117	0,4
18.05.2011	17	34	137	51	141	207	0,4
18.05.2011	57	72	182	60	119	217	0,4
18.05.2011	13	48	159	24	56	163	0,4
18.05.2011	24	56	163	33	30	132	0,4
18.05.2011	56	67	170	57	72	182	0,4
25.05.2011	30	42	151	57	72	182	0,4
25.05.2011	30	42	151	60	119	217	0,4
25.05.2011	33	30	132	60	119	217	0,4
25.05.2011	13	48	159	30	42	151	0,4
25.05.2011	13	48	159	56	67	170	0,4
25.05.2011	56	67	170	60	119	217	0,4
26.05.2011	15	59	166	23	21	117	0,4
26.05.2011	16	67	169	55	126	201	0,4
26.05.2011	23	21	117	55	126	201	0,4
27.05.2011	14	87	198	17	34	137	0,4
27.05.2011	27	46	152	60	119	217	0,4
27.05.2011	19	58	166	27	46	152	0,4
02.06.2011	56	67	170	57	72	182	0,4
16.05.2011	33	30	132	60	119	217	0,5
16.05.2011	14	87	198	23	21	117	0,5
16.05.2011	15	59	166	23	21	117	0,5
16.05.2011	15	59	166	53	152	218	0,5
16.05.2011	17	34	137	55	126	201	0,5
17.05.2011	14	87	198	23	21	117	0,5
17.05.2011	13	48	159	33	30	132	0,5
17.05.2011	14	87	198	16	67	169	0,5
18.05.2011	22	24	124	51	141	207	0,5
18.05.2011	13	48	159	33	30	132	0,5
25.05.2011	33	30	132	58	71	182	0,5
25.05.2011	13	48	159	60	119	217	0,5
25.05.2011	30	42	151	33	30	132	0,5

26.05.2011	30	42	151	57	72	182	0,5
26.05.2011	27	46	152	33	30	132	0,5
26.05.2011	14	87	198	16	67	169	0,5
26.05.2011	13	48	159	27	46	152	0,5
07.06.2011	19	58	166	27	46	152	0,5
16.06.2011	28	53	146	35	50	138	0,5
27.06.2011	28	53	138	35	50	138	0,5
28.06.2011	28	53	138	35	50	138	0,5
16.05.2011	27	46	152	56	67	170	0,6
16.05.2011	16	67	169	53	152	218	0,6
16.05.2011	22	24	124	55	126	201	0,6
16.05.2011	17	34	137	22	24	124	0,6
16.05.2011	22	24	124	54	199	238	0,6
17.05.2011	27	46	152	57	72	182	0,6
17.05.2011	16	67	169	17	34	137	0,6
18.05.2011	23	21	117	51	141	207	0,6
18.05.2011	56	67	170	60	119	217	0,6
25.05.2011	13	48	159	58	71	182	0,6
25.05.2011	30	42	151	58	71	182	0,6
25.05.2011	57	72	182	58	71	182	0,6
25.05.2011	13	48	159	27	46	152	0,6
26.05.2011	19	58	166	27	46	152	0,6
26.05.2011	13	48	159	57	72	182	0,6
26.05.2011	14	87	198	16	67	169	0,6
26.05.2011	16	67	169	23	21	117	0,6
26.05.2011	27	46	152	56	67	170	0,6
01.06.2011	24	56	163	57	72	182	0,6
01.06.2011	24	56	163	56	67	170	0,6
15.06.2011	28	53	146	35	50	138	0,6
23.06.2011	13	123	195	20	65	158	0,6
24.06.2011	28	53	146	35	50	138	0,6
24.06.2011	28	53	146	35	50	138	0,6
27.06.2011	20	65	158	13	123	195	0,6

28.06.2011	20	65	158	48	81	168	0,6
16.05.2011	17	34	137	53	152	218	0,7
16.05.2011	23	21	117	55	126	201	0,7
17.05.2011	30	42	151	60	119	217	0,7
17.05.2011	22	24	124	55	126	201	0,7
17.05.2011	51	141	207	53	152	218	0,7
18.05.2011	16	67	169	22	24	124	0,7
25.05.2011	24	56	163	27	46	152	0,7
25.05.2011	33	30	132	56	67	170	0,7
25.05.2011	56	67	170	57	72	182	0,7
25.05.2011	58	71	182	60	119	217	0,7
25.05.2011	30	42	151	56	67	170	0,7
25.05.2011	30	42	151	57	72	182	0,7
26.05.2011	14	87	198	15	59	166	0,7
27.05.2011	17	34	137	23	21	117	0,7
02.06.2011	24	56	163	57	72	182	0,7
02.06.2011	24	56	163	56	67	170	0,7
03.06.2011	24	42	151	27	71	182	0,7
03.06.2011	24	67	170	56	71	182	0,7
06.06.2011	19	58	166	56	67	170	0,7
07.06.2011	16	67	169	51	141	207	0,7
16.05.2011	16	67	169	53	152	218	0,8
16.05.2011	16	67	169	54	199	238	0,8
16.05.2011	22	24	124	53	152	218	0,8
17.05.2011	59	89	191	60	119	217	0,8
17.05.2011	22	24	124	54	199	238	0,8
17.05.2011	51	141	207	55	126	201	0,8
25.05.2011	19	58	166	27	46	152	0,8
25.05.2011	13	48	159	56	67	170	0,8
25.05.2011	30	42	151	56	67	170	0,8
25.05.2011	56	67	170	58	71	182	0,8
25.05.2011	56	67	170	60	119	217	0,8
25.05.2011	30	42	151	60	119	217	0,8

25.05.2011	13	48	159	60	119	217	0,8
26.05.2011	56	67	170	57	72	182	0,8
26.05.2011	15	59	166	55	126	201	0,8
27.05.2011	30	42	151	56	67	170	0,8
02.06.2011	27	46	152	33	42	151	0,8
06.06.2011	19	58	166	27	46	152	0,8
06.06.2011	19	58	166	27	46	152	0,8
07.06.2011	33	30	132	56	67	170	0,8
07.06.2011	22	24	124	23	21	117	0,8
07.06.2011	33	30	132	56	67	170	0,8
17.06.2011	28	53	146	35	50	138	0,8
29.06.2011	20	65	158	48	81	168	0,8
16.05.2011	19	58	166	56	67	170	0,9
16.05.2011	16	67	169	51	141	207	0,9
16.05.2011	17	34	137	53	152	218	0,9
16.05.2011	17	34	137	54	199	238	0,9
16.05.2011	22	24	124	54	199	238	0,9
16.05.2011	16	67	169	55	126	201	0,9
16.05.2011	14	87	198	23	21	117	0,9
17.05.2011	24	56	163	30	42	151	0,9
17.05.2011	51	141	207	54	199	238	0,9
17.05.2011	54	199	238	55	126	201	0,9
17.05.2011	19	58	166	33	30	132	0,9
18.05.2011	30	42	151	56	67	170	0,9
18.05.2011	27	46	152	59	89	191	0,9
18.05.2011	30	42	151	59	89	191	0,9
25.05.2011	22	24	124	23	21	117	0,9
26.05.2011	30	42	151	56	67	170	0,9
26.05.2011	27	46	152	33	30	132	0,9
26.05.2011	15	59	166	16	67	169	0,9
26.05.2011	33	30	132	56	67	170	0,9
26.05.2011	33	30	132	57	72	182	0,9
27.05.2011	22	24	124	55	126	201	0,9

02.06.2011	27	46	152	56	67	170	0,9
07.06.2011	19	58	166	27	46	152	0,9
07.06.2011	33	30	132	56	67	170	0,9
16.06.2011	13	123	195	48	81	168	0,9
16.05.2011	17	34	137	51	141	207	1,0
16.05.2011	22	24	124	53	152	218	1,0
16.05.2011	23	21	117	54	199	238	1,0
16.05.2011	17	34	137	55	126	201	1,0
16.05.2011	23	21	117	53	152	218	1,0
16.05.2011	57	72	182	60	119	217	1,0
16.05.2011	59	89	191	60	119	217	1,0
16.05.2011	13	48	159	30	42	151	1,0
17.05.2011	14	87	198	17	34	137	1,0
17.05.2011	19	58	166	24	56	163	1,0
18.05.2011	16	67	169	17	34	137	1,0
18.05.2011	27	46	152	57	72	182	1,0
18.05.2011	30	42	151	57	72	182	1,0
18.05.2011	56	67	170	57	72	182	1,0
18.05.2011	57	72	182	59	89	191	1,0
18.05.2011	27	46	152	56	67	170	1,0
25.05.2011	14	87	198	22	24	124	1,0
25.05.2011	27	46	152	30	42	151	1,0
25.05.2011	27	46	152	56	67	170	1,0
26.05.2011	27	46	152	33	30	132	1,0
26.05.2011	19	58	166	24	56	163	1,0
26.05.2011	24	56	163	33	30	132	1,0
26.05.2011	13	48	159	33	30	132	1,0
27.05.2011	17	34	137	55	126	201	1,0
07.06.2011	15	59	166	23	21	117	1,0
07.06.2011	15	59	166	53	152	218	1,0
15.06.2011	28	53	146	35	50	138	1,0
27.06.2011	28	53	138	35	50	138	1,0
16.05.2011	15	59	166	16	67	169	1,1

16.05.2011	22	24	124	51	141	207	1,1
16.05.2011	23	21	117	51	141	207	1,1
16.05.2011	23	21	117	53	152	218	1,1
16.05.2011	22	24	124	55	126	201	1,1
16.05.2011	13	48	159	60	119	217	1,1
16.05.2011	30	42	151	60	119	217	1,1
17.05.2011	13	48	159	30	42	151	1,1
17.05.2011	27	46	152	30	42	151	1,1
17.05.2011	14	87	198	22	24	124	1,1
26.05.2011	24	56	163	33	30	132	1,1
26.05.2011	19	58	166	27	46	152	1,1
26.05.2011	24	56	163	33	30	132	1,1
26.05.2011	27	46	152	33	30	132	1,1
02.06.2011	24	56	163	57	72	182	1,1
03.06.2011	15	59	166	16	67	169	1,1
07.06.2011	23	21	117	53	152	218	1,1
16.06.2011	28	53	146	35	50	138	1,1
22.06.2011	20	65	158	48	81	168	1,1
27.06.2011	13	123	195	20	65	158	1,1
27.06.2011	13	123	195	48	81	168	1,1
16.05.2011	14	87	198	16	67	169	1,2
16.05.2011	15	59	166	17	34	137	1,2
16.05.2011	15	59	166	22	24	124	1,2
16.05.2011	23	21	117	55	126	201	1,2
16.05.2011	33	30	132	60	119	217	1,2
17.05.2011	51	141	207	54	199	238	1,2
18.05.2011	56	67	170	59	89	191	1,2
26.05.2011	33	30	132	60	119	217	1,2
26.05.2011	16	67	169	53	152	218	1,2
01.06.2011	27	46	152	56	67	170	1,2
01.06.2011	33	30	132	56	67	170	1,2
02.06.2011	33	30	132	56	67	170	1,2
02.06.2011	16	67	169	53	152	218	1,2

06.06.2011	19	58	166	56	67	170	1,2
15.06.2011	13	123	195	48	81	168	1,2
16.06.2011	28	53	146	35	50	138	1,2
16.05.2011	14	87	198	17	34	137	1,3
16.05.2011	14	87	198	22	24	124	1,3
16.05.2011	14	87	198	23	21	117	1,3
16.05.2011	15	59	166	23	21	117	1,3
16.05.2011	16	67	169	17	34	137	1,3
17.05.2011	15	59	166	55	126	201	1,3
18.05.2011	27	46	152	60	119	217	1,3
18.05.2011	56	67	170	60	119	217	1,3
18.05.2011	59	89	191	60	119	217	1,3
18.05.2011	30	42	151	57	72	182	1,3
25.05.2011	56	67	170	60	119	217	1,3
25.05.2011	27	46	152	60	119	217	1,3
26.05.2011	22	24	124	53	152	218	1,3
27.05.2011	17	34	137	55	126	201	1,3
02.06.2011	27	46	152	57	72	182	1,3
06.06.2011	27	46	152	33	30	132	1,3
07.06.2011	16	67	169	52	147	212	1,3
15.06.2011	13	123	195	48	81	168	1,3
22.06.2011	28	53	146	35	50	138	1,3
16.05.2011	14	87	198	53	152	218	1,4
16.05.2011	14	87	198	54	199	238	1,4
17.05.2011	14	87	198	15	59	166	1,4
18.05.2011	30	42	151	60	119	217	1,4
18.05.2011	27	46	152	57	72	182	1,4
26.05.2011	19	58	166	24	56	163	1,4
26.05.2011	19	58	166	27	46	152	1,4
26.05.2011	19	58	166	27	46	152	1,4
26.05.2011	17	34	137	55	126	201	1,4
02.06.2011	24	58	166	56	42	151	1,4
02.06.2011	19	58	166	57	72	182	1,4

07.06.2011	15	59	166	23	21	117	1,4
15.06.2011	28	53	146	35	50	138	1,4
16.06.2011	13	123	195	48	81	168	1,4
16.06.2011	28	53	146	35	50	138	1,4
16.05.2011	14	87	198	15	59	166	1,5
16.05.2011	14	87	198	51	141	207	1,5
16.05.2011	14	87	198	55	126	201	1,5
17.05.2011	19	58	166	33	30	132	1,5
18.05.2011	30	42	151	60	119	217	1,5
26.05.2011	17	34	137	53	152	218	1,5
26.05.2011	19	58	166	24	56	163	1,5
26.05.2011	19	58	166	33	30	132	1,5
26.05.2011	27	46	152	33	30	132	1,5
27.05.2011	30	42	151	60	119	217	1,5
27.05.2011	14	87	198	23	21	117	1,5
27.05.2011	23	21	117	55	126	201	1,5
06.06.2011	33	30	132	56	67	170	1,5
06.06.2011	22	24	124	53	152	218	1,5
07.06.2011	19	58	166	27	46	152	1,5
17.05.2011	13	48	159	19	58	166	1,6
18.05.2011	57	72	182	59	89	191	1,6
18.05.2011	27	46	152	60	119	217	1,6
26.05.2011	24	56	163	33	30	132	1,6
26.05.2011	14	87	198	55	126	201	1,6
27.05.2011	13	48	159	60	119	217	1,6
27.05.2011	27	46	152	60	119	217	1,6
27.05.2011	33	30	132	60	119	217	1,6
27.05.2011	57	72	182	60	119	217	1,6
07.06.2011	24	56	163	27	46	152	1,6
28.06.2011	28	53	138	35	50	138	1,6
16.05.2011	57	72	182	60	119	217	1,7
17.05.2011	24	56	163	60	119	217	1,7
17.05.2011	14	87	198	55	126	201	1,7

18.05.2011	17	34	137	22	24	124	1,7
18.05.2011	59	89	191	60	119	217	1,7
25.05.2011	16	67	169	51	141	207	1,7
25.05.2011	56	67	170	58	71	182	1,7
27.05.2011	14	87	198	55	126	201	1,7
01.06.2011	56	67	170	57	72	182	1,7
02.06.2011	19	58	166	24	56	163	1,7
02.06.2011	22	24	124	51	141	207	1,7
06.06.2011	15	59	166	51	141	207	1,7
16.06.2011	13	123	195	48	81	168	1,7
16.05.2011	56	67	170	60	119	217	1,8
17.05.2011	19	58	166	24	56	163	1,8
17.05.2011	24	56	163	30	42	151	1,8
17.05.2011	33	30	132	56	67	170	1,8
17.05.2011	24	56	163	56	67	170	1,8
17.05.2011	24	56	163	59	89	191	1,8
25.05.2011	19	58	166	24	56	163	1,8
26.05.2011	13	48	159	30	42	151	1,8
26.05.2011	13	48	159	57	72	182	1,8
26.05.2011	19	58	166	33	30	132	1,8
26.05.2011	56	67	170	57	72	182	1,8
02.06.2011	15	59	166	16	67	169	1,8
02.06.2011	51	141	207	53	152	218	1,8
02.06.2011	19	58	166	56	67	170	1,8
02.06.2011	23	21	117	53	152	218	1,8
07.06.2011	14	87	198	15	59	166	1,8
16.05.2011	13	48	159	57	72	182	1,9
16.05.2011	27	46	152	57	72	182	1,9
16.05.2011	33	30	132	57	72	182	1,9
17.05.2011	13	48	159	60	119	217	1,9
17.05.2011	16	67	169	17	34	137	1,9
26.05.2011	56	67	170	60	119	217	1,9
26.05.2011	17	34	137	22	24	124	1,9

26.05.2011	24	56	163	27	46	152	1,9
26.05.2011	13	48	159	57	72	182	1,9
27.05.2011	17	34	137	55	126	201	1,9
27.05.2011	51	141	207	52	147	212	1,9
27.05.2011	13	48	159	56	67	170	1,9
01.06.2011	15	59	166	22	24	124	1,9
16.06.2011	28	53	146	35	50	138	1,9
22.06.2011	28	53	146	35	50	138	1,9
28.06.2011	28	53	138	35	50	138	1,9
16.05.2011	13	48	159	56	67	170	2,0
16.05.2011	27	46	152	56	67	170	2,0
16.05.2011	33	30	132	56	67	170	2,0
17.05.2011	13	48	159	30	42	151	2,0
17.05.2011	13	48	159	56	67	170	2,0
17.05.2011	33	30	132	60	119	217	2,0
17.05.2011	30	42	151	59	89	191	2,0
17.05.2011	14	87	198	16	67	169	2,0
17.05.2011	16	67	169	22	24	124	2,0
25.05.2011	24	56	163	27	46	152	2,0
25.05.2011	19	58	166	33	30	132	2,0
26.05.2011	19	58	166	24	56	163	2,0
26.05.2011	15	59	166	17	34	137	2,0
26.05.2011	53	152	218	55	126	201	2,0
26.05.2011	27	46	152	33	30	132	2,0
27.05.2011	23	21	117	55	126	201	2,0
01.06.2011	24	56	163	56	67	170	2,0
02.06.2011	33	56	163	57	67	170	2,0
17.05.2011	30	42	151	33	30	132	2,1
17.05.2011	24	56	163	56	67	170	2,1
17.05.2011	23	21	117	54	199	238	2,1
17.05.2011	23	21	117	55	126	201	2,1
25.05.2011	27	46	152	33	30	132	2,1
26.05.2011	23	21	117	55	126	201	2,1

26.05.2011	19	58	166	56	67	170	2,1
26.05.2011	56	67	170	60	119	217	2,1
01.06.2011	56	67	170	57	72	182	2,1
02.06.2011	27	58	166	56	30	132	2,1
06.06.2011	19	58	166	56	67	170	2,1
06.06.2011	27	46	152	56	67	170	2,1
28.06.2011	13	123	195	20	65	158	2,1
16.05.2011	15	59	166	54	199	238	2,2
16.05.2011	17	34	137	54	199	238	2,2
17.05.2011	24	56	163	59	89	191	2,2
18.05.2011	14	87	198	52	147	212	2,2
25.05.2011	27	46	152	56	67	170	2,2
26.05.2011	33	30	132	57	72	182	2,2
26.05.2011	13	48	159	60	119	217	2,2
27.05.2011	15	59	166	53	152	218	2,2
27.05.2011	22	24	124	23	21	117	2,2
02.06.2011	19	58	166	56	56	163	2,2
02.06.2011	14	87	198	23	21	117	2,2
06.06.2011	24	56	163	57	72	182	2,2
06.06.2011	19	58	166	33	30	132	2,2
07.06.2011	33	30	132	56	67	170	2,2
16.06.2011	28	53	146	35	50	138	2,2
28.06.2011	20	65	158	48	81	168	2,2
16.05.2011	14	87	198	54	199	238	2,3
16.05.2011	22	24	124	54	199	238	2,3
17.05.2011	15	59	166	53	152	218	2,3
17.05.2011	27	46	152	57	72	182	2,3
18.05.2011	52	147	212	55	126	201	2,3
25.05.2011	24	56	163	33	30	132	2,3
25.05.2011	24	56	163	56	67	170	2,3
26.05.2011	19	58	166	33	30	132	2,3
27.05.2011	17	34	137	23	21	117	2,3
27.05.2011	14	87	198	55	126	201	2,3

01.06.2011	33	30	132	56	67	170	2,3
02.06.2011	19	58	166	24	56	163	2,3
02.06.2011	19	58	166	33	30	132	2,3
07.06.2011	27	46	152	33	30	132	2,3
16.06.2011	13	123	195	48	81	168	2,3
16.05.2011	14	87	198	55	126	201	2,4
17.05.2011	13	48	159	59	89	191	2,4
26.05.2011	33	30	132	56	67	170	2,4
26.05.2011	27	46	152	57	72	182	2,4
01.06.2011	27	46	152	33	30	132	2,4
02.06.2011	19	58	166	24	56	163	2,4
06.06.2011	27	46	152	33	30	132	2,4
16.05.2011	15	59	166	55	126	201	2,5
16.05.2011	22	24	124	55	126	201	2,5
16.05.2011	19	58	166	24	56	163	2,5
16.05.2011	19	58	166	57	72	182	2,5
16.05.2011	19	58	166	59	89	191	2,5
17.05.2011	33	30	132	59	89	191	2,5
18.05.2011	19	58	166	33	30	132	2,5
18.05.2011	13	48	159	19	58	166	2,5
25.05.2011	19	58	166	33	30	132	2,5
25.05.2011	19	58	166	56	67	170	2,5
25.05.2011	30	42	151	33	30	132	2,5
25.05.2011	13	48	159	60	119	217	2,5
26.05.2011	24	56	163	27	46	152	2,5
26.05.2011	13	48	159	56	67	170	2,5
02.06.2011	27	58	166	33	30	132	2,5
16.05.2011	17	34	137	55	126	201	2,6
16.05.2011	13	48	159	19	58	166	2,6
16.05.2011	19	58	166	33	30	132	2,6
16.05.2011	19	58	166	60	119	217	2,6
17.05.2011	24	56	163	30	42	151	2,6
18.05.2011	19	58	166	33	30	132	2,6

25.05.2011	24	56	163	60	119	217	2,6
27.05.2011	14	87	198	17	34	137	2,6
27.05.2011	14	87	198	22	24	124	2,6
27.05.2011	13	48	159	30	42	151	2,6
27.05.2011	27	46	152	60	119	217	2,6
02.06.2011	24	56	163	33	42	151	2,6
02.06.2011	19	58	166	27	46	152	2,6
03.06.2011	22	24	124	23	21	117	2,6
06.06.2011	14	87	198	53	152	218	2,6
07.06.2011	14	87	198	53	152	218	2,6
15.06.2011	28	53	146	35	50	138	2,6
27.06.2011	28	53	138	35	50	138	2,6
16.05.2011	19	58	166	30	42	151	2,7
16.05.2011	13	48	159	27	46	152	2,7
16.05.2011	24	56	163	27	46	152	2,7
16.05.2011	27	46	152	57	72	182	2,7
16.05.2011	27	46	152	59	89	191	2,7
16.05.2011	27	46	152	59	89	191	2,7
16.05.2011	13	48	159	59	89	191	2,7
17.05.2011	22	24	124	23	21	117	2,7
18.05.2011	13	48	159	19	58	166	2,7
18.05.2011	19	58	166	24	56	163	2,7
25.05.2011	57	72	182	60	119	217	2,7
26.05.2011	13	48	159	33	30	132	2,7
26.05.2011	30	42	151	33	30	132	2,7
26.05.2011	27	46	152	60	119	217	2,7
01.06.2011	24	56	163	27	46	152	2,7
01.06.2011	24	56	163	33	30	132	2,7
02.06.2011	24	56	163	27	46	152	2,7
22.06.2011	28	53	146	35	50	138	2,7
27.06.2011	13	123	195	20	65	158	2,7
16.05.2011	17	34	137	51	141	207	2,8
16.05.2011	19	58	166	30	42	151	2,8

16.05.2011	27	46	152	33	30	132	2,8
16.05.2011	27	46	152	60	119	217	2,8
16.05.2011	13	48	159	27	46	152	2,8
16.05.2011	27	46	152	30	42	151	2,8
16.05.2011	27	46	152	33	30	132	2,8
16.05.2011	27	46	152	57	72	182	2,8
17.05.2011	23	21	117	51	141	207	2,8
18.05.2011	19	58	166	24	56	163	2,8
26.05.2011	17	34	137	52	147	212	2,8
01.06.2011	23	21	117	52	147	212	2,8
01.06.2011	24	56	163	33	30	132	2,8
02.06.2011	33	58	166	56	67	170	2,8
06.06.2011	15	59	166	23	21	117	2,8
07.06.2011	19	58	166	27	46	152	2,8
29.06.2011	20	65	158	48	81	168	2,8
16.05.2011	15	59	166	51	141	207	2,9
16.05.2011	27	46	152	30	42	151	2,9
16.05.2011	13	48	159	19	58	166	2,9
16.05.2011	19	58	166	30	42	151	2,9
16.05.2011	19	58	166	57	72	182	2,9
16.05.2011	19	58	166	59	89	191	2,9
16.05.2011	19	58	166	60	119	217	2,9
16.05.2011	27	46	152	60	119	217	2,9
17.05.2011	15	59	166	16	67	169	2,9
27.05.2011	24	56	163	60	119	217	2,9
27.05.2011	33	30	132	60	119	217	2,9
16.05.2011	14	87	198	51	141	207	3,0
16.05.2011	22	24	124	51	141	207	3,0
16.05.2011	19	58	166	33	30	132	3,0
16.05.2011	30	42	151	59	89	191	3,0
25.05.2011	16	67	169	17	34	137	3,0
25.05.2011	23	21	117	53	152	218	3,0
25.05.2011	33	30	132	57	72	182	3,0

27.05.2011	19	58	166	60	119	217	3,0
02.06.2011	16	67	169	23	21	117	3,0
02.06.2011	19	58	166	57	72	182	3,0
06.06.2011	14	87	198	22	24	124	3,0
07.06.2011	22	24	124	23	21	117	3,0
27.06.2011	13	123	195	48	81	168	3,0
16.05.2011	56	67	170	57	72	182	3,1
25.05.2011	17	34	137	23	21	117	3,1
25.05.2011	14	87	198	22	24	124	3,1
25.05.2011	33	30	132	56	67	170	3,1
25.05.2011	27	46	152	30	42	151	3,1
26.05.2011	56	67	170	57	72	182	3,1
02.06.2011	33	46	152	56	67	170	3,1
07.06.2011	16	67	169	22	24	124	3,1
16.05.2011	13	48	159	56	67	170	3,2
16.05.2011	24	56	163	56	67	170	3,2
16.05.2011	33	30	132	56	67	170	3,2
16.05.2011	56	67	170	59	89	191	3,2
25.05.2011	33	30	132	60	119	217	3,2
26.05.2011	17	34	137	55	126	201	3,2
27.05.2011	23	21	117	53	152	218	3,2
01.06.2011	24	56	163	56	67	170	3,2
03.06.2011	24	67	170	27	72	182	3,2
16.05.2011	54	199	238	55	126	201	3,3
16.05.2011	56	67	170	60	119	217	3,3
17.05.2011	22	24	124	53	152	218	3,3
26.05.2011	13	48	159	33	30	132	3,3
01.06.2011	27	46	152	57	72	182	3,3
01.06.2011	33	30	132	57	72	182	3,3
01.06.2011	24	56	163	56	67	170	3,3
01.06.2011	24	56	163	57	72	182	3,3
03.06.2011	27	67	170	57	71	182	3,3
16.05.2011	30	42	151	56	67	170	3,4

17.05.2011	19	58	166	60	119	217	3,4
17.05.2011	23	21	117	53	152	218	3,4
18.05.2011	13	48	159	59	89	191	3,4
18.05.2011	30	42	151	59	89	191	3,4
18.05.2011	56	67	170	59	89	191	3,4
18.05.2011	57	72	182	59	89	191	3,4
25.05.2011	17	34	137	55	126	201	3,4
27.05.2011	27	46	152	33	30	132	3,4
02.06.2011	16	67	169	52	147	212	3,4
07.06.2011	16	67	169	22	24	124	3,4
07.06.2011	24	56	163	57	72	182	3,4
15.06.2011	28	53	146	35	50	138	3,4
23.06.2011	28	53	146	35	50	138	3,4
17.05.2011	14	87	198	53	152	218	3,5
17.05.2011	53	152	218	55	126	201	3,5
18.05.2011	33	30	132	59	89	191	3,5
18.05.2011	59	89	191	60	119	217	3,5
25.05.2011	13	48	159	56	67	170	3,5
26.05.2011	24	56	163	56	67	170	3,5
26.05.2011	30	42	151	56	67	170	3,5
26.05.2011	27	46	152	56	67	170	3,5
27.05.2011	24	56	163	33	30	132	3,5
02.06.2011	56	42	151	57	67	170	3,5
03.06.2011	27	42	151	57	71	182	3,5
17.05.2011	19	58	166	30	42	151	3,6
17.05.2011	17	34	137	53	152	218	3,6
25.05.2011	27	46	152	33	30	132	3,6
25.05.2011	24	56	163	56	67	170	3,6
26.05.2011	19	58	166	33	30	132	3,6
26.05.2011	24	56	163	56	67	170	3,6
27.05.2011	19	58	166	33	30	132	3,6
01.06.2011	14	87	198	15	59	166	3,6
01.06.2011	27	46	152	57	72	182	3,6

02.06.2011	27	46	152	57	72	182	3,6
03.06.2011	24	30	132	27	72	182	3,6
06.06.2011	24	56	163	57	72	182	3,6
07.06.2011	30	42	151	58	71	182	3,6
16.05.2011	51	141	207	54	199	238	3,7
17.05.2011	51	141	207	54	199	238	3,7
17.05.2011	56	67	170	60	119	217	3,7
18.05.2011	51	141	207	53	152	218	3,7
25.05.2011	56	67	170	57	72	182	3,7
26.05.2011	13	48	159	56	67	170	3,7
26.05.2011	14	87	198	51	141	207	3,7
26.05.2011	33	30	132	56	67	170	3,7
01.06.2011	14	87	198	53	152	218	3,7
02.06.2011	24	56	163	57	42	151	3,7
06.06.2011	16	67	169	52	147	212	3,7
07.06.2011	19	58	166	57	72	182	3,7
18.05.2011	24	56	163	59	89	191	3,8
26.05.2011	24	56	163	57	72	182	3,8
26.05.2011	13	48	159	27	46	152	3,8
27.05.2011	33	30	132	60	119	217	3,8
01.06.2011	56	67	170	57	72	182	3,8
17.05.2011	30	42	151	56	67	170	3,9
17.05.2011	16	67	169	53	152	218	3,9
17.05.2011	52	147	212	55	126	201	3,9
26.05.2011	13	48	159	24	56	163	3,9
02.06.2011	24	56	163	27	46	152	3,9
02.06.2011	14	87	198	53	152	218	3,9
16.05.2011	24	56	163	57	72	182	4,0
16.05.2011	17	34	137	23	21	117	4,0
16.05.2011	22	24	124	23	21	117	4,0
17.05.2011	15	59	166	16	67	169	4,0
17.05.2011	19	58	166	59	89	191	4,0
17.05.2011	27	46	152	59	89	191	4,0

26.05.2011	13	48	159	30	42	151	4,0
01.06.2011	14	87	198	52	147	212	4,0
16.05.2011	23	21	117	54	199	238	4,1
17.05.2011	57	72	182	59	89	191	4,1
26.05.2011	27	46	152	33	30	132	4,1
26.05.2011	14	87	198	17	34	137	4,1
26.05.2011	30	42	151	57	72	182	4,1
26.05.2011	30	42	151	60	119	217	4,1
02.06.2011	27	56	163	57	30	132	4,1
02.06.2011	24	58	166	27	30	132	4,1
16.05.2011	24	56	163	56	67	170	4,2
26.05.2011	13	48	159	24	56	163	4,2
26.05.2011	24	56	163	30	42	151	4,2
26.05.2011	13	48	159	30	42	151	4,2
27.05.2011	14	87	198	23	21	117	4,2
06.06.2011	15	59	166	51	141	207	4,2
07.06.2011	27	46	152	57	72	182	4,2
27.06.2011	28	53	138	35	50	138	4,2
16.05.2011	13	48	159	24	56	163	4,3
16.05.2011	24	56	163	33	30	132	4,3
17.05.2011	56	67	170	59	89	191	4,3
25.05.2011	30	42	151	33	30	132	4,3
25.05.2011	30	42	151	56	67	170	4,3
26.05.2011	27	46	152	30	42	151	4,3
26.05.2011	27	46	152	57	72	182	4,3
01.06.2011	15	59	166	23	21	117	4,3
01.06.2011	33	30	132	57	72	182	4,3
02.06.2011	14	87	198	15	59	166	4,3
02.06.2011	24	46	152	27	42	151	4,3
03.06.2011	52	147	212	53	152	218	4,3
29.06.2011	28	53	138	35	50	138	4,3
16.05.2011	24	56	163	27	46	152	4,4
16.05.2011	24	56	163	60	119	217	4,4

17.05.2011	27	46	152	30	42	151	4,4
17.05.2011	16	67	169	17	34	137	4,4
17.05.2011	16	67	169	23	21	117	4,4
18.05.2011	13	48	159	24	56	163	4,4
18.05.2011	24	56	163	30	42	151	4,4
18.05.2011	24	56	163	33	30	132	4,4
18.05.2011	24	56	163	56	67	170	4,4
18.05.2011	24	56	163	57	72	182	4,4
18.05.2011	24	56	163	60	119	217	4,4
26.05.2011	22	24	124	53	152	218	4,4
26.05.2011	17	34	137	55	126	201	4,4
06.06.2011	51	141	207	53	152	218	4,4
06.06.2011	16	67	169	52	147	212	4,4
16.05.2011	59	89	191	60	119	217	4,5
17.05.2011	30	42	151	57	72	182	4,5
17.05.2011	27	46	152	60	119	217	4,5
17.05.2011	57	72	182	60	119	217	4,5
25.05.2011	56	67	170	60	119	217	4,5
02.06.2011	24	58	166	33	67	170	4,5
06.06.2011	33	30	132	56	67	170	4,5
22.06.2011	28	53	146	35	50	138	4,5
25.05.2011	33	30	132	57	72	182	4,6
25.05.2011	56	67	170	57	72	182	4,6
25.05.2011	33	30	132	60	119	217	4,6
26.05.2011	30	42	151	60	119	217	4,6
27.05.2011	14	87	198	15	59	166	4,6
01.06.2011	19	58	166	56	67	170	4,6
02.06.2011	27	46	152	33	30	132	4,6
02.06.2011	27	46	152	57	72	182	4,6
06.06.2011	24	56	163	57	72	182	4,6
06.06.2011	19	58	166	27	46	152	4,6
07.06.2011	19	58	166	27	46	152	4,6
16.05.2011	14	87	198	17	34	137	4,7

16.05.2011	14	87	198	22	24	124	4,7
16.05.2011	14	87	198	54	199	238	4,7
16.05.2011	19	58	166	24	56	163	4,7
17.05.2011	15	59	166	17	34	137	4,7
18.05.2011	22	24	124	52	147	212	4,7
25.05.2011	24	56	163	27	46	152	4,7
06.06.2011	19	58	166	57	72	182	4,7
07.06.2011	33	30	132	56	67	170	4,7
16.05.2011	13	48	159	30	42	151	4,8
16.05.2011	27	46	152	30	42	151	4,8
16.05.2011	30	42	151	33	30	132	4,8
17.05.2011	14	87	198	22	24	124	4,8
17.05.2011	14	87	198	15	59	166	4,8
17.05.2011	15	59	166	22	24	124	4,8
17.05.2011	14	87	198	17	34	137	4,8
17.05.2011	14	87	198	23	21	117	4,8
25.05.2011	15	59	166	52	147	212	4,8
26.05.2011	30	42	151	57	72	182	4,8
01.06.2011	19	58	166	33	30	132	4,8
02.06.2011	19	58	166	33	46	152	4,8
02.06.2011	27	46	152	57	72	182	4,8
06.06.2011	27	46	152	57	72	182	4,8
16.05.2011	24	56	163	33	30	132	4,9
17.05.2011	15	59	166	22	24	124	4,9
17.05.2011	17	34	137	22	24	124	4,9
17.05.2011	24	56	163	60	119	217	4,9
17.05.2011	57	72	182	60	119	217	4,9
25.05.2011	19	58	166	27	46	152	4,9
26.05.2011	27	46	152	56	67	170	4,9
26.05.2011	13	48	159	19	58	166	4,9
02.06.2011	24	56	163	57	72	182	4,9
16.05.2011	30	42	151	60	119	217	5,0
17.05.2011	30	42	151	60	119	217	5,0

26.05.2011	13	48	159	27	46	152	5,0
02.06.2011	14	87	198	53	152	218	5,0
03.06.2011	51	141	207	52	147	212	5,0
16.05.2011	56	67	170	57	72	182	5,1
18.05.2011	16	67	169	52	147	212	5,1
02.06.2011	14	87	198	16	67	169	5,1
06.06.2011	23	21	117	53	152	218	5,1
07.06.2011	19	58	166	33	30	132	5,1
18.05.2011	52	147	212	53	152	218	5,2
26.05.2011	30	42	151	56	67	170	5,2
01.06.2011	33	30	132	56	67	170	5,2
17.05.2011	27	46	152	60	119	217	5,3
25.05.2011	16	67	169	53	152	218	5,3
26.05.2011	13	48	159	56	67	170	5,3
26.05.2011	56	67	170	57	72	182	5,3
02.06.2011	27	56	163	56	30	132	5,3
15.06.2011	28	53	146	35	50	138	5,3
16.05.2011	51	141	207	55	126	201	5,4
27.05.2011	15	59	166	23	21	117	5,4
07.06.2011	16	67	169	23	21	117	5,4
18.05.2011	17	34	137	52	147	212	5,5
26.05.2011	19	58	166	56	67	170	5,5
26.05.2011	19	58	166	57	72	182	5,5
02.06.2011	56	67	170	57	72	182	5,5
02.06.2011	15	59	166	22	24	124	5,5
03.06.2011	19	42	151	33	30	132	5,5
06.06.2011	24	56	163	33	30	132	5,5
07.06.2011	24	56	163	57	72	182	5,5
07.06.2011	22	24	124	23	21	117	5,5
23.06.2011	28	53	146	35	50	138	5,5
16.05.2011	33	30	132	56	67	170	5,6
16.05.2011	22	24	124	23	21	117	5,6
17.05.2011	16	67	169	53	152	218	5,6

17.05.2011	14	87	198	22	24	124	5,6
17.05.2011	22	24	124	23	21	117	5,6
17.05.2011	22	24	124	55	126	201	5,6
25.05.2011	27	46	152	60	119	217	5,6
26.05.2011	30	42	151	56	67	170	5,6
26.05.2011	30	42	151	57	72	182	5,6
02.06.2011	19	58	166	57	46	152	5,6
02.06.2011	19	58	166	27	56	163	5,6
02.06.2011	14	87	198	53	152	218	5,6
06.06.2011	14	87	198	23	21	117	5,6
06.06.2011	24	56	163	27	46	152	5,6
07.06.2011	33	30	132	56	67	170	5,6
25.05.2011	24	56	163	33	30	132	5,7
26.05.2011	30	42	151	60	119	217	5,7
26.05.2011	30	42	151	33	30	132	5,7
27.05.2011	14	87	198	53	152	218	5,7
01.06.2011	27	46	152	56	67	170	5,7
01.06.2011	27	46	152	57	72	182	5,7
02.06.2011	22	24	124	23	21	117	5,7
03.06.2011	22	24	124	23	21	117	5,7
06.06.2011	24	56	163	27	46	152	5,7
25.05.2011	17	34	137	53	152	218	5,8
25.05.2011	53	152	218	55	126	201	5,8
16.05.2011	16	67	169	51	141	207	5,9
16.05.2011	24	56	163	30	42	151	5,9
16.05.2011	24	56	163	56	67	170	5,9
25.05.2011	27	46	152	57	72	182	5,9
26.05.2011	13	48	159	19	58	166	5,9
26.05.2011	19	58	166	30	42	151	5,9
26.05.2011	23	21	117	53	152	218	5,9
26.05.2011	24	56	163	57	72	182	5,9
01.06.2011	33	30	132	57	72	182	5,9
01.06.2011	19	58	166	27	46	152	5,9

02.06.2011	19	58	166	33	46	152	5,9
03.06.2011	16	67	169	53	152	218	5,9
06.06.2011	19	58	166	27	46	152	5,9
28.06.2011	13	123	195	48	81	168	5,9
16.05.2011	14	87	198	22	24	124	6,0
17.05.2011	22	24	124	51	141	207	6,0
26.05.2011	27	46	152	56	67	170	6,0
26.05.2011	17	34	137	53	152	218	6,0
26.05.2011	24	56	163	30	42	151	6,0
26.05.2011	30	42	151	33	30	132	6,0
26.05.2011	33	30	132	57	72	182	6,0
01.06.2011	33	30	132	56	67	170	6,0
06.06.2011	15	59	166	53	152	218	6,0
06.06.2011	24	56	163	33	30	132	6,0
07.06.2011	19	58	166	24	56	163	6,0
16.05.2011	27	46	152	60	119	217	6,1
25.05.2011	19	58	166	33	30	132	6,1
25.05.2011	27	46	152	56	67	170	6,1
26.05.2011	27	46	152	57	72	182	6,1
23.06.2011	13	123	195	20	65	158	6,1
17.05.2011	24	56	163	27	46	152	6,2
17.05.2011	24	56	163	57	72	182	6,2
17.05.2011	16	67	169	51	141	207	6,2
25.05.2011	19	58	166	24	56	163	6,2
26.05.2011	30	42	151	33	30	132	6,2
26.05.2011	33	30	132	57	72	182	6,2
26.05.2011	17	34	137	55	126	201	6,2
01.06.2011	22	24	124	53	152	218	6,2
07.06.2011	16	67	169	53	152	218	6,2
07.06.2011	24	56	163	57	72	182	6,2
16.06.2011	28	53	146	35	50	138	6,2
23.06.2011	28	53	146	35	50	138	6,2
17.05.2011	13	48	159	27	46	152	6,3

17.05.2011	14	87	198	16	67	169	6,3
25.05.2011	19	58	166	30	42	151	6,3
26.05.2011	13	48	159	33	30	132	6,3
01.06.2011	16	67	169	22	24	124	6,3
06.06.2011	22	24	124	23	21	117	6,3
17.05.2011	27	46	152	33	30	132	6,4
17.05.2011	13	48	159	57	72	182	6,4
17.05.2011	56	67	170	60	119	217	6,4
17.05.2011	15	59	166	17	34	137	6,4
17.05.2011	15	59	166	23	21	117	6,4
25.05.2011	19	58	166	33	30	132	6,4
26.05.2011	13	48	159	27	46	152	6,4
26.05.2011	27	46	152	30	42	151	6,4
26.05.2011	24	56	163	56	67	170	6,4
26.05.2011	33	30	132	56	67	170	6,4
07.06.2011	14	87	198	53	152	218	6,4
07.06.2011	27	46	152	57	72	182	6,4
22.06.2011	28	53	146	35	50	138	6,4
16.05.2011	30	42	151	57	72	182	6,5
16.05.2011	24	56	163	27	46	152	6,5
17.05.2011	33	30	132	57	72	182	6,5
17.05.2011	57	72	182	60	119	217	6,5
17.05.2011	19	58	166	33	30	132	6,5
25.05.2011	27	46	152	30	42	151	6,5
25.05.2011	13	48	159	57	72	182	6,5
26.05.2011	30	42	151	56	67	170	6,5
01.06.2011	56	67	170	57	72	182	6,5
02.06.2011	19	56	163	27	46	152	6,5
03.06.2011	15	59	166	51	141	207	6,5
22.06.2011	20	65	158	48	81	168	6,5
23.06.2011	28	53	146	35	50	138	6,5
16.05.2011	30	42	151	56	67	170	6,6
16.05.2011	23	21	117	54	199	238	6,6

16.05.2011	13	48	159	27	46	152	6,6
17.05.2011	14	87	198	17	34	137	6,6
25.05.2011	24	56	163	30	42	151	6,6
25.05.2011	24	56	163	33	30	132	6,6
26.05.2011	30	42	151	33	30	132	6,6
01.06.2011	52	147	212	55	126	201	6,6
01.06.2011	19	58	166	24	56	163	6,6
01.06.2011	19	58	166	57	72	182	6,6
02.06.2011	24	58	166	56	42	151	6,6
07.06.2011	19	58	166	57	72	182	6,6
16.05.2011	27	46	152	30	42	151	6,7
17.05.2011	14	87	198	22	24	124	6,7
17.05.2011	17	34	137	22	24	124	6,7
17.05.2011	22	24	124	23	21	117	6,7
18.05.2011	14	87	198	53	152	218	6,7
25.05.2011	27	46	152	60	119	217	6,7
26.05.2011	13	48	159	30	42	151	6,7
27.05.2011	15	59	166	55	126	201	6,7
01.06.2011	14	87	198	51	141	207	6,7
02.06.2011	19	58	166	56	67	170	6,7
06.06.2011	19	58	166	33	30	132	6,7
06.06.2011	27	46	152	33	30	132	6,7
07.06.2011	19	58	166	24	56	163	6,7
16.05.2011	19	58	166	24	56	163	6,8
16.05.2011	27	46	152	57	72	182	6,8
17.05.2011	14	87	198	23	21	117	6,8
18.05.2011	53	152	218	55	126	201	6,8
25.05.2011	19	58	166	30	42	151	6,8
25.05.2011	27	46	152	57	72	182	6,8
25.05.2011	24	56	163	60	119	217	6,8
25.05.2011	30	42	151	57	72	182	6,8
25.05.2011	56	67	170	57	72	182	6,8
26.05.2011	24	56	163	30	42	151	6,8

26.05.2011	27	46	152	30	42	151	6,8
26.05.2011	13	48	159	24	56	163	6,8
07.06.2011	15	59	166	22	24	124	6,8
16.05.2011	51	141	207	54	199	238	6,9
16.05.2011	27	46	152	33	30	132	6,9
25.05.2011	24	56	163	57	72	182	6,9
26.05.2011	13	48	159	24	56	163	6,9
26.05.2011	24	56	163	57	72	182	6,9
26.05.2011	27	46	152	57	72	182	6,9
26.05.2011	13	48	159	33	30	132	6,9
26.05.2011	22	24	124	53	152	218	6,9
01.06.2011	33	30	132	57	72	182	6,9
03.06.2011	15	59	166	53	152	218	6,9
07.06.2011	24	56	163	57	72	182	6,9
17.05.2011	14	87	198	17	34	137	7,0
17.05.2011	14	87	198	55	126	201	7,0
25.05.2011	19	58	166	60	119	217	7,0
25.05.2011	57	72	182	60	119	217	7,0
26.05.2011	13	48	159	27	46	152	7,0
01.06.2011	24	56	163	57	72	182	7,0
06.06.2011	24	56	163	57	72	182	7,0
24.06.2011	20	65	158	48	81	168	7,0
28.06.2011	28	53	138	35	50	138	7,0
16.05.2011	19	58	166	27	46	152	7,1
16.05.2011	19	58	166	33	30	132	7,1
16.05.2011	13	48	159	60	119	217	7,1
17.05.2011	14	87	198	16	67	169	7,1
17.05.2011	23	21	117	54	199	238	7,1
25.05.2011	19	58	166	57	72	182	7,1
26.05.2011	13	48	159	60	119	217	7,1
26.05.2011	30	42	151	60	119	217	7,1
02.06.2011	16	67	169	22	24	124	7,1
02.06.2011	14	87	198	51	141	207	7,1

23.06.2011	28	53	146	35	50	138	7,1
23.06.2011	28	53	146	35	50	138	7,1
16.05.2011	13	48	159	19	58	166	7,2
26.05.2011	22	24	124	23	21	117	7,2
26.05.2011	57	72	182	60	119	217	7,2
26.05.2011	17	34	137	53	152	218	7,2
26.05.2011	27	46	152	30	42	151	7,2
27.05.2011	15	59	166	17	34	137	7,2
27.05.2011	15	59	166	22	24	124	7,2
01.06.2011	24	56	163	27	46	152	7,2
01.06.2011	14	87	198	15	59	166	7,2
16.05.2011	19	58	166	60	119	217	7,3
16.05.2011	30	42	151	60	119	217	7,3
16.05.2011	33	30	132	57	72	182	7,3
17.05.2011	16	67	169	54	199	238	7,3
17.05.2011	13	48	159	57	72	182	7,3
26.05.2011	53	152	218	55	126	201	7,3
01.06.2011	27	46	152	57	72	182	7,3
01.06.2011	19	58	166	57	72	182	7,3
07.06.2011	15	59	166	53	152	218	7,3
07.06.2011	24	56	163	27	46	152	7,3
23.06.2011	28	53	146	35	50	138	7,3
16.05.2011	13	48	159	33	30	132	7,4
16.05.2011	30	42	151	33	30	132	7,4
25.05.2011	13	48	159	27	46	152	7,4
26.05.2011	17	34	137	23	21	117	7,4
26.05.2011	27	46	152	30	42	151	7,4
01.06.2011	27	46	152	33	30	132	7,4
02.06.2011	16	67	169	52	147	212	7,4
06.06.2011	19	58	166	24	56	163	7,4
16.05.2011	19	58	166	57	72	182	7,5
25.05.2011	24	56	163	27	46	152	7,5
26.05.2011	24	56	163	30	42	151	7,5

06.06.2011	22	24	124	23	21	117	7,5
16.05.2011	19	58	166	30	42	151	7,6
17.05.2011	51	141	207	52	147	212	7,6
17.05.2011	13	48	159	27	46	152	7,6
25.05.2011	13	48	159	24	56	163	7,6
25.05.2011	24	56	163	30	42	151	7,6
25.05.2011	24	56	163	33	30	132	7,6
25.05.2011	24	56	163	57	72	182	7,6
25.05.2011	27	46	152	57	72	182	7,6
01.06.2011	14	87	198	23	21	117	7,6
06.06.2011	27	46	152	57	72	182	7,6
07.06.2011	16	67	169	23	21	117	7,6
28.06.2011	28	53	138	35	50	138	7,6
16.05.2011	13	48	159	19	58	166	7,7
17.05.2011	57	72	182	60	119	217	7,7
25.05.2011	17	34	137	55	126	201	7,7
25.05.2011	24	56	163	60	119	217	7,7
01.06.2011	16	67	169	23	21	117	7,7
06.06.2011	15	59	166	53	152	218	7,7
17.06.2011	20	65	158	48	81	168	7,7
29.06.2011	28	53	138	35	50	138	7,7
17.05.2011	23	21	117	53	152	218	7,8
25.05.2011	17	34	137	55	126	201	7,8
26.05.2011	22	24	124	53	152	218	7,8
02.06.2011	14	87	198	16	67	169	7,8
02.06.2011	19	58	166	56	67	170	7,8
06.06.2011	23	21	117	53	152	218	7,8
07.06.2011	19	58	166	24	56	163	7,8
17.05.2011	19	58	166	27	46	152	7,9
17.05.2011	19	58	166	57	72	182	7,9
17.05.2011	51	141	207	53	152	218	7,9
17.05.2011	52	147	212	53	152	218	7,9
26.05.2011	15	59	166	53	152	218	7,9

01.06.2011	27	46	152	33	30	132	7,9
02.06.2011	19	58	166	24	56	163	7,9
07.06.2011	27	46	152	56	67	170	7,9
07.06.2011	15	59	166	53	152	218	8,0
26.05.2011	15	59	166	55	126	201	8,1
26.05.2011	22	24	124	55	126	201	8,1
06.06.2011	14	87	198	51	141	207	8,1
06.06.2011	19	58	166	33	30	132	8,1
16.05.2011	23	21	117	55	126	201	8,2
16.05.2011	51	141	207	52	147	212	8,2
17.05.2011	27	46	152	56	67	170	8,2
17.05.2011	30	42	151	60	119	217	8,2
17.05.2011	57	72	182	60	119	217	8,2
17.05.2011	59	89	191	60	119	217	8,2
26.05.2011	19	58	166	30	42	151	8,2
26.05.2011	19	58	166	57	72	182	8,2
26.05.2011	17	34	137	55	126	201	8,2
06.06.2011	24	56	163	57	72	182	8,2
06.06.2011	14	87	198	23	21	117	8,2
27.06.2011	13	123	195	20	65	158	8,2
27.06.2011	13	123	195	48	81	168	8,2
16.05.2011	14	87	198	23	21	117	8,3
16.05.2011	15	59	166	23	21	117	8,3
16.05.2011	23	21	117	53	152	218	8,3
16.05.2011	23	21	117	54	199	238	8,3
17.05.2011	56	67	170	57	72	182	8,3
17.05.2011	53	152	218	54	199	238	8,3
17.05.2011	13	48	159	56	67	170	8,3
17.05.2011	13	48	159	60	119	217	8,3
17.05.2011	27	46	152	60	119	217	8,3
17.05.2011	56	67	170	60	119	217	8,3
26.05.2011	13	48	159	19	58	166	8,3
07.06.2011	15	59	166	23	21	117	8,3

22.06.2011	28	53	146	35	50	138	8,3
16.05.2011	16	67	169	23	21	117	8,4
16.05.2011	17	34	137	23	21	117	8,4
16.05.2011	22	24	124	23	21	117	8,4
16.05.2011	23	21	117	51	141	207	8,4
17.05.2011	16	67	169	17	34	137	8,4
17.05.2011	14	87	198	23	21	117	8,4
26.05.2011	19	58	166	30	42	151	8,4
01.06.2011	27	46	152	57	72	182	8,4
07.06.2011	22	24	124	53	152	218	8,4
27.06.2011	28	53	138	35	50	138	8,4
18.05.2011	23	21	117	55	126	201	8,5
26.05.2011	22	24	124	55	126	201	8,5
27.05.2011	30	42	151	57	72	182	8,5
01.06.2011	15	59	166	22	24	124	8,5
01.06.2011	14	87	198	22	24	124	8,5
01.06.2011	16	67	169	52	147	212	8,5
02.06.2011	14	87	198	53	152	218	8,5
16.05.2011	19	58	166	57	72	182	8,6
17.05.2011	27	46	152	56	67	170	8,6
17.05.2011	30	42	151	56	67	170	8,6
18.05.2011	23	21	117	51	141	207	8,6
18.05.2011	14	87	198	55	126	201	8,6
25.05.2011	19	58	166	30	42	151	8,6
25.05.2011	16	67	169	51	141	207	8,6
01.06.2011	30	42	151	58	71	182	8,6
02.06.2011	24	58	166	27	42	151	8,6
03.06.2011	51	141	207	53	152	218	8,6
03.06.2011	22	24	124	23	21	117	8,6
06.06.2011	33	30	132	56	67	170	8,6
06.06.2011	27	46	152	33	30	132	8,6
07.06.2011	24	56	163	27	46	152	8,6
16.05.2011	16	67	169	17	34	137	8,7

17.05.2011	33	30	132	56	67	170	8,7
17.05.2011	54	199	238	55	126	201	8,7
18.05.2011	14	87	198	51	141	207	8,7
25.05.2011	19	58	166	56	67	170	8,7
25.05.2011	24	56	163	30	42	151	8,7
25.05.2011	30	42	151	60	119	217	8,7
26.05.2011	17	34	137	55	126	201	8,7
26.05.2011	33	30	132	60	119	217	8,7
27.05.2011	16	67	169	22	24	124	8,7
02.06.2011	14	87	198	23	21	117	8,7
02.06.2011	56	67	170	57	72	182	8,7
06.06.2011	24	56	163	27	46	152	8,7
06.06.2011	14	87	198	22	24	124	8,7
16.05.2011	15	59	166	16	67	169	8,8
16.05.2011	16	67	169	22	24	124	8,8
16.05.2011	19	58	166	56	67	170	8,8
17.05.2011	22	24	124	23	21	117	8,8
25.05.2011	24	56	163	56	67	170	8,8
25.05.2011	19	58	166	56	67	170	8,8
25.05.2011	19	58	166	57	72	182	8,8
26.05.2011	30	42	151	33	30	132	8,8
26.05.2011	33	30	132	57	72	182	8,8
07.06.2011	15	59	166	22	24	124	8,8
16.05.2011	14	87	198	16	67	169	8,9
18.05.2011	15	59	166	54	199	238	8,9
25.05.2011	24	56	163	56	67	170	8,9
25.05.2011	24	56	163	57	72	182	8,9
25.05.2011	19	58	166	60	119	217	8,9
25.05.2011	22	24	124	53	152	218	8,9
26.05.2011	53	152	218	55	126	201	8,9
01.06.2011	22	24	124	23	21	117	8,9
03.06.2011	14	87	198	15	59	166	8,9
28.06.2011	13	123	195	48	81	168	8,9

18.05.2011	23	21	117	55	126	201	9,0
25.05.2011	24	56	163	60	119	217	9,0
25.05.2011	17	67	169	55	126	201	9,0
26.05.2011	19	58	166	30	42	151	9,0
26.05.2011	24	56	163	30	42	151	9,0
26.05.2011	19	58	166	57	72	182	9,0
26.05.2011	19	58	166	60	119	217	9,0
26.05.2011	24	56	163	60	119	217	9,0
01.06.2011	24	56	163	27	46	152	9,0
02.06.2011	14	87	198	15	59	166	9,0
23.06.2011	13	123	195	20	65	158	9,0
16.05.2011	15	59	166	52	147	212	9,1
17.05.2011	15	59	166	23	21	117	9,1
18.05.2011	51	141	207	54	199	238	9,1
18.05.2011	22	24	124	23	21	117	9,1
18.05.2011	23	21	117	54	199	238	9,1
25.05.2011	24	56	163	27	46	152	9,1
25.05.2011	19	58	166	27	46	152	9,1
26.05.2011	24	56	163	57	72	182	9,1
01.06.2011	24	56	163	33	30	132	9,1
01.06.2011	19	58	166	27	46	152	9,1
01.06.2011	19	58	166	56	67	170	9,1
02.06.2011	23	21	117	52	147	212	9,1
02.06.2011	27	46	152	56	67	170	9,1
06.06.2011	33	30	132	57	72	182	9,1
06.06.2011	19	58	166	56	67	170	9,1
17.05.2011	15	59	166	51	141	207	9,2
18.05.2011	15	59	166	55	126	201	9,2
18.05.2011	16	67	169	55	126	201	9,2
18.05.2011	17	34	137	55	126	201	9,2
25.05.2011	16	67	169	17	34	137	9,2
25.05.2011	19	58	166	27	46	152	9,2
25.05.2011	13	48	159	19	58	166	9,2

25.05.2011	19	58	166	30	42	151	9,2
25.05.2011	19	58	166	33	30	132	9,2
25.05.2011	19	58	166	57	72	182	9,2
25.05.2011	17	67	169	23	126	201	9,2
25.05.2011	30	42	151	56	67	170	9,2
25.05.2011	33	30	132	60	119	217	9,2
26.05.2011	23	21	117	51	141	207	9,2
26.05.2011	27	46	152	30	42	151	9,2
26.05.2011	27	46	152	60	119	217	9,2
27.05.2011	56	67	170	57	72	182	9,2
01.06.2011	15	59	166	53	152	218	9,2
01.06.2011	22	24	124	53	152	218	9,2
02.06.2011	14	87	198	22	24	124	9,2
06.06.2011	19	58	166	24	56	163	9,2
17.05.2011	15	59	166	22	24	124	9,3
18.05.2011	16	67	169	54	199	238	9,3
18.05.2011	15	59	166	22	24	124	9,3
18.05.2011	16	67	169	22	24	124	9,3
18.05.2011	17	34	137	22	24	124	9,3
18.05.2011	15	59	166	54	199	238	9,3
18.05.2011	16	67	169	54	199	238	9,3
18.05.2011	17	34	137	54	199	238	9,3
25.05.2011	19	58	166	57	72	182	9,3
25.05.2011	24	56	163	57	72	182	9,3
25.05.2011	19	58	166	60	119	217	9,3
26.05.2011	27	46	152	57	72	182	9,3
16.05.2011	16	67	169	54	199	238	9,4
17.05.2011	22	24	124	54	199	238	9,4
17.05.2011	56	67	170	57	72	182	9,4
18.05.2011	15	59	166	54	199	238	9,4
18.05.2011	17	34	137	54	199	238	9,4
25.05.2011	13	48	159	19	58	166	9,4
25.05.2011	13	48	159	24	56	163	9,4

25.05.2011	19	58	166	30	42	151	9,4
25.05.2011	19	58	166	33	30	132	9,4
25.05.2011	24	56	163	33	30	132	9,4
25.05.2011	24	56	163	60	119	217	9,4
25.05.2011	13	48	159	27	46	152	9,4
25.05.2011	19	58	166	60	119	217	9,4
25.05.2011	27	46	152	30	42	151	9,4
25.05.2011	27	46	152	33	30	132	9,4
25.05.2011	27	46	152	57	72	182	9,4
26.05.2011	13	48	159	56	67	170	9,4
26.05.2011	52	147	212	55	126	201	9,4
26.05.2011	30	42	151	56	67	170	9,4
26.05.2011	16	67	169	17	34	137	9,4
02.06.2011	24	56	163	33	42	151	9,4
02.06.2011	16	67	169	53	152	218	9,4
16.05.2011	16	67	169	54	199	238	9,5
17.05.2011	52	147	212	54	199	238	9,5
25.05.2011	24	56	163	30	42	151	9,5
26.05.2011	17	34	137	55	126	201	9,5
26.05.2011	13	48	159	57	72	182	9,5
26.05.2011	56	67	170	57	72	182	9,5
26.05.2011	56	67	170	60	119	217	9,5
01.06.2011	14	87	198	51	141	207	9,5
03.06.2011	16	67	169	53	152	218	9,5
06.06.2011	30	42	151	58	71	182	9,5
27.06.2011	28	53	138	35	50	138	9,5
16.05.2011	16	67	169	17	34	137	9,6
17.05.2011	27	46	152	30	42	151	9,6
25.05.2011	19	58	166	58	71	182	9,6
25.05.2011	24	56	163	58	71	182	9,6
25.05.2011	27	46	152	60	119	217	9,6
26.05.2011	13	48	159	60	119	217	9,6
01.06.2011	15	59	166	53	152	218	9,6

02.06.2011	24	56	163	56	67	170	9,6
07.06.2011	14	87	198	15	59	166	9,6
07.06.2011	51	141	207	52	147	212	9,6
16.05.2011	16	67	169	23	21	117	9,7
17.05.2011	30	42	151	57	72	182	9,7
17.05.2011	15	59	166	55	126	201	9,7
18.05.2011	22	24	124	54	199	238	9,7
25.05.2011	16	67	169	23	21	117	9,7
25.05.2011	33	30	132	56	67	170	9,7
26.05.2011	17	34	137	55	126	201	9,7
27.05.2011	15	59	166	23	21	117	9,7
27.05.2011	51	141	207	52	147	212	9,7
03.06.2011	16	67	169	51	141	207	9,7
06.06.2011	24	56	163	33	30	132	9,7
06.06.2011	16	67	169	52	147	212	9,7
07.06.2011	24	56	163	27	46	152	9,7
29.06.2011	13	123	195	48	81	168	9,7
17.05.2011	13	48	159	24	56	163	9,8
17.05.2011	24	56	163	57	72	182	9,8
17.05.2011	53	152	218	54	199	238	9,8
18.05.2011	23	21	117	54	199	238	9,8
26.05.2011	14	87	198	23	21	117	9,8
27.05.2011	14	87	198	15	59	166	9,8
02.06.2011	22	24	124	23	21	117	9,8
03.06.2011	15	59	166	51	141	207	9,8
06.06.2011	24	56	163	33	30	132	9,8
07.06.2011	14	87	198	23	21	117	9,8
07.06.2011	16	67	169	53	152	218	9,8
16.05.2011	33	30	132	57	72	182	9,9
26.05.2011	14	87	198	17	34	137	9,9
07.06.2011	19	58	166	24	56	163	9,9
07.06.2011	19	58	166	24	56	163	9,9
17.05.2011	53	152	218	55	126	201	10,0

25.05.2011	19	58	166	60	119	217	10,0
27.05.2011	15	59	166	17	34	137	10,0
01.06.2011	27	46	152	56	67	170	10,0
07.06.2011	14	87	198	23	21	117	10,0
16.05.2011	16	67	169	22	24	124	10,1
16.05.2011	24	56	163	57	72	182	10,1
16.05.2011	27	46	152	59	89	191	10,1
17.05.2011	23	21	117	55	126	201	10,1
17.05.2011	30	42	151	33	30	132	10,1
17.05.2011	24	56	163	27	46	152	10,1
17.05.2011	24	56	163	30	42	151	10,1
17.05.2011	15	59	166	54	199	238	10,1
26.05.2011	17	34	137	23	21	117	10,1
01.06.2011	27	46	152	56	67	170	10,1
06.06.2011	15	59	166	53	152	218	10,1
07.06.2011	16	67	169	23	21	117	10,1
07.06.2011	23	21	117	53	152	218	10,1
28.06.2011	20	65	158	48	81	168	10,1
17.05.2011	24	56	163	33	30	132	10,2
17.05.2011	24	56	163	27	46	152	10,2
25.05.2011	16	67	169	53	152	218	10,2
06.06.2011	14	87	198	23	21	117	10,2
17.05.2011	24	56	163	30	42	151	10,3
25.05.2011	19	58	166	30	42	151	10,3
06.06.2011	24	56	163	57	72	182	10,3
07.06.2011	24	56	163	27	46	152	10,3
17.05.2011	53	152	218	54	199	238	10,4
18.05.2011	24	56	163	59	89	191	10,4
25.05.2011	13	48	159	30	42	151	10,4
25.05.2011	19	58	166	56	67	170	10,4
25.05.2011	33	30	132	60	119	217	10,4
26.05.2011	33	30	132	56	67	170	10,4
26.05.2011	53	152	218	55	126	201	10,4

26.05.2011	15	59	166	17	34	137	10,4
27.05.2011	15	59	166	55	126	201	10,4
06.06.2011	23	21	117	53	152	218	10,4
07.06.2011	14	87	198	53	152	218	10,4
23.06.2011	13	123	195	20	65	158	10,4
28.06.2011	28	53	138	35	50	138	10,4
16.05.2011	14	87	198	16	67	169	10,5
16.05.2011	51	141	207	55	126	201	10,5
17.05.2011	22	24	124	53	152	218	10,5
26.05.2011	16	67	169	53	152	218	10,5
02.06.2011	14	87	198	51	141	207	10,5
03.06.2011	16	67	169	53	152	218	10,5
06.06.2011	15	59	166	16	67	169	10,5
28.06.2011	20	65	158	48	81	168	10,5
17.05.2011	17	34	137	22	24	124	10,6
17.05.2011	51	141	207	53	152	218	10,6
18.05.2011	33	30	132	59	89	191	10,6
25.05.2011	16	67	169	55	126	201	10,6
25.05.2011	16	67	169	55	126	201	10,6
25.05.2011	24	56	163	30	42	151	10,6
25.05.2011	30	42	151	57	72	182	10,6
26.05.2011	24	56	163	56	67	170	10,6
26.05.2011	23	21	117	53	152	218	10,6
01.06.2011	24	56	163	27	46	152	10,6
01.06.2011	19	58	166	33	30	132	10,6
07.06.2011	19	58	166	56	67	170	10,6
07.06.2011	22	24	124	23	21	117	10,6
07.06.2011	14	87	198	22	24	124	10,6
23.06.2011	20	65	158	48	81	168	10,6
18.05.2011	13	48	159	59	89	191	10,7
25.05.2011	13	48	159	19	58	166	10,7
25.05.2011	30	42	151	33	30	132	10,7
25.05.2011	33	30	132	56	67	170	10,7

26.05.2011	27	46	152	56	67	170	10,7
26.05.2011	24	56	163	30	42	151	10,7
26.05.2011	27	46	152	30	42	151	10,7
01.06.2011	51	141	207	52	147	212	10,7
02.06.2011	15	59	166	22	24	124	10,7
03.06.2011	23	21	117	53	152	218	10,7
06.06.2011	19	58	166	27	46	152	10,7
07.06.2011	16	67	169	53	152	218	10,7
17.05.2011	15	59	166	16	67	169	10,8
25.05.2011	13	48	159	33	30	132	10,8
26.05.2011	24	56	163	27	46	152	10,8
01.06.2011	19	58	166	57	72	182	10,8
02.06.2011	51	141	207	52	147	212	10,8
16.05.2011	14	87	198	53	152	218	10,9
17.05.2011	19	58	166	56	67	170	10,9
26.05.2011	23	21	117	55	126	201	10,9
26.05.2011	19	58	166	27	46	152	10,9
02.06.2011	15	59	166	23	21	117	10,9
03.06.2011	33	107	266	56	71	182	10,9
06.06.2011	24	56	163	27	46	152	10,9
06.06.2011	33	30	132	56	67	170	10,9
17.06.2011	28	53	146	35	50	138	10,9
17.05.2011	14	87	198	15	59	166	11,0
17.05.2011	14	87	198	55	126	201	11,0
17.05.2011	56	67	170	57	72	182	11,0
17.05.2011	19	58	166	56	67	170	11,0
18.05.2011	24	56	163	27	46	152	11,0
25.05.2011	24	56	163	33	30	132	11,0
25.05.2011	33	30	132	57	72	182	11,0
26.05.2011	16	67	169	17	34	137	11,0
07.06.2011	16	67	169	51	141	207	11,0
07.06.2011	14	87	198	15	59	166	11,0
24.06.2011	13	123	195	20	65	158	11,0

16.05.2011	19	58	166	24	56	163	11,1
16.05.2011	24	56	163	27	46	152	11,1
17.05.2011	13	48	159	33	30	132	11,1
17.05.2011	27	46	152	33	30	132	11,1
17.05.2011	19	58	166	33	30	132	11,1
17.05.2011	16	67	169	22	24	124	11,1
18.05.2011	24	56	163	30	42	151	11,1
25.05.2011	13	48	159	33	30	132	11,1
26.05.2011	14	87	198	53	152	218	11,1
27.05.2011	13	48	159	57	72	182	11,1
01.06.2011	16	67	169	23	21	117	11,1
28.06.2011	28	53	138	35	50	138	11,1
17.05.2011	14	87	198	15	59	166	11,2
18.05.2011	27	46	152	33	30	132	11,2
25.05.2011	27	46	152	33	30	132	11,2
26.05.2011	14	87	198	17	34	137	11,2
26.05.2011	16	67	169	22	24	124	11,2
26.05.2011	15	59	166	52	147	212	11,2
26.05.2011	13	48	159	24	56	163	11,2
01.06.2011	51	141	207	53	152	218	11,2
02.06.2011	22	24	124	53	152	218	11,2
16.05.2011	16	67	169	55	126	201	11,3
16.05.2011	19	58	166	57	72	182	11,3
17.05.2011	13	48	159	24	56	163	11,3
17.05.2011	24	56	163	27	46	152	11,3
18.05.2011	13	48	159	27	46	152	11,3
18.05.2011	30	42	151	33	30	132	11,3
25.05.2011	19	58	166	27	46	152	11,3
26.05.2011	13	48	159	19	58	166	11,3
26.05.2011	24	56	163	56	67	170	11,3
02.06.2011	14	87	198	53	152	218	11,3
02.06.2011	51	141	207	53	152	218	11,3
02.06.2011	23	21	117	51	141	207	11,3

07.06.2011	27	46	152	33	30	132	11,3
18.05.2011	13	48	159	30	42	151	11,4
25.05.2011	17	34	137	53	152	218	11,4
26.05.2011	14	87	198	22	24	124	11,4
26.05.2011	19	58	166	56	67	170	11,4
02.06.2011	51	141	207	53	152	218	11,4
07.06.2011	15	59	166	22	24	124	11,4
16.05.2011	14	87	198	23	21	117	11,5
17.05.2011	14	87	198	22	24	124	11,5
01.06.2011	19	58	166	24	56	163	11,5
01.06.2011	19	58	166	33	30	132	11,5
01.06.2011	19	58	166	57	72	182	11,5
01.06.2011	14	87	198	52	147	212	11,5
01.06.2011	22	24	124	52	147	212	11,5
06.06.2011	14	87	198	53	152	218	11,5
06.06.2011	19	58	166	24	56	163	11,5
07.06.2011	23	21	117	52	147	212	11,5
07.06.2011	14	87	198	23	21	117	11,5
18.05.2011	24	56	163	56	67	170	11,6
26.05.2011	16	67	169	23	21	117	11,6
26.05.2011	23	21	117	53	152	218	11,6
07.06.2011	15	59	166	53	152	218	11,6
16.05.2011	16	67	169	53	152	218	11,7
25.05.2011	27	46	152	33	30	132	11,7
26.05.2011	14	87	198	16	67	169	11,7
26.05.2011	23	21	117	55	126	201	11,7
02.06.2011	14	87	198	15	59	166	11,7
06.06.2011	27	46	152	56	67	170	11,7
17.05.2011	22	24	124	54	199	238	11,8
17.05.2011	33	30	132	57	72	182	11,8
17.05.2011	15	59	166	23	21	117	11,8
26.05.2011	19	58	166	56	67	170	11,8
26.05.2011	16	67	169	22	24	124	11,8

26.05.2011	30	42	151	33	30	132	11,8
02.06.2011	19	58	166	33	30	132	11,8
06.06.2011	33	30	132	57	72	182	11,8
07.06.2011	19	58	166	24	56	163	11,8
07.06.2011	19	58	166	33	30	132	11,8
07.06.2011	27	46	152	56	67	170	11,8
16.05.2011	27	46	152	60	119	217	11,9
18.05.2011	33	30	132	56	67	170	11,9
18.05.2011	24	56	163	57	72	182	11,9
27.05.2011	23	21	117	53	152	218	11,9
01.06.2011	27	46	152	56	67	170	11,9
06.06.2011	19	58	166	33	30	132	11,9
23.06.2011	20	65	158	48	81	168	11,9
24.06.2011	13	123	195	48	81	168	11,9
16.05.2011	24	56	163	60	119	217	12,0
17.05.2011	51	141	207	53	152	218	12,0
18.05.2011	23	21	117	52	147	212	12,0
18.05.2011	13	48	159	56	67	170	12,0
18.05.2011	24	56	163	60	119	217	12,0
26.05.2011	15	59	166	17	34	137	12,0
26.05.2011	17	34	137	23	21	117	12,0
27.05.2011	14	87	198	53	152	218	12,0
02.06.2011	15	59	166	23	21	117	12,0
07.06.2011	27	46	152	56	67	170	12,0
17.06.2011	13	123	195	48	81	168	12,0
16.05.2011	22	24	124	54	199	238	12,1
16.05.2011	24	56	163	57	72	182	12,1
18.05.2011	14	87	198	52	147	212	12,1
26.05.2011	33	30	132	60	119	217	12,1
26.05.2011	15	59	166	53	152	218	12,1
26.05.2011	19	58	166	33	30	132	12,1
26.05.2011	24	56	163	33	30	132	12,1
16.05.2011	27	46	152	56	67	170	12,2

16.05.2011	24	56	163	30	42	151	12,2
17.05.2011	53	152	218	54	199	238	12,2
17.05.2011	13	48	159	60	119	217	12,2
18.05.2011	33	30	132	57	72	182	12,2
26.05.2011	33	30	132	56	67	170	12,2
26.05.2011	33	30	132	57	72	182	12,2
27.05.2011	17	34	137	53	152	218	12,2
02.06.2011	16	67	169	23	21	117	12,2
07.06.2011	19	58	166	56	67	170	12,2
16.05.2011	13	48	159	24	56	163	12,3
18.05.2011	13	48	159	57	72	182	12,3
18.05.2011	33	30	132	60	119	217	12,3
25.05.2011	53	152	218	55	126	201	12,3
26.05.2011	24	56	163	60	119	217	12,3
02.06.2011	19	58	166	56	67	170	12,3
02.06.2011	16	67	169	22	24	124	12,3
07.06.2011	23	21	117	53	152	218	12,3
16.05.2011	33	30	132	60	119	217	12,4
18.05.2011	53	152	218	54	199	238	12,4
18.05.2011	13	48	159	60	119	217	12,4
26.05.2011	16	67	169	53	152	218	12,4
26.05.2011	27	46	152	60	119	217	12,4
26.05.2011	33	30	132	60	119	217	12,4
02.06.2011	14	87	198	23	21	117	12,4
02.06.2011	16	67	169	23	21	117	12,4
07.06.2011	23	21	117	53	152	218	12,4
07.06.2011	14	87	198	16	67	169	12,4
07.06.2011	19	58	166	56	67	170	12,4
16.06.2011	20	65	158	46	118	192	12,4
23.06.2011	13	123	195	20	65	158	12,4
18.05.2011	22	24	124	53	152	218	12,5
25.05.2011	17	34	137	23	21	117	12,5
26.05.2011	14	87	198	53	152	218	12,5

03.06.2011	16	67	169	51	141	207	12,5
06.06.2011	16	67	169	53	152	218	12,5
23.06.2011	13	123	195	48	81	168	12,5
17.05.2011	16	67	169	51	141	207	12,6
18.05.2011	53	152	218	55	126	201	12,6
26.05.2011	14	87	198	16	67	169	12,6
27.05.2011	53	152	218	55	126	201	12,6
06.06.2011	19	58	166	56	67	170	12,6
07.06.2011	22	24	124	53	152	218	12,6
07.06.2011	27	46	152	33	30	132	12,6
16.05.2011	13	48	159	27	46	152	12,7
17.05.2011	14	87	198	53	152	218	12,7
17.05.2011	16	67	169	54	199	238	12,7
25.05.2011	16	67	169	22	24	124	12,7
25.05.2011	17	34	137	23	21	117	12,7
02.06.2011	15	59	166	53	152	218	12,7
06.06.2011	14	87	198	53	152	218	12,7
06.06.2011	27	46	152	56	67	170	12,7
25.05.2011	23	21	117	53	152	218	12,8
01.06.2011	19	58	166	56	67	170	12,8
02.06.2011	52	147	212	53	152	218	12,8
02.06.2011	23	21	117	53	152	218	12,8
02.06.2011	51	141	207	53	152	218	12,8
07.06.2011	19	58	166	57	72	182	12,8
28.06.2011	28	53	138	35	50	138	12,8
16.05.2011	58	71	182	59	89	191	12,9
26.05.2011	19	58	166	57	72	182	12,9
26.05.2011	24	56	163	57	72	182	12,9
02.06.2011	27	46	152	33	30	132	12,9
06.06.2011	33	30	132	56	67	170	12,9
16.05.2011	54	199	238	55	126	201	13,0
26.05.2011	24	56	163	56	67	170	13,0
26.05.2011	27	46	152	56	67	170	13,0

26.05.2011	27	46	152	57	72	182	13,0
01.06.2011	24	56	163	27	46	152	13,0
03.06.2011	19	42	151	27	30	132	13,0
07.06.2011	19	58	166	33	30	132	13,0
16.05.2011	24	56	163	60	119	217	13,1
16.05.2011	27	46	152	30	42	151	13,1
17.05.2011	51	141	207	52	147	212	13,1
26.05.2011	24	56	163	57	72	182	13,1
26.05.2011	27	46	152	60	119	217	13,1
26.05.2011	24	56	163	60	119	217	13,1
01.06.2011	27	46	152	57	72	182	13,1
02.06.2011	14	87	198	16	67	169	13,1
02.06.2011	24	56	163	33	30	132	13,1
03.06.2011	52	147	212	53	152	218	13,1
06.06.2011	27	46	152	56	67	170	13,1
24.06.2011	28	53	146	35	50	138	13,1
16.05.2011	24	56	163	57	72	182	13,2
16.05.2011	24	56	163	59	89	191	13,2
16.05.2011	24	56	163	30	42	151	13,2
16.05.2011	24	56	163	33	30	132	13,2
17.05.2011	23	21	117	55	126	201	13,2
17.05.2011	24	56	163	60	119	217	13,2
17.05.2011	33	30	132	60	119	217	13,2
18.05.2011	19	58	166	59	89	191	13,2
25.05.2011	17	34	137	53	152	218	13,2
26.05.2011	24	56	163	60	119	217	13,2
26.05.2011	19	58	166	60	119	217	13,2
16.05.2011	13	48	159	24	56	163	13,3
16.05.2011	19	58	166	24	56	163	13,3
16.05.2011	24	56	163	27	46	152	13,3
16.05.2011	24	56	163	30	42	151	13,3
16.05.2011	13	48	159	24	56	163	13,3
16.05.2011	24	56	163	57	72	182	13,3

16.05.2011	24	56	163	59	89	191	13,3
17.05.2011	33	30	132	60	119	217	13,3
17.05.2011	13	48	159	24	56	163	13,3
17.05.2011	13	48	159	33	30	132	13,3
17.05.2011	24	56	163	57	72	182	13,3
17.05.2011	33	30	132	57	72	182	13,3
17.05.2011	24	56	163	59	89	191	13,3
17.05.2011	33	30	132	59	89	191	13,3
17.05.2011	56	67	170	60	119	217	13,3
17.05.2011	22	24	124	51	141	207	13,3
18.05.2011	13	48	159	30	42	151	13,3
25.05.2011	14	87	198	55	126	201	13,3
26.05.2011	22	24	124	55	126	201	13,3
06.06.2011	16	67	169	23	21	117	13,3
16.06.2011	20	65	158	48	81	168	13,3
16.05.2011	24	56	163	33	30	132	13,4
16.05.2011	19	58	166	56	67	170	13,4
16.05.2011	22	24	124	53	152	218	13,4
17.05.2011	27	46	152	60	119	217	13,4
17.05.2011	30	42	151	60	119	217	13,4
17.05.2011	24	56	163	27	46	152	13,4
17.05.2011	24	56	163	30	42	151	13,4
17.05.2011	27	46	152	33	30	132	13,4
17.05.2011	30	42	151	33	30	132	13,4
17.05.2011	22	24	124	54	199	238	13,4
18.05.2011	13	48	159	27	46	152	13,4
18.05.2011	24	56	163	30	42	151	13,4
18.05.2011	13	48	159	56	67	170	13,4
18.05.2011	13	48	159	59	89	191	13,4
25.05.2011	27	46	152	30	42	151	13,4
26.05.2011	19	58	166	60	119	217	13,4
01.06.2011	51	141	207	53	152	218	13,4
01.06.2011	19	58	166	24	56	163	13,4

17.05.2011	13	48	159	56	67	170	13,5
17.05.2011	56	67	170	57	72	182	13,5
17.05.2011	56	67	170	59	89	191	13,5
17.05.2011	19	58	166	60	119	217	13,5
25.05.2011	16	67	169	17	34	137	13,5
01.06.2011	14	87	198	51	141	207	13,5
02.06.2011	14	87	198	51	141	207	13,5
03.06.2011	51	141	207	53	152	218	13,5
06.06.2011	19	58	166	57	72	182	13,5
07.06.2011	27	46	152	57	72	182	13,5
17.05.2011	27	46	152	56	67	170	13,6
17.05.2011	30	42	151	56	67	170	13,6
17.05.2011	15	59	166	51	141	207	13,6
17.05.2011	15	59	166	54	199	238	13,6
17.05.2011	24	56	163	60	119	217	13,6
17.05.2011	33	30	132	60	119	217	13,6
18.05.2011	24	56	163	27	46	152	13,6
18.05.2011	24	56	163	56	67	170	13,6
18.05.2011	24	56	163	59	89	191	13,6
25.05.2011	16	67	169	23	21	117	13,6
27.05.2011	15	59	166	51	141	207	13,6
03.06.2011	16	67	169	52	147	212	13,6
07.06.2011	51	141	207	52	147	212	13,6
18.05.2011	19	58	166	27	46	152	13,7
25.05.2011	16	67	169	17	34	137	13,7
25.05.2011	27	46	152	56	67	170	13,7
25.05.2011	27	46	152	57	72	182	13,7
25.05.2011	27	46	152	60	119	217	13,7
25.05.2011	14	87	198	23	21	117	13,7
06.06.2011	23	21	117	51	141	207	13,7
27.06.2011	48	81	168	13	123	195	13,7
16.05.2011	24	56	163	60	119	217	13,8
17.05.2011	13	48	159	60	119	217	13,8

17.05.2011	53	152	218	54	199	238	13,8
18.05.2011	30	42	151	33	30	132	13,8
18.05.2011	13	48	159	57	72	182	13,8
18.05.2011	19	58	166	30	42	151	13,8
02.06.2011	33	30	132	56	67	170	13,8
06.06.2011	16	67	169	51	141	207	13,8
07.06.2011	22	24	124	53	152	218	13,8
27.06.2011	20	65	158	48	81	168	13,8
28.06.2011	13	123	195	20	65	158	13,8
28.06.2011	13	123	195	48	81	168	13,8
17.05.2011	51	141	207	53	152	218	13,9
18.05.2011	27	46	152	33	30	132	13,9
18.05.2011	33	30	132	56	67	170	13,9
18.05.2011	33	30	132	59	89	191	13,9
18.05.2011	13	48	159	60	119	217	13,9
18.05.2011	51	141	207	54	199	238	13,9
01.06.2011	23	21	117	53	152	218	13,9
01.06.2011	19	58	166	24	56	163	13,9
02.06.2011	16	67	169	51	141	207	13,9
16.05.2011	27	46	152	33	30	132	14,0
18.05.2011	24	56	163	57	72	182	14,0
18.05.2011	22	24	124	51	141	207	14,0
26.05.2011	15	59	166	17	34	137	14,0
26.05.2011	17	34	137	22	24	124	14,0
26.05.2011	19	58	166	56	67	170	14,0
26.05.2011	19	58	166	57	72	182	14,0
26.05.2011	14	87	198	17	34	137	14,0
26.05.2011	13	48	159	24	56	163	14,0
26.05.2011	13	48	159	27	46	152	14,0
26.05.2011	24	56	163	57	72	182	14,0
06.06.2011	33	30	132	57	72	182	14,0
18.05.2011	19	58	166	30	42	151	14,1
18.05.2011	24	56	163	60	119	217	14,1

18.05.2011	51	141	207	55	126	201	14,1
26.05.2011	19	58	166	60	119	217	14,1
26.05.2011	24	56	163	60	119	217	14,1
26.05.2011	27	46	152	57	72	182	14,1
26.05.2011	27	46	152	60	119	217	14,1
26.05.2011	14	87	198	53	152	218	14,1
07.06.2011	19	58	166	56	67	170	14,1
07.06.2011	15	59	166	16	67	169	14,1
16.05.2011	19	58	166	27	46	152	14,2
17.05.2011	14	87	198	53	152	218	14,2
27.05.2011	16	67	169	22	24	124	14,2
02.06.2011	14	87	198	22	24	124	14,2
02.06.2011	23	21	117	53	152	218	14,2
02.06.2011	33	30	132	57	72	182	14,2
06.06.2011	51	141	207	53	152	218	14,2
17.05.2011	14	87	198	51	141	207	14,3
18.05.2011	19	58	166	27	46	152	14,3
18.05.2011	19	58	166	56	67	170	14,3
18.05.2011	33	30	132	57	72	182	14,3
18.05.2011	19	58	166	59	89	191	14,3
25.05.2011	15	59	166	22	24	124	14,3
26.05.2011	14	87	198	52	147	212	14,3
01.06.2011	16	67	169	51	141	207	14,3
02.06.2011	15	59	166	16	67	169	14,3
06.06.2011	19	58	166	33	30	132	14,3
06.06.2011	27	46	152	57	72	182	14,3
16.06.2011	13	123	195	20	65	158	14,3
17.06.2011	13	123	195	34	47	141	14,3
17.06.2011	28	53	146	35	50	138	14,3
17.05.2011	19	58	166	56	67	170	14,4
18.05.2011	33	30	132	60	119	217	14,4
18.05.2011	19	58	166	56	67	170	14,4
25.05.2011	19	58	166	60	119	217	14,4

25.05.2011	16	67	169	23	21	117	14,4
25.05.2011	53	152	218	55	126	201	14,4
26.05.2011	15	59	166	22	24	124	14,4
01.06.2011	23	21	117	53	152	218	14,4
06.06.2011	16	67	169	52	147	212	14,4
07.06.2011	16	67	169	53	152	218	14,4
27.06.2011	20	65	158	48	81	168	14,4
18.05.2011	19	58	166	57	72	182	14,5
18.05.2011	14	87	198	15	59	166	14,5
18.05.2011	14	87	198	16	67	169	14,5
18.05.2011	14	87	198	17	34	137	14,5
25.05.2011	19	58	166	56	67	170	14,5
26.05.2011	14	87	198	16	67	169	14,5
02.06.2011	19	58	166	56	56	163	14,5
06.06.2011	23	21	117	51	141	207	14,5
07.06.2011	24	56	163	27	46	152	14,5
17.05.2011	51	141	207	53	152	218	14,6
17.05.2011	19	58	166	24	56	163	14,6
17.05.2011	19	58	166	33	30	132	14,6
18.05.2011	51	141	207	53	152	218	14,6
18.05.2011	19	58	166	60	119	217	14,6
26.05.2011	16	67	169	23	21	117	14,6
01.06.2011	27	46	152	33	30	132	14,6
02.06.2011	19	58	166	24	56	163	14,6
06.06.2011	16	67	169	51	141	207	14,6
06.06.2011	15	59	166	51	141	207	14,6
06.06.2011	23	21	117	53	152	218	14,6
17.05.2011	23	21	117	51	141	207	14,7
18.05.2011	53	152	218	55	126	201	14,7
18.05.2011	14	87	198	23	21	117	14,7
18.05.2011	19	58	166	57	72	182	14,7
26.05.2011	19	58	166	56	67	170	14,7
01.06.2011	23	21	117	53	152	218	14,7

03.06.2011	16	67	169	51	141	207	14,7
03.06.2011	14	87	198	15	59	166	14,7
03.06.2011	52	147	212	53	152	218	14,7
06.06.2011	33	30	132	56	67	170	14,7
16.05.2011	52	147	212	55	126	201	14,8
18.05.2011	19	58	166	60	119	217	14,8
01.06.2011	19	58	166	27	46	152	14,8
03.06.2011	19	42	151	27	67	170	14,8
03.06.2011	19	30	132	56	72	182	14,8
06.06.2011	27	46	152	33	30	132	14,8
06.06.2011	14	87	198	23	21	117	14,8
25.05.2011	14	87	198	15	59	166	14,9
25.05.2011	51	141	207	53	152	218	14,9
26.05.2011	19	58	166	33	30	132	14,9
27.05.2011	13	48	159	33	30	132	14,9
27.05.2011	33	30	132	56	67	170	14,9
27.06.2011	13	123	195	48	81	168	14,9
16.05.2011	24	56	163	59	89	191	15,0
18.05.2011	13	48	159	19	58	166	15,0
18.05.2011	19	58	166	30	42	151	15,0
18.05.2011	19	58	166	56	67	170	15,0
18.05.2011	19	58	166	57	72	182	15,0
18.05.2011	19	58	166	60	119	217	15,0
25.05.2011	16	67	169	23	21	117	15,0
26.05.2011	19	58	166	24	56	163	15,0
27.05.2011	33	30	132	57	72	182	15,0
01.06.2011	30	42	151	58	71	182	15,0
07.06.2011	27	46	152	56	67	170	15,0
17.06.2011	13	123	195	48	81	168	15,0
17.06.2011	13	123	195	20	65	158	15,0
18.05.2011	19	58	166	33	30	132	15,1
26.05.2011	13	48	159	33	30	132	15,1
26.05.2011	33	30	132	57	72	182	15,1

26.05.2011	33	30	132	60	119	217	15,1
27.05.2011	19	58	166	57	72	182	15,1
06.06.2011	27	46	152	57	72	182	15,1
18.05.2011	13	48	159	27	46	152	15,2
25.05.2011	52	147	212	55	126	201	15,2
27.05.2011	24	56	163	57	72	182	15,2
27.05.2011	33	30	132	57	72	182	15,2
07.06.2011	19	58	166	56	67	170	15,2
17.05.2011	14	87	198	54	199	238	15,3
17.05.2011	17	34	137	51	141	207	15,3
17.05.2011	22	24	124	51	141	207	15,3
17.05.2011	17	34	137	53	152	218	15,3
17.05.2011	22	24	124	53	152	218	15,3
18.05.2011	27	46	152	30	42	151	15,3
18.05.2011	27	46	152	33	30	132	15,3
18.05.2011	27	46	152	56	67	170	15,3
18.05.2011	27	46	152	57	72	182	15,3
18.05.2011	27	46	152	60	119	217	15,3
07.06.2011	19	58	166	33	30	132	15,3
16.05.2011	33	30	132	59	89	191	15,4
17.05.2011	15	59	166	16	67	169	15,4
27.05.2011	16	67	169	53	152	218	15,4
02.06.2011	19	58	166	33	30	132	15,4
03.06.2011	19	42	151	24	30	132	15,4
07.06.2011	27	46	152	56	67	170	15,4
16.05.2011	27	46	152	57	72	182	15,5
17.05.2011	16	67	169	51	141	207	15,5
17.05.2011	16	67	169	53	152	218	15,5
25.05.2011	14	87	198	51	141	207	15,5
27.05.2011	14	87	198	53	152	218	15,5
27.05.2011	27	46	152	57	72	182	15,5
07.06.2011	27	46	152	33	30	132	15,5
28.06.2011	13	123	195	48	81	168	15,5

25.05.2011	15	59	166	16	67	169	15,6
25.05.2011	15	59	166	22	24	124	15,6
01.06.2011	23	21	117	53	152	218	15,6
02.06.2011	23	21	117	51	141	207	15,6
06.06.2011	33	30	132	57	72	182	15,6
26.05.2011	17	34	137	53	152	218	15,7
03.06.2011	15	59	166	51	141	207	15,7
06.06.2011	14	87	198	53	152	218	15,7
07.06.2011	19	58	166	56	67	170	15,7
16.05.2011	24	56	163	56	67	170	15,8
26.05.2011	17	34	137	53	152	218	15,8
27.05.2011	17	34	137	53	152	218	15,8
02.06.2011	22	24	124	51	141	207	15,8
27.05.2011	22	24	124	53	152	218	15,9
01.06.2011	14	87	198	23	21	117	15,9
07.06.2011	56	67	170	57	72	182	15,9
15.06.2011	13	123	195	48	81	168	15,9
28.06.2011	13	123	195	20	65	158	15,9
25.05.2011	15	59	166	16	67	169	16,0
25.05.2011	24	56	163	60	119	217	16,0
07.06.2011	23	21	117	53	152	218	16,0
16.05.2011	15	59	166	51	141	207	16,1
27.05.2011	14	87	198	17	34	137	16,1
01.06.2011	16	67	169	23	21	117	16,1
17.05.2011	15	59	166	54	199	238	16,2
26.05.2011	16	67	169	51	141	207	16,2
26.05.2011	15	59	166	55	126	201	16,2
02.06.2011	14	87	198	16	67	169	16,2
03.06.2011	15	59	166	16	67	169	16,2
03.06.2011	19	42	151	24	30	132	16,2
06.06.2011	22	24	124	23	21	117	16,2
07.06.2011	14	87	198	22	24	124	16,2
25.05.2011	51	141	207	52	147	212	16,3

25.05.2011	24	56	163	30	42	151	16,3
27.05.2011	16	67	169	53	152	218	16,3
02.06.2011	14	87	198	51	141	207	16,3
03.06.2011	19	42	151	57	67	170	16,3
06.06.2011	56	67	170	57	72	182	16,3
07.06.2011	30	42	151	58	71	182	16,3
22.06.2011	13	123	195	48	81	168	16,3
17.05.2011	15	59	166	53	152	218	16,4
25.05.2011	14	87	198	15	59	166	16,4
25.05.2011	13	48	159	19	58	166	16,4
25.05.2011	24	56	163	56	67	170	16,4
25.05.2011	19	58	166	57	72	182	16,4
26.05.2011	22	24	124	23	21	117	16,4
02.06.2011	19	58	166	27	46	152	16,4
02.06.2011	14	87	198	52	147	212	16,4
06.06.2011	24	56	163	27	46	152	16,4
17.05.2011	13	48	159	59	89	191	16,5
17.05.2011	15	59	166	23	21	117	16,5
17.05.2011	16	67	169	23	21	117	16,5
17.05.2011	22	24	124	23	21	117	16,5
17.05.2011	15	59	166	51	141	207	16,5
18.05.2011	22	24	124	23	21	117	16,5
26.05.2011	13	48	159	60	119	217	16,5
27.05.2011	15	59	166	53	152	218	16,5
06.06.2011	19	58	166	24	56	163	16,5
06.06.2011	24	56	163	56	67	170	16,5
17.05.2011	13	48	159	19	58	166	16,6
17.05.2011	17	34	137	23	21	117	16,6
18.05.2011	14	87	198	22	24	124	16,6
25.05.2011	19	58	166	24	56	163	16,6
25.05.2011	19	58	166	57	72	182	16,6
26.05.2011	30	42	151	60	119	217	16,6
26.05.2011	17	34	137	23	21	117	16,6

27.05.2011	53	152	218	55	126	201	16,6
17.05.2011	24	56	163	57	72	182	16,7
17.05.2011	19	58	166	24	56	163	16,7
17.05.2011	13	48	159	24	56	163	16,7
25.05.2011	13	48	159	24	56	163	16,7
26.05.2011	19	58	166	27	46	152	16,7
06.06.2011	15	59	166	16	67	169	16,7
07.06.2011	22	24	124	53	152	218	16,7
07.06.2011	14	87	198	51	141	207	16,7
07.06.2011	33	30	132	57	72	182	16,7
29.06.2011	28	53	138	35	50	138	16,7
17.05.2011	19	58	166	24	56	163	16,8
17.05.2011	33	30	132	57	72	182	16,8
01.06.2011	14	87	198	53	152	218	16,8
07.06.2011	24	56	163	33	30	132	16,8
17.05.2011	19	58	166	33	30	132	16,9
25.05.2011	51	141	207	52	147	212	16,9
26.05.2011	13	48	159	60	119	217	16,9
26.05.2011	56	67	170	60	119	217	16,9
27.05.2011	23	21	117	53	152	218	16,9
06.06.2011	16	67	169	51	141	207	16,9
07.06.2011	19	58	166	33	30	132	16,9
16.05.2011	56	67	170	60	119	217	17,0
17.05.2011	54	199	238	55	126	201	17,0
17.05.2011	13	48	159	30	42	151	17,0
18.05.2011	14	87	198	16	67	169	17,0
18.05.2011	16	67	169	23	21	117	17,0
07.06.2011	56	67	170	57	72	182	17,0
07.06.2011	15	59	166	51	141	207	17,0
17.05.2011	23	21	117	53	152	218	17,1
17.05.2011	13	48	159	19	58	166	17,1
17.05.2011	13	48	159	24	56	163	17,1
25.05.2011	33	30	132	57	72	182	17,1

26.05.2011	57	72	182	60	119	217	17,1
26.05.2011	22	24	124	55	126	201	17,1
26.05.2011	22	24	124	23	21	117	17,1
01.06.2011	52	147	212	53	152	218	17,1
01.06.2011	16	67	169	23	21	117	17,2
02.06.2011	19	58	166	33	46	152	17,2
07.06.2011	33	30	132	57	72	182	17,2
07.06.2011	27	46	152	33	30	132	17,2
25.05.2011	24	56	163	27	46	152	17,3
26.05.2011	14	87	198	52	147	212	17,3
26.05.2011	30	42	151	60	119	217	17,3
26.05.2011	57	72	182	60	119	217	17,3
26.05.2011	19	58	166	33	30	132	17,3
01.06.2011	19	58	166	56	67	170	17,3
01.06.2011	19	58	166	57	72	182	17,3
06.06.2011	24	56	163	33	30	132	17,3
16.05.2011	13	48	159	24	56	163	17,4
17.05.2011	14	87	198	54	199	238	17,4
17.05.2011	23	21	117	54	199	238	17,4
17.05.2011	54	199	238	55	126	201	17,4
18.05.2011	19	58	166	59	89	191	17,4
25.05.2011	22	24	124	53	152	218	17,4
25.05.2011	17	34	137	22	24	124	17,4
07.06.2011	27	46	152	56	67	170	17,4
07.06.2011	23	21	117	53	152	218	17,4
17.05.2011	16	67	169	55	126	201	17,5
18.05.2011	17	34	137	23	21	117	17,5
02.06.2011	16	67	169	52	147	212	17,5
06.06.2011	19	58	166	57	72	182	17,5
23.06.2011	13	123	195	20	65	158	17,5
16.05.2011	51	141	207	53	152	218	17,6
17.05.2011	13	48	159	30	42	151	17,6
17.05.2011	14	87	198	23	21	117	17,6

18.05.2011	14	87	198	17	34	137	17,6
18.05.2011	27	46	152	59	89	191	17,6
26.05.2011	14	87	198	22	24	124	17,6
16.05.2011	13	48	159	33	30	132	17,7
25.05.2011	27	46	152	56	67	170	17,7
26.05.2011	19	58	166	30	42	151	17,7
26.05.2011	24	56	163	30	42	151	17,7
27.05.2011	16	67	169	23	21	117	17,7
16.05.2011	22	24	124	53	152	218	17,8
26.05.2011	51	141	207	52	147	212	17,8
02.06.2011	15	59	166	51	141	207	17,8
06.06.2011	51	141	207	52	147	212	17,8
06.06.2011	14	87	198	23	21	117	17,8
22.06.2011	13	123	195	48	81	168	17,8
16.05.2011	15	59	166	22	24	124	17,9
17.05.2011	51	141	207	54	199	238	17,9
17.05.2011	15	59	166	17	34	137	17,9
25.05.2011	14	87	198	51	141	207	17,9
26.05.2011	14	87	198	51	141	207	17,9
27.05.2011	14	87	198	55	126	201	17,9
02.06.2011	19	58	166	27	46	152	17,9
06.06.2011	19	58	166	33	30	132	17,9
06.06.2011	51	141	207	52	147	212	17,9
07.06.2011	19	58	166	33	30	132	17,9
07.06.2011	51	141	207	52	147	212	17,9
24.06.2011	13	123	195	48	81	168	17,9
16.05.2011	24	56	163	30	42	151	18,0
16.05.2011	14	87	198	54	199	238	18,0
17.05.2011	52	147	212	54	199	238	18,0
17.05.2011	15	59	166	55	126	201	18,0
17.05.2011	13	48	159	24	56	163	18,0
17.05.2011	13	48	159	27	46	152	18,0
25.05.2011	23	21	117	55	126	201	18,0

26.05.2011	15	59	166	51	141	207	18,0
27.05.2011	15	59	166	16	67	169	18,0
01.06.2011	14	87	198	52	147	212	18,0
06.06.2011	33	30	132	57	72	182	18,0
17.05.2011	15	59	166	23	21	117	18,1
17.05.2011	13	48	159	56	67	170	18,1
26.05.2011	23	21	117	53	152	218	18,1
27.05.2011	57	72	182	60	119	217	18,1
06.06.2011	51	141	207	52	147	212	18,1
28.06.2011	13	123	195	48	81	168	18,1
16.05.2011	23	21	117	53	152	218	18,2
17.05.2011	23	21	117	54	199	238	18,2
17.05.2011	13	48	159	57	72	182	18,2
25.05.2011	23	21	117	53	152	218	18,2
25.05.2011	27	46	152	57	72	182	18,2
25.05.2011	27	46	152	60	119	217	18,2
26.05.2011	14	87	198	23	21	117	18,2
26.05.2011	16	67	169	23	21	117	18,2
26.05.2011	22	24	124	51	141	207	18,2
02.06.2011	16	67	169	52	147	212	18,2
06.06.2011	16	67	169	53	152	218	18,2
17.06.2011	13	123	195	20	65	158	18,2
28.06.2011	13	123	195	20	65	158	18,2
16.05.2011	17	34	137	53	152	218	18,3
17.05.2011	15	59	166	16	67	169	18,3
17.05.2011	19	58	166	57	72	182	18,3
17.05.2011	15	59	166	22	24	124	18,3
17.05.2011	24	56	163	30	42	151	18,3
17.05.2011	27	46	152	30	42	151	18,3
17.05.2011	13	48	159	59	89	191	18,3
17.05.2011	13	48	159	60	119	217	18,3
25.05.2011	13	48	159	27	46	152	18,3
25.05.2011	27	46	152	33	30	132	18,3

25.05.2011	16	67	169	22	24	124	18,3
26.05.2011	16	67	169	51	141	207	18,3
27.05.2011	13	48	159	27	46	152	18,3
27.05.2011	27	46	152	56	67	170	18,3
02.06.2011	22	24	124	23	21	117	18,3
16.06.2011	13	123	195	20	65	158	18,3
16.05.2011	15	59	166	17	34	137	18,4
16.05.2011	15	59	166	54	199	238	18,4
16.05.2011	53	152	218	54	199	238	18,4
16.05.2011	30	42	151	33	30	132	18,4
17.05.2011	15	59	166	17	34	137	18,4
17.05.2011	33	30	132	60	119	217	18,4
17.05.2011	52	147	212	53	152	218	18,4
17.05.2011	24	56	163	33	30	132	18,4
17.05.2011	27	46	152	33	30	132	18,4
17.05.2011	30	42	151	56	67	170	18,4
25.05.2011	27	46	152	30	42	151	18,4
26.05.2011	22	24	124	23	21	117	18,4
26.05.2011	19	58	166	24	56	163	18,4
26.05.2011	19	58	166	27	46	152	18,4
27.05.2011	13	48	159	24	56	163	18,4
27.05.2011	27	46	152	57	72	182	18,4
06.06.2011	24	56	163	56	67	170	18,4
07.06.2011	14	87	198	16	67	169	18,4
23.06.2011	13	123	195	20	65	158	18,4
16.05.2011	13	48	159	60	119	217	18,5
16.05.2011	30	42	151	60	119	217	18,5
17.05.2011	19	58	166	56	67	170	18,5
17.05.2011	33	30	132	56	67	170	18,5
17.05.2011	30	42	151	57	72	182	18,5
25.05.2011	15	59	166	51	141	207	18,5
26.05.2011	15	59	166	23	21	117	18,5
27.05.2011	13	48	159	19	58	166	18,5

27.05.2011	19	58	166	56	67	170	18,5
27.05.2011	24	56	163	56	67	170	18,5
27.05.2011	24	56	163	57	72	182	18,5
02.06.2011	19	58	166	24	56	163	18,5
06.06.2011	15	59	166	16	67	169	18,5
16.05.2011	57	72	182	60	119	217	18,6
17.05.2011	33	30	132	57	72	182	18,6
17.05.2011	30	42	151	59	89	191	18,6
17.05.2011	30	42	151	60	119	217	18,6
25.05.2011	27	46	152	58	71	182	18,6
26.05.2011	14	87	198	15	59	166	18,6
26.05.2011	14	87	198	22	24	124	18,6
27.05.2011	19	58	166	57	72	182	18,6
02.06.2011	19	58	166	57	72	182	18,6
03.06.2011	16	67	169	52	147	212	18,6
07.06.2011	19	58	166	57	72	182	18,6
28.06.2011	13	123	195	48	81	168	18,6
17.05.2011	33	30	132	59	89	191	18,7
17.05.2011	33	30	132	60	119	217	18,7
27.05.2011	13	48	159	60	119	217	18,7
27.05.2011	56	67	170	60	119	217	18,7
03.06.2011	14	87	198	51	141	207	18,7
06.06.2011	15	59	166	16	67	169	18,7
06.06.2011	22	24	124	53	152	218	18,7
06.06.2011	30	42	151	58	71	182	18,7
23.06.2011	28	53	146	35	50	138	18,7
26.05.2011	33	30	132	60	119	217	18,8
27.05.2011	57	72	182	60	119	217	18,8
16.05.2011	14	87	198	53	152	218	18,9
16.05.2011	19	58	166	33	30	132	18,9
16.05.2011	24	56	163	27	46	152	18,9
17.05.2011	22	24	124	52	147	212	18,9
17.05.2011	16	67	169	22	24	124	18,9

06.06.2011	22	24	124	23	21	117	18,9
07.06.2011	30	42	151	58	71	182	18,9
07.06.2011	22	24	124	23	21	117	18,9
07.06.2011	24	56	163	56	67	170	18,9
07.06.2011	24	56	163	33	30	132	18,9
16.05.2011	14	87	198	15	59	166	19,0
16.05.2011	51	141	207	52	147	212	19,0
17.05.2011	15	59	166	53	152	218	19,0
26.05.2011	23	21	117	53	152	218	19,0
26.05.2011	15	59	166	52	147	212	19,0
01.06.2011	19	58	166	27	46	152	19,0
03.06.2011	19	42	151	24	30	132	19,0
16.05.2011	57	72	182	60	119	217	19,1
16.05.2011	23	21	117	55	126	201	19,1
03.06.2011	19	42	151	57	67	170	19,1
16.05.2011	56	67	170	59	89	191	19,2
17.05.2011	33	30	132	57	72	182	19,2
25.05.2011	53	152	218	55	126	201	19,2
02.06.2011	24	56	163	57	72	182	19,2
06.06.2011	19	58	166	56	67	170	19,2
17.06.2011	20	65	158	48	81	168	19,2
24.06.2011	13	123	195	20	65	158	19,2
16.05.2011	23	21	117	53	152	218	19,3
16.05.2011	19	58	166	24	56	163	19,3
17.05.2011	27	46	152	57	72	182	19,3
17.05.2011	30	42	151	57	72	182	19,3
25.05.2011	23	21	117	55	126	201	19,3
02.06.2011	52	147	212	53	152	218	19,3
06.06.2011	15	59	166	16	67	169	19,3
07.06.2011	15	59	166	23	21	117	19,3
16.05.2011	15	59	166	23	21	117	19,4
18.05.2011	19	58	166	24	56	163	19,4
25.05.2011	13	48	159	27	46	152	19,4

26.05.2011	14	87	198	17	34	137	19,4
02.06.2011	30	56	163	57	30	132	19,4
02.06.2011	24	56	163	33	30	132	19,4
17.05.2011	15	59	166	53	152	218	19,5
25.05.2011	22	24	124	55	126	201	19,5
07.06.2011	15	59	166	22	24	124	19,5
07.06.2011	24	56	163	56	67	170	19,5
17.05.2011	14	87	198	15	59	166	19,6
17.05.2011	15	59	166	55	126	201	19,6
18.05.2011	52	147	212	55	126	201	19,6
18.05.2011	24	56	163	27	46	152	19,6
26.05.2011	24	56	163	60	119	217	19,6
02.06.2011	14	87	198	15	59	166	19,6
02.06.2011	22	24	124	53	152	218	19,6
06.06.2011	19	58	166	24	56	163	19,6
17.05.2011	13	48	159	57	72	182	19,7
18.05.2011	51	141	207	52	147	212	19,7
26.05.2011	17	34	137	22	24	124	19,7
26.05.2011	16	67	169	55	126	201	19,7
02.06.2011	16	67	169	23	21	117	19,7
03.06.2011	15	59	166	52	147	212	19,7
26.05.2011	14	87	198	55	126	201	19,8
17.05.2011	15	59	166	23	21	117	19,9
17.05.2011	17	34	137	51	141	207	19,9
01.06.2011	15	59	166	53	152	218	19,9
07.06.2011	14	87	198	16	67	169	19,9
22.06.2011	13	123	195	48	81	168	19,9
16.05.2011	51	141	207	54	199	238	20,0
17.05.2011	15	59	166	55	126	201	20,0
17.05.2011	17	34	137	55	126	201	20,0
17.05.2011	23	21	117	51	141	207	20,0
17.05.2011	17	34	137	54	199	238	20,0
17.05.2011	23	21	117	54	199	238	20,0

25.05.2011	22	24	124	23	21	117	20,0
01.06.2011	14	87	198	53	152	218	20,0
01.06.2011	16	67	169	22	24	124	20,0
02.06.2011	23	21	117	53	152	218	20,0
07.06.2011	22	24	124	53	152	218	20,0
16.06.2011	20	65	158	48	81	168	20,0
16.05.2011	57	72	182	59	89	191	20,1
17.05.2011	22	24	124	53	152	218	20,1
17.05.2011	14	87	198	55	126	201	20,1
26.05.2011	19	58	166	60	119	217	20,1
26.05.2011	56	67	170	60	119	217	20,1
02.06.2011	14	87	198	22	24	124	20,1
06.06.2011	24	56	163	27	46	152	20,1
06.06.2011	24	56	163	56	67	170	20,1
06.06.2011	22	24	124	51	141	207	20,1
22.06.2011	13	123	195	48	81	168	20,1
03.06.2011	14	87	198	51	141	207	20,2
25.05.2011	22	24	124	51	141	207	20,3
01.06.2011	15	59	166	22	24	124	20,3
03.06.2011	51	141	207	53	152	218	20,3
06.06.2011	15	59	166	52	147	212	20,3
07.06.2011	33	30	132	57	72	182	20,3
16.05.2011	17	34	137	53	152	218	20,4
16.05.2011	53	152	218	54	199	238	20,4
26.05.2011	27	46	152	60	119	217	20,4
27.05.2011	17	34	137	23	21	117	20,4
07.06.2011	14	87	198	15	59	166	20,4
22.06.2011	13	123	195	20	65	158	20,4
16.05.2011	15	59	166	53	152	218	20,5
16.05.2011	22	24	124	53	152	218	20,5
16.05.2011	14	87	198	54	199	238	20,5
16.05.2011	15	59	166	54	199	238	20,5
16.05.2011	54	199	238	55	126	201	20,5

18.05.2011	23	21	117	53	152	218	20,5
25.05.2011	14	87	198	51	141	207	20,5
25.05.2011	53	152	218	55	126	201	20,5
06.06.2011	14	87	198	15	59	166	20,5
16.05.2011	14	87	198	53	152	218	20,6
16.05.2011	16	67	169	54	199	238	20,6
16.05.2011	17	34	137	54	199	238	20,6
16.05.2011	22	24	124	54	199	238	20,6
17.05.2011	17	34	137	53	152	218	20,6
01.06.2011	15	59	166	16	67	169	20,6
28.06.2011	20	65	158	48	81	168	20,6
16.05.2011	23	21	117	54	199	238	20,7
18.05.2011	17	34	137	53	152	218	20,7
02.06.2011	51	141	207	52	147	212	20,7
07.06.2011	27	46	152	33	30	132	20,7
16.05.2011	53	152	218	54	199	238	20,8
18.05.2011	15	59	166	53	152	218	20,8
18.05.2011	16	67	169	53	152	218	20,8
25.05.2011	22	24	124	51	141	207	20,8
25.05.2011	22	24	124	53	152	218	20,8
25.05.2011	15	59	166	23	21	117	20,8
01.06.2011	27	46	152	33	30	132	20,8
02.06.2011	16	67	169	51	141	207	20,8
02.06.2011	22	24	124	51	141	207	20,8
17.05.2011	58	71	182	59	89	191	20,9
26.05.2011	19	58	166	30	42	151	20,9
26.05.2011	19	58	166	57	72	182	20,9
02.06.2011	23	21	117	52	147	212	20,9
03.06.2011	14	87	198	15	59	166	20,9
06.06.2011	22	24	124	53	152	218	20,9
07.06.2011	56	67	170	57	72	182	20,9
23.06.2011	20	65	158	48	81	168	20,9
17.05.2011	15	59	166	55	126	201	21,0

26.05.2011	22	24	124	53	152	218	21,0
02.06.2011	14	87	198	16	67	169	21,0
29.06.2011	13	123	195	48	81	168	21,0
17.05.2011	33	30	132	56	67	170	21,1
25.05.2011	51	141	207	52	147	212	21,1
27.05.2011	14	87	198	16	67	169	21,1
07.06.2011	23	21	117	51	141	207	21,1
16.05.2011	13	48	159	56	67	170	21,2
17.05.2011	22	24	124	53	152	218	21,2
26.05.2011	15	59	166	55	126	201	21,2
25.05.2011	22	24	124	23	21	117	21,3
26.05.2011	19	58	166	56	67	170	21,3
27.05.2011	17	34	137	53	152	218	21,3
02.06.2011	23	21	117	52	147	212	21,3
02.06.2011	30	42	151	58	30	132	21,3
02.06.2011	33	56	163	56	72	182	21,3
02.06.2011	24	56	163	56	67	170	21,3
07.06.2011	14	87	198	52	147	212	21,3
07.06.2011	27	46	152	57	72	182	21,3
16.05.2011	15	59	166	55	126	201	21,4
17.05.2011	56	67	170	60	119	217	21,4
17.05.2011	15	59	166	51	141	207	21,4
06.06.2011	22	24	124	53	152	218	21,4
16.05.2011	53	152	218	55	126	201	21,5
17.05.2011	14	87	198	15	59	166	21,5
26.05.2011	16	67	169	23	21	117	21,5
01.06.2011	16	67	169	53	152	218	21,5
06.06.2011	51	141	207	53	152	218	21,5
17.05.2011	19	58	166	30	42	151	21,6
17.05.2011	19	58	166	57	72	182	21,6
17.05.2011	19	58	166	59	89	191	21,6
26.05.2011	27	46	152	60	119	217	21,6
06.06.2011	19	58	166	24	56	163	21,6

16.05.2011	13	48	159	57	72	182	21,7
17.05.2011	13	48	159	19	58	166	21,7
17.05.2011	19	58	166	27	46	152	21,7
17.05.2011	19	58	166	56	67	170	21,7
17.05.2011	24	56	163	59	89	191	21,7
26.05.2011	13	48	159	19	58	166	21,7
02.06.2011	27	56	163	56	46	152	21,7
07.06.2011	51	141	207	52	147	212	21,7
29.06.2011	20	65	158	48	81	168	21,7
17.05.2011	19	58	166	33	30	132	21,8
17.05.2011	13	48	159	24	56	163	21,8
17.05.2011	24	56	163	27	46	152	21,8
17.05.2011	24	56	163	30	42	151	21,8
17.05.2011	24	56	163	56	67	170	21,8
17.05.2011	24	56	163	57	72	182	21,8
17.05.2011	33	30	132	57	72	182	21,8
17.05.2011	33	30	132	59	89	191	21,8
25.05.2011	17	34	137	22	24	124	21,8
01.06.2011	15	59	166	23	21	117	21,8
02.06.2011	52	147	212	53	152	218	21,8
03.06.2011	23	21	117	52	147	212	21,8
06.06.2011	15	59	166	52	147	212	21,8
06.06.2011	24	56	163	56	67	170	21,8
07.06.2011	15	59	166	53	152	218	21,8
17.05.2011	22	24	124	55	126	201	21,9
17.05.2011	19	58	166	30	42	151	21,9
17.05.2011	13	48	159	33	30	132	21,9
17.05.2011	27	46	152	33	30	132	21,9
17.05.2011	30	42	151	33	30	132	21,9
17.05.2011	33	30	132	56	67	170	21,9
27.05.2011	16	67	169	53	152	218	21,9
03.06.2011	14	87	198	16	67	169	21,9
16.05.2011	30	42	151	56	67	170	22,0

24.06.2011	13	123	195	20	65	158	22,0
17.05.2011	52	147	212	53	152	218	22,1
26.05.2011	15	59	166	51	141	207	22,1
26.05.2011	14	87	198	55	126	201	22,1
26.05.2011	17	34	137	53	152	218	22,1
27.05.2011	23	21	117	55	126	201	22,1
02.06.2011	52	147	212	53	152	218	22,1
07.06.2011	24	56	163	56	67	170	22,1
24.06.2011	20	65	158	48	81	168	22,1
17.05.2011	13	48	159	19	58	166	22,2
26.05.2011	22	24	124	51	141	207	22,2
01.06.2011	19	58	166	33	30	132	22,2
02.06.2011	15	59	166	16	67	169	22,2
03.06.2011	15	59	166	16	67	169	22,2
07.06.2011	16	67	169	23	21	117	22,2
07.06.2011	24	56	163	56	67	170	22,2
23.06.2011	20	65	158	48	81	168	22,2
25.05.2011	14	87	198	23	21	117	22,3
02.06.2011	14	87	198	52	147	212	22,3
03.06.2011	19	42	151	27	67	170	22,3
07.06.2011	24	56	163	33	30	132	22,3
06.06.2011	16	67	169	52	147	212	22,4
07.06.2011	16	67	169	53	152	218	22,4
07.06.2011	24	56	163	56	67	170	22,4
17.05.2011	15	59	166	54	199	238	22,5
26.05.2011	51	141	207	52	147	212	22,5
06.06.2011	14	87	198	51	141	207	22,5
16.05.2011	30	42	151	57	72	182	22,6
25.05.2011	24	56	163	57	72	182	22,6
06.06.2011	24	56	163	56	67	170	22,6
18.05.2011	23	21	117	51	141	207	22,7
25.05.2011	17	34	137	23	21	117	22,7
26.05.2011	16	67	169	22	24	124	22,7

07.06.2011	14	87	198	15	59	166	22,7
17.05.2011	15	59	166	17	34	137	22,8
17.05.2011	51	141	207	54	199	238	22,8
01.06.2011	19	58	166	33	30	132	22,8
07.06.2011	16	67	169	22	24	124	22,8
15.06.2011	13	123	195	48	81	168	22,8
28.06.2011	20	65	158	48	81	168	22,8
18.05.2011	15	59	166	51	141	207	22,9
18.05.2011	16	67	169	51	141	207	22,9
18.05.2011	17	34	137	51	141	207	22,9
26.05.2011	17	34	137	51	141	207	22,9
26.05.2011	53	152	218	55	126	201	22,9
27.05.2011	53	152	218	55	126	201	22,9
06.06.2011	30	42	151	58	71	182	22,9
07.06.2011	22	24	124	53	152	218	22,9
07.06.2011	24	56	163	33	30	132	22,9
29.06.2011	13	123	195	20	65	158	22,9
16.05.2011	53	152	218	55	126	201	23,0
17.05.2011	53	152	218	55	126	201	23,0
18.05.2011	14	87	198	53	152	218	23,0
18.05.2011	23	21	117	53	152	218	23,0
25.05.2011	13	48	159	24	56	163	23,0
26.05.2011	53	152	218	55	126	201	23,0
27.05.2011	19	58	166	30	42	151	23,0
01.06.2011	19	58	166	27	46	152	23,0
01.06.2011	14	87	198	53	152	218	23,0
06.06.2011	23	21	117	52	147	212	23,0
17.05.2011	14	87	198	23	21	117	23,1
17.05.2011	24	56	163	60	119	217	23,1
25.05.2011	22	24	124	55	126	201	23,1
26.05.2011	14	87	198	55	126	201	23,1
26.05.2011	17	34	137	53	152	218	23,1
27.05.2011	24	56	163	30	42	151	23,1

06.06.2011	19	58	166	57	72	182	23,1
07.06.2011	30	42	151	58	71	182	23,1
16.05.2011	53	152	218	54	199	238	23,2
25.05.2011	13	48	159	19	58	166	23,2
26.05.2011	24	56	163	60	119	217	23,2
26.05.2011	16	67	169	55	126	201	23,2
27.05.2011	30	42	151	33	30	132	23,2
02.06.2011	15	59	166	23	21	117	23,2
03.06.2011	51	141	207	52	147	212	23,2
07.06.2011	33	30	132	57	72	182	23,2
17.05.2011	13	48	159	33	30	132	23,3
26.05.2011	33	30	132	60	119	217	23,3
26.05.2011	16	67	169	17	34	137	23,3
23.06.2011	20	65	158	34	47	141	23,3
17.05.2011	16	67	169	22	24	124	23,4
25.05.2011	14	87	198	53	152	218	23,4
25.05.2011	14	87	198	52	147	212	23,4
27.05.2011	27	46	152	30	42	151	23,4
07.06.2011	56	67	170	57	72	182	23,4
27.05.2011	22	24	124	53	152	218	23,5
01.06.2011	19	58	166	57	72	182	23,5
02.06.2011	23	21	117	52	147	212	23,5
03.06.2011	23	21	117	53	152	218	23,5
06.06.2011	51	141	207	53	152	218	23,5
07.06.2011	33	30	132	57	72	182	23,5
22.06.2011	13	123	195	48	81	168	23,5
18.05.2011	22	24	124	55	126	201	23,6
25.05.2011	14	87	198	53	152	218	23,6
02.06.2011	24	56	163	27	46	152	23,6
03.06.2011	23	21	117	53	152	218	23,6
06.06.2011	56	67	170	57	72	182	23,6
29.06.2011	13	123	195	20	65	158	23,6
18.05.2011	22	24	124	51	141	207	23,7

18.05.2011	14	87	198	55	126	201	23,7
01.06.2011	14	87	198	16	67	169	23,7
02.06.2011	16	67	169	23	21	117	23,7
02.06.2011	14	87	198	52	147	212	23,7
06.06.2011	27	46	152	57	72	182	23,7
16.05.2011	15	59	166	55	126	201	23,8
17.05.2011	19	58	166	27	46	152	23,8
18.05.2011	14	87	198	22	24	124	23,8
18.05.2011	14	87	198	54	199	238	23,8
25.05.2011	17	34	137	23	21	117	23,8
27.05.2011	19	58	166	56	67	170	23,8
06.06.2011	27	46	152	33	30	132	23,8
07.06.2011	52	147	212	53	152	218	23,8
17.05.2011	19	58	166	57	72	182	23,9
25.05.2011	23	21	117	55	126	201	23,9
27.05.2011	24	56	163	56	67	170	23,9
27.05.2011	33	30	132	56	67	170	23,9
29.06.2011	13	123	195	48	81	168	23,9
26.05.2011	51	141	207	52	147	212	24,0
01.06.2011	52	147	212	53	152	218	24,1
01.06.2011	19	58	166	24	56	163	24,1
03.06.2011	19	42	151	33	67	170	24,1
22.06.2011	13	123	195	20	65	158	24,1
18.05.2011	16	67	169	55	126	201	24,2
26.05.2011	14	87	198	53	152	218	24,2
27.05.2011	27	46	152	56	67	170	24,2
02.06.2011	15	59	166	16	67	169	24,2
03.06.2011	24	30	132	33	72	182	24,2
17.05.2011	16	67	169	51	141	207	24,3
18.05.2011	16	67	169	51	141	207	24,3
07.06.2011	33	30	132	57	72	182	24,3
28.06.2011	13	123	195	20	65	158	24,3
17.05.2011	16	67	169	54	199	238	24,4

17.05.2011	15	59	166	22	24	124	24,5
25.05.2011	15	59	166	51	141	207	24,5
07.06.2011	24	56	163	33	30	132	24,5
06.06.2011	16	67	169	52	147	212	24,6
06.06.2011	19	58	166	57	72	182	24,6
06.06.2011	27	46	152	57	72	182	24,6
16.06.2011	13	123	195	34	47	141	24,6
16.06.2011	13	123	195	20	65	158	24,6
29.06.2011	13	123	195	20	65	158	24,6
16.05.2011	22	24	124	55	126	201	24,7
17.05.2011	14	87	198	51	141	207	24,7
01.06.2011	14	87	198	53	152	218	24,7
02.06.2011	15	59	166	16	67	169	24,7
17.05.2011	23	21	117	51	141	207	24,8
17.05.2011	14	87	198	54	199	238	24,8
27.05.2011	15	59	166	22	24	124	24,8
02.06.2011	15	59	166	51	141	207	24,8
17.05.2011	23	21	117	52	147	212	24,9
17.05.2011	22	24	124	55	126	201	24,9
17.05.2011	19	58	166	60	119	217	24,9
18.05.2011	17	34	137	55	126	201	24,9
07.06.2011	24	56	163	33	30	132	24,9
07.06.2011	56	67	170	57	72	182	24,9
18.05.2011	17	34	137	51	141	207	25,0
01.06.2011	51	141	207	52	147	212	25,0
02.06.2011	16	67	169	23	21	117	25,0
02.06.2011	15	59	166	53	152	218	25,0
07.06.2011	24	56	163	56	67	170	25,0
07.06.2011	14	87	198	53	152	218	25,0
25.05.2011	15	59	166	23	21	117	25,1
26.05.2011	16	67	169	53	152	218	25,1
27.05.2011	22	24	124	51	141	207	25,1
02.06.2011	16	67	169	53	152	218	25,1

03.06.2011	19	30	132	33	67	170	25,1
03.06.2011	16	67	169	52	147	212	25,1
06.06.2011	14	87	198	23	21	117	25,1
06.06.2011	27	46	152	56	67	170	25,1
03.06.2011	30	42	151	58	67	170	25,2
23.06.2011	13	123	195	34	47	141	25,2
26.05.2011	53	152	218	55	126	201	25,3
02.06.2011	22	24	124	53	152	218	25,3
26.05.2011	14	87	198	23	21	117	25,4
01.06.2011	16	67	169	22	24	124	25,4
17.05.2011	14	87	198	53	152	218	25,5
25.05.2011	15	59	166	22	24	124	25,5
26.05.2011	14	87	198	53	152	218	25,5
02.06.2011	22	24	124	23	21	117	25,5
02.06.2011	14	87	198	23	21	117	25,5
23.06.2011	20	65	158	48	81	168	25,5
16.05.2011	19	58	166	27	46	152	25,6
17.05.2011	23	21	117	53	152	218	25,6
26.05.2011	23	21	117	55	126	201	25,6
27.05.2011	13	48	159	19	58	166	25,6
01.06.2011	15	59	166	16	67	169	25,6
01.06.2011	23	21	117	51	141	207	25,6
01.06.2011	16	67	169	53	152	218	25,6
23.06.2011	20	65	158	48	81	168	25,6
16.05.2011	19	58	166	56	67	170	25,7
17.05.2011	14	87	198	53	152	218	25,7
17.05.2011	53	152	218	55	126	201	25,7
26.05.2011	23	21	117	53	152	218	25,7
27.05.2011	13	48	159	24	56	163	25,7
03.06.2011	15	59	166	53	152	218	25,7
16.05.2011	27	46	152	56	67	170	25,8
16.05.2011	33	30	132	60	119	217	25,8
27.05.2011	13	48	159	33	30	132	25,8

02.06.2011	24	58	166	56	67	170	25,8
06.06.2011	19	58	166	57	72	182	25,8
15.06.2011	13	123	195	34	47	141	25,8
28.06.2011	13	123	195	20	65	158	25,8
07.06.2011	15	59	166	16	67	169	25,9
16.06.2011	20	65	158	48	81	168	25,9
16.05.2011	19	58	166	60	119	217	26,0
17.05.2011	14	87	198	53	152	218	26,0
17.05.2011	17	34	137	53	152	218	26,0
26.05.2011	15	59	166	53	152	218	26,0
27.05.2011	13	48	159	27	46	152	26,0
27.05.2011	30	42	151	60	119	217	26,0
01.06.2011	14	87	198	51	141	207	26,0
01.06.2011	16	67	169	51	141	207	26,0
07.06.2011	22	24	124	23	21	117	26,0
23.06.2011	20	65	158	34	47	141	26,0
17.05.2011	16	67	169	17	34	137	26,1
17.05.2011	15	59	166	53	152	218	26,1
17.05.2011	23	21	117	52	147	212	26,1
25.05.2011	17	34	137	53	152	218	26,1
17.05.2011	15	59	166	53	152	218	26,2
17.05.2011	57	72	182	59	89	191	26,2
26.05.2011	51	141	207	55	126	201	26,2
01.06.2011	14	87	198	16	67	169	26,2
02.06.2011	51	141	207	53	152	218	26,2
06.06.2011	14	87	198	22	24	124	26,2
17.05.2011	13	48	159	19	58	166	26,3
02.06.2011	15	59	166	51	141	207	26,3
23.06.2011	13	123	195	48	81	168	26,3
24.06.2011	20	65	158	48	81	168	26,3
26.05.2011	53	152	218	55	126	201	26,4
02.06.2011	16	67	169	53	152	218	26,4
06.06.2011	22	24	124	23	21	117	26,4

25.05.2011	51	141	207	55	126	201	26,5
25.05.2011	16	67	169	53	152	218	26,5
06.06.2011	56	67	170	57	72	182	26,5
17.05.2011	19	58	166	27	46	152	26,6
17.05.2011	19	58	166	30	42	151	26,6
17.05.2011	13	48	159	56	67	170	26,6
06.06.2011	56	67	170	57	72	182	26,6
22.06.2011	20	65	158	46	118	192	26,6
16.05.2011	23	21	117	51	141	207	26,7
17.05.2011	19	58	166	33	30	132	26,7
17.05.2011	13	48	159	33	30	132	26,7
25.05.2011	17	67	169	22	126	201	26,7
01.06.2011	33	30	132	56	67	170	26,7
06.06.2011	56	67	170	57	72	182	26,7
07.06.2011	16	67	169	23	21	117	26,7
16.05.2011	52	147	212	54	199	238	26,8
17.05.2011	30	42	151	57	72	182	26,8
26.05.2011	15	59	166	23	21	117	26,8
27.05.2011	56	67	170	60	119	217	26,8
01.06.2011	30	42	151	56	67	170	26,8
01.06.2011	19	58	166	27	46	152	26,8
01.06.2011	19	58	166	33	30	132	26,8
17.05.2011	19	58	166	30	42	151	26,9
01.06.2011	51	141	207	53	152	218	26,9
28.06.2011	13	123	195	48	81	168	26,9
17.05.2011	53	152	218	55	126	201	27,1
27.05.2011	14	87	198	16	67	169	27,1
27.05.2011	16	67	169	23	21	117	27,1
27.05.2011	16	67	169	55	126	201	27,1
28.06.2011	13	123	195	20	65	158	27,1
17.05.2011	52	147	212	55	126	201	27,2
17.05.2011	22	24	124	53	152	218	27,2
25.05.2011	52	147	212	53	152	218	27,2

26.05.2011	14	87	198	23	21	117	27,2
27.05.2011	16	67	169	17	34	137	27,2
16.05.2011	51	141	207	53	152	218	27,3
16.05.2011	51	141	207	55	126	201	27,3
01.06.2011	23	21	117	51	141	207	27,3
01.06.2011	19	58	166	56	67	170	27,3
02.06.2011	19	58	166	56	46	152	27,3
16.05.2011	14	87	198	51	141	207	27,4
16.05.2011	15	59	166	51	141	207	27,4
17.05.2011	58	71	182	59	89	191	27,4
17.05.2011	15	59	166	53	152	218	27,4
26.05.2011	51	141	207	53	152	218	27,4
15.06.2011	20	65	158	46	118	192	27,4
16.05.2011	16	67	169	51	141	207	27,5
16.05.2011	17	34	137	51	141	207	27,5
16.05.2011	22	24	124	51	141	207	27,5
17.05.2011	24	56	163	56	67	170	27,5
02.06.2011	16	67	169	53	152	218	27,5
02.06.2011	15	59	166	22	24	124	27,5
16.05.2011	23	21	117	51	141	207	27,6
17.05.2011	56	67	170	57	72	182	27,6
25.05.2011	14	87	198	53	152	218	27,6
02.06.2011	15	59	166	52	147	212	27,6
06.06.2011	16	67	169	51	141	207	27,6
17.05.2011	23	21	117	52	147	212	27,7
17.05.2011	33	30	132	56	67	170	27,7
17.05.2011	19	58	166	56	67	170	27,7
02.06.2011	16	67	169	51	141	207	27,7
03.06.2011	27	30	132	33	71	182	27,7
23.06.2011	13	123	195	48	81	168	27,7
17.05.2011	13	48	159	57	72	182	27,8
17.05.2011	24	56	163	59	89	191	27,8
17.05.2011	19	58	166	60	119	217	27,8

03.06.2011	14	87	198	53	152	218	27,8
24.06.2011	13	123	195	48	81	168	27,8
16.05.2011	16	67	169	53	152	218	27,9
17.05.2011	51	141	207	55	126	201	27,9
17.05.2011	13	48	159	19	58	166	27,9
17.05.2011	27	46	152	57	72	182	27,9
17.05.2011	13	48	159	19	58	166	27,9
17.05.2011	19	58	166	57	72	182	27,9
17.05.2011	19	58	166	59	89	191	27,9
18.05.2011	53	152	218	54	199	238	27,9
26.05.2011	14	87	198	51	141	207	27,9
07.06.2011	30	42	151	58	71	182	27,9
16.05.2011	15	59	166	16	67	169	28,0
17.05.2011	19	58	166	27	46	152	28,0
17.05.2011	19	58	166	27	46	152	28,0
17.05.2011	19	58	166	30	42	151	28,0
25.05.2011	15	59	166	17	34	137	28,0
26.05.2011	17	34	137	51	141	207	28,0
26.05.2011	14	87	198	53	152	218	28,0
01.06.2011	14	87	198	52	147	212	28,0
06.06.2011	52	147	212	53	152	218	28,0
17.05.2011	14	87	198	16	67	169	28,1
17.05.2011	16	67	169	17	34	137	28,1
17.05.2011	52	147	212	54	199	238	28,1
26.05.2011	15	59	166	23	21	117	28,1
06.06.2011	30	42	151	58	71	182	28,1
07.06.2011	51	141	207	52	147	212	28,1
17.05.2011	16	67	169	23	21	117	28,2
17.05.2011	15	59	166	16	67	169	28,2
25.05.2011	22	24	124	23	21	117	28,2
26.05.2011	22	24	124	51	141	207	28,2
16.05.2011	30	42	151	56	67	170	28,3
17.05.2011	30	42	151	59	89	191	28,3

17.05.2011	52	147	212	55	126	201	28,3
01.06.2011	15	59	166	23	21	117	28,3
02.06.2011	15	59	166	22	24	124	28,3
03.06.2011	33	42	151	56	72	182	28,3
16.05.2011	13	48	159	56	67	170	28,4
16.05.2011	33	30	132	56	67	170	28,4
16.05.2011	56	67	170	57	72	182	28,4
16.05.2011	56	67	170	59	89	191	28,4
26.05.2011	16	67	169	22	24	124	28,4
06.06.2011	15	59	166	16	67	169	28,4
26.05.2011	19	58	166	30	42	151	28,5
02.06.2011	14	87	198	15	59	166	28,5
16.05.2011	56	67	170	60	119	217	28,6
17.05.2011	59	89	191	60	119	217	28,6
27.05.2011	13	48	159	60	119	217	28,6
01.06.2011	24	56	163	33	30	132	28,7
17.05.2011	19	58	166	60	119	217	28,8
17.05.2011	22	24	124	52	147	212	28,8
26.05.2011	16	67	169	23	21	117	28,8
01.06.2011	33	30	132	57	72	182	28,8
06.06.2011	16	67	169	53	152	218	28,8
06.06.2011	22	24	124	23	21	117	28,8
07.06.2011	14	87	198	53	152	218	28,8
17.05.2011	51	141	207	52	147	212	28,9
27.05.2011	22	24	124	53	152	218	28,9
25.05.2011	15	59	166	17	34	137	29,0
26.05.2011	16	67	169	53	152	218	29,0
26.05.2011	51	141	207	53	152	218	29,1
26.05.2011	51	141	207	52	147	212	29,1
01.06.2011	15	59	166	23	21	117	29,1
06.06.2011	15	59	166	22	24	124	29,1
07.06.2011	56	67	170	57	72	182	29,1
23.06.2011	13	123	195	34	47	141	29,1

06.06.2011	15	59	166	22	24	124	29,2
17.05.2011	24	56	163	57	72	182	29,3
25.05.2011	13	48	159	33	30	132	29,3
26.05.2011	15	59	166	53	152	218	29,3
26.05.2011	16	67	169	53	152	218	29,3
06.06.2011	24	56	163	33	30	132	29,4
23.06.2011	13	123	195	34	47	141	29,4
16.05.2011	24	56	163	56	67	170	29,5
25.05.2011	16	67	169	22	24	124	29,5
01.06.2011	14	87	198	16	67	169	29,5
07.06.2011	15	59	166	16	67	169	29,5
01.06.2011	22	24	124	23	21	117	29,6
25.05.2011	16	67	169	52	147	212	29,7
02.06.2011	27	46	152	33	30	132	29,7
03.06.2011	14	87	198	16	67	169	29,7
03.06.2011	15	59	166	52	147	212	29,7
27.06.2011	13	123	195	20	65	158	29,7
17.05.2011	27	46	152	60	119	217	29,8
25.05.2011	16	67	169	22	24	124	29,8
07.06.2011	15	59	166	16	67	169	29,8
17.05.2011	13	48	159	60	119	217	29,9
26.05.2011	16	67	169	52	147	212	29,9
16.05.2011	19	58	166	60	119	217	30,0
02.06.2011	15	59	166	22	24	124	30,0
23.06.2011	13	123	195	48	81	168	30,0
02.06.2011	24	56	163	33	30	132	30,1
02.06.2011	27	46	152	56	67	170	30,1
22.06.2011	20	65	158	48	81	168	30,1
25.05.2011	14	87	198	16	67	169	30,2
02.06.2011	14	87	198	22	24	124	30,2
02.06.2011	33	30	132	57	72	182	30,2
15.06.2011	13	123	195	48	81	168	30,3
16.05.2011	24	56	163	56	67	170	30,4

17.05.2011	52	147	212	54	199	238	30,4
07.06.2011	14	87	198	22	24	124	30,4
25.05.2011	14	87	198	16	67	169	30,5
02.06.2011	22	24	124	52	147	212	30,5
07.06.2011	15	59	166	52	147	212	30,5
16.05.2011	14	87	198	55	126	201	30,6
25.05.2011	15	59	166	23	21	117	30,6
25.05.2011	15	59	166	51	141	207	30,6
03.06.2011	23	21	117	52	147	212	30,6
16.05.2011	24	56	163	60	119	217	30,7
27.05.2011	22	24	124	23	21	117	30,7
02.06.2011	24	56	163	56	67	170	30,7
06.06.2011	24	56	163	56	67	170	30,7
28.06.2011	20	65	158	34	47	141	30,7
28.06.2011	34	47	141	48	81	168	30,7
17.05.2011	15	59	166	23	21	117	30,8
25.05.2011	13	48	159	56	67	170	30,8
25.05.2011	13	48	159	57	72	182	30,8
25.05.2011	13	48	159	60	119	217	30,8
25.05.2011	16	67	169	22	24	124	30,8
02.06.2011	56	67	170	57	72	182	30,8
07.06.2011	15	59	166	52	147	212	30,8
16.05.2011	19	58	166	59	89	191	30,9
17.05.2011	30	42	151	60	119	217	30,9
25.05.2011	13	48	159	30	42	151	30,9
01.06.2011	14	87	198	51	141	207	30,9
02.06.2011	15	59	166	53	152	218	30,9
06.06.2011	15	59	166	52	147	212	30,9
07.06.2011	14	87	198	51	141	207	30,9
26.05.2011	17	34	137	53	152	218	31,0
01.06.2011	15	59	166	16	67	169	31,0
06.06.2011	14	87	198	16	67	169	31,0
29.06.2011	20	65	158	34	47	141	31,0

02.06.2011	14	87	198	16	67	169	31,1
06.06.2011	30	42	151	58	71	182	31,1
17.05.2011	14	87	198	22	24	124	31,2
01.06.2011	23	21	117	51	141	207	31,2
03.06.2011	14	87	198	16	67	169	31,2
25.05.2011	23	21	117	55	126	201	31,3
02.06.2011	33	30	132	56	67	170	31,3
16.05.2011	19	58	166	59	89	191	31,4
17.05.2011	54	199	238	55	126	201	31,4
06.06.2011	33	30	132	57	72	182	31,4
25.05.2011	14	87	198	51	141	207	31,5
02.06.2011	16	67	169	52	147	212	31,5
03.06.2011	33	30	132	57	71	182	31,5
07.06.2011	15	59	166	22	24	124	31,5
22.06.2011	13	123	195	34	47	141	31,5
25.05.2011	14	87	198	16	67	169	31,6
06.06.2011	14	87	198	53	152	218	31,6
25.05.2011	14	87	198	22	24	124	31,7
17.05.2011	23	21	117	51	141	207	31,8
17.05.2011	23	21	117	53	152	218	31,8
25.05.2011	14	87	198	52	147	212	31,8
01.06.2011	14	87	198	23	21	117	31,8
17.06.2011	34	47	141	48	81	168	31,8
17.05.2011	59	89	191	60	119	217	31,9
18.05.2011	23	21	117	55	126	201	31,9
25.05.2011	13	48	159	19	58	166	31,9
25.05.2011	15	59	166	51	141	207	31,9
06.06.2011	52	147	212	53	152	218	31,9
27.06.2011	20	65	158	48	81	168	31,9
16.05.2011	22	24	124	51	141	207	32,0
18.05.2011	14	87	198	23	21	117	32,0
18.05.2011	22	24	124	55	126	201	32,0
25.05.2011	17	34	137	55	126	201	32,0

26.05.2011	33	30	132	56	67	170	32,0
06.06.2011	14	87	198	22	24	124	32,0
07.06.2011	19	58	166	57	72	182	32,0
17.05.2011	19	58	166	24	56	163	32,1
18.05.2011	14	87	198	22	24	124	32,1
25.05.2011	13	48	159	24	56	163	32,1
25.05.2011	17	34	137	22	24	124	32,1
02.06.2011	51	141	207	52	147	212	32,1
23.06.2011	20	65	158	48	81	168	32,1
17.05.2011	22	24	124	51	141	207	32,2
18.05.2011	17	34	137	55	126	201	32,2
26.05.2011	52	147	212	53	152	218	32,2
26.05.2011	13	48	159	19	58	166	32,2
26.05.2011	19	58	166	57	72	182	32,2
26.05.2011	19	58	166	60	119	217	32,2
02.06.2011	15	59	166	53	152	218	32,2
07.06.2011	27	46	152	57	72	182	32,2
16.06.2011	34	47	141	48	81	168	32,2
18.05.2011	14	87	198	17	34	137	32,3
18.05.2011	15	59	166	55	126	201	32,3
18.05.2011	16	67	169	55	126	201	32,3
25.05.2011	13	48	159	27	46	152	32,3
26.05.2011	22	24	124	52	147	212	32,3
02.06.2011	14	87	198	15	59	166	32,3
02.06.2011	30	56	163	58	30	132	32,3
03.06.2011	30	87	218	58	71	182	32,3
17.05.2011	22	24	124	54	199	238	32,4
18.05.2011	14	87	198	15	59	166	32,4
18.05.2011	14	87	198	16	67	169	32,4
26.05.2011	16	67	169	55	126	201	32,4
27.05.2011	22	24	124	55	126	201	32,4
17.05.2011	19	58	166	59	89	191	32,5
18.05.2011	51	141	207	55	126	201	32,5

25.05.2011	15	59	166	51	141	207	32,5
25.05.2011	51	141	207	52	147	212	32,5
26.05.2011	23	21	117	51	141	207	32,5
17.06.2011	34	47	141	48	81	168	32,5
18.05.2011	14	87	198	51	141	207	32,6
17.05.2011	59	89	191	60	119	217	32,7
26.05.2011	16	67	169	55	126	201	32,7
01.06.2011	22	24	124	23	21	117	32,7
02.06.2011	19	58	166	56	67	170	32,7
03.06.2011	15	59	166	53	152	218	32,7
06.06.2011	15	59	166	52	147	212	32,7
06.06.2011	56	67	170	57	72	182	32,7
27.05.2011	14	87	198	22	24	124	32,8
27.05.2011	17	34	137	22	24	124	32,8
23.06.2011	13	123	195	48	81	168	32,8
17.05.2011	14	87	198	17	34	137	32,9
27.05.2011	22	24	124	23	21	117	32,9
01.06.2011	22	24	124	51	141	207	32,9
29.06.2011	34	47	141	48	81	168	32,9
16.05.2011	13	48	159	19	58	166	33,0
17.05.2011	19	58	166	30	42	151	33,0
17.05.2011	23	21	117	53	152	218	33,1
26.05.2011	24	56	163	56	67	170	33,1
26.05.2011	27	46	152	56	67	170	33,1
17.05.2011	14	87	198	51	141	207	33,2
17.05.2011	14	87	198	52	147	212	33,2
16.06.2011	13	123	195	34	47	141	33,2
17.06.2011	20	65	158	48	81	168	33,2
23.06.2011	13	123	195	48	81	168	33,2
16.05.2011	23	21	117	52	147	212	33,3
17.05.2011	16	67	169	17	34	137	33,3
17.05.2011	16	67	169	55	126	201	33,3
03.06.2011	19	30	132	56	67	170	33,3

07.06.2011	51	141	207	53	152	218	33,3
07.06.2011	14	87	198	22	24	124	33,3
16.05.2011	30	42	151	59	89	191	33,4
17.05.2011	16	67	169	23	21	117	33,4
17.05.2011	19	58	166	30	42	151	33,4
26.05.2011	15	59	166	22	24	124	33,4
26.05.2011	17	34	137	23	21	117	33,4
27.05.2011	15	59	166	52	147	212	33,4
06.06.2011	14	87	198	22	24	124	33,5
17.05.2011	16	67	169	22	24	124	33,6
25.05.2011	15	59	166	16	67	169	33,6
25.05.2011	16	67	169	52	147	212	33,6
26.05.2011	14	87	198	23	21	117	33,6
17.05.2011	19	58	166	24	56	163	33,7
17.05.2011	17	34	137	53	152	218	33,7
07.06.2011	30	42	151	58	71	182	33,7
22.06.2011	20	65	158	48	81	168	33,7
27.06.2011	20	65	158	34	47	141	33,7
16.05.2011	19	58	166	30	42	151	33,8
17.05.2011	23	21	117	53	152	218	33,8
02.06.2011	19	58	166	57	72	182	33,8
07.06.2011	30	42	151	57	72	182	33,8
17.05.2011	15	59	166	51	141	207	33,9
17.05.2011	56	67	170	57	72	182	33,9
26.05.2011	23	21	117	51	141	207	33,9
01.06.2011	14	87	198	23	21	117	33,9
02.06.2011	22	24	124	23	21	117	33,9
28.06.2011	20	65	158	34	47	141	33,9
17.05.2011	15	59	166	52	147	212	34,0
17.05.2011	33	30	132	57	72	182	34,0
25.05.2011	14	87	198	17	34	137	34,0
26.05.2011	16	67	169	52	147	212	34,0
02.06.2011	19	58	166	33	30	132	34,0

03.06.2011	14	87	198	22	24	124	34,0
17.05.2011	16	67	169	53	152	218	34,1
18.05.2011	23	21	117	52	147	212	34,1
28.06.2011	34	47	141	48	81	168	34,1
17.05.2011	27	46	152	56	67	170	34,2
18.05.2011	22	24	124	52	147	212	34,2
25.05.2011	14	87	198	52	147	212	34,2
26.05.2011	16	67	169	23	21	117	34,2
17.05.2011	27	46	152	33	30	132	34,3
18.05.2011	52	147	212	53	152	218	34,3
06.06.2011	14	87	198	22	24	124	34,3
07.06.2011	14	87	198	51	141	207	34,3
07.06.2011	14	87	198	52	147	212	34,3
18.05.2011	17	34	137	52	147	212	34,4
25.05.2011	13	48	159	33	30	132	34,4
26.05.2011	17	34	137	23	21	117	34,4
01.06.2011	15	59	166	51	141	207	34,4
18.05.2011	15	59	166	52	147	212	34,5
18.05.2011	16	67	169	52	147	212	34,5
25.05.2011	13	48	159	56	67	170	34,5
27.05.2011	14	87	198	22	24	124	34,5
01.06.2011	14	87	198	55	126	201	34,5
17.05.2011	22	24	124	51	141	207	34,6
25.05.2011	23	21	117	51	141	207	34,6
23.06.2011	13	123	195	34	47	141	34,6
18.05.2011	14	87	198	53	152	218	34,7
25.05.2011	22	24	124	53	152	218	34,7
25.05.2011	22	24	124	55	126	201	34,7
27.05.2011	16	67	169	51	141	207	34,7
01.06.2011	16	67	169	23	21	117	34,7
18.05.2011	51	141	207	52	147	212	34,8
25.05.2011	14	87	198	53	152	218	34,8
07.06.2011	14	87	198	23	21	117	34,8

17.05.2011	51	141	207	55	126	201	34,9
17.05.2011	52	147	212	55	126	201	34,9
01.06.2011	16	67	169	51	141	207	34,9
03.06.2011	19	30	132	56	67	170	34,9
17.05.2011	13	48	159	57	72	182	35,0
25.05.2011	14	87	198	17	34	137	35,0
22.06.2011	13	123	195	34	47	141	35,0
16.05.2011	19	58	166	56	67	170	35,1
17.05.2011	15	59	166	54	199	238	35,1
25.05.2011	15	59	166	16	67	169	35,1
27.05.2011	15	59	166	16	67	169	35,2
16.05.2011	33	30	132	56	67	170	35,3
07.06.2011	14	87	198	16	67	169	35,4
07.06.2011	51	141	207	52	147	212	35,4
07.06.2011	16	67	169	51	141	207	35,4
23.06.2011	13	123	195	48	81	168	35,4
07.06.2011	14	87	198	16	67	169	35,5
17.05.2011	52	147	212	54	199	238	35,6
25.05.2011	15	59	166	16	67	169	35,6
26.05.2011	14	87	198	22	24	124	35,6
03.06.2011	14	87	198	22	24	124	35,6
16.05.2011	15	59	166	54	199	238	35,7
17.05.2011	16	67	169	22	24	124	35,7
17.05.2011	14	87	198	16	67	169	35,7
17.05.2011	16	67	169	55	126	201	35,8
26.05.2011	51	141	207	55	126	201	35,8
01.06.2011	15	59	166	53	152	218	35,8
22.06.2011	34	47	141	48	81	168	35,8
17.05.2011	14	87	198	17	34	137	35,9
17.05.2011	17	34	137	55	126	201	35,9
27.05.2011	30	42	151	58	71	182	35,9
02.06.2011	14	87	198	16	67	169	35,9
07.06.2011	14	87	198	51	141	207	36,0

07.06.2011	16	67	169	52	147	212	36,0
17.05.2011	16	67	169	23	21	117	36,1
27.05.2011	16	67	169	17	34	137	36,1
27.05.2011	14	87	198	16	67	169	36,1
02.06.2011	51	141	207	53	152	218	36,1
07.06.2011	16	67	169	22	24	124	36,1
23.06.2011	13	123	195	20	65	158	36,1
16.05.2011	53	152	218	55	126	201	36,2
17.05.2011	14	87	198	16	67	169	36,2
26.05.2011	16	67	169	17	34	137	36,2
26.05.2011	14	87	198	17	34	137	36,2
01.06.2011	52	147	212	53	152	218	36,2
02.06.2011	14	87	198	23	21	117	36,2
17.05.2011	17	34	137	23	21	117	36,3
25.05.2011	14	87	198	15	59	166	36,3
26.05.2011	14	87	198	55	126	201	36,3
25.05.2011	15	59	166	23	21	117	36,4
26.05.2011	23	21	117	51	141	207	36,4
22.06.2011	13	123	195	34	47	141	36,4
02.06.2011	15	59	166	23	21	117	36,5
03.06.2011	14	87	198	51	141	207	36,5
18.05.2011	15	59	166	53	152	218	36,6
02.06.2011	14	87	198	15	59	166	36,6
02.06.2011	33	30	132	57	72	182	36,6
25.05.2011	52	147	212	55	126	201	36,7
02.06.2011	14	87	198	22	24	124	36,7
16.05.2011	24	56	163	59	89	191	36,8
25.05.2011	51	141	207	53	152	218	36,8
26.05.2011	23	21	117	55	126	201	36,9
03.06.2011	27	67	170	33	71	182	36,9
17.05.2011	14	87	198	51	141	207	37,0
26.05.2011	23	21	117	53	152	218	37,0
07.06.2011	15	59	166	23	21	117	37,0

17.05.2011	15	59	166	51	141	207	37,1
17.05.2011	17	34	137	51	141	207	37,1
25.05.2011	23	21	117	53	152	218	37,1
27.05.2011	16	67	169	17	34	137	37,1
27.05.2011	16	67	169	22	24	124	37,1
02.06.2011	14	87	198	22	24	124	37,1
06.06.2011	52	147	212	53	152	218	37,1
07.06.2011	19	58	166	57	72	182	37,1
17.05.2011	14	87	198	51	141	207	37,2
17.05.2011	14	87	198	54	199	238	37,2
18.05.2011	14	87	198	51	141	207	37,2
25.05.2011	22	24	124	52	147	212	37,2
23.06.2011	34	47	141	48	81	168	37,2
17.05.2011	15	59	166	54	199	238	37,3
17.05.2011	17	34	137	54	199	238	37,3
06.06.2011	30	42	151	58	71	182	37,3
07.06.2011	16	67	169	22	24	124	37,3
07.06.2011	14	87	198	51	141	207	37,3
25.05.2011	16	67	169	55	126	201	37,4
27.05.2011	16	67	169	55	126	201	37,4
23.06.2011	13	123	195	34	47	141	37,4
01.06.2011	51	141	207	52	147	212	37,5
03.06.2011	14	87	198	22	24	124	37,5
07.06.2011	22	24	124	52	147	212	37,5
17.05.2011	57	72	182	59	89	191	37,6
26.05.2011	14	87	198	15	59	166	37,6
27.05.2011	16	67	169	23	21	117	37,6
03.06.2011	51	141	207	52	147	212	37,6
17.05.2011	30	42	151	56	67	170	37,7
25.05.2011	22	24	124	55	126	201	37,7
26.05.2011	23	21	117	51	141	207	37,7
27.05.2011	16	67	169	55	126	201	37,7
03.06.2011	14	87	198	23	21	117	37,7

17.05.2011	15	59	166	22	24	124	37,8
17.05.2011	27	46	152	59	89	191	37,8
25.05.2011	51	141	207	52	147	212	37,8
01.06.2011	22	24	124	23	21	117	37,8
17.05.2011	15	59	166	52	147	212	37,9
01.06.2011	23	21	117	52	147	212	37,9
22.06.2011	13	123	195	48	81	168	37,9
16.05.2011	27	46	152	59	89	191	38,0
16.05.2011	33	30	132	59	89	191	38,0
16.05.2011	14	87	198	51	141	207	38,0
26.05.2011	23	21	117	52	147	212	38,0
02.06.2011	19	58	166	57	72	182	38,0
16.06.2011	34	47	141	48	81	168	38,0
16.05.2011	13	48	159	59	89	191	38,1
17.05.2011	16	67	169	53	152	218	38,1
18.05.2011	23	21	117	53	152	218	38,1
26.05.2011	19	58	166	60	119	217	38,1
02.06.2011	19	58	166	27	46	152	38,1
16.05.2011	59	89	191	60	119	217	38,2
18.05.2011	22	24	124	53	152	218	38,2
18.05.2011	22	24	124	53	152	218	38,2
01.06.2011	23	21	117	53	152	218	38,2
02.06.2011	14	87	198	53	152	218	38,2
06.06.2011	15	59	166	51	141	207	38,2
16.06.2011	13	123	195	34	47	141	38,2
06.06.2011	15	59	166	51	141	207	38,3
23.06.2011	13	123	195	48	81	168	38,3
18.05.2011	17	34	137	53	152	218	38,4
27.05.2011	15	59	166	22	24	124	38,4
01.06.2011	15	59	166	23	21	117	38,4
16.06.2011	34	47	141	48	81	168	38,4
17.05.2011	14	87	198	53	152	218	38,5
18.05.2011	15	59	166	53	152	218	38,5

18.05.2011	16	67	169	53	152	218	38,5
01.06.2011	23	21	117	51	141	207	38,5
02.06.2011	30	42	151	58	71	182	38,5
24.06.2011	20	65	158	34	47	141	38,5
17.05.2011	24	56	163	33	30	132	38,6
16.06.2011	13	123	195	34	47	141	38,6
16.05.2011	59	89	191	60	119	217	38,7
17.05.2011	13	48	159	56	67	170	38,7
25.05.2011	13	48	159	30	42	151	38,7
16.05.2011	22	24	124	52	147	212	38,8
17.05.2011	27	46	152	56	67	170	38,8
17.05.2011	56	67	170	60	119	217	38,8
18.05.2011	16	67	169	53	152	218	38,8
18.05.2011	51	141	207	53	152	218	38,8
16.06.2011	13	123	195	48	81	168	38,8
22.06.2011	20	65	158	48	81	168	38,8
17.05.2011	33	30	132	60	119	217	38,9
17.05.2011	33	30	132	59	89	191	39,0
25.05.2011	13	48	159	57	72	182	39,0
25.05.2011	13	48	159	60	119	217	39,0
03.06.2011	14	87	198	53	152	218	39,0
01.06.2011	14	87	198	23	21	117	39,1
16.05.2011	22	24	124	55	126	201	39,2
17.06.2011	20	65	158	34	47	141	39,2
17.05.2011	17	34	137	51	141	207	39,3
25.05.2011	15	59	166	22	24	124	39,3
25.05.2011	14	87	198	55	126	201	39,3
03.06.2011	14	87	198	23	21	117	39,3
06.06.2011	14	87	198	15	59	166	39,3
17.05.2011	16	67	169	22	24	124	39,4
17.05.2011	51	141	207	55	126	201	39,4
25.05.2011	22	24	124	51	141	207	39,4
26.05.2011	16	67	169	55	126	201	39,4

07.06.2011	15	59	166	51	141	207	39,4
16.05.2011	57	72	182	59	89	191	39,5
16.05.2011	17	34	137	53	152	218	39,5
17.05.2011	17	34	137	22	24	124	39,5
17.05.2011	23	21	117	51	141	207	39,5
18.05.2011	17	34	137	53	152	218	39,5
03.06.2011	30	42	151	58	71	182	39,5
07.06.2011	15	59	166	16	67	169	39,5
07.06.2011	51	141	207	53	152	218	39,5
17.05.2011	30	42	151	33	30	132	39,6
26.05.2011	22	24	124	55	126	201	39,6
26.05.2011	16	67	169	53	152	218	39,6
02.06.2011	16	67	169	52	147	212	39,6
02.06.2011	15	59	166	53	152	218	39,6
06.06.2011	30	42	151	57	72	182	39,6
07.06.2011	14	87	198	15	59	166	39,6
22.06.2011	13	123	195	20	65	158	39,6
16.05.2011	56	67	170	59	89	191	39,7
16.05.2011	17	34	137	55	126	201	39,7
16.05.2011	54	199	238	55	126	201	39,7
17.05.2011	22	24	124	51	141	207	39,7
26.05.2011	14	87	198	51	141	207	39,7
06.06.2011	16	67	169	22	24	124	39,7
16.05.2011	16	67	169	53	152	218	39,8
03.06.2011	27	42	151	33	67	170	39,8
03.06.2011	33	30	132	56	71	182	39,8
17.05.2011	19	58	166	60	119	217	39,9
18.05.2011	51	141	207	54	199	238	39,9
26.05.2011	14	87	198	22	24	124	39,9
02.06.2011	15	59	166	51	141	207	39,9
02.06.2011	14	87	198	53	152	218	39,9
03.06.2011	24	42	151	33	71	182	39,9
07.06.2011	15	59	166	51	141	207	39,9

07.06.2011	16	67	169	53	152	218	39,9
07.06.2011	15	59	166	53	152	218	39,9
28.06.2011	20	65	158	34	47	141	39,9
28.06.2011	34	47	141	48	81	168	39,9
16.05.2011	51	141	207	55	126	201	40,0
26.05.2011	14	87	198	53	152	218	40,0
03.06.2011	14	87	198	52	147	212	40,0
07.06.2011	14	87	198	23	21	117	40,0
17.05.2011	22	24	124	53	152	218	40,1
17.05.2011	19	58	166	24	56	163	40,1
17.05.2011	19	58	166	27	46	152	40,1
18.05.2011	54	199	238	55	126	201	40,1
26.05.2011	14	87	198	16	67	169	40,1
06.06.2011	14	87	198	15	59	166	40,1
17.05.2011	14	87	198	51	141	207	40,2
17.05.2011	23	21	117	51	141	207	40,2
17.05.2011	51	141	207	55	126	201	40,2
17.05.2011	51	141	207	53	152	218	40,2
17.05.2011	19	58	166	56	67	170	40,2
26.05.2011	16	67	169	17	34	137	40,2
02.06.2011	27	46	152	33	30	132	40,2
17.05.2011	19	58	166	57	72	182	40,3
02.06.2011	30	56	163	56	30	132	40,3
16.05.2011	14	87	198	55	126	201	40,4
17.05.2011	14	87	198	54	199	238	40,4
17.05.2011	19	58	166	59	89	191	40,4
17.05.2011	19	58	166	60	119	217	40,4
03.06.2011	24	30	132	33	71	182	40,4
06.06.2011	14	87	198	51	141	207	40,4
06.06.2011	14	87	198	15	59	166	40,4
07.06.2011	15	59	166	51	141	207	40,4
07.06.2011	51	141	207	53	152	218	40,4
17.05.2011	17	34	137	54	199	238	40,5

17.05.2011	54	199	238	55	126	201	40,5
25.05.2011	15	59	166	17	147	212	40,5
02.06.2011	56	67	170	57	72	182	40,5
03.06.2011	23	21	117	52	147	212	40,5
06.06.2011	15	59	166	53	152	218	40,5
07.06.2011	24	56	163	30	42	151	40,5
07.06.2011	16	67	169	23	21	117	40,5
01.06.2011	14	87	198	23	21	117	40,6
06.06.2011	23	21	117	53	152	218	40,6
17.05.2011	13	48	159	60	119	217	40,7
17.05.2011	23	21	117	54	199	238	40,7
02.06.2011	33	30	132	56	67	170	40,7
03.06.2011	15	59	166	22	24	124	40,7
06.06.2011	14	87	198	52	147	212	40,7
07.06.2011	27	46	152	57	72	182	40,7
16.05.2011	23	21	117	55	126	201	40,8
17.05.2011	52	147	212	55	126	201	40,8
17.05.2011	22	24	124	54	199	238	40,8
25.05.2011	14	87	198	17	34	137	40,8
02.06.2011	24	56	163	33	30	132	40,8
17.05.2011	33	30	132	60	119	217	40,9
25.05.2011	15	59	166	53	152	218	40,9
01.06.2011	16	67	169	23	21	117	40,9
02.06.2011	16	67	169	53	152	218	40,9
06.06.2011	23	21	117	51	141	207	40,9
03.06.2011	33	112	278	57	71	182	41,0
07.06.2011	23	21	117	51	141	207	41,0
16.06.2011	13	123	195	34	47	141	41,0
16.06.2011	20	65	158	34	47	141	41,0
17.05.2011	24	56	163	60	119	217	41,1
16.06.2011	34	47	141	48	81	168	41,1
25.05.2011	14	87	198	23	21	117	41,2
16.05.2011	51	141	207	53	152	218	41,3

17.05.2011	16	67	169	55	126	201	41,4
17.05.2011	53	152	218	54	199	238	41,4
18.05.2011	54	199	238	55	126	201	41,4
23.06.2011	20	65	158	34	47	141	41,4
28.06.2011	20	65	158	34	47	141	41,4
29.06.2011	34	47	141	48	81	168	41,4
17.05.2011	16	67	169	51	141	207	41,5
18.05.2011	14	87	198	54	199	238	41,5
25.05.2011	14	87	198	22	24	124	41,5
01.06.2011	23	21	117	51	141	207	41,5
06.06.2011	51	141	207	52	147	212	41,5
15.06.2011	13	123	195	34	47	141	41,5
29.06.2011	20	65	158	34	47	141	41,5
25.05.2011	51	141	207	52	147	212	41,6
26.05.2011	14	87	198	55	126	201	41,6
26.05.2011	14	87	198	17	34	137	41,6
01.06.2011	14	87	198	16	67	169	41,6
02.06.2011	19	58	166	27	46	152	41,6
03.06.2011	14	87	198	53	152	218	41,6
17.06.2011	13	123	195	48	81	168	41,6
06.06.2011	15	59	166	52	147	212	41,7
17.05.2011	14	87	198	16	67	169	41,8
25.05.2011	16	67	169	23	21	117	41,8
26.05.2011	16	67	169	52	147	212	41,8
07.06.2011	24	56	163	57	72	182	41,8
29.06.2011	20	65	158	34	47	141	41,8
25.05.2011	16	67	169	51	141	207	41,9
03.06.2011	15	59	166	22	24	124	41,9
01.06.2011	15	59	166	53	152	218	42,0
07.06.2011	14	87	198	53	152	218	42,0
01.06.2011	16	67	169	52	147	212	42,1
15.06.2011	13	123	195	34	47	141	42,1
16.05.2011	15	59	166	23	21	117	42,2

17.05.2011	22	24	124	52	147	212	42,2
17.05.2011	58	71	182	59	89	191	42,2
26.05.2011	16	67	169	17	34	137	42,2
26.05.2011	22	24	124	52	147	212	42,2
02.06.2011	19	58	166	24	56	163	42,2
06.06.2011	22	24	124	51	141	207	42,2
17.05.2011	14	87	198	51	141	207	42,3
02.06.2011	15	59	166	51	141	207	42,3
07.06.2011	24	56	163	57	72	182	42,3
16.05.2011	56	67	170	60	119	217	42,4
02.06.2011	16	67	169	22	24	124	42,4
16.05.2011	13	48	159	56	67	170	42,5
16.05.2011	56	67	170	57	72	182	42,5
16.05.2011	56	67	170	59	89	191	42,5
16.05.2011	56	67	170	57	72	182	42,5
28.06.2011	13	123	195	34	47	141	42,5
16.05.2011	19	58	166	56	67	170	42,6
16.05.2011	27	46	152	56	67	170	42,6
16.05.2011	30	42	151	56	67	170	42,6
16.05.2011	33	30	132	56	67	170	42,6
16.05.2011	30	42	151	56	67	170	42,6
17.05.2011	15	59	166	16	67	169	42,6
16.05.2011	13	48	159	56	67	170	42,7
03.06.2011	14	87	198	52	147	212	42,7
15.06.2011	28	53	146	46	118	192	42,7
25.05.2011	17	34	137	22	24	124	42,8
25.05.2011	14	87	198	55	126	201	42,8
01.06.2011	15	59	166	16	67	169	42,8
02.06.2011	15	59	166	23	21	117	42,8
06.06.2011	15	59	166	23	21	117	42,8
02.06.2011	14	87	198	51	141	207	43,0
06.06.2011	15	59	166	53	152	218	43,0
17.05.2011	15	59	166	53	152	218	43,1

26.05.2011	16	67	169	51	141	207	43,1
26.05.2011	30	42	151	56	67	170	43,1
06.06.2011	15	59	166	52	147	212	43,1
02.06.2011	16	67	169	22	24	124	43,2
03.06.2011	15	59	166	23	21	117	43,2
24.06.2011	20	65	158	34	47	141	43,2
27.06.2011	13	123	195	48	81	168	43,2
25.05.2011	23	21	117	51	141	207	43,3
23.06.2011	13	123	195	34	47	141	43,3
25.05.2011	16	67	169	51	141	207	43,4
17.05.2011	15	59	166	51	141	207	43,5
17.05.2011	14	87	198	54	199	238	43,5
25.05.2011	17	34	137	51	141	207	43,5
16.06.2011	34	47	141	48	81	168	43,5
17.05.2011	17	34	137	55	126	201	43,6
17.05.2011	24	56	163	56	67	170	43,6
17.05.2011	30	42	151	56	67	170	43,6
25.05.2011	14	87	198	17	34	137	43,6
25.05.2011	15	59	166	17	34	137	43,6
17.05.2011	24	56	163	33	30	132	43,7
17.05.2011	30	42	151	33	30	132	43,7
18.05.2011	52	147	212	54	199	238	43,7
26.05.2011	22	24	124	53	152	218	43,7
28.06.2011	13	123	195	34	47	141	43,7
01.06.2011	16	67	169	22	24	124	43,8
01.06.2011	51	141	207	52	147	212	43,8
16.06.2011	13	123	195	34	47	141	43,8
29.06.2011	13	123	195	34	47	141	43,8
06.06.2011	24	56	163	30	42	151	43,9
17.06.2011	13	123	195	34	47	141	43,9
26.05.2011	16	67	169	22	24	124	44,0
01.06.2011	51	141	207	55	126	201	44,0
06.06.2011	30	42	151	57	72	182	44,0

23.06.2011	20	65	158	34	47	141	44,1
26.05.2011	14	87	198	53	152	218	44,2
27.05.2011	23	21	117	51	141	207	44,2
27.05.2011	51	141	207	53	152	218	44,2
03.06.2011	14	87	198	23	21	117	44,2
06.06.2011	14	87	198	51	141	207	44,2
17.05.2011	13	48	159	56	67	170	44,3
02.06.2011	27	46	152	30	42	151	44,3
17.05.2011	16	67	169	54	199	238	44,4
28.06.2011	13	123	195	34	47	141	44,4
26.05.2011	23	21	117	52	147	212	44,5
26.05.2011	22	24	124	53	152	218	44,5
06.06.2011	14	87	198	53	152	218	44,5
25.05.2011	22	24	124	23	21	117	44,6
25.05.2011	23	21	117	55	126	201	44,6
27.06.2011	20	65	158	34	47	141	44,7
16.05.2011	14	87	198	52	147	212	44,8
26.05.2011	14	87	198	55	126	201	44,8
02.06.2011	27	46	152	56	67	170	44,8
17.05.2011	13	48	159	59	89	191	44,9
06.06.2011	30	42	151	57	72	182	44,9
07.06.2011	30	42	151	57	72	182	44,9
16.06.2011	13	123	195	34	47	141	45,0
17.05.2011	30	42	151	59	89	191	45,2
17.05.2011	33	30	132	59	89	191	45,2
23.06.2011	20	65	158	48	81	168	45,2
17.05.2011	27	46	152	59	89	191	45,3
07.06.2011	14	87	198	51	141	207	45,3
15.06.2011	35	50	138	46	118	192	45,3
27.06.2011	34	47	141	13	123	195	45,3
25.05.2011	14	87	198	23	21	117	45,4
25.05.2011	15	59	166	22	24	124	45,4
25.05.2011	15	59	166	53	152	218	45,5

17.05.2011	13	48	159	59	89	191	45,6
25.05.2011	16	67	169	53	152	218	45,6
26.05.2011	14	87	198	52	147	212	45,6
01.06.2011	16	67	169	52	147	212	45,6
01.06.2011	14	87	198	16	67	169	45,6
27.06.2011	20	65	158	34	47	141	45,6
25.05.2011	15	59	166	52	147	212	45,7
01.06.2011	22	24	124	53	152	218	45,7
27.06.2011	34	47	141	48	81	168	45,7
17.05.2011	51	141	207	53	152	218	45,8
06.06.2011	16	67	169	22	24	124	45,8
16.05.2011	58	71	182	60	119	217	45,9
17.05.2011	19	58	166	57	72	182	45,9
17.05.2011	53	152	218	55	126	201	45,9
25.05.2011	15	59	166	55	126	201	45,9
06.06.2011	51	141	207	53	152	218	45,9
15.06.2011	34	47	141	48	81	168	46,0
22.06.2011	13	123	195	34	47	141	46,0
17.05.2011	15	59	166	54	199	238	46,1
01.06.2011	30	42	151	58	71	182	46,1
02.06.2011	16	67	169	22	24	124	46,1
25.05.2011	51	141	207	55	126	201	46,2
29.06.2011	13	123	195	34	47	141	46,3
18.05.2011	14	87	198	54	199	238	46,4
18.05.2011	23	21	117	54	199	238	46,5
07.06.2011	15	59	166	51	141	207	46,5
28.06.2011	20	65	158	34	47	141	46,5
25.05.2011	22	24	124	55	126	201	46,6
26.05.2011	17	34	137	22	24	124	46,6
23.06.2011	13	123	195	34	47	141	46,6
24.06.2011	13	123	195	34	47	141	46,6
27.06.2011	20	65	158	34	47	141	46,6
27.05.2011	51	141	207	52	147	212	46,7

25.05.2011	14	87	198	55	126	201	46,8
26.05.2011	15	59	166	23	21	117	46,8
26.05.2011	13	48	159	56	67	170	46,8
01.06.2011	23	21	117	53	152	218	46,8
06.06.2011	15	59	166	23	21	117	46,8
15.06.2011	13	123	195	34	47	141	46,8
26.05.2011	56	67	170	57	72	182	46,9
26.05.2011	56	67	170	60	119	217	46,9
01.06.2011	15	59	166	51	141	207	46,9
03.06.2011	22	24	124	51	141	207	46,9
23.06.2011	20	65	158	34	47	141	46,9
26.05.2011	22	24	124	23	21	117	47,0
28.06.2011	13	123	195	34	47	141	47,0
17.05.2011	16	67	169	23	21	117	47,1
07.06.2011	22	24	124	51	141	207	47,1
07.06.2011	24	56	163	30	42	151	47,2
23.06.2011	20	65	158	34	47	141	47,2
25.05.2011	51	141	207	53	152	218	47,3
26.05.2011	14	87	198	15	59	166	47,3
03.06.2011	15	59	166	52	147	212	47,3
18.05.2011	53	152	218	54	199	238	47,4
25.05.2011	15	59	166	55	126	201	47,4
07.06.2011	14	87	198	16	67	169	47,4
06.06.2011	24	56	163	30	42	151	47,5
07.06.2011	15	59	166	22	24	124	47,5
17.05.2011	16	67	169	55	126	201	47,6
23.06.2011	20	65	158	34	47	141	47,6
16.05.2011	15	59	166	22	24	124	47,7
17.05.2011	23	21	117	52	147	212	47,7
17.05.2011	16	67	169	54	199	238	47,7
02.06.2011	23	21	117	51	141	207	47,7
03.06.2011	27	72	182	56	71	182	47,7
15.06.2011	34	47	141	48	81	168	47,7

16.06.2011	20	65	158	34	47	141	47,7
17.05.2011	14	87	198	52	147	212	47,8
17.05.2011	52	147	212	55	126	201	47,8
03.06.2011	22	24	124	51	141	207	47,8
17.05.2011	17	34	137	54	199	238	47,9
07.06.2011	15	59	166	23	21	117	47,9
07.06.2011	14	87	198	22	24	124	48,0
18.05.2011	15	59	166	51	141	207	48,1
01.06.2011	51	141	207	53	152	218	48,1
17.06.2011	34	47	141	48	81	168	48,1
25.05.2011	15	59	166	52	147	212	48,2
27.05.2011	15	59	166	16	67	169	48,2
02.06.2011	23	21	117	51	141	207	48,2
17.05.2011	17	34	137	55	126	201	48,3
18.05.2011	15	59	166	55	126	201	48,3
06.06.2011	15	59	166	23	21	117	48,3
06.06.2011	15	59	166	23	21	117	48,3
17.05.2011	53	152	218	54	199	238	48,4
17.05.2011	58	71	182	60	119	217	48,4
27.05.2011	14	87	198	51	141	207	48,4
02.06.2011	30	46	152	33	30	132	48,4
06.06.2011	15	59	166	22	24	124	48,4
17.05.2011	51	141	207	54	199	238	48,5
02.06.2011	24	56	163	30	42	151	48,5
17.05.2011	53	152	218	55	126	201	48,6
02.06.2011	30	42	151	33	30	132	48,6
25.05.2011	14	87	198	16	67	169	48,7
26.05.2011	22	24	124	52	147	212	48,7
17.05.2011	53	152	218	54	199	238	48,8
02.06.2011	30	42	151	57	72	182	48,8
03.06.2011	23	21	117	51	141	207	48,8
06.06.2011	14	87	198	15	59	166	48,8
17.05.2011	14	87	198	17	34	137	48,9

16.05.2011	52	147	212	53	152	218	49,0
06.06.2011	23	21	117	51	141	207	49,0
16.06.2011	20	65	158	34	47	141	49,0
22.06.2011	13	123	195	34	47	141	49,0
02.06.2011	22	24	124	52	147	212	49,1
02.06.2011	16	67	169	22	24	124	49,1
02.06.2011	27	58	166	30	30	132	49,2
02.06.2011	27	46	152	30	42	151	49,2
03.06.2011	15	59	166	23	21	117	49,2
27.06.2011	34	47	141	48	81	168	49,2
27.06.2011	20	65	158	34	47	141	49,2
16.05.2011	16	67	169	55	126	201	49,3
16.05.2011	27	46	152	56	67	170	49,3
26.05.2011	15	59	166	16	67	169	49,3
01.06.2011	16	67	169	53	152	218	49,3
02.06.2011	15	59	166	53	152	218	49,3
17.06.2011	13	123	195	20	65	158	49,3
27.06.2011	34	47	141	48	81	168	49,3
27.06.2011	13	123	195	34	47	141	49,3
06.06.2011	22	24	124	52	147	212	49,4
27.06.2011	20	65	158	34	47	141	49,4
17.05.2011	14	87	198	52	147	212	49,5
17.05.2011	15	59	166	17	34	137	49,5
01.06.2011	22	24	124	53	152	218	49,5
02.06.2011	19	58	166	30	42	151	49,5
26.05.2011	22	24	124	51	141	207	49,6
26.05.2011	23	21	117	53	152	218	49,7
15.06.2011	34	47	141	48	81	168	49,7
06.06.2011	15	59	166	53	152	218	49,8
27.05.2011	17	34	137	22	24	124	50,0
27.05.2011	30	42	151	57	72	182	50,0
27.05.2011	13	48	159	30	42	151	50,1
27.05.2011	30	42	151	56	67	170	50,1

03.06.2011	15	59	166	22	24	124	50,1
17.06.2011	20	65	158	34	47	141	50,1
27.06.2011	34	47	141	48	81	168	50,1
07.06.2011	14	87	198	22	24	124	50,2
16.05.2011	14	87	198	17	34	137	50,3
25.05.2011	14	87	198	16	67	169	50,3
25.05.2011	16	67	169	23	21	117	50,3
07.06.2011	24	56	163	30	42	151	50,3
27.06.2011	13	123	195	34	47	141	50,3
17.05.2011	24	56	163	57	72	182	50,4
07.06.2011	15	59	166	16	67	169	50,4
15.06.2011	34	47	141	48	81	168	50,4
24.06.2011	13	123	195	34	47	141	50,4
27.06.2011	13	123	195	34	47	141	50,4
28.06.2011	13	123	195	34	47	141	50,4
17.05.2011	16	67	169	54	199	238	50,5
02.06.2011	30	42	151	57	72	182	50,5
16.05.2011	14	87	198	16	67	169	50,6
16.05.2011	27	46	152	59	89	191	50,6
01.06.2011	23	21	117	52	147	212	50,6
27.06.2011	13	123	195	34	47	141	50,6
03.06.2011	24	42	151	56	71	182	50,7
06.06.2011	30	42	151	57	72	182	50,7
17.05.2011	57	72	182	59	89	191	50,8
03.06.2011	16	67	169	22	24	124	50,8
03.06.2011	56	122	302	57	71	182	50,8
26.05.2011	14	87	198	17	34	137	50,9
27.05.2011	22	24	124	52	147	212	50,9
02.06.2011	30	46	152	56	30	132	50,9
07.06.2011	14	87	198	52	147	212	50,9
25.05.2011	52	147	212	53	152	218	51,0
02.06.2011	30	42	151	56	67	170	51,0
22.06.2011	34	47	141	48	81	168	51,0

27.05.2011	22	24	124	51	141	207	51,1
25.05.2011	23	21	117	52	147	212	51,2
02.06.2011	14	87	198	51	141	207	51,2
28.06.2011	13	123	195	20	65	158	51,2
01.06.2011	16	67	169	53	152	218	51,3
01.06.2011	15	59	166	16	67	169	51,3
28.06.2011	13	123	195	48	81	168	51,3
17.05.2011	30	42	151	57	72	182	51,4
26.05.2011	23	21	117	55	126	201	51,4
27.05.2011	22	24	124	55	126	201	51,5
01.06.2011	16	67	169	53	152	218	51,5
22.06.2011	20	65	158	34	47	141	51,5
25.05.2011	15	59	166	23	21	117	51,6
01.06.2011	23	21	117	52	147	212	51,6
15.06.2011	13	123	195	34	47	141	51,6
01.06.2011	16	67	169	52	147	212	51,7
02.06.2011	24	56	163	30	42	151	51,7
07.06.2011	16	67	169	51	141	207	51,7
06.06.2011	14	87	198	15	59	166	51,8
27.06.2011	34	47	141	48	81	168	51,8
02.06.2011	27	46	152	30	42	151	51,9
28.06.2011	20	65	158	34	47	141	51,9
28.06.2011	34	47	141	48	81	168	52,0
16.05.2011	15	59	166	51	141	207	52,1
16.05.2011	51	141	207	53	152	218	52,1
17.05.2011	52	147	212	53	152	218	52,1
26.05.2011	15	59	166	16	67	169	52,2
26.05.2011	17	34	137	22	24	124	52,2
01.06.2011	16	67	169	51	141	207	52,2
02.06.2011	16	67	169	51	141	207	52,2
03.06.2011	15	59	166	23	21	117	52,2
03.06.2011	24	67	170	56	72	182	52,2
15.06.2011	34	47	141	48	81	168	52,2

16.05.2011	17	34	137	22	24	124	52,3
26.05.2011	16	67	169	51	141	207	52,3
02.06.2011	15	59	166	52	147	212	52,3
06.06.2011	23	21	117	51	141	207	52,3
07.06.2011	19	58	166	30	42	151	52,3
03.06.2011	56	67	170	57	71	182	52,4
25.05.2011	16	67	169	17	21	117	52,5
06.06.2011	23	21	117	53	152	218	52,5
26.05.2011	16	67	169	17	34	137	52,6
07.06.2011	23	21	117	51	141	207	52,6
28.06.2011	20	65	158	34	47	141	52,6
16.05.2011	16	67	169	22	24	124	52,7
26.05.2011	14	87	198	16	67	169	52,7
02.06.2011	15	59	166	52	147	212	52,7
02.06.2011	30	42	151	57	72	182	52,7
03.06.2011	16	67	169	23	21	117	52,7
06.06.2011	24	56	163	30	42	151	52,7
26.05.2011	15	59	166	16	67	169	52,8
06.06.2011	30	42	151	33	30	132	52,8
28.06.2011	13	123	195	34	47	141	52,8
22.06.2011	13	123	195	20	65	158	53,0
17.05.2011	16	67	169	52	147	212	53,1
02.06.2011	14	87	198	22	24	124	53,1
27.05.2011	16	67	169	51	141	207	53,2
07.06.2011	14	87	198	23	21	117	53,2
17.05.2011	56	67	170	59	89	191	53,3
27.05.2011	16	67	169	52	147	212	53,3
02.06.2011	14	87	198	52	147	212	53,3
02.06.2011	23	21	117	53	152	218	53,3
07.06.2011	30	42	151	57	72	182	53,3
17.05.2011	17	34	137	23	21	117	53,4
25.05.2011	14	87	198	23	21	117	53,4
07.06.2011	22	24	124	51	141	207	53,4

17.05.2011	57	72	182	58	71	182	53,5
25.05.2011	52	147	212	53	152	218	53,5
26.05.2011	15	59	166	53	152	218	53,5
27.05.2011	16	67	169	51	141	207	53,6
06.06.2011	30	42	151	33	30	132	53,6
16.05.2011	14	87	198	15	59	166	53,7
01.06.2011	51	141	207	53	152	218	53,7
02.06.2011	15	59	166	23	21	117	53,7
01.06.2011	15	59	166	51	141	207	53,8
25.05.2011	17	34	137	51	141	207	53,9
25.05.2011	15	59	166	53	152	218	54,0
03.06.2011	30	92	230	57	71	182	54,0
16.06.2011	13	123	195	34	47	141	54,0
18.05.2011	14	87	198	15	59	166	54,1
18.05.2011	15	59	166	23	21	117	54,1
25.05.2011	15	59	166	53	152	218	54,1
03.06.2011	24	30	132	30	71	182	54,1
07.06.2011	16	67	169	22	24	124	54,1
16.06.2011	34	47	141	48	81	168	54,1
23.06.2011	34	47	141	48	81	168	54,1
25.05.2011	22	24	124	52	147	212	54,3
07.06.2011	22	24	124	51	141	207	54,3
07.06.2011	51	141	207	53	152	218	54,3
16.06.2011	20	65	158	34	47	141	54,3
17.05.2011	15	59	166	52	147	212	54,5
17.05.2011	16	67	169	53	152	218	54,6
25.05.2011	16	67	169	17	34	137	54,6
03.06.2011	24	30	132	30	71	182	54,6
17.05.2011	17	34	137	23	21	117	54,7
17.05.2011	51	141	207	55	126	201	54,7
06.06.2011	27	46	152	30	42	151	54,7
17.05.2011	17	34	137	53	152	218	54,8
17.05.2011	54	199	238	55	126	201	54,9

26.05.2011	17	34	137	23	21	117	54,9
06.06.2011	30	42	151	57	72	182	54,9
23.06.2011	34	47	141	48	81	168	54,9
17.05.2011	24	56	163	59	89	191	55,0
26.05.2011	22	24	124	23	21	117	55,0
06.06.2011	14	87	198	51	141	207	55,0
07.06.2011	27	46	152	30	42	151	55,0
01.06.2011	22	24	124	51	141	207	55,1
03.06.2011	27	42	151	30	30	132	55,1
03.06.2011	30	67	170	56	72	182	55,1
03.06.2011	23	21	117	51	141	207	55,2
02.06.2011	30	42	151	58	71	182	55,3
26.05.2011	23	21	117	52	147	212	55,4
01.06.2011	15	59	166	51	141	207	55,4
23.06.2011	34	47	141	48	81	168	55,4
26.05.2011	16	67	169	55	126	201	55,5
03.06.2011	16	67	169	22	24	124	55,5
03.06.2011	27	42	151	56	30	132	55,5
06.06.2011	15	59	166	23	21	117	55,5
07.06.2011	24	56	163	30	42	151	55,5
07.06.2011	24	56	163	58	71	182	55,5
27.06.2011	13	123	195	20	65	158	55,5
25.05.2011	14	87	198	53	152	218	55,6
22.06.2011	20	65	158	48	81	168	55,6
06.06.2011	16	67	169	53	152	218	55,7
07.06.2011	14	87	198	52	147	212	55,7
26.05.2011	52	147	212	55	126	201	55,8
26.05.2011	51	141	207	53	152	218	55,8
22.06.2011	34	47	141	48	81	168	55,8
16.05.2011	17	34	137	23	21	117	55,9
26.05.2011	52	147	212	53	152	218	55,9
03.06.2011	22	24	124	53	152	218	55,9
24.06.2011	20	65	158	34	47	141	55,9

26.05.2011	22	24	124	51	141	207	56,0
06.06.2011	24	56	163	30	42	151	56,1
24.06.2011	13	123	195	34	47	141	56,1
29.06.2011	13	123	195	34	47	141	56,1
01.06.2011	56	67	170	58	71	182	56,2
25.05.2011	23	21	117	51	141	207	56,3
25.05.2011	17	34	137	51	141	207	56,3
26.05.2011	22	24	124	52	147	212	56,3
02.06.2011	22	24	124	23	21	117	56,3
03.06.2011	14	87	198	52	147	212	56,3
06.06.2011	14	87	198	16	67	169	56,3
07.06.2011	19	58	166	30	42	151	56,3
07.06.2011	24	56	163	30	42	151	56,3
22.06.2011	34	47	141	48	81	168	56,3
16.05.2011	16	67	169	23	21	117	56,4
02.06.2011	14	87	198	23	21	117	56,4
25.05.2011	23	21	117	51	141	207	56,5
27.05.2011	15	59	166	23	21	117	56,5
06.06.2011	22	24	124	53	152	218	56,5
25.05.2011	15	59	166	55	126	201	56,6
25.05.2011	22	24	124	51	141	207	56,6
17.05.2011	24	56	163	60	119	217	56,7
17.05.2011	51	141	207	55	126	201	56,7
06.06.2011	16	67	169	51	141	207	56,7
07.06.2011	27	46	152	30	42	151	56,7
07.06.2011	15	59	166	52	147	212	56,7
17.05.2011	52	147	212	53	152	218	56,8
17.05.2011	22	24	124	55	126	201	56,8
27.05.2011	15	59	166	53	152	218	56,8
06.06.2011	51	141	207	53	152	218	56,8
17.05.2011	59	89	191	60	119	217	56,9
06.06.2011	24	56	163	30	42	151	56,9
07.06.2011	24	56	163	30	42	151	56,9

15.06.2011	20	65	158	46	118	192	56,9
22.06.2011	34	47	141	48	81	168	56,9
17.05.2011	53	152	218	55	126	201	57,0
26.05.2011	17	34	137	23	21	117	57,0
02.06.2011	14	87	198	23	21	117	57,0
07.06.2011	19	58	166	30	42	151	57,0
23.06.2011	13	123	195	34	47	141	57,0
17.05.2011	51	141	207	55	126	201	57,1
07.06.2011	27	46	152	58	71	182	57,1
16.05.2011	13	48	159	59	89	191	57,2
16.05.2011	30	42	151	59	89	191	57,2
17.05.2011	15	59	166	51	141	207	57,2
03.06.2011	27	77	194	30	71	182	57,2
16.05.2011	57	72	182	59	89	191	57,3
17.05.2011	54	199	238	55	126	201	57,3
02.06.2011	51	141	207	52	147	212	57,3
07.06.2011	30	42	151	33	30	132	57,3
17.06.2011	13	123	195	34	47	141	57,3
16.05.2011	27	46	152	58	71	182	57,4
25.05.2011	14	87	198	17	34	137	57,4
07.06.2011	27	46	152	30	42	151	57,4
16.05.2011	15	59	166	53	152	218	57,5
17.05.2011	57	72	182	58	71	182	57,5
17.05.2011	14	87	198	22	24	124	57,5
26.05.2011	23	21	117	55	126	201	57,5
26.05.2011	14	87	198	22	24	124	57,5
02.06.2011	24	56	163	30	42	151	57,5
17.05.2011	27	46	152	58	71	182	57,6
17.05.2011	30	42	151	60	119	217	57,6
25.05.2011	15	59	166	55	126	201	57,6
01.06.2011	15	59	166	51	141	207	57,6
03.06.2011	16	67	169	22	24	124	57,6
07.06.2011	51	141	207	53	152	218	57,6

23.06.2011	34	47	141	48	81	168	57,6
17.05.2011	57	72	182	60	119	217	57,7
25.05.2011	15	59	166	16	67	169	57,7
06.06.2011	14	87	198	16	67	169	57,7
07.06.2011	51	141	207	53	152	218	57,7
17.05.2011	19	58	166	60	119	217	57,8
25.05.2011	16	67	169	53	152	218	57,8
28.06.2011	34	47	141	48	81	168	57,8
17.05.2011	51	141	207	52	147	212	57,9
25.05.2011	16	67	169	52	147	212	57,9
25.05.2011	16	67	169	55	126	201	57,9
22.06.2011	20	65	158	34	47	141	57,9
17.05.2011	15	59	166	22	24	124	58,0
17.05.2011	52	147	212	54	199	238	58,0
22.06.2011	13	123	195	34	47	141	58,0
02.06.2011	57	46	152	58	42	151	58,1
02.06.2011	30	42	151	56	67	170	58,1
23.06.2011	34	47	141	48	81	168	58,1
03.06.2011	16	67	169	23	21	117	58,2
01.06.2011	16	67	169	55	126	201	58,3
01.06.2011	24	56	163	30	42	151	58,3
02.06.2011	52	147	212	53	152	218	58,3
17.05.2011	27	46	152	59	89	191	58,4
02.06.2011	22	24	124	53	152	218	58,4
23.06.2011	34	47	141	48	81	168	58,4
17.05.2011	17	34	137	54	199	238	58,5
17.05.2011	13	48	159	59	89	191	58,5
18.05.2011	52	147	212	54	199	238	58,5
26.05.2011	51	141	207	53	152	218	58,5
02.06.2011	56	56	163	57	71	182	58,5
03.06.2011	27	42	151	30	30	132	58,5
07.06.2011	27	46	152	30	42	151	58,5
01.06.2011	16	67	169	53	152	218	58,6

06.06.2011	14	87	198	51	141	207	58,6
25.05.2011	15	59	166	17	34	137	58,7
25.05.2011	23	21	117	51	141	207	58,7
06.06.2011	15	59	166	22	24	124	58,7
06.06.2011	16	67	169	23	21	117	58,7
16.06.2011	34	47	141	48	81	168	58,7
16.05.2011	17	34	137	54	199	238	58,8
26.05.2011	14	87	198	15	59	166	58,8
01.06.2011	22	24	124	51	141	207	58,8
16.05.2011	52	147	212	55	126	201	58,9
26.05.2011	14	87	198	23	21	117	58,9
01.06.2011	30	42	151	56	67	170	58,9
28.06.2011	34	47	141	48	81	168	58,9
17.05.2011	58	71	182	60	119	217	59,0
25.05.2011	17	34	137	52	147	212	59,1
02.06.2011	16	67	169	51	141	207	59,2
16.05.2011	16	67	169	54	199	238	59,4
01.06.2011	22	24	124	51	141	207	59,4
07.06.2011	30	42	151	57	72	182	59,4
27.06.2011	34	47	141	48	81	168	59,4
17.05.2011	30	42	151	59	89	191	59,5
26.05.2011	23	21	117	52	147	212	59,5
01.06.2011	24	56	163	30	42	151	59,5
01.06.2011	30	42	151	33	30	132	59,5
01.06.2011	30	42	151	57	72	182	59,5
26.05.2011	51	141	207	53	152	218	59,6
06.06.2011	15	59	166	51	141	207	59,6
17.05.2011	16	67	169	53	152	218	59,7
26.05.2011	16	67	169	22	24	124	59,7
17.05.2011	17	34	137	22	24	124	59,8
17.05.2011	24	56	163	56	67	170	59,8
25.05.2011	15	59	166	55	126	201	59,8
25.05.2011	17	34	137	52	147	212	59,8

06.06.2011	22	24	124	52	147	212	59,8
26.05.2011	22	24	124	55	126	201	60,0
01.06.2011	27	46	152	30	42	151	60,0
01.06.2011	30	42	151	57	72	182	60,0
06.06.2011	14	87	198	52	147	212	60,0
17.05.2011	56	67	170	59	89	191	60,1
25.05.2011	14	87	198	55	126	201	60,1
01.06.2011	24	56	163	30	42	151	60,1
02.06.2011	15	59	166	22	24	124	60,1
26.05.2011	14	87	198	22	24	124	60,2
26.05.2011	22	24	124	23	21	117	60,2
24.06.2011	34	47	141	48	81	168	60,2
26.05.2011	15	59	166	22	24	124	60,3
01.06.2011	14	87	198	15	59	166	60,3
22.06.2011	46	118	192	48	81	168	60,3
28.06.2011	34	47	141	48	81	168	60,3
29.06.2011	34	47	141	48	81	168	60,3
25.05.2011	22	24	124	52	147	212	60,4
06.06.2011	16	67	169	23	21	117	60,4
25.05.2011	17	67	169	51	126	201	60,5
07.06.2011	19	58	166	58	71	182	60,5
27.05.2011	14	87	198	15	59	166	60,6
02.06.2011	16	67	169	51	141	207	60,6
17.05.2011	30	42	151	56	67	170	60,7
01.06.2011	16	67	169	51	141	207	60,7
25.05.2011	52	147	212	55	126	201	60,8
02.06.2011	23	21	117	53	152	218	60,8
06.06.2011	24	56	163	30	42	151	60,8
07.06.2011	23	21	117	51	141	207	60,9
07.06.2011	19	58	166	30	42	151	60,9
23.06.2011	20	65	158	34	47	141	60,9
26.05.2011	15	59	166	16	67	169	61,0
16.05.2011	56	67	170	60	119	217	61,1

01.06.2011	30	42	151	33	30	132	61,1
07.06.2011	22	24	124	51	141	207	61,1
25.05.2011	14	87	198	51	141	207	61,2
26.05.2011	16	67	169	51	141	207	61,2
24.06.2011	34	47	141	48	81	168	61,3
17.05.2011	19	58	166	59	89	191	61,5
18.05.2011	51	141	207	52	147	212	61,5
01.06.2011	51	141	207	53	152	218	61,5
17.05.2011	58	71	182	59	89	191	61,6
25.05.2011	16	67	169	55	126	201	61,6
02.06.2011	30	56	163	33	67	170	61,6
02.06.2011	14	87	198	52	147	212	61,6
03.06.2011	30	30	132	57	67	170	61,6
17.05.2011	14	87	198	54	199	238	61,7
07.06.2011	19	58	166	30	42	151	61,7
16.05.2011	58	71	182	60	119	217	61,8
25.05.2011	22	24	124	52	147	212	61,8
02.06.2011	15	59	166	52	147	212	61,8
03.06.2011	24	67	170	30	72	182	61,8
17.05.2011	15	59	166	17	34	137	61,9
01.06.2011	14	87	198	15	59	166	61,9
01.06.2011	30	42	151	56	67	170	61,9
02.06.2011	27	56	163	30	46	152	61,9
07.06.2011	16	67	169	51	141	207	61,9
22.06.2011	13	123	195	20	65	158	61,9
16.05.2011	13	48	159	58	71	182	62,0
16.05.2011	57	72	182	58	71	182	62,0
17.05.2011	30	42	151	58	71	182	62,0
17.05.2011	58	71	182	60	119	217	62,0
25.05.2011	14	87	198	52	147	212	62,0
01.06.2011	14	87	198	15	59	166	62,0
07.06.2011	22	24	124	51	141	207	62,0
16.05.2011	30	42	151	58	71	182	62,1

16.05.2011	33	30	132	58	71	182	62,1
16.05.2011	58	71	182	59	89	191	62,1
02.06.2011	22	24	124	53	152	218	62,2
07.06.2011	27	46	152	30	42	151	62,2
17.05.2011	15	59	166	16	67	169	62,3
06.06.2011	30	42	151	33	30	132	62,4
06.06.2011	57	72	182	58	71	182	62,4
06.06.2011	27	46	152	30	42	151	62,5
17.05.2011	17	34	137	54	199	238	62,6
17.05.2011	22	24	124	54	199	238	62,6
06.06.2011	30	42	151	57	72	182	62,6
25.05.2011	14	87	198	15	59	166	62,7
01.06.2011	27	46	152	30	42	151	62,7
26.05.2011	15	59	166	23	21	117	62,8
27.05.2011	16	67	169	52	147	212	62,8
07.06.2011	30	42	151	56	67	170	62,9
18.05.2011	22	24	124	54	199	238	63,0
03.06.2011	22	24	124	51	141	207	63,0
06.06.2011	16	67	169	23	21	117	63,0
07.06.2011	16	67	169	52	147	212	63,0
02.06.2011	14	87	198	52	147	212	63,2
25.05.2011	51	141	207	55	126	201	63,3
01.06.2011	14	87	198	52	147	212	63,3
06.06.2011	14	87	198	16	67	169	63,3
17.05.2011	16	67	169	54	199	238	63,4
26.05.2011	17	34	137	51	141	207	63,4
01.06.2011	14	87	198	22	24	124	63,4
18.05.2011	16	67	169	54	199	238	63,5
02.06.2011	30	42	151	57	72	182	63,6
03.06.2011	16	67	169	23	21	117	63,6
06.06.2011	16	67	169	22	24	124	63,6
15.06.2011	34	47	141	48	81	168	63,6
17.05.2011	24	56	163	58	71	182	63,7

17.05.2011	22	24	124	55	126	201	63,7
25.05.2011	14	87	198	52	147	212	63,7
02.06.2011	24	56	163	30	42	151	63,7
25.05.2011	14	87	198	15	59	166	63,8
06.06.2011	15	59	166	22	24	124	63,8
16.05.2011	13	48	159	58	71	182	63,9
17.05.2011	13	48	159	58	71	182	63,9
17.05.2011	22	24	124	23	21	117	63,9
16.05.2011	30	42	151	58	71	182	64,0
16.05.2011	57	72	182	58	71	182	64,0
17.05.2011	33	30	132	58	71	182	64,0
17.05.2011	15	59	166	55	126	201	64,0
18.05.2011	17	34	137	54	199	238	64,0
03.06.2011	23	21	117	51	141	207	64,1
06.06.2011	24	56	163	58	71	182	64,2
07.06.2011	16	67	169	51	141	207	64,2
26.05.2011	13	48	159	30	42	151	64,3
26.05.2011	13	48	159	57	72	182	64,3
26.05.2011	13	48	159	60	119	217	64,3
27.05.2011	17	34	137	51	141	207	64,4
03.06.2011	22	24	124	53	152	218	64,4
16.05.2011	33	30	132	59	89	191	64,5
25.05.2011	16	67	169	51	141	207	64,5
16.05.2011	27	46	152	58	71	182	64,6
16.05.2011	19	58	166	59	89	191	64,6
26.05.2011	17	34	137	52	147	212	64,6
03.06.2011	22	24	124	52	147	212	64,6
06.06.2011	15	59	166	22	24	124	64,6
16.05.2011	19	58	166	58	71	182	64,7
02.06.2011	16	67	169	51	141	207	64,7
02.06.2011	27	46	152	30	42	151	64,7
17.05.2011	15	59	166	52	147	212	64,8
01.06.2011	14	87	198	51	141	207	64,8

01.06.2011	15	59	166	52	147	212	64,8
02.06.2011	30	42	151	33	30	132	64,8
06.06.2011	16	67	169	53	152	218	64,8
06.06.2011	27	46	152	30	42	151	64,8
17.05.2011	15	59	166	54	199	238	64,9
06.06.2011	22	24	124	51	141	207	64,9
06.06.2011	27	46	152	30	42	151	64,9
17.05.2011	16	67	169	51	141	207	65,0
17.05.2011	16	67	169	52	147	212	65,0
27.05.2011	30	42	151	33	30	132	65,0
02.06.2011	52	147	212	53	152	218	65,0
06.06.2011	16	67	169	23	21	117	65,0
06.06.2011	14	87	198	16	67	169	65,0
07.06.2011	27	46	152	30	42	151	65,0
17.05.2011	57	72	182	58	71	182	65,1
17.05.2011	58	71	182	59	89	191	65,1
17.05.2011	58	71	182	60	119	217	65,1
02.06.2011	51	141	207	53	152	218	65,1
02.06.2011	16	67	169	22	24	124	65,1
17.05.2011	17	34	137	51	141	207	65,2
17.05.2011	56	67	170	58	71	182	65,2
18.05.2011	52	147	212	53	152	218	65,2
01.06.2011	14	87	198	22	24	124	65,2
01.06.2011	30	42	151	57	72	182	65,2
07.06.2011	19	58	166	30	42	151	65,2
17.05.2011	24	56	163	58	71	182	65,3
17.05.2011	27	46	152	58	71	182	65,3
26.05.2011	14	87	198	23	21	117	65,3
26.05.2011	15	59	166	51	141	207	65,3
06.06.2011	19	58	166	30	42	151	65,3
17.05.2011	19	58	166	58	71	182	65,4
25.05.2011	51	141	207	55	126	201	65,4
26.05.2011	17	34	137	51	141	207	65,4

02.06.2011	19	56	163	30	46	152	65,4
07.06.2011	23	21	117	51	141	207	65,4
06.06.2011	19	58	166	30	42	151	65,5
07.06.2011	15	59	166	51	141	207	65,6
17.05.2011	56	67	170	58	71	182	65,7
16.05.2011	14	87	198	51	141	207	65,9
26.05.2011	51	141	207	55	126	201	65,9
01.06.2011	15	59	166	52	147	212	65,9
02.06.2011	24	58	166	30	72	182	66,0
18.05.2011	15	59	166	52	147	212	66,1
27.05.2011	51	141	207	55	126	201	66,1
27.05.2011	51	141	207	53	152	218	66,1
26.05.2011	14	87	198	52	147	212	66,3
01.06.2011	52	147	212	53	152	218	66,3
02.06.2011	23	21	117	51	141	207	66,3
02.06.2011	27	46	152	30	30	132	66,3
03.06.2011	22	24	124	53	152	218	66,3
06.06.2011	30	42	151	33	30	132	66,3
07.06.2011	57	72	182	58	71	182	66,3
17.05.2011	22	24	124	52	147	212	66,4
01.06.2011	19	58	166	30	42	151	66,4
06.06.2011	30	42	151	56	67	170	66,4
07.06.2011	52	147	212	53	152	218	66,5
16.05.2011	19	58	166	58	71	182	66,6
16.05.2011	23	21	117	51	141	207	66,6
26.05.2011	22	24	124	55	126	201	66,7
16.05.2011	27	46	152	58	71	182	66,8
17.05.2011	15	59	166	52	147	212	66,8
07.06.2011	23	21	117	51	141	207	66,8
26.05.2011	23	21	117	51	141	207	66,9
27.05.2011	15	59	166	51	141	207	66,9
26.05.2011	17	34	137	22	24	124	67,0
06.06.2011	23	21	117	52	147	212	67,0

16.06.2011	20	65	158	34	47	141	67,0
16.05.2011	56	67	170	58	71	182	67,1
06.06.2011	19	58	166	30	42	151	67,1
17.05.2011	16	67	169	51	141	207	67,2
16.05.2011	24	56	163	58	71	182	67,3
16.05.2011	22	24	124	51	141	207	67,3
06.06.2011	22	24	124	51	141	207	67,3
16.05.2011	13	48	159	58	71	182	67,4
16.05.2011	33	30	132	58	71	182	67,4
16.05.2011	57	72	182	58	71	182	67,4
16.05.2011	58	71	182	59	89	191	67,4
16.05.2011	58	71	182	60	119	217	67,4
17.05.2011	17	34	137	51	141	207	67,4
25.05.2011	17	34	137	53	152	218	67,4
26.05.2011	14	87	198	51	141	207	67,4
07.06.2011	57	72	182	58	71	182	67,4
07.06.2011	52	147	212	53	152	218	67,4
28.06.2011	20	65	158	34	47	141	67,4
17.05.2011	17	34	137	54	199	238	67,5
02.06.2011	19	58	166	30	42	151	67,5
06.06.2011	30	42	151	56	67	170	67,5
16.05.2011	30	42	151	58	71	182	67,6
06.06.2011	22	24	124	51	141	207	67,6
06.06.2011	27	46	152	30	42	151	67,6
16.05.2011	17	34	137	51	141	207	67,8
17.05.2011	17	34	137	53	152	218	67,8
25.05.2011	16	67	169	55	126	201	67,8
27.05.2011	51	141	207	53	152	218	67,8
22.06.2011	13	123	195	20	65	158	67,8
16.05.2011	51	141	207	54	199	238	67,9
26.05.2011	17	34	137	51	141	207	67,9
02.06.2011	24	56	163	30	42	151	67,9
06.06.2011	22	24	124	53	152	218	67,9

06.06.2011	52	147	212	53	152	218	68,0
07.06.2011	30	42	151	33	30	132	68,0
17.05.2011	22	24	124	54	199	238	68,1
27.05.2011	52	147	212	53	152	218	68,1
06.06.2011	19	58	166	30	42	151	68,1
06.06.2011	19	58	166	30	42	151	68,3
27.05.2011	27	46	152	30	42	151	68,4
02.06.2011	22	24	124	53	152	218	68,4
06.06.2011	23	21	117	51	141	207	68,4
17.05.2011	14	87	198	15	59	166	68,5
26.05.2011	51	141	207	53	152	218	68,5
27.05.2011	19	58	166	30	42	151	68,5
27.05.2011	24	56	163	30	42	151	68,5
02.06.2011	30	58	166	33	67	170	68,5
17.05.2011	56	67	170	60	119	217	68,6
17.05.2011	17	34	137	51	141	207	68,6
01.06.2011	23	21	117	52	147	212	68,6
01.06.2011	27	46	152	30	42	151	68,6
24.06.2011	34	47	141	48	81	168	68,6
17.05.2011	17	34	137	22	24	124	68,7
02.06.2011	15	59	166	16	67	169	68,7
03.06.2011	22	24	124	52	147	212	68,7
07.06.2011	30	42	151	56	67	170	68,7
27.05.2011	30	42	151	60	119	217	68,8
06.06.2011	14	87	198	16	67	169	68,8
17.05.2011	30	42	151	58	71	182	68,9
17.05.2011	24	56	163	58	71	182	69,0
01.06.2011	22	24	124	52	147	212	69,0
02.06.2011	30	58	166	57	67	170	69,0
06.06.2011	19	58	166	30	42	151	69,0
06.06.2011	27	46	152	30	42	151	69,0
07.06.2011	30	42	151	56	67	170	69,0
17.05.2011	16	67	169	51	141	207	69,1

02.06.2011	15	59	166	16	67	169	69,1
17.05.2011	16	67	169	22	24	124	69,2
17.05.2011	17	34	137	54	199	238	69,2
01.06.2011	14	87	198	53	152	218	69,2
26.05.2011	13	48	159	58	71	182	69,3
16.05.2011	24	56	163	59	89	191	69,4
17.05.2011	17	34	137	55	126	201	69,4
17.05.2011	17	34	137	55	126	201	69,4
17.05.2011	23	21	117	55	126	201	69,4
17.05.2011	30	42	151	58	71	182	69,5
17.05.2011	33	30	132	59	89	191	69,6
02.06.2011	51	141	207	52	147	212	69,6
17.05.2011	16	67	169	54	199	238	69,7
17.05.2011	14	87	198	55	126	201	69,7
18.05.2011	22	24	124	52	147	212	69,7
25.05.2011	51	141	207	53	152	218	69,7
07.06.2011	14	87	198	52	147	212	69,7
17.05.2011	24	56	163	59	89	191	69,8
17.05.2011	16	67	169	55	126	201	69,8
18.05.2011	52	147	212	55	126	201	69,8
02.06.2011	51	141	207	52	147	212	69,8
02.06.2011	22	24	124	51	141	207	69,8
18.05.2011	52	147	212	54	199	238	69,9
25.05.2011	16	67	169	52	147	212	69,9
27.05.2011	15	59	166	51	141	207	69,9
06.06.2011	27	46	152	58	71	182	69,9
07.06.2011	30	42	151	33	30	132	69,9
16.05.2011	30	42	151	58	71	182	70,0
16.05.2011	33	30	132	58	71	182	70,0
01.06.2011	30	42	151	58	71	182	70,0
03.06.2011	19	30	132	30	67	170	70,0
15.06.2011	20	65	158	28	53	146	70,0
27.06.2011	28	53	138	46	118	192	70,0

16.05.2011	13	48	159	58	71	182	70,1
16.05.2011	19	58	166	58	71	182	70,1
16.05.2011	27	46	152	58	71	182	70,1
16.05.2011	57	72	182	58	71	182	70,1
16.05.2011	58	71	182	59	89	191	70,1
16.05.2011	15	59	166	52	147	212	70,1
16.05.2011	52	147	212	53	152	218	70,1
16.05.2011	17	34	137	55	126	201	70,1
26.05.2011	15	59	166	52	147	212	70,1
03.06.2011	19	30	132	30	67	170	70,1
25.05.2011	23	21	117	53	152	218	70,2
27.05.2011	15	59	166	52	147	212	70,2
16.06.2011	28	53	146	46	118	192	70,2
16.05.2011	58	71	182	60	119	217	70,3
02.06.2011	14	87	198	16	67	169	70,3
06.06.2011	16	67	169	53	152	218	70,3
17.05.2011	24	56	163	58	71	182	70,4
17.05.2011	17	34	137	55	126	201	70,5
17.05.2011	22	24	124	55	126	201	70,5
26.05.2011	51	141	207	55	126	201	70,5
16.05.2011	17	34	137	51	141	207	70,6
02.06.2011	24	46	152	30	42	151	70,6
18.05.2011	15	59	166	22	24	124	70,7
07.06.2011	15	59	166	52	147	212	70,7
16.05.2011	16	67	169	55	126	201	70,8
17.05.2011	58	71	182	59	89	191	70,8
25.05.2011	17	34	137	51	141	207	70,8
01.06.2011	19	58	166	30	42	151	70,8
02.06.2011	15	59	166	51	141	207	70,8
06.06.2011	22	24	124	51	141	207	70,8
16.05.2011	33	30	132	58	71	182	70,9
01.06.2011	30	42	151	58	71	182	70,9
16.05.2011	19	58	166	58	71	182	71,0

27.05.2011	14	87	198	51	141	207	71,0
06.06.2011	30	42	151	56	67	170	71,0
16.06.2011	35	50	138	46	118	192	71,0
18.05.2011	15	59	166	16	67	169	71,1
01.06.2011	30	42	151	57	72	182	71,1
06.06.2011	57	72	182	58	71	182	71,1
26.05.2011	16	67	169	52	147	212	71,2
17.05.2011	33	30	132	59	89	191	71,3
17.05.2011	56	67	170	59	89	191	71,3
17.05.2011	17	34	137	23	21	117	71,3
26.05.2011	51	141	207	52	147	212	71,3
01.06.2011	30	42	151	33	30	132	71,3
17.05.2011	16	67	169	55	126	201	71,4
07.06.2011	52	147	212	53	152	218	71,4
07.06.2011	24	56	163	58	71	182	71,4
16.05.2011	16	67	169	51	141	207	71,5
26.05.2011	16	67	169	51	141	207	71,5
01.06.2011	27	46	152	30	42	151	71,5
01.06.2011	30	42	151	33	30	132	71,5
01.06.2011	30	42	151	56	67	170	71,5
01.06.2011	19	58	166	30	42	151	71,5
06.06.2011	23	21	117	52	147	212	71,5
18.05.2011	15	59	166	17	34	137	71,6
02.06.2011	30	56	163	56	67	170	71,6
28.06.2011	34	47	141	48	81	168	71,6
17.05.2011	19	58	166	59	89	191	71,7
25.05.2011	15	59	166	52	147	212	71,7
01.06.2011	30	42	151	58	71	182	71,7
07.06.2011	23	21	117	52	147	212	71,7
17.05.2011	16	67	169	23	21	117	71,8
25.05.2011	23	21	117	52	147	212	71,8
26.05.2011	51	141	207	55	126	201	71,8
27.05.2011	22	24	124	52	147	212	71,8

01.06.2011	15	59	166	52	147	212	71,8
17.05.2011	17	34	137	52	147	212	71,9
26.05.2011	15	59	166	51	141	207	71,9
26.05.2011	23	21	117	51	141	207	71,9
01.06.2011	14	87	198	22	24	124	71,9
07.06.2011	14	87	198	52	147	212	71,9
07.06.2011	24	56	163	58	71	182	71,9
17.05.2011	23	21	117	52	147	212	72,0
26.05.2011	14	87	198	51	141	207	72,0
26.05.2011	52	147	212	53	152	218	72,0
01.06.2011	27	46	152	30	42	151	72,0
06.06.2011	57	72	182	58	71	182	72,0
26.05.2011	16	67	169	52	147	212	72,1
27.05.2011	23	21	117	51	141	207	72,1
02.06.2011	14	87	198	51	141	207	72,1
25.05.2011	23	21	117	52	147	212	72,2
17.06.2011	20	65	158	34	47	141	72,2
26.05.2011	15	59	166	22	24	124	72,3
01.06.2011	27	46	152	30	42	151	72,3
02.06.2011	16	67	169	53	152	218	72,3
06.06.2011	16	67	169	51	141	207	72,3
17.05.2011	30	42	151	58	71	182	72,4
17.05.2011	33	30	132	58	71	182	72,4
25.05.2011	22	24	124	52	147	212	72,4
07.06.2011	24	56	163	58	71	182	72,4
17.05.2011	27	46	152	58	71	182	72,5
06.06.2011	24	56	163	58	71	182	72,6
15.06.2011	20	65	158	35	50	138	72,6
01.06.2011	14	87	198	16	67	169	72,7
01.06.2011	30	42	151	56	67	170	72,8
02.06.2011	19	56	163	30	46	152	72,8
17.05.2011	13	48	159	58	71	182	72,9
07.06.2011	15	59	166	52	147	212	72,9

07.06.2011	24	56	163	58	71	182	72,9
17.05.2011	15	59	166	55	126	201	73,0
26.05.2011	13	48	159	33	30	132	73,1
06.06.2011	14	87	198	52	147	212	73,1
17.05.2011	16	67	169	55	126	201	73,2
07.06.2011	24	56	163	58	71	182	73,2
17.05.2011	17	34	137	51	141	207	73,3
17.05.2011	17	34	137	52	147	212	73,3
26.05.2011	13	48	159	19	58	166	73,3
26.05.2011	13	48	159	24	56	163	73,3
27.05.2011	51	141	207	55	126	201	73,3
06.06.2011	30	42	151	33	30	132	73,3
26.05.2011	13	48	159	56	67	170	73,4
27.05.2011	17	34	137	51	141	207	73,4
27.05.2011	22	24	124	51	141	207	73,4
27.05.2011	15	59	166	52	147	212	73,4
07.06.2011	16	67	169	52	147	212	73,4
07.06.2011	52	147	212	53	152	218	73,4
26.05.2011	52	147	212	53	152	218	73,5
26.05.2011	13	48	159	27	46	152	73,5
07.06.2011	22	24	124	51	141	207	73,5
07.06.2011	23	21	117	52	147	212	73,5
06.06.2011	57	72	182	58	71	182	73,6
17.05.2011	14	87	198	55	126	201	73,7
02.06.2011	14	87	198	51	141	207	73,7
06.06.2011	57	72	182	58	71	182	73,7
17.05.2011	58	71	182	60	119	217	73,8
25.05.2011	51	141	207	55	126	201	73,8
07.06.2011	22	24	124	52	147	212	73,9
17.05.2011	52	147	212	54	199	238	74,0
26.05.2011	15	59	166	53	152	218	74,0
26.05.2011	15	59	166	55	126	201	74,0
27.05.2011	52	147	212	53	152	218	74,1

01.06.2011	24	56	163	30	42	151	74,1
07.06.2011	57	72	182	58	71	182	74,3
07.06.2011	56	67	170	58	71	182	74,4
25.05.2011	51	141	207	55	126	201	74,5
06.06.2011	24	56	163	58	71	182	74,5
17.05.2011	16	67	169	52	147	212	74,6
26.05.2011	15	59	166	17	34	137	74,6
17.05.2011	17	34	137	52	147	212	74,7
25.05.2011	15	59	166	23	21	117	74,7
25.05.2011	23	21	117	52	147	212	74,7
01.06.2011	30	42	151	57	72	182	74,7
02.06.2011	22	24	124	52	147	212	74,7
06.06.2011	52	147	212	53	152	218	74,7
07.06.2011	30	42	151	56	67	170	74,8
26.05.2011	16	67	169	52	147	212	74,9
17.05.2011	52	147	212	54	199	238	75,0
17.05.2011	51	141	207	52	147	212	75,0
16.05.2011	24	56	163	58	71	182	75,1
17.05.2011	17	34	137	52	147	212	75,1
17.05.2011	51	141	207	52	147	212	75,1
17.05.2011	14	87	198	51	141	207	75,1
01.06.2011	23	21	117	55	126	201	75,1
17.05.2011	14	87	198	22	24	124	75,2
26.05.2011	14	87	198	52	147	212	75,2
26.05.2011	51	141	207	52	147	212	75,2
26.05.2011	14	87	198	52	147	212	75,3
06.06.2011	14	87	198	52	147	212	75,5
06.06.2011	19	58	166	58	71	182	75,6
16.05.2011	24	56	163	58	71	182	75,7
17.05.2011	14	87	198	54	199	238	75,7
17.05.2011	16	67	169	52	147	212	75,7
07.06.2011	30	42	151	56	67	170	75,7
07.06.2011	22	24	124	52	147	212	75,8

28.06.2011	13	123	195	34	47	141	75,8
17.05.2011	14	87	198	55	126	201	75,9
02.06.2011	23	21	117	51	141	207	75,9
25.05.2011	15	59	166	52	147	212	76,0
25.05.2011	52	147	212	53	152	218	76,1
17.05.2011	14	87	198	52	147	212	76,2
25.05.2011	17	34	137	55	126	201	76,2
25.05.2011	15	59	166	53	152	218	76,2
27.05.2011	15	59	166	17	34	137	76,2
07.06.2011	16	67	169	52	147	212	76,2
16.05.2011	16	67	169	51	141	207	76,3
16.05.2011	30	42	151	58	71	182	76,3
07.06.2011	19	58	166	58	71	182	76,4
02.06.2011	30	56	163	58	67	170	76,6
07.06.2011	30	42	151	33	30	132	76,8
01.06.2011	30	42	151	33	30	132	76,9
26.05.2011	51	141	207	55	126	201	77,0
02.06.2011	16	67	169	51	141	207	77,1
06.06.2011	16	67	169	22	24	124	77,1
07.06.2011	16	67	169	52	147	212	77,1
16.05.2011	13	48	159	58	71	182	77,2
01.06.2011	30	42	151	56	67	170	77,2
01.06.2011	30	42	151	57	72	182	77,2
06.06.2011	30	42	151	56	67	170	77,2
06.06.2011	24	56	163	58	71	182	77,3
07.06.2011	33	30	132	58	71	182	77,3
27.05.2011	14	87	198	52	147	212	77,6
07.06.2011	27	46	152	58	71	182	77,6
16.05.2011	58	71	182	59	89	191	77,8
17.05.2011	22	24	124	53	152	218	77,8
17.05.2011	14	87	198	23	21	117	77,8
06.06.2011	30	42	151	56	67	170	77,8
07.06.2011	23	21	117	52	147	212	77,8

27.06.2011	35	50	138	46	118	192	77,8
18.05.2011	23	21	117	52	147	212	77,9
26.05.2011	51	141	207	53	152	218	77,9
26.05.2011	15	59	166	17	34	137	78,0
16.05.2011	58	71	182	60	119	217	78,1
17.05.2011	13	48	159	58	71	182	78,1
17.05.2011	27	46	152	58	71	182	78,1
18.05.2011	15	59	166	52	147	212	78,1
18.05.2011	16	67	169	52	147	212	78,1
18.05.2011	17	34	137	52	147	212	78,1
27.05.2011	15	59	166	55	126	201	78,1
07.06.2011	57	72	182	58	71	182	78,1
06.06.2011	51	141	207	52	147	212	78,3
17.05.2011	27	46	152	58	71	182	78,5
17.05.2011	23	21	117	54	199	238	78,5
26.05.2011	51	141	207	55	126	201	78,5
02.06.2011	33	58	166	58	67	170	78,5
07.06.2011	30	42	151	33	30	132	78,5
17.05.2011	17	34	137	53	152	218	78,6
26.05.2011	22	24	124	51	141	207	78,6
07.06.2011	22	24	124	52	147	212	78,6
16.05.2011	17	34	137	52	147	212	78,7
17.05.2011	57	72	182	58	71	182	78,7
27.05.2011	23	21	117	52	147	212	78,7
01.06.2011	27	46	152	56	67	170	78,7
06.06.2011	23	21	117	52	147	212	78,7
15.06.2011	20	65	158	46	118	192	78,9
17.05.2011	16	67	169	53	152	218	79,1
25.05.2011	23	21	117	55	126	201	79,1
25.05.2011	23	21	117	52	147	212	79,1
22.06.2011	20	65	158	46	118	192	79,1
17.05.2011	30	42	151	58	71	182	79,2
17.05.2011	16	67	169	52	147	212	79,2

07.06.2011	52	147	212	53	152	218	79,2
07.06.2011	19	58	166	58	71	182	79,2
02.06.2011	27	56	163	58	30	132	79,3
01.06.2011	33	30	132	58	71	182	79,4
06.06.2011	57	72	182	58	71	182	79,4
07.06.2011	22	24	124	52	147	212	79,5
16.05.2011	16	67	169	52	147	212	79,6
27.05.2011	23	21	117	51	141	207	79,6
02.06.2011	33	56	163	57	72	182	79,6
06.06.2011	16	67	169	22	24	124	79,6
06.06.2011	33	30	132	58	71	182	79,6
07.06.2011	27	46	152	58	71	182	79,6
22.06.2011	13	123	195	46	118	192	79,6
25.05.2011	16	67	169	52	147	212	79,7
27.05.2011	14	87	198	51	141	207	79,7
17.05.2011	16	67	169	52	147	212	79,8
27.05.2011	17	34	137	51	141	207	79,8
03.06.2011	22	24	124	52	147	212	79,8
27.05.2011	14	87	198	52	147	212	79,9
27.05.2011	23	21	117	52	147	212	79,9
27.05.2011	51	141	207	55	126	201	79,9
27.05.2011	52	147	212	55	126	201	79,9
15.06.2011	20	65	158	28	53	146	79,9
16.06.2011	28	53	146	46	118	192	79,9
25.05.2011	17	67	169	52	126	201	80,0
27.05.2011	17	34	137	52	147	212	80,0
02.06.2011	27	56	163	57	42	151	80,0
27.05.2011	52	147	212	55	126	201	80,1
27.05.2011	17	34	137	52	147	212	80,1
27.05.2011	22	24	124	52	147	212	80,1
01.06.2011	24	56	163	30	42	151	80,1
06.06.2011	14	87	198	52	147	212	80,1
07.06.2011	23	21	117	52	147	212	80,1

17.05.2011	56	67	170	58	71	182	80,2
26.05.2011	15	59	166	55	126	201	80,2
01.06.2011	24	56	163	56	67	170	80,2
01.06.2011	33	30	132	56	67	170	80,2
01.06.2011	56	67	170	57	72	182	80,2
17.05.2011	51	141	207	52	147	212	80,3
01.06.2011	27	46	152	58	71	182	80,3
15.06.2011	20	65	158	46	118	192	80,3
02.06.2011	22	24	124	52	147	212	80,4
07.06.2011	30	42	151	33	30	132	80,4
07.06.2011	30	42	151	56	67	170	80,4
22.06.2011	20	65	158	46	118	192	80,4
27.05.2011	16	67	169	52	147	212	80,6
27.05.2011	57	72	182	58	71	182	80,6
16.06.2011	35	50	138	46	118	192	80,6
17.05.2011	52	147	212	53	152	218	80,7
26.05.2011	23	21	117	52	147	212	80,7
27.05.2011	13	48	159	58	71	182	80,7
27.05.2011	56	67	170	58	71	182	80,7
03.06.2011	19	30	132	30	67	170	80,8
06.06.2011	24	56	163	58	71	182	80,9
06.06.2011	27	46	152	58	71	182	81,0
02.06.2011	56	56	163	58	67	170	81,2
06.06.2011	22	24	124	52	147	212	81,2
06.06.2011	16	67	169	23	21	117	81,4
15.06.2011	20	65	158	46	118	192	81,5
01.06.2011	19	58	166	30	42	151	81,6
06.06.2011	19	58	166	58	71	182	81,6
26.05.2011	14	87	198	15	59	166	81,7
06.06.2011	24	56	163	58	71	182	81,7
02.06.2011	23	21	117	51	141	207	81,8
07.06.2011	19	58	166	58	71	182	81,8
17.05.2011	24	56	163	58	71	182	82,0

03.06.2011	27	42	151	58	67	170	82,0
06.06.2011	14	87	198	52	147	212	82,0
26.05.2011	17	34	137	51	141	207	82,1
07.06.2011	27	46	152	58	71	182	82,1
22.06.2011	20	65	158	46	118	192	82,1
02.06.2011	19	58	166	30	42	151	82,2
07.06.2011	27	46	152	58	71	182	82,2
16.05.2011	14	87	198	52	147	212	82,3
02.06.2011	19	58	166	30	42	151	82,3
06.06.2011	51	141	207	52	147	212	82,3
07.06.2011	19	58	166	58	71	182	82,3
16.05.2011	27	46	152	58	71	182	82,4
25.05.2011	16	67	169	52	147	212	82,4
25.05.2011	24	56	163	58	71	182	82,4
25.05.2011	57	72	182	58	71	182	82,4
16.06.2011	20	65	158	28	53	146	82,4
25.05.2011	13	48	159	58	71	182	82,6
02.06.2011	22	24	124	52	147	212	82,6
17.05.2011	52	147	212	53	152	218	82,7
26.05.2011	15	59	166	52	147	212	82,7
01.06.2011	22	24	124	52	147	212	82,7
06.06.2011	22	24	124	52	147	212	82,8
15.06.2011	20	65	158	35	50	138	82,8
26.05.2011	52	147	212	53	152	218	82,9
22.06.2011	20	65	158	34	47	141	82,9
25.05.2011	17	34	137	52	147	212	83,0
16.05.2011	23	21	117	52	147	212	83,1
25.05.2011	15	59	166	17	34	137	83,1
17.05.2011	13	48	159	58	71	182	83,2
01.06.2011	16	67	169	52	147	212	83,2
16.05.2011	24	56	163	58	71	182	83,3
26.05.2011	22	24	124	52	147	212	83,3
02.06.2011	27	46	152	58	71	182	83,3

06.06.2011	16	67	169	22	24	124	83,3
16.06.2011	20	65	158	35	50	138	83,3
02.06.2011	24	56	163	58	71	182	83,4
17.05.2011	22	24	124	51	141	207	83,5
17.05.2011	22	24	124	52	147	212	83,5
17.05.2011	30	42	151	58	71	182	83,5
17.05.2011	33	30	132	58	71	182	83,6
26.05.2011	15	59	166	52	147	212	83,8
26.05.2011	22	24	124	52	147	212	83,9
06.06.2011	52	147	212	53	152	218	83,9
15.06.2011	20	65	158	28	53	146	83,9
25.05.2011	52	147	212	55	126	201	84,0
02.06.2011	24	58	166	57	72	182	84,0
26.05.2011	17	34	137	52	147	212	84,1
02.06.2011	15	59	166	51	141	207	84,2
15.06.2011	20	65	158	35	50	138	84,2
16.05.2011	22	24	124	52	147	212	84,3
26.05.2011	22	24	124	52	147	212	84,3
26.05.2011	17	34	137	52	147	212	84,3
03.06.2011	57	132	326	58	71	182	84,5
03.06.2011	24	30	132	58	71	182	84,6
25.05.2011	52	147	212	53	152	218	84,7
26.05.2011	52	147	212	53	152	218	84,7
17.06.2011	28	53	146	46	118	192	84,7
16.05.2011	17	34	137	52	147	212	84,8
16.05.2011	52	147	212	54	199	238	84,8
16.05.2011	51	141	207	52	147	212	84,9
26.05.2011	51	141	207	52	147	212	84,9
07.06.2011	19	58	166	58	71	182	84,9
17.05.2011	14	87	198	53	152	218	85,0
25.05.2011	58	71	182	60	119	217	85,0
02.06.2011	16	67	169	23	21	117	85,0
27.05.2011	13	48	159	58	71	182	85,1

06.06.2011	23	21	117	52	147	212	85,1
16.05.2011	15	59	166	17	34	137	85,2
01.06.2011	57	72	182	58	71	182	85,3
03.06.2011	57	72	182	58	71	182	85,3
25.05.2011	17	34	137	52	147	212	85,4
25.05.2011	56	67	170	58	71	182	85,4
26.05.2011	17	34	137	51	141	207	85,4
01.06.2011	56	67	170	58	71	182	85,4
01.06.2011	56	67	170	58	71	182	85,4
03.06.2011	24	42	151	58	71	182	85,4
01.06.2011	27	46	152	58	71	182	85,5
01.06.2011	33	30	132	58	71	182	85,5
26.05.2011	52	147	212	53	152	218	85,6
02.06.2011	19	58	166	57	42	151	85,6
26.05.2011	17	34	137	52	147	212	85,7
02.06.2011	27	46	152	58	30	132	85,7
06.06.2011	23	21	117	52	147	212	85,7
17.05.2011	13	48	159	58	71	182	85,9
03.06.2011	30	30	132	33	67	170	85,9
27.05.2011	23	21	117	52	147	212	86,0
15.06.2011	20	65	158	35	50	138	86,0
16.05.2011	15	59	166	16	67	169	86,1
06.06.2011	30	42	151	33	30	132	86,1
17.05.2011	23	21	117	55	126	201	86,2
06.06.2011	27	46	152	58	71	182	86,2
17.05.2011	57	72	182	59	89	191	86,3
07.06.2011	23	21	117	52	147	212	86,3
17.05.2011	19	58	166	59	89	191	86,4
26.05.2011	14	87	198	51	141	207	86,4
02.06.2011	15	59	166	52	147	212	86,4
23.06.2011	34	47	141	48	81	168	86,4
26.05.2011	52	147	212	55	126	201	86,6
07.06.2011	15	59	166	52	147	212	86,6

27.05.2011	56	67	170	58	71	182	86,7
06.06.2011	33	30	132	58	71	182	86,7
02.06.2011	57	72	182	58	71	182	86,8
27.05.2011	52	147	212	53	152	218	86,9
17.05.2011	13	48	159	58	71	182	87,1
26.05.2011	17	34	137	52	147	212	87,1
15.06.2011	20	65	158	46	118	192	87,2
27.05.2011	30	42	151	58	71	182	87,3
06.06.2011	30	42	151	56	67	170	87,4
03.06.2011	27	82	206	58	71	182	87,5
06.06.2011	22	24	124	52	147	212	87,5
06.06.2011	33	30	132	58	71	182	87,5
07.06.2011	27	46	152	58	71	182	87,5
02.06.2011	24	46	152	58	42	151	87,8
01.06.2011	15	59	166	52	147	212	87,9
01.06.2011	30	42	151	33	30	132	88,0
02.06.2011	33	46	152	58	67	170	88,0
06.06.2011	27	46	152	58	71	182	88,0
01.06.2011	24	56	163	58	71	182	88,1
01.06.2011	24	56	163	58	71	182	88,3
25.05.2011	52	147	212	55	126	201	88,4
26.05.2011	17	34	137	52	147	212	88,5
01.06.2011	57	72	182	58	71	182	88,7
02.06.2011	57	56	163	58	72	182	88,7
22.06.2011	20	65	158	34	47	141	88,7
16.05.2011	24	56	163	58	71	182	88,8
25.05.2011	52	147	212	55	126	201	88,8
25.05.2011	51	141	207	53	152	218	88,8
25.05.2011	15	59	166	55	126	201	88,9
02.06.2011	30	42	151	56	67	170	89,0
17.05.2011	33	30	132	58	71	182	89,1
26.05.2011	15	59	166	51	141	207	89,1
06.06.2011	19	58	166	58	71	182	89,1

16.05.2011	33	30	132	58	71	182	89,2
17.05.2011	24	56	163	58	71	182	89,3
02.06.2011	30	42	151	33	30	132	89,3
01.06.2011	22	24	124	52	147	212	89,5
06.06.2011	33	30	132	58	71	182	89,5
25.05.2011	27	46	152	58	71	182	89,6
27.05.2011	14	87	198	52	147	212	89,7
02.06.2011	22	24	124	51	141	207	89,7
07.06.2011	33	30	132	58	71	182	89,7
28.06.2011	13	123	195	34	47	141	89,8
22.06.2011	34	47	141	48	81	168	89,9
25.05.2011	17	34	137	52	147	212	90,0
02.06.2011	24	58	166	58	30	132	90,0
06.06.2011	33	30	132	58	71	182	90,0
16.05.2011	56	67	170	58	71	182	90,4
16.05.2011	52	147	212	54	199	238	90,5
26.05.2011	52	147	212	55	126	201	90,6
03.06.2011	24	67	170	58	71	182	90,6
02.06.2011	19	58	166	30	42	151	90,7
07.06.2011	56	67	170	58	71	182	90,7
15.06.2011	20	65	158	35	50	138	90,8
23.06.2011	46	118	192	48	81	168	90,8
15.06.2011	20	65	158	28	53	146	90,9
03.06.2011	27	30	132	58	67	170	91,0
07.06.2011	33	30	132	58	71	182	91,0
02.06.2011	56	58	166	58	72	182	91,1
03.06.2011	56	67	170	58	71	182	91,1
15.06.2011	20	65	158	28	53	146	91,1
26.05.2011	23	21	117	52	147	212	91,3
18.05.2011	14	87	198	52	147	212	91,4
25.05.2011	22	24	124	51	141	207	91,5
28.06.2011	28	53	138	46	118	192	91,5
26.05.2011	52	147	212	53	152	218	91,6

26.05.2011	52	147	212	55	126	201	91,6
07.06.2011	56	67	170	58	71	182	91,6
01.06.2011	19	58	166	56	67	170	91,7
17.05.2011	14	87	198	52	147	212	91,8
15.06.2011	20	65	158	35	50	138	91,8
26.05.2011	52	147	212	55	126	201	91,9
26.05.2011	16	67	169	52	147	212	92,0
23.06.2011	46	118	192	48	81	168	92,0
26.05.2011	52	147	212	55	126	201	92,1
02.06.2011	19	58	166	58	42	151	92,1
25.05.2011	30	42	151	58	71	182	92,2
26.05.2011	14	87	198	52	147	212	92,2
26.05.2011	17	34	137	52	147	212	92,2
26.05.2011	15	59	166	52	147	212	92,3
02.06.2011	56	67	170	58	71	182	92,3
26.05.2011	23	21	117	52	147	212	92,4
26.05.2011	52	147	212	55	126	201	92,4
01.06.2011	19	58	166	30	42	151	92,4
25.05.2011	33	30	132	58	71	182	92,5
26.05.2011	14	87	198	52	147	212	92,5
15.06.2011	20	65	158	28	53	146	92,5
17.05.2011	17	34	137	52	147	212	92,6
17.05.2011	22	24	124	52	147	212	92,6
16.05.2011	16	67	169	52	147	212	92,7
17.05.2011	15	59	166	52	147	212	92,7
07.06.2011	33	30	132	58	71	182	92,7
22.06.2011	20	65	158	34	47	141	92,7
27.06.2011	13	123	195	34	47	141	92,8
25.05.2011	23	21	117	52	147	212	93,0
06.06.2011	33	30	132	58	71	182	93,1
27.05.2011	57	72	182	58	71	182	93,6
02.06.2011	30	42	151	33	30	132	93,7
07.06.2011	22	24	124	52	147	212	93,7

17.05.2011	16	67	169	52	147	212	93,8
06.06.2011	19	58	166	58	71	182	93,8
06.06.2011	56	67	170	58	71	182	93,8
01.06.2011	15	59	166	55	126	201	93,9
01.06.2011	23	21	117	51	141	207	93,9
02.06.2011	30	42	151	58	71	182	94,0
07.06.2011	56	67	170	58	71	182	94,0
03.06.2011	30	97	242	33	71	182	94,1
06.06.2011	56	67	170	58	71	182	94,2
01.06.2011	52	147	212	53	152	218	94,3
06.06.2011	27	46	152	58	71	182	94,3
02.06.2011	30	42	151	56	67	170	94,4
06.06.2011	56	67	170	58	71	182	94,5
16.05.2011	56	67	170	58	71	182	94,6
25.05.2011	17	34	137	51	141	207	94,6
07.06.2011	33	30	132	58	71	182	94,8
02.06.2011	22	24	124	51	141	207	94,9
03.06.2011	30	30	132	33	72	182	94,9
27.05.2011	33	30	132	58	71	182	95,3
07.06.2011	56	67	170	58	71	182	95,3
17.05.2011	17	34	137	52	147	212	95,4
17.06.2011	35	50	138	46	118	192	95,4
01.06.2011	22	24	124	55	126	201	95,5
17.05.2011	15	59	166	52	147	212	95,7
02.06.2011	19	56	163	58	46	152	95,7
17.05.2011	16	67	169	52	147	212	95,8
01.06.2011	19	58	166	30	42	151	96,1
01.06.2011	51	141	207	52	147	212	96,1
06.06.2011	19	58	166	58	71	182	96,4
06.06.2011	27	46	152	58	71	182	96,4
06.06.2011	22	24	124	52	147	212	97,0
16.05.2011	57	72	182	58	71	182	97,2
17.05.2011	56	67	170	59	89	191	97,2

07.06.2011	30	42	151	57	72	182	97,2
07.06.2011	33	30	132	58	71	182	97,2
07.06.2011	56	67	170	58	71	182	97,3
27.05.2011	57	72	182	58	71	182	97,6
07.06.2011	57	72	182	58	71	182	97,6
27.05.2011	13	48	159	58	71	182	97,7
27.05.2011	27	46	152	58	71	182	97,7
27.05.2011	30	42	151	58	71	182	97,7
27.05.2011	33	30	132	58	71	182	97,7
06.06.2011	56	67	170	58	71	182	98,2
25.05.2011	19	58	166	58	71	182	98,5
06.06.2011	56	67	170	58	71	182	98,5
06.06.2011	19	58	166	58	71	182	98,5
17.05.2011	19	58	166	58	71	182	98,7
27.05.2011	27	46	152	58	71	182	98,7
27.05.2011	24	56	163	58	71	182	98,8
07.06.2011	30	42	151	57	72	182	98,8
27.05.2011	19	58	166	58	71	182	98,9
02.06.2011	22	24	124	51	141	207	98,9
27.05.2011	58	71	182	60	119	217	99,1
27.05.2011	58	71	182	60	119	217	99,1
06.06.2011	56	67	170	58	71	182	99,6
24.06.2011	46	118	192	48	81	168	99,6
16.05.2011	56	67	170	59	89	191	99,7
01.06.2011	53	152	218	55	126	201	100,0
03.06.2011	19	42	151	58	72	182	100,0
01.06.2011	19	58	166	58	71	182	100,8
17.05.2011	19	58	166	58	71	182	101,0
02.06.2011	23	21	117	52	147	212	101,3
17.05.2011	14	87	198	52	147	212	101,6
23.06.2011	46	118	192	48	81	168	101,7
28.06.2011	35	50	138	46	118	192	101,8
17.05.2011	19	58	166	58	71	182	102,2

26.05.2011	23	21	117	52	147	212	102,2
23.06.2011	20	65	158	46	118	192	102,2
17.05.2011	22	24	124	52	147	212	103,0
24.06.2011	46	118	192	48	81	168	103,0
02.06.2011	57	72	182	58	71	182	103,4
01.06.2011	16	67	169	51	141	207	103,5
02.06.2011	19	58	166	58	71	182	103,7
27.05.2011	17	34	137	52	147	212	104,2
23.06.2011	46	118	192	48	81	168	104,2
03.06.2011	19	42	151	58	72	182	104,3
25.05.2011	15	59	166	51	141	207	104,8
03.06.2011	30	102	254	56	71	182	104,8
23.06.2011	46	118	192	48	81	168	104,8
28.06.2011	13	123	195	46	118	192	105,0
17.05.2011	19	58	166	58	71	182	105,4
16.05.2011	56	67	170	58	71	182	105,6
25.05.2011	23	21	117	51	141	207	105,6
03.06.2011	19	67	170	58	72	182	105,6
01.06.2011	24	56	163	58	71	182	105,7
01.06.2011	57	72	182	58	71	182	105,7
17.05.2011	57	72	182	58	71	182	105,9
17.05.2011	19	58	166	58	71	182	106,0
25.05.2011	16	67	169	51	141	207	106,0
24.06.2011	20	65	158	46	118	192	106,0
27.06.2011	13	123	195	46	118	192	106,0
16.05.2011	51	141	207	52	147	212	106,1
27.05.2011	52	147	212	55	126	201	106,1
16.05.2011	15	59	166	52	147	212	106,2
16.05.2011	52	147	212	53	152	218	106,2
16.05.2011	52	147	212	54	199	238	106,2
16.05.2011	52	147	212	55	126	201	106,2
16.06.2011	20	65	158	46	118	192	106,3
16.05.2011	17	34	137	52	147	212	106,4

16.05.2011	22	24	124	52	147	212	106,4
16.05.2011	23	21	117	52	147	212	106,4
23.06.2011	46	118	192	48	81	168	106,4
16.05.2011	16	67	169	52	147	212	106,5
17.05.2011	23	21	117	52	147	212	106,9
24.06.2011	46	118	192	48	81	168	107,2
29.06.2011	46	118	192	48	81	168	107,2
17.05.2011	33	30	132	58	71	182	107,4
17.05.2011	15	59	166	52	147	212	107,5
17.05.2011	17	34	137	52	147	212	107,5
16.05.2011	14	87	198	52	147	212	107,6
17.05.2011	14	87	198	52	147	212	107,6
01.06.2011	56	67	170	58	71	182	107,6
03.06.2011	33	67	170	58	71	182	107,7
17.05.2011	52	147	212	53	152	218	107,8
22.06.2011	46	118	192	48	81	168	107,8
16.05.2011	19	58	166	58	71	182	107,9
16.05.2011	22	24	124	52	147	212	108,1
16.05.2011	23	21	117	52	147	212	108,1
18.05.2011	27	46	152	58	71	182	108,1
18.05.2011	56	67	170	58	71	182	108,1
18.05.2011	58	71	182	59	89	191	108,1
16.05.2011	14	87	198	52	147	212	108,2
16.05.2011	15	59	166	52	147	212	108,2
16.05.2011	16	67	169	52	147	212	108,2
16.05.2011	17	34	137	52	147	212	108,2
16.05.2011	52	147	212	55	126	201	108,2
17.05.2011	14	87	198	52	147	212	108,2
18.05.2011	30	42	151	58	71	182	108,2
18.05.2011	57	72	182	58	71	182	108,2
16.05.2011	52	147	212	53	152	218	108,3
18.05.2011	58	71	182	60	119	217	108,3
27.05.2011	19	58	166	58	71	182	108,3

26.05.2011	13	48	159	58	71	182	108,4
26.05.2011	57	72	182	58	71	182	108,4
26.05.2011	58	71	182	60	119	217	108,4
27.05.2011	24	56	163	58	71	182	108,4
27.05.2011	33	30	132	58	71	182	108,4
17.05.2011	17	34	137	52	147	212	108,5
17.05.2011	23	21	117	52	147	212	108,6
17.05.2011	52	147	212	55	126	201	108,6
27.05.2011	27	46	152	58	71	182	108,6
28.06.2011	46	118	192	48	81	168	108,6
17.05.2011	22	24	124	52	147	212	108,8
23.06.2011	46	118	192	48	81	168	110,0
02.06.2011	27	46	152	58	71	182	110,2
01.06.2011	19	58	166	58	71	182	110,6
02.06.2011	24	56	163	58	71	182	110,6
02.06.2011	57	72	182	58	71	182	110,7
02.06.2011	56	46	152	58	42	151	110,8
27.06.2011	46	118	192	48	81	168	110,9
28.06.2011	46	118	192	48	81	168	110,9
27.06.2011	46	118	192	48	81	168	111,0
16.05.2011	52	147	212	54	199	238	111,1
27.05.2011	58	71	182	60	119	217	111,1
02.06.2011	56	67	170	58	71	182	111,5
23.06.2011	20	65	158	46	118	192	111,5
17.06.2011	20	65	158	46	118	192	111,7
01.06.2011	19	58	166	58	71	182	112,2
28.06.2011	46	118	192	48	81	168	112,2
22.06.2011	20	65	158	46	118	192	112,4
26.05.2011	30	42	151	58	71	182	112,5
22.06.2011	46	118	192	48	81	168	112,5
16.05.2011	56	67	170	58	71	182	112,6
17.05.2011	33	30	132	58	71	182	112,6
17.05.2011	56	67	170	58	71	182	112,6

27.06.2011	13	123	195	46	118	192	112,8
16.05.2011	52	147	212	55	126	201	113,0
16.06.2011	20	65	158	46	118	192	113,0
27.06.2011	46	118	192	48	81	168	113,0
16.06.2011	46	118	192	48	81	168	113,1
27.06.2011	20	65	158	46	118	192	113,1
16.06.2011	13	123	195	46	118	192	113,2
28.06.2011	20	65	158	46	118	192	113,2
16.05.2011	15	59	166	52	147	212	113,3
16.05.2011	17	34	137	52	147	212	113,3
27.06.2011	13	123	195	46	118	192	113,3
27.06.2011	20	65	158	46	118	192	113,3
16.05.2011	14	87	198	52	147	212	113,4
16.05.2011	22	24	124	52	147	212	113,4
27.06.2011	46	118	192	48	81	168	113,4
27.06.2011	20	65	158	46	118	192	113,6
27.06.2011	46	118	192	48	81	168	113,6
16.05.2011	51	141	207	52	147	212	113,7
17.06.2011	20	65	158	46	118	192	113,7
03.06.2011	30	42	151	56	72	182	114,0
26.05.2011	58	71	182	60	119	217	114,2
07.06.2011	57	72	182	58	71	182	114,2
15.06.2011	46	118	192	48	81	168	114,2
15.06.2011	46	118	192	48	81	168	114,2
29.06.2011	13	123	195	46	118	192	114,2
15.06.2011	13	123	195	46	118	192	114,4
22.06.2011	34	47	141	46	118	192	115,0
24.06.2011	13	123	195	46	118	192	115,0
26.05.2011	30	42	151	58	71	182	115,5
22.06.2011	20	65	158	46	118	192	115,5
15.06.2011	46	118	192	48	81	168	115,7
16.05.2011	16	67	169	52	147	212	115,9
22.06.2011	20	65	158	34	47	141	116,0

15.06.2011	46	118	192	48	81	168	116,1
24.06.2011	13	123	195	46	118	192	116,4
17.05.2011	56	67	170	58	71	182	116,8
01.06.2011	27	46	152	58	71	182	117,2
01.06.2011	24	56	163	58	71	182	117,5
22.06.2011	46	118	192	48	81	168	117,5
01.06.2011	56	67	170	58	71	182	118,1
28.06.2011	20	65	158	46	118	192	118,8
16.05.2011	52	147	212	53	152	218	118,9
17.05.2011	57	72	182	58	71	182	118,9
16.06.2011	20	65	158	46	118	192	119,0
26.05.2011	58	71	182	60	119	217	119,1
26.05.2011	57	72	182	58	71	182	119,3
28.06.2011	13	123	195	46	118	192	119,3
18.05.2011	13	48	159	58	71	182	119,8
26.05.2011	33	30	132	58	71	182	119,8
18.05.2011	24	56	163	58	71	182	119,9
01.06.2011	33	30	132	58	71	182	120,0
27.06.2011	46	118	192	13	123	195	120,0
18.05.2011	33	30	132	58	71	182	120,2
26.05.2011	56	67	170	58	71	182	120,3
26.05.2011	13	48	159	58	71	182	120,4
27.06.2011	20	65	158	46	118	192	120,5
22.06.2011	46	118	192	48	81	168	120,6
26.05.2011	27	46	152	58	71	182	120,7
26.05.2011	24	56	163	58	71	182	120,8
26.05.2011	27	46	152	58	71	182	120,8
03.06.2011	33	117	290	58	71	182	120,9
23.06.2011	20	65	158	46	118	192	120,9
22.06.2011	13	123	195	46	118	192	121,5
18.05.2011	19	58	166	58	71	182	121,6
26.05.2011	33	30	132	58	71	182	121,8
02.06.2011	33	30	132	58	71	182	122,7

29.06.2011	20	65	158	46	118	192	122,7
16.05.2011	14	87	198	52	147	212	122,8
16.05.2011	16	67	169	52	147	212	122,8
16.05.2011	17	34	137	52	147	212	122,8
16.05.2011	51	141	207	52	147	212	122,8
16.05.2011	52	147	212	53	152	218	122,8
16.05.2011	52	147	212	55	126	201	122,8
16.05.2011	15	59	166	52	147	212	122,9
16.05.2011	22	24	124	52	147	212	122,9
16.05.2011	52	147	212	54	199	238	122,9
26.05.2011	30	42	151	58	71	182	122,9
26.05.2011	57	72	182	58	71	182	122,9
26.05.2011	58	71	182	60	119	217	123,0
01.06.2011	57	72	182	58	71	182	123,1
29.06.2011	46	118	192	48	81	168	123,1
28.06.2011	13	123	195	46	118	192	123,4
28.06.2011	46	118	192	48	81	168	123,6
23.06.2011	20	65	158	46	118	192	123,8
02.06.2011	19	58	166	58	71	182	124,4
23.06.2011	20	65	158	46	118	192	124,4
27.06.2011	13	123	195	46	118	192	124,4
29.06.2011	20	65	158	46	118	192	124,7
23.06.2011	34	47	141	46	118	192	124,9
01.06.2011	27	46	152	58	71	182	125,6
17.05.2011	52	147	212	55	126	201	125,9
17.05.2011	58	71	182	60	119	217	125,9
16.06.2011	13	123	195	46	118	192	125,9
28.06.2011	13	123	195	46	118	192	125,9
28.06.2011	13	123	195	46	118	192	126,0
28.06.2011	20	65	158	46	118	192	126,0
16.06.2011	20	65	158	46	118	192	126,4
03.06.2011	33	30	132	58	71	182	126,5
24.06.2011	20	65	158	46	118	192	126,5

23.06.2011	13	123	195	46	118	192	126,6
24.06.2011	20	65	158	46	118	192	126,8
17.06.2011	13	123	195	46	118	192	127,3
25.05.2011	13	48	159	58	71	182	127,8
25.05.2011	33	30	132	58	71	182	127,8
17.05.2011	56	67	170	58	71	182	127,9
25.05.2011	30	42	151	58	71	182	127,9
25.05.2011	57	72	182	58	71	182	127,9
25.05.2011	58	71	182	60	119	217	127,9
28.06.2011	46	118	192	48	81	168	127,9
02.06.2011	19	58	166	58	71	182	128,0
25.05.2011	13	48	159	56	67	170	128,1
25.05.2011	33	30	132	56	67	170	128,1
28.06.2011	20	65	158	46	118	192	128,1
25.05.2011	30	42	151	56	67	170	128,2
25.05.2011	56	67	170	57	72	182	128,2
25.05.2011	56	67	170	60	119	217	128,2
29.06.2011	13	123	195	46	118	192	128,3
28.06.2011	13	123	195	46	118	192	128,4
23.06.2011	13	123	195	46	118	192	128,5
01.06.2011	27	46	152	58	71	182	128,6
29.06.2011	13	123	195	46	118	192	128,7
26.05.2011	58	71	182	60	119	217	129,3
28.06.2011	20	65	158	46	118	192	129,3
28.06.2011	46	118	192	48	81	168	129,3
25.05.2011	57	72	182	58	71	182	129,4
17.06.2011	13	123	195	46	118	192	129,7
23.06.2011	13	123	195	46	118	192	129,9
23.06.2011	13	123	195	46	118	192	129,9
02.06.2011	33	56	163	58	71	182	130,0
15.06.2011	13	123	195	46	118	192	130,0
26.05.2011	56	67	170	58	71	182	130,2
02.06.2011	27	56	163	58	42	151	130,3

15.06.2011	13	123	195	46	118	192	130,3
24.06.2011	13	123	195	46	118	192	130,3
16.05.2011	23	21	117	52	147	212	130,4
01.06.2011	24	56	163	58	71	182	130,4
01.06.2011	33	30	132	58	71	182	130,4
01.06.2011	57	72	182	58	71	182	130,4
01.06.2011	19	58	166	58	71	182	130,4
02.06.2011	27	46	152	58	71	182	130,4
26.05.2011	13	48	159	58	71	182	130,6
01.06.2011	27	46	152	58	71	182	130,6
16.06.2011	13	123	195	46	118	192	130,6
26.05.2011	30	42	151	58	71	182	130,7
26.05.2011	19	58	166	58	71	182	130,8
26.05.2011	24	56	163	58	71	182	130,8
26.05.2011	19	58	166	58	71	182	130,9
26.05.2011	57	72	182	58	71	182	131,2
26.05.2011	33	30	132	58	71	182	131,3
03.06.2011	56	127	314	58	71	182	131,4
26.05.2011	24	56	163	58	71	182	131,5
23.06.2011	35	50	138	46	118	192	131,6
26.05.2011	27	46	152	58	71	182	131,7
03.06.2011	56	72	182	58	71	182	131,7
16.06.2011	46	118	192	48	81	168	131,7
25.05.2011	27	46	152	58	71	182	131,8
28.06.2011	13	123	195	46	118	192	131,8
16.05.2011	58	71	182	59	89	191	132,0
23.06.2011	13	123	195	46	118	192	132,0
25.05.2011	13	48	159	58	71	182	132,2
22.06.2011	13	123	195	46	118	192	132,3
25.05.2011	56	67	170	58	71	182	132,5
25.05.2011	30	42	151	58	71	182	132,6
17.06.2011	46	118	192	48	81	168	132,6
28.06.2011	20	65	158	46	118	192	132,6

25.05.2011	56	67	170	58	71	182	132,7
25.05.2011	57	72	182	58	71	182	132,7
25.05.2011	58	71	182	60	119	217	132,7
25.05.2011	30	42	151	58	71	182	132,8
26.05.2011	33	30	132	58	71	182	132,9
25.05.2011	58	71	182	60	119	217	133,0
23.06.2011	13	123	195	46	118	192	133,3
02.06.2011	33	30	132	58	71	182	133,5
17.05.2011	13	48	159	58	71	182	133,6
17.05.2011	27	46	152	58	71	182	133,6
17.05.2011	30	42	151	58	71	182	133,6
02.06.2011	57	72	182	58	71	182	133,6
17.05.2011	57	72	182	58	71	182	133,7
17.05.2011	58	71	182	59	89	191	133,7
17.05.2011	58	71	182	60	119	217	133,8
26.05.2011	24	56	163	58	71	182	133,8
02.06.2011	24	58	166	58	71	182	133,8
01.06.2011	33	30	132	58	71	182	134,0
26.05.2011	19	58	166	58	71	182	134,1
26.05.2011	56	67	170	58	71	182	134,1
26.05.2011	27	46	152	58	71	182	134,3
25.05.2011	24	56	163	58	71	182	134,4
25.05.2011	24	56	163	56	67	170	134,7
23.06.2011	20	65	158	46	118	192	134,7
25.05.2011	33	30	132	58	71	182	134,8
25.05.2011	19	58	166	58	71	182	135,5
22.06.2011	28	53	146	46	118	192	135,5
02.06.2011	19	58	166	58	30	132	135,6
25.05.2011	19	58	166	56	67	170	135,7
25.05.2011	27	46	152	58	71	182	135,7
22.06.2011	35	50	138	46	118	192	135,7
25.05.2011	27	46	152	56	67	170	135,9
23.06.2011	46	118	192	48	81	168	136,0

23.06.2011	13	123	195	46	118	192	136,3
18.05.2011	57	72	182	58	71	182	136,7
18.05.2011	58	71	182	60	119	217	136,7
01.06.2011	57	72	182	58	71	182	136,7
23.06.2011	28	53	146	46	118	192	136,8
18.05.2011	56	67	170	58	71	182	136,9
15.06.2011	28	53	146	46	118	192	136,9
23.06.2011	20	65	158	46	118	192	136,9
18.05.2011	30	42	151	58	71	182	137,1
27.06.2011	20	65	158	46	118	192	137,1
18.05.2011	27	46	152	58	71	182	137,2
22.06.2011	46	118	192	48	81	168	137,2
16.06.2011	13	123	195	46	118	192	137,3
22.06.2011	13	123	195	46	118	192	137,3
16.06.2011	46	118	192	48	81	168	137,7
18.05.2011	58	71	182	59	89	191	138,0
01.06.2011	56	67	170	58	71	182	138,1
16.06.2011	13	123	195	46	118	192	138,2
26.05.2011	19	58	166	58	71	182	138,8
16.06.2011	46	118	192	48	81	168	139,0
16.06.2011	46	118	192	48	81	168	139,6
02.06.2011	33	30	132	58	71	182	139,7
15.06.2011	35	50	138	46	118	192	139,7
02.06.2011	56	67	170	58	71	182	140,0
16.06.2011	13	123	195	46	118	192	140,4
22.06.2011	13	123	195	46	118	192	140,9
29.06.2011	46	118	192	48	81	168	140,9
25.05.2011	33	30	132	58	71	182	141,2
01.06.2011	33	30	132	58	71	182	141,2
25.05.2011	19	58	166	58	71	182	141,3
25.05.2011	24	56	163	58	71	182	141,5
01.06.2011	19	58	166	58	71	182	141,7
25.05.2011	19	58	166	58	71	182	141,8

28.06.2011	35	50	138	46	118	192	143,1
29.06.2011	20	65	158	46	118	192	143,3
17.06.2011	46	118	192	48	81	168	144,5
15.06.2011	13	123	195	46	118	192	144,9
28.06.2011	28	53	138	46	118	192	144,9
22.06.2011	13	123	195	46	118	192	145,1
17.05.2011	24	56	163	58	71	182	145,4
17.05.2011	33	30	132	58	71	182	145,4
26.05.2011	58	71	182	60	119	217	145,4
26.05.2011	56	67	170	58	71	182	145,5
26.05.2011	57	72	182	58	71	182	145,5
17.05.2011	56	67	170	58	71	182	145,6
15.06.2011	46	118	192	48	81	168	145,9
01.06.2011	19	58	166	58	71	182	146,0
25.05.2011	27	46	152	58	71	182	146,1
29.06.2011	35	50	138	46	118	192	146,4
25.05.2011	24	56	163	58	71	182	146,5
18.05.2011	24	56	163	58	71	182	147,6
18.05.2011	33	30	132	58	71	182	147,6
23.06.2011	20	65	158	46	118	192	147,7
18.05.2011	13	48	159	58	71	182	147,8
23.06.2011	13	123	195	46	118	192	148,3
16.05.2011	19	58	166	58	71	182	149,0
18.05.2011	19	58	166	58	71	182	149,8
16.05.2011	24	56	163	58	71	182	149,9
26.05.2011	30	42	151	58	71	182	149,9
26.05.2011	58	71	182	60	119	217	150,5
26.05.2011	13	48	159	58	71	182	151,4
16.05.2011	30	42	151	58	71	182	151,5
02.06.2011	24	56	163	58	71	182	151,8
23.06.2011	28	53	146	46	118	192	151,9
25.05.2011	57	72	182	58	71	182	152,3
25.05.2011	58	71	182	60	119	217	152,3

25.05.2011	30	42	151	58	71	182	152,4
26.05.2011	56	67	170	58	71	182	153,1
26.05.2011	58	71	182	60	119	217	153,4
16.05.2011	13	48	159	58	71	182	153,9
16.05.2011	27	46	152	58	71	182	153,9
16.05.2011	33	30	132	58	71	182	153,9
16.05.2011	57	72	182	58	71	182	153,9
29.06.2011	28	53	138	46	118	192	153,9
16.05.2011	58	71	182	60	119	217	154,0
16.05.2011	56	67	170	58	71	182	154,1
28.06.2011	28	53	138	46	118	192	154,3
27.06.2011	34	47	141	46	118	192	154,4
26.05.2011	33	30	132	58	71	182	154,6
28.06.2011	35	50	138	46	118	192	154,7
26.05.2011	24	56	163	58	71	182	154,9
26.05.2011	27	46	152	58	71	182	154,9
15.06.2011	34	47	141	46	118	192	154,9
17.06.2011	35	50	138	46	118	192	154,9
16.06.2011	28	53	146	46	118	192	155,0
17.05.2011	13	48	159	58	71	182	155,1
17.05.2011	27	46	152	58	71	182	155,1
17.05.2011	30	42	151	58	71	182	155,1
17.05.2011	56	67	170	58	71	182	155,1
17.05.2011	57	72	182	58	71	182	155,2
17.05.2011	58	71	182	59	89	191	155,2
18.05.2011	24	56	163	58	71	182	155,2
18.05.2011	58	71	182	59	89	191	155,2
26.05.2011	56	67	170	58	71	182	155,2
16.06.2011	35	50	138	46	118	192	155,4
17.06.2011	28	53	146	46	118	192	155,4
26.05.2011	19	58	166	58	71	182	155,9
28.06.2011	20	65	158	46	118	192	156,1
28.06.2011	46	118	192	48	81	168	156,2

25.05.2011	33	30	132	58	71	182	156,3
25.05.2011	56	67	170	58	71	182	156,3
23.06.2011	34	47	141	46	118	192	156,3
27.06.2011	13	123	195	46	118	192	156,5
23.06.2011	35	50	138	46	118	192	157,4
25.05.2011	27	46	152	58	71	182	158,1
17.05.2011	19	58	166	58	71	182	158,3
18.05.2011	13	48	159	58	71	182	158,5
18.05.2011	30	42	151	58	71	182	158,5
18.05.2011	33	30	132	58	71	182	158,5
18.05.2011	56	67	170	58	71	182	158,5
18.05.2011	57	72	182	58	71	182	158,5
25.05.2011	24	56	163	58	71	182	158,5
18.05.2011	58	71	182	60	119	217	158,6
17.06.2011	35	50	138	46	118	192	158,6
25.05.2011	19	58	166	58	71	182	158,7
17.06.2011	34	47	141	46	118	192	158,8
15.06.2011	34	47	141	46	118	192	159,2
27.06.2011	34	47	141	46	118	192	159,4
27.06.2011	34	47	141	46	118	192	159,8
23.06.2011	28	53	146	46	118	192	159,9
28.06.2011	34	47	141	46	118	192	160,0
24.06.2011	34	47	141	46	118	192	160,2
26.05.2011	13	48	159	58	71	182	160,3
26.05.2011	30	42	151	58	71	182	160,3
23.06.2011	34	47	141	46	118	192	160,3
23.06.2011	34	47	141	46	118	192	160,3
27.06.2011	34	47	141	46	118	192	160,3
26.05.2011	57	72	182	58	71	182	160,4
17.05.2011	58	71	182	60	119	217	160,6
16.06.2011	34	47	141	46	118	192	161,1
22.06.2011	34	47	141	46	118	192	161,1
26.05.2011	13	48	159	58	71	182	161,2

15.06.2011	34	47	141	46	118	192	161,6
29.06.2011	28	53	138	46	118	192	161,6
28.06.2011	34	47	141	46	118	192	161,9
28.06.2011	34	47	141	46	118	192	161,9
15.06.2011	34	47	141	46	118	192	162,0
16.06.2011	28	53	146	46	118	192	162,0
23.06.2011	34	47	141	46	118	192	162,5
26.05.2011	30	42	151	58	71	182	162,7
26.05.2011	57	72	182	58	71	182	162,8
25.05.2011	13	48	159	58	71	182	162,9
26.05.2011	56	67	170	58	71	182	162,9
23.06.2011	34	47	141	46	118	192	163,0
16.06.2011	34	47	141	46	118	192	163,3
28.06.2011	35	50	138	46	118	192	163,4
22.06.2011	35	50	138	46	118	192	163,9
24.06.2011	34	47	141	46	118	192	164,3
29.06.2011	34	47	141	46	118	192	164,4
22.06.2011	34	47	141	46	118	192	164,5
16.06.2011	34	47	141	46	118	192	164,7
28.06.2011	34	47	141	46	118	192	164,7
24.06.2011	35	50	138	46	118	192	165,1
16.06.2011	34	47	141	46	118	192	165,2
26.05.2011	24	56	163	58	71	182	165,3
26.05.2011	33	30	132	58	71	182	165,3
28.06.2011	35	50	138	46	118	192	165,3
26.05.2011	27	46	152	58	71	182	165,4
26.05.2011	27	46	152	58	71	182	165,7
02.06.2011	19	58	166	58	71	182	165,9
28.06.2011	28	53	138	46	118	192	166,1
29.06.2011	34	47	141	46	118	192	166,1
26.05.2011	19	58	166	58	71	182	166,2
22.06.2011	28	53	146	46	118	192	166,2
27.06.2011	35	50	138	46	118	192	166,3

22.06.2011	35	50	138	46	118	192	166,5
28.06.2011	28	53	138	46	118	192	166,6
27.06.2011	28	53	138	46	118	192	166,8
16.06.2011	35	50	138	46	118	192	166,9
15.06.2011	35	50	138	46	118	192	167,1
22.06.2011	35	50	138	46	118	192	167,3
23.06.2011	35	50	138	46	118	192	167,3
28.06.2011	34	47	141	46	118	192	167,3
26.05.2011	24	56	163	58	71	182	167,6
26.05.2011	33	30	132	58	71	182	167,7
23.06.2011	35	50	138	46	118	192	168,2
16.06.2011	28	53	146	46	118	192	168,3
22.06.2011	34	47	141	46	118	192	168,3
23.06.2011	35	50	138	46	118	192	169,1
28.06.2011	28	53	138	46	118	192	169,2
16.06.2011	34	47	141	46	118	192	169,3
27.06.2011	34	47	141	46	118	192	169,3
27.06.2011	35	50	138	46	118	192	169,3
22.06.2011	28	53	146	46	118	192	169,6
24.06.2011	34	47	141	46	118	192	169,8
28.06.2011	13	123	195	46	118	192	169,8
24.06.2011	35	50	138	46	118	192	169,9
16.06.2011	28	53	146	46	118	192	170,0
16.06.2011	35	50	138	46	118	192	170,2
28.06.2011	28	53	138	46	118	192	170,3
24.06.2011	28	53	146	46	118	192	170,4
15.06.2011	35	50	138	46	118	192	170,5
16.06.2011	35	50	138	46	118	192	170,6
15.06.2011	35	50	138	46	118	192	170,7
23.06.2011	28	53	146	46	118	192	170,7
27.06.2011	35	50	138	46	118	192	170,7
18.05.2011	19	58	166	58	71	182	170,9
15.06.2011	28	53	146	46	118	192	171,0

15.06.2011	28	53	146	46	118	192	171,0
17.05.2011	24	56	163	58	71	182	171,1
18.05.2011	27	46	152	58	71	182	171,1
17.05.2011	33	30	132	58	71	182	171,2
15.06.2011	28	53	146	46	118	192	171,2
15.06.2011	35	50	138	46	118	192	171,3
22.06.2011	34	47	141	46	118	192	171,3
27.06.2011	28	53	138	46	118	192	171,5
17.05.2011	19	58	166	58	71	182	171,7
15.06.2011	28	53	146	46	118	192	171,9
22.06.2011	35	50	138	46	118	192	172,0
02.06.2011	56	67	170	58	71	182	172,1
29.06.2011	28	53	138	46	118	192	172,4
23.06.2011	35	50	138	46	118	192	172,6
23.06.2011	35	50	138	46	118	192	172,6
16.06.2011	28	53	146	46	118	192	172,7
27.06.2011	28	53	138	46	118	192	172,8
17.06.2011	28	53	146	46	118	192	172,9
28.06.2011	28	53	138	46	118	192	172,9
16.06.2011	34	47	141	46	118	192	173,0
22.06.2011	28	53	146	46	118	192	173,0
29.06.2011	34	47	141	46	118	192	173,2
22.06.2011	28	53	146	46	118	192	173,3
27.06.2011	35	50	138	46	118	192	173,9
28.06.2011	46	118	192	48	81	168	173,9
27.06.2011	28	53	138	46	118	192	174,1
16.06.2011	35	50	138	46	118	192	174,2
23.06.2011	28	53	146	46	118	192	174,3
16.06.2011	28	53	146	46	118	192	174,7
23.06.2011	28	53	146	46	118	192	174,8
28.06.2011	35	50	138	46	118	192	174,8
29.06.2011	35	50	138	46	118	192	175,4
27.06.2011	28	53	138	46	118	192	175,6

27.06.2011	35	50	138	46	118	192	175,8
23.06.2011	28	53	146	46	118	192	175,9
23.06.2011	28	53	146	46	118	192	176,3
28.06.2011	35	50	138	46	118	192	176,5
23.06.2011	35	50	138	46	118	192	176,6
24.06.2011	28	53	146	46	118	192	176,7
23.06.2011	34	47	141	46	118	192	177,4
29.06.2011	35	50	138	46	118	192	177,9
28.06.2011	20	65	158	46	118	192	179,1
28.06.2011	35	50	138	46	118	192	179,6
17.06.2011	34	47	141	46	118	192	179,8
26.05.2011	19	58	166	58	71	182	180,4
24.06.2011	35	50	138	46	118	192	180,6
24.06.2011	28	53	146	46	118	192	181,2
16.06.2011	20	65	158	48	81	168	181,5
28.06.2011	34	47	141	46	118	192	183,6
15.06.2011	20	65	158	48	81	168	184,8
15.06.2011	20	65	158	48	81	168	186,3
25.05.2011	13	48	159	58	71	182	187,8
15.06.2011	20	65	158	48	81	168	188,1
15.06.2011	34	47	141	46	118	192	189,4
23.06.2011	34	47	141	46	118	192	192,4
16.06.2011	46	118	192	48	81	168	193,1
28.06.2011	34	47	141	46	118	192	194,7
15.06.2011	13	123	195	20	65	158	194,9
15.06.2011	20	65	158	48	81	168	196,2
27.06.2011	46	118	192	48	81	168	196,3
22.06.2011	34	47	141	46	118	192	196,4
17.06.2011	20	65	158	46	118	192	197,6
15.06.2011	13	123	195	20	65	158	198,3
15.06.2011	20	65	158	48	81	168	198,5
15.06.2011	20	65	158	48	81	168	199,3
15.06.2011	13	123	195	20	65	158	199,7

17.06.2011	46	118	192	48	81	168	202,8
27.06.2011	20	65	158	46	118	192	204,6
15.06.2011	13	123	195	20	65	158	205,4
16.06.2011	46	118	192	48	81	168	206,3
16.06.2011	13	123	195	46	118	192	206,4
16.06.2011	20	65	158	46	118	192	206,4
22.06.2011	20	65	158	28	53	146	215,2
22.06.2011	20	65	158	35	50	138	215,3
22.06.2011	28	53	146	46	118	192	216,3
16.06.2011	13	123	195	20	65	158	219,2
22.06.2011	35	50	138	46	118	192	220,7
15.06.2011	46	118	192	48	81	168	225,5
27.06.2011	13	123	195	28	53	146	226,3
15.06.2011	13	123	195	20	65	158	227,5
17.06.2011	34	47	141	46	118	192	230,8
15.06.2011	20	65	158	34	47	141	230,9
16.06.2011	13	123	195	46	118	192	231,0
17.06.2011	13	123	195	46	118	192	233,4
15.06.2011	20	65	158	34	47	141	233,8
27.06.2011	13	123	195	35	50	138	234,3
15.06.2011	20	65	158	34	47	141	236,6
22.06.2011	20	65	158	28	53	146	238,9
22.06.2011	20	65	158	35	50	138	238,9
16.06.2011	20	65	158	34	47	141	240,2
22.06.2011	20	65	158	28	53	146	240,3
22.06.2011	35	50	138	48	81	168	240,5
23.06.2011	28	53	146	48	81	168	240,5
22.06.2011	28	53	146	48	81	168	240,6
22.06.2011	20	65	158	35	50	138	243,3
22.06.2011	20	65	158	28	53	146	243,8
15.06.2011	20	65	158	34	47	141	244,0
16.06.2011	34	47	141	46	118	192	244,2
22.06.2011	20	65	158	35	50	138	244,8

15.06.2011	20	65	158	34	47	141	245,2
28.06.2011	34	47	141	46	118	192	245,5
28.06.2011	13	123	195	35	50	138	245,5
23.06.2011	35	50	138	48	81	168	246,3
28.06.2011	13	123	195	28	53	146	247,3
27.06.2011	34	47	141	46	118	192	248,0
16.06.2011	34	47	141	46	118	192	251,7
15.06.2011	13	123	195	46	118	192	254,3
23.06.2011	35	50	138	48	81	168	255,1
23.06.2011	20	65	158	28	53	146	255,8
16.06.2011	20	65	158	28	53	146	257,1
16.06.2011	20	65	158	35	50	138	257,4
23.06.2011	28	53	146	48	81	168	258,1
29.06.2011	28	53	138	48	81	168	259,4
24.06.2011	35	50	138	48	81	168	259,8
28.06.2011	13	123	195	28	53	146	259,9
15.06.2011	20	65	158	34	47	141	260,6
17.06.2011	20	65	158	35	50	138	260,8
23.06.2011	28	53	146	48	81	168	261,3
17.06.2011	20	65	158	28	53	146	261,4
17.06.2011	20	65	158	35	50	138	262,4
16.06.2011	28	53	146	48	81	168	262,5
23.06.2011	35	50	138	48	81	168	262,8
23.06.2011	35	50	138	48	81	168	262,9
16.06.2011	35	50	138	48	81	168	263,4
28.06.2011	28	53	138	48	81	168	263,6
29.06.2011	13	123	195	28	53	146	263,9
27.06.2011	28	53	138	48	81	168	265,3
23.06.2011	28	53	146	48	81	168	266,2
23.06.2011	35	50	138	48	81	168	266,6
23.06.2011	35	50	138	48	81	168	267,0
15.06.2011	28	53	146	48	81	168	267,3
23.06.2011	35	50	138	48	81	168	267,5

28.06.2011	20	65	158	28	53	146	268,0
27.06.2011	28	53	138	48	81	168	268,8
23.06.2011	28	53	146	48	81	168	268,9
28.06.2011	28	53	138	48	81	168	269,0
28.06.2011	28	53	138	48	81	168	269,1
22.06.2011	20	65	158	35	50	138	269,2
27.06.2011	13	123	195	35	50	138	269,2
28.06.2011	20	65	158	28	53	146	269,2
15.06.2011	35	50	138	48	81	168	270,0
27.06.2011	20	65	158	35	50	138	270,2
27.06.2011	35	50	138	48	81	168	270,2
29.06.2011	13	123	195	35	50	138	270,4
27.06.2011	13	123	195	28	53	146	270,5
28.06.2011	28	53	138	48	81	168	270,9
22.06.2011	28	53	146	48	81	168	271,0
22.06.2011	20	65	158	35	50	138	271,0
28.06.2011	13	123	195	35	50	138	271,0
24.06.2011	35	50	138	48	81	168	271,1
27.06.2011	35	50	138	48	81	168	271,1
22.06.2011	35	50	138	48	81	168	271,4
24.06.2011	28	53	146	48	81	168	271,6
24.06.2011	35	50	138	48	81	168	271,8
23.06.2011	28	53	146	48	81	168	272,0
24.06.2011	28	53	146	48	81	168	272,3
24.06.2011	28	53	146	48	81	168	272,3
15.06.2011	35	50	138	48	81	168	272,4
23.06.2011	13	123	195	28	53	146	272,6
22.06.2011	35	50	138	48	81	168	272,8
28.06.2011	28	53	138	48	81	168	272,9
27.06.2011	20	65	158	28	53	146	273,0
17.06.2011	13	123	195	35	50	138	273,2
28.06.2011	35	50	138	48	81	168	273,2
27.06.2011	35	50	138	48	81	168	273,5

23.06.2011	20	65	158	35	50	138	273,6
27.06.2011	13	123	195	35	50	138	273,6
23.06.2011	28	53	146	48	81	168	273,7
23.06.2011	28	53	146	48	81	168	273,7
23.06.2011	20	65	158	35	50	138	273,8
23.06.2011	20	65	158	35	50	138	273,9
22.06.2011	13	123	195	28	53	146	274,0
22.06.2011	13	123	195	35	50	138	274,0
27.06.2011	13	123	195	35	50	138	274,0
28.06.2011	13	123	195	35	50	138	274,4
15.06.2011	35	50	138	48	81	168	274,5
27.06.2011	13	123	195	28	53	146	274,5
27.06.2011	20	65	158	35	50	138	274,5
23.06.2011	13	123	195	35	50	138	274,6
27.06.2011	35	50	138	48	81	168	274,6
28.06.2011	35	50	138	48	81	168	274,8
22.06.2011	35	50	138	48	81	168	275,4
22.06.2011	20	65	158	28	53	146	275,4
27.06.2011	28	53	138	48	81	168	275,4
27.06.2011	35	50	138	48	81	168	275,4
27.06.2011	20	65	158	28	53	146	275,4
15.06.2011	28	53	146	48	81	168	275,5
27.06.2011	28	53	138	48	81	168	275,6
15.06.2011	35	50	138	48	81	168	275,8
22.06.2011	13	123	195	35	50	138	275,9
27.06.2011	28	53	138	48	81	168	275,9
27.06.2011	20	65	158	28	53	146	276,0
15.06.2011	28	53	146	48	81	168	276,1
22.06.2011	13	123	195	28	53	146	276,1
29.06.2011	35	50	138	48	81	168	276,2
15.06.2011	13	123	195	28	53	146	276,3
17.06.2011	20	65	158	28	53	146	276,6
28.06.2011	35	50	138	48	81	168	276,7

24.06.2011	20	65	158	35	50	138	276,9
16.06.2011	13	123	195	35	50	138	277,0
23.06.2011	20	65	158	28	53	146	277,0
16.06.2011	20	65	158	28	53	146	277,1
16.06.2011	20	65	158	35	50	138	277,2
15.06.2011	28	53	146	48	81	168	277,3
22.06.2011	35	50	138	48	81	168	277,3
15.06.2011	13	123	195	28	53	146	277,4
16.06.2011	35	50	138	48	81	168	277,4
24.06.2011	20	65	158	28	53	146	277,4
16.06.2011	13	123	195	35	50	138	277,5
15.06.2011	28	53	146	48	81	168	277,6
22.06.2011	28	53	146	48	81	168	277,6
15.06.2011	13	123	195	35	50	138	277,9
17.06.2011	13	123	195	35	50	138	277,9
23.06.2011	20	65	158	35	50	138	277,9
24.06.2011	13	123	195	35	50	138	277,9
22.06.2011	20	65	158	28	53	146	278,0
29.06.2011	13	123	195	28	53	146	278,1
24.06.2011	13	123	195	28	53	146	278,3
22.06.2011	28	53	146	48	81	168	278,4
17.06.2011	20	65	158	28	53	146	278,5
17.06.2011	13	123	195	28	53	146	278,5
16.06.2011	20	65	158	35	50	138	278,6
23.06.2011	13	123	195	35	50	138	278,6
15.06.2011	28	53	146	48	81	168	278,7
16.06.2011	13	123	195	28	53	146	278,7
27.06.2011	13	123	195	28	53	146	278,7
28.06.2011	35	50	138	48	81	168	278,7
16.06.2011	20	65	158	28	53	146	278,9
28.06.2011	13	123	195	28	53	146	278,9
22.06.2011	28	53	146	48	81	168	279,0
28.06.2011	20	65	158	35	50	138	279,0

16.06.2011	28	53	146	48	81	168	279,1
15.06.2011	35	50	138	48	81	168	279,2
16.06.2011	13	123	195	28	53	146	279,2
23.06.2011	35	50	138	48	81	168	279,3
28.06.2011	20	65	158	35	50	138	279,3
28.06.2011	35	50	138	48	81	168	279,3
27.06.2011	35	50	138	48	81	168	279,4
27.06.2011	13	123	195	28	53	146	279,4
15.06.2011	13	123	195	35	50	138	279,5
27.06.2011	20	65	158	35	50	138	279,5
27.06.2011	20	65	158	28	53	146	279,7
27.06.2011	28	53	138	48	81	168	279,7
28.06.2011	13	123	195	28	53	146	279,7
29.06.2011	20	65	158	28	53	146	279,8
23.06.2011	20	65	158	35	50	138	279,9
23.06.2011	20	65	158	28	53	146	280,0
16.06.2011	13	123	195	28	53	146	280,1
28.06.2011	13	123	195	28	53	146	280,2
23.06.2011	20	65	158	35	50	138	280,3
15.06.2011	35	50	138	48	81	168	280,4
16.06.2011	13	123	195	35	50	138	280,4
27.06.2011	35	50	138	13	123	195	280,4
27.06.2011	28	53	138	13	123	195	280,5
28.06.2011	20	65	158	28	53	146	280,5
28.06.2011	13	123	195	35	50	138	280,5
16.06.2011	20	65	158	28	53	146	280,6
23.06.2011	13	123	195	28	53	146	280,6
29.06.2011	13	123	195	35	50	138	280,6
16.06.2011	28	53	146	48	81	168	280,7
28.06.2011	20	65	158	35	50	138	280,8
16.06.2011	35	50	138	48	81	168	281,0
27.06.2011	20	65	158	28	53	146	281,0
27.06.2011	20	65	158	35	50	138	281,0

23.06.2011	20	65	158	28	53	146	281,2
27.06.2011	20	65	158	35	50	138	281,2
28.06.2011	13	123	195	28	53	146	281,3
28.06.2011	13	123	195	35	50	138	281,4
17.06.2011	35	50	138	48	81	168	281,6
27.06.2011	13	123	195	35	50	138	281,6
28.06.2011	13	123	195	28	53	146	282,3
24.06.2011	13	123	195	35	50	138	283,0
24.06.2011	13	123	195	28	53	146	283,5
23.06.2011	13	123	195	35	50	138	283,8
24.06.2011	13	123	195	35	50	138	284,0
17.06.2011	28	53	146	48	81	168	284,3
16.06.2011	13	123	195	28	53	146	284,9
16.06.2011	20	65	158	28	53	146	284,9
16.06.2011	28	53	146	48	81	168	284,9
23.06.2011	20	65	158	28	53	146	285,1
29.06.2011	35	50	138	48	81	168	285,1
28.06.2011	13	123	195	28	53	146	285,3
16.06.2011	13	123	195	35	50	138	285,4
16.06.2011	20	65	158	35	50	138	285,4
16.06.2011	35	50	138	48	81	168	285,4
23.06.2011	20	65	158	28	53	146	285,6
24.06.2011	20	65	158	35	50	138	285,8
28.06.2011	20	65	158	28	53	146	285,8
22.06.2011	13	123	195	35	50	138	286,5
28.06.2011	13	123	195	35	50	138	286,5
28.06.2011	35	50	138	48	81	168	286,7
22.06.2011	35	50	138	48	81	168	286,8
28.06.2011	20	65	158	35	50	138	286,8
22.06.2011	28	53	146	48	81	168	286,9
23.06.2011	20	65	158	28	53	146	287,0
23.06.2011	13	123	195	28	53	146	287,1
17.06.2011	13	123	195	28	53	146	287,3

29.06.2011	13	123	195	28	53	146	287,4
29.06.2011	20	65	158	35	50	138	287,7
15.06.2011	34	47	141	46	118	192	287,9
23.06.2011	13	123	195	35	50	138	288,4
28.06.2011	20	65	158	28	53	146	288,6
15.06.2011	13	123	195	35	50	138	288,9
16.06.2011	28	53	146	48	81	168	289,0
23.06.2011	13	123	195	35	50	138	289,0
29.06.2011	20	65	158	28	53	146	289,0
29.06.2011	28	53	138	48	81	168	289,2
16.06.2011	13	123	195	28	53	146	289,4
17.06.2011	20	65	158	35	50	138	289,4
28.06.2011	35	50	138	48	81	168	289,4
28.06.2011	13	123	195	35	50	138	289,5
28.06.2011	20	65	158	35	50	138	289,5
15.06.2011	13	123	195	28	53	146	289,9
28.06.2011	20	65	158	28	53	146	290,5
28.06.2011	28	53	138	48	81	168	290,5
22.06.2011	13	123	195	28	53	146	290,8
23.06.2011	13	123	195	35	50	138	291,1
29.06.2011	13	123	195	35	50	138	291,1
22.06.2011	13	123	195	35	50	138	291,4
23.06.2011	20	65	158	35	50	138	291,5
17.06.2011	35	50	138	48	81	168	291,6
23.06.2011	13	123	195	28	53	146	291,7
23.06.2011	13	123	195	28	53	146	291,8
23.06.2011	34	47	141	35	50	138	292,0
17.06.2011	28	53	146	48	81	168	292,2
24.06.2011	20	65	158	35	50	138	292,3
29.06.2011	20	65	158	35	50	138	292,4
16.06.2011	28	53	146	48	81	168	292,5
29.06.2011	35	50	138	48	81	168	292,7
16.06.2011	13	123	195	28	53	146	292,8

22.06.2011	13	123	195	28	53	146	292,8
24.06.2011	20	65	158	28	53	146	292,8
29.06.2011	28	53	138	48	81	168	292,8
16.06.2011	35	50	138	48	81	168	293,0
16.06.2011	13	123	195	35	50	138	293,2
23.06.2011	13	123	195	35	50	138	293,3
28.06.2011	28	53	138	48	81	168	293,7
28.06.2011	20	65	158	35	50	138	293,8
28.06.2011	20	65	158	28	53	146	293,8
23.06.2011	13	123	195	28	53	146	293,9
28.06.2011	13	123	195	35	50	138	294,0
23.06.2011	28	53	146	34	47	141	294,9
17.06.2011	35	50	138	48	81	168	295,2
22.06.2011	13	123	195	35	50	138	295,3
29.06.2011	20	65	158	28	53	146	295,3
15.06.2011	13	123	195	35	50	138	295,6
15.06.2011	13	123	195	28	53	146	295,7
17.06.2011	28	53	146	48	81	168	295,7
15.06.2011	13	123	195	28	53	146	295,8
28.06.2011	20	65	158	35	50	138	296,2
16.06.2011	13	123	195	35	50	138	296,4
29.06.2011	20	65	158	35	50	138	296,5
24.06.2011	13	123	195	28	53	146	296,6
28.06.2011	20	65	158	35	50	138	296,6
28.06.2011	35	50	138	48	81	168	296,7
16.06.2011	35	50	138	48	81	168	298,0
23.06.2011	20	65	158	35	50	138	298,1
23.06.2011	13	123	195	28	53	146	298,2
24.06.2011	20	65	158	28	53	146	298,2
15.06.2011	13	123	195	35	50	138	298,4
16.06.2011	13	123	195	28	53	146	298,4
22.06.2011	13	123	195	28	53	146	298,4
28.06.2011	20	65	158	28	53	146	298,4

28.06.2011	28	53	138	48	81	168	298,5
27.06.2011	20	65	158	28	53	146	298,8
16.06.2011	28	53	146	48	81	168	300,0
23.06.2011	13	123	195	28	53	146	300,0
22.06.2011	13	123	195	35	50	138	300,2
22.06.2011	13	123	195	28	53	146	300,4
16.06.2011	13	123	195	28	53	146	300,7
27.06.2011	20	65	158	35	50	138	301,1
16.06.2011	13	123	195	35	50	138	301,6
23.06.2011	20	65	158	28	53	146	304,7
23.06.2011	13	123	195	35	50	138	309,4
17.06.2011	34	47	141	35	50	138	311,5
17.06.2011	28	53	146	34	47	141	313,4
23.06.2011	28	53	146	34	47	141	315,3
29.06.2011	34	47	141	35	50	138	316,3
17.06.2011	13	123	195	28	53	146	316,8
27.06.2011	28	53	138	34	47	141	317,1
27.06.2011	34	47	141	35	50	138	319,2
28.06.2011	34	47	141	35	50	138	319,2
29.06.2011	28	53	138	34	47	141	319,7
22.06.2011	34	47	141	35	50	138	319,9
22.06.2011	28	53	146	34	47	141	320,0
28.06.2011	28	53	138	34	47	141	320,2
23.06.2011	34	47	141	35	50	138	320,4
28.06.2011	34	47	141	35	50	138	320,4
16.06.2011	28	53	146	34	47	141	320,9
27.06.2011	28	53	138	34	47	141	320,9
28.06.2011	28	53	138	34	47	141	320,9
15.06.2011	34	47	141	35	50	138	321,2
16.06.2011	34	47	141	35	50	138	321,2
23.06.2011	34	47	141	35	50	138	321,2
15.06.2011	28	53	146	34	47	141	321,5
15.06.2011	34	47	141	35	50	138	321,6

23.06.2011	34	47	141	35	50	138	321,7
16.06.2011	34	47	141	35	50	138	321,9
22.06.2011	34	47	141	35	50	138	322,3
23.06.2011	34	47	141	35	50	138	322,4
15.06.2011	28	53	146	34	47	141	322,6
16.06.2011	28	53	146	34	47	141	322,9
16.06.2011	28	53	146	34	47	141	323,4
27.06.2011	34	47	141	35	50	138	323,6
16.06.2011	28	53	146	34	47	141	323,8
16.06.2011	28	53	146	34	47	141	324,0
16.06.2011	34	47	141	35	50	138	324,0
29.06.2011	28	53	138	34	47	141	324,0
16.06.2011	34	47	141	35	50	138	324,1
23.06.2011	34	47	141	35	50	138	324,1
17.06.2011	34	47	141	35	50	138	324,3
27.06.2011	34	47	141	35	50	138	324,4
27.06.2011	28	53	138	34	47	141	324,5
23.06.2011	28	53	146	34	47	141	324,6
15.06.2011	34	47	141	35	50	138	324,7
27.06.2011	28	53	138	34	47	141	325,1
27.06.2011	34	47	141	35	50	138	325,1
27.06.2011	34	47	141	35	50	138	325,3
15.06.2011	28	53	146	34	47	141	325,5
15.06.2011	34	47	141	35	50	138	325,7
17.06.2011	28	53	146	34	47	141	325,7
16.06.2011	28	53	146	34	47	141	326,0
22.06.2011	34	47	141	35	50	138	326,3
23.06.2011	28	53	146	34	47	141	326,3
16.06.2011	34	47	141	35	50	138	326,4
23.06.2011	28	53	146	34	47	141	326,4
15.06.2011	28	53	146	34	47	141	326,7
16.06.2011	34	47	141	35	50	138	326,8
23.06.2011	28	53	146	34	47	141	326,9

22.06.2011	28	53	146	34	47	141	327,2
28.06.2011	28	53	138	34	47	141	327,3
17.06.2011	13	123	195	35	50	138	327,7
28.06.2011	28	53	138	34	47	141	328,0
27.06.2011	28	53	138	34	47	141	328,1
15.06.2011	28	53	146	34	47	141	328,3
24.06.2011	34	47	141	35	50	138	328,4
15.06.2011	34	47	141	35	50	138	328,7
27.06.2011	28	53	138	34	47	141	328,7
28.06.2011	28	53	138	34	47	141	328,7
16.06.2011	28	53	146	34	47	141	328,9
29.06.2011	28	53	138	34	47	141	329,0
23.06.2011	28	53	146	34	47	141	329,1
22.06.2011	28	53	146	34	47	141	329,3
15.06.2011	28	53	146	34	47	141	330,0
16.06.2011	34	47	141	35	50	138	330,1
22.06.2011	34	47	141	35	50	138	330,3
22.06.2011	28	53	146	34	47	141	330,4
27.06.2011	34	47	141	35	50	138	330,4
28.06.2011	34	47	141	35	50	138	330,6
24.06.2011	34	47	141	35	50	138	330,9
23.06.2011	28	53	146	34	47	141	331,2
24.06.2011	28	53	146	34	47	141	331,3
16.06.2011	28	53	146	34	47	141	331,5
22.06.2011	34	47	141	35	50	138	331,7
17.06.2011	34	47	141	35	50	138	332,0
22.06.2011	28	53	146	34	47	141	332,4
29.06.2011	34	47	141	35	50	138	332,5
15.06.2011	34	47	141	35	50	138	332,6
17.06.2011	28	53	146	34	47	141	332,6
23.06.2011	34	47	141	35	50	138	332,7
24.06.2011	34	47	141	35	50	138	332,7
28.06.2011	28	53	138	34	47	141	333,0

24.06.2011	28	53	146	34	47	141	333,2
28.06.2011	34	47	141	35	50	138	333,3
23.06.2011	34	47	141	35	50	138	333,4
28.06.2011	34	47	141	35	50	138	333,4
22.06.2011	34	47	141	35	50	138	334,2
22.06.2011	28	53	146	34	47	141	334,5
28.06.2011	28	53	138	34	47	141	335,1
28.06.2011	28	53	138	34	47	141	335,2
28.06.2011	34	47	141	35	50	138	335,6
29.06.2011	34	47	141	35	50	138	336,4
28.06.2011	34	47	141	35	50	138	337,1
24.06.2011	28	53	146	34	47	141	340,9
28.06.2011	34	47	141	35	50	138	346,3
02.06.2010	171	13	99	183	47	149	359,57
02.06.2010	171	13	99	184	34	133	37,86
02.06.2010	183	47	149	184	34	133	369,36
02.06.2010	171	13	99	185	88	181	247,04
02.06.2010	183	47	149	185	88	181	123,25
02.06.2010	184	34	133	185	88	181	263,44
02.06.2010	171	13	99	186	45	146	145,26
02.06.2010	183	47	149	186	45	146	477,07
02.06.2010	184	34	133	186	45	146	116,56
02.06.2010	185	88	181	186	45	146	377,55
02.06.2010	171	13	99	187	75	174	102,41
02.06.2010	183	47	149	187	75	174	434,49
02.06.2010	184	34	133	187	75	174	71,97
02.06.2010	185	88	181	187	75	174	333,25
02.06.2010	186	45	146	187	75	174	44,68
02.06.2010	171	13	99	188	49	147	115,10
02.06.2010	183	47	149	188	49	147	444,86
02.06.2010	184	34	133	188	49	147	84,41
02.06.2010	185	88	181	188	49	147	344,78
02.06.2010	186	45	146	188	49	147	32,77

02.06.2010	187	75	174	188	49	147	12,70
02.06.2010	171	13	99	189	39	141	86,82
02.06.2010	183	47	149	189	39	141	322,94
02.06.2010	184	34	133	189	39	141	123,19
02.06.2010	185	88	181	189	39	141	200,94
02.06.2010	186	45	146	189	39	141	229,90
02.06.2010	187	75	174	189	39	141	188,47
02.06.2010	188	49	147	189	39	141	201,08
02.06.2010	171	13	99	190	25	122	79,92
02.06.2010	183	47	149	190	25	122	324,35
02.06.2010	184	34	133	190	25	122	116,23
02.06.2010	185	88	181	190	25	122	202,85
02.06.2010	186	45	146	190	25	122	223,27
02.06.2010	187	75	174	190	25	122	181,69
02.06.2010	188	49	147	190	25	122	194,31
02.06.2010	189	39	141	190	25	122	6,96
02.06.2010	171	13	99	192	101	192	52,24
02.06.2010	183	47	149	192	101	192	389,66
02.06.2010	184	34	133	192	101	192	20,96
02.06.2010	185	88	181	192	101	192	284,36
02.06.2010	186	45	146	192	101	192	96,06
02.06.2010	187	75	174	192	101	192	51,73
02.06.2010	188	49	147	192	101	192	64,34
02.06.2010	189	39	141	192	101	192	139,06
02.06.2010	190	25	122	192	101	192	132,16
02.06.2010	171	13	99	183	47	149	352,05
02.06.2010	171	13	99	184	34	133	36,29
02.06.2010	183	47	149	184	34	133	365,10
02.06.2010	171	13	99	185	88	181	249,67
02.06.2010	183	47	149	185	88	181	110,33
02.06.2010	184	34	133	185	88	181	267,73
02.06.2010	171	13	99	186	45	146	183,09
02.06.2010	183	47	149	186	45	146	498,03

02.06.2010	184	34	133	186	45	146	151,41
02.06.2010	185	88	181	186	45	146	410,45
02.06.2010	171	13	99	187	75	174	94,44
02.06.2010	183	47	149	187	75	174	420,36
02.06.2010	184	34	133	187	75	174	62,63
02.06.2010	185	88	181	187	75	174	327,04
02.06.2010	186	45	146	187	75	174	88,99
02.06.2010	171	13	99	188	49	147	156,57
02.06.2010	183	47	149	188	49	147	479,48
02.06.2010	184	34	133	188	49	147	126,17
02.06.2010	185	88	181	188	49	147	389,03
02.06.2010	186	45	146	188	49	147	27,96
02.06.2010	187	75	174	188	49	147	63,59
02.06.2010	171	13	99	189	39	141	76,53
02.06.2010	183	47	149	189	39	141	322,94
02.06.2010	184	34	133	189	39	141	112,45
02.06.2010	185	88	181	189	39	141	213,59
02.06.2010	186	45	146	189	39	141	257,42
02.06.2010	187	75	174	189	39	141	169,88
02.06.2010	188	49	147	189	39	141	230,17
02.06.2010	171	13	99	190	25	122	80,82
02.06.2010	183	47	149	190	25	122	318,31
02.06.2010	184	34	133	190	25	122	116,52
02.06.2010	185	88	181	190	25	122	208,80
02.06.2010	186	45	146	190	25	122	262,29
02.06.2010	187	75	174	190	25	122	174,54
02.06.2010	188	49	147	190	25	122	235,10
02.06.2010	189	39	141	190	25	122	5,55
02.06.2010	171	13	99	192	101	192	67,10
02.06.2010	183	47	149	192	101	192	394,10
02.06.2010	184	34	133	192	101	192	33,85
02.06.2010	185	88	181	192	101	192	299,21
02.06.2010	186	45	146	192	101	192	117,58

02.06.2010	187	75	174	192	101	192	28,83
02.06.2010	188	49	147	192	101	192	92,41
02.06.2010	189	39	141	192	101	192	143,41
02.06.2010	190	25	122	192	101	192	147,84
02.06.2010	171	13	99	183	47	149	357,13
02.06.2010	171	13	99	184	34	133	40,80
02.06.2010	183	47	149	184	34	133	357,21
02.06.2010	171	13	99	185	88	181	249,78
02.06.2010	183	47	149	185	88	181	114,39
02.06.2010	184	34	133	185	88	181	256,17
02.06.2010	171	13	99	186	45	146	108,32
02.06.2010	183	47	149	186	45	146	426,12
02.06.2010	184	34	133	186	45	146	79,22
02.06.2010	185	88	181	186	45	146	330,80
02.06.2010	171	13	99	187	75	174	119,03
02.06.2010	183	47	149	187	75	174	441,48
02.06.2010	184	34	133	187	75	174	92,75
02.06.2010	185	88	181	187	75	174	345,76
02.06.2010	186	45	146	187	75	174	15,40
02.06.2010	171	13	99	188	49	147	134,39
02.06.2010	183	47	149	188	49	147	454,73
02.06.2010	184	34	133	188	49	147	108,04
02.06.2010	185	88	181	188	49	147	360,12
02.06.2010	186	45	146	188	49	147	29,39
02.06.2010	187	75	174	188	49	147	15,47
02.06.2010	171	13	99	189	39	141	78,87
02.06.2010	183	47	149	189	39	141	320,12
02.06.2010	184	34	133	189	39	141	114,15
02.06.2010	185	88	181	189	39	141	206,38
02.06.2010	186	45	146	189	39	141	187,18
02.06.2010	187	75	174	189	39	141	197,72
02.06.2010	188	49	147	189	39	141	213,00
02.06.2010	171	13	99	190	25	122	73,57

02.06.2010	183	47	149	190	25	122	321,70
02.06.2010	184	34	133	190	25	122	108,88
02.06.2010	185	88	181	190	25	122	208,23
02.06.2010	186	45	146	190	25	122	181,88
02.06.2010	187	75	174	190	25	122	192,46
02.06.2010	188	49	147	190	25	122	207,74
02.06.2010	189	39	141	190	25	122	5,32
02.06.2010	171	13	99	192	101	192	60,63
02.06.2010	183	47	149	192	101	192	381,21
02.06.2010	184	34	133	192	101	192	27,55
02.06.2010	185	88	181	192	101	192	282,17
02.06.2010	186	45	146	192	101	192	51,69
02.06.2010	187	75	174	192	101	192	65,25
02.06.2010	188	49	147	192	101	192	80,50
02.06.2010	189	39	141	192	101	192	138,37
02.06.2010	190	25	122	192	101	192	133,05
02.06.2010	171	13	99	183	47	149	366,27
02.06.2010	171	13	99	184	34	133	43,28
02.06.2010	183	47	149	184	34	133	366,76
02.06.2010	171	13	99	185	88	181	295,33
02.06.2010	183	47	149	185	88	181	73,39
02.06.2010	184	34	133	185	88	181	298,90
02.06.2010	171	13	99	186	45	146	186,12
02.06.2010	183	47	149	186	45	146	502,16
02.06.2010	184	34	133	186	45	146	155,73
02.06.2010	185	88	181	186	45	146	440,40
02.06.2010	171	13	99	188	49	147	133,57
02.06.2010	183	47	149	188	49	147	457,76
02.06.2010	184	34	133	188	49	147	103,43
02.06.2010	185	88	181	188	49	147	393,76
02.06.2010	186	45	146	188	49	147	52,76
02.06.2010	171	13	99	189	39	141	60,37
02.06.2010	183	47	149	189	39	141	340,69

02.06.2010	184	34	133	189	39	141	99,71
02.06.2010	185	88	181	189	39	141	267,58
02.06.2010	186	45	146	189	39	141	246,02
02.06.2010	188	49	147	189	39	141	193,66
02.06.2010	171	13	99	190	25	122	60,43
02.06.2010	183	47	149	190	25	122	340,77
02.06.2010	184	34	133	190	25	122	99,79
02.06.2010	185	88	181	190	25	122	267,66
02.06.2010	186	45	146	190	25	122	246,07
02.06.2010	188	49	147	190	25	122	193,71
02.06.2010	189	39	141	190	25	122	0,13
02.06.2010	171	13	99	192	101	192	74,25
02.06.2010	183	47	149	192	101	192	391,95
02.06.2010	184	34	133	192	101	192	35,49
02.06.2010	185	88	181	192	101	192	326,19
02.06.2010	186	45	146	192	101	192	121,16
02.06.2010	188	49	147	192	101	192	69,69
02.06.2010	189	39	141	192	101	192	133,54
02.06.2010	190	25	122	192	101	192	133,61
02.06.2010	171	13	99	183	47	149	351,49
02.06.2010	171	13	99	184	34	133	52,67
02.06.2010	183	47	149	184	34	133	372,05
02.06.2010	171	13	99	185	88	181	234,34
02.06.2010	183	47	149	185	88	181	124,35
02.06.2010	184	34	133	185	88	181	262,77
02.06.2010	171	13	99	186	45	146	192,41
02.06.2010	183	47	149	186	45	146	498,30
02.06.2010	184	34	133	186	45	146	144,64
02.06.2010	185	88	181	186	45	146	399,69
02.06.2010	171	13	99	187	75	174	103,72
02.06.2010	183	47	149	187	75	174	419,36
02.06.2010	184	34	133	187	75	174	54,89
02.06.2010	185	88	181	187	75	174	314,44

02.06.2010	186	45	146	187	75	174	89,79
02.06.2010	171	13	99	188	49	147	139,44
02.06.2010	183	47	149	188	49	147	453,17
02.06.2010	184	34	133	188	49	147	91,95
02.06.2010	185	88	181	188	49	147	350,44
02.06.2010	186	45	146	188	49	147	53,03
02.06.2010	187	75	174	188	49	147	37,10
02.06.2010	171	13	99	189	39	141	67,96
02.06.2010	183	47	149	189	39	141	329,26
02.06.2010	184	34	133	189	39	141	120,50
02.06.2010	185	88	181	189	39	141	205,68
02.06.2010	186	45	146	189	39	141	258,42
02.06.2010	187	75	174	189	39	141	171,02
02.06.2010	188	49	147	189	39	141	205,82
02.06.2010	171	13	99	190	25	122	64,33
02.06.2010	183	47	149	190	25	122	331,78
02.06.2010	184	34	133	190	25	122	116,93
02.06.2010	185	88	181	190	25	122	208,36
02.06.2010	186	45	146	190	25	122	254,49
02.06.2010	187	75	174	190	25	122	167,20
02.06.2010	188	49	147	190	25	122	201,92
02.06.2010	189	39	141	190	25	122	4,03
02.06.2010	171	13	99	192	101	192	79,65
02.06.2010	183	47	149	192	101	192	393,38
02.06.2010	184	34	133	192	101	192	28,21
02.06.2010	185	88	181	192	101	192	287,23
02.06.2010	186	45	146	192	101	192	116,77
02.06.2010	187	75	174	192	101	192	27,50
02.06.2010	188	49	147	192	101	192	64,49
02.06.2010	189	39	141	192	101	192	147,60
02.06.2010	190	25	122	192	101	192	143,92
02.06.2010	171	13	99	183	47	149	343,54
02.06.2010	171	13	99	184	34	133	59,98

02.06.2010	183	47	149	184	34	133	366,55
02.06.2010	171	13	99	185	88	181	222,40
02.06.2010	183	47	149	185	88	181	126,18
02.06.2010	184	34	133	185	88	181	253,92
02.06.2010	171	13	99	186	45	146	184,39
02.06.2010	183	47	149	186	45	146	491,76
02.06.2010	184	34	133	186	45	146	133,84
02.06.2010	185	88	181	186	45	146	385,36
02.06.2010	171	13	99	187	75	174	105,72
02.06.2010	183	47	149	187	75	174	418,58
02.06.2010	184	34	133	187	75	174	54,39
02.06.2010	185	88	181	187	75	174	307,93
02.06.2010	186	45	146	187	75	174	79,95
02.06.2010	171	13	99	188	49	147	120,30
02.06.2010	183	47	149	188	49	147	432,10
02.06.2010	184	34	133	188	49	147	69,17
02.06.2010	185	88	181	188	49	147	322,33
02.06.2010	186	45	146	188	49	147	64,99
02.06.2010	187	75	174	188	49	147	14,96
02.06.2010	171	13	99	189	39	141	66,22
02.06.2010	183	47	149	189	39	141	315,27
02.06.2010	184	34	133	189	39	141	125,47
02.06.2010	185	88	181	189	39	141	189,30
02.06.2010	186	45	146	189	39	141	249,01
02.06.2010	187	75	174	189	39	141	171,45
02.06.2010	188	49	147	189	39	141	185,79
02.06.2010	171	13	99	190	25	122	73,72
02.06.2010	183	47	149	190	25	122	305,56
02.06.2010	184	34	133	190	25	122	132,00
02.06.2010	185	88	181	190	25	122	179,48
02.06.2010	186	45	146	190	25	122	257,51
02.06.2010	187	75	174	190	25	122	179,39
02.06.2010	188	49	147	190	25	122	193,88

02.06.2010	189	39	141	190	25	122	10,53
02.06.2010	171	13	99	192	101	192	84,91
02.06.2010	183	47	149	192	101	192	394,04
02.06.2010	184	34	133	192	101	192	29,69
02.06.2010	185	88	181	192	101	192	282,99
02.06.2010	186	45	146	192	101	192	104,16
02.06.2010	187	75	174	192	101	192	24,94
02.06.2010	188	49	147	192	101	192	39,54
02.06.2010	189	39	141	192	101	192	151,13
02.06.2010	190	25	122	192	101	192	158,45
03.06.2010	171	13	99	183	47	149	317,78
03.06.2010	171	13	99	184	34	133	48,99
03.06.2010	183	47	149	184	34	133	331,45
03.06.2010	171	13	99	185	88	181	218,77
03.06.2010	183	47	149	185	88	181	99,63
03.06.2010	184	34	133	185	88	181	232,02
03.06.2010	171	13	99	186	45	146	193,47
03.06.2010	183	47	149	186	45	146	477,92
03.06.2010	184	34	133	186	45	146	155,91
03.06.2010	185	88	181	186	45	146	380,47
03.06.2010	171	13	99	187	75	174	113,76
03.06.2010	183	47	149	187	75	174	404,99
03.06.2010	184	34	133	187	75	174	76,63
03.06.2010	185	88	181	187	75	174	306,17
03.06.2010	186	45	146	187	75	174	80,13
03.06.2010	171	13	99	188	49	147	139,32
03.06.2010	183	47	149	188	49	147	433,97
03.06.2010	184	34	133	188	49	147	105,20
03.06.2010	185	88	181	188	49	147	335,18
03.06.2010	186	45	146	188	49	147	54,86
03.06.2010	187	75	174	188	49	147	29,01
03.06.2010	171	13	99	189	39	141	68,45
03.06.2010	183	47	149	189	39	141	279,04

03.06.2010	184	34	133	189	39	141	114,12
03.06.2010	185	88	181	189	39	141	185,46
03.06.2010	186	45	146	189	39	141	261,51
03.06.2010	187	75	174	189	39	141	182,10
03.06.2010	188	49	147	189	39	141	207,01
03.06.2010	171	13	99	190	25	122	67,36
03.06.2010	183	47	149	190	25	122	279,22
03.06.2010	184	34	133	190	25	122	112,97
03.06.2010	185	88	181	190	25	122	185,46
03.06.2010	186	45	146	190	25	122	260,46
03.06.2010	187	75	174	190	25	122	181,03
03.06.2010	188	49	147	190	25	122	205,98
03.06.2010	189	39	141	190	25	122	1,16
03.06.2010	171	13	99	192	101	192	86,89
03.06.2010	183	47	149	192	101	192	374,10
03.06.2010	184	34	133	192	101	192	45,76
03.06.2010	185	88	181	192	101	192	275,14
03.06.2010	186	45	146	192	101	192	110,18
03.06.2010	187	75	174	192	101	192	31,23
03.06.2010	188	49	147	192	101	192	60,18
03.06.2010	189	39	141	192	101	192	155,19
03.06.2010	190	25	122	192	101	192	154,08
03.06.2010	171	13	99	183	47	149	330,61
03.06.2010	171	13	99	184	34	133	64,36
03.06.2010	183	47	149	184	34	133	363,52
03.06.2010	171	13	99	185	88	181	216,29
03.06.2010	183	47	149	185	88	181	117,40
03.06.2010	184	34	133	185	88	181	256,11
03.06.2010	171	13	99	186	45	146	195,73
03.06.2010	183	47	149	186	45	146	487,53
03.06.2010	184	34	133	186	45	146	136,00
03.06.2010	185	88	181	186	45	146	387,03
03.06.2010	171	13	99	187	75	174	114,82

03.06.2010	183	47	149	187	75	174	414,79
03.06.2010	184	34	133	187	75	174	55,04
03.06.2010	185	88	181	187	75	174	309,81
03.06.2010	186	45	146	187	75	174	81,38
03.06.2010	171	13	99	188	49	147	187,59
03.06.2010	183	47	149	188	49	147	481,45
03.06.2010	184	34	133	188	49	147	128,28
03.06.2010	185	88	181	188	49	147	380,20
03.06.2010	186	45	146	188	49	147	8,41
03.06.2010	187	75	174	188	49	147	73,48
03.06.2010	171	13	99	189	39	141	66,04
03.06.2010	183	47	149	189	39	141	303,14
03.06.2010	184	34	133	189	39	141	130,33
03.06.2010	185	88	181	189	39	141	185,74
03.06.2010	186	45	146	189	39	141	260,26
03.06.2010	187	75	174	189	39	141	180,11
03.06.2010	188	49	147	189	39	141	252,00
03.06.2010	171	13	99	190	25	122	66,25
03.06.2010	183	47	149	190	25	122	302,95
03.06.2010	184	34	133	190	25	122	130,53
03.06.2010	185	88	181	190	25	122	185,56
03.06.2010	186	45	146	190	25	122	260,49
03.06.2010	187	75	174	190	25	122	180,33
03.06.2010	188	49	147	190	25	122	252,23
03.06.2010	189	39	141	190	25	122	0,23
03.06.2010	171	13	99	192	101	192	70,68
03.06.2010	183	47	149	192	101	192	367,37
03.06.2010	184	34	133	192	101	192	6,32
03.06.2010	185	88	181	192	101	192	260,70
03.06.2010	186	45	146	192	101	192	130,28
03.06.2010	187	75	174	192	101	192	49,67
03.06.2010	188	49	147	192	101	192	122,63
03.06.2010	189	39	141	192	101	192	136,65

03.06.2010	190	25	122	192	101	192	136,85
03.06.2010	171	13	99	183	47	149	334,68
03.06.2010	171	13	99	184	34	133	44,68
03.06.2010	183	47	149	184	34	133	344,43
03.06.2010	171	13	99	185	88	181	220,17
03.06.2010	183	47	149	185	88	181	118,05
03.06.2010	184	34	133	185	88	181	235,69
03.06.2010	171	13	99	186	45	146	193,09
03.06.2010	183	47	149	186	45	146	486,84
03.06.2010	184	34	133	186	45	146	157,95
03.06.2010	185	88	181	186	45	146	386,96
03.06.2010	171	13	99	187	75	174	114,52
03.06.2010	183	47	149	187	75	174	417,09
03.06.2010	184	34	133	187	75	174	79,43
03.06.2010	185	88	181	187	75	174	312,48
03.06.2010	186	45	146	187	75	174	79,00
03.06.2010	171	13	99	188	49	147	123,74
03.06.2010	183	47	149	188	49	147	424,50
03.06.2010	184	34	133	188	49	147	88,36
03.06.2010	185	88	181	188	49	147	320,64
03.06.2010	186	45	146	188	49	147	69,84
03.06.2010	187	75	174	188	49	147	9,22
03.06.2010	171	13	99	189	39	141	64,57
03.06.2010	183	47	149	189	39	141	310,73
03.06.2010	184	34	133	189	39	141	107,94
03.06.2010	185	88	181	189	39	141	192,69
03.06.2010	186	45	146	189	39	141	255,85
03.06.2010	187	75	174	189	39	141	178,10
03.06.2010	188	49	147	189	39	141	187,28
03.06.2010	171	13	99	190	25	122	64,08
03.06.2010	183	47	149	190	25	122	310,86
03.06.2010	184	34	133	190	25	122	107,45
03.06.2010	185	88	181	190	25	122	192,83

03.06.2010	186	45	146	190	25	122	255,36
03.06.2010	187	75	174	190	25	122	177,61
03.06.2010	188	49	147	190	25	122	186,79
03.06.2010	189	39	141	190	25	122	0,49
03.06.2010	171	13	99	192	101	192	83,15
03.06.2010	183	47	149	192	101	192	382,20
03.06.2010	184	34	133	192	101	192	44,06
03.06.2010	185	88	181	192	101	192	276,63
03.06.2010	186	45	146	192	101	192	113,93
03.06.2010	187	75	174	192	101	192	36,00
03.06.2010	188	49	147	192	101	192	44,62
03.06.2010	189	39	141	192	101	192	147,70
03.06.2010	190	25	122	192	101	192	147,20
03.06.2010	171	13	99	183	47	149	355,04
03.06.2010	171	13	99	184	34	133	81,24
03.06.2010	183	47	149	184	34	133	351,47
03.06.2010	171	13	99	185	88	181	220,45
03.06.2010	183	47	149	185	88	181	136,30
03.06.2010	184	34	133	185	88	181	228,63
03.06.2010	171	13	99	186	45	146	216,62
03.06.2010	183	47	149	186	45	146	492,64
03.06.2010	184	34	133	186	45	146	158,33
03.06.2010	185	88	181	186	45	146	381,10
03.06.2010	171	13	99	187	75	174	171,37
03.06.2010	183	47	149	187	75	174	457,15
03.06.2010	184	34	133	187	75	174	114,97
03.06.2010	185	88	181	187	75	174	341,10
03.06.2010	186	45	146	187	75	174	45,34
03.06.2010	171	13	99	188	49	147	131,48
03.06.2010	183	47	149	188	49	147	417,20
03.06.2010	184	34	133	188	49	147	71,27
03.06.2010	185	88	181	188	49	147	298,53
03.06.2010	186	45	146	188	49	147	88,14

03.06.2010	187	75	174	188	49	147	43,79
03.06.2010	171	13	99	189	39	141	42,09
03.06.2010	183	47	149	189	39	141	320,90
03.06.2010	184	34	133	189	39	141	105,95
03.06.2010	185	88	181	189	39	141	184,92
03.06.2010	186	45	146	189	39	141	254,17
03.06.2010	187	75	174	189	39	141	208,84
03.06.2010	188	49	147	189	39	141	167,02
03.06.2010	171	13	99	190	25	122	51,43
03.06.2010	183	47	149	190	25	122	312,44
03.06.2010	184	34	133	190	25	122	111,61
03.06.2010	185	88	181	190	25	122	176,32
03.06.2010	186	45	146	190	25	122	261,91
03.06.2010	187	75	174	190	25	122	216,63
03.06.2010	188	49	147	190	25	122	174,44
03.06.2010	189	39	141	190	25	122	9,50
03.06.2010	171	13	99	192	101	192	102,80
03.06.2010	183	47	149	192	101	192	376,76
03.06.2010	184	34	133	192	101	192	30,20
03.06.2010	185	88	181	192	101	192	256,80
03.06.2010	186	45	146	192	101	192	128,16
03.06.2010	187	75	174	192	101	192	85,15
03.06.2010	188	49	147	192	101	192	41,79
03.06.2010	189	39	141	192	101	192	132,90
03.06.2010	190	25	122	192	101	192	139,32
03.06.2010	171	13	99	183	47	149	355,59
03.06.2010	171	13	99	184	34	133	78,13
03.06.2010	183	47	149	184	34	133	348,86
03.06.2010	171	13	99	185	88	181	227,60
03.06.2010	183	47	149	185	88	181	129,77
03.06.2010	184	34	133	185	88	181	231,55
03.06.2010	171	13	99	186	45	146	216,13
03.06.2010	183	47	149	186	45	146	493,56

03.06.2010	184	34	133	186	45	146	162,47
03.06.2010	185	88	181	186	45	146	387,39
03.06.2010	171	13	99	187	75	174	129,74
03.06.2010	183	47	149	187	75	174	416,51
03.06.2010	184	34	133	187	75	174	73,78
03.06.2010	185	88	181	187	75	174	303,51
03.06.2010	186	45	146	187	75	174	89,62
03.06.2010	171	13	99	188	49	147	139,81
03.06.2010	183	47	149	188	49	147	423,30
03.06.2010	184	34	133	188	49	147	82,93
03.06.2010	185	88	181	188	49	147	311,54
03.06.2010	186	45	146	188	49	147	79,88
03.06.2010	187	75	174	188	49	147	10,11
03.06.2010	171	13	99	189	39	141	50,66
03.06.2010	183	47	149	189	39	141	315,09
03.06.2010	184	34	133	189	39	141	108,55
03.06.2010	185	88	181	189	39	141	185,51
03.06.2010	186	45	146	189	39	141	261,83
03.06.2010	187	75	174	189	39	141	173,11
03.06.2010	188	49	147	189	39	141	183,20
03.06.2010	171	13	99	190	25	122	51,59
03.06.2010	183	47	149	190	25	122	314,07
03.06.2010	184	34	133	190	25	122	108,94
03.06.2010	185	88	181	190	25	122	184,48
03.06.2010	186	45	146	190	25	122	262,51
03.06.2010	187	75	174	190	25	122	173,74
03.06.2010	188	49	147	190	25	122	183,82
03.06.2010	189	39	141	190	25	122	1,05
03.06.2010	171	13	99	192	101	192	97,94
03.06.2010	183	47	149	192	101	192	372,94
03.06.2010	184	34	133	192	101	192	28,84
03.06.2010	185	88	181	192	101	192	258,26
03.06.2010	186	45	146	192	101	192	133,65

03.06.2010	187	75	174	192	101	192	45,50
03.06.2010	188	49	147	192	101	192	54,30
03.06.2010	189	39	141	192	101	192	134,61
03.06.2010	190	25	122	192	101	192	135,09
03.06.2010	171	13	99	183	47	149	340,34
03.06.2010	171	13	99	184	34	133	109,10
03.06.2010	183	47	149	184	34	133	360,51
03.06.2010	171	13	99	185	88	181	202,33
03.06.2010	183	47	149	185	88	181	138,43
03.06.2010	184	34	133	185	88	181	236,56
03.06.2010	171	13	99	186	45	146	241,99
03.06.2010	183	47	149	186	45	146	496,16
03.06.2010	184	34	133	186	45	146	149,23
03.06.2010	185	88	181	186	45	146	381,74
03.06.2010	171	13	99	187	75	174	160,53
03.06.2010	183	47	149	187	75	174	420,51
03.06.2010	184	34	133	187	75	174	64,74
03.06.2010	185	88	181	187	75	174	300,22
03.06.2010	186	45	146	187	75	174	84,97
03.06.2010	171	13	99	188	49	147	135,19
03.06.2010	183	47	149	188	49	147	394,82
03.06.2010	184	34	133	188	49	147	36,36
03.06.2010	185	88	181	188	49	147	272,60
03.06.2010	186	45	146	188	49	147	113,45
03.06.2010	187	75	174	188	49	147	28,55
03.06.2010	171	13	99	189	39	141	25,28
03.06.2010	183	47	149	189	39	141	316,48
03.06.2010	184	34	133	189	39	141	120,24
03.06.2010	185	88	181	189	39	141	178,19
03.06.2010	186	45	146	189	39	141	260,31
03.06.2010	187	75	174	189	39	141	177,03
03.06.2010	188	49	147	189	39	141	150,22
03.06.2010	171	13	99	190	25	122	24,68

03.06.2010	183	47	149	190	25	122	317,12
03.06.2010	184	34	133	190	25	122	120,09
03.06.2010	185	88	181	190	25	122	178,83
03.06.2010	186	45	146	190	25	122	260,00
03.06.2010	187	75	174	190	25	122	176,76
03.06.2010	188	49	147	190	25	122	149,98
03.06.2010	189	39	141	190	25	122	0,64
03.06.2010	171	13	99	192	101	192	122,11
03.06.2010	183	47	149	192	101	192	384,01
03.06.2010	184	34	133	192	101	192	23,77
03.06.2010	185	88	181	192	101	192	260,29
03.06.2010	186	45	146	192	101	192	127,22
03.06.2010	187	75	174	192	101	192	42,25
03.06.2010	188	49	147	192	101	192	13,88
03.06.2010	189	39	141	192	101	192	136,59
03.06.2010	190	25	122	192	101	192	136,36
03.06.2010	171	13	99	183	47	149	344,78
03.06.2010	171	13	99	184	34	133	100,81
03.06.2010	183	47	149	184	34	133	356,76
03.06.2010	171	13	99	185	88	181	210,46
03.06.2010	183	47	149	185	88	181	134,55
03.06.2010	184	34	133	185	88	181	233,77
03.06.2010	171	13	99	186	45	146	247,55
03.06.2010	183	47	149	186	45	146	502,07
03.06.2010	184	34	133	186	45	146	163,47
03.06.2010	185	88	181	186	45	146	390,22
03.06.2010	171	13	99	187	75	174	190,70
03.06.2010	183	47	149	187	75	174	451,90
03.06.2010	184	34	133	187	75	174	105,92
03.06.2010	185	88	181	187	75	174	335,80
03.06.2010	186	45	146	187	75	174	58,01
03.06.2010	171	13	99	188	49	147	145,41
03.06.2010	183	47	149	188	49	147	408,63

03.06.2010	184	34	133	188	49	147	57,25
03.06.2010	185	88	181	188	49	147	289,22
03.06.2010	186	45	146	188	49	147	106,69
03.06.2010	187	75	174	188	49	147	48,81
03.06.2010	171	13	99	189	39	141	20,91
03.06.2010	183	47	149	189	39	141	325,35
03.06.2010	184	34	133	189	39	141	109,62
03.06.2010	185	88	181	189	39	141	190,86
03.06.2010	186	45	146	189	39	141	262,99
03.06.2010	187	75	174	189	39	141	205,52
03.06.2010	188	49	147	189	39	141	158,79
03.06.2010	171	13	99	190	25	122	21,39
03.06.2010	183	47	149	190	25	122	324,56
03.06.2010	184	34	133	190	25	122	109,00
03.06.2010	185	88	181	190	25	122	190,08
03.06.2010	186	45	146	190	25	122	262,69
03.06.2010	187	75	174	190	25	122	205,19
03.06.2010	188	49	147	190	25	122	158,38
03.06.2010	189	39	141	190	25	122	1,08
03.06.2010	171	13	99	192	101	192	122,62
03.06.2010	183	47	149	192	101	192	389,63
03.06.2010	184	34	133	192	101	192	34,29
03.06.2010	185	88	181	192	101	192	267,87
03.06.2010	186	45	146	192	101	192	130,73
03.06.2010	187	75	174	192	101	192	72,77
03.06.2010	188	49	147	192	101	192	24,11
03.06.2010	189	39	141	192	101	192	135,24
03.06.2010	190	25	122	192	101	192	134,79
03.06.2010	171	13	99	183	47	149	351,45
03.06.2010	171	13	99	184	34	133	102,05
03.06.2010	183	47	149	184	34	133	360,28
03.06.2010	171	13	99	185	88	181	216,59
03.06.2010	183	47	149	185	88	181	135,15

03.06.2010	184	34	133	185	88	181	237,07
03.06.2010	171	13	99	186	45	146	237,46
03.06.2010	183	47	149	186	45	146	499,70
03.06.2010	184	34	133	186	45	146	154,97
03.06.2010	185	88	181	186	45	146	386,38
03.06.2010	171	13	99	187	75	174	228,50
03.06.2010	183	47	149	187	75	174	491,88
03.06.2010	184	34	133	187	75	174	145,99
03.06.2010	185	88	181	187	75	174	377,90
03.06.2010	186	45	146	187	75	174	9,09
03.06.2010	171	13	99	188	49	147	140,30
03.06.2010	183	47	149	188	49	147	405,99
03.06.2010	184	34	133	188	49	147	50,67
03.06.2010	185	88	181	188	49	147	286,05
03.06.2010	186	45	146	188	49	147	104,44
03.06.2010	187	75	174	188	49	147	95,43
03.06.2010	171	13	99	189	39	141	27,76
03.06.2010	183	47	149	189	39	141	324,61
03.06.2010	184	34	133	189	39	141	110,73
03.06.2010	185	88	181	189	39	141	189,57
03.06.2010	186	45	146	189	39	141	255,83
03.06.2010	187	75	174	189	39	141	246,77
03.06.2010	188	49	147	189	39	141	154,86
03.06.2010	171	13	99	190	25	122	31,60
03.06.2010	183	47	149	190	25	122	321,74
03.06.2010	184	34	133	190	25	122	114,71
03.06.2010	185	88	181	190	25	122	186,64
03.06.2010	186	45	146	190	25	122	260,30
03.06.2010	187	75	174	190	25	122	251,24
03.06.2010	188	49	147	190	25	122	159,18
03.06.2010	189	39	141	190	25	122	4,56
03.06.2010	171	13	99	192	101	192	109,81
03.06.2010	183	47	149	192	101	192	379,42

03.06.2010	184	34	133	192	101	192	19,23
03.06.2010	185	88	181	192	101	192	256,23
03.06.2010	186	45	146	192	101	192	137,78
03.06.2010	187	75	174	192	101	192	128,74
03.06.2010	188	49	147	192	101	192	33,52
03.06.2010	189	39	141	192	101	192	122,31
03.06.2010	190	25	122	192	101	192	126,55
04.06.2010	171	13	99	183	47	149	347,95
04.06.2010	171	13	99	184	34	133	100,72
04.06.2010	183	47	149	184	34	133	358,41
04.06.2010	171	13	99	185	88	181	217,43
04.06.2010	183	47	149	185	88	181	130,83
04.06.2010	184	34	133	185	88	181	239,03
04.06.2010	171	13	99	186	45	146	241,56
04.06.2010	183	47	149	186	45	146	501,17
04.06.2010	184	34	133	186	45	146	159,19
04.06.2010	185	88	181	186	45	146	391,87
04.06.2010	171	13	99	187	75	174	230,57
04.06.2010	183	47	149	187	75	174	493,07
04.06.2010	184	34	133	187	75	174	148,92
04.06.2010	185	88	181	187	75	174	382,67
04.06.2010	186	45	146	187	75	174	11,01
04.06.2010	171	13	99	188	49	147	133,08
04.06.2010	183	47	149	188	49	147	389,97
04.06.2010	184	34	133	188	49	147	38,68
04.06.2010	185	88	181	188	49	147	274,17
04.06.2010	186	45	146	188	49	147	120,76
04.06.2010	187	75	174	188	49	147	110,64
04.06.2010	171	13	99	189	39	141	22,32
04.06.2010	183	47	149	189	39	141	326,89
04.06.2010	184	34	133	189	39	141	109,16
04.06.2010	185	88	181	189	39	141	196,17
04.06.2010	186	45	146	189	39	141	257,56

04.06.2010	187	75	174	189	39	141	246,63
04.06.2010	188	49	147	189	39	141	144,57
04.06.2010	171	13	99	190	25	122	22,55
04.06.2010	183	47	149	190	25	122	327,22
04.06.2010	184	34	133	190	25	122	110,54
04.06.2010	185	88	181	190	25	122	196,47
04.06.2010	186	45	146	190	25	122	258,69
04.06.2010	187	75	174	190	25	122	247,76
04.06.2010	188	49	147	190	25	122	145,88
04.06.2010	189	39	141	190	25	122	1,47
04.06.2010	171	13	99	192	101	192	116,90
04.06.2010	183	47	149	192	101	192	383,17
04.06.2010	184	34	133	192	101	192	26,05
04.06.2010	185	88	181	192	101	192	264,84
04.06.2010	186	45	146	192	101	192	133,90
04.06.2010	187	75	174	192	101	192	123,47
04.06.2010	188	49	147	192	101	192	16,19
04.06.2010	189	39	141	192	101	192	128,66
04.06.2010	190	25	122	192	101	192	129,95
04.06.2010	171	13	99	183	47	149	258,37
04.06.2010	171	13	99	184	34	133	165,22
04.06.2010	183	47	149	184	34	133	362,90
04.06.2010	171	13	99	185	88	181	119,26
04.06.2010	183	47	149	185	88	181	140,20
04.06.2010	184	34	133	185	88	181	235,15
04.06.2010	171	13	99	186	45	146	324,29
04.06.2010	183	47	149	186	45	146	504,30
04.06.2010	184	34	133	186	45	146	159,62
04.06.2010	185	88	181	186	45	146	387,98
04.06.2010	171	13	99	187	75	174	231,86
04.06.2010	183	47	149	187	75	174	425,08
04.06.2010	184	34	133	187	75	174	68,01
04.06.2010	185	88	181	187	75	174	301,44

04.06.2010	186	45	146	187	75	174	92,49
04.06.2010	171	13	99	188	49	147	217,01
04.06.2010	183	47	149	188	49	147	411,18
04.06.2010	184	34	133	188	49	147	52,95
04.06.2010	185	88	181	188	49	147	286,70
04.06.2010	186	45	146	188	49	147	107,27
04.06.2010	187	75	174	188	49	147	15,06
04.06.2010	171	13	99	189	39	141	109,85
04.06.2010	183	47	149	189	39	141	358,71
04.06.2010	184	34	133	189	39	141	98,63
04.06.2010	185	88	181	189	39	141	218,96
04.06.2010	186	45	146	189	39	141	238,31
04.06.2010	187	75	174	189	39	141	149,30
04.06.2010	188	49	147	189	39	141	136,75
04.06.2010	171	13	99	190	25	122	80,60
04.06.2010	183	47	149	190	25	122	331,51
04.06.2010	184	34	133	190	25	122	110,02
04.06.2010	185	88	181	190	25	122	191,42
04.06.2010	186	45	146	190	25	122	259,64
04.06.2010	187	75	174	190	25	122	168,47
04.06.2010	188	49	147	190	25	122	154,82
04.06.2010	189	39	141	190	25	122	29,25
04.06.2010	171	13	99	192	101	192	189,49
04.06.2010	183	47	149	192	101	192	387,30
04.06.2010	184	34	133	192	101	192	25,71
04.06.2010	185	88	181	192	101	192	260,65
04.06.2010	186	45	146	192	101	192	134,81
04.06.2010	187	75	174	192	101	192	42,61
04.06.2010	188	49	147	192	101	192	27,60
04.06.2010	189	39	141	192	101	192	113,45
04.06.2010	190	25	122	192	101	192	129,25
04.06.2010	171	13	99	183	47	149	261,41
04.06.2010	171	13	99	184	34	133	158,96

04.06.2010	183	47	149	184	34	133	359,96
04.06.2010	171	13	99	185	88	181	122,96
04.06.2010	183	47	149	185	88	181	139,10
04.06.2010	184	34	133	185	88	181	233,71
04.06.2010	171	13	99	186	45	146	322,14
04.06.2010	183	47	149	186	45	146	500,08
04.06.2010	184	34	133	186	45	146	163,36
04.06.2010	185	88	181	186	45	146	387,57
04.06.2010	171	13	99	187	75	174	215,83
04.06.2010	183	47	149	187	75	174	411,47
04.06.2010	184	34	133	187	75	174	57,79
04.06.2010	185	88	181	187	75	174	289,37
04.06.2010	186	45	146	187	75	174	106,46
04.06.2010	171	13	99	188	49	147	227,42
04.06.2010	183	47	149	188	49	147	422,72
04.06.2010	184	34	133	188	49	147	69,67
04.06.2010	185	88	181	188	49	147	301,16
04.06.2010	186	45	146	188	49	147	95,18
04.06.2010	187	75	174	188	49	147	11,92
04.06.2010	171	13	99	189	39	141	76,00
04.06.2010	183	47	149	189	39	141	330,73
04.06.2010	184	34	133	189	39	141	108,32
04.06.2010	185	88	181	189	39	141	191,82
04.06.2010	186	45	146	189	39	141	263,52
04.06.2010	187	75	174	189	39	141	158,39
04.06.2010	188	49	147	189	39	141	168,86
04.06.2010	171	13	99	190	25	122	75,68
04.06.2010	183	47	149	190	25	122	330,51
04.06.2010	184	34	133	190	25	122	108,66
04.06.2010	185	88	181	190	25	122	191,59
04.06.2010	186	45	146	190	25	122	263,90
04.06.2010	187	75	174	190	25	122	158,76
04.06.2010	188	49	147	190	25	122	169,23

04.06.2010	189	39	141	190	25	122	0,38
04.06.2010	171	13	99	192	101	192	185,70
04.06.2010	183	47	149	192	101	192	386,81
04.06.2010	184	34	133	192	101	192	28,38
04.06.2010	185	88	181	192	101	192	261,84
04.06.2010	186	45	146	192	101	192	136,54
04.06.2010	187	75	174	192	101	192	30,13
04.06.2010	188	49	147	192	101	192	41,80
04.06.2010	189	39	141	192	101	192	129,60
04.06.2010	190	25	122	192	101	192	129,96
09.06.2010	171	13	99	183	47	149	123,03
09.06.2010	171	13	99	184	34	133	256,47
09.06.2010	183	47	149	184	34	133	361,34
09.06.2010	171	13	99	185	88	181	13,52
09.06.2010	183	47	149	185	88	181	123,15
09.06.2010	184	34	133	185	88	181	248,87
09.06.2010	171	13	99	186	45	146	399,07
09.06.2010	183	47	149	186	45	146	494,14
09.06.2010	184	34	133	186	45	146	147,29
09.06.2010	185	88	181	186	45	146	390,14
09.06.2010	171	13	99	187	75	174	332,48
09.06.2010	183	47	149	187	75	174	432,50
09.06.2010	184	34	133	187	75	174	77,81
09.06.2010	185	88	181	187	75	174	324,19
09.06.2010	186	45	146	187	75	174	69,88
09.06.2010	171	13	99	188	49	147	248,02
09.06.2010	183	47	149	188	49	147	365,75
09.06.2010	184	34	133	188	49	147	77,29
09.06.2010	185	88	181	188	49	147	244,19
09.06.2010	186	45	146	188	49	147	200,27
09.06.2010	187	75	174	188	49	147	133,70
09.06.2010	171	13	99	189	39	141	206,16
09.06.2010	183	47	149	189	39	141	327,08

09.06.2010	184	34	133	189	39	141	110,53
09.06.2010	185	88	181	189	39	141	204,06
09.06.2010	186	45	146	189	39	141	247,36
09.06.2010	187	75	174	189	39	141	178,85
09.06.2010	188	49	147	189	39	141	50,57
09.06.2010	171	13	99	190	25	122	203,52
09.06.2010	183	47	149	190	25	122	324,59
09.06.2010	184	34	133	190	25	122	112,74
09.06.2010	185	88	181	190	25	122	201,53
09.06.2010	186	45	146	190	25	122	250,15
09.06.2010	187	75	174	190	25	122	181,54
09.06.2010	188	49	147	190	25	122	53,64
09.06.2010	189	39	141	190	25	122	3,09
09.06.2010	171	13	99	183	47	149	111,92
09.06.2010	171	13	99	184	34	133	260,35
09.06.2010	183	47	149	184	34	133	352,11
09.06.2010	171	13	99	185	88	181	7,92
09.06.2010	183	47	149	185	88	181	112,37
09.06.2010	184	34	133	185	88	181	255,04
09.06.2010	171	13	99	186	45	146	399,13
09.06.2010	183	47	149	186	45	146	481,30
09.06.2010	184	34	133	186	45	146	143,52
09.06.2010	185	88	181	186	45	146	393,16
09.06.2010	171	13	99	187	75	174	333,77
09.06.2010	183	47	149	187	75	174	421,05
09.06.2010	184	34	133	187	75	174	74,99
09.06.2010	185	88	181	187	75	174	328,15
09.06.2010	186	45	146	187	75	174	69,13
09.06.2010	171	13	99	188	49	147	258,12
09.06.2010	183	47	149	188	49	147	361,96
09.06.2010	184	34	133	188	49	147	69,08
09.06.2010	185	88	181	188	49	147	254,57
09.06.2010	186	45	146	188	49	147	186,10

09.06.2010	187	75	174	188	49	147	120,06
09.06.2010	171	13	99	189	39	141	210,42
09.06.2010	183	47	149	189	39	141	319,05
09.06.2010	184	34	133	189	39	141	110,27
09.06.2010	185	88	181	189	39	141	208,04
09.06.2010	186	45	146	189	39	141	243,37
09.06.2010	187	75	174	189	39	141	175,41
09.06.2010	188	49	147	189	39	141	59,96
09.06.2010	171	13	99	190	25	122	208,07
09.06.2010	183	47	149	190	25	122	316,82
09.06.2010	184	34	133	190	25	122	111,83
09.06.2010	185	88	181	190	25	122	205,74
09.06.2010	186	45	146	190	25	122	245,49
09.06.2010	187	75	174	190	25	122	177,45
09.06.2010	188	49	147	190	25	122	62,38
09.06.2010	189	39	141	190	25	122	2,55
09.06.2010	171	13	99	183	47	149	114,68
09.06.2010	171	13	99	184	34	133	279,71
09.06.2010	183	47	149	184	34	133	373,81
09.06.2010	171	13	99	185	88	181	6,42
09.06.2010	183	47	149	185	88	181	115,31
09.06.2010	184	34	133	185	88	181	275,20
09.06.2010	171	13	99	186	45	146	413,61
09.06.2010	183	47	149	186	45	146	497,73
09.06.2010	184	34	133	186	45	146	139,99
09.06.2010	185	88	181	186	45	146	408,57
09.06.2010	171	13	99	187	75	174	342,79
09.06.2010	183	47	149	187	75	174	433,11
09.06.2010	184	34	133	187	75	174	64,53
09.06.2010	185	88	181	187	75	174	338,07
09.06.2010	186	45	146	187	75	174	76,42
09.06.2010	171	13	99	188	49	147	252,55
09.06.2010	183	47	149	188	49	147	361,21

09.06.2010	184	34	133	188	49	147	90,37
09.06.2010	185	88	181	188	49	147	249,76
09.06.2010	186	45	146	188	49	147	215,33
09.06.2010	187	75	174	188	49	147	140,84
09.06.2010	171	13	99	189	39	141	210,91
09.06.2010	183	47	149	189	39	141	322,51
09.06.2010	184	34	133	189	39	141	124,12
09.06.2010	185	88	181	189	39	141	208,75
09.06.2010	186	45	146	189	39	141	258,27
09.06.2010	187	75	174	189	39	141	182,30
09.06.2010	188	49	147	189	39	141	47,93
09.06.2010	171	13	99	190	25	122	210,82
09.06.2010	183	47	149	190	25	122	322,61
09.06.2010	184	34	133	190	25	122	126,02
09.06.2010	185	88	181	190	25	122	208,71
09.06.2010	186	45	146	190	25	122	259,99
09.06.2010	187	75	174	190	25	122	184,06
09.06.2010	188	49	147	190	25	122	49,11
09.06.2010	189	39	141	190	25	122	1,99
09.06.2010	171	13	99	183	47	149	124,31
09.06.2010	171	13	99	184	34	133	259,82
09.06.2010	183	47	149	184	34	133	360,91
09.06.2010	171	13	99	185	88	181	7,83
09.06.2010	183	47	149	185	88	181	126,95
09.06.2010	184	34	133	185	88	181	253,00
09.06.2010	171	13	99	186	45	146	387,61
09.06.2010	183	47	149	186	45	146	480,86
09.06.2010	184	34	133	186	45	146	129,90
09.06.2010	185	88	181	186	45	146	380,52
09.06.2010	171	13	99	187	75	174	314,22
09.06.2010	183	47	149	187	75	174	410,42
09.06.2010	184	34	133	187	75	174	56,50
09.06.2010	185	88	181	187	75	174	307,19

09.06.2010	186	45	146	187	75	174	73,73
09.06.2010	171	13	99	188	49	147	252,50
09.06.2010	183	47	149	188	49	147	368,50
09.06.2010	184	34	133	188	49	147	77,23
09.06.2010	185	88	181	188	49	147	247,16
09.06.2010	186	45	146	188	49	147	178,47
09.06.2010	187	75	174	188	49	147	117,02
09.06.2010	171	13	99	189	39	141	174,88
09.06.2010	183	47	149	189	39	141	298,00
09.06.2010	184	34	133	189	39	141	146,53
09.06.2010	185	88	181	189	39	141	171,29
09.06.2010	186	45	146	189	39	141	268,68
09.06.2010	187	75	174	189	39	141	199,87
09.06.2010	188	49	147	189	39	141	95,60
09.06.2010	171	13	99	190	25	122	190,64
09.06.2010	183	47	149	190	25	122	312,79
09.06.2010	184	34	133	190	25	122	131,78
09.06.2010	185	88	181	190	25	122	186,64
09.06.2010	186	45	146	190	25	122	251,42
09.06.2010	187	75	174	190	25	122	183,74
09.06.2010	188	49	147	190	25	122	76,84
09.06.2010	189	39	141	190	25	122	19,02
09.06.2010	171	13	99	183	47	149	122,86
09.06.2010	171	13	99	184	34	133	255,18
09.06.2010	183	47	149	184	34	133	358,58
09.06.2010	171	13	99	185	88	181	4,63
09.06.2010	183	47	149	185	88	181	123,31
09.06.2010	184	34	133	185	88	181	251,98
09.06.2010	171	13	99	186	45	146	384,38
09.06.2010	183	47	149	186	45	146	477,90
09.06.2010	184	34	133	186	45	146	134,04
09.06.2010	185	88	181	186	45	146	380,81
09.06.2010	171	13	99	187	75	174	330,70

09.06.2010	183	47	149	187	75	174	426,38
09.06.2010	184	34	133	187	75	174	80,69
09.06.2010	185	88	181	187	75	174	327,18
09.06.2010	186	45	146	187	75	174	53,99
09.06.2010	171	13	99	188	49	147	251,04
09.06.2010	183	47	149	188	49	147	367,01
09.06.2010	184	34	133	188	49	147	70,77
09.06.2010	185	88	181	188	49	147	248,89
09.06.2010	186	45	146	188	49	147	180,48
09.06.2010	187	75	174	188	49	147	134,39
09.06.2010	171	13	99	189	39	141	173,22
09.06.2010	183	47	149	189	39	141	295,25
09.06.2010	184	34	133	189	39	141	138,54
09.06.2010	185	88	181	189	39	141	172,21
09.06.2010	186	45	146	189	39	141	268,12
09.06.2010	187	75	174	189	39	141	217,19
09.06.2010	188	49	147	189	39	141	94,49
09.06.2010	171	13	99	190	25	122	188,90
09.06.2010	183	47	149	190	25	122	309,92
09.06.2010	184	34	133	190	25	122	121,33
09.06.2010	185	88	181	190	25	122	187,57
09.06.2010	186	45	146	190	25	122	249,10
09.06.2010	187	75	174	190	25	122	198,80
09.06.2010	188	49	147	190	25	122	74,27
09.06.2010	189	39	141	190	25	122	20,23
09.06.2010	171	13	99	183	47	149	128,80
09.06.2010	171	13	99	184	34	133	261,38
09.06.2010	183	47	149	184	34	133	369,17
09.06.2010	171	13	99	185	88	181	9,99
09.06.2010	183	47	149	185	88	181	135,74
09.06.2010	184	34	133	185	88	181	251,48
09.06.2010	171	13	99	186	45	146	412,18
09.06.2010	183	47	149	186	45	146	505,33

09.06.2010	184	34	133	186	45	146	159,90
09.06.2010	185	88	181	186	45	146	402,20
09.06.2010	171	13	99	187	75	174	333,76
09.06.2010	183	47	149	187	75	174	434,12
09.06.2010	184	34	133	187	75	174	76,89
09.06.2010	185	88	181	187	75	174	323,78
09.06.2010	186	45	146	187	75	174	83,12
09.06.2010	171	13	99	188	49	147	252,62
09.06.2010	183	47	149	188	49	147	373,20
09.06.2010	184	34	133	188	49	147	67,87
09.06.2010	185	88	181	188	49	147	243,41
09.06.2010	186	45	146	188	49	147	209,71
09.06.2010	187	75	174	188	49	147	129,78
09.06.2010	171	13	99	189	39	141	177,32
09.06.2010	183	47	149	189	39	141	305,04
09.06.2010	184	34	133	189	39	141	141,09
09.06.2010	185	88	181	189	39	141	169,54
09.06.2010	186	45	146	189	39	141	298,41
09.06.2010	187	75	174	189	39	141	215,73
09.06.2010	188	49	147	189	39	141	94,41
09.06.2010	171	13	99	190	25	122	203,00
09.06.2010	183	47	149	190	25	122	328,70
09.06.2010	184	34	133	190	25	122	112,72
09.06.2010	185	88	181	190	25	122	194,55
09.06.2010	186	45	146	190	25	122	267,36
09.06.2010	187	75	174	190	25	122	185,25
09.06.2010	188	49	147	190	25	122	61,28
09.06.2010	189	39	141	190	25	122	33,20
10.06.2010	171	13	99	183	47	149	119,23
10.06.2010	171	13	99	184	34	133	264,49
10.06.2010	183	47	149	184	34	133	366,05
10.06.2010	171	13	99	185	88	181	8,99
10.06.2010	183	47	149	185	88	181	128,21

10.06.2010	184	34	133	185	88	181	257,18
10.06.2010	171	13	99	186	45	146	409,07
10.06.2010	183	47	149	186	45	146	497,65
10.06.2010	184	34	133	186	45	146	154,54
10.06.2010	185	88	181	186	45	146	402,71
10.06.2010	171	13	99	187	75	174	334,75
10.06.2010	183	47	149	187	75	174	429,53
10.06.2010	184	34	133	187	75	174	75,53
10.06.2010	185	88	181	187	75	174	327,95
10.06.2010	186	45	146	187	75	174	79,07
10.06.2010	171	13	99	188	49	147	260,87
10.06.2010	183	47	149	188	49	147	373,17
10.06.2010	184	34	133	188	49	147	65,00
10.06.2010	185	88	181	188	49	147	252,49
10.06.2010	186	45	146	188	49	147	199,95
10.06.2010	187	75	174	188	49	147	124,53
10.06.2010	171	13	99	189	39	141	181,13
10.06.2010	183	47	149	189	39	141	299,67
10.06.2010	184	34	133	189	39	141	140,38
10.06.2010	185	88	181	189	39	141	172,17
10.06.2010	186	45	146	189	39	141	292,70
10.06.2010	187	75	174	189	39	141	214,07
10.06.2010	188	49	147	189	39	141	99,45
10.06.2010	171	13	99	190	25	122	199,20
10.06.2010	183	47	149	190	25	122	317,30
10.06.2010	184	34	133	190	25	122	128,36
10.06.2010	185	88	181	190	25	122	190,29
10.06.2010	186	45	146	190	25	122	278,42
10.06.2010	187	75	174	190	25	122	200,36
10.06.2010	188	49	147	190	25	122	82,13
10.06.2010	189	39	141	190	25	122	19,30
10.06.2010	189	39	141	190	25	122	81,52
10.06.2010	171	13	99	183	47	149	129,34

10.06.2010	171	13	99	184	34	133	261,54
10.06.2010	183	47	149	184	34	133	368,29
10.06.2010	171	13	99	185	88	181	2,86
10.06.2010	183	47	149	185	88	181	132,02
10.06.2010	184	34	133	185	88	181	258,88
10.06.2010	171	13	99	186	45	146	400,44
10.06.2010	183	47	149	186	45	146	496,56
10.06.2010	184	34	133	186	45	146	143,18
10.06.2010	185	88	181	186	45	146	397,91
10.06.2010	171	13	99	187	75	174	333,70
10.06.2010	183	47	149	187	75	174	434,40
10.06.2010	184	34	133	187	75	174	74,62
10.06.2010	185	88	181	187	75	174	331,11
10.06.2010	186	45	146	187	75	174	68,57
10.06.2010	171	13	99	188	49	147	254,01
10.06.2010	183	47	149	188	49	147	375,77
10.06.2010	184	34	133	188	49	147	79,07
10.06.2010	185	88	181	188	49	147	251,16
10.06.2010	186	45	146	188	49	147	196,54
10.06.2010	187	75	174	188	49	147	133,71
10.06.2010	171	13	99	189	39	141	184,04
10.06.2010	183	47	149	189	39	141	312,02
10.06.2010	184	34	133	189	39	141	140,19
10.06.2010	185	88	181	189	39	141	181,22
10.06.2010	186	45	146	189	39	141	276,70
10.06.2010	187	75	174	189	39	141	209,95
10.06.2010	188	49	147	189	39	141	86,07
10.06.2010	171	13	99	190	25	122	206,00
10.06.2010	183	47	149	190	25	122	331,30
10.06.2010	184	34	133	190	25	122	109,32
10.06.2010	185	88	181	190	25	122	203,15
10.06.2010	186	45	146	190	25	122	243,48
10.06.2010	187	75	174	190	25	122	177,18

10.06.2010	188	49	147	190	25	122	53,37
10.06.2010	189	39	141	190	25	122	33,56
10.06.2010	171	13	99	183	47	149	122,46
10.06.2010	171	13	99	184	34	133	259,52
10.06.2010	183	47	149	184	34	133	362,85
10.06.2010	171	13	99	185	88	181	6,14
10.06.2010	183	47	149	185	88	181	117,51
10.06.2010	184	34	133	185	88	181	265,65
10.06.2010	171	13	99	186	45	146	389,93
10.06.2010	183	47	149	186	45	146	485,94
10.06.2010	184	34	133	186	45	146	132,82
10.06.2010	185	88	181	186	45	146	396,01
10.06.2010	171	13	99	187	75	174	328,13
10.06.2010	183	47	149	187	75	174	426,92
10.06.2010	184	34	133	187	75	174	70,33
10.06.2010	185	88	181	187	75	174	334,23
10.06.2010	186	45	146	187	75	174	62,51
10.06.2010	171	13	99	188	49	147	246,02
10.06.2010	183	47	149	188	49	147	363,33
10.06.2010	184	34	133	188	49	147	83,41
10.06.2010	185	88	181	188	49	147	251,95
10.06.2010	186	45	146	188	49	147	190,62
10.06.2010	187	75	174	188	49	147	135,14
10.06.2010	171	13	99	189	39	141	169,38
10.06.2010	183	47	149	189	39	141	291,65
10.06.2010	184	34	133	189	39	141	154,56
10.06.2010	185	88	181	189	39	141	174,66
10.06.2010	186	45	146	189	39	141	280,42
10.06.2010	187	75	174	189	39	141	220,49
10.06.2010	188	49	147	189	39	141	95,61
10.06.2010	171	13	99	190	25	122	192,27
10.06.2010	183	47	149	190	25	122	313,41
10.06.2010	184	34	133	190	25	122	128,38

10.06.2010	185	88	181	190	25	122	197,86
10.06.2010	186	45	146	190	25	122	251,16
10.06.2010	187	75	174	190	25	122	192,09
10.06.2010	188	49	147	190	25	122	64,90
10.06.2010	189	39	141	190	25	122	30,71
10.06.2010	171	13	99	183	47	149	118,83
10.06.2010	171	13	99	184	34	133	260,52
10.06.2010	183	47	149	184	34	133	359,18
10.06.2010	171	13	99	185	88	181	0,37
10.06.2010	183	47	149	185	88	181	118,53
10.06.2010	184	34	133	185	88	181	260,62
10.06.2010	171	13	99	186	45	146	407,85
10.06.2010	183	47	149	186	45	146	496,66
10.06.2010	184	34	133	186	45	146	151,68
10.06.2010	185	88	181	186	45	146	407,92
10.06.2010	171	13	99	187	75	174	331,10
10.06.2010	183	47	149	187	75	174	426,31
10.06.2010	184	34	133	187	75	174	71,28
10.06.2010	185	88	181	187	75	174	331,19
10.06.2010	186	45	146	187	75	174	81,77
10.06.2010	171	13	99	188	49	147	251,20
10.06.2010	183	47	149	188	49	147	363,94
10.06.2010	184	34	133	188	49	147	81,78
10.06.2010	185	88	181	188	49	147	251,40
10.06.2010	186	45	146	188	49	147	207,75
10.06.2010	187	75	174	188	49	147	128,96
10.06.2010	171	13	99	189	39	141	164,46
10.06.2010	183	47	149	189	39	141	282,57
10.06.2010	184	34	133	189	39	141	149,96
10.06.2010	185	88	181	189	39	141	164,73
10.06.2010	186	45	146	189	39	141	297,68
10.06.2010	187	75	174	189	39	141	215,98
10.06.2010	188	49	147	189	39	141	99,94

10.06.2010	171	13	99	190	25	122	192,83
10.06.2010	183	47	149	190	25	122	310,26
10.06.2010	184	34	133	190	25	122	132,33
10.06.2010	185	88	181	190	25	122	193,09
10.06.2010	186	45	146	190	25	122	275,39
10.06.2010	187	75	174	190	25	122	194,27
10.06.2010	188	49	147	190	25	122	72,28
10.06.2010	189	39	141	190	25	122	29,78
10.06.2010	171	13	99	183	47	149	118,53
10.06.2010	171	13	99	184	34	133	266,75
10.06.2010	183	47	149	184	34	133	363,71
10.06.2010	171	13	99	185	88	181	1,60
10.06.2010	183	47	149	185	88	181	116,93
10.06.2010	184	34	133	185	88	181	267,90
10.06.2010	171	13	99	186	45	146	383,09
10.06.2010	183	47	149	186	45	146	473,89
10.06.2010	184	34	133	186	45	146	117,99
10.06.2010	185	88	181	186	45	146	384,17
10.06.2010	171	13	99	187	75	174	330,97
10.06.2010	183	47	149	187	75	174	424,00
10.06.2010	184	34	133	187	75	174	65,41
10.06.2010	185	88	181	187	75	174	332,07
10.06.2010	186	45	146	187	75	174	52,59
10.06.2010	171	13	99	188	49	147	259,35
10.06.2010	183	47	149	188	49	147	368,02
10.06.2010	184	34	133	188	49	147	62,56
10.06.2010	185	88	181	188	49	147	260,73
10.06.2010	186	45	146	188	49	147	155,18
10.06.2010	187	75	174	188	49	147	108,36
10.06.2010	171	13	99	189	39	141	164,04
10.06.2010	183	47	149	189	39	141	280,99
10.06.2010	184	34	133	189	39	141	148,30
10.06.2010	185	88	181	189	39	141	165,58

10.06.2010	186	45	146	189	39	141	261,61
10.06.2010	187	75	174	189	39	141	210,68
10.06.2010	188	49	147	189	39	141	111,39
10.06.2010	171	13	99	190	25	122	195,37
10.06.2010	183	47	149	190	25	122	311,95
10.06.2010	184	34	133	190	25	122	133,87
10.06.2010	185	88	181	190	25	122	196,91
10.06.2010	186	45	146	190	25	122	241,51
10.06.2010	187	75	174	190	25	122	192,34
10.06.2010	188	49	147	190	25	122	87,30
10.06.2010	189	39	141	190	25	122	31,46
10.06.2010	171	13	99	183	47	149	122,87
10.06.2010	171	13	99	184	34	133	261,17
10.06.2010	183	47	149	184	34	133	363,70
10.06.2010	171	13	99	185	88	181	6,35
10.06.2010	183	47	149	185	88	181	118,44
10.06.2010	184	34	133	185	88	181	267,51
10.06.2010	171	13	99	187	75	174	326,29
10.06.2010	183	47	149	187	75	174	424,62
10.06.2010	184	34	133	187	75	174	66,47
10.06.2010	185	88	181	187	75	174	332,64
10.06.2010	171	13	99	188	49	147	246,35
10.06.2010	183	47	149	188	49	147	363,50
10.06.2010	184	34	133	188	49	147	84,26
10.06.2010	185	88	181	188	49	147	252,20
10.06.2010	187	75	174	188	49	147	132,35
10.06.2010	171	13	99	189	39	141	164,72
10.06.2010	183	47	149	189	39	141	286,92
10.06.2010	184	34	133	189	39	141	150,55
10.06.2010	185	88	181	189	39	141	169,85
10.06.2010	187	75	174	189	39	141	213,36
10.06.2010	188	49	147	189	39	141	94,44
10.06.2010	171	13	99	190	25	122	199,06

10.06.2010	183	47	149	190	25	122	319,48
10.06.2010	184	34	133	190	25	122	118,91
10.06.2010	185	88	181	190	25	122	204,58
10.06.2010	187	75	174	190	25	122	178,13
10.06.2010	188	49	147	190	25	122	54,23
10.06.2010	189	39	141	190	25	122	40,28
10.06.2010	171	13	99	183	47	149	121,57
10.06.2010	171	13	99	184	34	133	261,62
10.06.2010	183	47	149	184	34	133	360,97
10.06.2010	171	13	99	185	88	181	1,90
10.06.2010	183	47	149	185	88	181	119,69
10.06.2010	184	34	133	185	88	181	263,17
10.06.2010	171	13	99	186	45	146	406,63
10.06.2010	183	47	149	186	45	146	494,52
10.06.2010	184	34	133	186	45	146	150,48
10.06.2010	185	88	181	186	45	146	408,03
10.06.2010	171	13	99	187	75	174	330,54
10.06.2010	183	47	149	187	75	174	425,11
10.06.2010	184	34	133	187	75	174	70,49
10.06.2010	185	88	181	187	75	174	332,04
10.06.2010	186	45	146	187	75	174	80,67
10.06.2010	171	13	99	188	49	147	249,08
10.06.2010	183	47	149	188	49	147	363,25
10.06.2010	184	34	133	188	49	147	80,00
10.06.2010	185	88	181	188	49	147	250,89
10.06.2010	186	45	146	188	49	147	208,83
10.06.2010	187	75	174	188	49	147	131,34
10.06.2010	171	13	99	189	39	141	164,15
10.06.2010	183	47	149	189	39	141	283,70
10.06.2010	184	34	133	189	39	141	140,99
10.06.2010	185	88	181	189	39	141	166,04
10.06.2010	186	45	146	189	39	141	289,21
10.06.2010	187	75	174	189	39	141	208,60

10.06.2010	188	49	147	189	39	141	92,86
10.06.2010	171	13	99	190	25	122	201,61
10.06.2010	183	47	149	190	25	122	319,75
10.06.2010	184	34	133	190	25	122	116,13
10.06.2010	185	88	181	190	25	122	203,48
10.06.2010	186	45	146	190	25	122	258,94
10.06.2010	187	75	174	190	25	122	179,15
10.06.2010	188	49	147	190	25	122	55,18
10.06.2010	189	39	141	190	25	122	39,03
10.06.2010	171	13	99	183	47	149	123,31
10.06.2010	171	13	99	184	34	133	254,60
10.06.2010	183	47	149	184	34	133	356,60
10.06.2010	171	13	99	185	88	181	4,47
10.06.2010	183	47	149	185	88	181	118,98
10.06.2010	184	34	133	185	88	181	258,57
10.06.2010	171	13	99	186	45	146	409,30
10.06.2010	183	47	149	186	45	146	499,46
10.06.2010	184	34	133	186	45	146	160,03
10.06.2010	185	88	181	186	45	146	412,98
10.06.2010	171	13	99	187	75	174	343,37
10.06.2010	183	47	149	187	75	174	439,15
10.06.2010	184	34	133	187	75	174	90,90
10.06.2010	185	88	181	187	75	174	347,20
10.06.2010	186	45	146	187	75	174	69,76
10.06.2010	171	13	99	188	49	147	253,53
10.06.2010	183	47	149	188	49	147	369,34
10.06.2010	184	34	133	188	49	147	74,83
10.06.2010	185	88	181	188	49	147	257,92
10.06.2010	186	45	146	188	49	147	206,19
10.06.2010	187	75	174	188	49	147	139,05
10.06.2010	171	13	99	189	39	141	158,46
10.06.2010	183	47	149	189	39	141	280,14
10.06.2010	184	34	133	189	39	141	138,89

10.06.2010	185	88	181	189	39	141	162,93
10.06.2010	186	45	146	189	39	141	296,52
10.06.2010	187	75	174	189	39	141	226,77
10.06.2010	188	49	147	189	39	141	103,84
10.06.2010	171	13	99	190	25	122	202,47
10.06.2010	183	47	149	190	25	122	322,60
10.06.2010	184	34	133	190	25	122	110,65
10.06.2010	185	88	181	190	25	122	206,93
10.06.2010	186	45	146	190	25	122	260,98
10.06.2010	187	75	174	190	25	122	191,91
10.06.2010	188	49	147	190	25	122	59,82
10.06.2010	189	39	141	190	25	122	45,81
11.06.2010	171	13	99	183	47	149	118,77
11.06.2010	171	13	99	184	34	133	265,18
11.06.2010	183	47	149	184	34	133	366,21
11.06.2010	171	13	99	185	88	181	7,70
11.06.2010	183	47	149	185	88	181	114,76
11.06.2010	184	34	133	185	88	181	272,47
11.06.2010	171	13	99	186	45	146	407,84
11.06.2010	183	47	149	186	45	146	498,94
11.06.2010	184	34	133	186	45	146	148,31
11.06.2010	185	88	181	186	45	146	415,37
11.06.2010	171	13	99	187	75	174	343,34
11.06.2010	183	47	149	187	75	174	439,53
11.06.2010	184	34	133	187	75	174	80,23
11.06.2010	185	88	181	187	75	174	350,76
11.06.2010	186	45	146	187	75	174	68,72
11.06.2010	171	13	99	188	49	147	256,83
11.06.2010	183	47	149	188	49	147	369,97
11.06.2010	184	34	133	188	49	147	75,65
11.06.2010	185	88	181	188	49	147	263,12
11.06.2010	186	45	146	188	49	147	200,95
11.06.2010	187	75	174	188	49	147	134,64

11.06.2010	171	13	99	189	39	141	131,46
11.06.2010	183	47	149	189	39	141	250,19
11.06.2010	184	34	133	189	39	141	176,21
11.06.2010	185	88	181	189	39	141	136,03
11.06.2010	186	45	146	189	39	141	324,07
11.06.2010	187	75	174	189	39	141	255,43
11.06.2010	188	49	147	189	39	141	139,59
11.06.2010	171	13	99	190	25	122	206,81
11.06.2010	183	47	149	190	25	122	323,65
11.06.2010	184	34	133	190	25	122	116,87
11.06.2010	185	88	181	190	25	122	212,41
11.06.2010	186	45	146	190	25	122	257,12
11.06.2010	187	75	174	190	25	122	189,07
11.06.2010	188	49	147	190	25	122	60,35
11.06.2010	171	13	99	183	47	149	111,42
11.06.2010	171	13	99	184	34	133	263,09
11.06.2010	183	47	149	184	34	133	355,34
11.06.2010	171	13	99	185	88	181	2,89
11.06.2010	183	47	149	185	88	181	108,56
11.06.2010	184	34	133	185	88	181	265,54
11.06.2010	171	13	99	186	45	146	414,70
11.06.2010	183	47	149	186	45	146	496,78
11.06.2010	184	34	133	186	45	146	157,05
11.06.2010	185	88	181	186	45	146	416,94
11.06.2010	171	13	99	187	75	174	334,87
11.06.2010	183	47	149	187	75	174	422,25
11.06.2010	184	34	133	187	75	174	74,05
11.06.2010	185	88	181	187	75	174	337,22
11.06.2010	186	45	146	187	75	174	83,20
11.06.2010	171	13	99	188	49	147	248,28
11.06.2010	183	47	149	188	49	147	354,19
11.06.2010	184	34	133	188	49	147	84,22
11.06.2010	185	88	181	188	49	147	251,09

11.06.2010	186	45	146	188	49	147	219,53
11.06.2010	187	75	174	188	49	147	140,71
11.06.2010	171	13	99	189	39	141	163,26
11.06.2010	183	47	149	189	39	141	273,38
11.06.2010	184	34	133	189	39	141	144,20
11.06.2010	185	88	181	189	39	141	166,14
11.06.2010	186	45	146	189	39	141	298,91
11.06.2010	187	75	174	189	39	141	216,02
11.06.2010	188	49	147	189	39	141	92,54
11.06.2010	171	13	99	190	25	122	200,74
11.06.2010	183	47	149	190	25	122	309,68
11.06.2010	184	34	133	190	25	122	118,49
11.06.2010	185	88	181	190	25	122	203,60
11.06.2010	186	45	146	190	25	122	267,88
11.06.2010	187	75	174	190	25	122	186,17
11.06.2010	188	49	147	190	25	122	54,15
11.06.2010	189	39	141	190	25	122	39,31
11.06.2010	171	13	99	183	47	149	131,66
11.06.2010	171	13	99	184	34	133	265,34
11.06.2010	183	47	149	184	34	133	372,86
11.06.2010	171	13	99	185	88	181	1,45
11.06.2010	183	47	149	185	88	181	130,21
11.06.2010	184	34	133	185	88	181	266,37
11.06.2010	171	13	99	186	45	146	414,22
11.06.2010	183	47	149	186	45	146	508,77
11.06.2010	184	34	133	186	45	146	154,82
11.06.2010	185	88	181	186	45	146	415,11
11.06.2010	171	13	99	187	75	174	340,68
11.06.2010	183	47	149	187	75	174	442,09
11.06.2010	184	34	133	187	75	174	77,50
11.06.2010	185	88	181	187	75	174	341,65
11.06.2010	186	45	146	187	75	174	77,81
11.06.2010	171	13	99	188	49	147	258,65

11.06.2010	183	47	149	188	49	147	380,94
11.06.2010	184	34	133	188	49	147	73,85
11.06.2010	185	88	181	188	49	147	259,93
11.06.2010	186	45	146	188	49	147	204,80
11.06.2010	187	75	174	188	49	147	130,16
11.06.2010	171	13	99	189	39	141	133,41
11.06.2010	183	47	149	189	39	141	264,38
11.06.2010	184	34	133	189	39	141	172,78
11.06.2010	185	88	181	189	39	141	134,84
11.06.2010	186	45	146	189	39	141	327,16
11.06.2010	187	75	174	189	39	141	249,38
11.06.2010	188	49	147	189	39	141	138,56
11.06.2010	171	13	99	190	25	122	200,57
11.06.2010	183	47	149	190	25	122	328,52
11.06.2010	184	34	133	190	25	122	119,84
11.06.2010	185	88	181	190	25	122	201,94
11.06.2010	186	45	146	190	25	122	267,97
11.06.2010	187	75	174	190	25	122	190,87
11.06.2010	188	49	147	190	25	122	68,43
11.06.2010	189	39	141	190	25	122	72,15
14.06.2010	171	13	99	183	47	149	103,42
14.06.2010	171	13	99	184	34	133	264,02
14.06.2010	183	47	149	184	34	133	349,21
14.06.2010	171	13	99	185	88	181	1,25
14.06.2010	183	47	149	185	88	181	104,61
14.06.2010	184	34	133	185	88	181	262,86
14.06.2010	171	13	99	186	45	146	385,12
14.06.2010	183	47	149	186	45	146	464,17
14.06.2010	184	34	133	186	45	146	123,58
14.06.2010	185	88	181	186	45	146	384,00
14.06.2010	171	13	99	187	75	174	323,64
14.06.2010	183	47	149	187	75	174	404,26
14.06.2010	184	34	133	187	75	174	62,60

14.06.2010	185	88	181	187	75	174	322,52
14.06.2010	186	45	146	187	75	174	61,62
14.06.2010	171	13	99	188	49	147	248,93
14.06.2010	183	47	149	188	49	147	347,28
14.06.2010	184	34	133	188	49	147	86,25
14.06.2010	185	88	181	188	49	147	247,68
14.06.2010	186	45	146	188	49	147	185,57
14.06.2010	187	75	174	188	49	147	134,83
14.06.2010	171	13	99	189	39	141	170,18
14.06.2010	183	47	149	189	39	141	272,59
14.06.2010	184	34	133	189	39	141	145,22
14.06.2010	185	88	181	189	39	141	168,95
14.06.2010	186	45	146	189	39	141	263,74
14.06.2010	187	75	174	189	39	141	206,01
14.06.2010	188	49	147	189	39	141	88,47
14.06.2010	171	13	99	190	25	122	212,11
14.06.2010	183	47	149	190	25	122	312,53
14.06.2010	184	34	133	190	25	122	109,78
14.06.2010	185	88	181	190	25	122	210,86
14.06.2010	186	45	146	190	25	122	221,51
14.06.2010	187	75	174	190	25	122	166,62
14.06.2010	188	49	147	190	25	122	41,04
14.06.2010	189	39	141	190	25	122	47,49
14.06.2010	171	13	99	192	101	192	16,44
14.06.2010	183	47	149	192	101	192	112,57
14.06.2010	184	34	133	192	101	192	248,57
14.06.2010	185	88	181	192	101	192	15,53
14.06.2010	186	45	146	192	101	192	369,26
14.06.2010	187	75	174	192	101	192	307,84
14.06.2010	188	49	147	192	101	192	236,28
14.06.2010	189	39	141	192	101	192	160,05
14.06.2010	190	25	122	192	101	192	200,40
14.06.2010	171	13	99	183	47	149	110,90

14.06.2010	171	13	99	184	34	133	260,20
14.06.2010	183	47	149	184	34	133	353,96
14.06.2010	171	13	99	185	88	181	1,24
14.06.2010	183	47	149	185	88	181	109,71
14.06.2010	184	34	133	185	88	181	260,91
14.06.2010	171	13	99	186	45	146	417,25
14.06.2010	183	47	149	186	45	146	497,24
14.06.2010	184	34	133	186	45	146	168,41
14.06.2010	185	88	181	186	45	146	417,77
14.06.2010	171	13	99	187	75	174	331,27
14.06.2010	183	47	149	187	75	174	418,34
14.06.2010	184	34	133	187	75	174	76,59
14.06.2010	185	88	181	187	75	174	331,89
14.06.2010	186	45	146	187	75	174	91,94
14.06.2010	171	13	99	188	49	147	250,61
14.06.2010	183	47	149	188	49	147	355,80
14.06.2010	184	34	133	188	49	147	72,47
14.06.2010	185	88	181	188	49	147	251,58
14.06.2010	186	45	146	188	49	147	223,97
14.06.2010	187	75	174	188	49	147	135,33
14.06.2010	171	13	99	189	39	141	164,74
14.06.2010	183	47	149	189	39	141	274,32
14.06.2010	184	34	133	189	39	141	135,56
14.06.2010	185	88	181	189	39	141	165,83
14.06.2010	186	45	146	189	39	141	303,52
14.06.2010	187	75	174	189	39	141	211,60
14.06.2010	188	49	147	189	39	141	93,87
14.06.2010	171	13	99	190	25	122	192,39
14.06.2010	183	47	149	190	25	122	301,70
14.06.2010	184	34	133	190	25	122	121,72
14.06.2010	185	88	181	190	25	122	193,48
14.06.2010	186	45	146	190	25	122	287,03
14.06.2010	187	75	174	190	25	122	195,66

14.06.2010	188	49	147	190	25	122	69,55
14.06.2010	189	39	141	190	25	122	27,76
14.06.2010	171	13	99	192	101	192	14,15
14.06.2010	183	47	149	192	101	192	116,79
14.06.2010	184	34	133	192	101	192	248,09
14.06.2010	185	88	181	192	101	192	14,31
14.06.2010	186	45	146	192	101	192	403,97
14.06.2010	187	75	174	192	101	192	318,48
14.06.2010	188	49	147	192	101	192	241,09
14.06.2010	189	39	141	192	101	192	157,53
14.06.2010	190	25	122	192	101	192	184,95
14.06.2010	171	13	99	183	47	149	104,14
14.06.2010	171	13	99	184	34	133	263,07
14.06.2010	183	47	149	184	34	133	348,84
14.06.2010	171	13	99	185	88	181	2,09
14.06.2010	183	47	149	185	88	181	102,06
14.06.2010	184	34	133	185	88	181	264,73
14.06.2010	171	13	99	186	45	146	419,41
14.06.2010	183	47	149	186	45	146	495,43
14.06.2010	184	34	133	186	45	146	161,85
14.06.2010	185	88	181	186	45	146	420,90
14.06.2010	171	13	99	187	75	174	341,87
14.06.2010	183	47	149	187	75	174	423,01
14.06.2010	184	34	133	187	75	174	80,83
14.06.2010	185	88	181	187	75	174	343,45
14.06.2010	186	45	146	187	75	174	81,47
14.06.2010	171	13	99	188	49	147	244,85
14.06.2010	183	47	149	188	49	147	344,28
14.06.2010	184	34	133	188	49	147	89,71
14.06.2010	185	88	181	188	49	147	246,84
14.06.2010	186	45	146	188	49	147	230,47
14.06.2010	187	75	174	188	49	147	152,17
14.06.2010	171	13	99	189	39	141	165,02

14.06.2010	183	47	149	189	39	141	267,86
14.06.2010	184	34	133	189	39	141	143,19
14.06.2010	185	88	181	189	39	141	167,08
14.06.2010	186	45	146	189	39	141	302,46
14.06.2010	187	75	174	189	39	141	221,11
14.06.2010	188	49	147	189	39	141	85,93
14.06.2010	171	13	99	190	25	122	196,80
14.06.2010	183	47	149	190	25	122	299,45
14.06.2010	184	34	133	190	25	122	130,31
14.06.2010	185	88	181	190	25	122	198,86
14.06.2010	186	45	146	190	25	122	284,08
14.06.2010	187	75	174	190	25	122	203,67
14.06.2010	188	49	147	190	25	122	58,28
14.06.2010	189	39	141	190	25	122	31,81
14.06.2010	171	13	99	192	101	192	15,42
14.06.2010	183	47	149	192	101	192	111,13
14.06.2010	184	34	133	192	101	192	249,33
14.06.2010	185	88	181	192	101	192	16,45
14.06.2010	186	45	146	192	101	192	404,86
14.06.2010	187	75	174	192	101	192	327,70
14.06.2010	188	49	147	192	101	192	234,43
14.06.2010	189	39	141	192	101	192	156,73
14.06.2010	190	25	122	192	101	192	188,32
14.06.2010	171	13	99	183	47	149	125,43
14.06.2010	171	13	99	184	34	133	249,10
14.06.2010	183	47	149	184	34	133	363,74
14.06.2010	171	13	99	185	88	181	20,78
14.06.2010	183	47	149	185	88	181	119,25
14.06.2010	184	34	133	185	88	181	265,61
14.06.2010	171	13	99	186	45	146	394,06
14.06.2010	183	47	149	186	45	146	499,44
14.06.2010	184	34	133	186	45	146	151,80
14.06.2010	185	88	181	186	45	146	412,08

14.06.2010	171	13	99	187	75	174	380,33
14.06.2010	183	47	149	187	75	174	487,17
14.06.2010	184	34	133	187	75	174	136,50
14.06.2010	185	88	181	187	75	174	398,14
14.06.2010	186	45	146	187	75	174	16,12
14.06.2010	171	13	99	188	49	147	240,43
14.06.2010	183	47	149	188	49	147	364,65
14.06.2010	184	34	133	188	49	147	83,02
14.06.2010	185	88	181	188	49	147	251,98
14.06.2010	186	45	146	188	49	147	212,78
14.06.2010	187	75	174	188	49	147	196,66
14.06.2010	171	13	99	189	39	141	128,98
14.06.2010	183	47	149	189	39	141	253,40
14.06.2010	184	34	133	189	39	141	170,52
14.06.2010	185	88	181	189	39	141	134,74
14.06.2010	186	45	146	189	39	141	321,75
14.06.2010	187	75	174	189	39	141	306,11
14.06.2010	188	49	147	189	39	141	127,19
14.06.2010	171	13	99	190	25	122	204,41
14.06.2010	183	47	149	190	25	122	329,69
14.06.2010	184	34	133	190	25	122	109,71
14.06.2010	185	88	181	190	25	122	214,18
14.06.2010	186	45	146	190	25	122	251,70
14.06.2010	187	75	174	190	25	122	235,63
14.06.2010	188	49	147	190	25	122	43,34
14.06.2010	189	39	141	190	25	122	84,93
14.06.2010	171	13	99	192	101	192	21,02
14.06.2010	183	47	149	192	101	192	119,27
14.06.2010	184	34	133	192	101	192	265,76
14.06.2010	185	88	181	192	101	192	0,27
14.06.2010	186	45	146	192	101	192	412,26
14.06.2010	187	75	174	192	101	192	398,31
14.06.2010	188	49	147	192	101	192	252,07

14.06.2010	189	39	141	192	101	192	134,76
14.06.2010	190	25	122	192	101	192	214,24
14.06.2010	171	13	99	183	47	149	122,82
14.06.2010	171	13	99	184	34	133	268,91
14.06.2010	183	47	149	184	34	133	368,47
14.06.2010	171	13	99	185	88	181	1,14
14.06.2010	183	47	149	185	88	181	121,68
14.06.2010	184	34	133	185	88	181	269,74
14.06.2010	171	13	99	186	45	146	417,66
14.06.2010	183	47	149	186	45	146	503,06
14.06.2010	184	34	133	186	45	146	157,38
14.06.2010	185	88	181	186	45	146	418,35
14.06.2010	171	13	99	187	75	174	333,12
14.06.2010	183	47	149	187	75	174	428,00
14.06.2010	184	34	133	187	75	174	65,84
14.06.2010	185	88	181	187	75	174	333,90
14.06.2010	186	45	146	187	75	174	93,13
14.06.2010	171	13	99	188	49	147	251,58
14.06.2010	183	47	149	188	49	147	367,15
14.06.2010	184	34	133	188	49	147	86,95
14.06.2010	185	88	181	188	49	147	252,62
14.06.2010	186	45	146	188	49	147	227,12
14.06.2010	187	75	174	188	49	147	135,90
14.06.2010	171	13	99	189	39	141	137,19
14.06.2010	183	47	149	189	39	141	258,99
14.06.2010	184	34	133	189	39	141	170,54
14.06.2010	185	88	181	189	39	141	138,31
14.06.2010	186	45	146	189	39	141	327,88
14.06.2010	187	75	174	189	39	141	235,42
14.06.2010	188	49	147	189	39	141	122,91
14.06.2010	171	13	99	190	25	122	201,83
14.06.2010	183	47	149	190	25	122	321,14
14.06.2010	184	34	133	190	25	122	122,86

14.06.2010	185	88	181	190	25	122	202,92
14.06.2010	186	45	146	190	25	122	275,57
14.06.2010	187	75	174	190	25	122	182,56
14.06.2010	188	49	147	190	25	122	56,24
14.06.2010	189	39	141	190	25	122	67,96
14.06.2010	171	13	99	192	101	192	1,11
14.06.2010	183	47	149	192	101	192	121,71
14.06.2010	184	34	133	192	101	192	269,74
14.06.2010	185	88	181	192	101	192	0,00
14.06.2010	186	45	146	192	101	192	418,36
14.06.2010	187	75	174	192	101	192	333,90
14.06.2010	188	49	147	192	101	192	252,60
14.06.2010	189	39	141	192	101	192	138,29
14.06.2010	190	25	122	192	101	192	202,89
14.06.2010	171	13	99	183	47	149	121,60
14.06.2010	171	13	99	184	34	133	265,94
14.06.2010	183	47	149	184	34	133	365,57
14.06.2010	171	13	99	185	88	181	2,30
14.06.2010	183	47	149	185	88	181	119,58
14.06.2010	184	34	133	185	88	181	266,72
14.06.2010	171	13	99	186	45	146	419,14
14.06.2010	183	47	149	186	45	146	506,47
14.06.2010	184	34	133	186	45	146	159,64
14.06.2010	185	88	181	186	45	146	419,63
14.06.2010	171	13	99	187	75	174	347,42
14.06.2010	183	47	149	187	75	174	441,50
14.06.2010	184	34	133	187	75	174	83,44
14.06.2010	185	88	181	187	75	174	348,07
14.06.2010	186	45	146	187	75	174	77,29
14.06.2010	171	13	99	188	49	147	251,25
14.06.2010	183	47	149	188	49	147	366,56
14.06.2010	184	34	133	188	49	147	88,33
14.06.2010	185	88	181	188	49	147	252,71

14.06.2010	186	45	146	188	49	147	226,54
14.06.2010	187	75	174	188	49	147	151,23
14.06.2010	171	13	99	189	39	141	127,52
14.06.2010	183	47	149	189	39	141	248,38
14.06.2010	184	34	133	189	39	141	173,60
14.06.2010	185	88	181	189	39	141	129,36
14.06.2010	186	45	146	189	39	141	333,13
14.06.2010	187	75	174	189	39	141	256,28
14.06.2010	188	49	147	189	39	141	130,85
14.06.2010	171	13	99	190	25	122	218,61
14.06.2010	183	47	149	190	25	122	336,60
14.06.2010	184	34	133	190	25	122	112,62
14.06.2010	185	88	181	190	25	122	220,22
14.06.2010	186	45	146	190	25	122	261,46
14.06.2010	187	75	174	190	25	122	184,91
14.06.2010	188	49	147	190	25	122	38,67
14.06.2010	189	39	141	190	25	122	94,46
14.06.2010	171	13	99	192	101	192	2,29
14.06.2010	183	47	149	192	101	192	119,59
14.06.2010	184	34	133	192	101	192	266,72
14.06.2010	185	88	181	192	101	192	0,00
14.06.2010	186	45	146	192	101	192	419,63
14.06.2010	187	75	174	192	101	192	348,07
14.06.2010	188	49	147	192	101	192	252,71
14.06.2010	189	39	141	192	101	192	129,35
14.06.2010	190	25	122	192	101	192	220,22
15.06.2010	171	13	99	183	47	149	1,10
15.06.2010	171	13	99	184	34	133	265,32
15.06.2010	183	47	149	184	34	133	265,85
15.06.2010	171	13	99	185	88	181	1,18
15.06.2010	183	47	149	185	88	181	0,10
15.06.2010	184	34	133	185	88	181	265,91
15.06.2010	171	13	99	186	45	146	412,85

15.06.2010	183	47	149	186	45	146	413,24
15.06.2010	184	34	133	186	45	146	154,09
15.06.2010	185	88	181	186	45	146	413,29
15.06.2010	171	13	99	187	75	174	342,47
15.06.2010	183	47	149	187	75	174	342,94
15.06.2010	184	34	133	187	75	174	79,60
15.06.2010	185	88	181	187	75	174	342,99
15.06.2010	186	45	146	187	75	174	75,07
15.06.2010	171	13	99	188	49	147	247,18
15.06.2010	183	47	149	188	49	147	248,01
15.06.2010	184	34	133	188	49	147	90,06
15.06.2010	185	88	181	188	49	147	248,08
15.06.2010	186	45	146	188	49	147	224,89
15.06.2010	187	75	174	188	49	147	152,30
15.06.2010	171	13	99	189	39	141	137,06
15.06.2010	183	47	149	189	39	141	138,03
15.06.2010	184	34	133	189	39	141	167,15
15.06.2010	185	88	181	189	39	141	138,11
15.06.2010	186	45	146	189	39	141	320,91
15.06.2010	187	75	174	189	39	141	245,93
15.06.2010	188	49	147	189	39	141	117,53
15.06.2010	171	13	99	190	25	122	219,76
15.06.2010	183	47	149	190	25	122	220,63
15.06.2010	184	34	133	190	25	122	107,91
15.06.2010	185	88	181	190	25	122	220,70
15.06.2010	186	45	146	190	25	122	251,66
15.06.2010	187	75	174	190	25	122	177,71
15.06.2010	188	49	147	190	25	122	30,97
15.06.2010	189	39	141	190	25	122	87,32
15.06.2010	171	13	99	192	101	192	1,16
15.06.2010	183	47	149	192	101	192	0,00
15.06.2010	184	34	133	192	101	192	265,88
15.06.2010	185	88	181	192	101	192	0,10

15.06.2010	186	45	146	192	101	192	413,27
15.06.2010	187	75	174	192	101	192	342,97
15.06.2010	188	49	147	192	101	192	248,05
15.06.2010	189	39	141	192	101	192	138,08
15.06.2010	190	25	122	192	101	192	220,67
15.06.2010	171	13	99	183	47	149	131,50
15.06.2010	171	13	99	184	34	133	259,27
15.06.2010	183	47	149	184	34	133	370,01
15.06.2010	171	13	99	185	88	181	4,57
15.06.2010	183	47	149	185	88	181	132,18
15.06.2010	184	34	133	185	88	181	261,73
15.06.2010	171	13	99	186	45	146	415,24
15.06.2010	183	47	149	186	45	146	513,05
15.06.2010	184	34	133	186	45	146	162,41
15.06.2010	185	88	181	186	45	146	418,21
15.06.2010	171	13	99	187	75	174	328,69
15.06.2010	183	47	149	187	75	174	434,61
15.06.2010	184	34	133	187	75	174	70,96
15.06.2010	185	88	181	187	75	174	331,34
15.06.2010	186	45	146	187	75	174	92,41
15.06.2010	171	13	99	188	49	147	252,89
15.06.2010	183	47	149	188	49	147	378,30
15.06.2010	184	34	133	188	49	147	81,41
15.06.2010	185	88	181	188	49	147	254,04
15.06.2010	186	45	146	188	49	147	218,74
15.06.2010	187	75	174	188	49	147	130,06
15.06.2010	171	13	99	189	39	141	133,42
15.06.2010	183	47	149	189	39	141	264,71
15.06.2010	184	34	133	189	39	141	168,88
15.06.2010	185	88	181	189	39	141	133,26
15.06.2010	186	45	146	189	39	141	330,75
15.06.2010	187	75	174	189	39	141	238,42
15.06.2010	188	49	147	189	39	141	131,25

15.06.2010	171	13	99	190	25	122	212,64
15.06.2010	183	47	149	190	25	122	341,25
15.06.2010	184	34	133	190	25	122	109,96
15.06.2010	185	88	181	190	25	122	213,31
15.06.2010	186	45	146	190	25	122	261,79
15.06.2010	187	75	174	190	25	122	170,64
15.06.2010	188	49	147	190	25	122	47,54
15.06.2010	189	39	141	190	25	122	85,28
15.06.2010	171	13	99	192	101	192	5,22
15.06.2010	183	47	149	192	101	192	133,79
15.06.2010	184	34	133	192	101	192	260,65
15.06.2010	185	88	181	192	101	192	1,62
15.06.2010	186	45	146	192	101	192	417,29
15.06.2010	187	75	174	192	101	192	330,31
15.06.2010	188	49	147	192	101	192	252,63
15.06.2010	189	39	141	192	101	192	131,69
15.06.2010	190	25	122	192	101	192	211,83
15.06.2010	171	13	99	183	47	149	125,26
15.06.2010	171	13	99	184	34	133	263,68
15.06.2010	183	47	149	184	34	133	368,01
15.06.2010	171	13	99	185	88	181	0,00
15.06.2010	183	47	149	185	88	181	125,30
15.06.2010	184	34	133	185	88	181	263,65
15.06.2010	171	13	99	186	45	146	407,61
15.06.2010	183	47	149	186	45	146	500,45
15.06.2010	184	34	133	186	45	146	149,81
15.06.2010	185	88	181	186	45	146	407,59
15.06.2010	171	13	99	187	75	174	333,71
15.06.2010	183	47	149	187	75	174	432,84
15.06.2010	184	34	133	187	75	174	72,13
15.06.2010	185	88	181	187	75	174	333,69
15.06.2010	186	45	146	187	75	174	78,12
15.06.2010	171	13	99	188	49	147	256,34

15.06.2010	183	47	149	188	49	147	374,09
15.06.2010	184	34	133	188	49	147	73,81
15.06.2010	185	88	181	188	49	147	256,31
15.06.2010	186	45	146	188	49	147	200,57
15.06.2010	187	75	174	188	49	147	125,98
15.06.2010	171	13	99	189	39	141	131,78
15.06.2010	183	47	149	189	39	141	256,60
15.06.2010	184	34	133	189	39	141	170,66
15.06.2010	185	88	181	189	39	141	131,74
15.06.2010	186	45	146	189	39	141	320,14
15.06.2010	187	75	174	189	39	141	242,05
15.06.2010	188	49	147	189	39	141	136,59
15.06.2010	171	13	99	190	25	122	203,14
15.06.2010	183	47	149	190	25	122	325,76
15.06.2010	184	34	133	190	25	122	118,83
15.06.2010	185	88	181	190	25	122	203,10
15.06.2010	186	45	146	190	25	122	261,15
15.06.2010	187	75	174	190	25	122	184,08
15.06.2010	188	49	147	190	25	122	64,77
15.06.2010	189	39	141	190	25	122	75,14
15.06.2010	171	13	99	192	101	192	6,37
15.06.2010	183	47	149	192	101	192	130,14
15.06.2010	184	34	133	192	101	192	257,30
15.06.2010	185	88	181	192	101	192	6,34
15.06.2010	186	45	146	192	101	192	401,28
15.06.2010	187	75	174	192	101	192	327,35
15.06.2010	188	49	147	192	101	192	250,25
15.06.2010	189	39	141	192	101	192	126,56
15.06.2010	190	25	122	192	101	192	197,43
15.06.2010	171	13	99	183	47	149	118,80
15.06.2010	171	13	99	184	34	133	262,76
15.06.2010	183	47	149	184	34	133	360,54
15.06.2010	171	13	99	185	88	181	7,23

15.06.2010	183	47	149	185	88	181	125,76
15.06.2010	184	34	133	185	88	181	256,23
15.06.2010	171	13	99	186	45	146	413,21
15.06.2010	183	47	149	186	45	146	499,39
15.06.2010	184	34	133	186	45	146	156,50
15.06.2010	185	88	181	186	45	146	407,13
15.06.2010	171	13	99	187	75	174	348,36
15.06.2010	183	47	149	187	75	174	440,18
15.06.2010	184	34	133	187	75	174	87,97
15.06.2010	185	88	181	187	75	174	342,05
15.06.2010	186	45	146	187	75	174	69,23
15.06.2010	171	13	99	188	49	147	251,39
15.06.2010	183	47	149	188	49	147	363,26
15.06.2010	184	34	133	188	49	147	79,33
15.06.2010	185	88	181	188	49	147	244,23
15.06.2010	186	45	146	188	49	147	213,92
15.06.2010	187	75	174	188	49	147	146,60
15.06.2010	171	13	99	189	39	141	169,10
15.06.2010	183	47	149	189	39	141	286,24
15.06.2010	184	34	133	189	39	141	140,77
15.06.2010	185	88	181	189	39	141	161,89
15.06.2010	186	45	146	189	39	141	294,64
15.06.2010	187	75	174	189	39	141	225,43
15.06.2010	188	49	147	189	39	141	91,76
15.06.2010	171	13	99	190	25	122	202,87
15.06.2010	183	47	149	190	25	122	318,45
15.06.2010	184	34	133	190	25	122	115,07
15.06.2010	185	88	181	190	25	122	195,64
15.06.2010	186	45	146	190	25	122	264,26
15.06.2010	187	75	174	190	25	122	195,43
15.06.2010	188	49	147	190	25	122	55,81
15.06.2010	189	39	141	190	25	122	36,52
15.06.2010	171	13	99	192	101	192	1,35

15.06.2010	183	47	149	192	101	192	119,86
15.06.2010	184	34	133	192	101	192	261,41
15.06.2010	185	88	181	192	101	192	6,01
15.06.2010	186	45	146	192	101	192	411,88
15.06.2010	187	75	174	192	101	192	347,02
15.06.2010	188	49	147	192	101	192	250,09
15.06.2010	189	39	141	192	101	192	167,89
15.06.2010	190	25	122	192	101	192	201,62
15.06.2010	171	13	99	183	47	149	116,02
15.06.2010	171	13	99	184	34	133	265,57
15.06.2010	183	47	149	184	34	133	362,09
15.06.2010	171	13	99	185	88	181	2,08
15.06.2010	183	47	149	185	88	181	118,08
15.06.2010	184	34	133	185	88	181	263,82
15.06.2010	171	13	99	186	45	146	402,45
15.06.2010	183	47	149	186	45	146	489,79
15.06.2010	184	34	133	186	45	146	141,39
15.06.2010	185	88	181	186	45	146	400,83
15.06.2010	171	13	99	187	75	174	335,25
15.06.2010	183	47	149	187	75	174	427,57
15.06.2010	184	34	133	187	75	174	71,18
15.06.2010	185	88	181	187	75	174	333,56
15.06.2010	186	45	146	187	75	174	70,68
15.06.2010	171	13	99	188	49	147	260,76
15.06.2010	183	47	149	188	49	147	370,19
15.06.2010	184	34	133	188	49	147	77,30
15.06.2010	185	88	181	188	49	147	258,76
15.06.2010	186	45	146	188	49	147	192,01
15.06.2010	187	75	174	188	49	147	125,44
15.06.2010	171	13	99	189	39	141	166,98
15.06.2010	183	47	149	189	39	141	281,74
15.06.2010	184	34	133	189	39	141	144,68
15.06.2010	185	88	181	189	39	141	164,91

15.06.2010	186	45	146	189	39	141	283,21
15.06.2010	187	75	174	189	39	141	212,70
15.06.2010	188	49	147	189	39	141	103,84
15.06.2010	171	13	99	190	25	122	206,12
15.06.2010	183	47	149	190	25	122	319,72
15.06.2010	184	34	133	190	25	122	119,10
15.06.2010	185	88	181	190	25	122	204,06
15.06.2010	186	45	146	190	25	122	251,58
15.06.2010	187	75	174	190	25	122	182,12
15.06.2010	188	49	147	190	25	122	64,82
15.06.2010	189	39	141	190	25	122	40,87
15.06.2010	171	13	99	192	101	192	18,19
15.06.2010	183	47	149	192	101	192	128,49
15.06.2010	184	34	133	192	101	192	247,66
15.06.2010	185	88	181	192	101	192	16,71
15.06.2010	186	45	146	192	101	192	384,29
15.06.2010	187	75	174	192	101	192	317,20
15.06.2010	188	49	147	192	101	192	244,64
15.06.2010	189	39	141	192	101	192	153,25
15.06.2010	190	25	122	192	101	192	191,55
15.06.2010	171	13	99	183	47	149	117,76
15.06.2010	171	13	99	184	34	133	261,52
15.06.2010	183	47	149	184	34	133	358,64
15.06.2010	171	13	99	185	88	181	6,07
15.06.2010	183	47	149	185	88	181	121,96
15.06.2010	184	34	133	185	88	181	255,48
15.06.2010	171	13	99	186	45	146	415,96
15.06.2010	183	47	149	186	45	146	501,86
15.06.2010	184	34	133	186	45	146	160,03
15.06.2010	185	88	181	186	45	146	409,89
15.06.2010	171	13	99	187	75	174	333,71
15.06.2010	183	47	149	187	75	174	426,04
15.06.2010	184	34	133	187	75	174	73,93

15.06.2010	185	88	181	187	75	174	327,64
15.06.2010	186	45	146	187	75	174	86,65
15.06.2010	171	13	99	188	49	147	248,36
15.06.2010	183	47	149	188	49	147	360,01
15.06.2010	184	34	133	188	49	147	83,94
15.06.2010	185	88	181	188	49	147	242,86
15.06.2010	186	45	146	188	49	147	221,39
15.06.2010	187	75	174	188	49	147	138,40
15.06.2010	171	13	99	189	39	141	165,77
15.06.2010	183	47	149	189	39	141	281,88
15.06.2010	184	34	133	189	39	141	140,51
15.06.2010	185	88	181	189	39	141	160,82
15.06.2010	186	45	146	189	39	141	297,94
15.06.2010	187	75	174	189	39	141	211,41
15.06.2010	188	49	147	189	39	141	89,66
15.06.2010	171	13	99	190	25	122	204,89
15.06.2010	183	47	149	190	25	122	319,72
15.06.2010	184	34	133	190	25	122	115,53
15.06.2010	185	88	181	190	25	122	199,72
15.06.2010	186	45	146	190	25	122	266,66
15.06.2010	187	75	174	190	25	122	181,24
15.06.2010	188	49	147	190	25	122	50,25
15.06.2010	189	39	141	190	25	122	40,67
15.06.2010	171	13	99	192	101	192	31,21
15.06.2010	183	47	149	192	101	192	140,40
15.06.2010	184	34	133	192	101	192	230,60
15.06.2010	185	88	181	192	101	192	25,15
15.06.2010	186	45	146	192	101	192	384,74
15.06.2010	187	75	174	192	101	192	302,59
15.06.2010	188	49	147	192	101	192	220,75
15.06.2010	189	39	141	192	101	192	141,69
15.06.2010	190	25	122	192	101	192	179,34
15.06.2010	171	13	99	183	47	149	128,91

15.06.2010	171	13	99	184	34	133	261,93
15.06.2010	183	47	149	184	34	133	367,50
15.06.2010	171	13	99	185	88	181	8,58
15.06.2010	183	47	149	185	88	181	136,13
15.06.2010	184	34	133	185	88	181	253,45
15.06.2010	171	13	99	186	45	146	418,35
15.06.2010	183	47	149	186	45	146	510,83
15.06.2010	184	34	133	186	45	146	162,41
15.06.2010	185	88	181	186	45	146	410,13
15.06.2010	171	13	99	187	75	174	333,45
15.06.2010	183	47	149	187	75	174	433,35
15.06.2010	184	34	133	187	75	174	73,53
15.06.2010	185	88	181	187	75	174	325,06
15.06.2010	186	45	146	187	75	174	89,32
15.06.2010	171	13	99	188	49	147	248,70
15.06.2010	183	47	149	188	49	147	370,61
15.06.2010	184	34	133	188	49	147	85,87
15.06.2010	185	88	181	188	49	147	240,26
15.06.2010	186	45	146	188	49	147	225,78
15.06.2010	187	75	174	188	49	147	140,53
15.06.2010	171	13	99	189	39	141	136,74
15.06.2010	183	47	149	189	39	141	264,82
15.06.2010	184	34	133	189	39	141	165,91
15.06.2010	185	88	181	189	39	141	128,99
15.06.2010	186	45	146	189	39	141	327,67
15.06.2010	187	75	174	189	39	141	238,35
15.06.2010	188	49	147	189	39	141	121,00
15.06.2010	171	13	99	190	25	122	204,38
15.06.2010	183	47	149	190	25	122	329,79
15.06.2010	184	34	133	190	25	122	116,80
15.06.2010	185	88	181	190	25	122	196,17
15.06.2010	186	45	146	190	25	122	270,70
15.06.2010	187	75	174	190	25	122	182,72

15.06.2010	188	49	147	190	25	122	50,66
15.06.2010	189	39	141	190	25	122	71,60
15.06.2010	171	13	99	192	101	192	8,34
15.06.2010	183	47	149	192	101	192	136,04
15.06.2010	184	34	133	192	101	192	253,73
15.06.2010	185	88	181	192	101	192	0,33
15.06.2010	186	45	146	192	101	192	410,42
15.06.2010	187	75	174	192	101	192	325,35
15.06.2010	188	49	147	192	101	192	240,46
15.06.2010	189	39	141	192	101	192	129,11
15.06.2010	190	25	122	192	101	192	196,34
15.06.2010	171	13	99	183	47	149	125,05
15.06.2010	171	13	99	184	34	133	264,81
15.06.2010	183	47	149	184	34	133	367,55
15.06.2010	171	13	99	185	88	181	7,15
15.06.2010	183	47	149	185	88	181	131,54
15.06.2010	184	34	133	185	88	181	257,95
15.06.2010	171	13	99	186	45	146	413,43
15.06.2010	183	47	149	186	45	146	504,11
15.06.2010	184	34	133	186	45	146	154,53
15.06.2010	185	88	181	186	45	146	406,88
15.06.2010	171	13	99	187	75	174	342,33
15.06.2010	183	47	149	187	75	174	438,63
15.06.2010	184	34	133	187	75	174	80,39
15.06.2010	185	88	181	187	75	174	335,63
15.06.2010	186	45	146	187	75	174	74,29
15.06.2010	171	13	99	188	49	147	253,28
15.06.2010	183	47	149	188	49	147	371,22
15.06.2010	184	34	133	188	49	147	82,70
15.06.2010	185	88	181	188	49	147	246,13
15.06.2010	186	45	146	188	49	147	214,70
15.06.2010	187	75	174	188	49	147	144,35
15.06.2010	171	13	99	189	39	141	137,38

15.06.2010	183	47	149	189	39	141	261,65
15.06.2010	184	34	133	189	39	141	167,35
15.06.2010	185	88	181	189	39	141	130,55
15.06.2010	186	45	146	189	39	141	321,34
15.06.2010	187	75	174	189	39	141	247,06
15.06.2010	188	49	147	189	39	141	125,53
15.06.2010	171	13	99	190	25	122	206,28
15.06.2010	183	47	149	190	25	122	327,94
15.06.2010	184	34	133	190	25	122	116,64
15.06.2010	185	88	181	190	25	122	199,21
15.06.2010	186	45	146	190	25	122	263,15
15.06.2010	187	75	174	190	25	122	190,25
15.06.2010	188	49	147	190	25	122	54,13
15.06.2010	189	39	141	190	25	122	72,90
15.06.2010	171	13	99	192	101	192	7,98
15.06.2010	183	47	149	192	101	192	132,32
15.06.2010	184	34	133	192	101	192	257,18
15.06.2010	185	88	181	192	101	192	0,83
15.06.2010	186	45	146	192	101	192	406,15
15.06.2010	187	75	174	192	101	192	334,88
15.06.2010	188	49	147	192	101	192	245,30
15.06.2010	189	39	141	192	101	192	129,74
15.06.2010	190	25	122	192	101	192	198,38
16.06.2010	171	13	99	183	47	149	127,68
16.06.2010	171	13	99	184	34	133	262,15
16.06.2010	183	47	149	184	34	133	368,07
16.06.2010	171	13	99	185	88	181	5,96
16.06.2010	183	47	149	185	88	181	132,58
16.06.2010	184	34	133	185	88	181	256,21
16.06.2010	171	13	99	186	45	146	410,27
16.06.2010	183	47	149	186	45	146	503,57
16.06.2010	184	34	133	186	45	146	154,60
16.06.2010	185	88	181	186	45	146	404,47

16.06.2010	171	13	99	187	75	174	334,11
16.06.2010	183	47	149	187	75	174	434,29
16.06.2010	184	34	133	187	75	174	74,31
16.06.2010	185	88	181	187	75	174	328,23
16.06.2010	186	45	146	187	75	174	80,76
16.06.2010	171	13	99	188	49	147	249,62
16.06.2010	183	47	149	188	49	147	370,31
16.06.2010	184	34	133	188	49	147	79,84
16.06.2010	185	88	181	188	49	147	243,79
16.06.2010	186	45	146	188	49	147	213,83
16.06.2010	187	75	174	188	49	147	136,20
16.06.2010	171	13	99	189	39	141	134,38
16.06.2010	183	47	149	189	39	141	261,42
16.06.2010	184	34	133	189	39	141	166,50
16.06.2010	185	88	181	189	39	141	129,12
16.06.2010	186	45	146	189	39	141	320,77
16.06.2010	187	75	174	189	39	141	240,06
16.06.2010	188	49	147	189	39	141	125,15
16.06.2010	171	13	99	190	25	122	204,06
16.06.2010	183	47	149	190	25	122	328,62
16.06.2010	184	34	133	190	25	122	115,47
16.06.2010	185	88	181	190	25	122	198,44
16.06.2010	186	45	146	190	25	122	262,68
16.06.2010	187	75	174	190	25	122	182,87
16.06.2010	188	49	147	190	25	122	53,62
16.06.2010	189	39	141	190	25	122	73,53
16.06.2010	171	13	99	192	101	192	6,47
16.06.2010	183	47	149	192	101	192	133,44
16.06.2010	184	34	133	192	101	192	255,85
16.06.2010	185	88	181	192	101	192	0,98
16.06.2010	186	45	146	192	101	192	404,23
16.06.2010	187	75	174	192	101	192	327,92
16.06.2010	188	49	147	192	101	192	243,16

16.06.2010	189	39	141	192	101	192	128,31
16.06.2010	190	25	122	192	101	192	197,72
16.06.2010	171	13	99	183	47	149	111,25
16.06.2010	171	13	99	184	34	133	265,74
16.06.2010	183	47	149	184	34	133	358,91
16.06.2010	171	13	99	185	88	181	6,94
16.06.2010	183	47	149	185	88	181	117,69
16.06.2010	184	34	133	185	88	181	259,09
16.06.2010	171	13	99	186	45	146	416,10
16.06.2010	183	47	149	186	45	146	498,37
16.06.2010	184	34	133	186	45	146	157,43
16.06.2010	185	88	181	186	45	146	409,80
16.06.2010	171	13	99	187	75	174	335,67
16.06.2010	183	47	149	187	75	174	423,41
16.06.2010	184	34	133	187	75	174	73,36
16.06.2010	185	88	181	187	75	174	329,19
16.06.2010	186	45	146	187	75	174	84,14
16.06.2010	171	13	99	188	49	147	253,16
16.06.2010	183	47	149	188	49	147	359,02
16.06.2010	184	34	133	188	49	147	80,99
16.06.2010	185	88	181	188	49	147	246,22
16.06.2010	186	45	146	188	49	147	218,01
16.06.2010	187	75	174	188	49	147	138,49
16.06.2010	171	13	99	189	39	141	134,07
16.06.2010	183	47	149	189	39	141	244,99
16.06.2010	184	34	133	189	39	141	170,16
16.06.2010	185	88	181	189	39	141	127,44
16.06.2010	186	45	146	189	39	141	327,41
16.06.2010	187	75	174	189	39	141	243,27
16.06.2010	188	49	147	189	39	141	128,96
16.06.2010	171	13	99	190	25	122	206,73
16.06.2010	183	47	149	190	25	122	315,29
16.06.2010	184	34	133	190	25	122	112,38

16.06.2010	185	88	181	190	25	122	199,85
16.06.2010	186	45	146	190	25	122	263,30
16.06.2010	187	75	174	190	25	122	180,67
16.06.2010	188	49	147	190	25	122	51,88
16.06.2010	189	39	141	190	25	122	77,86
16.06.2010	171	13	99	192	101	192	5,96
16.06.2010	183	47	149	192	101	192	116,04
16.06.2010	184	34	133	192	101	192	259,78
16.06.2010	185	88	181	192	101	192	1,92
16.06.2010	186	45	146	192	101	192	410,23
16.06.2010	187	75	174	192	101	192	329,75
16.06.2010	188	49	147	192	101	192	247,44
16.06.2010	189	39	141	192	101	192	129,02
16.06.2010	190	25	122	192	101	192	201,21
16.06.2010	171	13	99	183	47	149	121,85
16.06.2010	171	13	99	184	34	133	257,80
16.06.2010	183	47	149	184	34	133	358,58
16.06.2010	171	13	99	185	88	181	20,12
16.06.2010	183	47	149	185	88	181	129,00
16.06.2010	184	34	133	185	88	181	241,04
16.06.2010	171	13	99	186	45	146	415,34
16.06.2010	183	47	149	186	45	146	504,26
16.06.2010	184	34	133	186	45	146	163,12
16.06.2010	185	88	181	186	45	146	397,19
16.06.2010	171	13	99	187	75	174	337,06
16.06.2010	183	47	149	187	75	174	431,85
16.06.2010	184	34	133	187	75	174	81,69
16.06.2010	185	88	181	187	75	174	319,52
16.06.2010	186	45	146	187	75	174	81,67
16.06.2010	171	13	99	188	49	147	252,05
16.06.2010	183	47	149	188	49	147	367,34
16.06.2010	184	34	133	188	49	147	81,14
16.06.2010	185	88	181	188	49	147	239,78

16.06.2010	186	45	146	188	49	147	217,58
16.06.2010	187	75	174	188	49	147	140,44
16.06.2010	171	13	99	189	39	141	143,74
16.06.2010	183	47	149	189	39	141	264,44
16.06.2010	184	34	133	189	39	141	152,59
16.06.2010	185	88	181	189	39	141	135,75
16.06.2010	186	45	146	189	39	141	314,65
16.06.2010	187	75	174	189	39	141	233,01
16.06.2010	188	49	147	189	39	141	115,69
16.06.2010	171	13	99	190	25	122	203,34
16.06.2010	183	47	149	190	25	122	322,28
16.06.2010	184	34	133	190	25	122	114,30
16.06.2010	185	88	181	190	25	122	193,34
16.06.2010	186	45	146	190	25	122	267,98
16.06.2010	187	75	174	190	25	122	187,87
16.06.2010	188	49	147	190	25	122	56,17
16.06.2010	189	39	141	190	25	122	61,57
16.06.2010	171	13	99	192	101	192	6,45
16.06.2010	183	47	149	192	101	192	126,97
16.06.2010	184	34	133	192	101	192	251,35
16.06.2010	185	88	181	192	101	192	15,31
16.06.2010	186	45	146	192	101	192	408,99
16.06.2010	187	75	174	192	101	192	330,65
16.06.2010	188	49	147	192	101	192	245,83
16.06.2010	189	39	141	192	101	192	138,06
16.06.2010	190	25	122	192	101	192	197,40
21.06.2010	171	13	99	183	47	149	120,58
21.06.2010	171	13	99	184	34	133	258,19
21.06.2010	183	47	149	184	34	133	358,89
21.06.2010	171	13	99	185	88	181	1,72
21.06.2010	183	47	149	185	88	181	119,13
21.06.2010	184	34	133	185	88	181	259,89
21.06.2010	171	13	99	186	45	146	405,22

21.06.2010	183	47	149	186	45	146	494,80
21.06.2010	184	34	133	186	45	146	152,72
21.06.2010	185	88	181	186	45	146	406,88
21.06.2010	171	13	99	187	75	174	330,05
21.06.2010	183	47	149	187	75	174	425,33
21.06.2010	184	34	133	187	75	174	74,27
21.06.2010	185	88	181	187	75	174	331,73
21.06.2010	186	45	146	187	75	174	78,67
21.06.2010	171	13	99	188	49	147	250,63
21.06.2010	183	47	149	188	49	147	365,27
21.06.2010	184	34	133	188	49	147	80,85
21.06.2010	185	88	181	188	49	147	252,32
21.06.2010	186	45	146	188	49	147	209,09
21.06.2010	187	75	174	188	49	147	135,02
21.06.2010	171	13	99	189	39	141	133,13
21.06.2010	183	47	149	189	39	141	253,36
21.06.2010	184	34	133	189	39	141	165,76
21.06.2010	185	88	181	189	39	141	134,68
21.06.2010	186	45	146	189	39	141	317,87
21.06.2010	187	75	174	189	39	141	239,21
21.06.2010	188	49	147	189	39	141	127,93
21.06.2010	171	13	99	190	25	122	203,83
21.06.2010	183	47	149	190	25	122	321,86
21.06.2010	184	34	133	190	25	122	114,05
21.06.2010	185	88	181	190	25	122	205,47
21.06.2010	186	45	146	190	25	122	257,92
21.06.2010	187	75	174	190	25	122	180,88
21.06.2010	188	49	147	190	25	122	54,41
21.06.2010	189	39	141	190	25	122	75,16
21.06.2010	171	13	99	192	101	192	4,24
21.06.2010	183	47	149	192	101	192	122,76
21.06.2010	184	34	133	192	101	192	254,21
21.06.2010	185	88	181	192	101	192	5,81

21.06.2010	186	45	146	192	101	192	401,08
21.06.2010	187	75	174	192	101	192	325,98
21.06.2010	188	49	147	192	101	192	247,30
21.06.2010	189	39	141	192	101	192	130,68
21.06.2010	190	25	122	192	101	192	200,86
21.06.2010	171	13	99	183	47	149	116,05
21.06.2010	171	13	99	184	34	133	262,24
21.06.2010	183	47	149	184	34	133	355,25
21.06.2010	171	13	99	185	88	181	0,69
21.06.2010	183	47	149	185	88	181	116,66
21.06.2010	184	34	133	185	88	181	262,02
21.06.2010	171	13	99	186	45	146	412,70
21.06.2010	183	47	149	186	45	146	494,05
21.06.2010	184	34	133	186	45	146	156,09
21.06.2010	185	88	181	186	45	146	412,56
21.06.2010	171	13	99	187	75	174	333,67
21.06.2010	183	47	149	187	75	174	421,40
21.06.2010	184	34	133	187	75	174	73,42
21.06.2010	185	88	181	187	75	174	333,48
21.06.2010	186	45	146	187	75	174	83,06
21.06.2010	171	13	99	188	49	147	260,21
21.06.2010	183	47	149	188	49	147	367,35
21.06.2010	184	34	133	188	49	147	75,72
21.06.2010	185	88	181	188	49	147	259,81
21.06.2010	186	45	146	188	49	147	204,13
21.06.2010	187	75	174	188	49	147	125,77
21.06.2010	171	13	99	189	39	141	133,06
21.06.2010	183	47	149	189	39	141	248,23
21.06.2010	184	34	133	189	39	141	169,37
21.06.2010	185	88	181	189	39	141	132,51
21.06.2010	186	45	146	189	39	141	324,91
21.06.2010	187	75	174	189	39	141	241,85
21.06.2010	188	49	147	189	39	141	139,11

21.06.2010	171	13	99	190	25	122	203,75
21.06.2010	183	47	149	190	25	122	316,02
21.06.2010	184	34	133	190	25	122	117,23
21.06.2010	185	88	181	190	25	122	203,27
21.06.2010	186	45	146	190	25	122	264,96
21.06.2010	187	75	174	190	25	122	183,20
21.06.2010	188	49	147	190	25	122	66,46
21.06.2010	189	39	141	190	25	122	74,98
21.06.2010	171	13	99	192	101	192	6,33
21.06.2010	183	47	149	192	101	192	117,62
21.06.2010	184	34	133	192	101	192	256,98
21.06.2010	185	88	181	192	101	192	6,53
21.06.2010	186	45	146	192	101	192	407,03
21.06.2010	187	75	174	192	101	192	328,21
21.06.2010	188	49	147	192	101	192	256,20
21.06.2010	189	39	141	192	101	192	130,77
21.06.2010	190	25	122	192	101	192	200,55
21.06.2010	171	13	99	183	47	149	136,88
21.06.2010	171	13	99	184	34	133	255,07
21.06.2010	183	47	149	184	34	133	367,81
21.06.2010	171	13	99	185	88	181	6,71
21.06.2010	183	47	149	185	88	181	130,51
21.06.2010	184	34	133	185	88	181	261,19
21.06.2010	171	13	99	186	45	146	400,06
21.06.2010	183	47	149	186	45	146	500,79
21.06.2010	184	34	133	186	45	146	149,31
21.06.2010	185	88	181	186	45	146	405,83
21.06.2010	171	13	99	187	75	174	328,33
21.06.2010	183	47	149	187	75	174	434,72
21.06.2010	184	34	133	187	75	174	75,36
21.06.2010	185	88	181	187	75	174	334,26
21.06.2010	186	45	146	187	75	174	74,04
21.06.2010	171	13	99	188	49	147	244,96

21.06.2010	183	47	149	188	49	147	374,36
21.06.2010	184	34	133	188	49	147	82,27
21.06.2010	185	88	181	188	49	147	251,64
21.06.2010	186	45	146	188	49	147	206,66
21.06.2010	187	75	174	188	49	147	137,77
21.06.2010	171	13	99	189	39	141	125,66
21.06.2010	183	47	149	189	39	141	261,91
21.06.2010	184	34	133	189	39	141	167,39
21.06.2010	185	88	181	189	39	141	132,28
21.06.2010	186	45	146	189	39	141	315,97
21.06.2010	187	75	174	189	39	141	242,00
21.06.2010	188	49	147	189	39	141	128,56
21.06.2010	171	13	99	190	25	122	196,47
21.06.2010	183	47	149	190	25	122	329,73
21.06.2010	184	34	133	190	25	122	114,25
21.06.2010	185	88	181	190	25	122	203,18
21.06.2010	186	45	146	190	25	122	254,78
21.06.2010	187	75	174	190	25	122	182,63
21.06.2010	188	49	147	190	25	122	54,51
21.06.2010	189	39	141	190	25	122	75,15
21.06.2010	171	13	99	192	101	192	5,74
21.06.2010	183	47	149	192	101	192	131,40
21.06.2010	184	34	133	192	101	192	260,25
21.06.2010	185	88	181	192	101	192	0,99
21.06.2010	186	45	146	192	101	192	404,93
21.06.2010	187	75	174	192	101	192	333,34
21.06.2010	188	49	147	192	101	192	250,65
21.06.2010	189	39	141	192	101	192	131,34
21.06.2010	190	25	122	192	101	192	202,20
21.06.2010	171	13	99	183	47	149	11,07
21.06.2010	171	13	99	184	34	133	326,35
21.06.2010	183	47	149	184	34	133	336,73
21.06.2010	171	13	99	185	88	181	1,38

21.06.2010	183	47	149	185	88	181	10,17
21.06.2010	184	34	133	185	88	181	327,59
21.06.2010	171	13	99	186	45	146	102,15
21.06.2010	183	47	149	186	45	146	110,82
21.06.2010	184	34	133	186	45	146	290,71
21.06.2010	185	88	181	186	45	146	102,23
21.06.2010	171	13	99	187	75	174	399,97
21.06.2010	183	47	149	187	75	174	410,06
21.06.2010	184	34	133	187	75	174	77,82
21.06.2010	185	88	181	187	75	174	401,25
21.06.2010	186	45	146	187	75	174	368,44
21.06.2010	171	13	99	188	49	147	252,59
21.06.2010	183	47	149	188	49	147	263,19
21.06.2010	184	34	133	188	49	147	76,00
21.06.2010	185	88	181	188	49	147	253,78
21.06.2010	186	45	146	188	49	147	215,21
21.06.2010	187	75	174	188	49	147	153,37
21.06.2010	171	13	99	189	39	141	192,62
21.06.2010	183	47	149	189	39	141	203,51
21.06.2010	184	34	133	189	39	141	190,12
21.06.2010	185	88	181	189	39	141	193,37
21.06.2010	186	45	146	189	39	141	115,46
21.06.2010	187	75	174	189	39	141	266,97
21.06.2010	188	49	147	189	39	141	121,17
21.06.2010	171	13	99	190	25	122	245,04
21.06.2010	183	47	149	190	25	122	256,10
21.06.2010	184	34	133	190	25	122	135,00
21.06.2010	185	88	181	190	25	122	245,96
21.06.2010	186	45	146	190	25	122	177,58
21.06.2010	187	75	174	190	25	122	209,27
21.06.2010	188	49	147	190	25	122	79,55
21.06.2010	189	39	141	190	25	122	63,16
21.06.2010	171	13	99	192	101	192	128,80

21.06.2010	183	47	149	192	101	192	139,35
21.06.2010	184	34	133	192	101	192	239,98
21.06.2010	185	88	181	192	101	192	129,39
21.06.2010	186	45	146	192	101	192	51,38
21.06.2010	187	75	174	192	101	192	317,78
21.06.2010	188	49	147	192	101	192	164,91
21.06.2010	189	39	141	192	101	192	67,11
21.06.2010	190	25	122	192	101	192	127,36
21.06.2010	171	13	99	183	47	149	7,39
21.06.2010	171	13	99	184	34	133	325,89
21.06.2010	183	47	149	184	34	133	333,21
21.06.2010	171	13	99	185	88	181	121,67
21.06.2010	183	47	149	185	88	181	128,10
21.06.2010	184	34	133	185	88	181	242,01
21.06.2010	171	13	99	186	45	146	105,84
21.06.2010	183	47	149	186	45	146	112,75
21.06.2010	184	34	133	186	45	146	239,07
21.06.2010	185	88	181	186	45	146	23,71
21.06.2010	171	13	99	187	75	174	398,44
21.06.2010	183	47	149	187	75	174	405,68
21.06.2010	184	34	133	187	75	174	76,59
21.06.2010	185	88	181	187	75	174	318,54
21.06.2010	186	45	146	187	75	174	315,05
21.06.2010	171	13	99	188	49	147	250,87
21.06.2010	183	47	149	188	49	147	258,25
21.06.2010	184	34	133	188	49	147	78,65
21.06.2010	185	88	181	188	49	147	163,50
21.06.2010	186	45	146	188	49	147	160,56
21.06.2010	187	75	174	188	49	147	155,06
21.06.2010	171	13	99	189	39	141	197,45
21.06.2010	183	47	149	189	39	141	204,36
21.06.2010	184	34	133	189	39	141	180,99
21.06.2010	185	88	181	189	39	141	79,55

21.06.2010	186	45	146	189	39	141	91,62
21.06.2010	187	75	174	189	39	141	256,53
21.06.2010	188	49	147	189	39	141	108,10
21.06.2010	171	13	99	190	25	122	246,06
21.06.2010	183	47	149	190	25	122	253,25
21.06.2010	184	34	133	190	25	122	131,61
21.06.2010	185	88	181	190	25	122	134,24
21.06.2010	186	45	146	190	25	122	141,95
21.06.2010	187	75	174	190	25	122	204,64
21.06.2010	188	49	147	190	25	122	71,71
21.06.2010	189	39	141	190	25	122	57,15
21.06.2010	171	13	99	192	101	192	0,00
21.06.2010	183	47	149	192	101	192	7,33
21.06.2010	184	34	133	192	101	192	325,96
21.06.2010	185	88	181	192	101	192	121,69
21.06.2010	186	45	146	192	101	192	105,87
21.06.2010	187	75	174	192	101	192	398,51
21.06.2010	188	49	147	192	101	192	250,94
21.06.2010	189	39	141	192	101	192	197,49
21.06.2010	190	25	122	192	101	192	246,11
21.06.2010	171	13	99	183	47	149	129,36
21.06.2010	171	13	99	184	34	133	259,10
21.06.2010	183	47	149	184	34	133	368,19
21.06.2010	171	13	99	185	88	181	5,04
21.06.2010	183	47	149	185	88	181	127,82
21.06.2010	184	34	133	185	88	181	263,41
21.06.2010	171	13	99	186	45	146	399,14
21.06.2010	183	47	149	186	45	146	496,94
21.06.2010	184	34	133	186	45	146	145,59
21.06.2010	185	88	181	186	45	146	403,73
21.06.2010	171	13	99	187	75	174	327,41
21.06.2010	183	47	149	187	75	174	431,09
21.06.2010	184	34	133	187	75	174	70,60

21.06.2010	185	88	181	187	75	174	331,87
21.06.2010	186	45	146	187	75	174	75,22
21.06.2010	171	13	99	188	49	147	248,64
21.06.2010	183	47	149	188	49	147	372,50
21.06.2010	184	34	133	188	49	147	83,91
21.06.2010	185	88	181	188	49	147	251,89
21.06.2010	186	45	146	188	49	147	206,65
21.06.2010	187	75	174	188	49	147	135,93
21.06.2010	171	13	99	189	39	141	128,90
21.06.2010	183	47	149	189	39	141	258,03
21.06.2010	184	34	133	189	39	141	169,74
21.06.2010	185	88	181	189	39	141	131,01
21.06.2010	186	45	146	189	39	141	314,93
21.06.2010	187	75	174	189	39	141	239,71
21.06.2010	188	49	147	189	39	141	129,45
21.06.2010	171	13	99	190	25	122	198,76
21.06.2010	183	47	149	190	25	122	326,34
21.06.2010	184	34	133	190	25	122	122,88
21.06.2010	185	88	181	190	25	122	201,42
21.06.2010	186	45	146	190	25	122	260,53
21.06.2010	187	75	174	190	25	122	186,82
21.06.2010	188	49	147	190	25	122	59,58
21.06.2010	189	39	141	190	25	122	72,84
22.06.2010	171	13	99	183	47	149	5,99
22.06.2010	171	13	99	184	34	133	258,69
22.06.2010	183	47	149	184	34	133	264,64
22.06.2010	171	13	99	185	88	181	5,99
22.06.2010	183	47	149	185	88	181	0,00
22.06.2010	184	34	133	185	88	181	264,65
22.06.2010	171	13	99	186	45	146	403,08
22.06.2010	183	47	149	186	45	146	408,89
22.06.2010	184	34	133	186	45	146	150,35
22.06.2010	185	88	181	186	45	146	408,91

22.06.2010	171	13	99	187	75	174	327,79
22.06.2010	183	47	149	187	75	174	333,69
22.06.2010	184	34	133	187	75	174	71,05
22.06.2010	185	88	181	187	75	174	333,70
22.06.2010	186	45	146	187	75	174	79,83
22.06.2010	171	13	99	188	49	147	249,30
22.06.2010	183	47	149	188	49	147	255,15
22.06.2010	184	34	133	188	49	147	83,78
22.06.2010	185	88	181	188	49	147	255,15
22.06.2010	186	45	146	188	49	147	210,88
22.06.2010	187	75	174	188	49	147	134,91
22.06.2010	171	13	99	189	39	141	130,27
22.06.2010	183	47	149	189	39	141	135,58
22.06.2010	184	34	133	189	39	141	168,22
22.06.2010	185	88	181	189	39	141	135,58
22.06.2010	186	45	146	189	39	141	318,19
22.06.2010	187	75	174	189	39	141	238,39
22.06.2010	188	49	147	189	39	141	128,64
22.06.2010	171	13	99	190	25	122	200,25
22.06.2010	183	47	149	190	25	122	205,90
22.06.2010	184	34	133	190	25	122	117,03
22.06.2010	185	88	181	190	25	122	205,90
22.06.2010	186	45	146	190	25	122	259,74
22.06.2010	187	75	174	190	25	122	180,91
22.06.2010	188	49	147	190	25	122	55,89
22.06.2010	189	39	141	190	25	122	74,13
22.06.2010	171	13	99	192	101	192	4,38
22.06.2010	183	47	149	192	101	192	2,70
22.06.2010	184	34	133	192	101	192	262,86
22.06.2010	185	88	181	192	101	192	2,69
22.06.2010	186	45	146	192	101	192	407,39
22.06.2010	187	75	174	192	101	192	332,02
22.06.2010	188	49	147	192	101	192	252,82

22.06.2010	189	39	141	192	101	192	132,99
22.06.2010	190	25	122	192	101	192	203,43
22.06.2010	171	13	99	183	47	149	123,55
22.06.2010	171	13	99	184	34	133	261,22
22.06.2010	183	47	149	184	34	133	362,59
22.06.2010	171	13	99	185	88	181	2,06
22.06.2010	183	47	149	185	88	181	125,50
22.06.2010	184	34	133	185	88	181	259,32
22.06.2010	171	13	99	186	45	146	420,30
22.06.2010	183	47	149	186	45	146	508,38
22.06.2010	184	34	133	186	45	146	165,93
22.06.2010	185	88	181	186	45	146	418,53
22.06.2010	171	13	99	187	75	174	332,52
22.06.2010	183	47	149	187	75	174	428,45
22.06.2010	184	34	133	187	75	174	73,36
22.06.2010	185	88	181	187	75	174	330,67
22.06.2010	186	45	146	187	75	174	93,21
22.06.2010	171	13	99	188	49	147	250,29
22.06.2010	183	47	149	188	49	147	366,52
22.06.2010	184	34	133	188	49	147	80,02
22.06.2010	185	88	181	188	49	147	248,24
22.06.2010	186	45	146	188	49	147	224,04
22.06.2010	187	75	174	188	49	147	134,24
22.06.2010	171	13	99	189	39	141	133,55
22.06.2010	183	47	149	189	39	141	256,57
22.06.2010	184	34	133	189	39	141	169,28
22.06.2010	185	88	181	189	39	141	131,53
22.06.2010	186	45	146	189	39	141	334,82
22.06.2010	187	75	174	189	39	141	241,67
22.06.2010	188	49	147	189	39	141	128,37
22.06.2010	171	13	99	190	25	122	202,01
22.06.2010	183	47	149	190	25	122	322,31
22.06.2010	184	34	133	190	25	122	116,74

22.06.2010	185	88	181	190	25	122	199,95
22.06.2010	186	45	146	190	25	122	275,32
22.06.2010	187	75	174	190	25	122	183,01
22.06.2010	188	49	147	190	25	122	56,47
22.06.2010	189	39	141	190	25	122	73,49
22.06.2010	171	13	99	192	101	192	5,92
22.06.2010	183	47	149	192	101	192	127,80
22.06.2010	184	34	133	192	101	192	255,31
22.06.2010	185	88	181	192	101	192	4,15
22.06.2010	186	45	146	192	101	192	414,41
22.06.2010	187	75	174	192	101	192	326,60
22.06.2010	188	49	147	192	101	192	244,77
22.06.2010	189	39	141	192	101	192	128,91
22.06.2010	190	25	122	192	101	192	196,82
22.06.2010	171	13	99	183	47	149	120,11
22.06.2010	171	13	99	184	34	133	260,74
22.06.2010	183	47	149	184	34	133	360,98
22.06.2010	171	13	99	185	88	181	5,44
22.06.2010	183	47	149	185	88	181	124,35
22.06.2010	184	34	133	185	88	181	255,31
22.06.2010	171	13	99	186	45	146	411,46
22.06.2010	183	47	149	186	45	146	500,61
22.06.2010	184	34	133	186	45	146	156,37
22.06.2010	185	88	181	186	45	146	406,08
22.06.2010	171	13	99	187	75	174	331,74
22.06.2010	183	47	149	187	75	174	427,30
22.06.2010	184	34	133	187	75	174	72,70
22.06.2010	185	88	181	187	75	174	326,31
22.06.2010	186	45	146	187	75	174	84,27
22.06.2010	171	13	99	188	49	147	249,58
22.06.2010	183	47	149	188	49	147	364,08
22.06.2010	184	34	133	188	49	147	84,01
22.06.2010	185	88	181	188	49	147	244,42

22.06.2010	186	45	146	188	49	147	217,04
22.06.2010	187	75	174	188	49	147	136,50
22.06.2010	171	13	99	189	39	141	131,55
22.06.2010	183	47	149	189	39	141	251,42
22.06.2010	184	34	133	189	39	141	170,77
22.06.2010	185	88	181	189	39	141	127,11
22.06.2010	186	45	146	189	39	141	326,57
22.06.2010	187	75	174	189	39	141	242,32
22.06.2010	188	49	147	189	39	141	128,74
22.06.2010	171	13	99	190	25	122	200,03
22.06.2010	183	47	149	190	25	122	317,87
22.06.2010	184	34	133	190	25	122	119,47
22.06.2010	185	88	181	190	25	122	195,13
22.06.2010	186	45	146	190	25	122	267,90
22.06.2010	187	75	174	190	25	122	184,63
22.06.2010	188	49	147	190	25	122	57,04
22.06.2010	189	39	141	190	25	122	73,12
22.06.2010	171	13	99	192	101	192	4,17
22.06.2010	183	47	149	192	101	192	122,96
22.06.2010	184	34	133	192	101	192	256,61
22.06.2010	185	88	181	192	101	192	1,44
22.06.2010	186	45	146	192	101	192	407,29
22.06.2010	187	75	174	192	101	192	327,58
22.06.2010	188	49	147	192	101	192	245,84
22.06.2010	189	39	141	192	101	192	128,51
22.06.2010	190	25	122	192	101	192	196,56
22.06.2010	171	13	99	183	47	149	0,00
22.06.2010	171	13	99	184	34	133	333,80
22.06.2010	183	47	149	184	34	133	333,82
22.06.2010	171	13	99	185	88	181	6,63
22.06.2010	183	47	149	185	88	181	6,66
22.06.2010	184	34	133	185	88	181	327,17
22.06.2010	171	13	99	186	45	146	159,42

22.06.2010	183	47	149	186	45	146	159,44
22.06.2010	184	34	133	186	45	146	176,65
22.06.2010	185	88	181	186	45	146	152,81
22.06.2010	171	13	99	187	75	174	384,48
22.06.2010	183	47	149	187	75	174	384,50
22.06.2010	184	34	133	187	75	174	51,83
22.06.2010	185	88	181	187	75	174	377,85
22.06.2010	186	45	146	187	75	174	226,22
22.06.2010	171	13	99	188	49	147	263,14
22.06.2010	183	47	149	188	49	147	263,16
22.06.2010	184	34	133	188	49	147	71,84
22.06.2010	185	88	181	188	49	147	256,52
22.06.2010	186	45	146	188	49	147	109,15
22.06.2010	187	75	174	188	49	147	123,60
22.06.2010	171	13	99	189	39	141	203,79
22.06.2010	183	47	149	189	39	141	203,83
22.06.2010	184	34	133	189	39	141	182,22
22.06.2010	185	88	181	189	39	141	198,08
22.06.2010	186	45	146	189	39	141	118,11
22.06.2010	187	75	174	189	39	141	231,82
22.06.2010	188	49	147	189	39	141	119,33
22.06.2010	171	13	99	190	25	122	254,64
22.06.2010	183	47	149	190	25	122	254,67
22.06.2010	184	34	133	190	25	122	127,95
22.06.2010	185	88	181	190	25	122	248,48
22.06.2010	186	45	146	190	25	122	133,55
22.06.2010	187	75	174	190	25	122	174,91
22.06.2010	188	49	147	190	25	122	78,55
22.06.2010	189	39	141	190	25	122	61,00
22.06.2010	171	13	99	192	101	192	145,25
22.06.2010	183	47	149	192	101	192	145,28
22.06.2010	184	34	133	192	101	192	226,03
22.06.2010	185	88	181	192	101	192	139,84

22.06.2010	186	45	146	192	101	192	104,81
22.06.2010	187	75	174	192	101	192	277,50
22.06.2010	188	49	147	192	101	192	156,19
22.06.2010	189	39	141	192	101	192	60,11
22.06.2010	190	25	122	192	101	192	117,78
22.06.2010	171	13	99	183	47	149	6,09
22.06.2010	171	13	99	184	34	133	322,71
22.06.2010	183	47	149	184	34	133	328,79
22.06.2010	171	13	99	185	88	181	0,00
22.06.2010	183	47	149	185	88	181	6,07
22.06.2010	184	34	133	185	88	181	322,73
22.06.2010	171	13	99	186	45	146	40,81
22.06.2010	183	47	149	186	45	146	37,63
22.06.2010	184	34	133	186	45	146	346,48
22.06.2010	185	88	181	186	45	146	40,81
22.06.2010	171	13	99	187	75	174	385,90
22.06.2010	183	47	149	187	75	174	391,96
22.06.2010	184	34	133	187	75	174	64,52
22.06.2010	185	88	181	187	75	174	385,91
22.06.2010	186	45	146	187	75	174	408,18
22.06.2010	171	13	99	188	49	147	254,21
22.06.2010	183	47	149	188	49	147	260,29
22.06.2010	184	34	133	188	49	147	70,04
22.06.2010	185	88	181	188	49	147	254,22
22.06.2010	186	45	146	188	49	147	279,96
22.06.2010	187	75	174	188	49	147	134,54
22.06.2010	171	13	99	189	39	141	199,24
22.06.2010	183	47	149	189	39	141	204,70
22.06.2010	184	34	133	189	39	141	177,26
22.06.2010	185	88	181	189	39	141	199,25
22.06.2010	186	45	146	189	39	141	235,99
22.06.2010	187	75	174	189	39	141	238,83
22.06.2010	188	49	147	189	39	141	115,64

22.06.2010	171	13	99	190	25	122	251,20
22.06.2010	183	47	149	190	25	122	257,01
22.06.2010	184	34	133	190	25	122	120,82
22.06.2010	185	88	181	190	25	122	251,20
22.06.2010	186	45	146	190	25	122	284,62
22.06.2010	187	75	174	190	25	122	178,96
22.06.2010	188	49	147	190	25	122	73,77
22.06.2010	189	39	141	190	25	122	63,49
22.06.2010	171	13	99	192	101	192	155,73
22.06.2010	183	47	149	192	101	192	160,97
22.06.2010	184	34	133	192	101	192	211,41
22.06.2010	185	88	181	192	101	192	155,73
22.06.2010	186	45	146	192	101	192	193,80
22.06.2010	187	75	174	192	101	192	274,98
22.06.2010	188	49	147	192	101	192	144,20
22.06.2010	189	39	141	192	101	192	45,72
22.06.2010	190	25	122	192	101	192	106,83
22.06.2010	171	13	99	183	47	149	4,88
22.06.2010	171	13	99	184	34	133	328,01
22.06.2010	183	47	149	184	34	133	332,88
22.06.2010	171	13	99	185	88	181	4,73
22.06.2010	183	47	149	185	88	181	0,21
22.06.2010	184	34	133	185	88	181	332,72
22.06.2010	171	13	99	186	45	146	102,58
22.06.2010	183	47	149	186	45	146	104,78
22.06.2010	184	34	133	186	45	146	293,08
22.06.2010	185	88	181	186	45	146	104,57
22.06.2010	171	13	99	187	75	174	399,62
22.06.2010	183	47	149	187	75	174	404,50
22.06.2010	184	34	133	187	75	174	75,14
22.06.2010	185	88	181	187	75	174	404,36
22.06.2010	186	45	146	187	75	174	368,20
22.06.2010	171	13	99	188	49	147	259,18

22.06.2010	183	47	149	188	49	147	264,03
22.06.2010	184	34	133	188	49	147	70,33
22.06.2010	185	88	181	188	49	147	263,87
22.06.2010	186	45	146	188	49	147	223,51
22.06.2010	187	75	174	188	49	147	145,03
22.06.2010	171	13	99	189	39	141	200,35
22.06.2010	183	47	149	189	39	141	204,49
22.06.2010	184	34	133	189	39	141	181,21
22.06.2010	185	88	181	189	39	141	204,29
22.06.2010	186	45	146	189	39	141	126,23
22.06.2010	187	75	174	189	39	141	255,00
22.06.2010	188	49	147	189	39	141	118,92
22.06.2010	171	13	99	190	25	122	243,34
22.06.2010	183	47	149	190	25	122	247,74
22.06.2010	184	34	133	190	25	122	139,16
22.06.2010	185	88	181	190	25	122	247,54
22.06.2010	186	45	146	190	25	122	175,66
22.06.2010	187	75	174	190	25	122	210,53
22.06.2010	188	49	147	190	25	122	87,38
22.06.2010	189	39	141	190	25	122	49,77
22.06.2010	171	13	99	192	101	192	122,08
22.06.2010	183	47	149	192	101	192	125,84
22.06.2010	184	34	133	192	101	192	242,56
22.06.2010	185	88	181	192	101	192	125,62
22.06.2010	186	45	146	192	101	192	50,53
22.06.2010	187	75	174	192	101	192	317,67
22.06.2010	188	49	147	192	101	192	173,02
22.06.2010	189	39	141	192	101	192	81,47
22.06.2010	190	25	122	192	101	192	129,14
22.06.2010	171	13	99	183	47	149	8,43
22.06.2010	171	13	99	184	34	133	326,20
22.06.2010	183	47	149	184	34	133	334,60
22.06.2010	171	13	99	185	88	181	129,31

22.06.2010	183	47	149	185	88	181	136,39
22.06.2010	184	34	133	185	88	181	239,09
22.06.2010	171	13	99	186	45	146	129,81
22.06.2010	183	47	149	186	45	146	123,33
22.06.2010	184	34	133	186	45	146	430,09
22.06.2010	185	88	181	186	45	146	258,91
22.06.2010	171	13	99	187	75	174	398,32
22.06.2010	183	47	149	187	75	174	406,68
22.06.2010	184	34	133	187	75	174	75,26
22.06.2010	185	88	181	187	75	174	314,33
22.06.2010	186	45	146	187	75	174	496,52
22.06.2010	171	13	99	188	49	147	255,02
22.06.2010	183	47	149	188	49	147	263,45
22.06.2010	184	34	133	188	49	147	73,20
22.06.2010	185	88	181	188	49	147	166,87
22.06.2010	186	45	146	188	49	147	364,33
22.06.2010	187	75	174	188	49	147	148,22
22.06.2010	171	13	99	189	39	141	198,73
22.06.2010	183	47	149	189	39	141	206,38
22.06.2010	184	34	133	189	39	141	183,56
22.06.2010	185	88	181	189	39	141	73,37
22.06.2010	186	45	146	189	39	141	326,48
22.06.2010	187	75	174	189	39	141	257,12
22.06.2010	188	49	147	189	39	141	118,14
22.06.2010	171	13	99	190	25	122	243,34
22.06.2010	183	47	149	190	25	122	251,38
22.06.2010	184	34	133	190	25	122	137,79
22.06.2010	185	88	181	190	25	122	124,30
22.06.2010	186	45	146	190	25	122	367,66
22.06.2010	187	75	174	190	25	122	208,72
22.06.2010	188	49	147	190	25	122	83,66
22.06.2010	189	39	141	190	25	122	53,16
22.06.2010	171	13	99	192	101	192	0,00

22.06.2010	183	47	149	192	101	192	8,41
22.06.2010	184	34	133	192	101	192	326,23
22.06.2010	185	88	181	192	101	192	129,30
22.06.2010	186	45	146	192	101	192	129,83
22.06.2010	187	75	174	192	101	192	398,36
22.06.2010	188	49	147	192	101	192	255,05
22.06.2010	189	39	141	192	101	192	198,73
22.06.2010	190	25	122	192	101	192	243,35
22.06.2010	171	13	99	183	47	149	6,89
22.06.2010	171	13	99	184	34	133	319,46
22.06.2010	183	47	149	184	34	133	326,28
22.06.2010	171	13	99	185	88	181	0,00
22.06.2010	183	47	149	185	88	181	6,92
22.06.2010	184	34	133	185	88	181	319,44
22.06.2010	171	13	99	186	45	146	169,18
22.06.2010	183	47	149	186	45	146	175,91
22.06.2010	184	34	133	186	45	146	151,26
22.06.2010	185	88	181	186	45	146	169,16
22.06.2010	171	13	99	187	75	174	399,21
22.06.2010	183	47	149	187	75	174	405,95
22.06.2010	184	34	133	187	75	174	83,18
22.06.2010	185	88	181	187	75	174	399,19
22.06.2010	186	45	146	187	75	174	230,04
22.06.2010	171	13	99	188	49	147	258,00
22.06.2010	183	47	149	188	49	147	264,86
22.06.2010	184	34	133	188	49	147	62,91
22.06.2010	185	88	181	188	49	147	257,98
22.06.2010	186	45	146	188	49	147	92,33
22.06.2010	187	75	174	188	49	147	145,79
22.06.2010	171	13	99	189	39	141	197,83
22.06.2010	183	47	149	189	39	141	204,25
22.06.2010	184	34	133	189	39	141	177,81
22.06.2010	185	88	181	189	39	141	197,79

22.06.2010	186	45	146	189	39	141	111,15
22.06.2010	187	75	174	189	39	141	259,01
22.06.2010	188	49	147	189	39	141	122,15
22.06.2010	171	13	99	190	25	122	246,76
22.06.2010	183	47	149	190	25	122	253,48
22.06.2010	184	34	133	190	25	122	126,60
22.06.2010	185	88	181	190	25	122	246,73
22.06.2010	186	45	146	190	25	122	118,99
22.06.2010	187	75	174	190	25	122	204,44
22.06.2010	188	49	147	190	25	122	82,28
22.06.2010	189	39	141	190	25	122	59,15
22.06.2010	171	13	99	192	101	192	129,58
22.06.2010	183	47	149	192	101	192	135,61
22.06.2010	184	34	133	192	101	192	231,15
22.06.2010	185	88	181	192	101	192	129,53
22.06.2010	186	45	146	192	101	192	113,37
22.06.2010	187	75	174	192	101	192	314,31
22.06.2010	188	49	147	192	101	192	169,31
22.06.2010	189	39	141	192	101	192	71,74
22.06.2010	190	25	122	192	101	192	127,97
23.06.2010	171	13	99	183	47	149	6,08
23.06.2010	171	13	99	184	34	133	321,07
23.06.2010	183	47	149	184	34	133	326,70
23.06.2010	171	13	99	185	88	181	123,46
23.06.2010	183	47	149	185	88	181	129,34
23.06.2010	184	34	133	185	88	181	235,67
23.06.2010	171	13	99	186	45	146	80,44
23.06.2010	183	47	149	186	45	146	86,47
23.06.2010	184	34	133	186	45	146	255,39
23.06.2010	185	88	181	186	45	146	44,58
23.06.2010	171	13	99	187	75	174	399,49
23.06.2010	183	47	149	187	75	174	404,96
23.06.2010	184	34	133	187	75	174	81,39

23.06.2010	185	88	181	187	75	174	317,04
23.06.2010	186	45	146	187	75	174	336,17
23.06.2010	171	13	99	188	49	147	254,88
23.06.2010	183	47	149	188	49	147	260,62
23.06.2010	184	34	133	188	49	147	67,69
23.06.2010	185	88	181	188	49	147	168,87
23.06.2010	186	45	146	188	49	147	187,73
23.06.2010	187	75	174	188	49	147	148,83
23.06.2010	171	13	99	189	39	141	194,34
23.06.2010	183	47	149	189	39	141	200,36
23.06.2010	184	34	133	189	39	141	182,42
23.06.2010	185	88	181	189	39	141	73,48
23.06.2010	186	45	146	189	39	141	113,90
23.06.2010	187	75	174	189	39	141	261,79
23.06.2010	188	49	147	189	39	141	122,26
23.06.2010	171	13	99	190	25	122	246,60
23.06.2010	183	47	149	190	25	122	252,68
23.06.2010	184	34	133	190	25	122	129,34
23.06.2010	185	88	181	190	25	122	132,33
23.06.2010	186	45	146	190	25	122	167,69
23.06.2010	187	75	174	190	25	122	205,05
23.06.2010	188	49	147	190	25	122	82,39
23.06.2010	189	39	141	190	25	122	62,11
23.06.2010	171	13	99	192	101	192	0,30
23.06.2010	183	47	149	192	101	192	6,28
23.06.2010	184	34	133	192	101	192	320,98
23.06.2010	185	88	181	192	101	192	123,21
23.06.2010	186	45	146	192	101	192	80,22
23.06.2010	187	75	174	192	101	192	399,42
23.06.2010	188	49	147	192	101	192	254,78
23.06.2010	189	39	141	192	101	192	194,12
23.06.2010	190	25	122	192	101	192	246,41
23.06.2010	171	13	99	183	47	149	1,68

23.06.2010	171	13	99	184	34	133	330,64
23.06.2010	183	47	149	184	34	133	331,68
23.06.2010	171	13	99	185	88	181	128,28
23.06.2010	183	47	149	185	88	181	129,90
23.06.2010	184	34	133	185	88	181	239,92
23.06.2010	171	13	99	186	45	146	108,96
23.06.2010	183	47	149	186	45	146	110,64
23.06.2010	184	34	133	186	45	146	285,73
23.06.2010	185	88	181	186	45	146	45,81
23.06.2010	171	13	99	187	75	174	405,75
23.06.2010	183	47	149	187	75	174	406,69
23.06.2010	184	34	133	187	75	174	79,39
23.06.2010	185	88	181	187	75	174	319,25
23.06.2010	186	45	146	187	75	174	365,06
23.06.2010	171	13	99	188	49	147	261,96
23.06.2010	183	47	149	188	49	147	263,06
23.06.2010	184	34	133	188	49	147	70,39
23.06.2010	185	88	181	188	49	147	170,34
23.06.2010	186	45	146	188	49	147	216,11
23.06.2010	187	75	174	188	49	147	149,23
23.06.2010	171	13	99	189	39	141	201,91
23.06.2010	183	47	149	189	39	141	203,46
23.06.2010	184	34	133	189	39	141	182,51
23.06.2010	185	88	181	189	39	141	76,56
23.06.2010	186	45	146	189	39	141	116,98
23.06.2010	187	75	174	189	39	141	260,80
23.06.2010	188	49	147	189	39	141	119,64
23.06.2010	171	13	99	190	25	122	251,57
23.06.2010	183	47	149	190	25	122	253,02
23.06.2010	184	34	133	190	25	122	131,74
23.06.2010	185	88	181	190	25	122	132,38
23.06.2010	186	45	146	190	25	122	174,97
23.06.2010	187	75	174	190	25	122	207,39

23.06.2010	188	49	147	190	25	122	81,23
23.06.2010	189	39	141	190	25	122	58,67
23.06.2010	171	13	99	192	101	192	0,00
23.06.2010	183	47	149	192	101	192	1,68
23.06.2010	184	34	133	192	101	192	330,59
23.06.2010	185	88	181	192	101	192	128,26
23.06.2010	186	45	146	192	101	192	108,97
23.06.2010	187	75	174	192	101	192	405,70
23.06.2010	188	49	147	192	101	192	261,91
23.06.2010	189	39	141	192	101	192	201,89
23.06.2010	190	25	122	192	101	192	251,54
23.06.2010	171	13	99	183	47	149	132,35
23.06.2010	171	13	99	184	34	133	236,95
23.06.2010	183	47	149	184	34	133	330,28
23.06.2010	171	13	99	185	88	181	132,32
23.06.2010	183	47	149	185	88	181	0,00
23.06.2010	184	34	133	185	88	181	330,27
23.06.2010	171	13	99	186	45	146	259,88
23.06.2010	183	47	149	186	45	146	128,28
23.06.2010	184	34	133	186	45	146	430,68
23.06.2010	185	88	181	186	45	146	128,31
23.06.2010	171	13	99	187	75	174	315,22
23.06.2010	183	47	149	187	75	174	404,04
23.06.2010	184	34	133	187	75	174	78,33
23.06.2010	185	88	181	187	75	174	404,03
23.06.2010	186	45	146	187	75	174	497,56
23.06.2010	171	13	99	188	49	147	165,79
23.06.2010	183	47	149	188	49	147	259,38
23.06.2010	184	34	133	188	49	147	72,42
23.06.2010	185	88	181	188	49	147	259,37
23.06.2010	186	45	146	188	49	147	364,86
23.06.2010	187	75	174	188	49	147	150,00
23.06.2010	171	13	99	189	39	141	73,72

23.06.2010	183	47	149	189	39	141	202,80
23.06.2010	184	34	133	189	39	141	180,87
23.06.2010	185	88	181	189	39	141	202,78
23.06.2010	186	45	146	189	39	141	327,92
23.06.2010	187	75	174	189	39	141	258,24
23.06.2010	188	49	147	189	39	141	117,06
23.06.2010	171	13	99	190	25	122	127,72
23.06.2010	183	47	149	190	25	122	250,53
23.06.2010	184	34	133	190	25	122	131,81
23.06.2010	185	88	181	190	25	122	250,51
23.06.2010	186	45	146	190	25	122	371,44
23.06.2010	187	75	174	190	25	122	206,80
23.06.2010	188	49	147	190	25	122	80,83
23.06.2010	189	39	141	190	25	122	56,49
23.06.2010	171	13	99	192	101	192	132,52
23.06.2010	183	47	149	192	101	192	0,27
23.06.2010	184	34	133	192	101	192	330,55
23.06.2010	185	88	181	192	101	192	0,29
23.06.2010	186	45	146	192	101	192	128,14
23.06.2010	187	75	174	192	101	192	404,31
23.06.2010	188	49	147	192	101	192	259,65
23.06.2010	189	39	141	192	101	192	203,00
23.06.2010	190	25	122	192	101	192	250,76
02.06.2010	170	49	148	172	19	106	0,651558712
02.06.2010	170	49	148	173	16	105	72,14
02.06.2010	172	19	106	173	16	105	71,52436017
02.06.2010	170	49	148	174	125	211	236,236116
02.06.2010	172	19	106	174	125	211	236,8841544
02.06.2010	173	16	105	174	125	211	307,5502506
02.06.2010	170	49	148	175	52	150	54,69774975
02.06.2010	172	19	106	175	52	150	54,13
02.06.2010	173	16	105	175	52	150	20,51887267
02.06.2010	174	125	211	175	52	150	288,2969234

02.06.2010	170	49	148	176	37	136	27,75839918
02.06.2010	172	19	106	176	37	136	27,21055458
02.06.2010	173	16	105	176	37	136	45,6552304
02.06.2010	174	125	211	176	37	136	261,8987889
02.06.2010	175	52	150	176	37	136	27,06677258
02.06.2010	170	49	148	177	13	97	48,96154198
02.06.2010	172	19	106	177	13	97	48,32939187
02.06.2010	173	16	105	177	13	97	23,50
02.06.2010	174	125	211	177	13	97	284,9568406
02.06.2010	175	52	150	177	13	97	13,49127296
02.06.2010	176	37	136	177	13	97	23,96053836
02.06.2010	170	49	148	178	26	122	56,78956449
02.06.2010	172	19	106	178	26	122	56,20883675
02.06.2010	173	16	105	178	26	122	17,94210836
02.06.2010	174	125	211	178	26	122	290,7136496
02.06.2010	175	52	150	178	26	122	2,58
02.06.2010	176	37	136	178	26	122	29,27173582
02.06.2010	177	13	97	178	26	122	13,52303724
02.06.2010	170	49	148	179	54	153	64,52846318
02.06.2010	172	19	106	179	54	153	63,92641134
02.06.2010	173	16	105	179	54	153	9,019253611
02.06.2010	174	125	211	179	54	153	299,2570076
02.06.2010	175	52	150	179	54	153	11,52607492
02.06.2010	176	37	136	179	54	153	37,45513167
02.06.2010	177	13	97	179	54	153	17,31990892
02.06.2010	178	26	122	179	54	153	8,950894483
02.06.2010	170	49	148	180	67	166	17,63644211
02.06.2010	172	19	106	180	67	166	18,18189885
02.06.2010	173	16	105	180	67	166	89,29182161
02.06.2010	174	125	211	180	67	166	220,3779321
02.06.2010	175	52	150	180	67	166	72,27935437
02.06.2010	176	37	136	180	67	166	45,3920342
02.06.2010	177	13	97	180	67	166	65,90618089

02.06.2010	178	26	122	180	67	166	74,33288135
02.06.2010	179	54	153	180	67	166	81,90991635
02.06.2010	170	49	148	181	60	156	56,90261787
02.06.2010	172	19	106	181	60	156	57,46
02.06.2010	173	16	105	181	60	156	128,2986106
02.06.2010	174	125	211	181	60	156	184,8843787
02.06.2010	175	52	150	181	60	156	111,5723688
02.06.2010	176	37	136	181	60	156	84,65623238
02.06.2010	177	13	97	181	60	156	104,8248942
02.06.2010	178	26	122	181	60	156	113,6190442
02.06.2010	179	54	153	181	60	156	121,11
02.06.2010	180	67	166	181	60	156	39,29310192
02.06.2010	170	49	148	182	13	96	63,91397611
02.06.2010	172	19	106	182	13	96	63,31292894
02.06.2010	173	16	105	182	13	96	9,658568011
02.06.2010	174	125	211	182	13	96	298,6091943
02.06.2010	175	52	150	182	13	96	10,875704
02.06.2010	176	37	136	182	13	96	36,81653284
02.06.2010	177	13	97	182	13	96	16,84588747
02.06.2010	178	26	122	182	13	96	8,299520998
02.06.2010	179	54	153	182	13	96	0,658453705
02.06.2010	180	67	166	182	13	96	81,30542484
02.06.2010	181	60	156	182	13	96	120,5113448
02.06.2010	170	49	148	191	128	203	72,09191091
02.06.2010	172	19	106	191	128	203	71,48381086
02.06.2010	173	16	105	191	128	203	3,452960364
02.06.2010	174	125	211	191	128	203	306,9787222
02.06.2010	175	52	150	191	128	203	19,06272758
02.06.2010	176	37	136	191	128	203	45,15241566
02.06.2010	177	13	97	191	128	203	24,07130152
02.06.2010	178	26	122	191	128	203	16,51885687
02.06.2010	179	54	153	191	128	203	7,724562871
02.06.2010	180	67	166	191	128	203	89,40151

02.06.2010	181	60	156	191	128	203	128,5404963
02.06.2010	182	13	96	191	128	203	8,374817611
02.06.2010	170	49	148	193	171	232	57,14963142
02.06.2010	172	19	106	193	171	232	57,71072214
02.06.2010	173	16	105	193	171	232	128,58
02.06.2010	174	125	211	193	171	232	184,5110427
02.06.2010	175	52	150	193	171	232	111,8260415
02.06.2010	176	37	136	193	171	232	84,90058822
02.06.2010	177	13	97	193	171	232	105,1115701
02.06.2010	178	26	122	193	171	232	113,8771207
02.06.2010	179	54	153	193	171	232	121,3796423
02.06.2010	180	67	166	193	171	232	39,54721233
02.06.2010	181	60	156	193	171	232	0,4032189
02.06.2010	182	13	96	193	171	232	120,7816921
02.06.2010	191	128	203	193	171	232	128,8165713
02.06.2010	170	49	148	194	81	184	25,53808122
02.06.2010	172	19	106	194	81	184	25,01149208
02.06.2010	173	16	105	194	81	184	48,27637803
02.06.2010	174	125	211	194	81	184	259,2867531
02.06.2010	175	52	150	194	81	184	29,5146564
02.06.2010	176	37	136	194	81	184	2,656012683
02.06.2010	177	13	97	194	81	184	26,60565832
02.06.2010	178	26	122	194	81	184	31,76299848
02.06.2010	179	54	153	194	81	184	40,02826315
02.06.2010	180	67	166	194	81	184	43,16953763
02.06.2010	181	60	156	194	81	184	82,38793733
02.06.2010	182	13	96	194	81	184	39,38700884
02.06.2010	191	128	203	194	81	184	47,73439315
02.06.2010	193	171	232	194	81	184	82,62681262
02.06.2010	170	49	148	172	19	106	14,0761325
02.06.2010	170	49	148	173	16	105	72,74609834
02.06.2010	172	19	106	173	16	105	59,06327295
02.06.2010	170	49	148	174	125	211	257,4440141

02.06.2010	172	19	106	174	125	211	271,008299
02.06.2010	173	16	105	174	125	211	330,0715121
02.06.2010	170	49	148	175	52	150	67,46453231
02.06.2010	172	19	106	175	52	150	53,57385209
02.06.2010	173	16	105	175	52	150	7,127336831
02.06.2010	174	125	211	175	52	150	324,4360256
02.06.2010	170	49	148	176	37	136	26,10027075
02.06.2010	172	19	106	176	37	136	12,05318049
02.06.2010	173	16	105	176	37	136	47,22426123
02.06.2010	174	125	211	176	37	136	282,897836
02.06.2010	175	52	150	176	37	136	41,58045221
02.06.2010	170	49	148	177	13	97	108,6444874
02.06.2010	172	19	106	177	13	97	95,76210706
02.06.2010	173	16	105	177	13	97	39,11038572
02.06.2010	174	125	211	177	13	97	365,6252578
02.06.2010	175	52	150	177	13	97	46,11938268
02.06.2010	176	37	136	177	13	97	84,53429712
02.06.2010	170	49	148	178	26	122	46,62678332
02.06.2010	172	19	106	178	26	122	32,65768862
02.06.2010	173	16	105	178	26	122	26,89016292
02.06.2010	174	125	211	178	26	122	303,4707637
02.06.2010	175	52	150	178	26	122	20,96716739
02.06.2010	176	37	136	178	26	122	20,63103868
02.06.2010	177	13	97	178	26	122	65,22120234
02.06.2010	170	49	148	179	54	153	67,22841359
02.06.2010	172	19	106	179	54	153	53,31196702
02.06.2010	173	16	105	179	54	153	7,800198718
02.06.2010	174	125	211	179	54	153	324,1238229
02.06.2010	175	52	150	179	54	153	0,748342606
02.06.2010	176	37	136	179	54	153	41,30211777
02.06.2010	177	13	97	179	54	153	46,72044958
02.06.2010	178	26	122	179	54	153	20,67455513
02.06.2010	170	49	148	180	67	166	4,140499453

02.06.2010	172	19	106	180	67	166	12,08237133
02.06.2010	173	16	105	180	67	166	69,68924526
02.06.2010	174	125	211	180	67	166	260,7369806
02.06.2010	175	52	150	180	67	166	64,61477152
02.06.2010	176	37	136	180	67	166	23,71423863
02.06.2010	177	13	97	180	67	166	105,1278961
02.06.2010	178	26	122	180	67	166	43,94099428
02.06.2010	179	54	153	180	67	166	64,411476
02.06.2010	170	49	148	181	60	156	1,757590436
02.06.2010	172	19	106	181	60	156	15,33292554
02.06.2010	173	16	105	181	60	156	73,66525938
02.06.2010	174	125	211	181	60	156	256,6447031
02.06.2010	175	52	150	181	60	156	68,48446129
02.06.2010	176	37	136	181	60	156	27,27851933
02.06.2010	177	13	97	181	60	156	109,283935
02.06.2010	178	26	122	181	60	156	47,71101818
02.06.2010	179	54	153	181	60	156	68,26344998
02.06.2010	180	67	166	181	60	156	4,196835369
02.06.2010	170	49	148	182	13	96	72,89135066
02.06.2010	172	19	106	182	13	96	59,19449377
02.06.2010	173	16	105	182	13	96	0,315210714
02.06.2010	174	125	211	182	13	96	330,2021422
02.06.2010	175	52	150	182	13	96	7,064965865
02.06.2010	176	37	136	182	13	96	47,34336711
02.06.2010	177	13	97	182	13	96	39,11881447
02.06.2010	178	26	122	182	13	96	26,98120687
02.06.2010	179	54	153	182	13	96	7,724562871
02.06.2010	180	67	166	182	13	96	69,84512316
02.06.2010	181	60	156	182	13	96	73,81610632
02.06.2010	170	49	148	191	128	203	73,12419184
02.06.2010	172	19	106	191	128	203	59,38287238
02.06.2010	173	16	105	191	128	203	1,257255811
02.06.2010	174	125	211	191	128	203	330,3809924

02.06.2010	175	52	150	191	128	203	6,731058911
02.06.2010	176	37	136	191	128	203	47,49489614
02.06.2010	177	13	97	191	128	203	39,38769682
02.06.2010	178	26	122	191	128	203	27,05208523
02.06.2010	179	54	153	191	128	203	7,34207869
02.06.2010	180	67	166	191	128	203	70,11430595
02.06.2010	181	60	156	191	128	203	74,06768884
02.06.2010	182	13	96	191	128	203	0,950396061
02.06.2010	170	49	148	193	171	232	33,1492097
02.06.2010	172	19	106	193	171	232	45,56730384
02.06.2010	173	16	105	193	171	232	98,82446561
02.06.2010	174	125	211	193	171	232	237,1580023
02.06.2010	175	52	150	193	171	232	94,82352551
02.06.2010	176	37	136	193	171	232	56,4102296
02.06.2010	177	13	97	193	171	232	130,2761792
02.06.2010	178	26	122	193	171	232	75,26836798
02.06.2010	179	54	153	193	171	232	94,75787127
02.06.2010	180	67	166	193	171	232	33,62898268
02.06.2010	181	60	156	193	171	232	31,4403026
02.06.2010	182	13	96	193	171	232	99,03154318
02.06.2010	191	128	203	193	171	232	99,47573609
02.06.2010	170	49	148	194	81	184	33,7900218
02.06.2010	172	19	106	194	81	184	46,38059365
02.06.2010	173	16	105	194	81	184	99,94177173
02.06.2010	174	125	211	194	81	184	235,887345
02.06.2010	175	52	150	194	81	184	95,90150866
02.06.2010	176	37	136	194	81	184	57,31873233
02.06.2010	177	13	97	194	81	184	131,4952205
02.06.2010	178	26	122	194	81	184	76,28205839
02.06.2010	179	54	153	194	81	184	95,83046652
02.06.2010	180	67	166	194	81	184	34,3990862
02.06.2010	181	60	156	194	81	184	32,09638201
02.06.2010	182	13	96	194	81	184	100,1473959

02.06.2010	191	128	203	194	81	184	100,5863021
02.06.2010	193	171	232	194	81	184	1,278627129
02.06.2010	170	49	148	172	19	106	13,81576472
02.06.2010	170	49	148	173	16	105	54,4320624
02.06.2010	172	19	106	173	16	105	67,204494
02.06.2010	170	49	148	174	125	211	61,48276588
02.06.2010	172	19	106	174	125	211	47,68355925
02.06.2010	173	16	105	174	125	211	112,6025627
02.06.2010	170	49	148	175	52	150	47,37426462
02.06.2010	172	19	106	175	52	150	59,76185349
02.06.2010	173	16	105	175	52	150	8,210893951
02.06.2010	174	125	211	175	52	150	104,6488349
02.06.2010	170	49	148	176	37	136	28,31685885
02.06.2010	172	19	106	176	37	136	41,10504166
02.06.2010	173	16	105	176	37	136	26,15161168
02.06.2010	174	125	211	176	37	136	87,2469278
02.06.2010	175	52	150	176	37	136	19,13721058
02.06.2010	170	49	148	177	13	97	77,95264133
02.06.2010	172	19	106	177	13	97	88,90898004
02.06.2010	173	16	105	177	13	97	29,34970147
02.06.2010	174	125	211	177	13	97	129,87363
02.06.2010	175	52	150	177	13	97	32,63043392
02.06.2010	176	37	136	177	13	97	50,84663337
02.06.2010	170	49	148	178	26	122	16,69291615
02.06.2010	172	19	106	178	26	122	30,23066306
02.06.2010	173	16	105	178	26	122	38,06665467
02.06.2010	174	125	211	178	26	122	77,52849678
02.06.2010	175	52	150	178	26	122	31,46098077
02.06.2010	176	37	136	178	26	122	12,41204157
02.06.2010	177	13	97	178	26	122	63,21419925
02.06.2010	170	49	148	179	54	153	32,27080986
02.06.2010	172	19	106	179	54	153	45,52307845
02.06.2010	173	16	105	179	54	153	22,51576326

02.06.2010	174	125	211	179	54	153	92,19018152
02.06.2010	175	52	150	179	54	153	16,62025067
02.06.2010	176	37	136	179	54	153	5,572596063
02.06.2010	177	13	97	179	54	153	49,18168762
02.06.2010	178	26	122	179	54	153	15,66338057
02.06.2010	170	49	148	180	67	166	1,567433337
02.06.2010	172	19	106	180	67	166	12,53154059
02.06.2010	173	16	105	180	67	166	55,98502393
02.06.2010	174	125	211	180	67	166	60,21447238
02.06.2010	175	52	150	180	67	166	48,93986054
02.06.2010	176	37	136	180	67	166	29,87677005
02.06.2010	177	13	97	180	67	166	79,50800098
02.06.2010	178	26	122	180	67	166	18,19406605
02.06.2010	179	54	153	180	67	166	33,79750575
02.06.2010	170	49	148	181	60	156	15,10561783
02.06.2010	172	19	106	181	60	156	1,28917994
02.06.2010	173	16	105	181	60	156	68,44145111
02.06.2010	174	125	211	181	60	156	46,4001617
02.06.2010	175	52	150	181	60	156	60,97685134
02.06.2010	176	37	136	181	60	156	42,3533424
02.06.2010	177	13	97	181	60	156	90,01189001
02.06.2010	178	26	122	181	60	156	31,51691604
02.06.2010	179	54	153	181	60	156	46,79327872
02.06.2010	180	67	166	181	60	156	13,81413016
02.06.2010	170	49	148	182	13	96	54,35891611
02.06.2010	172	19	106	182	13	96	67,13672041
02.06.2010	173	16	105	182	13	96	0,134406299
02.06.2010	174	125	211	182	13	96	112,546237
02.06.2010	175	52	150	182	13	96	8,17395866
02.06.2010	176	37	136	182	13	96	26,08053729
02.06.2010	177	13	97	182	13	96	29,4417299
02.06.2010	178	26	122	182	13	96	37,98969756
02.06.2010	179	54	153	182	13	96	22,43558818

02.06.2010	180	67	166	182	13	96	55,91172838
02.06.2010	181	60	156	182	13	96	68,37391283
02.06.2010	170	49	148	191	128	203	50,81109243
02.06.2010	172	19	106	191	128	203	63,4963442
02.06.2010	173	16	105	191	128	203	3,792068373
02.06.2010	174	125	211	191	128	203	108,8147876
02.06.2010	175	52	150	191	128	203	4,567837206
02.06.2010	176	37	136	191	128	203	22,50031312
02.06.2010	177	13	97	191	128	203	31,32128234
02.06.2010	178	26	122	191	128	203	34,54790995
02.06.2010	179	54	153	191	128	203	19,12824071
02.06.2010	180	67	166	191	128	203	52,36837531
02.06.2010	181	60	156	191	128	203	64,72874
02.06.2010	182	13	96	191	128	203	3,736881898
02.06.2010	170	49	148	193	171	232	43,27966338
02.06.2010	172	19	106	193	171	232	29,4904697
02.06.2010	173	16	105	193	171	232	94,85485962
02.06.2010	174	125	211	193	171	232	18,20746539
02.06.2010	175	52	150	193	171	232	86,99576897
02.06.2010	176	37	136	193	171	232	69,26985667
02.06.2010	177	13	97	193	171	232	113,3493838
02.06.2010	178	26	122	193	171	232	59,34810584
02.06.2010	179	54	153	193	171	232	74,12735915
02.06.2010	180	67	166	193	171	232	42,02190988
02.06.2010	181	60	156	193	171	232	28,21011817
02.06.2010	182	13	96	193	171	232	94,79561134
02.06.2010	191	128	203	193	171	232	91,07546822
02.06.2010	170	49	148	194	81	184	62,86925616
02.06.2010	172	19	106	194	81	184	49,06050883
02.06.2010	173	16	105	194	81	184	114,1621508
02.06.2010	174	125	211	194	81	184	1,678734147
02.06.2010	175	52	150	194	81	184	106,2197605
02.06.2010	176	37	136	194	81	184	88,76424465

02.06.2010	177	13	97	194	81	184	131,5237585
02.06.2010	178	26	122	194	81	184	78,97305188
02.06.2010	179	54	153	194	81	184	93,68630103
02.06.2010	180	67	166	194	81	184	61,58683807
02.06.2010	181	60	156	194	81	184	47,77420835
02.06.2010	182	13	96	194	81	184	114,1054076
02.06.2010	191	128	203	194	81	184	110,375154
02.06.2010	193	171	232	194	81	184	19,62654159
02.06.2010	170	49	148	172	19	106	14,06200822
02.06.2010	170	49	148	173	16	105	57,10377839
02.06.2010	172	19	106	173	16	105	45,74318541
02.06.2010	170	49	148	174	125	211	277,7674556
02.06.2010	172	19	106	174	125	211	289,0919544
02.06.2010	173	16	105	174	125	211	334,653725
02.06.2010	170	49	148	175	52	150	56,85886913
02.06.2010	172	19	106	175	52	150	45,44970445
02.06.2010	173	16	105	175	52	150	0,414268038
02.06.2010	174	125	211	175	52	150	334,3833042
02.06.2010	170	49	148	176	37	136	30,26187019
02.06.2010	172	19	106	176	37	136	17,53422781
02.06.2010	173	16	105	176	37	136	28,44400612
02.06.2010	174	125	211	176	37	136	306,6172568
02.06.2010	175	52	150	176	37	136	28,12979686
02.06.2010	170	49	148	177	13	97	94,85843048
02.06.2010	172	19	106	177	13	97	84,81005573
02.06.2010	173	16	105	177	13	97	39,72064335
02.06.2010	174	125	211	177	13	97	372,4425006
02.06.2010	175	52	150	177	13	97	40,07449547
02.06.2010	176	37	136	177	13	97	67,9284573
02.06.2010	170	49	148	178	26	122	22,38298745
02.06.2010	172	19	106	178	26	122	10,31343952
02.06.2010	173	16	105	178	26	122	35,50870979
02.06.2010	174	125	211	178	26	122	299,1973296

02.06.2010	175	52	150	178	26	122	35,22419625
02.06.2010	176	37	136	178	26	122	7,893438374
02.06.2010	177	13	97	178	26	122	74,49729098
02.06.2010	170	49	148	179	54	153	54,04704445
02.06.2010	172	19	106	179	54	153	42,29080931
02.06.2010	173	16	105	179	54	153	4,078959945
02.06.2010	174	125	211	179	54	153	331,3428802
02.06.2010	175	52	150	179	54	153	3,685772673
02.06.2010	176	37	136	179	54	153	24,85099429
02.06.2010	177	13	97	179	54	153	43,6452253
02.06.2010	178	26	122	179	54	153	32,14727847
02.06.2010	170	49	148	180	67	166	14,03114224
02.06.2010	172	19	106	180	67	166	0,23279854
02.06.2010	173	16	105	180	67	166	45,91091779
02.06.2010	174	125	211	180	67	166	288,9390035
02.06.2010	175	52	150	180	67	166	45,61643686
02.06.2010	176	37	136	180	67	166	17,68273106
02.06.2010	177	13	97	180	67	166	84,99293723
02.06.2010	178	26	122	180	67	166	10,49574656
02.06.2010	179	54	153	180	67	166	42,4513314
02.06.2010	170	49	148	181	60	156	52,50024315
02.06.2010	172	19	106	181	60	156	66,5603013
02.06.2010	173	16	105	181	60	156	105,1844158
02.06.2010	174	125	211	181	60	156	238,7106402
02.06.2010	175	52	150	181	60	156	105,0348471
02.06.2010	176	37	136	181	60	156	81,6326596
02.06.2010	177	13	97	181	60	156	138,2791976
02.06.2010	178	26	122	181	60	156	73,85953321
02.06.2010	179	54	153	181	60	156	102,9421527
02.06.2010	180	67	166	181	60	156	66,53111845
02.06.2010	170	49	148	182	13	96	56,99540312
02.06.2010	172	19	106	182	13	96	45,64543616
02.06.2010	173	16	105	182	13	96	0,134406299

02.06.2010	174	125	211	182	13	96	334,5500783
02.06.2010	175	52	150	182	13	96	0,4032189
02.06.2010	176	37	136	182	13	96	28,35224358
02.06.2010	177	13	97	182	13	96	39,79765774
02.06.2010	178	26	122	182	13	96	35,40859931
02.06.2010	179	54	153	182	13	96	4,034428479
02.06.2010	180	67	166	182	13	96	45,81323068
02.06.2010	181	60	156	182	13	96	105,0648123
02.06.2010	170	49	148	191	128	203	56,84552343
02.06.2010	172	19	106	191	128	203	45,40019203
02.06.2010	173	16	105	191	128	203	0,615927043
02.06.2010	174	125	211	191	128	203	334,350604
02.06.2010	175	52	150	191	128	203	0,23279854
02.06.2010	176	37	136	191	128	203	28,06373287
02.06.2010	177	13	97	191	128	203	40,18006592
02.06.2010	178	26	122	191	128	203	35,18224503
02.06.2010	179	54	153	191	128	203	3,535677726
02.06.2010	180	67	166	191	128	203	45,56621359
02.06.2010	181	60	156	191	128	203	105,082263
02.06.2010	182	13	96	191	128	203	0,630421427
02.06.2010	170	49	148	193	171	232	32,67607614
02.06.2010	172	19	106	193	171	232	46,62503981
02.06.2010	173	16	105	193	171	232	88,10607201
02.06.2010	174	125	211	193	171	232	249,5490682
02.06.2010	175	52	150	193	171	232	87,91014582
02.06.2010	176	37	136	193	171	232	62,6966132
02.06.2010	177	13	97	193	171	232	123,6317338
02.06.2010	178	26	122	193	171	232	54,81009149
02.06.2010	179	54	153	193	171	232	85,45413659
02.06.2010	180	67	166	193	171	232	46,57027979
02.06.2010	181	60	156	193	171	232	21,01063251
02.06.2010	182	13	96	193	171	232	87,99056014
02.06.2010	191	128	203	193	171	232	87,92920337

02.06.2010	170	49	148	194	81	184	60,83945371
02.06.2010	172	19	106	194	81	184	74,89784278
02.06.2010	173	16	105	194	81	184	113,8341221
02.06.2010	174	125	211	194	81	184	231,2118685
02.06.2010	175	52	150	194	81	184	113,6847703
02.06.2010	176	37	136	194	81	184	90,17671147
02.06.2010	177	13	97	194	81	184	146,7825719
02.06.2010	178	26	122	194	81	184	82,38097527
02.06.2010	179	54	153	194	81	184	111,5896113
02.06.2010	180	67	166	194	81	184	74,861534
02.06.2010	181	60	156	194	81	184	8,649648482
02.06.2010	182	13	96	194	81	184	113,7144816
02.06.2010	191	128	203	194	81	184	113,7322332
02.06.2010	193	171	232	194	81	184	28,73168486
02.06.2010	170	49	148	172	19	106	1,383797553
02.06.2010	170	49	148	173	16	105	69,2122628
02.06.2010	172	19	106	173	16	105	69,12078379
02.06.2010	170	49	148	174	125	211	224,1742365
02.06.2010	172	19	106	174	125	211	223,9449157
02.06.2010	173	16	105	174	125	211	291,7942353
02.06.2010	170	49	148	175	52	150	39,5586306
02.06.2010	172	19	106	175	52	150	39,2226969
02.06.2010	173	16	105	175	52	150	31,20629482
02.06.2010	174	125	211	175	52	150	260,6294461
02.06.2010	170	49	148	176	37	136	27,60372963
02.06.2010	172	19	106	176	37	136	27,15439719
02.06.2010	173	16	105	176	37	136	43,33983191
02.06.2010	174	125	211	176	37	136	248,4581798
02.06.2010	175	52	150	176	37	136	12,33722448
02.06.2010	170	49	148	177	13	97	105,3492492
02.06.2010	172	19	106	177	13	97	105,6301411
02.06.2010	173	16	105	177	13	97	43,01568586
02.06.2010	174	125	211	177	13	97	329,4945017

02.06.2010	175	52	150	177	13	97	72,07919267
02.06.2010	176	37	136	177	13	97	83,12396709
02.06.2010	170	49	148	178	26	122	24,38798515
02.06.2010	172	19	106	178	26	122	23,91752459
02.06.2010	173	16	105	178	26	122	46,44919159
02.06.2010	174	125	211	178	26	122	245,3759194
02.06.2010	175	52	150	178	26	122	15,56010536
02.06.2010	176	37	136	178	26	122	3,25363202
02.06.2010	177	13	97	178	26	122	85,85838248
02.06.2010	170	49	148	179	54	153	63,79826901
02.06.2010	172	19	106	179	54	153	63,68462103
02.06.2010	173	16	105	179	54	153	5,528659009
02.06.2010	174	125	211	179	54	153	286,2664246
02.06.2010	175	52	150	179	54	153	25,68391504
02.06.2010	176	37	136	179	54	153	37,81298564
02.06.2010	177	13	97	179	54	153	47,86789373
02.06.2010	178	26	122	179	54	153	40,92633209
02.06.2010	170	49	148	180	67	166	12,6062792
02.06.2010	172	19	106	180	67	166	11,961028
02.06.2010	173	16	105	180	67	166	58,17293471
02.06.2010	174	125	211	180	67	166	233,9284601
02.06.2010	175	52	150	180	67	166	27,6240099
02.06.2010	176	37	136	180	67	166	15,36646718
02.06.2010	177	13	97	180	67	166	96,34231951
02.06.2010	178	26	122	180	67	166	12,11223758
02.06.2010	179	54	153	180	67	166	52,67492468
02.06.2010	170	49	148	181	60	156	39,51796649
02.06.2010	172	19	106	181	60	156	40,15274328
02.06.2010	173	16	105	181	60	156	106,8729139
02.06.2010	174	125	211	181	60	156	193,6657199
02.06.2010	175	52	150	181	60	156	78,68561964
02.06.2010	176	37	136	181	60	156	67,02927207
02.06.2010	177	13	97	181	60	156	138,7168737

02.06.2010	178	26	122	181	60	156	63,84822701
02.06.2010	179	54	153	181	60	156	101,6675053
02.06.2010	180	67	166	181	60	156	52,09887087
02.06.2010	170	49	148	182	13	96	69,04808891
02.06.2010	172	19	106	182	13	96	68,95396877
02.06.2010	173	16	105	182	13	96	0,23279854
02.06.2010	174	125	211	182	13	96	291,6077794
02.06.2010	175	52	150	182	13	96	31,01525482
02.06.2010	176	37	136	182	13	96	43,15467947
02.06.2010	177	13	97	182	13	96	43,22766549
02.06.2010	178	26	122	182	13	96	46,26623298
02.06.2010	179	54	153	182	13	96	5,341699348
02.06.2010	180	67	166	182	13	96	57,99659096
02.06.2010	181	60	156	182	13	96	106,7288852
02.06.2010	170	49	148	191	128	203	67,65525049
02.06.2010	172	19	106	191	128	203	67,60008914
02.06.2010	173	16	105	191	128	203	2,366467108
02.06.2010	174	125	211	191	128	203	290,5784939
02.06.2010	175	52	150	191	128	203	30,15557429
02.06.2010	176	37	136	191	128	203	42,14071567
02.06.2010	177	13	97	191	128	203	43,09519562
02.06.2010	178	26	122	191	128	203	45,20289887
02.06.2010	179	54	153	191	128	203	4,774735328
02.06.2010	180	67	166	191	128	203	56,80093561
02.06.2010	181	60	156	191	128	203	105,0746125
02.06.2010	182	13	96	191	128	203	2,366467108
02.06.2010	170	49	148	193	171	232	19,79038634
02.06.2010	172	19	106	193	171	232	20,69115033
02.06.2010	173	16	105	193	171	232	86,21307688
02.06.2010	174	125	211	193	171	232	211,6719581
02.06.2010	175	52	150	193	171	232	58,1880716
02.06.2010	176	37	136	193	171	232	46,77455103
02.06.2010	177	13	97	193	171	232	118,9308558

02.06.2010	178	26	122	193	171	232	43,65743384
02.06.2010	179	54	153	193	171	232	80,99627412
02.06.2010	180	67	166	193	171	232	32,31150931
02.06.2010	181	60	156	193	171	232	20,67455513
02.06.2010	182	13	96	193	171	232	86,06779592
02.06.2010	191	128	203	193	171	232	84,43005367
02.06.2010	170	49	148	194	81	184	50,45905004
02.06.2010	172	19	106	194	81	184	51,23419358
02.06.2010	173	16	105	194	81	184	115,9393604
02.06.2010	174	125	211	194	81	184	190,5027832
02.06.2010	175	52	150	194	81	184	88,87682005
02.06.2010	176	37	136	194	81	184	77,59633178
02.06.2010	177	13	97	194	81	184	145,5780315
02.06.2010	178	26	122	194	81	184	74,48607485
02.06.2010	179	54	153	194	81	184	110,8871931
02.06.2010	180	67	166	194	81	184	63,05317414
02.06.2010	181	60	156	194	81	184	12,22469446
02.06.2010	182	13	96	194	81	184	115,8071542
02.06.2010	191	128	203	194	81	184	114,0309734
02.06.2010	193	171	232	194	81	184	30,82933059
02.06.2010	170	49	148	172	19	106	78,51870635
02.06.2010	170	49	148	173	16	105	120,1450991
02.06.2010	172	19	106	173	16	105	49,67363004
02.06.2010	170	49	148	174	125	211	235,2058213
02.06.2010	172	19	106	174	125	211	308,2819086
02.06.2010	173	16	105	174	125	211	354,438944
02.06.2010	170	49	148	175	52	150	82,6651194
02.06.2010	172	19	106	175	52	150	5,112742155
02.06.2010	173	16	105	175	52	150	44,58299717
02.06.2010	174	125	211	175	52	150	313,1409646
02.06.2010	170	49	148	176	37	136	36,55814297
02.06.2010	172	19	106	176	37	136	42,07056744
02.06.2010	173	16	105	176	37	136	86,48802587

02.06.2010	174	125	211	176	37	136	268,0415138
02.06.2010	175	52	150	176	37	136	46,39480808
02.06.2010	170	49	148	177	13	97	116,4405458
02.06.2010	172	19	106	177	13	97	47,10744279
02.06.2010	173	16	105	177	13	97	4,02658484
02.06.2010	174	125	211	177	13	97	350,903524
02.06.2010	175	52	150	177	13	97	42,06079748
02.06.2010	176	37	136	177	13	97	83,05200082
02.06.2010	170	49	148	178	26	122	36,88026579
02.06.2010	172	19	106	178	26	122	41,74770885
02.06.2010	173	16	105	178	26	122	86,19081042
02.06.2010	174	125	211	178	26	122	268,3451146
02.06.2010	175	52	150	178	26	122	46,07313866
02.06.2010	176	37	136	178	26	122	0,315210714
02.06.2010	177	13	97	178	26	122	82,75799836
02.06.2010	170	49	148	179	54	153	120,2337038
02.06.2010	172	19	106	179	54	153	49,89307771
02.06.2010	173	16	105	179	54	153	0,329226853
02.06.2010	174	125	211	179	54	153	354,5587751
02.06.2010	175	52	150	179	54	153	44,80490001
02.06.2010	176	37	136	179	54	153	86,62045469
02.06.2010	177	13	97	179	54	153	3,998446236
02.06.2010	178	26	122	179	54	153	86,32369521
02.06.2010	170	49	148	180	67	166	78,42443477
02.06.2010	172	19	106	180	67	166	0,164613426
02.06.2010	173	16	105	180	67	166	49,65726197
02.06.2010	174	125	211	180	67	166	308,2340587
02.06.2010	175	52	150	180	67	166	5,113625413
02.06.2010	176	37	136	180	67	166	41,98298626
02.06.2010	177	13	97	180	67	166	47,08068703
02.06.2010	178	26	122	180	67	166	41,66009938
02.06.2010	179	54	153	180	67	166	49,87578561
02.06.2010	170	49	148	181	60	156	1,444476955

02.06.2010	172	19	106	181	60	156	79,88100094
02.06.2010	173	16	105	181	60	156	121,3229245
02.06.2010	174	125	211	181	60	156	234,1730573
02.06.2010	175	52	150	181	60	156	84,00888344
02.06.2010	176	37	136	181	60	156	37,94616207
02.06.2010	177	13	97	181	60	156	117,6072789
02.06.2010	178	26	122	181	60	156	38,26804879
02.06.2010	179	54	153	181	60	156	121,4093677
02.06.2010	180	67	166	181	60	156	79,78601819
02.06.2010	170	49	148	182	13	96	82,64801742
02.06.2010	172	19	106	182	13	96	5,06124943
02.06.2010	173	16	105	182	13	96	44,6381716
02.06.2010	174	125	211	182	13	96	313,1057863
02.06.2010	175	52	150	182	13	96	0
02.06.2010	176	37	136	182	13	96	46,37299786
02.06.2010	177	13	97	182	13	96	42,11873988
02.06.2010	178	26	122	182	13	96	46,05127417
02.06.2010	179	54	153	182	13	96	44,86030489
02.06.2010	180	67	166	182	13	96	5,063925688
02.06.2010	181	60	156	182	13	96	83,99216262
02.06.2010	170	49	148	191	128	203	81,89833677
02.06.2010	172	19	106	191	128	203	4,029948269
02.06.2010	173	16	105	191	128	203	45,68726958
02.06.2010	174	125	211	191	128	203	312,1732365
02.06.2010	175	52	150	191	128	203	1,108342742
02.06.2010	176	37	136	191	128	203	45,57334926
02.06.2010	177	13	97	191	128	203	43,1699561
02.06.2010	178	26	122	191	128	203	45,25122992
02.06.2010	179	54	153	191	128	203	45,90973733
02.06.2010	180	67	166	191	128	203	4,038903721
02.06.2010	181	60	156	191	128	203	83,2471001
02.06.2010	182	13	96	191	128	203	1,04974676
02.06.2010	170	49	148	193	171	232	0,134406299

02.06.2010	172	19	106	193	171	232	78,4301933
02.06.2010	173	16	105	193	171	232	120,0635385
02.06.2010	174	125	211	193	171	232	235,2810017
02.06.2010	175	52	150	193	171	232	82,57711303
02.06.2010	176	37	136	193	171	232	36,46921209
02.06.2010	177	13	97	193	171	232	116,3594936
02.06.2010	178	26	122	193	171	232	36,79125426
02.06.2010	179	54	153	193	171	232	120,1522786
02.06.2010	180	67	166	193	171	232	78,33604581
02.06.2010	181	60	156	193	171	232	1,532467601
02.06.2010	182	13	96	193	171	232	82,56004753
02.06.2010	191	128	203	193	171	232	81,81033362
02.06.2010	170	49	148	194	81	184	46,53855739
02.06.2010	172	19	106	194	81	184	124,3434806
02.06.2010	173	16	105	194	81	184	162,5062212
02.06.2010	174	125	211	194	81	184	200,2366318
02.06.2010	175	52	150	194	81	184	128,2120568
02.06.2010	176	37	136	194	81	184	82,82018699
02.06.2010	177	13	97	194	81	184	158,6162907
02.06.2010	178	26	122	194	81	184	83,13874399
02.06.2010	179	54	153	194	81	184	162,5500145
02.06.2010	180	67	166	194	81	184	124,2388325
02.06.2010	181	60	156	194	81	184	45,10558078
02.06.2010	182	13	96	194	81	184	128,2010662
02.06.2010	191	128	203	194	81	184	127,5217621
02.06.2010	193	171	232	194	81	184	46,62775191
03.06.2010	170	49	148	172	19	106	60,58534489
03.06.2010	170	49	148	173	16	105	88,08546332
03.06.2010	172	19	106	173	16	105	30,2736578
03.06.2010	170	49	148	174	125	211	242,5889809
03.06.2010	172	19	106	174	125	211	292,0126967
03.06.2010	173	16	105	174	125	211	322,2760391
03.06.2010	170	49	148	175	52	150	60,39585634

03.06.2010	172	19	106	175	52	150	0,190079212
03.06.2010	173	16	105	175	52	150	30,44176591
03.06.2010	174	125	211	175	52	150	291,8471637
03.06.2010	170	49	148	176	37	136	32,05090064
03.06.2010	172	19	106	176	37	136	28,53911351
03.06.2010	173	16	105	176	37	136	56,68720143
03.06.2010	174	125	211	176	37	136	268,382155
03.06.2010	175	52	150	176	37	136	28,34985411
03.06.2010	170	49	148	177	13	97	113,0188762
03.06.2010	172	19	106	177	13	97	61,48555713
03.06.2010	173	16	105	177	13	97	32,99736253
03.06.2010	174	125	211	177	13	97	352,2589377
03.06.2010	175	52	150	177	13	97	61,62693741
03.06.2010	176	37	136	177	13	97	84,07734497
03.06.2010	170	49	148	178	26	122	31,38236
03.06.2010	172	19	106	178	26	122	29,20392505
03.06.2010	173	16	105	178	26	122	57,38857837
03.06.2010	174	125	211	178	26	122	267,709296
03.06.2010	175	52	150	178	26	122	29,01433251
03.06.2010	176	37	136	178	26	122	0,698395621
03.06.2010	177	13	97	178	26	122	84,7641405
03.06.2010	170	49	148	179	54	153	111,3750244
03.06.2010	172	19	106	179	54	153	60,28111313
03.06.2010	173	16	105	179	54	153	32,15991978
03.06.2010	174	125	211	179	54	153	350,8109351
03.06.2010	175	52	150	179	54	153	60,41948147
03.06.2010	176	37	136	179	54	153	82,56858072
03.06.2010	177	13	97	179	54	153	1,840434058
03.06.2010	178	26	122	179	54	153	83,25355576
03.06.2010	170	49	148	180	67	166	62,98258275
03.06.2010	172	19	106	180	67	166	2,841668329
03.06.2010	173	16	105	180	67	166	27,43765611
03.06.2010	174	125	211	180	67	166	294,8522409

03.06.2010	175	52	150	180	67	166	3,005416231
03.06.2010	176	37	136	180	67	166	30,93696114
03.06.2010	177	13	97	180	67	166	58,70248041
03.06.2010	178	26	122	180	67	166	31,61220744
03.06.2010	179	54	153	180	67	166	57,51207723
03.06.2010	170	49	148	181	60	156	2,033936391
03.06.2010	172	19	106	181	60	156	61,8673527
03.06.2010	173	16	105	181	60	156	89,61403346
03.06.2010	174	125	211	181	60	156	240,6013741
03.06.2010	175	52	150	181	60	156	61,67784871
03.06.2010	176	37	136	181	60	156	33,36878331
03.06.2010	177	13	97	181	60	156	114,797852
03.06.2010	178	26	122	181	60	156	32,6905853
03.06.2010	179	54	153	181	60	156	113,1612848
03.06.2010	180	67	166	181	60	156	64,30207253
03.06.2010	170	49	148	182	13	96	88,05341288
03.06.2010	172	19	106	182	13	96	30,23454704
03.06.2010	173	16	105	182	13	96	0
03.06.2010	174	125	211	182	13	96	322,2364762
03.06.2010	175	52	150	182	13	96	30,40272286
03.06.2010	176	37	136	182	13	96	56,6528533
03.06.2010	177	13	97	182	13	96	33,03853394
03.06.2010	178	26	122	182	13	96	57,35425656
03.06.2010	179	54	153	182	13	96	32,20089965
03.06.2010	180	67	166	182	13	96	27,39845308
03.06.2010	181	60	156	182	13	96	89,58157202
03.06.2010	170	49	148	191	128	203	87,08294014
03.06.2010	172	19	106	191	128	203	30,03702656
03.06.2010	173	16	105	191	128	203	2,137863074
03.06.2010	174	125	211	191	128	203	322,0246483
03.06.2010	175	52	150	191	128	203	30,19837683
03.06.2010	176	37	136	191	128	203	55,87391294
03.06.2010	177	13	97	191	128	203	32,367091

03.06.2010	178	26	122	191	128	203	56,57611242
03.06.2010	179	54	153	191	128	203	31,42852144
03.06.2010	180	67	166	191	128	203	27,19710731
03.06.2010	181	60	156	191	128	203	88,6399103
03.06.2010	182	13	96	191	128	203	2,144191247
03.06.2010	170	49	148	193	171	232	51,81768156
03.06.2010	172	19	106	193	171	232	112,3121271
03.06.2010	173	16	105	193	171	232	139,762648
03.06.2010	174	125	211	193	171	232	202,1285531
03.06.2010	175	52	150	193	171	232	112,1224894
03.06.2010	176	37	136	193	171	232	83,82180763
03.06.2010	177	13	97	193	171	232	163,135643
03.06.2010	178	26	122	193	171	232	83,14428465
03.06.2010	179	54	153	193	171	232	161,4187767
03.06.2010	180	67	166	193	171	232	114,7556707
03.06.2010	181	60	156	193	171	232	50,45376906
03.06.2010	182	13	96	193	171	232	139,7315263
03.06.2010	191	128	203	193	171	232	138,6881551
03.06.2010	170	49	148	194	81	184	51,29427624
03.06.2010	172	19	106	194	81	184	111,8781684
03.06.2010	173	16	105	194	81	184	138,8353321
03.06.2010	174	125	211	194	81	184	205,8497754
03.06.2010	175	52	150	194	81	184	111,6888047
03.06.2010	176	37	136	194	81	184	83,34046848
03.06.2010	177	13	97	194	81	184	161,4984958
03.06.2010	178	26	122	194	81	184	82,67440653
03.06.2010	179	54	153	194	81	184	159,7643764
03.06.2010	180	67	166	194	81	184	114,2599805
03.06.2010	181	60	156	194	81	184	50,07441946
03.06.2010	182	13	96	194	81	184	138,8051413
03.06.2010	191	128	203	194	81	184	137,7002546
03.06.2010	193	171	232	194	81	184	4,69461023
03.06.2010	170	49	148	172	19	106	55,72125987

03.06.2010	170	49	148	173	16	105	111,3902296
03.06.2010	172	19	106	173	16	105	60,36017684
03.06.2010	170	49	148	174	125	211	227,3653296
03.06.2010	172	19	106	174	125	211	280,2781472
03.06.2010	173	16	105	174	125	211	338,6297175
03.06.2010	170	49	148	175	52	150	76,19616309
03.06.2010	172	19	106	175	52	150	20,49905384
03.06.2010	173	16	105	175	52	150	43,69114479
03.06.2010	174	125	211	175	52	150	300,2770301
03.06.2010	170	49	148	176	37	136	32,51604809
03.06.2010	172	19	106	176	37	136	23,99726529
03.06.2010	173	16	105	176	37	136	83,15150868
03.06.2010	174	125	211	176	37	136	256,3888511
03.06.2010	175	52	150	176	37	136	44,39010966
03.06.2010	170	49	148	177	13	97	102,8915122
03.06.2010	172	19	106	177	13	97	51,08907044
03.06.2010	173	16	105	177	13	97	9,392106047
03.06.2010	174	125	211	177	13	97	329,9348075
03.06.2010	175	52	150	177	13	97	34,34284802
03.06.2010	176	37	136	177	13	97	74,09762142
03.06.2010	170	49	148	178	26	122	31,19833405
03.06.2010	172	19	106	178	26	122	25,18137092
03.06.2010	173	16	105	178	26	122	84,12320556
03.06.2010	174	125	211	178	26	122	255,2814418
03.06.2010	175	52	150	178	26	122	45,60752553
03.06.2010	176	37	136	178	26	122	1,337325799
03.06.2010	177	13	97	178	26	122	75,0983098
03.06.2010	170	49	148	179	54	153	103,9085447
03.06.2010	172	19	106	179	54	153	52,44688136
03.06.2010	173	16	105	179	54	153	7,925414238
03.06.2010	174	125	211	179	54	153	331,0322936
03.06.2010	175	52	150	179	54	153	35,92673105
03.06.2010	176	37	136	179	54	153	75,33344217

03.06.2010	177	13	97	179	54	153	1,692132079
03.06.2010	178	26	122	179	54	153	76,32041346
03.06.2010	170	49	148	180	67	166	55,64745459
03.06.2010	172	19	106	180	67	166	0,23279854
03.06.2010	173	16	105	180	67	166	60,30568189
03.06.2010	174	125	211	180	67	166	280,2682372
03.06.2010	175	52	150	180	67	166	20,56196759
03.06.2010	176	37	136	180	67	166	23,96732296
03.06.2010	177	13	97	180	67	166	51,04078133
03.06.2010	178	26	122	180	67	166	25,14619387
03.06.2010	179	54	153	180	67	166	52,3941548
03.06.2010	170	49	148	181	60	156	2,944694493
03.06.2010	172	19	106	181	60	156	58,46210557
03.06.2010	173	16	105	181	60	156	113,6922783
03.06.2010	174	125	211	181	60	156	225,1987281
03.06.2010	175	52	150	181	60	156	78,91767507
03.06.2010	176	37	136	181	60	156	35,38384652
03.06.2010	177	13	97	181	60	156	105,2609869
03.06.2010	178	26	122	181	60	156	34,06198798
03.06.2010	179	54	153	181	60	156	106,2550447
03.06.2010	180	67	166	181	60	156	58,3840298
03.06.2010	170	49	148	182	13	96	97,22756088
03.06.2010	172	19	106	182	13	96	44,29396268
03.06.2010	173	16	105	182	13	96	16,79218328
03.06.2010	174	125	211	182	13	96	323,8798624
03.06.2010	175	52	150	182	13	96	26,90342788
03.06.2010	176	37	136	182	13	96	67,67740921
03.06.2010	177	13	97	182	13	96	7,51474265
03.06.2010	178	26	122	182	13	96	68,72520199
03.06.2010	179	54	153	182	13	96	9,165287697
03.06.2010	180	67	166	182	13	96	44,26101698
03.06.2010	181	60	156	182	13	96	99,68028273
03.06.2010	170	49	148	191	128	203	79,94644443

03.06.2010	172	19	106	191	128	203	24,52427011
03.06.2010	173	16	105	191	128	203	38,72021328
03.06.2010	174	125	211	191	128	203	304,6929752
03.06.2010	175	52	150	191	128	203	4,977571778
03.06.2010	176	37	136	191	128	203	48,52104658
03.06.2010	177	13	97	191	128	203	29,38046088
03.06.2010	178	26	122	191	128	203	49,70380589
03.06.2010	179	54	153	191	128	203	30,972123
03.06.2010	180	67	166	191	128	203	24,55776342
03.06.2010	181	60	156	191	128	203	82,61866809
03.06.2010	182	13	96	191	128	203	21,93006733
03.06.2010	170	49	148	193	171	232	38,71321434
03.06.2010	172	19	106	193	171	232	93,35937444
03.06.2010	173	16	105	193	171	232	144,9252094
03.06.2010	174	125	211	193	171	232	198,2738485
03.06.2010	175	52	150	193	171	232	113,5790097
03.06.2010	176	37	136	193	171	232	70,99375876
03.06.2010	177	13	97	193	171	232	137,1319096
03.06.2010	178	26	122	193	171	232	69,6627995
03.06.2010	179	54	153	193	171	232	137,930794
03.06.2010	180	67	166	193	171	232	93,25870007
03.06.2010	181	60	156	193	171	232	35,77707873
03.06.2010	182	13	96	193	171	232	132,2635163
03.06.2010	191	128	203	193	171	232	116,9348713
03.06.2010	170	49	148	194	81	184	41,58371053
03.06.2010	172	19	106	194	81	184	96,37475311
03.06.2010	173	16	105	194	81	184	147,991041
03.06.2010	174	125	211	194	81	184	195,4502151
03.06.2010	175	52	150	194	81	184	116,6117764
03.06.2010	176	37	136	194	81	184	73,93122345
03.06.2010	177	13	97	194	81	184	140,2037463
03.06.2010	178	26	122	194	81	184	72,6013641
03.06.2010	179	54	153	194	81	184	141,0013374

03.06.2010	180	67	166	194	81	184	96,27535552
03.06.2010	181	60	156	194	81	184	38,65530793
03.06.2010	182	13	96	194	81	184	135,3370999
03.06.2010	191	128	203	194	81	184	119,9818849
03.06.2010	193	171	232	194	81	184	3,073763646
03.06.2010	170	49	148	172	19	106	74,98022618
03.06.2010	170	49	148	173	16	105	107,1957443
03.06.2010	172	19	106	173	16	105	34,69648584
03.06.2010	170	49	148	174	125	211	218,2638302
03.06.2010	172	19	106	174	125	211	289,5188137
03.06.2010	173	16	105	174	125	211	323,6837886
03.06.2010	170	49	148	175	52	150	72,77533335
03.06.2010	172	19	106	175	52	150	2,220756536
03.06.2010	173	16	105	175	52	150	36,59160603
03.06.2010	174	125	211	175	52	150	287,4582669
03.06.2010	170	49	148	176	37	136	30,95286917
03.06.2010	172	19	106	176	37	136	45,63168111
03.06.2010	173	16	105	176	37	136	79,49834395
03.06.2010	174	125	211	176	37	136	244,1854173
03.06.2010	175	52	150	176	37	136	43,49990907
03.06.2010	170	49	148	177	13	97	30,54322172
03.06.2010	172	19	106	177	13	97	46,50127776
03.06.2010	173	16	105	177	13	97	80,46605578
03.06.2010	174	125	211	177	13	97	243,2230456
03.06.2010	175	52	150	177	13	97	44,38156265
03.06.2010	176	37	136	177	13	97	1,224499668
03.06.2010	170	49	148	179	54	153	105,5371071
03.06.2010	172	19	106	179	54	153	33,39502972
03.06.2010	173	16	105	179	54	153	1,85266299
03.06.2010	174	125	211	179	54	153	322,1640506
03.06.2010	175	52	150	179	54	153	35,24624229
03.06.2010	176	37	136	179	54	153	77,98455753
03.06.2010	177	13	97	179	54	153	78,9625287

03.06.2010	170	49	148	180	67	166	38,17233568
03.06.2010	172	19	106	180	67	166	39,30562814
03.06.2010	173	16	105	180	67	166	73,60386457
03.06.2010	174	125	211	180	67	166	250,2247901
03.06.2010	175	52	150	180	67	166	37,23406276
03.06.2010	176	37	136	180	67	166	7,225510672
03.06.2010	177	13	97	180	67	166	7,691752079
03.06.2010	179	54	153	180	67	166	72,15196332
03.06.2010	170	49	148	181	60	156	10,54425775
03.06.2010	172	19	106	181	60	156	83,28799554
03.06.2010	173	16	105	181	60	156	114,433448
03.06.2010	174	125	211	181	60	156	213,419514
03.06.2010	175	52	150	181	60	156	81,07140228
03.06.2010	176	37	136	181	60	156	40,68583265
03.06.2010	177	13	97	181	60	156	40,40625367
03.06.2010	179	54	153	181	60	156	112,7212199
03.06.2010	180	67	166	181	60	156	47,8974157
03.06.2010	170	49	148	182	13	96	103,4434405
03.06.2010	172	19	106	182	13	96	31,82592468
03.06.2010	173	16	105	182	13	96	4,20543545
03.06.2010	174	125	211	182	13	96	320,2427186
03.06.2010	175	52	150	182	13	96	33,6117883
03.06.2010	176	37	136	182	13	96	76,08750016
03.06.2010	177	13	97	182	13	96	77,07870257
03.06.2010	179	54	153	182	13	96	2,353070097
03.06.2010	180	67	166	182	13	96	70,33919707
03.06.2010	181	60	156	182	13	96	110,5560652
03.06.2010	170	49	148	191	128	203	90,35647824
03.06.2010	172	19	106	191	128	203	15,50078203
03.06.2010	173	16	105	191	128	203	23,55947298
03.06.2010	174	125	211	191	128	203	303,7829771
03.06.2010	175	52	150	191	128	203	17,7166674
03.06.2010	176	37	136	191	128	203	60,58713392

03.06.2010	177	13	97	191	128	203	61,38513912
03.06.2010	179	54	153	191	128	203	22,86902556
03.06.2010	180	67	166	191	128	203	53,96525922
03.06.2010	181	60	156	191	128	203	98,76714137
03.06.2010	182	13	96	191	128	203	22,19006899
03.06.2010	170	49	148	193	171	232	23,87481166
03.06.2010	172	19	106	193	171	232	98,27168802
03.06.2010	173	16	105	193	171	232	129,5355315
03.06.2010	174	125	211	193	171	232	199,322141
03.06.2010	175	52	150	193	171	232	96,05660088
03.06.2010	176	37	136	193	171	232	54,82788665
03.06.2010	177	13	97	193	171	232	54,41090077
03.06.2010	179	54	153	193	171	232	127,8214159
03.06.2010	180	67	166	193	171	232	62,04703465
03.06.2010	181	60	156	193	171	232	15,10442187
03.06.2010	182	13	96	193	171	232	125,6530705
03.06.2010	191	128	203	193	171	232	113,7313993
03.06.2010	170	49	148	194	81	184	44,58279456
03.06.2010	172	19	106	194	81	184	117,7656157
03.06.2010	173	16	105	194	81	184	147,8271953
03.06.2010	174	125	211	194	81	184	187,256799
03.06.2010	175	52	150	194	81	184	115,549558
03.06.2010	176	37	136	194	81	184	75,4263678
03.06.2010	177	13	97	194	81	184	75,07936394
03.06.2010	179	54	153	194	81	184	146,0651459
03.06.2010	180	67	166	194	81	184	82,65080425
03.06.2010	181	60	156	194	81	184	34,84948272
03.06.2010	182	13	96	194	81	184	143,8332677
03.06.2010	191	128	203	194	81	184	133,2656933
03.06.2010	193	171	232	194	81	184	20,98934155
03.06.2010	170	49	148	172	19	106	34,7187369
03.06.2010	170	49	148	173	16	105	78,51393219
03.06.2010	172	19	106	173	16	105	43,80181505

03.06.2010	170	49	148	174	125	211	224,0309914
03.06.2010	172	19	106	174	125	211	258,2765952
03.06.2010	173	16	105	174	125	211	301,4714731
03.06.2010	170	49	148	175	52	150	33,16378423
03.06.2010	172	19	106	175	52	150	1,561660083
03.06.2010	173	16	105	175	52	150	45,3532147
03.06.2010	174	125	211	175	52	150	256,7164902
03.06.2010	170	49	148	176	37	136	10,61299243
03.06.2010	172	19	106	176	37	136	26,8420856
03.06.2010	173	16	105	176	37	136	70,08280098
03.06.2010	174	125	211	176	37	136	231,529667
03.06.2010	175	52	150	176	37	136	25,29482285
03.06.2010	170	49	148	177	13	97	98,28786352
03.06.2010	172	19	106	177	13	97	63,99158608
03.06.2010	173	16	105	177	13	97	22,04652571
03.06.2010	174	125	211	177	13	97	319,1848539
03.06.2010	175	52	150	177	13	97	65,4903575
03.06.2010	176	37	136	177	13	97	89,11664751
03.06.2010	170	49	148	178	26	122	16,14610747
03.06.2010	172	19	106	178	26	122	18,87822488
03.06.2010	173	16	105	178	26	122	62,61963404
03.06.2010	174	125	211	178	26	122	240,1680746
03.06.2010	175	52	150	178	26	122	17,35637632
03.06.2010	176	37	136	178	26	122	11,26369701
03.06.2010	177	13	97	178	26	122	82,83256459
03.06.2010	170	49	148	179	54	153	86,06790086
03.06.2010	172	19	106	179	54	153	51,35446449
03.06.2010	173	16	105	179	54	153	7,554303664
03.06.2010	174	125	211	179	54	153	308,9642144
03.06.2010	175	52	150	179	54	153	52,90642406
03.06.2010	176	37	136	179	54	153	77,61861996
03.06.2010	177	13	97	179	54	153	15,98870137
03.06.2010	178	26	122	179	54	153	70,1647231

03.06.2010	170	49	148	180	67	166	19,2904649
03.06.2010	172	19	106	180	67	166	51,43530838
03.06.2010	173	16	105	180	67	166	94,47108948
03.06.2010	174	125	211	180	67	166	207,0192571
03.06.2010	175	52	150	180	67	166	49,88665046
03.06.2010	176	37	136	180	67	166	24,5941494
03.06.2010	177	13	97	180	67	166	112,8957715
03.06.2010	178	26	122	180	67	166	34,32259024
03.06.2010	179	54	153	180	67	166	101,9809959
03.06.2010	170	49	148	181	60	156	32,50354528
03.06.2010	172	19	106	181	60	156	65,51869423
03.06.2010	173	16	105	181	60	156	108,5366865
03.06.2010	174	125	211	181	60	156	192,9394828
03.06.2010	175	52	150	181	60	156	63,96779758
03.06.2010	176	37	136	181	60	156	38,68193697
03.06.2010	177	13	97	181	60	156	126,7720406
03.06.2010	178	26	122	181	60	156	48,13791231
03.06.2010	179	54	153	181	60	156	116,0386109
03.06.2010	180	67	166	181	60	156	14,09537009
03.06.2010	170	49	148	182	13	96	62,34540209
03.06.2010	172	19	106	182	13	96	27,62826033
03.06.2010	173	16	105	182	13	96	16,17907984
03.06.2010	174	125	211	182	13	96	285,5519491
03.06.2010	175	52	150	182	13	96	29,18180233
03.06.2010	176	37	136	182	13	96	54,05205792
03.06.2010	177	13	97	182	13	96	37,13531992
03.06.2010	178	26	122	182	13	96	46,44141252
03.06.2010	179	54	153	182	13	96	23,72832739
03.06.2010	180	67	166	182	13	96	78,53469482
03.06.2010	181	60	156	182	13	96	92,6218866
03.06.2010	170	49	148	191	128	203	46,61341475
03.06.2010	172	19	106	191	128	203	13,0913392
03.06.2010	173	16	105	191	128	203	33,17617435

03.06.2010	174	125	211	191	128	203	270,6112056
03.06.2010	175	52	150	191	128	203	14,53981733
03.06.2010	176	37	136	191	128	203	39,61248046
03.06.2010	177	13	97	191	128	203	54,61181191
03.06.2010	178	26	122	191	128	203	30,46786564
03.06.2010	179	54	153	191	128	203	40,57467746
03.06.2010	180	67	166	191	128	203	64,1318079
03.06.2010	181	60	156	191	128	203	78,16647945
03.06.2010	182	13	96	191	128	203	17,5032923
03.06.2010	170	49	148	193	171	232	67,24936982
03.06.2010	172	19	106	193	171	232	96,51720046
03.06.2010	173	16	105	193	171	232	136,7476275
03.06.2010	174	125	211	193	171	232	168,8755656
03.06.2010	175	52	150	193	171	232	95,05941979
03.06.2010	176	37	136	193	171	232	70,63725617
03.06.2010	177	13	97	193	171	232	152,3096199
03.06.2010	178	26	122	193	171	232	81,44162282
03.06.2010	179	54	153	193	171	232	143,9535081
03.06.2010	180	67	166	193	171	232	47,98850783
03.06.2010	181	60	156	193	171	232	36,54862942
03.06.2010	182	13	96	193	171	232	121,7267037
03.06.2010	191	128	203	193	171	232	109,5987573
03.06.2010	170	49	148	194	81	184	82,66719543
03.06.2010	172	19	106	194	81	184	112,133163
03.06.2010	173	16	105	194	81	184	152,1009948
03.06.2010	174	125	211	194	81	184	155,6007634
03.06.2010	175	52	150	194	81	184	110,6790383
03.06.2010	176	37	136	194	81	184	86,25989627
03.06.2010	177	13	97	194	81	184	167,2741617
03.06.2010	178	26	122	194	81	184	97,0317609
03.06.2010	179	54	153	194	81	184	159,2584212
03.06.2010	180	67	166	194	81	184	63,37873455
03.06.2010	181	60	156	194	81	184	51,31082625

03.06.2010	182	13	96	194	81	184	137,1969053
03.06.2010	191	128	203	194	81	184	125,2176296
03.06.2010	193	171	232	194	81	184	15,63192072
03.06.2010	170	49	148	172	19	106	65,99431447
03.06.2010	170	49	148	173	16	105	126,3761623
03.06.2010	172	19	106	173	16	105	69,9823286
03.06.2010	170	49	148	174	125	211	198,4816563
03.06.2010	172	19	106	174	125	211	255,1233209
03.06.2010	173	16	105	174	125	211	322,8342519
03.06.2010	170	49	148	175	52	150	85,30904547
03.06.2010	172	19	106	175	52	150	24,94060916
03.06.2010	173	16	105	175	52	150	45,07382951
03.06.2010	174	125	211	175	52	150	278,8441636
03.06.2010	170	49	148	176	37	136	65,01445433
03.06.2010	172	19	106	176	37	136	4,931081214
03.06.2010	173	16	105	176	37	136	67,66940087
03.06.2010	174	125	211	176	37	136	256,2996337
03.06.2010	175	52	150	176	37	136	22,70750582
03.06.2010	170	49	148	177	13	97	132,4959484
03.06.2010	172	19	106	177	13	97	76,41533037
03.06.2010	173	16	105	177	13	97	6,438889474
03.06.2010	174	125	211	177	13	97	329,1664892
03.06.2010	175	52	150	177	13	97	51,5111158
03.06.2010	176	37	136	177	13	97	74,10651962
03.06.2010	170	49	148	178	26	122	48,91973895
03.06.2010	172	19	106	178	26	122	17,26061591
03.06.2010	173	16	105	178	26	122	84,54295156
03.06.2010	174	125	211	178	26	122	238,9319892
03.06.2010	175	52	150	178	26	122	39,93043555
03.06.2010	176	37	136	178	26	122	17,42104756
03.06.2010	177	13	97	178	26	122	90,96332998
03.06.2010	170	49	148	179	54	153	120,1939187
03.06.2010	172	19	106	179	54	153	64,088484

03.06.2010	173	16	105	179	54	153	6,197281956
03.06.2010	174	125	211	179	54	153	316,6546249
03.06.2010	175	52	150	179	54	153	39,15101174
03.06.2010	176	37	136	179	54	153	61,66246987
03.06.2010	177	13	97	179	54	153	12,5311802
03.06.2010	178	26	122	179	54	153	78,45345347
03.06.2010	170	49	148	180	67	166	29,37323532
03.06.2010	172	19	106	180	67	166	36,97443779
03.06.2010	173	16	105	180	67	166	101,6806533
03.06.2010	174	125	211	180	67	166	221,1535488
03.06.2010	175	52	150	180	67	166	58,2554024
03.06.2010	176	37	136	180	67	166	36,5896312
03.06.2010	177	13	97	180	67	166	108,0176029
03.06.2010	178	26	122	180	67	166	19,7327949
03.06.2010	179	54	153	180	67	166	95,50194218
03.06.2010	170	49	148	181	60	156	5,676975899
03.06.2010	172	19	106	181	60	156	69,36152974
03.06.2010	173	16	105	181	60	156	127,685419
03.06.2010	174	125	211	181	60	156	198,6656514
03.06.2010	175	52	150	181	60	156	87,62517677
03.06.2010	176	37	136	181	60	156	68,04649962
03.06.2010	177	13	97	181	60	156	133,7146463
03.06.2010	178	26	122	181	60	156	52,52982695
03.06.2010	179	54	153	181	60	156	121,5281964
03.06.2010	180	67	166	181	60	156	33,37785012
03.06.2010	170	49	148	182	13	96	99,04449395
03.06.2010	172	19	106	182	13	96	36,43452103
03.06.2010	173	16	105	182	13	96	35,29054902
03.06.2010	174	125	211	182	13	96	291,4782445
03.06.2010	175	52	150	182	13	96	13,86666611
03.06.2010	176	37	136	182	13	96	35,28222975
03.06.2010	177	13	97	182	13	96	41,53513503
03.06.2010	178	26	122	182	13	96	52,70295368

03.06.2010	179	54	153	182	13	96	30,08720373
03.06.2010	180	67	166	182	13	96	71,5829329
03.06.2010	181	60	156	182	13	96	101,4451082
03.06.2010	170	49	148	191	128	203	74,17157804
03.06.2010	172	19	106	191	128	203	10,38761591
03.06.2010	173	16	105	191	128	203	59,60718621
03.06.2010	174	125	211	191	128	203	265,2164969
03.06.2010	175	52	150	191	128	203	14,61819131
03.06.2010	176	37	136	191	128	203	9,239883258
03.06.2010	177	13	97	191	128	203	66,03809778
03.06.2010	178	26	122	191	128	203	26,54686046
03.06.2010	179	54	153	191	128	203	53,73445532
03.06.2010	180	67	166	191	128	203	45,82939493
03.06.2010	181	60	156	191	128	203	77,09827011
03.06.2010	182	13	96	191	128	203	26,26224811
03.06.2010	170	49	148	193	171	232	20,70467866
03.06.2010	172	19	106	193	171	232	86,69784584
03.06.2010	173	16	105	193	171	232	145,6586689
03.06.2010	174	125	211	193	171	232	182,1692335
03.06.2010	175	52	150	193	171	232	105,6024747
03.06.2010	176	37	136	193	171	232	85,66163857
03.06.2010	177	13	97	193	171	232	151,6581988
03.06.2010	178	26	122	193	171	232	69,61649525
03.06.2010	179	54	153	193	171	232	139,5102108
03.06.2010	180	67	166	193	171	232	50,01205863
03.06.2010	181	60	156	193	171	232	18,04901961
03.06.2010	182	13	96	193	171	232	119,4014461
03.06.2010	191	128	203	193	171	232	94,78989412
03.06.2010	170	49	148	194	81	184	29,17406315
03.06.2010	172	19	106	194	81	184	93,5020202
03.06.2010	173	16	105	194	81	184	148,2450003
03.06.2010	174	125	211	194	81	184	185,0477997
03.06.2010	175	52	150	194	81	184	110,3942608

03.06.2010	176	37	136	194	81	184	91,87793859
03.06.2010	177	13	97	194	81	184	154,0270981
03.06.2010	178	26	122	194	81	184	76,89755488
03.06.2010	179	54	153	194	81	184	142,1873505
03.06.2010	180	67	166	194	81	184	57,92880341
03.06.2010	181	60	156	194	81	184	24,56180897
03.06.2010	182	13	96	194	81	184	124,2609322
03.06.2010	191	128	203	194	81	184	100,765381
03.06.2010	193	171	232	194	81	184	12,83914377
03.06.2010	170	49	148	172	19	106	13,26543198
03.06.2010	170	49	148	173	16	105	90,78466537
03.06.2010	172	19	106	173	16	105	78,79562884
03.06.2010	170	49	148	174	125	211	230,6087033
03.06.2010	172	19	106	174	125	211	243,7225056
03.06.2010	173	16	105	174	125	211	319,2917769
03.06.2010	170	49	148	175	52	150	38,30732814
03.06.2010	172	19	106	175	52	150	26,2543364
03.06.2010	173	16	105	175	52	150	52,58963166
03.06.2010	174	125	211	175	52	150	268,2859
03.06.2010	170	49	148	176	37	136	11,91411589
03.06.2010	172	19	106	176	37	136	3,966694033
03.06.2010	173	16	105	176	37	136	78,95669459
03.06.2010	174	125	211	176	37	136	242,4085156
03.06.2010	175	52	150	176	37	136	26,4182754
03.06.2010	170	49	148	177	13	97	74,76712112
03.06.2010	172	19	106	177	13	97	62,83153105
03.06.2010	173	16	105	177	13	97	16,01805089
03.06.2010	174	125	211	177	13	97	303,5361335
03.06.2010	175	52	150	177	13	97	36,59876389
03.06.2010	176	37	136	177	13	97	62,94406456
03.06.2010	170	49	148	178	26	122	18,98937945
03.06.2010	172	19	106	178	26	122	5,904706018
03.06.2010	173	16	105	178	26	122	72,99245932

03.06.2010	174	125	211	178	26	122	249,561863
03.06.2010	175	52	150	178	26	122	20,54658692
03.06.2010	176	37	136	178	26	122	7,723393458
03.06.2010	177	13	97	178	26	122	57,05321833
03.06.2010	170	49	148	179	54	153	79,53054843
03.06.2010	172	19	106	179	54	153	67,22901819
03.06.2010	173	16	105	179	54	153	12,31560753
03.06.2010	174	125	211	179	54	153	309,011542
03.06.2010	175	52	150	179	54	153	41,22331274
03.06.2010	176	37	136	179	54	153	67,63635645
03.06.2010	177	13	97	179	54	153	6,648698053
03.06.2010	178	26	122	179	54	153	61,37115874
03.06.2010	170	49	148	180	67	166	16,27482147
03.06.2010	172	19	106	180	67	166	28,97040418
03.06.2010	173	16	105	180	67	166	102,7316158
03.06.2010	174	125	211	180	67	166	216,9119476
03.06.2010	175	52	150	180	67	166	51,44874075
03.06.2010	176	37	136	180	67	166	26,55332439
03.06.2010	177	13	97	180	67	166	86,82511734
03.06.2010	178	26	122	180	67	166	34,22983059
03.06.2010	179	54	153	180	67	166	92,14117994
03.06.2010	170	49	148	181	60	156	41,93390398
03.06.2010	172	19	106	181	60	156	54,57044746
03.06.2010	173	16	105	181	60	156	124,4907834
03.06.2010	174	125	211	181	60	156	195,081982
03.06.2010	175	52	150	181	60	156	75,30675956
03.06.2010	176	37	136	181	60	156	51,91068157
03.06.2010	177	13	97	181	60	156	108,9522869
03.06.2010	178	26	122	181	60	156	59,63316866
03.06.2010	179	54	153	181	60	156	114,7292995
03.06.2010	180	67	166	181	60	156	25,68215658
03.06.2010	170	49	148	182	13	96	71,37733842
03.06.2010	172	19	106	182	13	96	58,89818405

03.06.2010	173	16	105	182	13	96	20,91519231
03.06.2010	174	125	211	182	13	96	301,3117062
03.06.2010	175	52	150	182	13	96	33,13190265
03.06.2010	176	37	136	182	13	96	59,46487726
03.06.2010	177	13	97	182	13	96	7,932249439
03.06.2010	178	26	122	182	13	96	53,01642827
03.06.2010	179	54	153	182	13	96	8,642335534
03.06.2010	180	67	166	182	13	96	84,40159156
03.06.2010	181	60	156	182	13	96	107,4989535
03.06.2010	170	49	148	191	128	203	27,18199195
03.06.2010	172	19	106	191	128	203	14,50467554
03.06.2010	173	16	105	191	128	203	64,32721179
03.06.2010	174	125	211	191	128	203	257,7467284
03.06.2010	175	52	150	191	128	203	11,95687388
03.06.2010	176	37	136	191	128	203	15,33881534
03.06.2010	177	13	97	191	128	203	48,39112128
03.06.2010	178	26	122	191	128	203	8,664777098
03.06.2010	179	54	153	191	128	203	52,72437252
03.06.2010	180	67	166	191	128	203	41,57197937
03.06.2010	181	60	156	191	128	203	66,45844505
03.06.2010	182	13	96	191	128	203	44,40211339
03.06.2010	170	49	148	193	171	232	57,39495241
03.06.2010	172	19	106	193	171	232	69,71302491
03.06.2010	173	16	105	193	171	232	136,2426483
03.06.2010	174	125	211	193	171	232	185,1572036
03.06.2010	175	52	150	193	171	232	88,93980816
03.06.2010	176	37	136	193	171	232	66,82386028
03.06.2010	177	13	97	193	171	232	121,0885519
03.06.2010	178	26	122	193	171	232	74,52850539
03.06.2010	179	54	153	193	171	232	127,1231383
03.06.2010	180	67	166	193	171	232	41,12744929
03.06.2010	181	60	156	193	171	232	15,80517508
03.06.2010	182	13	96	193	171	232	120,3017851

03.06.2010	191	128	203	193	171	232	80,88099419
03.06.2010	170	49	148	194	81	184	74,79025243
03.06.2010	172	19	106	194	81	184	87,30897096
03.06.2010	173	16	105	194	81	184	153,4111764
03.06.2010	174	125	211	194	81	184	169,8669656
03.06.2010	175	52	150	194	81	184	106,6784335
03.06.2010	176	37	136	194	81	184	84,49870843
03.06.2010	177	13	97	194	81	184	138,4258959
03.06.2010	178	26	122	194	81	184	92,21369302
03.06.2010	179	54	153	194	81	184	144,5368379
03.06.2010	180	67	166	194	81	184	58,52495421
03.06.2010	181	60	156	194	81	184	32,86227161
03.06.2010	182	13	96	194	81	184	137,8462258
03.06.2010	191	128	203	194	81	184	98,64580049
03.06.2010	193	171	232	194	81	184	17,77393097
03.06.2010	170	49	148	172	19	106	8,53453546
03.06.2010	170	49	148	173	16	105	90,0622507
03.06.2010	172	19	106	173	16	105	82,47072429
03.06.2010	170	49	148	174	125	211	225,3624743
03.06.2010	172	19	106	174	125	211	233,3070184
03.06.2010	173	16	105	174	125	211	315,259983
03.06.2010	170	49	148	175	52	150	29,13146093
03.06.2010	172	19	106	175	52	150	20,67062274
03.06.2010	173	16	105	175	52	150	63,37367501
03.06.2010	174	125	211	175	52	150	253,5556413
03.06.2010	170	49	148	176	37	136	29,68082486
03.06.2010	172	19	106	176	37	136	21,17528493
03.06.2010	173	16	105	176	37	136	66,00717883
03.06.2010	174	125	211	176	37	136	252,4180371
03.06.2010	175	52	150	176	37	136	5,306921738
03.06.2010	170	49	148	177	13	97	96,04541025
03.06.2010	172	19	106	177	13	97	88,50841097
03.06.2010	173	16	105	177	13	97	6,136497692

03.06.2010	174	125	211	177	13	97	321,1575326
03.06.2010	175	52	150	177	13	97	69,49377623
03.06.2010	176	37	136	177	13	97	72,14388828
03.06.2010	170	49	148	178	26	122	17,96826745
03.06.2010	172	19	106	178	26	122	9,452977674
03.06.2010	173	16	105	178	26	122	74,80136258
03.06.2010	174	125	211	178	26	122	241,8151999
03.06.2010	175	52	150	178	26	122	11,74037299
03.06.2010	176	37	136	178	26	122	11,72266444
03.06.2010	177	13	97	178	26	122	80,89997701
03.06.2010	170	49	148	179	54	153	81,05864432
03.06.2010	172	19	106	179	54	153	73,52055319
03.06.2010	173	16	105	179	54	153	9,047251361
03.06.2010	174	125	211	179	54	153	306,221003
03.06.2010	175	52	150	179	54	153	54,64802143
03.06.2010	176	37	136	179	54	153	57,48686446
03.06.2010	177	13	97	179	54	153	14,9915753
03.06.2010	178	26	122	179	54	153	65,97720372
03.06.2010	170	49	148	180	67	166	22,0473451
03.06.2010	172	19	106	180	67	166	30,34265013
03.06.2010	173	16	105	180	67	166	107,4847945
03.06.2010	174	125	211	180	67	166	208,3811798
03.06.2010	175	52	150	180	67	166	50,2302076
03.06.2010	176	37	136	180	67	166	51,4503208
03.06.2010	177	13	97	180	67	166	113,2298692
03.06.2010	178	26	122	180	67	166	39,75292122
03.06.2010	179	54	153	180	67	166	98,44731627
03.06.2010	170	49	148	181	60	156	38,28067448
03.06.2010	172	19	106	181	60	156	46,62019639
03.06.2010	173	16	105	181	60	156	122,064306
03.06.2010	174	125	211	181	60	156	195,40633
03.06.2010	175	52	150	181	60	156	66,41929075
03.06.2010	176	37	136	181	60	156	67,72804002

03.06.2010	177	13	97	181	60	156	127,6622848
03.06.2010	178	26	122	181	60	156	56,0332433
03.06.2010	179	54	153	181	60	156	113,0735686
03.06.2010	180	67	166	181	60	156	16,28037052
03.06.2010	170	49	148	182	13	96	46,18494616
03.06.2010	172	19	106	182	13	96	38,28893202
03.06.2010	173	16	105	182	13	96	44,34715447
03.06.2010	174	125	211	182	13	96	271,5320633
03.06.2010	175	52	150	182	13	96	19,21540079
03.06.2010	176	37	136	182	13	96	22,64177747
03.06.2010	177	13	97	182	13	96	50,44016126
03.06.2010	178	26	122	182	13	96	30,46149107
03.06.2010	179	54	153	182	13	96	35,51964623
03.06.2010	180	67	166	182	13	96	65,29089514
03.06.2010	181	60	156	182	13	96	80,86211862
03.06.2010	170	49	148	191	128	203	17,28493235
03.06.2010	172	19	106	191	128	203	8,758094367
03.06.2010	173	16	105	191	128	203	75,17674895
03.06.2010	174	125	211	191	128	203	241,3091159
03.06.2010	175	52	150	191	128	203	12,25753032
03.06.2010	176	37	136	191	128	203	12,42804116
03.06.2010	177	13	97	191	128	203	81,26942248
03.06.2010	178	26	122	191	128	203	0,754353486
03.06.2010	179	54	153	191	128	203	66,33424097
03.06.2010	180	67	166	191	128	203	39,03201529
03.06.2010	181	60	156	191	128	203	55,31174434
03.06.2010	182	13	96	191	128	203	30,83020953
03.06.2010	170	49	148	193	171	232	55,84707108
03.06.2010	172	19	106	193	171	232	64,24719549
03.06.2010	173	16	105	193	171	232	138,8588491
03.06.2010	174	125	211	193	171	232	180,9202409
03.06.2010	175	52	150	193	171	232	84,11397101
03.06.2010	176	37	136	193	171	232	85,38587979

03.06.2010	177	13	97	193	171	232	144,3430425
03.06.2010	178	26	122	193	171	232	73,67856199
03.06.2010	179	54	153	193	171	232	129,9208101
03.06.2010	180	67	166	193	171	232	33,94218152
03.06.2010	181	60	156	193	171	232	17,69498627
03.06.2010	182	13	96	193	171	232	98,32943889
03.06.2010	191	128	203	193	171	232	72,96207335
03.06.2010	170	49	148	194	81	184	81,7777015
03.06.2010	172	19	106	194	81	184	90,0468044
03.06.2010	173	16	105	194	81	184	161,0074716
03.06.2010	174	125	211	194	81	184	167,2796964
03.06.2010	175	52	150	194	81	184	109,4229911
03.06.2010	176	37	136	194	81	184	111,0529562
03.06.2010	177	13	97	194	81	184	166,2035393
03.06.2010	178	26	122	194	81	184	99,40970162
03.06.2010	179	54	153	194	81	184	152,2492665
03.06.2010	180	67	166	194	81	184	59,73449052
03.06.2010	181	60	156	194	81	184	43,52139499
03.06.2010	182	13	96	194	81	184	122,4365039
03.06.2010	191	128	203	194	81	184	98,67775158
03.06.2010	193	171	232	194	81	184	26,38286455
03.06.2010	170	49	148	172	19	106	15,62122729
03.06.2010	170	49	148	173	16	105	107,7176564
03.06.2010	172	19	106	173	16	105	94,02919114
03.06.2010	170	49	148	174	125	211	230,4818026
03.06.2010	172	19	106	174	125	211	244,7222245
03.06.2010	173	16	105	174	125	211	338,1680669
03.06.2010	170	49	148	175	52	150	62,45208648
03.06.2010	172	19	106	175	52	150	47,53215659
03.06.2010	173	16	105	175	52	150	48,28872508
03.06.2010	174	125	211	175	52	150	292,1725248
03.06.2010	170	49	148	176	37	136	63,51263005
03.06.2010	172	19	106	176	37	136	48,70823855

03.06.2010	173	16	105	176	37	136	46,73494718
03.06.2010	174	125	211	176	37	136	293,4016258
03.06.2010	175	52	150	176	37	136	1,731704037
03.06.2010	170	49	148	177	13	97	30,35291849
03.06.2010	172	19	106	177	13	97	15,65126592
03.06.2010	173	16	105	177	13	97	78,40629262
03.06.2010	174	125	211	177	13	97	260,3312983
03.06.2010	175	52	150	177	13	97	32,10834008
03.06.2010	176	37	136	177	13	97	33,20515732
03.06.2010	170	49	148	178	26	122	19,84075552
03.06.2010	172	19	106	178	26	122	4,264091498
03.06.2010	173	16	105	178	26	122	90,08155485
03.06.2010	174	125	211	178	26	122	248,8579737
03.06.2010	175	52	150	178	26	122	43,34702152
03.06.2010	176	37	136	178	26	122	44,54712249
03.06.2010	177	13	97	178	26	122	11,68793969
03.06.2010	170	49	148	179	54	153	101,9738215
03.06.2010	172	19	106	179	54	153	88,26907985
03.06.2010	173	16	105	179	54	153	5,760686001
03.06.2010	174	125	211	179	54	153	332,4332588
03.06.2010	175	52	150	179	54	153	42,65086972
03.06.2010	176	37	136	179	54	153	41,07800443
03.06.2010	177	13	97	179	54	153	72,64669835
03.06.2010	178	26	122	179	54	153	84,32252141
03.06.2010	170	49	148	180	67	166	14,71119779
03.06.2010	172	19	106	180	67	166	30,33014481
03.06.2010	173	16	105	180	67	166	120,8895571
03.06.2010	174	125	211	180	67	166	217,4106552
03.06.2010	175	52	150	180	67	166	76,67923583
03.06.2010	176	37	136	180	67	166	77,6617233
03.06.2010	177	13	97	180	67	166	44,70964504
03.06.2010	178	26	122	180	67	166	34,5370581
03.06.2010	179	54	153	180	67	166	115,1808666

03.06.2010	181	60	156	180	67	166	19,00245566
03.06.2010	170	49	148	181	60	156	33,67193022
03.06.2010	172	19	106	181	60	156	49,25802939
03.06.2010	173	16	105	181	60	156	137,5881389
03.06.2010	174	125	211	181	60	156	201,922329
03.06.2010	175	52	150	181	60	156	94,84757465
03.06.2010	176	37	136	181	60	156	95,7326739
03.06.2010	177	13	97	181	60	156	63,18740214
03.06.2010	178	26	122	181	60	156	53,42908238
03.06.2010	179	54	153	181	60	156	131,9488627
03.06.2010	170	49	148	182	13	96	87,18333825
03.06.2010	172	19	106	182	13	96	73,55451535
03.06.2010	173	16	105	182	13	96	20,5349339
03.06.2010	174	125	211	182	13	96	317,6410183
03.06.2010	175	52	150	182	13	96	28,92906322
03.06.2010	176	37	136	182	13	96	27,2614612
03.06.2010	177	13	97	182	13	96	57,95756436
03.06.2010	178	26	122	182	13	96	69,64321798
03.06.2010	179	54	153	182	13	96	14,79232774
03.06.2010	180	67	166	182	13	96	100,4058692
03.06.2010	181	60	156	182	13	96	117,2718651
03.06.2010	170	49	148	191	128	203	39,76859609
03.06.2010	172	19	106	191	128	203	24,46176196
03.06.2010	173	16	105	191	128	203	70,88336464
03.06.2010	174	125	211	191	128	203	268,9229583
03.06.2010	175	52	150	191	128	203	23,2697639
03.06.2010	176	37	136	191	128	203	24,57247132
03.06.2010	177	13	97	191	128	203	9,882291179
03.06.2010	178	26	122	191	128	203	20,22089068
03.06.2010	179	54	153	191	128	203	65,15365316
03.06.2010	180	67	166	191	128	203	54,30330561
03.06.2010	181	60	156	191	128	203	72,92517234
03.06.2010	182	13	96	191	128	203	50,73851173

03.06.2010	170	49	148	193	171	232	35,34451268
03.06.2010	172	19	106	193	171	232	50,83846115
03.06.2010	173	16	105	193	171	232	138,0220512
03.06.2010	174	125	211	193	171	232	202,0858725
03.06.2010	175	52	150	193	171	232	95,86637105
03.06.2010	176	37	136	193	171	232	96,70699066
03.06.2010	177	13	97	193	171	232	64,41722525
03.06.2010	178	26	122	193	171	232	54,96050935
03.06.2010	179	54	153	193	171	232	132,4098532
03.06.2010	180	67	166	193	171	232	20,84077926
03.06.2010	181	60	156	193	171	232	3,002409311
03.06.2010	182	13	96	193	171	232	117,7808779
03.06.2010	191	128	203	193	171	232	74,21431009
03.06.2010	170	49	148	194	81	184	44,58826446
03.06.2010	172	19	106	194	81	184	59,93390771
03.06.2010	173	16	105	194	81	184	145,0182315
03.06.2010	174	125	211	194	81	184	197,0256995
03.06.2010	175	52	150	194	81	184	104,0642459
03.06.2010	176	37	136	194	81	184	104,82735
03.06.2010	177	13	97	194	81	184	73,02549203
03.06.2010	178	26	122	194	81	184	63,98960992
03.06.2010	179	54	153	194	81	184	139,4734634
03.06.2010	180	67	166	194	81	184	30,28544083
03.06.2010	181	60	156	194	81	184	12,14388998
03.06.2010	182	13	96	194	81	184	124,9724203
03.06.2010	191	128	203	194	81	184	82,88042179
03.06.2010	193	171	232	194	81	184	9,598059183
04.06.2010	170	49	148	172	19	106	28,88109612
04.06.2010	170	49	148	173	16	105	104,1186537
04.06.2010	172	19	106	173	16	105	78,6806834
04.06.2010	170	49	148	174	125	211	217,8518408
04.06.2010	172	19	106	174	125	211	243,7486319
04.06.2010	173	16	105	174	125	211	321,8254375

04.06.2010	170	49	148	175	52	150	55,96106007
04.06.2010	172	19	106	175	52	150	27,89052172
04.06.2010	173	16	105	175	52	150	51,97198084
04.06.2010	174	125	211	175	52	150	271,5303335
04.06.2010	170	49	148	176	37	136	43,20049314
04.06.2010	172	19	106	176	37	136	14,37519205
04.06.2010	173	16	105	176	37	136	66,15814771
04.06.2010	174	125	211	176	37	136	257,4167337
04.06.2010	175	52	150	176	37	136	14,27936477
04.06.2010	170	49	148	177	13	97	111,702067
04.06.2010	172	19	106	177	13	97	86,23418541
04.06.2010	173	16	105	177	13	97	7,590684289
04.06.2010	174	125	211	177	13	97	329,4165702
04.06.2010	175	52	150	177	13	97	59,38842401
04.06.2010	176	37	136	177	13	97	73,6104297
04.06.2010	170	49	148	178	26	122	25,78342364
04.06.2010	172	19	106	178	26	122	3,261949798
04.06.2010	173	16	105	178	26	122	80,81295443
04.06.2010	174	125	211	178	26	122	241,3309374
04.06.2010	175	52	150	178	26	122	30,57174628
04.06.2010	176	37	136	178	26	122	17,41741779
04.06.2010	177	13	97	178	26	122	88,38550515
04.06.2010	170	49	148	179	54	153	102,6920426
04.06.2010	172	19	106	179	54	153	77,15694295
04.06.2010	173	16	105	179	54	153	1,615673304
04.06.2010	174	125	211	179	54	153	320,3657545
04.06.2010	175	52	150	179	54	153	50,3941182
04.06.2010	176	37	136	179	54	153	64,58988403
04.06.2010	177	13	97	179	54	153	9,079640489
04.06.2010	178	26	122	179	54	153	79,30643697
04.06.2010	170	49	148	180	67	166	5,590397345
04.06.2010	172	19	106	180	67	166	23,55525529
04.06.2010	173	16	105	180	67	166	98,58928854

04.06.2010	174	125	211	180	67	166	223,3244094
04.06.2010	175	52	150	180	67	166	50,40899274
04.06.2010	176	37	136	180	67	166	37,79685823
04.06.2010	177	13	97	180	67	166	106,1751922
04.06.2010	178	26	122	180	67	166	20,39745685
04.06.2010	179	54	153	180	67	166	97,15669813
04.06.2010	170	49	148	181	60	156	16,96397069
04.06.2010	172	19	106	181	60	156	44,55989475
04.06.2010	173	16	105	181	60	156	115,2512665
04.06.2010	174	125	211	181	60	156	208,8647305
04.06.2010	175	52	150	181	60	156	70,15320053
04.06.2010	176	37	136	181	60	156	58,48388633
04.06.2010	177	13	97	181	60	156	122,7470639
04.06.2010	178	26	122	181	60	156	41,32398139
04.06.2010	179	54	153	181	60	156	113,922442
04.06.2010	180	67	166	181	60	156	21,33506859
04.06.2010	170	49	148	182	13	96	81,93819673
04.06.2010	172	19	106	182	13	96	54,68139891
04.06.2010	173	16	105	182	13	96	26,15351126
04.06.2010	174	125	211	182	13	96	298,4242118
04.06.2010	175	52	150	182	13	96	26,95977301
04.06.2010	176	37	136	182	13	96	41,21279402
04.06.2010	177	13	97	182	13	96	33,17739949
04.06.2010	178	26	122	182	13	96	57,1835232
04.06.2010	179	54	153	182	13	96	24,54120651
04.06.2010	180	67	166	182	13	96	76,3472742
04.06.2010	181	60	156	182	13	96	94,90855116
04.06.2010	170	49	148	191	128	203	48,59061937
04.06.2010	172	19	106	191	128	203	19,96872367
04.06.2010	173	16	105	191	128	203	60,44489061
04.06.2010	174	125	211	191	128	203	263,2641006
04.06.2010	175	52	150	191	128	203	8,483582554
04.06.2010	176	37	136	191	128	203	5,854007721

04.06.2010	177	13	97	191	128	203	67,86972513
04.06.2010	178	26	122	191	128	203	22,87119778
04.06.2010	179	54	153	191	128	203	58,86942234
04.06.2010	180	67	166	191	128	203	43,11635949
04.06.2010	181	60	156	191	128	203	63,48218848
04.06.2010	182	13	96	191	128	203	35,37082522
04.06.2010	170	49	148	193	171	232	36,51722951
04.06.2010	172	19	106	193	171	232	64,88024734
04.06.2010	173	16	105	193	171	232	134,5485609
04.06.2010	174	125	211	193	171	232	192,4880762
04.06.2010	175	52	150	193	171	232	90,53868151
04.06.2010	176	37	136	193	171	232	78,89025832
04.06.2010	177	13	97	193	171	232	141,970086
04.06.2010	178	26	122	193	171	232	61,6621769
04.06.2010	179	54	153	193	171	232	133,2705394
04.06.2010	180	67	166	193	171	232	41,43269041
04.06.2010	181	60	156	193	171	232	20,42909439
04.06.2010	182	13	96	193	171	232	114,984727
04.06.2010	191	128	203	193	171	232	83,91098438
04.06.2010	170	49	148	194	81	184	74,8156705
04.06.2010	172	19	106	194	81	184	103,5650037
04.06.2010	173	16	105	194	81	184	172,7354188
04.06.2010	174	125	211	194	81	184	161,6937395
04.06.2010	175	52	150	194	81	184	129,5359499
04.06.2010	176	37	136	194	81	184	117,7154438
04.06.2010	177	13	97	194	81	184	180,0635135
04.06.2010	178	26	122	194	81	184	100,3842314
04.06.2010	179	54	153	194	81	184	171,5096012
04.06.2010	180	67	166	194	81	184	80,01352772
04.06.2010	181	60	156	194	81	184	59,39222619
04.06.2010	182	13	96	194	81	184	153,8614027
04.06.2010	191	128	203	194	81	184	122,8289751
04.06.2010	193	171	232	194	81	184	39,00215156

04.06.2010	170	49	148	172	19	106	12,07938064
04.06.2010	170	49	148	173	16	105	89,15348289
04.06.2010	172	19	106	173	16	105	80,3117288
04.06.2010	170	49	148	174	125	211	224,1662987
04.06.2010	172	19	106	174	125	211	234,5882063
04.06.2010	173	16	105	174	125	211	312,516903
04.06.2010	170	49	148	175	52	150	52,58224568
04.06.2010	172	19	106	175	52	150	42,55164207
04.06.2010	173	16	105	175	52	150	38,55774506
04.06.2010	174	125	211	175	52	150	276,7400746
04.06.2010	170	49	148	176	37	136	45,30349714
04.06.2010	172	19	106	176	37	136	35,48122666
04.06.2010	173	16	105	176	37	136	45,06020065
04.06.2010	174	125	211	176	37	136	269,4694306
04.06.2010	175	52	150	176	37	136	7,316815285
04.06.2010	170	49	148	177	13	97	81,1938657
04.06.2010	172	19	106	177	13	97	72,72045373
04.06.2010	173	16	105	177	13	97	8,547754599
04.06.2010	174	125	211	177	13	97	304,2243919
04.06.2010	175	52	150	177	13	97	31,87059347
04.06.2010	176	37	136	177	13	97	37,87873817
04.06.2010	170	49	148	178	26	122	23,99971175
04.06.2010	172	19	106	178	26	122	13,1930584
04.06.2010	173	16	105	178	26	122	67,32400695
04.06.2010	174	125	211	178	26	122	247,6896972
04.06.2010	175	52	150	178	26	122	29,36616176
04.06.2010	176	37	136	178	26	122	22,34946818
04.06.2010	177	13	97	178	26	122	59,89048804
04.06.2010	170	49	148	179	54	153	93,53698376
04.06.2010	172	19	106	179	54	153	84,73547082
04.06.2010	173	16	105	179	54	153	4,429294306
04.06.2010	174	125	211	179	54	153	316,7985001
04.06.2010	175	52	150	179	54	153	42,96746793

04.06.2010	176	37	136	179	54	153	49,48844708
04.06.2010	177	13	97	179	54	153	12,61165188
04.06.2010	178	26	122	179	54	153	71,75219612
04.06.2010	170	49	148	180	67	166	44,33207975
04.06.2010	172	19	106	180	67	166	56,4064666
04.06.2010	173	16	105	180	67	166	125,6421795
04.06.2010	174	125	211	180	67	166	188,5026674
04.06.2010	175	52	150	180	67	166	93,23719589
04.06.2010	176	37	136	180	67	166	86,01997943
04.06.2010	177	13	97	180	67	166	117,1570074
04.06.2010	178	26	122	180	67	166	67,2997187
04.06.2010	179	54	153	180	67	166	129,7571186
04.06.2010	170	49	148	181	60	156	33,92661022
04.06.2010	172	19	106	181	60	156	46,00074022
04.06.2010	173	16	105	181	60	156	116,5594805
04.06.2010	174	125	211	181	60	156	196,5943816
04.06.2010	175	52	150	181	60	156	83,33873437
04.06.2010	176	37	136	181	60	156	76,08168303
04.06.2010	177	13	97	181	60	156	108,127759
04.06.2010	178	26	122	181	60	156	56,98510111
04.06.2010	179	54	153	181	60	156	120,7391325
04.06.2010	180	67	166	181	60	156	10,40586039
04.06.2010	170	49	148	182	13	96	74,36629034
04.06.2010	172	19	106	182	13	96	65,34116341
04.06.2010	173	16	105	182	13	96	15,00000802
04.06.2010	174	125	211	182	13	96	298,0767819
04.06.2010	175	52	150	182	13	96	23,65856327
04.06.2010	176	37	136	182	13	96	30,06122422
04.06.2010	177	13	97	182	13	96	8,611448451
04.06.2010	178	26	122	182	13	96	52,32989801
04.06.2010	179	54	153	182	13	96	19,42903387
04.06.2010	180	67	166	182	13	96	111,9289797
04.06.2010	181	60	156	182	13	96	102,6001739

04.06.2010	170	49	148	191	128	203	33,50547144
04.06.2010	172	19	106	191	128	203	22,94414354
04.06.2010	173	16	105	191	128	203	57,86710236
04.06.2010	174	125	211	191	128	203	257,4153653
04.06.2010	175	52	150	191	128	203	19,65459496
04.06.2010	176	37	136	191	128	203	12,80815172
04.06.2010	177	13	97	191	128	203	50,63925688
04.06.2010	178	26	122	191	128	203	9,754416258
04.06.2010	179	54	153	191	128	203	62,29518136
04.06.2010	180	67	166	191	128	203	75,98141607
04.06.2010	181	60	156	191	128	203	65,77887435
04.06.2010	182	13	96	191	128	203	42,86886776
04.06.2010	170	49	148	193	171	232	62,63434523
04.06.2010	172	19	106	193	171	232	74,65250652
04.06.2010	173	16	105	193	171	232	139,8470908
04.06.2010	174	125	211	193	171	232	178,524937
04.06.2010	175	52	150	193	171	232	109,5360631
04.06.2010	176	37	136	193	171	232	102,4638033
04.06.2010	177	13	97	193	171	232	131,3021603
04.06.2010	178	26	122	193	171	232	84,94175096
04.06.2010	179	54	153	193	171	232	143,7945157
04.06.2010	180	67	166	193	171	232	18,76207962
04.06.2010	181	60	156	193	171	232	28,92422325
04.06.2010	182	13	96	193	171	232	126,7689055
04.06.2010	191	128	203	193	171	232	93,20560868
04.06.2010	170	49	148	194	81	184	63,17753794
04.06.2010	172	19	106	194	81	184	75,25108538
04.06.2010	173	16	105	194	81	184	144,121789
04.06.2010	174	125	211	194	81	184	171,8611737
04.06.2010	175	52	150	194	81	184	112,316872
04.06.2010	176	37	136	194	81	184	105,1180578
04.06.2010	177	13	97	194	81	184	135,6029683
04.06.2010	178	26	122	194	81	184	86,39126333

04.06.2010	179	54	153	194	81	184	148,1725067
04.06.2010	180	67	166	194	81	184	19,14428908
04.06.2010	181	60	156	194	81	184	29,40734898
04.06.2010	182	13	96	194	81	184	130,6336585
04.06.2010	191	128	203	194	81	184	95,12187479
04.06.2010	193	171	232	194	81	184	8,943827854
04.06.2010	170	49	148	172	19	106	17,39614254
04.06.2010	170	49	148	173	16	105	104,8898015
04.06.2010	172	19	106	173	16	105	92,69134386
04.06.2010	170	49	148	174	125	211	230,1494298
04.06.2010	172	19	106	174	125	211	242,6807636
04.06.2010	173	16	105	174	125	211	334,9110495
04.06.2010	170	49	148	175	52	150	65,13826295
04.06.2010	172	19	106	175	52	150	51,58418535
04.06.2010	173	16	105	175	52	150	41,49042147
04.06.2010	174	125	211	175	52	150	294,23754
04.06.2010	170	49	148	176	37	136	42,14789552
04.06.2010	172	19	106	176	37	136	25,30821018
04.06.2010	173	16	105	176	37	136	71,99537157
04.06.2010	174	125	211	176	37	136	266,0608608
04.06.2010	175	52	150	176	37	136	30,79605886
04.06.2010	170	49	148	177	13	97	105,0267202
04.06.2010	172	19	106	177	13	97	92,71833285
04.06.2010	173	16	105	177	13	97	0,881361048
04.06.2010	174	125	211	177	13	97	335,009664
04.06.2010	175	52	150	177	13	97	41,43334442
04.06.2010	176	37	136	177	13	97	71,86653451
04.06.2010	170	49	148	178	26	122	31,63876909
04.06.2010	172	19	106	178	26	122	14,88819015
04.06.2010	173	16	105	178	26	122	79,60115952
04.06.2010	174	125	211	178	26	122	256,7667644
04.06.2010	175	52	150	178	26	122	38,12581053
04.06.2010	176	37	136	178	26	122	10,51552149

04.06.2010	177	13	97	178	26	122	79,55598472
04.06.2010	170	49	148	179	54	153	97,04097621
04.06.2010	172	19	106	179	54	153	84,74037413
04.06.2010	173	16	105	179	54	153	7,954991027
04.06.2010	174	125	211	179	54	153	327,0109973
04.06.2010	175	52	150	179	54	153	33,55907571
04.06.2010	176	37	136	179	54	153	64,13631472
04.06.2010	177	13	97	179	54	153	7,999151162
04.06.2010	178	26	122	179	54	153	71,65721595
04.06.2010	170	49	148	180	67	166	21,90884531
04.06.2010	172	19	106	180	67	166	39,14385908
04.06.2010	173	16	105	180	67	166	124,092468
04.06.2010	174	125	211	180	67	166	212,5643601
04.06.2010	175	52	150	180	67	166	85,70022247
04.06.2010	176	37	136	180	67	166	64,05556643
04.06.2010	177	13	97	180	67	166	124,3086803
04.06.2010	178	26	122	180	67	166	53,54737542
04.06.2010	179	54	153	180	67	166	116,4011325
04.06.2010	170	49	148	181	60	156	29,89052272
04.06.2010	172	19	106	181	60	156	47,2298077
04.06.2010	173	16	105	181	60	156	130,2232669
04.06.2010	174	125	211	181	60	156	207,9833372
04.06.2010	175	52	150	181	60	156	92,61491362
04.06.2010	176	37	136	181	60	156	71,96211703
04.06.2010	177	13	97	181	60	156	130,476192
04.06.2010	178	26	122	181	60	156	61,47586065
04.06.2010	179	54	153	181	60	156	122,6293795
04.06.2010	180	67	166	181	60	156	8,24382988
04.06.2010	170	49	148	182	13	96	85,53538149
04.06.2010	172	19	106	182	13	96	72,91266139
04.06.2010	173	16	105	182	13	96	19,85236103
04.06.2010	174	125	211	182	13	96	315,332562
04.06.2010	175	52	150	182	13	96	21,64942983

04.06.2010	176	37	136	182	13	96	52,28525996
04.06.2010	177	13	97	182	13	96	19,82732107
04.06.2010	178	26	122	182	13	96	59,74908063
04.06.2010	179	54	153	182	13	96	11,91032461
04.06.2010	180	67	166	182	13	96	105,2547654
04.06.2010	181	60	156	182	13	96	111,6896134
04.06.2010	170	49	148	191	128	203	48,77930351
04.06.2010	172	19	106	191	128	203	32,39610077
04.06.2010	173	16	105	191	128	203	64,75873508
04.06.2010	174	125	211	191	128	203	273,646622
04.06.2010	175	52	150	191	128	203	23,96769982
04.06.2010	176	37	136	191	128	203	7,592469004
04.06.2010	177	13	97	191	128	203	64,60107061
04.06.2010	178	26	122	191	128	203	17,51464167
04.06.2010	179	54	153	191	128	203	56,95053614
04.06.2010	180	67	166	191	128	203	70,6205669
04.06.2010	181	60	156	191	128	203	78,38607214
04.06.2010	182	13	96	191	128	203	45,16851643
04.06.2010	170	49	148	193	171	232	43,10064474
04.06.2010	172	19	106	193	171	232	60,46132612
04.06.2010	173	16	105	193	171	232	141,4821517
04.06.2010	174	125	211	193	171	232	199,6784612
04.06.2010	175	52	150	193	171	232	104,8416956
04.06.2010	176	37	136	193	171	232	85,09574814
04.06.2010	177	13	97	193	171	232	141,7779106
04.06.2010	178	26	122	193	171	232	74,62957458
04.06.2010	179	54	153	193	171	232	134,0218858
04.06.2010	180	67	166	193	171	232	21,44465046
04.06.2010	181	60	156	193	171	232	13,24362503
04.06.2010	182	13	96	193	171	232	123,3478623
04.06.2010	191	128	203	193	171	232	91,41639325
04.06.2010	170	49	148	194	81	184	67,79142526
04.06.2010	172	19	106	194	81	184	84,9904929

04.06.2010	173	16	105	194	81	184	166,182968
04.06.2010	174	125	211	194	81	184	179,535325
04.06.2010	175	52	150	194	81	184	130,01036
04.06.2010	176	37	136	194	81	184	109,9304271
04.06.2010	177	13	97	194	81	184	166,50524
04.06.2010	178	26	122	194	81	184	99,42728179
04.06.2010	179	54	153	194	81	184	158,8143952
04.06.2010	180	67	166	194	81	184	45,88572812
04.06.2010	181	60	156	194	81	184	38,02095047
04.06.2010	182	13	96	194	81	184	148,2966597
04.06.2010	191	128	203	194	81	184	116,4054391
04.06.2010	193	171	232	194	81	184	25,20109163
09.06.2010	170	49	148	172	19	106	18,74
09.06.2010	170	49	148	173	16	105	158,13
09.06.2010	172	19	106	173	16	105	144,68
09.06.2010	170	49	148	174	125	211	197,19
09.06.2010	172	19	106	174	125	211	211,09
09.06.2010	173	16	105	174	125	211	355,25
09.06.2010	170	49	148	175	52	150	92,23
09.06.2010	172	19	106	175	52	150	75,03
09.06.2010	173	16	105	175	52	150	79,04
09.06.2010	174	125	211	175	52	150	284,38
09.06.2010	170	49	148	176	37	136	43,82
09.06.2010	172	19	106	176	37	136	25,35
09.06.2010	173	16	105	176	37	136	124,47
09.06.2010	174	125	211	176	37	136	233,83
09.06.2010	175	52	150	176	37	136	50,70
09.06.2010	170	49	148	177	13	97	69,04
09.06.2010	172	19	106	177	13	97	51,48
09.06.2010	173	16	105	177	13	97	99,28
09.06.2010	174	125	211	177	13	97	260,84
09.06.2010	175	52	150	177	13	97	23,67
09.06.2010	176	37	136	177	13	97	27,05

09.06.2010	170	49	148	178	26	122	68,65
09.06.2010	172	19	106	178	26	122	50,97
09.06.2010	173	16	105	178	26	122	100,35
09.06.2010	174	125	211	178	26	122	260,08
09.06.2010	175	52	150	178	26	122	24,34
09.06.2010	176	37	136	178	26	122	26,36
09.06.2010	177	13	97	178	26	122	1,24
09.06.2010	170	49	148	179	54	153	124,78
09.06.2010	172	19	106	179	54	153	109,42
09.06.2010	173	16	105	179	54	153	40,40
09.06.2010	174	125	211	179	54	153	320,43
09.06.2010	175	52	150	179	54	153	39,01
09.06.2010	176	37	136	179	54	153	87,11
09.06.2010	177	13	97	179	54	153	60,71
09.06.2010	178	26	122	179	54	153	61,67
09.06.2010	170	49	148	180	67	166	38,82
09.06.2010	172	19	106	180	67	166	20,16
09.06.2010	173	16	105	180	67	166	129,81
09.06.2010	174	125	211	180	67	166	228,19
09.06.2010	175	52	150	180	67	166	56,35
09.06.2010	176	37	136	180	67	166	5,66
09.06.2010	177	13	97	180	67	166	32,71
09.06.2010	178	26	122	180	67	166	32,01
09.06.2010	179	54	153	180	67	166	92,66
09.06.2010	170	49	148	181	60	156	5,14
09.06.2010	172	19	106	181	60	156	23,09
09.06.2010	173	16	105	181	60	156	163,25
09.06.2010	174	125	211	181	60	156	192,10
09.06.2010	175	52	150	181	60	156	97,21
09.06.2010	176	37	136	181	60	156	48,39
09.06.2010	177	13	97	181	60	156	73,93
09.06.2010	178	26	122	181	60	156	73,52
09.06.2010	179	54	153	181	60	156	129,91

09.06.2010	180	67	166	181	60	156	43,25
09.06.2010	170	49	148	182	13	96	119,52
09.06.2010	172	19	106	182	13	96	104,14
09.06.2010	173	16	105	182	13	96	44,89
09.06.2010	174	125	211	182	13	96	315,15
09.06.2010	175	52	150	182	13	96	34,20
09.06.2010	176	37	136	182	13	96	81,87
09.06.2010	177	13	97	182	13	96	55,55
09.06.2010	178	26	122	182	13	96	56,52
09.06.2010	179	54	153	182	13	96	5,28
09.06.2010	180	67	166	182	13	96	87,41
09.06.2010	181	60	156	182	13	96	124,66
09.06.2010	170	49	148	191	128	203	80,47
09.06.2010	172	19	106	191	128	203	63,42
09.06.2010	173	16	105	191	128	203	87,11
09.06.2010	174	125	211	191	128	203	273,34
09.06.2010	175	52	150	191	128	203	11,81
09.06.2010	176	37	136	191	128	203	39,51
09.06.2010	177	13	97	191	128	203	12,59
09.06.2010	178	26	122	191	128	203	13,50
09.06.2010	179	54	153	191	128	203	48,18
09.06.2010	180	67	166	191	128	203	45,15
09.06.2010	181	60	156	191	128	203	85,46
09.06.2010	182	13	96	191	128	203	43,05
09.06.2010	170	49	148	193	171	232	36,45
09.06.2010	172	19	106	193	171	232	55,14
09.06.2010	173	16	105	193	171	232	188,56
09.06.2010	174	125	211	193	171	232	170,44
09.06.2010	175	52	150	193	171	232	127,66
09.06.2010	176	37	136	193	171	232	80,25
09.06.2010	177	13	97	193	171	232	104,90
09.06.2010	178	26	122	193	171	232	104,61
09.06.2010	179	54	153	193	171	232	157,99

09.06.2010	180	67	166	193	171	232	75,27
09.06.2010	181	60	156	193	171	232	32,16
09.06.2010	182	13	96	193	171	232	152,83
09.06.2010	191	128	203	193	171	232	115,85
09.06.2010	170	49	148	194	81	184	26,30
09.06.2010	172	19	106	194	81	184	44,75
09.06.2010	173	16	105	194	81	184	181,65
09.06.2010	174	125	211	194	81	184	175,12
09.06.2010	175	52	150	194	81	184	118,34
09.06.2010	176	37	136	194	81	184	70,04
09.06.2010	177	13	97	194	81	184	95,28
09.06.2010	178	26	122	194	81	184	94,92
09.06.2010	179	54	153	194	81	184	149,85
09.06.2010	180	67	166	194	81	184	64,91
09.06.2010	181	60	156	194	81	184	21,66
09.06.2010	182	13	96	194	81	184	144,64
09.06.2010	191	128	203	194	81	184	106,55
09.06.2010	193	171	232	194	81	184	11,09
09.06.2010	170	49	148	172	19	106	19,95967494
09.06.2010	170	49	148	173	16	105	128,5515633
09.06.2010	172	19	106	173	16	105	112,6944528
09.06.2010	170	49	148	174	125	211	225,9518215
09.06.2010	172	19	106	174	125	211	240,4031298
09.06.2010	173	16	105	174	125	211	353,0687981
09.06.2010	170	49	148	175	52	150	82,63839942
09.06.2010	172	19	106	175	52	150	63,74940537
09.06.2010	173	16	105	175	52	150	56,24441961
09.06.2010	174	125	211	175	52	150	300,9805443
09.06.2010	170	49	148	176	37	136	41,46831888
09.06.2010	172	19	106	176	37	136	21,67444844
09.06.2010	173	16	105	176	37	136	94,57734976
09.06.2010	174	125	211	176	37	136	259,0938289
09.06.2010	175	52	150	176	37	136	42,70885754

09.06.2010	170	49	148	177	13	97	67,77650074
09.06.2010	172	19	106	177	13	97	48,74837009
09.06.2010	173	16	105	177	13	97	68,72211332
09.06.2010	174	125	211	177	13	97	286,4154731
09.06.2010	175	52	150	177	13	97	15,01836292
09.06.2010	176	37	136	177	13	97	27,72942364
09.06.2010	170	49	148	178	26	122	28,23828061
09.06.2010	172	19	106	178	26	122	8,294077618
09.06.2010	173	16	105	178	26	122	106,9456579
09.06.2010	174	125	211	178	26	122	246,2215564
09.06.2010	175	52	150	178	26	122	56,39509602
09.06.2010	176	37	136	178	26	122	13,79875586
09.06.2010	177	13	97	178	26	122	41,38186411
09.06.2010	170	49	148	179	54	153	118,115458
09.06.2010	172	19	106	179	54	153	101,6986847
09.06.2010	173	16	105	179	54	153	12,04568426
09.06.2010	174	125	211	179	54	153	341,9097217
09.06.2010	175	52	150	179	54	153	44,20291977
09.06.2010	176	37	136	179	54	153	83,07864213
09.06.2010	177	13	97	179	54	153	56,85926627
09.06.2010	178	26	122	179	54	153	95,69086704
09.06.2010	170	49	148	180	67	166	7,291464346
09.06.2010	172	19	106	180	67	166	13,56039008
09.06.2010	173	16	105	180	67	166	124,8120088
09.06.2010	174	125	211	180	67	166	228,7287222
09.06.2010	175	52	150	180	67	166	77,16074753
09.06.2010	176	37	136	180	67	166	35,23035002
09.06.2010	177	13	97	180	67	166	62,18866352
09.06.2010	178	26	122	180	67	166	21,64442263
09.06.2010	179	54	153	180	67	166	114,0834783
09.06.2010	170	49	148	181	60	156	3,38826526
09.06.2010	172	19	106	181	60	156	23,2957565
09.06.2010	173	16	105	181	60	156	131,6562364

09.06.2010	174	125	211	181	60	156	223,1716946
09.06.2010	175	52	150	181	60	156	86,00921575
09.06.2010	176	37	136	181	60	156	44,84137419
09.06.2010	177	13	97	181	60	156	71,15528418
09.06.2010	178	26	122	181	60	156	31,5594464
09.06.2010	179	54	153	181	60	156	121,2823423
09.06.2010	180	67	166	181	60	156	10,27834776
09.06.2010	170	49	148	182	13	96	107,5937332
09.06.2010	172	19	106	182	13	96	91,30145853
09.06.2010	173	16	105	182	13	96	21,58382724
09.06.2010	174	125	211	182	13	96	331,6022428
09.06.2010	175	52	150	182	13	96	35,77127152
09.06.2010	176	37	136	182	13	96	72,99382051
09.06.2010	177	13	97	182	13	96	47,36406304
09.06.2010	178	26	122	182	13	96	85,41930124
09.06.2010	179	54	153	182	13	96	10,53697388
09.06.2010	180	67	166	182	13	96	103,6082974
09.06.2010	181	60	156	182	13	96	110,7552359
09.06.2010	170	49	148	191	128	203	79,48453803
09.06.2010	172	19	106	191	128	203	60,55730993
09.06.2010	173	16	105	191	128	203	58,88652766
09.06.2010	174	125	211	191	128	203	297,8386592
09.06.2010	175	52	150	191	128	203	3,20327182
09.06.2010	176	37	136	191	128	203	39,50482167
09.06.2010	177	13	97	191	128	203	11,81782248
09.06.2010	178	26	122	191	128	203	53,1916222
09.06.2010	179	54	153	191	128	203	46,86387461
09.06.2010	180	67	166	191	128	203	73,97818471
09.06.2010	181	60	156	191	128	203	82,85753227
09.06.2010	182	13	96	191	128	203	38,13706227
09.06.2010	170	49	148	193	171	232	43,51776285
09.06.2010	172	19	106	193	171	232	63,45870721
09.06.2010	173	16	105	193	171	232	165,1893069

09.06.2010	174	125	211	193	171	232	199,661271
09.06.2010	175	52	150	193	171	232	124,5213257
09.06.2010	176	37	136	193	171	232	84,76328801
09.06.2010	177	13	97	193	171	232	110,0209776
09.06.2010	178	26	122	193	171	232	71,7480418
09.06.2010	179	54	153	193	171	232	155,8510741
09.06.2010	180	67	166	193	171	232	50,42457941
09.06.2010	181	60	156	193	171	232	40,23510451
09.06.2010	182	13	96	193	171	232	145,3820516
09.06.2010	191	128	203	193	171	232	121,4549647
09.06.2010	170	49	148	194	81	184	25,59443237
09.06.2010	172	19	106	194	81	184	45,48079612
09.06.2010	173	16	105	194	81	184	151,6168596
09.06.2010	174	125	211	194	81	184	206,6233605
09.06.2010	175	52	150	194	81	184	107,9402258
09.06.2010	176	37	136	194	81	184	67,059181
09.06.2010	177	13	97	194	81	184	93,17764604
09.06.2010	178	26	122	194	81	184	53,70889871
09.06.2010	179	54	153	194	81	184	141,6843231
09.06.2010	180	67	166	194	81	184	32,13266454
09.06.2010	181	60	156	194	81	184	22,21813788
09.06.2010	182	13	96	194	81	184	131,1517639
09.06.2010	191	128	203	194	81	184	104,8119682
09.06.2010	193	171	232	194	81	184	18,75798706
09.06.2010	170	49	148	172	19	106	11,6337174
09.06.2010	170	49	148	173	16	105	104,4321382
09.06.2010	172	19	106	173	16	105	98,27982205
09.06.2010	170	49	148	174	125	211	259,0976637
09.06.2010	172	19	106	174	125	211	265,8403379
09.06.2010	173	16	105	174	125	211	363,5288165
09.06.2010	170	49	148	175	52	150	54,12486793
09.06.2010	172	19	106	175	52	150	45,88316902
09.06.2010	173	16	105	175	52	150	53,98099033

09.06.2010	174	125	211	175	52	150	311,6403609
09.06.2010	170	49	148	176	37	136	17,71641248
09.06.2010	172	19	106	176	37	136	6,156336746
09.06.2010	173	16	105	176	37	136	94,4359457
09.06.2010	174	125	211	176	37	136	270,4048327
09.06.2010	175	52	150	176	37	136	41,26995723
09.06.2010	170	49	148	177	13	97	31,77906147
09.06.2010	172	19	106	177	13	97	22,73612774
09.06.2010	173	16	105	177	13	97	76,35011355
09.06.2010	174	125	211	177	13	97	288,4583094
09.06.2010	175	52	150	177	13	97	23,18498934
09.06.2010	176	37	136	177	13	97	18,16027576
09.06.2010	170	49	148	178	26	122	24,79477498
09.06.2010	172	19	106	178	26	122	14,9469233
09.06.2010	173	16	105	178	26	122	84,22155506
09.06.2010	174	125	211	178	26	122	280,5288418
09.06.2010	175	52	150	178	26	122	31,11179244
09.06.2010	176	37	136	178	26	122	10,22725083
09.06.2010	177	13	97	178	26	122	7,945902202
09.06.2010	170	49	148	179	54	153	107,7899141
09.06.2010	172	19	106	179	54	153	102,0170821
09.06.2010	173	16	105	179	54	153	5,286457953
09.06.2010	174	125	211	179	54	153	366,8454438
09.06.2010	175	52	150	179	54	153	58,30631423
09.06.2010	176	37	136	179	54	153	98,354192
09.06.2010	177	13	97	179	54	153	80,34934065
09.06.2010	178	26	122	179	54	153	88,16786916
09.06.2010	170	49	148	180	67	166	11,21910246
09.06.2010	172	19	106	180	67	166	22,48103574
09.06.2010	173	16	105	180	67	166	113,8441196
09.06.2010	174	125	211	180	67	166	249,9799643
09.06.2010	175	52	150	180	67	166	64,80028669
09.06.2010	176	37	136	180	67	166	28,6367439

09.06.2010	177	13	97	180	67	166	42,84273283
09.06.2010	178	26	122	180	67	166	36,00057124
09.06.2010	179	54	153	180	67	166	116,964761
09.06.2010	170	49	148	181	60	156	21,73728452
09.06.2010	172	19	106	181	60	156	32,99626758
09.06.2010	173	16	105	181	60	156	122,4380163
09.06.2010	174	125	211	181	60	156	242,2335279
09.06.2010	175	52	150	181	60	156	74,69085186
09.06.2010	176	37	136	181	60	156	39,15193457
09.06.2010	177	13	97	181	60	156	53,14388386
09.06.2010	178	26	122	181	60	156	46,46766165
09.06.2010	179	54	153	181	60	156	125,3309379
09.06.2010	180	67	166	181	60	156	10,54340108
09.06.2010	170	49	148	182	13	96	50,02406755
09.06.2010	172	19	106	182	13	96	41,8280093
09.06.2010	173	16	105	182	13	96	57,61109334
09.06.2010	174	125	211	182	13	96	307,620993
09.06.2010	175	52	150	182	13	96	4,101044349
09.06.2010	176	37	136	182	13	96	37,29587133
09.06.2010	177	13	97	182	13	96	19,16739398
09.06.2010	178	26	122	182	13	96	27,10812137
09.06.2010	179	54	153	182	13	96	61,82828583
09.06.2010	180	67	166	182	13	96	60,71238347
09.06.2010	181	60	156	182	13	96	70,62683383
09.06.2010	170	49	148	191	128	203	44,99058912
09.06.2010	172	19	106	191	128	203	35,79247589
09.06.2010	173	16	105	191	128	203	65,11836119
09.06.2010	174	125	211	191	128	203	301,1126808
09.06.2010	175	52	150	191	128	203	11,14032842
09.06.2010	176	37	136	191	128	203	30,75673136
09.06.2010	177	13	97	191	128	203	13,23270808
09.06.2010	178	26	122	191	128	203	20,84662943
09.06.2010	179	54	153	191	128	203	69,44241671

09.06.2010	180	67	166	191	128	203	56,0050263
09.06.2010	181	60	156	191	128	203	66,23230983
09.06.2010	182	13	96	191	128	203	7,869371209
09.06.2010	170	49	148	193	171	232	54,00926135
09.06.2010	172	19	106	193	171	232	65,14436401
09.06.2010	173	16	105	193	171	232	151,1894642
09.06.2010	174	125	211	193	171	232	218,4484054
09.06.2010	175	52	150	193	171	232	106,1796158
09.06.2010	176	37	136	193	171	232	71,28451975
09.06.2010	177	13	97	193	171	232	85,25238082
09.06.2010	178	26	122	193	171	232	78,72481155
09.06.2010	179	54	153	193	171	232	153,5781222
09.06.2010	180	67	166	193	171	232	42,80022953
09.06.2010	181	60	156	193	171	232	32,27346878
09.06.2010	182	13	96	193	171	232	102,1728367
09.06.2010	191	128	203	193	171	232	98,22461686
09.06.2010	170	49	148	194	81	184	41,2789297
09.06.2010	172	19	106	194	81	184	52,63572765
09.06.2010	173	16	105	194	81	184	138,2117699
09.06.2010	174	125	211	194	81	184	229,7850135
09.06.2010	175	52	150	194	81	184	92,96904264
09.06.2010	176	37	136	194	81	184	58,79196431
09.06.2010	177	13	97	194	81	184	72,20908882
09.06.2010	178	26	122	194	81	184	65,84269557
09.06.2010	179	54	153	194	81	184	140,6896941
09.06.2010	180	67	166	194	81	184	30,15961769
09.06.2010	181	60	156	194	81	184	19,64586132
09.06.2010	182	13	96	194	81	184	88,96920427
09.06.2010	191	128	203	194	81	184	85,11612563
09.06.2010	193	171	232	194	81	184	13,2286119
09.06.2010	172	19	106	173	16	105	95,40201394
09.06.2010	172	19	106	174	125	211	87,35933889
09.06.2010	173	16	105	174	125	211	167,9644291

09.06.2010	172	19	106	175	52	150	43,12159647
09.06.2010	173	16	105	175	52	150	53,82078712
09.06.2010	174	125	211	175	52	150	125,1400608
09.06.2010	172	19	106	176	37	136	20,28065919
09.06.2010	173	16	105	176	37	136	77,91433623
09.06.2010	174	125	211	176	37	136	106,7120001
09.06.2010	175	52	150	176	37	136	24,23472685
09.06.2010	172	19	106	177	13	97	47,92918101
09.06.2010	173	16	105	177	13	97	48,90579666
09.06.2010	174	125	211	177	13	97	129,0733377
09.06.2010	175	52	150	177	13	97	4,946626618
09.06.2010	176	37	136	177	13	97	29,18087374
09.06.2010	172	19	106	178	26	122	10,52067405
09.06.2010	173	16	105	178	26	122	85,14424279
09.06.2010	174	125	211	178	26	122	93,27497021
09.06.2010	175	52	150	178	26	122	33,74053258
09.06.2010	176	37	136	178	26	122	13,84841534
09.06.2010	177	13	97	178	26	122	38,38494268
09.06.2010	172	19	106	179	54	153	92,35942857
09.06.2010	173	16	105	179	54	153	7,138733486
09.06.2010	174	125	211	179	54	153	167,4897461
09.06.2010	175	52	150	179	54	153	49,92447786
09.06.2010	176	37	136	179	54	153	74,15763305
09.06.2010	177	13	97	179	54	153	44,97773834
09.06.2010	178	26	122	179	54	153	82,3006772
09.06.2010	172	19	106	180	67	166	20,16027294
09.06.2010	173	16	105	180	67	166	108,2779354
09.06.2010	174	125	211	180	67	166	67,47597856
09.06.2010	175	52	150	180	67	166	59,41016924
09.06.2010	176	37	136	180	67	166	39,27056764
09.06.2010	177	13	97	180	67	166	63,82544648
09.06.2010	178	26	122	180	67	166	26,13969298
09.06.2010	179	54	153	180	67	166	106,2371915

09.06.2010	172	19	106	181	60	156	24,11984621
09.06.2010	173	16	105	181	60	156	111,2957208
09.06.2010	174	125	211	181	60	156	63,52770318
09.06.2010	175	52	150	181	60	156	63,03024957
09.06.2010	176	37	136	181	60	156	43,19735676
09.06.2010	177	13	97	181	60	156	67,38395202
09.06.2010	178	26	122	181	60	156	29,96205544
09.06.2010	179	54	153	181	60	156	109,4117641
09.06.2010	180	67	166	181	60	156	3,971245606
09.06.2010	172	19	106	182	13	96	83,25436946
09.06.2010	173	16	105	182	13	96	13,68306303
09.06.2010	174	125	211	182	13	96	159,0596204
09.06.2010	175	52	150	182	13	96	40,86901977
09.06.2010	176	37	136	182	13	96	65,09401315
09.06.2010	177	13	97	182	13	96	35,92270818
09.06.2010	178	26	122	182	13	96	73,20820105
09.06.2010	179	54	153	182	13	96	9,106460776
09.06.2010	180	67	166	182	13	96	97,26554992
09.06.2010	181	60	156	182	13	96	100,4782161
09.06.2010	172	19	106	191	128	203	32,40139783
09.06.2010	173	16	105	191	128	203	65,76335575
09.06.2010	174	125	211	191	128	203	117,166914
09.06.2010	175	52	150	191	128	203	11,94704931
09.06.2010	176	37	136	191	128	203	12,59409264
09.06.2010	177	13	97	191	128	203	16,86009041
09.06.2010	178	26	122	191	128	203	24,08649397
09.06.2010	179	54	153	191	128	203	61,7667511
09.06.2010	180	67	166	191	128	203	50,22607151
09.06.2010	181	60	156	191	128	203	54,03559528
09.06.2010	182	13	96	191	128	203	52,74261452
09.06.2010	172	19	106	193	171	232	79,98851923
09.06.2010	173	16	105	193	171	232	160,9032881
09.06.2010	174	125	211	193	171	232	7,426480202

09.06.2010	175	52	150	193	171	232	117,740916
09.06.2010	176	37	136	193	171	232	99,29751508
09.06.2010	177	13	97	193	171	232	121,6916747
09.06.2010	178	26	122	193	171	232	85,85070235
09.06.2010	179	54	153	193	171	232	160,3355618
09.06.2010	180	67	166	193	171	232	60,07239918
09.06.2010	181	60	156	193	171	232	56,12054538
09.06.2010	182	13	96	193	171	232	151,8647558
09.06.2010	191	128	203	193	171	232	109,7413241
09.06.2010	172	19	106	194	81	184	72,10124454
09.06.2010	173	16	105	194	81	184	154,6889224
09.06.2010	174	125	211	194	81	184	15,30816362
09.06.2010	175	52	150	194	81	184	110,4745385
09.06.2010	176	37	136	194	81	184	91,5695631
09.06.2010	177	13	97	194	81	184	114,519531
09.06.2010	178	26	122	194	81	184	78,23018207
09.06.2010	179	54	153	194	81	184	153,8904005
09.06.2010	180	67	166	194	81	184	52,30287797
09.06.2010	181	60	156	194	81	184	48,37282547
09.06.2010	182	13	96	194	81	184	145,3166766
09.06.2010	191	128	203	194	81	184	102,2063807
09.06.2010	193	171	232	194	81	184	8,137965714
09.06.2010	170	49	148	172	19	106	7,604356389
09.06.2010	170	49	148	173	16	105	92,10877559
09.06.2010	172	19	106	173	16	105	85,37556515
09.06.2010	170	49	148	174	125	211	258,66076
09.06.2010	172	19	106	174	125	211	265,4492778
09.06.2010	173	16	105	174	125	211	350,7659383
09.06.2010	170	49	148	175	52	150	44,04939644
09.06.2010	172	19	106	175	52	150	36,6894734
09.06.2010	173	16	105	175	52	150	50,27604145
09.06.2010	174	125	211	175	52	150	301,6063294
09.06.2010	170	49	148	176	37	136	8,039137313

09.06.2010	172	19	106	176	37	136	0,445775266
09.06.2010	173	16	105	176	37	136	84,94121927
09.06.2010	174	125	211	176	37	136	265,8880207
09.06.2010	175	52	150	176	37	136	36,24113286
09.06.2010	170	49	148	177	13	97	27,05375465
09.06.2010	172	19	106	177	13	97	19,4819602
09.06.2010	173	16	105	177	13	97	70,32301506
09.06.2010	174	125	211	177	13	97	282,0559676
09.06.2010	175	52	150	177	13	97	20,04795106
09.06.2010	176	37	136	177	13	97	19,06296449
09.06.2010	170	49	148	178	26	122	10,94648343
09.06.2010	172	19	106	178	26	122	3,890824435
09.06.2010	173	16	105	178	26	122	83,89887354
09.06.2010	174	125	211	178	26	122	267,1754854
09.06.2010	175	52	150	178	26	122	34,52371742
09.06.2010	176	37	136	178	26	122	3,63145825
09.06.2010	177	13	97	178	26	122	16,28924507
09.06.2010	170	49	148	179	54	153	89,65932876
09.06.2010	172	19	106	179	54	153	82,9050482
09.06.2010	173	16	105	179	54	153	2,514511624
09.06.2010	174	125	211	179	54	153	348,3109764
09.06.2010	175	52	150	179	54	153	47,76106638
09.06.2010	176	37	136	179	54	153	82,47006714
09.06.2010	177	13	97	179	54	153	67,80821141
09.06.2010	178	26	122	179	54	153	81,41017434
09.06.2010	170	49	148	180	67	166	20,54834529
09.06.2010	172	19	106	180	67	166	28,08577147
09.06.2010	173	16	105	180	67	166	109,499609
09.06.2010	174	125	211	180	67	166	242,344585
09.06.2010	175	52	150	180	67	166	63,55670179
09.06.2010	176	37	136	180	67	166	28,5049114
09.06.2010	177	13	97	180	67	166	47,56729911
09.06.2010	178	26	122	180	67	166	31,47978037

09.06.2010	179	54	153	180	67	166	107,1278076
09.06.2010	170	49	148	181	60	156	33,95455362
09.06.2010	172	19	106	181	60	156	41,35926672
09.06.2010	173	16	105	181	60	156	119,7938703
09.06.2010	174	125	211	181	60	156	234,3935083
09.06.2010	175	52	150	181	60	156	75,7603378
09.06.2010	176	37	136	181	60	156	41,76176987
09.06.2010	177	13	97	181	60	156	60,77602611
09.06.2010	178	26	122	181	60	156	44,89572813
09.06.2010	179	54	153	181	60	156	117,4954782
09.06.2010	180	67	166	181	60	156	13,65762459
09.06.2010	170	49	148	182	13	96	42,48557415
09.06.2010	172	19	106	182	13	96	34,98748565
09.06.2010	173	16	105	182	13	96	53,30078361
09.06.2010	174	125	211	182	13	96	299,258879
09.06.2010	175	52	150	182	13	96	3,794449577
09.06.2010	176	37	136	182	13	96	34,53980407
09.06.2010	177	13	97	182	13	96	17,27160177
09.06.2010	178	26	122	182	13	96	32,52285315
09.06.2010	179	54	153	182	13	96	50,78566541
09.06.2010	180	67	166	182	13	96	62,3855206
09.06.2010	181	60	156	182	13	96	74,89748099
09.06.2010	170	49	148	191	128	203	36,18950463
09.06.2010	172	19	106	191	128	203	28,58654855
09.06.2010	173	16	105	191	128	203	62,22010089
09.06.2010	174	125	211	191	128	203	291,1312436
09.06.2010	175	52	150	191	128	203	12,5221669
09.06.2010	176	37	136	191	128	203	28,15419002
09.06.2010	177	13	97	191	128	203	9,355489511
09.06.2010	178	26	122	191	128	203	25,57112971
09.06.2010	179	54	153	191	128	203	59,70696373
09.06.2010	180	67	166	191	128	203	56,60651808
09.06.2010	181	60	156	191	128	203	69,63919726

09.06.2010	182	13	96	191	128	203	9,016749583
09.06.2010	170	49	148	193	171	232	62,3966681
09.06.2010	172	19	106	193	171	232	69,62518775
09.06.2010	173	16	105	193	171	232	142,8463617
09.06.2010	174	125	211	193	171	232	220,2110947
09.06.2010	175	52	150	193	171	232	102,3496693
09.06.2010	176	37	136	193	171	232	70,00775051
09.06.2010	177	13	97	193	171	232	88,83300684
09.06.2010	178	26	122	193	171	232	73,28102152
09.06.2010	179	54	153	193	171	232	140,7010574
09.06.2010	180	67	166	193	171	232	42,33563763
09.06.2010	181	60	156	193	171	232	28,68134074
09.06.2010	182	13	96	193	171	232	101,9209273
09.06.2010	191	128	203	193	171	232	97,41230458
09.06.2010	170	49	148	194	81	184	49,3263796
09.06.2010	172	19	106	194	81	184	56,72471354
09.06.2010	173	16	105	194	81	184	133,3582803
09.06.2010	174	125	211	194	81	184	224,1108878
09.06.2010	175	52	150	194	81	184	90,68930006
09.06.2010	176	37	136	194	81	184	57,12504928
09.06.2010	177	13	97	194	81	184	76,12067303
09.06.2010	178	26	122	194	81	184	60,26807563
09.06.2010	179	54	153	194	81	184	131,122215
09.06.2010	180	67	166	194	81	184	28,93796041
09.06.2010	181	60	156	194	81	184	15,37234414
09.06.2010	182	13	96	194	81	184	89,97620917
09.06.2010	191	128	203	194	81	184	84,93074428
09.06.2010	193	171	232	194	81	184	13,8692714
09.06.2010	170	49	148	172	19	106	7,578179525
09.06.2010	170	49	148	173	16	105	103,3058223
09.06.2010	172	19	106	173	16	105	105,7496605
09.06.2010	170	49	148	174	125	211	240,1210961
09.06.2010	172	19	106	174	125	211	239,4427014

09.06.2010	173	16	105	174	125	211	342,145486
09.06.2010	170	49	148	175	52	150	65,13444949
09.06.2010	172	19	106	175	52	150	69,64931348
09.06.2010	173	16	105	175	52	150	45,29801391
09.06.2010	174	125	211	175	52	150	299,2418553
09.06.2010	170	49	148	176	37	136	7,709346655
09.06.2010	172	19	106	176	37	136	0,190079212
09.06.2010	173	16	105	176	37	136	105,6719904
09.06.2010	174	125	211	176	37	136	239,5653079
09.06.2010	175	52	150	176	37	136	69,62869039
09.06.2010	170	49	148	177	13	97	55,24818329
09.06.2010	172	19	106	177	13	97	59,80143959
09.06.2010	173	16	105	177	13	97	53,00228549
09.06.2010	174	125	211	177	13	97	290,1336743
09.06.2010	175	52	150	177	13	97	9,88686018
09.06.2010	176	37	136	177	13	97	59,78338735
09.06.2010	170	49	148	179	54	153	89,1860495
09.06.2010	172	19	106	179	54	153	92,22735633
09.06.2010	173	16	105	179	54	153	16,09624169
09.06.2010	174	125	211	179	54	153	326,9598935
09.06.2010	175	52	150	179	54	153	29,20949177
09.06.2010	176	37	136	179	54	153	92,16485096
09.06.2010	177	13	97	179	54	153	37,17142244
09.06.2010	170	49	148	180	67	166	123,5198284
09.06.2010	172	19	106	180	67	166	125,6685607
09.06.2010	173	16	105	180	67	166	20,61110858
09.06.2010	174	125	211	180	67	166	362,6890187
09.06.2010	175	52	150	180	67	166	65,56258852
09.06.2010	176	37	136	180	67	166	125,5831792
09.06.2010	177	13	97	180	67	166	73,5466557
09.06.2010	179	54	153	180	67	166	36,43253769
09.06.2010	170	49	148	181	60	156	7,966337475
09.06.2010	172	19	106	181	60	156	6,227814203

09.06.2010	173	16	105	181	60	156	110,5208056
09.06.2010	174	125	211	181	60	156	233,7912495
09.06.2010	175	52	150	181	60	156	73,01528689
09.06.2010	176	37	136	181	60	156	6,398078706
09.06.2010	177	13	97	181	60	156	63,12898078
09.06.2010	179	54	153	181	60	156	96,64728901
09.06.2010	180	67	166	181	60	156	130,6043382
09.06.2010	170	49	148	182	13	96	68,5196673
09.06.2010	172	19	106	182	13	96	72,30371881
09.06.2010	173	16	105	182	13	96	37,98945979
09.06.2010	174	125	211	182	13	96	305,0080242
09.06.2010	175	52	150	182	13	96	8,495286254
09.06.2010	176	37	136	182	13	96	72,26235688
09.06.2010	177	13	97	182	13	96	15,04510285
09.06.2010	179	54	153	182	13	96	22,12729409
09.06.2010	180	67	166	182	13	96	58,50936416
09.06.2010	181	60	156	182	13	96	76,22294911
09.06.2010	170	49	148	191	128	203	62,1653475
09.06.2010	172	19	106	191	128	203	66,44675556
09.06.2010	173	16	105	191	128	203	45,98561832
09.06.2010	174	125	211	191	128	203	297,4261398
09.06.2010	175	52	150	191	128	203	3,966694033
09.06.2010	176	37	136	191	128	203	66,41976672
09.06.2010	177	13	97	191	128	203	7,301367867
09.06.2010	179	54	153	191	128	203	30,04153692
09.06.2010	180	67	166	191	128	203	66,46585187
09.06.2010	181	60	156	191	128	203	69,99536332
09.06.2010	182	13	96	191	128	203	8,03407966
09.06.2010	170	49	148	193	171	232	21,93377392
09.06.2010	172	19	106	193	171	232	16,54016836
09.06.2010	173	16	105	193	171	232	121,7336787
09.06.2010	174	125	211	193	171	232	225,6754421
09.06.2010	175	52	150	193	171	232	86,17828629

09.06.2010	176	37	136	193	171	232	16,56990389
09.06.2010	177	13	97	193	171	232	76,32242538
09.06.2010	179	54	153	193	171	232	108,512092
09.06.2010	180	67	166	193	171	232	141,4396584
09.06.2010	181	60	156	193	171	232	14,15611663
09.06.2010	182	13	96	193	171	232	88,80799008
09.06.2010	191	128	203	193	171	232	82,98650298
09.06.2010	170	49	148	194	81	184	39,23478517
09.06.2010	172	19	106	194	81	184	33,89384722
09.06.2010	173	16	105	194	81	184	138,1391439
09.06.2010	174	125	211	194	81	184	213,0325438
09.06.2010	175	52	150	194	81	184	103,5372653
09.06.2010	176	37	136	194	81	184	33,91116494
09.06.2010	177	13	97	194	81	184	93,69449573
09.06.2010	179	54	153	194	81	184	125,319154
09.06.2010	180	67	166	194	81	184	157,5545997
09.06.2010	181	60	156	194	81	184	31,32387769
09.06.2010	182	13	96	194	81	184	105,9821587
09.06.2010	191	128	203	194	81	184	100,3037575
09.06.2010	193	171	232	194	81	184	17,41923277
10.06.2010	170	49	148	172	19	106	9,519629228
10.06.2010	170	49	148	173	16	105	100,7652017
10.06.2010	172	19	106	173	16	105	110,2792838
10.06.2010	170	49	148	174	125	211	245,069645
10.06.2010	172	19	106	174	125	211	235,9976376
10.06.2010	173	16	105	174	125	211	343,2974234
10.06.2010	170	49	148	175	52	150	58,54871718
10.06.2010	172	19	106	175	52	150	67,7269731
10.06.2010	173	16	105	175	52	150	48,95997386
10.06.2010	174	125	211	175	52	150	295,490103
10.06.2010	170	49	148	176	37	136	7,753740896
10.06.2010	172	19	106	176	37	136	2,103791362
10.06.2010	173	16	105	176	37	136	108,4169488

10.06.2010	174	125	211	176	37	136	238,0612754
10.06.2010	175	52	150	176	37	136	66,21907999
10.06.2010	170	49	148	177	13	97	33,32070156
10.06.2010	172	19	106	177	13	97	42,54399958
10.06.2010	173	16	105	177	13	97	69,8989005
10.06.2010	174	125	211	177	13	97	273,4676272
10.06.2010	175	52	150	177	13	97	25,22939077
10.06.2010	176	37	136	177	13	97	41,0036152
10.06.2010	170	49	148	179	54	153	86,17529909
10.06.2010	172	19	106	179	54	153	95,69199974
10.06.2010	173	16	105	179	54	153	15,62411813
10.06.2010	174	125	211	179	54	153	327,8512679
10.06.2010	175	52	150	179	54	153	33,36891866
10.06.2010	176	37	136	179	54	153	93,88715391
10.06.2010	177	13	97	179	54	153	54,62578601
10.06.2010	170	49	148	180	67	166	11,12369469
10.06.2010	172	19	106	180	67	166	20,18199099
10.06.2010	173	16	105	180	67	166	90,93010855
10.06.2010	174	125	211	180	67	166	253,491992
10.06.2010	175	52	150	180	67	166	47,55058591
10.06.2010	176	37	136	180	67	166	18,68344261
10.06.2010	177	13	97	180	67	166	22,36320504
10.06.2010	179	54	153	180	67	166	76,05152181
10.06.2010	170	49	148	181	60	156	13,67084527
10.06.2010	172	19	106	181	60	156	4,188217627
10.06.2010	173	16	105	181	60	156	114,3935801
10.06.2010	174	125	211	181	60	156	232,2781583
10.06.2010	175	52	150	181	60	156	71,89945637
10.06.2010	176	37	136	181	60	156	5,980702788
10.06.2010	177	13	97	181	60	156	46,72518595
10.06.2010	179	54	153	181	60	156	99,84253273
10.06.2010	180	67	166	181	60	156	24,36204561
10.06.2010	170	49	148	182	13	96	70,7403736

10.06.2010	172	19	106	182	13	96	80,12994293
10.06.2010	173	16	105	182	13	96	34,89312837
10.06.2010	174	125	211	182	13	96	309,5999157
10.06.2010	175	52	150	182	13	96	14,20325498
10.06.2010	176	37	136	182	13	96	78,4874677
10.06.2010	177	13	97	182	13	96	37,77964807
10.06.2010	179	54	153	182	13	96	19,41368616
10.06.2010	180	67	166	182	13	96	60,02140526
10.06.2010	181	60	156	182	13	96	84,31818298
10.06.2010	170	49	148	191	128	203	57,9480569
10.06.2010	172	19	106	191	128	203	67,0442282
10.06.2010	173	16	105	191	128	203	50,7150075
10.06.2010	174	125	211	191	128	203	294,039609
10.06.2010	175	52	150	191	128	203	2,033936391
10.06.2010	176	37	136	191	128	203	65,57939425
10.06.2010	177	13	97	191	128	203	24,68798976
10.06.2010	179	54	153	191	128	203	35,15296501
10.06.2010	180	67	166	191	128	203	46,89585848
10.06.2010	181	60	156	191	128	203	71,20515453
10.06.2010	182	13	96	191	128	203	15,86250601
10.06.2010	170	49	148	193	171	232	20,20972027
10.06.2010	172	19	106	193	171	232	11,38096803
10.06.2010	173	16	105	193	171	232	120,1313779
10.06.2010	174	125	211	193	171	232	228,4842662
10.06.2010	175	52	150	193	171	232	78,75796311
10.06.2010	176	37	136	193	171	232	12,59480982
10.06.2010	177	13	97	193	171	232	53,5304201
10.06.2010	179	54	153	193	171	232	105,8445132
10.06.2010	180	67	166	193	171	232	31,26066325
10.06.2010	181	60	156	193	171	232	7,600792126
10.06.2010	182	13	96	193	171	232	90,86104461
10.06.2010	191	128	203	193	171	232	78,14556119
10.06.2010	170	49	148	194	81	184	25,93588885

10.06.2010	172	19	106	194	81	184	16,87321079
10.06.2010	173	16	105	194	81	184	125,8731683
10.06.2010	174	125	211	194	81	184	223,3811274
10.06.2010	175	52	150	194	81	184	84,46984173
10.06.2010	176	37	136	194	81	184	18,25577001
10.06.2010	177	13	97	194	81	184	59,24795266
10.06.2010	179	54	153	194	81	184	111,6128398
10.06.2010	180	67	166	194	81	184	36,93924312
10.06.2010	181	60	156	194	81	184	12,82929078
10.06.2010	182	13	96	194	81	184	96,62583787
10.06.2010	191	128	203	194	81	184	83,83522251
10.06.2010	193	171	232	194	81	184	5,77790782
10.06.2010	170	49	148	172	19	106	32,55824429
10.06.2010	170	49	148	173	16	105	121,7795992
10.06.2010	172	19	106	173	16	105	98,19610579
10.06.2010	170	49	148	174	125	211	212,7183844
10.06.2010	172	19	106	174	125	211	238,1936942
10.06.2010	173	16	105	174	125	211	334,3804274
10.06.2010	170	49	148	175	52	150	70,28331485
10.06.2010	172	19	106	175	52	150	43,38701148
10.06.2010	173	16	105	175	52	150	55,07895718
10.06.2010	174	125	211	175	52	150	281,1277011
10.06.2010	170	49	148	176	37	136	24,10467479
10.06.2010	172	19	106	176	37	136	8,68820038
10.06.2010	173	16	105	176	37	136	102,6589652
10.06.2010	174	125	211	176	37	136	232,4071485
10.06.2010	175	52	150	176	37	136	48,72816943
10.06.2010	170	49	148	177	13	97	62,61357549
10.06.2010	172	19	106	177	13	97	34,03851155
10.06.2010	173	16	105	177	13	97	64,93653678
10.06.2010	174	125	211	177	13	97	272,1653898
10.06.2010	175	52	150	177	13	97	9,97055611
10.06.2010	176	37	136	177	13	97	40,04969452

10.06.2010	170	49	148	178	26	122	54,6825552
10.06.2010	172	19	106	178	26	122	23,54240583
10.06.2010	173	16	105	178	26	122	77,20568593
10.06.2010	174	125	211	178	26	122	261,4208062
10.06.2010	175	52	150	178	26	122	22,43458166
10.06.2010	176	37	136	178	26	122	30,88333899
10.06.2010	177	13	97	178	26	122	12,47446874
10.06.2010	170	49	148	179	54	153	72,44873599
10.06.2010	172	19	106	179	54	153	47,02540118
10.06.2010	173	16	105	179	54	153	51,17068666
10.06.2010	174	125	211	179	54	153	284,0878656
10.06.2010	175	52	150	179	54	153	5,228906042
10.06.2010	176	37	136	179	54	153	51,7705955
10.06.2010	177	13	97	179	54	153	14,71917749
10.06.2010	178	26	122	179	54	153	27,14391714
10.06.2010	170	49	148	180	67	166	15,08227944
10.06.2010	172	19	106	180	67	166	17,48625445
10.06.2010	173	16	105	180	67	166	110,0181863
10.06.2010	174	125	211	180	67	166	224,5397535
10.06.2010	175	52	150	180	67	166	56,88428084
10.06.2010	176	37	136	180	67	166	9,063211244
10.06.2010	177	13	97	180	67	166	48,58532121
10.06.2010	178	26	122	180	67	166	39,87973308
10.06.2010	179	54	153	180	67	166	59,58096503
10.06.2010	170	49	148	181	60	156	10,99136229
10.06.2010	172	19	106	181	60	156	43,48391748
10.06.2010	173	16	105	181	60	156	129,7526982
10.06.2010	174	125	211	181	60	156	205,5862437
10.06.2010	175	52	150	181	60	156	79,71483433
10.06.2010	176	37	136	181	60	156	34,94796416
10.06.2010	177	13	97	181	60	156	72,5314105
10.06.2010	178	26	122	181	60	156	65,21503921
10.06.2010	179	54	153	181	60	156	81,50099241

10.06.2010	180	67	166	181	60	156	25,99884815
10.06.2010	170	49	148	182	13	96	108,7065324
10.06.2010	172	19	106	182	13	96	84,92781956
10.06.2010	173	16	105	182	13	96	13,27972333
10.06.2010	174	125	211	182	13	96	321,2204841
10.06.2010	175	52	150	182	13	96	41,89446739
10.06.2010	176	37	136	182	13	96	89,3887789
10.06.2010	177	13	97	182	13	96	51,79623655
10.06.2010	178	26	122	182	13	96	64,13377968
10.06.2010	179	54	153	182	13	96	37,90472129
10.06.2010	180	67	166	182	13	96	96,79325468
10.06.2010	181	60	156	182	13	96	116,8518817
10.06.2010	170	49	148	191	128	203	61,68795271
10.06.2010	172	19	106	191	128	203	33,14130681
10.06.2010	173	16	105	191	128	203	65,74302496
10.06.2010	174	125	211	191	128	203	271,255057
10.06.2010	175	52	150	191	128	203	10,71884975
10.06.2010	176	37	136	191	128	203	39,1244711
10.06.2010	177	13	97	191	128	203	0,926331495
10.06.2010	178	26	122	191	128	203	11,79219017
10.06.2010	179	54	153	191	128	203	15,35352997
10.06.2010	180	67	166	191	128	203	47,6566531
10.06.2010	181	60	156	191	128	203	71,6134626
10.06.2010	182	13	96	191	128	203	52,58765644
10.06.2010	170	49	148	193	171	232	5,900880488
10.06.2010	172	19	106	193	171	232	38,43973182
10.06.2010	173	16	105	193	171	232	126,7690837
10.06.2010	174	125	211	193	171	232	207,9832287
10.06.2010	175	52	150	193	171	232	75,82576407
10.06.2010	176	37	136	193	171	232	30,00453197
10.06.2010	177	13	97	193	171	232	68,32105045
10.06.2010	178	26	122	193	171	232	60,55455047
10.06.2010	179	54	153	193	171	232	77,84863478

10.06.2010	180	67	166	193	171	232	20,97492024
10.06.2010	181	60	156	193	171	232	5,372890787
10.06.2010	182	13	96	193	171	232	113,7619718
10.06.2010	191	128	203	193	171	232	67,39735526
10.06.2010	170	49	148	194	81	184	33,98061339
10.06.2010	172	19	106	194	81	184	66,46360952
10.06.2010	173	16	105	194	81	184	151,5253253
10.06.2010	174	125	211	194	81	184	186,191345
10.06.2010	175	52	150	194	81	184	102,8111558
10.06.2010	176	37	136	194	81	184	58,0810975
10.06.2010	177	13	97	194	81	184	95,84686553
10.06.2010	178	26	122	194	81	184	88,56640895
10.06.2010	179	54	153	194	81	184	104,3657779
10.06.2010	180	67	166	194	81	184	49,03804228
10.06.2010	181	60	156	194	81	184	23,38244732
10.06.2010	182	13	96	194	81	184	138,8430414
10.06.2010	191	128	203	194	81	184	94,93200787
10.06.2010	193	171	232	194	81	184	28,07998198
10.06.2010	170	49	148	172	19	106	38,13978588
10.06.2010	170	49	148	173	16	105	131,3366893
10.06.2010	172	19	106	173	16	105	101,2573301
10.06.2010	170	49	148	174	125	211	213,8927727
10.06.2010	172	19	106	174	125	211	240,5380145
10.06.2010	173	16	105	174	125	211	341,759437
10.06.2010	170	49	148	175	52	150	83,80154655
10.06.2010	172	19	106	175	52	150	50,80647028
10.06.2010	173	16	105	175	52	150	50,77392557
10.06.2010	174	125	211	175	52	150	291,2960826
10.06.2010	170	49	148	176	37	136	31,88646062
10.06.2010	172	19	106	176	37	136	6,398784544
10.06.2010	173	16	105	176	37	136	106,6817356
10.06.2010	174	125	211	176	37	136	235,0859169
10.06.2010	175	52	150	176	37	136	56,51645069

10.06.2010	170	49	148	177	13	97	70,81171387
10.06.2010	172	19	106	177	13	97	34,71665553
10.06.2010	173	16	105	177	13	97	68,78720879
10.06.2010	174	125	211	177	13	97	274,2061133
10.06.2010	175	52	150	177	13	97	18,44992689
10.06.2010	176	37	136	177	13	97	40,90348305
10.06.2010	170	49	148	178	26	122	54,52292239
10.06.2010	172	19	106	178	26	122	16,81556568
10.06.2010	173	16	105	178	26	122	86,85850496
10.06.2010	174	125	211	178	26	122	255,6685702
10.06.2010	175	52	150	178	26	122	36,09466106
10.06.2010	176	37	136	178	26	122	23,19978885
10.06.2010	177	13	97	178	26	122	18,55926653
10.06.2010	170	49	148	179	54	153	99,77791786
10.06.2010	172	19	106	179	54	153	66,00225235
10.06.2010	173	16	105	179	54	153	37,79829206
10.06.2010	174	125	211	179	54	153	306,0672568
10.06.2010	175	52	150	179	54	153	16,01212888
10.06.2010	176	37	136	179	54	153	71,91886315
10.06.2010	177	13	97	179	54	153	31,88121967
10.06.2010	178	26	122	179	54	153	50,39886778
10.06.2010	170	49	148	180	67	166	30,4393921
10.06.2010	172	19	106	180	67	166	7,869371209
10.06.2010	173	16	105	180	67	166	107,9037348
10.06.2010	174	125	211	180	67	166	233,8811745
10.06.2010	175	52	150	180	67	166	57,81658491
10.06.2010	176	37	136	180	67	166	1,466199631
10.06.2010	177	13	97	180	67	166	42,31675148
10.06.2010	178	26	122	180	67	166	24,66438003
10.06.2010	179	54	153	180	67	166	73,26228379
10.06.2010	170	49	148	181	60	156	1,21710081
10.06.2010	172	19	106	181	60	156	37,85249859
10.06.2010	173	16	105	181	60	156	130,447044

10.06.2010	174	125	211	181	60	156	214,9876559
10.06.2010	175	52	150	181	60	156	83,06896525
10.06.2010	176	37	136	181	60	156	31,64933043
10.06.2010	177	13	97	181	60	156	70,25709271
10.06.2010	178	26	122	181	60	156	54,13254401
10.06.2010	179	54	153	181	60	156	99,05580169
10.06.2010	180	67	166	181	60	156	30,21362741
10.06.2010	170	49	148	182	13	96	130,5739045
10.06.2010	172	19	106	182	13	96	99,94886615
10.06.2010	173	16	105	182	13	96	2,907652836
10.06.2010	174	125	211	182	13	96	340,48558
10.06.2010	175	52	150	182	13	96	49,29258283
10.06.2010	176	37	136	182	13	96	105,4574818
10.06.2010	177	13	97	182	13	96	67,122996
10.06.2010	178	26	122	182	13	96	85,31915642
10.06.2010	179	54	153	182	13	96	35,82703229
10.06.2010	180	67	166	182	13	96	106,6993874
10.06.2010	181	60	156	182	13	96	129,7024972
10.06.2010	170	49	148	191	128	203	61,78517409
10.06.2010	172	19	106	191	128	203	24,31175542
10.06.2010	173	16	105	191	128	203	80,35023997
10.06.2010	174	125	211	191	128	203	262,7474377
10.06.2010	175	52	150	191	128	203	29,80639636
10.06.2010	176	37	136	191	128	203	30,68234126
10.06.2010	177	13	97	191	128	203	11,58548001
10.06.2010	178	26	122	191	128	203	7,517146214
10.06.2010	179	54	153	191	128	203	43,41760389
10.06.2010	180	67	166	191	128	203	32,14193953
10.06.2010	181	60	156	191	128	203	61,35481973
10.06.2010	182	13	96	191	128	203	78,70329571
10.06.2010	170	49	148	194	81	184	25,32622727
10.06.2010	172	19	106	194	81	184	63,43721067
10.06.2010	173	16	105	194	81	184	153,9862775

10.06.2010	174	125	211	194	81	184	197,0413776
10.06.2010	175	52	150	194	81	184	108,0410142
10.06.2010	176	37	136	194	81	184	57,14149124
10.06.2010	177	13	97	194	81	184	95,88511899
10.06.2010	178	26	122	194	81	184	79,84520475
10.06.2010	179	54	153	194	81	184	124,0527918
10.06.2010	180	67	166	194	81	184	55,68315288
10.06.2010	181	60	156	194	81	184	25,73011944
10.06.2010	182	13	96	194	81	184	153,4450863
10.06.2010	191	128	203	194	81	184	87,08434039
10.06.2010	170	49	148	172	19	106	75,20666048
10.06.2010	170	49	148	173	16	105	112,0772865
10.06.2010	172	19	106	173	16	105	37,3216552
10.06.2010	170	49	148	174	125	211	236,2917799
10.06.2010	172	19	106	174	125	211	311,3359836
10.06.2010	173	16	105	174	125	211	348,3631873
10.06.2010	170	49	148	175	52	150	57,41800321
10.06.2010	172	19	106	175	52	150	18,05952588
10.06.2010	173	16	105	175	52	150	55,37188849
10.06.2010	174	125	211	175	52	150	293,3611863
10.06.2010	170	49	148	176	37	136	34,24526402
10.06.2010	172	19	106	176	37	136	43,38742785
10.06.2010	173	16	105	176	37	136	80,69886771
10.06.2010	174	125	211	176	37	136	268,6309387
10.06.2010	175	52	150	176	37	136	25,40527956
10.06.2010	170	49	148	177	13	97	49,16157314
10.06.2010	172	19	106	177	13	97	27,84417172
10.06.2010	173	16	105	177	13	97	65,08818491
10.06.2010	174	125	211	177	13	97	284,3079278
10.06.2010	175	52	150	177	13	97	10,25943634
10.06.2010	176	37	136	177	13	97	15,71605686
10.06.2010	170	49	148	178	26	122	24,26731706
10.06.2010	172	19	106	178	26	122	54,02564841

10.06.2010	173	16	105	178	26	122	91,34715313
10.06.2010	174	125	211	178	26	122	257,8513141
10.06.2010	175	52	150	178	26	122	35,98262746
10.06.2010	176	37	136	178	26	122	10,78060858
10.06.2010	177	13	97	178	26	122	26,4838397
10.06.2010	170	49	148	179	54	153	106,5812721
10.06.2010	172	19	106	179	54	153	32,51618698
10.06.2010	173	16	105	179	54	153	6,538420987
10.06.2010	174	125	211	179	54	153	342,8584449
10.06.2010	175	52	150	179	54	153	50,41526382
10.06.2010	176	37	136	179	54	153	75,81879511
10.06.2010	177	13	97	179	54	153	60,35426562
10.06.2010	178	26	122	179	54	153	86,36637596
10.06.2010	170	49	148	180	67	166	18,80055414
10.06.2010	172	19	106	180	67	166	58,00593475
10.06.2010	173	16	105	180	67	166	95,26330366
10.06.2010	174	125	211	180	67	166	253,4486593
10.06.2010	175	52	150	180	67	166	39,96378709
10.06.2010	176	37	136	180	67	166	15,57374096
10.06.2010	177	13	97	180	67	166	30,95987195
10.06.2010	178	26	122	180	67	166	5,628238732
10.06.2010	179	54	153	180	67	166	90,09478958
10.06.2010	170	49	148	181	60	156	16,04312467
10.06.2010	172	19	106	181	60	156	87,41288114
10.06.2010	173	16	105	181	60	156	123,4100172
10.06.2010	174	125	211	181	60	156	226,0041034
10.06.2010	175	52	150	181	60	156	70,23291853
10.06.2010	176	37	136	181	60	156	48,98579537
10.06.2010	177	13	97	181	60	156	62,98007297
10.06.2010	178	26	122	181	60	156	39,72610061
10.06.2010	179	54	153	181	60	156	117,5866172
10.06.2010	180	67	166	181	60	156	34,11511496
10.06.2010	170	49	148	191	128	203	48,6378126

10.06.2010	172	19	106	191	128	203	28,20739645
10.06.2010	173	16	105	191	128	203	65,47463257
10.06.2010	174	125	211	191	128	203	283,8442126
10.06.2010	175	52	150	191	128	203	10,5103664
10.06.2010	176	37	136	191	128	203	15,28247505
10.06.2010	177	13	97	191	128	203	0,585863479
10.06.2010	178	26	122	191	128	203	26,03842394
10.06.2010	179	54	153	191	128	203	60,70836639
10.06.2010	180	67	166	191	128	203	30,4718676
10.06.2010	181	60	156	191	128	203	62,42373231
10.06.2010	170	49	148	193	171	232	36,15017288
10.06.2010	172	19	106	193	171	232	104,9306979
10.06.2010	173	16	105	193	171	232	139,8119502
10.06.2010	174	125	211	193	171	232	212,8846869
10.06.2010	175	52	150	193	171	232	88,4410305
10.06.2010	176	37	136	193	171	232	68,63517946
10.06.2010	177	13	97	193	171	232	81,96305114
10.06.2010	178	26	122	193	171	232	59,73607822
10.06.2010	179	54	153	193	171	232	133,7392663
10.06.2010	180	67	166	193	171	232	54,11018022
10.06.2010	181	60	156	193	171	232	20,13225122
10.06.2010	191	128	203	193	171	232	81,39219832
10.06.2010	170	49	148	194	81	184	56,8655408
10.06.2010	172	19	106	194	81	184	127,7923338
10.06.2010	173	16	105	194	81	184	162,6849323
10.06.2010	174	125	211	194	81	184	191,713214
10.06.2010	175	52	150	194	81	184	111,1659954
10.06.2010	176	37	136	194	81	184	90,53229637
10.06.2010	177	13	97	194	81	184	104,3837348
10.06.2010	178	26	122	194	81	184	81,06221007
10.06.2010	179	54	153	194	81	184	156,5924245
10.06.2010	180	67	166	194	81	184	75,49914245
10.06.2010	181	60	156	194	81	184	41,55111579

10.06.2010	191	128	203	194	81	184	103,8199711
10.06.2010	193	171	232	194	81	184	22,89645936
10.06.2010	170	49	148	172	19	106	7,805986504
10.06.2010	170	49	148	173	16	105	115,3115192
10.06.2010	172	19	106	173	16	105	122,2419202
10.06.2010	170	49	148	174	125	211	235,1377812
10.06.2010	172	19	106	174	125	211	228,3672599
10.06.2010	173	16	105	174	125	211	350,4352572
10.06.2010	170	49	148	175	52	150	58,4107883
10.06.2010	172	19	106	175	52	150	66,04719286
10.06.2010	173	16	105	175	52	150	61,07868004
10.06.2010	174	125	211	175	52	150	291,5824718
10.06.2010	170	49	148	176	37	136	3,097183137
10.06.2010	172	19	106	176	37	136	10,84826211
10.06.2010	173	16	105	176	37	136	112,9951773
10.06.2010	174	125	211	176	37	136	237,4423764
10.06.2010	175	52	150	176	37	136	55,63195116
10.06.2010	170	49	148	177	13	97	54,76269192
10.06.2010	172	19	106	177	13	97	62,44557773
10.06.2010	173	16	105	177	13	97	65,14353208
10.06.2010	174	125	211	177	13	97	287,5992074
10.06.2010	175	52	150	177	13	97	4,077852585
10.06.2010	176	37	136	177	13	97	51,94120982
10.06.2010	170	49	148	178	26	122	40,87465518
10.06.2010	172	19	106	178	26	122	48,62945493
10.06.2010	173	16	105	178	26	122	78,57367462
10.06.2010	174	125	211	178	26	122	273,4168229
10.06.2010	175	52	150	178	26	122	18,16723773
10.06.2010	176	37	136	178	26	122	37,97816432
10.06.2010	177	13	97	178	26	122	14,22422577
10.06.2010	170	49	148	179	54	153	104,5630912
10.06.2010	172	19	106	179	54	153	111,6228339
10.06.2010	173	16	105	179	54	153	11,49232813

10.06.2010	174	125	211	179	54	153	339,5869261
10.06.2010	175	52	150	179	54	153	49,63697626
10.06.2010	176	37	136	179	54	153	102,1731904
10.06.2010	177	13	97	179	54	153	53,70738511
10.06.2010	178	26	122	179	54	153	67,24305677
10.06.2010	170	49	148	180	67	166	10,96462178
10.06.2010	172	19	106	180	67	166	5,692864472
10.06.2010	173	16	105	180	67	166	121,4890954
10.06.2010	174	125	211	180	67	166	229,6868783
10.06.2010	175	52	150	180	67	166	66,91935666
10.06.2010	176	37	136	180	67	166	13,95820911
10.06.2010	177	13	97	180	67	166	63,49698434
10.06.2010	178	26	122	180	67	166	50,08569207
10.06.2010	179	54	153	180	67	166	111,0957303
10.06.2010	170	49	148	181	60	156	27,00713916
10.06.2010	172	19	106	181	60	156	20,80477543
10.06.2010	173	16	105	181	60	156	130,814276
10.06.2010	174	125	211	181	60	156	223,4670363
10.06.2010	175	52	150	181	60	156	79,88659794
10.06.2010	176	37	136	181	60	156	29,94954201
10.06.2010	177	13	97	181	60	156	76,77935618
10.06.2010	178	26	122	181	60	156	64,12124379
10.06.2010	179	54	153	181	60	156	121,0005732
10.06.2010	180	67	166	181	60	156	16,05803764
10.06.2010	170	49	148	182	13	96	81,11361737
10.06.2010	172	19	106	182	13	96	88,58670183
10.06.2010	173	16	105	182	13	96	38,66745678
10.06.2010	174	125	211	182	13	96	314,9743029
10.06.2010	175	52	150	182	13	96	23,43531001
10.06.2010	176	37	136	182	13	96	78,45702249
10.06.2010	177	13	97	182	13	96	27,47384444
10.06.2010	178	26	122	182	13	96	41,57284846
10.06.2010	179	54	153	182	13	96	27,20159048

10.06.2010	180	67	166	182	13	96	88,92508107
10.06.2010	181	60	156	182	13	96	100,7420721
10.06.2010	170	49	148	191	128	203	58,77168094
10.06.2010	172	19	106	191	128	203	66,40378579
10.06.2010	173	16	105	191	128	203	60,6865654
10.06.2010	174	125	211	191	128	203	291,9704251
10.06.2010	175	52	150	191	128	203	0,391858334
10.06.2010	176	37	136	191	128	203	55,99647777
10.06.2010	177	13	97	191	128	203	4,473933306
10.06.2010	178	26	122	191	128	203	18,55464244
10.06.2010	179	54	153	191	128	203	49,24409117
10.06.2010	180	67	166	191	128	203	67,26044981
10.06.2010	181	60	156	191	128	203	80,19912529
10.06.2010	182	13	96	191	128	203	23,04391994
10.06.2010	170	49	148	193	171	232	30,25425802
10.06.2010	172	19	106	193	171	232	22,70830136
10.06.2010	173	16	105	193	171	232	139,5444888
10.06.2010	174	125	211	193	171	232	213,677908
10.06.2010	175	52	150	193	171	232	86,69893977
10.06.2010	176	37	136	193	171	232	33,35186102
10.06.2010	177	13	97	193	171	232	83,36116669
10.06.2010	178	26	122	193	171	232	70,07803212
10.06.2010	179	54	153	193	171	232	129,4976974
10.06.2010	180	67	166	193	171	232	20,01571109
10.06.2010	181	60	156	193	171	232	10,08584731
10.06.2010	182	13	96	193	171	232	108,3041261
10.06.2010	191	128	203	193	171	232	87,03168577
10.06.2010	170	49	148	194	81	184	44,92991724
10.06.2010	172	19	106	194	81	184	37,97209905
10.06.2010	173	16	105	194	81	184	146,8632245
10.06.2010	174	125	211	194	81	184	211,8507222
10.06.2010	175	52	150	194	81	184	98,00012105
10.06.2010	176	37	136	194	81	184	47,96619826

10.06.2010	177	13	97	194	81	184	94,99796963
10.06.2010	178	26	122	194	81	184	82,53695973
10.06.2010	179	54	153	194	81	184	137,4632835
10.06.2010	180	67	166	194	81	184	34,02152414
10.06.2010	181	60	156	194	81	184	18,48074405
10.06.2010	182	13	96	194	81	184	118,3219049
10.06.2010	191	128	203	194	81	184	98,30219867
10.06.2010	193	171	232	194	81	184	16,52131729
10.06.2010	172	19	106	173	16	105	108,4408569
10.06.2010	172	19	106	174	125	211	191,920666
10.06.2010	173	16	105	174	125	211	299,0007031
10.06.2010	172	19	106	175	52	150	55,52428201
10.06.2010	173	16	105	175	52	150	56,82271728
10.06.2010	174	125	211	175	52	150	247,3414468
10.06.2010	172	19	106	177	13	97	33,27092147
10.06.2010	173	16	105	177	13	97	77,31546433
10.06.2010	174	125	211	177	13	97	225,0382946
10.06.2010	175	52	150	177	13	97	22,30314505
10.06.2010	172	19	106	178	26	122	32,18041624
10.06.2010	173	16	105	178	26	122	79,98699476
10.06.2010	174	125	211	178	26	122	223,476939
10.06.2010	175	52	150	178	26	122	24,12040794
10.06.2010	177	13	97	178	26	122	3,72962346
10.06.2010	172	19	106	179	54	153	101,2865401
10.06.2010	173	16	105	179	54	153	7,56983154
10.06.2010	174	125	211	179	54	153	292,1963284
10.06.2010	175	52	150	179	54	153	49,26884713
10.06.2010	177	13	97	179	54	153	69,90103265
10.06.2010	178	26	122	179	54	153	72,52356452
10.06.2010	172	19	106	180	67	166	15,36293993
10.06.2010	173	16	105	180	67	166	122,026413
10.06.2010	174	125	211	180	67	166	177,4015
10.06.2010	175	52	150	180	67	166	70,51118976

10.06.2010	177	13	97	180	67	166	48,38962801
10.06.2010	178	26	122	180	67	166	47,48424493
10.06.2010	179	54	153	180	67	166	115,0416254
10.06.2010	172	19	106	181	60	156	45,85175922
10.06.2010	173	16	105	181	60	156	144,7992573
10.06.2010	174	125	211	181	60	156	155,5055914
10.06.2010	175	52	150	181	60	156	98,16220362
10.06.2010	177	13	97	181	60	156	76,97985158
10.06.2010	178	26	122	181	60	156	76,78641442
10.06.2010	179	54	153	181	60	156	138,3801469
10.06.2010	180	67	166	181	60	156	31,2392742
10.06.2010	172	19	106	182	13	96	55,52330593
10.06.2010	173	16	105	182	13	96	56,84210706
10.06.2010	174	125	211	182	13	96	247,3384522
10.06.2010	175	52	150	182	13	96	0
10.06.2010	177	13	97	182	13	96	22,30010743
10.06.2010	178	26	122	182	13	96	24,11235535
10.06.2010	179	54	153	182	13	96	49,28790991
10.06.2010	180	67	166	182	13	96	70,51208646
10.06.2010	181	60	156	182	13	96	98,16951865
10.06.2010	172	19	106	191	128	203	53,57368349
10.06.2010	173	16	105	191	128	203	56,3926935
10.06.2010	174	125	211	191	128	203	245,4788581
10.06.2010	175	52	150	191	128	203	5,611362416
10.06.2010	177	13	97	191	128	203	21,04306513
10.06.2010	178	26	122	191	128	203	23,59471874
10.06.2010	179	54	153	191	128	203	48,93275431
10.06.2010	180	67	166	191	128	203	68,22215407
10.06.2010	181	60	156	191	128	203	94,95655271
10.06.2010	182	13	96	191	128	203	5,645064596
10.06.2010	172	19	106	193	171	232	43,68463213
10.06.2010	173	16	105	193	171	232	144,6058136
10.06.2010	174	125	211	193	171	232	155,0639255

10.06.2010	175	52	150	193	171	232	96,92697993
10.06.2010	177	13	97	193	171	232	75,46282374
10.06.2010	178	26	122	193	171	232	75,09181462
10.06.2010	179	54	153	193	171	232	138,0768158
10.06.2010	180	67	166	193	171	232	28,71391718
10.06.2010	181	60	156	193	171	232	3,971245606
10.06.2010	182	13	96	193	171	232	96,93294384
10.06.2010	191	128	203	193	171	232	93,9062489
10.06.2010	172	19	106	194	81	184	59,89614343
10.06.2010	173	16	105	194	81	184	157,9395843
10.06.2010	174	125	211	194	81	184	143,9284389
10.06.2010	175	52	150	194	81	184	112,2282141
10.06.2010	177	13	97	194	81	184	91,13331921
10.06.2010	178	26	122	194	81	184	90,95339958
10.06.2010	179	54	153	194	81	184	151,6628443
10.06.2010	180	67	166	194	81	184	45,03593911
10.06.2010	181	60	156	194	81	184	14,16695958
10.06.2010	182	13	96	194	81	184	112,2361012
10.06.2010	191	128	203	194	81	184	108,9339637
10.06.2010	193	171	232	194	81	184	16,33299251
10.06.2010	170	49	148	172	19	106	5,06124943
10.06.2010	170	49	148	173	16	105	111,4504625
10.06.2010	172	19	106	173	16	105	116,0977158
10.06.2010	170	49	148	174	125	211	230,009042
10.06.2010	172	19	106	174	125	211	225,3362404
10.06.2010	173	16	105	174	125	211	341,433935
10.06.2010	170	49	148	175	52	150	60,66490546
10.06.2010	172	19	106	175	52	150	65,64327182
10.06.2010	173	16	105	175	52	150	54,1190267
10.06.2010	174	125	211	175	52	150	289,698149
10.06.2010	170	49	148	176	37	136	5,785718974
10.06.2010	172	19	106	176	37	136	10,73653138
10.06.2010	173	16	105	176	37	136	107,0990943

10.06.2010	174	125	211	176	37	136	234,5497764
10.06.2010	175	52	150	176	37	136	55,58078353
10.06.2010	170	49	148	177	13	97	41,44467894
10.06.2010	172	19	106	177	13	97	46,4922446
10.06.2010	173	16	105	177	13	97	77,46042745
10.06.2010	174	125	211	177	13	97	267,6718251
10.06.2010	175	52	150	177	13	97	23,34107714
10.06.2010	176	37	136	177	13	97	35,80534385
10.06.2010	170	49	148	178	26	122	14,76084719
10.06.2010	172	19	106	178	26	122	19,81045819
10.06.2010	173	16	105	178	26	122	98,70136494
10.06.2010	174	125	211	178	26	122	243,2757741
10.06.2010	175	52	150	178	26	122	46,58453328
10.06.2010	176	37	136	178	26	122	9,179074534
10.06.2010	177	13	97	178	26	122	26,68396676
10.06.2010	170	49	148	179	54	153	96,67233269
10.06.2010	172	19	106	179	54	153	101,3820938
10.06.2010	173	16	105	179	54	153	15,1438388
10.06.2010	174	125	211	179	54	153	326,6802838
10.06.2010	175	52	150	179	54	153	39,00261473
10.06.2010	176	37	136	179	54	153	92,20889324
10.06.2010	177	13	97	179	54	153	62,34091068
10.06.2010	178	26	122	179	54	153	83,71737831
10.06.2010	170	49	148	180	67	166	20,8591909
10.06.2010	172	19	106	180	67	166	15,97287544
10.06.2010	173	16	105	180	67	166	128,726226
10.06.2010	174	125	211	180	67	166	213,3683395
10.06.2010	175	52	150	180	67	166	80,17952592
10.06.2010	176	37	136	180	67	166	26,63839958
10.06.2010	177	13	97	180	67	166	62,14253146
10.06.2010	178	26	122	180	67	166	35,52091769
10.06.2010	179	54	153	180	67	166	114,3590298
10.06.2010	170	49	148	181	60	156	29,96024659

10.06.2010	172	19	106	181	60	156	25,11762119
10.06.2010	173	16	105	181	60	156	135,9786616
10.06.2010	174	125	211	181	60	156	207,1355892
10.06.2010	175	52	150	181	60	156	88,54162291
10.06.2010	176	37	136	181	60	156	35,74575913
10.06.2010	177	13	97	181	60	156	71,08378044
10.06.2010	178	26	122	181	60	156	44,54043079
10.06.2010	179	54	153	181	60	156	121,8332873
10.06.2010	180	67	166	181	60	156	9,154440615
10.06.2010	170	49	148	182	13	96	69,93636506
10.06.2010	172	19	106	182	13	96	74,92262157
10.06.2010	173	16	105	182	13	96	46,50924102
10.06.2010	174	125	211	182	13	96	298,7427878
10.06.2010	175	52	150	182	13	96	9,294951217
10.06.2010	176	37	136	182	13	96	64,81450293
10.06.2010	177	13	97	182	13	96	31,60492051
10.06.2010	178	26	122	182	13	96	55,78071958
10.06.2010	179	54	153	182	13	96	31,64248023
10.06.2010	180	67	166	182	13	96	89,47104436
10.06.2010	181	60	156	182	13	96	97,81404047
10.06.2010	170	49	148	191	128	203	60,42516209
10.06.2010	172	19	106	191	128	203	65,41307588
10.06.2010	173	16	105	191	128	203	54,65025274
10.06.2010	174	125	211	191	128	203	289,3511673
10.06.2010	175	52	150	191	128	203	0,71753329
10.06.2010	176	37	136	191	128	203	55,30945807
10.06.2010	177	13	97	191	128	203	22,81781991
10.06.2010	178	26	122	191	128	203	46,29043509
10.06.2010	179	54	153	191	128	203	39,54401462
10.06.2010	180	67	166	191	128	203	80,01155217
10.06.2010	181	60	156	191	128	203	88,40430696
10.06.2010	182	13	96	191	128	203	9,511560739
10.06.2010	170	49	148	193	171	232	30,50430852

10.06.2010	172	19	106	193	171	232	25,66386144
10.06.2010	173	16	105	193	171	232	136,4168989
10.06.2010	174	125	211	193	171	232	206,7729644
10.06.2010	175	52	150	193	171	232	89,0446048
10.06.2010	176	37	136	193	171	232	36,29019877
10.06.2010	177	13	97	193	171	232	71,61875981
10.06.2010	178	26	122	193	171	232	45,08024166
10.06.2010	179	54	153	193	171	232	122,2849169
10.06.2010	180	67	166	193	171	232	9,701025511
10.06.2010	181	60	156	193	171	232	0,55417137
10.06.2010	182	13	96	193	171	232	98,31542926
10.06.2010	191	128	203	193	171	232	88,90903084
10.06.2010	170	49	148	194	81	184	59,87012424
10.06.2010	172	19	106	194	81	184	55,25978986
10.06.2010	173	16	105	194	81	184	158,3600556
10.06.2010	174	125	211	194	81	184	192,988422
10.06.2010	175	52	150	194	81	184	115,2341017
10.06.2010	176	37	136	194	81	184	65,6179485
10.06.2010	177	13	97	194	81	184	99,98112356
10.06.2010	178	26	122	194	81	184	73,99143107
10.06.2010	179	54	153	194	81	184	145,1407886
10.06.2010	180	67	166	194	81	184	39,51213759
10.06.2010	181	60	156	194	81	184	30,47660998
10.06.2010	182	13	96	194	81	184	124,3231393
10.06.2010	191	128	203	194	81	184	115,1996859
10.06.2010	193	171	232	194	81	184	29,9385319
10.06.2010	170	49	148	172	19	106	13,73379857
10.06.2010	170	49	148	173	16	105	116,63021
10.06.2010	172	19	106	173	16	105	105,1547854
10.06.2010	170	49	148	174	125	211	220,7078726
10.06.2010	172	19	106	174	125	211	233,7639329
10.06.2010	173	16	105	174	125	211	335,4348409
10.06.2010	170	49	148	175	52	150	62,48216255

10.06.2010	172	19	106	175	52	150	49,25234454
10.06.2010	173	16	105	175	52	150	60,14865882
10.06.2010	174	125	211	175	52	150	283,0162024
10.06.2010	170	49	148	177	13	97	40,0440558
10.06.2010	172	19	106	177	13	97	26,69648832
10.06.2010	173	16	105	177	13	97	80,30357443
10.06.2010	174	125	211	177	13	97	260,4579089
10.06.2010	175	52	150	177	13	97	22,56225036
10.06.2010	170	49	148	178	26	122	29,60449406
10.06.2010	172	19	106	178	26	122	15,95759987
10.06.2010	173	16	105	178	26	122	94,35341399
10.06.2010	174	125	211	178	26	122	248,1672841
10.06.2010	175	52	150	178	26	122	35,66607357
10.06.2010	177	13	97	178	26	122	14,08767821
10.06.2010	170	49	148	179	54	153	93,42784838
10.06.2010	172	19	106	179	54	153	81,15441926
10.06.2010	173	16	105	179	54	153	26,23041476
10.06.2010	174	125	211	179	54	153	313,7654978
10.06.2010	175	52	150	179	54	153	34,19577328
10.06.2010	177	13	97	179	54	153	55,32366412
10.06.2010	178	26	122	179	54	153	69,18844159
10.06.2010	170	49	148	180	67	166	13,06785944
10.06.2010	172	19	106	180	67	166	26,79847262
10.06.2010	173	16	105	180	67	166	128,1473675
10.06.2010	174	125	211	180	67	166	208,2370472
10.06.2010	175	52	150	180	67	166	75,31227674
10.06.2010	177	13	97	180	67	166	52,98149045
10.06.2010	178	26	122	180	67	166	42,61442829
10.06.2010	179	54	153	180	67	166	105,5837413
10.06.2010	170	49	148	181	60	156	22,11136827
10.06.2010	172	19	106	181	60	156	35,58087894
10.06.2010	173	16	105	181	60	156	133,008124
10.06.2010	174	125	211	181	60	156	202,5071224

10.06.2010	175	52	150	181	60	156	82,64697917
10.06.2010	177	13	97	181	60	156	60,76413535
10.06.2010	178	26	122	181	60	156	51,53828804
10.06.2010	179	54	153	181	60	156	111,4791893
10.06.2010	180	67	166	181	60	156	10,21487879
10.06.2010	170	49	148	182	13	96	73,95082989
10.06.2010	172	19	106	182	13	96	61,49760215
10.06.2010	173	16	105	182	13	96	45,01206583
10.06.2010	174	125	211	182	13	96	294,5256386
10.06.2010	175	52	150	182	13	96	15,51359644
10.06.2010	177	13	97	182	13	96	35,62324817
10.06.2010	178	26	122	182	13	96	49,54499531
10.06.2010	179	54	153	182	13	96	19,71699648
10.06.2010	180	67	166	182	13	96	86,28884454
10.06.2010	181	60	156	182	13	96	92,58843104
10.06.2010	170	49	148	191	128	203	61,64063998
10.06.2010	172	19	106	191	128	203	48,46554008
10.06.2010	173	16	105	191	128	203	60,39540767
10.06.2010	174	125	211	191	128	203	282,2232748
10.06.2010	175	52	150	191	128	203	1,132527613
10.06.2010	177	13	97	191	128	203	21,79869643
10.06.2010	178	26	122	191	128	203	35,06356147
10.06.2010	179	54	153	191	128	203	34,54189609
10.06.2010	180	67	166	191	128	203	74,44264945
10.06.2010	181	60	156	191	128	203	81,71411154
10.06.2010	182	13	96	191	128	203	15,57606073
10.06.2010	170	49	148	193	171	232	41,90891023
10.06.2010	172	19	106	193	171	232	55,32431718
10.06.2010	173	16	105	193	171	232	148,9709067
10.06.2010	174	125	211	193	171	232	186,886559
10.06.2010	175	52	150	193	171	232	101,4114015
10.06.2010	177	13	97	193	171	232	79,96485841
10.06.2010	178	26	122	193	171	232	71,27463561

10.06.2010	179	54	153	193	171	232	128,7504318
10.06.2010	180	67	166	193	171	232	29,56541462
10.06.2010	181	60	156	193	171	232	19,79791567
10.06.2010	182	13	96	193	171	232	110,3952017
10.06.2010	191	128	203	193	171	232	100,4351469
10.06.2010	170	49	148	194	81	184	54,81388168
10.06.2010	172	19	106	194	81	184	68,26047275
10.06.2010	173	16	105	194	81	184	160,377357
10.06.2010	174	125	211	194	81	184	176,4585294
10.06.2010	175	52	150	194	81	184	114,0952747
10.06.2010	177	13	97	194	81	184	92,80484926
10.06.2010	178	26	122	194	81	184	84,2121704
10.06.2010	179	54	153	194	81	184	140,7989231
10.06.2010	180	67	166	194	81	184	42,31611113
10.06.2010	181	60	156	194	81	184	32,71074924
10.06.2010	182	13	96	194	81	184	122,7051124
10.06.2010	191	128	203	194	81	184	113,1051175
10.06.2010	193	171	232	194	81	184	12,93865721
11.06.2010	170	49	148	172	19	106	8,617215456
11.06.2010	170	49	148	173	16	105	122,8858064
11.06.2010	172	19	106	173	16	105	115,4365469
11.06.2010	170	49	148	174	125	211	194,9351057
11.06.2010	172	19	106	174	125	211	202,4338256
11.06.2010	173	16	105	174	125	211	317,8097997
11.06.2010	170	49	148	175	52	150	64,72887955
11.06.2010	172	19	106	175	52	150	56,45576597
11.06.2010	173	16	105	175	52	150	62,0900373
11.06.2010	174	125	211	175	52	150	258,0052074
11.06.2010	170	49	148	176	37	136	26,95441188
11.06.2010	172	19	106	176	37	136	18,33796526
11.06.2010	173	16	105	176	37	136	100,0222209
11.06.2010	174	125	211	176	37	136	218,8057988
11.06.2010	175	52	150	176	37	136	39,21279325

11.06.2010	170	49	148	177	13	97	59,60726198
11.06.2010	172	19	106	177	13	97	51,26441991
11.06.2010	173	16	105	177	13	97	67,47410445
11.06.2010	174	125	211	177	13	97	252,6067629
11.06.2010	175	52	150	177	13	97	5,451331299
11.06.2010	176	37	136	177	13	97	33,84424293
11.06.2010	170	49	148	178	26	122	34,15890565
11.06.2010	172	19	106	178	26	122	25,54197149
11.06.2010	173	16	105	178	26	122	94,71897184
11.06.2010	174	125	211	178	26	122	225,0012645
11.06.2010	175	52	150	178	26	122	33,14117054
11.06.2010	176	37	136	178	26	122	7,231758412
11.06.2010	177	13	97	178	26	122	27,69862429
11.06.2010	170	49	148	179	54	153	108,5570738
11.06.2010	172	19	106	179	54	153	101,044847
11.06.2010	173	16	105	179	54	153	14,45664565
11.06.2010	174	125	211	179	54	153	303,4524285
11.06.2010	175	52	150	179	54	153	47,79235534
11.06.2010	176	37	136	179	54	153	85,56584163
11.06.2010	177	13	97	179	54	153	53,14286407
11.06.2010	178	26	122	179	54	153	80,28754446
11.06.2010	170	49	148	180	67	166	4,153567869
11.06.2010	172	19	106	180	67	166	11,9360816
11.06.2010	173	16	105	180	67	166	124,1134293
11.06.2010	174	125	211	180	67	166	193,8814953
11.06.2010	175	52	150	180	67	166	66,91868177
11.06.2010	176	37	136	180	67	166	30,02288972
11.06.2010	177	13	97	180	67	166	61,90799987
11.06.2010	178	26	122	180	67	166	37,25382841
11.06.2010	179	54	153	180	67	166	109,858344
11.06.2010	170	49	148	181	60	156	13,22519745
11.06.2010	172	19	106	181	60	156	21,58591957
11.06.2010	173	16	105	181	60	156	132,2348989

11.06.2010	174	125	211	181	60	156	186,3703412
11.06.2010	175	52	150	181	60	156	76,14576562
11.06.2010	176	37	136	181	60	156	39,70972659
11.06.2010	177	13	97	181	60	156	71,22709657
11.06.2010	178	26	122	181	60	156	46,94052231
11.06.2010	179	54	153	181	60	156	118,0964914
11.06.2010	180	67	166	181	60	156	9,691710139
11.06.2010	170	49	148	182	13	96	94,27670245
11.06.2010	172	19	106	182	13	96	86,79468286
11.06.2010	173	16	105	182	13	96	28,64242085
11.06.2010	174	125	211	182	13	96	289,1832207
11.06.2010	175	52	150	182	13	96	34,62586461
11.06.2010	176	37	136	182	13	96	71,52979026
11.06.2010	177	13	97	182	13	96	39,80684861
11.06.2010	178	26	122	182	13	96	66,46048371
11.06.2010	179	54	153	182	13	96	14,28537281
11.06.2010	180	67	166	182	13	96	95,57393734
11.06.2010	181	60	156	182	13	96	103,8365001
11.06.2010	170	49	148	191	128	203	62,53649412
11.06.2010	172	19	106	191	128	203	54,20974488
11.06.2010	173	16	105	191	128	203	64,69817258
11.06.2010	174	125	211	191	128	203	255,5683397
11.06.2010	175	52	150	191	128	203	2,613157133
11.06.2010	176	37	136	191	128	203	36,81113499
11.06.2010	177	13	97	191	128	203	2,96913221
11.06.2010	178	26	122	191	128	203	30,6402148
11.06.2010	179	54	153	191	128	203	50,40487132
11.06.2010	180	67	166	191	128	203	64,80641957
11.06.2010	181	60	156	191	128	203	74,09786522
11.06.2010	182	13	96	191	128	203	37,22447931
11.06.2010	170	49	148	193	171	232	19,1834096
11.06.2010	172	19	106	193	171	232	27,62499082
11.06.2010	173	16	105	193	171	232	137,5201754

11.06.2010	174	125	211	193	171	232	181,6445326
11.06.2010	175	52	150	193	171	232	82,02463117
11.06.2010	176	37	136	193	171	232	45,77358441
11.06.2010	177	13	97	193	171	232	77,14956738
11.06.2010	178	26	122	193	171	232	53,00450087
11.06.2010	179	54	153	193	171	232	123,4540705
11.06.2010	180	67	166	193	171	232	15,75365686
11.06.2010	181	60	156	193	171	232	6,063199137
11.06.2010	182	13	96	193	171	232	109,219737
11.06.2010	191	128	203	193	171	232	80,00675419
11.06.2010	170	49	148	194	81	184	44,63705866
11.06.2010	172	19	106	194	81	184	53,07108958
11.06.2010	173	16	105	194	81	184	158,8576142
11.06.2010	174	125	211	194	81	184	165,1604608
11.06.2010	175	52	150	194	81	184	106,2118944
11.06.2010	176	37	136	194	81	184	71,11846174
11.06.2010	177	13	97	194	81	184	101,5575461
11.06.2010	178	26	122	194	81	184	78,34088846
11.06.2010	179	54	153	194	81	184	145,1789634
11.06.2010	180	67	166	194	81	184	41,16016008
11.06.2010	181	60	156	194	81	184	31,48523159
11.06.2010	182	13	96	194	81	184	131,1458064
11.06.2010	191	128	203	194	81	184	104,3396807
11.06.2010	193	171	232	194	81	184	25,45926389
11.06.2010	170	49	148	172	19	106	6,222010088
11.06.2010	170	49	148	173	16	105	101,2057126
11.06.2010	172	19	106	173	16	105	97,19610884
11.06.2010	170	49	148	174	125	211	212,2139703
11.06.2010	172	19	106	174	125	211	216,6529706
11.06.2010	173	16	105	174	125	211	313,29591
11.06.2010	170	49	148	175	52	150	70,10567409
11.06.2010	172	19	106	175	52	150	65,17375203
11.06.2010	173	16	105	175	52	150	36,12392792

11.06.2010	174	125	211	175	52	150	281,658335
11.06.2010	170	49	148	176	37	136	20,72233947
11.06.2010	172	19	106	176	37	136	14,63116138
11.06.2010	173	16	105	176	37	136	85,99571986
11.06.2010	174	125	211	176	37	136	229,4613545
11.06.2010	175	52	150	176	37	136	52,29769682
11.06.2010	170	49	148	177	13	97	47,08039925
11.06.2010	172	19	106	177	13	97	42,13653579
11.06.2010	173	16	105	177	13	97	56,5016653
11.06.2010	174	125	211	177	13	97	258,7029578
11.06.2010	175	52	150	177	13	97	23,04568374
11.06.2010	176	37	136	177	13	97	29,65753509
11.06.2010	170	49	148	178	26	122	34,6779975
11.06.2010	172	19	106	178	26	122	29,08864106
11.06.2010	173	16	105	178	26	122	71,22652591
11.06.2010	174	125	211	178	26	122	244,8369301
11.06.2010	175	52	150	178	26	122	36,96283214
11.06.2010	176	37	136	178	26	122	15,37586923
11.06.2010	177	13	97	178	26	122	14,73880144
11.06.2010	170	49	148	179	54	153	116,7770324
11.06.2010	172	19	106	179	54	153	113,1113464
11.06.2010	173	16	105	179	54	153	17,4407388
11.06.2010	174	125	211	179	54	153	328,3311802
11.06.2010	175	52	150	179	54	153	53,53607247
11.06.2010	176	37	136	179	54	153	102,5352267
11.06.2010	177	13	97	179	54	153	73,30850307
11.06.2010	178	26	122	179	54	153	88,04356464
11.06.2010	170	49	148	180	67	166	9,740980097
11.06.2010	172	19	106	180	67	166	15,94485904
11.06.2010	173	16	105	180	67	166	107,1767415
11.06.2010	174	125	211	180	67	166	206,2089645
11.06.2010	175	52	150	180	67	166	77,63770239
11.06.2010	176	37	136	180	67	166	30,21512215

11.06.2010	177	13	97	180	67	166	54,8199784
11.06.2010	178	26	122	180	67	166	43,41916415
11.06.2010	179	54	153	180	67	166	122,1515925
11.06.2010	170	49	148	181	60	156	33,36959537
11.06.2010	172	19	106	181	60	156	39,57027386
11.06.2010	173	16	105	181	60	156	127,5867333
11.06.2010	174	125	211	181	60	156	187,7846257
11.06.2010	175	52	150	181	60	156	100,258226
11.06.2010	176	37	136	181	60	156	54,05113881
11.06.2010	177	13	97	181	60	156	77,83726333
11.06.2010	178	26	122	181	60	156	67,17088469
11.06.2010	179	54	153	181	60	156	141,5264194
11.06.2010	180	67	166	181	60	156	23,88540251
11.06.2010	170	49	148	182	13	96	88,30667791
11.06.2010	172	19	106	182	13	96	84,22595208
11.06.2010	173	16	105	182	13	96	13,01313994
11.06.2010	174	125	211	182	13	96	300,4659523
11.06.2010	175	52	150	182	13	96	24,06360786
11.06.2010	176	37	136	182	13	96	72,98781869
11.06.2010	177	13	97	182	13	96	43,53166712
11.06.2010	178	26	122	182	13	96	58,26625492
11.06.2010	179	54	153	182	13	96	29,91589566
11.06.2010	180	67	166	182	13	96	94,45177398
11.06.2010	181	60	156	182	13	96	115,2358261
11.06.2010	170	49	148	191	128	203	46,48534714
11.06.2010	172	19	106	191	128	203	41,48160362
11.06.2010	173	16	105	191	128	203	57,35370536
11.06.2010	174	125	211	191	128	203	258,0048048
11.06.2010	175	52	150	191	128	203	23,6932804
11.06.2010	176	37	136	191	128	203	28,87186856
11.06.2010	177	13	97	191	128	203	0,945632141
11.06.2010	178	26	122	191	128	203	13,87382948
11.06.2010	179	54	153	191	128	203	74,18734672

11.06.2010	180	67	166	191	128	203	54,31378369
11.06.2010	181	60	156	191	128	203	77,40974458
11.06.2010	182	13	96	191	128	203	44,39255136
11.06.2010	170	49	148	193	171	232	23,83031657
11.06.2010	172	19	106	193	171	232	30,03296666
11.06.2010	173	16	105	193	171	232	119,6758479
11.06.2010	174	125	211	193	171	232	194,5138727
11.06.2010	175	52	150	193	171	232	91,40142285
11.06.2010	176	37	136	193	171	232	44,51649468
11.06.2010	177	13	97	193	171	232	68,78858754
11.06.2010	178	26	122	193	171	232	57,77587338
11.06.2010	179	54	153	193	171	232	134,0551752
11.06.2010	180	67	166	193	171	232	14,39214726
11.06.2010	181	60	156	193	171	232	9,538586988
11.06.2010	182	13	96	193	171	232	107,1552066
11.06.2010	191	128	203	193	171	232	68,32732999
11.06.2010	170	49	148	194	81	184	46,52060088
11.06.2010	172	19	106	194	81	184	52,63375416
11.06.2010	173	16	105	194	81	184	141,1478303
11.06.2010	174	125	211	194	81	184	175,3653032
11.06.2010	175	52	150	194	81	184	114,0770254
11.06.2010	176	37	136	194	81	184	67,2376835
11.06.2010	177	13	97	194	81	184	91,6307002
11.06.2010	178	26	122	194	81	184	80,74552853
11.06.2010	179	54	153	194	81	184	154,8484826
11.06.2010	180	67	166	194	81	184	37,33254443
11.06.2010	181	60	156	194	81	184	13,81903325
11.06.2010	182	13	96	194	81	184	128,8771401
11.06.2010	191	128	203	194	81	184	91,19425353
11.06.2010	193	171	232	194	81	184	22,97718841
11.06.2010	170	49	148	172	19	106	12,46142847
11.06.2010	170	49	148	173	16	105	108,8282341
11.06.2010	172	19	106	173	16	105	99,33184815

11.06.2010	170	49	148	174	125	211	243,0330166
11.06.2010	172	19	106	174	125	211	251,3594019
11.06.2010	173	16	105	174	125	211	350,4832086
11.06.2010	170	49	148	175	52	150	69,59131979
11.06.2010	172	19	106	175	52	150	59,14167384
11.06.2010	173	16	105	175	52	150	41,10789821
11.06.2010	174	125	211	175	52	150	309,6454834
11.06.2010	170	49	148	176	37	136	24,73222008
11.06.2010	172	19	106	176	37	136	12,38216898
11.06.2010	173	16	105	176	37	136	89,04759718
11.06.2010	174	125	211	176	37	136	261,4561674
11.06.2010	175	52	150	176	37	136	48,24278183
11.06.2010	170	49	148	177	13	97	54,8548979
11.06.2010	172	19	106	177	13	97	43,49596364
11.06.2010	173	16	105	177	13	97	58,46843982
11.06.2010	174	125	211	177	13	97	292,7108935
11.06.2010	175	52	150	177	13	97	17,43711314
11.06.2010	176	37	136	177	13	97	31,83003967
11.06.2010	170	49	148	178	26	122	38,79595415
11.06.2010	172	19	106	178	26	122	26,92507433
11.06.2010	173	16	105	178	26	122	74,87601137
11.06.2010	174	125	211	178	26	122	275,8811677
11.06.2010	175	52	150	178	26	122	33,81460569
11.06.2010	176	37	136	178	26	122	14,92575758
11.06.2010	177	13	97	178	26	122	16,96450313
11.06.2010	170	49	148	179	54	153	109,3109176
11.06.2010	172	19	106	179	54	153	99,80697258
11.06.2010	173	16	105	179	54	153	0,484608806
11.06.2010	174	125	211	179	54	153	350,9512441
11.06.2010	175	52	150	179	54	153	41,55828883
11.06.2010	176	37	136	179	54	153	89,51298123
11.06.2010	177	13	97	179	54	153	58,91213801
11.06.2010	178	26	122	179	54	153	75,33218321

11.06.2010	170	49	148	180	67	166	17,63823455
11.06.2010	172	19	106	180	67	166	5,320520534
11.06.2010	173	16	105	180	67	166	96,66037234
11.06.2010	174	125	211	180	67	166	253,8269646
11.06.2010	175	52	150	180	67	166	56,02808469
11.06.2010	176	37	136	180	67	166	7,996892473
11.06.2010	177	13	97	180	67	166	39,79584201
11.06.2010	178	26	122	180	67	166	22,91932862
11.06.2010	179	54	153	180	67	166	97,12954749
11.06.2010	170	49	148	181	60	156	16,5603616
11.06.2010	172	19	106	181	60	156	28,94919506
11.06.2010	173	16	105	181	60	156	123,4463147
11.06.2010	174	125	211	181	60	156	230,7885719
11.06.2010	175	52	150	181	60	156	85,1778651
11.06.2010	176	37	136	181	60	156	41,2810084
11.06.2010	177	13	97	181	60	156	71,07412256
11.06.2010	178	26	122	181	60	156	55,28185197
11.06.2010	179	54	153	181	60	156	123,933104
11.06.2010	180	67	166	181	60	156	33,98194244
11.06.2010	170	49	148	182	13	96	97,72271592
11.06.2010	172	19	106	182	13	96	87,90654959
11.06.2010	173	16	105	182	13	96	11,9587623
11.06.2010	174	125	211	182	13	96	338,8411611
11.06.2010	175	52	150	182	13	96	29,26818699
11.06.2010	176	37	136	182	13	96	77,38657921
11.06.2010	177	13	97	182	13	96	46,56494573
11.06.2010	178	26	122	182	13	96	63,07866793
11.06.2010	179	54	153	182	13	96	12,38472189
11.06.2010	180	67	166	182	13	96	85,0745164
11.06.2010	181	60	156	182	13	96	112,666116
11.06.2010	170	49	148	191	128	203	63,30080127
11.06.2010	172	19	106	191	128	203	51,93999251
11.06.2010	173	16	105	191	128	203	51,28370962

11.06.2010	174	125	211	191	128	203	300,6763559
11.06.2010	175	52	150	191	128	203	11,45651107
11.06.2010	176	37	136	191	128	203	40,19197861
11.06.2010	177	13	97	191	128	203	8,454786604
11.06.2010	178	26	122	191	128	203	25,27731939
11.06.2010	179	54	153	191	128	203	51,70958153
11.06.2010	180	67	166	191	128	203	48,17514416
11.06.2010	181	60	156	191	128	203	79,48885619
11.06.2010	182	13	96	191	128	203	39,32550103
11.06.2010	170	49	148	193	171	232	14,72929935
11.06.2010	172	19	106	193	171	232	27,18099503
11.06.2010	173	16	105	193	171	232	120,297618
11.06.2010	174	125	211	193	171	232	234,2968985
11.06.2010	175	52	150	193	171	232	82,31779647
11.06.2010	176	37	136	193	171	232	39,36131577
11.06.2010	177	13	97	193	171	232	68,53060781
11.06.2010	178	26	122	193	171	232	53,01710976
11.06.2010	179	54	153	193	171	232	120,7849451
11.06.2010	180	67	166	193	171	232	32,36416068
11.06.2010	181	60	156	193	171	232	3,561132899
11.06.2010	182	13	96	193	171	232	109,5956255
11.06.2010	191	128	203	193	171	232	76,91305828
11.06.2010	170	49	148	194	81	184	63,58447971
11.06.2010	172	19	106	194	81	184	75,85410984
11.06.2010	173	16	105	194	81	184	167,9229355
11.06.2010	174	125	211	194	81	184	196,9907629
11.06.2010	175	52	150	194	81	184	131,3340071
11.06.2010	176	37	136	194	81	184	88,2316705
11.06.2010	177	13	97	194	81	184	117,9502011
11.06.2010	178	26	122	194	81	184	102,3314437
11.06.2010	179	54	153	194	81	184	168,4118983
11.06.2010	180	67	166	194	81	184	80,70323281
11.06.2010	181	60	156	194	81	184	47,05161247

11.06.2010	182	13	96	194	81	184	157,7394935
11.06.2010	191	128	203	194	81	184	126,3193282
11.06.2010	193	171	232	194	81	184	49,42552967
14.06.2010	170	49	148	172	19	106	4,319861133
14.06.2010	170	49	148	173	16	105	133,9898352
14.06.2010	172	19	106	173	16	105	137,9676904
14.06.2010	170	49	148	174	125	211	195,3627125
14.06.2010	172	19	106	174	125	211	191,1149593
14.06.2010	173	16	105	174	125	211	327,3207359
14.06.2010	170	49	148	175	52	150	90,32833358
14.06.2010	172	19	106	175	52	150	94,40681666
14.06.2010	173	16	105	175	52	150	44,21272712
14.06.2010	174	125	211	175	52	150	284,8713228
14.06.2010	170	49	148	176	37	136	24,18547908
14.06.2010	172	19	106	176	37	136	28,50427765
14.06.2010	173	16	105	176	37	136	112,3189629
14.06.2010	174	125	211	176	37	136	219,152388
14.06.2010	175	52	150	176	37	136	68,17043276
14.06.2010	170	49	148	177	13	97	27,97459768
14.06.2010	172	19	106	177	13	97	32,28256342
14.06.2010	173	16	105	177	13	97	107,9041534
14.06.2010	174	125	211	177	13	97	223,2058921
14.06.2010	175	52	150	177	13	97	63,76881367
14.06.2010	176	37	136	177	13	97	4,432352154
14.06.2010	170	49	148	178	26	122	17,26427864
14.06.2010	172	19	106	178	26	122	21,58299025
14.06.2010	173	16	105	178	26	122	118,2511179
14.06.2010	174	125	211	178	26	122	212,3980193
14.06.2010	175	52	150	178	26	122	74,2270884
14.06.2010	176	37	136	178	26	122	6,9359381
14.06.2010	177	13	97	178	26	122	10,80947574
14.06.2010	170	49	148	179	54	153	99,04864331
14.06.2010	172	19	106	179	54	153	103,1339573

14.06.2010	173	16	105	179	54	153	35,83005754
14.06.2010	174	125	211	179	54	153	293,597818
14.06.2010	175	52	150	179	54	153	8,736409493
14.06.2010	176	37	136	179	54	153	76,77170903
14.06.2010	177	13	97	179	54	153	72,38200427
14.06.2010	178	26	122	179	54	153	82,88750535
14.06.2010	170	49	148	180	67	166	20,11699101
14.06.2010	172	19	106	180	67	166	16,33133336
14.06.2010	173	16	105	180	67	166	147,9779791
14.06.2010	174	125	211	180	67	166	179,4772572
14.06.2010	175	52	150	180	67	166	105,4446762
14.06.2010	176	37	136	180	67	166	43,26108497
14.06.2010	177	13	97	180	67	166	46,49020462
14.06.2010	178	26	122	180	67	166	36,40947339
14.06.2010	179	54	153	180	67	166	114,1596585
14.06.2010	170	49	148	181	60	156	9,979611198
14.06.2010	172	19	106	181	60	156	6,170259325
14.06.2010	173	16	105	181	60	156	141,2394711
14.06.2010	174	125	211	181	60	156	186,9641876
14.06.2010	175	52	150	181	60	156	98,05420905
14.06.2010	176	37	136	181	60	156	33,69700235
14.06.2010	177	13	97	181	60	156	37,2043339
14.06.2010	178	26	122	181	60	156	26,7725069
14.06.2010	179	54	153	181	60	156	106,7904785
14.06.2010	180	67	166	181	60	156	10,1874301
14.06.2010	170	49	148	182	13	96	63,38764122
14.06.2010	172	19	106	182	13	96	67,64403487
14.06.2010	173	16	105	182	13	96	73,70332176
14.06.2010	174	125	211	182	13	96	258,7498963
14.06.2010	175	52	150	182	13	96	29,74375279
14.06.2010	176	37	136	182	13	96	39,8864141
14.06.2010	177	13	97	182	13	96	35,66138809
14.06.2010	178	26	122	182	13	96	46,44919159

14.06.2010	179	54	153	182	13	96	37,87468415
14.06.2010	180	67	166	182	13	96	80,57083153
14.06.2010	181	60	156	182	13	96	72,07123479
14.06.2010	170	49	148	191	128	203	31,7324137
14.06.2010	172	19	106	191	128	203	36,02414808
14.06.2010	173	16	105	191	128	203	103,9344025
14.06.2010	174	125	211	191	128	203	227,046138
14.06.2010	175	52	150	191	128	203	59,79366042
14.06.2010	176	37	136	191	128	203	8,386134574
14.06.2010	177	13	97	191	128	203	3,975791967
14.06.2010	178	26	122	191	128	203	14,69584002
14.06.2010	179	54	153	191	128	203	68,40541258
14.06.2010	180	67	166	191	128	203	49,87596671
14.06.2010	181	60	156	191	128	203	40,78406302
14.06.2010	182	13	96	191	128	203	31,75489283
14.06.2010	170	49	148	193	171	232	32,35857839
14.06.2010	172	19	106	193	171	232	28,31813474
14.06.2010	173	16	105	193	171	232	159,3250204
14.06.2010	174	125	211	193	171	232	167,9977135
14.06.2010	175	52	150	193	171	232	117,2672437
14.06.2010	176	37	136	193	171	232	55,81398611
14.06.2010	177	13	97	193	171	232	59,0546318
14.06.2010	178	26	122	193	171	232	48,94650438
14.06.2010	179	54	153	193	171	232	125,9535127
14.06.2010	180	67	166	193	171	232	12,56537175
14.06.2010	181	60	156	193	171	232	22,39004837
14.06.2010	182	13	96	193	171	232	92,94460463
14.06.2010	191	128	203	193	171	232	62,423877
14.06.2010	170	49	148	194	81	184	52,82056435
14.06.2010	172	19	106	194	81	184	48,92075445
14.06.2010	173	16	105	194	81	184	173,9682272
14.06.2010	174	125	211	194	81	184	154,9251694
14.06.2010	175	52	150	194	81	184	133,4232187

14.06.2010	176	37	136	194	81	184	75,58594936
14.06.2010	177	13	97	194	81	184	78,49535045
14.06.2010	178	26	122	194	81	184	68,85224272
14.06.2010	179	54	153	194	81	184	141,9693861
14.06.2010	180	67	166	194	81	184	32,71074924
14.06.2010	181	60	156	194	81	184	42,85601305
14.06.2010	182	13	96	194	81	184	111,0117522
14.06.2010	191	128	203	194	81	184	81,6110803
14.06.2010	193	171	232	194	81	184	20,79478744
14.06.2010	170	49	148	172	19	106	6,628969944
14.06.2010	170	49	148	173	16	105	119,7645359
14.06.2010	172	19	106	173	16	105	115,16675
14.06.2010	170	49	148	174	125	211	224,9626223
14.06.2010	172	19	106	174	125	211	229,01461
14.06.2010	173	16	105	174	125	211	343,9556219
14.06.2010	170	49	148	175	52	150	62,1885909
14.06.2010	172	19	106	175	52	150	56,56908726
14.06.2010	173	16	105	175	52	150	61,58654474
14.06.2010	174	125	211	175	52	150	283,3833723
14.06.2010	170	49	148	177	13	97	45,91455734
14.06.2010	172	19	106	177	13	97	39,86228922
14.06.2010	173	16	105	177	13	97	79,3606889
14.06.2010	174	125	211	177	13	97	265,4966736
14.06.2010	175	52	150	177	13	97	17,91767556
14.06.2010	170	49	148	178	26	122	19,87668783
14.06.2010	172	19	106	178	26	122	13,58168844
14.06.2010	173	16	105	178	26	122	103,0212672
14.06.2010	174	125	211	178	26	122	240,9359175
14.06.2010	175	52	150	178	26	122	43,34764665
14.06.2010	177	13	97	178	26	122	26,32442688
14.06.2010	170	49	148	179	54	153	106,0705873
14.06.2010	172	19	106	179	54	153	101,7143596
14.06.2010	173	16	105	179	54	153	14,63239603

14.06.2010	174	125	211	179	54	153	330,7094527
14.06.2010	175	52	150	179	54	153	50,1387745
14.06.2010	177	13	97	179	54	153	67,43232515
14.06.2010	178	26	122	179	54	153	89,95899099
14.06.2010	170	49	148	180	67	166	21,64421398
14.06.2010	172	19	106	180	67	166	28,21347994
14.06.2010	173	16	105	180	67	166	137,8395419
14.06.2010	174	125	211	180	67	166	209,981853
14.06.2010	175	52	150	180	67	166	82,70062338
14.06.2010	177	13	97	180	67	166	67,14943317
14.06.2010	178	26	122	180	67	166	41,46102135
14.06.2010	179	54	153	180	67	166	123,6957542
14.06.2010	170	49	148	181	60	156	13,45842704
14.06.2010	172	19	106	181	60	156	6,881025003
14.06.2010	173	16	105	181	60	156	109,7662604
14.06.2010	174	125	211	181	60	156	234,2033536
14.06.2010	175	52	150	181	60	156	50,34641839
14.06.2010	177	13	97	181	60	156	33,29873696
14.06.2010	178	26	122	181	60	156	7,007197392
14.06.2010	179	54	153	181	60	156	96,56650696
14.06.2010	180	67	166	181	60	156	35,08545101
14.06.2010	170	49	148	182	13	96	80,54807066
14.06.2010	172	19	106	182	13	96	75,41019942
14.06.2010	173	16	105	182	13	96	41,13952677
14.06.2010	174	125	211	182	13	96	303,3987261
14.06.2010	175	52	150	182	13	96	20,45008527
14.06.2010	177	13	97	182	13	96	38,24963378
14.06.2010	178	26	122	182	13	96	62,68119611
14.06.2010	179	54	153	182	13	96	30,07849634
14.06.2010	180	67	166	182	13	96	100,0499859
14.06.2010	181	60	156	182	13	96	69,5832721
14.06.2010	170	49	148	191	128	203	55,8674462
14.06.2010	172	19	106	191	128	203	50,36982564

14.06.2010	173	16	105	191	128	203	66,49139564
14.06.2010	174	125	211	191	128	203	277,8521854
14.06.2010	175	52	150	191	128	203	6,543254288
14.06.2010	177	13	97	191	128	203	13,12303907
14.06.2010	178	26	122	191	128	203	37,32068711
14.06.2010	179	54	153	191	128	203	54,34287886
14.06.2010	180	67	166	191	128	203	76,2382935
14.06.2010	181	60	156	191	128	203	44,28702878
14.06.2010	182	13	96	191	128	203	25,55222484
14.06.2010	170	49	148	193	171	232	35,92849092
14.06.2010	172	19	106	193	171	232	42,23545552
14.06.2010	173	16	105	193	171	232	152,9909487
14.06.2010	174	125	211	193	171	232	196,0029611
14.06.2010	175	52	150	193	171	232	97,70672419
14.06.2010	177	13	97	193	171	232	81,81822742
14.06.2010	178	26	122	193	171	232	55,76557714
14.06.2010	179	54	153	193	171	232	138,8095663
14.06.2010	180	67	166	193	171	232	15,1998016
14.06.2010	181	60	156	193	171	232	49,07891637
14.06.2010	182	13	96	193	171	232	115,2298297
14.06.2010	191	128	203	193	171	232	91,27414955
14.06.2010	170	49	148	194	81	184	43,17769698
14.06.2010	172	19	106	194	81	184	49,73705082
14.06.2010	173	16	105	194	81	184	156,7688314
14.06.2010	174	125	211	194	81	184	196,2326225
14.06.2010	175	52	150	194	81	184	103,5888981
14.06.2010	177	13	97	194	81	184	88,485293
14.06.2010	178	26	122	194	81	184	62,97906903
14.06.2010	179	54	153	194	81	184	142,3835725
14.06.2010	180	67	166	194	81	184	21,53355038
14.06.2010	181	60	156	194	81	184	56,61313972
14.06.2010	182	13	96	194	81	184	120,2155974
14.06.2010	191	128	203	194	81	184	97,07689825

14.06.2010	193	171	232	194	81	184	10,24754389
14.06.2010	170	49	148	172	19	106	26,22059885
14.06.2010	170	49	148	173	16	105	117,6002897
14.06.2010	172	19	106	173	16	105	95,32922569
14.06.2010	170	49	148	174	125	211	207,7816199
14.06.2010	172	19	106	174	125	211	229,7053605
14.06.2010	173	16	105	174	125	211	324,8384222
14.06.2010	170	49	148	175	52	150	70,18209992
14.06.2010	172	19	106	175	52	150	46,70826812
14.06.2010	173	16	105	175	52	150	48,73706616
14.06.2010	174	125	211	175	52	150	276,4006981
14.06.2010	170	49	148	176	37	136	35,37184667
14.06.2010	172	19	106	176	37	136	9,395952119
14.06.2010	173	16	105	176	37	136	86,5932341
14.06.2010	174	125	211	176	37	136	238,744163
14.06.2010	175	52	150	176	37	136	37,86204236
14.06.2010	170	49	148	177	13	97	52,52948305
14.06.2010	172	19	106	177	13	97	26,90040606
14.06.2010	173	16	105	177	13	97	70,37565715
14.06.2010	174	125	211	177	13	97	255,8575991
14.06.2010	175	52	150	177	13	97	22,07314033
14.06.2010	176	37	136	177	13	97	17,51154712
14.06.2010	170	49	148	178	26	122	38,85771906
14.06.2010	172	19	106	178	26	122	13,12613604
14.06.2010	173	16	105	178	26	122	82,83621754
14.06.2010	174	125	211	178	26	122	242,5489139
14.06.2010	175	52	150	178	26	122	34,09988751
14.06.2010	176	37	136	178	26	122	3,806333257
14.06.2010	177	13	97	178	26	122	13,79122602
14.06.2010	170	49	148	179	54	153	83,73782155
14.06.2010	172	19	106	179	54	153	60,14407846
14.06.2010	173	16	105	179	54	153	35,782885
14.06.2010	174	125	211	179	54	153	289,8471312

14.06.2010	175	52	150	179	54	153	13,56771516
14.06.2010	176	37	136	179	54	153	51,15709299
14.06.2010	177	13	97	179	54	153	34,6106009
14.06.2010	178	26	122	179	54	153	47,3647305
14.06.2010	170	49	148	180	67	166	19,05798867
14.06.2010	172	19	106	180	67	166	7,167775946
14.06.2010	173	16	105	180	67	166	101,3645853
14.06.2010	174	125	211	180	67	166	223,4894884
14.06.2010	175	52	150	180	67	166	52,98677521
14.06.2010	176	37	136	180	67	166	16,45558017
14.06.2010	177	13	97	180	67	166	33,87238761
14.06.2010	178	26	122	180	67	166	20,08621097
14.06.2010	179	54	153	180	67	166	66,51170137
14.06.2010	170	49	148	181	60	156	7,95442328
14.06.2010	172	19	106	181	60	156	18,27357328
14.06.2010	173	16	105	181	60	156	110,8122682
14.06.2010	174	125	211	181	60	156	214,1642399
14.06.2010	175	52	150	181	60	156	62,95432403
14.06.2010	176	37	136	181	60	156	27,47548823
14.06.2010	177	13	97	181	60	156	44,73953495
14.06.2010	178	26	122	181	60	156	31,01205114
14.06.2010	179	54	153	181	60	156	76,52193428
14.06.2010	180	67	166	181	60	156	11,10703606
14.06.2010	170	49	148	182	13	96	84,99825076
14.06.2010	172	19	106	182	13	96	60,94929285
14.06.2010	173	16	105	182	13	96	35,91290055
14.06.2010	174	125	211	182	13	96	290,5680493
14.06.2010	175	52	150	182	13	96	14,95960836
14.06.2010	176	37	136	182	13	96	51,82561222
14.06.2010	177	13	97	182	13	96	34,90244618
14.06.2010	178	26	122	182	13	96	48,02011884
14.06.2010	179	54	153	182	13	96	3,146370812
14.06.2010	180	67	166	182	13	96	67,4659381

14.06.2010	181	60	156	182	13	96	77,66631725
14.06.2010	170	49	148	191	128	203	64,6133736
14.06.2010	172	19	106	191	128	203	39,58008805
14.06.2010	173	16	105	191	128	203	57,59651053
14.06.2010	174	125	211	191	128	203	268,8549009
14.06.2010	175	52	150	191	128	203	10,69522877
14.06.2010	176	37	136	191	128	203	30,27574627
14.06.2010	177	13	97	191	128	203	13,01452808
14.06.2010	178	26	122	191	128	203	26,48247544
14.06.2010	179	54	153	191	128	203	21,82644095
14.06.2010	180	67	166	191	128	203	46,37105002
14.06.2010	181	60	156	191	128	203	56,98842966
14.06.2010	182	13	96	191	128	203	21,92388828
14.06.2010	170	49	148	193	171	232	20,14817234
14.06.2010	172	19	106	193	171	232	46,30877342
14.06.2010	173	16	105	193	171	232	134,7425
14.06.2010	174	125	211	193	171	232	193,0503564
14.06.2010	175	52	150	193	171	232	88,59669359
14.06.2010	176	37	136	193	171	232	55,32872516
14.06.2010	177	13	97	193	171	232	72,20871355
14.06.2010	178	26	122	193	171	232	58,70263428
14.06.2010	179	54	153	193	171	232	102,0329737
14.06.2010	180	67	166	193	171	232	39,16692749
14.06.2010	181	60	156	193	171	232	28,08995202
14.06.2010	182	13	96	193	171	232	103,5410165
14.06.2010	191	128	203	193	171	232	83,89289821
14.06.2010	170	49	148	194	81	184	36,55900771
14.06.2010	172	19	106	194	81	184	62,2650875
14.06.2010	173	16	105	194	81	184	146,4090607
14.06.2010	174	125	211	194	81	184	186,4436793
14.06.2010	175	52	150	194	81	184	102,025493
14.06.2010	176	37	136	194	81	184	70,95570683
14.06.2010	177	13	97	194	81	184	87,27399626

14.06.2010	178	26	122	194	81	184	74,12394723
14.06.2010	179	54	153	194	81	184	115,1947461
14.06.2010	180	67	166	194	81	184	55,23657428
14.06.2010	181	60	156	194	81	184	44,35082051
14.06.2010	182	13	96	194	81	184	116,9615947
14.06.2010	191	128	203	194	81	184	98,34041552
14.06.2010	193	171	232	194	81	184	16,95385105
14.06.2010	170	49	148	172	19	106	19,69247242
14.06.2010	170	49	148	173	16	105	119,9835034
14.06.2010	172	19	106	173	16	105	105,0349331
14.06.2010	170	49	148	174	125	211	219,5242918
14.06.2010	172	19	106	174	125	211	235,4022649
14.06.2010	173	16	105	174	125	211	339,5066687
14.06.2010	170	49	148	175	52	150	60,60174232
14.06.2010	172	19	106	175	52	150	42,94465324
14.06.2010	173	16	105	175	52	150	63,76045608
14.06.2010	174	125	211	175	52	150	278,2896964
14.06.2010	170	49	148	176	37	136	30,4699408
14.06.2010	172	19	106	176	37	136	10,83117986
14.06.2010	173	16	105	176	37	136	98,59790023
14.06.2010	174	125	211	176	37	136	243,5657254
14.06.2010	175	52	150	176	37	136	35,15450667
14.06.2010	170	49	148	177	13	97	51,28370962
14.06.2010	172	19	106	177	13	97	32,95778412
14.06.2010	173	16	105	177	13	97	74,13198936
14.06.2010	174	125	211	177	13	97	268,094836
14.06.2010	175	52	150	177	13	97	10,42320639
14.06.2010	176	37	136	177	13	97	24,7570421
14.06.2010	170	49	148	178	26	122	34,97354199
14.06.2010	172	19	106	178	26	122	15,35823567
14.06.2010	173	16	105	178	26	122	93,1072904
14.06.2010	174	125	211	178	26	122	249,1665517
14.06.2010	175	52	150	178	26	122	29,57091328

14.06.2010	176	37	136	178	26	122	5,614580863
14.06.2010	177	13	97	178	26	122	19,16126683
14.06.2010	170	49	148	179	54	153	104,7883096
14.06.2010	172	19	106	179	54	153	90,52561145
14.06.2010	173	16	105	179	54	153	15,84227849
14.06.2010	174	125	211	179	54	153	324,2700943
14.06.2010	175	52	150	179	54	153	50,69567957
14.06.2010	176	37	136	179	54	153	84,75550864
14.06.2010	177	13	97	179	54	153	60,81517484
14.06.2010	178	26	122	179	54	153	79,38964982
14.06.2010	170	49	148	180	67	166	12,67701524
14.06.2010	172	19	106	180	67	166	7,022648782
14.06.2010	173	16	105	180	67	166	110,3628781
14.06.2010	174	125	211	180	67	166	229,5245251
14.06.2010	175	52	150	180	67	166	49,17930003
14.06.2010	176	37	136	180	67	166	17,79881484
14.06.2010	177	13	97	180	67	166	39,43697056
14.06.2010	178	26	122	180	67	166	22,3583577
14.06.2010	179	54	153	180	67	166	95,56902278
14.06.2010	170	49	148	181	60	156	15,47716411
14.06.2010	172	19	106	181	60	156	4,217231926
14.06.2010	173	16	105	181	60	156	108,1802067
14.06.2010	174	125	211	181	60	156	231,8912636
14.06.2010	175	52	150	181	60	156	46,6399544
14.06.2010	176	37	136	181	60	156	15,01505468
14.06.2010	177	13	97	181	60	156	36,80671798
14.06.2010	178	26	122	181	60	156	19,5562321
14.06.2010	179	54	153	181	60	156	93,49047549
14.06.2010	180	67	166	181	60	156	2,80487625
14.06.2010	170	49	148	182	13	96	79,24103485
14.06.2010	172	19	106	182	13	96	63,41720238
14.06.2010	173	16	105	182	13	96	41,76490592
14.06.2010	174	125	211	182	13	96	298,4766001

14.06.2010	175	52	150	182	13	96	22,37753894
14.06.2010	176	37	136	182	13	96	56,84798626
14.06.2010	177	13	97	182	13	96	32,56656603
14.06.2010	178	26	122	182	13	96	51,38259853
14.06.2010	179	54	153	182	13	96	28,3192511
14.06.2010	180	67	166	182	13	96	68,98631659
14.06.2010	181	60	156	182	13	96	66,69972726
14.06.2010	170	49	148	191	128	203	77,76272756
14.06.2010	172	19	106	191	128	203	61,71583997
14.06.2010	173	16	105	191	128	203	43,60909698
14.06.2010	174	125	211	191	128	203	296,8844715
14.06.2010	175	52	150	191	128	203	20,37220013
14.06.2010	176	37	136	191	128	203	54,98918021
14.06.2010	177	13	97	191	128	203	30,61455705
14.06.2010	178	26	122	191	128	203	49,50204279
14.06.2010	179	54	153	191	128	203	30,34547799
14.06.2010	180	67	166	191	128	203	67,36706015
14.06.2010	181	60	156	191	128	203	65,04931668
14.06.2010	182	13	96	191	128	203	2,090871334
14.06.2010	170	49	148	193	171	232	10,0067273
14.06.2010	172	19	106	193	171	232	29,68599788
14.06.2010	173	16	105	193	171	232	127,6961004
14.06.2010	174	125	211	193	171	232	212,1196929
14.06.2010	175	52	150	193	171	232	69,77162947
14.06.2010	176	37	136	193	171	232	40,47526945
14.06.2010	177	13	97	193	171	232	60,75149888
14.06.2010	178	26	122	193	171	232	44,9224783
14.06.2010	179	54	153	193	171	232	112,2731954
14.06.2010	180	67	166	193	171	232	22,6784495
14.06.2010	181	60	156	193	171	232	25,47398315
14.06.2010	182	13	96	193	171	232	87,56413131
14.06.2010	191	128	203	193	171	232	86,18703765
14.06.2010	170	49	148	194	81	184	22,42894432

14.06.2010	172	19	106	194	81	184	42,11702421
14.06.2010	173	16	105	194	81	184	138,102653
14.06.2010	174	125	211	194	81	184	202,7116599
14.06.2010	175	52	150	194	81	184	81,66070416
14.06.2010	176	37	136	194	81	184	52,8977163
14.06.2010	177	13	97	194	81	184	72,88379129
14.06.2010	178	26	122	194	81	184	57,34913802
14.06.2010	179	54	153	194	81	184	122,5024025
14.06.2010	180	67	166	194	81	184	35,10578316
14.06.2010	181	60	156	194	81	184	37,9035298
14.06.2010	182	13	96	194	81	184	98,67784312
14.06.2010	191	128	203	194	81	184	97,39658647
14.06.2010	193	171	232	194	81	184	12,43240111
14.06.2010	170	49	148	172	19	106	15,33351462
14.06.2010	170	49	148	173	16	105	117,5199221
14.06.2010	172	19	106	173	16	105	106,4565775
14.06.2010	170	49	148	174	125	211	238,4801473
14.06.2010	172	19	106	174	125	211	247,3124677
14.06.2010	173	16	105	174	125	211	353,251822
14.06.2010	170	49	148	175	52	150	70,84984314
14.06.2010	172	19	106	175	52	150	57,17783647
14.06.2010	173	16	105	175	52	150	54,06859907
14.06.2010	174	125	211	175	52	150	300,7271204
14.06.2010	170	49	148	176	37	136	28,82819313
14.06.2010	172	19	106	176	37	136	13,51769269
14.06.2010	173	16	105	176	37	136	98,54424814
14.06.2010	174	125	211	176	37	136	254,748944
14.06.2010	175	52	150	176	37	136	46,71445592
14.06.2010	170	49	148	177	13	97	56,80626256
14.06.2010	172	19	106	177	13	97	41,8898317
14.06.2010	173	16	105	177	13	97	73,75642914
14.06.2010	174	125	211	177	13	97	281,6156639
14.06.2010	175	52	150	177	13	97	19,69224308

14.06.2010	176	37	136	177	13	97	29,57457849
14.06.2010	170	49	148	178	26	122	46,80312224
14.06.2010	172	19	106	178	26	122	31,96892763
14.06.2010	173	16	105	178	26	122	79,93853528
14.06.2010	174	125	211	178	26	122	273,9529958
14.06.2010	175	52	150	178	26	122	26,84746916
14.06.2010	176	37	136	178	26	122	20,13673732
14.06.2010	177	13	97	178	26	122	10,01665148
14.06.2010	170	49	148	179	54	153	112,0610057
14.06.2010	172	19	106	179	54	153	101,0051495
14.06.2010	173	16	105	179	54	153	5,461263887
14.06.2010	174	125	211	179	54	153	347,8467501
14.06.2010	175	52	150	179	54	153	49,00330936
14.06.2010	176	37	136	179	54	153	93,16358886
14.06.2010	177	13	97	179	54	153	68,66649359
14.06.2010	178	26	122	179	54	153	74,66557276
14.06.2010	170	49	148	180	67	166	25,72327308
14.06.2010	172	19	106	180	67	166	10,69142767
14.06.2010	173	16	105	180	67	166	102,2837241
14.06.2010	174	125	211	180	67	166	250,9783489
14.06.2010	175	52	150	180	67	166	50,64398347
14.06.2010	176	37	136	180	67	166	3,950722236
14.06.2010	177	13	97	180	67	166	33,48362805
14.06.2010	178	26	122	180	67	166	24,08686897
14.06.2010	179	54	153	180	67	166	96,89109553
14.06.2010	170	49	148	181	60	156	18,2537908
14.06.2010	172	19	106	181	60	156	3,079635215
14.06.2010	173	16	105	181	60	156	103,7056748
14.06.2010	174	125	211	181	60	156	249,8981097
14.06.2010	175	52	150	181	60	156	54,14697543
14.06.2010	176	37	136	181	60	156	10,79986192
14.06.2010	177	13	97	181	60	156	38,82097438
14.06.2010	178	26	122	181	60	156	28,89235288

14.06.2010	179	54	153	181	60	156	98,25937079
14.06.2010	180	67	166	181	60	156	8,479322658
14.06.2010	170	49	148	182	13	96	105,2144672
14.06.2010	172	19	106	182	13	96	93,75916052
14.06.2010	173	16	105	182	13	96	13,13060814
14.06.2010	174	125	211	182	13	96	340,2930357
14.06.2010	175	52	150	182	13	96	40,97077943
14.06.2010	176	37	136	182	13	96	85,55000582
14.06.2010	177	13	97	182	13	96	60,64986549
14.06.2010	178	26	122	182	13	96	66,82548229
14.06.2010	179	54	153	182	13	96	8,08172016
14.06.2010	180	67	166	182	13	96	89,31543874
14.06.2010	181	60	156	182	13	96	90,96397542
14.06.2010	170	49	148	191	128	203	96,49417589
14.06.2010	172	19	106	191	128	203	83,82126884
14.06.2010	173	16	105	191	128	203	27,51490978
14.06.2010	174	125	211	191	128	203	328,6215342
14.06.2010	175	52	150	191	128	203	27,91447681
14.06.2010	176	37	136	191	128	203	74,26893719
14.06.2010	177	13	97	191	128	203	47,38532179
14.06.2010	178	26	122	191	128	203	54,67883849
14.06.2010	179	54	153	191	128	203	23,16862098
14.06.2010	180	67	166	191	128	203	78,15665665
14.06.2010	181	60	156	191	128	203	80,87859311
14.06.2010	182	13	96	191	128	203	15,36617328
14.06.2010	170	49	148	193	171	232	12,50556547
14.06.2010	172	19	106	193	171	232	26,90577795
14.06.2010	173	16	105	193	171	232	129,8079595
14.06.2010	174	125	211	193	171	232	227,3475908
14.06.2010	175	52	150	193	171	232	83,29808072
14.06.2010	176	37	136	193	171	232	39,9824292
14.06.2010	177	13	97	193	171	232	68,783335
14.06.2010	178	26	122	193	171	232	58,83158889

14.06.2010	179	54	153	193	171	232	124,3537227
14.06.2010	180	67	166	193	171	232	36,47763208
14.06.2010	181	60	156	193	171	232	29,96567281
14.06.2010	182	13	96	193	171	232	117,5855802
14.06.2010	191	128	203	193	171	232	108,994891
14.06.2010	170	49	148	194	81	184	109,3871599
14.06.2010	172	19	106	194	81	184	121,3163727
14.06.2010	173	16	105	194	81	184	226,9066764
14.06.2010	174	125	211	194	81	184	135,7512944
14.06.2010	175	52	150	194	81	184	178,2535651
14.06.2010	176	37	136	194	81	184	131,7958461
14.06.2010	177	13	97	194	81	184	161,1352168
14.06.2010	178	26	122	194	81	184	151,9325152
14.06.2010	179	54	153	194	81	184	221,4474012
14.06.2010	180	67	166	194	81	184	127,8456871
14.06.2010	181	60	156	194	81	184	124,2749969
14.06.2010	182	13	96	194	81	184	214,5474008
14.06.2010	191	128	203	194	81	184	205,1356311
14.06.2010	193	171	232	194	81	184	97,16311275
14.06.2010	170	49	148	172	19	106	27,31921702
14.06.2010	170	49	148	173	16	105	125,937161
14.06.2010	172	19	106	173	16	105	107,5026085
14.06.2010	170	49	148	174	125	211	5,703960138
14.06.2010	172	19	106	174	125	211	33,0226733
14.06.2010	173	16	105	174	125	211	130,1500002
14.06.2010	170	49	148	175	52	150	78,82674537
14.06.2010	172	19	106	175	52	150	55,70050696
14.06.2010	173	16	105	175	52	150	55,00067721
14.06.2010	174	125	211	175	52	150	83,95392341
14.06.2010	170	49	148	176	37	136	39,73030673
14.06.2010	172	19	106	176	37	136	12,82436145
14.06.2010	173	16	105	176	37	136	97,28579229
14.06.2010	174	125	211	176	37	136	45,40586185

14.06.2010	175	52	150	176	37	136	44,00467185
14.06.2010	170	49	148	177	13	97	70,87406178
14.06.2010	172	19	106	177	13	97	48,42293579
14.06.2010	173	16	105	177	13	97	60,30972579
14.06.2010	174	125	211	177	13	97	75,94449544
14.06.2010	175	52	150	177	13	97	8,164560444
14.06.2010	176	37	136	177	13	97	37,25091879
14.06.2010	170	49	148	178	26	122	47,05103655
14.06.2010	172	19	106	178	26	122	21,53606701
14.06.2010	173	16	105	178	26	122	87,78084605
14.06.2010	174	125	211	178	26	122	52,5970166
14.06.2010	175	52	150	178	26	122	34,46728949
14.06.2010	176	37	136	178	26	122	9,583461366
14.06.2010	177	13	97	178	26	122	27,67154474
14.06.2010	170	49	148	179	54	153	119,7990728
14.06.2010	172	19	106	179	54	153	99,11933813
14.06.2010	173	16	105	179	54	153	15,14622441
14.06.2010	174	125	211	179	54	153	124,4135601
14.06.2010	175	52	150	179	54	153	44,28172566
14.06.2010	176	37	136	179	54	153	88,0096516
14.06.2010	177	13	97	179	54	153	50,7958911
14.06.2010	178	26	122	179	54	153	78,42621996
14.06.2010	170	49	148	180	67	166	14,41127638
14.06.2010	172	19	106	180	67	166	12,90790409
14.06.2010	173	16	105	180	67	166	115,7268682
14.06.2010	174	125	211	180	67	166	20,11452136
14.06.2010	175	52	150	180	67	166	66,19540972
14.06.2010	176	37	136	180	67	166	25,41772033
14.06.2010	177	13	97	180	67	166	58,47956172
14.06.2010	178	26	122	180	67	166	33,21358892
14.06.2010	179	54	153	180	67	166	108,4814554
14.06.2010	170	49	148	181	60	156	9,986397127
14.06.2010	172	19	106	181	60	156	17,40289117

14.06.2010	173	16	105	181	60	156	118,1581217
14.06.2010	174	125	211	181	60	156	15,67087543
14.06.2010	175	52	150	181	60	156	69,59644646
14.06.2010	176	37	136	181	60	156	29,74436014
14.06.2010	177	13	97	181	60	156	61,76046263
14.06.2010	178	26	122	181	60	156	37,201299
14.06.2010	179	54	153	181	60	156	111,3506917
14.06.2010	180	67	166	181	60	156	4,553974237
14.06.2010	170	49	148	182	13	96	108,9060169
14.06.2010	172	19	106	182	13	96	87,81361314
14.06.2010	173	16	105	182	13	96	23,8111676
14.06.2010	174	125	211	182	13	96	113,6194417
14.06.2010	175	52	150	182	13	96	32,88754902
14.06.2010	176	37	136	182	13	96	76,62791839
14.06.2010	177	13	97	182	13	96	39,44006244
14.06.2010	178	26	122	182	13	96	67,04544072
14.06.2010	179	54	153	182	13	96	11,39960359
14.06.2010	180	67	166	182	13	96	97,35229321
14.06.2010	181	60	156	182	13	96	100,3106912
14.06.2010	170	49	148	191	128	203	104,8026175
14.06.2010	172	19	106	191	128	203	83,66012144
14.06.2010	173	16	105	191	128	203	27,13709461
14.06.2010	174	125	211	191	128	203	109,5368878
14.06.2010	175	52	150	191	128	203	28,82380628
14.06.2010	176	37	136	191	128	203	72,49528704
14.06.2010	177	13	97	191	128	203	35,29400414
14.06.2010	178	26	122	191	128	203	62,91198394
14.06.2010	179	54	153	191	128	203	15,51563413
14.06.2010	180	67	166	191	128	203	93,20851593
14.06.2010	181	60	156	191	128	203	96,18017528
14.06.2010	182	13	96	191	128	203	4,154655048
14.06.2010	170	49	148	193	171	232	20,51094744
14.06.2010	172	19	106	193	171	232	47,45855807

14.06.2010	173	16	105	193	171	232	138,1191666
14.06.2010	174	125	211	193	171	232	15,03969861
14.06.2010	175	52	150	193	171	232	95,28999075
14.06.2010	176	37	136	193	171	232	59,41488219
14.06.2010	177	13	97	193	171	232	87,15074876
14.06.2010	178	26	122	193	171	232	65,83665922
14.06.2010	179	54	153	193	171	232	133,7179563
14.06.2010	180	67	166	193	171	232	34,64372896
14.06.2010	181	60	156	193	171	232	30,09560848
14.06.2010	182	13	96	193	171	232	123,317359
14.06.2010	191	128	203	193	171	232	119,3303155
14.06.2010	170	49	148	194	81	184	102,9180643
14.06.2010	172	19	106	194	81	184	127,2567195
14.06.2010	173	16	105	194	81	184	227,5081825
14.06.2010	174	125	211	194	81	184	98,05973595
14.06.2010	175	52	150	194	81	184	181,6426927
14.06.2010	176	37	136	194	81	184	140,0083724
14.06.2010	177	13	97	194	81	184	173,7533511
14.06.2010	178	26	122	194	81	184	148,6393917
14.06.2010	179	54	153	194	81	184	222,4095329
14.06.2010	180	67	166	194	81	184	115,6209053
14.06.2010	181	60	156	194	81	184	112,0560082
14.06.2010	182	13	96	194	81	184	211,6712327
14.06.2010	191	128	203	194	81	184	207,5937826
14.06.2010	193	171	232	194	81	184	89,51812736
15.06.2010	170	49	148	172	19	106	4,462815414
15.06.2010	170	49	148	173	16	105	102,8125615
15.06.2010	172	19	106	173	16	105	99,84533719
15.06.2010	170	49	148	174	125	211	229,1760633
15.06.2010	172	19	106	174	125	211	231,6066062
15.06.2010	173	16	105	174	125	211	330,891905
15.06.2010	170	49	148	175	52	150	26,95441188
15.06.2010	172	19	106	175	52	150	22,83087936

15.06.2010	173	16	105	175	52	150	79,68440466
15.06.2010	174	125	211	175	52	150	251,2599509
15.06.2010	170	49	148	177	13	97	58,81024408
15.06.2010	172	19	106	177	13	97	54,8469112
15.06.2010	173	16	105	177	13	97	52,37018632
15.06.2010	174	125	211	177	13	97	281,1705266
15.06.2010	175	52	150	177	13	97	32,10566749
15.06.2010	170	49	148	178	26	122	34,93995109
15.06.2010	172	19	106	178	26	122	30,84924713
15.06.2010	173	16	105	178	26	122	72,78513779
15.06.2010	174	125	211	178	26	122	258,4466095
15.06.2010	175	52	150	178	26	122	8,023954792
15.06.2010	177	13	97	178	26	122	24,11066958
15.06.2010	170	49	148	179	54	153	105,3263544
15.06.2010	172	19	106	179	54	153	102,289773
15.06.2010	173	16	105	179	54	153	3,284027543
15.06.2010	174	125	211	179	54	153	333,1152158
15.06.2010	175	52	150	179	54	153	81,85911947
15.06.2010	177	13	97	179	54	153	53,86960234
15.06.2010	178	26	122	179	54	153	74,83003604
15.06.2010	170	49	148	180	67	166	1,903166628
15.06.2010	172	19	106	180	67	166	2,912308809
15.06.2010	173	16	105	180	67	166	102,333518
15.06.2010	174	125	211	180	67	166	229,3505376
15.06.2010	175	52	150	180	67	166	25,72327308
15.06.2010	177	13	97	180	67	166	57,70884394
15.06.2010	178	26	122	180	67	166	33,73731996
15.06.2010	179	54	153	180	67	166	104,8095121
15.06.2010	170	49	148	181	60	156	8,750872047
15.06.2010	172	19	106	181	60	156	4,64042495
15.06.2010	173	16	105	181	60	156	95,42288828
15.06.2010	174	125	211	181	60	156	235,8374074
15.06.2010	175	52	150	181	60	156	18,23968268

15.06.2010	177	13	97	181	60	156	50,21599961
15.06.2010	178	26	122	181	60	156	26,2474547
15.06.2010	179	54	153	181	60	156	97,83698523
15.06.2010	180	67	166	181	60	156	7,49548639
15.06.2010	170	49	148	182	13	96	95,37924102
15.06.2010	172	19	106	182	13	96	92,34710525
15.06.2010	173	16	105	182	13	96	7,709346655
15.06.2010	174	125	211	182	13	96	323,256568
15.06.2010	175	52	150	182	13	96	72,02246579
15.06.2010	177	13	97	182	13	96	44,77222941
15.06.2010	178	26	122	182	13	96	65,08762982
15.06.2010	179	54	153	182	13	96	9,946974301
15.06.2010	180	67	166	182	13	96	94,86352467
15.06.2010	181	60	156	182	13	96	87,89935669
15.06.2010	170	49	148	191	128	203	70,46275109
15.06.2010	172	19	106	191	128	203	66,82575262
15.06.2010	173	16	105	191	128	203	37,68461201
15.06.2010	174	125	211	191	128	203	295,2797818
15.06.2010	175	52	150	191	128	203	44,7427651
15.06.2010	177	13	97	191	128	203	14,71365354
15.06.2010	178	26	122	191	128	203	37,07848157
15.06.2010	179	54	153	191	128	203	39,15585635
15.06.2010	180	67	166	191	128	203	69,58586823
15.06.2010	181	60	156	191	128	203	62,19999152
15.06.2010	182	13	96	191	128	203	30,12755513
15.06.2010	170	49	148	193	171	232	15,79602856
15.06.2010	172	19	106	193	171	232	20,14480977
15.06.2010	173	16	105	193	171	232	116,4184356
15.06.2010	174	125	211	193	171	232	217,8457665
15.06.2010	175	52	150	193	171	232	42,69732974
15.06.2010	177	13	97	193	171	232	74,34891954
15.06.2010	178	26	122	193	171	232	50,64585614
15.06.2010	179	54	153	193	171	232	119,0765482

15.06.2010	180	67	166	193	171	232	17,25224103
15.06.2010	181	60	156	193	171	232	24,54378277
15.06.2010	182	13	96	193	171	232	109,1578595
15.06.2010	191	128	203	193	171	232	85,50322027
15.06.2010	170	49	148	194	81	184	18,33944288
15.06.2010	172	19	106	194	81	184	22,22017047
15.06.2010	173	16	105	194	81	184	120,6535941
15.06.2010	174	125	211	194	81	184	212,6383702
15.06.2010	175	52	150	194	81	184	45,04355984
15.06.2010	177	13	97	194	81	184	77,05907145
15.06.2010	178	26	122	194	81	184	53,06513236
15.06.2010	179	54	153	194	81	184	123,2422954
15.06.2010	180	67	166	194	81	184	19,35030653
15.06.2010	181	60	156	194	81	184	26,84393632
15.06.2010	182	13	96	194	81	184	113,302757
15.06.2010	191	128	203	194	81	184	88,79715746
15.06.2010	193	171	232	194	81	184	5,94738385
15.06.2010	170	49	148	172	19	106	19,94903745
15.06.2010	170	49	148	173	16	105	133,0134208
15.06.2010	172	19	106	173	16	105	117,5741724
15.06.2010	170	49	148	174	125	211	231,7449154
15.06.2010	172	19	106	174	125	211	247,1362375
15.06.2010	173	16	105	174	125	211	364,4899122
15.06.2010	170	49	148	175	52	150	65,79651715
15.06.2010	172	19	106	175	52	150	48,03074524
15.06.2010	173	16	105	175	52	150	70,82179016
15.06.2010	174	125	211	175	52	150	294,9491052
15.06.2010	170	49	148	176	37	136	40,8418263
15.06.2010	172	19	106	176	37	136	21,11120399
15.06.2010	173	16	105	176	37	136	99,9483691
15.06.2010	174	125	211	176	37	136	266,2439532
15.06.2010	175	52	150	176	37	136	29,14850929
15.06.2010	170	49	148	177	13	97	61,5505282

15.06.2010	172	19	106	177	13	97	43,11447402
15.06.2010	173	16	105	177	13	97	76,80476279
15.06.2010	174	125	211	177	13	97	289,5828258
15.06.2010	175	52	150	177	13	97	6,305646886
15.06.2010	176	37	136	177	13	97	23,41390921
15.06.2010	170	49	148	178	26	122	51,48726254
15.06.2010	172	19	106	178	26	122	32,08258952
15.06.2010	173	16	105	178	26	122	89,59699771
15.06.2010	174	125	211	178	26	122	277,3348529
15.06.2010	175	52	150	178	26	122	18,9520032
15.06.2010	176	37	136	178	26	122	11,22594374
15.06.2010	177	13	97	178	26	122	12,80039198
15.06.2010	170	49	148	179	54	153	128,5774881
15.06.2010	172	19	106	179	54	153	112,2029395
15.06.2010	173	16	105	179	54	153	10,72432575
15.06.2010	174	125	211	179	54	153	359,337524
15.06.2010	175	52	150	179	54	153	64,57107223
15.06.2010	176	37	136	179	54	153	93,65086275
15.06.2010	177	13	97	179	54	153	70,26647724
15.06.2010	178	26	122	179	54	153	82,93881591
15.06.2010	170	49	148	180	67	166	11,67402087
15.06.2010	172	19	106	180	67	166	8,378591631
15.06.2010	173	16	105	180	67	166	123,2167143
15.06.2010	174	125	211	180	67	166	241,2733289
15.06.2010	175	52	150	180	67	166	54,80185103
15.06.2010	176	37	136	180	67	166	29,17112173
15.06.2010	177	13	97	180	67	166	50,28232911
15.06.2010	178	26	122	180	67	166	39,88539504
15.06.2010	179	54	153	180	67	166	118,3222485
15.06.2010	170	49	148	181	60	156	15,06430222
15.06.2010	172	19	106	181	60	156	34,97857784
15.06.2010	173	16	105	181	60	156	144,6162446
15.06.2010	174	125	211	181	60	156	221,5889943

15.06.2010	175	52	150	181	60	156	79,41746285
15.06.2010	176	37	136	181	60	156	55,69426335
15.06.2010	177	13	97	181	60	156	75,58224476
15.06.2010	178	26	122	181	60	156	66,11540022
15.06.2010	179	54	153	181	60	156	140,8461952
15.06.2010	180	67	166	181	60	156	26,63636503
15.06.2010	170	49	148	182	13	96	97,84866329
15.06.2010	172	19	106	182	13	96	82,17139952
15.06.2010	173	16	105	182	13	96	35,40285922
15.06.2010	174	125	211	182	13	96	329,1337784
15.06.2010	175	52	150	182	13	96	35,94394887
15.06.2010	176	37	136	182	13	96	64,86924816
15.06.2010	177	13	97	182	13	96	42,12645951
15.06.2010	178	26	122	182	13	96	54,88913695
15.06.2010	179	54	153	182	13	96	31,03476099
15.06.2010	180	67	166	182	13	96	87,87556456
15.06.2010	181	60	156	182	13	96	109,8772529
15.06.2010	170	49	148	191	128	203	81,85056749
15.06.2010	172	19	106	191	128	203	64,03053212
15.06.2010	173	16	105	191	128	203	56,43464294
15.06.2010	174	125	211	191	128	203	310,7274829
15.06.2010	175	52	150	191	128	203	16,0715318
15.06.2010	176	37	136	191	128	203	44,54266147
15.06.2010	177	13	97	191	128	203	21,14796748
15.06.2010	178	26	122	191	128	203	33,65757574
15.06.2010	179	54	153	191	128	203	49,32299181
15.06.2010	180	67	166	191	128	203	70,87253243
15.06.2010	181	60	156	191	128	203	95,35575224
15.06.2010	182	13	96	191	128	203	23,51188424
15.06.2010	170	49	148	193	171	232	18,68682646
15.06.2010	172	19	106	193	171	232	38,49772786
15.06.2010	173	16	105	193	171	232	146,5708866
15.06.2010	174	125	211	193	171	232	220,3996948

15.06.2010	175	52	150	193	171	232	82,17299339
15.06.2010	176	37	136	193	171	232	59,04292981
15.06.2010	177	13	97	193	171	232	78,50559108
15.06.2010	178	26	122	193	171	232	69,32238642
15.06.2010	179	54	153	193	171	232	143,0292078
15.06.2010	180	67	166	193	171	232	30,1268056
15.06.2010	181	60	156	193	171	232	3,858186626
15.06.2010	182	13	96	193	171	232	112,0188824
15.06.2010	191	128	203	193	171	232	98,0396073
15.06.2010	170	49	148	194	81	184	34,06079466
15.06.2010	172	19	106	194	81	184	53,97965169
15.06.2010	173	16	105	194	81	184	160,4565804
15.06.2010	174	125	211	194	81	184	209,067563
15.06.2010	175	52	150	194	81	184	97,42222561
15.06.2010	176	37	136	194	81	184	74,61644151
15.06.2010	177	13	97	194	81	184	93,91673266
15.06.2010	178	26	122	194	81	184	84,89138504
15.06.2010	179	54	153	194	81	184	157,3581524
15.06.2010	180	67	166	194	81	184	45,62989958
15.06.2010	181	60	156	194	81	184	19,00221799
15.06.2010	182	13	96	194	81	184	126,324119
15.06.2010	191	128	203	194	81	184	113,1890986
15.06.2010	193	171	232	194	81	184	15,57895995
15.06.2010	170	49	148	172	19	106	10,69311722
15.06.2010	170	49	148	173	16	105	92,20007667
15.06.2010	172	19	106	173	16	105	101,4642941
15.06.2010	170	49	148	174	125	211	259,6901543
15.06.2010	172	19	106	174	125	211	250,6025556
15.06.2010	173	16	105	174	125	211	351,8885534
15.06.2010	170	49	148	175	52	150	53,26425175
15.06.2010	172	19	106	175	52	150	63,35970572
15.06.2010	173	16	105	175	52	150	41,00097168
15.06.2010	174	125	211	175	52	150	312,2663912

15.06.2010	170	49	148	176	37	136	16,4085818
15.06.2010	172	19	106	176	37	136	27,10062325
15.06.2010	173	16	105	176	37	136	78,5142198
15.06.2010	174	125	211	176	37	136	274,0025963
15.06.2010	175	52	150	176	37	136	38,26379996
15.06.2010	170	49	148	177	13	97	41,46799215
15.06.2010	172	19	106	177	13	97	52,02270454
15.06.2010	173	16	105	177	13	97	55,61433207
15.06.2010	174	125	211	177	13	97	298,8111719
15.06.2010	175	52	150	177	13	97	14,70321375
15.06.2010	176	37	136	177	13	97	25,41523266
15.06.2010	170	49	148	178	26	122	28,16894403
15.06.2010	172	19	106	178	26	122	38,80421843
15.06.2010	173	16	105	178	26	122	67,60656926
15.06.2010	174	125	211	178	26	122	285,6214516
15.06.2010	175	52	150	178	26	122	26,8264335
15.06.2010	176	37	136	178	26	122	11,97197286
15.06.2010	177	13	97	178	26	122	13,44734863
15.06.2010	170	49	148	179	54	153	92,949415
15.06.2010	172	19	106	179	54	153	102,142819
15.06.2010	173	16	105	179	54	153	1,463116138
15.06.2010	174	125	211	179	54	153	352,6251548
15.06.2010	175	52	150	179	54	153	42,03222612
15.06.2010	176	37	136	179	54	153	79,39329053
15.06.2010	177	13	97	179	54	153	56,67660434
15.06.2010	178	26	122	179	54	153	68,56341885
15.06.2010	170	49	148	180	67	166	5,13477804
15.06.2010	172	19	106	180	67	166	15,11607848
15.06.2010	173	16	105	180	67	166	90,4447546
15.06.2010	174	125	211	180	67	166	261,6486097
15.06.2010	175	52	150	180	67	166	50,72925379
15.06.2010	176	37	136	180	67	166	12,75656699
15.06.2010	177	13	97	180	67	166	38,17044264

15.06.2010	178	26	122	180	67	166	24,72856768
15.06.2010	179	54	153	180	67	166	91,2592053
15.06.2010	170	49	148	181	60	156	19,5065174
15.06.2010	172	19	106	181	60	156	8,880992252
15.06.2010	173	16	105	181	60	156	109,9054869
15.06.2010	174	125	211	181	60	156	242,4460165
15.06.2010	175	52	150	181	60	156	72,16404291
15.06.2010	176	37	136	181	60	156	35,90032275
15.06.2010	177	13	97	181	60	156	60,90148044
15.06.2010	178	26	122	181	60	156	47,6630968
15.06.2010	179	54	153	181	60	156	110,5494064
15.06.2010	180	67	166	181	60	156	23,6107917
15.06.2010	170	49	148	182	13	96	73,44987635
15.06.2010	172	19	106	182	13	96	83,83096662
15.06.2010	173	16	105	182	13	96	30,41980109
15.06.2010	174	125	211	182	13	96	330,8207464
15.06.2010	175	52	150	182	13	96	21,29523507
15.06.2010	176	37	136	182	13	96	57,64949275
15.06.2010	177	13	97	182	13	96	32,27388859
15.06.2010	178	26	122	182	13	96	45,70614635
15.06.2010	179	54	153	182	13	96	31,82989778
15.06.2010	180	67	166	182	13	96	70,38784909
15.06.2010	181	60	156	182	13	96	92,69748284
15.06.2010	170	49	148	191	128	203	70,72875326
15.06.2010	172	19	106	191	128	203	81,07474465
15.06.2010	173	16	105	191	128	203	30,96774818
15.06.2010	174	125	211	191	128	203	328,3957545
15.06.2010	175	52	150	191	128	203	18,3851901
15.06.2010	176	37	136	191	128	203	55,01225391
15.06.2010	177	13	97	191	128	203	29,69436409
15.06.2010	178	26	122	191	128	203	43,09435723
15.06.2010	179	54	153	191	128	203	32,33707758
15.06.2010	180	67	166	191	128	203	67,7345079

15.06.2010	181	60	156	191	128	203	89,93509091
15.06.2010	182	13	96	191	128	203	2,926232276
15.06.2010	170	49	148	193	171	232	49,10191609
15.06.2010	172	19	106	193	171	232	38,41622674
15.06.2010	173	16	105	193	171	232	135,8809802
15.06.2010	174	125	211	193	171	232	220,1848419
15.06.2010	175	52	150	193	171	232	100,3209559
15.06.2010	176	37	136	193	171	232	65,4943571
15.06.2010	177	13	97	193	171	232	90,05658402
15.06.2010	178	26	122	193	171	232	77,05491018
15.06.2010	179	54	153	193	171	232	136,3629911
15.06.2010	180	67	166	193	171	232	53,38823966
15.06.2010	181	60	156	193	171	232	29,78608576
15.06.2010	182	13	96	193	171	232	121,2973109
15.06.2010	191	128	203	193	171	232	118,4663257
15.06.2010	170	49	148	194	81	184	54,36348544
15.06.2010	172	19	106	194	81	184	44,35407897
15.06.2010	173	16	105	194	81	184	133,7420691
15.06.2010	174	125	211	194	81	184	228,3200292
15.06.2010	175	52	150	194	81	184	101,2726274
15.06.2010	176	37	136	194	81	184	70,04219086
15.06.2010	177	13	97	194	81	184	92,86337371
15.06.2010	178	26	122	194	81	184	80,70211358
15.06.2010	179	54	153	194	81	184	134,0610371
15.06.2010	180	67	166	194	81	184	59,25763263
15.06.2010	181	60	156	194	81	184	37,16716975
15.06.2010	182	13	96	194	81	184	122,5578375
15.06.2010	191	128	203	194	81	184	119,6549018
15.06.2010	193	171	232	194	81	184	15,82915953
15.06.2010	170	49	148	172	19	106	34,4522177
15.06.2010	170	49	148	173	16	105	115,6161007
15.06.2010	172	19	106	173	16	105	86,94809923
15.06.2010	170	49	148	174	125	211	223,5734367

15.06.2010	172	19	106	174	125	211	252,8202866
15.06.2010	173	16	105	174	125	211	338,8303514
15.06.2010	170	49	148	175	52	150	79,76444888
15.06.2010	172	19	106	175	52	150	47,0476769
15.06.2010	173	16	105	175	52	150	43,49513298
15.06.2010	174	125	211	175	52	150	299,6650927
15.06.2010	170	49	148	176	37	136	46,74808778
15.06.2010	172	19	106	176	37	136	12,74168887
15.06.2010	173	16	105	176	37	136	75,26380768
15.06.2010	174	125	211	176	37	136	265,296366
15.06.2010	175	52	150	176	37	136	34,40827529
15.06.2010	170	49	148	177	13	97	61,1641708
15.06.2010	172	19	106	177	13	97	27,5257408
15.06.2010	173	16	105	177	13	97	61,99963172
15.06.2010	174	125	211	177	13	97	279,8836527
15.06.2010	175	52	150	177	13	97	19,8006529
15.06.2010	176	37	136	177	13	97	14,78713654
15.06.2010	170	49	148	178	26	122	53,4687114
15.06.2010	172	19	106	178	26	122	19,44436946
15.06.2010	173	16	105	178	26	122	69,72189971
15.06.2010	174	125	211	178	26	122	271,6700117
15.06.2010	175	52	150	178	26	122	28,00299699
15.06.2010	176	37	136	178	26	122	6,743125354
15.06.2010	177	13	97	178	26	122	8,215293038
15.06.2010	170	49	148	179	54	153	120,2398639
15.06.2010	172	19	106	179	54	153	90,95325062
15.06.2010	173	16	105	179	54	153	5,507379117
15.06.2010	174	125	211	179	54	153	343,2270604
15.06.2010	175	52	150	179	54	153	46,38088577
15.06.2010	176	37	136	179	54	153	79,04290399
15.06.2010	177	13	97	179	54	153	65,41362821
15.06.2010	178	26	122	179	54	153	73,2974131
15.06.2010	170	49	148	180	67	166	23,3379811

15.06.2010	172	19	106	180	67	166	11,17069186
15.06.2010	173	16	105	180	67	166	96,3321935
15.06.2010	174	125	211	180	67	166	242,7452942
15.06.2010	175	52	150	180	67	166	57,71189598
15.06.2010	176	37	136	180	67	166	23,75876102
15.06.2010	177	13	97	180	67	166	38,46075668
15.06.2010	178	26	122	180	67	166	30,50016275
15.06.2010	179	54	153	180	67	166	100,566724
15.06.2010	170	49	148	181	60	156	10,91797853
15.06.2010	172	19	106	181	60	156	44,8281784
15.06.2010	173	16	105	181	60	156	123,0046774
15.06.2010	174	125	211	181	60	156	217,484719
15.06.2010	175	52	150	181	60	156	88,96392486
15.06.2010	176	37	136	181	60	156	56,79457443
15.06.2010	177	13	97	181	60	156	70,90559734
15.06.2010	178	26	122	181	60	156	63,44931227
15.06.2010	179	54	153	181	60	156	127,8140311
15.06.2010	180	67	166	181	60	156	33,8691875
15.06.2010	170	49	148	182	13	96	78,2776798
15.06.2010	172	19	106	182	13	96	45,22387529
15.06.2010	173	16	105	182	13	96	46,03214648
15.06.2010	174	125	211	182	13	96	297,6635001
15.06.2010	175	52	150	182	13	96	2,585356799
15.06.2010	176	37	136	182	13	96	32,52382519
15.06.2010	177	13	97	182	13	96	17,79399314
15.06.2010	178	26	122	182	13	96	26,00874774
15.06.2010	179	54	153	182	13	96	48,85294609
15.06.2010	180	67	166	182	13	96	56,00357476
15.06.2010	181	60	156	182	13	96	87,62651682
15.06.2010	170	49	148	191	128	203	78,6225158
15.06.2010	172	19	106	191	128	203	45,51375193
15.06.2010	173	16	105	191	128	203	46,01173485
15.06.2010	174	125	211	191	128	203	297,9062502

15.06.2010	175	52	150	191	128	203	2,691484058
15.06.2010	176	37	136	191	128	203	32,80436254
15.06.2010	177	13	97	191	128	203	18,05577434
15.06.2010	178	26	122	191	128	203	26,26706277
15.06.2010	179	54	153	191	128	203	48,78606179
15.06.2010	180	67	166	191	128	203	56,31383377
15.06.2010	181	60	156	191	128	203	87,9918433
15.06.2010	182	13	96	191	128	203	0,465597081
15.06.2010	170	49	148	193	171	232	11,48879076
15.06.2010	172	19	106	193	171	232	23,16355224
15.06.2010	173	16	105	193	171	232	106,7123387
15.06.2010	174	125	211	193	171	232	232,1181818
15.06.2010	175	52	150	193	171	232	69,30107952
15.06.2010	176	37	136	193	171	232	35,67949344
15.06.2010	177	13	97	193	171	232	50,29364492
15.06.2010	178	26	122	193	171	232	42,42302306
15.06.2010	179	54	153	193	171	232	111,1385693
15.06.2010	180	67	166	193	171	232	11,99307949
15.06.2010	181	60	156	193	171	232	22,28329176
15.06.2010	182	13	96	193	171	232	67,68494953
15.06.2010	191	128	203	193	171	232	68,010119
15.06.2010	170	49	148	194	81	184	34,88587947
15.06.2010	172	19	106	194	81	184	68,86313039
15.06.2010	173	16	105	194	81	184	149,5635959
15.06.2010	174	125	211	194	81	184	191,3491422
15.06.2010	175	52	150	194	81	184	114,6496768
15.06.2010	176	37	136	194	81	184	81,40640192
15.06.2010	177	13	97	194	81	184	95,96129779
15.06.2010	178	26	122	194	81	184	88,14860703
15.06.2010	179	54	153	194	81	184	154,3512257
15.06.2010	180	67	166	194	81	184	57,70054784
15.06.2010	181	60	156	194	81	184	26,57270676
15.06.2010	182	13	96	194	81	184	113,1592493

15.06.2010	191	128	203	194	81	184	113,5020419
15.06.2010	193	171	232	194	81	184	45,73074363
15.06.2010	170	49	148	172	19	106	8,908410622
15.06.2010	170	49	148	173	16	105	116,2315343
15.06.2010	172	19	106	173	16	105	108,2831908
15.06.2010	170	49	148	174	125	211	241,1318508
15.06.2010	172	19	106	174	125	211	248,8587541
15.06.2010	173	16	105	174	125	211	357,1181333
15.06.2010	170	49	148	175	52	150	68,39167863
15.06.2010	172	19	106	175	52	150	59,82529946
15.06.2010	173	16	105	175	52	150	50,78157457
15.06.2010	174	125	211	175	52	150	307,5812042
15.06.2010	170	49	148	176	37	136	32,77943432
15.06.2010	172	19	106	176	37	136	23,89183038
15.06.2010	173	16	105	176	37	136	86,85101726
15.06.2010	174	125	211	176	37	136	270,7459534
15.06.2010	175	52	150	176	37	136	36,8572366
15.06.2010	170	49	148	177	13	97	60,33121381
15.06.2010	172	19	106	177	13	97	51,55756288
15.06.2010	173	16	105	177	13	97	60,9140858
15.06.2010	174	125	211	177	13	97	298,1953479
15.06.2010	175	52	150	177	13	97	10,25283112
15.06.2010	176	37	136	177	13	97	27,92418248
15.06.2010	170	49	148	178	26	122	43,78923422
15.06.2010	172	19	106	178	26	122	34,88070075
15.06.2010	173	16	105	178	26	122	79,02621827
15.06.2010	174	125	211	178	26	122	279,8774724
15.06.2010	175	52	150	178	26	122	28,26625522
15.06.2010	176	37	136	178	26	122	11,19855353
15.06.2010	177	13	97	178	26	122	18,38666393
15.06.2010	170	49	148	179	54	153	96,27352602
15.06.2010	172	19	106	179	54	153	88,21103996
15.06.2010	173	16	105	179	54	153	20,23808105

15.06.2010	174	125	211	179	54	153	336,9831578
15.06.2010	175	52	150	179	54	153	30,73734261
15.06.2010	176	37	136	179	54	153	66,61361178
15.06.2010	177	13	97	179	54	153	40,95369001
15.06.2010	178	26	122	179	54	153	58,89841409
15.06.2010	170	49	148	180	67	166	4,834891846
15.06.2010	172	19	106	180	67	166	4,128483732
15.06.2010	173	16	105	180	67	166	111,7026735
15.06.2010	174	125	211	180	67	166	245,5361053
15.06.2010	175	52	150	180	67	166	63,62275216
15.06.2010	176	37	136	180	67	166	27,95037096
15.06.2010	177	13	97	180	67	166	55,50654737
15.06.2010	178	26	122	180	67	166	38,98014425
15.06.2010	179	54	153	180	67	166	91,69460402
15.06.2010	170	49	148	181	60	156	21,97306649
15.06.2010	172	19	106	181	60	156	30,80133783
15.06.2010	173	16	105	181	60	156	134,8275412
15.06.2010	174	125	211	181	60	156	224,6114666
15.06.2010	175	52	150	181	60	156	88,91487224
15.06.2010	176	37	136	181	60	156	54,44019291
15.06.2010	177	13	97	181	60	156	81,48115193
15.06.2010	178	26	122	181	60	156	65,56575714
15.06.2010	179	54	153	181	60	156	115,2982804
15.06.2010	180	67	166	181	60	156	26,67347119
15.06.2010	170	49	148	182	13	96	81,31458956
15.06.2010	172	19	106	182	13	96	72,69523495
15.06.2010	173	16	105	182	13	96	40,41206537
15.06.2010	174	125	211	182	13	96	319,9190972
15.06.2010	175	52	150	182	13	96	12,9943855
15.06.2010	176	37	136	182	13	96	49,43996483
15.06.2010	177	13	97	182	13	96	21,72439922
15.06.2010	178	26	122	182	13	96	40,08351019
15.06.2010	179	54	153	182	13	96	21,7025599

15.06.2010	180	67	166	182	13	96	76,53090468
15.06.2010	181	60	156	182	13	96	101,9087853
15.06.2010	170	49	148	191	128	203	83,13113856
15.06.2010	172	19	106	191	128	203	74,46121145
15.06.2010	173	16	105	191	128	203	40,14689405
15.06.2010	174	125	211	191	128	203	321,2890319
15.06.2010	175	52	150	191	128	203	15,04930481
15.06.2010	176	37	136	191	128	203	51,04378967
15.06.2010	177	13	97	191	128	203	23,17154475
15.06.2010	178	26	122	191	128	203	41,41197477
15.06.2010	179	54	153	191	128	203	22,17052186
15.06.2010	180	67	166	191	128	203	78,33287486
15.06.2010	181	60	156	191	128	203	103,865811
15.06.2010	182	13	96	191	128	203	2,560783943
15.06.2010	170	49	148	194	81	184	30,62429183
15.06.2010	172	19	106	194	81	184	39,22822343
15.06.2010	173	16	105	194	81	184	140,2265507
15.06.2010	174	125	211	194	81	184	221,5616206
15.06.2010	175	52	150	194	81	184	95,76257868
15.06.2010	176	37	136	194	81	184	62,40158974
15.06.2010	177	13	97	194	81	184	88,83229508
15.06.2010	178	26	122	194	81	184	73,59208269
15.06.2010	179	54	153	194	81	184	121,0446452
15.06.2010	180	67	166	194	81	184	35,12970341
15.06.2010	181	60	156	194	81	184	9,364657066
15.06.2010	182	13	96	194	81	184	108,7403452
15.06.2010	191	128	203	194	81	184	110,7927444
15.06.2010	170	49	148	172	19	106	34,53470423
15.06.2010	170	49	148	173	16	105	130,7454165
15.06.2010	172	19	106	173	16	105	104,6258732
15.06.2010	170	49	148	174	125	211	221,9977304
15.06.2010	172	19	106	174	125	211	248,3279972
15.06.2010	173	16	105	174	125	211	352,0384012

15.06.2010	170	49	148	175	52	150	82,46535744
15.06.2010	172	19	106	175	52	150	51,33212212
15.06.2010	173	16	105	175	52	150	56,08440077
15.06.2010	174	125	211	175	52	150	299,5199988
15.06.2010	170	49	148	176	37	136	47,09699163
15.06.2010	172	19	106	176	37	136	12,64526871
15.06.2010	173	16	105	176	37	136	95,13796871
15.06.2010	174	125	211	176	37	136	259,3562591
15.06.2010	175	52	150	176	37	136	40,269886
15.06.2010	170	49	148	177	13	97	70,43807045
15.06.2010	172	19	106	177	13	97	38,39541274
15.06.2010	173	16	105	177	13	97	68,71849875
15.06.2010	174	125	211	177	13	97	286,4325023
15.06.2010	175	52	150	177	13	97	13,13473488
15.06.2010	176	37	136	177	13	97	27,13742746
15.06.2010	170	49	148	178	26	122	55,72012515
15.06.2010	172	19	106	178	26	122	22,5346101
15.06.2010	173	16	105	178	26	122	84,00619543
15.06.2010	174	125	211	178	26	122	270,4012752
15.06.2010	175	52	150	178	26	122	29,13688649
15.06.2010	176	37	136	178	26	122	11,1945199
15.06.2010	177	13	97	178	26	122	16,0315787
15.06.2010	170	49	148	179	54	153	112,6353663
15.06.2010	172	19	106	179	54	153	85,96268013
15.06.2010	173	16	105	179	54	153	18,6776403
15.06.2010	174	125	211	179	54	153	333,5723919
15.06.2010	175	52	150	179	54	153	37,87074895
15.06.2010	176	37	136	179	54	153	76,48751835
15.06.2010	177	13	97	179	54	153	50,24063623
15.06.2010	178	26	122	179	54	153	65,37398631
15.06.2010	170	49	148	180	67	166	27,01265702
15.06.2010	172	19	106	180	67	166	7,704658697
15.06.2010	173	16	105	180	67	166	108,934627

15.06.2010	174	125	211	180	67	166	243,3106727
15.06.2010	175	52	150	180	67	166	57,12251933
15.06.2010	176	37	136	180	67	166	20,09542746
15.06.2010	177	13	97	180	67	166	44,49010948
15.06.2010	178	26	122	180	67	166	29,08708843
15.06.2010	179	54	153	180	67	166	90,35952714
15.06.2010	170	49	148	181	60	156	10,12963492
15.06.2010	172	19	106	181	60	156	24,45400646
15.06.2010	173	16	105	181	60	156	123,2017589
15.06.2010	174	125	211	181	60	156	228,9170915
15.06.2010	175	52	150	181	60	156	73,37353044
15.06.2010	176	37	136	181	60	156	37,05789118
15.06.2010	177	13	97	181	60	156	61,05996992
15.06.2010	178	26	122	181	60	156	45,99858026
15.06.2010	179	54	153	181	60	156	104,8628442
15.06.2010	180	67	166	181	60	156	17,03463981
15.06.2010	170	49	148	182	13	96	91,60723626
15.06.2010	172	19	106	182	13	96	60,44847692
15.06.2010	173	16	105	182	13	96	48,71129824
15.06.2010	174	125	211	182	13	96	308,5285709
15.06.2010	175	52	150	182	13	96	9,183009828
15.06.2010	176	37	136	182	13	96	49,18976783
15.06.2010	177	13	97	182	13	96	22,10462696
15.06.2010	178	26	122	182	13	96	38,13279884
15.06.2010	179	54	153	182	13	96	31,25314984
15.06.2010	180	67	166	182	13	96	66,29991804
15.06.2010	181	60	156	182	13	96	82,54905154
15.06.2010	170	49	148	191	128	203	91,29384054
15.06.2010	172	19	106	191	128	203	62,87291968
15.06.2010	173	16	105	191	128	203	42,12742437
15.06.2010	174	125	211	191	128	203	311,003864
15.06.2010	175	52	150	191	128	203	15,03549397
15.06.2010	176	37	136	191	128	203	53,02477585

15.06.2010	177	13	97	191	128	203	26,76879546
15.06.2010	178	26	122	191	128	203	41,88045093
15.06.2010	179	54	153	191	128	203	23,52667008
15.06.2010	180	67	166	191	128	203	67,71256796
15.06.2010	181	60	156	191	128	203	82,94066729
15.06.2010	182	13	96	191	128	203	11,92283127
15.06.2010	170	49	148	193	171	232	18,24141585
15.06.2010	172	19	106	193	171	232	52,62139673
15.06.2010	173	16	105	193	171	232	144,2505869
15.06.2010	174	125	211	193	171	232	211,3520155
15.06.2010	175	52	150	193	171	232	98,80837795
15.06.2010	176	37	136	193	171	232	65,083744
15.06.2010	177	13	97	193	171	232	87,30141844
15.06.2010	178	26	122	193	171	232	73,18703809
15.06.2010	179	54	153	193	171	232	126,6464713
15.06.2010	180	67	166	193	171	232	45,0012284
15.06.2010	181	60	156	193	171	232	28,35112852
15.06.2010	182	13	96	193	171	232	107,840851
15.06.2010	191	128	203	193	171	232	106,4124903
15.06.2010	170	49	148	194	81	184	22,68402483
15.06.2010	172	19	106	194	81	184	57,20286964
15.06.2010	173	16	105	194	81	184	150,3032666
15.06.2010	174	125	211	194	81	184	205,2413686
15.06.2010	175	52	150	194	81	184	104,3627487
15.06.2010	176	37	136	194	81	184	69,77991432
15.06.2010	177	13	97	194	81	184	92,67438647
15.06.2010	178	26	122	194	81	184	78,27387181
15.06.2010	179	54	153	194	81	184	132,6450592
15.06.2010	180	67	166	194	81	184	49,69653628
15.06.2010	181	60	156	194	81	184	32,75352194
15.06.2010	182	13	96	194	81	184	113,4330247
15.06.2010	191	128	203	194	81	184	112,2471262
15.06.2010	193	171	232	194	81	184	6,147527294

15.06.2010	170	49	148	172	19	106	7,12
15.06.2010	170	49	148	173	16	105	85,79
15.06.2010	172	19	106	173	16	105	83,93
15.06.2010	170	49	148	174	125	211	260,31
15.06.2010	172	19	106	174	125	211	262,13
15.06.2010	173	16	105	174	125	211	345,98
15.06.2010	170	49	148	175	52	150	57,15
15.06.2010	172	19	106	175	52	150	54,06
15.06.2010	173	16	105	175	52	150	31,92
15.06.2010	174	125	211	175	52	150	315,86
15.06.2010	170	49	148	176	37	136	20,57
15.06.2010	172	19	106	176	37	136	16,15
15.06.2010	173	16	105	176	37	136	68,57
15.06.2010	174	125	211	176	37	136	277,86
15.06.2010	175	52	150	176	37	136	38,06
15.06.2010	170	49	148	177	13	97	49,24
15.06.2010	172	19	106	177	13	97	45,68
15.06.2010	173	16	105	177	13	97	40,86
15.06.2010	174	125	211	177	13	97	307,15
15.06.2010	175	52	150	177	13	97	8,99
15.06.2010	176	37	136	177	13	97	29,57
15.06.2010	170	49	148	178	26	122	37,19
15.06.2010	172	19	106	178	26	122	33,66
15.06.2010	173	16	105	178	26	122	51,42
15.06.2010	174	125	211	178	26	122	295,43
15.06.2010	175	52	150	178	26	122	20,45
15.06.2010	176	37	136	178	26	122	17,61
15.06.2010	177	13	97	178	26	122	12,07
15.06.2010	170	49	148	179	54	153	87,50
15.06.2010	172	19	106	179	54	153	85,58
15.06.2010	173	16	105	179	54	153	1,85
15.06.2010	174	125	211	179	54	153	347,66
15.06.2010	175	52	150	179	54	153	33,29

15.06.2010	176	37	136	179	54	153	70,17
15.06.2010	177	13	97	179	54	153	42,25
15.06.2010	178	26	122	179	54	153	52,95
15.06.2010	170	49	148	180	67	166	13,78
15.06.2010	172	19	106	180	67	166	8,07
15.06.2010	173	16	105	180	67	166	76,86
15.06.2010	174	125	211	180	67	166	269,48
15.06.2010	175	52	150	180	67	166	46,44
15.06.2010	176	37	136	180	67	166	8,38
15.06.2010	177	13	97	180	67	166	37,91
15.06.2010	178	26	122	180	67	166	25,99
15.06.2010	179	54	153	180	67	166	78,47
15.06.2010	170	49	148	181	60	156	15,51
15.06.2010	172	19	106	181	60	156	21,43
15.06.2010	173	16	105	181	60	156	99,25
15.06.2010	174	125	211	181	60	156	247,87
15.06.2010	175	52	150	181	60	156	71,87
15.06.2010	176	37	136	181	60	156	36,06
15.06.2010	177	13	97	181	60	156	64,28
15.06.2010	178	26	122	181	60	156	52,31
15.06.2010	179	54	153	181	60	156	101,02
15.06.2010	180	67	166	181	60	156	29,08
15.06.2010	170	49	148	182	13	96	72,00
15.06.2010	172	19	106	182	13	96	69,04
15.06.2010	173	16	105	182	13	96	19,16
15.06.2010	174	125	211	182	13	96	330,83
15.06.2010	175	52	150	182	13	96	15,00
15.06.2010	176	37	136	182	13	96	53,06
15.06.2010	177	13	97	182	13	96	23,73
15.06.2010	178	26	122	182	13	96	35,45
15.06.2010	179	54	153	182	13	96	20,10
15.06.2010	180	67	166	182	13	96	61,44
15.06.2010	181	60	156	182	13	96	86,53

15.06.2010	170	49	148	191	128	203	69,91
15.06.2010	172	19	106	191	128	203	66,84
15.06.2010	173	16	105	191	128	203	21,47
15.06.2010	174	125	211	191	128	203	328,55
15.06.2010	175	52	150	191	128	203	12,79
15.06.2010	176	37	136	191	128	203	50,82
15.06.2010	177	13	97	191	128	203	21,41
15.06.2010	178	26	122	191	128	203	33,21
15.06.2010	179	54	153	191	128	203	22,46
15.06.2010	180	67	166	191	128	203	59,20
15.06.2010	181	60	156	191	128	203	84,53
15.06.2010	182	13	96	191	128	203	2,43
15.06.2010	170	49	148	193	171	232	34,10
15.06.2010	172	19	106	193	171	232	39,31
15.06.2010	173	16	105	193	171	232	117,48
15.06.2010	174	125	211	193	171	232	231,07
15.06.2010	175	52	150	193	171	232	90,61
15.06.2010	176	37	136	193	171	232	54,65
15.06.2010	177	13	97	193	171	232	83,07
15.06.2010	178	26	122	193	171	232	71,10
15.06.2010	179	54	153	193	171	232	119,26
15.06.2010	180	67	166	193	171	232	47,26
15.06.2010	181	60	156	193	171	232	18,79
15.06.2010	182	13	96	193	171	232	105,22
15.06.2010	191	128	203	193	171	232	103,24
15.06.2010	170	49	148	194	81	184	45,40
15.06.2010	172	19	106	194	81	184	50,88
15.06.2010	173	16	105	194	81	184	127,11
15.06.2010	174	125	211	194	81	184	223,89
15.06.2010	175	52	150	194	81	184	101,22
15.06.2010	176	37	136	194	81	184	65,96
15.06.2010	177	13	97	194	81	184	93,93
15.06.2010	178	26	122	194	81	184	82,05

15.06.2010	179	54	153	194	81	184	128,92
15.06.2010	180	67	166	194	81	184	58,77
15.06.2010	181	60	156	194	81	184	29,90
15.06.2010	182	13	96	194	81	184	115,63
15.06.2010	191	128	203	194	81	184	113,72
15.06.2010	193	171	232	194	81	184	11,72
15.06.2010	170	49	148	172	19	106	16,56090703
15.06.2010	170	49	148	173	16	105	102,1502911
15.06.2010	172	19	106	173	16	105	87,04860097
15.06.2010	170	49	148	174	125	211	219,8476456
15.06.2010	172	19	106	174	125	211	234,9744468
15.06.2010	173	16	105	174	125	211	321,9242447
15.06.2010	170	49	148	175	52	150	76,96025393
15.06.2010	172	19	106	175	52	150	60,59622731
15.06.2010	173	16	105	175	52	150	33,60506936
15.06.2010	174	125	211	175	52	150	294,2930829
15.06.2010	170	49	148	176	37	136	28,9537189
15.06.2010	172	19	106	176	37	136	12,99264761
15.06.2010	173	16	105	176	37	136	79,26223378
15.06.2010	174	125	211	176	37	136	244,1031922
15.06.2010	175	52	150	176	37	136	50,24108569
15.06.2010	170	49	148	177	13	97	51,90563529
15.06.2010	172	19	106	177	13	97	35,35460573
15.06.2010	173	16	105	177	13	97	56,61146443
15.06.2010	174	125	211	177	13	97	268,3159466
15.06.2010	175	52	150	177	13	97	25,97729916
15.06.2010	176	37	136	177	13	97	24,30413787
15.06.2010	170	49	148	178	26	122	43,58703261
15.06.2010	172	19	106	178	26	122	27,17085768
15.06.2010	173	16	105	178	26	122	66,99463109
15.06.2010	174	125	211	178	26	122	258,234625
15.06.2010	175	52	150	178	26	122	36,16266378
15.06.2010	176	37	136	178	26	122	14,86815587

15.06.2010	177	13	97	178	26	122	10,43100268
15.06.2010	170	49	148	179	54	153	125,3812682
15.06.2010	172	19	106	179	54	153	110,4828778
15.06.2010	173	16	105	179	54	153	23,54413229
15.06.2010	174	125	211	179	54	153	345,2230196
15.06.2010	175	52	150	179	54	153	55,90187297
15.06.2010	176	37	136	179	54	153	102,804171
15.06.2010	177	13	97	179	54	153	79,97400734
15.06.2010	178	26	122	179	54	153	90,38811177
15.06.2010	170	49	148	180	67	166	19,80703829
15.06.2010	172	19	106	180	67	166	3,335876634
15.06.2010	173	16	105	180	67	166	84,55908279
15.06.2010	174	125	211	180	67	166	237,6192768
15.06.2010	175	52	150	180	67	166	57,57925729
15.06.2010	176	37	136	180	67	166	9,707075708
15.06.2010	177	13	97	180	67	166	32,17845139
15.06.2010	178	26	122	180	67	166	23,84907143
15.06.2010	179	54	153	180	67	166	108,0397601
15.06.2010	170	49	148	181	60	156	12,86514748
15.06.2010	172	19	106	181	60	156	29,30704636
15.06.2010	173	16	105	181	60	156	113,1954026
15.06.2010	174	125	211	181	60	156	209,4779426
15.06.2010	175	52	150	181	60	156	89,26991835
15.06.2010	176	37	136	181	60	156	41,81904664
15.06.2010	177	13	97	181	60	156	64,54469852
15.06.2010	178	26	122	181	60	156	56,42279778
15.06.2010	179	54	153	181	60	156	136,168375
15.06.2010	180	67	166	181	60	156	32,59719944
15.06.2010	170	49	148	182	13	96	79,4045529
15.06.2010	172	19	106	182	13	96	63,1175333
15.06.2010	173	16	105	182	13	96	30,50490073
15.06.2010	174	125	211	182	13	96	297,0973506
15.06.2010	175	52	150	182	13	96	3,146370812

15.06.2010	176	37	136	182	13	96	53,00313757
15.06.2010	177	13	97	182	13	96	28,81064171
15.06.2010	178	26	122	182	13	96	39,07757712
15.06.2010	179	54	153	182	13	96	52,75545721
15.06.2010	180	67	166	182	13	96	60,14573043
15.06.2010	181	60	156	182	13	96	91,62000415
15.06.2010	170	49	148	194	81	184	38,04066343
15.06.2010	172	19	106	194	81	184	54,4880388
15.06.2010	173	16	105	194	81	184	136,353883
15.06.2010	174	125	211	194	81	184	189,051718
15.06.2010	175	52	150	194	81	184	114,0251908
15.06.2010	176	37	136	194	81	184	66,98263063
15.06.2010	177	13	97	194	81	184	89,63479455
15.06.2010	178	26	122	194	81	184	81,61462191
15.06.2010	179	54	153	194	81	184	158,8610257
15.06.2010	180	67	166	194	81	184	57,78556548
15.06.2010	181	60	156	194	81	184	25,19177103
15.06.2010	182	13	96	194	81	184	116,2714323
16.06.2010	170	49	148	172	19	106	9,172183705
16.06.2010	170	49	148	173	16	105	117,2653181
16.06.2010	172	19	106	173	16	105	109,3248814
16.06.2010	170	49	148	174	125	211	255,6690472
16.06.2010	172	19	106	174	125	211	263,896904
16.06.2010	173	16	105	174	125	211	372,8742536
16.06.2010	170	49	148	175	52	150	62,57973778
16.06.2010	172	19	106	175	52	150	54,02222092
16.06.2010	173	16	105	175	52	150	56,44600555
16.06.2010	174	125	211	175	52	150	317,8576857
16.06.2010	170	49	148	176	37	136	17,29198556
16.06.2010	172	19	106	176	37	136	8,122411908
16.06.2010	173	16	105	176	37	136	102,6433906
16.06.2010	174	125	211	176	37	136	271,0961742
16.06.2010	175	52	150	176	37	136	46,78613605

16.06.2010	170	49	148	177	13	97	41,69390877
16.06.2010	172	19	106	177	13	97	32,7781943
16.06.2010	173	16	105	177	13	97	78,30779102
16.06.2010	174	125	211	177	13	97	296,1937037
16.06.2010	175	52	150	177	13	97	21,93130293
16.06.2010	176	37	136	177	13	97	25,15409504
16.06.2010	170	49	148	178	26	122	34,19550913
16.06.2010	172	19	106	178	26	122	25,04884167
16.06.2010	173	16	105	178	26	122	87,55309322
16.06.2010	174	125	211	178	26	122	287,4544177
16.06.2010	175	52	150	178	26	122	31,12311305
16.06.2010	176	37	136	178	26	122	17,02084787
16.06.2010	177	13	97	178	26	122	9,299323141
16.06.2010	170	49	148	179	54	153	117,2757162
16.06.2010	172	19	106	179	54	153	109,2246575
16.06.2010	173	16	105	179	54	153	2,787108355
16.06.2010	174	125	211	179	54	153	372,9358261
16.06.2010	175	52	150	179	54	153	55,98768595
16.06.2010	176	37	136	179	54	153	102,4240384
16.06.2010	177	13	97	179	54	153	77,90314828
16.06.2010	178	26	122	179	54	153	87,10995586
16.06.2010	170	49	148	180	67	166	6,183420221
16.06.2010	172	19	106	180	67	166	15,35470653
16.06.2010	173	16	105	180	67	166	122,6256965
16.06.2010	174	125	211	180	67	166	250,2485773
16.06.2010	175	52	150	180	67	166	68,37860239
16.06.2010	176	37	136	180	67	166	23,47574462
16.06.2010	177	13	97	180	67	166	47,72256514
16.06.2010	178	26	122	180	67	166	40,35614902
16.06.2010	179	54	153	180	67	166	122,706327
16.06.2010	170	49	148	181	60	156	21,96011389
16.06.2010	172	19	106	181	60	156	31,05629089
16.06.2010	173	16	105	181	60	156	135,3049603

16.06.2010	174	125	211	181	60	156	238,1333354
16.06.2010	175	52	150	181	60	156	82,5668304
16.06.2010	176	37	136	181	60	156	39,16323746
16.06.2010	177	13	97	181	60	156	62,70554473
16.06.2010	178	26	122	181	60	156	55,83849837
16.06.2010	179	54	153	181	60	156	135,5874472
16.06.2010	180	67	166	181	60	156	15,84142323
16.06.2010	170	49	148	182	13	96	70,6549003
16.06.2010	172	19	106	182	13	96	61,66788952
16.06.2010	173	16	105	182	13	96	54,72300961
16.06.2010	174	125	211	182	13	96	324,1972733
16.06.2010	175	52	150	182	13	96	12,97351545
16.06.2010	176	37	136	182	13	96	53,84528414
16.06.2010	177	13	97	182	13	96	28,98536598
16.06.2010	178	26	122	182	13	96	36,96478704
16.06.2010	179	54	153	182	13	96	53,64310174
16.06.2010	180	67	166	182	13	96	76,7032625
16.06.2010	181	60	156	182	13	96	91,63967011
16.06.2010	170	49	148	191	128	203	70,12531968
16.06.2010	172	19	106	191	128	203	61,16143872
16.06.2010	173	16	105	191	128	203	54,60006759
16.06.2010	174	125	211	191	128	203	323,8486536
16.06.2010	175	52	150	191	128	203	12,02842521
16.06.2010	176	37	136	191	128	203	53,37097993
16.06.2010	177	13	97	191	128	203	28,43717787
16.06.2010	178	26	122	191	128	203	36,53478711
16.06.2010	179	54	153	191	128	203	53,56559007
16.06.2010	180	67	166	191	128	203	76,15940587
16.06.2010	181	60	156	191	128	203	91,04655374
16.06.2010	182	13	96	191	128	203	0,95513622
16.06.2010	170	49	148	193	171	232	33,77879277
16.06.2010	172	19	106	193	171	232	42,60128478
16.06.2010	173	16	105	193	171	232	142,5349502

16.06.2010	174	125	211	193	171	232	232,8173186
16.06.2010	175	52	150	193	171	232	91,73163503
16.06.2010	176	37	136	193	171	232	50,57750319
16.06.2010	177	13	97	193	171	232	72,94412049
16.06.2010	178	26	122	193	171	232	66,77470821
16.06.2010	179	54	153	193	171	232	143,0203979
16.06.2010	180	67	166	193	171	232	27,89181711
16.06.2010	181	60	156	193	171	232	12,57902235
16.06.2010	182	13	96	193	171	232	101,5938271
16.06.2010	191	128	203	193	171	232	100,9422631
16.06.2010	170	49	148	194	81	184	44,93182704
16.06.2010	172	19	106	194	81	184	53,84763258
16.06.2010	173	16	105	194	81	184	152,8892191
16.06.2010	174	125	211	194	81	184	224,0579223
16.06.2010	175	52	150	194	81	184	102,8251239
16.06.2010	176	37	136	194	81	184	61,85903024
16.06.2010	177	13	97	194	81	184	84,24788013
16.06.2010	178	26	122	194	81	184	78,10925878
16.06.2010	179	54	153	194	81	184	153,4582715
16.06.2010	180	67	166	194	81	184	38,93574435
16.06.2010	181	60	156	194	81	184	23,2126334
16.06.2010	182	13	96	194	81	184	112,8408729
16.06.2010	191	128	203	194	81	184	112,1797928
16.06.2010	193	171	232	194	81	184	11,3364364
16.06.2010	170	49	148	172	19	106	11,86511532
16.06.2010	170	49	148	173	16	105	127,2376602
16.06.2010	172	19	106	173	16	105	120,835674
16.06.2010	170	49	148	174	125	211	238,7048697
16.06.2010	172	19	106	174	125	211	246,4680999
16.06.2010	173	16	105	174	125	211	365,6434397
16.06.2010	170	49	148	175	52	150	60,20517129
16.06.2010	172	19	106	175	52	150	51,59328989
16.06.2010	173	16	105	175	52	150	71,13795461

16.06.2010	174	125	211	175	52	150	298,0610847
16.06.2010	170	49	148	176	37	136	22,75241024
16.06.2010	172	19	106	176	37	136	11,49468577
16.06.2010	173	16	105	176	37	136	111,4294698
16.06.2010	174	125	211	176	37	136	257,1703428
16.06.2010	175	52	150	176	37	136	41,12657079
16.06.2010	170	49	148	177	13	97	44,42916076
16.06.2010	172	19	106	177	13	97	34,24895646
16.06.2010	173	16	105	177	13	97	90,62593322
16.06.2010	174	125	211	177	13	97	280,0145674
16.06.2010	175	52	150	177	13	97	19,49169411
16.06.2010	176	37	136	177	13	97	22,98937154
16.06.2010	170	49	148	178	26	122	33,56499655
16.06.2010	172	19	106	178	26	122	23,7458315
16.06.2010	173	16	105	178	26	122	98,55272629
16.06.2010	174	125	211	178	26	122	270,0718264
16.06.2010	175	52	150	178	26	122	28,11293396
16.06.2010	176	37	136	178	26	122	13,01869162
16.06.2010	177	13	97	178	26	122	10,89686176
16.06.2010	170	49	148	179	54	153	111,0156984
16.06.2010	172	19	106	179	54	153	103,8953743
16.06.2010	173	16	105	179	54	153	18,64835948
16.06.2010	174	125	211	179	54	153	349,7184126
16.06.2010	175	52	150	179	54	153	53,25882493
16.06.2010	176	37	136	179	54	153	94,06117397
16.06.2010	177	13	97	179	54	153	72,66702428
16.06.2010	178	26	122	179	54	153	81,08237586
16.06.2010	170	49	148	180	67	166	9,076157983
16.06.2010	172	19	106	180	67	166	20,4173743
16.06.2010	173	16	105	180	67	166	135,2369848
16.06.2010	174	125	211	180	67	166	230,4616778
16.06.2010	175	52	150	180	67	166	69,06286942
16.06.2010	176	37	136	180	67	166	31,66117206

16.06.2010	177	13	97	180	67	166	53,50468162
16.06.2010	178	26	122	180	67	166	42,63667821
16.06.2010	179	54	153	180	67	166	119,3211941
16.06.2010	170	49	148	181	60	156	25,10449202
16.06.2010	172	19	106	181	60	156	36,84632946
16.06.2010	173	16	105	181	60	156	146,0637854
16.06.2010	174	125	211	181	60	156	220,1167136
16.06.2010	175	52	150	181	60	156	82,99809399
16.06.2010	176	37	136	181	60	156	47,84222406
16.06.2010	177	13	97	181	60	156	68,85525995
16.06.2010	178	26	122	181	60	156	57,9680831
16.06.2010	179	54	153	181	60	156	131,0999972
16.06.2010	180	67	166	181	60	156	16,56199781
16.06.2010	170	49	148	182	13	96	72,15202592
16.06.2010	172	19	106	182	13	96	62,95511315
16.06.2010	173	16	105	182	13	96	63,48901777
16.06.2010	174	125	211	182	13	96	309,1836319
16.06.2010	175	52	150	182	13	96	12,54306783
16.06.2010	176	37	136	182	13	96	52,0212287
16.06.2010	177	13	97	182	13	96	29,22973949
16.06.2010	178	26	122	182	13	96	39,21452081
16.06.2010	179	54	153	182	13	96	44,97311919
16.06.2010	180	67	166	182	13	96	81,10114449
16.06.2010	181	60	156	182	13	96	95,34324778
16.06.2010	170	49	148	191	128	203	71,82642989
16.06.2010	172	19	106	191	128	203	62,70914579
16.06.2010	173	16	105	191	128	203	63,29281
16.06.2010	174	125	211	191	128	203	308,9915186
16.06.2010	175	52	150	191	128	203	12,04868331
16.06.2010	176	37	136	191	128	203	51,82169062
16.06.2010	177	13	97	191	128	203	29,09205655
16.06.2010	178	26	122	191	128	203	38,97863803
16.06.2010	179	54	153	191	128	203	44,8095365

16.06.2010	180	67	166	191	128	203	80,76157943
16.06.2010	181	60	156	191	128	203	94,93881066
16.06.2010	182	13	96	191	128	203	0,760316849
16.06.2010	170	49	148	193	171	232	32,38132019
16.06.2010	172	19	106	193	171	232	44,14249532
16.06.2010	173	16	105	193	171	232	151,4648084
16.06.2010	174	125	211	193	171	232	215,4543888
16.06.2010	175	52	150	193	171	232	89,61418465
16.06.2010	176	37	136	193	171	232	55,10084581
16.06.2010	177	13	97	193	171	232	75,90333247
16.06.2010	178	26	122	193	171	232	65,03306843
16.06.2010	179	54	153	193	171	232	136,8789759
16.06.2010	180	67	166	193	171	232	23,85266916
16.06.2010	181	60	156	193	171	232	7,29951198
16.06.2010	182	13	96	193	171	232	102,0252274
16.06.2010	191	128	203	193	171	232	101,6014285
16.06.2010	170	49	148	194	81	184	46,57890998
16.06.2010	172	19	106	194	81	184	58,16190948
16.06.2010	173	16	105	194	81	184	165,2211549
16.06.2010	174	125	211	194	81	184	202,9815938
16.06.2010	175	52	150	194	81	184	104,2126507
16.06.2010	176	37	136	194	81	184	69,32837984
16.06.2010	177	13	97	194	81	184	90,44984772
16.06.2010	178	26	122	194	81	184	79,56682674
16.06.2010	179	54	153	194	81	184	150,9810561
16.06.2010	180	67	166	194	81	184	37,74412733
16.06.2010	181	60	156	194	81	184	21,60515948
16.06.2010	182	13	96	194	81	184	116,6352439
16.06.2010	191	128	203	194	81	184	116,2079854
16.06.2010	193	171	232	194	81	184	14,62004488
16.06.2010	170	49	148	172	19	106	8,313657108
16.06.2010	170	49	148	173	16	105	118,8403676
16.06.2010	172	19	106	173	16	105	112,6278279

16.06.2010	170	49	148	174	125	211	243,5666896
16.06.2010	172	19	106	174	125	211	249,0340011
16.06.2010	173	16	105	174	125	211	361,351706
16.06.2010	170	49	148	175	52	150	71,04628527
16.06.2010	172	19	106	175	52	150	63,91284551
16.06.2010	173	16	105	175	52	150	51,02502885
16.06.2010	174	125	211	175	52	150	311,1181277
16.06.2010	170	49	148	176	37	136	33,63462267
16.06.2010	172	19	106	176	37	136	25,39140982
16.06.2010	173	16	105	176	37	136	92,80538457
16.06.2010	174	125	211	176	37	136	269,0943358
16.06.2010	175	52	150	176	37	136	42,05499885
16.06.2010	170	49	148	177	13	97	57,30935531
16.06.2010	172	19	106	177	13	97	49,57789127
16.06.2010	173	16	105	177	13	97	68,06534616
16.06.2010	174	125	211	177	13	97	294,7482045
16.06.2010	175	52	150	177	13	97	17,09762249
16.06.2010	176	37	136	177	13	97	25,83014942
16.06.2010	170	49	148	178	26	122	47,90486406
16.06.2010	172	19	106	178	26	122	39,87950659
16.06.2010	173	16	105	178	26	122	78,92831867
16.06.2010	174	125	211	178	26	122	283,7927198
16.06.2010	175	52	150	178	26	122	27,90347297
16.06.2010	176	37	136	178	26	122	15,15874269
16.06.2010	177	13	97	178	26	122	10,98355256
16.06.2010	170	49	148	179	54	153	109,0132039
16.06.2010	172	19	106	179	54	153	102,7807972
16.06.2010	173	16	105	179	54	153	9,848414693
16.06.2010	174	125	211	179	54	153	351,5146183
16.06.2010	175	52	150	179	54	153	41,44478791
16.06.2010	176	37	136	179	54	153	83,06581187
16.06.2010	177	13	97	179	54	153	58,53012424
16.06.2010	178	26	122	179	54	153	69,33118094

16.06.2010	170	49	148	180	67	166	16,79756143
16.06.2010	172	19	106	180	67	166	8,485179464
16.06.2010	173	16	105	180	67	166	106,7421714
16.06.2010	174	125	211	180	67	166	254,612169
16.06.2010	175	52	150	180	67	166	57,0984792
16.06.2010	176	37	136	180	67	166	17,10132013
16.06.2010	177	13	97	180	67	166	42,02233977
16.06.2010	178	26	122	180	67	166	31,92340615
16.06.2010	179	54	153	180	67	166	96,90307401
16.06.2010	170	49	148	181	60	156	22,12688588
16.06.2010	172	19	106	181	60	156	30,17413949
16.06.2010	173	16	105	181	60	156	139,4431193
16.06.2010	174	125	211	181	60	156	225,2360667
16.06.2010	175	52	150	181	60	156	92,79769537
16.06.2010	176	37	136	181	60	156	55,53469236
16.06.2010	177	13	97	181	60	156	79,41507438
16.06.2010	178	26	122	181	60	156	70,00723442
16.06.2010	179	54	153	181	60	156	129,6779467
16.06.2010	180	67	166	181	60	156	38,46533599
16.06.2010	170	49	148	182	13	96	81,77482969
16.06.2010	172	19	106	182	13	96	74,51735456
16.06.2010	173	16	105	182	13	96	42,87813761
16.06.2010	174	125	211	182	13	96	320,9047167
16.06.2010	175	52	150	182	13	96	10,88773993
16.06.2010	176	37	136	182	13	96	51,88561933
16.06.2010	177	13	97	182	13	96	26,15920918
16.06.2010	178	26	122	182	13	96	37,13094147
16.06.2010	179	54	153	182	13	96	33,86105252
16.06.2010	180	67	166	182	13	96	67,48829281
16.06.2010	181	60	156	182	13	96	103,5982277
16.06.2010	170	49	148	191	128	203	83,35303973
16.06.2010	172	19	106	191	128	203	76,03132848
16.06.2010	173	16	105	191	128	203	42,4153574

16.06.2010	174	125	211	191	128	203	322,0639428
16.06.2010	175	52	150	191	128	203	12,67060101
16.06.2010	176	37	136	191	128	203	53,14286407
16.06.2010	177	13	97	191	128	203	27,34152537
16.06.2010	178	26	122	191	128	203	38,27276916
16.06.2010	179	54	153	191	128	203	33,61555032
16.06.2010	180	67	166	191	128	203	68,91380754
16.06.2010	181	60	156	191	128	203	105,2215924
16.06.2010	182	13	96	191	128	203	1,998093294
16.06.2010	170	49	148	193	171	232	17,49709864
16.06.2010	172	19	106	193	171	232	25,75836336
16.06.2010	173	16	105	193	171	232	133,9345796
16.06.2010	174	125	211	193	171	232	230,8116228
16.06.2010	175	52	150	193	171	232	87,57991234
16.06.2010	176	37	136	193	171	232	51,12883487
16.06.2010	177	13	97	193	171	232	74,50620206
16.06.2010	178	26	122	193	171	232	65,33355996
16.06.2010	179	54	153	193	171	232	124,1798564
16.06.2010	180	67	166	193	171	232	34,19867871
16.06.2010	181	60	156	193	171	232	5,601695979
16.06.2010	182	13	96	193	171	232	98,40781003
16.06.2010	191	128	203	193	171	232	100,0553123
16.06.2010	170	49	148	194	81	184	34,50343494
16.06.2010	172	19	106	194	81	184	42,81552714
16.06.2010	173	16	105	194	81	184	147,2415034
16.06.2010	174	125	211	194	81	184	222,1582252
16.06.2010	175	52	150	194	81	184	102,8120343
16.06.2010	176	37	136	194	81	184	68,06647414
16.06.2010	177	13	97	194	81	184	90,66091002
16.06.2010	178	26	122	194	81	184	81,92722753
16.06.2010	179	54	153	194	81	184	137,6268008
16.06.2010	180	67	166	194	81	184	51,2941882
16.06.2010	181	60	156	194	81	184	14,52521116

16.06.2010	182	13	96	194	81	184	113,6963698
16.06.2010	191	128	203	194	81	184	115,4230094
16.06.2010	193	171	232	194	81	184	17,24098089
21.06.2010	170	49	148	172	19	106	17,15984765
21.06.2010	170	49	148	173	16	105	114,9242244
21.06.2010	172	19	106	173	16	105	101,7298545
21.06.2010	170	49	148	174	125	211	231,9274663
21.06.2010	172	19	106	174	125	211	245,7264041
21.06.2010	173	16	105	174	125	211	346,8443569
21.06.2010	170	49	148	175	52	150	69,66027107
21.06.2010	172	19	106	175	52	150	54,69230001
21.06.2010	173	16	105	175	52	150	48,57704748
21.06.2010	174	125	211	175	52	150	300,3952238
21.06.2010	170	49	148	176	37	136	8,991168677
21.06.2010	172	19	106	176	37	136	8,185553326
21.06.2010	173	16	105	176	37	136	107,6304552
21.06.2010	174	125	211	176	37	136	239,3269388
21.06.2010	175	52	150	176	37	136	61,49422391
21.06.2010	170	49	148	177	37	136	41,0213444
21.06.2010	172	19	106	177	13	97	26,09542532
21.06.2010	173	16	105	177	13	97	75,76284148
21.06.2010	174	125	211	177	13	97	271,7981199
21.06.2010	175	52	150	177	13	97	28,74519982
21.06.2010	176	37	136	177	13	97	32,75090199
21.06.2010	170	49	148	178	26	122	33,45421134
21.06.2010	172	19	106	178	26	122	17,23023762
21.06.2010	173	16	105	178	26	122	85,29443277
21.06.2010	174	125	211	178	26	122	262,7805752
21.06.2010	175	52	150	178	26	122	37,62968356
21.06.2010	176	37	136	178	26	122	24,68579446
21.06.2010	177	13	97	178	26	122	9,759507894
21.06.2010	170	49	148	179	54	153	107,2847301
21.06.2010	172	19	106	179	54	153	94,28398363

21.06.2010	173	16	105	179	54	153	7,770032731
21.06.2010	174	125	211	179	54	153	339,2121316
21.06.2010	175	52	150	179	54	153	41,76663605
21.06.2010	176	37	136	179	54	153	100,0704322
21.06.2010	177	13	97	179	54	153	68,3996024
21.06.2010	178	26	122	179	54	153	77,99625493
21.06.2010	170	49	148	180	67	166	1,681422274
21.06.2010	172	19	106	180	67	166	18,74064398
21.06.2010	173	16	105	180	67	166	116,5378967
21.06.2010	174	125	211	180	67	166	230,3218921
21.06.2010	175	52	150	180	67	166	71,33372995
21.06.2010	176	37	136	180	67	166	10,60021851
21.06.2010	177	13	97	180	67	166	42,69976247
21.06.2010	178	26	122	180	67	166	35,12083167
21.06.2010	179	54	153	180	67	166	108,8924141
21.06.2010	170	49	148	181	60	156	16,03608543
21.06.2010	172	19	106	181	60	156	32,98216675
21.06.2010	173	16	105	181	60	156	129,7486606
21.06.2010	174	125	211	181	60	156	217,4598401
21.06.2010	175	52	150	181	60	156	85,38265329
21.06.2010	176	37	136	181	60	156	24,92974195
21.06.2010	177	13	97	181	60	156	56,88436024
21.06.2010	178	26	122	181	60	156	49,49018097
21.06.2010	179	54	153	181	60	156	122,045398
21.06.2010	180	67	166	181	60	156	14,37362111
21.06.2010	170	49	148	191	128	203	73,48288782
21.06.2010	172	19	106	191	128	203	58,75108308
21.06.2010	173	16	105	191	128	203	44,25805781
21.06.2010	174	125	211	191	128	203	304,4765217
21.06.2010	175	52	150	191	128	203	4,319861133
21.06.2010	176	37	136	191	128	203	65,42633064
21.06.2010	177	13	97	191	128	203	32,71696165
21.06.2010	178	26	122	191	128	203	41,76436524

21.06.2010	179	54	153	191	128	203	37,45766372
21.06.2010	180	67	166	191	128	203	75,15181358
21.06.2010	181	60	156	191	128	203	89,11624209
21.06.2010	170	49	148	193	171	232	33,66361342
21.06.2010	172	19	106	193	171	232	50,80166992
21.06.2010	173	16	105	193	171	232	142,2181252
21.06.2010	174	125	211	193	171	232	207,8165678
21.06.2010	175	52	150	193	171	232	100,4035752
21.06.2010	176	37	136	193	171	232	42,61591198
21.06.2010	177	13	97	193	171	232	72,71256603
21.06.2010	178	26	122	193	171	232	66,38201834
21.06.2010	179	54	153	193	171	232	134,4503108
21.06.2010	180	67	166	193	171	232	32,13997233
21.06.2010	181	60	156	193	171	232	18,8585977
21.06.2010	191	128	203	193	171	232	103,8426326
21.06.2010	170	49	148	194	81	184	53,02153919
21.06.2010	172	19	106	194	81	184	70,18132771
21.06.2010	173	16	105	194	81	184	160,3553906
21.06.2010	174	125	211	194	81	184	192,9861754
21.06.2010	175	52	150	194	81	184	119,5993296
21.06.2010	176	37	136	194	81	184	62,0034923
21.06.2010	177	13	97	194	81	184	92,13118042
21.06.2010	178	26	122	194	81	184	85,88194463
21.06.2010	179	54	153	194	81	184	152,5878561
21.06.2010	180	67	166	194	81	184	51,45786925
21.06.2010	181	60	156	194	81	184	37,60591236
21.06.2010	191	128	203	194	81	184	122,9505459
21.06.2010	193	171	232	194	81	184	19,50003359
21.06.2010	170	49	148	172	19	106	2,491053162
21.06.2010	170	49	148	173	16	105	112,8052466
21.06.2010	172	19	106	173	16	105	114,0224183
21.06.2010	170	49	148	174	125	211	251,4781381
21.06.2010	172	19	106	174	125	211	250,2605603

21.06.2010	173	16	105	174	125	211	364,2756522
21.06.2010	170	49	148	175	52	150	59,48705007
21.06.2010	172	19	106	175	52	150	60,97122212
21.06.2010	173	16	105	175	52	150	54,22390589
21.06.2010	174	125	211	175	52	150	310,6928889
21.06.2010	170	49	148	176	37	136	9,442939364
21.06.2010	172	19	106	176	37	136	11,49939961
21.06.2010	173	16	105	176	37	136	104,1678307
21.06.2010	174	125	211	176	37	136	260,2551982
21.06.2010	175	52	150	176	37	136	50,47381597
21.06.2010	170	49	148	177	13	97	41,61584565
21.06.2010	172	19	106	177	13	97	43,34243698
21.06.2010	173	16	105	177	13	97	73,10708189
21.06.2010	174	125	211	177	13	97	292,1582108
21.06.2010	175	52	150	177	13	97	18,88587873
21.06.2010	176	37	136	177	13	97	32,31961511
21.06.2010	170	49	148	178	26	122	28,0081574
21.06.2010	172	19	106	178	26	122	29,96250764
21.06.2010	173	16	105	178	26	122	87,33276231
21.06.2010	174	125	211	178	26	122	277,8685041
21.06.2010	175	52	150	178	26	122	33,124541
21.06.2010	176	37	136	178	26	122	18,56899769
21.06.2010	177	13	97	178	26	122	14,31726795
21.06.2010	170	49	148	179	54	153	98,07700552
21.06.2010	172	19	106	179	54	153	99,308794
21.06.2010	173	16	105	179	54	153	14,73910785
21.06.2010	174	125	211	179	54	153	349,5524412
21.06.2010	175	52	150	179	54	153	39,57917521
21.06.2010	176	37	136	179	54	153	89,42878482
21.06.2010	177	13	97	179	54	153	58,46380507
21.06.2010	178	26	122	179	54	153	72,65285267
21.06.2010	170	49	148	180	67	166	32,04230404
21.06.2010	172	19	106	180	67	166	29,61044304

21.06.2010	173	16	105	180	67	166	135,9231513
21.06.2010	174	125	211	180	67	166	231,4452678
21.06.2010	175	52	150	180	67	166	85,93756354
21.06.2010	176	37	136	180	67	166	40,72045096
21.06.2010	177	13	97	180	67	166	70,25902114
21.06.2010	178	26	122	180	67	166	58,30213137
21.06.2010	179	54	153	180	67	166	121,564387
21.06.2010	170	49	148	181	60	156	32,6499434
21.06.2010	172	19	106	181	60	156	30,24918218
21.06.2010	173	16	105	181	60	156	137,3810574
21.06.2010	174	125	211	181	60	156	229,8044901
21.06.2010	175	52	150	181	60	156	87,19908461
21.06.2010	176	37	136	181	60	156	41,48018824
21.06.2010	177	13	97	181	60	156	71,35632867
21.06.2010	178	26	122	181	60	156	59,23438279
21.06.2010	179	54	153	181	60	156	122,9991699
21.06.2010	180	67	166	181	60	156	1,71071291
21.06.2010	170	49	148	191	128	203	72,37152118
21.06.2010	172	19	106	191	128	203	74,04231898
21.06.2010	173	16	105	191	128	203	45,0963682
21.06.2010	174	125	211	191	128	203	322,6436877
21.06.2010	175	52	150	191	128	203	14,48473441
21.06.2010	176	37	136	191	128	203	63,09849727
21.06.2010	177	13	97	191	128	203	30,77933614
21.06.2010	178	26	122	191	128	203	44,88969205
21.06.2010	179	54	153	191	128	203	31,4800673
21.06.2010	180	67	166	191	128	203	99,95952941
21.06.2010	181	60	156	191	128	203	101,1739796
21.06.2010	170	49	148	193	171	232	16,63491776
21.06.2010	172	19	106	193	171	232	14,35695859
21.06.2010	173	16	105	193	171	232	126,1349955
21.06.2010	174	125	211	193	171	232	238,6639425
21.06.2010	175	52	150	193	171	232	74,0838454

21.06.2010	176	37	136	193	171	232	25,83329643
21.06.2010	177	13	97	193	171	232	57,10346203
21.06.2010	178	26	122	193	171	232	44,14157451
21.06.2010	179	54	153	193	171	232	111,5141458
21.06.2010	180	67	166	193	171	232	15,92473612
21.06.2010	181	60	156	193	171	232	16,27815113
21.06.2010	191	128	203	193	171	232	87,57248633
21.06.2010	170	49	148	194	81	184	68,17003527
21.06.2010	172	19	106	194	81	184	65,88623691
21.06.2010	173	16	105	194	81	184	171,6556577
21.06.2010	174	125	211	194	81	184	201,5735154
21.06.2010	175	52	150	194	81	184	122,9620058
21.06.2010	176	37	136	194	81	184	77,31844336
21.06.2010	177	13	97	194	81	184	107,4889122
21.06.2010	178	26	122	194	81	184	95,33348938
21.06.2010	179	54	153	194	81	184	157,5591
21.06.2010	180	67	166	194	81	184	37,23103029
21.06.2010	181	60	156	194	81	184	36,15716832
21.06.2010	191	128	203	194	81	184	137,087969
21.06.2010	193	171	232	194	81	184	51,53881382
21.06.2010	170	49	148	172	19	106	5,606531281
21.06.2010	170	49	148	173	16	105	121,5047819
21.06.2010	172	19	106	173	16	105	118,4806972
21.06.2010	170	49	148	174	125	211	235,1100448
21.06.2010	172	19	106	174	125	211	238,7331721
21.06.2010	173	16	105	174	125	211	356,1792307
21.06.2010	170	49	148	175	52	150	59,06786067
21.06.2010	172	19	106	175	52	150	55,31468371
21.06.2010	173	16	105	175	52	150	64,26785893
21.06.2010	174	125	211	175	52	150	294,0425733
21.06.2010	170	49	148	176	37	136	17,62055827
21.06.2010	172	19	106	176	37	136	12,51350803
21.06.2010	173	16	105	176	37	136	107,7403784

21.06.2010	174	125	211	176	37	136	250,5271779
21.06.2010	175	52	150	176	37	136	43,85127837
21.06.2010	170	49	148	177	13	97	45,02952065
21.06.2010	172	19	106	177	13	97	40,67761757
21.06.2010	173	16	105	177	13	97	80,45830998
21.06.2010	174	125	211	177	13	97	279,0960006
21.06.2010	175	52	150	177	13	97	16,26927052
21.06.2010	176	37	136	177	13	97	28,57627764
21.06.2010	170	49	148	178	26	122	31,01612849
21.06.2010	172	19	106	178	26	122	26,25863654
21.06.2010	173	16	105	178	26	122	94,96677785
21.06.2010	174	125	211	178	26	122	264,2699082
21.06.2010	175	52	150	178	26	122	30,723528
21.06.2010	176	37	136	178	26	122	13,86862012
21.06.2010	177	13	97	178	26	122	14,88303238
21.06.2010	170	49	148	179	54	153	108,8919164
21.06.2010	172	19	106	179	54	153	105,5993099
21.06.2010	173	16	105	179	54	153	14,34531479
21.06.2010	174	125	211	179	54	153	343,9270491
21.06.2010	175	52	150	179	54	153	50,75310736
21.06.2010	176	37	136	179	54	153	94,51687632
21.06.2010	177	13	97	179	54	153	66,78985658
21.06.2010	178	26	122	179	54	153	81,46441123
21.06.2010	170	49	148	180	67	166	10,38109225
21.06.2010	172	19	106	180	67	166	15,95873189
21.06.2010	173	16	105	180	67	166	126,4790777
21.06.2010	174	125	211	180	67	166	229,702372
21.06.2010	175	52	150	180	67	166	65,81593937
21.06.2010	176	37	136	180	67	166	27,25201667
21.06.2010	177	13	97	180	67	166	52,98626381
21.06.2010	178	26	122	180	67	166	39,85571747
21.06.2010	179	54	153	180	67	166	114,4225547
21.06.2010	170	49	148	181	60	156	25,27714072

21.06.2010	172	19	106	181	60	156	30,76759547
21.06.2010	173	16	105	181	60	156	140,9609739
21.06.2010	174	125	211	181	60	156	215,5656241
21.06.2010	175	52	150	181	60	156	81,43513445
21.06.2010	176	37	136	181	60	156	42,8119406
21.06.2010	177	13	97	181	60	156	68,90705711
21.06.2010	178	26	122	181	60	156	55,73860209
21.06.2010	179	54	153	181	60	156	129,2964853
21.06.2010	180	67	166	181	60	156	15,93890983
21.06.2010	170	49	148	191	128	203	66,16729454
21.06.2010	172	19	106	191	128	203	62,15946264
21.06.2010	173	16	105	191	128	203	59,26784443
21.06.2010	174	125	211	191	128	203	300,8213574
21.06.2010	175	52	150	191	128	203	7,949311729
21.06.2010	176	37	136	191	128	203	50,32111557
21.06.2010	177	13	97	191	128	203	21,86324117
21.06.2010	178	26	122	191	128	203	36,74175121
21.06.2010	179	54	153	191	128	203	45,30608898
21.06.2010	180	67	166	191	128	203	73,28527382
21.06.2010	181	60	156	191	128	203	89,00224426
21.06.2010	170	49	148	193	171	232	17,04206162
21.06.2010	172	19	106	193	171	232	22,63619173
21.06.2010	173	16	105	193	171	232	132,7232778
21.06.2010	174	125	211	193	171	232	223,5661644
21.06.2010	175	52	150	193	171	232	72,71778319
21.06.2010	176	37	136	193	171	232	34,33298371
21.06.2010	177	13	97	193	171	232	60,12455169
21.06.2010	178	26	122	193	171	232	47,04642897
21.06.2010	179	54	153	193	171	232	120,8832059
21.06.2010	180	67	166	193	171	232	7,192934943
21.06.2010	181	60	156	193	171	232	8,784868146
21.06.2010	191	128	203	193	171	232	80,2555312
21.06.2010	170	49	148	194	81	184	50,70895161

21.06.2010	172	19	106	194	81	184	56,11507286
21.06.2010	173	16	105	194	81	184	163,6101781
21.06.2010	174	125	211	194	81	184	195,1830776
21.06.2010	175	52	150	194	81	184	106,1244772
21.06.2010	176	37	136	194	81	184	68,29658776
21.06.2010	177	13	97	194	81	184	94,20318853
21.06.2010	178	26	122	194	81	184	81,23846326
21.06.2010	179	54	153	194	81	184	152,6111773
21.06.2010	180	67	166	194	81	184	41,39190343
21.06.2010	181	60	156	194	81	184	25,5150811
21.06.2010	191	128	203	194	81	184	113,8311862
21.06.2010	193	171	232	194	81	184	34,19999928
21.06.2010	170	49	148	172	19	106	22,63299926
21.06.2010	170	49	148	173	16	105	121,1870883
21.06.2010	172	19	106	173	16	105	102,589609
21.06.2010	170	49	148	174	125	211	245,396275
21.06.2010	172	19	106	174	125	211	265,5615442
21.06.2010	173	16	105	174	125	211	366,4417383
21.06.2010	170	49	148	175	52	150	69,88675251
21.06.2010	172	19	106	175	52	150	48,33808171
21.06.2010	173	16	105	175	52	150	57,96356417
21.06.2010	174	125	211	175	52	150	313,7823812
21.06.2010	170	49	148	176	37	136	29,88870955
21.06.2010	172	19	106	176	37	136	7,784550199
21.06.2010	173	16	105	176	37	136	95,04226717
21.06.2010	174	125	211	176	37	136	273,3441677
21.06.2010	175	52	150	176	37	136	40,56632855
21.06.2010	170	49	148	177	13	97	53,74907765
21.06.2010	172	19	106	177	13	97	32,84330079
21.06.2010	173	16	105	177	13	97	70,34138007
21.06.2010	174	125	211	177	13	97	298,3562538
21.06.2010	175	52	150	177	13	97	16,37965633
21.06.2010	176	37	136	177	13	97	25,09171597

21.06.2010	170	49	148	178	26	122	47,33745233
21.06.2010	172	19	106	178	26	122	25,48195992
21.06.2010	173	16	105	178	26	122	78,66736549
21.06.2010	174	125	211	178	26	122	290,9473898
21.06.2010	175	52	150	178	26	122	22,86231012
21.06.2010	176	37	136	178	26	122	17,72278433
21.06.2010	177	13	97	178	26	122	8,522355743
21.06.2010	170	49	148	179	54	153	102,7150848
21.06.2010	172	19	106	179	54	153	83,13950449
21.06.2010	173	16	105	179	54	153	20,6815442
21.06.2010	174	125	211	179	54	153	348,0900145
21.06.2010	175	52	150	179	54	153	37,35914921
21.06.2010	176	37	136	179	54	153	75,46713264
21.06.2010	177	13	97	179	54	153	50,47524758
21.06.2010	178	26	122	179	54	153	58,6165583
21.06.2010	170	49	148	180	67	166	16,03439555
21.06.2010	172	19	106	180	67	166	38,28362381
21.06.2010	173	16	105	180	67	166	132,4595736
21.06.2010	174	125	211	180	67	166	234,2462362
21.06.2010	175	52	150	180	67	166	84,02474096
21.06.2010	176	37	136	180	67	166	45,1137904
21.06.2010	177	13	97	180	67	166	67,67247085
21.06.2010	178	26	122	180	67	166	62,01856811
21.06.2010	179	54	153	180	67	166	114,9309049
21.06.2010	170	49	148	181	60	156	28,42303978
21.06.2010	172	19	106	181	60	156	50,45081505
21.06.2010	173	16	105	181	60	156	141,359266
21.06.2010	174	125	211	181	60	156	226,5603878
21.06.2010	175	52	150	181	60	156	95,09575791
21.06.2010	176	37	136	181	60	156	57,03936386
21.06.2010	177	13	97	181	60	156	78,71706652
21.06.2010	178	26	122	181	60	156	73,54223428
21.06.2010	179	54	153	181	60	156	124,5718019

21.06.2010	180	67	166	181	60	156	12,39966411
21.06.2010	170	49	148	182	13	96	102,1370709
21.06.2010	172	19	106	182	13	96	82,53619368
21.06.2010	173	16	105	182	13	96	21,30371653
21.06.2010	174	125	211	182	13	96	347,5077679
21.06.2010	175	52	150	182	13	96	36,73302292
21.06.2010	176	37	136	182	13	96	74,86117203
21.06.2010	177	13	97	182	13	96	49,8640127
21.06.2010	178	26	122	182	13	96	57,99962785
21.06.2010	179	54	153	182	13	96	0,630421427
21.06.2010	180	67	166	182	13	96	114,3784977
21.06.2010	181	60	156	182	13	96	124,0397942
21.06.2010	170	49	148	191	128	203	74,58048018
21.06.2010	172	19	106	191	128	203	53,92892624
21.06.2010	173	16	105	191	128	203	50,20205747
21.06.2010	174	125	211	191	128	203	319,4368254
21.06.2010	175	52	150	191	128	203	8,469730058
21.06.2010	176	37	136	191	128	203	46,16773253
21.06.2010	177	13	97	191	128	203	21,08872965
21.06.2010	178	26	122	191	128	203	28,91500945
21.06.2010	179	54	153	191	128	203	29,82563318
21.06.2010	180	67	166	191	128	203	87,95934798
21.06.2010	181	60	156	191	128	203	98,52527278
21.06.2010	182	13	96	191	128	203	29,20299716
21.06.2010	170	49	148	193	171	232	21,22513376
21.06.2010	172	19	106	193	171	232	43,36264699
21.06.2010	173	16	105	193	171	232	136,1044808
21.06.2010	174	125	211	193	171	232	230,9958693
21.06.2010	175	52	150	193	171	232	88,61040495
21.06.2010	176	37	136	193	171	232	50,07730549
21.06.2010	177	13	97	193	171	232	72,23622793
21.06.2010	178	26	122	193	171	232	66,80087757
21.06.2010	179	54	153	193	171	232	118,8927239

21.06.2010	180	67	166	193	171	232	5,198588267
21.06.2010	181	60	156	193	171	232	7,201719827
21.06.2010	182	13	96	193	171	232	118,3490784
21.06.2010	191	128	203	193	171	232	92,31858906
21.06.2010	170	49	148	194	81	184	32,98244061
21.06.2010	172	19	106	194	81	184	54,9045211
21.06.2010	173	16	105	194	81	184	144,5245888
21.06.2010	174	125	211	194	81	184	224,134827
21.06.2010	175	52	150	194	81	184	99,10466546
21.06.2010	176	37	136	194	81	184	61,39838076
21.06.2010	177	13	97	194	81	184	82,73567543
21.06.2010	178	26	122	194	81	184	77,73844738
21.06.2010	179	54	153	194	81	184	128,026569
21.06.2010	180	67	166	194	81	184	16,97248781
21.06.2010	181	60	156	194	81	184	4,584614453
21.06.2010	182	13	96	194	81	184	127,5026008
21.06.2010	191	128	203	194	81	184	102,3464922
21.06.2010	193	171	232	194	81	184	11,77571018
21.06.2010	170	49	148	172	19	106	14,49720083
21.06.2010	170	49	148	173	16	105	125,39229
21.06.2010	172	19	106	173	16	105	113,9824858
21.06.2010	170	49	148	174	125	211	224,4397474
21.06.2010	172	19	106	174	125	211	233,7625806
21.06.2010	173	16	105	174	125	211	346,9378221
21.06.2010	170	49	148	175	52	150	77,6607347
21.06.2010	172	19	106	175	52	150	64,13983546
21.06.2010	173	16	105	175	52	150	55,82863004
21.06.2010	174	125	211	175	52	150	293,0602928
21.06.2010	170	49	148	176	37	136	39,02032717
21.06.2010	172	19	106	176	37	136	24,5239018
21.06.2010	173	16	105	176	37	136	96,78186925
21.06.2010	174	125	211	176	37	136	250,4749301
21.06.2010	175	52	150	176	37	136	42,90361939

21.06.2010	170	49	148	177	13	97	62,17442799
21.06.2010	172	19	106	177	13	97	48,17092537
21.06.2010	173	16	105	177	13	97	72,2459805
21.06.2010	174	125	211	177	13	97	275,9763774
21.06.2010	175	52	150	177	13	97	17,09392405
21.06.2010	176	37	136	177	13	97	25,89929535
21.06.2010	170	49	148	178	26	122	47,88977757
21.06.2010	172	19	106	178	26	122	33,45097123
21.06.2010	173	16	105	178	26	122	88,3123546
21.06.2010	174	125	211	178	26	122	259,4278355
21.06.2010	175	52	150	178	26	122	33,67716071
21.06.2010	176	37	136	178	26	122	9,542847293
21.06.2010	177	13	97	178	26	122	16,58761071
21.06.2010	170	49	148	179	54	153	99,53256147
21.06.2010	172	19	106	179	54	153	86,25544585
21.06.2010	173	16	105	179	54	153	37,63688399
21.06.2010	174	125	211	179	54	153	314,9403188
21.06.2010	175	52	150	179	54	153	22,29382836
21.06.2010	176	37	136	179	54	153	65,14131355
21.06.2010	177	13	97	179	54	153	39,25285307
21.06.2010	178	26	122	179	54	153	55,81689902
21.06.2010	170	49	148	180	67	166	25,47912402
21.06.2010	172	19	106	180	67	166	11,15653254
21.06.2010	173	16	105	180	67	166	107,7621736
21.06.2010	174	125	211	180	67	166	239,1910313
21.06.2010	175	52	150	180	67	166	55,6146569
21.06.2010	176	37	136	180	67	166	13,8718762
21.06.2010	177	13	97	180	67	166	38,97759524
21.06.2010	178	26	122	180	67	166	23,24587945
21.06.2010	179	54	153	180	67	166	77,90303234
21.06.2010	170	49	148	181	60	156	16,64523129
21.06.2010	172	19	106	181	60	156	30,38890476
21.06.2010	173	16	105	181	60	156	141,7824339

21.06.2010	174	125	211	181	60	156	209,6152969
21.06.2010	175	52	150	181	60	156	94,2258143
21.06.2010	176	37	136	181	60	156	54,49972439
21.06.2010	177	13	97	181	60	156	78,52422792
21.06.2010	178	26	122	181	60	156	63,70731013
21.06.2010	179	54	153	181	60	156	116,1528248
21.06.2010	180	67	166	181	60	156	40,645074
21.06.2010	170	49	148	182	13	96	99,42723637
21.06.2010	172	19	106	182	13	96	86,16817133
21.06.2010	173	16	105	182	13	96	37,470683
21.06.2010	174	125	211	182	13	96	314,940663
21.06.2010	175	52	150	182	13	96	22,24008011
21.06.2010	176	37	136	182	13	96	65,10344823
21.06.2010	177	13	97	182	13	96	39,21993334
21.06.2010	178	26	122	182	13	96	55,78930116
21.06.2010	179	54	153	182	13	96	0,300541623
21.06.2010	180	67	166	182	13	96	77,84231107
21.06.2010	181	60	156	182	13	96	116,0498583
21.06.2010	170	49	148	191	128	203	86,32390448
21.06.2010	172	19	106	191	128	203	73,41082128
21.06.2010	173	16	105	191	128	203	44,0639529
21.06.2010	174	125	211	191	128	203	304,0811022
21.06.2010	175	52	150	191	128	203	11,82393541
21.06.2010	176	37	136	191	128	203	53,62331322
21.06.2010	177	13	97	191	128	203	28,32945578
21.06.2010	178	26	122	191	128	203	44,71045314
21.06.2010	179	54	153	191	128	203	13,97373117
21.06.2010	180	67	166	191	128	203	65,68880169
21.06.2010	181	60	156	191	128	203	102,968297
21.06.2010	182	13	96	191	128	203	13,78958856
21.06.2010	170	49	148	193	171	232	5,74891427
21.06.2010	172	19	106	193	171	232	20,24232058
21.06.2010	173	16	105	193	171	232	130,2168161

21.06.2010	174	125	211	193	171	232	220,7132337
21.06.2010	175	52	150	193	171	232	83,18143013
21.06.2010	176	37	136	193	171	232	44,7633518
21.06.2010	177	13	97	193	171	232	67,82978751
21.06.2010	178	26	122	193	171	232	53,63552402
21.06.2010	179	54	153	193	171	232	104,9662433
21.06.2010	180	67	166	193	171	232	31,17994414
21.06.2010	181	60	156	193	171	232	11,57143795
21.06.2010	182	13	96	193	171	232	104,8557808
21.06.2010	191	128	203	193	171	232	91,66480092
21.06.2010	170	49	148	194	81	184	223,9145423
21.06.2010	172	19	106	194	81	184	233,2250633
21.06.2010	173	16	105	194	81	184	346,3894561
21.06.2010	174	125	211	194	81	184	0,585863479
21.06.2010	175	52	150	194	81	184	292,5026655
21.06.2010	176	37	136	194	81	184	249,9210967
21.06.2010	177	13	97	194	81	184	275,4191377
21.06.2010	178	26	122	194	81	184	258,8714185
21.06.2010	179	54	153	194	81	184	314,3800706
21.06.2010	180	67	166	194	81	184	238,6440535
21.06.2010	181	60	156	194	81	184	209,0997474
21.06.2010	182	13	96	194	81	184	314,3805877
21.06.2010	191	128	203	194	81	184	303,5262389
21.06.2010	193	171	232	194	81	184	220,1933949
21.06.2010	170	49	148	172	19	106	6,856038877
21.06.2010	170	49	148	173	16	105	128,8600379
21.06.2010	172	19	106	173	16	105	123,3297736
21.06.2010	170	49	148	174	125	211	203,5868601
21.06.2010	172	19	106	174	125	211	209,3545853
21.06.2010	173	16	105	174	125	211	332,3951494
21.06.2010	170	49	148	175	52	150	69,24912057
21.06.2010	172	19	106	175	52	150	62,82182664
21.06.2010	173	16	105	175	52	150	65,12467216

21.06.2010	174	125	211	175	52	150	271,3224792
21.06.2010	170	49	148	176	37	136	40,09590212
21.06.2010	172	19	106	176	37	136	33,27309327
21.06.2010	173	16	105	176	37	136	96,31925314
21.06.2010	174	125	211	176	37	136	239,7077859
21.06.2010	175	52	150	176	37	136	31,92934742
21.06.2010	170	49	148	177	13	97	50,57946762
21.06.2010	172	19	106	177	13	97	44,08075861
21.06.2010	173	16	105	177	13	97	82,26203601
21.06.2010	174	125	211	177	13	97	252,603509
21.06.2010	175	52	150	177	13	97	18,7904622
21.06.2010	176	37	136	177	13	97	14,16217695
21.06.2010	170	49	148	178	26	122	47,77515367
21.06.2010	172	19	106	178	26	122	41,01253582
21.06.2010	173	16	105	178	26	122	88,72556795
21.06.2010	174	125	211	178	26	122	247,8636571
21.06.2010	175	52	150	178	26	122	23,95130069
21.06.2010	176	37	136	178	26	122	8,160134007
21.06.2010	177	13	97	178	26	122	7,825632959
21.06.2010	170	49	148	179	54	153	111,9520572
21.06.2010	172	19	106	179	54	153	106,1151144
21.06.2010	173	16	105	179	54	153	19,47709141
21.06.2010	174	125	211	179	54	153	315,4681071
21.06.2010	175	52	150	179	54	153	46,11389852
21.06.2010	176	37	136	179	54	153	77,72124918
21.06.2010	177	13	97	179	54	153	63,83634253
21.06.2010	178	26	122	179	54	153	69,94618003
21.06.2010	170	49	148	180	67	166	20,98977188
21.06.2010	172	19	106	180	67	166	14,13440576
21.06.2010	173	16	105	180	67	166	112,1824499
21.06.2010	174	125	211	180	67	166	221,5986956
21.06.2010	175	52	150	180	67	166	49,79077706
21.06.2010	176	37	136	180	67	166	19,22973249

21.06.2010	177	13	97	180	67	166	31,02137001
21.06.2010	178	26	122	180	67	166	27,13975727
21.06.2010	179	54	153	180	67	166	94,32526494
21.06.2010	170	49	148	181	60	156	19,80703829
21.06.2010	172	19	106	181	60	156	26,62160987
21.06.2010	173	16	105	181	60	156	144,0422038
21.06.2010	174	125	211	181	60	156	188,9963351
21.06.2010	175	52	150	181	60	156	87,39236738
21.06.2010	176	37	136	181	60	156	59,58543709
21.06.2010	177	13	97	181	60	156	69,07293929
21.06.2010	178	26	122	181	60	156	67,02630739
21.06.2010	179	54	153	181	60	156	128,1239642
21.06.2010	180	67	166	181	60	156	40,69160441
21.06.2010	170	49	148	182	13	96	108,9265009
21.06.2010	172	19	106	182	13	96	103,0837614
21.06.2010	173	16	105	182	13	96	22,20146354
21.06.2010	174	125	211	182	13	96	312,4373243
21.06.2010	175	52	150	182	13	96	43,16367869
21.06.2010	176	37	136	182	13	96	74,72385905
21.06.2010	177	13	97	182	13	96	60,82267485
21.06.2010	178	26	122	182	13	96	66,96678402
21.06.2010	179	54	153	182	13	96	3,035321608
21.06.2010	180	67	166	182	13	96	91,29384054
21.06.2010	181	60	156	182	13	96	125,1339254
21.06.2010	170	49	148	191	128	203	79,85561162
21.06.2010	172	19	106	191	128	203	73,43198125
21.06.2010	173	16	105	191	128	203	56,32810722
21.06.2010	174	125	211	191	128	203	281,8150772
21.06.2010	175	52	150	191	128	203	10,61043888
21.06.2010	176	37	136	191	128	203	42,21941288
21.06.2010	177	13	97	191	128	203	29,38522571
21.06.2010	178	26	122	191	128	203	34,12305702
21.06.2010	179	54	153	191	128	203	36,96075496

21.06.2010	180	67	166	191	128	203	60,35276902
21.06.2010	181	60	156	191	128	203	97,92133137
21.06.2010	182	13	96	191	128	203	34,13549586
21.06.2010	170	49	148	193	171	232	13,1862102
21.06.2010	172	19	106	193	171	232	19,95560168
21.06.2010	173	16	105	193	171	232	138,25568
21.06.2010	174	125	211	193	171	232	194,4023469
21.06.2010	175	52	150	193	171	232	80,87596858
21.06.2010	176	37	136	193	171	232	52,87559896
21.06.2010	177	13	97	193	171	232	62,47970495
21.06.2010	178	26	122	193	171	232	60,33241153
21.06.2010	179	54	153	193	171	232	122,0753682
21.06.2010	180	67	166	193	171	232	33,99496432
21.06.2010	181	60	156	193	171	232	6,710899958
21.06.2010	182	13	96	193	171	232	119,0741967
21.06.2010	191	128	203	193	171	232	91,42731071
21.06.2010	170	49	148	194	81	184	36,65905651
21.06.2010	172	19	106	194	81	184	43,51547964
21.06.2010	173	16	105	194	81	184	160,161871
21.06.2010	174	125	211	194	81	184	174,1160601
21.06.2010	175	52	150	194	81	184	104,5353584
21.06.2010	176	37	136	194	81	184	76,67534846
21.06.2010	177	13	97	194	81	184	86,26450351
21.06.2010	178	26	122	194	81	184	84,18749718
21.06.2010	179	54	153	194	81	184	144,672103
21.06.2010	180	67	166	194	81	184	57,6471425
21.06.2010	181	60	156	194	81	184	17,20033082
21.06.2010	182	13	96	194	81	184	141,7016623
21.06.2010	191	128	203	194	81	184	115,043235
21.06.2010	193	171	232	194	81	184	23,86194501
22.06.2010	170	49	148	172	19	106	9,520103632
22.06.2010	170	49	148	173	16	105	110,6443078
22.06.2010	172	19	106	173	16	105	101,2981772

22.06.2010	170	49	148	174	125	211	240,3304162
22.06.2010	172	19	106	174	125	211	249,3381409
22.06.2010	173	16	105	174	125	211	350,09068
22.06.2010	170	49	148	175	52	150	32,14039388
22.06.2010	172	19	106	175	52	150	23,3193962
22.06.2010	173	16	105	175	52	150	84,0414553
22.06.2010	174	125	211	175	52	150	266,4261909
22.06.2010	170	49	148	177	13	97	57,6206564
22.06.2010	172	19	106	177	13	97	48,14879411
22.06.2010	173	16	105	177	13	97	57,36866478
22.06.2010	174	125	211	177	13	97	293,7293396
22.06.2010	175	52	150	177	13	97	27,38031507
22.06.2010	170	49	148	178	26	122	44,49619976
22.06.2010	172	19	106	178	26	122	35,34809029
22.06.2010	173	16	105	178	26	122	72,67392261
22.06.2010	174	125	211	178	26	122	278,5104806
22.06.2010	175	52	150	178	26	122	12,68734246
22.06.2010	177	13	97	178	26	122	15,34411423
22.06.2010	170	49	148	179	54	153	109,7997468
22.06.2010	172	19	106	179	54	153	100,4528624
22.06.2010	173	16	105	179	54	153	0,850060079
22.06.2010	174	125	211	179	54	153	349,2454016
22.06.2010	175	52	150	179	54	153	83,2021679
22.06.2010	177	13	97	179	54	153	56,5423358
22.06.2010	178	26	122	179	54	153	71,84459917
22.06.2010	170	49	148	180	67	166	17,7444315
22.06.2010	172	19	106	180	67	166	8,893696449
22.06.2010	173	16	105	180	67	166	94,92996218
22.06.2010	174	125	211	180	67	166	255,19904
22.06.2010	175	52	150	180	67	166	14,47756135
22.06.2010	177	13	97	180	67	166	40,24082869
22.06.2010	178	26	122	180	67	166	26,75428214
22.06.2010	179	54	153	180	67	166	94,08402591

22.06.2010	170	49	148	181	60	156	16,29728347
22.06.2010	172	19	106	181	60	156	25,02936188
22.06.2010	173	16	105	181	60	156	123,5848935
22.06.2010	174	125	211	181	60	156	229,8974479
22.06.2010	175	52	150	181	60	156	48,24596465
22.06.2010	177	13	97	181	60	156	72,79611968
22.06.2010	178	26	122	181	60	156	60,37685982
22.06.2010	179	54	153	181	60	156	122,7477998
22.06.2010	180	67	166	181	60	156	33,77090348
22.06.2010	170	49	148	182	13	96	83,9480058
22.06.2010	172	19	106	182	13	96	74,4283305
22.06.2010	173	16	105	182	13	96	32,49492944
22.06.2010	174	125	211	182	13	96	320,5840459
22.06.2010	175	52	150	182	13	96	54,3255899
22.06.2010	177	13	97	182	13	96	26,94670339
22.06.2010	178	26	122	182	13	96	42,07368046
22.06.2010	179	54	153	182	13	96	31,73782153
22.06.2010	180	67	166	182	13	96	66,90957016
22.06.2010	181	60	156	182	13	96	98,53104827
22.06.2010	170	49	148	191	128	203	74,74210952
22.06.2010	172	19	106	191	128	203	65,22501072
22.06.2010	173	16	105	191	128	203	38,69524461
22.06.2010	174	125	211	191	128	203	312,3130449
22.06.2010	175	52	150	191	128	203	45,88730288
22.06.2010	177	13	97	191	128	203	18,74281274
22.06.2010	178	26	122	191	128	203	34,08677321
22.06.2010	179	54	153	191	128	203	37,87599579
22.06.2010	180	67	166	191	128	203	57,93160998
22.06.2010	181	60	156	191	128	203	89,12896144
22.06.2010	182	13	96	191	128	203	9,583461366
22.06.2010	170	49	148	193	171	232	10,76929167
22.06.2010	172	19	106	193	171	232	18,69890664
22.06.2010	173	16	105	193	171	232	116,9699735

22.06.2010	174	125	211	193	171	232	235,9828061
22.06.2010	175	52	150	193	171	232	42,01352607
22.06.2010	177	13	97	193	171	232	66,18128538
22.06.2010	178	26	122	193	171	232	53,97379475
22.06.2010	179	54	153	193	171	232	116,1318655
22.06.2010	180	67	166	193	171	232	27,552308
22.06.2010	181	60	156	193	171	232	6,687979691
22.06.2010	182	13	96	193	171	232	91,85183356
22.06.2010	191	128	203	193	171	232	82,44476301
22.06.2010	170	49	148	194	81	184	31,32416604
22.06.2010	172	19	106	194	81	184	39,11315701
22.06.2010	173	16	105	194	81	184	132,8490185
22.06.2010	174	125	211	194	81	184	225,8392826
22.06.2010	175	52	150	194	81	184	62,38334878
22.06.2010	177	13	97	194	81	184	85,2891377
22.06.2010	178	26	122	194	81	184	74,00040306
22.06.2010	179	54	153	194	81	184	132,0279727
22.06.2010	180	67	166	194	81	184	48,00695011
22.06.2010	181	60	156	194	81	184	15,57490089
22.06.2010	182	13	96	194	81	184	109,9940051
22.06.2010	191	128	203	194	81	184	100,4472873
22.06.2010	193	171	232	194	81	184	20,64307501
22.06.2010	170	49	148	172	19	106	15,14771522
22.06.2010	170	49	148	173	16	105	93,86430216
22.06.2010	172	19	106	173	16	105	104,4206341
22.06.2010	170	49	148	174	125	211	242,8940881
22.06.2010	172	19	106	174	125	211	232,0082057
22.06.2010	173	16	105	174	125	211	336,3557333
22.06.2010	170	49	148	175	52	150	52,88439576
22.06.2010	172	19	106	175	52	150	65,7178088
22.06.2010	173	16	105	175	52	150	43,90243479
22.06.2010	174	125	211	175	52	150	295,4918301
22.06.2010	170	49	148	176	37	136	18,83559356

22.06.2010	172	19	106	176	37	136	33,63462267
22.06.2010	173	16	105	176	37	136	78,63779398
22.06.2010	174	125	211	176	37	136	259,9173201
22.06.2010	175	52	150	176	37	136	35,71845839
22.06.2010	170	49	148	177	13	97	42,90782979
22.06.2010	172	19	106	177	13	97	56,84147144
22.06.2010	173	16	105	177	13	97	56,5432144
22.06.2010	174	125	211	177	13	97	284,3830862
22.06.2010	175	52	150	177	13	97	12,68271406
22.06.2010	176	37	136	177	13	97	24,6031457
22.06.2010	170	49	148	178	26	122	31,10482387
22.06.2010	172	19	106	178	26	122	45,4526854
22.06.2010	173	16	105	178	26	122	67,40204576
22.06.2010	174	125	211	178	26	122	272,3231844
22.06.2010	175	52	150	178	26	122	23,7640829
22.06.2010	176	37	136	178	26	122	12,54774774
22.06.2010	177	13	97	178	26	122	12,07938064
22.06.2010	170	49	148	179	54	153	101,8519554
22.06.2010	172	19	106	179	54	153	112,905132
22.06.2010	173	16	105	179	54	153	9,554198821
22.06.2010	174	125	211	179	54	153	344,6355755
22.06.2010	175	52	150	179	54	153	50,53551763
22.06.2010	176	37	136	179	54	153	85,95112111
22.06.2010	177	13	97	179	54	153	62,93035873
22.06.2010	178	26	122	179	54	153	74,28717782
22.06.2010	170	49	148	180	67	166	21,12745621
22.06.2010	172	19	106	180	67	166	5,980702788
22.06.2010	173	16	105	180	67	166	108,9865207
22.06.2010	174	125	211	180	67	166	227,7338954
22.06.2010	175	52	150	180	67	166	71,10264764
22.06.2010	176	37	136	180	67	166	39,56981732
22.06.2010	177	13	97	180	67	166	62,54479864
22.06.2010	178	26	122	180	67	166	51,27921814

22.06.2010	179	54	153	180	67	166	117,6156117
22.06.2010	170	49	148	181	60	156	41,12195835
22.06.2010	172	19	106	181	60	156	26,03460784
22.06.2010	173	16	105	181	60	156	123,022924
22.06.2010	174	125	211	181	60	156	216,8663246
22.06.2010	175	52	150	181	60	156	88,35013872
22.06.2010	176	37	136	181	60	156	59,12617002
22.06.2010	177	13	97	181	60	156	81,00357821
22.06.2010	178	26	122	181	60	156	70,29539431
22.06.2010	179	54	153	181	60	156	132,075922
22.06.2010	180	67	166	181	60	156	20,12731536
22.06.2010	170	49	148	182	13	96	61,22159011
22.06.2010	172	19	106	182	13	96	73,98355679
22.06.2010	173	16	105	182	13	96	36,76780083
22.06.2010	174	125	211	182	13	96	303,7946918
22.06.2010	175	52	150	182	13	96	8,337525153
22.06.2010	176	37	136	182	13	96	43,95086007
22.06.2010	177	13	97	182	13	96	20,16632052
22.06.2010	178	26	122	182	13	96	31,81116312
22.06.2010	179	54	153	182	13	96	42,7871431
22.06.2010	180	67	166	182	13	96	79,32294988
22.06.2010	181	60	156	182	13	96	96,32783335
22.06.2010	170	49	148	191	128	203	62,18423343
22.06.2010	172	19	106	191	128	203	74,85236355
22.06.2010	173	16	105	191	128	203	35,57021526
22.06.2010	174	125	211	191	128	203	304,815635
22.06.2010	175	52	150	191	128	203	9,325027334
22.06.2010	176	37	136	191	128	203	45,00704882
22.06.2010	177	13	97	191	128	203	21,31855095
22.06.2010	178	26	122	191	128	203	32,91198368
22.06.2010	179	54	153	191	128	203	41,62072889
22.06.2010	180	67	166	191	128	203	80,16200635
22.06.2010	181	60	156	191	128	203	97,0548906

22.06.2010	182	13	96	191	128	203	1,202166492
22.06.2010	170	49	148	193	171	232	34,81745844
22.06.2010	172	19	106	193	171	232	19,67205061
22.06.2010	173	16	105	193	171	232	119,8996106
22.06.2010	174	125	211	193	171	232	218,2622369
22.06.2010	175	52	150	193	171	232	83,72919177
22.06.2010	176	37	136	193	171	232	53,1939146
22.06.2010	177	13	97	193	171	232	75,7585494
22.06.2010	178	26	122	193	171	232	64,72078549
22.06.2010	179	54	153	193	171	232	128,7881699
22.06.2010	180	67	166	193	171	232	13,6913121
22.06.2010	181	60	156	193	171	232	7,08029119
22.06.2010	182	13	96	193	171	232	91,83826191
22.06.2010	191	128	203	193	171	232	92,61954608
22.06.2010	170	49	148	194	81	184	55,94063831
22.06.2010	172	19	106	194	81	184	40,83452741
22.06.2010	173	16	105	194	81	184	138,9662348
22.06.2010	174	125	211	194	81	184	202,6256215
22.06.2010	175	52	150	194	81	184	104,4595525
22.06.2010	176	37	136	194	81	184	74,42013834
22.06.2010	177	13	97	194	81	184	96,87328821
22.06.2010	178	26	122	194	81	184	85,93556651
22.06.2010	179	54	153	194	81	184	148,0731703
22.06.2010	180	67	166	194	81	184	34,86684392
22.06.2010	181	60	156	194	81	184	16,15896911
22.06.2010	182	13	96	194	81	184	112,4627396
22.06.2010	191	128	203	194	81	184	113,1971581
22.06.2010	193	171	232	194	81	184	21,23513202
22.06.2010	170	49	148	173	16	105	97,67038644
22.06.2010	170	49	148	174	125	211	260,0405893
22.06.2010	173	16	105	174	125	211	357,5982099
22.06.2010	170	49	148	175	52	150	53,55378499
22.06.2010	173	16	105	175	52	150	45,0167813

22.06.2010	174	125	211	175	52	150	313,4930065
22.06.2010	170	49	148	176	37	136	17,02721477
22.06.2010	173	16	105	176	37	136	81,95401405
22.06.2010	174	125	211	176	37	136	276,336084
22.06.2010	175	52	150	176	37	136	37,21222346
22.06.2010	170	49	148	177	13	97	38,33761533
22.06.2010	173	16	105	177	13	97	61,96298067
22.06.2010	174	125	211	177	13	97	297,4460308
22.06.2010	175	52	150	177	13	97	17,03066252
22.06.2010	176	37	136	177	13	97	21,37905324
22.06.2010	170	49	148	178	26	122	27,20740088
22.06.2010	173	16	105	178	26	122	73,55611174
22.06.2010	174	125	211	178	26	122	285,6996157
22.06.2010	175	52	150	178	26	122	28,5397465
22.06.2010	176	37	136	178	26	122	10,24269486
22.06.2010	177	13	97	178	26	122	11,77877797
22.06.2010	170	49	148	179	54	153	62,34004134
22.06.2010	173	16	105	179	54	153	38,72861033
22.06.2010	174	125	211	179	54	153	321,8356536
22.06.2010	175	52	150	179	54	153	9,920605385
22.06.2010	176	37	136	179	54	153	45,59742391
22.06.2010	177	13	97	179	54	153	24,39576164
22.06.2010	178	26	122	179	54	153	36,16491169
22.06.2010	170	49	148	180	67	166	22,0807094
22.06.2010	173	16	105	180	67	166	112,9811874
22.06.2010	174	125	211	180	67	166	245,6245804
22.06.2010	175	52	150	180	67	166	71,2113066
22.06.2010	176	37	136	180	67	166	37,79960635
22.06.2010	177	13	97	180	67	166	57,99931639
22.06.2010	178	26	122	180	67	166	47,9437841
22.06.2010	179	54	153	180	67	166	80,71783741
22.06.2010	170	49	148	181	60	156	44,74024156
22.06.2010	173	16	105	181	60	156	134,6571368

22.06.2010	174	125	211	181	60	156	226,3646706
22.06.2010	175	52	150	181	60	156	94,04796917
22.06.2010	176	37	136	181	60	156	60,98714553
22.06.2010	177	13	97	181	60	156	81,23384894
22.06.2010	178	26	122	181	60	156	71,16772331
22.06.2010	179	54	153	181	60	156	103,6769286
22.06.2010	180	67	166	181	60	156	23,25656252
22.06.2010	170	49	148	182	13	96	12,84195752
22.06.2010	173	16	105	182	13	96	103,7111618
22.06.2010	174	125	211	182	13	96	254,2619433
22.06.2010	175	52	150	182	13	96	61,31432144
22.06.2010	176	37	136	182	13	96	27,68117243
22.06.2010	177	13	97	182	13	96	47,83297205
22.06.2010	178	26	122	182	13	96	37,78634184
22.06.2010	179	54	153	182	13	96	70,72849784
22.06.2010	180	67	166	182	13	96	10,18343947
22.06.2010	181	60	156	182	13	96	33,44030362
22.06.2010	170	49	148	191	128	203	64,3928225
22.06.2010	173	16	105	191	128	203	38,9824614
22.06.2010	174	125	211	191	128	203	323,3992238
22.06.2010	175	52	150	191	128	203	13,34959746
22.06.2010	176	37	136	191	128	203	47,48861982
22.06.2010	177	13	97	191	128	203	26,11739561
22.06.2010	178	26	122	191	128	203	37,75034886
22.06.2010	179	54	153	191	128	203	3,939274161
22.06.2010	180	67	166	191	128	203	83,29298406
22.06.2010	181	60	156	191	128	203	106,3578546
22.06.2010	182	13	96	191	128	203	73,23367487
22.06.2010	170	49	148	193	171	232	38,79874791
22.06.2010	173	16	105	193	171	232	126,4342568
22.06.2010	174	125	211	193	171	232	234,7997276
22.06.2010	175	52	150	193	171	232	86,32003289
22.06.2010	176	37	136	193	171	232	54,35318313

22.06.2010	177	13	97	193	171	232	74,04884523
22.06.2010	178	26	122	193	171	232	64,40614698
22.06.2010	179	54	153	193	171	232	96,03064416
22.06.2010	180	67	166	193	171	232	16,73075023
22.06.2010	181	60	156	193	171	232	8,450512192
22.06.2010	182	13	96	193	171	232	26,68125862
22.06.2010	191	128	203	193	171	232	98,81541662
22.06.2010	170	49	148	194	81	184	53,10919997
22.06.2010	173	16	105	194	81	184	140,3395836
22.06.2010	174	125	211	194	81	184	223,2559242
22.06.2010	175	52	150	194	81	184	100,8805911
22.06.2010	176	37	136	194	81	184	68,97669241
22.06.2010	177	13	97	194	81	184	88,7604795
22.06.2010	178	26	122	194	81	184	79,06906834
22.06.2010	179	54	153	194	81	184	110,6298165
22.06.2010	180	67	166	194	81	184	31,20817616
22.06.2010	181	60	156	194	81	184	9,022257525
22.06.2010	182	13	96	194	81	184	41,2970875
22.06.2010	191	128	203	194	81	184	113,4576274
22.06.2010	193	171	232	194	81	184	14,71242572
22.06.2010	170	49	148	172	19	106	18,91503063
22.06.2010	170	49	148	173	16	105	36,41195412
22.06.2010	172	19	106	173	16	105	50,22031638
22.06.2010	170	49	148	174	125	211	70,28068022
22.06.2010	172	19	106	174	125	211	51,37046762
22.06.2010	173	16	105	174	125	211	97,24860065
22.06.2010	170	49	148	175	52	150	45,71118542
22.06.2010	172	19	106	175	52	150	62,75133486
22.06.2010	173	16	105	175	52	150	18,15579881
22.06.2010	174	125	211	175	52	150	112,3733529
22.06.2010	170	49	148	176	37	136	11,28692859
22.06.2010	172	19	106	176	37	136	29,16864451
22.06.2010	173	16	105	176	37	136	26,22955386

22.06.2010	174	125	211	176	37	136	80,25384298
22.06.2010	175	52	150	176	37	136	34,42507185
22.06.2010	170	49	148	177	13	97	41,4587338
22.06.2010	172	19	106	177	13	97	58,38016195
22.06.2010	173	16	105	177	13	97	14,7908011
22.06.2010	174	125	211	177	13	97	107,9987029
22.06.2010	175	52	150	177	13	97	4,387290104
22.06.2010	176	37	136	177	13	97	30,17324144
22.06.2010	170	49	148	178	26	122	30,57529151
22.06.2010	172	19	106	178	26	122	47,87553534
22.06.2010	173	16	105	178	26	122	14,15356413
22.06.2010	174	125	211	178	26	122	98,12359517
22.06.2010	175	52	150	178	26	122	15,13667972
22.06.2010	176	37	136	178	26	122	19,29023078
22.06.2010	177	13	97	178	26	122	10,93244683
22.06.2010	170	49	148	179	54	153	93,97826508
22.06.2010	172	19	106	179	54	153	108,7863535
22.06.2010	173	16	105	179	54	153	58,56637885
22.06.2010	174	125	211	179	54	153	154,1569364
22.06.2010	175	52	150	179	54	153	50,0113362
22.06.2010	176	37	136	179	54	153	82,91986411
22.06.2010	177	13	97	179	54	153	53,64411202
22.06.2010	178	26	122	179	54	153	64,33949696
22.06.2010	170	49	148	180	67	166	9,286687524
22.06.2010	172	19	106	180	67	166	9,872231934
22.06.2010	173	16	105	180	67	166	41,59446119
22.06.2010	174	125	211	180	67	166	61,17391669
22.06.2010	175	52	150	180	67	166	53,16911747
22.06.2010	176	37	136	180	67	166	19,31222569
22.06.2010	177	13	97	180	67	166	48,81854404
22.06.2010	178	26	122	180	67	166	38,18688534
22.06.2010	179	54	153	180	67	166	100,0381133
22.06.2010	170	49	148	181	60	156	30,86285913

22.06.2010	172	19	106	181	60	156	11,97800712
22.06.2010	173	16	105	181	60	156	59,85699722
22.06.2010	174	125	211	181	60	156	39,49876215
22.06.2010	175	52	150	181	60	156	73,63478304
22.06.2010	176	37	136	181	60	156	40,78583476
22.06.2010	177	13	97	181	60	156	69,24755533
22.06.2010	178	26	122	181	60	156	59,02273268
22.06.2010	179	54	153	181	60	156	118,155599
22.06.2010	180	67	166	181	60	156	21,68715517
22.06.2010	170	49	148	182	13	96	79,85810001
22.06.2010	172	19	106	182	13	96	95,07827935
22.06.2010	173	16	105	182	13	96	44,93856095
22.06.2010	174	125	211	182	13	96	141,480811
22.06.2010	175	52	150	182	13	96	35,67556928
22.06.2010	176	37	136	182	13	96	68,73952632
22.06.2010	177	13	97	182	13	96	39,31068347
22.06.2010	178	26	122	182	13	96	50,03814953
22.06.2010	179	54	153	182	13	96	14,34027671
22.06.2010	180	67	166	182	13	96	86,15349466
22.06.2010	181	60	156	182	13	96	104,7664131
22.06.2010	170	49	148	191	128	203	61,86202354
22.06.2010	172	19	106	191	128	203	77,21463538
22.06.2010	173	16	105	191	128	203	27,29308482
22.06.2010	174	125	211	191	128	203	124,4058642
22.06.2010	175	52	150	191	128	203	18,67304553
22.06.2010	176	37	136	191	128	203	50,7636065
22.06.2010	177	13	97	191	128	203	21,79828206
22.06.2010	178	26	122	191	128	203	32,25667193
22.06.2010	179	54	153	191	128	203	32,16665978
22.06.2010	180	67	166	191	128	203	68,20056967
22.06.2010	181	60	156	191	128	203	87,1226571
22.06.2010	182	13	96	191	128	203	17,99990927
22.06.2010	170	49	148	193	171	232	46,33958107

22.06.2010	172	19	106	193	171	232	27,53968358
22.06.2010	173	16	105	193	171	232	72,75329956
22.06.2010	174	125	211	193	171	232	24,66346447
22.06.2010	175	52	150	193	171	232	87,71028069
22.06.2010	176	37	136	193	171	232	55,87067967
22.06.2010	177	13	97	193	171	232	83,33537442
22.06.2010	178	26	122	193	171	232	73,49173754
22.06.2010	179	54	153	193	171	232	130,161659
22.06.2010	180	67	166	193	171	232	37,0898075
22.06.2010	181	60	156	193	171	232	15,59055144
22.06.2010	182	13	96	193	171	232	117,2403203
22.06.2010	191	128	203	193	171	232	99,98482753
22.06.2010	170	49	148	194	81	184	56,05604838
22.06.2010	172	19	106	194	81	184	37,15428723
22.06.2010	173	16	105	194	81	184	82,79068049
22.06.2010	174	125	211	194	81	184	14,55502943
22.06.2010	175	52	150	194	81	184	97,81907308
22.06.2010	176	37	136	194	81	184	65,82630011
22.06.2010	177	13	97	194	81	184	93,44399241
22.06.2010	178	26	122	194	81	184	83,58083614
22.06.2010	179	54	153	194	81	184	139,9876941
22.06.2010	180	67	166	194	81	184	46,86984916
22.06.2010	181	60	156	194	81	184	25,19428076
22.06.2010	182	13	96	194	81	184	127,1713388
22.06.2010	191	128	203	194	81	184	109,9926501
22.06.2010	193	171	232	194	81	184	10,10910517
22.06.2010	170	49	148	172	19	106	23,5326202
22.06.2010	170	49	148	173	16	105	97,14247288
22.06.2010	172	19	106	173	16	105	116,8716686
22.06.2010	170	49	148	174	125	211	247,0009334
22.06.2010	172	19	106	174	125	211	230,0101808
22.06.2010	173	16	105	174	125	211	343,8546083
22.06.2010	170	49	148	175	52	150	45,1488147

22.06.2010	172	19	106	175	52	150	67,46982058
22.06.2010	173	16	105	175	52	150	54,4133078
22.06.2010	174	125	211	175	52	150	290,0337535
22.06.2010	170	49	148	176	37	136	6,979428102
22.06.2010	172	19	106	176	37	136	30,46267714
22.06.2010	173	16	105	176	37	136	91,09098798
22.06.2010	174	125	211	176	37	136	252,8134626
22.06.2010	175	52	150	176	37	136	38,44678054
22.06.2010	170	49	148	177	13	97	42,51277852
22.06.2010	172	19	106	177	13	97	65,91652745
22.06.2010	173	16	105	177	13	97	63,49655758
22.06.2010	174	125	211	177	13	97	283,1052
22.06.2010	175	52	150	177	13	97	11,92321006
22.06.2010	176	37	136	177	13	97	35,53286719
22.06.2010	170	49	148	178	26	122	21,55346566
22.06.2010	172	19	106	178	26	122	44,80792386
22.06.2010	173	16	105	178	26	122	77,9269135
22.06.2010	174	125	211	178	26	122	266,0386402
22.06.2010	175	52	150	178	26	122	24,21123473
22.06.2010	176	37	136	178	26	122	14,61232012
22.06.2010	177	13	97	178	26	122	21,14369594
22.06.2010	170	49	148	179	54	153	77,0378525
22.06.2010	172	19	106	179	54	153	98,75772129
22.06.2010	173	16	105	179	54	153	25,91306754
22.06.2010	174	125	211	179	54	153	321,7388125
22.06.2010	175	52	150	179	54	153	32,04540472
22.06.2010	176	37	136	179	54	153	70,43274855
22.06.2010	177	13	97	179	54	153	39,04011392
22.06.2010	178	26	122	179	54	153	56,25204732
22.06.2010	170	49	148	180	67	166	9,262339814
22.06.2010	172	19	106	180	67	166	14,41910886
22.06.2010	173	16	105	180	67	166	105,3676814
22.06.2010	174	125	211	180	67	166	239,3614697

22.06.2010	175	52	150	180	67	166	54,15956849
22.06.2010	176	37	136	180	67	166	16,24259956
22.06.2010	177	13	97	180	67	166	51,77486989
22.06.2010	178	26	122	180	67	166	30,794299
22.06.2010	179	54	153	180	67	166	85,90534261
22.06.2010	170	49	148	181	60	156	41,41175666
22.06.2010	172	19	106	181	60	156	18,3320536
22.06.2010	173	16	105	181	60	156	134,8135389
22.06.2010	174	125	211	181	60	156	214,1221023
22.06.2010	175	52	150	181	60	156	85,79307942
22.06.2010	176	37	136	181	60	156	48,39149459
22.06.2010	177	13	97	181	60	156	83,92454644
22.06.2010	178	26	122	181	60	156	62,90444586
22.06.2010	179	54	153	181	60	156	117,0708963
22.06.2010	180	67	166	181	60	156	32,14980713
22.06.2010	170	49	148	182	13	96	76,97339782
22.06.2010	172	19	106	182	13	96	98,68461651
22.06.2010	173	16	105	182	13	96	25,89859783
22.06.2010	174	125	211	182	13	96	321,6940312
22.06.2010	175	52	150	182	13	96	31,98785227
22.06.2010	176	37	136	182	13	96	70,37097232
22.06.2010	177	13	97	182	13	96	39,00909865
22.06.2010	178	26	122	182	13	96	56,19533667
22.06.2010	179	54	153	182	13	96	0,134406299
22.06.2010	180	67	166	182	13	96	85,83807597
22.06.2010	181	60	156	182	13	96	116,9972293
22.06.2010	170	49	148	191	128	203	63,82955041
22.06.2010	172	19	106	191	128	203	85,55517917
22.06.2010	173	16	105	191	128	203	36,17627396
22.06.2010	174	125	211	191	128	203	308,9818133
22.06.2010	175	52	150	191	128	203	18,98890378
22.06.2010	176	37	136	191	128	203	57,25613723
22.06.2010	177	13	97	191	128	203	27,32632462

22.06.2010	178	26	122	191	128	203	43,18104396
22.06.2010	179	54	153	191	128	203	13,22656333
22.06.2010	180	67	166	191	128	203	72,6805096
22.06.2010	181	60	156	191	128	203	103,8748113
22.06.2010	182	13	96	191	128	203	13,15809531
22.06.2010	170	49	148	193	171	232	29,98435559
22.06.2010	172	19	106	193	171	232	6,48780198
22.06.2010	173	16	105	193	171	232	122,8747069
22.06.2010	174	125	211	193	171	232	224,9995182
22.06.2010	175	52	150	193	171	232	73,89847337
22.06.2010	176	37	136	193	171	232	36,92995005
22.06.2010	177	13	97	193	171	232	72,4010322
22.06.2010	178	26	122	193	171	232	51,29568496
22.06.2010	179	54	153	193	171	232	105,0897409
22.06.2010	180	67	166	193	171	232	20,80911654
22.06.2010	181	60	156	193	171	232	11,99985588
22.06.2010	182	13	96	193	171	232	105,0156254
22.06.2010	191	128	203	193	171	232	91,90030141
22.06.2010	170	49	148	194	81	184	246,8158992
22.06.2010	172	19	106	194	81	184	229,7888461
22.06.2010	173	16	105	194	81	184	343,6832954
22.06.2010	174	125	211	194	81	184	0,570237637
22.06.2010	175	52	150	194	81	184	289,8763607
22.06.2010	176	37	136	194	81	184	252,6367436
22.06.2010	177	13	97	194	81	184	282,9664262
22.06.2010	178	26	122	194	81	184	265,8749415
22.06.2010	179	54	153	194	81	184	321,5897045
22.06.2010	180	67	166	194	81	184	239,164898
22.06.2010	181	60	156	194	81	184	213,8793645
22.06.2010	182	13	96	194	81	184	321,544762
22.06.2010	191	128	203	194	81	184	308,8266337
22.06.2010	193	171	232	194	81	184	224,7687287
22.06.2010	170	49	148	172	19	106	11,03442161

22.06.2010	170	49	148	173	16	105	47,09641627
22.06.2010	172	19	106	173	16	105	54,15222984
22.06.2010	170	49	148	174	125	211	61,45668355
22.06.2010	172	19	106	174	125	211	50,62998079
22.06.2010	173	16	105	174	125	211	100,3867508
22.06.2010	170	49	148	175	52	150	51,59425277
22.06.2010	172	19	106	175	52	150	60,726813
22.06.2010	173	16	105	175	52	150	15,89748715
22.06.2010	174	125	211	175	52	150	109,8695664
22.06.2010	170	49	148	176	37	136	11,9273759
22.06.2010	172	19	106	176	37	136	22,44907116
22.06.2010	173	16	105	176	37	136	37,37558633
22.06.2010	174	125	211	176	37	136	73,078474
22.06.2010	175	52	150	176	37	136	40,07269228
22.06.2010	170	49	148	177	13	97	42,61061285
22.06.2010	172	19	106	177	13	97	51,70626254
22.06.2010	173	16	105	177	13	97	13,80399159
22.06.2010	174	125	211	177	13	97	100,9945519
22.06.2010	175	52	150	177	13	97	9,02425958
22.06.2010	176	37	136	177	13	97	31,18544777
22.06.2010	170	49	148	178	26	122	32,51785365
22.06.2010	172	19	106	178	26	122	42,25170585
22.06.2010	173	16	105	178	26	122	21,28590157
22.06.2010	174	125	211	178	26	122	92,3713591
22.06.2010	175	52	150	178	26	122	19,41694275
22.06.2010	176	37	136	178	26	122	20,77762294
22.06.2010	177	13	97	178	26	122	10,8428487
22.06.2010	170	49	148	179	54	153	102,0903221
22.06.2010	172	19	106	179	54	153	109,7301297
22.06.2010	173	16	105	179	54	153	55,616281
22.06.2010	174	125	211	179	54	153	155,0747597
22.06.2010	175	52	150	179	54	153	52,35569645
22.06.2010	176	37	136	179	54	153	91,33271532

22.06.2010	177	13	97	179	54	153	60,55842859
22.06.2010	178	26	122	179	54	153	71,39695038
22.06.2010	170	49	148	180	67	166	6,310658471
22.06.2010	172	19	106	180	67	166	17,30138537
22.06.2010	173	16	105	180	67	166	43,02177491
22.06.2010	174	125	211	180	67	166	67,76503861
22.06.2010	175	52	150	180	67	166	46,17575331
22.06.2010	176	37	136	180	67	166	6,103289295
22.06.2010	177	13	97	180	67	166	37,28085279
22.06.2010	178	26	122	180	67	166	26,87151376
22.06.2010	179	54	153	180	67	166	97,33512705
22.06.2010	170	49	148	181	60	156	31,83755878
22.06.2010	172	19	106	181	60	156	20,87563932
22.06.2010	173	16	105	181	60	156	71,84654786
22.06.2010	174	125	211	181	60	156	29,84848918
22.06.2010	175	52	150	181	60	156	80,35788379
22.06.2010	176	37	136	181	60	156	43,29082747
22.06.2010	177	13	97	181	60	156	71,41554513
22.06.2010	178	26	122	181	60	156	62,56725144
22.06.2010	179	54	153	181	60	156	127,2182431
22.06.2010	180	67	166	181	60	156	38,14025953
22.06.2010	170	49	148	182	13	96	79,51992864
22.06.2010	172	19	106	182	13	96	87,38384009
22.06.2010	173	16	105	182	13	96	33,51948686
22.06.2010	174	125	211	182	13	96	133,7431835
22.06.2010	175	52	150	182	13	96	30,00468249
22.06.2010	176	37	136	182	13	96	68,69858233
22.06.2010	177	13	97	182	13	96	37,97269373
22.06.2010	178	26	122	182	13	96	48,81539855
22.06.2010	179	54	153	182	13	96	22,63738879
22.06.2010	180	67	166	182	13	96	74,70796181
22.06.2010	181	60	156	182	13	96	105,3647668
22.06.2010	170	49	148	191	128	203	63,55130109

22.06.2010	172	19	106	191	128	203	71,59447768
22.06.2010	173	16	105	191	128	203	18,44111253
22.06.2010	174	125	211	191	128	203	118,7477948
22.06.2010	175	52	150	191	128	203	14,99759916
22.06.2010	176	37	136	191	128	203	52,70449612
22.06.2010	177	13	97	191	128	203	22,17255882
22.06.2010	178	26	122	191	128	203	32,9873697
22.06.2010	179	54	153	191	128	203	38,62819288
22.06.2010	180	67	166	191	128	203	58,71155802
22.06.2010	181	60	156	191	128	203	89,9819813
22.06.2010	182	13	96	191	128	203	15,99632613
22.06.2010	170	49	148	193	171	232	22,74923408
22.06.2010	172	19	106	193	171	232	11,74652622
22.06.2010	173	16	105	193	171	232	63,64610195
22.06.2010	174	125	211	193	171	232	38,9748143
22.06.2010	175	52	150	193	171	232	71,5385028
22.06.2010	176	37	136	193	171	232	34,14396227
22.06.2010	177	13	97	193	171	232	62,56089906
22.06.2010	178	26	122	193	171	232	53,52982952
22.06.2010	179	54	153	193	171	232	119,1995969
22.06.2010	180	67	166	193	171	232	29,03829368
22.06.2010	181	60	156	193	171	232	9,147037502
22.06.2010	182	13	96	193	171	232	97,13931143
22.06.2010	191	128	203	193	171	232	81,58949529
22.06.2010	170	49	148	194	81	184	39,40328773
22.06.2010	172	19	106	194	81	184	28,38583413
22.06.2010	173	16	105	194	81	184	77,63031432
22.06.2010	174	125	211	194	81	184	22,9543768
22.06.2010	175	52	150	194	81	184	86,91557742
22.06.2010	176	37	136	194	81	184	50,6829387
22.06.2010	177	13	97	194	81	184	78,04701986
22.06.2010	178	26	122	194	81	184	69,53931791
22.06.2010	179	54	153	194	81	184	132,6385218

22.06.2010	180	67	166	194	81	184	45,68628105
22.06.2010	181	60	156	194	81	184	7,739749173
22.06.2010	182	13	96	194	81	184	111,0752398
22.06.2010	191	128	203	194	81	184	95,93484452
22.06.2010	193	171	232	194	81	184	16,65553844
22.06.2010	170	49	148	172	19	106	19,87986857
22.06.2010	170	49	148	173	16	105	98,22231789
22.06.2010	172	19	106	173	16	105	114,1612805
22.06.2010	170	49	148	174	125	211	256,9584654
22.06.2010	172	19	106	174	125	211	243,6790665
22.06.2010	173	16	105	174	125	211	354,7405464
22.06.2010	170	49	148	175	52	150	45,51395038
22.06.2010	172	19	106	175	52	150	64,44351097
22.06.2010	173	16	105	175	52	150	57,03461298
22.06.2010	174	125	211	175	52	150	298,7889231
22.06.2010	170	49	148	176	37	136	15,45087963
22.06.2010	172	19	106	176	37	136	35,32380652
22.06.2010	173	16	105	176	37	136	86,5229007
22.06.2010	174	125	211	176	37	136	268,2656145
22.06.2010	175	52	150	176	37	136	31,31017763
22.06.2010	170	49	148	177	13	97	40,06457693
22.06.2010	172	19	106	177	13	97	59,07244802
22.06.2010	173	16	105	177	13	97	61,82134614
22.06.2010	174	125	211	177	13	97	293,5959568
22.06.2010	175	52	150	177	13	97	5,460436862
22.06.2010	176	37	136	177	13	97	25,8815027
22.06.2010	170	49	148	178	26	122	29,95451785
22.06.2010	172	19	106	178	26	122	49,83194003
22.06.2010	173	16	105	178	26	122	78,24166958
22.06.2010	174	125	211	178	26	122	278,0226234
22.06.2010	175	52	150	178	26	122	21,20895639
22.06.2010	176	37	136	178	26	122	14,56433512
22.06.2010	177	13	97	178	26	122	16,65445378

22.06.2010	170	49	148	179	54	153	73,54260274
22.06.2010	172	19	106	179	54	153	91,9718259
22.06.2010	173	16	105	179	54	153	33,45421134
22.06.2010	174	125	211	179	54	153	326,4426887
22.06.2010	175	52	150	179	54	153	28,24867441
22.06.2010	176	37	136	179	54	153	59,55875136
22.06.2010	177	13	97	179	54	153	33,68828955
22.06.2010	178	26	122	179	54	153	48,43160885
22.06.2010	170	49	148	180	67	166	7,318049669
22.06.2010	172	19	106	180	67	166	27,18498247
22.06.2010	173	16	105	180	67	166	92,40244948
22.06.2010	174	125	211	180	67	166	262,4101869
22.06.2010	175	52	150	180	67	166	38,58608007
22.06.2010	176	37	136	180	67	166	8,139075562
22.06.2010	177	13	97	180	67	166	33,12481368
22.06.2010	178	26	122	180	67	166	22,67705545
22.06.2010	179	54	153	180	67	166	66,74879923
22.06.2010	170	49	148	181	60	156	30,1091112
22.06.2010	172	19	106	181	60	156	10,47334734
22.06.2010	173	16	105	181	60	156	121,3017043
22.06.2010	174	125	211	181	60	156	239,4596762
22.06.2010	175	52	150	181	60	156	73,68714305
22.06.2010	176	37	136	181	60	156	45,47086499
22.06.2010	177	13	97	181	60	156	68,40283768
22.06.2010	178	26	122	181	60	156	60,0262207
22.06.2010	179	54	153	181	60	156	100,7420273
22.06.2010	180	67	166	181	60	156	37,34971878
22.06.2010	170	49	148	182	13	96	62,60989679
22.06.2010	172	19	106	182	13	96	80,81759279
22.06.2010	173	16	105	182	13	96	39,76144094
22.06.2010	174	125	211	182	13	96	316,7185997
22.06.2010	175	52	150	182	13	96	17,93102957
22.06.2010	176	37	136	182	13	96	49,01381474

22.06.2010	177	13	97	182	13	96	23,20562816
22.06.2010	178	26	122	182	13	96	38,92565168
22.06.2010	179	54	153	182	13	96	11,27371649
22.06.2010	180	67	166	182	13	96	55,94984113
22.06.2010	181	60	156	182	13	96	89,5060688
22.06.2010	170	49	148	191	128	203	71,00349118
22.06.2010	172	19	106	191	128	203	89,18868267
22.06.2010	173	16	105	191	128	203	33,26630592
22.06.2010	174	125	211	191	128	203	324,745703
22.06.2010	175	52	150	191	128	203	26,08867482
22.06.2010	176	37	136	191	128	203	57,31621093
22.06.2010	177	13	97	191	128	203	31,45337164
22.06.2010	178	26	122	191	128	203	46,76450835
22.06.2010	179	54	153	191	128	203	3,688222506
22.06.2010	180	67	166	191	128	203	64,32271834
22.06.2010	181	60	156	191	128	203	97,81542562
22.06.2010	182	13	96	191	128	203	8,396898453
22.06.2010	170	49	148	193	171	232	37,88541444
22.06.2010	172	19	106	193	171	232	18,00793643
22.06.2010	173	16	105	193	171	232	129,7510971
22.06.2010	174	125	211	193	171	232	232,1202247
22.06.2010	175	52	150	193	171	232	82,06327407
22.06.2010	176	37	136	193	171	232	53,3317017
22.06.2010	177	13	97	193	171	232	76,7498222
22.06.2010	178	26	122	193	171	232	67,83098598
22.06.2010	179	54	153	193	171	232	109,2245748
22.06.2010	180	67	166	193	171	232	45,1930066
22.06.2010	181	60	156	193	171	232	8,523945388
22.06.2010	182	13	96	193	171	232	97,99638815
22.06.2010	191	128	203	193	171	232	106,3141938
22.06.2010	170	49	148	194	81	184	256,9569891
22.06.2010	172	19	106	194	81	184	243,6746555
22.06.2010	173	16	105	194	81	184	354,7406228

22.06.2010	174	125	211	194	81	184	0,095039606
22.06.2010	175	52	150	194	81	184	298,7906613
22.06.2010	176	37	136	194	81	184	268,2661532
22.06.2010	177	13	97	194	81	184	293,5974181
22.06.2010	178	26	122	194	81	184	278,0251087
22.06.2010	179	54	153	194	81	184	326,4453727
22.06.2010	180	67	166	194	81	184	262,4096534
22.06.2010	181	60	156	194	81	184	239,4532636
22.06.2010	182	13	96	194	81	184	316,7203964
22.06.2010	191	128	203	194	81	184	324,7478725
22.06.2010	193	171	232	194	81	184	232,1129284
22.06.2010	170	49	148	172	19	106	12,49111151
22.06.2010	170	49	148	173	16	105	34,86023732
22.06.2010	172	19	106	173	16	105	41,31337898
22.06.2010	170	49	148	174	125	211	69,91905634
22.06.2010	172	19	106	174	125	211	57,52872259
22.06.2010	173	16	105	174	125	211	92,12470956
22.06.2010	170	49	148	175	52	150	48,19595144
22.06.2010	172	19	106	175	52	150	58,34920993
22.06.2010	173	16	105	175	52	150	23,66428938
22.06.2010	174	125	211	175	52	150	113,3785058
22.06.2010	170	49	148	176	37	136	11,5107834
22.06.2010	172	19	106	176	37	136	23,77472308
22.06.2010	173	16	105	176	37	136	29,12898034
22.06.2010	174	125	211	176	37	136	81,30292519
22.06.2010	175	52	150	176	37	136	37,96365363
22.06.2010	170	49	148	177	13	97	38,86492438
22.06.2010	172	19	106	177	13	97	49,90927795
22.06.2010	173	16	105	177	13	97	22,7690778
22.06.2010	174	125	211	177	13	97	106,3644786
22.06.2010	175	52	150	177	13	97	11,05731802
22.06.2010	176	37	136	177	13	97	27,98638044
22.06.2010	170	49	148	178	26	122	28,0651812

22.06.2010	172	19	106	178	26	122	39,04127073
22.06.2010	173	16	105	178	26	122	19,13744657
22.06.2010	174	125	211	178	26	122	95,69478426
22.06.2010	175	52	150	178	26	122	20,45406006
22.06.2010	176	37	136	178	26	122	17,51309446
22.06.2010	177	13	97	178	26	122	10,87487344
22.06.2010	170	49	148	179	54	153	96,12883836
22.06.2010	172	19	106	179	54	153	104,3556514
22.06.2010	173	16	105	179	54	153	63,29851815
22.06.2010	174	125	211	179	54	153	153,7649779
22.06.2010	175	52	150	179	54	153	49,93478946
22.06.2010	176	37	136	179	54	153	87,0461106
22.06.2010	177	13	97	179	54	153	60,95003384
22.06.2010	178	26	122	179	54	153	69,86348451
22.06.2010	170	49	148	180	67	166	6,852085371
22.06.2010	172	19	106	180	67	166	5,71345356
22.06.2010	173	16	105	180	67	166	38,58994233
22.06.2010	174	125	211	180	67	166	63,06785581
22.06.2010	175	52	150	180	67	166	54,01043202
22.06.2010	176	37	136	180	67	166	18,29654354
22.06.2010	177	13	97	180	67	166	45,11569242
22.06.2010	178	26	122	180	67	166	34,24829712
22.06.2010	179	54	153	180	67	166	101,0219602
22.06.2010	170	49	148	181	60	156	27,43568082
22.06.2010	172	19	106	181	60	156	15,03128815
22.06.2010	173	16	105	181	60	156	50,48329967
22.06.2010	174	125	211	181	60	156	43,39492179
22.06.2010	175	52	150	181	60	156	70,38541087
22.06.2010	176	37	136	181	60	156	38,41493354
22.06.2010	177	13	97	181	60	156	63,00086527
22.06.2010	178	26	122	181	60	156	52,30253258
22.06.2010	179	54	153	181	60	156	113,5853319
22.06.2010	180	67	166	181	60	156	20,74172722

22.06.2010	170	49	148	182	13	96	48,40437209
22.06.2010	172	19	106	182	13	96	58,58095151
22.06.2010	173	16	105	182	13	96	23,94262535
22.06.2010	174	125	211	182	13	96	113,6367709
22.06.2010	175	52	150	182	13	96	0,285118818
22.06.2010	176	37	136	182	13	96	38,1481923
22.06.2010	177	13	97	182	13	96	11,15450831
22.06.2010	178	26	122	182	13	96	20,63651061
22.06.2010	179	54	153	182	13	96	49,81680254
22.06.2010	180	67	166	182	13	96	54,23148467
22.06.2010	181	60	156	182	13	96	70,63629712
22.06.2010	170	49	148	191	128	203	77,48928263
22.06.2010	172	19	106	191	128	203	86,10929231
22.06.2010	173	16	105	191	128	203	45,53646959
22.06.2010	174	125	211	191	128	203	137,1727413
22.06.2010	175	52	150	191	128	203	31,12369349
22.06.2010	176	37	136	191	128	203	68,22652309
22.06.2010	177	13	97	191	128	203	42,16632176
22.06.2010	178	26	122	191	128	203	51,00236506
22.06.2010	179	54	153	191	128	203	18,86530196
22.06.2010	180	67	166	191	128	203	82,56261852
22.06.2010	181	60	156	191	128	203	96,01992086
22.06.2010	182	13	96	191	128	203	31,01525482
22.06.2010	170	49	148	193	171	232	43,66239905
22.06.2010	172	19	106	193	171	232	31,19529395
22.06.2010	173	16	105	193	171	232	66,63394805
22.06.2010	174	125	211	193	171	232	26,48503338
22.06.2010	175	52	150	193	171	232	87,18825929
22.06.2010	176	37	136	193	171	232	54,94670257
22.06.2010	177	13	97	193	171	232	79,92887376
22.06.2010	178	26	122	193	171	232	69,22857397
22.06.2010	179	54	153	193	171	232	129,2454432
22.06.2010	180	67	166	193	171	232	36,81947679

22.06.2010	181	60	156	193	171	232	16,92879228
22.06.2010	182	13	96	193	171	232	87,44227408
22.06.2010	191	128	203	193	171	232	112,0673329
22.06.2010	170	49	148	194	81	184	50,35484984
22.06.2010	172	19	106	194	81	184	37,87754586
22.06.2010	173	16	105	194	81	184	72,34380855
22.06.2010	174	125	211	194	81	184	20,06033725
22.06.2010	175	52	150	194	81	184	93,34408667
22.06.2010	176	37	136	194	81	184	61,6072942
22.06.2010	177	13	97	194	81	184	86,31684131
22.06.2010	178	26	122	194	81	184	75,66979191
22.06.2010	179	54	153	194	81	184	134,5311726
22.06.2010	180	67	166	194	81	184	43,51651748
22.06.2010	181	60	156	194	81	184	23,40426282
22.06.2010	182	13	96	194	81	184	93,60141961
22.06.2010	191	128	203	194	81	184	117,6133461
22.06.2010	193	171	232	194	81	184	6,714936591
23.06.2010	170	49	148	172	19	106	15,46256714
23.06.2010	170	49	148	173	16	105	99,69894767
23.06.2010	172	19	106	173	16	105	93,66436463
23.06.2010	170	49	148	174	125	211	240,6351778
23.06.2010	172	19	106	174	125	211	246,6295541
23.06.2010	173	16	105	174	125	211	339,9468667
23.06.2010	170	49	148	175	52	150	51,22290922
23.06.2010	172	19	106	175	52	150	42,6100829
23.06.2010	173	16	105	175	52	150	51,61814409
23.06.2010	174	125	211	175	52	150	289,2328326
23.06.2010	170	49	148	176	37	136	13,16427202
23.06.2010	172	19	106	176	37	136	5,372890787
23.06.2010	173	16	105	176	37	136	90,5643173
23.06.2010	174	125	211	176	37	136	249,4231851
23.06.2010	175	52	150	176	37	136	40,22073435
23.06.2010	170	49	148	177	13	97	48,8006861

23.06.2010	172	19	106	177	13	97	37,24994887
23.06.2010	173	16	105	177	13	97	60,84940004
23.06.2010	174	125	211	177	13	97	282,5492165
23.06.2010	175	52	150	177	13	97	11,91866384
23.06.2010	176	37	136	177	13	97	36,29505194
23.06.2010	170	49	148	178	26	122	33,79857475
23.06.2010	172	19	106	178	26	122	21,74746265
23.06.2010	173	16	105	178	26	122	73,79120079
23.06.2010	174	125	211	178	26	122	267,6673707
23.06.2010	175	52	150	178	26	122	22,17703948
23.06.2010	176	37	136	178	26	122	20,96135086
23.06.2010	177	13	97	178	26	122	15,51330532
23.06.2010	170	49	148	179	54	153	77,34869445
23.06.2010	172	19	106	179	54	153	69,84777422
23.06.2010	173	16	105	179	54	153	24,81061661
23.06.2010	174	125	211	179	54	153	316,4710713
23.06.2010	175	52	150	179	54	153	27,30268056
23.06.2010	176	37	136	179	54	153	67,18298598
23.06.2010	177	13	97	179	54	153	36,04119405
23.06.2010	178	26	122	179	54	153	49,33416148
23.06.2010	170	49	148	180	67	166	6,279809844
23.06.2010	172	19	106	180	67	166	9,185960193
23.06.2010	173	16	105	180	67	166	96,86923215
23.06.2010	174	125	211	180	67	166	243,0974908
23.06.2010	175	52	150	180	67	166	47,19403109
23.06.2010	176	37	136	180	67	166	7,469532621
23.06.2010	177	13	97	180	67	166	43,73164625
23.06.2010	178	26	122	180	67	166	28,42971256
23.06.2010	179	54	153	180	67	166	73,88502697
23.06.2010	170	49	148	181	60	156	27,7525414
23.06.2010	172	19	106	181	60	156	41,55013755
23.06.2010	173	16	105	181	60	156	124,5970323
23.06.2010	174	125	211	181	60	156	218,6515391

23.06.2010	175	52	150	181	60	156	78,33604581
23.06.2010	176	37	136	181	60	156	40,66162672
23.06.2010	177	13	97	181	60	156	76,54388628
23.06.2010	178	26	122	181	60	156	61,51977644
23.06.2010	179	54	153	181	60	156	103,5335139
23.06.2010	180	67	166	181	60	156	33,24240341
23.06.2010	170	49	148	182	13	96	77,45628775
23.06.2010	172	19	106	182	13	96	69,51599858
23.06.2010	173	16	105	182	13	96	26,0231562
23.06.2010	174	125	211	182	13	96	316,1355749
23.06.2010	175	52	150	182	13	96	26,90577795
23.06.2010	176	37	136	182	13	96	67,01640174
23.06.2010	177	13	97	182	13	96	35,01909652
23.06.2010	178	26	122	182	13	96	48,72705722
23.06.2010	179	54	153	182	13	96	2,411834823
23.06.2010	180	67	166	182	13	96	73,82736307
23.06.2010	181	60	156	182	13	96	103,8780721
23.06.2010	170	49	148	191	128	203	65,66067594
23.06.2010	172	19	106	191	128	203	57,77204299
23.06.2010	173	16	105	191	128	203	36,60443982
23.06.2010	174	125	211	191	128	203	304,4013097
23.06.2010	175	52	150	191	128	203	15,20425784
23.06.2010	176	37	136	191	128	203	55,19780539
23.06.2010	177	13	97	191	128	203	24,53273977
23.06.2010	178	26	122	191	128	203	37,27140255
23.06.2010	179	54	153	191	128	203	12,09880689
23.06.2010	180	67	166	191	128	203	61,99686361
23.06.2010	181	60	156	191	128	203	92,22975577
23.06.2010	182	13	96	191	128	203	11,83080869
23.06.2010	170	49	148	193	171	232	37,76960519
23.06.2010	172	19	106	193	171	232	51,31117832
23.06.2010	173	16	105	193	171	232	134,0445289
23.06.2010	174	125	211	193	171	232	210,7162027

23.06.2010	175	52	150	193	171	232	88,29593724
23.06.2010	176	37	136	193	171	232	50,62248732
23.06.2010	177	13	97	193	171	232	86,56642232
23.06.2010	178	26	122	193	171	232	71,51425657
23.06.2010	179	54	153	193	171	232	113,299329
23.06.2010	180	67	166	193	171	232	43,18104396
23.06.2010	181	60	156	193	171	232	10,02656584
23.06.2010	182	13	96	193	171	232	113,6914441
23.06.2010	191	128	203	193	171	232	102,084571
23.06.2010	170	49	148	194	81	184	240,8879263
23.06.2010	172	19	106	194	81	184	246,8661772
23.06.2010	173	16	105	194	81	184	340,1912133
23.06.2010	174	125	211	194	81	184	0,368086811
23.06.2010	175	52	150	194	81	184	289,4686267
23.06.2010	176	37	136	194	81	184	249,6650113
23.06.2010	177	13	97	194	81	184	282,7750759
23.06.2010	178	26	122	194	81	184	267,8978871
23.06.2010	179	54	153	194	81	184	316,7086036
23.06.2010	180	67	166	194	81	184	243,3437474
23.06.2010	181	60	156	194	81	184	218,9233974
23.06.2010	182	13	96	194	81	184	316,3709894
23.06.2010	191	128	203	194	81	184	304,6380979
23.06.2010	193	171	232	194	81	184	210,995032
23.06.2010	170	49	148	172	19	106	18,25082157
23.06.2010	170	49	148	173	16	105	77,38010101
23.06.2010	172	19	106	173	16	105	92,37561265
23.06.2010	170	49	148	174	125	211	197,4504028
23.06.2010	172	19	106	174	125	211	181,2875691
23.06.2010	173	16	105	174	125	211	273,6097331
23.06.2010	170	49	148	175	52	150	53,46153135
23.06.2010	172	19	106	175	52	150	69,61688449
23.06.2010	173	16	105	175	52	150	24,99126033
23.06.2010	174	125	211	175	52	150	250,7139045

23.06.2010	170	49	148	176	37	136	13,34587556
23.06.2010	172	19	106	176	37	136	31,06385191
23.06.2010	173	16	105	176	37	136	64,74373928
23.06.2010	174	125	211	176	37	136	210,7468281
23.06.2010	175	52	150	176	37	136	40,4210048
23.06.2010	170	49	148	177	13	97	37,78359276
23.06.2010	172	19	106	177	13	97	54,72408248
23.06.2010	173	16	105	177	13	97	41,15642929
23.06.2010	174	125	211	177	13	97	235,2338728
23.06.2010	175	52	150	177	13	97	16,25149475
23.06.2010	176	37	136	177	13	97	24,52224433
23.06.2010	170	49	148	178	26	122	36,36479125
23.06.2010	172	19	106	178	26	122	53,2557721
23.06.2010	173	16	105	178	26	122	42,31184184
23.06.2010	174	125	211	178	26	122	233,8123626
23.06.2010	175	52	150	178	26	122	17,48909525
23.06.2010	176	37	136	178	26	122	23,12882104
23.06.2010	177	13	97	178	26	122	1,481520837
23.06.2010	170	49	148	179	54	153	51,62164372
23.06.2010	172	19	106	179	54	153	69,68205145
23.06.2010	173	16	105	179	54	153	57,55838964
23.06.2010	174	125	211	179	54	153	241,1107605
23.06.2010	175	52	150	179	54	153	37,35854477
23.06.2010	176	37	136	179	54	153	40,75902901
23.06.2010	177	13	97	179	54	153	30,69705716
23.06.2010	178	26	122	179	54	153	31,32849109
23.06.2010	170	49	148	180	67	166	9,752564094
23.06.2010	172	19	106	180	67	166	8,592021934
23.06.2010	173	16	105	180	67	166	85,7247764
23.06.2010	174	125	211	180	67	166	188,3763871
23.06.2010	175	52	150	180	67	166	62,33917199
23.06.2010	176	37	136	180	67	166	22,88225315
23.06.2010	177	13	97	180	67	166	47,01483572

23.06.2010	178	26	122	180	67	166	45,56690739
23.06.2010	179	54	153	180	67	166	61,09154455
23.06.2010	170	49	148	181	60	156	43,37160305
23.06.2010	172	19	106	181	60	156	25,35652416
23.06.2010	173	16	105	181	60	156	112,1459298
23.06.2010	174	125	211	181	60	156	163,0223481
23.06.2010	175	52	150	181	60	156	91,33963784
23.06.2010	176	37	136	181	60	156	55,47920216
23.06.2010	177	13	97	181	60	156	77,71915724
23.06.2010	178	26	122	181	60	156	76,23616087
23.06.2010	179	54	153	181	60	156	94,9688703
23.06.2010	180	67	166	181	60	156	33,91436109
23.06.2010	170	49	148	182	13	96	53,41352693
23.06.2010	172	19	106	182	13	96	69,57003034
23.06.2010	173	16	105	182	13	96	25,03766068
23.06.2010	174	125	211	182	13	96	250,66638
23.06.2010	175	52	150	182	13	96	0
23.06.2010	176	37	136	182	13	96	40,37270833
23.06.2010	177	13	97	182	13	96	16,20390445
23.06.2010	178	26	122	182	13	96	17,44125669
23.06.2010	179	54	153	182	13	96	37,33532673
23.06.2010	180	67	166	182	13	96	62,29177387
23.06.2010	181	60	156	182	13	96	91,29522568
23.06.2010	170	49	148	191	128	203	76,389676
23.06.2010	172	19	106	191	128	203	91,49772416
23.06.2010	173	16	105	191	128	203	1,310030564
23.06.2010	174	125	211	191	128	203	272,7556887
23.06.2010	175	52	150	191	128	203	23,82747364
23.06.2010	176	37	136	191	128	203	63,7036237
23.06.2010	177	13	97	191	128	203	40,02307278
23.06.2010	178	26	122	191	128	203	41,19350272
23.06.2010	179	54	153	191	128	203	56,24859491
23.06.2010	180	67	166	191	128	203	84,78923183

23.06.2010	181	60	156	191	128	203	111,4295914
23.06.2010	182	13	96	191	128	203	23,87424416
23.06.2010	170	49	148	193	171	232	31,55801533
23.06.2010	172	19	106	193	171	232	13,34689073
23.06.2010	173	16	105	193	171	232	103,3289025
23.06.2010	174	125	211	193	171	232	170,4914379
23.06.2010	175	52	150	193	171	232	81,47261571
23.06.2010	176	37	136	193	171	232	44,10144952
23.06.2010	177	13	97	193	171	232	67,157907
23.06.2010	178	26	122	193	171	232	65,67731904
23.06.2010	179	54	153	193	171	232	83,02551414
23.06.2010	180	67	166	193	171	232	21,93830335
23.06.2010	181	60	156	193	171	232	12,17953966
23.06.2010	182	13	96	193	171	232	81,42681526
23.06.2010	191	128	203	193	171	232	102,5293684
23.06.2010	170	49	148	194	81	184	49,73596118
23.06.2010	172	19	106	194	81	184	31,7584482
23.06.2010	173	16	105	194	81	184	117,3688729
23.06.2010	174	125	211	194	81	184	158,8694404
23.06.2010	175	52	150	194	81	184	97,01844835
23.06.2010	176	37	136	194	81	184	61,72703524
23.06.2010	177	13	97	194	81	184	83,67496558
23.06.2010	178	26	122	194	81	184	82,19360097
23.06.2010	179	54	153	194	81	184	101,3462272
23.06.2010	180	67	166	194	81	184	40,30979159
23.06.2010	181	60	156	194	81	184	6,403723241
23.06.2010	182	13	96	194	81	184	96,97458781
23.06.2010	191	128	203	194	81	184	116,6882025
23.06.2010	193	171	232	194	81	184	18,56705187
23.06.2010	170	49	148	172	19	106	30,9535987
23.06.2010	170	49	148	173	16	105	66,47719832
23.06.2010	172	19	106	173	16	105	94,25370556
23.06.2010	170	49	148	174	125	211	259,4877663

23.06.2010	172	19	106	174	125	211	238,0126667
23.06.2010	173	16	105	174	125	211	324,8089741
23.06.2010	170	49	148	175	52	150	43,80923811
23.06.2010	172	19	106	175	52	150	71,97931092
23.06.2010	173	16	105	175	52	150	22,6696855
23.06.2010	174	125	211	175	52	150	302,5171872
23.06.2010	170	49	148	176	37	136	9,2750086
23.06.2010	172	19	106	176	37	136	39,52345174
23.06.2010	173	16	105	176	37	136	57,26086973
23.06.2010	174	125	211	176	37	136	268,3252713
23.06.2010	175	52	150	176	37	136	34,60459793
23.06.2010	170	49	148	177	13	97	31,5851945
23.06.2010	172	19	106	177	13	97	61,31977186
23.06.2010	173	16	105	177	13	97	35,61006076
23.06.2010	174	125	211	177	13	97	289,2156092
23.06.2010	175	52	150	177	13	97	13,60029714
23.06.2010	176	37	136	177	13	97	22,32682434
23.06.2010	170	49	148	178	26	122	18,67981638
23.06.2010	172	19	106	178	26	122	47,71499368
23.06.2010	173	16	105	178	26	122	47,80558317
23.06.2010	174	125	211	178	26	122	277,8736076
23.06.2010	175	52	150	178	26	122	25,13613424
23.06.2010	176	37	136	178	26	122	9,558924645
23.06.2010	177	13	97	178	26	122	13,62948806
23.06.2010	170	49	148	179	54	153	86,07083931
23.06.2010	172	19	106	179	54	153	109,8361424
23.06.2010	173	16	105	179	54	153	27,34697575
23.06.2010	174	125	211	179	54	153	345,4970475
23.06.2010	175	52	150	179	54	153	44,90598759
23.06.2010	176	37	136	179	54	153	77,45010693
23.06.2010	177	13	97	179	54	153	58,44356236
23.06.2010	178	26	122	179	54	153	68,00341169
23.06.2010	170	49	148	180	67	166	12,15541329

23.06.2010	172	19	106	180	67	166	18,80944017
23.06.2010	173	16	105	180	67	166	76,90642274
23.06.2010	174	125	211	180	67	166	251,1159342
23.06.2010	175	52	150	180	67	166	54,33373634
23.06.2010	176	37	136	180	67	166	20,82733933
23.06.2010	177	13	97	180	67	166	42,93665998
23.06.2010	178	26	122	180	67	166	29,47729645
23.06.2010	179	54	153	180	67	166	94,63788
23.06.2010	170	49	148	181	60	156	50,89031468
23.06.2010	172	19	106	181	60	156	20,46906898
23.06.2010	173	16	105	181	60	156	114,663148
23.06.2010	174	125	211	181	60	156	220,8885041
23.06.2010	175	52	150	181	60	156	92,43939236
23.06.2010	176	37	136	181	60	156	59,7422018
23.06.2010	177	13	97	181	60	156	81,72013565
23.06.2010	178	26	122	181	60	156	68,13895694
23.06.2010	179	54	153	181	60	156	129,5042189
23.06.2010	180	67	166	181	60	156	38,91520822
23.06.2010	170	49	148	182	13	96	66,70724269
23.06.2010	172	19	106	182	13	96	94,54372633
23.06.2010	173	16	105	182	13	96	0,425030039
23.06.2010	174	125	211	182	13	96	324,9735184
23.06.2010	175	52	150	182	13	96	22,90513659
23.06.2010	176	37	136	182	13	96	57,48490039
23.06.2010	177	13	97	182	13	96	35,78831174
23.06.2010	178	26	122	182	13	96	48,03967713
23.06.2010	179	54	153	182	13	96	27,50095444
23.06.2010	180	67	166	182	13	96	77,16777088
23.06.2010	181	60	156	182	13	96	114,9578584
23.06.2010	170	49	148	191	128	203	54,28317538
23.06.2010	172	19	106	191	128	203	80,85351704
23.06.2010	173	16	105	191	128	203	14,15675468
23.06.2010	174	125	211	191	128	203	313,5915443

23.06.2010	175	52	150	191	128	203	11,87424702
23.06.2010	176	37	136	191	128	203	45,2876438
23.06.2010	177	13	97	191	128	203	25,38215911
23.06.2010	178	26	122	191	128	203	35,73021543
23.06.2010	179	54	153	191	128	203	33,06190084
23.06.2010	180	67	166	191	128	203	64,03060265
23.06.2010	181	60	156	191	128	203	101,1705423
23.06.2010	182	13	96	191	128	203	14,53204992
23.06.2010	170	49	148	193	171	232	36,31284135
23.06.2010	172	19	106	193	171	232	5,390513748
23.06.2010	173	16	105	193	171	232	99,4683355
23.06.2010	174	125	211	193	171	232	234,0406793
23.06.2010	175	52	150	193	171	232	77,25708725
23.06.2010	176	37	136	193	171	232	44,91282594
23.06.2010	177	13	97	193	171	232	66,69194011
23.06.2010	178	26	122	193	171	232	53,08087499
23.06.2010	179	54	153	193	171	232	114,6338402
23.06.2010	180	67	166	193	171	232	24,18099704
23.06.2010	181	60	156	193	171	232	15,19534405
23.06.2010	182	13	96	193	171	232	99,76279882
23.06.2010	191	128	203	193	171	232	85,99409181
23.06.2010	170	49	148	194	81	184	256,8848296
23.06.2010	172	19	106	194	81	184	235,5627528
23.06.2010	173	16	105	194	81	184	322,1402743
23.06.2010	174	125	211	194	81	184	3,042752026
23.06.2010	175	52	150	194	81	184	299,8682567
23.06.2010	176	37	136	194	81	184	265,7054309
23.06.2010	177	13	97	194	81	184	286,5525608
23.06.2010	178	26	122	194	81	184	275,2511076
23.06.2010	179	54	153	194	81	184	342,910248
23.06.2010	180	67	166	194	81	184	248,5694173
23.06.2010	181	60	156	194	81	184	218,5308389
23.06.2010	182	13	96	194	81	184	322,3029722

23.06.2010	191	128	203	194	81	184	310,9636366
23.06.2010	193	171	232	194	81	184	231,6191832
25.06.2009	106	25	122	112	17	111	81,57
25.06.2009	106	25	122	112	17	111	108,25
25.06.2009	106	25	122	112	17	111	3,92
25.06.2009	106	25	122	112	17	111	26,70
25.06.2009	106	25	122	112	17	111	15,32
26.06.2009	106	25	122	112	17	111	6,07
26.06.2009	106	25	122	112	17	111	81,99
26.06.2009	106	25	122	112	17	111	93,28
17.06.2009	106	25	122	112	17	111	895,55
10.06.2009	106	25	122	112	17	111	880,99
10.06.2009	106	25	122	112	17	111	897,16
10.06.2009	106	25	122	112	17	111	898,31
10.06.2009	106	25	122	112	17	111	894,16
10.06.2009	106	25	122	112	17	111	953,74
10.06.2009	106	25	122	112	17	111	933,02
11.06.2009	106	25	122	112	17	111	922,30
11.06.2009	106	25	122	112	17	111	896,96
11.06.2009	106	25	122	112	17	111	907,11
11.06.2009	106	25	122	112	17	111	901,70
11.06.2009	106	25	122	112	17	111	923,76
11.06.2009	106	25	122	112	17	111	897,55
11.06.2009	106	25	122	112	17	111	932,80
12.06.2009	106	25	122	112	17	111	921,40
12.06.2009	106	25	122	112	17	111	898,02
12.06.2009	106	25	122	112	17	111	889,98
03.06.2009	106	25	122	112	17	111	815,52
03.06.2009	106	25	122	112	17	111	899,37
04.06.2009	106	25	122	112	17	111	898,23
04.06.2009	106	25	122	112	17	111	889,88
04.06.2009	106	25	122	112	17	111	874,18
04.06.2009	106	25	122	112	17	111	913,63

04.06.2009	106	25	122	112	17	111	712,14
04.06.2009	106	25	122	112	17	111	892,71
04.06.2009	106	25	122	112	17	111	802,78
04.06.2009	106	25	122	112	17	111	866,40
05.06.2009	106	25	122	112	17	111	883,74
05.06.2009	106	25	122	112	17	111	852,97
05.06.2009	106	25	122	112	17	111	898,44
25.06.2009	106	25	122	113	19	106	22,70
25.06.2009	106	25	122	113	19	106	17,67
25.06.2009	106	25	122	113	19	106	83,08
25.06.2009	106	25	122	113	19	106	110,05
25.06.2009	106	25	122	113	19	106	118,39
26.06.2009	106	25	122	113	19	106	104,79
26.06.2009	106	25	122	113	19	106	155,56
26.06.2009	106	25	122	113	19	106	24,47
17.06.2009	106	25	122	113	19	106	1023,64
10.06.2009	106	25	122	113	19	106	976,89
10.06.2009	106	25	122	113	19	106	933,98
10.06.2009	106	25	122	113	19	106	945,98
10.06.2009	106	25	122	113	19	106	1012,33
10.06.2009	106	25	122	113	19	106	1023,39
10.06.2009	106	25	122	113	19	106	1018,56
11.06.2009	106	25	122	113	19	106	957,20
11.06.2009	106	25	122	113	19	106	960,70
11.06.2009	106	25	122	113	19	106	1012,85
11.06.2009	106	25	122	113	19	106	954,43
11.06.2009	106	25	122	113	19	106	1025,25
11.06.2009	106	25	122	113	19	106	1011,46
11.06.2009	106	25	122	113	19	106	983,13
11.06.2009	106	25	122	113	19	106	1018,82
12.06.2009	106	25	122	113	19	106	995,45
12.06.2009	106	25	122	113	19	106	960,60
12.06.2009	106	25	122	113	19	106	1006,02

03.06.2009	106	25	122	113	19	106	914,77
03.06.2009	106	25	122	113	19	106	997,99
04.06.2009	106	25	122	113	19	106	1016,62
04.06.2009	106	25	122	113	19	106	1012,23
04.06.2009	106	25	122	113	19	106	986,55
04.06.2009	106	25	122	113	19	106	977,39
04.06.2009	106	25	122	113	19	106	858,80
04.06.2009	106	25	122	113	19	106	968,86
04.06.2009	106	25	122	113	19	106	1015,53
04.06.2009	106	25	122	113	19	106	1031,81
05.06.2009	106	25	122	113	19	106	1005,50
05.06.2009	106	25	122	113	19	106	957,27
05.06.2009	106	25	122	113	19	106	1004,08
24.06.2009	112	17	111	113	19	106	0,00
24.06.2009	112	17	111	113	19	106	81,77
24.06.2009	112	17	111	113	19	106	84,39
24.06.2009	112	17	111	113	19	106	82,56
24.06.2009	112	17	111	113	19	106	90,93
24.06.2009	112	17	111	113	19	106	99,63
25.06.2009	112	17	111	113	19	106	79,96
25.06.2009	112	17	111	113	19	106	125,58
25.06.2009	112	17	111	113	19	106	95,34
25.06.2009	112	17	111	113	19	106	88,01
25.06.2009	112	17	111	113	19	106	92,33
25.06.2009	112	17	111	113	19	106	80,64
25.06.2009	112	17	111	113	19	106	87,40
25.06.2009	112	17	111	113	19	106	105,39
26.06.2009	112	17	111	113	19	106	100,47
26.06.2009	112	17	111	113	19	106	85,63
26.06.2009	112	17	111	113	19	106	102,29
17.06.2009	112	17	111	113	19	106	136,80
17.06.2009	112	17	111	113	19	106	113,42
17.06.2009	112	17	111	113	19	106	56,28

17.06.2009	112	17	111	113	19	106	103,36
17.06.2009	112	17	111	113	19	106	112,51
17.06.2009	112	17	111	113	19	106	36,67
18.06.2009	112	17	111	113	19	106	66,37
18.06.2009	112	17	111	113	19	106	108,03
18.06.2009	112	17	111	113	19	106	45,51
18.06.2009	112	17	111	113	19	106	84,76
18.06.2009	112	17	111	113	19	106	87,71
18.06.2009	112	17	111	113	19	106	57,05
18.06.2009	112	17	111	113	19	106	129,83
18.06.2009	112	17	111	113	19	106	54,27
19.06.2009	112	17	111	113	19	106	84,00
19.06.2009	112	17	111	113	19	106	56,34
19.06.2009	112	17	111	113	19	106	26,91
10.06.2009	112	17	111	113	19	106	95,90
10.06.2009	112	17	111	113	19	106	47,35
10.06.2009	112	17	111	113	19	106	54,15
10.06.2009	112	17	111	113	19	106	127,37
10.06.2009	112	17	111	113	19	106	70,69
10.06.2009	112	17	111	113	19	106	85,73
11.06.2009	112	17	111	113	19	106	47,85
11.06.2009	112	17	111	113	19	106	72,88
11.06.2009	112	17	111	113	19	106	107,02
11.06.2009	112	17	111	113	19	106	60,18
11.06.2009	112	17	111	113	19	106	90,76
11.06.2009	112	17	111	113	19	106	85,65
11.06.2009	112	17	111	113	19	106	86,17
12.06.2009	112	17	111	113	19	106	74,04
12.06.2009	112	17	111	113	19	106	78,44
12.06.2009	112	17	111	113	19	106	121,78
03.06.2009	112	17	111	113	19	106	124,38
03.06.2009	112	17	111	113	19	106	57,68
03.06.2009	112	17	111	113	19	106	66,67

03.06.2009	112	17	111	113	19	106	99,64
03.06.2009	112	17	111	113	19	106	99,63
03.06.2009	112	17	111	113	19	106	100,55
04.06.2009	112	17	111	113	19	106	126,60
04.06.2009	112	17	111	113	19	106	127,06
04.06.2009	112	17	111	113	19	106	120,98
04.06.2009	112	17	111	113	19	106	64,29
04.06.2009	112	17	111	113	19	106	150,00
04.06.2009	112	17	111	113	19	106	80,52
04.06.2009	112	17	111	113	19	106	215,27
04.06.2009	112	17	111	113	19	106	171,64
05.06.2009	112	17	111	113	19	106	132,60
05.06.2009	112	17	111	113	19	106	114,13
05.06.2009	112	17	111	113	19	106	112,46
17.06.2009	106	25	122	114	17	108	614,06
10.06.2009	106	25	122	114	17	108	590,84
03.06.2009	106	25	122	114	17	108	739,63
03.06.2009	106	25	122	114	17	108	836,55
04.06.2009	106	25	122	114	17	108	817,06
04.06.2009	106	25	122	114	17	108	795,39
04.06.2009	106	25	122	114	17	108	780,39
04.06.2009	106	25	122	114	17	108	835,06
04.06.2009	106	25	122	114	17	108	711,05
04.06.2009	106	25	122	114	17	108	611,66
04.06.2009	106	25	122	114	17	108	616,42
04.06.2009	106	25	122	114	17	108	667,24
05.06.2009	106	25	122	114	17	108	612,09
05.06.2009	106	25	122	114	17	108	874,04
05.06.2009	106	25	122	114	17	108	599,80
17.06.2009	112	17	111	114	17	108	281,50
17.06.2009	112	17	111	114	17	108	91,76
17.06.2009	112	17	111	114	17	108	210,23
17.06.2009	112	17	111	114	17	108	104,50

17.06.2009	112	17	111	114	17	108	167,31
17.06.2009	112	17	111	114	17	108	119,73
18.06.2009	112	17	111	114	17	108	176,85
18.06.2009	112	17	111	114	17	108	149,65
18.06.2009	112	17	111	114	17	108	57,95
18.06.2009	112	17	111	114	17	108	80,66
18.06.2009	112	17	111	114	17	108	92,96
18.06.2009	112	17	111	114	17	108	134,76
18.06.2009	112	17	111	114	17	108	207,42
18.06.2009	112	17	111	114	17	108	178,55
19.06.2009	112	17	111	114	17	108	89,10
19.06.2009	112	17	111	114	17	108	153,10
19.06.2009	112	17	111	114	17	108	62,67
10.06.2009	112	17	111	114	17	108	290,82
03.06.2009	112	17	111	114	17	108	53,48
03.06.2009	112	17	111	114	17	108	46,27
03.06.2009	112	17	111	114	17	108	64,58
03.06.2009	112	17	111	114	17	108	59,05
03.06.2009	112	17	111	114	17	108	80,50
03.06.2009	112	17	111	114	17	108	64,55
04.06.2009	112	17	111	114	17	108	83,77
04.06.2009	112	17	111	114	17	108	96,09
04.06.2009	112	17	111	114	17	108	96,96
04.06.2009	112	17	111	114	17	108	93,78
04.06.2009	112	17	111	114	17	108	5,77
04.06.2009	112	17	111	114	17	108	281,05
04.06.2009	112	17	111	114	17	108	186,39
04.06.2009	112	17	111	114	17	108	199,27
05.06.2009	112	17	111	114	17	108	273,81
05.06.2009	112	17	111	114	17	108	22,50
05.06.2009	112	17	111	114	17	108	298,64
17.06.2009	113	19	106	114	17	108	411,70
17.06.2009	113	19	106	114	17	108	51,29

17.06.2009	113	19	106	114	17	108	154,07
17.06.2009	113	19	106	114	17	108	7,63
17.06.2009	113	19	106	114	17	108	56,75
17.06.2009	113	19	106	114	17	108	97,15
18.06.2009	113	19	106	114	17	108	111,77
18.06.2009	113	19	106	114	17	108	45,05
18.06.2009	113	19	106	114	17	108	42,01
18.06.2009	113	19	106	114	17	108	71,73
18.06.2009	113	19	106	114	17	108	42,69
18.06.2009	113	19	106	114	17	108	77,81
18.06.2009	113	19	106	114	17	108	77,61
18.06.2009	113	19	106	114	17	108	125,60
19.06.2009	113	19	106	114	17	108	48,94
19.06.2009	113	19	106	114	17	108	106,67
19.06.2009	113	19	106	114	17	108	39,18
10.06.2009	113	19	106	114	17	108	386,63
03.06.2009	113	19	106	114	17	108	74,05
03.06.2009	113	19	106	114	17	108	32,54
03.06.2009	113	19	106	114	17	108	38,53
03.06.2009	113	19	106	114	17	108	76,84
03.06.2009	113	19	106	114	17	108	176,29
03.06.2009	113	19	106	114	17	108	165,09
04.06.2009	113	19	106	114	17	108	209,81
04.06.2009	113	19	106	114	17	108	222,76
04.06.2009	113	19	106	114	17	108	217,22
04.06.2009	113	19	106	114	17	108	154,78
04.06.2009	113	19	106	114	17	108	152,48
04.06.2009	113	19	106	114	17	108	357,96
04.06.2009	113	19	106	114	17	108	399,88
04.06.2009	113	19	106	114	17	108	367,66
05.06.2009	113	19	106	114	17	108	401,52
05.06.2009	113	19	106	114	17	108	91,72
05.06.2009	113	19	106	114	17	108	405,63

25.06.2009	106	25	122	115	14	98	80,28
25.06.2009	106	25	122	115	14	98	88,64
25.06.2009	106	25	122	115	14	98	0,74
25.06.2009	106	25	122	115	14	98	43,35
25.06.2009	106	25	122	115	14	98	43,06
26.06.2009	106	25	122	115	14	98	32,64
26.06.2009	106	25	122	115	14	98	84,46
26.06.2009	106	25	122	115	14	98	69,84
17.06.2009	106	25	122	115	14	98	941,87
10.06.2009	106	25	122	115	14	98	931,56
10.06.2009	106	25	122	115	14	98	915,11
10.06.2009	106	25	122	115	14	98	916,01
10.06.2009	106	25	122	115	14	98	945,55
10.06.2009	106	25	122	115	14	98	952,51
10.06.2009	106	25	122	115	14	98	919,54
11.06.2009	106	25	122	115	14	98	927,21
11.06.2009	106	25	122	115	14	98	951,59
11.06.2009	106	25	122	115	14	98	944,50
11.06.2009	106	25	122	115	14	98	957,21
11.06.2009	106	25	122	115	14	98	923,71
11.06.2009	106	25	122	115	14	98	941,37
11.06.2009	106	25	122	115	14	98	930,97
11.06.2009	106	25	122	115	14	98	932,33
12.06.2009	106	25	122	115	14	98	898,14
12.06.2009	106	25	122	115	14	98	913,75
12.06.2009	106	25	122	115	14	98	939,48
03.06.2009	106	25	122	115	14	98	859,96
04.06.2009	106	25	122	115	14	98	929,43
04.06.2009	106	25	122	115	14	98	919,39
04.06.2009	106	25	122	115	14	98	904,02
04.06.2009	106	25	122	115	14	98	852,21
04.06.2009	106	25	122	115	14	98	728,14
04.06.2009	106	25	122	115	14	98	895,30

04.06.2009	106	25	122	115	14	98	920,95
04.06.2009	106	25	122	115	14	98	942,50
05.06.2009	106	25	122	115	14	98	935,60
05.06.2009	106	25	122	115	14	98	922,24
05.06.2009	106	25	122	115	14	98	898,20
24.06.2009	112	17	111	115	14	98	0,00
24.06.2009	112	17	111	115	14	98	4,88
24.06.2009	112	17	111	115	14	98	5,13
24.06.2009	112	17	111	115	14	98	5,70
24.06.2009	112	17	111	115	14	98	23,55
24.06.2009	112	17	111	115	14	98	7,29
25.06.2009	112	17	111	115	14	98	2,83
25.06.2009	112	17	111	115	14	98	62,34
25.06.2009	112	17	111	115	14	98	25,93
25.06.2009	112	17	111	115	14	98	1,29
25.06.2009	112	17	111	115	14	98	21,51
25.06.2009	112	17	111	115	14	98	3,96
25.06.2009	112	17	111	115	14	98	18,86
25.06.2009	112	17	111	115	14	98	29,55
26.06.2009	112	17	111	115	14	98	27,15
26.06.2009	112	17	111	115	14	98	24,89
26.06.2009	112	17	111	115	14	98	26,97
17.06.2009	112	17	111	115	14	98	51,68
17.06.2009	112	17	111	115	14	98	30,44
17.06.2009	112	17	111	115	14	98	33,55
17.06.2009	112	17	111	115	14	98	7,21
18.06.2009	112	17	111	115	14	98	58,99
18.06.2009	112	17	111	115	14	98	20,05
18.06.2009	112	17	111	115	14	98	22,43
18.06.2009	112	17	111	115	14	98	8,68
18.06.2009	112	17	111	115	14	98	10,54
18.06.2009	112	17	111	115	14	98	53,32
18.06.2009	112	17	111	115	14	98	53,37

18.06.2009	112	17	111	115	14	98	53,03
19.06.2009	112	17	111	115	14	98	8,32
19.06.2009	112	17	111	115	14	98	34,74
19.06.2009	112	17	111	115	14	98	5,48
10.06.2009	112	17	111	115	14	98	58,09
10.06.2009	112	17	111	115	14	98	36,53
10.06.2009	112	17	111	115	14	98	29,68
10.06.2009	112	17	111	115	14	98	66,91
10.06.2009	112	17	111	115	14	98	1,47
10.06.2009	112	17	111	115	14	98	33,11
11.06.2009	112	17	111	115	14	98	5,01
11.06.2009	112	17	111	115	14	98	72,42
11.06.2009	112	17	111	115	14	98	39,77
11.06.2009	112	17	111	115	14	98	60,96
11.06.2009	112	17	111	115	14	98	18,91
11.06.2009	112	17	111	115	14	98	51,76
11.06.2009	112	17	111	115	14	98	0,49
12.06.2009	112	17	111	115	14	98	25,63
12.06.2009	112	17	111	115	14	98	30,25
12.06.2009	112	17	111	115	14	98	79,17
03.06.2009	112	17	111	115	14	98	47,65
03.06.2009	112	17	111	115	14	98	46,32
03.06.2009	112	17	111	115	14	98	62,34
03.06.2009	112	17	111	115	14	98	65,15
03.06.2009	112	17	111	115	14	98	56,14
04.06.2009	112	17	111	115	14	98	49,59
04.06.2009	112	17	111	115	14	98	43,77
04.06.2009	112	17	111	115	14	98	47,12
04.06.2009	112	17	111	115	14	98	67,04
04.06.2009	112	17	111	115	14	98	22,61
04.06.2009	112	17	111	115	14	98	18,49
04.06.2009	112	17	111	115	14	98	127,92
04.06.2009	112	17	111	115	14	98	97,56

05.06.2009	112	17	111	115	14	98	63,72
05.06.2009	112	17	111	115	14	98	95,59
05.06.2009	112	17	111	115	14	98	33,63
24.06.2009	113	19	106	115	14	98	0,00
24.06.2009	113	19	106	115	14	98	77,18
24.06.2009	113	19	106	115	14	98	79,37
24.06.2009	113	19	106	115	14	98	76,86
24.06.2009	113	19	106	115	14	98	71,09
24.06.2009	113	19	106	115	14	98	93,53
25.06.2009	113	19	106	115	14	98	80,87
25.06.2009	113	19	106	115	14	98	63,28
25.06.2009	113	19	106	115	14	98	76,54
25.06.2009	113	19	106	115	14	98	86,78
25.06.2009	113	19	106	115	14	98	72,10
25.06.2009	113	19	106	115	14	98	82,50
25.06.2009	113	19	106	115	14	98	68,54
25.06.2009	113	19	106	115	14	98	75,84
26.06.2009	113	19	106	115	14	98	75,14
26.06.2009	113	19	106	115	14	98	71,87
26.06.2009	113	19	106	115	14	98	82,95
17.06.2009	113	19	106	115	14	98	85,44
17.06.2009	113	19	106	115	14	98	75,73
17.06.2009	113	19	106	115	14	98	86,33
17.06.2009	113	19	106	115	14	98	30,36
18.06.2009	113	19	106	115	14	98	18,56
18.06.2009	113	19	106	115	14	98	94,03
18.06.2009	113	19	106	115	14	98	31,51
18.06.2009	113	19	106	115	14	98	76,28
18.06.2009	113	19	106	115	14	98	77,80
18.06.2009	113	19	106	115	14	98	29,20
18.06.2009	113	19	106	115	14	98	88,06
18.06.2009	113	19	106	115	14	98	17,72
19.06.2009	113	19	106	115	14	98	75,71

19.06.2009	113	19	106	115	14	98	46,89
19.06.2009	113	19	106	115	14	98	24,23
10.06.2009	113	19	106	115	14	98	54,06
10.06.2009	113	19	106	115	14	98	19,02
10.06.2009	113	19	106	115	14	98	30,01
10.06.2009	113	19	106	115	14	98	66,86
10.06.2009	113	19	106	115	14	98	71,77
10.06.2009	113	19	106	115	14	98	102,39
11.06.2009	113	19	106	115	14	98	45,20
11.06.2009	113	19	106	115	14	98	15,71
11.06.2009	113	19	106	115	14	98	68,39
11.06.2009	113	19	106	115	14	98	4,86
11.06.2009	113	19	106	115	14	98	104,26
11.06.2009	113	19	106	115	14	98	71,98
11.06.2009	113	19	106	115	14	98	64,34
11.06.2009	113	19	106	115	14	98	86,63
12.06.2009	113	19	106	115	14	98	97,98
12.06.2009	113	19	106	115	14	98	51,33
12.06.2009	113	19	106	115	14	98	72,10
03.06.2009	113	19	106	115	14	98	83,91
03.06.2009	113	19	106	115	14	98	32,39
03.06.2009	113	19	106	115	14	98	42,95
03.06.2009	113	19	106	115	14	98	81,05
03.06.2009	113	19	106	115	14	98	71,12
04.06.2009	113	19	106	115	14	98	87,31
04.06.2009	113	19	106	115	14	98	92,84
04.06.2009	113	19	106	115	14	98	82,82
04.06.2009	113	19	106	115	14	98	130,18
04.06.2009	113	19	106	115	14	98	131,44
04.06.2009	113	19	106	115	14	98	73,91
04.06.2009	113	19	106	115	14	98	96,65
04.06.2009	113	19	106	115	14	98	91,27
05.06.2009	113	19	106	115	14	98	71,39

05.06.2009	113	19	106	115	14	98	41,17
05.06.2009	113	19	106	115	14	98	105,93
17.06.2009	114	17	108	115	14	98	328,47
17.06.2009	114	17	108	115	14	98	75,87
17.06.2009	114	17	108	115	14	98	138,21
17.06.2009	114	17	108	115	14	98	117,57
18.06.2009	114	17	108	115	14	98	125,65
18.06.2009	114	17	108	115	14	98	133,50
18.06.2009	114	17	108	115	14	98	60,37
18.06.2009	114	17	108	115	14	98	74,21
18.06.2009	114	17	108	115	14	98	82,55
18.06.2009	114	17	108	115	14	98	91,03
18.06.2009	114	17	108	115	14	98	163,28
18.06.2009	114	17	108	115	14	98	134,98
19.06.2009	114	17	108	115	14	98	82,61
19.06.2009	114	17	108	115	14	98	123,56
19.06.2009	114	17	108	115	14	98	61,77
10.06.2009	114	17	108	115	14	98	343,50
03.06.2009	114	17	108	115	14	98	10,88
03.06.2009	114	17	108	115	14	98	0,13
03.06.2009	114	17	108	115	14	98	5,57
03.06.2009	114	17	108	115	14	98	7,36
03.06.2009	114	17	108	115	14	98	134,57
04.06.2009	114	17	108	115	14	98	126,37
04.06.2009	114	17	108	115	14	98	133,07
04.06.2009	114	17	108	115	14	98	137,17
04.06.2009	114	17	108	115	14	98	29,31
04.06.2009	114	17	108	115	14	98	27,62
04.06.2009	114	17	108	115	14	98	284,13
04.06.2009	114	17	108	115	14	98	307,04
04.06.2009	114	17	108	115	14	98	281,86
05.06.2009	114	17	108	115	14	98	330,20
05.06.2009	114	17	108	115	14	98	75,79

05.06.2009	114	17	108	115	14	98	299,80
17.06.2009	106	25	122	116	60	172	943,77
10.06.2009	106	25	122	116	60	172	917,46
10.06.2009	106	25	122	116	60	172	921,75
10.06.2009	106	25	122	116	60	172	931,62
10.06.2009	106	25	122	116	60	172	941,95
10.06.2009	106	25	122	116	60	172	918,34
10.06.2009	106	25	122	116	60	172	936,68
11.06.2009	106	25	122	116	60	172	928,18
11.06.2009	106	25	122	116	60	172	951,35
11.06.2009	106	25	122	116	60	172	945,46
11.06.2009	106	25	122	116	60	172	944,45
11.06.2009	106	25	122	116	60	172	937,66
11.06.2009	106	25	122	116	60	172	946,25
11.06.2009	106	25	122	116	60	172	950,29
11.06.2009	106	25	122	116	60	172	950,72
12.06.2009	106	25	122	116	60	172	916,15
12.06.2009	106	25	122	116	60	172	949,62
12.06.2009	106	25	122	116	60	172	934,09
03.06.2009	106	25	122	116	60	172	862,13
03.06.2009	106	25	122	116	60	172	935,27
04.06.2009	106	25	122	116	60	172	1020,48
04.06.2009	106	25	122	116	60	172	999,38
04.06.2009	106	25	122	116	60	172	988,21
04.06.2009	106	25	122	116	60	172	890,73
04.06.2009	106	25	122	116	60	172	764,04
04.06.2009	106	25	122	116	60	172	933,65
04.06.2009	106	25	122	116	60	172	933,50
04.06.2009	106	25	122	116	60	172	953,22
05.06.2009	106	25	122	116	60	172	922,27
05.06.2009	106	25	122	116	60	172	929,20
05.06.2009	106	25	122	116	60	172	921,53
17.06.2009	112	17	111	116	60	172	54,19

17.06.2009	112	17	111	116	60	172	424,01
17.06.2009	112	17	111	116	60	172	425,37
10.06.2009	112	17	111	116	60	172	52,36
10.06.2009	112	17	111	116	60	172	45,66
10.06.2009	112	17	111	116	60	172	51,64
10.06.2009	112	17	111	116	60	172	65,68
10.06.2009	112	17	111	116	60	172	36,25
10.06.2009	112	17	111	116	60	172	3,66
11.06.2009	112	17	111	116	60	172	6,23
11.06.2009	112	17	111	116	60	172	71,55
11.06.2009	112	17	111	116	60	172	40,22
11.06.2009	112	17	111	116	60	172	60,49
11.06.2009	112	17	111	116	60	172	22,49
11.06.2009	112	17	111	116	60	172	56,96
11.06.2009	112	17	111	116	60	172	24,06
12.06.2009	112	17	111	116	60	172	7,02
12.06.2009	112	17	111	116	60	172	79,22
12.06.2009	112	17	111	116	60	172	75,70
03.06.2009	112	17	111	116	60	172	54,14
03.06.2009	112	17	111	116	60	172	47,40
03.06.2009	112	17	111	116	60	172	42,49
03.06.2009	112	17	111	116	60	172	64,27
03.06.2009	112	17	111	116	60	172	57,70
03.06.2009	112	17	111	116	60	172	67,44
04.06.2009	112	17	111	116	60	172	127,47
04.06.2009	112	17	111	116	60	172	109,56
04.06.2009	112	17	111	116	60	172	124,65
04.06.2009	112	17	111	116	60	172	41,11
04.06.2009	112	17	111	116	60	172	74,53
04.06.2009	112	17	111	116	60	172	75,72
04.06.2009	112	17	111	116	60	172	142,03
04.06.2009	112	17	111	116	60	172	109,72
05.06.2009	112	17	111	116	60	172	61,47

05.06.2009	112	17	111	116	60	172	103,28
05.06.2009	112	17	111	116	60	172	63,50
17.06.2009	113	19	106	116	60	172	83,08
17.06.2009	113	19	106	116	60	172	378,43
17.06.2009	113	19	106	116	60	172	338,27
10.06.2009	113	19	106	116	60	172	71,04
10.06.2009	113	19	106	116	60	172	15,23
10.06.2009	113	19	106	116	60	172	20,30
10.06.2009	113	19	106	116	60	172	70,38
10.06.2009	113	19	106	116	60	172	106,93
10.06.2009	113	19	106	116	60	172	82,07
11.06.2009	113	19	106	116	60	172	45,40
11.06.2009	113	19	106	116	60	172	14,99
11.06.2009	113	19	106	116	60	172	67,51
11.06.2009	113	19	106	116	60	172	17,47
11.06.2009	113	19	106	116	60	172	87,59
11.06.2009	113	19	106	116	60	172	69,42
11.06.2009	113	19	106	116	60	172	37,83
11.06.2009	113	19	106	116	60	172	71,51
12.06.2009	113	19	106	116	60	172	79,43
12.06.2009	113	19	106	116	60	172	17,44
12.06.2009	113	19	106	116	60	172	77,03
03.06.2009	113	19	106	116	60	172	71,48
03.06.2009	113	19	106	116	60	172	34,15
03.06.2009	113	19	106	116	60	172	43,73
03.06.2009	113	19	106	116	60	172	79,74
03.06.2009	113	19	106	116	60	172	69,26
03.06.2009	113	19	106	116	60	172	74,46
04.06.2009	113	19	106	116	60	172	10,13
04.06.2009	113	19	106	116	60	172	42,09
04.06.2009	113	19	106	116	60	172	6,06
04.06.2009	113	19	106	116	60	172	90,82
04.06.2009	113	19	106	116	60	172	98,29

04.06.2009	113	19	106	116	60	172	53,00
04.06.2009	113	19	106	116	60	172	86,38
04.06.2009	113	19	106	116	60	172	82,51
05.06.2009	113	19	106	116	60	172	83,27
05.06.2009	113	19	106	116	60	172	37,91
05.06.2009	113	19	106	116	60	172	85,82
17.06.2009	114	17	108	116	60	172	330,47
17.06.2009	114	17	108	116	60	172	285,49
17.06.2009	114	17	108	116	60	172	342,22
10.06.2009	114	17	108	116	60	172	330,55
03.06.2009	114	17	108	116	60	172	5,46
03.06.2009	114	17	108	116	60	172	1,97
03.06.2009	114	17	108	116	60	172	22,75
03.06.2009	114	17	108	116	60	172	5,97
03.06.2009	114	17	108	116	60	172	136,40
03.06.2009	114	17	108	116	60	172	121,13
04.06.2009	114	17	108	116	60	172	211,12
04.06.2009	114	17	108	116	60	172	204,56
04.06.2009	114	17	108	116	60	172	220,49
04.06.2009	114	17	108	116	60	172	100,10
04.06.2009	114	17	108	116	60	172	79,54
04.06.2009	114	17	108	116	60	172	326,54
04.06.2009	114	17	108	116	60	172	320,24
04.06.2009	114	17	108	116	60	172	293,50
05.06.2009	114	17	108	116	60	172	319,10
05.06.2009	114	17	108	116	60	172	83,22
05.06.2009	114	17	108	116	60	172	325,57
17.06.2009	115	14	98	116	60	172	2,64
17.06.2009	115	14	98	116	60	172	406,30
10.06.2009	115	14	98	116	60	172	16,97
10.06.2009	115	14	98	116	60	172	9,37
10.06.2009	115	14	98	116	60	172	22,06
10.06.2009	115	14	98	116	60	172	4,29

10.06.2009	115	14	98	116	60	172	35,24
10.06.2009	115	14	98	116	60	172	34,79
11.06.2009	115	14	98	116	60	172	1,44
11.06.2009	115	14	98	116	60	172	1,10
11.06.2009	115	14	98	116	60	172	1,77
11.06.2009	115	14	98	116	60	172	22,33
11.06.2009	115	14	98	116	60	172	26,73
11.06.2009	115	14	98	116	60	172	8,64
11.06.2009	115	14	98	116	60	172	26,93
11.06.2009	115	14	98	116	60	172	24,50
12.06.2009	115	14	98	116	60	172	23,65
12.06.2009	115	14	98	116	60	172	49,48
12.06.2009	115	14	98	116	60	172	5,38
03.06.2009	115	14	98	116	60	172	15,73
03.06.2009	115	14	98	116	60	172	2,07
03.06.2009	115	14	98	116	60	172	19,89
03.06.2009	115	14	98	116	60	172	1,53
03.06.2009	115	14	98	116	60	172	2,20
04.06.2009	115	14	98	116	60	172	91,15
04.06.2009	115	14	98	116	60	172	88,49
04.06.2009	115	14	98	116	60	172	85,10
04.06.2009	115	14	98	116	60	172	70,89
04.06.2009	115	14	98	116	60	172	51,94
04.06.2009	115	14	98	116	60	172	59,18
04.06.2009	115	14	98	116	60	172	14,21
04.06.2009	115	14	98	116	60	172	12,26
05.06.2009	115	14	98	116	60	172	17,58
05.06.2009	115	14	98	116	60	172	7,89
05.06.2009	115	14	98	116	60	172	34,27
25.06.2009	106	25	122	117	29	130	121,55
25.06.2009	106	25	122	117	29	130	150,86
25.06.2009	106	25	122	117	29	130	72,11
25.06.2009	106	25	122	117	29	130	33,00

25.06.2009	106	25	122	117	29	130	39,84
26.06.2009	106	25	122	117	29	130	42,67
26.06.2009	106	25	122	117	29	130	14,21
26.06.2009	106	25	122	117	29	130	147,70
17.06.2009	106	25	122	117	29	130	961,41
10.06.2009	106	25	122	117	29	130	926,05
10.06.2009	106	25	122	117	29	130	931,66
10.06.2009	106	25	122	117	29	130	952,18
10.06.2009	106	25	122	117	29	130	930,46
10.06.2009	106	25	122	117	29	130	955,60
10.06.2009	106	25	122	117	29	130	948,94
11.06.2009	106	25	122	117	29	130	942,41
11.06.2009	106	25	122	117	29	130	948,46
11.06.2009	106	25	122	117	29	130	936,87
11.06.2009	106	25	122	117	29	130	950,96
11.06.2009	106	25	122	117	29	130	960,44
11.06.2009	106	25	122	117	29	130	934,46
11.06.2009	106	25	122	117	29	130	932,84
11.06.2009	106	25	122	117	29	130	936,88
12.06.2009	106	25	122	117	29	130	923,63
12.06.2009	106	25	122	117	29	130	948,21
12.06.2009	106	25	122	117	29	130	943,18
03.06.2009	106	25	122	117	29	130	860,57
03.06.2009	106	25	122	117	29	130	933,78
04.06.2009	106	25	122	117	29	130	929,28
04.06.2009	106	25	122	117	29	130	924,90
04.06.2009	106	25	122	117	29	130	909,90
04.06.2009	106	25	122	117	29	130	891,30
04.06.2009	106	25	122	117	29	130	765,78
04.06.2009	106	25	122	117	29	130	936,21
04.06.2009	106	25	122	117	29	130	934,15
04.06.2009	106	25	122	117	29	130	955,80
05.06.2009	106	25	122	117	29	130	923,97

05.06.2009	106	25	122	117	29	130	932,87
05.06.2009	106	25	122	117	29	130	921,52
24.06.2009	112	17	111	117	29	130	0,00
24.06.2009	112	17	111	117	29	130	71,59
24.06.2009	112	17	111	117	29	130	60,58
24.06.2009	112	17	111	117	29	130	68,98
24.06.2009	112	17	111	117	29	130	77,62
24.06.2009	112	17	111	117	29	130	56,94
25.06.2009	112	17	111	117	29	130	72,57
25.06.2009	112	17	111	117	29	130	63,23
25.06.2009	112	17	111	117	29	130	58,50
25.06.2009	112	17	111	117	29	130	51,64
25.06.2009	112	17	111	117	29	130	72,72
25.06.2009	112	17	111	117	29	130	57,16
25.06.2009	112	17	111	117	29	130	54,00
26.06.2009	112	17	111	117	29	130	48,67
26.06.2009	112	17	111	117	29	130	69,27
26.06.2009	112	17	111	117	29	130	72,71
17.06.2009	112	17	111	117	29	130	65,89
17.06.2009	112	17	111	117	29	130	54,77
17.06.2009	112	17	111	117	29	130	52,14
17.06.2009	112	17	111	117	29	130	37,42
17.06.2009	112	17	111	117	29	130	38,04
17.06.2009	112	17	111	117	29	130	20,11
18.06.2009	112	17	111	117	29	130	63,70
18.06.2009	112	17	111	117	29	130	35,11
18.06.2009	112	17	111	117	29	130	34,66
18.06.2009	112	17	111	117	29	130	12,22
18.06.2009	112	17	111	117	29	130	20,00
18.06.2009	112	17	111	117	29	130	55,67
18.06.2009	112	17	111	117	29	130	60,22
18.06.2009	112	17	111	117	29	130	53,78
19.06.2009	112	17	111	117	29	130	19,09

19.06.2009	112	17	111	117	29	130	39,68
19.06.2009	112	17	111	117	29	130	32,61
10.06.2009	112	17	111	117	29	130	57,12
10.06.2009	112	17	111	117	29	130	48,13
10.06.2009	112	17	111	117	29	130	63,84
10.06.2009	112	17	111	117	29	130	60,69
10.06.2009	112	17	111	117	29	130	1,88
10.06.2009	112	17	111	117	29	130	18,82
11.06.2009	112	17	111	117	29	130	21,71
11.06.2009	112	17	111	117	29	130	70,94
11.06.2009	112	17	111	117	29	130	37,05
11.06.2009	112	17	111	117	29	130	56,44
11.06.2009	112	17	111	117	29	130	14,01
11.06.2009	112	17	111	117	29	130	53,22
11.06.2009	112	17	111	117	29	130	4,42
12.06.2009	112	17	111	117	29	130	6,20
12.06.2009	112	17	111	117	29	130	78,95
12.06.2009	112	17	111	117	29	130	81,06
03.06.2009	112	17	111	117	29	130	50,08
03.06.2009	112	17	111	117	29	130	53,13
03.06.2009	112	17	111	117	29	130	63,34
03.06.2009	112	17	111	117	29	130	63,20
03.06.2009	112	17	111	117	29	130	56,57
03.06.2009	112	17	111	117	29	130	65,09
04.06.2009	112	17	111	117	29	130	54,09
04.06.2009	112	17	111	117	29	130	48,46
04.06.2009	112	17	111	117	29	130	51,78
04.06.2009	112	17	111	117	29	130	38,23
04.06.2009	112	17	111	117	29	130	76,33
04.06.2009	112	17	111	117	29	130	77,99
04.06.2009	112	17	111	117	29	130	142,55
04.06.2009	112	17	111	117	29	130	111,26
05.06.2009	112	17	111	117	29	130	61,70

05.06.2009	112	17	111	117	29	130	107,22
05.06.2009	112	17	111	117	29	130	63,69
24.06.2009	113	19	106	117	29	130	0,00
24.06.2009	113	19	106	117	29	130	139,73
24.06.2009	113	19	106	117	29	130	135,08
24.06.2009	113	19	106	117	29	130	136,97
24.06.2009	113	19	106	117	29	130	141,10
24.06.2009	113	19	106	117	29	130	146,12
25.06.2009	113	19	106	117	29	130	139,71
25.06.2009	113	19	106	117	29	130	69,01
25.06.2009	113	19	106	117	29	130	135,48
25.06.2009	113	19	106	117	29	130	133,53
25.06.2009	113	19	106	117	29	130	149,09
25.06.2009	113	19	106	117	29	130	128,96
25.06.2009	113	19	106	117	29	130	144,44
26.06.2009	113	19	106	117	29	130	134,66
26.06.2009	113	19	106	117	29	130	141,36
26.06.2009	113	19	106	117	29	130	164,05
17.06.2009	113	19	106	117	29	130	78,26
17.06.2009	113	19	106	117	29	130	69,85
17.06.2009	113	19	106	117	29	130	17,43
17.06.2009	113	19	106	117	29	130	66,85
17.06.2009	113	19	106	117	29	130	74,67
17.06.2009	113	19	106	117	29	130	21,20
18.06.2009	113	19	106	117	29	130	12,39
18.06.2009	113	19	106	117	29	130	73,46
18.06.2009	113	19	106	117	29	130	14,31
18.06.2009	113	19	106	117	29	130	74,66
18.06.2009	113	19	106	117	29	130	74,53
18.06.2009	113	19	106	117	29	130	11,01
18.06.2009	113	19	106	117	29	130	78,54
18.06.2009	113	19	106	117	29	130	1,04
19.06.2009	113	19	106	117	29	130	66,09

19.06.2009	113	19	106	117	29	130	39,50
19.06.2009	113	19	106	117	29	130	7,12
10.06.2009	113	19	106	117	29	130	62,47
10.06.2009	113	19	106	117	29	130	4,55
10.06.2009	113	19	106	117	29	130	10,69
10.06.2009	113	19	106	117	29	130	81,93
10.06.2009	113	19	106	117	29	130	68,91
10.06.2009	113	19	106	117	29	130	69,79
11.06.2009	113	19	106	117	29	130	28,84
11.06.2009	113	19	106	117	29	130	18,71
11.06.2009	113	19	106	117	29	130	76,26
11.06.2009	113	19	106	117	29	130	3,75
11.06.2009	113	19	106	117	29	130	67,65
11.06.2009	113	19	106	117	29	130	78,27
11.06.2009	113	19	106	117	29	130	63,03
11.06.2009	113	19	106	117	29	130	82,00
12.06.2009	113	19	106	117	29	130	72,09
12.06.2009	113	19	106	117	29	130	19,03
12.06.2009	113	19	106	117	29	130	68,39
03.06.2009	113	19	106	117	29	130	82,14
03.06.2009	113	19	106	117	29	130	20,11
03.06.2009	113	19	106	117	29	130	20,51
03.06.2009	113	19	106	117	29	130	78,51
03.06.2009	113	19	106	117	29	130	70,59
03.06.2009	113	19	106	117	29	130	74,72
04.06.2009	113	19	106	117	29	130	87,36
04.06.2009	113	19	106	117	29	130	87,33
04.06.2009	113	19	106	117	29	130	76,88
04.06.2009	113	19	106	117	29	130	89,36
04.06.2009	113	19	106	117	29	130	96,86
04.06.2009	113	19	106	117	29	130	52,14
04.06.2009	113	19	106	117	29	130	85,68
04.06.2009	113	19	106	117	29	130	79,76

05.06.2009	113	19	106	117	29	130	81,61
05.06.2009	113	19	106	117	29	130	36,63
05.06.2009	113	19	106	117	29	130	85,90
17.06.2009	114	17	108	117	29	130	347,37
17.06.2009	114	17	108	117	29	130	74,43
17.06.2009	114	17	108	117	29	130	160,33
17.06.2009	114	17	108	117	29	130	67,39
17.06.2009	114	17	108	117	29	130	129,28
17.06.2009	114	17	108	117	29	130	100,61
18.06.2009	114	17	108	117	29	130	118,81
18.06.2009	114	17	108	117	29	130	114,55
18.06.2009	114	17	108	117	29	130	50,21
18.06.2009	114	17	108	117	29	130	69,26
18.06.2009	114	17	108	117	29	130	74,00
18.06.2009	114	17	108	117	29	130	80,11
18.06.2009	114	17	108	117	29	130	153,87
18.06.2009	114	17	108	117	29	130	125,85
19.06.2009	114	17	108	117	29	130	78,36
19.06.2009	114	17	108	117	29	130	115,62
19.06.2009	114	17	108	117	29	130	38,44
10.06.2009	114	17	108	117	29	130	338,76
03.06.2009	114	17	108	117	29	130	10,14
03.06.2009	114	17	108	117	29	130	13,84
03.06.2009	114	17	108	117	29	130	18,05
03.06.2009	114	17	108	117	29	130	4,50
03.06.2009	114	17	108	117	29	130	135,08
03.06.2009	114	17	108	117	29	130	118,88
04.06.2009	114	17	108	117	29	130	128,83
04.06.2009	114	17	108	117	29	130	138,63
04.06.2009	114	17	108	117	29	130	142,93
04.06.2009	114	17	108	117	29	130	97,99
04.06.2009	114	17	108	117	29	130	81,31
04.06.2009	114	17	108	117	29	130	329,21

04.06.2009	114	17	108	117	29	130	320,86
04.06.2009	114	17	108	117	29	130	295,85
05.06.2009	114	17	108	117	29	130	320,55
05.06.2009	114	17	108	117	29	130	87,03
05.06.2009	114	17	108	117	29	130	325,58
24.06.2009	115	14	98	117	29	130	0,00
24.06.2009	115	14	98	117	29	130	73,57
24.06.2009	115	14	98	117	29	130	63,67
24.06.2009	115	14	98	117	29	130	72,78
24.06.2009	115	14	98	117	29	130	79,17
24.06.2009	115	14	98	117	29	130	64,09
25.06.2009	115	14	98	117	29	130	74,00
25.06.2009	115	14	98	117	29	130	22,62
25.06.2009	115	14	98	117	29	130	59,10
25.06.2009	115	14	98	117	29	130	64,25
25.06.2009	115	14	98	117	29	130	72,85
25.06.2009	115	14	98	117	29	130	69,69
25.06.2009	115	14	98	117	29	130	75,43
26.06.2009	115	14	98	117	29	130	72,32
26.06.2009	115	14	98	117	29	130	70,38
26.06.2009	115	14	98	117	29	130	81,82
17.06.2009	115	14	98	117	29	130	29,08
17.06.2009	115	14	98	117	29	130	9,47
17.06.2009	115	14	98	117	29	130	18,00
17.06.2009	115	14	98	117	29	130	16,96
18.06.2009	115	14	98	117	29	130	7,09
18.06.2009	115	14	98	117	29	130	21,48
18.06.2009	115	14	98	117	29	130	17,43
18.06.2009	115	14	98	117	29	130	5,08
18.06.2009	115	14	98	117	29	130	11,35
18.06.2009	115	14	98	117	29	130	18,43
18.06.2009	115	14	98	117	29	130	9,52
18.06.2009	115	14	98	117	29	130	18,51

19.06.2009	115	14	98	117	29	130	11,17
19.06.2009	115	14	98	117	29	130	8,98
19.06.2009	115	14	98	117	29	130	29,18
10.06.2009	115	14	98	117	29	130	8,73
10.06.2009	115	14	98	117	29	130	16,62
10.06.2009	115	14	98	117	29	130	37,54
10.06.2009	115	14	98	117	29	130	16,35
10.06.2009	115	14	98	117	29	130	3,27
10.06.2009	115	14	98	117	29	130	35,86
11.06.2009	115	14	98	117	29	130	17,76
11.06.2009	115	14	98	117	29	130	3,42
11.06.2009	115	14	98	117	29	130	11,61
11.06.2009	115	14	98	117	29	130	6,75
11.06.2009	115	14	98	117	29	130	37,00
11.06.2009	115	14	98	117	29	130	7,26
11.06.2009	115	14	98	117	29	130	1,89
11.06.2009	115	14	98	117	29	130	4,80
12.06.2009	115	14	98	117	29	130	25,99
12.06.2009	115	14	98	117	29	130	49,07
12.06.2009	115	14	98	117	29	130	3,79
03.06.2009	115	14	98	117	29	130	2,43
03.06.2009	115	14	98	117	29	130	13,69
03.06.2009	115	14	98	117	29	130	22,70
03.06.2009	115	14	98	117	29	130	3,12
03.06.2009	115	14	98	117	29	130	0,62
04.06.2009	115	14	98	117	29	130	5,86
04.06.2009	115	14	98	117	29	130	5,62
04.06.2009	115	14	98	117	29	130	5,95
04.06.2009	115	14	98	117	29	130	68,72
04.06.2009	115	14	98	117	29	130	53,73
04.06.2009	115	14	98	117	29	130	61,63
04.06.2009	115	14	98	117	29	130	14,68
04.06.2009	115	14	98	117	29	130	14,20

05.06.2009	115	14	98	117	29	130	15,51
05.06.2009	115	14	98	117	29	130	11,96
05.06.2009	115	14	98	117	29	130	34,41
17.06.2009	116	60	172	117	29	130	29,31
17.06.2009	116	60	172	117	29	130	393,76
17.06.2009	116	60	172	117	29	130	396,85
10.06.2009	116	60	172	117	29	130	9,01
10.06.2009	116	60	172	117	29	130	11,18
10.06.2009	116	60	172	117	29	130	21,35
10.06.2009	116	60	172	117	29	130	12,15
10.06.2009	116	60	172	117	29	130	38,03
10.06.2009	116	60	172	117	29	130	15,84
11.06.2009	116	60	172	117	29	130	17,54
11.06.2009	116	60	172	117	29	130	3,78
11.06.2009	116	60	172	117	29	130	13,36
11.06.2009	116	60	172	117	29	130	17,04
11.06.2009	116	60	172	117	29	130	29,50
11.06.2009	116	60	172	117	29	130	15,04
11.06.2009	116	60	172	117	29	130	25,85
11.06.2009	116	60	172	117	29	130	22,54
12.06.2009	116	60	172	117	29	130	12,83
12.06.2009	116	60	172	117	29	130	1,67
12.06.2009	116	60	172	117	29	130	9,11
03.06.2009	116	60	172	117	29	130	15,37
03.06.2009	116	60	172	117	29	130	15,02
03.06.2009	116	60	172	117	29	130	28,83
03.06.2009	116	60	172	117	29	130	1,60
03.06.2009	116	60	172	117	29	130	1,58
03.06.2009	116	60	172	117	29	130	2,36
04.06.2009	116	60	172	117	29	130	91,80
04.06.2009	116	60	172	117	29	130	84,11
04.06.2009	116	60	172	117	29	130	79,15
04.06.2009	116	60	172	117	29	130	3,14

04.06.2009	116	60	172	117	29	130	1,91
04.06.2009	116	60	172	117	29	130	2,74
04.06.2009	116	60	172	117	29	130	0,70
04.06.2009	116	60	172	117	29	130	2,76
05.06.2009	116	60	172	117	29	130	2,08
05.06.2009	116	60	172	117	29	130	4,07
05.06.2009	116	60	172	117	29	130	0,21
25.06.2009	106	25	122	118	41	141	991,99
25.06.2009	106	25	122	118	41	141	1036,13
25.06.2009	106	25	122	118	41	141	782,20
25.06.2009	106	25	122	118	41	141	736,80
25.06.2009	106	25	122	118	41	141	830,55
26.06.2009	106	25	122	118	41	141	737,69
26.06.2009	106	25	122	118	41	141	861,72
26.06.2009	106	25	122	118	41	141	1013,50
17.06.2009	106	25	122	118	41	141	6,17
10.06.2009	106	25	122	118	41	141	20,61
10.06.2009	106	25	122	118	41	141	600,97
10.06.2009	106	25	122	118	41	141	71,47
10.06.2009	106	25	122	118	41	141	62,62
10.06.2009	106	25	122	118	41	141	7,00
10.06.2009	106	25	122	118	41	141	18,40
11.06.2009	106	25	122	118	41	141	178,88
11.06.2009	106	25	122	118	41	141	22,63
11.06.2009	106	25	122	118	41	141	168,68
11.06.2009	106	25	122	118	41	141	104,69
11.06.2009	106	25	122	118	41	141	229,96
11.06.2009	106	25	122	118	41	141	80,44
11.06.2009	106	25	122	118	41	141	76,77
11.06.2009	106	25	122	118	41	141	78,22
12.06.2009	106	25	122	118	41	141	41,87
12.06.2009	106	25	122	118	41	141	41,98
12.06.2009	106	25	122	118	41	141	133,24

03.06.2009	106	25	122	118	41	141	39,92
03.06.2009	106	25	122	118	41	141	36,42
04.06.2009	106	25	122	118	41	141	17,63
04.06.2009	106	25	122	118	41	141	2,92
04.06.2009	106	25	122	118	41	141	43,27
04.06.2009	106	25	122	118	41	141	242,93
04.06.2009	106	25	122	118	41	141	41,83
04.06.2009	106	25	122	118	41	141	139,98
04.06.2009	106	25	122	118	41	141	5,14
04.06.2009	106	25	122	118	41	141	223,05
05.06.2009	106	25	122	118	41	141	41,69
05.06.2009	106	25	122	118	41	141	102,95
05.06.2009	106	25	122	118	41	141	216,86
24.06.2009	112	17	111	118	41	141	0,00
24.06.2009	112	17	111	118	41	141	769,62
24.06.2009	112	17	111	118	41	141	760,59
24.06.2009	112	17	111	118	41	141	934,60
24.06.2009	112	17	111	118	41	141	895,56
24.06.2009	112	17	111	118	41	141	909,82
25.06.2009	112	17	111	118	41	141	938,59
25.06.2009	112	17	111	118	41	141	902,98
25.06.2009	112	17	111	118	41	141	930,45
25.06.2009	112	17	111	118	41	141	911,67
25.06.2009	112	17	111	118	41	141	931,21
25.06.2009	112	17	111	118	41	141	784,93
25.06.2009	112	17	111	118	41	141	760,03
25.06.2009	112	17	111	118	41	141	841,02
26.06.2009	112	17	111	118	41	141	742,51
26.06.2009	112	17	111	118	41	141	913,16
26.06.2009	112	17	111	118	41	141	920,27
17.06.2009	112	17	111	118	41	141	896,83
17.06.2009	112	17	111	118	41	141	884,96
17.06.2009	112	17	111	118	41	141	815,86

17.06.2009	112	17	111	118	41	141	842,36
17.06.2009	112	17	111	118	41	141	744,37
17.06.2009	112	17	111	118	41	141	921,03
18.06.2009	112	17	111	118	41	141	720,05
18.06.2009	112	17	111	118	41	141	754,84
18.06.2009	112	17	111	118	41	141	853,30
18.06.2009	112	17	111	118	41	141	750,36
18.06.2009	112	17	111	118	41	141	761,63
18.06.2009	112	17	111	118	41	141	809,55
18.06.2009	112	17	111	118	41	141	900,09
18.06.2009	112	17	111	118	41	141	906,29
19.06.2009	112	17	111	118	41	141	910,90
19.06.2009	112	17	111	118	41	141	847,94
19.06.2009	112	17	111	118	41	141	834,83
10.06.2009	112	17	111	118	41	141	883,29
10.06.2009	112	17	111	118	41	141	297,95
10.06.2009	112	17	111	118	41	141	835,12
10.06.2009	112	17	111	118	41	141	841,78
10.06.2009	112	17	111	118	41	141	947,39
10.06.2009	112	17	111	118	41	141	916,28
11.06.2009	112	17	111	118	41	141	794,03
11.06.2009	112	17	111	118	41	141	879,53
11.06.2009	112	17	111	118	41	141	772,64
11.06.2009	112	17	111	118	41	141	807,03
11.06.2009	112	17	111	118	41	141	856,68
11.06.2009	112	17	111	118	41	141	844,68
11.06.2009	112	17	111	118	41	141	866,36
12.06.2009	112	17	111	118	41	141	951,07
12.06.2009	112	17	111	118	41	141	886,73
12.06.2009	112	17	111	118	41	141	771,47
03.06.2009	112	17	111	118	41	141	830,75
03.06.2009	112	17	111	118	41	141	896,54
04.06.2009	112	17	111	118	41	141	896,42

04.06.2009	112	17	111	118	41	141	891,65
04.06.2009	112	17	111	118	41	141	901,45
04.06.2009	112	17	111	118	41	141	775,78
04.06.2009	112	17	111	118	41	141	731,87
04.06.2009	112	17	111	118	41	141	769,60
04.06.2009	112	17	111	118	41	141	803,22
04.06.2009	112	17	111	118	41	141	691,21
05.06.2009	112	17	111	118	41	141	890,62
05.06.2009	112	17	111	118	41	141	759,56
05.06.2009	112	17	111	118	41	141	739,23
24.06.2009	113	19	106	118	41	141	0,00
24.06.2009	113	19	106	118	41	141	850,28
24.06.2009	113	19	106	118	41	141	844,23
24.06.2009	113	19	106	118	41	141	1017,14
24.06.2009	113	19	106	118	41	141	986,46
24.06.2009	113	19	106	118	41	141	1008,48
25.06.2009	113	19	106	118	41	141	1018,41
25.06.2009	113	19	106	118	41	141	1019,92
25.06.2009	113	19	106	118	41	141	1025,62
25.06.2009	113	19	106	118	41	141	999,45
25.06.2009	113	19	106	118	41	141	1022,10
25.06.2009	113	19	106	118	41	141	865,06
25.06.2009	113	19	106	118	41	141	846,79
25.06.2009	113	19	106	118	41	141	944,18
26.06.2009	113	19	106	118	41	141	841,99
26.06.2009	113	19	106	118	41	141	998,45
26.06.2009	113	19	106	118	41	141	1020,69
17.06.2009	113	19	106	118	41	141	1024,61
17.06.2009	113	19	106	118	41	141	998,29
17.06.2009	113	19	106	118	41	141	870,35
17.06.2009	113	19	106	118	41	141	944,71
17.06.2009	113	19	106	118	41	141	856,84
17.06.2009	113	19	106	118	41	141	949,67

18.06.2009	113	19	106	118	41	141	781,03
18.06.2009	113	19	106	118	41	141	862,70
18.06.2009	113	19	106	118	41	141	898,05
18.06.2009	113	19	106	118	41	141	831,73
18.06.2009	113	19	106	118	41	141	843,10
18.06.2009	113	19	106	118	41	141	865,10
18.06.2009	113	19	106	118	41	141	1021,09
18.06.2009	113	19	106	118	41	141	951,94
19.06.2009	113	19	106	118	41	141	983,30
19.06.2009	113	19	106	118	41	141	902,56
19.06.2009	113	19	106	118	41	141	854,15
10.06.2009	113	19	106	118	41	141	979,18
10.06.2009	113	19	106	118	41	141	337,92
10.06.2009	113	19	106	118	41	141	883,75
10.06.2009	113	19	106	118	41	141	961,69
10.06.2009	113	19	106	118	41	141	1017,08
10.06.2009	113	19	106	118	41	141	1001,78
11.06.2009	113	19	106	118	41	141	824,02
11.06.2009	113	19	106	118	41	141	943,82
11.06.2009	113	19	106	118	41	141	879,58
11.06.2009	113	19	106	118	41	141	861,20
11.06.2009	113	19	106	118	41	141	854,78
11.06.2009	113	19	106	118	41	141	945,41
11.06.2009	113	19	106	118	41	141	930,32
11.06.2009	113	19	106	118	41	141	952,51
12.06.2009	113	19	106	118	41	141	1025,09
12.06.2009	113	19	106	118	41	141	951,33
12.06.2009	113	19	106	118	41	141	889,85
03.06.2009	113	19	106	118	41	141	929,53
03.06.2009	113	19	106	118	41	141	995,84
04.06.2009	113	19	106	118	41	141	1013,97
04.06.2009	113	19	106	118	41	141	1014,08
04.06.2009	113	19	106	118	41	141	1015,32

04.06.2009	113	19	106	118	41	141	839,74
04.06.2009	113	19	106	118	41	141	879,83
04.06.2009	113	19	106	118	41	141	847,58
04.06.2009	113	19	106	118	41	141	1016,16
04.06.2009	113	19	106	118	41	141	861,91
05.06.2009	113	19	106	118	41	141	1014,54
05.06.2009	113	19	106	118	41	141	866,19
05.06.2009	113	19	106	118	41	141	849,93
17.06.2009	114	17	108	118	41	141	615,36
17.06.2009	114	17	108	118	41	141	969,45
17.06.2009	114	17	108	118	41	141	1017,62
17.06.2009	114	17	108	118	41	141	944,59
17.06.2009	114	17	108	118	41	141	910,39
17.06.2009	114	17	108	118	41	141	1040,31
18.06.2009	114	17	108	118	41	141	891,86
18.06.2009	114	17	108	118	41	141	904,15
18.06.2009	114	17	108	118	41	141	886,44
18.06.2009	114	17	108	118	41	141	783,55
18.06.2009	114	17	108	118	41	141	824,37
18.06.2009	114	17	108	118	41	141	939,67
18.06.2009	114	17	108	118	41	141	1093,66
18.06.2009	114	17	108	118	41	141	1072,83
19.06.2009	114	17	108	118	41	141	1000,00
19.06.2009	114	17	108	118	41	141	997,55
19.06.2009	114	17	108	118	41	141	861,02
10.06.2009	114	17	108	118	41	141	592,70
03.06.2009	114	17	108	118	41	141	753,67
03.06.2009	114	17	108	118	41	141	833,16
04.06.2009	114	17	108	118	41	141	815,69
04.06.2009	114	17	108	118	41	141	797,11
04.06.2009	114	17	108	118	41	141	806,75
04.06.2009	114	17	108	118	41	141	687,09
04.06.2009	114	17	108	118	41	141	730,49

04.06.2009	114	17	108	118	41	141	489,82
04.06.2009	114	17	108	118	41	141	616,90
04.06.2009	114	17	108	118	41	141	495,02
05.06.2009	114	17	108	118	41	141	617,48
05.06.2009	114	17	108	118	41	141	781,03
05.06.2009	114	17	108	118	41	141	448,33
24.06.2009	115	14	98	118	41	141	0,00
24.06.2009	115	14	98	118	41	141	773,89
24.06.2009	115	14	98	118	41	141	765,42
24.06.2009	115	14	98	118	41	141	940,29
24.06.2009	115	14	98	118	41	141	916,05
24.06.2009	115	14	98	118	41	141	915,38
25.06.2009	115	14	98	118	41	141	937,57
25.06.2009	115	14	98	118	41	141	961,39
25.06.2009	115	14	98	118	41	141	950,06
25.06.2009	115	14	98	118	41	141	912,92
25.06.2009	115	14	98	118	41	141	952,58
25.06.2009	115	14	98	118	41	141	782,75
25.06.2009	115	14	98	118	41	141	778,73
25.06.2009	115	14	98	118	41	141	869,93
26.06.2009	115	14	98	118	41	141	769,30
26.06.2009	115	14	98	118	41	141	931,28
26.06.2009	115	14	98	118	41	141	944,35
17.06.2009	115	14	98	118	41	141	943,00
17.06.2009	115	14	98	118	41	141	868,99
17.06.2009	115	14	98	118	41	141	772,18
17.06.2009	115	14	98	118	41	141	924,04
18.06.2009	115	14	98	118	41	141	766,24
18.06.2009	115	14	98	118	41	141	770,69
18.06.2009	115	14	98	118	41	141	873,07
18.06.2009	115	14	98	118	41	141	757,99
18.06.2009	115	14	98	118	41	141	769,54
18.06.2009	115	14	98	118	41	141	848,69

18.06.2009	115	14	98	118	41	141	933,10
18.06.2009	115	14	98	118	41	141	939,42
19.06.2009	115	14	98	118	41	141	917,63
19.06.2009	115	14	98	118	41	141	874,28
19.06.2009	115	14	98	118	41	141	840,21
10.06.2009	115	14	98	118	41	141	934,49
10.06.2009	115	14	98	118	41	141	319,59
10.06.2009	115	14	98	118	41	141	853,74
10.06.2009	115	14	98	118	41	141	894,83
10.06.2009	115	14	98	118	41	141	946,17
10.06.2009	115	14	98	118	41	141	902,55
11.06.2009	115	14	98	118	41	141	799,03
11.06.2009	115	14	98	118	41	141	934,92
11.06.2009	115	14	98	118	41	141	811,39
11.06.2009	115	14	98	118	41	141	863,78
11.06.2009	115	14	98	118	41	141	750,68
11.06.2009	115	14	98	118	41	141	874,61
11.06.2009	115	14	98	118	41	141	880,56
11.06.2009	115	14	98	118	41	141	865,89
12.06.2009	115	14	98	118	41	141	927,48
12.06.2009	115	14	98	118	41	141	903,61
12.06.2009	115	14	98	118	41	141	825,53
03.06.2009	115	14	98	118	41	141	876,63
04.06.2009	115	14	98	118	41	141	926,87
04.06.2009	115	14	98	118	41	141	921,24
04.06.2009	115	14	98	118	41	141	932,61
04.06.2009	115	14	98	118	41	141	709,67
04.06.2009	115	14	98	118	41	141	748,65
04.06.2009	115	14	98	118	41	141	773,68
04.06.2009	115	14	98	118	41	141	921,69
04.06.2009	115	14	98	118	41	141	776,87
05.06.2009	115	14	98	118	41	141	944,09
05.06.2009	115	14	98	118	41	141	832,40

05.06.2009	115	14	98	118	41	141	745,16
17.06.2009	116	60	172	118	41	141	944,89
17.06.2009	116	60	172	118	41	141	1225,09
17.06.2009	116	60	172	118	41	141	1250,38
10.06.2009	116	60	172	118	41	141	920,61
10.06.2009	116	60	172	118	41	141	327,17
10.06.2009	116	60	172	118	41	141	869,96
10.06.2009	116	60	172	118	41	141	891,33
10.06.2009	116	60	172	118	41	141	911,97
10.06.2009	116	60	172	118	41	141	919,94
11.06.2009	116	60	172	118	41	141	800,15
11.06.2009	116	60	172	118	41	141	934,66
11.06.2009	116	60	172	118	41	141	812,17
11.06.2009	116	60	172	118	41	141	852,01
11.06.2009	116	60	172	118	41	141	768,55
11.06.2009	116	60	172	118	41	141	879,14
11.06.2009	116	60	172	118	41	141	898,66
11.06.2009	116	60	172	118	41	141	883,54
12.06.2009	116	60	172	118	41	141	945,97
12.06.2009	116	60	172	118	41	141	940,95
12.06.2009	116	60	172	118	41	141	820,15
03.06.2009	116	60	172	118	41	141	878,78
03.06.2009	116	60	172	118	41	141	934,68
04.06.2009	116	60	172	118	41	141	1017,98
04.06.2009	116	60	172	118	41	141	1001,14
04.06.2009	116	60	172	118	41	141	1017,17
04.06.2009	116	60	172	118	41	141	761,83
04.06.2009	116	60	172	118	41	141	786,30
04.06.2009	116	60	172	118	41	141	815,72
04.06.2009	116	60	172	118	41	141	934,28
04.06.2009	116	60	172	118	41	141	788,46
05.06.2009	116	60	172	118	41	141	931,27
05.06.2009	116	60	172	118	41	141	839,56

05.06.2009	116	60	172	118	41	141	772,77
24.06.2009	117	29	130	118	41	141	0,00
24.06.2009	117	29	130	118	41	141	715,25
24.06.2009	117	29	130	118	41	141	711,92
24.06.2009	117	29	130	118	41	141	894,03
24.06.2009	117	29	130	118	41	141	865,59
24.06.2009	117	29	130	118	41	141	876,17
25.06.2009	117	29	130	118	41	141	894,56
25.06.2009	117	29	130	118	41	141	950,99
25.06.2009	117	29	130	118	41	141	875,25
25.06.2009	117	29	130	118	41	141	903,74
25.06.2009	117	29	130	118	41	141	725,70
25.06.2009	117	29	130	118	41	141	722,84
25.06.2009	117	29	130	118	41	141	820,15
26.06.2009	117	29	130	118	41	141	709,56
26.06.2009	117	29	130	118	41	141	874,17
26.06.2009	117	29	130	118	41	141	878,34
17.06.2009	117	29	130	118	41	141	962,67
17.06.2009	117	29	130	118	41	141	932,74
17.06.2009	117	29	130	118	41	141	860,09
17.06.2009	117	29	130	118	41	141	877,91
17.06.2009	117	29	130	118	41	141	782,18
17.06.2009	117	29	130	118	41	141	940,78
18.06.2009	117	29	130	118	41	141	773,12
18.06.2009	117	29	130	118	41	141	789,84
18.06.2009	117	29	130	118	41	141	887,92
18.06.2009	117	29	130	118	41	141	758,43
18.06.2009	117	29	130	118	41	141	769,79
18.06.2009	117	29	130	118	41	141	860,43
18.06.2009	117	29	130	118	41	141	942,60
18.06.2009	117	29	130	118	41	141	951,99
19.06.2009	117	29	130	118	41	141	923,38
19.06.2009	117	29	130	118	41	141	882,57

19.06.2009	117	29	130	118	41	141	861,27
10.06.2009	117	29	130	118	41	141	929,14
10.06.2009	117	29	130	118	41	141	336,18
10.06.2009	117	29	130	118	41	141	890,28
10.06.2009	117	29	130	118	41	141	880,01
10.06.2009	117	29	130	118	41	141	949,26
10.06.2009	117	29	130	118	41	141	932,11
11.06.2009	117	29	130	118	41	141	812,77
11.06.2009	117	29	130	118	41	141	931,81
11.06.2009	117	29	130	118	41	141	804,85
11.06.2009	117	29	130	118	41	141	857,67
11.06.2009	117	29	130	118	41	141	787,63
11.06.2009	117	29	130	118	41	141	867,82
11.06.2009	117	29	130	118	41	141	882,44
11.06.2009	117	29	130	118	41	141	870,51
12.06.2009	117	29	130	118	41	141	953,11
12.06.2009	117	29	130	118	41	141	939,58
12.06.2009	117	29	130	118	41	141	829,16
03.06.2009	117	29	130	118	41	141	877,24
03.06.2009	117	29	130	118	41	141	933,12
04.06.2009	117	29	130	118	41	141	926,61
04.06.2009	117	29	130	118	41	141	926,76
04.06.2009	117	29	130	118	41	141	938,52
04.06.2009	117	29	130	118	41	141	761,61
04.06.2009	117	29	130	118	41	141	788,07
04.06.2009	117	29	130	118	41	141	818,35
04.06.2009	117	29	130	118	41	141	934,93
04.06.2009	117	29	130	118	41	141	790,83
05.06.2009	117	29	130	118	41	141	932,92
05.06.2009	117	29	130	118	41	141	843,32
05.06.2009	117	29	130	118	41	141	772,79
25.06.2009	106	25	122	119	88	198	29,97
25.06.2009	106	25	122	119	88	198	136,62

25.06.2009	106	25	122	119	88	198	81,86
25.06.2009	106	25	122	119	88	198	114,48
25.06.2009	106	25	122	119	88	198	44,59
26.06.2009	106	25	122	119	88	198	52,08
26.06.2009	106	25	122	119	88	198	82,99
26.06.2009	106	25	122	119	88	198	54,40
17.06.2009	106	25	122	119	88	198	1091,75
10.06.2009	106	25	122	119	88	198	1057,26
10.06.2009	106	25	122	119	88	198	1069,50
10.06.2009	106	25	122	119	88	198	1025,01
10.06.2009	106	25	122	119	88	198	1087,95
10.06.2009	106	25	122	119	88	198	1091,15
10.06.2009	106	25	122	119	88	198	1067,72
11.06.2009	106	25	122	119	88	198	1017,48
11.06.2009	106	25	122	119	88	198	1028,11
11.06.2009	106	25	122	119	88	198	1057,12
11.06.2009	106	25	122	119	88	198	1066,58
11.06.2009	106	25	122	119	88	198	1086,03
11.06.2009	106	25	122	119	88	198	1067,85
11.06.2009	106	25	122	119	88	198	1024,41
11.06.2009	106	25	122	119	88	198	1032,10
12.06.2009	106	25	122	119	88	198	1045,69
12.06.2009	106	25	122	119	88	198	1039,12
12.06.2009	106	25	122	119	88	198	1023,37
03.06.2009	106	25	122	119	88	198	949,78
03.06.2009	106	25	122	119	88	198	1015,60
04.06.2009	106	25	122	119	88	198	1030,19
04.06.2009	106	25	122	119	88	198	1035,00
04.06.2009	106	25	122	119	88	198	1035,22
04.06.2009	106	25	122	119	88	198	927,89
04.06.2009	106	25	122	119	88	198	855,42
04.06.2009	106	25	122	119	88	198	1042,82
04.06.2009	106	25	122	119	88	198	1019,07

04.06.2009	106	25	122	119	88	198	1042,48
05.06.2009	106	25	122	119	88	198	1036,45
05.06.2009	106	25	122	119	88	198	1020,23
05.06.2009	106	25	122	119	88	198	1049,44
24.06.2009	112	17	111	119	88	198	0,00
24.06.2009	112	17	111	119	88	198	123,63
24.06.2009	112	17	111	119	88	198	125,12
24.06.2009	112	17	111	119	88	198	121,33
24.06.2009	112	17	111	119	88	198	121,68
24.06.2009	112	17	111	119	88	198	132,91
25.06.2009	112	17	111	119	88	198	119,96
25.06.2009	112	17	111	119	88	198	150,04
25.06.2009	112	17	111	119	88	198	121,38
25.06.2009	112	17	111	119	88	198	93,93
25.06.2009	112	17	111	119	88	198	238,58
25.06.2009	112	17	111	119	88	198	78,12
25.06.2009	112	17	111	119	88	198	99,69
25.06.2009	112	17	111	119	88	198	33,43
26.06.2009	112	17	111	119	88	198	49,41
26.06.2009	112	17	111	119	88	198	23,60
26.06.2009	112	17	111	119	88	198	67,36
17.06.2009	112	17	111	119	88	198	208,38
17.06.2009	112	17	111	119	88	198	171,74
17.06.2009	112	17	111	119	88	198	179,32
17.06.2009	112	17	111	119	88	198	116,60
17.06.2009	112	17	111	119	88	198	172,29
17.06.2009	112	17	111	119	88	198	144,98
18.06.2009	112	17	111	119	88	198	166,71
18.06.2009	112	17	111	119	88	198	158,44
18.06.2009	112	17	111	119	88	198	123,83
18.06.2009	112	17	111	119	88	198	122,59
18.06.2009	112	17	111	119	88	198	109,67
18.06.2009	112	17	111	119	88	198	183,44

18.06.2009	112	17	111	119	88	198	175,75
18.06.2009	112	17	111	119	88	198	160,44
19.06.2009	112	17	111	119	88	198	100,46
19.06.2009	112	17	111	119	88	198	151,00
19.06.2009	112	17	111	119	88	198	152,20
10.06.2009	112	17	111	119	88	198	187,78
10.06.2009	112	17	111	119	88	198	185,90
10.06.2009	112	17	111	119	88	198	137,83
10.06.2009	112	17	111	119	88	198	209,59
10.06.2009	112	17	111	119	88	198	143,09
10.06.2009	112	17	111	119	88	198	136,27
11.06.2009	112	17	111	119	88	198	96,51
11.06.2009	112	17	111	119	88	198	148,43
11.06.2009	112	17	111	119	88	198	153,54
11.06.2009	112	17	111	119	88	198	176,59
11.06.2009	112	17	111	119	88	198	146,73
11.06.2009	112	17	111	119	88	198	137,99
11.06.2009	112	17	111	119	88	198	100,08
12.06.2009	112	17	111	119	88	198	125,58
12.06.2009	112	17	111	119	88	198	162,95
12.06.2009	112	17	111	119	88	198	149,91
03.06.2009	112	17	111	119	88	198	178,13
03.06.2009	112	17	111	119	88	198	125,32
03.06.2009	112	17	111	119	88	198	153,25
03.06.2009	112	17	111	119	88	198	141,62
03.06.2009	112	17	111	119	88	198	147,06
03.06.2009	112	17	111	119	88	198	134,01
04.06.2009	112	17	111	119	88	198	143,99
04.06.2009	112	17	111	119	88	198	156,00
04.06.2009	112	17	111	119	88	198	174,42
04.06.2009	112	17	111	119	88	198	17,97
04.06.2009	112	17	111	119	88	198	159,96
04.06.2009	112	17	111	119	88	198	172,63

04.06.2009	112	17	111	119	88	198	225,10
04.06.2009	112	17	111	119	88	198	195,23
05.06.2009	112	17	111	119	88	198	168,24
05.06.2009	112	17	111	119	88	198	186,42
05.06.2009	112	17	111	119	88	198	174,12
24.06.2009	113	19	106	119	88	198	0,00
24.06.2009	113	19	106	119	88	198	44,61
24.06.2009	113	19	106	119	88	198	42,56
24.06.2009	113	19	106	119	88	198	40,64
24.06.2009	113	19	106	119	88	198	33,06
24.06.2009	113	19	106	119	88	198	33,47
25.06.2009	113	19	106	119	88	198	40,80
25.06.2009	113	19	106	119	88	198	24,53
25.06.2009	113	19	106	119	88	198	34,70
25.06.2009	113	19	106	119	88	198	7,79
25.06.2009	113	19	106	119	88	198	148,63
25.06.2009	113	19	106	119	88	198	91,37
25.06.2009	113	19	106	119	88	198	44,06
25.06.2009	113	19	106	119	88	198	74,21
26.06.2009	113	19	106	119	88	198	57,24
26.06.2009	113	19	106	119	88	198	73,52
26.06.2009	113	19	106	119	88	198	76,60
17.06.2009	113	19	106	119	88	198	71,59
17.06.2009	113	19	106	119	88	198	79,75
17.06.2009	113	19	106	119	88	198	123,20
17.06.2009	113	19	106	119	88	198	19,17
17.06.2009	113	19	106	119	88	198	61,50
17.06.2009	113	19	106	119	88	198	123,17
18.06.2009	113	19	106	119	88	198	102,27
18.06.2009	113	19	106	119	88	198	51,27
18.06.2009	113	19	106	119	88	198	83,39
18.06.2009	113	19	106	119	88	198	44,92
18.06.2009	113	19	106	119	88	198	26,23

18.06.2009	113	19	106	119	88	198	126,63
18.06.2009	113	19	106	119	88	198	45,93
18.06.2009	113	19	106	119	88	198	108,16
19.06.2009	113	19	106	119	88	198	59,17
19.06.2009	113	19	106	119	88	198	105,44
19.06.2009	113	19	106	119	88	198	138,12
10.06.2009	113	19	106	119	88	198	104,95
10.06.2009	113	19	106	119	88	198	140,92
10.06.2009	113	19	106	119	88	198	83,92
10.06.2009	113	19	106	119	88	198	82,32
10.06.2009	113	19	106	119	88	198	73,47
10.06.2009	113	19	106	119	88	198	56,36
11.06.2009	113	19	106	119	88	198	78,74
11.06.2009	113	19	106	119	88	198	75,55
11.06.2009	113	19	106	119	88	198	47,33
11.06.2009	113	19	106	119	88	198	117,06
11.06.2009	113	19	106	119	88	198	71,60
11.06.2009	113	19	106	119	88	198	56,52
11.06.2009	113	19	106	119	88	198	67,15
11.06.2009	113	19	106	119	88	198	15,39
12.06.2009	113	19	106	119	88	198	53,51
12.06.2009	113	19	106	119	88	198	85,35
12.06.2009	113	19	106	119	88	198	37,39
03.06.2009	113	19	106	119	88	198	53,88
03.06.2009	113	19	106	119	88	198	69,95
03.06.2009	113	19	106	119	88	198	89,90
03.06.2009	113	19	106	119	88	198	66,14
03.06.2009	113	19	106	119	88	198	80,96
03.06.2009	113	19	106	119	88	198	52,48
04.06.2009	113	19	106	119	88	198	19,00
04.06.2009	113	19	106	119	88	198	33,13
04.06.2009	113	19	106	119	88	198	53,59
04.06.2009	113	19	106	119	88	198	49,59

04.06.2009	113	19	106	119	88	198	43,73
04.06.2009	113	19	106	119	88	198	95,59
04.06.2009	113	19	106	119	88	198	33,32
04.06.2009	113	19	106	119	88	198	43,12
05.06.2009	113	19	106	119	88	198	36,03
05.06.2009	113	19	106	119	88	198	72,80
05.06.2009	113	19	106	119	88	198	67,50
17.06.2009	114	17	108	119	88	198	481,46
17.06.2009	114	17	108	119	88	198	131,00
17.06.2009	114	17	108	119	88	198	30,93
17.06.2009	114	17	108	119	88	198	13,48
17.06.2009	114	17	108	119	88	198	5,03
17.06.2009	114	17	108	119	88	198	26,01
18.06.2009	114	17	108	119	88	198	11,52
18.06.2009	114	17	108	119	88	198	13,26
18.06.2009	114	17	108	119	88	198	115,35
18.06.2009	114	17	108	119	88	198	114,75
18.06.2009	114	17	108	119	88	198	63,84
18.06.2009	114	17	108	119	88	198	48,92
18.06.2009	114	17	108	119	88	198	31,69
18.06.2009	114	17	108	119	88	198	19,20
19.06.2009	114	17	108	119	88	198	12,65
19.06.2009	114	17	108	119	88	198	3,81
19.06.2009	114	17	108	119	88	198	150,47
10.06.2009	114	17	108	119	88	198	472,33
03.06.2009	114	17	108	119	88	198	126,77
03.06.2009	114	17	108	119	88	198	84,34
03.06.2009	114	17	108	119	88	198	95,38
03.06.2009	114	17	108	119	88	198	91,91
03.06.2009	114	17	108	119	88	198	227,11
03.06.2009	114	17	108	119	88	198	196,15
04.06.2009	114	17	108	119	88	198	226,65
04.06.2009	114	17	108	119	88	198	250,46

04.06.2009	114	17	108	119	88	198	270,26
04.06.2009	114	17	108	119	88	198	111,66
04.06.2009	114	17	108	119	88	198	163,75
04.06.2009	114	17	108	119	88	198	437,17
04.06.2009	114	17	108	119	88	198	405,83
04.06.2009	114	17	108	119	88	198	383,98
05.06.2009	114	17	108	119	88	198	435,07
05.06.2009	114	17	108	119	88	198	164,17
05.06.2009	114	17	108	119	88	198	455,44
24.06.2009	115	14	98	119	88	198	0,00
24.06.2009	115	14	98	119	88	198	119,33
24.06.2009	115	14	98	119	88	198	120,27
24.06.2009	115	14	98	119	88	198	115,67
24.06.2009	115	14	98	119	88	198	103,27
24.06.2009	115	14	98	119	88	198	126,67
25.06.2009	115	14	98	119	88	198	120,64
25.06.2009	115	14	98	119	88	198	87,79
25.06.2009	115	14	98	119	88	198	106,61
25.06.2009	115	14	98	119	88	198	92,73
25.06.2009	115	14	98	119	88	198	217,25
25.06.2009	115	14	98	119	88	198	82,05
25.06.2009	115	14	98	119	88	198	83,17
25.06.2009	115	14	98	119	88	198	10,18
26.06.2009	115	14	98	119	88	198	33,18
26.06.2009	115	14	98	119	88	198	1,78
26.06.2009	115	14	98	119	88	198	41,04
17.06.2009	115	14	98	119	88	198	156,99
17.06.2009	115	14	98	119	88	198	87,33
17.06.2009	115	14	98	119	88	198	143,23
17.06.2009	115	14	98	119	88	198	143,11
18.06.2009	115	14	98	119	88	198	116,88
18.06.2009	115	14	98	119	88	198	143,10
18.06.2009	115	14	98	119	88	198	102,63

18.06.2009	115	14	98	119	88	198	114,79
18.06.2009	115	14	98	119	88	198	100,40
18.06.2009	115	14	98	119	88	198	137,52
18.06.2009	115	14	98	119	88	198	132,37
18.06.2009	115	14	98	119	88	198	118,64
19.06.2009	115	14	98	119	88	198	94,29
19.06.2009	115	14	98	119	88	198	121,06
19.06.2009	115	14	98	119	88	198	146,73
10.06.2009	115	14	98	119	88	198	130,55
10.06.2009	115	14	98	119	88	198	158,46
10.06.2009	115	14	98	119	88	198	112,88
10.06.2009	115	14	98	119	88	198	146,60
10.06.2009	115	14	98	119	88	198	144,02
10.06.2009	115	14	98	119	88	198	157,32
11.06.2009	115	14	98	119	88	198	91,51
11.06.2009	115	14	98	119	88	198	79,27
11.06.2009	115	14	98	119	88	198	114,17
11.06.2009	115	14	98	119	88	198	115,71
11.06.2009	115	14	98	119	88	198	173,14
11.06.2009	115	14	98	119	88	198	128,14
11.06.2009	115	14	98	119	88	198	94,35
11.06.2009	115	14	98	119	88	198	100,52
12.06.2009	115	14	98	119	88	198	150,42
12.06.2009	115	14	98	119	88	198	136,68
12.06.2009	115	14	98	119	88	198	83,99
03.06.2009	115	14	98	119	88	198	135,99
03.06.2009	115	14	98	119	88	198	84,22
03.06.2009	115	14	98	119	88	198	100,24
03.06.2009	115	14	98	119	88	198	90,61
03.06.2009	115	14	98	119	88	198	93,13
04.06.2009	115	14	98	119	88	198	102,24
04.06.2009	115	14	98	119	88	198	117,94
04.06.2009	115	14	98	119	88	198	134,25

04.06.2009	115	14	98	119	88	198	84,52
04.06.2009	115	14	98	119	88	198	138,43
04.06.2009	115	14	98	119	88	198	161,46
04.06.2009	115	14	98	119	88	198	98,80
04.06.2009	115	14	98	119	88	198	102,16
05.06.2009	115	14	98	119	88	198	105,97
05.06.2009	115	14	98	119	88	198	98,92
05.06.2009	115	14	98	119	88	198	159,41
17.06.2009	116	60	172	119	88	198	154,61
17.06.2009	116	60	172	119	88	198	301,59
17.06.2009	116	60	172	119	88	198	337,85
10.06.2009	116	60	172	119	88	198	142,00
10.06.2009	116	60	172	119	88	198	150,50
10.06.2009	116	60	172	119	88	198	94,30
10.06.2009	116	60	172	119	88	198	149,51
10.06.2009	116	60	172	119	88	198	179,22
10.06.2009	116	60	172	119	88	198	132,66
11.06.2009	116	60	172	119	88	198	90,37
11.06.2009	116	60	172	119	88	198	79,80
11.06.2009	116	60	172	119	88	198	113,49
11.06.2009	116	60	172	119	88	198	123,48
11.06.2009	116	60	172	119	88	198	152,67
11.06.2009	116	60	172	119	88	198	124,83
11.06.2009	116	60	172	119	88	198	81,10
11.06.2009	116	60	172	119	88	198	86,55
12.06.2009	116	60	172	119	88	198	130,19
12.06.2009	116	60	172	119	88	198	91,53
12.06.2009	116	60	172	119	88	198	89,37
03.06.2009	116	60	172	119	88	198	124,71
03.06.2009	116	60	172	119	88	198	84,38
03.06.2009	116	60	172	119	88	198	116,68
03.06.2009	116	60	172	119	88	198	90,25
03.06.2009	116	60	172	119	88	198	91,15

03.06.2009	116	60	172	119	88	198	80,66
04.06.2009	116	60	172	119	88	198	24,74
04.06.2009	116	60	172	119	88	198	73,63
04.06.2009	116	60	172	119	88	198	49,81
04.06.2009	116	60	172	119	88	198	44,04
04.06.2009	116	60	172	119	88	198	92,61
04.06.2009	116	60	172	119	88	198	110,70
04.06.2009	116	60	172	119	88	198	85,70
04.06.2009	116	60	172	119	88	198	90,49
05.06.2009	116	60	172	119	88	198	115,97
05.06.2009	116	60	172	119	88	198	91,54
05.06.2009	116	60	172	119	88	198	130,12
24.06.2009	117	29	130	119	88	198	0,00
24.06.2009	117	29	130	119	88	198	184,31
24.06.2009	117	29	130	119	88	198	177,60
24.06.2009	117	29	130	119	88	198	177,59
24.06.2009	117	29	130	119	88	198	174,05
24.06.2009	117	29	130	119	88	198	179,28
25.06.2009	117	29	130	119	88	198	180,16
25.06.2009	117	29	130	119	88	198	92,20
25.06.2009	117	29	130	119	88	198	142,62
25.06.2009	117	29	130	119	88	198	270,77
25.06.2009	117	29	130	119	88	198	99,65
25.06.2009	117	29	130	119	88	198	122,23
25.06.2009	117	29	130	119	88	198	72,31
26.06.2009	117	29	130	119	88	198	77,44
26.06.2009	117	29	130	119	88	198	68,94
26.06.2009	117	29	130	119	88	198	99,22
17.06.2009	117	29	130	119	88	198	148,12
17.06.2009	117	29	130	119	88	198	117,32
17.06.2009	117	29	130	119	88	198	129,44
17.06.2009	117	29	130	119	88	198	79,25
17.06.2009	117	29	130	119	88	198	134,26

17.06.2009	117	29	130	119	88	198	126,16
18.06.2009	117	29	130	119	88	198	109,94
18.06.2009	117	29	130	119	88	198	123,46
18.06.2009	117	29	130	119	88	198	89,69
18.06.2009	117	29	130	119	88	198	114,36
18.06.2009	117	29	130	119	88	198	98,64
18.06.2009	117	29	130	119	88	198	128,32
18.06.2009	117	29	130	119	88	198	122,90
18.06.2009	117	29	130	119	88	198	108,34
19.06.2009	117	29	130	119	88	198	90,54
19.06.2009	117	29	130	119	88	198	113,23
19.06.2009	117	29	130	119	88	198	131,55
10.06.2009	117	29	130	119	88	198	134,14
10.06.2009	117	29	130	119	88	198	142,07
10.06.2009	117	29	130	119	88	198	75,33
10.06.2009	117	29	130	119	88	198	159,92
10.06.2009	117	29	130	119	88	198	141,39
10.06.2009	117	29	130	119	88	198	122,90
11.06.2009	117	29	130	119	88	198	79,05
11.06.2009	117	29	130	119	88	198	81,94
11.06.2009	117	29	130	119	88	198	120,62
11.06.2009	117	29	130	119	88	198	120,79
11.06.2009	117	29	130	119	88	198	137,75
11.06.2009	117	29	130	119	88	198	134,62
11.06.2009	117	29	130	119	88	198	92,47
11.06.2009	117	29	130	119	88	198	95,82
12.06.2009	117	29	130	119	88	198	124,43
12.06.2009	117	29	130	119	88	198	92,72
12.06.2009	117	29	130	119	88	198	80,33
03.06.2009	117	29	130	119	88	198	134,02
03.06.2009	117	29	130	119	88	198	73,16
03.06.2009	117	29	130	119	88	198	90,19
03.06.2009	117	29	130	119	88	198	90,11

03.06.2009	117	29	130	119	88	198	92,57
03.06.2009	117	29	130	119	88	198	82,34
04.06.2009	117	29	130	119	88	198	101,53
04.06.2009	117	29	130	119	88	198	112,32
04.06.2009	117	29	130	119	88	198	128,32
04.06.2009	117	29	130	119	88	198	41,94
04.06.2009	117	29	130	119	88	198	90,77
04.06.2009	117	29	130	119	88	198	108,01
04.06.2009	117	29	130	119	88	198	85,06
04.06.2009	117	29	130	119	88	198	88,13
05.06.2009	117	29	130	119	88	198	114,52
05.06.2009	117	29	130	119	88	198	87,71
05.06.2009	117	29	130	119	88	198	130,10
24.06.2009	118	41	141	119	88	198	0,00
24.06.2009	118	41	141	119	88	198	893,23
24.06.2009	118	41	141	119	88	198	885,69
24.06.2009	118	41	141	119	88	198	1054,80
24.06.2009	118	41	141	119	88	198	1016,83
24.06.2009	118	41	141	119	88	198	1040,89
25.06.2009	118	41	141	119	88	198	1057,47
25.06.2009	118	41	141	119	88	198	1042,26
25.06.2009	118	41	141	119	88	198	1050,59
25.06.2009	118	41	141	119	88	198	1004,88
25.06.2009	118	41	141	119	88	198	1169,78
25.06.2009	118	41	141	119	88	198	822,77
25.06.2009	118	41	141	119	88	198	844,97
25.06.2009	118	41	141	119	88	198	874,32
26.06.2009	118	41	141	119	88	198	785,80
26.06.2009	118	41	141	119	88	198	929,50
26.06.2009	118	41	141	119	88	198	974,93
17.06.2009	118	41	141	119	88	198	1092,59
17.06.2009	118	41	141	119	88	198	1043,42
17.06.2009	118	41	141	119	88	198	987,45

17.06.2009	118	41	141	119	88	198	955,11
17.06.2009	118	41	141	119	88	198	915,42
17.06.2009	118	41	141	119	88	198	1064,78
18.06.2009	118	41	141	119	88	198	883,03
18.06.2009	118	41	141	119	88	198	913,28
18.06.2009	118	41	141	119	88	198	975,05
18.06.2009	118	41	141	119	88	198	872,75
18.06.2009	118	41	141	119	88	198	868,22
18.06.2009	118	41	141	119	88	198	985,62
18.06.2009	118	41	141	119	88	198	1064,15
18.06.2009	118	41	141	119	88	198	1057,69
19.06.2009	118	41	141	119	88	198	1011,12
19.06.2009	118	41	141	119	88	198	994,85
19.06.2009	118	41	141	119	88	198	985,35
10.06.2009	118	41	141	119	88	198	1060,89
10.06.2009	118	41	141	119	88	198	477,56
10.06.2009	118	41	141	119	88	198	963,78
10.06.2009	118	41	141	119	88	198	1038,45
10.06.2009	118	41	141	119	88	198	1084,93
10.06.2009	118	41	141	119	88	198	1051,13
11.06.2009	118	41	141	119	88	198	890,51
11.06.2009	118	41	141	119	88	198	1011,76
11.06.2009	118	41	141	119	88	198	925,40
11.06.2009	118	41	141	119	88	198	974,92
11.06.2009	118	41	141	119	88	198	920,89
11.06.2009	118	41	141	119	88	198	1001,91
11.06.2009	118	41	141	119	88	198	974,63
11.06.2009	118	41	141	119	88	198	966,10
12.06.2009	118	41	141	119	88	198	1075,83
12.06.2009	118	41	141	119	88	198	1031,17
12.06.2009	118	41	141	119	88	198	909,49
03.06.2009	118	41	141	119	88	198	967,35
03.06.2009	118	41	141	119	88	198	1015,23

04.06.2009	118	41	141	119	88	198	1027,30
04.06.2009	118	41	141	119	88	198	1036,91
04.06.2009	118	41	141	119	88	198	1064,66
04.06.2009	118	41	141	119	88	198	792,20
04.06.2009	118	41	141	119	88	198	878,24
04.06.2009	118	41	141	119	88	198	926,03
04.06.2009	118	41	141	119	88	198	1019,87
04.06.2009	118	41	141	119	88	198	878,95
05.06.2009	118	41	141	119	88	198	1046,20
05.06.2009	118	41	141	119	88	198	930,98
05.06.2009	118	41	141	119	88	198	902,88
17.06.2009	106	25	122	120	24	122	927,33
10.06.2009	106	25	122	120	24	122	924,78
10.06.2009	106	25	122	120	24	122	920,27
10.06.2009	106	25	122	120	24	122	949,71
10.06.2009	106	25	122	120	24	122	929,47
10.06.2009	106	25	122	120	24	122	940,67
10.06.2009	106	25	122	120	24	122	936,47
11.06.2009	106	25	122	120	24	122	937,00
11.06.2009	106	25	122	120	24	122	951,35
11.06.2009	106	25	122	120	24	122	935,65
11.06.2009	106	25	122	120	24	122	926,14
11.06.2009	106	25	122	120	24	122	953,15
11.06.2009	106	25	122	120	24	122	945,93
11.06.2009	106	25	122	120	24	122	941,86
11.06.2009	106	25	122	120	24	122	945,04
12.06.2009	106	25	122	120	24	122	932,74
12.06.2009	106	25	122	120	24	122	949,02
12.06.2009	106	25	122	120	24	122	946,46
03.06.2009	106	25	122	120	24	122	870,25
03.06.2009	106	25	122	120	24	122	990,85
04.06.2009	106	25	122	120	24	122	926,75
04.06.2009	106	25	122	120	24	122	934,49

04.06.2009	106	25	122	120	24	122	912,85
04.06.2009	106	25	122	120	24	122	892,41
04.06.2009	106	25	122	120	24	122	773,48
04.06.2009	106	25	122	120	24	122	935,63
04.06.2009	106	25	122	120	24	122	948,38
04.06.2009	106	25	122	120	24	122	953,01
05.06.2009	106	25	122	120	24	122	1001,34
05.06.2009	106	25	122	120	24	122	941,17
05.06.2009	106	25	122	120	24	122	933,95
17.06.2009	112	17	111	120	24	122	49,93
17.06.2009	112	17	111	120	24	122	54,72
17.06.2009	112	17	111	120	24	122	51,48
17.06.2009	112	17	111	120	24	122	33,43
17.06.2009	112	17	111	120	24	122	43,75
17.06.2009	112	17	111	120	24	122	23,39
18.06.2009	112	17	111	120	24	122	65,57
18.06.2009	112	17	111	120	24	122	35,52
18.06.2009	112	17	111	120	24	122	40,24
18.06.2009	112	17	111	120	24	122	21,34
18.06.2009	112	17	111	120	24	122	19,57
18.06.2009	112	17	111	120	24	122	56,14
18.06.2009	112	17	111	120	24	122	57,65
19.06.2009	112	17	111	120	24	122	18,58
19.06.2009	112	17	111	120	24	122	47,49
19.06.2009	112	17	111	120	24	122	28,53
10.06.2009	112	17	111	120	24	122	55,80
10.06.2009	112	17	111	120	24	122	42,09
10.06.2009	112	17	111	120	24	122	62,00
10.06.2009	112	17	111	120	24	122	58,74
10.06.2009	112	17	111	120	24	122	16,52
10.06.2009	112	17	111	120	24	122	3,46
11.06.2009	112	17	111	120	24	122	15,39
11.06.2009	112	17	111	120	24	122	72,77

11.06.2009	112	17	111	120	24	122	40,82
11.06.2009	112	17	111	120	24	122	40,80
11.06.2009	112	17	111	120	24	122	22,18
11.06.2009	112	17	111	120	24	122	57,16
11.06.2009	112	17	111	120	24	122	13,80
12.06.2009	112	17	111	120	24	122	20,02
12.06.2009	112	17	111	120	24	122	76,47
12.06.2009	112	17	111	120	24	122	82,57
03.06.2009	112	17	111	120	24	122	103,36
03.06.2009	112	17	111	120	24	122	64,38
03.06.2009	112	17	111	120	24	122	74,03
03.06.2009	112	17	111	120	24	122	62,63
03.06.2009	112	17	111	120	24	122	93,08
04.06.2009	112	17	111	120	24	122	49,86
04.06.2009	112	17	111	120	24	122	58,25
04.06.2009	112	17	111	120	24	122	52,90
04.06.2009	112	17	111	120	24	122	28,43
04.06.2009	112	17	111	120	24	122	82,89
04.06.2009	112	17	111	120	24	122	76,45
04.06.2009	112	17	111	120	24	122	152,54
04.06.2009	112	17	111	120	24	122	109,45
05.06.2009	112	17	111	120	24	122	120,49
05.06.2009	112	17	111	120	24	122	112,85
05.06.2009	112	17	111	120	24	122	63,69
17.06.2009	113	19	106	120	24	122	96,62
17.06.2009	113	19	106	120	24	122	63,79
17.06.2009	113	19	106	120	24	122	24,73
17.06.2009	113	19	106	120	24	122	70,35
17.06.2009	113	19	106	120	24	122	72,16
17.06.2009	113	19	106	120	24	122	16,10
18.06.2009	113	19	106	120	24	122	5,82
18.06.2009	113	19	106	120	24	122	74,22
18.06.2009	113	19	106	120	24	122	7,25

18.06.2009	113	19	106	120	24	122	66,92
18.06.2009	113	19	106	120	24	122	75,00
18.06.2009	113	19	106	120	24	122	1,18
18.06.2009	113	19	106	120	24	122	73,61
19.06.2009	113	19	106	120	24	122	66,73
19.06.2009	113	19	106	120	24	122	15,60
19.06.2009	113	19	106	120	24	122	3,06
10.06.2009	113	19	106	120	24	122	63,21
10.06.2009	113	19	106	120	24	122	14,86
10.06.2009	113	19	106	120	24	122	9,91
10.06.2009	113	19	106	120	24	122	82,88
10.06.2009	113	19	106	120	24	122	82,74
10.06.2009	113	19	106	120	24	122	82,27
11.06.2009	113	19	106	120	24	122	34,82
11.06.2009	113	19	106	120	24	122	16,53
11.06.2009	113	19	106	120	24	122	78,46
11.06.2009	113	19	106	120	24	122	28,60
11.06.2009	113	19	106	120	24	122	72,70
11.06.2009	113	19	106	120	24	122	69,84
11.06.2009	113	19	106	120	24	122	53,52
11.06.2009	113	19	106	120	24	122	74,70
12.06.2009	113	19	106	120	24	122	65,08
12.06.2009	113	19	106	120	24	122	15,51
12.06.2009	113	19	106	120	24	122	64,97
03.06.2009	113	19	106	120	24	122	47,31
03.06.2009	113	19	106	120	24	122	37,07
03.06.2009	113	19	106	120	24	122	33,29
03.06.2009	113	19	106	120	24	122	60,72
03.06.2009	113	19	106	120	24	122	7,56
04.06.2009	113	19	106	120	24	122	89,89
04.06.2009	113	19	106	120	24	122	77,89
04.06.2009	113	19	106	120	24	122	74,08
04.06.2009	113	19	106	120	24	122	85,75

04.06.2009	113	19	106	120	24	122	89,92
04.06.2009	113	19	106	120	24	122	51,34
04.06.2009	113	19	106	120	24	122	68,90
04.06.2009	113	19	106	120	24	122	82,67
05.06.2009	113	19	106	120	24	122	28,26
05.06.2009	113	19	106	120	24	122	30,53
05.06.2009	113	19	106	120	24	122	72,06
17.06.2009	114	17	108	120	24	122	315,08
17.06.2009	114	17	108	120	24	122	65,28
17.06.2009	114	17	108	120	24	122	163,96
17.06.2009	114	17	108	120	24	122	71,15
17.06.2009	114	17	108	120	24	122	128,62
17.06.2009	114	17	108	120	24	122	99,42
18.06.2009	114	17	108	120	24	122	114,33
18.06.2009	114	17	108	120	24	122	117,61
18.06.2009	114	17	108	120	24	122	46,36
18.06.2009	114	17	108	120	24	122	61,45
18.06.2009	114	17	108	120	24	122	74,50
18.06.2009	114	17	108	120	24	122	78,79
18.06.2009	114	17	108	120	24	122	150,74
19.06.2009	114	17	108	120	24	122	78,98
19.06.2009	114	17	108	120	24	122	107,56
19.06.2009	114	17	108	120	24	122	39,74
10.06.2009	114	17	108	120	24	122	337,44
03.06.2009	114	17	108	120	24	122	68,32
03.06.2009	114	17	108	120	24	122	1,47
03.06.2009	114	17	108	120	24	122	43,64
03.06.2009	114	17	108	120	24	122	142,41
03.06.2009	114	17	108	120	24	122	157,61
04.06.2009	114	17	108	120	24	122	125,04
04.06.2009	114	17	108	120	24	122	148,95
04.06.2009	114	17	108	120	24	122	145,08
04.06.2009	114	17	108	120	24	122	89,49

04.06.2009	114	17	108	120	24	122	87,71
04.06.2009	114	17	108	120	24	122	328,43
04.06.2009	114	17	108	120	24	122	333,91
04.06.2009	114	17	108	120	24	122	293,25
05.06.2009	114	17	108	120	24	122	393,60
05.06.2009	114	17	108	120	24	122	92,05
05.06.2009	114	17	108	120	24	122	337,13
17.06.2009	115	14	98	120	24	122	21,76
17.06.2009	115	14	98	120	24	122	8,07
17.06.2009	115	14	98	120	24	122	34,51
17.06.2009	115	14	98	120	24	122	18,88
18.06.2009	115	14	98	120	24	122	13,27
18.06.2009	115	14	98	120	24	122	29,62
18.06.2009	115	14	98	120	24	122	24,51
18.06.2009	115	14	98	120	24	122	13,32
18.06.2009	115	14	98	120	24	122	11,06
18.06.2009	115	14	98	120	24	122	29,56
18.06.2009	115	14	98	120	24	122	20,51
19.06.2009	115	14	98	120	24	122	10,75
19.06.2009	115	14	98	120	24	122	31,69
19.06.2009	115	14	98	120	24	122	25,38
10.06.2009	115	14	98	120	24	122	9,24
10.06.2009	115	14	98	120	24	122	6,13
10.06.2009	115	14	98	120	24	122	35,30
10.06.2009	115	14	98	120	24	122	16,72
10.06.2009	115	14	98	120	24	122	15,05
10.06.2009	115	14	98	120	24	122	34,47
11.06.2009	115	14	98	120	24	122	11,25
11.06.2009	115	14	98	120	24	122	0,86
11.06.2009	115	14	98	120	24	122	18,28
11.06.2009	115	14	98	120	24	122	32,13
11.06.2009	115	14	98	120	24	122	32,58
11.06.2009	115	14	98	120	24	122	8,75

11.06.2009	115	14	98	120	24	122	11,51
11.06.2009	115	14	98	120	24	122	14,29
12.06.2009	115	14	98	120	24	122	35,03
12.06.2009	115	14	98	120	24	122	46,90
12.06.2009	115	14	98	120	24	122	7,22
03.06.2009	115	14	98	120	24	122	79,00
03.06.2009	115	14	98	120	24	122	6,84
03.06.2009	115	14	98	120	24	122	48,24
03.06.2009	115	14	98	120	24	122	11,10
04.06.2009	115	14	98	120	24	122	3,63
04.06.2009	115	14	98	120	24	122	15,89
04.06.2009	115	14	98	120	24	122	8,85
04.06.2009	115	14	98	120	24	122	60,22
04.06.2009	115	14	98	120	24	122	60,28
04.06.2009	115	14	98	120	24	122	60,15
04.06.2009	115	14	98	120	24	122	27,81
04.06.2009	115	14	98	120	24	122	11,99
05.06.2009	115	14	98	120	24	122	66,91
05.06.2009	115	14	98	120	24	122	19,35
05.06.2009	115	14	98	120	24	122	40,29
17.06.2009	116	60	172	120	24	122	21,85
17.06.2009	116	60	172	120	24	122	400,49
17.06.2009	116	60	172	120	24	122	398,84
10.06.2009	116	60	172	120	24	122	7,98
10.06.2009	116	60	172	120	24	122	3,63
10.06.2009	116	60	172	120	24	122	18,84
10.06.2009	116	60	172	120	24	122	12,69
10.06.2009	116	60	172	120	24	122	28,48
10.06.2009	116	60	172	120	24	122	0,33
11.06.2009	116	60	172	120	24	122	11,02
11.06.2009	116	60	172	120	24	122	1,90
11.06.2009	116	60	172	120	24	122	20,05
11.06.2009	116	60	172	120	24	122	20,84

11.06.2009	116	60	172	120	24	122	17,97
11.06.2009	116	60	172	120	24	122	0,48
11.06.2009	116	60	172	120	24	122	17,31
11.06.2009	116	60	172	120	24	122	11,26
12.06.2009	116	60	172	120	24	122	26,87
12.06.2009	116	60	172	120	24	122	3,26
12.06.2009	116	60	172	120	24	122	12,49
03.06.2009	116	60	172	120	24	122	63,27
03.06.2009	116	60	172	120	24	122	22,87
03.06.2009	116	60	172	120	24	122	46,87
03.06.2009	116	60	172	120	24	122	8,97
03.06.2009	116	60	172	120	24	122	69,91
04.06.2009	116	60	172	120	24	122	93,98
04.06.2009	116	60	172	120	24	122	78,02
04.06.2009	116	60	172	120	24	122	76,49
04.06.2009	116	60	172	120	24	122	15,16
04.06.2009	116	60	172	120	24	122	9,65
04.06.2009	116	60	172	120	24	122	2,04
04.06.2009	116	60	172	120	24	122	18,71
04.06.2009	116	60	172	120	24	122	0,27
05.06.2009	116	60	172	120	24	122	82,70
05.06.2009	116	60	172	120	24	122	11,97
05.06.2009	116	60	172	120	24	122	14,14
17.06.2009	117	29	130	120	24	122	50,84
17.06.2009	117	29	130	120	24	122	9,18
17.06.2009	117	29	130	120	24	122	7,31
17.06.2009	117	29	130	120	24	122	4,29
17.06.2009	117	29	130	120	24	122	17,05
17.06.2009	117	29	130	120	24	122	5,11
18.06.2009	117	29	130	120	24	122	6,66
18.06.2009	117	29	130	120	24	122	14,05
18.06.2009	117	29	130	120	24	122	7,13
18.06.2009	117	29	130	120	24	122	9,15

18.06.2009	117	29	130	120	24	122	0,51
18.06.2009	117	29	130	120	24	122	11,59
18.06.2009	117	29	130	120	24	122	14,75
19.06.2009	117	29	130	120	24	122	0,67
19.06.2009	117	29	130	120	24	122	23,96
19.06.2009	117	29	130	120	24	122	4,36
10.06.2009	117	29	130	120	24	122	1,36
10.06.2009	117	29	130	120	24	122	11,54
10.06.2009	117	29	130	120	24	122	2,52
10.06.2009	117	29	130	120	24	122	1,98
10.06.2009	117	29	130	120	24	122	18,18
10.06.2009	117	29	130	120	24	122	15,85
11.06.2009	117	29	130	120	24	122	6,52
11.06.2009	117	29	130	120	24	122	2,94
11.06.2009	117	29	130	120	24	122	7,34
11.06.2009	117	29	130	120	24	122	25,46
11.06.2009	117	29	130	120	24	122	12,12
11.06.2009	117	29	130	120	24	122	15,00
11.06.2009	117	29	130	120	24	122	9,87
11.06.2009	117	29	130	120	24	122	11,49
12.06.2009	117	29	130	120	24	122	14,05
12.06.2009	117	29	130	120	24	122	4,18
12.06.2009	117	29	130	120	24	122	3,44
03.06.2009	117	29	130	120	24	122	78,46
03.06.2009	117	29	130	120	24	122	16,58
03.06.2009	117	29	130	120	24	122	45,56
03.06.2009	117	29	130	120	24	122	10,50
03.06.2009	117	29	130	120	24	122	69,98
04.06.2009	117	29	130	120	24	122	4,23
04.06.2009	117	29	130	120	24	122	10,34
04.06.2009	117	29	130	120	24	122	3,29
04.06.2009	117	29	130	120	24	122	12,03
04.06.2009	117	29	130	120	24	122	7,80

04.06.2009	117	29	130	120	24	122	1,59
04.06.2009	117	29	130	120	24	122	18,04
04.06.2009	117	29	130	120	24	122	2,91
05.06.2009	117	29	130	120	24	122	80,71
05.06.2009	117	29	130	120	24	122	8,43
05.06.2009	117	29	130	120	24	122	14,26
17.06.2009	118	41	141	120	24	122	928,35
17.06.2009	118	41	141	120	24	122	936,41
17.06.2009	118	41	141	120	24	122	855,37
17.06.2009	118	41	141	120	24	122	874,55
17.06.2009	118	41	141	120	24	122	786,47
17.06.2009	118	41	141	120	24	122	942,83
18.06.2009	118	41	141	120	24	122	777,91
18.06.2009	118	41	141	120	24	122	788,66
18.06.2009	118	41	141	120	24	122	893,44
18.06.2009	118	41	141	120	24	122	765,27
18.06.2009	118	41	141	120	24	122	769,37
18.06.2009	118	41	141	120	24	122	864,38
18.06.2009	118	41	141	120	24	122	949,33
19.06.2009	118	41	141	120	24	122	922,73
19.06.2009	118	41	141	120	24	122	895,43
19.06.2009	118	41	141	120	24	122	857,08
10.06.2009	118	41	141	120	24	122	927,86
10.06.2009	118	41	141	120	24	122	325,19
10.06.2009	118	41	141	120	24	122	887,83
10.06.2009	118	41	141	120	24	122	878,94
10.06.2009	118	41	141	120	24	122	934,35
10.06.2009	118	41	141	120	24	122	919,73
11.06.2009	118	41	141	120	24	122	807,93
11.06.2009	118	41	141	120	24	122	934,70
11.06.2009	118	41	141	120	24	122	804,53
11.06.2009	118	41	141	120	24	122	833,18
11.06.2009	118	41	141	120	24	122	782,15

11.06.2009	118	41	141	120	24	122	878,80
11.06.2009	118	41	141	120	24	122	891,19
11.06.2009	118	41	141	120	24	122	878,29
12.06.2009	118	41	141	120	24	122	961,88
12.06.2009	118	41	141	120	24	122	940,21
12.06.2009	118	41	141	120	24	122	832,35
03.06.2009	118	41	141	120	24	122	886,73
03.06.2009	118	41	141	120	24	122	988,62
04.06.2009	118	41	141	120	24	122	924,15
04.06.2009	118	41	141	120	24	122	936,35
04.06.2009	118	41	141	120	24	122	941,41
04.06.2009	118	41	141	120	24	122	759,73
04.06.2009	118	41	141	120	24	122	795,82
04.06.2009	118	41	141	120	24	122	817,65
04.06.2009	118	41	141	120	24	122	949,10
04.06.2009	118	41	141	120	24	122	788,22
05.06.2009	118	41	141	120	24	122	1009,25
05.06.2009	118	41	141	120	24	122	851,52
05.06.2009	118	41	141	120	24	122	783,73
17.06.2009	119	88	198	120	24	122	166,95
17.06.2009	119	88	198	120	24	122	117,24
17.06.2009	119	88	198	120	24	122	133,17
17.06.2009	119	88	198	120	24	122	83,17
17.06.2009	119	88	198	120	24	122	133,47
17.06.2009	119	88	198	120	24	122	125,17
18.06.2009	119	88	198	120	24	122	105,15
18.06.2009	119	88	198	120	24	122	125,34
18.06.2009	119	88	198	120	24	122	85,68
18.06.2009	119	88	198	120	24	122	107,89
18.06.2009	119	88	198	120	24	122	99,08
18.06.2009	119	88	198	120	24	122	127,63
18.06.2009	119	88	198	120	24	122	119,23
19.06.2009	119	88	198	120	24	122	91,14

19.06.2009	119	88	198	120	24	122	105,82
19.06.2009	119	88	198	120	24	122	135,07
10.06.2009	119	88	198	120	24	122	135,50
10.06.2009	119	88	198	120	24	122	152,67
10.06.2009	119	88	198	120	24	122	77,60
10.06.2009	119	88	198	120	24	122	161,23
10.06.2009	119	88	198	120	24	122	153,22
10.06.2009	119	88	198	120	24	122	132,91
11.06.2009	119	88	198	120	24	122	83,14
11.06.2009	119	88	198	120	24	122	79,28
11.06.2009	119	88	198	120	24	122	121,48
11.06.2009	119	88	198	120	24	122	143,32
11.06.2009	119	88	198	120	24	122	140,64
11.06.2009	119	88	198	120	24	122	125,22
11.06.2009	119	88	198	120	24	122	84,30
11.06.2009	119	88	198	120	24	122	89,16
12.06.2009	119	88	198	120	24	122	118,45
12.06.2009	119	88	198	120	24	122	92,87
12.06.2009	119	88	198	120	24	122	77,14
03.06.2009	119	88	198	120	24	122	92,29
03.06.2009	119	88	198	120	24	122	94,75
03.06.2009	119	88	198	120	24	122	70,19
03.06.2009	119	88	198	120	24	122	84,70
03.06.2009	119	88	198	120	24	122	57,38
04.06.2009	119	88	198	120	24	122	104,48
04.06.2009	119	88	198	120	24	122	102,16
04.06.2009	119	88	198	120	24	122	125,81
04.06.2009	119	88	198	120	24	122	36,44
04.06.2009	119	88	198	120	24	122	82,97
04.06.2009	119	88	198	120	24	122	108,85
04.06.2009	119	88	198	120	24	122	72,60
04.06.2009	119	88	198	120	24	122	90,73
05.06.2009	119	88	198	120	24	122	58,48

05.06.2009	119	88	198	120	24	122	79,63
05.06.2009	119	88	198	120	24	122	119,65
25.06.2009	106	25	122	121	40	136	69,36
17.06.2009	106	25	122	121	40	136	946,96
10.06.2009	106	25	122	121	40	136	1031,10
10.06.2009	106	25	122	121	40	136	920,72
10.06.2009	106	25	122	121	40	136	933,57
10.06.2009	106	25	122	121	40	136	942,56
10.06.2009	106	25	122	121	40	136	964,78
10.06.2009	106	25	122	121	40	136	937,18
11.06.2009	106	25	122	121	40	136	939,43
11.06.2009	106	25	122	121	40	136	941,42
11.06.2009	106	25	122	121	40	136	935,36
11.06.2009	106	25	122	121	40	136	941,55
11.06.2009	106	25	122	121	40	136	949,52
11.06.2009	106	25	122	121	40	136	932,04
11.06.2009	106	25	122	121	40	136	946,94
11.06.2009	106	25	122	121	40	136	945,74
12.06.2009	106	25	122	121	40	136	917,47
12.06.2009	106	25	122	121	40	136	953,67
12.06.2009	106	25	122	121	40	136	947,84
03.06.2009	106	25	122	121	40	136	863,31
03.06.2009	106	25	122	121	40	136	896,92
04.06.2009	106	25	122	121	40	136	906,75
04.06.2009	106	25	122	121	40	136	974,52
04.06.2009	106	25	122	121	40	136	890,67
04.06.2009	106	25	122	121	40	136	768,00
04.06.2009	106	25	122	121	40	136	934,16
04.06.2009	106	25	122	121	40	136	934,74
04.06.2009	106	25	122	121	40	136	956,09
05.06.2009	106	25	122	121	40	136	916,00
05.06.2009	106	25	122	121	40	136	928,83
05.06.2009	106	25	122	121	40	136	921,68

25.06.2009	112	17	111	121	40	136	15,57
17.06.2009	112	17	111	121	40	136	54,12
17.06.2009	112	17	111	121	40	136	41,23
17.06.2009	112	17	111	121	40	136	47,63
17.06.2009	112	17	111	121	40	136	38,32
17.06.2009	112	17	111	121	40	136	37,90
17.06.2009	112	17	111	121	40	136	20,89
18.06.2009	112	17	111	121	40	136	64,23
18.06.2009	112	17	111	121	40	136	32,22
18.06.2009	112	17	111	121	40	136	34,56
18.06.2009	112	17	111	121	40	136	26,07
18.06.2009	112	17	111	121	40	136	20,44
18.06.2009	112	17	111	121	40	136	60,52
18.06.2009	112	17	111	121	40	136	57,76
18.06.2009	112	17	111	121	40	136	53,05
19.06.2009	112	17	111	121	40	136	16,68
19.06.2009	112	17	111	121	40	136	44,70
19.06.2009	112	17	111	121	40	136	12,21
10.06.2009	112	17	111	121	40	136	150,28
10.06.2009	112	17	111	121	40	136	41,96
10.06.2009	112	17	111	121	40	136	52,63
10.06.2009	112	17	111	121	40	136	65,68
10.06.2009	112	17	111	121	40	136	11,22
10.06.2009	112	17	111	121	40	136	4,92
11.06.2009	112	17	111	121	40	136	18,36
11.06.2009	112	17	111	121	40	136	71,83
11.06.2009	112	17	111	121	40	136	40,00
11.06.2009	112	17	111	121	40	136	58,56
11.06.2009	112	17	111	121	40	136	12,43
11.06.2009	112	17	111	121	40	136	57,03
11.06.2009	112	17	111	121	40	136	15,94
12.06.2009	112	17	111	121	40	136	4,42
12.06.2009	112	17	111	121	40	136	78,75

12.06.2009	112	17	111	121	40	136	80,49
03.06.2009	112	17	111	121	40	136	56,07
03.06.2009	112	17	111	121	40	136	45,44
03.06.2009	112	17	111	121	40	136	59,10
03.06.2009	112	17	111	121	40	136	53,89
03.06.2009	112	17	111	121	40	136	61,51
03.06.2009	112	17	111	121	40	136	15,89
04.06.2009	112	17	111	121	40	136	37,34
04.06.2009	112	17	111	121	40	136	100,53
04.06.2009	112	17	111	121	40	136	36,15
04.06.2009	112	17	111	121	40	136	78,73
04.06.2009	112	17	111	121	40	136	77,23
04.06.2009	112	17	111	121	40	136	143,23
04.06.2009	112	17	111	121	40	136	111,58
05.06.2009	112	17	111	121	40	136	55,44
05.06.2009	112	17	111	121	40	136	103,52
05.06.2009	112	17	111	121	40	136	64,83
25.06.2009	113	19	106	121	40	136	78,82
17.06.2009	113	19	106	121	40	136	82,82
17.06.2009	113	19	106	121	40	136	90,58
17.06.2009	113	19	106	121	40	136	31,30
17.06.2009	113	19	106	121	40	136	65,14
17.06.2009	113	19	106	121	40	136	75,08
17.06.2009	113	19	106	121	40	136	29,29
18.06.2009	113	19	106	121	40	136	11,10
18.06.2009	113	19	106	121	40	136	76,04
18.06.2009	113	19	106	121	40	136	21,98
18.06.2009	113	19	106	121	40	136	66,94
18.06.2009	113	19	106	121	40	136	72,15
18.06.2009	113	19	106	121	40	136	20,40
18.06.2009	113	19	106	121	40	136	73,49
18.06.2009	113	19	106	121	40	136	4,88
19.06.2009	113	19	106	121	40	136	68,66

19.06.2009	113	19	106	121	40	136	24,56
19.06.2009	113	19	106	121	40	136	20,30
10.06.2009	113	19	106	121	40	136	54,81
10.06.2009	113	19	106	121	40	136	14,27
10.06.2009	113	19	106	121	40	136	18,64
10.06.2009	113	19	106	121	40	136	69,79
10.06.2009	113	19	106	121	40	136	60,31
10.06.2009	113	19	106	121	40	136	81,43
11.06.2009	113	19	106	121	40	136	31,75
11.06.2009	113	19	106	121	40	136	29,39
11.06.2009	113	19	106	121	40	136	78,60
11.06.2009	113	19	106	121	40	136	19,40
11.06.2009	113	19	106	121	40	136	75,97
11.06.2009	113	19	106	121	40	136	80,61
11.06.2009	113	19	106	121	40	136	44,62
11.06.2009	113	19	106	121	40	136	74,56
12.06.2009	113	19	106	121	40	136	77,99
12.06.2009	113	19	106	121	40	136	11,28
12.06.2009	113	19	106	121	40	136	61,99
03.06.2009	113	19	106	121	40	136	68,55
03.06.2009	113	19	106	121	40	136	32,51
03.06.2009	113	19	106	121	40	136	41,99
03.06.2009	113	19	106	121	40	136	86,30
03.06.2009	113	19	106	121	40	136	71,75
03.06.2009	113	19	106	121	40	136	101,11
04.06.2009	113	19	106	121	40	136	105,48
04.06.2009	113	19	106	121	40	136	42,47
04.06.2009	113	19	106	121	40	136	89,17
04.06.2009	113	19	106	121	40	136	95,09
04.06.2009	113	19	106	121	40	136	53,82
04.06.2009	113	19	106	121	40	136	85,25
04.06.2009	113	19	106	121	40	136	79,52
05.06.2009	113	19	106	121	40	136	89,66

05.06.2009	113	19	106	121	40	136	38,74
05.06.2009	113	19	106	121	40	136	86,09
17.06.2009	114	17	108	121	40	136	333,28
17.06.2009	114	17	108	121	40	136	89,29
17.06.2009	114	17	108	121	40	136	170,89
17.06.2009	114	17	108	121	40	136	66,18
17.06.2009	114	17	108	121	40	136	129,41
17.06.2009	114	17	108	121	40	136	99,11
18.06.2009	114	17	108	121	40	136	117,74
18.06.2009	114	17	108	121	40	136	118,56
18.06.2009	114	17	108	121	40	136	58,61
18.06.2009	114	17	108	121	40	136	55,33
18.06.2009	114	17	108	121	40	136	72,82
18.06.2009	114	17	108	121	40	136	78,91
18.06.2009	114	17	108	121	40	136	150,62
18.06.2009	114	17	108	121	40	136	125,85
19.06.2009	114	17	108	121	40	136	80,28
19.06.2009	114	17	108	121	40	136	108,49
19.06.2009	114	17	108	121	40	136	59,17
10.06.2009	114	17	108	121	40	136	440,54
03.06.2009	114	17	108	121	40	136	10,87
03.06.2009	114	17	108	121	40	136	0,83
03.06.2009	114	17	108	121	40	136	7,19
03.06.2009	114	17	108	121	40	136	10,73
03.06.2009	114	17	108	121	40	136	139,49
03.06.2009	114	17	108	121	40	136	67,40
04.06.2009	114	17	108	121	40	136	121,70
04.06.2009	114	17	108	121	40	136	196,72
04.06.2009	114	17	108	121	40	136	95,19
04.06.2009	114	17	108	121	40	136	83,69
04.06.2009	114	17	108	121	40	136	327,24
04.06.2009	114	17	108	121	40	136	321,49
04.06.2009	114	17	108	121	40	136	296,16

05.06.2009	114	17	108	121	40	136	312,44
05.06.2009	114	17	108	121	40	136	83,56
05.06.2009	114	17	108	121	40	136	325,88
25.06.2009	115	14	98	121	40	136	14,50
17.06.2009	115	14	98	121	40	136	8,03
17.06.2009	115	14	98	121	40	136	13,16
17.06.2009	115	14	98	121	40	136	16,41
17.06.2009	115	14	98	121	40	136	20,77
18.06.2009	115	14	98	121	40	136	8,36
18.06.2009	115	14	98	121	40	136	23,82
18.06.2009	115	14	98	121	40	136	13,34
18.06.2009	115	14	98	121	40	136	18,91
18.06.2009	115	14	98	121	40	136	10,82
18.06.2009	115	14	98	121	40	136	12,84
18.06.2009	115	14	98	121	40	136	20,61
18.06.2009	115	14	98	121	40	136	22,07
19.06.2009	115	14	98	121	40	136	9,00
19.06.2009	115	14	98	121	40	136	23,43
19.06.2009	115	14	98	121	40	136	6,89
10.06.2009	115	14	98	121	40	136	106,60
10.06.2009	115	14	98	121	40	136	6,28
10.06.2009	115	14	98	121	40	136	23,21
10.06.2009	115	14	98	121	40	136	3,42
10.06.2009	115	14	98	121	40	136	12,59
10.06.2009	115	14	98	121	40	136	32,86
11.06.2009	115	14	98	121	40	136	14,39
11.06.2009	115	14	98	121	40	136	13,85
11.06.2009	115	14	98	121	40	136	17,68
11.06.2009	115	14	98	121	40	136	24,23
11.06.2009	115	14	98	121	40	136	30,88
11.06.2009	115	14	98	121	40	136	9,65
11.06.2009	115	14	98	121	40	136	19,72
11.06.2009	115	14	98	121	40	136	16,40

12.06.2009	115	14	98	121	40	136	23,15
12.06.2009	115	14	98	121	40	136	49,68
12.06.2009	115	14	98	121	40	136	10,46
03.06.2009	115	14	98	121	40	136	21,05
03.06.2009	115	14	98	121	40	136	0,88
03.06.2009	115	14	98	121	40	136	3,25
03.06.2009	115	14	98	121	40	136	12,74
03.06.2009	115	14	98	121	40	136	5,58
04.06.2009	115	14	98	121	40	136	12,70
04.06.2009	115	14	98	121	40	136	77,50
04.06.2009	115	14	98	121	40	136	65,90
04.06.2009	115	14	98	121	40	136	56,13
04.06.2009	115	14	98	121	40	136	60,64
04.06.2009	115	14	98	121	40	136	15,37
04.06.2009	115	14	98	121	40	136	14,51
05.06.2009	115	14	98	121	40	136	21,44
05.06.2009	115	14	98	121	40	136	7,99
05.06.2009	115	14	98	121	40	136	35,38
17.06.2009	116	60	172	121	40	136	8,66
17.06.2009	116	60	172	121	40	136	408,09
17.06.2009	116	60	172	121	40	136	393,34
10.06.2009	116	60	172	121	40	136	123,28
10.06.2009	116	60	172	121	40	136	3,92
10.06.2009	116	60	172	121	40	136	2,00
10.06.2009	116	60	172	121	40	136	0,91
10.06.2009	116	60	172	121	40	136	46,82
10.06.2009	116	60	172	121	40	136	2,64
11.06.2009	116	60	172	121	40	136	14,21
11.06.2009	116	60	172	121	40	136	14,43
11.06.2009	116	60	172	121	40	136	19,45
11.06.2009	116	60	172	121	40	136	2,90
11.06.2009	116	60	172	121	40	136	13,32
11.06.2009	116	60	172	121	40	136	17,12

11.06.2009	116	60	172	121	40	136	7,98
11.06.2009	116	60	172	121	40	136	8,40
12.06.2009	116	60	172	121	40	136	2,94
12.06.2009	116	60	172	121	40	136	6,16
12.06.2009	116	60	172	121	40	136	15,06
03.06.2009	116	60	172	121	40	136	5,44
03.06.2009	116	60	172	121	40	136	2,58
03.06.2009	116	60	172	121	40	136	16,68
03.06.2009	116	60	172	121	40	136	12,54
03.06.2009	116	60	172	121	40	136	4,97
03.06.2009	116	60	172	121	40	136	56,14
04.06.2009	116	60	172	121	40	136	100,45
04.06.2009	116	60	172	121	40	136	48,52
04.06.2009	116	60	172	121	40	136	6,14
04.06.2009	116	60	172	121	40	136	4,40
04.06.2009	116	60	172	121	40	136	1,57
04.06.2009	116	60	172	121	40	136	1,25
04.06.2009	116	60	172	121	40	136	2,99
05.06.2009	116	60	172	121	40	136	6,82
05.06.2009	116	60	172	121	40	136	0,90
05.06.2009	116	60	172	121	40	136	1,39
25.06.2009	117	29	130	121	40	136	59,12
17.06.2009	117	29	130	121	40	136	21,06
17.06.2009	117	29	130	121	40	136	20,92
17.06.2009	117	29	130	121	40	136	14,42
17.06.2009	117	29	130	121	40	136	4,61
17.06.2009	117	29	130	121	40	136	1,74
17.06.2009	117	29	130	121	40	136	8,80
18.06.2009	117	29	130	121	40	136	1,34
18.06.2009	117	29	130	121	40	136	8,67
18.06.2009	117	29	130	121	40	136	8,62
18.06.2009	117	29	130	121	40	136	14,08
18.06.2009	117	29	130	121	40	136	2,78

18.06.2009	117	29	130	121	40	136	9,95
18.06.2009	117	29	130	121	40	136	14,81
18.06.2009	117	29	130	121	40	136	3,87
19.06.2009	117	29	130	121	40	136	2,57
19.06.2009	117	29	130	121	40	136	15,21
19.06.2009	117	29	130	121	40	136	24,05
10.06.2009	117	29	130	121	40	136	114,34
10.06.2009	117	29	130	121	40	136	11,03
10.06.2009	117	29	130	121	40	136	19,35
10.06.2009	117	29	130	121	40	136	12,96
10.06.2009	117	29	130	121	40	136	9,35
10.06.2009	117	29	130	121	40	136	13,91
11.06.2009	117	29	130	121	40	136	3,37
11.06.2009	117	29	130	121	40	136	10,68
11.06.2009	117	29	130	121	40	136	6,55
11.06.2009	117	29	130	121	40	136	18,49
11.06.2009	117	29	130	121	40	136	16,77
11.06.2009	117	29	130	121	40	136	2,43
11.06.2009	117	29	130	121	40	136	18,43
11.06.2009	117	29	130	121	40	136	14,14
12.06.2009	117	29	130	121	40	136	9,94
12.06.2009	117	29	130	121	40	136	7,78
12.06.2009	117	29	130	121	40	136	7,20
03.06.2009	117	29	130	121	40	136	20,79
03.06.2009	117	29	130	121	40	136	14,12
03.06.2009	117	29	130	121	40	136	22,19
03.06.2009	117	29	130	121	40	136	12,32
03.06.2009	117	29	130	121	40	136	5,33
03.06.2009	117	29	130	121	40	136	53,79
04.06.2009	117	29	130	121	40	136	18,15
04.06.2009	117	29	130	121	40	136	72,66
04.06.2009	117	29	130	121	40	136	3,12
04.06.2009	117	29	130	121	40	136	2,50

04.06.2009	117	29	130	121	40	136	2,12
04.06.2009	117	29	130	121	40	136	0,70
04.06.2009	117	29	130	121	40	136	0,32
05.06.2009	117	29	130	121	40	136	8,11
05.06.2009	117	29	130	121	40	136	4,15
05.06.2009	117	29	130	121	40	136	1,19
25.06.2009	118	41	141	121	40	136	922,69
17.06.2009	118	41	141	121	40	136	948,12
17.06.2009	118	41	141	121	40	136	912,31
17.06.2009	118	41	141	121	40	136	848,03
17.06.2009	118	41	141	121	40	136	879,92
17.06.2009	118	41	141	121	40	136	781,82
17.06.2009	118	41	141	121	40	136	941,35
18.06.2009	118	41	141	121	40	136	774,23
18.06.2009	118	41	141	121	40	136	786,66
18.06.2009	118	41	141	121	40	136	886,39
18.06.2009	118	41	141	121	40	136	764,79
18.06.2009	118	41	141	121	40	136	772,41
18.06.2009	118	41	141	121	40	136	860,77
18.06.2009	118	41	141	121	40	136	949,46
18.06.2009	118	41	141	121	40	136	953,20
19.06.2009	118	41	141	121	40	136	921,15
19.06.2009	118	41	141	121	40	136	891,96
19.06.2009	118	41	141	121	40	136	847,04
10.06.2009	118	41	141	121	40	136	1033,22
10.06.2009	118	41	141	121	40	136	325,56
10.06.2009	118	41	141	121	40	136	871,89
10.06.2009	118	41	141	121	40	136	891,91
10.06.2009	118	41	141	121	40	136	958,43
10.06.2009	118	41	141	121	40	136	920,42
11.06.2009	118	41	141	121	40	136	810,04
11.06.2009	118	41	141	121	40	136	924,91
11.06.2009	118	41	141	121	40	136	804,13

11.06.2009	118	41	141	121	40	136	849,13
11.06.2009	118	41	141	121	40	136	779,13
11.06.2009	118	41	141	121	40	136	865,41
11.06.2009	118	41	141	121	40	136	895,77
11.06.2009	118	41	141	121	40	136	878,86
12.06.2009	118	41	141	121	40	136	947,21
12.06.2009	118	41	141	121	40	136	944,79
12.06.2009	118	41	141	121	40	136	833,37
03.06.2009	118	41	141	121	40	136	880,17
03.06.2009	118	41	141	121	40	136	894,73
04.06.2009	118	41	141	121	40	136	908,61
04.06.2009	118	41	141	121	40	136	1001,95
04.06.2009	118	41	141	121	40	136	760,25
04.06.2009	118	41	141	121	40	136	790,34
04.06.2009	118	41	141	121	40	136	816,35
04.06.2009	118	41	141	121	40	136	935,51
04.06.2009	118	41	141	121	40	136	791,14
05.06.2009	118	41	141	121	40	136	924,89
05.06.2009	118	41	141	121	40	136	839,24
05.06.2009	118	41	141	121	40	136	773,17
25.06.2009	119	88	198	121	40	136	85,42
17.06.2009	119	88	198	121	40	136	154,37
17.06.2009	119	88	198	121	40	136	135,38
17.06.2009	119	88	198	121	40	136	140,17
17.06.2009	119	88	198	121	40	136	78,42
17.06.2009	119	88	198	121	40	136	134,41
17.06.2009	119	88	198	121	40	136	124,21
18.06.2009	119	88	198	121	40	136	108,82
18.06.2009	119	88	198	121	40	136	126,82
18.06.2009	119	88	198	121	40	136	89,52
18.06.2009	119	88	198	121	40	136	109,20
18.06.2009	119	88	198	121	40	136	96,10
18.06.2009	119	88	198	121	40	136	126,07

18.06.2009	119	88	198	121	40	136	119,11
18.06.2009	119	88	198	121	40	136	108,09
19.06.2009	119	88	198	121	40	136	92,38
19.06.2009	119	88	198	121	40	136	106,48
19.06.2009	119	88	198	121	40	136	140,14
10.06.2009	119	88	198	121	40	136	82,01
10.06.2009	119	88	198	121	40	136	152,34
10.06.2009	119	88	198	121	40	136	92,44
10.06.2009	119	88	198	121	40	136	149,09
10.06.2009	119	88	198	121	40	136	133,25
10.06.2009	119	88	198	121	40	136	132,63
11.06.2009	119	88	198	121	40	136	81,38
11.06.2009	119	88	198	121	40	136	87,36
11.06.2009	119	88	198	121	40	136	121,79
11.06.2009	119	88	198	121	40	136	126,31
11.06.2009	119	88	198	121	40	136	143,03
11.06.2009	119	88	198	121	40	136	136,98
11.06.2009	119	88	198	121	40	136	81,50
11.06.2009	119	88	198	121	40	136	89,20
12.06.2009	119	88	198	121	40	136	129,18
12.06.2009	119	88	198	121	40	136	88,78
12.06.2009	119	88	198	121	40	136	76,28
03.06.2009	119	88	198	121	40	136	122,14
03.06.2009	119	88	198	121	40	136	84,99
03.06.2009	119	88	198	121	40	136	102,55
03.06.2009	119	88	198	121	40	136	102,31
03.06.2009	119	88	198	121	40	136	88,74
03.06.2009	119	88	198	121	40	136	128,76
04.06.2009	119	88	198	121	40	136	130,10
04.06.2009	119	88	198	121	40	136	88,21
04.06.2009	119	88	198	121	40	136	41,07
04.06.2009	119	88	198	121	40	136	88,39
04.06.2009	119	88	198	121	40	136	109,95

04.06.2009	119	88	198	121	40	136	84,46
04.06.2009	119	88	198	121	40	136	87,82
05.06.2009	119	88	198	121	40	136	122,64
05.06.2009	119	88	198	121	40	136	91,82
05.06.2009	119	88	198	121	40	136	129,72
17.06.2009	120	24	122	121	40	136	29,78
17.06.2009	120	24	122	121	40	136	27,30
17.06.2009	120	24	122	121	40	136	7,71
17.06.2009	120	24	122	121	40	136	5,56
17.06.2009	120	24	122	121	40	136	18,79
17.06.2009	120	24	122	121	40	136	13,55
18.06.2009	120	24	122	121	40	136	5,33
18.06.2009	120	24	122	121	40	136	6,24
18.06.2009	120	24	122	121	40	136	14,81
18.06.2009	120	24	122	121	40	136	6,54
18.06.2009	120	24	122	121	40	136	3,11
18.06.2009	120	24	122	121	40	136	21,17
18.06.2009	120	24	122	121	40	136	0,13
19.06.2009	120	24	122	121	40	136	1,96
19.06.2009	120	24	122	121	40	136	8,97
19.06.2009	120	24	122	121	40	136	20,74
10.06.2009	120	24	122	121	40	136	115,30
10.06.2009	120	24	122	121	40	136	0,65
10.06.2009	120	24	122	121	40	136	16,84
10.06.2009	120	24	122	121	40	136	13,42
10.06.2009	120	24	122	121	40	136	27,00
10.06.2009	120	24	122	121	40	136	2,44
11.06.2009	120	24	122	121	40	136	3,19
11.06.2009	120	24	122	121	40	136	13,12
11.06.2009	120	24	122	121	40	136	0,91
11.06.2009	120	24	122	121	40	136	18,43
11.06.2009	120	24	122	121	40	136	4,74
11.06.2009	120	24	122	121	40	136	17,05

11.06.2009	120	24	122	121	40	136	9,38
11.06.2009	120	24	122	121	40	136	3,02
12.06.2009	120	24	122	121	40	136	23,99
12.06.2009	120	24	122	121	40	136	4,86
12.06.2009	120	24	122	121	40	136	4,66
03.06.2009	120	24	122	121	40	136	58,03
03.06.2009	120	24	122	121	40	136	8,03
03.06.2009	120	24	122	121	40	136	53,01
03.06.2009	120	24	122	121	40	136	11,10
03.06.2009	120	24	122	121	40	136	93,94
04.06.2009	120	24	122	121	40	136	27,98
04.06.2009	120	24	122	121	40	136	69,37
04.06.2009	120	24	122	121	40	136	9,09
04.06.2009	120	24	122	121	40	136	5,49
04.06.2009	120	24	122	121	40	136	2,48
04.06.2009	120	24	122	121	40	136	17,83
04.06.2009	120	24	122	121	40	136	3,16
05.06.2009	120	24	122	121	40	136	87,96
05.06.2009	120	24	122	121	40	136	12,35
05.06.2009	120	24	122	121	40	136	14,74
25.06.2009	106	25	122	122	34	129	66,59
25.06.2009	106	25	122	122	34	129	92,24
25.06.2009	106	25	122	122	34	129	14,30
25.06.2009	106	25	122	122	34	129	34,67
25.06.2009	106	25	122	122	34	129	41,94
26.06.2009	106	25	122	122	34	129	37,72
26.06.2009	106	25	122	122	34	129	90,35
26.06.2009	106	25	122	122	34	129	76,65
17.06.2009	106	25	122	122	34	129	944,17
10.06.2009	106	25	122	122	34	129	935,74
10.06.2009	106	25	122	122	34	129	930,55
10.06.2009	106	25	122	122	34	129	938,40
10.06.2009	106	25	122	122	34	129	941,06

10.06.2009	106	25	122	122	34	129	944,69
11.06.2009	106	25	122	122	34	129	929,27
11.06.2009	106	25	122	122	34	129	941,11
11.06.2009	106	25	122	122	34	129	935,09
11.06.2009	106	25	122	122	34	129	954,85
11.06.2009	106	25	122	122	34	129	948,86
11.06.2009	106	25	122	122	34	129	944,44
11.06.2009	106	25	122	122	34	129	943,04
12.06.2009	106	25	122	122	34	129	923,07
12.06.2009	106	25	122	122	34	129	952,90
12.06.2009	106	25	122	122	34	129	945,81
03.06.2009	106	25	122	122	34	129	859,56
03.06.2009	106	25	122	122	34	129	934,82
04.06.2009	106	25	122	122	34	129	923,47
04.06.2009	106	25	122	122	34	129	926,06
04.06.2009	106	25	122	122	34	129	899,89
04.06.2009	106	25	122	122	34	129	903,66
04.06.2009	106	25	122	122	34	129	772,83
04.06.2009	106	25	122	122	34	129	933,56
04.06.2009	106	25	122	122	34	129	898,27
04.06.2009	106	25	122	122	34	129	956,07
05.06.2009	106	25	122	122	34	129	919,16
05.06.2009	106	25	122	122	34	129	929,07
05.06.2009	106	25	122	122	34	129	931,85
24.06.2009	112	17	111	122	34	129	0,00
24.06.2009	112	17	111	122	34	129	16,26
24.06.2009	112	17	111	122	34	129	12,61
24.06.2009	112	17	111	122	34	129	18,24
24.06.2009	112	17	111	122	34	129	23,76
24.06.2009	112	17	111	122	34	129	16,79
25.06.2009	112	17	111	122	34	129	14,29
25.06.2009	112	17	111	122	34	129	63,73
25.06.2009	112	17	111	122	34	129	19,97

25.06.2009	112	17	111	122	34	129	18,06
25.06.2009	112	17	111	122	34	129	16,98
25.06.2009	112	17	111	122	34	129	10,65
25.06.2009	112	17	111	122	34	129	21,32
25.06.2009	112	17	111	122	34	129	30,68
26.06.2009	112	17	111	122	34	129	33,13
26.06.2009	112	17	111	122	34	129	16,47
26.06.2009	112	17	111	122	34	129	16,71
17.06.2009	112	17	111	122	34	129	53,85
17.06.2009	112	17	111	122	34	129	52,16
17.06.2009	112	17	111	122	34	129	54,44
17.06.2009	112	17	111	122	34	129	34,30
17.06.2009	112	17	111	122	34	129	39,99
17.06.2009	112	17	111	122	34	129	32,50
18.06.2009	112	17	111	122	34	129	65,40
18.06.2009	112	17	111	122	34	129	36,54
18.06.2009	112	17	111	122	34	129	26,32
18.06.2009	112	17	111	122	34	129	31,30
18.06.2009	112	17	111	122	34	129	22,68
18.06.2009	112	17	111	122	34	129	57,82
18.06.2009	112	17	111	122	34	129	58,10
18.06.2009	112	17	111	122	34	129	54,24
19.06.2009	112	17	111	122	34	129	12,06
19.06.2009	112	17	111	122	34	129	40,59
19.06.2009	112	17	111	122	34	129	17,40
10.06.2009	112	17	111	122	34	129	50,14
10.06.2009	112	17	111	122	34	129	46,63
10.06.2009	112	17	111	122	34	129	65,76
10.06.2009	112	17	111	122	34	129	16,19
10.06.2009	112	17	111	122	34	129	12,82
11.06.2009	112	17	111	122	34	129	7,16
11.06.2009	112	17	111	122	34	129	71,55
11.06.2009	112	17	111	122	34	129	39,46

11.06.2009	112	17	111	122	34	129	60,78
11.06.2009	112	17	111	122	34	129	20,73
11.06.2009	112	17	111	122	34	129	53,53
12.06.2009	112	17	111	122	34	129	4,38
12.06.2009	112	17	111	122	34	129	77,45
12.06.2009	112	17	111	122	34	129	74,72
03.06.2009	112	17	111	122	34	129	54,51
03.06.2009	112	17	111	122	34	129	53,15
03.06.2009	112	17	111	122	34	129	64,25
03.06.2009	112	17	111	122	34	129	36,18
03.06.2009	112	17	111	122	34	129	55,44
03.06.2009	112	17	111	122	34	129	65,65
04.06.2009	112	17	111	122	34	129	42,84
04.06.2009	112	17	111	122	34	129	52,86
04.06.2009	112	17	111	122	34	129	42,03
04.06.2009	112	17	111	122	34	129	17,84
04.06.2009	112	17	111	122	34	129	81,98
04.06.2009	112	17	111	122	34	129	79,89
04.06.2009	112	17	111	122	34	129	97,07
04.06.2009	112	17	111	122	34	129	111,35
05.06.2009	112	17	111	122	34	129	58,11
05.06.2009	112	17	111	122	34	129	104,02
05.06.2009	112	17	111	122	34	129	65,26
24.06.2009	113	19	106	122	34	129	0,00
24.06.2009	113	19	106	122	34	129	71,22
24.06.2009	113	19	106	122	34	129	77,44
24.06.2009	113	19	106	122	34	129	71,35
24.06.2009	113	19	106	122	34	129	69,41
24.06.2009	113	19	106	122	34	129	83,89
25.06.2009	113	19	106	122	34	129	68,83
25.06.2009	113	19	106	122	34	129	70,05
25.06.2009	113	19	106	122	34	129	76,69
25.06.2009	113	19	106	122	34	129	76,31

25.06.2009	113	19	106	122	34	129	76,90
25.06.2009	113	19	106	122	34	129	78,99
25.06.2009	113	19	106	122	34	129	76,13
25.06.2009	113	19	106	122	34	129	76,71
26.06.2009	113	19	106	122	34	129	67,36
26.06.2009	113	19	106	122	34	129	70,45
26.06.2009	113	19	106	122	34	129	85,79
17.06.2009	113	19	106	122	34	129	83,18
17.06.2009	113	19	106	122	34	129	74,15
17.06.2009	113	19	106	122	34	129	5,39
17.06.2009	113	19	106	122	34	129	69,13
17.06.2009	113	19	106	122	34	129	72,64
17.06.2009	113	19	106	122	34	129	7,15
18.06.2009	113	19	106	122	34	129	3,91
18.06.2009	113	19	106	122	34	129	71,56
18.06.2009	113	19	106	122	34	129	27,11
18.06.2009	113	19	106	122	34	129	65,92
18.06.2009	113	19	106	122	34	129	71,29
18.06.2009	113	19	106	122	34	129	2,43
18.06.2009	113	19	106	122	34	129	73,07
18.06.2009	113	19	106	122	34	129	21,34
19.06.2009	113	19	106	122	34	129	72,97
19.06.2009	113	19	106	122	34	129	31,56
19.06.2009	113	19	106	122	34	129	13,31
10.06.2009	113	19	106	122	34	129	2,88
10.06.2009	113	19	106	122	34	129	17,59
10.06.2009	113	19	106	122	34	129	73,99
10.06.2009	113	19	106	122	34	129	82,34
10.06.2009	113	19	106	122	34	129	73,87
11.06.2009	113	19	106	122	34	129	44,42
11.06.2009	113	19	106	122	34	129	29,51
11.06.2009	113	19	106	122	34	129	78,77
11.06.2009	113	19	106	122	34	129	0,64

11.06.2009	113	19	106	122	34	129	76,40
11.06.2009	113	19	106	122	34	129	70,60
11.06.2009	113	19	106	122	34	129	47,68
12.06.2009	113	19	106	122	34	129	72,51
12.06.2009	113	19	106	122	34	129	10,98
12.06.2009	113	19	106	122	34	129	61,98
03.06.2009	113	19	106	122	34	129	69,92
03.06.2009	113	19	106	122	34	129	23,58
03.06.2009	113	19	106	122	34	129	37,87
03.06.2009	113	19	106	122	34	129	83,72
03.06.2009	113	19	106	122	34	129	71,00
03.06.2009	113	19	106	122	34	129	73,83
04.06.2009	113	19	106	122	34	129	93,55
04.06.2009	113	19	106	122	34	129	86,40
04.06.2009	113	19	106	122	34	129	87,26
04.06.2009	113	19	106	122	34	129	74,07
04.06.2009	113	19	106	122	34	129	90,33
04.06.2009	113	19	106	122	34	129	57,02
04.06.2009	113	19	106	122	34	129	118,23
04.06.2009	113	19	106	122	34	129	79,43
05.06.2009	113	19	106	122	34	129	86,45
05.06.2009	113	19	106	122	34	129	38,90
05.06.2009	113	19	106	122	34	129	74,95
17.06.2009	114	17	108	122	34	129	330,78
17.06.2009	114	17	108	122	34	129	77,77
17.06.2009	114	17	108	122	34	129	155,83
17.06.2009	114	17	108	122	34	129	70,21
17.06.2009	114	17	108	122	34	129	128,07
17.06.2009	114	17	108	122	34	129	94,98
18.06.2009	114	17	108	122	34	129	113,80
18.06.2009	114	17	108	122	34	129	113,94
18.06.2009	114	17	108	122	34	129	58,51
18.06.2009	114	17	108	122	34	129	49,78

18.06.2009	114	17	108	122	34	129	70,83
18.06.2009	114	17	108	122	34	129	76,94
18.06.2009	114	17	108	122	34	129	150,22
18.06.2009	114	17	108	122	34	129	136,49
19.06.2009	114	17	108	122	34	129	82,73
19.06.2009	114	17	108	122	34	129	112,74
19.06.2009	114	17	108	122	34	129	52,47
03.06.2009	114	17	108	122	34	129	12,38
03.06.2009	114	17	108	122	34	129	11,17
03.06.2009	114	17	108	122	34	129	0,67
03.06.2009	114	17	108	122	34	129	23,07
03.06.2009	114	17	108	122	34	129	133,94
03.06.2009	114	17	108	122	34	129	119,75
04.06.2009	114	17	108	122	34	129	119,33
04.06.2009	114	17	108	122	34	129	141,47
04.06.2009	114	17	108	122	34	129	132,11
04.06.2009	114	17	108	122	34	129	94,56
04.06.2009	114	17	108	122	34	129	86,80
04.06.2009	114	17	108	122	34	129	327,16
04.06.2009	114	17	108	122	34	129	282,09
04.06.2009	114	17	108	122	34	129	296,06
05.06.2009	114	17	108	122	34	129	315,71
05.06.2009	114	17	108	122	34	129	84,08
05.06.2009	114	17	108	122	34	129	335,40
24.06.2009	115	14	98	122	34	129	0,00
24.06.2009	115	14	98	122	34	129	12,06
24.06.2009	115	14	98	122	34	129	9,37
24.06.2009	115	14	98	122	34	129	15,02
24.06.2009	115	14	98	122	34	129	2,75
24.06.2009	115	14	98	122	34	129	9,85
25.06.2009	115	14	98	122	34	129	16,65
25.06.2009	115	14	98	122	34	129	24,97
25.06.2009	115	14	98	122	34	129	9,65

25.06.2009	115	14	98	122	34	129	16,93
25.06.2009	115	14	98	122	34	129	13,77
25.06.2009	115	14	98	122	34	129	14,52
25.06.2009	115	14	98	122	34	129	17,71
25.06.2009	115	14	98	122	34	129	9,10
26.06.2009	115	14	98	122	34	129	10,45
26.06.2009	115	14	98	122	34	129	13,07
26.06.2009	115	14	98	122	34	129	15,16
17.06.2009	115	14	98	122	34	129	2,32
17.06.2009	115	14	98	122	34	129	10,83
17.06.2009	115	14	98	122	34	129	23,90
17.06.2009	115	14	98	122	34	129	26,98
18.06.2009	115	14	98	122	34	129	14,82
18.06.2009	115	14	98	122	34	129	26,37
18.06.2009	115	14	98	122	34	129	4,92
18.06.2009	115	14	98	122	34	129	24,43
18.06.2009	115	14	98	122	34	129	13,22
18.06.2009	115	14	98	122	34	129	27,37
18.06.2009	115	14	98	122	34	129	21,06
18.06.2009	115	14	98	122	34	129	3,68
19.06.2009	115	14	98	122	34	129	4,84
19.06.2009	115	14	98	122	34	129	16,00
19.06.2009	115	14	98	122	34	129	13,00
10.06.2009	115	14	98	122	34	129	20,63
10.06.2009	115	14	98	122	34	129	17,52
10.06.2009	115	14	98	122	34	129	9,42
10.06.2009	115	14	98	122	34	129	14,72
10.06.2009	115	14	98	122	34	129	35,59
11.06.2009	115	14	98	122	34	129	2,16
11.06.2009	115	14	98	122	34	129	14,02
11.06.2009	115	14	98	122	34	129	17,39
11.06.2009	115	14	98	122	34	129	5,06
11.06.2009	115	14	98	122	34	129	34,23

11.06.2009	115	14	98	122	34	129	6,37
11.06.2009	115	14	98	122	34	129	16,86
12.06.2009	115	14	98	122	34	129	25,84
12.06.2009	115	14	98	122	34	129	48,44
12.06.2009	115	14	98	122	34	129	14,19
03.06.2009	115	14	98	122	34	129	21,92
03.06.2009	115	14	98	122	34	129	11,02
03.06.2009	115	14	98	122	34	129	5,99
03.06.2009	115	14	98	122	34	129	28,96
03.06.2009	115	14	98	122	34	129	0,75
04.06.2009	115	14	98	122	34	129	7,10
04.06.2009	115	14	98	122	34	129	9,11
04.06.2009	115	14	98	122	34	129	5,22
04.06.2009	115	14	98	122	34	129	65,81
04.06.2009	115	14	98	122	34	129	59,38
04.06.2009	115	14	98	122	34	129	63,00
04.06.2009	115	14	98	122	34	129	40,20
04.06.2009	115	14	98	122	34	129	14,37
05.06.2009	115	14	98	122	34	129	19,06
05.06.2009	115	14	98	122	34	129	8,46
05.06.2009	115	14	98	122	34	129	40,10
17.06.2009	116	60	172	122	34	129	1,69
17.06.2009	116	60	172	122	34	129	383,15
17.06.2009	116	60	172	122	34	129	396,55
10.06.2009	116	60	172	122	34	129	15,56
10.06.2009	116	60	172	122	34	129	5,95
10.06.2009	116	60	172	122	34	129	5,19
10.06.2009	116	60	172	122	34	129	28,77
10.06.2009	116	60	172	122	34	129	9,59
11.06.2009	116	60	172	122	34	129	1,15
11.06.2009	116	60	172	122	34	129	14,57
11.06.2009	116	60	172	122	34	129	19,16
11.06.2009	116	60	172	122	34	129	17,32

11.06.2009	116	60	172	122	34	129	11,20
11.06.2009	116	60	172	122	34	129	2,38
11.06.2009	116	60	172	122	34	129	10,07
12.06.2009	116	60	172	122	34	129	11,12
12.06.2009	116	60	172	122	34	129	6,59
12.06.2009	116	60	172	122	34	129	17,18
03.06.2009	116	60	172	122	34	129	6,97
03.06.2009	116	60	172	122	34	129	12,09
03.06.2009	116	60	172	122	34	129	22,55
03.06.2009	116	60	172	122	34	129	28,10
03.06.2009	116	60	172	122	34	129	2,59
03.06.2009	116	60	172	122	34	129	1,90
04.06.2009	116	60	172	122	34	129	97,00
04.06.2009	116	60	172	122	34	129	85,74
04.06.2009	116	60	172	122	34	129	89,71
04.06.2009	116	60	172	122	34	129	23,27
04.06.2009	116	60	172	122	34	129	8,89
04.06.2009	116	60	172	122	34	129	5,08
04.06.2009	116	60	172	122	34	129	53,30
04.06.2009	116	60	172	122	34	129	3,09
05.06.2009	116	60	172	122	34	129	3,58
05.06.2009	116	60	172	122	34	129	1,30
05.06.2009	116	60	172	122	34	129	10,88
24.06.2009	117	29	130	122	34	129	0,00
24.06.2009	117	29	130	122	34	129	72,34
24.06.2009	117	29	130	122	34	129	60,56
24.06.2009	117	29	130	122	34	129	68,93
24.06.2009	117	29	130	122	34	129	81,87
24.06.2009	117	29	130	122	34	129	72,47
25.06.2009	117	29	130	122	34	129	75,68
25.06.2009	117	29	130	122	34	129	2,36
25.06.2009	117	29	130	122	34	129	61,01
25.06.2009	117	29	130	122	34	129	67,55

25.06.2009	117	29	130	122	34	129	70,81
25.06.2009	117	29	130	122	34	129	54,86
25.06.2009	117	29	130	122	34	129	70,38
26.06.2009	117	29	130	122	34	129	73,56
26.06.2009	117	29	130	122	34	129	76,69
26.06.2009	117	29	130	122	34	129	84,51
17.06.2009	117	29	130	122	34	129	27,76
17.06.2009	117	29	130	122	34	129	4,30
17.06.2009	117	29	130	122	34	129	12,04
17.06.2009	117	29	130	122	34	129	5,43
17.06.2009	117	29	130	122	34	129	6,02
17.06.2009	117	29	130	122	34	129	14,89
18.06.2009	117	29	130	122	34	129	8,48
18.06.2009	117	29	130	122	34	129	7,09
18.06.2009	117	29	130	122	34	129	12,85
18.06.2009	117	29	130	122	34	129	19,53
18.06.2009	117	29	130	122	34	129	3,36
18.06.2009	117	29	130	122	34	129	8,99
18.06.2009	117	29	130	122	34	129	15,14
18.06.2009	117	29	130	122	34	129	22,14
19.06.2009	117	29	130	122	34	129	7,12
19.06.2009	117	29	130	122	34	129	7,94
19.06.2009	117	29	130	122	34	129	16,70
10.06.2009	117	29	130	122	34	129	4,40
10.06.2009	117	29	130	122	34	129	21,64
10.06.2009	117	29	130	122	34	129	7,94
10.06.2009	117	29	130	122	34	129	17,83
10.06.2009	117	29	130	122	34	129	6,38
11.06.2009	117	29	130	122	34	129	16,44
11.06.2009	117	29	130	122	34	129	10,82
11.06.2009	117	29	130	122	34	129	6,13
11.06.2009	117	29	130	122	34	129	4,33
11.06.2009	117	29	130	122	34	129	22,38

11.06.2009	117	29	130	122	34	129	12,66
11.06.2009	117	29	130	122	34	129	15,80
12.06.2009	117	29	130	122	34	129	1,82
12.06.2009	117	29	130	122	34	129	8,11
12.06.2009	117	29	130	122	34	129	12,22
03.06.2009	117	29	130	122	34	129	21,92
03.06.2009	117	29	130	122	34	129	3,47
03.06.2009	117	29	130	122	34	129	17,40
03.06.2009	117	29	130	122	34	129	27,07
03.06.2009	117	29	130	122	34	129	1,14
03.06.2009	117	29	130	122	34	129	1,04
04.06.2009	117	29	130	122	34	129	11,31
04.06.2009	117	29	130	122	34	129	5,26
04.06.2009	117	29	130	122	34	129	10,82
04.06.2009	117	29	130	122	34	129	20,41
04.06.2009	117	29	130	122	34	129	7,08
04.06.2009	117	29	130	122	34	129	4,90
04.06.2009	117	29	130	122	34	129	53,56
04.06.2009	117	29	130	122	34	129	0,36
05.06.2009	117	29	130	122	34	129	4,85
05.06.2009	117	29	130	122	34	129	3,83
05.06.2009	117	29	130	122	34	129	10,97
24.06.2009	118	41	141	122	34	129	0,00
24.06.2009	118	41	141	122	34	129	779,06
24.06.2009	118	41	141	122	34	129	766,79
24.06.2009	118	41	141	122	34	129	947,47
24.06.2009	118	41	141	122	34	129	917,43
24.06.2009	118	41	141	122	34	129	924,81
25.06.2009	118	41	141	122	34	129	950,74
25.06.2009	118	41	141	122	34	129	949,88
25.06.2009	118	41	141	122	34	129	948,97
25.06.2009	118	41	141	122	34	129	925,44
25.06.2009	118	41	141	122	34	129	945,50

25.06.2009	118	41	141	122	34	129	788,53
25.06.2009	118	41	141	122	34	129	771,15
25.06.2009	118	41	141	122	34	129	871,58
26.06.2009	118	41	141	122	34	129	775,41
26.06.2009	118	41	141	122	34	129	929,06
26.06.2009	118	41	141	122	34	129	936,94
17.06.2009	118	41	141	122	34	129	945,30
17.06.2009	118	41	141	122	34	129	928,76
17.06.2009	118	41	141	122	34	129	867,15
17.06.2009	118	41	141	122	34	129	876,00
17.06.2009	118	41	141	122	34	129	784,35
17.06.2009	118	41	141	122	34	129	949,39
18.06.2009	118	41	141	122	34	129	778,62
18.06.2009	118	41	141	122	34	129	791,17
18.06.2009	118	41	141	122	34	129	877,85
18.06.2009	118	41	141	122	34	129	765,97
18.06.2009	118	41	141	122	34	129	772,83
18.06.2009	118	41	141	122	34	129	865,30
18.06.2009	118	41	141	122	34	129	949,92
18.06.2009	118	41	141	122	34	129	937,43
19.06.2009	118	41	141	122	34	129	918,06
19.06.2009	118	41	141	122	34	129	886,51
19.06.2009	118	41	141	122	34	129	851,22
10.06.2009	118	41	141	122	34	129	339,98
10.06.2009	118	41	141	122	34	129	868,65
10.06.2009	118	41	141	122	34	129	887,93
10.06.2009	118	41	141	122	34	129	934,75
10.06.2009	118	41	141	122	34	129	927,91
11.06.2009	118	41	141	122	34	129	801,16
11.06.2009	118	41	141	122	34	129	924,60
11.06.2009	118	41	141	122	34	129	803,80
11.06.2009	118	41	141	122	34	129	861,65
11.06.2009	118	41	141	122	34	129	779,62

11.06.2009	118	41	141	122	34	129	877,41
11.06.2009	118	41	141	122	34	129	891,86
12.06.2009	118	41	141	122	34	129	952,61
12.06.2009	118	41	141	122	34	129	943,98
12.06.2009	118	41	141	122	34	129	830,86
03.06.2009	118	41	141	122	34	129	876,20
03.06.2009	118	41	141	122	34	129	934,16
04.06.2009	118	41	141	122	34	129	920,99
04.06.2009	118	41	141	122	34	129	927,92
04.06.2009	118	41	141	122	34	129	928,36
04.06.2009	118	41	141	122	34	129	769,63
04.06.2009	118	41	141	122	34	129	795,14
04.06.2009	118	41	141	122	34	129	816,07
04.06.2009	118	41	141	122	34	129	898,83
04.06.2009	118	41	141	122	34	129	791,05
05.06.2009	118	41	141	122	34	129	928,09
05.06.2009	118	41	141	122	34	129	839,51
05.06.2009	118	41	141	122	34	129	782,26
24.06.2009	119	88	198	122	34	129	0,00
24.06.2009	119	88	198	122	34	129	114,79
24.06.2009	119	88	198	122	34	129	119,28
24.06.2009	119	88	198	122	34	129	111,55
24.06.2009	119	88	198	122	34	129	101,39
24.06.2009	119	88	198	122	34	129	116,94
25.06.2009	119	88	198	122	34	129	109,44
25.06.2009	119	88	198	122	34	129	93,02
25.06.2009	119	88	198	122	34	129	104,75
25.06.2009	119	88	198	122	34	129	82,98
25.06.2009	119	88	198	122	34	129	224,45
25.06.2009	119	88	198	122	34	129	67,56
25.06.2009	119	88	198	122	34	129	81,01
25.06.2009	119	88	198	122	34	129	2,75
26.06.2009	119	88	198	122	34	129	22,84

26.06.2009	119	88	198	122	34	129	12,85
26.06.2009	119	88	198	122	34	129	55,68
17.06.2009	119	88	198	122	34	129	154,74
17.06.2009	119	88	198	122	34	129	120,51
17.06.2009	119	88	198	122	34	129	124,90
17.06.2009	119	88	198	122	34	129	82,44
17.06.2009	119	88	198	122	34	129	133,02
17.06.2009	119	88	198	122	34	129	120,94
18.06.2009	119	88	198	122	34	129	104,50
18.06.2009	119	88	198	122	34	129	122,25
18.06.2009	119	88	198	122	34	129	98,04
18.06.2009	119	88	198	122	34	129	109,08
18.06.2009	119	88	198	122	34	129	95,52
18.06.2009	119	88	198	122	34	129	125,68
18.06.2009	119	88	198	122	34	129	118,70
18.06.2009	119	88	198	122	34	129	120,41
19.06.2009	119	88	198	122	34	129	94,65
19.06.2009	119	88	198	122	34	129	110,55
19.06.2009	119	88	198	122	34	129	137,26
10.06.2009	119	88	198	122	34	129	138,58
10.06.2009	119	88	198	122	34	129	96,40
10.06.2009	119	88	198	122	34	129	152,17
10.06.2009	119	88	198	122	34	129	152,84
10.06.2009	119	88	198	122	34	129	125,83
11.06.2009	119	88	198	122	34	129	89,37
11.06.2009	119	88	198	122	34	129	87,68
11.06.2009	119	88	198	122	34	129	122,07
11.06.2009	119	88	198	122	34	129	116,51
11.06.2009	119	88	198	122	34	129	141,79
11.06.2009	119	88	198	122	34	129	126,23
11.06.2009	119	88	198	122	34	129	85,28
12.06.2009	119	88	198	122	34	129	124,63
12.06.2009	119	88	198	122	34	129	89,83

12.06.2009	119	88	198	122	34	129	79,46
03.06.2009	119	88	198	122	34	129	123,62
03.06.2009	119	88	198	122	34	129	74,19
03.06.2009	119	88	198	122	34	129	95,32
03.06.2009	119	88	198	122	34	129	111,47
03.06.2009	119	88	198	122	34	129	93,70
03.06.2009	119	88	198	122	34	129	81,31
04.06.2009	119	88	198	122	34	129	108,83
04.06.2009	119	88	198	122	34	129	110,24
04.06.2009	119	88	198	122	34	129	139,02
04.06.2009	119	88	198	122	34	129	24,57
04.06.2009	119	88	198	122	34	129	83,72
04.06.2009	119	88	198	122	34	129	110,03
04.06.2009	119	88	198	122	34	129	129,39
04.06.2009	119	88	198	122	34	129	87,91
05.06.2009	119	88	198	122	34	129	119,36
05.06.2009	119	88	198	122	34	129	91,54
05.06.2009	119	88	198	122	34	129	120,82
17.06.2009	120	24	122	122	34	129	23,24
17.06.2009	120	24	122	122	34	129	12,62
17.06.2009	120	24	122	122	34	129	19,34
17.06.2009	120	24	122	122	34	129	2,81
17.06.2009	120	24	122	122	34	129	11,10
17.06.2009	120	24	122	122	34	129	9,93
18.06.2009	120	24	122	122	34	129	1,97
18.06.2009	120	24	122	122	34	129	7,49
18.06.2009	120	24	122	122	34	129	19,98
18.06.2009	120	24	122	122	34	129	12,01
18.06.2009	120	24	122	122	34	129	3,86
18.06.2009	120	24	122	122	34	129	3,47
18.06.2009	120	24	122	122	34	129	0,60
19.06.2009	120	24	122	122	34	129	6,57
19.06.2009	120	24	122	122	34	129	16,03

19.06.2009	120	24	122	122	34	129	13,46
10.06.2009	120	24	122	122	34	129	15,86
10.06.2009	120	24	122	122	34	129	19,18
10.06.2009	120	24	122	122	34	129	9,06
10.06.2009	120	24	122	122	34	129	0,39
10.06.2009	120	24	122	122	34	129	9,64
11.06.2009	120	24	122	122	34	129	9,93
11.06.2009	120	24	122	122	34	129	13,29
11.06.2009	120	24	122	122	34	129	1,48
11.06.2009	120	24	122	122	34	129	28,95
11.06.2009	120	24	122	122	34	129	10,38
11.06.2009	120	24	122	122	34	129	2,41
11.06.2009	120	24	122	122	34	129	8,19
12.06.2009	120	24	122	122	34	129	15,75
12.06.2009	120	24	122	122	34	129	4,61
12.06.2009	120	24	122	122	34	129	10,92
03.06.2009	120	24	122	122	34	129	57,61
03.06.2009	120	24	122	122	34	129	0,87
03.06.2009	120	24	122	122	34	129	51,75
03.06.2009	120	24	122	122	34	129	11,25
03.06.2009	120	24	122	122	34	129	69,13
04.06.2009	120	24	122	122	34	129	7,11
04.06.2009	120	24	122	122	34	129	8,53
04.06.2009	120	24	122	122	34	129	13,23
04.06.2009	120	24	122	122	34	129	12,01
04.06.2009	120	24	122	122	34	129	0,91
04.06.2009	120	24	122	122	34	129	5,96
04.06.2009	120	24	122	122	34	129	57,99
04.06.2009	120	24	122	122	34	129	3,24
05.06.2009	120	24	122	122	34	129	85,16
05.06.2009	120	24	122	122	34	129	12,14
05.06.2009	120	24	122	122	34	129	3,92
25.06.2009	121	40	136	122	34	129	2,84

17.06.2009	121	40	136	122	34	129	6,99
17.06.2009	121	40	136	122	34	129	16,70
17.06.2009	121	40	136	122	34	129	26,00
17.06.2009	121	40	136	122	34	129	4,03
17.06.2009	121	40	136	122	34	129	7,74
17.06.2009	121	40	136	122	34	129	22,46
18.06.2009	121	40	136	122	34	129	7,19
18.06.2009	121	40	136	122	34	129	4,65
18.06.2009	121	40	136	122	34	129	8,55
18.06.2009	121	40	136	122	34	129	5,59
18.06.2009	121	40	136	122	34	129	2,41
18.06.2009	121	40	136	122	34	129	18,10
18.06.2009	121	40	136	122	34	129	0,48
18.06.2009	121	40	136	122	34	129	25,74
19.06.2009	121	40	136	122	34	129	4,64
19.06.2009	121	40	136	122	34	129	7,44
19.06.2009	121	40	136	122	34	129	7,35
10.06.2009	121	40	136	122	34	129	15,32
10.06.2009	121	40	136	122	34	129	6,20
10.06.2009	121	40	136	122	34	129	6,10
10.06.2009	121	40	136	122	34	129	26,63
10.06.2009	121	40	136	122	34	129	7,97
11.06.2009	121	40	136	122	34	129	13,11
11.06.2009	121	40	136	122	34	129	0,33
11.06.2009	121	40	136	122	34	129	0,56
11.06.2009	121	40	136	122	34	129	19,32
11.06.2009	121	40	136	122	34	129	6,41
11.06.2009	121	40	136	122	34	129	14,76
11.06.2009	121	40	136	122	34	129	3,91
12.06.2009	121	40	136	122	34	129	8,26
12.06.2009	121	40	136	122	34	129	1,32
12.06.2009	121	40	136	122	34	129	6,76
03.06.2009	121	40	136	122	34	129	2,52

03.06.2009	121	40	136	122	34	129	11,58
03.06.2009	121	40	136	122	34	129	7,33
03.06.2009	121	40	136	122	34	129	18,26
03.06.2009	121	40	136	122	34	129	6,34
03.06.2009	121	40	136	122	34	129	54,51
04.06.2009	121	40	136	122	34	129	19,92
04.06.2009	121	40	136	122	34	129	79,99
04.06.2009	121	40	136	122	34	129	18,49
04.06.2009	121	40	136	122	34	129	4,86
04.06.2009	121	40	136	122	34	129	3,64
04.06.2009	121	40	136	122	34	129	54,24
04.06.2009	121	40	136	122	34	129	0,37
05.06.2009	121	40	136	122	34	129	3,29
05.06.2009	121	40	136	122	34	129	0,54
05.06.2009	121	40	136	122	34	129	11,28
25.06.2009	106	25	122	123	33	137	62,72
25.06.2009	106	25	122	123	33	137	87,30
25.06.2009	106	25	122	123	33	137	42,75
25.06.2009	106	25	122	123	33	137	42,36
26.06.2009	106	25	122	123	33	137	36,96
26.06.2009	106	25	122	123	33	137	86,44
26.06.2009	106	25	122	123	33	137	72,22
17.06.2009	106	25	122	123	33	137	934,43
10.06.2009	106	25	122	123	33	137	878,73
10.06.2009	106	25	122	123	33	137	886,86
10.06.2009	106	25	122	123	33	137	897,64
10.06.2009	106	25	122	123	33	137	926,51
10.06.2009	106	25	122	123	33	137	938,56
10.06.2009	106	25	122	123	33	137	954,16
11.06.2009	106	25	122	123	33	137	974,17
11.06.2009	106	25	122	123	33	137	950,90
11.06.2009	106	25	122	123	33	137	948,37
11.06.2009	106	25	122	123	33	137	978,91

11.06.2009	106	25	122	123	33	137	899,05
11.06.2009	106	25	122	123	33	137	888,88
11.06.2009	106	25	122	123	33	137	909,64
11.06.2009	106	25	122	123	33	137	907,46
12.06.2009	106	25	122	123	33	137	875,34
12.06.2009	106	25	122	123	33	137	1058,13
12.06.2009	106	25	122	123	33	137	894,78
03.06.2009	106	25	122	123	33	137	1156,09
04.06.2009	106	25	122	123	33	137	1147,22
04.06.2009	106	25	122	123	33	137	1057,27
04.06.2009	106	25	122	123	33	137	1091,39
04.06.2009	106	25	122	123	33	137	1079,21
04.06.2009	106	25	122	123	33	137	959,46
04.06.2009	106	25	122	123	33	137	1099,29
04.06.2009	106	25	122	123	33	137	1107,33
04.06.2009	106	25	122	123	33	137	1088,14
05.06.2009	106	25	122	123	33	137	1102,61
05.06.2009	106	25	122	123	33	137	1082,02
05.06.2009	106	25	122	123	33	137	1074,83
24.06.2009	112	17	111	123	33	137	0,00
24.06.2009	112	17	111	123	33	137	3,96
24.06.2009	112	17	111	123	33	137	13,67
24.06.2009	112	17	111	123	33	137	12,75
24.06.2009	112	17	111	123	33	137	17,96
25.06.2009	112	17	111	123	33	137	12,67
25.06.2009	112	17	111	123	33	137	60,47
25.06.2009	112	17	111	123	33	137	6,44
25.06.2009	112	17	111	123	33	137	22,58
25.06.2009	112	17	111	123	33	137	27,80
25.06.2009	112	17	111	123	33	137	43,81
25.06.2009	112	17	111	123	33	137	22,93
26.06.2009	112	17	111	123	33	137	31,61
26.06.2009	112	17	111	123	33	137	16,42

26.06.2009	112	17	111	123	33	137	21,29
17.06.2009	112	17	111	123	33	137	42,45
17.06.2009	112	17	111	123	33	137	11,90
17.06.2009	112	17	111	123	33	137	10,65
17.06.2009	112	17	111	123	33	137	32,64
17.06.2009	112	17	111	123	33	137	42,87
17.06.2009	112	17	111	123	33	137	917,01
18.06.2009	112	17	111	123	33	137	22,10
18.06.2009	112	17	111	123	33	137	25,29
18.06.2009	112	17	111	123	33	137	69,61
18.06.2009	112	17	111	123	33	137	74,20
18.06.2009	112	17	111	123	33	137	10,81
18.06.2009	112	17	111	123	33	137	9,07
18.06.2009	112	17	111	123	33	137	16,37
19.06.2009	112	17	111	123	33	137	70,67
19.06.2009	112	17	111	123	33	137	33,60
19.06.2009	112	17	111	123	33	137	5,61
10.06.2009	112	17	111	123	33	137	5,74
10.06.2009	112	17	111	123	33	137	10,93
10.06.2009	112	17	111	123	33	137	12,60
10.06.2009	112	17	111	123	33	137	54,58
10.06.2009	112	17	111	123	33	137	18,56
10.06.2009	112	17	111	123	33	137	23,71
11.06.2009	112	17	111	123	33	137	64,17
11.06.2009	112	17	111	123	33	137	71,22
11.06.2009	112	17	111	123	33	137	42,04
11.06.2009	112	17	111	123	33	137	77,21
11.06.2009	112	17	111	123	33	137	57,45
11.06.2009	112	17	111	123	33	137	32,39
11.06.2009	112	17	111	123	33	137	44,39
12.06.2009	112	17	111	123	33	137	69,70
12.06.2009	112	17	111	123	33	137	172,03
12.06.2009	112	17	111	123	33	137	10,04

03.06.2009	112	17	111	123	33	137	42,12
03.06.2009	112	17	111	123	33	137	46,21
03.06.2009	112	17	111	123	33	137	11,60
03.06.2009	112	17	111	123	33	137	1,08
03.06.2009	112	17	111	123	33	137	256,94
04.06.2009	112	17	111	123	33	137	249,00
04.06.2009	112	17	111	123	33	137	167,41
04.06.2009	112	17	111	123	33	137	228,61
04.06.2009	112	17	111	123	33	137	178,41
04.06.2009	112	17	111	123	33	137	260,21
04.06.2009	112	17	111	123	33	137	229,58
04.06.2009	112	17	111	123	33	137	317,38
04.06.2009	112	17	111	123	33	137	243,34
05.06.2009	112	17	111	123	33	137	235,15
05.06.2009	112	17	111	123	33	137	250,76
05.06.2009	112	17	111	123	33	137	199,42
24.06.2009	113	19	106	123	33	137	0,00
24.06.2009	113	19	106	123	33	137	78,12
24.06.2009	113	19	106	123	33	137	72,89
24.06.2009	113	19	106	123	33	137	79,35
24.06.2009	113	19	106	123	33	137	82,43
25.06.2009	113	19	106	123	33	137	70,88
25.06.2009	113	19	106	123	33	137	74,70
25.06.2009	113	19	106	123	33	137	88,90
25.06.2009	113	19	106	123	33	137	73,30
25.06.2009	113	19	106	123	33	137	70,19
25.06.2009	113	19	106	123	33	137	122,83
25.06.2009	113	19	106	123	33	137	67,70
26.06.2009	113	19	106	123	33	137	70,44
26.06.2009	113	19	106	123	33	137	72,99
26.06.2009	113	19	106	123	33	137	82,73
17.06.2009	113	19	106	123	33	137	94,39
17.06.2009	113	19	106	123	33	137	118,96

17.06.2009	113	19	106	123	33	137	62,72
17.06.2009	113	19	106	123	33	137	127,17
17.06.2009	113	19	106	123	33	137	144,65
17.06.2009	113	19	106	123	33	137	945,70
18.06.2009	113	19	106	123	33	137	92,53
18.06.2009	113	19	106	123	33	137	32,60
18.06.2009	113	19	106	123	33	137	122,80
18.06.2009	113	19	106	123	33	137	128,50
18.06.2009	113	19	106	123	33	137	65,19
18.06.2009	113	19	106	123	33	137	136,79
18.06.2009	113	19	106	123	33	137	66,49
19.06.2009	113	19	106	123	33	137	118,46
19.06.2009	113	19	106	123	33	137	46,85
19.06.2009	113	19	106	123	33	137	23,33
10.06.2009	113	19	106	123	33	137	98,35
10.06.2009	113	19	106	123	33	137	57,72
10.06.2009	113	19	106	123	33	137	61,85
10.06.2009	113	19	106	123	33	137	85,83
10.06.2009	113	19	106	123	33	137	84,83
10.06.2009	113	19	106	123	33	137	64,65
11.06.2009	113	19	106	123	33	137	17,65
11.06.2009	113	19	106	123	33	137	15,29
11.06.2009	113	19	106	123	33	137	65,02
11.06.2009	113	19	106	123	33	137	39,86
11.06.2009	113	19	106	123	33	137	138,44
11.06.2009	113	19	106	123	33	137	141,49
11.06.2009	113	19	106	123	33	137	81,43
11.06.2009	113	19	106	123	33	137	119,38
12.06.2009	113	19	106	123	33	137	131,96
12.06.2009	113	19	106	123	33	137	98,50
12.06.2009	113	19	106	123	33	137	120,54
03.06.2009	113	19	106	123	33	137	86,72
03.06.2009	113	19	106	123	33	137	31,30

03.06.2009	113	19	106	123	33	137	69,02
03.06.2009	113	19	106	123	33	137	98,81
03.06.2009	113	19	106	123	33	137	161,57
04.06.2009	113	19	106	123	33	137	141,04
04.06.2009	113	19	106	123	33	137	60,15
04.06.2009	113	19	106	123	33	137	107,94
04.06.2009	113	19	106	123	33	137	118,06
04.06.2009	113	19	106	123	33	137	113,44
04.06.2009	113	19	106	123	33	137	150,62
04.06.2009	113	19	106	123	33	137	110,74
04.06.2009	113	19	106	123	33	137	80,89
05.06.2009	113	19	106	123	33	137	102,58
05.06.2009	113	19	106	123	33	137	136,74
05.06.2009	113	19	106	123	33	137	90,35
17.06.2009	114	17	108	123	33	137	320,77
17.06.2009	114	17	108	123	33	137	92,28
17.06.2009	114	17	108	123	33	137	216,70
17.06.2009	114	17	108	123	33	137	129,69
17.06.2009	114	17	108	123	33	137	201,20
17.06.2009	114	17	108	123	33	137	1036,26
18.06.2009	114	17	108	123	33	137	131,75
18.06.2009	114	17	108	123	33	137	63,34
18.06.2009	114	17	108	123	33	137	68,53
18.06.2009	114	17	108	123	33	137	105,77
18.06.2009	114	17	108	123	33	137	142,98
18.06.2009	114	17	108	123	33	137	214,39
18.06.2009	114	17	108	123	33	137	187,80
19.06.2009	114	17	108	123	33	137	146,31
19.06.2009	114	17	108	123	33	137	124,50
19.06.2009	114	17	108	123	33	137	60,81
10.06.2009	114	17	108	123	33	137	288,30
03.06.2009	114	17	108	123	33	137	12,68
03.06.2009	114	17	108	123	33	137	1,25

03.06.2009	114	17	108	123	33	137	72,71
03.06.2009	114	17	108	123	33	137	57,99
03.06.2009	114	17	108	123	33	137	319,61
04.06.2009	114	17	108	123	33	137	330,83
04.06.2009	114	17	108	123	33	137	262,42
04.06.2009	114	17	108	123	33	137	325,13
04.06.2009	114	17	108	123	33	137	271,65
04.06.2009	114	17	108	123	33	137	263,34
04.06.2009	114	17	108	123	33	137	494,92
04.06.2009	114	17	108	123	33	137	496,48
04.06.2009	114	17	108	123	33	137	431,61
05.06.2009	114	17	108	123	33	137	502,67
05.06.2009	114	17	108	123	33	137	228,42
05.06.2009	114	17	108	123	33	137	481,33
24.06.2009	115	14	98	123	33	137	0,00
24.06.2009	115	14	98	123	33	137	0,96
24.06.2009	115	14	98	123	33	137	10,22
24.06.2009	115	14	98	123	33	137	10,92
24.06.2009	115	14	98	123	33	137	11,19
25.06.2009	115	14	98	123	33	137	15,17
25.06.2009	115	14	98	123	33	137	27,58
25.06.2009	115	14	98	123	33	137	21,33
25.06.2009	115	14	98	123	33	137	21,44
25.06.2009	115	14	98	123	33	137	8,71
25.06.2009	115	14	98	123	33	137	43,48
25.06.2009	115	14	98	123	33	137	10,42
26.06.2009	115	14	98	123	33	137	4,71
26.06.2009	115	14	98	123	33	137	9,66
26.06.2009	115	14	98	123	33	137	9,36
17.06.2009	115	14	98	123	33	137	9,52
17.06.2009	115	14	98	123	33	137	61,20
17.06.2009	115	14	98	123	33	137	75,76
17.06.2009	115	14	98	123	33	137	920,04

18.06.2009	115	14	98	123	33	137	2,06
18.06.2009	115	14	98	123	33	137	3,55
18.06.2009	115	14	98	123	33	137	70,67
18.06.2009	115	14	98	123	33	137	74,22
18.06.2009	115	14	98	123	33	137	63,86
18.06.2009	115	14	98	123	33	137	62,30
18.06.2009	115	14	98	123	33	137	67,90
19.06.2009	115	14	98	123	33	137	71,91
19.06.2009	115	14	98	123	33	137	1,14
19.06.2009	115	14	98	123	33	137	0,97
10.06.2009	115	14	98	123	33	137	62,81
10.06.2009	115	14	98	123	33	137	45,25
10.06.2009	115	14	98	123	33	137	40,87
10.06.2009	115	14	98	123	33	137	19,10
10.06.2009	115	14	98	123	33	137	17,09
10.06.2009	115	14	98	123	33	137	39,94
11.06.2009	115	14	98	123	33	137	60,97
11.06.2009	115	14	98	123	33	137	1,26
11.06.2009	115	14	98	123	33	137	6,86
11.06.2009	115	14	98	123	33	137	35,00
11.06.2009	115	14	98	123	33	137	40,31
11.06.2009	115	14	98	123	33	137	74,47
11.06.2009	115	14	98	123	33	137	73,54
11.06.2009	115	14	98	123	33	137	44,23
12.06.2009	115	14	98	123	33	137	47,05
12.06.2009	115	14	98	123	33	137	148,67
12.06.2009	115	14	98	123	33	137	83,91
03.06.2009	115	14	98	123	33	137	6,22
03.06.2009	115	14	98	123	33	137	1,10
03.06.2009	115	14	98	123	33	137	71,08
03.06.2009	115	14	98	123	33	137	64,10
04.06.2009	115	14	98	123	33	137	222,60
04.06.2009	115	14	98	123	33	137	142,92

04.06.2009	115	14	98	123	33	137	190,05
04.06.2009	115	14	98	123	33	137	245,44
04.06.2009	115	14	98	123	33	137	239,82
04.06.2009	115	14	98	123	33	137	219,12
04.06.2009	115	14	98	123	33	137	190,24
04.06.2009	115	14	98	123	33	137	150,06
05.06.2009	115	14	98	123	33	137	173,53
05.06.2009	115	14	98	123	33	137	162,95
05.06.2009	115	14	98	123	33	137	185,38
17.06.2009	116	60	172	123	33	137	12,15
17.06.2009	116	60	172	123	33	137	424,02
17.06.2009	116	60	172	123	33	137	433,52
10.06.2009	116	60	172	123	33	137	57,80
10.06.2009	116	60	172	123	33	137	54,58
10.06.2009	116	60	172	123	33	137	62,33
10.06.2009	116	60	172	123	33	137	15,46
10.06.2009	116	60	172	123	33	137	27,23
10.06.2009	116	60	172	123	33	137	20,51
11.06.2009	116	60	172	123	33	137	60,94
11.06.2009	116	60	172	123	33	137	0,45
11.06.2009	116	60	172	123	33	137	5,08
11.06.2009	116	60	172	123	33	137	57,33
11.06.2009	116	60	172	123	33	137	66,89
11.06.2009	116	60	172	123	33	137	73,56
11.06.2009	116	60	172	123	33	137	66,45
11.06.2009	116	60	172	123	33	137	48,07
12.06.2009	116	60	172	123	33	137	69,85
12.06.2009	116	60	172	123	33	137	108,51
12.06.2009	116	60	172	123	33	137	80,91
03.06.2009	116	60	172	123	33	137	15,94
03.06.2009	116	60	172	123	33	137	3,11
03.06.2009	116	60	172	123	33	137	51,56
03.06.2009	116	60	172	123	33	137	63,21

03.06.2009	116	60	172	123	33	137	233,34
04.06.2009	116	60	172	123	33	137	133,97
04.06.2009	116	60	172	123	33	137	57,91
04.06.2009	116	60	172	123	33	137	105,02
04.06.2009	116	60	172	123	33	137	190,61
04.06.2009	116	60	172	123	33	137	196,62
04.06.2009	116	60	172	123	33	137	168,66
04.06.2009	116	60	172	123	33	137	176,59
04.06.2009	116	60	172	123	33	137	138,20
05.06.2009	116	60	172	123	33	137	183,59
05.06.2009	116	60	172	123	33	137	155,37
05.06.2009	116	60	172	123	33	137	156,12
24.06.2009	117	29	130	123	33	137	0,00
24.06.2009	117	29	130	123	33	137	72,97
24.06.2009	117	29	130	123	33	137	69,76
24.06.2009	117	29	130	123	33	137	78,75
24.06.2009	117	29	130	123	33	137	73,20
25.06.2009	117	29	130	123	33	137	74,02
25.06.2009	117	29	130	123	33	137	6,21
25.06.2009	117	29	130	123	33	137	62,97
25.06.2009	117	29	130	123	33	137	63,73
25.06.2009	117	29	130	123	33	137	29,90
25.06.2009	117	29	130	123	33	137	64,57
26.06.2009	117	29	130	123	33	137	75,80
26.06.2009	117	29	130	123	33	137	72,69
26.06.2009	117	29	130	123	33	137	84,77
17.06.2009	117	29	130	123	33	137	31,08
17.06.2009	117	29	130	123	33	137	64,67
17.06.2009	117	29	130	123	33	137	60,78
17.06.2009	117	29	130	123	33	137	66,06
17.06.2009	117	29	130	123	33	137	75,18
17.06.2009	117	29	130	123	33	137	936,76
18.06.2009	117	29	130	123	33	137	20,45

18.06.2009	117	29	130	123	33	137	18,30
18.06.2009	117	29	130	123	33	137	67,09
18.06.2009	117	29	130	123	33	137	66,87
18.06.2009	117	29	130	123	33	137	64,96
18.06.2009	117	29	130	123	33	137	68,93
18.06.2009	117	29	130	123	33	137	65,84
19.06.2009	117	29	130	123	33	137	70,52
19.06.2009	117	29	130	123	33	137	9,58
19.06.2009	117	29	130	123	33	137	28,36
10.06.2009	117	29	130	123	33	137	62,26
10.06.2009	117	29	130	123	33	137	58,17
10.06.2009	117	29	130	123	33	137	72,12
10.06.2009	117	29	130	123	33	137	6,12
10.06.2009	117	29	130	123	33	137	20,25
10.06.2009	117	29	130	123	33	137	5,26
11.06.2009	117	29	130	123	33	137	43,54
11.06.2009	117	29	130	123	33	137	3,43
11.06.2009	117	29	130	123	33	137	18,43
11.06.2009	117	29	130	123	33	137	40,96
11.06.2009	117	29	130	123	33	137	71,64
11.06.2009	117	29	130	123	33	137	71,27
11.06.2009	117	29	130	123	33	137	74,43
11.06.2009	117	29	130	123	33	137	48,20
12.06.2009	117	29	130	123	33	137	67,20
12.06.2009	117	29	130	123	33	137	109,93
12.06.2009	117	29	130	123	33	137	85,43
03.06.2009	117	29	130	123	33	137	8,52
03.06.2009	117	29	130	123	33	137	12,73
03.06.2009	117	29	130	123	33	137	69,01
03.06.2009	117	29	130	123	33	137	62,14
03.06.2009	117	29	130	123	33	137	234,09
04.06.2009	117	29	130	123	33	137	224,18
04.06.2009	117	29	130	123	33	137	137,97

04.06.2009	117	29	130	123	33	137	184,10
04.06.2009	117	29	130	123	33	137	190,59
04.06.2009	117	29	130	123	33	137	194,80
04.06.2009	117	29	130	123	33	137	165,96
04.06.2009	117	29	130	123	33	137	176,00
04.06.2009	117	29	130	123	33	137	135,93
05.06.2009	117	29	130	123	33	137	182,17
05.06.2009	117	29	130	123	33	137	151,44
05.06.2009	117	29	130	123	33	137	156,09
24.06.2009	118	41	141	123	33	137	0,00
24.06.2009	118	41	141	123	33	137	772,98
24.06.2009	118	41	141	123	33	137	945,09
24.06.2009	118	41	141	123	33	137	907,17
24.06.2009	118	41	141	123	33	137	926,31
25.06.2009	118	41	141	123	33	137	948,68
25.06.2009	118	41	141	123	33	137	945,23
25.06.2009	118	41	141	123	33	137	936,88
25.06.2009	118	41	141	123	33	137	929,27
25.06.2009	118	41	141	123	33	137	957,22
25.06.2009	118	41	141	123	33	137	745,84
25.06.2009	118	41	141	123	33	137	779,14
26.06.2009	118	41	141	123	33	137	773,92
26.06.2009	118	41	141	123	33	137	927,32
26.06.2009	118	41	141	123	33	137	941,28
17.06.2009	118	41	141	123	33	137	935,60
17.06.2009	118	41	141	123	33	137	880,40
17.06.2009	118	41	141	123	33	137	812,63
17.06.2009	118	41	141	123	33	137	825,31
17.06.2009	118	41	141	123	33	137	717,54
17.06.2009	118	41	141	123	33	137	4,64
18.06.2009	118	41	141	123	33	137	772,47
18.06.2009	118	41	141	123	33	137	874,48
18.06.2009	118	41	141	123	33	137	715,76

18.06.2009	118	41	141	123	33	137	718,66
18.06.2009	118	41	141	123	33	137	803,90
18.06.2009	118	41	141	123	33	137	896,14
18.06.2009	118	41	141	123	33	137	903,83
19.06.2009	118	41	141	123	33	137	865,80
19.06.2009	118	41	141	123	33	137	873,39
19.06.2009	118	41	141	123	33	137	840,44
10.06.2009	118	41	141	123	33	137	880,91
10.06.2009	118	41	141	123	33	137	287,38
10.06.2009	118	41	141	123	33	137	833,97
10.06.2009	118	41	141	123	33	137	875,87
10.06.2009	118	41	141	123	33	137	932,25
10.06.2009	118	41	141	123	33	137	937,33
11.06.2009	118	41	141	123	33	137	840,20
11.06.2009	118	41	141	123	33	137	934,22
11.06.2009	118	41	141	123	33	137	814,57
11.06.2009	118	41	141	123	33	137	884,08
11.06.2009	118	41	141	123	33	137	720,68
11.06.2009	118	41	141	123	33	137	819,61
11.06.2009	118	41	141	123	33	137	854,87
11.06.2009	118	41	141	123	33	137	839,40
12.06.2009	118	41	141	123	33	137	903,29
12.06.2009	118	41	141	123	33	137	1049,35
12.06.2009	118	41	141	123	33	137	775,62
03.06.2009	118	41	141	123	33	137	1152,72
04.06.2009	118	41	141	123	33	137	1145,41
04.06.2009	118	41	141	123	33	137	1059,04
04.06.2009	118	41	141	123	33	137	1120,89
04.06.2009	118	41	141	123	33	137	951,88
04.06.2009	118	41	141	123	33	137	982,43
04.06.2009	118	41	141	123	33	137	983,39
04.06.2009	118	41	141	123	33	137	1108,26
04.06.2009	118	41	141	123	33	137	926,45

05.06.2009	118	41	141	123	33	137	1112,85
05.06.2009	118	41	141	123	33	137	993,67
05.06.2009	118	41	141	123	33	137	928,86
24.06.2009	119	88	198	123	33	137	0,00
24.06.2009	119	88	198	123	33	137	120,25
24.06.2009	119	88	198	123	33	137	112,74
24.06.2009	119	88	198	123	33	137	110,78
24.06.2009	119	88	198	123	33	137	115,51
25.06.2009	119	88	198	123	33	137	111,48
25.06.2009	119	88	198	123	33	137	97,61
25.06.2009	119	88	198	123	33	137	115,06
25.06.2009	119	88	198	123	33	137	80,15
25.06.2009	119	88	198	123	33	137	212,93
25.06.2009	119	88	198	123	33	137	89,82
25.06.2009	119	88	198	123	33	137	76,76
26.06.2009	119	88	198	123	33	137	30,38
26.06.2009	119	88	198	123	33	137	9,05
26.06.2009	119	88	198	123	33	137	49,51
17.06.2009	119	88	198	123	33	137	165,98
17.06.2009	119	88	198	123	33	137	180,71
17.06.2009	119	88	198	123	33	137	185,86
17.06.2009	119	88	198	123	33	137	142,60
17.06.2009	119	88	198	123	33	137	206,04
17.06.2009	119	88	198	123	33	137	1060,71
18.06.2009	119	88	198	123	33	137	141,43
18.06.2009	119	88	198	123	33	137	100,89
18.06.2009	119	88	198	123	33	137	167,48
18.06.2009	119	88	198	123	33	137	154,69
18.06.2009	119	88	198	123	33	137	191,83
18.06.2009	119	88	198	123	33	137	182,70
18.06.2009	119	88	198	123	33	137	169,24
19.06.2009	119	88	198	123	33	137	158,88
19.06.2009	119	88	198	123	33	137	122,02

19.06.2009	119	88	198	123	33	137	146,65
10.06.2009	119	88	198	123	33	137	191,92
10.06.2009	119	88	198	123	33	137	196,80
10.06.2009	119	88	198	123	33	137	144,23
10.06.2009	119	88	198	123	33	137	164,73
10.06.2009	119	88	198	123	33	137	155,16
10.06.2009	119	88	198	123	33	137	118,08
11.06.2009	119	88	198	123	33	137	70,87
11.06.2009	119	88	198	123	33	137	80,22
11.06.2009	119	88	198	123	33	137	111,53
11.06.2009	119	88	198	123	33	137	110,46
11.06.2009	119	88	198	123	33	137	209,27
11.06.2009	119	88	198	123	33	137	194,53
11.06.2009	119	88	198	123	33	137	143,86
11.06.2009	119	88	198	123	33	137	134,53
12.06.2009	119	88	198	123	33	137	185,44
12.06.2009	119	88	198	123	33	137	28,20
12.06.2009	119	88	198	123	33	137	150,42
03.06.2009	119	88	198	123	33	137	139,43
03.06.2009	119	88	198	123	33	137	83,71
03.06.2009	119	88	198	123	33	137	157,71
03.06.2009	119	88	198	123	33	137	140,58
03.06.2009	119	88	198	123	33	137	165,14
04.06.2009	119	88	198	123	33	137	135,22
04.06.2009	119	88	198	123	33	137	68,61
04.06.2009	119	88	198	123	33	137	56,24
04.06.2009	119	88	198	123	33	137	161,03
04.06.2009	119	88	198	123	33	137	104,22
04.06.2009	119	88	198	123	33	137	58,10
04.06.2009	119	88	198	123	33	137	92,43
04.06.2009	119	88	198	123	33	137	48,20
05.06.2009	119	88	198	123	33	137	67,68
05.06.2009	119	88	198	123	33	137	64,63

05.06.2009	119	88	198	123	33	137	26,03
17.06.2009	120	24	122	123	33	137	23,14
17.06.2009	120	24	122	123	33	137	63,48
17.06.2009	120	24	122	123	33	137	60,85
17.06.2009	120	24	122	123	33	137	61,77
17.06.2009	120	24	122	123	33	137	72,57
17.06.2009	120	24	122	123	33	137	938,83
18.06.2009	120	24	122	123	33	137	29,48
18.06.2009	120	24	122	123	33	137	25,42
18.06.2009	120	24	122	123	33	137	67,65
18.06.2009	120	24	122	123	33	137	66,72
18.06.2009	120	24	122	123	33	137	64,20
18.06.2009	120	24	122	123	33	137	65,55
19.06.2009	120	24	122	123	33	137	70,03
19.06.2009	120	24	122	123	33	137	31,76
19.06.2009	120	24	122	123	33	137	24,53
10.06.2009	120	24	122	123	33	137	60,97
10.06.2009	120	24	122	123	33	137	51,12
10.06.2009	120	24	122	123	33	137	70,54
10.06.2009	120	24	122	123	33	137	4,18
10.06.2009	120	24	122	123	33	137	2,19
10.06.2009	120	24	122	123	33	137	20,56
11.06.2009	120	24	122	123	33	137	49,97
11.06.2009	120	24	122	123	33	137	1,94
11.06.2009	120	24	122	123	33	137	25,12
11.06.2009	120	24	122	123	33	137	63,47
11.06.2009	120	24	122	123	33	137	71,11
11.06.2009	120	24	122	123	33	137	73,10
11.06.2009	120	24	122	123	33	137	74,45
11.06.2009	120	24	122	123	33	137	48,33
12.06.2009	120	24	122	123	33	137	68,06
12.06.2009	120	24	122	123	33	137	109,15
12.06.2009	120	24	122	123	33	137	86,60

03.06.2009	120	24	122	123	33	137	78,31
03.06.2009	120	24	122	123	33	137	72,34
03.06.2009	120	24	122	123	33	137	73,06
03.06.2009	120	24	122	123	33	137	168,03
04.06.2009	120	24	122	123	33	137	225,78
04.06.2009	120	24	122	123	33	137	130,17
04.06.2009	120	24	122	123	33	137	181,48
04.06.2009	120	24	122	123	33	137	192,16
04.06.2009	120	24	122	123	33	137	187,01
04.06.2009	120	24	122	123	33	137	166,83
04.06.2009	120	24	122	123	33	137	164,85
04.06.2009	120	24	122	123	33	137	138,45
05.06.2009	120	24	122	123	33	137	118,87
05.06.2009	120	24	122	123	33	137	143,60
05.06.2009	120	24	122	123	33	137	145,67
25.06.2009	121	40	136	123	33	137	7,29
17.06.2009	121	40	136	123	33	137	12,53
17.06.2009	121	40	136	123	33	137	52,69
17.06.2009	121	40	136	123	33	137	57,57
17.06.2009	121	40	136	123	33	137	65,40
17.06.2009	121	40	136	123	33	137	75,72
17.06.2009	121	40	136	123	33	137	937,31
18.06.2009	121	40	136	123	33	137	23,51
18.06.2009	121	40	136	123	33	137	12,56
18.06.2009	121	40	136	123	33	137	63,14
18.06.2009	121	40	136	123	33	137	69,51
18.06.2009	121	40	136	123	33	137	70,47
18.06.2009	121	40	136	123	33	137	65,66
18.06.2009	121	40	136	123	33	137	64,39
19.06.2009	121	40	136	123	33	137	69,41
19.06.2009	121	40	136	123	33	137	23,66
19.06.2009	121	40	136	123	33	137	6,60
10.06.2009	121	40	136	123	33	137	152,38

10.06.2009	121	40	136	123	33	137	51,07
10.06.2009	121	40	136	123	33	137	63,10
10.06.2009	121	40	136	123	33	137	16,05
10.06.2009	121	40	136	123	33	137	29,15
10.06.2009	121	40	136	123	33	137	18,82
11.06.2009	121	40	136	123	33	137	46,79
11.06.2009	121	40	136	123	33	137	14,11
11.06.2009	121	40	136	123	33	137	24,53
11.06.2009	121	40	136	123	33	137	59,21
11.06.2009	121	40	136	123	33	137	70,37
11.06.2009	121	40	136	123	33	137	69,87
11.06.2009	121	40	136	123	33	137	70,27
11.06.2009	121	40	136	123	33	137	47,14
12.06.2009	121	40	136	123	33	137	68,63
12.06.2009	121	40	136	123	33	137	104,56
12.06.2009	121	40	136	123	33	137	84,07
03.06.2009	121	40	136	123	33	137	20,56
03.06.2009	121	40	136	123	33	137	1,40
03.06.2009	121	40	136	123	33	137	67,83
03.06.2009	121	40	136	123	33	137	52,87
03.06.2009	121	40	136	123	33	137	260,71
04.06.2009	121	40	136	123	33	137	155,41
04.06.2009	121	40	136	123	33	137	135,40
04.06.2009	121	40	136	123	33	137	191,80
04.06.2009	121	40	136	123	33	137	192,47
04.06.2009	121	40	136	123	33	137	167,87
04.06.2009	121	40	136	123	33	137	175,35
04.06.2009	121	40	136	123	33	137	135,62
05.06.2009	121	40	136	123	33	137	190,28
05.06.2009	121	40	136	123	33	137	155,58
05.06.2009	121	40	136	123	33	137	155,70
24.06.2009	122	34	129	123	33	137	0,00
24.06.2009	122	34	129	123	33	137	12,70

24.06.2009	122	34	129	123	33	137	4,80
24.06.2009	122	34	129	123	33	137	11,01
24.06.2009	122	34	129	123	33	137	1,57
25.06.2009	122	34	129	123	33	137	2,07
25.06.2009	122	34	129	123	33	137	4,66
25.06.2009	122	34	129	123	33	137	14,09
25.06.2009	122	34	129	123	33	137	4,55
25.06.2009	122	34	129	123	33	137	22,48
25.06.2009	122	34	129	123	33	137	43,86
25.06.2009	122	34	129	123	33	137	9,79
26.06.2009	122	34	129	123	33	137	7,75
26.06.2009	122	34	129	123	33	137	4,41
26.06.2009	122	34	129	123	33	137	6,17
17.06.2009	122	34	129	123	33	137	11,52
17.06.2009	122	34	129	123	33	137	62,44
17.06.2009	122	34	129	123	33	137	61,60
17.06.2009	122	34	129	123	33	137	61,60
17.06.2009	122	34	129	123	33	137	74,54
17.06.2009	122	34	129	123	33	137	945,41
18.06.2009	122	34	129	123	33	137	25,74
18.06.2009	122	34	129	123	33	137	5,56
18.06.2009	122	34	129	123	33	137	60,94
18.06.2009	122	34	129	123	33	137	68,41
18.06.2009	122	34	129	123	33	137	66,22
18.06.2009	122	34	129	123	33	137	65,97
18.06.2009	122	34	129	123	33	137	69,46
19.06.2009	122	34	129	123	33	137	69,08
19.06.2009	122	34	129	123	33	137	16,26
19.06.2009	122	34	129	123	33	137	12,32
10.06.2009	122	34	129	123	33	137	60,46
10.06.2009	122	34	129	123	33	137	56,95
10.06.2009	122	34	129	123	33	137	12,71
10.06.2009	122	34	129	123	33	137	2,58

10.06.2009	122	34	129	123	33	137	10,92
11.06.2009	122	34	129	123	33	137	59,88
11.06.2009	122	34	129	123	33	137	14,25
11.06.2009	122	34	129	123	33	137	24,25
11.06.2009	122	34	129	123	33	137	40,01
11.06.2009	122	34	129	123	33	137	74,37
11.06.2009	122	34	129	123	33	137	73,10
11.06.2009	122	34	129	123	33	137	67,97
12.06.2009	122	34	129	123	33	137	67,99
12.06.2009	122	34	129	123	33	137	105,38
12.06.2009	122	34	129	123	33	137	77,91
03.06.2009	122	34	129	123	33	137	20,71
03.06.2009	122	34	129	123	33	137	10,19
03.06.2009	122	34	129	123	33	137	72,31
03.06.2009	122	34	129	123	33	137	35,13
03.06.2009	122	34	129	123	33	137	233,11
04.06.2009	122	34	129	123	33	137	227,58
04.06.2009	122	34	129	123	33	137	138,53
04.06.2009	122	34	129	123	33	137	194,71
04.06.2009	122	34	129	123	33	137	182,52
04.06.2009	122	34	129	123	33	137	187,73
04.06.2009	122	34	129	123	33	137	167,80
04.06.2009	122	34	129	123	33	137	221,79
04.06.2009	122	34	129	123	33	137	135,73
05.06.2009	122	34	129	123	33	137	187,00
05.06.2009	122	34	129	123	33	137	155,25
05.06.2009	122	34	129	123	33	137	146,85
25.06.2009	106	25	122	124	26	120	63,48
25.06.2009	106	25	122	124	26	120	88,54
25.06.2009	106	25	122	124	26	120	13,25
25.06.2009	106	25	122	124	26	120	42,85
25.06.2009	106	25	122	124	26	120	45,62
26.06.2009	106	25	122	124	26	120	38,29

26.06.2009	106	25	122	124	26	120	82,53
26.06.2009	106	25	122	124	26	120	70,13
17.06.2009	106	25	122	124	26	120	948,44
10.06.2009	106	25	122	124	26	120	941,71
10.06.2009	106	25	122	124	26	120	939,92
10.06.2009	106	25	122	124	26	120	946,70
11.06.2009	106	25	122	124	26	120	940,96
11.06.2009	106	25	122	124	26	120	951,43
11.06.2009	106	25	122	124	26	120	926,84
11.06.2009	106	25	122	124	26	120	953,77
11.06.2009	106	25	122	124	26	120	965,14
11.06.2009	106	25	122	124	26	120	950,76
11.06.2009	106	25	122	124	26	120	946,92
11.06.2009	106	25	122	124	26	120	950,50
12.06.2009	106	25	122	124	26	120	929,66
12.06.2009	106	25	122	124	26	120	959,50
12.06.2009	106	25	122	124	26	120	947,55
03.06.2009	106	25	122	124	26	120	864,82
03.06.2009	106	25	122	124	26	120	934,15
04.06.2009	106	25	122	124	26	120	929,52
04.06.2009	106	25	122	124	26	120	937,67
04.06.2009	106	25	122	124	26	120	918,25
04.06.2009	106	25	122	124	26	120	891,31
04.06.2009	106	25	122	124	26	120	772,94
04.06.2009	106	25	122	124	26	120	932,12
04.06.2009	106	25	122	124	26	120	934,86
04.06.2009	106	25	122	124	26	120	954,93
05.06.2009	106	25	122	124	26	120	933,40
05.06.2009	106	25	122	124	26	120	931,22
05.06.2009	106	25	122	124	26	120	931,80
24.06.2009	112	17	111	124	26	120	0,00
24.06.2009	112	17	111	124	26	120	6,57
24.06.2009	112	17	111	124	26	120	11,85

24.06.2009	112	17	111	124	26	120	11,57
24.06.2009	112	17	111	124	26	120	7,01
24.06.2009	112	17	111	124	26	120	21,63
25.06.2009	112	17	111	124	26	120	19,92
25.06.2009	112	17	111	124	26	120	59,09
25.06.2009	112	17	111	124	26	120	23,52
25.06.2009	112	17	111	124	26	120	21,10
25.06.2009	112	17	111	124	26	120	25,45
25.06.2009	112	17	111	124	26	120	9,63
25.06.2009	112	17	111	124	26	120	24,26
25.06.2009	112	17	111	124	26	120	35,27
26.06.2009	112	17	111	124	26	120	33,72
26.06.2009	112	17	111	124	26	120	19,15
26.06.2009	112	17	111	124	26	120	24,04
17.06.2009	112	17	111	124	26	120	54,62
17.06.2009	112	17	111	124	26	120	51,60
17.06.2009	112	17	111	124	26	120	49,99
17.06.2009	112	17	111	124	26	120	29,10
17.06.2009	112	17	111	124	26	120	36,83
17.06.2009	112	17	111	124	26	120	26,87
18.06.2009	112	17	111	124	26	120	58,54
18.06.2009	112	17	111	124	26	120	37,73
18.06.2009	112	17	111	124	26	120	35,40
18.06.2009	112	17	111	124	26	120	7,30
18.06.2009	112	17	111	124	26	120	10,94
18.06.2009	112	17	111	124	26	120	56,86
18.06.2009	112	17	111	124	26	120	58,13
18.06.2009	112	17	111	124	26	120	55,93
19.06.2009	112	17	111	124	26	120	18,74
19.06.2009	112	17	111	124	26	120	27,57
10.06.2009	112	17	111	124	26	120	66,96
10.06.2009	112	17	111	124	26	120	17,16
10.06.2009	112	17	111	124	26	120	14,60

11.06.2009	112	17	111	124	26	120	20,83
11.06.2009	112	17	111	124	26	120	71,09
11.06.2009	112	17	111	124	26	120	36,08
11.06.2009	112	17	111	124	26	120	61,22
11.06.2009	112	17	111	124	26	120	27,27
11.06.2009	112	17	111	124	26	120	56,39
11.06.2009	112	17	111	124	26	120	22,28
12.06.2009	112	17	111	124	26	120	15,35
12.06.2009	112	17	111	124	26	120	78,60
12.06.2009	112	17	111	124	26	120	76,57
03.06.2009	112	17	111	124	26	120	54,96
03.06.2009	112	17	111	124	26	120	53,19
03.06.2009	112	17	111	124	26	120	61,82
03.06.2009	112	17	111	124	26	120	60,56
03.06.2009	112	17	111	124	26	120	58,84
03.06.2009	112	17	111	124	26	120	64,52
04.06.2009	112	17	111	124	26	120	50,06
04.06.2009	112	17	111	124	26	120	58,27
04.06.2009	112	17	111	124	26	120	56,40
04.06.2009	112	17	111	124	26	120	34,14
04.06.2009	112	17	111	124	26	120	81,57
04.06.2009	112	17	111	124	26	120	77,27
04.06.2009	112	17	111	124	26	120	143,46
04.06.2009	112	17	111	124	26	120	110,40
05.06.2009	112	17	111	124	26	120	65,78
05.06.2009	112	17	111	124	26	120	110,52
05.06.2009	112	17	111	124	26	120	63,73
24.06.2009	113	19	106	124	26	120	0,00
24.06.2009	113	19	106	124	26	120	75,96
24.06.2009	113	19	106	124	26	120	77,53
24.06.2009	113	19	106	124	26	120	73,67
24.06.2009	113	19	106	124	26	120	85,95
24.06.2009	113	19	106	124	26	120	78,13

25.06.2009	113	19	106	124	26	120	69,00
25.06.2009	113	19	106	124	26	120	67,25
25.06.2009	113	19	106	124	26	120	78,18
25.06.2009	113	19	106	124	26	120	73,59
25.06.2009	113	19	106	124	26	120	71,55
25.06.2009	113	19	106	124	26	120	79,34
25.06.2009	113	19	106	124	26	120	67,29
25.06.2009	113	19	106	124	26	120	73,91
26.06.2009	113	19	106	124	26	120	66,77
26.06.2009	113	19	106	124	26	120	75,17
26.06.2009	113	19	106	124	26	120	81,58
17.06.2009	113	19	106	124	26	120	82,88
17.06.2009	113	19	106	124	26	120	82,85
17.06.2009	113	19	106	124	26	120	30,64
17.06.2009	113	19	106	124	26	120	83,17
17.06.2009	113	19	106	124	26	120	80,03
17.06.2009	113	19	106	124	26	120	11,28
18.06.2009	113	19	106	124	26	120	18,58
18.06.2009	113	19	106	124	26	120	70,30
18.06.2009	113	19	106	124	26	120	20,03
18.06.2009	113	19	106	124	26	120	77,46
18.06.2009	113	19	106	124	26	120	77,96
18.06.2009	113	19	106	124	26	120	18,96
18.06.2009	113	19	106	124	26	120	74,04
18.06.2009	113	19	106	124	26	120	8,96
19.06.2009	113	19	106	124	26	120	66,56
19.06.2009	113	19	106	124	26	120	2,00
10.06.2009	113	19	106	124	26	120	70,63
10.06.2009	113	19	106	124	26	120	83,49
10.06.2009	113	19	106	124	26	120	71,87
11.06.2009	113	19	106	124	26	120	28,68
11.06.2009	113	19	106	124	26	120	14,28
11.06.2009	113	19	106	124	26	120	87,34

11.06.2009	113	19	106	124	26	120	3,35
11.06.2009	113	19	106	124	26	120	64,01
11.06.2009	113	19	106	124	26	120	66,72
11.06.2009	113	19	106	124	26	120	43,88
11.06.2009	113	19	106	124	26	120	70,96
12.06.2009	113	19	106	124	26	120	67,21
12.06.2009	113	19	106	124	26	120	2,08
12.06.2009	113	19	106	124	26	120	60,51
03.06.2009	113	19	106	124	26	120	69,75
03.06.2009	113	19	106	124	26	120	16,50
03.06.2009	113	19	106	124	26	120	32,45
03.06.2009	113	19	106	124	26	120	70,21
03.06.2009	113	19	106	124	26	120	65,94
03.06.2009	113	19	106	124	26	120	73,92
04.06.2009	113	19	106	124	26	120	87,19
04.06.2009	113	19	106	124	26	120	74,57
04.06.2009	113	19	106	124	26	120	68,84
04.06.2009	113	19	106	124	26	120	88,06
04.06.2009	113	19	106	124	26	120	89,98
04.06.2009	113	19	106	124	26	120	56,19
04.06.2009	113	19	106	124	26	120	85,24
04.06.2009	113	19	106	124	26	120	80,50
05.06.2009	113	19	106	124	26	120	72,54
05.06.2009	113	19	106	124	26	120	43,07
05.06.2009	113	19	106	124	26	120	74,51
17.06.2009	114	17	108	124	26	120	334,64
17.06.2009	114	17	108	124	26	120	87,34
17.06.2009	114	17	108	124	26	120	168,54
17.06.2009	114	17	108	124	26	120	82,65
17.06.2009	114	17	108	124	26	120	132,56
17.06.2009	114	17	108	124	26	120	98,77
18.06.2009	114	17	108	124	26	120	125,95
18.06.2009	114	17	108	124	26	120	112,36

18.06.2009	114	17	108	124	26	120	57,16
18.06.2009	114	17	108	124	26	120	76,33
18.06.2009	114	17	108	124	26	120	82,03
18.06.2009	114	17	108	124	26	120	81,84
18.06.2009	114	17	108	124	26	120	150,93
18.06.2009	114	17	108	124	26	120	127,32
19.06.2009	114	17	108	124	26	120	78,87
19.06.2009	114	17	108	124	26	120	39,92
03.06.2009	114	17	108	124	26	120	9,91
03.06.2009	114	17	108	124	26	120	16,86
03.06.2009	114	17	108	124	26	120	6,31
03.06.2009	114	17	108	124	26	120	6,68
03.06.2009	114	17	108	124	26	120	138,08
03.06.2009	114	17	108	124	26	120	118,69
04.06.2009	114	17	108	124	26	120	126,68
04.06.2009	114	17	108	124	26	120	150,61
04.06.2009	114	17	108	124	26	120	149,73
04.06.2009	114	17	108	124	26	120	93,97
04.06.2009	114	17	108	124	26	120	86,37
04.06.2009	114	17	108	124	26	120	325,42
04.06.2009	114	17	108	124	26	120	321,65
04.06.2009	114	17	108	124	26	120	294,94
05.06.2009	114	17	108	124	26	120	329,10
05.06.2009	114	17	108	124	26	120	90,99
05.06.2009	114	17	108	124	26	120	335,16
24.06.2009	115	14	98	124	26	120	0,00
24.06.2009	115	14	98	124	26	120	1,84
24.06.2009	115	14	98	124	26	120	8,54
24.06.2009	115	14	98	124	26	120	7,90
24.06.2009	115	14	98	124	26	120	16,87
24.06.2009	115	14	98	124	26	120	15,45
25.06.2009	115	14	98	124	26	120	22,57
25.06.2009	115	14	98	124	26	120	9,43

25.06.2009	115	14	98	124	26	120	2,42
25.06.2009	115	14	98	124	26	120	19,94
25.06.2009	115	14	98	124	26	120	6,59
25.06.2009	115	14	98	124	26	120	13,48
25.06.2009	115	14	98	124	26	120	12,08
25.06.2009	115	14	98	124	26	120	13,44
26.06.2009	115	14	98	124	26	120	10,94
26.06.2009	115	14	98	124	26	120	5,80
26.06.2009	115	14	98	124	26	120	5,98
17.06.2009	115	14	98	124	26	120	11,62
17.06.2009	115	14	98	124	26	120	9,88
17.06.2009	115	14	98	124	26	120	6,78
17.06.2009	115	14	98	124	26	120	21,49
18.06.2009	115	14	98	124	26	120	0,47
18.06.2009	115	14	98	124	26	120	26,34
18.06.2009	115	14	98	124	26	120	14,76
18.06.2009	115	14	98	124	26	120	2,12
18.06.2009	115	14	98	124	26	120	1,28
18.06.2009	115	14	98	124	26	120	11,38
18.06.2009	115	14	98	124	26	120	18,28
18.06.2009	115	14	98	124	26	120	9,75
19.06.2009	115	14	98	124	26	120	10,89
19.06.2009	115	14	98	124	26	120	24,54
10.06.2009	115	14	98	124	26	120	5,91
10.06.2009	115	14	98	124	26	120	15,69
10.06.2009	115	14	98	124	26	120	37,18
11.06.2009	115	14	98	124	26	120	17,16
11.06.2009	115	14	98	124	26	120	1,93
11.06.2009	115	14	98	124	26	120	24,59
11.06.2009	115	14	98	124	26	120	8,04
11.06.2009	115	14	98	124	26	120	41,49
11.06.2009	115	14	98	124	26	120	14,35
11.06.2009	115	14	98	124	26	120	20,48

11.06.2009	115	14	98	124	26	120	22,74
12.06.2009	115	14	98	124	26	120	31,58
12.06.2009	115	14	98	124	26	120	51,07
12.06.2009	115	14	98	124	26	120	14,37
03.06.2009	115	14	98	124	26	120	19,88
03.06.2009	115	14	98	124	26	120	16,70
03.06.2009	115	14	98	124	26	120	10,58
03.06.2009	115	14	98	124	26	120	11,92
03.06.2009	115	14	98	124	26	120	5,39
04.06.2009	115	14	98	124	26	120	0,54
04.06.2009	115	14	98	124	26	120	18,29
04.06.2009	115	14	98	124	26	120	14,31
04.06.2009	115	14	98	124	26	120	64,66
04.06.2009	115	14	98	124	26	120	58,98
04.06.2009	115	14	98	124	26	120	60,40
04.06.2009	115	14	98	124	26	120	15,62
04.06.2009	115	14	98	124	26	120	13,29
05.06.2009	115	14	98	124	26	120	6,70
05.06.2009	115	14	98	124	26	120	15,26
05.06.2009	115	14	98	124	26	120	39,13
17.06.2009	116	60	172	124	26	120	12,36
17.06.2009	116	60	172	124	26	120	406,65
17.06.2009	116	60	172	124	26	120	415,60
10.06.2009	116	60	172	124	26	120	2,15
10.06.2009	116	60	172	124	26	120	27,93
10.06.2009	116	60	172	124	26	120	11,24
11.06.2009	116	60	172	124	26	120	17,08
11.06.2009	116	60	172	124	26	120	0,86
11.06.2009	116	60	172	124	26	120	26,31
11.06.2009	116	60	172	124	26	120	14,48
11.06.2009	116	60	172	124	26	120	34,74
11.06.2009	116	60	172	124	26	120	5,85
11.06.2009	116	60	172	124	26	120	6,83

11.06.2009	116	60	172	124	26	120	2,56
12.06.2009	116	60	172	124	26	120	22,17
12.06.2009	116	60	172	124	26	120	15,38
12.06.2009	116	60	172	124	26	120	17,86
03.06.2009	116	60	172	124	26	120	4,45
03.06.2009	116	60	172	124	26	120	18,22
03.06.2009	116	60	172	124	26	120	21,72
03.06.2009	116	60	172	124	26	120	10,41
03.06.2009	116	60	172	124	26	120	3,34
03.06.2009	116	60	172	124	26	120	2,98
04.06.2009	116	60	172	124	26	120	91,08
04.06.2009	116	60	172	124	26	120	72,98
04.06.2009	116	60	172	124	26	120	71,39
04.06.2009	116	60	172	124	26	120	8,23
04.06.2009	116	60	172	124	26	120	8,92
04.06.2009	116	60	172	124	26	120	3,27
04.06.2009	116	60	172	124	26	120	1,43
04.06.2009	116	60	172	124	26	120	2,12
05.06.2009	116	60	172	124	26	120	12,27
05.06.2009	116	60	172	124	26	120	8,89
05.06.2009	116	60	172	124	26	120	11,54
24.06.2009	117	29	130	124	26	120	0,00
24.06.2009	117	29	130	124	26	120	73,52
24.06.2009	117	29	130	124	26	120	60,83
24.06.2009	117	29	130	124	26	120	70,34
24.06.2009	117	29	130	124	26	120	75,54
24.06.2009	117	29	130	124	26	120	75,14
25.06.2009	117	29	130	124	26	120	72,60
25.06.2009	117	29	130	124	26	120	13,81
25.06.2009	117	29	130	124	26	120	63,08
25.06.2009	117	29	130	124	26	120	62,76
25.06.2009	117	29	130	124	26	120	70,68
25.06.2009	117	29	130	124	26	120	64,26

25.06.2009	117	29	130	124	26	120	71,67
26.06.2009	117	29	130	124	26	120	74,01
26.06.2009	117	29	130	124	26	120	68,62
26.06.2009	117	29	130	124	26	120	84,61
17.06.2009	117	29	130	124	26	120	17,62
17.06.2009	117	29	130	124	26	120	13,71
17.06.2009	117	29	130	124	26	120	13,38
17.06.2009	117	29	130	124	26	120	18,92
17.06.2009	117	29	130	124	26	120	12,14
17.06.2009	117	29	130	124	26	120	9,94
18.06.2009	117	29	130	124	26	120	7,31
18.06.2009	117	29	130	124	26	120	5,73
18.06.2009	117	29	130	124	26	120	6,99
18.06.2009	117	29	130	124	26	120	7,16
18.06.2009	117	29	130	124	26	120	10,23
18.06.2009	117	29	130	124	26	120	7,97
18.06.2009	117	29	130	124	26	120	12,09
18.06.2009	117	29	130	124	26	120	9,93
19.06.2009	117	29	130	124	26	120	0,52
19.06.2009	117	29	130	124	26	120	5,52
10.06.2009	117	29	130	124	26	120	11,40
10.06.2009	117	29	130	124	26	120	18,83
10.06.2009	117	29	130	124	26	120	5,44
11.06.2009	117	29	130	124	26	120	1,81
11.06.2009	117	29	130	124	26	120	4,43
11.06.2009	117	29	130	124	26	120	13,06
11.06.2009	117	29	130	124	26	120	5,49
11.06.2009	117	29	130	124	26	120	5,32
11.06.2009	117	29	130	124	26	120	20,88
11.06.2009	117	29	130	124	26	120	19,26
11.06.2009	117	29	130	124	26	120	20,45
12.06.2009	117	29	130	124	26	120	9,35
12.06.2009	117	29	130	124	26	120	16,98

12.06.2009	117	29	130	124	26	120	11,95
03.06.2009	117	29	130	124	26	120	19,70
03.06.2009	117	29	130	124	26	120	3,62
03.06.2009	117	29	130	124	26	120	12,13
03.06.2009	117	29	130	124	26	120	8,90
03.06.2009	117	29	130	124	26	120	4,80
03.06.2009	117	29	130	124	26	120	1,00
04.06.2009	117	29	130	124	26	120	5,33
04.06.2009	117	29	130	124	26	120	12,77
04.06.2009	117	29	130	124	26	120	8,71
04.06.2009	117	29	130	124	26	120	5,13
04.06.2009	117	29	130	124	26	120	7,17
04.06.2009	117	29	130	124	26	120	4,52
04.06.2009	117	29	130	124	26	120	0,99
04.06.2009	117	29	130	124	26	120	0,91
05.06.2009	117	29	130	124	26	120	10,22
05.06.2009	117	29	130	124	26	120	7,11
05.06.2009	117	29	130	124	26	120	11,65
24.06.2009	118	41	141	124	26	120	0,00
24.06.2009	118	41	141	124	26	120	774,91
24.06.2009	118	41	141	124	26	120	766,71
24.06.2009	118	41	141	124	26	120	944,01
24.06.2009	118	41	141	124	26	120	900,55
24.06.2009	118	41	141	124	26	120	930,82
25.06.2009	118	41	141	124	26	120	952,30
25.06.2009	118	41	141	124	26	120	954,85
25.06.2009	118	41	141	124	26	120	948,16
25.06.2009	118	41	141	124	26	120	928,53
25.06.2009	118	41	141	124	26	120	955,20
25.06.2009	118	41	141	124	26	120	787,95
25.06.2009	118	41	141	124	26	120	779,65
25.06.2009	118	41	141	124	26	120	875,81
26.06.2009	118	41	141	124	26	120	775,99

26.06.2009	118	41	141	124	26	120	926,57
26.06.2009	118	41	141	124	26	120	943,47
17.06.2009	118	41	141	124	26	120	949,63
17.06.2009	118	41	141	124	26	120	922,45
17.06.2009	118	41	141	124	26	120	850,24
17.06.2009	118	41	141	124	26	120	861,95
17.06.2009	118	41	141	124	26	120	777,90
17.06.2009	118	41	141	124	26	120	944,59
18.06.2009	118	41	141	124	26	120	765,94
18.06.2009	118	41	141	124	26	120	792,51
18.06.2009	118	41	141	124	26	120	887,67
18.06.2009	118	41	141	124	26	120	757,34
18.06.2009	118	41	141	124	26	120	768,97
18.06.2009	118	41	141	124	26	120	857,92
18.06.2009	118	41	141	124	26	120	948,35
18.06.2009	118	41	141	124	26	120	948,07
19.06.2009	118	41	141	124	26	120	922,86
19.06.2009	118	41	141	124	26	120	855,90
10.06.2009	118	41	141	124	26	120	891,18
10.06.2009	118	41	141	124	26	120	933,60
10.06.2009	118	41	141	124	26	120	929,91
11.06.2009	118	41	141	124	26	120	811,18
11.06.2009	118	41	141	124	26	120	934,74
11.06.2009	118	41	141	124	26	120	795,93
11.06.2009	118	41	141	124	26	120	860,73
11.06.2009	118	41	141	124	26	120	791,83
11.06.2009	118	41	141	124	26	120	883,46
11.06.2009	118	41	141	124	26	120	895,67
11.06.2009	118	41	141	124	26	120	883,43
12.06.2009	118	41	141	124	26	120	958,91
12.06.2009	118	41	141	124	26	120	950,31
12.06.2009	118	41	141	124	26	120	832,66
03.06.2009	118	41	141	124	26	120	881,39

03.06.2009	118	41	141	124	26	120	933,46
04.06.2009	118	41	141	124	26	120	926,96
04.06.2009	118	41	141	124	26	120	939,52
04.06.2009	118	41	141	124	26	120	946,77
04.06.2009	118	41	141	124	26	120	760,36
04.06.2009	118	41	141	124	26	120	795,22
04.06.2009	118	41	141	124	26	120	814,45
04.06.2009	118	41	141	124	26	120	935,64
04.06.2009	118	41	141	124	26	120	789,93
05.06.2009	118	41	141	124	26	120	942,17
05.06.2009	118	41	141	124	26	120	842,08
05.06.2009	118	41	141	124	26	120	781,89
24.06.2009	119	88	198	124	26	120	0,00
24.06.2009	119	88	198	124	26	120	118,33
24.06.2009	119	88	198	124	26	120	119,29
24.06.2009	119	88	198	124	26	120	113,31
24.06.2009	119	88	198	124	26	120	117,28
24.06.2009	119	88	198	124	26	120	111,34
25.06.2009	119	88	198	124	26	120	109,80
25.06.2009	119	88	198	124	26	120	91,48
25.06.2009	119	88	198	124	26	120	107,88
25.06.2009	119	88	198	124	26	120	80,36
25.06.2009	119	88	198	124	26	120	214,86
25.06.2009	119	88	198	124	26	120	68,61
25.06.2009	119	88	198	124	26	120	75,47
25.06.2009	119	88	198	124	26	120	3,25
26.06.2009	119	88	198	124	26	120	22,42
26.06.2009	119	88	198	124	26	120	4,45
26.06.2009	119	88	198	124	26	120	45,87
17.06.2009	119	88	198	124	26	120	154,30
17.06.2009	119	88	198	124	26	120	124,43
17.06.2009	119	88	198	124	26	120	137,85
17.06.2009	119	88	198	124	26	120	93,44

17.06.2009	119	88	198	124	26	120	137,59
17.06.2009	119	88	198	124	26	120	124,65
18.06.2009	119	88	198	124	26	120	117,16
18.06.2009	119	88	198	124	26	120	120,83
18.06.2009	119	88	198	124	26	120	88,49
18.06.2009	119	88	198	124	26	120	115,50
18.06.2009	119	88	198	124	26	120	100,74
18.06.2009	119	88	198	124	26	120	129,26
18.06.2009	119	88	198	124	26	120	119,49
18.06.2009	119	88	198	124	26	120	110,53
19.06.2009	119	88	198	124	26	120	91,04
19.06.2009	119	88	198	124	26	120	136,15
10.06.2009	119	88	198	124	26	120	149,27
10.06.2009	119	88	198	124	26	120	153,94
10.06.2009	119	88	198	124	26	120	123,84
11.06.2009	119	88	198	124	26	120	80,77
11.06.2009	119	88	198	124	26	120	79,96
11.06.2009	119	88	198	124	26	120	130,28
11.06.2009	119	88	198	124	26	120	116,74
11.06.2009	119	88	198	124	26	120	134,68
11.06.2009	119	88	198	124	26	120	121,44
11.06.2009	119	88	198	124	26	120	81,95
11.06.2009	119	88	198	124	26	120	85,89
12.06.2009	119	88	198	124	26	120	120,33
12.06.2009	119	88	198	124	26	120	85,66
12.06.2009	119	88	198	124	26	120	77,59
03.06.2009	119	88	198	124	26	120	123,29
03.06.2009	119	88	198	124	26	120	72,41
03.06.2009	119	88	198	124	26	120	94,98
03.06.2009	119	88	198	124	26	120	86,78
03.06.2009	119	88	198	124	26	120	89,19
03.06.2009	119	88	198	124	26	120	82,09
04.06.2009	119	88	198	124	26	120	102,05

04.06.2009	119	88	198	124	26	120	99,97
04.06.2009	119	88	198	124	26	120	120,86
04.06.2009	119	88	198	124	26	120	39,63
04.06.2009	119	88	198	124	26	120	83,75
04.06.2009	119	88	198	124	26	120	111,75
04.06.2009	119	88	198	124	26	120	84,31
04.06.2009	119	88	198	124	26	120	89,03
05.06.2009	119	88	198	124	26	120	106,24
05.06.2009	119	88	198	124	26	120	89,00
05.06.2009	119	88	198	124	26	120	121,31
17.06.2009	120	24	122	124	26	120	33,30
17.06.2009	120	24	122	124	26	120	22,34
17.06.2009	120	24	122	124	26	120	6,20
17.06.2009	120	24	122	124	26	120	17,95
17.06.2009	120	24	122	124	26	120	29,14
17.06.2009	120	24	122	124	26	120	4,83
18.06.2009	120	24	122	124	26	120	13,36
18.06.2009	120	24	122	124	26	120	9,75
18.06.2009	120	24	122	124	26	120	12,89
18.06.2009	120	24	122	124	26	120	15,38
18.06.2009	120	24	122	124	26	120	9,92
18.06.2009	120	24	122	124	26	120	19,56
18.06.2009	120	24	122	124	26	120	2,67
19.06.2009	120	24	122	124	26	120	0,16
19.06.2009	120	24	122	124	26	120	1,18
10.06.2009	120	24	122	124	26	120	12,25
10.06.2009	120	24	122	124	26	120	0,75
10.06.2009	120	24	122	124	26	120	11,31
11.06.2009	120	24	122	124	26	120	6,17
11.06.2009	120	24	122	124	26	120	2,75
11.06.2009	120	24	122	124	26	120	8,89
11.06.2009	120	24	122	124	26	120	27,66
11.06.2009	120	24	122	124	26	120	17,10

11.06.2009	120	24	122	124	26	120	5,89
11.06.2009	120	24	122	124	26	120	10,48
11.06.2009	120	24	122	124	26	120	9,02
12.06.2009	120	24	122	124	26	120	4,70
12.06.2009	120	24	122	124	26	120	13,53
12.06.2009	120	24	122	124	26	120	10,14
03.06.2009	120	24	122	124	26	120	59,26
03.06.2009	120	24	122	124	26	120	4,96
03.06.2009	120	24	122	124	26	120	37,05
03.06.2009	120	24	122	124	26	120	5,73
03.06.2009	120	24	122	124	26	120	69,13
04.06.2009	120	24	122	124	26	120	3,37
04.06.2009	120	24	122	124	26	120	5,33
04.06.2009	120	24	122	124	26	120	5,49
04.06.2009	120	24	122	124	26	120	6,94
04.06.2009	120	24	122	124	26	120	1,50
04.06.2009	120	24	122	124	26	120	4,88
04.06.2009	120	24	122	124	26	120	17,94
04.06.2009	120	24	122	124	26	120	2,21
05.06.2009	120	24	122	124	26	120	70,54
05.06.2009	120	24	122	124	26	120	13,03
05.06.2009	120	24	122	124	26	120	2,62
25.06.2009	121	40	136	124	26	120	6,07
17.06.2009	121	40	136	124	26	120	3,70
17.06.2009	121	40	136	124	26	120	11,09
17.06.2009	121	40	136	124	26	120	2,49
17.06.2009	121	40	136	124	26	120	22,95
17.06.2009	121	40	136	124	26	120	10,43
17.06.2009	121	40	136	124	26	120	18,27
18.06.2009	121	40	136	124	26	120	8,55
18.06.2009	121	40	136	124	26	120	6,59
18.06.2009	121	40	136	124	26	120	1,98
18.06.2009	121	40	136	124	26	120	21,03

18.06.2009	121	40	136	124	26	120	9,90
18.06.2009	121	40	136	124	26	120	3,71
18.06.2009	121	40	136	124	26	120	2,72
18.06.2009	121	40	136	124	26	120	13,79
19.06.2009	121	40	136	124	26	120	2,13
19.06.2009	121	40	136	124	26	120	20,11
10.06.2009	121	40	136	124	26	120	2,95
10.06.2009	121	40	136	124	26	120	27,70
10.06.2009	121	40	136	124	26	120	9,83
11.06.2009	121	40	136	124	26	120	3,08
11.06.2009	121	40	136	124	26	120	15,11
11.06.2009	121	40	136	124	26	120	8,75
11.06.2009	121	40	136	124	26	120	16,61
11.06.2009	121	40	136	124	26	120	21,82
11.06.2009	121	40	136	124	26	120	22,94
11.06.2009	121	40	136	124	26	120	1,30
11.06.2009	121	40	136	124	26	120	6,37
12.06.2009	121	40	136	124	26	120	19,29
12.06.2009	121	40	136	124	26	120	9,22
12.06.2009	121	40	136	124	26	120	5,65
03.06.2009	121	40	136	124	26	120	1,30
03.06.2009	121	40	136	124	26	120	17,03
03.06.2009	121	40	136	124	26	120	10,35
03.06.2009	121	40	136	124	26	120	17,07
03.06.2009	121	40	136	124	26	120	6,98
03.06.2009	121	40	136	124	26	120	53,39
04.06.2009	121	40	136	124	26	120	30,92
04.06.2009	121	40	136	124	26	120	64,15
04.06.2009	121	40	136	124	26	120	2,17
04.06.2009	121	40	136	124	26	120	5,12
04.06.2009	121	40	136	124	26	120	2,47
04.06.2009	121	40	136	124	26	120	0,33
04.06.2009	121	40	136	124	26	120	1,22

05.06.2009	121	40	136	124	26	120	17,56
05.06.2009	121	40	136	124	26	120	8,20
05.06.2009	121	40	136	124	26	120	12,12
24.06.2009	122	34	129	124	26	120	0,00
24.06.2009	122	34	129	124	26	120	10,24
24.06.2009	122	34	129	124	26	120	0,84
24.06.2009	122	34	129	124	26	120	7,11
24.06.2009	122	34	129	124	26	120	17,42
24.06.2009	122	34	129	124	26	120	6,47
25.06.2009	122	34	129	124	26	120	7,13
25.06.2009	122	34	129	124	26	120	16,12
25.06.2009	122	34	129	124	26	120	8,12
25.06.2009	122	34	129	124	26	120	3,24
25.06.2009	122	34	129	124	26	120	20,31
25.06.2009	122	34	129	124	26	120	1,05
25.06.2009	122	34	129	124	26	120	9,40
25.06.2009	122	34	129	124	26	120	5,25
26.06.2009	122	34	129	124	26	120	0,60
26.06.2009	122	34	129	124	26	120	9,79
26.06.2009	122	34	129	124	26	120	9,81
17.06.2009	122	34	129	124	26	120	10,69
17.06.2009	122	34	129	124	26	120	9,73
17.06.2009	122	34	129	124	26	120	25,28
17.06.2009	122	34	129	124	26	120	20,69
17.06.2009	122	34	129	124	26	120	18,16
17.06.2009	122	34	129	124	26	120	5,64
18.06.2009	122	34	129	124	26	120	14,87
18.06.2009	122	34	129	124	26	120	2,33
18.06.2009	122	34	129	124	26	120	9,87
18.06.2009	122	34	129	124	26	120	26,55
18.06.2009	122	34	129	124	26	120	12,27
18.06.2009	122	34	129	124	26	120	16,84
18.06.2009	122	34	129	124	26	120	3,05

18.06.2009	122	34	129	124	26	120	13,07
19.06.2009	122	34	129	124	26	120	6,73
19.06.2009	122	34	129	124	26	120	12,91
10.06.2009	122	34	129	124	26	120	3,70
10.06.2009	122	34	129	124	26	120	1,15
10.06.2009	122	34	129	124	26	120	2,01
11.06.2009	122	34	129	124	26	120	16,02
11.06.2009	122	34	129	124	26	120	15,24
11.06.2009	122	34	129	124	26	120	8,63
11.06.2009	122	34	129	124	26	120	2,99
11.06.2009	122	34	129	124	26	120	27,11
11.06.2009	122	34	129	124	26	120	8,23
11.06.2009	122	34	129	124	26	120	4,02
12.06.2009	122	34	129	124	26	120	11,06
12.06.2009	122	34	129	124	26	120	8,97
12.06.2009	122	34	129	124	26	120	1,92
03.06.2009	122	34	129	124	26	120	2,66
03.06.2009	122	34	129	124	26	120	7,09
03.06.2009	122	34	129	124	26	120	5,64
03.06.2009	122	34	129	124	26	120	25,70
03.06.2009	122	34	129	124	26	120	5,52
03.06.2009	122	34	129	124	26	120	1,13
04.06.2009	122	34	129	124	26	120	7,48
04.06.2009	122	34	129	124	26	120	12,87
04.06.2009	122	34	129	124	26	120	18,43
04.06.2009	122	34	129	124	26	120	16,59
04.06.2009	122	34	129	124	26	120	0,77
04.06.2009	122	34	129	124	26	120	2,62
04.06.2009	122	34	129	124	26	120	54,55
04.06.2009	122	34	129	124	26	120	1,14
05.06.2009	122	34	129	124	26	120	14,63
05.06.2009	122	34	129	124	26	120	7,67
05.06.2009	122	34	129	124	26	120	1,80

24.06.2009	123	33	137	124	26	120	0,00
24.06.2009	123	33	137	124	26	120	2,63
24.06.2009	123	33	137	124	26	120	2,31
24.06.2009	123	33	137	124	26	120	6,62
24.06.2009	123	33	137	124	26	120	4,91
25.06.2009	123	33	137	124	26	120	7,70
25.06.2009	123	33	137	124	26	120	18,27
25.06.2009	123	33	137	124	26	120	18,99
25.06.2009	123	33	137	124	26	120	1,65
25.06.2009	123	33	137	124	26	120	2,41
25.06.2009	123	33	137	124	26	120	43,50
25.06.2009	123	33	137	124	26	120	1,71
26.06.2009	123	33	137	124	26	120	8,05
26.06.2009	123	33	137	124	26	120	5,47
26.06.2009	123	33	137	124	26	120	3,66
17.06.2009	123	33	137	124	26	120	14,46
17.06.2009	123	33	137	124	26	120	62,75
17.06.2009	123	33	137	124	26	120	59,86
17.06.2009	123	33	137	124	26	120	61,54
17.06.2009	123	33	137	124	26	120	77,84
17.06.2009	123	33	137	124	26	120	940,60
18.06.2009	123	33	137	124	26	120	25,54
18.06.2009	123	33	137	124	26	120	14,28
18.06.2009	123	33	137	124	26	120	71,86
18.06.2009	123	33	137	124	26	120	73,01
18.06.2009	123	33	137	124	26	120	66,78
18.06.2009	123	33	137	124	26	120	66,21
18.06.2009	123	33	137	124	26	120	69,57
19.06.2009	123	33	137	124	26	120	70,09
19.06.2009	123	33	137	124	26	120	23,68
10.06.2009	123	33	137	124	26	120	15,47
10.06.2009	123	33	137	124	26	120	1,45
10.06.2009	123	33	137	124	26	120	9,36

11.06.2009	123	33	137	124	26	120	43,86
11.06.2009	123	33	137	124	26	120	1,01
11.06.2009	123	33	137	124	26	120	31,32
11.06.2009	123	33	137	124	26	120	42,90
11.06.2009	123	33	137	124	26	120	74,61
11.06.2009	123	33	137	124	26	120	75,10
11.06.2009	123	33	137	124	26	120	69,19
11.06.2009	123	33	137	124	26	120	49,01
12.06.2009	123	33	137	124	26	120	67,42
12.06.2009	123	33	137	124	26	120	99,34
12.06.2009	123	33	137	124	26	120	79,70
03.06.2009	123	33	137	124	26	120	19,28
03.06.2009	123	33	137	124	26	120	15,68
03.06.2009	123	33	137	124	26	120	69,24
03.06.2009	123	33	137	124	26	120	59,48
03.06.2009	123	33	137	124	26	120	233,42
04.06.2009	123	33	137	124	26	120	222,63
04.06.2009	123	33	137	124	26	120	125,73
04.06.2009	123	33	137	124	26	120	176,41
04.06.2009	123	33	137	124	26	120	191,61
04.06.2009	123	33	137	124	26	120	187,72
04.06.2009	123	33	137	124	26	120	169,60
04.06.2009	123	33	137	124	26	120	175,17
04.06.2009	123	33	137	124	26	120	136,84
05.06.2009	123	33	137	124	26	120	173,92
05.06.2009	123	33	137	124	26	120	151,96
05.06.2009	123	33	137	124	26	120	147,34
25.06.2009	106	25	122	125	25	117	78,41
25.06.2009	106	25	122	125	25	117	95,53
25.06.2009	106	25	122	125	25	117	26,45
25.06.2009	106	25	122	125	25	117	32,42
25.06.2009	106	25	122	125	25	117	33,93
26.06.2009	106	25	122	125	25	117	40,93

26.06.2009	106	25	122	125	25	117	65,31
26.06.2009	106	25	122	125	25	117	87,37
17.06.2009	106	25	122	125	25	117	940,44
10.06.2009	106	25	122	125	25	117	915,28
10.06.2009	106	25	122	125	25	117	920,94
10.06.2009	106	25	122	125	25	117	933,30
10.06.2009	106	25	122	125	25	117	940,59
10.06.2009	106	25	122	125	25	117	940,61
10.06.2009	106	25	122	125	25	117	947,13
11.06.2009	106	25	122	125	25	117	927,48
11.06.2009	106	25	122	125	25	117	937,48
11.06.2009	106	25	122	125	25	117	943,42
11.06.2009	106	25	122	125	25	117	944,84
11.06.2009	106	25	122	125	25	117	938,47
11.06.2009	106	25	122	125	25	117	931,13
11.06.2009	106	25	122	125	25	117	930,85
11.06.2009	106	25	122	125	25	117	903,36
12.06.2009	106	25	122	125	25	117	915,94
12.06.2009	106	25	122	125	25	117	940,90
12.06.2009	106	25	122	125	25	117	897,17
03.06.2009	106	25	122	125	25	117	835,64
03.06.2009	106	25	122	125	25	117	935,17
04.06.2009	106	25	122	125	25	117	923,91
04.06.2009	106	25	122	125	25	117	897,49
04.06.2009	106	25	122	125	25	117	889,83
04.06.2009	106	25	122	125	25	117	892,01
04.06.2009	106	25	122	125	25	117	777,45
04.06.2009	106	25	122	125	25	117	943,40
04.06.2009	106	25	122	125	25	117	923,79
04.06.2009	106	25	122	125	25	117	912,15
05.06.2009	106	25	122	125	25	117	892,36
05.06.2009	106	25	122	125	25	117	913,37
05.06.2009	106	25	122	125	25	117	918,64

24.06.2009	112	17	111	125	25	117	0,00
24.06.2009	112	17	111	125	25	117	2,22
24.06.2009	112	17	111	125	25	117	4,30
24.06.2009	112	17	111	125	25	117	1,59
24.06.2009	112	17	111	125	25	117	9,04
24.06.2009	112	17	111	125	25	117	14,48
25.06.2009	112	17	111	125	25	117	6,18
25.06.2009	112	17	111	125	25	117	55,04
25.06.2009	112	17	111	125	25	117	55,30
25.06.2009	112	17	111	125	25	117	3,46
25.06.2009	112	17	111	125	25	117	14,26
25.06.2009	112	17	111	125	25	117	27,88
25.06.2009	112	17	111	125	25	117	5,97
25.06.2009	112	17	111	125	25	117	18,91
26.06.2009	112	17	111	125	25	117	36,73
26.06.2009	112	17	111	125	25	117	17,51
26.06.2009	112	17	111	125	25	117	10,36
17.06.2009	112	17	111	125	25	117	53,67
17.06.2009	112	17	111	125	25	117	57,42
17.06.2009	112	17	111	125	25	117	56,62
17.06.2009	112	17	111	125	25	117	26,44
17.06.2009	112	17	111	125	25	117	33,03
17.06.2009	112	17	111	125	25	117	21,71
18.06.2009	112	17	111	125	25	117	62,33
18.06.2009	112	17	111	125	25	117	23,10
18.06.2009	112	17	111	125	25	117	2,71
18.06.2009	112	17	111	125	25	117	0,43
18.06.2009	112	17	111	125	25	117	59,02
18.06.2009	112	17	111	125	25	117	59,60
18.06.2009	112	17	111	125	25	117	50,50
19.06.2009	112	17	111	125	25	117	6,28
19.06.2009	112	17	111	125	25	117	40,14
19.06.2009	112	17	111	125	25	117	10,09

10.06.2009	112	17	111	125	25	117	47,58
10.06.2009	112	17	111	125	25	117	46,72
10.06.2009	112	17	111	125	25	117	51,72
10.06.2009	112	17	111	125	25	117	62,52
10.06.2009	112	17	111	125	25	117	15,76
10.06.2009	112	17	111	125	25	117	16,75
11.06.2009	112	17	111	125	25	117	5,37
11.06.2009	112	17	111	125	25	117	68,74
11.06.2009	112	17	111	125	25	117	38,90
11.06.2009	112	17	111	125	25	117	56,60
11.06.2009	112	17	111	125	25	117	9,91
11.06.2009	112	17	111	125	25	117	51,56
11.06.2009	112	17	111	125	25	117	42,84
12.06.2009	112	17	111	125	25	117	6,50
12.06.2009	112	17	111	125	25	117	74,19
12.06.2009	112	17	111	125	25	117	18,78
03.06.2009	112	17	111	125	25	117	54,78
03.06.2009	112	17	111	125	25	117	46,45
03.06.2009	112	17	111	125	25	117	60,78
03.06.2009	112	17	111	125	25	117	58,46
03.06.2009	112	17	111	125	25	117	21,04
03.06.2009	112	17	111	125	25	117	67,56
04.06.2009	112	17	111	125	25	117	47,52
04.06.2009	112	17	111	125	25	117	44,24
04.06.2009	112	17	111	125	25	117	23,38
04.06.2009	112	17	111	125	25	117	29,24
04.06.2009	112	17	111	125	25	117	82,96
04.06.2009	112	17	111	125	25	117	78,15
04.06.2009	112	17	111	125	25	117	129,19
04.06.2009	112	17	111	125	25	117	51,10
05.06.2009	112	17	111	125	25	117	16,55
05.06.2009	112	17	111	125	25	117	84,56
05.06.2009	112	17	111	125	25	117	62,11

24.06.2009	113	19	106	125	25	117	0,00
24.06.2009	113	19	106	125	25	117	79,58
24.06.2009	113	19	106	125	25	117	80,09
24.06.2009	113	19	106	125	25	117	84,15
24.06.2009	113	19	106	125	25	117	98,77
24.06.2009	113	19	106	125	25	117	88,75
25.06.2009	113	19	106	125	25	117	86,07
25.06.2009	113	19	106	125	25	117	80,29
25.06.2009	113	19	106	125	25	117	135,61
25.06.2009	113	19	106	125	25	117	85,33
25.06.2009	113	19	106	125	25	117	80,24
25.06.2009	113	19	106	125	25	117	108,04
25.06.2009	113	19	106	125	25	117	84,10
25.06.2009	113	19	106	125	25	117	87,69
26.06.2009	113	19	106	125	25	117	63,86
26.06.2009	113	19	106	125	25	117	101,44
26.06.2009	113	19	106	125	25	117	94,49
17.06.2009	113	19	106	125	25	117	85,07
17.06.2009	113	19	106	125	25	117	64,94
17.06.2009	113	19	106	125	25	117	15,41
17.06.2009	113	19	106	125	25	117	88,12
17.06.2009	113	19	106	125	25	117	83,73
17.06.2009	113	19	106	125	25	117	45,15
18.06.2009	113	19	106	125	25	117	13,93
18.06.2009	113	19	106	125	25	117	91,53
18.06.2009	113	19	106	125	25	117	82,90
18.06.2009	113	19	106	125	25	117	88,03
18.06.2009	113	19	106	125	25	117	26,79
18.06.2009	113	19	106	125	25	117	75,30
18.06.2009	113	19	106	125	25	117	10,14
19.06.2009	113	19	106	125	25	117	77,89
19.06.2009	113	19	106	125	25	117	42,84
19.06.2009	113	19	106	125	25	117	20,20

10.06.2009	113	19	106	125	25	117	70,41
10.06.2009	113	19	106	125	25	117	16,98
10.06.2009	113	19	106	125	25	117	18,10
10.06.2009	113	19	106	125	25	117	71,86
10.06.2009	113	19	106	125	25	117	82,84
10.06.2009	113	19	106	125	25	117	71,53
11.06.2009	113	19	106	125	25	117	45,35
11.06.2009	113	19	106	125	25	117	31,53
11.06.2009	113	19	106	125	25	117	69,45
11.06.2009	113	19	106	125	25	117	12,49
11.06.2009	113	19	106	125	25	117	86,83
11.06.2009	113	19	106	125	25	117	82,01
11.06.2009	113	19	106	125	25	117	64,34
11.06.2009	113	19	106	125	25	117	121,38
12.06.2009	113	19	106	125	25	117	79,58
12.06.2009	113	19	106	125	25	117	24,30
12.06.2009	113	19	106	125	25	117	110,44
03.06.2009	113	19	106	125	25	117	69,84
03.06.2009	113	19	106	125	25	117	30,38
03.06.2009	113	19	106	125	25	117	39,93
03.06.2009	113	19	106	125	25	117	86,02
03.06.2009	113	19	106	125	25	117	80,60
03.06.2009	113	19	106	125	25	117	74,67
04.06.2009	113	19	106	125	25	117	92,75
04.06.2009	113	19	106	125	25	117	115,37
04.06.2009	113	19	106	125	25	117	100,36
04.06.2009	113	19	106	125	25	117	86,25
04.06.2009	113	19	106	125	25	117	84,62
04.06.2009	113	19	106	125	25	117	43,34
04.06.2009	113	19	106	125	25	117	93,06
04.06.2009	113	19	106	125	25	117	121,69
05.06.2009	113	19	106	125	25	117	119,26
05.06.2009	113	19	106	125	25	117	46,32

05.06.2009	113	19	106	125	25	117	88,51
17.06.2009	114	17	108	125	25	117	327,43
17.06.2009	114	17	108	125	25	117	70,16
17.06.2009	114	17	108	125	25	117	155,40
17.06.2009	114	17	108	125	25	117	87,58
17.06.2009	114	17	108	125	25	117	136,45
17.06.2009	114	17	108	125	25	117	108,18
18.06.2009	114	17	108	125	25	117	120,67
18.06.2009	114	17	108	125	25	117	130,68
18.06.2009	114	17	108	125	25	117	77,97
18.06.2009	114	17	108	125	25	117	93,11
18.06.2009	114	17	108	125	25	117	84,29
18.06.2009	114	17	108	125	25	117	151,49
18.06.2009	114	17	108	125	25	117	132,61
19.06.2009	114	17	108	125	25	117	84,83
19.06.2009	114	17	108	125	25	117	116,62
19.06.2009	114	17	108	125	25	117	58,65
10.06.2009	114	17	108	125	25	117	327,83
03.06.2009	114	17	108	125	25	117	10,51
03.06.2009	114	17	108	125	25	117	2,17
03.06.2009	114	17	108	125	25	117	4,41
03.06.2009	114	17	108	125	25	117	9,21
03.06.2009	114	17	108	125	25	117	101,54
03.06.2009	114	17	108	125	25	117	121,16
04.06.2009	114	17	108	125	25	117	122,12
04.06.2009	114	17	108	125	25	117	117,77
04.06.2009	114	17	108	125	25	117	116,93
04.06.2009	114	17	108	125	25	117	89,79
04.06.2009	114	17	108	125	25	117	87,54
04.06.2009	114	17	108	125	25	117	335,61
04.06.2009	114	17	108	125	25	117	309,46
04.06.2009	114	17	108	125	25	117	246,39
05.06.2009	114	17	108	125	25	117	284,06

05.06.2009	114	17	108	125	25	117	65,00
05.06.2009	114	17	108	125	25	117	322,65
24.06.2009	115	14	98	125	25	117	0,00
24.06.2009	115	14	98	125	25	117	3,08
24.06.2009	115	14	98	125	25	117	1,30
24.06.2009	115	14	98	125	25	117	7,29
24.06.2009	115	14	98	125	25	117	32,58
24.06.2009	115	14	98	125	25	117	7,28
25.06.2009	115	14	98	125	25	117	5,51
25.06.2009	115	14	98	125	25	117	29,56
25.06.2009	115	14	98	125	25	117	60,44
25.06.2009	115	14	98	125	25	117	2,31
25.06.2009	115	14	98	125	25	117	15,73
25.06.2009	115	14	98	125	25	117	27,16
25.06.2009	115	14	98	125	25	117	16,18
25.06.2009	115	14	98	125	25	117	13,21
26.06.2009	115	14	98	125	25	117	15,30
26.06.2009	115	14	98	125	25	117	33,47
26.06.2009	115	14	98	125	25	117	26,78
17.06.2009	115	14	98	125	25	117	6,84
17.06.2009	115	14	98	125	25	117	14,10
17.06.2009	115	14	98	125	25	117	4,72
17.06.2009	115	14	98	125	25	117	26,45
18.06.2009	115	14	98	125	25	117	5,18
18.06.2009	115	14	98	125	25	117	3,12
18.06.2009	115	14	98	125	25	117	6,62
18.06.2009	115	14	98	125	25	117	10,76
18.06.2009	115	14	98	125	25	117	6,82
18.06.2009	115	14	98	125	25	117	13,90
18.06.2009	115	14	98	125	25	117	8,20
19.06.2009	115	14	98	125	25	117	2,31
19.06.2009	115	14	98	125	25	117	7,00
19.06.2009	115	14	98	125	25	117	5,05

10.06.2009	115	14	98	125	25	117	16,96
10.06.2009	115	14	98	125	25	117	10,21
10.06.2009	115	14	98	125	25	117	22,37
10.06.2009	115	14	98	125	25	117	5,04
10.06.2009	115	14	98	125	25	117	14,29
10.06.2009	115	14	98	125	25	117	34,99
11.06.2009	115	14	98	125	25	117	0,50
11.06.2009	115	14	98	125	25	117	16,52
11.06.2009	115	14	98	125	25	117	1,14
11.06.2009	115	14	98	125	25	117	17,22
11.06.2009	115	14	98	125	25	117	29,35
11.06.2009	115	14	98	125	25	117	10,25
11.06.2009	115	14	98	125	25	117	0,19
11.06.2009	115	14	98	125	25	117	42,62
12.06.2009	115	14	98	125	25	117	22,78
12.06.2009	115	14	98	125	25	117	44,00
12.06.2009	115	14	98	125	25	117	61,21
03.06.2009	115	14	98	125	25	117	20,35
03.06.2009	115	14	98	125	25	117	2,01
03.06.2009	115	14	98	125	25	117	3,16
03.06.2009	115	14	98	125	25	117	8,69
03.06.2009	115	14	98	125	25	117	37,41
04.06.2009	115	14	98	125	25	117	5,74
04.06.2009	115	14	98	125	25	117	24,89
04.06.2009	115	14	98	125	25	117	23,82
04.06.2009	115	14	98	125	25	117	60,51
04.06.2009	115	14	98	125	25	117	60,49
04.06.2009	115	14	98	125	25	117	63,05
04.06.2009	115	14	98	125	25	117	4,99
04.06.2009	115	14	98	125	25	117	49,35
05.06.2009	115	14	98	125	25	117	48,83
05.06.2009	115	14	98	125	25	117	11,08
05.06.2009	115	14	98	125	25	117	32,08

17.06.2009	116	60	172	125	25	117	5,89
17.06.2009	116	60	172	125	25	117	389,48
17.06.2009	116	60	172	125	25	117	420,28
10.06.2009	116	60	172	125	25	117	5,11
10.06.2009	116	60	172	125	25	117	1,96
10.06.2009	116	60	172	125	25	117	2,21
10.06.2009	116	60	172	125	25	117	3,52
10.06.2009	116	60	172	125	25	117	27,61
10.06.2009	116	60	172	125	25	117	13,80
11.06.2009	116	60	172	125	25	117	0,94
11.06.2009	116	60	172	125	25	117	16,90
11.06.2009	116	60	172	125	25	117	2,78
11.06.2009	116	60	172	125	25	117	6,32
11.06.2009	116	60	172	125	25	117	2,70
11.06.2009	116	60	172	125	25	117	16,61
11.06.2009	116	60	172	125	25	117	26,90
11.06.2009	116	60	172	125	25	117	49,87
12.06.2009	116	60	172	125	25	117	1,14
12.06.2009	116	60	172	125	25	117	8,75
12.06.2009	116	60	172	125	25	117	57,44
03.06.2009	116	60	172	125	25	117	5,06
03.06.2009	116	60	172	125	25	117	3,92
03.06.2009	116	60	172	125	25	117	18,61
03.06.2009	116	60	172	125	25	117	8,87
03.06.2009	116	60	172	125	25	117	38,62
03.06.2009	116	60	172	125	25	117	0,23
04.06.2009	116	60	172	125	25	117	96,75
04.06.2009	116	60	172	125	25	117	113,37
04.06.2009	116	60	172	125	25	117	103,56
04.06.2009	116	60	172	125	25	117	14,36
04.06.2009	116	60	172	125	25	117	13,72
04.06.2009	116	60	172	125	25	117	10,81
04.06.2009	116	60	172	125	25	117	14,52

04.06.2009	116	60	172	125	25	117	60,91
05.06.2009	116	60	172	125	25	117	45,06
05.06.2009	116	60	172	125	25	117	18,91
05.06.2009	116	60	172	125	25	117	2,92
24.06.2009	117	29	130	125	25	117	0,00
24.06.2009	117	29	130	125	25	117	73,29
24.06.2009	117	29	130	125	25	117	63,81
24.06.2009	117	29	130	125	25	117	67,96
24.06.2009	117	29	130	125	25	117	79,34
24.06.2009	117	29	130	125	25	117	71,36
25.06.2009	117	29	130	125	25	117	69,34
25.06.2009	117	29	130	125	25	117	11,30
25.06.2009	117	29	130	125	25	117	58,95
25.06.2009	117	29	130	125	25	117	65,45
25.06.2009	117	29	130	125	25	117	46,32
25.06.2009	117	29	130	125	25	117	63,13
25.06.2009	117	29	130	125	25	117	70,43
26.06.2009	117	29	130	125	25	117	74,84
26.06.2009	117	29	130	125	25	117	53,24
26.06.2009	117	29	130	125	25	117	83,06
17.06.2009	117	29	130	125	25	117	35,20
17.06.2009	117	29	130	125	25	117	5,01
17.06.2009	117	29	130	125	25	117	4,98
17.06.2009	117	29	130	125	25	117	23,45
17.06.2009	117	29	130	125	25	117	13,39
17.06.2009	117	29	130	125	25	117	23,97
18.06.2009	117	29	130	125	25	117	1,91
18.06.2009	117	29	130	125	25	117	19,66
18.06.2009	117	29	130	125	25	117	9,61
18.06.2009	117	29	130	125	25	117	20,04
18.06.2009	117	29	130	125	25	117	15,81
18.06.2009	117	29	130	125	25	117	6,28
18.06.2009	117	29	130	125	25	117	10,77

19.06.2009	117	29	130	125	25	117	12,84
19.06.2009	117	29	130	125	25	117	3,36
19.06.2009	117	29	130	125	25	117	24,58
10.06.2009	117	29	130	125	25	117	10,95
10.06.2009	117	29	130	125	25	117	12,79
10.06.2009	117	29	130	125	25	117	19,36
10.06.2009	117	29	130	125	25	117	12,41
10.06.2009	117	29	130	125	25	117	17,48
10.06.2009	117	29	130	125	25	117	2,07
11.06.2009	117	29	130	125	25	117	17,76
11.06.2009	117	29	130	125	25	117	13,14
11.06.2009	117	29	130	125	25	117	10,61
11.06.2009	117	29	130	125	25	117	11,24
11.06.2009	117	29	130	125	25	117	30,64
11.06.2009	117	29	130	125	25	117	4,12
11.06.2009	117	29	130	125	25	117	2,03
11.06.2009	117	29	130	125	25	117	46,94
12.06.2009	117	29	130	125	25	117	12,06
12.06.2009	117	29	130	125	25	117	7,31
12.06.2009	117	29	130	125	25	117	63,35
03.06.2009	117	29	130	125	25	117	20,23
03.06.2009	117	29	130	125	25	117	11,82
03.06.2009	117	29	130	125	25	117	19,83
03.06.2009	117	29	130	125	25	117	9,10
03.06.2009	117	29	130	125	25	117	37,74
03.06.2009	117	29	130	125	25	117	2,47
04.06.2009	117	29	130	125	25	117	6,84
04.06.2009	117	29	130	125	25	117	29,46
04.06.2009	117	29	130	125	25	117	28,40
04.06.2009	117	29	130	125	25	117	11,24
04.06.2009	117	29	130	125	25	117	12,25
04.06.2009	117	29	130	125	25	117	9,14
04.06.2009	117	29	130	125	25	117	14,79

04.06.2009	117	29	130	125	25	117	62,03
05.06.2009	117	29	130	125	25	117	45,38
05.06.2009	117	29	130	125	25	117	22,96
05.06.2009	117	29	130	125	25	117	2,93
24.06.2009	118	41	141	125	25	117	0,00
24.06.2009	118	41	141	125	25	117	771,84
24.06.2009	118	41	141	125	25	117	764,85
24.06.2009	118	41	141	125	25	117	933,01
24.06.2009	118	41	141	125	25	117	887,97
24.06.2009	118	41	141	125	25	117	919,77
25.06.2009	118	41	141	125	25	117	932,44
25.06.2009	118	41	141	125	25	117	939,69
25.06.2009	118	41	141	125	25	117	896,03
25.06.2009	118	41	141	125	25	117	914,54
25.06.2009	118	41	141	125	25	117	942,15
25.06.2009	118	41	141	125	25	117	758,63
25.06.2009	118	41	141	125	25	117	764,06
25.06.2009	118	41	141	125	25	117	857,25
26.06.2009	118	41	141	125	25	117	778,45
26.06.2009	118	41	141	125	25	117	898,57
26.06.2009	118	41	141	125	25	117	926,85
17.06.2009	118	41	141	125	25	117	941,53
17.06.2009	118	41	141	125	25	117	937,00
17.06.2009	118	41	141	125	25	117	865,05
17.06.2009	118	41	141	125	25	117	857,03
17.06.2009	118	41	141	125	25	117	774,03
17.06.2009	118	41	141	125	25	117	932,51
18.06.2009	118	41	141	125	25	117	771,25
18.06.2009	118	41	141	125	25	117	773,55
18.06.2009	118	41	141	125	25	117	751,60
18.06.2009	118	41	141	125	25	117	761,23
18.06.2009	118	41	141	125	25	117	855,47
18.06.2009	118	41	141	125	25	117	946,22

18.06.2009	118	41	141	125	25	117	943,28
19.06.2009	118	41	141	125	25	117	915,37
19.06.2009	118	41	141	125	25	117	881,27
19.06.2009	118	41	141	125	25	117	844,89
10.06.2009	118	41	141	125	25	117	918,33
10.06.2009	118	41	141	125	25	117	326,66
10.06.2009	118	41	141	125	25	117	871,58
10.06.2009	118	41	141	125	25	117	889,84
10.06.2009	118	41	141	125	25	117	934,29
10.06.2009	118	41	141	125	25	117	930,32
11.06.2009	118	41	141	125	25	117	799,36
11.06.2009	118	41	141	125	25	117	920,95
11.06.2009	118	41	141	125	25	117	810,37
11.06.2009	118	41	141	125	25	117	852,06
11.06.2009	118	41	141	125	25	117	769,82
11.06.2009	118	41	141	125	25	117	864,37
11.06.2009	118	41	141	125	25	117	880,43
11.06.2009	118	41	141	125	25	117	835,53
12.06.2009	118	41	141	125	25	117	945,72
12.06.2009	118	41	141	125	25	117	932,27
12.06.2009	118	41	141	125	25	117	779,94
03.06.2009	118	41	141	125	25	117	851,12
03.06.2009	118	41	141	125	25	117	934,59
04.06.2009	118	41	141	125	25	117	921,33
04.06.2009	118	41	141	125	25	117	899,37
04.06.2009	118	41	141	125	25	117	917,73
04.06.2009	118	41	141	125	25	117	759,54
04.06.2009	118	41	141	125	25	117	799,56
04.06.2009	118	41	141	125	25	117	825,04
04.06.2009	118	41	141	125	25	117	924,51
04.06.2009	118	41	141	125	25	117	740,24
05.06.2009	118	41	141	125	25	117	899,88
05.06.2009	118	41	141	125	25	117	823,16

05.06.2009	118	41	141	125	25	117	769,86
24.06.2009	119	88	198	125	25	117	0,00
24.06.2009	119	88	198	125	25	117	121,40
24.06.2009	119	88	198	125	25	117	120,85
24.06.2009	119	88	198	125	25	117	122,92
24.06.2009	119	88	198	125	25	117	128,94
24.06.2009	119	88	198	125	25	117	121,61
25.06.2009	119	88	198	125	25	117	125,98
25.06.2009	119	88	198	125	25	117	103,37
25.06.2009	119	88	198	125	25	117	166,89
25.06.2009	119	88	198	125	25	117	91,37
25.06.2009	119	88	198	125	25	117	227,81
25.06.2009	119	88	198	125	25	117	86,28
25.06.2009	119	88	198	125	25	117	98,94
25.06.2009	119	88	198	125	25	117	21,37
26.06.2009	119	88	198	125	25	117	18,13
26.06.2009	119	88	198	125	25	117	31,73
26.06.2009	119	88	198	125	25	117	67,74
17.06.2009	119	88	198	125	25	117	156,37
17.06.2009	119	88	198	125	25	117	114,34
17.06.2009	119	88	198	125	25	117	124,50
17.06.2009	119	88	198	125	25	117	98,30
17.06.2009	119	88	198	125	25	117	141,47
17.06.2009	119	88	198	125	25	117	132,28
18.06.2009	119	88	198	125	25	117	111,82
18.06.2009	119	88	198	125	25	117	140,38
18.06.2009	119	88	198	125	25	117	121,24
18.06.2009	119	88	198	125	25	117	110,02
18.06.2009	119	88	198	125	25	117	130,70
18.06.2009	119	88	198	125	25	117	120,27
18.06.2009	119	88	198	125	25	117	115,67
19.06.2009	119	88	198	125	25	117	96,46
19.06.2009	119	88	198	125	25	117	114,15

19.06.2009	119	88	198	125	25	117	142,44
10.06.2009	119	88	198	125	25	117	145,08
10.06.2009	119	88	198	125	25	117	150,94
10.06.2009	119	88	198	125	25	117	92,86
10.06.2009	119	88	198	125	25	117	151,63
10.06.2009	119	88	198	125	25	117	153,56
10.06.2009	119	88	198	125	25	117	124,37
11.06.2009	119	88	198	125	25	117	91,17
11.06.2009	119	88	198	125	25	117	91,37
11.06.2009	119	88	198	125	25	117	115,16
11.06.2009	119	88	198	125	25	117	124,26
11.06.2009	119	88	198	125	25	117	151,25
11.06.2009	119	88	198	125	25	117	138,28
11.06.2009	119	88	198	125	25	117	94,49
11.06.2009	119	88	198	125	25	117	136,38
12.06.2009	119	88	198	125	25	117	130,52
12.06.2009	119	88	198	125	25	117	99,92
12.06.2009	119	88	198	125	25	117	135,78
03.06.2009	119	88	198	125	25	117	123,43
03.06.2009	119	88	198	125	25	117	83,04
03.06.2009	119	88	198	125	25	117	99,78
03.06.2009	119	88	198	125	25	117	99,11
03.06.2009	119	88	198	125	25	117	126,32
03.06.2009	119	88	198	125	25	117	80,74
04.06.2009	119	88	198	125	25	117	107,42
04.06.2009	119	88	198	125	25	117	137,91
04.06.2009	119	88	198	125	25	117	153,33
04.06.2009	119	88	198	125	25	117	37,00
04.06.2009	119	88	198	125	25	117	80,08
04.06.2009	119	88	198	125	25	117	102,13
04.06.2009	119	88	198	125	25	117	96,60
04.06.2009	119	88	198	125	25	117	144,14
05.06.2009	119	88	198	125	25	117	154,47

05.06.2009	119	88	198	125	25	117	108,80
05.06.2009	119	88	198	125	25	117	133,03
17.06.2009	120	24	122	125	25	117	16,22
17.06.2009	120	24	122	125	25	117	5,82
17.06.2009	120	24	122	125	25	117	11,02
17.06.2009	120	24	122	125	25	117	22,00
17.06.2009	120	24	122	125	25	117	29,79
17.06.2009	120	24	122	125	25	117	29,07
18.06.2009	120	24	122	125	25	117	8,33
18.06.2009	120	24	122	125	25	117	29,09
18.06.2009	120	24	122	125	25	117	18,76
18.06.2009	120	24	122	125	25	117	19,60
18.06.2009	120	24	122	125	25	117	27,40
18.06.2009	120	24	122	125	25	117	8,58
19.06.2009	120	24	122	125	25	117	12,35
19.06.2009	120	24	122	125	25	117	27,28
19.06.2009	120	24	122	125	25	117	20,99
10.06.2009	120	24	122	125	25	117	9,61
10.06.2009	120	24	122	125	25	117	5,13
10.06.2009	120	24	122	125	25	117	16,85
10.06.2009	120	24	122	125	25	117	12,39
10.06.2009	120	24	122	125	25	117	1,37
10.06.2009	120	24	122	125	25	117	13,80
11.06.2009	120	24	122	125	25	117	11,24
11.06.2009	120	24	122	125	25	117	15,90
11.06.2009	120	24	122	125	25	117	17,43
11.06.2009	120	24	122	125	25	117	19,06
11.06.2009	120	24	122	125	25	117	18,78
11.06.2009	120	24	122	125	25	117	16,46
11.06.2009	120	24	122	125	25	117	11,56
11.06.2009	120	24	122	125	25	117	48,63
12.06.2009	120	24	122	125	25	117	26,12
12.06.2009	120	24	122	125	25	117	9,02

12.06.2009	120	24	122	125	25	117	65,14
03.06.2009	120	24	122	125	25	117	58,87
03.06.2009	120	24	122	125	25	117	5,13
03.06.2009	120	24	122	125	25	117	52,79
03.06.2009	120	24	122	125	25	117	42,39
03.06.2009	120	24	122	125	25	117	70,13
04.06.2009	120	24	122	125	25	117	2,97
04.06.2009	120	24	122	125	25	117	37,66
04.06.2009	120	24	122	125	25	117	29,64
04.06.2009	120	24	122	125	25	117	0,86
04.06.2009	120	24	122	125	25	117	6,33
04.06.2009	120	24	122	125	25	117	8,82
04.06.2009	120	24	122	125	25	117	24,59
04.06.2009	120	24	122	125	25	117	60,64
05.06.2009	120	24	122	125	25	117	109,57
05.06.2009	120	24	122	125	25	117	29,79
05.06.2009	120	24	122	125	25	117	16,61
25.06.2009	121	40	136	125	25	117	12,19
17.06.2009	121	40	136	125	25	117	14,46
17.06.2009	121	40	136	125	25	117	25,69
17.06.2009	121	40	136	125	25	117	18,64
17.06.2009	121	40	136	125	25	117	27,25
17.06.2009	121	40	136	125	25	117	11,85
17.06.2009	121	40	136	125	25	117	16,35
18.06.2009	121	40	136	125	25	117	3,20
18.06.2009	121	40	136	125	25	117	23,06
18.06.2009	121	40	136	125	25	117	23,37
18.06.2009	121	40	136	125	25	117	20,54
18.06.2009	121	40	136	125	25	117	7,36
18.06.2009	121	40	136	125	25	117	8,63
18.06.2009	121	40	136	125	25	117	14,08
19.06.2009	121	40	136	125	25	117	10,49
19.06.2009	121	40	136	125	25	117	18,48

19.06.2009	121	40	136	125	25	117	2,39
10.06.2009	121	40	136	125	25	117	123,49
10.06.2009	121	40	136	125	25	117	5,57
10.06.2009	121	40	136	125	25	117	1,02
10.06.2009	121	40	136	125	25	117	3,23
10.06.2009	121	40	136	125	25	117	26,46
10.06.2009	121	40	136	125	25	117	11,84
11.06.2009	121	40	136	125	25	117	14,40
11.06.2009	121	40	136	125	25	117	4,02
11.06.2009	121	40	136	125	25	117	16,79
11.06.2009	121	40	136	125	25	117	7,27
11.06.2009	121	40	136	125	25	117	14,04
11.06.2009	121	40	136	125	25	117	2,81
11.06.2009	121	40	136	125	25	117	19,72
11.06.2009	121	40	136	125	25	117	47,83
12.06.2009	121	40	136	125	25	117	2,14
12.06.2009	121	40	136	125	25	117	13,85
12.06.2009	121	40	136	125	25	117	63,51
03.06.2009	121	40	136	125	25	117	1,29
03.06.2009	121	40	136	125	25	117	2,30
03.06.2009	121	40	136	125	25	117	2,91
03.06.2009	121	40	136	125	25	117	4,61
03.06.2009	121	40	136	125	25	117	42,97
03.06.2009	121	40	136	125	25	117	56,23
04.06.2009	121	40	136	125	25	117	14,03
04.06.2009	121	40	136	125	25	117	85,55
04.06.2009	121	40	136	125	25	117	8,27
04.06.2009	121	40	136	125	25	117	10,62
04.06.2009	121	40	136	125	25	117	11,11
04.06.2009	121	40	136	125	25	117	15,47
04.06.2009	121	40	136	125	25	117	62,33
05.06.2009	121	40	136	125	25	117	38,93
05.06.2009	121	40	136	125	25	117	19,06

05.06.2009	121	40	136	125	25	117	3,49
24.06.2009	122	34	129	125	25	117	0,00
24.06.2009	122	34	129	125	25	117	15,04
24.06.2009	122	34	129	125	25	117	10,61
24.06.2009	122	34	129	125	25	117	19,33
24.06.2009	122	34	129	125	25	117	32,71
24.06.2009	122	34	129	125	25	117	5,85
25.06.2009	122	34	129	125	25	117	20,02
25.06.2009	122	34	129	125	25	117	10,37
25.06.2009	122	34	129	125	25	117	64,24
25.06.2009	122	34	129	125	25	117	14,63
25.06.2009	122	34	129	125	25	117	3,36
25.06.2009	122	34	129	125	25	117	29,90
25.06.2009	122	34	129	125	25	117	23,74
25.06.2009	122	34	129	125	25	117	19,09
26.06.2009	122	34	129	125	25	117	4,85
26.06.2009	122	34	129	125	25	117	30,98
26.06.2009	122	34	129	125	25	117	12,34
17.06.2009	122	34	129	125	25	117	7,50
17.06.2009	122	34	129	125	25	117	9,28
17.06.2009	122	34	129	125	25	117	10,34
17.06.2009	122	34	129	125	25	117	24,62
17.06.2009	122	34	129	125	25	117	19,23
17.06.2009	122	34	129	125	25	117	38,57
18.06.2009	122	34	129	125	25	117	10,06
18.06.2009	122	34	129	125	25	117	25,12
18.06.2009	122	34	129	125	25	117	28,60
18.06.2009	122	34	129	125	25	117	22,76
18.06.2009	122	34	129	125	25	117	24,64
18.06.2009	122	34	129	125	25	117	8,93
18.06.2009	122	34	129	125	25	117	11,87
19.06.2009	122	34	129	125	25	117	5,93
19.06.2009	122	34	129	125	25	117	11,28

19.06.2009	122	34	129	125	25	117	8,07
10.06.2009	122	34	129	125	25	117	17,13
10.06.2009	122	34	129	125	25	117	5,21
10.06.2009	122	34	129	125	25	117	7,34
10.06.2009	122	34	129	125	25	117	1,41
10.06.2009	122	34	129	125	25	117	4,47
11.06.2009	122	34	129	125	25	117	1,80
11.06.2009	122	34	129	125	25	117	3,70
11.06.2009	122	34	129	125	25	117	16,48
11.06.2009	122	34	129	125	25	117	12,52
11.06.2009	122	34	129	125	25	117	10,64
11.06.2009	122	34	129	125	25	117	14,34
11.06.2009	122	34	129	125	25	117	16,83
12.06.2009	122	34	129	125	25	117	10,40
12.06.2009	122	34	129	125	25	117	13,61
12.06.2009	122	34	129	125	25	117	58,21
03.06.2009	122	34	129	125	25	117	1,98
03.06.2009	122	34	129	125	25	117	9,35
03.06.2009	122	34	129	125	25	117	4,47
03.06.2009	122	34	129	125	25	117	22,67
03.06.2009	122	34	129	125	25	117	36,66
03.06.2009	122	34	129	125	25	117	2,10
04.06.2009	122	34	129	125	25	117	5,46
04.06.2009	122	34	129	125	25	117	29,14
04.06.2009	122	34	129	125	25	117	18,83
04.06.2009	122	34	129	125	25	117	12,66
04.06.2009	122	34	129	125	25	117	6,31
04.06.2009	122	34	129	125	25	117	13,86
04.06.2009	122	34	129	125	25	117	38,78
04.06.2009	122	34	129	125	25	117	62,06
05.06.2009	122	34	129	125	25	117	41,66
05.06.2009	122	34	129	125	25	117	19,54
05.06.2009	122	34	129	125	25	117	13,58

24.06.2009	123	33	137	125	25	117	0,00
24.06.2009	123	33	137	125	25	117	2,34
24.06.2009	123	33	137	125	25	117	14,87
24.06.2009	123	33	137	125	25	117	21,74
24.06.2009	123	33	137	125	25	117	7,39
25.06.2009	123	33	137	125	25	117	18,21
25.06.2009	123	33	137	125	25	117	5,95
25.06.2009	123	33	137	125	25	117	59,45
25.06.2009	123	33	137	125	25	117	19,14
25.06.2009	123	33	137	125	25	117	24,36
25.06.2009	123	33	137	125	25	117	16,51
25.06.2009	123	33	137	125	25	117	22,83
26.06.2009	123	33	137	125	25	117	12,26
26.06.2009	123	33	137	125	25	117	28,79
26.06.2009	123	33	137	125	25	117	18,44
17.06.2009	123	33	137	125	25	117	13,96
17.06.2009	123	33	137	125	25	117	66,81
17.06.2009	123	33	137	125	25	117	65,00
17.06.2009	123	33	137	125	25	117	59,08
17.06.2009	123	33	137	125	25	117	74,36
17.06.2009	123	33	137	125	25	117	928,44
18.06.2009	123	33	137	125	25	117	1,10
18.06.2009	123	33	137	125	25	117	68,26
18.06.2009	123	33	137	125	25	117	73,85
18.06.2009	123	33	137	125	25	117	69,38
18.06.2009	123	33	137	125	25	117	68,02
18.06.2009	123	33	137	125	25	117	64,35
19.06.2009	123	33	137	125	25	117	70,58
19.06.2009	123	33	137	125	25	117	7,88
19.06.2009	123	33	137	125	25	117	4,55
10.06.2009	123	33	137	125	25	117	52,96
10.06.2009	123	33	137	125	25	117	55,42
10.06.2009	123	33	137	125	25	117	62,14

10.06.2009	123	33	137	125	25	117	14,29
10.06.2009	123	33	137	125	25	117	2,84
10.06.2009	123	33	137	125	25	117	7,23
11.06.2009	123	33	137	125	25	117	61,04
11.06.2009	123	33	137	125	25	117	16,52
11.06.2009	123	33	137	125	25	117	7,82
11.06.2009	123	33	137	125	25	117	52,09
11.06.2009	123	33	137	125	25	117	69,44
11.06.2009	123	33	137	125	25	117	67,25
11.06.2009	123	33	137	125	25	117	73,35
11.06.2009	123	33	137	125	25	117	6,60
12.06.2009	123	33	137	125	25	117	68,84
12.06.2009	123	33	137	125	25	117	117,24
12.06.2009	123	33	137	125	25	117	26,36
03.06.2009	123	33	137	125	25	117	19,57
03.06.2009	123	33	137	125	25	117	0,93
03.06.2009	123	33	137	125	25	117	69,21
03.06.2009	123	33	137	125	25	117	57,44
03.06.2009	123	33	137	125	25	117	233,52
04.06.2009	123	33	137	125	25	117	228,33
04.06.2009	123	33	137	125	25	117	167,38
04.06.2009	123	33	137	125	25	117	208,30
04.06.2009	123	33	137	125	25	117	192,35
04.06.2009	123	33	137	125	25	117	183,74
04.06.2009	123	33	137	125	25	117	160,22
04.06.2009	123	33	137	125	25	117	188,44
04.06.2009	123	33	137	125	25	117	192,24
05.06.2009	123	33	137	125	25	117	221,80
05.06.2009	123	33	137	125	25	117	173,10
05.06.2009	123	33	137	125	25	117	159,02
24.06.2009	124	26	120	125	25	117	0,00
24.06.2009	124	26	120	125	25	117	4,91
24.06.2009	124	26	120	125	25	117	9,78

24.06.2009	124	26	120	125	25	117	12,85
24.06.2009	124	26	120	125	25	117	15,97
24.06.2009	124	26	120	125	25	117	12,29
25.06.2009	124	26	120	125	25	117	24,90
25.06.2009	124	26	120	125	25	117	20,16
25.06.2009	124	26	120	125	25	117	59,43
25.06.2009	124	26	120	125	25	117	17,65
25.06.2009	124	26	120	125	25	117	22,08
25.06.2009	124	26	120	125	25	117	29,31
25.06.2009	124	26	120	125	25	117	24,39
25.06.2009	124	26	120	125	25	117	24,33
26.06.2009	124	26	120	125	25	117	4,36
26.06.2009	124	26	120	125	25	117	28,25
26.06.2009	124	26	120	125	25	117	21,99
17.06.2009	124	26	120	125	25	117	18,15
17.06.2009	124	26	120	125	25	117	18,67
17.06.2009	124	26	120	125	25	117	17,20
17.06.2009	124	26	120	125	25	117	4,95
17.06.2009	124	26	120	125	25	117	3,93
17.06.2009	124	26	120	125	25	117	33,90
18.06.2009	124	26	120	125	25	117	5,39
18.06.2009	124	26	120	125	25	117	24,84
18.06.2009	124	26	120	125	25	117	5,76
18.06.2009	124	26	120	125	25	117	11,11
18.06.2009	124	26	120	125	25	117	7,84
18.06.2009	124	26	120	125	25	117	5,91
18.06.2009	124	26	120	125	25	117	5,54
19.06.2009	124	26	120	125	25	117	12,51
19.06.2009	124	26	120	125	25	117	20,26
10.06.2009	124	26	120	125	25	117	5,49
10.06.2009	124	26	120	125	25	117	1,60
10.06.2009	124	26	120	125	25	117	3,97
11.06.2009	124	26	120	125	25	117	17,21

11.06.2009	124	26	120	125	25	117	17,46
11.06.2009	124	26	120	125	25	117	23,54
11.06.2009	124	26	120	125	25	117	10,11
11.06.2009	124	26	120	125	25	117	35,80
11.06.2009	124	26	120	125	25	117	22,30
11.06.2009	124	26	120	125	25	117	20,47
11.06.2009	124	26	120	125	25	117	50,50
12.06.2009	124	26	120	125	25	117	21,42
12.06.2009	124	26	120	125	25	117	22,40
12.06.2009	124	26	120	125	25	117	60,12
03.06.2009	124	26	120	125	25	117	0,68
03.06.2009	124	26	120	125	25	117	14,75
03.06.2009	124	26	120	125	25	117	7,77
03.06.2009	124	26	120	125	25	117	15,89
03.06.2009	124	26	120	125	25	117	39,22
03.06.2009	124	26	120	125	25	117	3,15
04.06.2009	124	26	120	125	25	117	5,71
04.06.2009	124	26	120	125	25	117	41,72
04.06.2009	124	26	120	125	25	117	33,48
04.06.2009	124	26	120	125	25	117	6,13
04.06.2009	124	26	120	125	25	117	5,73
04.06.2009	124	26	120	125	25	117	13,58
04.06.2009	124	26	120	125	25	117	15,77
04.06.2009	124	26	120	125	25	117	61,22
05.06.2009	124	26	120	125	25	117	50,16
05.06.2009	124	26	120	125	25	117	26,03
05.06.2009	124	26	120	125	25	117	14,06
04.06.2008	55	88	188	69	31	131	1,59
05.06.2008	55	88	188	69	31	131	1,13
06.06.2008	55	88	188	69	31	131	1,23
05.06.2008	55	88	188	69	31	131	3,56
06.06.2008	55	88	188	69	31	131	1,45
05.06.2008	55	88	188	69	31	131	3,84

06.06.2008	55	88	188	69	31	131	1,47
04.06.2008	55	88	188	69	31	131	3,83
05.06.2008	55	88	188	69	31	131	0,99
04.06.2008	55	88	188	69	31	131	1,18
05.06.2008	55	88	188	69	31	131	0,67
04.06.2008	55	88	188	69	31	131	2,70
05.06.2008	55	88	188	69	31	131	2,20
04.06.2008	55	88	188	69	31	131	1,86
05.06.2008	55	88	188	69	31	131	0,76
04.06.2008	55	88	188	69	31	131	0,86
05.06.2008	55	88	188	69	31	131	1,60
11.06.2008	55	88	188	69	31	131	1,60
11.06.2008	55	88	188	69	31	131	1,28
11.06.2008	55	88	188	69	31	131	9,04
11.06.2008	55	88	188	69	31	131	9,62
11.06.2008	55	88	188	69	31	131	44,75
11.06.2008	55	88	188	69	31	131	4,20
12.06.2008	55	88	188	69	31	131	50,34
12.06.2008	55	88	188	69	31	131	59,52
12.06.2008	55	88	188	69	31	131	49,78
12.06.2008	55	88	188	69	31	131	54,20
12.06.2008	55	88	188	69	31	131	41,76
12.06.2008	55	88	188	69	31	131	52,84
12.06.2008	55	88	188	69	31	131	47,37
12.06.2008	55	88	188	69	31	131	24,63
12.06.2008	55	88	188	69	31	131	37,59
13.06.2008	55	88	188	69	31	131	46,09
13.06.2008	55	88	188	69	31	131	77,97
18.06.2008	55	88	188	69	31	131	1,81
18.06.2008	55	88	188	69	31	131	2,46
18.06.2008	55	88	188	69	31	131	5,61
18.06.2008	55	88	188	69	31	131	6,44
18.06.2008	55	88	188	69	31	131	4,09

18.06.2008	55	88	188	69	31	131	3,27
19.06.2008	55	88	188	69	31	131	0,38
19.06.2008	55	88	188	69	31	131	4,32
19.06.2008	55	88	188	69	31	131	3,84
19.06.2008	55	88	188	69	31	131	5,76
19.06.2008	55	88	188	69	31	131	1,57
19.06.2008	55	88	188	69	31	131	0,23
19.06.2008	55	88	188	69	31	131	0,66
19.06.2008	55	88	188	69	31	131	1,86
20.06.2008	55	88	188	69	31	131	3,35
20.06.2008	55	88	188	69	31	131	4,05
20.06.2008	55	88	188	69	31	131	2,85
04.06.2008	55	88	188	71	19	113	1,28
05.06.2008	55	88	188	71	19	113	75,08
06.06.2008	55	88	188	71	19	113	2,60
05.06.2008	55	88	188	71	19	113	12,84
06.06.2008	55	88	188	71	19	113	4,05
05.06.2008	55	88	188	71	19	113	9,05
06.06.2008	55	88	188	71	19	113	4,24
04.06.2008	55	88	188	71	19	113	6,84
05.06.2008	55	88	188	71	19	113	1,11
04.06.2008	55	88	188	71	19	113	2,00
05.06.2008	55	88	188	71	19	113	6,78
04.06.2008	55	88	188	71	19	113	6,02
05.06.2008	55	88	188	71	19	113	60,65
04.06.2008	55	88	188	71	19	113	5,26
05.06.2008	55	88	188	71	19	113	55,69
04.06.2008	55	88	188	71	19	113	68,52
05.06.2008	55	88	188	71	19	113	1,61
11.06.2008	55	88	188	71	19	113	4,88
11.06.2008	55	88	188	71	19	113	25,93
11.06.2008	55	88	188	71	19	113	187,02
11.06.2008	55	88	188	71	19	113	36,90

11.06.2008	55	88	188	71	19	113	57,72
11.06.2008	55	88	188	71	19	113	6,97
12.06.2008	55	88	188	71	19	113	150,72
12.06.2008	55	88	188	71	19	113	1,22
12.06.2008	55	88	188	71	19	113	126,30
12.06.2008	55	88	188	71	19	113	189,64
12.06.2008	55	88	188	71	19	113	56,70
12.06.2008	55	88	188	71	19	113	77,28
12.06.2008	55	88	188	71	19	113	56,37
12.06.2008	55	88	188	71	19	113	28,09
12.06.2008	55	88	188	71	19	113	35,99
13.06.2008	55	88	188	71	19	113	37,98
13.06.2008	55	88	188	71	19	113	80,07
18.06.2008	55	88	188	71	19	113	50,63
18.06.2008	55	88	188	71	19	113	53,67
18.06.2008	55	88	188	71	19	113	51,76
18.06.2008	55	88	188	71	19	113	48,54
18.06.2008	55	88	188	71	19	113	52,63
18.06.2008	55	88	188	71	19	113	50,59
19.06.2008	55	88	188	71	19	113	48,88
19.06.2008	55	88	188	71	19	113	47,36
19.06.2008	55	88	188	71	19	113	50,99
19.06.2008	55	88	188	71	19	113	48,45
19.06.2008	55	88	188	71	19	113	49,39
19.06.2008	55	88	188	71	19	113	51,61
19.06.2008	55	88	188	71	19	113	56,15
19.06.2008	55	88	188	71	19	113	47,15
20.06.2008	55	88	188	71	19	113	44,37
20.06.2008	55	88	188	71	19	113	52,21
20.06.2008	55	88	188	71	19	113	53,52
25.06.2008	55	88	188	71	19	113	46,24
25.06.2008	55	88	188	71	19	113	55,48
25.06.2008	55	88	188	71	19	113	46,78

25.06.2008	55	88	188	71	19	113	43,81
25.06.2008	55	88	188	71	19	113	48,82
25.06.2008	55	88	188	71	19	113	48,39
26.06.2008	55	88	188	71	19	113	49,08
26.06.2008	55	88	188	71	19	113	45,57
26.06.2008	55	88	188	71	19	113	49,88
26.06.2008	55	88	188	71	19	113	47,15
26.06.2008	55	88	188	71	19	113	51,68
26.06.2008	55	88	188	71	19	113	48,37
27.06.2008	55	88	188	71	19	113	47,22
27.06.2008	55	88	188	71	19	113	53,26
04.06.2008	69	31	131	71	19	113	0,33
05.06.2008	69	31	131	71	19	113	76,18
06.06.2008	69	31	131	71	19	113	3,82
05.06.2008	69	31	131	71	19	113	9,48
06.06.2008	69	31	131	71	19	113	3,02
05.06.2008	69	31	131	71	19	113	6,43
06.06.2008	69	31	131	71	19	113	2,77
04.06.2008	69	31	131	71	19	113	3,02
05.06.2008	69	31	131	71	19	113	0,87
04.06.2008	69	31	131	71	19	113	3,13
05.06.2008	69	31	131	71	19	113	6,34
04.06.2008	69	31	131	71	19	113	7,40
05.06.2008	69	31	131	71	19	113	58,76
04.06.2008	69	31	131	71	19	113	6,12
05.06.2008	69	31	131	71	19	113	55,18
04.06.2008	69	31	131	71	19	113	69,12
05.06.2008	69	31	131	71	19	113	1,95
11.06.2008	69	31	131	71	19	113	5,79
11.06.2008	69	31	131	71	19	113	24,81
11.06.2008	69	31	131	71	19	113	179,22
11.06.2008	69	31	131	71	19	113	35,98
11.06.2008	69	31	131	71	19	113	13,96

11.06.2008	69	31	131	71	19	113	3,76
12.06.2008	69	31	131	71	19	113	108,53
12.06.2008	69	31	131	71	19	113	58,70
12.06.2008	69	31	131	71	19	113	89,20
12.06.2008	69	31	131	71	19	113	144,60
12.06.2008	69	31	131	71	19	113	29,16
12.06.2008	69	31	131	71	19	113	121,11
12.06.2008	69	31	131	71	19	113	97,64
12.06.2008	69	31	131	71	19	113	28,20
12.06.2008	69	31	131	71	19	113	1,69
13.06.2008	69	31	131	71	19	113	17,43
13.06.2008	69	31	131	71	19	113	9,24
18.06.2008	69	31	131	71	19	113	50,28
18.06.2008	69	31	131	71	19	113	51,84
18.06.2008	69	31	131	71	19	113	47,15
18.06.2008	69	31	131	71	19	113	44,57
18.06.2008	69	31	131	71	19	113	50,47
18.06.2008	69	31	131	71	19	113	48,16
19.06.2008	69	31	131	71	19	113	48,51
19.06.2008	69	31	131	71	19	113	44,60
19.06.2008	69	31	131	71	19	113	48,06
19.06.2008	69	31	131	71	19	113	52,29
19.06.2008	69	31	131	71	19	113	48,45
19.06.2008	69	31	131	71	19	113	51,44
19.06.2008	69	31	131	71	19	113	55,81
19.06.2008	69	31	131	71	19	113	48,98
20.06.2008	69	31	131	71	19	113	46,21
20.06.2008	69	31	131	71	19	113	55,49
20.06.2008	69	31	131	71	19	113	51,29
04.06.2008	50	20	108	51	22	116	18,26
04.06.2008	50	20	108	56	254	276	50,07
04.06.2008	51	22	116	56	254	276	51,88
04.06.2008	50	20	108	57	27	116	83,27

04.06.2008	51	22	116	57	27	116	72,76
04.06.2008	56	254	276	57	27	116	51,71
04.06.2008	50	20	108	63	115	203	699,07
04.06.2008	51	22	116	63	115	203	704,89
04.06.2008	56	254	276	63	115	203	747,90
04.06.2008	57	27	116	63	115	203	776,63
04.06.2008	50	20	108	64	38	140	822,06
04.06.2008	51	22	116	64	38	140	828,38
04.06.2008	56	254	276	64	38	140	870,53
04.06.2008	57	27	116	64	38	140	900,39
06.06.2008	50	20	108	63	115	203	658,92
04.06.2008	50	20	108	65	24	123	77,80
04.06.2008	51	22	116	65	24	123	67,30
04.06.2008	56	254	276	65	24	123	47,62
04.06.2008	57	27	116	65	24	123	5,49
04.06.2008	63	115	203	64	38	140	125,13
04.06.2008	63	115	203	65	24	123	771,32
04.06.2008	50	20	108	70	25	123	114,85
04.06.2008	51	22	116	70	25	123	103,53
04.06.2008	56	254	276	70	25	123	80,94
04.06.2008	57	27	116	70	25	123	31,69
04.06.2008	63	115	203	70	25	123	805,27
04.06.2008	63	115	203	64	38	140	17,20
04.06.2008	65	24	123	70	25	123	37,10
04.06.2008	50	20	108	51	22	116	17,18
04.06.2008	50	20	108	56	254	276	61,77
04.06.2008	51	22	116	56	254	276	54,41
04.06.2008	50	20	108	57	27	116	115,00
04.06.2008	51	22	116	57	27	116	102,18
04.06.2008	56	254	276	57	27	116	59,63
04.06.2008	50	20	108	63	115	203	714,92
04.06.2008	51	22	116	63	115	203	722,97
04.06.2008	56	254	276	63	115	203	776,43

04.06.2008	57	27	116	63	115	203	821,42
04.06.2008	50	20	108	64	38	140	732,04
04.06.2008	51	22	116	64	38	140	740,12
04.06.2008	56	254	276	64	38	140	793,56
04.06.2008	57	27	116	64	38	140	838,60
04.06.2008	63	115	203	65	24	123	803,75
04.06.2008	50	20	108	65	24	123	95,02
04.06.2008	51	22	116	65	24	123	82,72
04.06.2008	56	254	276	65	24	123	39,94
04.06.2008	57	27	116	65	24	123	20,23
04.06.2008	63	115	203	70	25	123	863,60
04.06.2008	63	115	203	64	38	140	18,33
04.06.2008	50	20	108	70	25	123	161,91
04.06.2008	51	22	116	70	25	123	148,53
04.06.2008	56	254	276	70	25	123	106,15
04.06.2008	57	27	116	70	25	123	47,14
04.06.2008	63	115	203	65	24	123	747,10
04.06.2008	63	115	203	70	25	123	832,25
04.06.2008	65	24	123	70	25	123	67,36
04.06.2008	50	20	108	51	22	116	12,11
04.06.2008	50	20	108	56	254	276	53,02
04.06.2008	51	22	116	56	254	276	41,46
04.06.2008	50	20	108	57	27	116	88,45
04.06.2008	51	22	116	57	27	116	77,31
04.06.2008	56	254	276	57	27	116	55,93
04.06.2008	50	20	108	63	115	203	674,91
04.06.2008	51	22	116	63	115	203	686,89
04.06.2008	56	254	276	63	115	203	723,82
04.06.2008	57	27	116	63	115	203	761,25
04.06.2008	50	20	108	64	38	140	692,95
04.06.2008	51	22	116	64	38	140	704,93
04.06.2008	56	254	276	64	38	140	741,76
04.06.2008	57	27	116	64	38	140	779,37

04.06.2008	63	115	203	64	38	140	30,40
04.06.2008	50	20	108	65	24	123	74,34
04.06.2008	51	22	116	65	24	123	63,35
04.06.2008	56	254	276	65	24	123	46,19
04.06.2008	57	27	116	65	24	123	14,22
04.06.2008	63	115	203	65	24	123	785,53
04.06.2008	63	115	203	70	25	123	766,67
04.06.2008	50	20	108	70	25	123	170,05
04.06.2008	51	22	116	70	25	123	160,04
04.06.2008	56	254	276	70	25	123	140,58
04.06.2008	57	27	116	70	25	123	85,27
04.06.2008	63	115	203	64	38	140	17,45
04.06.2008	63	115	203	65	24	123	802,21
04.06.2008	65	24	123	70	25	123	97,83
04.06.2008	50	20	108	51	22	116	33,47
04.06.2008	50	20	108	56	254	276	63,07
04.06.2008	51	22	116	56	254	276	87,15
04.06.2008	50	20	108	57	27	116	101,22
04.06.2008	51	22	116	57	27	116	69,92
04.06.2008	56	254	276	57	27	116	156,41
04.06.2008	50	20	108	63	115	203	699,46
04.06.2008	51	22	116	63	115	203	732,90
04.06.2008	56	254	276	63	115	203	661,64
04.06.2008	57	27	116	63	115	203	797,90
04.06.2008	50	20	108	64	38	140	727,93
04.06.2008	51	22	116	64	38	140	761,34
04.06.2008	56	254	276	64	38	140	689,26
04.06.2008	57	27	116	64	38	140	826,67
04.06.2008	63	115	203	70	25	123	762,48
04.06.2008	50	20	108	65	24	123	87,04
04.06.2008	51	22	116	65	24	123	54,71
04.06.2008	56	254	276	65	24	123	140,43
04.06.2008	57	27	116	65	24	123	16,61

04.06.2008	63	115	203	64	38	140	164,67
04.06.2008	63	115	203	65	24	123	726,17
04.06.2008	50	20	108	70	25	123	71,89
04.06.2008	51	22	116	70	25	123	41,73
04.06.2008	56	254	276	70	25	123	108,70
04.06.2008	57	27	116	70	25	123	60,59
04.06.2008	63	115	203	70	25	123	699,53
05.06.2008	63	115	203	64	38	140	168,44
04.06.2008	65	24	123	70	25	123	44,82
04.06.2008	50	20	108	51	22	116	56,20
04.06.2008	50	20	108	56	254	276	45,40
04.06.2008	51	22	116	56	254	276	82,55
04.06.2008	50	20	108	57	27	116	119,01
04.06.2008	51	22	116	57	27	116	83,39
04.06.2008	56	254	276	57	27	116	159,15
04.06.2008	50	20	108	63	115	203	705,66
04.06.2008	51	22	116	63	115	203	758,88
04.06.2008	56	254	276	63	115	203	678,87
04.06.2008	57	27	116	63	115	203	818,68
04.06.2008	50	20	108	64	38	140	723,03
04.06.2008	51	22	116	64	38	140	776,28
04.06.2008	56	254	276	64	38	140	696,30
04.06.2008	57	27	116	64	38	140	835,93
05.06.2008	63	115	203	65	24	123	745,97
04.06.2008	50	20	108	65	24	123	101,65
04.06.2008	51	22	116	65	24	123	67,87
04.06.2008	56	254	276	65	24	123	141,95
04.06.2008	57	27	116	65	24	123	17,36
05.06.2008	63	115	203	70	25	123	692,66
05.06.2008	63	115	203	64	38	140	37,18
04.06.2008	50	20	108	70	25	123	58,65
04.06.2008	51	22	116	70	25	123	5,13
04.06.2008	56	254	276	70	25	123	86,93

04.06.2008	57	27	116	70	25	123	78,26
05.06.2008	63	115	203	65	24	123	766,16
05.06.2008	63	115	203	70	25	123	789,23
04.06.2008	65	24	123	70	25	123	62,78
04.06.2008	50	20	108	51	22	116	20,61
04.06.2008	50	20	108	56	254	276	64,36
04.06.2008	51	22	116	56	254	276	59,72
04.06.2008	50	20	108	57	27	116	117,72
04.06.2008	51	22	116	57	27	116	99,18
04.06.2008	56	254	276	57	27	116	84,52
04.06.2008	50	20	108	63	115	203	633,81
04.06.2008	51	22	116	63	115	203	648,58
04.06.2008	56	254	276	63	115	203	693,52
04.06.2008	57	27	116	63	115	203	744,75
04.06.2008	50	20	108	64	38	140	797,86
04.06.2008	51	22	116	64	38	140	812,88
04.06.2008	56	254	276	64	38	140	857,00
04.06.2008	57	27	116	64	38	140	909,31
05.06.2008	63	115	203	64	38	140	47,47
04.06.2008	50	20	108	65	24	123	94,07
04.06.2008	51	22	116	65	24	123	77,63
04.06.2008	56	254	276	65	24	123	55,13
04.06.2008	57	27	116	65	24	123	29,39
05.06.2008	63	115	203	65	24	123	785,10
05.06.2008	63	115	203	70	25	123	860,83
04.06.2008	50	20	108	70	25	123	70,48
04.06.2008	51	22	116	70	25	123	65,31
04.06.2008	56	254	276	70	25	123	6,18
04.06.2008	57	27	116	70	25	123	82,47
05.06.2008	63	115	203	64	38	140	124,84
05.06.2008	63	115	203	65	24	123	782,59
04.06.2008	65	24	123	70	25	123	53,20
05.06.2008	50	20	108	51	22	116	15,23

05.06.2008	50	20	108	56	254	276	75,26
05.06.2008	51	22	116	56	254	276	70,16
05.06.2008	50	20	108	57	27	116	107,74
05.06.2008	51	22	116	57	27	116	95,40
05.06.2008	56	254	276	57	27	116	58,64
05.06.2008	50	20	108	63	115	203	626,43
05.06.2008	51	22	116	63	115	203	636,87
05.06.2008	56	254	276	63	115	203	698,52
05.06.2008	57	27	116	63	115	203	731,52
05.06.2008	50	20	108	64	38	140	791,91
05.06.2008	51	22	116	64	38	140	802,82
05.06.2008	56	254	276	64	38	140	862,93
05.06.2008	57	27	116	64	38	140	897,84
05.06.2008	63	115	203	70	25	123	782,97
05.06.2008	50	20	108	65	24	123	122,94
05.06.2008	51	22	116	65	24	123	110,39
05.06.2008	56	254	276	65	24	123	71,81
05.06.2008	57	27	116	65	24	123	15,31
05.06.2008	63	115	203	65	24	123	769,93
05.06.2008	63	115	203	70	25	123	706,25
05.06.2008	50	20	108	70	25	123	67,46
05.06.2008	51	22	116	70	25	123	60,98
05.06.2008	56	254	276	70	25	123	10,89
05.06.2008	57	27	116	70	25	123	54,97
05.06.2008	63	115	203	64	38	140	172,63
05.06.2008	63	115	203	65	24	123	780,87
05.06.2008	65	24	123	70	25	123	69,24
05.06.2008	50	20	108	51	22	116	21,23
05.06.2008	50	20	108	56	254	276	64,45
05.06.2008	51	22	116	56	254	276	85,35
05.06.2008	50	20	108	57	27	116	108,09
05.06.2008	51	22	116	57	27	116	92,16
05.06.2008	56	254	276	57	27	116	168,45

05.06.2008	50	20	108	63	115	203	683,17
05.06.2008	51	22	116	63	115	203	695,47
05.06.2008	56	254	276	63	115	203	638,08
05.06.2008	57	27	116	63	115	203	786,77
05.06.2008	50	20	108	64	38	140	717,60
05.06.2008	51	22	116	64	38	140	730,23
05.06.2008	56	254	276	64	38	140	671,49
05.06.2008	57	27	116	64	38	140	821,74
05.06.2008	63	115	203	70	25	123	724,82
05.06.2008	50	20	108	65	24	123	84,37
05.06.2008	51	22	116	65	24	123	70,75
05.06.2008	56	254	276	65	24	123	142,94
05.06.2008	57	27	116	65	24	123	26,28
05.06.2008	63	115	203	64	38	140	201,57
05.06.2008	63	115	203	65	24	123	771,90
05.06.2008	50	20	108	70	25	123	112,93
05.06.2008	51	22	116	70	25	123	95,98
05.06.2008	56	254	276	70	25	123	174,36
05.06.2008	57	27	116	70	25	123	8,85
05.06.2008	63	115	203	70	25	123	717,58
05.06.2008	63	115	203	64	38	140	125,41
05.06.2008	65	24	123	70	25	123	33,81
05.06.2008	50	20	108	51	22	116	68,69
05.06.2008	50	20	108	56	254	276	82,48
05.06.2008	51	22	116	56	254	276	120,69
05.06.2008	50	20	108	57	27	116	120,02
05.06.2008	51	22	116	57	27	116	76,23
05.06.2008	56	254	276	57	27	116	191,72
05.06.2008	50	20	108	63	115	203	694,58
05.06.2008	51	22	116	63	115	203	758,13
05.06.2008	56	254	276	63	115	203	645,92
05.06.2008	57	27	116	63	115	203	812,06
05.06.2008	50	20	108	64	38	140	739,90

05.06.2008	51	22	116	64	38	140	802,92
05.06.2008	56	254	276	64	38	140	689,69
05.06.2008	57	27	116	64	38	140	857,79
05.06.2008	63	115	203	65	24	123	723,86
05.06.2008	50	20	108	65	24	123	90,91
05.06.2008	51	22	116	65	24	123	45,41
05.06.2008	56	254	276	65	24	123	159,99
05.06.2008	57	27	116	65	24	123	31,89
05.06.2008	63	115	203	70	25	123	703,19
06.06.2008	63	115	203	51	22	116	689,40
05.06.2008	50	20	108	70	25	123	172,53
05.06.2008	51	22	116	70	25	123	128,49
05.06.2008	56	254	276	70	25	123	245,43
05.06.2008	57	27	116	70	25	123	53,72
06.06.2008	63	115	203	56	254	276	548,07
06.06.2008	63	115	203	57	27	116	760,13
05.06.2008	65	24	123	70	25	123	85,49
05.06.2008	50	20	108	51	22	116	73,01
05.06.2008	50	20	108	56	254	276	68,55
05.06.2008	51	22	116	56	254	276	117,43
05.06.2008	50	20	108	57	27	116	112,11
05.06.2008	51	22	116	57	27	116	61,21
05.06.2008	56	254	276	57	27	116	171,52
05.06.2008	50	20	108	63	115	203	680,54
05.06.2008	51	22	116	63	115	203	750,64
05.06.2008	56	254	276	63	115	203	639,50
05.06.2008	57	27	116	63	115	203	790,15
05.06.2008	50	20	108	64	38	140	804,23
05.06.2008	51	22	116	64	38	140	873,82
05.06.2008	56	254	276	64	38	140	761,56
05.06.2008	57	27	116	64	38	140	914,27
06.06.2008	63	115	203	64	38	140	98,62
05.06.2008	50	20	108	65	24	123	103,38

05.06.2008	51	22	116	65	24	123	50,61
05.06.2008	56	254	276	65	24	123	161,50
05.06.2008	57	27	116	65	24	123	10,63
06.06.2008	63	115	203	65	24	123	786,09
06.06.2008	63	115	203	70	25	123	725,41
05.06.2008	50	20	108	70	25	123	103,87
05.06.2008	51	22	116	70	25	123	51,44
05.06.2008	56	254	276	70	25	123	162,17
05.06.2008	57	27	116	70	25	123	9,82
06.06.2008	63	115	203	64	38	140	45,73
06.06.2008	63	115	203	65	24	123	795,82
05.06.2008	65	24	123	70	25	123	0,85
05.06.2008	50	20	108	51	22	116	32,58
05.06.2008	50	20	108	56	254	276	110,96
05.06.2008	51	22	116	56	254	276	143,53
06.06.2008	63	115	203	70	25	123	854,01
05.06.2008	50	20	108	57	27	116	112,90
05.06.2008	51	22	116	57	27	116	82,05
05.06.2008	56	254	276	57	27	116	222,01
06.06.2008	63	115	203	64	38	140	33,87
05.06.2008	50	20	108	63	115	203	667,98
05.06.2008	51	22	116	63	115	203	695,99
05.06.2008	56	254	276	63	115	203	574,90
05.06.2008	57	27	116	63	115	203	777,34
06.06.2008	63	115	203	65	24	123	796,65
05.06.2008	50	20	108	64	38	140	870,10
05.06.2008	51	22	116	64	38	140	897,64
05.06.2008	50	20	108	65	24	123	104,86
05.06.2008	51	22	116	65	24	123	74,29
05.06.2008	56	254	276	65	24	123	213,78
05.06.2008	57	27	116	65	24	123	8,28
06.06.2008	63	115	203	70	25	123	824,71
11.06.2008	63	115	203	64	38	140	13,31

05.06.2008	50	20	108	70	25	123	39,27
05.06.2008	51	22	116	70	25	123	13,09
05.06.2008	56	254	276	70	25	123	148,95
05.06.2008	57	27	116	70	25	123	73,69
11.06.2008	63	115	203	65	24	123	968,02
11.06.2008	63	115	203	70	25	123	956,01
05.06.2008	65	24	123	70	25	123	65,61
05.06.2008	50	20	108	51	22	116	24,02
05.06.2008	50	20	108	56	254	276	111,08
05.06.2008	51	22	116	56	254	276	133,42
05.06.2008	50	20	108	57	27	116	105,46
05.06.2008	51	22	116	57	27	116	81,50
05.06.2008	56	254	276	57	27	116	210,47
05.06.2008	50	20	108	63	115	203	693,75
05.06.2008	51	22	116	63	115	203	717,64
05.06.2008	56	254	276	63	115	203	599,96
05.06.2008	57	27	116	63	115	203	799,12
05.06.2008	50	20	108	64	38	140	858,14
05.06.2008	51	22	116	64	38	140	882,15
05.06.2008	56	254	276	64	38	140	759,81
05.06.2008	57	27	116	64	38	140	963,59
11.06.2008	63	115	203	64	38	140	180,84
05.06.2008	50	20	108	65	24	123	87,94
05.06.2008	51	22	116	65	24	123	63,94
05.06.2008	56	254	276	65	24	123	195,56
05.06.2008	57	27	116	65	24	123	19,96
11.06.2008	63	115	203	65	24	123	858,83
11.06.2008	63	115	203	70	25	123	767,13
05.06.2008	50	20	108	70	25	123	35,14
05.06.2008	51	22	116	70	25	123	20,56
05.06.2008	56	254	276	70	25	123	132,91
05.06.2008	57	27	116	70	25	123	77,56
11.06.2008	63	115	203	64	38	140	45,46

11.06.2008	63	115	203	65	24	123	962,48
05.06.2008	65	24	123	70	25	123	63,42
05.06.2008	50	20	108	51	22	116	26,01
05.06.2008	50	20	108	56	254	276	117,74
05.06.2008	51	22	116	56	254	276	142,97
05.06.2008	50	20	108	57	27	116	101,96
05.06.2008	51	22	116	57	27	116	75,99
05.06.2008	56	254	276	57	27	116	216,93
05.06.2008	50	20	108	63	115	203	681,99
05.06.2008	51	22	116	63	115	203	707,17
05.06.2008	56	254	276	63	115	203	583,35
05.06.2008	57	27	116	63	115	203	781,99
05.06.2008	50	20	108	64	38	140	883,40
05.06.2008	51	22	116	64	38	140	908,64
05.06.2008	56	254	276	64	38	140	783,31
05.06.2008	57	27	116	64	38	140	983,54
11.06.2008	63	115	203	70	25	123	902,42
05.06.2008	50	20	108	65	24	123	91,42
05.06.2008	51	22	116	65	24	123	65,49
05.06.2008	56	254	276	65	24	123	206,29
05.06.2008	57	27	116	65	24	123	10,65
11.06.2008	63	115	203	64	38	140	57,99
11.06.2008	63	115	203	65	24	123	1019,66
05.06.2008	50	20	108	70	25	123	35,66
05.06.2008	51	22	116	70	25	123	11,37
05.06.2008	56	254	276	70	25	123	150,42
05.06.2008	57	27	116	70	25	123	66,91
11.06.2008	63	115	203	70	25	123	992,99
11.06.2008	63	115	203	64	38	140	170,98
05.06.2008	65	24	123	70	25	123	56,28
05.06.2008	50	20	108	51	22	116	32,63
05.06.2008	50	20	108	56	254	276	163,44
05.06.2008	51	22	116	56	254	276	178,33

05.06.2008	50	20	108	57	27	116	101,28
05.06.2008	51	22	116	57	27	116	82,34
05.06.2008	56	254	276	57	27	116	260,44
05.06.2008	50	20	108	63	115	203	647,55
05.06.2008	51	22	116	63	115	203	674,12
05.06.2008	56	254	276	63	115	203	515,35
05.06.2008	57	27	116	63	115	203	747,43
05.06.2008	50	20	108	64	38	140	769,83
05.06.2008	51	22	116	64	38	140	795,60
05.06.2008	56	254	276	64	38	140	632,30
05.06.2008	57	27	116	64	38	140	870,24
11.06.2008	63	115	203	65	24	123	828,54
05.06.2008	50	20	108	65	24	123	76,37
05.06.2008	51	22	116	65	24	123	54,89
05.06.2008	56	254	276	65	24	123	233,18
05.06.2008	57	27	116	65	24	123	27,63
11.06.2008	63	115	203	70	25	123	800,45
11.06.2008	63	115	203	64	38	140	88,37
05.06.2008	50	20	108	70	25	123	64,33
05.06.2008	51	22	116	70	25	123	31,93
05.06.2008	56	254	276	70	25	123	200,95
05.06.2008	57	27	116	70	25	123	68,44
11.06.2008	63	115	203	65	24	123	893,49
11.06.2008	63	115	203	70	25	123	884,60
05.06.2008	65	24	123	70	25	123	42,74
06.06.2008	50	20	108	51	22	116	36,99
12.06.2008	63	115	203	64	38	140	28,75
06.06.2008	50	20	108	56	254	276	131,15
06.06.2008	51	22	116	56	254	276	167,97
12.06.2008	63	115	203	65	24	123	862,92
06.06.2008	50	20	108	57	27	116	104,02
06.06.2008	51	22	116	57	27	116	70,76
06.06.2008	56	254	276	57	27	116	233,25

12.06.2008	63	115	203	70	25	123	848,52
12.06.2008	63	115	203	64	38	140	41,12
06.06.2008	50	20	108	64	38	140	755,45
06.06.2008	51	22	116	64	38	140	786,46
06.06.2008	56	254	276	64	38	140	641,94
06.06.2008	57	27	116	64	38	140	857,22
12.06.2008	63	115	203	65	24	123	943,06
06.06.2008	50	20	108	65	24	123	132,38
06.06.2008	51	22	116	65	24	123	97,81
06.06.2008	56	254	276	65	24	123	262,22
06.06.2008	57	27	116	65	24	123	29,15
12.06.2008	63	115	203	70	25	123	930,83
12.06.2008	63	115	203	64	38	140	35,05
06.06.2008	50	20	108	70	25	123	69,24
06.06.2008	51	22	116	70	25	123	55,13
06.06.2008	56	254	276	70	25	123	185,96
06.06.2008	57	27	116	70	25	123	57,60
12.06.2008	63	115	203	64	38	140	36,74
12.06.2008	63	115	203	65	24	123	940,96
06.06.2008	65	24	123	70	25	123	85,10
06.06.2008	50	20	108	51	22	116	33,79
06.06.2008	50	20	108	56	254	276	59,44
06.06.2008	51	22	116	56	254	276	85,79
06.06.2008	50	20	108	57	27	116	111,44
06.06.2008	51	22	116	57	27	116	77,86
06.06.2008	56	254	276	57	27	116	160,26
06.06.2008	50	20	108	63	115	203	696,94
06.06.2008	51	22	116	63	115	203	730,46
06.06.2008	56	254	276	63	115	203	665,00
06.06.2008	57	27	116	63	115	203	806,04
06.06.2008	50	20	108	64	38	140	740,55
06.06.2008	51	22	116	64	38	140	774,15
06.06.2008	56	254	276	64	38	140	707,53

06.06.2008	57	27	116	64	38	140	850,02
12.06.2008	63	115	203	65	24	123	864,80
06.06.2008	50	20	108	65	24	123	100,64
06.06.2008	51	22	116	65	24	123	66,98
06.06.2008	56	254	276	65	24	123	149,25
06.06.2008	57	27	116	65	24	123	11,03
12.06.2008	63	115	203	70	25	123	997,99
12.06.2008	63	115	203	70	25	123	1020,57
06.06.2008	50	20	108	70	25	123	168,26
06.06.2008	51	22	116	70	25	123	136,09
06.06.2008	56	254	276	70	25	123	220,90
06.06.2008	57	27	116	70	25	123	62,28
12.06.2008	63	115	203	64	38	140	188,04
12.06.2008	63	115	203	65	24	123	780,94
06.06.2008	65	24	123	70	25	123	72,80
06.06.2008	50	20	108	51	22	116	30,30
06.06.2008	50	20	108	56	254	276	41,39
06.06.2008	51	22	116	56	254	276	69,56
06.06.2008	50	20	108	57	27	116	103,50
06.06.2008	51	22	116	57	27	116	75,70
06.06.2008	56	254	276	57	27	116	134,68
06.06.2008	50	20	108	63	115	203	685,00
06.06.2008	51	22	116	63	115	203	710,59
06.06.2008	56	254	276	63	115	203	666,19
06.06.2008	57	27	116	63	115	203	786,11
06.06.2008	50	20	108	64	38	140	718,58
06.06.2008	51	22	116	64	38	140	744,26
06.06.2008	56	254	276	64	38	140	699,49
06.06.2008	57	27	116	64	38	140	819,80
12.06.2008	63	115	203	70	25	123	793,26
06.06.2008	50	20	108	65	24	123	116,38
06.06.2008	51	22	116	65	24	123	87,53
06.06.2008	56	254	276	65	24	123	149,08

06.06.2008	57	27	116	65	24	123	15,48
12.06.2008	63	115	203	64	38	140	190,94
12.06.2008	63	115	203	65	24	123	779,29
06.06.2008	50	20	108	70	25	123	149,61
06.06.2008	51	22	116	70	25	123	119,89
06.06.2008	56	254	276	70	25	123	183,89
06.06.2008	57	27	116	70	25	123	50,83
12.06.2008	63	115	203	70	25	123	713,09
12.06.2008	63	115	203	64	38	140	164,16
06.06.2008	65	24	123	70	25	123	35,46
11.06.2008	50	20	108	51	22	116	27,40
11.06.2008	50	20	108	56	254	276	18,89
11.06.2008	51	22	116	56	254	276	38,80
11.06.2008	50	20	108	57	27	116	93,35
11.06.2008	51	22	116	57	27	116	66,01
11.06.2008	56	254	276	57	27	116	101,26
11.06.2008	50	20	108	63	115	203	878,38
11.06.2008	51	22	116	63	115	203	905,72
11.06.2008	56	254	276	63	115	203	870,18
11.06.2008	57	27	116	63	115	203	971,16
11.06.2008	50	20	108	64	38	140	865,07
11.06.2008	51	22	116	64	38	140	892,41
11.06.2008	56	254	276	64	38	140	856,87
11.06.2008	57	27	116	64	38	140	957,84
12.06.2008	63	115	203	65	24	123	803,95
11.06.2008	50	20	108	65	24	123	90,72
11.06.2008	51	22	116	65	24	123	64,32
11.06.2008	56	254	276	65	24	123	102,86
11.06.2008	57	27	116	65	24	123	25,75
12.06.2008	63	115	203	70	25	123	727,12
12.06.2008	63	115	203	64	38	140	202,31
11.06.2008	50	20	108	70	25	123	77,76
11.06.2008	51	22	116	70	25	123	50,70

11.06.2008	56	254	276	70	25	123	88,68
11.06.2008	57	27	116	70	25	123	21,78
12.06.2008	63	115	203	65	24	123	764,56
12.06.2008	63	115	203	70	25	123	752,89
11.06.2008	65	24	123	70	25	123	15,46
11.06.2008	50	20	108	51	22	116	49,91
11.06.2008	50	20	108	56	254	276	29,03
11.06.2008	51	22	116	56	254	276	62,83
11.06.2008	50	20	108	57	27	116	82,24
11.06.2008	51	22	116	57	27	116	63,58
11.06.2008	56	254	276	57	27	116	108,88
11.06.2008	50	20	108	63	115	203	704,15
11.06.2008	51	22	116	63	115	203	741,16
11.06.2008	56	254	276	63	115	203	680,36
11.06.2008	57	27	116	63	115	203	785,82
11.06.2008	50	20	108	64	38	140	881,61
11.06.2008	51	22	116	64	38	140	916,97
11.06.2008	56	254	276	64	38	140	857,06
11.06.2008	57	27	116	64	38	140	963,61
12.06.2008	63	115	203	64	38	140	20,51
11.06.2008	50	20	108	65	24	123	187,17
11.06.2008	51	22	116	65	24	123	137,53
11.06.2008	56	254	276	65	24	123	197,79
11.06.2008	57	27	116	65	24	123	142,13
12.06.2008	63	115	203	65	24	123	765,96
12.06.2008	63	115	203	70	25	123	764,16
11.06.2008	50	20	108	70	25	123	63,01
11.06.2008	51	22	116	70	25	123	45,15
11.06.2008	56	254	276	70	25	123	88,88
11.06.2008	57	27	116	70	25	123	20,34
13.06.2008	63	115	203	64	38	140	134,98
13.06.2008	63	115	203	65	24	123	801,25
11.06.2008	65	24	123	70	25	123	144,86

11.06.2008	50	20	108	51	22	116	162,66
11.06.2008	50	20	108	56	254	276	1,14
11.06.2008	51	22	116	56	254	276	163,59
11.06.2008	50	20	108	57	27	116	226,93
11.06.2008	51	22	116	57	27	116	72,15
11.06.2008	56	254	276	57	27	116	227,74
11.06.2008	50	20	108	63	115	203	771,67
11.06.2008	51	22	116	63	115	203	899,50
11.06.2008	56	254	276	63	115	203	771,42
11.06.2008	57	27	116	63	115	203	971,59
11.06.2008	50	20	108	64	38	140	753,84
11.06.2008	51	22	116	64	38	140	886,72
11.06.2008	56	254	276	64	38	140	753,53
11.06.2008	57	27	116	64	38	140	958,87
13.06.2008	63	115	203	70	25	123	785,76
11.06.2008	50	20	108	65	24	123	214,34
11.06.2008	51	22	116	65	24	123	63,36
11.06.2008	56	254	276	65	24	123	215,13
11.06.2008	57	27	116	65	24	123	13,81
13.06.2008	63	115	203	64	38	140	27,56
13.06.2008	63	115	203	65	24	123	823,51
11.06.2008	50	20	108	70	25	123	163,93
11.06.2008	51	22	116	70	25	123	3,54
11.06.2008	56	254	276	70	25	123	164,86
11.06.2008	57	27	116	70	25	123	69,36
13.06.2008	63	115	203	70	25	123	787,49
18.06.2008	63	115	203	64	38	140	184,43
11.06.2008	65	24	123	70	25	123	60,26
11.06.2008	50	20	108	51	22	116	34,34
11.06.2008	50	20	108	56	254	276	27,26
11.06.2008	51	22	116	56	254	276	42,17
11.06.2008	50	20	108	57	27	116	109,96
11.06.2008	51	22	116	57	27	116	77,44

11.06.2008	56	254	276	57	27	116	118,33
11.06.2008	50	20	108	63	115	203	937,22
11.06.2008	51	22	116	63	115	203	965,11
11.06.2008	56	254	276	63	115	203	923,10
11.06.2008	57	27	116	63	115	203	1040,82
11.06.2008	50	20	108	64	38	140	881,47
11.06.2008	51	22	116	64	38	140	909,02
11.06.2008	56	254	276	64	38	140	866,96
11.06.2008	57	27	116	64	38	140	984,49
18.06.2008	63	115	203	65	24	123	778,04
11.06.2008	50	20	108	65	24	123	83,10
11.06.2008	51	22	116	65	24	123	62,98
11.06.2008	56	254	276	65	24	123	102,59
11.06.2008	57	27	116	65	24	123	53,75
18.06.2008	63	115	203	70	25	123	712,94
18.06.2008	63	115	203	64	38	140	197,21
11.06.2008	50	20	108	70	25	123	59,70
11.06.2008	51	22	116	70	25	123	27,90
11.06.2008	56	254	276	70	25	123	69,95
11.06.2008	57	27	116	70	25	123	50,27
18.06.2008	63	115	203	65	24	123	778,04
18.06.2008	63	115	203	70	25	123	717,91
11.06.2008	65	24	123	70	25	123	42,37
11.06.2008	50	20	108	51	22	116	102,14
11.06.2008	50	20	108	57	27	116	192,03
11.06.2008	51	22	116	57	27	116	98,11
11.06.2008	50	20	108	63	115	203	707,13
11.06.2008	51	22	116	63	115	203	774,51
11.06.2008	57	27	116	63	115	203	870,43
11.06.2008	50	20	108	64	38	140	537,54
11.06.2008	51	22	116	64	38	140	603,53
11.06.2008	57	27	116	64	38	140	699,48
18.06.2008	63	115	203	64	38	140	191,07

11.06.2008	50	20	108	65	24	123	136,29
11.06.2008	51	22	116	65	24	123	57,05
11.06.2008	57	27	116	65	24	123	58,42
18.06.2008	63	115	203	65	24	123	771,64
18.06.2008	63	115	203	70	25	123	706,60
11.06.2008	50	20	108	70	25	123	131,53
11.06.2008	51	22	116	70	25	123	30,15
11.06.2008	57	27	116	70	25	123	70,14
18.06.2008	63	115	203	64	38	140	189,53
18.06.2008	63	115	203	65	24	123	774,10
11.06.2008	65	24	123	70	25	123	44,49
11.06.2008	50	20	108	51	22	116	68,56
11.06.2008	50	20	108	56	254	276	2,51
11.06.2008	51	22	116	56	254	276	67,88
11.06.2008	50	20	108	57	27	116	124,16
11.06.2008	51	22	116	57	27	116	55,66
11.06.2008	56	254	276	57	27	116	123,39
11.06.2008	50	20	108	63	115	203	797,06
11.06.2008	51	22	116	63	115	203	864,64
11.06.2008	56	254	276	63	115	203	797,35
11.06.2008	57	27	116	63	115	203	918,96
11.06.2008	50	20	108	64	38	140	884,09
11.06.2008	51	22	116	64	38	140	951,45
11.06.2008	56	254	276	64	38	140	884,33
11.06.2008	57	27	116	64	38	140	1005,53
18.06.2008	63	115	203	70	25	123	729,17
11.06.2008	50	20	108	65	24	123	99,87
11.06.2008	51	22	116	65	24	123	48,71
11.06.2008	56	254	276	65	24	123	100,31
11.06.2008	57	27	116	65	24	123	58,65
18.06.2008	63	115	203	64	38	140	190,46
18.06.2008	63	115	203	65	24	123	774,94
11.06.2008	50	20	108	70	25	123	89,89

11.06.2008	51	22	116	70	25	123	38,92
11.06.2008	56	254	276	70	25	123	90,24
11.06.2008	57	27	116	70	25	123	58,02
18.06.2008	63	115	203	70	25	123	726,74
18.06.2008	63	115	203	64	38	140	211,51
11.06.2008	65	24	123	70	25	123	10,58
12.06.2008	50	20	108	51	22	116	113,28
12.06.2008	50	20	108	56	254	276	72,13
12.06.2008	51	22	116	56	254	276	168,95
12.06.2008	50	20	108	57	27	116	205,44
12.06.2008	51	22	116	57	27	116	93,02
12.06.2008	56	254	276	57	27	116	254,01
12.06.2008	50	20	108	63	115	203	693,32
12.06.2008	51	22	116	63	115	203	794,37
12.06.2008	56	254	276	63	115	203	683,11
12.06.2008	57	27	116	63	115	203	884,77
12.06.2008	50	20	108	64	38	140	685,93
12.06.2008	51	22	116	64	38	140	788,84
12.06.2008	56	254	276	64	38	140	672,87
12.06.2008	57	27	116	64	38	140	879,93
18.06.2008	63	115	203	65	24	123	749,01
12.06.2008	50	20	108	65	24	123	175,08
12.06.2008	51	22	116	65	24	123	69,37
12.06.2008	56	254	276	65	24	123	217,70
12.06.2008	57	27	116	65	24	123	40,47
18.06.2008	63	115	203	70	25	123	709,03
19.06.2008	63	115	203	64	38	140	181,55
12.06.2008	50	20	108	70	25	123	161,11
12.06.2008	51	22	116	70	25	123	55,15
12.06.2008	56	254	276	70	25	123	205,42
12.06.2008	57	27	116	70	25	123	49,46
19.06.2008	63	115	203	65	24	123	779,36
19.06.2008	63	115	203	70	25	123	741,37

12.06.2008	65	24	123	70	25	123	14,40
12.06.2008	50	20	108	51	22	116	74,70
12.06.2008	50	20	108	56	254	276	16,22
12.06.2008	51	22	116	56	254	276	86,11
12.06.2008	50	20	108	57	27	116	113,43
12.06.2008	51	22	116	57	27	116	56,74
12.06.2008	56	254	276	57	27	116	118,52
12.06.2008	50	20	108	63	115	203	852,17
12.06.2008	51	22	116	63	115	203	924,97
12.06.2008	56	254	276	63	115	203	844,94
12.06.2008	57	27	116	63	115	203	963,21
12.06.2008	50	20	108	64	38	140	893,00
12.06.2008	51	22	116	64	38	140	965,70
12.06.2008	56	254	276	64	38	140	885,84
12.06.2008	57	27	116	64	38	140	1004,15
19.06.2008	63	115	203	64	38	140	182,67
12.06.2008	50	20	108	65	24	123	92,60
12.06.2008	51	22	116	65	24	123	18,12
12.06.2008	56	254	276	65	24	123	103,49
12.06.2008	57	27	116	65	24	123	47,63
19.06.2008	63	115	203	65	24	123	778,07
19.06.2008	63	115	203	70	25	123	874,50
12.06.2008	50	20	108	70	25	123	79,46
12.06.2008	51	22	116	70	25	123	8,18
12.06.2008	56	254	276	70	25	123	89,87
12.06.2008	57	27	116	70	25	123	48,59
19.06.2008	63	115	203	64	38	140	191,64
19.06.2008	63	115	203	65	24	123	782,09
12.06.2008	65	24	123	70	25	123	13,90
12.06.2008	50	20	108	51	22	116	81,40
12.06.2008	50	20	108	51	22	116	168,77
12.06.2008	50	20	108	56	254	276	610,48
12.06.2008	50	20	108	56	254	276	475,96

12.06.2008	51	22	116	56	254	276	675,29
12.06.2008	51	22	116	56	254	276	631,13
12.06.2008	50	20	108	57	27	116	113,27
12.06.2008	50	20	108	57	27	116	239,60
12.06.2008	51	22	116	57	27	116	33,71
12.06.2008	51	22	116	57	27	116	72,70
12.06.2008	56	254	276	57	27	116	708,24
12.06.2008	56	254	276	57	27	116	703,64
12.06.2008	50	20	108	63	115	203	772,99
12.06.2008	50	20	108	63	115	203	704,57
12.06.2008	51	22	116	63	115	203	839,99
12.06.2008	51	22	116	63	115	203	861,82
12.06.2008	56	254	276	63	115	203	166,99
12.06.2008	56	254	276	63	115	203	230,91
12.06.2008	57	27	116	63	115	203	873,17
12.06.2008	57	27	116	63	115	203	934,39
12.06.2008	50	20	108	64	38	140	806,82
12.06.2008	50	20	108	64	38	140	740,96
12.06.2008	51	22	116	64	38	140	873,24
12.06.2008	51	22	116	64	38	140	897,71
12.06.2008	56	254	276	64	38	140	198,78
12.06.2008	56	254	276	64	38	140	266,60
12.06.2008	57	27	116	64	38	140	906,34
12.06.2008	57	27	116	64	38	140	970,23
19.06.2008	63	115	203	70	25	123	876,09
19.06.2008	63	115	203	64	38	140	187,81
12.06.2008	50	20	108	65	24	123	179,45
12.06.2008	50	20	108	65	24	123	171,34
12.06.2008	51	22	116	65	24	123	102,05
12.06.2008	51	22	116	65	24	123	3,06
12.06.2008	56	254	276	65	24	123	775,73
12.06.2008	56	254	276	65	24	123	634,11
12.06.2008	57	27	116	65	24	123	68,36

12.06.2008	57	27	116	65	24	123	69,79
19.06.2008	63	115	203	65	24	123	779,93
19.06.2008	63	115	203	70	25	123	717,96
19.06.2008	63	115	203	64	38	140	189,77
19.06.2008	63	115	203	65	24	123	781,13
12.06.2008	50	20	108	70	25	123	245,17
12.06.2008	50	20	108	70	25	123	332,63
12.06.2008	51	22	116	70	25	123	165,44
12.06.2008	51	22	116	70	25	123	163,99
12.06.2008	56	254	276	70	25	123	831,90
12.06.2008	56	254	276	70	25	123	789,66
12.06.2008	57	27	116	70	25	123	132,18
12.06.2008	57	27	116	70	25	123	94,22
19.06.2008	63	115	203	70	25	123	713,77
19.06.2008	63	115	203	64	38	140	189,37
19.06.2008	63	115	203	65	24	123	782,74
19.06.2008	63	115	203	70	25	123	731,52
12.06.2008	65	24	123	70	25	123	67,30
12.06.2008	65	24	123	70	25	123	161,35
12.06.2008	50	20	108	51	22	116	102,30
12.06.2008	50	20	108	56	254	276	80,12
12.06.2008	51	22	116	56	254	276	181,65
12.06.2008	50	20	108	57	27	116	128,94
12.06.2008	51	22	116	57	27	116	26,84
12.06.2008	56	254	276	57	27	116	207,92
12.06.2008	50	20	108	63	115	203	597,68
12.06.2008	51	22	116	63	115	203	680,76
12.06.2008	56	254	276	63	115	203	548,57
12.06.2008	57	27	116	63	115	203	705,16
12.06.2008	50	20	108	64	38	140	783,68
12.06.2008	51	22	116	64	38	140	868,38
12.06.2008	56	254	276	64	38	140	731,17
12.06.2008	57	27	116	64	38	140	892,94

19.06.2008	63	115	203	64	38	140	183,56
12.06.2008	50	20	108	65	24	123	202,22
12.06.2008	51	22	116	65	24	123	102,80
12.06.2008	56	254	276	65	24	123	278,77
12.06.2008	57	27	116	65	24	123	76,64
19.06.2008	63	115	203	65	24	123	782,81
19.06.2008	63	115	203	70	25	123	725,70
12.06.2008	50	20	108	70	25	123	213,42
12.06.2008	51	22	116	70	25	123	114,72
12.06.2008	56	254	276	70	25	123	289,39
12.06.2008	57	27	116	70	25	123	88,75
19.06.2008	63	115	203	64	38	140	15,44
19.06.2008	63	115	203	65	24	123	746,40
12.06.2008	65	24	123	70	25	123	12,35
12.06.2008	50	20	108	51	22	116	72,75
12.06.2008	50	20	108	56	254	276	91,72
12.06.2008	51	22	116	56	254	276	164,42
12.06.2008	50	20	108	57	27	116	139,62
12.06.2008	51	22	116	57	27	116	69,97
12.06.2008	56	254	276	57	27	116	230,69
12.06.2008	50	20	108	63	115	203	624,34
12.06.2008	51	22	116	63	115	203	681,65
12.06.2008	56	254	276	63	115	203	554,00
12.06.2008	57	27	116	63	115	203	750,19
12.06.2008	50	20	108	64	38	140	808,67
12.06.2008	51	22	116	64	38	140	868,57
12.06.2008	56	254	276	64	38	140	733,56
12.06.2008	57	27	116	64	38	140	937,68
19.06.2008	63	115	203	70	25	123	714,90
12.06.2008	50	20	108	65	24	123	170,49
12.06.2008	51	22	116	65	24	123	100,61
12.06.2008	56	254	276	65	24	123	261,40
12.06.2008	57	27	116	65	24	123	30,90

20.06.2008	63	115	203	64	38	140	63,24
20.06.2008	63	115	203	65	24	123	719,02
12.06.2008	50	20	108	70	25	123	108,02
12.06.2008	51	22	116	70	25	123	35,49
12.06.2008	56	254	276	70	25	123	199,74
12.06.2008	57	27	116	70	25	123	37,21
20.06.2008	63	115	203	70	25	123	688,78
20.06.2008	63	115	203	64	38	140	53,94
12.06.2008	65	24	123	70	25	123	66,59
12.06.2008	50	20	108	51	22	116	96,21
12.06.2008	50	20	108	56	254	276	74,43
12.06.2008	51	22	116	56	254	276	169,54
12.06.2008	50	20	108	57	27	116	136,30
12.06.2008	51	22	116	57	27	116	43,15
12.06.2008	56	254	276	57	27	116	210,54
12.06.2008	50	20	108	63	115	203	626,56
12.06.2008	51	22	116	63	115	203	703,31
12.06.2008	56	254	276	63	115	203	560,95
12.06.2008	57	27	116	63	115	203	746,00
12.06.2008	50	20	108	64	38	140	789,76
12.06.2008	51	22	116	64	38	140	867,39
12.06.2008	56	254	276	64	38	140	723,03
12.06.2008	57	27	116	64	38	140	910,12
20.06.2008	63	115	203	70	25	123	818,22
12.06.2008	50	20	108	65	24	123	196,56
12.06.2008	51	22	116	65	24	123	103,47
12.06.2008	56	254	276	65	24	123	270,94
12.06.2008	57	27	116	65	24	123	60,70
20.06.2008	63	115	203	64	38	140	55,66
20.06.2008	63	115	203	65	24	123	778,63
12.06.2008	50	20	108	70	25	123	124,20
12.06.2008	51	22	116	70	25	123	28,01
12.06.2008	56	254	276	70	25	123	197,50

12.06.2008	57	27	116	70	25	123	20,84
20.06.2008	63	115	203	70	25	123	835,49
25.06.2008	63	115	203	64	38	140	384,14
12.06.2008	65	24	123	70	25	123	77,32
12.06.2008	50	20	108	51	22	116	69,37
12.06.2008	50	20	108	56	254	276	70,79
12.06.2008	51	22	116	56	254	276	2,13
12.06.2008	50	20	108	57	27	116	119,60
12.06.2008	51	22	116	57	27	116	77,27
12.06.2008	56	254	276	57	27	116	78,34
12.06.2008	50	20	108	63	115	203	660,93
12.06.2008	51	22	116	63	115	203	726,54
12.06.2008	56	254	276	63	115	203	727,37
12.06.2008	57	27	116	63	115	203	775,73
12.06.2008	50	20	108	64	38	140	858,15
12.06.2008	51	22	116	64	38	140	922,25
12.06.2008	56	254	276	64	38	140	922,96
12.06.2008	57	27	116	64	38	140	974,51
25.06.2008	63	115	203	65	24	123	580,46
12.06.2008	50	20	108	65	24	123	106,71
12.06.2008	51	22	116	65	24	123	63,56
12.06.2008	56	254	276	65	24	123	64,71
12.06.2008	57	27	116	65	24	123	14,04
25.06.2008	63	115	203	70	25	123	537,30
25.06.2008	63	115	203	64	38	140	473,28
12.06.2008	50	20	108	70	25	123	94,25
12.06.2008	51	22	116	70	25	123	52,75
12.06.2008	56	254	276	70	25	123	54,10
12.06.2008	57	27	116	70	25	123	26,50
25.06.2008	63	115	203	65	24	123	498,84
25.06.2008	63	115	203	70	25	123	441,02
12.06.2008	65	24	123	70	25	123	12,69
12.06.2008	50	20	108	51	22	116	44,28

12.06.2008	50	20	108	56	254	276	63,21
12.06.2008	51	22	116	56	254	276	19,23
12.06.2008	50	20	108	57	27	116	92,29
12.06.2008	51	22	116	57	27	116	58,68
12.06.2008	56	254	276	57	27	116	45,98
12.06.2008	50	20	108	63	115	203	677,31
12.06.2008	51	22	116	63	115	203	714,01
12.06.2008	56	254	276	63	115	203	731,99
12.06.2008	57	27	116	63	115	203	769,36
12.06.2008	50	20	108	64	38	140	682,66
12.06.2008	51	22	116	64	38	140	718,64
12.06.2008	56	254	276	64	38	140	736,43
12.06.2008	57	27	116	64	38	140	774,49
25.06.2008	63	115	203	64	38	140	387,23
12.06.2008	50	20	108	65	24	123	89,90
12.06.2008	51	22	116	65	24	123	66,64
12.06.2008	56	254	276	65	24	123	59,87
12.06.2008	57	27	116	65	24	123	23,40
25.06.2008	63	115	203	65	24	123	575,69
25.06.2008	63	115	203	70	25	123	514,08
12.06.2008	50	20	108	70	25	123	87,99
12.06.2008	51	22	116	70	25	123	64,69
12.06.2008	56	254	276	70	25	123	58,11
12.06.2008	57	27	116	70	25	123	22,85
25.06.2008	63	115	203	64	38	140	381,83
25.06.2008	63	115	203	65	24	123	581,93
12.06.2008	65	24	123	70	25	123	2,00
13.06.2008	50	20	108	51	22	116	49,10
13.06.2008	50	20	108	56	254	276	57,42
13.06.2008	51	22	116	56	254	276	9,53
13.06.2008	50	20	108	57	27	116	70,59
13.06.2008	51	22	116	57	27	116	57,84
13.06.2008	56	254	276	57	27	116	54,65

13.06.2008	50	20	108	63	115	203	716,83
13.06.2008	51	22	116	63	115	203	752,86
13.06.2008	56	254	276	63	115	203	762,26
13.06.2008	57	27	116	63	115	203	786,41
13.06.2008	50	20	108	64	38	140	847,62
13.06.2008	51	22	116	64	38	140	882,10
13.06.2008	56	254	276	64	38	140	891,41
13.06.2008	57	27	116	64	38	140	917,62
25.06.2008	63	115	203	70	25	123	555,38
13.06.2008	50	20	108	65	24	123	84,97
13.06.2008	51	22	116	65	24	123	54,63
13.06.2008	56	254	276	65	24	123	47,51
13.06.2008	57	27	116	65	24	123	27,12
25.06.2008	63	115	203	64	38	140	370,99
25.06.2008	63	115	203	65	24	123	591,21
13.06.2008	50	20	108	70	25	123	70,16
13.06.2008	51	22	116	70	25	123	58,45
13.06.2008	56	254	276	70	25	123	55,45
13.06.2008	57	27	116	70	25	123	1,40
25.06.2008	63	115	203	70	25	123	549,84
25.06.2008	63	115	203	64	38	140	202,66
13.06.2008	65	24	123	70	25	123	28,52
13.06.2008	50	20	108	51	22	116	35,20
13.06.2008	50	20	108	56	254	276	81,50
13.06.2008	51	22	116	56	254	276	47,89
13.06.2008	50	20	108	57	27	116	112,73
13.06.2008	51	22	116	57	27	116	81,74
13.06.2008	56	254	276	57	27	116	35,89
13.06.2008	50	20	108	63	115	203	720,30
13.06.2008	51	22	116	63	115	203	753,83
13.06.2008	56	254	276	63	115	203	801,52
13.06.2008	57	27	116	63	115	203	832,57
13.06.2008	50	20	108	64	38	140	696,66

13.06.2008	51	22	116	64	38	140	730,39
13.06.2008	56	254	276	64	38	140	777,99
13.06.2008	57	27	116	64	38	140	808,71
25.06.2008	63	115	203	65	24	123	771,26
13.06.2008	50	20	108	65	24	123	105,97
13.06.2008	51	22	116	65	24	123	70,90
13.06.2008	56	254	276	65	24	123	28,54
13.06.2008	57	27	116	65	24	123	37,90
25.06.2008	63	115	203	70	25	123	725,24
26.06.2008	63	115	203	64	38	140	215,94
13.06.2008	50	20	108	70	25	123	69,51
13.06.2008	51	22	116	70	25	123	44,68
13.06.2008	56	254	276	70	25	123	29,34
13.06.2008	57	27	116	70	25	123	45,85
26.06.2008	63	115	203	64	38	140	373,32
26.06.2008	63	115	203	65	24	123	587,64
13.06.2008	65	24	123	70	25	123	56,98
18.06.2008	50	20	108	51	22	116	21,28
18.06.2008	50	20	108	56	254	276	65,64
18.06.2008	51	22	116	56	254	276	81,97
18.06.2008	50	20	108	57	27	116	111,85
18.06.2008	51	22	116	57	27	116	103,17
18.06.2008	56	254	276	57	27	116	176,02
18.06.2008	50	20	108	63	115	203	685,35
18.06.2008	51	22	116	63	115	203	687,85
18.06.2008	56	254	276	63	115	203	635,39
18.06.2008	57	27	116	63	115	203	789,32
18.06.2008	50	20	108	64	38	140	869,74
18.06.2008	51	22	116	64	38	140	872,27
18.06.2008	56	254	276	64	38	140	819,26
18.06.2008	57	27	116	64	38	140	973,66
26.06.2008	63	115	203	70	25	123	530,19
18.06.2008	50	20	108	65	24	123	100,30

18.06.2008	51	22	116	65	24	123	91,58
18.06.2008	56	254	276	65	24	123	164,66
18.06.2008	57	27	116	65	24	123	11,63
26.06.2008	63	115	203	64	38	140	378,80
26.06.2008	63	115	203	65	24	123	580,66
18.06.2008	50	20	108	70	25	123	32,80
18.06.2008	51	22	116	70	25	123	25,38
18.06.2008	56	254	276	70	25	123	98,36
18.06.2008	57	27	116	70	25	123	79,77
26.06.2008	63	115	203	70	25	123	523,27
26.06.2008	63	115	203	64	38	140	363,16
18.06.2008	65	24	123	70	25	123	68,15
18.06.2008	50	20	108	51	22	116	26,45
18.06.2008	50	20	108	56	254	276	83,21
18.06.2008	51	22	116	56	254	276	106,30
18.06.2008	50	20	108	57	27	116	95,60
18.06.2008	51	22	116	57	27	116	84,16
18.06.2008	56	254	276	57	27	116	175,06
18.06.2008	50	20	108	63	115	203	695,86
18.06.2008	51	22	116	63	115	203	702,92
18.06.2008	56	254	276	63	115	203	636,63
18.06.2008	57	27	116	63	115	203	787,06
18.06.2008	50	20	108	64	38	140	893,06
18.06.2008	51	22	116	64	38	140	900,01
18.06.2008	56	254	276	64	38	140	833,29
18.06.2008	57	27	116	64	38	140	984,13
26.06.2008	63	115	203	65	24	123	601,62
18.06.2008	50	20	108	65	24	123	86,90
18.06.2008	51	22	116	65	24	123	75,14
18.06.2008	56	254	276	65	24	123	166,97
18.06.2008	57	27	116	65	24	123	9,03
26.06.2008	63	115	203	70	25	123	532,72
26.06.2008	63	115	203	64	38	140	361,46

18.06.2008	50	20	108	70	25	123	23,05
18.06.2008	51	22	116	70	25	123	24,33
18.06.2008	56	254	276	70	25	123	104,56
18.06.2008	57	27	116	70	25	123	72,56
26.06.2008	63	115	203	65	24	123	596,19
26.06.2008	63	115	203	70	25	123	530,50
18.06.2008	65	24	123	70	25	123	63,89
18.06.2008	51	22	116	56	254	276	112,50
18.06.2008	51	22	116	57	27	116	81,29
18.06.2008	56	254	276	57	27	116	183,41
18.06.2008	51	22	116	63	115	203	698,65
18.06.2008	56	254	276	63	115	203	625,87
18.06.2008	57	27	116	63	115	203	779,25
18.06.2008	51	22	116	64	38	140	889,67
18.06.2008	56	254	276	64	38	140	816,17
18.06.2008	57	27	116	64	38	140	970,19
26.06.2008	63	115	203	64	38	140	366,16
18.06.2008	51	22	116	65	24	123	73,36
18.06.2008	56	254	276	65	24	123	175,20
18.06.2008	57	27	116	65	24	123	8,27
26.06.2008	63	115	203	65	24	123	589,32
26.06.2008	63	115	203	70	25	123	540,36
18.06.2008	51	22	116	70	25	123	16,62
18.06.2008	56	254	276	70	25	123	108,49
18.06.2008	57	27	116	70	25	123	77,17
26.06.2008	63	115	203	64	38	140	256,29
26.06.2008	63	115	203	65	24	123	696,38
18.06.2008	65	24	123	70	25	123	68,93
18.06.2008	50	20	108	51	22	116	16,84
18.06.2008	50	20	108	56	254	276	190,44
18.06.2008	51	22	116	56	254	276	206,06
18.06.2008	50	20	108	57	27	116	93,85
18.06.2008	51	22	116	57	27	116	79,35

18.06.2008	56	254	276	57	27	116	284,06
18.06.2008	50	20	108	63	115	203	689,14
18.06.2008	51	22	116	63	115	203	701,38
18.06.2008	56	254	276	63	115	203	515,71
18.06.2008	57	27	116	63	115	203	779,79
18.06.2008	50	20	108	64	38	140	878,67
18.06.2008	51	22	116	64	38	140	890,89
18.06.2008	56	254	276	64	38	140	704,07
18.06.2008	57	27	116	64	38	140	969,22
26.06.2008	63	115	203	70	25	123	658,28
18.06.2008	50	20	108	65	24	123	88,16
18.06.2008	51	22	116	65	24	123	73,62
18.06.2008	56	254	276	65	24	123	278,42
18.06.2008	57	27	116	65	24	123	5,73
27.06.2008	63	115	203	64	38	140	181,07
27.06.2008	63	115	203	65	24	123	775,85
18.06.2008	50	20	108	70	25	123	43,76
18.06.2008	51	22	116	70	25	123	40,69
18.06.2008	56	254	276	70	25	123	223,33
18.06.2008	57	27	116	70	25	123	66,95
27.06.2008	63	115	203	70	25	123	722,12
27.06.2008	63	115	203	64	38	140	185,64
18.06.2008	65	24	123	70	25	123	62,15
18.06.2008	50	20	108	51	22	116	23,81
18.06.2008	50	20	108	56	254	276	152,59
18.06.2008	51	22	116	56	254	276	169,60
18.06.2008	50	20	108	57	27	116	100,85
18.06.2008	51	22	116	57	27	116	86,44
18.06.2008	56	254	276	57	27	116	253,44
18.06.2008	50	20	108	63	115	203	691,29
18.06.2008	51	22	116	63	115	203	701,06
18.06.2008	56	254	276	63	115	203	549,53
18.06.2008	57	27	116	63	115	203	787,06

18.06.2008	50	20	108	64	38	140	881,74
18.06.2008	51	22	116	64	38	140	891,37
18.06.2008	56	254	276	64	38	140	739,56
18.06.2008	57	27	116	64	38	140	977,28
27.06.2008	63	115	203	65	24	123	778,10
18.06.2008	50	20	108	65	24	123	88,14
18.06.2008	51	22	116	65	24	123	74,05
18.06.2008	56	254	276	65	24	123	240,73
18.06.2008	57	27	116	65	24	123	12,71
27.06.2008	63	115	203	70	25	123	725,91
04.06.2008	64	38	140	65	24	123	895,05
18.06.2008	50	20	108	70	25	123	36,62
18.06.2008	51	22	116	70	25	123	40,67
18.06.2008	56	254	276	70	25	123	184,04
18.06.2008	57	27	116	70	25	123	73,64
04.06.2008	64	38	140	70	25	123	929,30
04.06.2008	64	38	140	65	24	123	820,92
18.06.2008	65	24	123	70	25	123	61,52
18.06.2008	50	20	108	51	22	116	23,97
18.06.2008	50	20	108	56	254	276	143,70
18.06.2008	51	22	116	56	254	276	153,41
18.06.2008	50	20	108	57	27	116	106,23
18.06.2008	51	22	116	57	27	116	102,24
18.06.2008	56	254	276	57	27	116	249,81
18.06.2008	50	20	108	63	115	203	659,95
18.06.2008	51	22	116	63	115	203	656,82
18.06.2008	56	254	276	63	115	203	537,54
18.06.2008	57	27	116	63	115	203	757,85
18.06.2008	50	20	108	64	38	140	871,46
18.06.2008	51	22	116	64	38	140	868,17
18.06.2008	56	254	276	64	38	140	747,98
18.06.2008	57	27	116	64	38	140	969,00
04.06.2008	64	38	140	70	25	123	880,80

18.06.2008	50	20	108	65	24	123	96,07
18.06.2008	51	22	116	65	24	123	92,80
18.06.2008	56	254	276	65	24	123	239,58
18.06.2008	57	27	116	65	24	123	10,32
04.06.2008	64	38	140	65	24	123	765,21
04.06.2008	64	38	140	70	25	123	850,50
18.06.2008	50	20	108	70	25	123	50,86
18.06.2008	51	22	116	70	25	123	64,55
18.06.2008	56	254	276	70	25	123	183,74
18.06.2008	57	27	116	70	25	123	75,09
04.06.2008	64	38	140	65	24	123	814,17
04.06.2008	64	38	140	70	25	123	794,74
18.06.2008	65	24	123	70	25	123	65,26
19.06.2008	50	20	108	51	22	116	23,03
19.06.2008	50	20	108	56	254	276	122,67
19.06.2008	51	22	116	56	254	276	135,95
19.06.2008	50	20	108	57	27	116	102,68
19.06.2008	51	22	116	57	27	116	93,41
19.06.2008	56	254	276	57	27	116	225,33
19.06.2008	50	20	108	63	115	203	693,43
19.06.2008	51	22	116	63	115	203	696,48
19.06.2008	56	254	276	63	115	203	583,80
19.06.2008	57	27	116	63	115	203	788,90
19.06.2008	50	20	108	64	38	140	874,96
19.06.2008	51	22	116	64	38	140	878,01
19.06.2008	56	254	276	64	38	140	764,60
19.06.2008	57	27	116	64	38	140	970,36
04.06.2008	64	38	140	65	24	123	819,49
19.06.2008	50	20	108	65	24	123	91,86
19.06.2008	51	22	116	65	24	123	83,30
19.06.2008	56	254	276	65	24	123	214,49
19.06.2008	57	27	116	65	24	123	10,97
04.06.2008	64	38	140	70	25	123	779,87

04.06.2008	64	38	140	65	24	123	890,50
19.06.2008	50	20	108	70	25	123	50,05
19.06.2008	51	22	116	70	25	123	58,82
19.06.2008	56	254	276	70	25	123	163,52
19.06.2008	57	27	116	70	25	123	72,24
04.06.2008	64	38	140	70	25	123	862,98
05.06.2008	64	38	140	65	24	123	912,44
19.06.2008	65	24	123	70	25	123	61,86
19.06.2008	50	20	108	51	22	116	27,27
19.06.2008	50	20	108	56	254	276	67,71
19.06.2008	51	22	116	56	254	276	61,14
19.06.2008	50	20	108	57	27	116	112,15
19.06.2008	51	22	116	57	27	116	92,09
19.06.2008	56	254	276	57	27	116	60,05
19.06.2008	50	20	108	63	115	203	683,01
19.06.2008	51	22	116	63	115	203	697,96
19.06.2008	56	254	276	63	115	203	750,29
19.06.2008	57	27	116	63	115	203	789,32
19.06.2008	50	20	108	64	38	140	865,67
19.06.2008	51	22	116	64	38	140	880,49
19.06.2008	56	254	276	64	38	140	932,97
19.06.2008	57	27	116	64	38	140	971,72
05.06.2008	64	38	140	70	25	123	857,50
19.06.2008	50	20	108	65	24	123	101,44
19.06.2008	51	22	116	65	24	123	80,93
19.06.2008	56	254	276	65	24	123	53,11
19.06.2008	57	27	116	65	24	123	11,25
05.06.2008	64	38	140	65	24	123	800,86
05.06.2008	64	38	140	70	25	123	824,32
19.06.2008	50	20	108	70	25	123	206,54
19.06.2008	51	22	116	70	25	123	184,18
19.06.2008	56	254	276	70	25	123	153,24
19.06.2008	57	27	116	70	25	123	95,34

05.06.2008	64	38	140	65	24	123	830,56
05.06.2008	64	38	140	70	25	123	906,88
19.06.2008	65	24	123	70	25	123	105,41
19.06.2008	50	20	108	51	22	116	22,84
19.06.2008	50	20	108	56	254	276	29,12
19.06.2008	51	22	116	56	254	276	32,29
19.06.2008	50	20	108	57	27	116	98,81
19.06.2008	51	22	116	57	27	116	83,23
19.06.2008	56	254	276	57	27	116	76,36
19.06.2008	50	20	108	63	115	203	695,93
19.06.2008	51	22	116	63	115	203	707,39
19.06.2008	56	254	276	63	115	203	724,09
19.06.2008	57	27	116	63	115	203	790,13
19.06.2008	50	20	108	64	38	140	887,56
19.06.2008	51	22	116	64	38	140	899,01
19.06.2008	56	254	276	64	38	140	915,69
19.06.2008	57	27	116	64	38	140	981,72
05.06.2008	64	38	140	65	24	123	906,59
19.06.2008	50	20	108	65	24	123	90,83
19.06.2008	51	22	116	65	24	123	75,13
19.06.2008	56	254	276	65	24	123	68,89
19.06.2008	57	27	116	65	24	123	8,10
05.06.2008	64	38	140	70	25	123	906,98
05.06.2008	64	38	140	56	254	276	777,95
19.06.2008	50	20	108	70	25	123	197,01
19.06.2008	51	22	116	70	25	123	178,46
19.06.2008	56	254	276	70	25	123	176,46
19.06.2008	57	27	116	70	25	123	100,20
05.06.2008	64	38	140	57	27	116	978,62
05.06.2008	64	38	140	63	115	203	203,05
19.06.2008	65	24	123	70	25	123	107,56
19.06.2008	50	20	108	51	22	116	27,83
19.06.2008	50	20	108	56	254	276	24,97

19.06.2008	51	22	116	56	254	276	32,94
19.06.2008	50	20	108	57	27	116	101,60
19.06.2008	51	22	116	57	27	116	89,67
19.06.2008	56	254	276	57	27	116	78,55
19.06.2008	50	20	108	63	115	203	693,27
19.06.2008	51	22	116	63	115	203	698,76
19.06.2008	56	254	276	63	115	203	718,22
19.06.2008	57	27	116	63	115	203	788,11
19.06.2008	50	20	108	64	38	140	881,07
19.06.2008	51	22	116	64	38	140	886,51
19.06.2008	56	254	276	64	38	140	906,02
19.06.2008	57	27	116	64	38	140	975,81
05.06.2008	64	38	140	65	24	123	971,33
19.06.2008	50	20	108	65	24	123	93,55
19.06.2008	51	22	116	65	24	123	81,43
19.06.2008	56	254	276	65	24	123	70,76
19.06.2008	57	27	116	65	24	123	8,26
05.06.2008	64	38	140	70	25	123	908,12
05.06.2008	64	38	140	65	24	123	945,85
19.06.2008	50	20	108	70	25	123	24,69
19.06.2008	51	22	116	70	25	123	33,32
19.06.2008	56	254	276	70	25	123	0,71
19.06.2008	57	27	116	70	25	123	79,09
05.06.2008	64	38	140	70	25	123	888,00
05.06.2008	64	38	140	65	24	123	973,43
19.06.2008	65	24	123	70	25	123	71,32
19.06.2008	50	20	108	51	22	116	26,17
19.06.2008	50	20	108	56	254	276	31,71
19.06.2008	51	22	116	56	254	276	33,29
19.06.2008	50	20	108	57	27	116	102,31
19.06.2008	51	22	116	57	27	116	84,33
19.06.2008	56	254	276	57	27	116	75,94
19.06.2008	50	20	108	63	115	203	688,59

19.06.2008	51	22	116	63	115	203	701,92
19.06.2008	56	254	276	63	115	203	719,91
19.06.2008	57	27	116	63	115	203	785,87
19.06.2008	50	20	108	64	38	140	878,36
19.06.2008	51	22	116	64	38	140	891,60
19.06.2008	56	254	276	64	38	140	909,68
19.06.2008	57	27	116	64	38	140	975,48
05.06.2008	64	38	140	70	25	123	919,02
19.06.2008	50	20	108	65	24	123	97,51
19.06.2008	51	22	116	65	24	123	79,52
19.06.2008	56	254	276	65	24	123	71,37
19.06.2008	57	27	116	65	24	123	4,82
05.06.2008	64	38	140	65	24	123	846,20
05.06.2008	64	38	140	70	25	123	824,06
19.06.2008	50	20	108	70	25	123	25,19
19.06.2008	51	22	116	70	25	123	25,14
19.06.2008	56	254	276	70	25	123	8,45
19.06.2008	57	27	116	70	25	123	78,66
06.06.2008	64	38	140	65	24	123	883,45
06.06.2008	64	38	140	70	25	123	821,34
19.06.2008	65	24	123	70	25	123	73,95
19.06.2008	50	20	108	51	22	116	23,42
19.06.2008	50	20	108	56	254	276	121,82
19.06.2008	51	22	116	56	254	276	140,90
19.06.2008	50	20	108	57	27	116	109,54
19.06.2008	51	22	116	57	27	116	95,49
19.06.2008	56	254	276	57	27	116	230,56
19.06.2008	50	20	108	63	115	203	692,49
19.06.2008	51	22	116	63	115	203	702,78
19.06.2008	56	254	276	63	115	203	585,80
19.06.2008	57	27	116	63	115	203	798,00
19.06.2008	50	20	108	64	38	140	880,72
19.06.2008	51	22	116	64	38	140	891,49

19.06.2008	56	254	276	64	38	140	771,82
19.06.2008	57	27	116	64	38	140	986,82
06.06.2008	64	38	140	65	24	123	839,74
19.06.2008	50	20	108	65	24	123	94,89
19.06.2008	51	22	116	65	24	123	80,28
19.06.2008	56	254	276	65	24	123	216,29
19.06.2008	57	27	116	65	24	123	15,27
06.06.2008	64	38	140	70	25	123	898,53
06.06.2008	64	38	140	65	24	123	830,39
19.06.2008	50	20	108	70	25	123	39,38
19.06.2008	51	22	116	70	25	123	39,37
19.06.2008	56	254	276	70	25	123	155,97
19.06.2008	57	27	116	70	25	123	75,56
06.06.2008	64	38	140	70	25	123	858,52
11.06.2008	64	38	140	65	24	123	954,72
19.06.2008	65	24	123	70	25	123	62,44
19.06.2008	50	20	108	51	22	116	25,97
19.06.2008	50	20	108	56	254	276	118,44
19.06.2008	51	22	116	56	254	276	138,79
19.06.2008	50	20	108	57	27	116	103,43
19.06.2008	51	22	116	57	27	116	88,29
19.06.2008	56	254	276	57	27	116	221,55
19.06.2008	50	20	108	63	115	203	693,16
19.06.2008	51	22	116	63	115	203	703,28
19.06.2008	56	254	276	63	115	203	588,88
19.06.2008	57	27	116	63	115	203	791,25
19.06.2008	50	20	108	64	38	140	876,72
19.06.2008	51	22	116	64	38	140	886,75
19.06.2008	56	254	276	64	38	140	771,92
19.06.2008	57	27	116	64	38	140	974,66
11.06.2008	64	38	140	70	25	123	942,70
19.06.2008	50	20	108	65	24	123	94,94
19.06.2008	51	22	116	65	24	123	79,76

19.06.2008	56	254	276	65	24	123	213,14
19.06.2008	57	27	116	65	24	123	8,56
11.06.2008	64	38	140	65	24	123	1030,43
11.06.2008	64	38	140	70	25	123	944,61
19.06.2008	50	20	108	70	25	123	33,20
19.06.2008	51	22	116	70	25	123	38,34
19.06.2008	56	254	276	70	25	123	146,15
19.06.2008	57	27	116	70	25	123	77,09
11.06.2008	64	38	140	65	24	123	949,30
11.06.2008	64	38	140	70	25	123	889,53
19.06.2008	65	24	123	70	25	123	69,13
19.06.2008	50	20	108	51	22	116	7,83
19.06.2008	50	20	108	56	254	276	63,58
19.06.2008	51	22	116	56	254	276	69,28
19.06.2008	50	20	108	57	27	116	92,50
19.06.2008	51	22	116	57	27	116	91,64
19.06.2008	56	254	276	57	27	116	74,37
19.06.2008	50	20	108	63	115	203	665,94
19.06.2008	51	22	116	63	115	203	665,09
19.06.2008	56	254	276	63	115	203	717,28
19.06.2008	57	27	116	63	115	203	755,54
19.06.2008	50	20	108	64	38	140	679,52
19.06.2008	51	22	116	64	38	140	678,75
19.06.2008	56	254	276	64	38	140	730,45
19.06.2008	57	27	116	64	38	140	769,35
11.06.2008	64	38	140	65	24	123	964,08
19.06.2008	50	20	108	65	24	123	81,71
19.06.2008	51	22	116	65	24	123	81,53
19.06.2008	56	254	276	65	24	123	61,72
19.06.2008	57	27	116	65	24	123	12,97
11.06.2008	64	38	140	70	25	123	936,89
11.06.2008	64	38	140	65	24	123	657,66
19.06.2008	50	20	108	70	25	123	58,77

19.06.2008	51	22	116	70	25	123	64,22
19.06.2008	56	254	276	70	25	123	5,66
19.06.2008	57	27	116	70	25	123	71,14
11.06.2008	64	38	140	70	25	123	629,48
11.06.2008	64	38	140	65	24	123	980,92
19.06.2008	65	24	123	70	25	123	58,32
20.06.2008	50	20	108	51	22	116	9,73
20.06.2008	50	20	108	56	254	276	57,60
20.06.2008	51	22	116	56	254	276	65,92
20.06.2008	50	20	108	57	27	116	94,45
20.06.2008	51	22	116	57	27	116	96,44
20.06.2008	56	254	276	57	27	116	69,87
20.06.2008	50	20	108	63	115	203	640,87
20.06.2008	51	22	116	63	115	203	636,78
20.06.2008	56	254	276	63	115	203	690,83
20.06.2008	57	27	116	63	115	203	731,99
20.06.2008	50	20	108	64	38	140	703,89
20.06.2008	51	22	116	64	38	140	699,87
20.06.2008	56	254	276	64	38	140	753,59
20.06.2008	57	27	116	64	38	140	795,15
11.06.2008	64	38	140	70	25	123	971,95
20.06.2008	50	20	108	65	24	123	80,70
20.06.2008	51	22	116	65	24	123	82,93
20.06.2008	56	254	276	65	24	123	58,47
20.06.2008	57	27	116	65	24	123	13,85
12.06.2008	64	38	140	65	24	123	857,00
12.06.2008	64	38	140	70	25	123	842,61
20.06.2008	50	20	108	70	25	123	56,43
20.06.2008	51	22	116	70	25	123	64,92
20.06.2008	56	254	276	70	25	123	2,39
20.06.2008	57	27	116	70	25	123	72,04
12.06.2008	64	38	140	65	24	123	983,79
12.06.2008	64	38	140	70	25	123	971,59

20.06.2008	65	24	123	70	25	123	60,51
20.06.2008	50	20	108	51	22	116	26,75
20.06.2008	50	20	108	56	254	276	36,54
20.06.2008	51	22	116	56	254	276	35,64
20.06.2008	50	20	108	57	27	116	104,13
20.06.2008	51	22	116	57	27	116	86,79
20.06.2008	56	254	276	57	27	116	71,84
20.06.2008	50	20	108	63	115	203	686,24
20.06.2008	51	22	116	63	115	203	698,93
20.06.2008	56	254	276	63	115	203	722,70
20.06.2008	57	27	116	63	115	203	785,46
20.06.2008	50	20	108	64	38	140	739,31
20.06.2008	51	22	116	64	38	140	752,30
20.06.2008	56	254	276	64	38	140	775,74
20.06.2008	57	27	116	64	38	140	838,89
12.06.2008	64	38	140	65	24	123	974,02
20.06.2008	50	20	108	70	25	123	146,10
20.06.2008	51	22	116	70	25	123	125,05
20.06.2008	56	254	276	70	25	123	116,60
20.06.2008	57	27	116	70	25	123	46,18
12.06.2008	64	38	140	65	24	123	900,68
12.06.2008	64	38	140	70	25	123	1030,60
20.06.2008	50	20	108	51	22	116	26,81
20.06.2008	50	20	108	56	254	276	39,02
20.06.2008	51	22	116	56	254	276	35,08
20.06.2008	50	20	108	57	27	116	107,62
20.06.2008	51	22	116	57	27	116	87,97
20.06.2008	56	254	276	57	27	116	73,46
20.06.2008	50	20	108	63	115	203	686,58
20.06.2008	51	22	116	63	115	203	702,11
20.06.2008	56	254	276	63	115	203	725,41
20.06.2008	57	27	116	63	115	203	789,75
20.06.2008	50	20	108	64	38	140	739,91

20.06.2008	51	22	116	64	38	140	755,90
20.06.2008	56	254	276	64	38	140	778,66
20.06.2008	57	27	116	64	38	140	843,66
12.06.2008	64	38	140	70	25	123	1056,08
20.06.2008	50	20	108	65	24	123	97,17
20.06.2008	51	22	116	65	24	123	76,96
20.06.2008	56	254	276	65	24	123	64,21
20.06.2008	57	27	116	65	24	123	11,15
12.06.2008	64	38	140	65	24	123	968,84
12.06.2008	64	38	140	70	25	123	981,15
20.06.2008	50	20	108	70	25	123	163,88
20.06.2008	51	22	116	70	25	123	140,80
20.06.2008	56	254	276	70	25	123	132,87
20.06.2008	57	27	116	70	25	123	60,51
12.06.2008	64	38	140	65	24	123	967,19
12.06.2008	64	38	140	70	25	123	900,75
20.06.2008	65	24	123	70	25	123	68,70
25.06.2008	50	20	108	51	22	116	32,53
25.06.2008	50	20	108	56	254	276	42,44
25.06.2008	51	22	116	56	254	276	26,14
25.06.2008	50	20	108	57	27	116	115,80
25.06.2008	51	22	116	57	27	116	86,13
25.06.2008	56	254	276	57	27	116	76,33
25.06.2008	50	20	108	63	115	203	480,53
25.06.2008	51	22	116	63	115	203	506,49
25.06.2008	56	254	276	63	115	203	522,97
25.06.2008	57	27	116	63	115	203	591,97
25.06.2008	50	20	108	64	38	140	864,66
25.06.2008	51	22	116	64	38	140	890,39
25.06.2008	56	254	276	64	38	140	907,10
25.06.2008	57	27	116	64	38	140	975,61
12.06.2008	64	38	140	65	24	123	968,12
25.06.2008	50	20	108	65	24	123	104,44

25.06.2008	51	22	116	65	24	123	74,59
25.06.2008	56	254	276	65	24	123	65,54
25.06.2008	57	27	116	65	24	123	11,55
12.06.2008	64	38	140	70	25	123	891,27
12.06.2008	64	38	140	65	24	123	962,97
25.06.2008	50	20	108	70	25	123	57,31
25.06.2008	51	22	116	70	25	123	42,18
25.06.2008	56	254	276	70	25	123	16,60
25.06.2008	57	27	116	70	25	123	68,94
12.06.2008	64	38	140	70	25	123	951,09
12.06.2008	64	38	140	65	24	123	771,68
25.06.2008	65	24	123	70	25	123	59,34
25.06.2008	50	20	108	51	22	116	43,23
25.06.2008	50	20	108	56	254	276	44,28
25.06.2008	51	22	116	56	254	276	26,28
25.06.2008	50	20	108	57	27	116	113,94
25.06.2008	51	22	116	57	27	116	73,12
25.06.2008	56	254	276	57	27	116	73,74
25.06.2008	50	20	108	63	115	203	390,63
25.06.2008	51	22	116	63	115	203	425,69
25.06.2008	56	254	276	63	115	203	434,87
25.06.2008	57	27	116	63	115	203	497,90
25.06.2008	50	20	108	64	38	140	862,37
25.06.2008	51	22	116	64	38	140	898,60
25.06.2008	56	254	276	64	38	140	906,64
25.06.2008	57	27	116	64	38	140	971,05
12.06.2008	64	38	140	70	25	123	769,86
25.06.2008	50	20	108	65	24	123	114,54
25.06.2008	51	22	116	65	24	123	73,92
25.06.2008	56	254	276	65	24	123	74,09
25.06.2008	57	27	116	65	24	123	1,39
13.06.2008	64	38	140	65	24	123	931,56
13.06.2008	64	38	140	70	25	123	917,02

25.06.2008	50	20	108	70	25	123	50,52
25.06.2008	51	22	116	70	25	123	34,16
25.06.2008	56	254	276	70	25	123	8,45
25.06.2008	57	27	116	70	25	123	72,36
13.06.2008	64	38	140	65	24	123	800,30
13.06.2008	64	38	140	70	25	123	763,51
25.06.2008	65	24	123	70	25	123	72,56
25.06.2008	50	20	108	51	22	116	33,96
25.06.2008	50	20	108	56	254	276	44,38
25.06.2008	51	22	116	56	254	276	32,11
25.06.2008	50	20	108	57	27	116	114,60
25.06.2008	51	22	116	57	27	116	85,99
25.06.2008	56	254	276	57	27	116	73,82
25.06.2008	50	20	108	63	115	203	473,63
25.06.2008	51	22	116	63	115	203	498,51
25.06.2008	56	254	276	63	115	203	517,98
25.06.2008	57	27	116	63	115	203	584,38
25.06.2008	50	20	108	64	38	140	860,86
25.06.2008	51	22	116	64	38	140	885,46
25.06.2008	56	254	276	64	38	140	905,21
25.06.2008	57	27	116	64	38	140	971,21
18.06.2008	64	38	140	65	24	123	962,40
25.06.2008	50	20	108	65	24	123	106,66
25.06.2008	51	22	116	65	24	123	77,44
25.06.2008	56	254	276	65	24	123	66,90
25.06.2008	57	27	116	65	24	123	8,91
18.06.2008	64	38	140	70	25	123	897,37
18.06.2008	64	38	140	65	24	123	975,11
25.06.2008	50	20	108	70	25	123	41,25
25.06.2008	51	22	116	70	25	123	22,19
25.06.2008	56	254	276	70	25	123	10,55
25.06.2008	57	27	116	70	25	123	73,58
18.06.2008	64	38	140	70	25	123	915,11

18.06.2008	64	38	140	65	24	123	962,61
25.06.2008	65	24	123	70	25	123	65,90
25.06.2008	50	20	108	51	22	116	28,57
25.06.2008	50	20	108	56	254	276	118,79
25.06.2008	51	22	116	56	254	276	144,64
25.06.2008	50	20	108	57	27	116	103,97
25.06.2008	51	22	116	57	27	116	81,16
25.06.2008	56	254	276	57	27	116	222,62
25.06.2008	50	20	108	63	115	203	512,49
25.06.2008	51	22	116	63	115	203	530,24
25.06.2008	56	254	276	63	115	203	407,73
25.06.2008	57	27	116	63	115	203	610,78
25.06.2008	50	20	108	64	38	140	894,31
25.06.2008	51	22	116	64	38	140	911,77
25.06.2008	56	254	276	64	38	140	788,29
25.06.2008	57	27	116	64	38	140	992,04
18.06.2008	64	38	140	70	25	123	897,67
25.06.2008	50	20	108	65	24	123	70,29
25.06.2008	51	22	116	65	24	123	53,24
25.06.2008	56	254	276	65	24	123	187,50
25.06.2008	57	27	116	65	24	123	37,67
18.06.2008	64	38	140	65	24	123	963,54
18.06.2008	64	38	140	70	25	123	918,66
25.06.2008	50	20	108	70	25	123	43,51
25.06.2008	51	22	116	70	25	123	39,70
25.06.2008	56	254	276	70	25	123	156,48
25.06.2008	57	27	116	70	25	123	70,27
18.06.2008	64	38	140	65	24	123	965,20
18.06.2008	64	38	140	70	25	123	917,20
25.06.2008	65	24	123	70	25	123	32,71
25.06.2008	50	20	108	51	22	116	32,81
25.06.2008	50	20	108	56	254	276	116,66
25.06.2008	51	22	116	56	254	276	145,39

25.06.2008	50	20	108	57	27	116	113,82
25.06.2008	51	22	116	57	27	116	89,69
25.06.2008	56	254	276	57	27	116	230,32
25.06.2008	50	20	108	63	115	203	515,09
25.06.2008	51	22	116	63	115	203	533,77
25.06.2008	56	254	276	63	115	203	411,55
25.06.2008	57	27	116	63	115	203	623,22
25.06.2008	50	20	108	64	38	140	886,02
25.06.2008	51	22	116	64	38	140	904,15
25.06.2008	56	254	276	64	38	140	781,67
25.06.2008	57	27	116	64	38	140	993,36
18.06.2008	64	38	140	65	24	123	960,22
25.06.2008	50	20	108	65	24	123	78,75
25.06.2008	51	22	116	65	24	123	58,04
25.06.2008	56	254	276	65	24	123	194,91
25.06.2008	57	27	116	65	24	123	35,72
18.06.2008	64	38	140	70	25	123	920,52
19.06.2008	64	38	140	65	24	123	960,85
25.06.2008	50	20	108	70	25	123	35,27
25.06.2008	51	22	116	70	25	123	26,96
25.06.2008	56	254	276	70	25	123	151,13
25.06.2008	57	27	116	70	25	123	79,28
19.06.2008	64	38	140	70	25	123	922,82
19.06.2008	64	38	140	65	24	123	960,47
25.06.2008	65	24	123	70	25	123	43,78
25.06.2008	50	20	108	51	22	116	18,91
25.06.2008	50	20	108	56	254	276	115,99
25.06.2008	51	22	116	56	254	276	128,75
25.06.2008	50	20	108	57	27	116	114,22
25.06.2008	51	22	116	57	27	116	105,67
25.06.2008	56	254	276	57	27	116	229,66
25.06.2008	50	20	108	63	115	203	675,80
25.06.2008	51	22	116	63	115	203	680,77

25.06.2008	56	254	276	63	115	203	572,40
25.06.2008	57	27	116	63	115	203	785,87
25.06.2008	50	20	108	64	38	140	878,42
25.06.2008	51	22	116	64	38	140	883,43
25.06.2008	56	254	276	64	38	140	774,20
25.06.2008	57	27	116	64	38	140	988,50
19.06.2008	64	38	140	70	25	123	1056,23
25.06.2008	50	20	108	65	24	123	100,67
25.06.2008	51	22	116	65	24	123	91,36
25.06.2008	56	254	276	65	24	123	216,47
25.06.2008	57	27	116	65	24	123	14,73
19.06.2008	64	38	140	65	24	123	973,69
19.06.2008	64	38	140	70	25	123	1067,12
25.06.2008	50	20	108	70	25	123	49,62
25.06.2008	51	22	116	70	25	123	50,16
25.06.2008	56	254	276	70	25	123	160,95
25.06.2008	57	27	116	70	25	123	70,64
19.06.2008	64	38	140	65	24	123	967,63
19.06.2008	64	38	140	70	25	123	905,76
25.06.2008	65	24	123	70	25	123	59,51
19.06.2008	64	38	140	65	24	123	970,74
26.06.2008	50	20	108	51	22	116	25,10
26.06.2008	50	20	108	56	254	276	56,10
26.06.2008	51	22	116	56	254	276	79,81
26.06.2008	50	20	108	57	27	116	107,53
26.06.2008	51	22	116	57	27	116	91,32
26.06.2008	56	254	276	57	27	116	161,07
26.06.2008	50	20	108	63	115	203	498,10
26.06.2008	51	22	116	63	115	203	509,82
26.06.2008	56	254	276	63	115	203	456,85
26.06.2008	57	27	116	63	115	203	600,79
26.06.2008	50	20	108	64	38	140	871,26
26.06.2008	51	22	116	64	38	140	882,40

26.06.2008	56	254	276	64	38	140	829,99
26.06.2008	57	27	116	64	38	140	973,03
19.06.2008	64	38	140	70	25	123	903,53
26.06.2008	50	20	108	65	24	123	95,37
26.06.2008	51	22	116	65	24	123	78,35
26.06.2008	56	254	276	65	24	123	149,63
26.06.2008	57	27	116	65	24	123	13,22
19.06.2008	64	38	140	65	24	123	971,56
19.06.2008	64	38	140	70	25	123	919,59
26.06.2008	50	20	108	70	25	123	32,80
26.06.2008	51	22	116	70	25	123	25,94
26.06.2008	56	254	276	70	25	123	86,42
26.06.2008	57	27	116	70	25	123	75,06
19.06.2008	64	38	140	65	24	123	966,24
19.06.2008	64	38	140	70	25	123	909,26
26.06.2008	65	24	123	70	25	123	63,27
26.06.2008	50	20	108	51	22	116	24,57
26.06.2008	50	20	108	56	254	276	116,70
26.06.2008	51	22	116	56	254	276	138,88
26.06.2008	50	20	108	57	27	116	105,77
26.06.2008	51	22	116	57	27	116	86,61
26.06.2008	56	254	276	57	27	116	222,12
26.06.2008	50	20	108	63	115	203	491,96
26.06.2008	51	22	116	63	115	203	506,73
26.06.2008	56	254	276	63	115	203	390,66
26.06.2008	57	27	116	63	115	203	592,56
26.06.2008	50	20	108	64	38	140	870,73
26.06.2008	51	22	116	64	38	140	885,20
26.06.2008	56	254	276	64	38	140	768,16
26.06.2008	57	27	116	64	38	140	970,69
19.06.2008	64	38	140	65	24	123	760,13
26.06.2008	50	20	108	65	24	123	92,82
26.06.2008	51	22	116	65	24	123	74,21

26.06.2008	56	254	276	65	24	123	209,07
26.06.2008	57	27	116	65	24	123	13,10
19.06.2008	64	38	140	70	25	123	728,13
20.06.2008	64	38	140	65	24	123	782,17
26.06.2008	50	20	108	70	25	123	33,06
26.06.2008	51	22	116	70	25	123	19,07
26.06.2008	56	254	276	70	25	123	149,58
26.06.2008	57	27	116	70	25	123	72,71
20.06.2008	64	38	140	70	25	123	751,53
20.06.2008	64	38	140	70	25	123	871,90
26.06.2008	65	24	123	70	25	123	59,76
26.06.2008	50	20	108	51	22	116	31,11
26.06.2008	50	20	108	56	254	276	64,65
26.06.2008	51	22	116	56	254	276	94,93
26.06.2008	50	20	108	57	27	116	108,21
26.06.2008	51	22	116	57	27	116	84,97
26.06.2008	56	254	276	57	27	116	170,45
26.06.2008	50	20	108	63	115	203	505,29
26.06.2008	51	22	116	63	115	203	523,20
26.06.2008	56	254	276	63	115	203	457,79
26.06.2008	57	27	116	63	115	203	607,87
26.06.2008	50	20	108	64	38	140	868,29
26.06.2008	51	22	116	64	38	140	885,53
26.06.2008	56	254	276	64	38	140	820,67
26.06.2008	57	27	116	64	38	140	969,89
20.06.2008	64	38	140	65	24	123	832,56
26.06.2008	50	20	108	65	24	123	102,41
26.06.2008	51	22	116	65	24	123	78,79
26.06.2008	56	254	276	65	24	123	165,01
26.06.2008	57	27	116	65	24	123	6,28
20.06.2008	64	38	140	70	25	123	889,99
25.06.2008	64	38	140	65	24	123	964,12
26.06.2008	50	20	108	70	25	123	36,41

26.06.2008	51	22	116	70	25	123	9,69
26.06.2008	56	254	276	70	25	123	101,04
26.06.2008	57	27	116	70	25	123	75,70
25.06.2008	64	38	140	70	25	123	921,43
25.06.2008	64	38	140	65	24	123	971,98
26.06.2008	65	24	123	70	25	123	69,56
26.06.2008	50	20	108	51	22	116	31,08
26.06.2008	50	20	108	56	254	276	115,90
26.06.2008	51	22	116	56	254	276	145,15
26.06.2008	50	20	108	57	27	116	108,25
26.06.2008	51	22	116	57	27	116	81,47
26.06.2008	56	254	276	57	27	116	224,01
26.06.2008	50	20	108	63	115	203	503,06
26.06.2008	51	22	116	63	115	203	525,05
26.06.2008	56	254	276	63	115	203	400,00
26.06.2008	57	27	116	63	115	203	605,83
26.06.2008	50	20	108	64	38	140	864,47
26.06.2008	51	22	116	64	38	140	886,05
26.06.2008	56	254	276	64	38	140	760,61
26.06.2008	57	27	116	64	38	140	966,47
25.06.2008	64	38	140	70	25	123	912,42
26.06.2008	50	20	108	65	24	123	98,72
26.06.2008	51	22	116	65	24	123	71,78
26.06.2008	56	254	276	65	24	123	214,55
26.06.2008	57	27	116	65	24	123	9,70
25.06.2008	64	38	140	65	24	123	962,47
25.06.2008	64	38	140	70	25	123	901,27
26.06.2008	50	20	108	70	25	123	35,85
26.06.2008	51	22	116	70	25	123	5,54
26.06.2008	56	254	276	70	25	123	150,50
26.06.2008	57	27	116	70	25	123	75,94
25.06.2008	64	38	140	65	24	123	963,66
25.06.2008	64	38	140	70	25	123	937,21

26.06.2008	65	24	123	70	25	123	66,24
26.06.2008	50	20	108	51	22	116	25,40
26.06.2008	50	20	108	56	254	276	137,49
26.06.2008	51	22	116	56	254	276	160,84
26.06.2008	50	20	108	57	27	116	101,30
26.06.2008	51	22	116	57	27	116	81,64
26.06.2008	56	254	276	57	27	116	238,03
26.06.2008	50	20	108	63	115	203	498,52
26.06.2008	51	22	116	63	115	203	513,74
26.06.2008	56	254	276	63	115	203	384,29
26.06.2008	57	27	116	63	115	203	594,79
26.06.2008	50	20	108	64	38	140	864,67
26.06.2008	51	22	116	64	38	140	879,63
26.06.2008	56	254	276	64	38	140	747,90
26.06.2008	57	27	116	64	38	140	960,43
25.06.2008	64	38	140	65	24	123	961,79
26.06.2008	50	20	108	65	24	123	94,67
26.06.2008	51	22	116	65	24	123	75,75
26.06.2008	56	254	276	65	24	123	231,09
26.06.2008	57	27	116	65	24	123	7,41
25.06.2008	64	38	140	70	25	123	920,69
25.06.2008	64	38	140	65	24	123	973,88
26.06.2008	50	20	108	70	25	123	42,80
26.06.2008	51	22	116	70	25	123	40,38
26.06.2008	56	254	276	70	25	123	171,77
26.06.2008	57	27	116	70	25	123	69,18
25.06.2008	64	38	140	70	25	123	927,83
26.06.2008	64	38	140	65	24	123	959,84
26.06.2008	65	24	123	70	25	123	61,82
26.06.2008	50	20	108	51	22	116	28,20
26.06.2008	50	20	108	56	254	276	139,64
26.06.2008	51	22	116	56	254	276	164,27
26.06.2008	50	20	108	57	27	116	111,70

26.06.2008	51	22	116	57	27	116	91,38
26.06.2008	56	254	276	57	27	116	250,90
26.06.2008	50	20	108	63	115	203	612,81
26.06.2008	51	22	116	63	115	203	627,61
26.06.2008	56	254	276	63	115	203	493,38
26.06.2008	57	27	116	63	115	203	718,31
26.06.2008	50	20	108	64	38	140	869,10
26.06.2008	51	22	116	64	38	140	883,70
26.06.2008	56	254	276	64	38	140	748,38
26.06.2008	57	27	116	64	38	140	974,22
26.06.2008	64	38	140	70	25	123	903,22
26.06.2008	50	20	108	65	24	123	88,38
26.06.2008	51	22	116	65	24	123	68,87
26.06.2008	56	254	276	65	24	123	227,57
26.06.2008	57	27	116	65	24	123	23,33
26.06.2008	64	38	140	65	24	123	958,95
26.06.2008	64	38	140	70	25	123	901,93
26.06.2008	50	20	108	70	25	123	45,58
26.06.2008	51	22	116	70	25	123	41,61
26.06.2008	56	254	276	70	25	123	179,98
26.06.2008	57	27	116	70	25	123	72,98
26.06.2008	64	38	140	65	24	123	963,62
26.06.2008	64	38	140	70	25	123	895,12
26.06.2008	65	24	123	70	25	123	50,55
26.06.2008	64	38	140	65	24	123	956,87
26.06.2008	64	38	140	70	25	123	891,46
27.06.2008	50	20	108	51	22	116	26,96
27.06.2008	50	20	108	56	254	276	59,71
27.06.2008	51	22	116	56	254	276	56,88
27.06.2008	50	20	108	57	27	116	108,64
27.06.2008	51	22	116	57	27	116	89,85
27.06.2008	56	254	276	57	27	116	64,65
27.06.2008	50	20	108	63	115	203	682,58

27.06.2008	51	22	116	63	115	203	696,53
27.06.2008	56	254	276	63	115	203	741,27
27.06.2008	57	27	116	63	115	203	785,90
27.06.2008	50	20	108	64	38	140	863,64
27.06.2008	51	22	116	64	38	140	877,51
27.06.2008	56	254	276	64	38	140	922,32
27.06.2008	57	27	116	64	38	140	966,81
26.06.2008	64	38	140	65	24	123	955,07
27.06.2008	50	20	108	65	24	123	99,03
27.06.2008	51	22	116	65	24	123	79,83
27.06.2008	56	254	276	65	24	123	57,84
27.06.2008	57	27	116	65	24	123	10,06
26.06.2008	64	38	140	70	25	123	906,51
26.06.2008	64	38	140	65	24	123	952,42
27.06.2008	50	20	108	70	25	123	39,62
27.06.2008	51	22	116	70	25	123	36,65
27.06.2008	56	254	276	70	25	123	21,04
27.06.2008	57	27	116	70	25	123	73,84
26.06.2008	64	38	140	70	25	123	914,57
27.06.2008	64	38	140	65	24	123	956,76
27.06.2008	65	24	123	70	25	123	65,16
27.06.2008	50	20	108	51	22	116	27,87
27.06.2008	50	20	108	56	254	276	63,84
27.06.2008	51	22	116	56	254	276	63,02
27.06.2008	50	20	108	57	27	116	108,61
27.06.2008	51	22	116	57	27	116	90,66
27.06.2008	56	254	276	57	27	116	63,77
27.06.2008	50	20	108	63	115	203	689,04
27.06.2008	51	22	116	63	115	203	701,61
27.06.2008	56	254	276	63	115	203	751,72
27.06.2008	57	27	116	63	115	203	791,85
27.06.2008	50	20	108	64	38	140	874,63
27.06.2008	51	22	116	64	38	140	887,25

27.06.2008	56	254	276	64	38	140	937,21
27.06.2008	57	27	116	64	38	140	977,47
27.06.2008	64	38	140	70	25	123	903,18
27.06.2008	50	20	108	65	24	123	95,56
27.06.2008	51	22	116	65	24	123	76,96
27.06.2008	56	254	276	65	24	123	55,69
27.06.2008	57	27	116	65	24	123	13,75
27.06.2008	64	38	140	65	24	123	963,72
27.06.2008	64	38	140	70	25	123	911,49
27.06.2008	50	20	108	70	25	123	36,87
27.06.2008	51	22	116	70	25	123	35,37
27.06.2008	56	254	276	70	25	123	28,58
27.06.2008	57	27	116	70	25	123	75,18
26.06.2008	n	n	n	n	n	n	n
27.06.2008	n	n	n	n	n	n	n
27.06.2008	65	24	123	70	25	123	63,24
30.05.2007	31	191	228	37	217	230	1,330554485
30.05.2007	29	240	260	30	122	198	101,0363545
30.05.2007	29	240	260	30	122	198	111,0417314
30.05.2007	29	240	260	30	122	198	111,3324792
30.05.2007	29	240	260	30	122	198	100,8759351
30.05.2007	29	240	260	30	122	198	116,9216232
31.05.2007	29	240	260	30	122	198	170,2905808
31.05.2007	29	240	260	30	122	198	131,5352269
31.05.2007	29	240	260	30	122	198	6,33779507
06.06.2007	29	240	260	30	122	198	204,4740626
06.06.2007	29	240	260	30	122	198	155,1065589
06.06.2007	29	240	260	30	122	198	153,2064391
06.06.2007	29	240	260	30	122	198	181,4304338
06.06.2007	29	240	260	30	122	198	146,2747535
07.06.2007	29	240	260	30	122	198	25,218648
07.06.2007	29	240	260	30	122	198	136,7871872
07.06.2007	29	240	260	30	122	198	164,6062931

11.06.2007	29	240	260	30	122	198	8,146285893
12.06.2007	29	240	260	30	122	198	6,466884778
11.06.2007	29	240	260	30	122	198	13,73116757
12.06.2007	29	240	260	30	122	198	11,21064569
11.06.2007	29	240	260	30	122	198	7,141263606
12.06.2007	29	240	260	30	122	198	10,71126297
11.06.2007	29	240	260	30	122	198	16,54480953
12.06.2007	29	240	260	30	122	198	14,43100607
11.06.2007	29	240	260	30	122	198	74,35371817
12.06.2007	29	240	260	30	122	198	24,57467674
12.06.2007	29	240	260	30	122	198	15,31140853
13.06.2007	29	240	260	30	122	198	18,80535792
12.06.2007	29	240	260	30	122	198	36,67248245
13.06.2007	29	240	260	30	122	198	4,514131179
12.06.2007	29	240	260	30	122	198	2,66450108
13.06.2007	29	240	260	30	122	198	11,11760297
19.06.2007	29	240	260	30	122	198	37,27467406
18.06.2007	29	240	260	30	122	198	86,85080926
18.06.2007	29	240	260	30	122	198	103,6490458
19.06.2007	29	240	260	30	122	198	43,95137385
18.06.2007	29	240	260	30	122	198	54,52184556
19.06.2007	29	240	260	30	122	198	38,89988609
18.06.2007	29	240	260	30	122	198	39,93292374
19.06.2007	29	240	260	30	122	198	36,13842749
18.06.2007	29	240	260	30	122	198	51,11425817
19.06.2007	29	240	260	30	122	198	88,96666613
18.06.2007	29	240	260	30	122	198	8,604103035
19.06.2007	29	240	260	30	122	198	106,9532167
19.06.2007	29	240	260	30	122	198	5,012832468
20.06.2007	29	240	260	30	122	198	108,4643018
19.06.2007	29	240	260	30	122	198	55,78719637
20.06.2007	29	240	260	30	122	198	104,0005169
25.06.2007	29	240	260	30	122	198	34,98877645

25.06.2007	29	240	260	30	122	198	70,08389648
26.06.2007	29	240	260	30	122	198	34,6006824
25.06.2007	29	240	260	30	122	198	56,17781389
26.06.2007	29	240	260	30	122	198	18,75750553
25.06.2007	29	240	260	30	122	198	55,72871607
26.06.2007	29	240	260	30	122	198	12,22432502
26.06.2007	29	240	260	30	122	198	21,74621661
25.06.2007	29	240	260	30	122	198	48,34406091
26.06.2007	29	240	260	30	122	198	9,267214474
26.06.2007	29	240	260	30	122	198	23,19492163
27.06.2007	29	240	260	30	122	198	19,31503175
26.06.2007	29	240	260	30	122	198	18,6548972
27.06.2007	29	240	260	30	122	198	18,7254556
26.06.2007	29	240	260	30	122	198	31,83642394
25.06.2007	36	128	206	30	122	198	912,4551985
30.05.2007	30	122	198	34	173	226	3,050164345
30.05.2007	29	240	260	31	191	228	842,0168523
30.05.2007	29	240	260	31	191	228	835,8110854
30.05.2007	29	240	260	31	191	228	853,3834493
30.05.2007	29	240	260	31	191	228	860,8502754
30.05.2007	29	240	260	31	191	228	916,3325482
31.05.2007	29	240	260	31	191	228	888,0823323
31.05.2007	29	240	260	31	191	228	865,1787507
31.05.2007	29	240	260	31	191	228	865,6943261
06.06.2007	29	240	260	31	191	228	959,985766
06.06.2007	29	240	260	31	191	228	864,8077929
06.06.2007	29	240	260	31	191	228	902,0180533
06.06.2007	29	240	260	31	191	228	917,8668028
06.06.2007	29	240	260	31	191	228	880,4474553
07.06.2007	29	240	260	31	191	228	893,7415416
07.06.2007	29	240	260	31	191	228	918,1806947
07.06.2007	29	240	260	31	191	228	885,7250553
07.06.2007	29	240	260	31	191	228	906,1362144

11.06.2007	29	240	260	31	191	228	870,8412628
12.06.2007	29	240	260	31	191	228	853,5185745
11.06.2007	29	240	260	31	191	228	818,3266012
12.06.2007	29	240	260	31	191	228	835,2765943
11.06.2007	29	240	260	31	191	228	875,0214527
12.06.2007	29	240	260	31	191	228	831,3133854
11.06.2007	29	240	260	31	191	228	848,0926021
12.06.2007	29	240	260	31	191	228	1349,986962
11.06.2007	29	240	260	31	191	228	953,6408298
12.06.2007	29	240	260	31	191	228	1368,567499
12.06.2007	29	240	260	31	191	228	883,503816
13.06.2007	29	240	260	31	191	228	811,1889332
12.06.2007	29	240	260	31	191	228	912,3584292
13.06.2007	29	240	260	31	191	228	793,0822229
12.06.2007	29	240	260	31	191	228	860,3582224
13.06.2007	29	240	260	31	191	228	836,4711857
19.06.2007	29	240	260	31	191	228	852,215946
18.06.2007	29	240	260	31	191	228	932,9017886
18.06.2007	29	240	260	31	191	228	876,2700342
19.06.2007	29	240	260	31	191	228	908,734925
18.06.2007	29	240	260	31	191	228	749,1594541
19.06.2007	29	240	260	31	191	228	878,4866406
18.06.2007	29	240	260	31	191	228	805,216006
19.06.2007	29	240	260	31	191	228	765,298956
18.06.2007	29	240	260	31	191	228	917,3071986
19.06.2007	29	240	260	31	191	228	214,3844736
19.06.2007	29	240	260	31	191	228	1041,608069
19.06.2007	29	240	260	31	191	228	844,8077702
20.06.2007	29	240	260	31	191	228	1044,02438
19.06.2007	29	240	260	31	191	228	827,1304568
20.06.2007	29	240	260	31	191	228	232,4896445
25.06.2007	29	240	260	31	191	228	695,3682595
25.06.2007	29	240	260	31	191	228	730,6105173

26.06.2007	29	240	260	31	191	228	682,4818734
25.06.2007	29	240	260	31	191	228	717,7473045
26.06.2007	29	240	260	31	191	228	668,2410632
25.06.2007	29	240	260	31	191	228	712,150604
26.06.2007	29	240	260	31	191	228	673,7652686
25.06.2007	29	240	260	31	191	228	715,7814414
26.06.2007	29	240	260	31	191	228	674,5662861
25.06.2007	29	240	260	31	191	228	712,9283787
26.06.2007	29	240	260	31	191	228	649,9312981
27.06.2007	29	240	260	31	191	228	667,5486272
26.06.2007	29	240	260	31	191	228	672,5316815
27.06.2007	29	240	260	31	191	228	668,6480606
26.06.2007	29	240	260	31	191	228	680,278922
27.06.2007	29	240	260	31	191	228	23,64156766
30.05.2007	29	240	260	34	173	226	41,62235651
30.05.2007	30	122	198	31	191	228	745,5530908
30.05.2007	30	122	198	31	191	228	727,4205669
30.05.2007	30	122	198	31	191	228	746,6596476
30.05.2007	30	122	198	31	191	228	762,4152791
30.05.2007	30	122	198	31	191	228	804,2194811
31.05.2007	30	122	198	31	191	228	718,3758981
31.05.2007	30	122	198	31	191	228	733,6436918
31.05.2007	30	122	198	31	191	228	861,6248343
06.06.2007	30	122	198	31	191	228	757,5240896
06.06.2007	30	122	198	31	191	228	711,2881335
06.06.2007	30	122	198	31	191	228	750,9071283
06.06.2007	30	122	198	31	191	228	738,0988811
06.06.2007	30	122	198	31	191	228	736,905354
07.06.2007	30	122	198	31	191	228	898,117701
07.06.2007	30	122	198	31	191	228	752,4168754
07.06.2007	30	122	198	31	191	228	744,3619053
11.06.2007	30	122	198	31	191	228	863,0296972
12.06.2007	30	122	198	31	191	228	859,5734222

11.06.2007	30	122	198	31	191	228	804,8246325
12.06.2007	30	122	198	31	191	228	826,267602
11.06.2007	30	122	198	31	191	228	868,4606002
12.06.2007	30	122	198	31	191	228	823,0422541
11.06.2007	30	122	198	31	191	228	832,2708391
12.06.2007	30	122	198	31	191	228	1335,658322
11.06.2007	30	122	198	31	191	228	879,5468581
11.06.2007	30	122	198	31	191	228	134,7453155
12.06.2007	30	122	198	31	191	228	1350,837085
12.06.2007	30	122	198	31	191	228	868,7392452
13.06.2007	30	122	198	31	191	228	792,3846101
12.06.2007	30	122	198	31	191	228	875,9730744
13.06.2007	30	122	198	31	191	228	788,6491144
12.06.2007	30	122	198	31	191	228	857,8555987
13.06.2007	30	122	198	31	191	228	845,9153895
19.06.2007	30	122	198	31	191	228	815,2392382
18.06.2007	30	122	198	31	191	228	848,5898514
18.06.2007	30	122	198	31	191	228	773,383141
19.06.2007	30	122	198	31	191	228	865,5502956
18.06.2007	30	122	198	31	191	228	694,7977708
19.06.2007	30	122	198	31	191	228	839,6369844
18.06.2007	30	122	198	31	191	228	765,3106287
19.06.2007	30	122	198	31	191	228	729,1625018
18.06.2007	30	122	198	31	191	228	866,1948811
19.06.2007	30	122	198	31	191	228	125,5926729
19.06.2007	30	122	198	31	191	228	934,6576221
19.06.2007	30	122	198	31	191	228	839,8071426
20.06.2007	30	122	198	31	191	228	935,7853629
19.06.2007	30	122	198	31	191	228	882,9173582
20.06.2007	30	122	198	31	191	228	128,7330321
25.06.2007	30	122	198	31	191	228	662,5972948
25.06.2007	30	122	198	31	191	228	665,4343317
26.06.2007	30	122	198	31	191	228	651,5770119

25.06.2007	30	122	198	31	191	228	662,9581583
26.06.2007	30	122	198	31	191	228	650,7950823
25.06.2007	30	122	198	31	191	228	659,4739512
26.06.2007	30	122	198	31	191	228	662,2679456
26.06.2007	30	122	198	31	191	228	657,1271152
25.06.2007	30	122	198	31	191	228	669,3955959
26.06.2007	30	122	198	31	191	228	659,1973733
27.06.2007	30	122	198	31	191	228	648,5052717
26.06.2007	30	122	198	31	191	228	655,8875265
27.06.2007	30	122	198	31	191	228	650,2927245
26.06.2007	30	122	198	31	191	228	653,0679574
12.06.2007	32	194	230	31	191	228	955,5482075
12.06.2007	33	162	220	31	191	228	715,525985
12.06.2007	34	173	226	31	191	228	752,223359
25.06.2007	36	128	206	31	191	228	273,169422
30.05.2007	29	240	260	30	122	198	42,52499357
30.05.2007	29	240	260	32	194	230	100,3185249
30.05.2007	29	240	260	32	194	230	105,9445243
30.05.2007	29	240	260	32	194	230	87,88239968
30.05.2007	29	240	260	32	194	230	94,20453088
30.05.2007	29	240	260	32	194	230	128,5248252
31.05.2007	29	240	260	32	194	230	31,2117938
31.05.2007	29	240	260	32	194	230	157,4224858
31.05.2007	29	240	260	32	194	230	119,8411748
06.06.2007	29	240	260	32	194	230	36,00709404
06.06.2007	29	240	260	32	194	230	59,67019114
06.06.2007	29	240	260	32	194	230	75,16605479
06.06.2007	29	240	260	32	194	230	39,92523245
06.06.2007	29	240	260	32	194	230	62,63477787
07.06.2007	29	240	260	32	194	230	66,02838587
07.06.2007	29	240	260	32	194	230	134,5874583
07.06.2007	29	240	260	32	194	230	74,37187729
07.06.2007	29	240	260	32	194	230	49,83148688

11.06.2007	29	240	260	32	194	230	64,86465301
12.06.2007	29	240	260	32	194	230	102,1424653
11.06.2007	29	240	260	32	194	230	101,6679939
12.06.2007	29	240	260	32	194	230	140,4376047
11.06.2007	29	240	260	32	194	230	76,23610163
12.06.2007	29	240	260	32	194	230	108,0746173
11.06.2007	29	240	260	32	194	230	93,3975829
12.06.2007	29	240	260	32	194	230	66,45477532
11.06.2007	29	240	260	32	194	230	54,36630993
12.06.2007	29	240	260	32	194	230	106,2470536
12.06.2007	29	240	260	32	194	230	104,9137817
13.06.2007	29	240	260	32	194	230	104,5290506
12.06.2007	29	240	260	32	194	230	66,69011169
13.06.2007	29	240	260	32	194	230	109,6224077
12.06.2007	29	240	260	32	194	230	119,1943682
13.06.2007	29	240	260	32	194	230	119,9658863
19.06.2007	29	240	260	32	194	230	76,34691927
18.06.2007	29	240	260	32	194	230	215,5168662
18.06.2007	29	240	260	32	194	230	17,05106948
19.06.2007	29	240	260	32	194	230	75,92451159
18.06.2007	29	240	260	32	194	230	73,96908791
19.06.2007	29	240	260	32	194	230	43,02282466
18.06.2007	29	240	260	32	194	230	95,75229702
19.06.2007	29	240	260	32	194	230	85,08014333
18.06.2007	29	240	260	32	194	230	71,00151936
19.06.2007	29	240	260	32	194	230	54,76277439
18.06.2007	29	240	260	32	194	230	89,88887947
19.06.2007	29	240	260	32	194	230	130,4054917
19.06.2007	29	240	260	32	194	230	100,9895881
20.06.2007	29	240	260	32	194	230	30,23454704
19.06.2007	29	240	260	32	194	230	114,8695876
20.06.2007	29	240	260	32	194	230	114,1820083
25.06.2007	29	240	260	32	194	230	110,9854274

25.06.2007	29	240	260	32	194	230	39,41428937
26.06.2007	29	240	260	32	194	230	91,17572984
25.06.2007	29	240	260	32	194	230	85,03936623
26.06.2007	29	240	260	32	194	230	98,66868914
25.06.2007	29	240	260	32	194	230	87,79051798
26.06.2007	29	240	260	32	194	230	88,05197674
25.06.2007	29	240	260	32	194	230	83,88633024
26.06.2007	29	240	260	32	194	230	93,11893112
25.06.2007	29	240	260	32	194	230	85,73794621
26.06.2007	29	240	260	32	194	230	141,1829263
26.06.2007	29	240	260	32	194	230	100,9352833
27.06.2007	29	240	260	32	194	230	97,56388425
26.06.2007	29	240	260	32	194	230	111,4312936
27.06.2007	29	240	260	32	194	230	95,37228023
26.06.2007	29	240	260	32	194	230	92,97919492
27.06.2007	29	240	260	32	194	230	114,9415927
30.05.2007	33	162	220	36	128	206	77,73507776
30.05.2007	30	122	198	32	194	230	197,9948506
30.05.2007	30	122	198	32	194	230	215,1906775
30.05.2007	30	122	198	32	194	230	195,3071074
30.05.2007	30	122	198	32	194	230	193,0748252
30.05.2007	30	122	198	32	194	230	242,2164679
31.05.2007	30	122	198	32	194	230	201,0976082
31.05.2007	30	122	198	32	194	230	27,72160482
31.05.2007	30	122	198	32	194	230	123,1570654
06.06.2007	30	122	198	32	194	230	235,2307049
06.06.2007	30	122	198	32	194	230	212,4182184
06.06.2007	30	122	198	32	194	230	226,3151462
06.06.2007	30	122	198	32	194	230	219,1326036
06.06.2007	30	122	198	32	194	230	206,379287
07.06.2007	30	122	198	32	194	230	111,3793226
07.06.2007	30	122	198	32	194	230	206,8490249
07.06.2007	30	122	198	32	194	230	208,8051508

11.06.2007	30	122	198	32	194	230	72,83835659
12.06.2007	30	122	198	32	194	230	96,0051038
11.06.2007	30	122	198	32	194	230	113,9858141
12.06.2007	30	122	198	32	194	230	149,3534634
11.06.2007	30	122	198	32	194	230	82,88913994
12.06.2007	30	122	198	32	194	230	117,1073846
11.06.2007	30	122	198	32	194	230	109,1081583
12.06.2007	30	122	198	32	194	230	80,8395515
11.06.2007	30	122	198	32	194	230	126,2744863
12.06.2007	30	122	198	32	194	230	128,2726999
12.06.2007	30	122	198	32	194	230	119,0759793
13.06.2007	30	122	198	32	194	230	123,3307257
12.06.2007	30	122	198	32	194	230	103,3513221
13.06.2007	30	122	198	32	194	230	114,1099196
12.06.2007	30	122	198	32	194	230	121,577315
13.06.2007	30	122	198	32	194	230	111,7169852
19.06.2007	30	122	198	32	194	230	112,2478504
18.06.2007	30	122	198	32	194	230	134,5682963
18.06.2007	30	122	198	32	194	230	120,5462298
19.06.2007	30	122	198	32	194	230	118,5020795
18.06.2007	30	122	198	32	194	230	128,128476
19.06.2007	30	122	198	32	194	230	80,85329361
18.06.2007	30	122	198	32	194	230	134,4216213
19.06.2007	30	122	198	32	194	230	119,6165099
18.06.2007	30	122	198	32	194	230	121,0020288
19.06.2007	30	122	198	32	194	230	131,6460822
18.06.2007	30	122	198	32	194	230	97,09364492
19.06.2007	30	122	198	32	194	230	33,29873696
19.06.2007	30	122	198	32	194	230	105,9738061
20.06.2007	30	122	198	32	194	230	83,34052267
19.06.2007	30	122	198	32	194	230	59,50906283
20.06.2007	30	122	198	32	194	230	23,22702642
25.06.2007	30	122	198	32	194	230	143,3148101

25.06.2007	30	122	198	32	194	230	105,340332
26.06.2007	30	122	198	32	194	230	124,2208735
25.06.2007	30	122	198	32	194	230	140,3530024
26.06.2007	30	122	198	32	194	230	117,0582423
25.06.2007	30	122	198	32	194	230	140,1163598
26.06.2007	30	122	198	32	194	230	100,111132
26.06.2007	30	122	198	32	194	230	112,3105186
25.06.2007	30	122	198	32	194	230	129,746224
26.06.2007	30	122	198	32	194	230	131,9518405
26.06.2007	30	122	198	32	194	230	116,3987269
27.06.2007	30	122	198	32	194	230	116,8060345
26.06.2007	30	122	198	32	194	230	129,038588
27.06.2007	30	122	198	32	194	230	114,0946018
26.06.2007	30	122	198	32	194	230	122,7922745
30.05.2007	29	240	260	32	194	230	85,63284745
30.05.2007	31	191	228	32	194	230	942,0321738
30.05.2007	31	191	228	32	194	230	941,6209934
30.05.2007	31	191	228	32	194	230	940,59265
30.05.2007	31	191	228	32	194	230	954,7833609
30.05.2007	31	191	228	32	194	230	1044,680029
31.05.2007	31	191	228	32	194	230	919,1410278
31.05.2007	31	191	228	32	194	230	708,2306174
31.05.2007	31	191	228	32	194	230	983,9842816
06.06.2007	31	191	228	32	194	230	992,279386
06.06.2007	31	191	228	32	194	230	923,3456027
06.06.2007	31	191	228	32	194	230	976,5522609
06.06.2007	31	191	228	32	194	230	956,6172222
06.06.2007	31	191	228	32	194	230	942,3030822
07.06.2007	31	191	228	32	194	230	957,7861501
07.06.2007	31	191	228	32	194	230	789,6629324
07.06.2007	31	191	228	32	194	230	958,5014226
07.06.2007	31	191	228	32	194	230	952,6326772
11.06.2007	31	191	228	32	194	230	935,5674841

11.06.2007	31	191	228	32	194	230	915,8022615
12.06.2007	31	191	228	32	194	230	975,6098838
11.06.2007	31	191	228	32	194	230	951,2487738
12.06.2007	31	191	228	32	194	230	938,9931367
11.06.2007	31	191	228	32	194	230	941,3775899
12.06.2007	31	191	228	32	194	230	1415,096152
11.06.2007	31	191	228	32	194	230	1005,472393
12.06.2007	31	191	228	32	194	230	1471,741076
12.06.2007	31	191	228	32	194	230	987,3497687
13.06.2007	31	191	228	32	194	230	915,7119616
12.06.2007	31	191	228	32	194	230	978,318156
13.06.2007	31	191	228	32	194	230	902,4073315
12.06.2007	31	191	228	32	194	230	978,8162862
13.06.2007	31	191	228	32	194	230	955,3520151
19.06.2007	31	191	228	32	194	230	927,05159
18.06.2007	31	191	228	32	194	230	717,8586628
18.06.2007	31	191	228	32	194	230	892,7629976
19.06.2007	31	191	228	32	194	230	984,049133
18.06.2007	31	191	228	32	194	230	821,4169576
19.06.2007	31	191	228	32	194	230	919,9752289
18.06.2007	31	191	228	32	194	230	896,164465
19.06.2007	31	191	228	32	194	230	845,8281366
18.06.2007	31	191	228	32	194	230	985,9948562
19.06.2007	31	191	228	32	194	230	252,1046968
19.06.2007	31	191	228	32	194	230	913,9882386
19.06.2007	31	191	228	32	194	230	945,7254443
19.06.2007	31	191	228	32	194	230	703,8289817
20.06.2007	31	191	228	32	194	230	1016,833592
19.06.2007	31	191	228	32	194	230	941,5751017
20.06.2007	31	191	228	32	194	230	120,5114198
25.06.2007	31	191	228	32	194	230	805,8770971
25.06.2007	31	191	228	32	194	230	769,1374534
26.06.2007	31	191	228	32	194	230	773,2397592

25.06.2007	31	191	228	32	194	230	802,780994
26.06.2007	31	191	228	32	194	230	765,8824318
25.06.2007	31	191	228	32	194	230	799,2972475
26.06.2007	31	191	228	32	194	230	760,7186536
25.06.2007	31	191	228	32	194	230	798,787813
26.06.2007	31	191	228	32	194	230	767,36407
25.06.2007	31	191	228	32	194	230	798,3093105
26.06.2007	31	191	228	32	194	230	790,5080682
27.06.2007	31	191	228	32	194	230	762,2315713
26.06.2007	31	191	228	32	194	230	783,4958148
27.06.2007	31	191	228	32	194	230	762,0666295
26.06.2007	31	191	228	32	194	230	772,6776676
27.06.2007	31	191	228	32	194	230	138,4224375
25.06.2007	36	128	206	32	194	230	1054,912041
30.05.2007	30	122	198	39	83	181	95,28269165
30.05.2007	29	240	260	33	162	220	434,4972978
30.05.2007	29	240	260	33	162	220	381,559814
30.05.2007	29	240	260	33	162	220	394,5148426
30.05.2007	29	240	260	33	162	220	369,6725494
30.05.2007	29	240	260	33	162	220	391,7280546
31.05.2007	29	240	260	33	162	220	423,2797963
31.05.2007	29	240	260	33	162	220	396,7174244
31.05.2007	29	240	260	33	162	220	374,3877291
06.06.2007	29	240	260	33	162	220	216,1276706
06.06.2007	29	240	260	33	162	220	194,3817627
06.06.2007	29	240	260	33	162	220	154,5660177
06.06.2007	29	240	260	33	162	220	189,2864016
06.06.2007	29	240	260	33	162	220	196,6088767
07.06.2007	29	240	260	33	162	220	204,0772341
07.06.2007	29	240	260	33	162	220	190,4466603
07.06.2007	29	240	260	33	162	220	185,0629308
07.06.2007	29	240	260	33	162	220	203,4825266
11.06.2007	29	240	260	33	162	220	9,337127394

12.06.2007	29	240	260	33	162	220	139,255143
11.06.2007	29	240	260	33	162	220	156,7185814
12.06.2007	29	240	260	33	162	220	148,4602611
11.06.2007	29	240	260	33	162	220	156,9407522
12.06.2007	29	240	260	33	162	220	149,6094872
11.06.2007	29	240	260	33	162	220	172,9316343
12.06.2007	29	240	260	33	162	220	140,0169848
11.06.2007	29	240	260	33	162	220	208,5661425
12.06.2007	29	240	260	33	162	220	145,9916008
12.06.2007	29	240	260	33	162	220	171,3756749
13.06.2007	29	240	260	33	162	220	150,1493437
12.06.2007	29	240	260	33	162	220	207,4424412
13.06.2007	29	240	260	33	162	220	160,5556811
12.06.2007	29	240	260	33	162	220	161,1167454
13.06.2007	29	240	260	33	162	220	145,8530077
19.06.2007	29	240	260	33	162	220	183,7707214
18.06.2007	29	240	260	33	162	220	214,1833868
18.06.2007	29	240	260	33	162	220	245,7389016
19.06.2007	29	240	260	33	162	220	184,0233808
18.06.2007	29	240	260	33	162	220	189,8895135
19.06.2007	29	240	260	33	162	220	188,7084315
18.06.2007	29	240	260	33	162	220	198,8657373
19.06.2007	29	240	260	33	162	220	143,6231157
18.06.2007	29	240	260	33	162	220	134,4695904
19.06.2007	29	240	260	33	162	220	193,8847564
18.06.2007	29	240	260	33	162	220	149,7136263
19.06.2007	29	240	260	33	162	220	239,2842118
19.06.2007	29	240	260	33	162	220	144,7990702
20.06.2007	29	240	260	33	162	220	221,6647385
19.06.2007	29	240	260	33	162	220	141,5943418
20.06.2007	29	240	260	33	162	220	107,224558
25.06.2007	29	240	260	33	162	220	146,3772233
25.06.2007	29	240	260	33	162	220	189,7785066

26.06.2007	29	240	260	33	162	220	151,5539357
25.06.2007	29	240	260	33	162	220	166,1029412
26.06.2007	29	240	260	33	162	220	140,1903454
25.06.2007	29	240	260	33	162	220	182,7957551
26.06.2007	29	240	260	33	162	220	139,7117445
25.06.2007	29	240	260	33	162	220	176,2928591
26.06.2007	29	240	260	33	162	220	148,2149285
25.06.2007	29	240	260	33	162	220	170,8921003
26.06.2007	29	240	260	33	162	220	118,0803904
26.06.2007	29	240	260	33	162	220	147,3449867
27.06.2007	29	240	260	33	162	220	135,533876
26.06.2007	29	240	260	33	162	220	133,738726
27.06.2007	29	240	260	33	162	220	133,4988163
26.06.2007	29	240	260	33	162	220	155,5225512
27.06.2007	29	240	260	33	162	220	140,0425285
30.05.2007	34	173	226	39	83	181	95,99273098
30.05.2007	30	122	198	33	162	220	334,2577401
30.05.2007	30	122	198	33	162	220	271,5249112
30.05.2007	30	122	198	33	162	220	284,5847658
30.05.2007	30	122	198	33	162	220	269,6267763
30.05.2007	30	122	198	33	162	220	276,9303437
31.05.2007	30	122	198	33	162	220	253,1796955
31.05.2007	30	122	198	33	162	220	265,280738
31.05.2007	30	122	198	33	162	220	369,8671872
06.06.2007	30	122	198	33	162	220	12,95784084
06.06.2007	30	122	198	33	162	220	59,06663731
06.06.2007	30	122	198	33	162	220	1,469276654
06.06.2007	30	122	198	33	162	220	8,016071053
06.06.2007	30	122	198	33	162	220	67,74997496
07.06.2007	30	122	198	33	162	220	172,6700948
07.06.2007	30	122	198	33	162	220	67,83624569
07.06.2007	30	122	198	33	162	220	62,22402037
11.06.2007	30	122	198	33	162	220	16,65391142

12.06.2007	30	122	198	33	162	220	145,5494255
11.06.2007	30	122	198	33	162	220	143,5106864
12.06.2007	30	122	198	33	162	220	140,8480229
11.06.2007	30	122	198	33	162	220	150,9180467
12.06.2007	30	122	198	33	162	220	140,3176666
11.06.2007	30	122	198	33	162	220	158,3400911
12.06.2007	30	122	198	33	162	220	125,8228911
12.06.2007	30	122	198	33	162	220	124,8701438
12.06.2007	30	122	198	33	162	220	157,6082797
13.06.2007	30	122	198	33	162	220	131,6937591
12.06.2007	30	122	198	33	162	220	170,779561
13.06.2007	30	122	198	33	162	220	156,0447302
12.06.2007	30	122	198	33	162	220	158,8480047
13.06.2007	30	122	198	33	162	220	153,8749924
19.06.2007	30	122	198	33	162	220	148,39539
18.06.2007	30	122	198	33	162	220	132,0930181
18.06.2007	30	122	198	33	162	220	142,2216183
19.06.2007	30	122	198	33	162	220	142,5942527
18.06.2007	30	122	198	33	162	220	136,0543663
19.06.2007	30	122	198	33	162	220	150,1545171
18.06.2007	30	122	198	33	162	220	159,0313947
19.06.2007	30	122	198	33	162	220	107,8657242
18.06.2007	30	122	198	33	162	220	85,59497187
19.06.2007	30	122	198	33	162	220	106,0420988
18.06.2007	30	122	198	33	162	220	143,3986098
19.06.2007	30	122	198	33	162	220	132,3328144
19.06.2007	30	122	198	33	162	220	139,9877586
20.06.2007	30	122	198	33	162	220	115,2832381
19.06.2007	30	122	198	33	162	220	197,3028632
20.06.2007	30	122	198	33	162	220	7,234880261
25.06.2007	30	122	198	33	162	220	114,2565812
25.06.2007	30	122	198	33	162	220	124,207639
26.06.2007	30	122	198	33	162	220	121,9675521

25.06.2007	30	122	198	33	162	220	111,6787761
26.06.2007	30	122	198	33	162	220	123,1499877
25.06.2007	30	122	198	33	162	220	130,6782488
26.06.2007	30	122	198	33	162	220	128,3433083
26.06.2007	30	122	198	33	162	220	131,1000317
25.06.2007	30	122	198	33	162	220	127,6220906
26.06.2007	30	122	198	33	162	220	127,3122128
26.06.2007	30	122	198	33	162	220	133,2972404
27.06.2007	30	122	198	33	162	220	116,7535934
26.06.2007	30	122	198	33	162	220	116,9975767
27.06.2007	30	122	198	33	162	220	115,5144543
26.06.2007	30	122	198	33	162	220	129,8786374
30.05.2007	32	194	230	34	173	226	125,2827502
30.05.2007	31	191	228	33	162	220	420,7776141
30.05.2007	31	191	228	33	162	220	457,1136624
30.05.2007	31	191	228	33	162	220	465,4768538
30.05.2007	31	191	228	33	162	220	494,2576269
30.05.2007	31	191	228	33	162	220	528,6469232
31.05.2007	31	191	228	33	162	220	465,3836331
31.05.2007	31	191	228	33	162	220	468,798346
31.05.2007	31	191	228	33	162	220	494,5941823
06.06.2007	31	191	228	33	162	220	745,186334
06.06.2007	31	191	228	33	162	220	672,3800997
06.06.2007	31	191	228	33	162	220	749,47021
06.06.2007	31	191	228	33	162	220	730,0944709
06.06.2007	31	191	228	33	162	220	684,8923233
07.06.2007	31	191	228	33	162	220	691,1544218
07.06.2007	31	191	228	33	162	220	728,8833944
07.06.2007	31	191	228	33	162	220	701,6649061
07.06.2007	31	191	228	33	162	220	703,9139402
11.06.2007	31	191	228	33	162	220	879,6688721
11.06.2007	31	191	228	33	162	220	662,2803159
12.06.2007	31	191	228	33	162	220	689,2002263

11.06.2007	31	191	228	33	162	220	720,2433445
12.06.2007	31	191	228	33	162	220	684,7049125
11.06.2007	31	191	228	33	162	220	678,4298154
12.06.2007	31	191	228	33	162	220	1216,483489
11.06.2007	31	191	228	33	162	220	745,1083787
12.06.2007	31	191	228	33	162	220	1228,343339
12.06.2007	31	191	228	33	162	220	715,0883165
13.06.2007	31	191	228	33	162	220	664,3665203
12.06.2007	31	191	228	33	162	220	707,6694066
13.06.2007	31	191	228	33	162	220	637,5932774
12.06.2007	31	191	228	33	162	220	703,0758607
13.06.2007	31	191	228	33	162	220	695,3153575
19.06.2007	31	191	228	33	162	220	671,5510271
18.06.2007	31	191	228	33	162	220	718,8229683
18.06.2007	31	191	228	33	162	220	634,4728935
19.06.2007	31	191	228	33	162	220	726,2470579
18.06.2007	31	191	228	33	162	220	564,7319773
19.06.2007	31	191	228	33	162	220	690,3548769
18.06.2007	31	191	228	33	162	220	607,646497
19.06.2007	31	191	228	33	162	220	623,303184
18.06.2007	31	191	228	33	162	220	787,4740722
19.06.2007	31	191	228	33	162	220	24,89965129
19.06.2007	31	191	228	33	162	220	802,3425816
20.06.2007	31	191	228	33	162	220	823,2542278
19.06.2007	31	191	228	33	162	220	685,9035569
20.06.2007	31	191	228	33	162	220	125,2758287
25.06.2007	31	191	228	33	162	220	548,9974673
25.06.2007	31	191	228	33	162	220	541,4546408
26.06.2007	31	191	228	33	162	220	531,0029838
25.06.2007	31	191	228	33	162	220	551,6542644
26.06.2007	31	191	228	33	162	220	528,1380072
25.06.2007	31	191	228	33	162	220	529,3864572
26.06.2007	31	191	228	33	162	220	534,0775168

25.06.2007	31	191	228	33	162	220	540,3075583
26.06.2007	31	191	228	33	162	220	526,3659941
25.06.2007	31	191	228	33	162	220	542,2197856
26.06.2007	31	191	228	33	162	220	532,2486762
27.06.2007	31	191	228	33	162	220	532,2542086
26.06.2007	31	191	228	33	162	220	538,9081396
27.06.2007	31	191	228	33	162	220	535,50862
26.06.2007	31	191	228	33	162	220	524,89687
27.06.2007	31	191	228	33	162	220	117,306135
30.05.2007	30	122	198	32	194	230	125,3563398
30.05.2007	32	194	230	33	162	220	532,1584704
30.05.2007	32	194	230	33	162	220	486,645475
30.05.2007	32	194	230	33	162	220	479,8844197
30.05.2007	32	194	230	33	162	220	462,6937607
30.05.2007	32	194	230	33	162	220	518,9358662
31.05.2007	32	194	230	33	162	220	454,2121656
31.05.2007	32	194	230	33	162	220	240,6102149
31.05.2007	32	194	230	33	162	220	490,8834113
06.06.2007	32	194	230	33	162	220	247,3072996
06.06.2007	32	194	230	33	162	220	253,9177267
06.06.2007	32	194	230	33	162	220	227,7204097
06.06.2007	32	194	230	33	162	220	227,074382
06.06.2007	32	194	230	33	162	220	259,1202878
07.06.2007	32	194	230	33	162	220	269,5573551
07.06.2007	32	194	230	33	162	220	81,95125864
07.06.2007	32	194	230	33	162	220	259,0315231
07.06.2007	32	194	230	33	162	220	251,7547322
11.06.2007	32	194	230	33	162	220	56,38756776
12.06.2007	32	194	230	33	162	220	241,2380794
11.06.2007	32	194	230	33	162	220	256,8687602
12.06.2007	32	194	230	33	162	220	288,3683484
11.06.2007	32	194	230	33	162	220	232,4666434
12.06.2007	32	194	230	33	162	220	257,4247339

11.06.2007	32	194	230	33	162	220	265,876878
12.06.2007	32	194	230	33	162	220	206,3138455
11.06.2007	32	194	230	33	162	220	261,0099403
12.06.2007	32	194	230	33	162	220	252,230137
12.06.2007	32	194	230	33	162	220	276,2808705
13.06.2007	32	194	230	33	162	220	253,4228201
12.06.2007	32	194	230	33	162	220	274,1300098
13.06.2007	32	194	230	33	162	220	269,4773914
12.06.2007	32	194	230	33	162	220	280,0993592
13.06.2007	32	194	230	33	162	220	265,566272
19.06.2007	32	194	230	33	162	220	260,0634961
18.06.2007	32	194	230	33	162	220	6,735753985
18.06.2007	32	194	230	33	162	220	262,6992364
19.06.2007	32	194	230	33	162	220	259,9405678
18.06.2007	32	194	230	33	162	220	263,8502307
19.06.2007	32	194	230	33	162	220	230,9967295
18.06.2007	32	194	230	33	162	220	292,8053352
19.06.2007	32	194	230	33	162	220	222,5349485
18.06.2007	32	194	230	33	162	220	199,4896727
19.06.2007	32	194	230	33	162	220	229,3631595
18.06.2007	32	194	230	33	162	220	239,1783049
19.06.2007	32	194	230	33	162	220	114,7094974
19.06.2007	32	194	230	33	162	220	244,9312633
20.06.2007	32	194	230	33	162	220	193,740236
19.06.2007	32	194	230	33	162	220	256,442113
20.06.2007	32	194	230	33	162	220	16,02199768
25.06.2007	32	194	230	33	162	220	257,0985595
25.06.2007	32	194	230	33	162	220	227,8452409
26.06.2007	32	194	230	33	162	220	242,2508665
25.06.2007	32	194	230	33	162	220	251,1289548
26.06.2007	32	194	230	33	162	220	237,8573635
25.06.2007	32	194	230	33	162	220	269,9678262
26.06.2007	32	194	230	33	162	220	226,8441505

25.06.2007	32	194	230	33	162	220	258,5862292
26.06.2007	32	194	230	33	162	220	241,1678086
25.06.2007	32	194	230	33	162	220	256,0943017
26.06.2007	32	194	230	33	162	220	258,2619239
26.06.2007	32	194	230	33	162	220	248,1427877
27.06.2007	32	194	230	33	162	220	230,3119308
26.06.2007	32	194	230	33	162	220	245,054183
27.06.2007	32	194	230	33	162	220	226,6581631
26.06.2007	32	194	230	33	162	220	247,7894329
27.06.2007	32	194	230	33	162	220	254,4881337
25.06.2007	36	128	206	33	162	220	801,9253418
30.05.2007	29	240	260	39	83	181	137,6116393
30.05.2007	29	240	260	34	173	226	37,22654178
30.05.2007	29	240	260	34	173	226	31,6797103
30.05.2007	29	240	260	34	173	226	55,19608715
30.05.2007	29	240	260	34	173	226	101,3597288
30.05.2007	29	240	260	34	173	226	118,4033302
31.05.2007	29	240	260	34	173	226	155,2476547
31.05.2007	29	240	260	34	173	226	90,51129207
31.05.2007	29	240	260	34	173	226	100,0994923
06.06.2007	29	240	260	34	173	226	136,3498752
06.06.2007	29	240	260	34	173	226	144,4449441
06.06.2007	29	240	260	34	173	226	134,2570933
06.06.2007	29	240	260	34	173	226	177,3253901
06.06.2007	29	240	260	34	173	226	124,3756567
07.06.2007	29	240	260	34	173	226	157,5287707
07.06.2007	29	240	260	34	173	226	133,210612
07.06.2007	29	240	260	34	173	226	147,4613224
07.06.2007	29	240	260	34	173	226	150,8147691
11.06.2007	29	240	260	34	173	226	117,0543069
12.06.2007	29	240	260	34	173	226	108,1989915
11.06.2007	29	240	260	34	173	226	117,6609515
12.06.2007	29	240	260	34	173	226	73,62680928

11.06.2007	29	240	260	34	173	226	101,7232839
12.06.2007	29	240	260	34	173	226	84,80771263
11.06.2007	29	240	260	34	173	226	91,21623934
12.06.2007	29	240	260	34	173	226	2,389258648
11.06.2007	29	240	260	34	173	226	140,0268545
12.06.2007	29	240	260	34	173	226	16,68425631
12.06.2007	29	240	260	34	173	226	107,5010121
13.06.2007	29	240	260	34	173	226	37,52439995
12.06.2007	29	240	260	34	173	226	130,021163
13.06.2007	29	240	260	34	173	226	80,73064923
12.06.2007	29	240	260	34	173	226	117,2655491
13.06.2007	29	240	260	34	173	226	76,73970037
19.06.2007	29	240	260	34	173	226	139,4318802
18.06.2007	29	240	260	34	173	226	118,7452086
18.06.2007	29	240	260	34	173	226	178,3497153
19.06.2007	29	240	260	34	173	226	138,2532627
18.06.2007	29	240	260	34	173	226	142,1536779
19.06.2007	29	240	260	34	173	226	77,60401407
18.06.2007	29	240	260	34	173	226	162,2351965
19.06.2007	29	240	260	34	173	226	57,33464613
18.06.2007	29	240	260	34	173	226	169,495244
19.06.2007	29	240	260	34	173	226	110,253972
18.06.2007	29	240	260	34	173	226	112,4735817
19.06.2007	29	240	260	34	173	226	222,4498165
19.06.2007	29	240	260	34	173	226	124,2095297
20.06.2007	29	240	260	34	173	226	119,5361378
19.06.2007	29	240	260	34	173	226	101,7532922
20.06.2007	29	240	260	34	173	226	253,6777469
25.06.2007	29	240	260	34	173	226	103,8063109
25.06.2007	29	240	260	34	173	226	156,3493957
26.06.2007	29	240	260	34	173	226	113,3129209
25.06.2007	29	240	260	34	173	226	146,8744791
26.06.2007	29	240	260	34	173	226	107,4087996

25.06.2007	29	240	260	34	173	226	144,7976666
26.06.2007	29	240	260	34	173	226	104,502951
25.06.2007	29	240	260	34	173	226	145,2054653
26.06.2007	29	240	260	34	173	226	112,9644772
25.06.2007	29	240	260	34	173	226	147,4780131
26.06.2007	29	240	260	34	173	226	84,79482443
26.06.2007	29	240	260	34	173	226	120,8270026
27.06.2007	29	240	260	34	173	226	97,71060682
26.06.2007	29	240	260	34	173	226	109,0629069
27.06.2007	29	240	260	34	173	226	95,36143554
26.06.2007	29	240	260	34	173	226	119,5427116
27.06.2007	29	240	260	34	173	226	102,619496
30.05.2007	32	194	230	39	83	181	219,7319804
30.05.2007	30	122	198	34	173	226	63,81044368
30.05.2007	30	122	198	34	173	226	79,36222541
30.05.2007	30	122	198	34	173	226	56,13647704
30.05.2007	30	122	198	34	173	226	7,075824731
30.05.2007	30	122	198	34	173	226	2,543086536
31.05.2007	30	122	198	34	173	226	18,24092067
31.05.2007	30	122	198	34	173	226	41,03488391
31.05.2007	30	122	198	34	173	226	94,66927555
06.06.2007	30	122	198	34	173	226	68,12629625
06.06.2007	30	122	198	34	173	226	10,93409913
06.06.2007	30	122	198	34	173	226	21,67569861
06.06.2007	30	122	198	34	173	226	4,181742668
06.06.2007	30	122	198	34	173	226	22,40214761
07.06.2007	30	122	198	34	173	226	109,9003914
07.06.2007	30	122	198	34	173	226	12,05467917
07.06.2007	30	122	198	34	173	226	13,80791708
11.06.2007	30	122	198	34	173	226	109,3752686
12.06.2007	30	122	198	34	173	226	113,2359317
11.06.2007	30	122	198	34	173	226	103,935706
12.06.2007	30	122	198	34	173	226	62,71411492

11.06.2007	30	122	198	34	173	226	94,61964863
12.06.2007	30	122	198	34	173	226	78,47313863
11.06.2007	30	122	198	34	173	226	74,67192358
12.06.2007	30	122	198	34	173	226	13,30825999
11.06.2007	30	122	198	34	173	226	67,04975169
12.06.2007	30	122	198	34	173	226	8,301697352
12.06.2007	30	122	198	34	173	226	92,19527619
13.06.2007	30	122	198	34	173	226	19,22667909
12.06.2007	30	122	198	34	173	226	94,99968107
13.06.2007	30	122	198	34	173	226	76,7357572
12.06.2007	30	122	198	34	173	226	114,6575549
13.06.2007	30	122	198	34	173	226	87,77400302
19.06.2007	30	122	198	34	173	226	102,2347894
18.06.2007	30	122	198	34	173	226	33,8025832
18.06.2007	30	122	198	34	173	226	78,89111703
19.06.2007	30	122	198	34	173	226	94,32253576
18.06.2007	30	122	198	34	173	226	88,77787926
19.06.2007	30	122	198	34	173	226	39,44773383
18.06.2007	30	122	198	34	173	226	123,0051915
19.06.2007	30	122	198	34	173	226	22,10401402
18.06.2007	30	122	198	34	173	226	118,9631292
19.06.2007	30	122	198	34	173	226	22,34542632
18.06.2007	30	122	198	34	173	226	104,3244003
19.06.2007	30	122	198	34	173	226	115,9547851
19.06.2007	30	122	198	34	173	226	119,1979298
20.06.2007	30	122	198	34	173	226	13,76565938
19.06.2007	30	122	198	34	173	226	156,554148
20.06.2007	30	122	198	34	173	226	150,3459282
25.06.2007	30	122	198	34	173	226	68,83912276
25.06.2007	30	122	198	34	173	226	86,45888311
26.06.2007	30	122	198	34	173	226	78,9874618
25.06.2007	30	122	198	34	173	226	90,7116074
26.06.2007	30	122	198	34	173	226	88,69105003

25.06.2007	30	122	198	34	173	226	89,07508177
26.06.2007	30	122	198	34	173	226	92,27919972
26.06.2007	30	122	198	34	173	226	91,91882649
25.06.2007	30	122	198	34	173	226	99,57021538
26.06.2007	30	122	198	34	173	226	93,55981892
26.06.2007	30	122	198	34	173	226	101,2217762
27.06.2007	30	122	198	34	173	226	78,77103636
26.06.2007	30	122	198	34	173	226	90,57987479
27.06.2007	30	122	198	34	173	226	76,88122596
26.06.2007	30	122	198	34	173	226	89,06078273
30.05.2007	33	162	220	39	83	181	282,8054835
30.05.2007	31	191	228	34	173	226	806,393299
30.05.2007	31	191	228	34	173	226	804,8341496
30.05.2007	31	191	228	34	173	226	800,3003248
30.05.2007	31	191	228	34	173	226	763,8593593
30.05.2007	31	191	228	34	173	226	803,4588497
31.05.2007	31	191	228	34	173	226	732,8460953
31.05.2007	31	191	228	34	173	226	774,6724879
31.05.2007	31	191	228	34	173	226	772,2541901
06.06.2007	31	191	228	34	173	226	824,8055683
06.06.2007	31	191	228	34	173	226	722,2045726
06.06.2007	31	191	228	34	173	226	771,7035653
06.06.2007	31	191	228	34	173	226	742,2768167
06.06.2007	31	191	228	34	173	226	759,3044846
07.06.2007	31	191	228	34	173	226	737,884552
07.06.2007	31	191	228	34	173	226	791,397728
07.06.2007	31	191	228	34	173	226	740,7668029
07.06.2007	31	191	228	34	173	226	758,0001388
11.06.2007	31	191	228	34	173	226	753,9074012
11.06.2007	31	191	228	34	173	226	703,6208957
12.06.2007	31	191	228	34	173	226	768,6516858
11.06.2007	31	191	228	34	173	226	778,618509
12.06.2007	31	191	228	34	173	226	748,416967

11.06.2007	31	191	228	34	173	226	761,4852949
12.06.2007	31	191	228	34	173	226	1348,959422
11.06.2007	31	191	228	34	173	226	817,3097135
12.06.2007	31	191	228	34	173	226	1355,133063
12.06.2007	31	191	228	34	173	226	779,5199944
13.06.2007	31	191	228	34	173	226	774,1391891
12.06.2007	31	191	228	34	173	226	783,6831304
13.06.2007	31	191	228	34	173	226	715,8948404
12.06.2007	31	191	228	34	173	226	744,5155619
13.06.2007	31	191	228	34	173	226	767,3324764
19.06.2007	31	191	228	34	173	226	715,4433081
18.06.2007	31	191	228	34	173	226	815,1449454
18.06.2007	31	191	228	34	173	226	698,514875
19.06.2007	31	191	228	34	173	226	773,9799709
18.06.2007	31	191	228	34	173	226	607,681993
19.06.2007	31	191	228	34	173	226	802,389204
18.06.2007	31	191	228	34	173	226	644,6161387
19.06.2007	31	191	228	34	173	226	708,640567
18.06.2007	31	191	228	34	173	226	749,6943174
19.06.2007	31	191	228	34	173	226	104,1788424
19.06.2007	31	191	228	34	173	226	819,6678808
19.06.2007	31	191	228	34	173	226	721,2151947
20.06.2007	31	191	228	34	173	226	924,4961411
19.06.2007	31	191	228	34	173	226	728,4582152
20.06.2007	31	191	228	34	173	226	39,66454932
25.06.2007	31	191	228	34	173	226	600,100318
25.06.2007	31	191	228	34	173	226	583,2635062
26.06.2007	31	191	228	34	173	226	577,3302498
25.06.2007	31	191	228	34	173	226	575,8029265
26.06.2007	31	191	228	34	173	226	566,97924
25.06.2007	31	191	228	34	173	226	575,8272563
26.06.2007	31	191	228	34	173	226	576,5405746
25.06.2007	31	191	228	34	173	226	583,5102264

26.06.2007	31	191	228	34	173	226	572,2930131
25.06.2007	31	191	228	34	173	226	575,0804395
26.06.2007	31	191	228	34	173	226	571,5157227
27.06.2007	31	191	228	34	173	226	576,4502094
26.06.2007	31	191	228	34	173	226	570,808808
27.06.2007	31	191	228	34	173	226	579,6813298
26.06.2007	31	191	228	34	173	226	566,4377508
27.06.2007	31	191	228	34	173	226	79,02004594
30.05.2007	36	128	206	39	83	181	355,8325498
30.05.2007	32	194	230	34	173	226	135,6921299
30.05.2007	32	194	230	34	173	226	136,7987426
30.05.2007	32	194	230	34	173	226	140,3949239
30.05.2007	32	194	230	34	173	226	192,4605761
30.05.2007	32	194	230	34	173	226	243,3167608
31.05.2007	32	194	230	34	173	226	186,2970228
31.05.2007	32	194	230	34	173	226	67,29609484
31.05.2007	32	194	230	34	173	226	212,6843268
06.06.2007	32	194	230	34	173	226	167,5074608
06.06.2007	32	194	230	34	173	226	201,572776
06.06.2007	32	194	230	34	173	226	206,3217039
06.06.2007	32	194	230	34	173	226	214,9837485
06.06.2007	32	194	230	34	173	226	184,0882579
07.06.2007	32	194	230	34	173	226	220,0897723
07.06.2007	32	194	230	34	173	226	1,914995
07.06.2007	32	194	230	34	173	226	218,1927631
07.06.2007	32	194	230	34	173	226	195,0414411
11.06.2007	32	194	230	34	173	226	181,6668087
12.06.2007	32	194	230	34	173	226	206,441295
11.06.2007	32	194	230	34	173	226	212,1834076
12.06.2007	32	194	230	34	173	226	208,9354903
11.06.2007	32	194	230	34	173	226	176,1308022
12.06.2007	32	194	230	34	173	226	190,8526865
11.06.2007	32	194	230	34	173	226	181,7354097

12.06.2007	32	194	230	34	173	226	67,93091722
11.06.2007	32	194	230	34	173	226	188,9865853
12.06.2007	32	194	230	34	173	226	121,8931764
12.06.2007	32	194	230	34	173	226	208,3566444
13.06.2007	32	194	230	34	173	226	141,7669204
12.06.2007	32	194	230	34	173	226	194,9020884
13.06.2007	32	194	230	34	173	226	187,338035
12.06.2007	32	194	230	34	173	226	234,4089028
13.06.2007	32	194	230	34	173	226	189,0982959
19.06.2007	32	194	230	34	173	226	212,0989118
18.06.2007	32	194	230	34	173	226	100,8997054
18.06.2007	32	194	230	34	173	226	194,5494862
19.06.2007	32	194	230	34	173	226	211,3566951
18.06.2007	32	194	230	34	173	226	213,7351151
19.06.2007	32	194	230	34	173	226	117,6794894
18.06.2007	32	194	230	34	173	226	251,5613098
19.06.2007	32	194	230	34	173	226	138,2600572
18.06.2007	32	194	230	34	173	226	236,5063414
19.06.2007	32	194	230	34	173	226	149,8836661
18.06.2007	32	194	230	34	173	226	200,2672361
19.06.2007	32	194	230	34	173	226	94,81647629
19.06.2007	32	194	230	34	173	226	224,7137477
20.06.2007	32	194	230	34	173	226	93,0003217
19.06.2007	32	194	230	34	173	226	213,8139584
20.06.2007	32	194	230	34	173	226	147,3120639
25.06.2007	32	194	230	34	173	226	208,7057848
25.06.2007	32	194	230	34	173	226	191,5352024
26.06.2007	32	194	230	34	173	226	202,9412067
25.06.2007	32	194	230	34	173	226	230,3095777
26.06.2007	32	194	230	34	173	226	205,4410956
25.06.2007	32	194	230	34	173	226	227,4142282
26.06.2007	32	194	230	34	173	226	191,800333
25.06.2007	32	194	230	34	173	226	221,552305

26.06.2007	32	194	230	34	173	226	203,5894333
25.06.2007	32	194	230	34	173	226	228,4269172
26.06.2007	32	194	230	34	173	226	224,1557616
26.06.2007	32	194	230	34	173	226	217,0769516
27.06.2007	32	194	230	34	173	226	195,1109181
26.06.2007	32	194	230	34	173	226	218,9136601
27.06.2007	32	194	230	34	173	226	190,361057
26.06.2007	32	194	230	34	173	226	211,8286354
27.06.2007	32	194	230	34	173	226	216,6796931
30.05.2007	30	122	198	33	162	220	377,7497608
30.05.2007	33	162	220	34	173	226	397,5376079
30.05.2007	33	162	220	34	173	226	350,1143899
30.05.2007	33	162	220	34	173	226	339,8917419
30.05.2007	33	162	220	34	173	226	270,5438733
30.05.2007	33	162	220	34	173	226	275,9840523
31.05.2007	33	162	220	34	173	226	268,120693
31.05.2007	33	162	220	34	173	226	306,3015333
31.05.2007	33	162	220	34	173	226	278,3530783
06.06.2007	33	162	220	34	173	226	79,8820186
06.06.2007	33	162	220	34	173	226	67,4012417
06.06.2007	33	162	220	34	173	226	23,14014368
06.06.2007	33	162	220	34	173	226	12,19621458
06.06.2007	33	162	220	34	173	226	86,30559136
07.06.2007	33	162	220	34	173	226	63,16009313
07.06.2007	33	162	220	34	173	226	83,80219326
07.06.2007	33	162	220	34	173	226	57,14354615
07.06.2007	33	162	220	34	173	226	70,75492826
11.06.2007	33	162	220	34	173	226	126,0263522
12.06.2007	33	162	220	34	173	226	63,9536756
11.06.2007	33	162	220	34	173	226	55,49173706
12.06.2007	33	162	220	34	173	226	95,99545972
11.06.2007	33	162	220	34	173	226	79,480106
12.06.2007	33	162	220	34	173	226	77,7329862

11.06.2007	33	162	220	34	173	226	100,9055241
12.06.2007	33	162	220	34	173	226	138,4249824
11.06.2007	33	162	220	34	173	226	78,86122854
12.06.2007	33	162	220	34	173	226	130,5381358
12.06.2007	33	162	220	34	173	226	84,27264286
13.06.2007	33	162	220	34	173	226	115,5529974
12.06.2007	33	162	220	34	173	226	89,71326028
13.06.2007	33	162	220	34	173	226	98,17135882
12.06.2007	33	162	220	34	173	226	64,14391927
13.06.2007	33	162	220	34	173	226	97,35507663
19.06.2007	33	162	220	34	173	226	70,26763416
18.06.2007	33	162	220	34	173	226	98,31092738
18.06.2007	33	162	220	34	173	226	82,18909522
19.06.2007	33	162	220	34	173	226	68,77598077
18.06.2007	33	162	220	34	173	226	68,01529845
19.06.2007	33	162	220	34	173	226	115,3783167
18.06.2007	33	162	220	34	173	226	54,64248409
19.06.2007	33	162	220	34	173	226	86,29533432
18.06.2007	33	162	220	34	173	226	38,68263748
19.06.2007	33	162	220	34	173	226	83,93326376
18.06.2007	33	162	220	34	173	226	64,92846861
19.06.2007	33	162	220	34	173	226	23,49093773
19.06.2007	33	162	220	34	173	226	45,28245786
20.06.2007	33	162	220	34	173	226	102,743265
19.06.2007	33	162	220	34	173	226	53,36082454
20.06.2007	33	162	220	34	173	226	148,4853256
25.06.2007	33	162	220	34	173	226	63,62807581
25.06.2007	33	162	220	34	173	226	52,22493375
26.06.2007	33	162	220	34	173	226	62,28336309
25.06.2007	33	162	220	34	173	226	39,66010847
26.06.2007	33	162	220	34	173	226	53,22760997
25.06.2007	33	162	220	34	173	226	60,66177865
26.06.2007	33	162	220	34	173	226	55,99938119

25.06.2007	33	162	220	34	173	226	56,70998244
26.06.2007	33	162	220	34	173	226	61,2056539
25.06.2007	33	162	220	34	173	226	50,6991538
26.06.2007	33	162	220	34	173	226	54,83628792
26.06.2007	33	162	220	34	173	226	53,01591715
27.06.2007	33	162	220	34	173	226	58,96317213
26.06.2007	33	162	220	34	173	226	43,86013466
27.06.2007	33	162	220	34	173	226	59,48135579
26.06.2007	33	162	220	34	173	226	56,34678555
27.06.2007	33	162	220	34	173	226	52,28258219
25.06.2007	36	128	206	34	173	226	846,2732021
30.05.2007	33	162	220	34	173	226	378,2069809
30.05.2007	29	240	260	36	128	206	385,2741759
30.05.2007	29	240	260	36	128	206	382,9515468
30.05.2007	29	240	260	36	128	206	423,4180955
30.05.2007	29	240	260	36	128	206	403,0977591
30.05.2007	29	240	260	36	128	206	413,0323529
31.05.2007	29	240	260	36	128	206	455,0724406
31.05.2007	29	240	260	36	128	206	444,1413829
31.05.2007	29	240	260	36	128	206	421,3040028
06.06.2007	29	240	260	36	128	206	1053,416037
06.06.2007	29	240	260	36	128	206	982,6940587
06.06.2007	29	240	260	36	128	206	973,4736739
06.06.2007	29	240	260	36	128	206	999,561445
06.06.2007	29	240	260	36	128	206	962,0706254
07.06.2007	29	240	260	36	128	206	959,8666498
07.06.2007	29	240	260	36	128	206	949,4965139
07.06.2007	29	240	260	36	128	206	978,5456005
07.06.2007	29	240	260	36	128	206	986,8546371
11.06.2007	29	240	260	36	128	206	951,244083
12.06.2007	29	240	260	36	128	206	917,2573085
11.06.2007	29	240	260	36	128	206	896,5916412
12.06.2007	29	240	260	36	128	206	954,4070983

11.06.2007	29	240	260	36	128	206	957,1066585
12.06.2007	29	240	260	36	128	206	881,8121602
11.06.2007	29	240	260	36	128	206	956,7218544
12.06.2007	29	240	260	36	128	206	855,5570078
11.06.2007	29	240	260	36	128	206	1053,37794
12.06.2007	29	240	260	36	128	206	940,1528154
12.06.2007	29	240	260	36	128	206	961,8639688
13.06.2007	29	240	260	36	128	206	948,4434822
12.06.2007	29	240	260	36	128	206	960,4367803
13.06.2007	29	240	260	36	128	206	945,5989486
12.06.2007	29	240	260	36	128	206	906,1417965
13.06.2007	29	240	260	36	128	206	928,3696112
19.06.2007	29	240	260	36	128	206	979,8460657
18.06.2007	29	240	260	36	128	206	927,4663857
18.06.2007	29	240	260	36	128	206	1026,733271
19.06.2007	29	240	260	36	128	206	975,3673778
18.06.2007	29	240	260	36	128	206	978,7951261
19.06.2007	29	240	260	36	128	206	989,765064
18.06.2007	29	240	260	36	128	206	955,1831258
19.06.2007	29	240	260	36	128	206	891,5860527
18.06.2007	29	240	260	36	128	206	1017,873677
19.06.2007	29	240	260	36	128	206	871,9373538
18.06.2007	29	240	260	36	128	206	949,3933117
19.06.2007	29	240	260	36	128	206	1452,59153
19.06.2007	29	240	260	36	128	206	931,4672722
20.06.2007	29	240	260	36	128	206	1439,274601
19.06.2007	29	240	260	36	128	206	944,5070203
20.06.2007	29	240	260	36	128	206	988,2707148
25.06.2007	29	240	260	36	128	206	946,5945062
25.06.2007	29	240	260	36	128	206	998,5226216
26.06.2007	29	240	260	36	128	206	950,5029849
25.06.2007	29	240	260	36	128	206	978,8410631
26.06.2007	29	240	260	36	128	206	920,0877782

25.06.2007	29	240	260	36	128	206	984,7823396
26.06.2007	29	240	260	36	128	206	931,8018152
25.06.2007	29	240	260	36	128	206	984,0143992
26.06.2007	29	240	260	36	128	206	937,2393443
25.06.2007	29	240	260	36	128	206	969,5075987
26.06.2007	29	240	260	36	128	206	902,4962403
26.06.2007	29	240	260	36	128	206	928,3139084
27.06.2007	29	240	260	36	128	206	924,8657429
26.06.2007	29	240	260	36	128	206	945,8283305
27.06.2007	29	240	260	36	128	206	928,7649902
26.06.2007	29	240	260	36	128	206	951,6798831
27.06.2007	29	240	260	36	128	206	926,5122008
30.05.2007	29	240	260	33	162	220	419,5975684
30.05.2007	30	122	198	36	128	206	286,4851917
30.05.2007	30	122	198	36	128	206	272,8908009
30.05.2007	30	122	198	36	128	206	313,2985913
30.05.2007	30	122	198	36	128	206	302,771932
30.05.2007	30	122	198	36	128	206	297,7967748
31.05.2007	30	122	198	36	128	206	284,802668
31.05.2007	30	122	198	36	128	206	313,2094205
31.05.2007	30	122	198	36	128	206	416,6319105
06.06.2007	30	122	198	36	128	206	850,7242432
06.06.2007	30	122	198	36	128	206	829,8516426
06.06.2007	30	122	198	36	128	206	822,5133078
06.06.2007	30	122	198	36	128	206	820,0832198
06.06.2007	30	122	198	36	128	206	818,9468065
07.06.2007	30	122	198	36	128	206	929,5061424
07.06.2007	30	122	198	36	128	206	845,6652141
07.06.2007	30	122	198	36	128	206	825,2722706
11.06.2007	30	122	198	36	128	206	943,4275002
12.06.2007	30	122	198	36	128	206	923,3451038
11.06.2007	30	122	198	36	128	206	883,1459609
12.06.2007	30	122	198	36	128	206	945,445232

11.06.2007	30	122	198	36	128	206	950,54397
12.06.2007	30	122	198	36	128	206	873,4715713
11.06.2007	30	122	198	36	128	206	940,9954967
12.06.2007	30	122	198	36	128	206	841,4806622
11.06.2007	30	122	198	36	128	206	979,0415944
12.06.2007	30	122	198	36	128	206	918,0245124
12.06.2007	30	122	198	36	128	206	947,0880682
13.06.2007	30	122	198	36	128	206	929,6385249
12.06.2007	30	122	198	36	128	206	924,0291388
13.06.2007	30	122	198	36	128	206	941,13659
12.06.2007	30	122	198	36	128	206	903,6541679
13.06.2007	30	122	198	36	128	206	937,7455119
19.06.2007	30	122	198	36	128	206	942,9391874
18.06.2007	30	122	198	36	128	206	843,1074829
18.06.2007	30	122	198	36	128	206	923,458778
19.06.2007	30	122	198	36	128	206	932,1354727
18.06.2007	30	122	198	36	128	206	924,2793977
19.06.2007	30	122	198	36	128	206	950,952071
18.06.2007	30	122	198	36	128	206	915,2769128
19.06.2007	30	122	198	36	128	206	855,4955927
18.06.2007	30	122	198	36	128	206	966,766942
19.06.2007	30	122	198	36	128	206	783,0964063
18.06.2007	30	122	198	36	128	206	942,1058333
19.06.2007	30	122	198	36	128	206	1350,507735
19.06.2007	30	122	198	36	128	206	926,4778302
20.06.2007	30	122	198	36	128	206	1331,68388
19.06.2007	30	122	198	36	128	206	1000,284961
20.06.2007	30	122	198	36	128	206	884,3264731
25.06.2007	30	122	198	36	128	206	930,4004608
26.06.2007	30	122	198	36	128	206	917,7054195
25.06.2007	30	122	198	36	128	206	922,9118671
26.06.2007	30	122	198	36	128	206	901,7984565
25.06.2007	30	122	198	36	128	206	930,0696092

26.06.2007	30	122	198	36	128	206	919,8380089
26.06.2007	30	122	198	36	128	206	918,0595832
25.06.2007	30	122	198	36	128	206	923,4794552
26.06.2007	30	122	198	36	128	206	911,6859036
26.06.2007	30	122	198	36	128	206	911,7548027
27.06.2007	30	122	198	36	128	206	905,5570975
26.06.2007	30	122	198	36	128	206	928,0947329
27.06.2007	30	122	198	36	128	206	910,0651715
26.06.2007	30	122	198	36	128	206	922,2794795
30.05.2007	36	128	206	37	217	230	429,7999824
30.05.2007	31	191	228	36	128	206	460,408187
30.05.2007	31	191	228	36	128	206	455,8298066
30.05.2007	31	191	228	36	128	206	438,4126656
30.05.2007	31	191	228	36	128	206	462,8828865
30.05.2007	31	191	228	36	128	206	509,1306029
31.05.2007	31	191	228	36	128	206	434,8068316
31.05.2007	31	191	228	36	128	206	423,8813846
31.05.2007	31	191	228	36	128	206	451,6789652
06.06.2007	31	191	228	36	128	206	93,62269542
06.06.2007	31	191	228	36	128	206	120,5468667
06.06.2007	31	191	228	36	128	206	71,71542828
06.06.2007	31	191	228	36	128	206	82,43276547
06.06.2007	31	191	228	36	128	206	82,7377497
07.06.2007	31	191	228	36	128	206	67,27475037
07.06.2007	31	191	228	36	128	206	31,65717779
07.06.2007	31	191	228	36	128	206	93,78054501
07.06.2007	31	191	228	36	128	206	81,00407999
11.06.2007	31	191	228	36	128	206	80,4246239
12.06.2007	31	191	228	36	128	206	65,09429067
11.06.2007	31	191	228	36	128	206	80,36193022
12.06.2007	31	191	228	36	128	206	119,318431
11.06.2007	31	191	228	36	128	206	82,0864951
12.06.2007	31	191	228	36	128	206	51,21744246

11.06.2007	31	191	228	36	128	206	110,0102633
12.06.2007	31	191	228	36	128	206	612,8753225
11.06.2007	31	191	228	36	128	206	116,1756463
12.06.2007	31	191	228	36	128	206	556,4380561
12.06.2007	31	191	228	36	128	206	78,39678792
13.06.2007	31	191	228	36	128	206	137,9239178
12.06.2007	31	191	228	36	128	206	48,28273903
13.06.2007	31	191	228	36	128	206	156,03049
12.06.2007	31	191	228	36	128	206	47,9101432
13.06.2007	31	191	228	36	128	206	92,43186815
19.06.2007	31	191	228	36	128	206	128,2850222
18.06.2007	31	191	228	36	128	206	5,816858698
18.06.2007	31	191	228	36	128	206	153,7469429
19.06.2007	31	191	228	36	128	206	66,82818555
18.06.2007	31	191	228	36	128	206	235,1661673
19.06.2007	31	191	228	36	128	206	112,2550924
18.06.2007	31	191	228	36	128	206	149,9675597
19.06.2007	31	191	228	36	128	206	130,6174779
18.06.2007	31	191	228	36	128	206	100,8859632
19.06.2007	31	191	228	36	128	206	657,5536983
19.06.2007	31	191	228	36	128	206	550,1631915
19.06.2007	31	191	228	36	128	206	89,84053296
20.06.2007	31	191	228	36	128	206	454,4153664
19.06.2007	31	191	228	36	128	206	118,9189694
20.06.2007	31	191	228	36	128	206	755,8407681
25.06.2007	31	191	228	36	128	206	289,8630395
26.06.2007	31	191	228	36	128	206	289,6531229
25.06.2007	31	191	228	36	128	206	280,5692798
26.06.2007	31	191	228	36	128	206	277,7094044
25.06.2007	31	191	228	36	128	206	294,6900961
26.06.2007	31	191	228	36	128	206	280,2554101
25.06.2007	31	191	228	36	128	206	287,2752538
26.06.2007	31	191	228	36	128	206	286,9240986

25.06.2007	31	191	228	36	128	206	278,994733
26.06.2007	31	191	228	36	128	206	276,4572925
27.06.2007	31	191	228	36	128	206	279,7932591
26.06.2007	31	191	228	36	128	206	298,2098416
27.06.2007	31	191	228	36	128	206	283,917919
26.06.2007	31	191	228	36	128	206	295,7776991
27.06.2007	31	191	228	36	128	206	903,0288343
30.05.2007	31	191	228	36	128	206	431,0873437
30.05.2007	32	194	230	36	128	206	484,2456235
30.05.2007	32	194	230	36	128	206	488,0199098
30.05.2007	32	194	230	36	128	206	508,604743
30.05.2007	32	194	230	36	128	206	495,8275366
30.05.2007	32	194	230	36	128	206	539,9523644
31.05.2007	32	194	230	36	128	206	485,8971223
31.05.2007	32	194	230	36	128	206	289,4203036
31.05.2007	32	194	230	36	128	206	536,8009766
06.06.2007	32	194	230	36	128	206	1085,604004
06.06.2007	32	194	230	36	128	206	1041,510707
06.06.2007	32	194	230	36	128	206	1048,064011
06.06.2007	32	194	230	36	128	206	1038,414015
06.06.2007	32	194	230	36	128	206	1024,056352
07.06.2007	32	194	230	36	128	206	1024,104991
07.06.2007	32	194	230	36	128	206	821,16016
07.06.2007	32	194	230	36	128	206	1051,516528
07.06.2007	32	194	230	36	128	206	1033,462251
11.06.2007	32	194	230	36	128	206	1015,977804
12.06.2007	32	194	230	36	128	206	1019,344105
11.06.2007	32	194	230	36	128	206	994,5940696
12.06.2007	32	194	230	36	128	206	1094,773274
11.06.2007	32	194	230	36	128	206	1033,334468
12.06.2007	32	194	230	36	128	206	989,5718127
11.06.2007	32	194	230	36	128	206	1050,074653
12.06.2007	32	194	230	36	128	206	921,4740222

11.06.2007	32	194	230	36	128	206	1104,155236
12.06.2007	32	194	230	36	128	206	1046,203652
12.06.2007	32	194	230	36	128	206	1065,663314
13.06.2007	32	194	230	36	128	206	1052,938543
12.06.2007	32	194	230	36	128	206	1026,438518
13.06.2007	32	194	230	36	128	206	1055,140537
12.06.2007	32	194	230	36	128	206	1024,781392
13.06.2007	32	194	230	36	128	206	1047,401779
19.06.2007	32	194	230	36	128	206	1054,865992
18.06.2007	32	194	230	36	128	206	712,4611033
18.06.2007	32	194	230	36	128	206	1043,358878
19.06.2007	32	194	230	36	128	206	1050,626949
18.06.2007	32	194	230	36	128	206	1051,811124
19.06.2007	32	194	230	36	128	206	1031,417886
18.06.2007	32	194	230	36	128	206	1046,063553
19.06.2007	32	194	230	36	128	206	973,059433
18.06.2007	32	194	230	36	128	206	1086,687778
19.06.2007	32	194	230	36	128	206	906,897075
18.06.2007	32	194	230	36	128	206	1039,195684
19.06.2007	32	194	230	36	128	206	1339,086245
19.06.2007	32	194	230	36	128	206	1032,448912
20.06.2007	32	194	230	36	128	206	1414,944692
19.06.2007	32	194	230	36	128	206	1059,124203
20.06.2007	32	194	230	36	128	206	875,7512889
25.06.2007	32	194	230	36	128	206	1035,663478
26.06.2007	32	194	230	36	128	206	1041,629107
25.06.2007	32	194	230	36	128	206	1063,17057
26.06.2007	32	194	230	36	128	206	1018,755185
25.06.2007	32	194	230	36	128	206	1069,816353
26.06.2007	32	194	230	36	128	206	1019,817631
25.06.2007	32	194	230	36	128	206	1064,974864
26.06.2007	32	194	230	36	128	206	1030,222159
25.06.2007	32	194	230	36	128	206	1053,195932

26.06.2007	32	194	230	36	128	206	1043,557363
26.06.2007	32	194	230	36	128	206	1028,131739
27.06.2007	32	194	230	36	128	206	1021,787218
26.06.2007	32	194	230	36	128	206	1057,130756
27.06.2007	32	194	230	36	128	206	1023,912394
26.06.2007	32	194	230	36	128	206	1044,628021
27.06.2007	32	194	230	36	128	206	1041,443987
30.05.2007	30	122	198	36	128	206	451,1084914
30.05.2007	33	162	220	36	128	206	57,2834226
30.05.2007	33	162	220	36	128	206	1,487605138
30.05.2007	33	162	220	36	128	206	29,07870281
30.05.2007	33	162	220	36	128	206	34,22336494
30.05.2007	33	162	220	36	128	206	22,25550798
31.05.2007	33	162	220	36	128	206	33,52056473
31.05.2007	33	162	220	36	128	206	51,65173075
31.05.2007	33	162	220	36	128	206	48,80688623
06.06.2007	33	162	220	36	128	206	838,4302377
06.06.2007	33	162	220	36	128	206	789,4668872
06.06.2007	33	162	220	36	128	206	821,0748747
06.06.2007	33	162	220	36	128	206	812,0745336
06.06.2007	33	162	220	36	128	206	766,1861045
07.06.2007	33	162	220	36	128	206	756,9060085
07.06.2007	33	162	220	36	128	206	760,0769017
07.06.2007	33	162	220	36	128	206	794,1812426
07.06.2007	33	162	220	36	128	206	784,4297463
11.06.2007	33	162	220	36	128	206	960,0649914
12.06.2007	33	162	220	36	128	206	778,9649045
11.06.2007	33	162	220	36	128	206	740,2383379
12.06.2007	33	162	220	36	128	206	808,0605793
11.06.2007	33	162	220	36	128	206	802,3033926
12.06.2007	33	162	220	36	128	206	734,8516554
11.06.2007	33	162	220	36	128	206	786,3044594
12.06.2007	33	162	220	36	128	206	715,7361626

11.06.2007	33	162	220	36	128	206	845,5491146
12.06.2007	33	162	220	36	128	206	794,3566008
12.06.2007	33	162	220	36	128	206	793,4828089
13.06.2007	33	162	220	36	128	206	801,0168386
12.06.2007	33	162	220	36	128	206	755,5324412
13.06.2007	33	162	220	36	128	206	788,4169533
12.06.2007	33	162	220	36	128	206	748,2293756
13.06.2007	33	162	220	36	128	206	786,6860329
19.06.2007	33	162	220	36	128	206	798,5785688
18.06.2007	33	162	220	36	128	206	713,4054454
18.06.2007	33	162	220	36	128	206	783,2756357
19.06.2007	33	162	220	36	128	206	792,9984115
18.06.2007	33	162	220	36	128	206	791,5082018
19.06.2007	33	162	220	36	128	206	801,3896261
18.06.2007	33	162	220	36	128	206	757,5402998
19.06.2007	33	162	220	36	128	206	750,6105653
18.06.2007	33	162	220	36	128	206	888,2786278
19.06.2007	33	162	220	36	128	206	678,6105937
18.06.2007	33	162	220	36	128	206	802,2026475
19.06.2007	33	162	220	36	128	206	1225,006412
19.06.2007	33	162	220	36	128	206	789,5276439
20.06.2007	33	162	220	36	128	206	1226,582513
19.06.2007	33	162	220	36	128	206	803,0739308
20.06.2007	33	162	220	36	128	206	881,1010353
25.06.2007	33	162	220	36	128	206	809,1643826
26.06.2007	33	162	220	36	128	206	801,3716372
25.06.2007	33	162	220	36	128	206	814,0142822
26.06.2007	33	162	220	36	128	206	782,5875231
25.06.2007	33	162	220	36	128	206	803,5365398
26.06.2007	33	162	220	36	128	206	794,0485895
25.06.2007	33	162	220	36	128	206	807,883936
26.06.2007	33	162	220	36	128	206	790,5757086
25.06.2007	33	162	220	36	128	206	799,4822958

26.06.2007	33	162	220	36	128	206	787,7193139
26.06.2007	33	162	220	36	128	206	781,6623567
27.06.2007	33	162	220	36	128	206	792,2757862
26.06.2007	33	162	220	36	128	206	813,0754786
27.06.2007	33	162	220	36	128	206	798,6900738
26.06.2007	33	162	220	36	128	206	799,2758213
27.06.2007	33	162	220	36	128	206	788,0640817
30.05.2007	34	173	226	36	128	206	451,7505312
30.05.2007	34	173	226	36	128	206	348,7592012
30.05.2007	34	173	226	36	128	206	351,5005109
30.05.2007	34	173	226	36	128	206	368,724681
30.05.2007	34	173	226	36	128	206	303,4696624
30.05.2007	34	173	226	36	128	206	296,786367
31.05.2007	34	173	226	36	128	206	300,1320967
31.05.2007	34	173	226	36	128	206	354,0808402
31.05.2007	34	173	226	36	128	206	324,1189042
06.06.2007	34	173	226	36	128	206	918,1087017
06.06.2007	34	173	226	36	128	206	840,7814485
06.06.2007	34	173	226	36	128	206	843,3377944
06.06.2007	34	173	226	36	128	206	824,2626094
06.06.2007	34	173	226	36	128	206	841,3389389
07.06.2007	34	173	226	36	128	206	804,3341064
07.06.2007	34	173	226	36	128	206	822,898129
07.06.2007	34	173	226	36	128	206	833,9763619
07.06.2007	34	173	226	36	128	206	838,8993844
11.06.2007	34	173	226	36	128	206	834,3205168
12.06.2007	34	173	226	36	128	206	816,5077909
11.06.2007	34	173	226	36	128	206	782,4668089
12.06.2007	34	173	226	36	128	206	887,9475587
11.06.2007	34	173	226	36	128	206	860,6290113
12.06.2007	34	173	226	36	128	206	799,0953427
11.06.2007	34	173	226	36	128	206	870,6471516
12.06.2007	34	173	226	36	128	206	853,8714292

11.06.2007	34	173	226	36	128	206	915,2457077
12.06.2007	34	173	226	36	128	206	924,3135471
12.06.2007	34	173	226	36	128	206	857,7674648
13.06.2007	34	173	226	36	128	206	911,4886488
12.06.2007	34	173	226	36	128	206	831,8467474
13.06.2007	34	173	226	36	128	206	869,3403277
12.06.2007	34	173	226	36	128	206	790,6154275
13.06.2007	34	173	226	36	128	206	859,5523951
19.06.2007	34	173	226	36	128	206	843,4329139
18.06.2007	34	173	226	36	128	206	809,6742719
18.06.2007	34	173	226	36	128	206	849,5993179
19.06.2007	34	173	226	36	128	206	840,3934341
18.06.2007	34	173	226	36	128	206	838,4348378
19.06.2007	34	173	226	36	128	206	913,9115271
18.06.2007	34	173	226	36	128	206	794,5315281
19.06.2007	34	173	226	36	128	206	835,3335433
18.06.2007	34	173	226	36	128	206	850,4531098
19.06.2007	34	173	226	36	128	206	761,7043494
18.06.2007	34	173	226	36	128	206	840,6897626
19.06.2007	34	173	226	36	128	206	1246,729413
19.06.2007	34	173	226	36	128	206	808,2675812
20.06.2007	34	173	226	36	128	206	1322,181397
19.06.2007	34	173	226	36	128	206	846,3709852
20.06.2007	34	173	226	36	128	206	736,9770378
25.06.2007	34	173	226	36	128	206	844,9534282
26.06.2007	34	173	226	36	128	206	840,0962753
25.06.2007	34	173	226	36	128	206	833,0005212
26.06.2007	34	173	226	36	128	206	814,2826403
25.06.2007	34	173	226	36	128	206	842,5452932
26.06.2007	34	173	226	36	128	206	829,8256935
25.06.2007	34	173	226	36	128	206	844,456193
26.06.2007	34	173	226	36	128	206	828,4576357
25.06.2007	34	173	226	36	128	206	825,2442293

26.06.2007	34	173	226	36	128	206	819,9098441
26.06.2007	34	173	226	36	128	206	812,0271492
27.06.2007	34	173	226	36	128	206	829,3868746
26.06.2007	34	173	226	36	128	206	838,9525561
27.06.2007	34	173	226	36	128	206	835,4602738
26.06.2007	34	173	226	36	128	206	833,3946222
27.06.2007	34	173	226	36	128	206	825,4877914
30.05.2007	29	240	260	36	128	206	493,3246423
30.05.2007	29	240	260	37	217	230	909,5983567
30.05.2007	29	240	260	37	217	230	902,0430471
30.05.2007	29	240	260	37	217	230	908,1121352
30.05.2007	29	240	260	37	217	230	906,6822562
30.05.2007	29	240	260	37	217	230	937,0869216
31.05.2007	29	240	260	37	217	230	1385,381443
31.05.2007	29	240	260	37	217	230	1393,916794
31.05.2007	29	240	260	37	217	230	1311,198434
06.06.2007	29	240	260	37	217	230	1470,915469
06.06.2007	29	240	260	37	217	230	1395,164322
06.06.2007	29	240	260	37	217	230	1420,730713
06.06.2007	29	240	260	37	217	230	1423,130586
06.06.2007	29	240	260	37	217	230	1400,276372
07.06.2007	29	240	260	37	217	230	1386,304605
07.06.2007	29	240	260	37	217	230	1382,04756
07.06.2007	29	240	260	37	217	230	1395,037682
07.06.2007	29	240	260	37	217	230	1397,781962
11.06.2007	29	240	260	37	217	230	1345,097834
12.06.2007	29	240	260	37	217	230	1328,575139
11.06.2007	29	240	260	37	217	230	1368,04431
12.06.2007	29	240	260	37	217	230	1361,595287
11.06.2007	29	240	260	37	217	230	1418,778485
12.06.2007	29	240	260	37	217	230	1367,908503
11.06.2007	29	240	260	37	217	230	1372,747378
12.06.2007	29	240	260	37	217	230	1353,34042

11.06.2007	29	240	260	37	217	230	1454,845017
12.06.2007	29	240	260	37	217	230	1366,164373
12.06.2007	29	240	260	37	217	230	1360,271918
13.06.2007	29	240	260	37	217	230	1376,092204
12.06.2007	29	240	260	37	217	230	1371,912693
13.06.2007	29	240	260	37	217	230	1352,704475
12.06.2007	29	240	260	37	217	230	1331,633157
13.06.2007	29	240	260	37	217	230	1347,478817
19.06.2007	29	240	260	37	217	230	1375,16508
18.06.2007	29	240	260	37	217	230	1468,186837
18.06.2007	29	240	260	37	217	230	1444,798347
19.06.2007	29	240	260	37	217	230	1385,831327
18.06.2007	29	240	260	37	217	230	1395,193462
19.06.2007	29	240	260	37	217	230	1364,002413
18.06.2007	29	240	260	37	217	230	1405,060593
19.06.2007	29	240	260	37	217	230	1378,651801
18.06.2007	29	240	260	37	217	230	1389,803879
19.06.2007	29	240	260	37	217	230	1453,182563
18.06.2007	29	240	260	37	217	230	1360,735925
19.06.2007	29	240	260	37	217	230	1458,020554
19.06.2007	29	240	260	37	217	230	1343,565552
20.06.2007	29	240	260	37	217	230	1466,139819
19.06.2007	29	240	260	37	217	230	1363,748688
20.06.2007	29	240	260	37	217	230	1457,893072
25.06.2007	29	240	260	37	217	230	1362,113625
25.06.2007	29	240	260	37	217	230	1399,515403
26.06.2007	29	240	260	37	217	230	1350,877645
25.06.2007	29	240	260	37	217	230	1362,000004
26.06.2007	29	240	260	37	217	230	1350,252421
25.06.2007	29	240	260	37	217	230	1397,268174
26.06.2007	29	240	260	37	217	230	1347,332899
25.06.2007	29	240	260	37	217	230	1378,273165
26.06.2007	29	240	260	37	217	230	1351,547618

25.06.2007	29	240	260	37	217	230	1383,692258
26.06.2007	29	240	260	37	217	230	1320,169893
26.06.2007	29	240	260	37	217	230	1355,040884
27.06.2007	29	240	260	37	217	230	1344,672718
26.06.2007	29	240	260	37	217	230	1343,608006
27.06.2007	29	240	260	37	217	230	1337,231418
26.06.2007	29	240	260	37	217	230	1348,913896
27.06.2007	29	240	260	37	217	230	1340,947037
30.05.2007	33	162	220	37	217	230	495,6851497
30.05.2007	30	122	198	37	217	230	813,0281192
30.05.2007	30	122	198	37	217	230	793,9639642
30.05.2007	30	122	198	37	217	230	801,33071
30.05.2007	30	122	198	37	217	230	808,4209235
30.05.2007	30	122	198	37	217	230	824,9397686
31.05.2007	30	122	198	37	217	230	1222,609339
31.05.2007	30	122	198	37	217	230	1269,48165
31.05.2007	30	122	198	37	217	230	1308,705365
06.06.2007	30	122	198	37	217	230	1291,511135
06.06.2007	30	122	198	37	217	230	1259,127545
06.06.2007	30	122	198	37	217	230	1289,061893
06.06.2007	30	122	198	37	217	230	1261,507096
06.06.2007	30	122	198	37	217	230	1274,180359
07.06.2007	30	122	198	37	217	230	1367,953681
07.06.2007	30	122	198	37	217	230	1281,400769
07.06.2007	30	122	198	37	217	230	1256,111384
11.06.2007	30	122	198	37	217	230	1338,357171
12.06.2007	30	122	198	37	217	230	1335,016718
11.06.2007	30	122	198	37	217	230	1356,187255
12.06.2007	30	122	198	37	217	230	1355,414295
11.06.2007	30	122	198	37	217	230	1413,656684
12.06.2007	30	122	198	37	217	230	1357,798814
11.06.2007	30	122	198	37	217	230	1359,500645
12.06.2007	30	122	198	37	217	230	1338,999751

11.06.2007	30	122	198	37	217	230	1388,715901
12.06.2007	30	122	198	37	217	230	1348,797505
12.06.2007	30	122	198	37	217	230	1347,613275
13.06.2007	30	122	198	37	217	230	1358,355833
12.06.2007	30	122	198	37	217	230	1336,078511
13.06.2007	30	122	198	37	217	230	1348,249722
12.06.2007	30	122	198	37	217	230	1329,540501
13.06.2007	30	122	198	37	217	230	1354,356373
19.06.2007	30	122	198	37	217	230	1342,130806
18.06.2007	30	122	198	37	217	230	1381,717371
18.06.2007	30	122	198	37	217	230	1343,471847
19.06.2007	30	122	198	37	217	230	1347,410174
18.06.2007	30	122	198	37	217	230	1343,29783
19.06.2007	30	122	198	37	217	230	1327,867952
18.06.2007	30	122	198	37	217	230	1367,19682
19.06.2007	30	122	198	37	217	230	1344,904676
18.06.2007	30	122	198	37	217	230	1341,684183
19.06.2007	30	122	198	37	217	230	1369,9103
18.06.2007	30	122	198	37	217	230	1355,368549
19.06.2007	30	122	198	37	217	230	1356,046188
19.06.2007	30	122	198	37	217	230	1338,9679
20.06.2007	30	122	198	37	217	230	1360,980213
19.06.2007	30	122	198	37	217	230	1416,213833
20.06.2007	30	122	198	37	217	230	1357,965827
25.06.2007	30	122	198	37	217	230	1332,420795
25.06.2007	30	122	198	37	217	230	1340,687904
26.06.2007	30	122	198	37	217	230	1323,534141
25.06.2007	30	122	198	37	217	230	1311,191828
26.06.2007	30	122	198	37	217	230	1334,305014
25.06.2007	30	122	198	37	217	230	1349,124774
26.06.2007	30	122	198	37	217	230	1336,825142
26.06.2007	30	122	198	37	217	230	1336,796656
25.06.2007	30	122	198	37	217	230	1345,091042

26.06.2007	30	122	198	37	217	230	1329,247282
26.06.2007	30	122	198	37	217	230	1344,548477
27.06.2007	30	122	198	37	217	230	1326,631678
26.06.2007	30	122	198	37	217	230	1328,83331
27.06.2007	30	122	198	37	217	230	1319,844095
26.06.2007	30	122	198	37	217	230	1325,20608
30.05.2007	31	191	228	33	162	220	496,9956785
30.05.2007	31	191	228	37	217	230	67,60336268
30.05.2007	31	191	228	37	217	230	67,17451531
30.05.2007	31	191	228	37	217	230	54,7306018
30.05.2007	31	191	228	37	217	230	46,37319264
30.05.2007	31	191	228	37	217	230	20,7602267
31.05.2007	31	191	228	37	217	230	546,7162243
31.05.2007	31	191	228	37	217	230	631,4931357
31.05.2007	31	191	228	37	217	230	543,6447405
06.06.2007	31	191	228	37	217	230	651,1304913
06.06.2007	31	191	228	37	217	230	650,0951865
06.06.2007	31	191	228	37	217	230	656,5754494
06.06.2007	31	191	228	37	217	230	623,0831442
06.06.2007	31	191	228	37	217	230	633,4603566
07.06.2007	31	191	228	37	217	230	618,9949612
07.06.2007	31	191	228	37	217	230	591,7963265
07.06.2007	31	191	228	37	217	230	642,6638181
07.06.2007	31	191	228	37	217	230	618,6783452
11.06.2007	31	191	228	37	217	230	579,055348
12.06.2007	31	191	228	37	217	230	555,5119693
11.06.2007	31	191	228	37	217	230	658,716802
12.06.2007	31	191	228	37	217	230	644,0851427
11.06.2007	31	191	228	37	217	230	677,6598367
12.06.2007	31	191	228	37	217	230	651,920595
11.06.2007	31	191	228	37	217	230	642,1742286
12.06.2007	31	191	228	37	217	230	10,29239882
11.06.2007	31	191	228	37	217	230	672,2545773

12.06.2007	31	191	228	37	217	230	28,79684382
12.06.2007	31	191	228	37	217	230	596,564713
13.06.2007	31	191	228	37	217	230	672,0785673
12.06.2007	31	191	228	37	217	230	591,2507622
13.06.2007	31	191	228	37	217	230	666,2945566
12.06.2007	31	191	228	37	217	230	579,4697048
13.06.2007	31	191	228	37	217	230	629,362146
19.06.2007	31	191	228	37	217	230	645,6382161
18.06.2007	31	191	228	37	217	230	653,6216577
18.06.2007	31	191	228	37	217	230	671,1687161
19.06.2007	31	191	228	37	217	230	594,1056607
18.06.2007	31	191	228	37	217	230	752,3585904
19.06.2007	31	191	228	37	217	230	600,577583
18.06.2007	31	191	228	37	217	230	707,7925981
19.06.2007	31	191	228	37	217	230	710,5536985
18.06.2007	31	191	228	37	217	230	600,2530507
19.06.2007	31	191	228	37	217	230	1258,182745
19.06.2007	31	191	228	37	217	230	557,3720358
19.06.2007	31	191	228	37	217	230	614,9580071
20.06.2007	31	191	228	37	217	230	560,5899868
19.06.2007	31	191	228	37	217	230	655,6870248
20.06.2007	31	191	228	37	217	230	1239,510997
25.06.2007	31	191	228	37	217	230	694,8978004
25.06.2007	31	191	228	37	217	230	699,435547
26.06.2007	31	191	228	37	217	230	694,5091059
25.06.2007	31	191	228	37	217	230	679,1907557
26.06.2007	31	191	228	37	217	230	703,0208085
25.06.2007	31	191	228	37	217	230	712,9992743
26.06.2007	31	191	228	37	217	230	697,536726
25.06.2007	31	191	228	37	217	230	692,4013042
26.06.2007	31	191	228	37	217	230	700,5104204
25.06.2007	31	191	228	37	217	230	699,6078828
26.06.2007	31	191	228	37	217	230	692,1595471

27.06.2007	31	191	228	37	217	230	702,3378216
26.06.2007	31	191	228	37	217	230	694,9333045
27.06.2007	31	191	228	37	217	230	690,1688578
26.06.2007	31	191	228	37	217	230	692,7195735
27.06.2007	31	191	228	37	217	230	1319,653636
30.05.2007	32	194	230	33	162	220	502,5347576
30.05.2007	32	194	230	37	217	230	1009,596508
30.05.2007	32	194	230	37	217	230	1007,909443
30.05.2007	32	194	230	37	217	230	995,3121393
30.05.2007	32	194	230	37	217	230	1000,66579
30.05.2007	32	194	230	37	217	230	1065,4305
31.05.2007	32	194	230	37	217	230	1416,427593
31.05.2007	32	194	230	37	217	230	1241,803479
31.05.2007	32	194	230	37	217	230	1430,227596
06.06.2007	32	194	230	37	217	230	1506,64808
06.06.2007	32	194	230	37	217	230	1454,25638
06.06.2007	32	194	230	37	217	230	1494,136993
06.06.2007	32	194	230	37	217	230	1462,955252
06.06.2007	32	194	230	37	217	230	1462,105557
07.06.2007	32	194	230	37	217	230	1452,111703
07.06.2007	32	194	230	37	217	230	1273,782713
07.06.2007	32	194	230	37	217	230	1468,733651
07.06.2007	32	194	230	37	217	230	1447,57023
11.06.2007	32	194	230	37	217	230	1405,634871
12.06.2007	32	194	230	37	217	230	1428,398271
11.06.2007	32	194	230	37	217	230	1469,638891
12.06.2007	32	194	230	37	217	230	1496,020487
11.06.2007	32	194	230	37	217	230	1489,823605
12.06.2007	32	194	230	37	217	230	1472,662826
11.06.2007	32	194	230	37	217	230	1462,40944
12.06.2007	32	194	230	37	217	230	1418,541412
11.06.2007	32	194	230	37	217	230	1509,091542
12.06.2007	32	194	230	37	217	230	1468,80385

12.06.2007	32	194	230	37	217	230	1463,653519
13.06.2007	32	194	230	37	217	230	1474,457691
12.06.2007	32	194	230	37	217	230	1437,567674
13.06.2007	32	194	230	37	217	230	1458,584403
12.06.2007	32	194	230	37	217	230	1448,695384
13.06.2007	32	194	230	37	217	230	1465,161106
19.06.2007	32	194	230	37	217	230	1450,767325
18.06.2007	32	194	230	37	217	230	1270,565504
18.06.2007	32	194	230	37	217	230	1461,819697
19.06.2007	32	194	230	37	217	230	1460,530481
18.06.2007	32	194	230	37	217	230	1468,335534
19.06.2007	32	194	230	37	217	230	1406,972989
18.06.2007	32	194	230	37	217	230	1500,796953
19.06.2007	32	194	230	37	217	230	1463,730839
18.06.2007	32	194	230	37	217	230	1460,668183
19.06.2007	32	194	230	37	217	230	1501,390379
18.06.2007	32	194	230	37	217	230	1447,082833
19.06.2007	32	194	230	37	217	230	1344,732907
19.06.2007	32	194	230	37	217	230	1440,371906
20.06.2007	32	194	230	37	217	230	1444,070059
19.06.2007	32	194	230	37	217	230	1474,919708
20.06.2007	32	194	230	37	217	230	1356,670097
25.06.2007	32	194	230	37	217	230	1472,568754
25.06.2007	32	194	230	37	217	230	1438,924858
26.06.2007	32	194	230	37	217	230	1438,265069
25.06.2007	32	194	230	37	217	230	1445,350112
26.06.2007	32	194	230	37	217	230	1443,815261
25.06.2007	32	194	230	37	217	230	1484,857435
26.06.2007	32	194	230	37	217	230	1430,11583
25.06.2007	32	194	230	37	217	230	1462,061342
26.06.2007	32	194	230	37	217	230	1441,331145
25.06.2007	32	194	230	37	217	230	1468,994942
26.06.2007	32	194	230	37	217	230	1456,050386

26.06.2007	32	194	230	37	217	230	1454,621441
27.06.2007	32	194	230	37	217	230	1432,838002
26.06.2007	32	194	230	37	217	230	1450,763278
27.06.2007	32	194	230	37	217	230	1425,628014
26.06.2007	32	194	230	37	217	230	1437,781064
27.06.2007	32	194	230	37	217	230	1450,586164
30.05.2007	31	191	228	38	166	226	566,3943595
30.05.2007	33	162	220	37	217	230	487,1525701
30.05.2007	33	162	220	37	217	230	524,0024176
30.05.2007	33	162	220	37	217	230	519,8387931
30.05.2007	33	162	220	37	217	230	540,4814412
30.05.2007	33	162	220	37	217	230	549,2973687
31.05.2007	33	162	220	37	217	230	979,8533251
31.05.2007	33	162	220	37	217	230	1029,550276
31.05.2007	33	162	220	37	217	230	976,0452665
06.06.2007	33	162	220	37	217	230	1278,586177
06.06.2007	33	162	220	37	217	230	1206,1621
06.06.2007	33	162	220	37	217	230	1287,616199
06.06.2007	33	162	220	37	217	230	1253,848841
06.06.2007	33	162	220	37	217	230	1210,000568
07.06.2007	33	162	220	37	217	230	1188,629081
07.06.2007	33	162	220	37	217	230	1197,393729
07.06.2007	33	162	220	37	217	230	1217,222921
07.06.2007	33	162	220	37	217	230	1200,948425
11.06.2007	33	162	220	37	217	230	1354,432087
12.06.2007	33	162	220	37	217	230	1191,017503
11.06.2007	33	162	220	37	217	230	1217,322883
12.06.2007	33	162	220	37	217	230	1216,039238
11.06.2007	33	162	220	37	217	230	1265,869246
12.06.2007	33	162	220	37	217	230	1220,637008
11.06.2007	33	162	220	37	217	230	1202,697697
12.06.2007	33	162	220	37	217	230	1219,518964
11.06.2007	33	162	220	37	217	230	1262,371125

12.06.2007	33	162	220	37	217	230	1226,875631
12.06.2007	33	162	220	37	217	230	1191,43979
13.06.2007	33	162	220	37	217	230	1227,982339
12.06.2007	33	162	220	37	217	230	1168,886858
13.06.2007	33	162	220	37	217	230	1193,584158
12.06.2007	33	162	220	37	217	230	1171,813105
13.06.2007	33	162	220	37	217	230	1202,729765
19.06.2007	33	162	220	37	217	230	1195,141206
18.06.2007	33	162	220	37	217	230	1269,057432
18.06.2007	33	162	220	37	217	230	1203,125302
19.06.2007	33	162	220	37	217	230	1206,085762
18.06.2007	33	162	220	37	217	230	1208,517347
19.06.2007	33	162	220	37	217	230	1182,358081
18.06.2007	33	162	220	37	217	230	1213,715652
19.06.2007	33	162	220	37	217	230	1253,522666
18.06.2007	33	162	220	37	217	230	1278,884622
19.06.2007	33	162	220	37	217	230	1282,9425
18.06.2007	33	162	220	37	217	230	1213,38183
19.06.2007	33	162	220	37	217	230	1230,696503
19.06.2007	33	162	220	37	217	230	1200,124966
20.06.2007	33	162	220	37	217	230	1262,876911
19.06.2007	33	162	220	37	217	230	1228,781047
20.06.2007	33	162	220	37	217	230	1356,977083
25.06.2007	33	162	220	37	217	230	1218,783204
25.06.2007	33	162	220	37	217	230	1217,793821
26.06.2007	33	162	220	37	217	230	1201,754088
25.06.2007	33	162	220	37	217	230	1200,760426
26.06.2007	33	162	220	37	217	230	1211,781329
25.06.2007	33	162	220	37	217	230	1219,273143
26.06.2007	33	162	220	37	217	230	1209,974284
25.06.2007	33	162	220	37	217	230	1210,20691
26.06.2007	33	162	220	37	217	230	1206,687359
25.06.2007	33	162	220	37	217	230	1218,418353

26.06.2007	33	162	220	37	217	230	1202,909767
26.06.2007	33	162	220	37	217	230	1212,33779
27.06.2007	33	162	220	37	217	230	1210,699588
26.06.2007	33	162	220	37	217	230	1213,684916
27.06.2007	33	162	220	37	217	230	1204,723683
26.06.2007	33	162	220	37	217	230	1195,415731
27.06.2007	33	162	220	37	217	230	1203,779719
30.05.2007	37	217	230	38	166	226	567,3143922
30.05.2007	34	173	226	37	217	230	873,9479008
30.05.2007	34	173	226	37	217	230	871,1538606
30.05.2007	34	173	226	37	217	230	855,0123746
30.05.2007	34	173	226	37	217	230	809,9145368
30.05.2007	34	173	226	37	217	230	824,1751957
31.05.2007	34	173	226	37	217	230	1234,196322
31.05.2007	34	173	226	37	217	230	1307,885849
31.05.2007	34	173	226	37	217	230	1232,295292
06.06.2007	34	173	226	37	217	230	1350,279908
06.06.2007	34	173	226	37	217	230	1269,531985
06.06.2007	34	173	226	37	217	230	1310,641932
06.06.2007	34	173	226	37	217	230	1265,483117
06.06.2007	34	173	226	37	217	230	1295,13605
07.06.2007	34	173	226	37	217	230	1247,961462
07.06.2007	34	173	226	37	217	230	1275,68866
07.06.2007	34	173	226	37	217	230	1269,427445
07.06.2007	34	173	226	37	217	230	1268,113228
11.06.2007	34	173	226	37	217	230	1232,54399
12.06.2007	34	173	226	37	217	230	1241,266843
11.06.2007	34	173	226	37	217	230	1269,405658
12.06.2007	34	173	226	37	217	230	1309,763127
11.06.2007	34	173	226	37	217	230	1340,584295
12.06.2007	34	173	226	37	217	230	1296,39413
11.06.2007	34	173	226	37	217	230	1301,117875
12.06.2007	34	173	226	37	217	230	1352,297357

11.06.2007	34	173	226	37	217	230	1341,227216
12.06.2007	34	173	226	37	217	230	1352,941615
12.06.2007	34	173	226	37	217	230	1271,038901
13.06.2007	34	173	226	37	217	230	1343,316483
12.06.2007	34	173	226	37	217	230	1257,206213
13.06.2007	34	173	226	37	217	230	1288,50073
12.06.2007	34	173	226	37	217	230	1227,734758
13.06.2007	34	173	226	37	217	230	1293,841715
19.06.2007	34	173	226	37	217	230	1256,88645
18.06.2007	34	173	226	37	217	230	1352,046439
18.06.2007	34	173	226	37	217	230	1281,733326
19.06.2007	34	173	226	37	217	230	1268,058452
18.06.2007	34	173	226	37	217	230	1269,895021
19.06.2007	34	173	226	37	217	230	1296,94203
18.06.2007	34	173	226	37	217	230	1263,559682
19.06.2007	34	173	226	37	217	230	1328,589879
18.06.2007	34	173	226	37	217	230	1240,559957
19.06.2007	34	173	226	37	217	230	1353,132949
18.06.2007	34	173	226	37	217	230	1268,192416
19.06.2007	34	173	226	37	217	230	1252,451988
19.06.2007	34	173	226	37	217	230	1231,830903
20.06.2007	34	173	226	37	217	230	1352,797221
19.06.2007	34	173	226	37	217	230	1280,321293
20.06.2007	34	173	226	37	217	230	1209,360926
25.06.2007	34	173	226	37	217	230	1276,503285
25.06.2007	34	173	226	37	217	230	1265,002143
26.06.2007	34	173	226	37	217	230	1255,445273
25.06.2007	34	173	226	37	217	230	1231,406287
26.06.2007	34	173	226	37	217	230	1256,601407
25.06.2007	34	173	226	37	217	230	1272,54969
26.06.2007	34	173	226	37	217	230	1258,642263
25.06.2007	34	173	226	37	217	230	1259,706691
26.06.2007	34	173	226	37	217	230	1259,275536

25.06.2007	34	173	226	37	217	230	1258,260445
26.06.2007	34	173	226	37	217	230	1248,74135
26.06.2007	34	173	226	37	217	230	1255,974222
27.06.2007	34	173	226	37	217	230	1261,850956
26.06.2007	34	173	226	37	217	230	1250,647777
27.06.2007	34	173	226	37	217	230	1255,524523
26.06.2007	34	173	226	37	217	230	1243,592372
27.06.2007	34	173	226	37	217	230	1251,747716
30.05.2007	32	194	230	36	128	206	575,0069282
30.05.2007	36	128	206	37	217	230	527,6446835
30.05.2007	36	128	206	37	217	230	522,728141
30.05.2007	36	128	206	37	217	230	492,6232103
30.05.2007	36	128	206	37	217	230	509,1914246
30.05.2007	36	128	206	37	217	230	529,7338842
31.05.2007	36	128	206	37	217	230	954,0652109
31.05.2007	36	128	206	37	217	230	998,6662509
31.05.2007	36	128	206	37	217	230	944,0870251
06.06.2007	36	128	206	37	217	230	599,4371351
06.06.2007	36	128	206	37	217	230	553,5769075
06.06.2007	36	128	206	37	217	230	607,1724975
06.06.2007	36	128	206	37	217	230	560,4167664
06.06.2007	36	128	206	37	217	230	568,1144752
07.06.2007	36	128	206	37	217	230	566,7407272
07.06.2007	36	128	206	37	217	230	567,9729871
07.06.2007	36	128	206	37	217	230	574,3027538
07.06.2007	36	128	206	37	217	230	562,1529235
11.06.2007	36	128	206	37	217	230	526,5922537
12.06.2007	36	128	206	37	217	230	498,7966284
11.06.2007	36	128	206	37	217	230	591,1486803
12.06.2007	36	128	206	37	217	230	562,7466744
11.06.2007	36	128	206	37	217	230	626,5192349
12.06.2007	36	128	206	37	217	230	611,1940986
11.06.2007	36	128	206	37	217	230	557,7588742

12.06.2007	36	128	206	37	217	230	611,3822737
11.06.2007	36	128	206	37	217	230	675,2138431
12.06.2007	36	128	206	37	217	230	569,6961212
12.06.2007	36	128	206	37	217	230	548,5592869
13.06.2007	36	128	206	37	217	230	569,6253639
12.06.2007	36	128	206	37	217	230	557,6066754
13.06.2007	36	128	206	37	217	230	538,4780202
12.06.2007	36	128	206	37	217	230	537,6519628
13.06.2007	36	128	206	37	217	230	562,140977
19.06.2007	36	128	206	37	217	230	555,1828054
18.06.2007	36	128	206	37	217	230	656,0016062
18.06.2007	36	128	206	37	217	230	542,6730884
19.06.2007	36	128	206	37	217	230	554,6365416
18.06.2007	36	128	206	37	217	230	558,7686397
19.06.2007	36	128	206	37	217	230	517,240123
18.06.2007	36	128	206	37	217	230	607,8273298
19.06.2007	36	128	206	37	217	230	595,7408122
18.06.2007	36	128	206	37	217	230	531,8857232
19.06.2007	36	128	206	37	217	230	732,8537986
18.06.2007	36	128	206	37	217	230	560,5248562
19.06.2007	36	128	206	37	217	230	7,279065996
19.06.2007	36	128	206	37	217	230	539,5433847
20.06.2007	36	128	206	37	217	230	170,7075891
19.06.2007	36	128	206	37	217	230	564,8126791
20.06.2007	36	128	206	37	217	230	592,3227005
25.06.2007	36	128	206	37	217	230	561,2644949
25.06.2007	36	128	206	37	217	230	559,9322578
26.06.2007	36	128	206	37	217	230	549,1792074
25.06.2007	36	128	206	37	217	230	547,6479157
26.06.2007	36	128	206	37	217	230	564,3375146
25.06.2007	36	128	206	37	217	230	566,0077579
26.06.2007	36	128	206	37	217	230	554,1527575
25.06.2007	36	128	206	37	217	230	545,8939877

26.06.2007	36	128	206	37	217	230	558,3701825
25.06.2007	36	128	206	37	217	230	564,2752336
26.06.2007	36	128	206	37	217	230	553,1271274
26.06.2007	36	128	206	37	217	230	561,4669508
27.06.2007	36	128	206	37	217	230	562,3498554
26.06.2007	36	128	206	37	217	230	551,0707798
27.06.2007	36	128	206	37	217	230	545,5089435
26.06.2007	36	128	206	37	217	230	548,6393219
27.06.2007	36	128	206	37	217	230	554,0269828
30.05.2007	37	217	230	39	83	181	777,1491863
30.05.2007	29	240	260	38	166	226	1346,103877
30.05.2007	29	240	260	38	166	226	1344,780116
30.05.2007	29	240	260	38	166	226	1317,58713
30.05.2007	29	240	260	38	166	226	1301,679365
30.05.2007	29	240	260	38	166	226	1362,413796
31.05.2007	29	240	260	38	166	226	1415,770691
31.05.2007	29	240	260	38	166	226	1386,727588
31.05.2007	29	240	260	38	166	226	1317,16935
06.06.2007	29	240	260	38	166	226	1463,152808
06.06.2007	29	240	260	38	166	226	1396,392326
06.06.2007	29	240	260	38	166	226	1413,792658
06.06.2007	29	240	260	38	166	226	1408,139765
06.06.2007	29	240	260	38	166	226	1402,341387
07.06.2007	29	240	260	38	166	226	1382,744603
07.06.2007	29	240	260	38	166	226	1381,068473
07.06.2007	29	240	260	38	166	226	1397,318059
07.06.2007	29	240	260	38	166	226	1392,730528
11.06.2007	29	240	260	38	166	226	1355,895892
12.06.2007	29	240	260	38	166	226	1349,428196
11.06.2007	29	240	260	38	166	226	1346,775896
12.06.2007	29	240	260	38	166	226	1356,941138
11.06.2007	29	240	260	38	166	226	1329,917125
12.06.2007	29	240	260	38	166	226	1368,833591

11.06.2007	29	240	260	38	166	226	1370,982877
12.06.2007	29	240	260	38	166	226	1355,295356
11.06.2007	29	240	260	38	166	226	1416,661758
12.06.2007	29	240	260	38	166	226	1373,603599
12.06.2007	29	240	260	38	166	226	1358,893516
13.06.2007	29	240	260	38	166	226	1403,96806
12.06.2007	29	240	260	38	166	226	1393,500524
13.06.2007	29	240	260	38	166	226	1356,309472
12.06.2007	29	240	260	38	166	226	1347,510537
13.06.2007	29	240	260	38	166	226	1349,795751
19.06.2007	29	240	260	38	166	226	1380,707574
18.06.2007	29	240	260	38	166	226	1052,079435
18.06.2007	29	240	260	38	166	226	1451,773735
19.06.2007	29	240	260	38	166	226	1383,757594
18.06.2007	29	240	260	38	166	226	1392,157311
19.06.2007	29	240	260	38	166	226	1430,903321
18.06.2007	29	240	260	38	166	226	1402,368864
19.06.2007	29	240	260	38	166	226	1208,823633
18.06.2007	29	240	260	38	166	226	1398,850763
19.06.2007	29	240	260	38	166	226	1018,760336
18.06.2007	29	240	260	38	166	226	1352,947687
19.06.2007	29	240	260	38	166	226	1461,456746
19.06.2007	29	240	260	38	166	226	1346,07305
20.06.2007	29	240	260	38	166	226	1466,953355
19.06.2007	29	240	260	38	166	226	1354,880767
20.06.2007	29	240	260	38	166	226	1042,888242
25.06.2007	29	240	260	38	166	226	1339,576653
25.06.2007	29	240	260	38	166	226	1372,844379
26.06.2007	29	240	260	38	166	226	1329,344582
25.06.2007	29	240	260	38	166	226	1337,727013
26.06.2007	29	240	260	38	166	226	1324,691516
25.06.2007	29	240	260	38	166	226	1363,641134
26.06.2007	29	240	260	38	166	226	1330,53273

25.06.2007	29	240	260	38	166	226	1364,440641
26.06.2007	29	240	260	38	166	226	1327,329967
25.06.2007	29	240	260	38	166	226	1356,353052
26.06.2007	29	240	260	38	166	226	1302,228724
26.06.2007	29	240	260	38	166	226	1327,467567
27.06.2007	29	240	260	38	166	226	1320,538725
26.06.2007	29	240	260	38	166	226	1322,566455
27.06.2007	29	240	260	38	166	226	1318,623564
26.06.2007	29	240	260	38	166	226	1332,067737
27.06.2007	29	240	260	38	166	226	1321,180657
30.05.2007	31	191	228	39	83	181	778,4673603
30.05.2007	30	122	198	38	166	226	1261,13059
30.05.2007	30	122	198	38	166	226	1247,312976
30.05.2007	30	122	198	38	166	226	1224,648873
30.05.2007	30	122	198	38	166	226	1214,683115
30.05.2007	30	122	198	38	166	226	1265,217313
31.05.2007	30	122	198	38	166	226	1259,494947
31.05.2007	30	122	198	38	166	226	1261,844385
31.05.2007	30	122	198	38	166	226	1314,641523
06.06.2007	30	122	198	38	166	226	1284,010606
06.06.2007	30	122	198	38	166	226	1260,251569
06.06.2007	30	122	198	38	166	226	1281,134336
06.06.2007	30	122	198	38	166	226	1247,113182
06.06.2007	30	122	198	38	166	226	1275,687382
07.06.2007	30	122	198	38	166	226	1366,98906
07.06.2007	30	122	198	38	166	226	1283,605752
07.06.2007	30	122	198	38	166	226	1250,721966
11.06.2007	30	122	198	38	166	226	1349,235493
12.06.2007	30	122	198	38	166	226	1355,895159
11.06.2007	30	122	198	38	166	226	1334,098347
12.06.2007	30	122	198	38	166	226	1350,703179
11.06.2007	30	122	198	38	166	226	1324,634617
12.06.2007	30	122	198	38	166	226	1358,743403

11.06.2007	30	122	198	38	166	226	1357,655329
12.06.2007	30	122	198	38	166	226	1340,967363
11.06.2007	30	122	198	38	166	226	1348,17888
12.06.2007	30	122	198	38	166	226	1355,801473
12.06.2007	30	122	198	38	166	226	1346,228793
13.06.2007	30	122	198	38	166	226	1386,1024
12.06.2007	30	122	198	38	166	226	1357,950848
13.06.2007	30	122	198	38	166	226	1351,857104
12.06.2007	30	122	198	38	166	226	1345,42841
13.06.2007	30	122	198	38	166	226	1356,750135
19.06.2007	30	122	198	38	166	226	1347,496658
18.06.2007	30	122	198	38	166	226	967,3227109
18.06.2007	30	122	198	38	166	226	1350,867167
19.06.2007	30	122	198	38	166	226	1345,349155
18.06.2007	30	122	198	38	166	226	1339,803883
19.06.2007	30	122	198	38	166	226	1394,694863
18.06.2007	30	122	198	38	166	226	1364,365192
19.06.2007	30	122	198	38	166	226	1172,744081
18.06.2007	30	122	198	38	166	226	1350,567236
19.06.2007	30	122	198	38	166	226	929,7947089
18.06.2007	30	122	198	38	166	226	1347,391039
19.06.2007	30	122	198	38	166	226	1359,595969
19.06.2007	30	122	198	38	166	226	1341,480033
20.06.2007	30	122	198	38	166	226	1361,830232
19.06.2007	30	122	198	38	166	226	1407,754213
20.06.2007	30	122	198	38	166	226	938,9318882
25.06.2007	30	122	198	38	166	226	1309,366209
25.06.2007	30	122	198	38	166	226	1312,692646
26.06.2007	30	122	198	38	166	226	1301,416484
25.06.2007	30	122	198	38	166	226	1286,401558
26.06.2007	30	122	198	38	166	226	1308,534505
25.06.2007	30	122	198	38	166	226	1314,88337
26.06.2007	30	122	198	38	166	226	1319,897727

26.06.2007	30	122	198	38	166	226	1312,101931
25.06.2007	30	122	198	38	166	226	1316,862097
26.06.2007	30	122	198	38	166	226	1311,350736
26.06.2007	30	122	198	38	166	226	1316,558761
27.06.2007	30	122	198	38	166	226	1302,301105
26.06.2007	30	122	198	38	166	226	1307,437027
27.06.2007	30	122	198	38	166	226	1301,100839
26.06.2007	30	122	198	38	166	226	1307,754673
30.05.2007	30	122	198	37	217	230	871,1312312
30.05.2007	31	191	228	38	166	226	578,4969095
30.05.2007	31	191	228	38	166	226	586,3434436
30.05.2007	31	191	228	38	166	226	556,1276153
30.05.2007	31	191	228	38	166	226	544,8143079
30.05.2007	31	191	228	38	166	226	554,5786192
31.05.2007	31	191	228	38	166	226	630,7921926
31.05.2007	31	191	228	38	166	226	618,9399678
31.05.2007	31	191	228	38	166	226	545,3952115
06.06.2007	31	191	228	38	166	226	646,078872
06.06.2007	31	191	228	38	166	226	650,3788642
06.06.2007	31	191	228	38	166	226	642,144409
06.06.2007	31	191	228	38	166	226	614,1689369
06.06.2007	31	191	228	38	166	226	630,8464892
07.06.2007	31	191	228	38	166	226	623,2443897
07.06.2007	31	191	228	38	166	226	591,4201826
07.06.2007	31	191	228	38	166	226	644,0656213
07.06.2007	31	191	228	38	166	226	611,595144
11.06.2007	31	191	228	38	166	226	599,39663
12.06.2007	31	191	228	38	166	226	623,3761511
11.06.2007	31	191	228	38	166	226	572,8253486
12.06.2007	31	191	228	38	166	226	636,2355715
11.06.2007	31	191	228	38	166	226	577,9953724
12.06.2007	31	191	228	38	166	226	649,5232318
11.06.2007	31	191	228	38	166	226	635,6246442

12.06.2007	31	191	228	38	166	226	5,332391033
11.06.2007	31	191	228	38	166	226	585,4056172
12.06.2007	31	191	228	38	166	226	7,622152708
12.06.2007	31	191	228	38	166	226	594,8952959
13.06.2007	31	191	228	38	166	226	686,1276006
12.06.2007	31	191	228	38	166	226	633,4889382
13.06.2007	31	191	228	38	166	226	671,3752901
12.06.2007	31	191	228	38	166	226	597,4846159
13.06.2007	31	191	228	38	166	226	626,0754306
19.06.2007	31	191	228	38	166	226	644,2955006
18.06.2007	31	191	228	38	166	226	120,8313384
18.06.2007	31	191	228	38	166	226	688,2249752
19.06.2007	31	191	228	38	166	226	592,6638748
18.06.2007	31	191	228	38	166	226	735,4172461
19.06.2007	31	191	228	38	166	226	657,5712946
18.06.2007	31	191	228	38	166	226	699,2249909
19.06.2007	31	191	228	38	166	226	445,7389038
18.06.2007	31	191	228	38	166	226	601,7569538
19.06.2007	31	191	228	38	166	226	804,6310803
19.06.2007	31	191	228	38	166	226	562,9912192
19.06.2007	31	191	228	38	166	226	618,6104017
20.06.2007	31	191	228	38	166	226	562,3370056
19.06.2007	31	191	228	38	166	226	634,363578
20.06.2007	31	191	228	38	166	226	810,4796737
25.06.2007	31	191	228	38	166	226	665,4652319
25.06.2007	31	191	228	38	166	226	663,663128
26.06.2007	31	191	228	38	166	226	666,5157528
25.06.2007	31	191	228	38	166	226	648,9765361
26.06.2007	31	191	228	38	166	226	673,0956659
25.06.2007	31	191	228	38	166	226	674,145196
26.06.2007	31	191	228	38	166	226	676,1294325
25.06.2007	31	191	228	38	166	226	671,0052028
26.06.2007	31	191	228	38	166	226	669,7586158

25.06.2007	31	191	228	38	166	226	664,6185843
26.06.2007	31	191	228	38	166	226	668,9218044
27.06.2007	31	191	228	38	166	226	671,655635
26.06.2007	31	191	228	38	166	226	666,9658952
27.06.2007	31	191	228	38	166	226	667,3894038
26.06.2007	31	191	228	38	166	226	669,4217999
27.06.2007	31	191	228	38	166	226	1299,61733
30.05.2007	34	173	226	37	217	230	871,329381
30.05.2007	32	194	230	38	166	226	1444,826567
30.05.2007	32	194	230	38	166	226	1448,313734
30.05.2007	32	194	230	38	166	226	1404,419972
30.05.2007	32	194	230	38	166	226	1393,694773
30.05.2007	32	194	230	38	166	226	1487,571916
31.05.2007	32	194	230	38	166	226	1446,320538
31.05.2007	32	194	230	38	166	226	1234,18537
31.05.2007	32	194	230	38	166	226	1436,275739
06.06.2007	32	194	230	38	166	226	1498,895486
06.06.2007	32	194	230	38	166	226	1455,494892
06.06.2007	32	194	230	38	166	226	1487,388057
06.06.2007	32	194	230	38	166	226	1447,94566
06.06.2007	32	194	230	38	166	226	1464,239439
07.06.2007	32	194	230	38	166	226	1448,490112
07.06.2007	32	194	230	38	166	226	1272,862278
07.06.2007	32	194	230	38	166	226	1471,02275
07.06.2007	32	194	230	38	166	226	1442,510365
11.06.2007	32	194	230	38	166	226	1416,028436
12.06.2007	32	194	230	38	166	226	1447,208183
11.06.2007	32	194	230	38	166	226	1448,023236
12.06.2007	32	194	230	38	166	226	1491,602455
11.06.2007	32	194	230	38	166	226	1401,827724
12.06.2007	32	194	230	38	166	226	1473,725844
11.06.2007	32	194	230	38	166	226	1460,849194
12.06.2007	32	194	230	38	166	226	1420,399386

11.06.2007	32	194	230	38	166	226	1471,027282
12.06.2007	32	194	230	38	166	226	1476,877661
12.06.2007	32	194	230	38	166	226	1462,287357
13.06.2007	32	194	230	38	166	226	1503,027976
12.06.2007	32	194	230	38	166	226	1458,736722
13.06.2007	32	194	230	38	166	226	1462,099781
12.06.2007	32	194	230	38	166	226	1464,431608
13.06.2007	32	194	230	38	166	226	1467,671312
19.06.2007	32	194	230	38	166	226	1456,411412
18.06.2007	32	194	230	38	166	226	837,391969
18.06.2007	32	194	230	38	166	226	1468,774571
19.06.2007	32	194	230	38	166	226	1458,449223
18.06.2007	32	194	230	38	166	226	1465,57439
19.06.2007	32	194	230	38	166	226	1473,883478
18.06.2007	32	194	230	38	166	226	1498,118467
19.06.2007	32	194	230	38	166	226	1290,35103
18.06.2007	32	194	230	38	166	226	1469,752596
19.06.2007	32	194	230	38	166	226	1055,443574
18.06.2007	32	194	230	38	166	226	1439,951395
19.06.2007	32	194	230	38	166	226	1348,395076
19.06.2007	32	194	230	38	166	226	1442,814365
20.06.2007	32	194	230	38	166	226	1444,911118
19.06.2007	32	194	230	38	166	226	1466,655275
20.06.2007	32	194	230	38	166	226	930,4293666
25.06.2007	32	194	230	38	166	226	1450,287041
25.06.2007	32	194	230	38	166	226	1412,208809
26.06.2007	32	194	230	38	166	226	1417,406726
25.06.2007	32	194	230	38	166	226	1421,417311
26.06.2007	32	194	230	38	166	226	1418,892385
25.06.2007	32	194	230	38	166	226	1451,338043
26.06.2007	32	194	230	38	166	226	1413,904699
25.06.2007	32	194	230	38	166	226	1448,309876
26.06.2007	32	194	230	38	166	226	1417,800777

25.06.2007	32	194	230	38	166	226	1441,876858
26.06.2007	32	194	230	38	166	226	1439,007581
26.06.2007	32	194	230	38	166	226	1427,353582
27.06.2007	32	194	230	38	166	226	1409,870978
26.06.2007	32	194	230	38	166	226	1430,608183
27.06.2007	32	194	230	38	166	226	1407,698814
26.06.2007	32	194	230	38	166	226	1421,665529
27.06.2007	32	194	230	38	166	226	1431,67979
30.05.2007	30	122	198	31	191	228	872,4518961
30.05.2007	33	162	220	38	166	226	971,0156611
30.05.2007	33	162	220	38	166	226	997,8681042
30.05.2007	33	162	220	38	166	226	971,7968293
30.05.2007	33	162	220	38	166	226	972,1590121
30.05.2007	33	162	220	38	166	226	1014,177243
31.05.2007	33	162	220	38	166	226	1027,923447
31.05.2007	33	162	220	38	166	226	1020,573853
31.05.2007	33	162	220	38	166	226	980,8120328
06.06.2007	33	162	220	38	166	226	1271,088549
06.06.2007	33	162	220	38	166	226	1207,325896
06.06.2007	33	162	220	38	166	226	1279,685348
06.06.2007	33	162	220	38	166	226	1239,4742
06.06.2007	33	162	220	38	166	226	1211,687153
07.06.2007	33	162	220	38	166	226	1185,686101
07.06.2007	33	162	220	38	166	226	1196,44953
07.06.2007	33	162	220	38	166	226	1219,452025
07.06.2007	33	162	220	38	166	226	1195,682759
11.06.2007	33	162	220	38	166	226	1365,232846
12.06.2007	33	162	220	38	166	226	1214,272163
11.06.2007	33	162	220	38	166	226	1191,499168
12.06.2007	33	162	220	38	166	226	1211,200619
11.06.2007	33	162	220	38	166	226	1175,893255
12.06.2007	33	162	220	38	166	226	1221,412466
11.06.2007	33	162	220	38	166	226	1200,665991

12.06.2007	33	162	220	38	166	226	1221,805821
11.06.2007	33	162	220	38	166	226	1218,511379
12.06.2007	33	162	220	38	166	226	1233,199447
12.06.2007	33	162	220	38	166	226	1190,039616
13.06.2007	33	162	220	38	166	226	1255,339388
12.06.2007	33	162	220	38	166	226	1192,134384
13.06.2007	33	162	220	38	166	226	1197,263227
12.06.2007	33	162	220	38	166	226	1187,830074
13.06.2007	33	162	220	38	166	226	1204,884983
19.06.2007	33	162	220	38	166	226	1200,289145
18.06.2007	33	162	220	38	166	226	838,1998116
18.06.2007	33	162	220	38	166	226	1211,011956
19.06.2007	33	162	220	38	166	226	1204,037353
18.06.2007	33	162	220	38	166	226	1204,515719
19.06.2007	33	162	220	38	166	226	1248,945324
18.06.2007	33	162	220	38	166	226	1210,39443
19.06.2007	33	162	220	38	166	226	1067,889397
18.06.2007	33	162	220	38	166	226	1287,175931
19.06.2007	33	162	220	38	166	226	826,3933838
18.06.2007	33	162	220	38	166	226	1204,864487
19.06.2007	33	162	220	38	166	226	1234,405585
19.06.2007	33	162	220	38	166	226	1202,681002
20.06.2007	33	162	220	38	166	226	1263,815064
19.06.2007	33	162	220	38	166	226	1218,936382
20.06.2007	33	162	220	38	166	226	935,7314385
25.06.2007	33	162	220	38	166	226	1195,422731
25.06.2007	33	162	220	38	166	226	1189,203629
26.06.2007	33	162	220	38	166	226	1179,489633
25.06.2007	33	162	220	38	166	226	1175,612938
26.06.2007	33	162	220	38	166	226	1185,761265
25.06.2007	33	162	220	38	166	226	1184,722744
26.06.2007	33	162	220	38	166	226	1192,652197
25.06.2007	33	162	220	38	166	226	1194,815424

26.06.2007	33	162	220	38	166	226	1181,546336
25.06.2007	33	162	220	38	166	226	1189,746257
26.06.2007	33	162	220	38	166	226	1184,639196
26.06.2007	33	162	220	38	166	226	1184,019122
27.06.2007	33	162	220	38	166	226	1185,994932
26.06.2007	33	162	220	38	166	226	1191,662841
27.06.2007	33	162	220	38	166	226	1185,80585
26.06.2007	33	162	220	38	166	226	1177,878554
27.06.2007	33	162	220	38	166	226	1183,271411
30.05.2007	31	191	228	34	173	226	872,6504448
30.05.2007	34	173	226	38	166	226	1314,619976
30.05.2007	34	173	226	38	166	226	1316,798851
30.05.2007	34	173	226	38	166	226	1271,11202
30.05.2007	34	173	226	38	166	226	1218,202013
30.05.2007	34	173	226	38	166	226	1265,224102
31.05.2007	34	173	226	38	166	226	1269,370414
31.05.2007	34	173	226	38	166	226	1300,404772
31.05.2007	34	173	226	38	166	226	1237,882403
06.06.2007	34	173	226	38	166	226	1342,678751
06.06.2007	34	173	226	38	166	226	1270,661
06.06.2007	34	173	226	38	166	226	1302,739247
06.06.2007	34	173	226	38	166	226	1251,078757
06.06.2007	34	173	226	38	166	226	1296,705053
07.06.2007	34	173	226	38	166	226	1245,286683
07.06.2007	34	173	226	38	166	226	1274,768368
07.06.2007	34	173	226	38	166	226	1271,630905
07.06.2007	34	173	226	38	166	226	1262,751882
11.06.2007	34	173	226	38	166	226	1243,937336
12.06.2007	34	173	226	38	166	226	1267,849973
11.06.2007	34	173	226	38	166	226	1240,21472
12.06.2007	34	173	226	38	166	226	1304,788298
11.06.2007	34	173	226	38	166	226	1249,662425
12.06.2007	34	173	226	38	166	226	1297,064798

11.06.2007	34	173	226	38	166	226	1298,884015
12.06.2007	34	173	226	38	166	226	1354,268626
11.06.2007	34	173	226	38	166	226	1297,184288
12.06.2007	34	173	226	38	166	226	1360,127654
12.06.2007	34	173	226	38	166	226	1269,618196
13.06.2007	34	173	226	38	166	226	1370,806403
12.06.2007	34	173	226	38	166	226	1280,97651
13.06.2007	34	173	226	38	166	226	1292,26557
12.06.2007	34	173	226	38	166	226	1243,959763
13.06.2007	34	173	226	38	166	226	1295,662993
19.06.2007	34	173	226	38	166	226	1261,641054
18.06.2007	34	173	226	38	166	226	934,0098133
18.06.2007	34	173	226	38	166	226	1290,094627
19.06.2007	34	173	226	38	166	226	1266,031367
18.06.2007	34	173	226	38	166	226	1264,936908
19.06.2007	34	173	226	38	166	226	1363,622389
18.06.2007	34	173	226	38	166	226	1259,956022
19.06.2007	34	173	226	38	166	226	1152,617924
18.06.2007	34	173	226	38	166	226	1248,796803
19.06.2007	34	173	226	38	166	226	908,7594905
18.06.2007	34	173	226	38	166	226	1258,570525
19.06.2007	34	173	226	38	166	226	1256,194817
19.06.2007	34	173	226	38	166	226	1234,467488
20.06.2007	34	173	226	38	166	226	1353,662938
19.06.2007	34	173	226	38	166	226	1270,125562
20.06.2007	34	173	226	38	166	226	791,4177582
25.06.2007	34	173	226	38	166	226	1252,300471
25.06.2007	34	173	226	38	166	226	1235,512107
26.06.2007	34	173	226	38	166	226	1232,204919
25.06.2007	34	173	226	38	166	226	1205,654652
26.06.2007	34	173	226	38	166	226	1229,908958
25.06.2007	34	173	226	38	166	226	1237,296142
26.06.2007	34	173	226	38	166	226	1240,694015

25.06.2007	34	173	226	38	166	226	1243,409064
26.06.2007	34	173	226	38	166	226	1233,09096
25.06.2007	34	173	226	38	166	226	1228,510669
26.06.2007	34	173	226	38	166	226	1229,634201
26.06.2007	34	173	226	38	166	226	1227,000966
27.06.2007	34	173	226	38	166	226	1236,194688
26.06.2007	34	173	226	38	166	226	1227,801272
27.06.2007	34	173	226	38	166	226	1235,937625
26.06.2007	34	173	226	38	166	226	1225,112253
27.06.2007	34	173	226	38	166	226	1230,618447
30.05.2007	29	240	260	37	217	230	912,1679048
30.05.2007	36	128	206	38	166	226	996,5429651
30.05.2007	36	128	206	38	166	226	996,9060516
30.05.2007	36	128	206	38	166	226	949,8667624
30.05.2007	36	128	206	38	166	226	948,048545
30.05.2007	36	128	206	38	166	226	999,7861211
31.05.2007	36	128	206	38	166	226	1005,424609
31.05.2007	36	128	206	38	166	226	989,1574739
31.05.2007	36	128	206	38	166	226	948,5213957
06.06.2007	36	128	206	38	166	226	595,2796076
06.06.2007	36	128	206	38	166	226	553,5976782
06.06.2007	36	128	206	38	166	226	591,8002185
06.06.2007	36	128	206	38	166	226	552,939824
06.06.2007	36	128	206	38	166	226	564,6760982
07.06.2007	36	128	206	38	166	226	572,0632842
07.06.2007	36	128	206	38	166	226	567,6441432
07.06.2007	36	128	206	38	166	226	575,4656313
07.06.2007	36	128	206	38	166	226	554,8677718
11.06.2007	36	128	206	38	166	226	548,5353614
12.06.2007	36	128	206	38	166	226	572,3170028
11.06.2007	36	128	206	38	166	226	495,96176
12.06.2007	36	128	206	38	166	226	554,2014262
11.06.2007	36	128	206	38	166	226	527,7167136

12.06.2007	36	128	206	38	166	226	608,4514116
11.06.2007	36	128	206	38	166	226	550,1691183
12.06.2007	36	128	206	38	166	226	617,8191687
11.06.2007	36	128	206	38	166	226	576,8226797
12.06.2007	36	128	206	38	166	226	557,7781937
12.06.2007	36	128	206	38	166	226	546,8797784
13.06.2007	36	128	206	38	166	226	578,1210572
12.06.2007	36	128	206	38	166	226	601,4756634
13.06.2007	36	128	206	38	166	226	543,876467
12.06.2007	36	128	206	38	166	226	555,6628237
13.06.2007	36	128	206	38	166	226	557,6339777
19.06.2007	36	128	206	38	166	226	551,6023087
18.06.2007	36	128	206	38	166	226	125,8588515
18.06.2007	36	128	206	38	166	226	562,4615724
19.06.2007	36	128	206	38	166	226	553,3600901
18.06.2007	36	128	206	38	166	226	535,0630564
19.06.2007	36	128	206	38	166	226	569,3822724
18.06.2007	36	128	206	38	166	226	597,1379521
19.06.2007	36	128	206	38	166	226	317,2938777
18.06.2007	36	128	206	38	166	226	531,3238884
19.06.2007	36	128	206	38	166	226	153,028995
18.06.2007	36	128	206	38	166	226	532,7978299
19.06.2007	36	128	206	38	166	226	13,22724622
19.06.2007	36	128	206	38	166	226	543,3196337
20.06.2007	36	128	206	38	166	226	172,7799651
19.06.2007	36	128	206	38	166	226	540,6711059
20.06.2007	36	128	206	38	166	226	54,73068432
25.06.2007	36	128	206	38	166	226	521,3382499
25.06.2007	36	128	206	38	166	226	509,9444366
26.06.2007	36	128	206	38	166	226	510,3123928
25.06.2007	36	128	206	38	166	226	510,5551878
26.06.2007	36	128	206	38	166	226	527,4192436
25.06.2007	36	128	206	38	166	226	520,9237767

26.06.2007	36	128	206	38	166	226	525,0498027
25.06.2007	36	128	206	38	166	226	511,843226
26.06.2007	36	128	206	38	166	226	515,9468695
25.06.2007	36	128	206	38	166	226	517,8914643
26.06.2007	36	128	206	38	166	226	520,1318721
26.06.2007	36	128	206	38	166	226	523,6173871
27.06.2007	36	128	206	38	166	226	520,7903847
26.06.2007	36	128	206	38	166	226	509,7576567
27.06.2007	36	128	206	38	166	226	515,5325472
26.06.2007	36	128	206	38	166	226	512,9035893
27.06.2007	36	128	206	38	166	226	518,0646677
30.05.2007	29	240	260	31	191	228	913,4899436
30.05.2007	37	217	230	38	166	226	528,0459531
30.05.2007	37	217	230	38	166	226	528,3503616
30.05.2007	37	217	230	38	166	226	517,5255195
30.05.2007	37	217	230	38	166	226	508,3322671
30.05.2007	37	217	230	38	166	226	540,6771201
31.05.2007	37	217	230	38	166	226	149,1554497
31.05.2007	37	217	230	38	166	226	15,98587646
31.05.2007	37	217	230	38	166	226	9,846121538
06.06.2007	37	217	230	38	166	226	8,470263265
06.06.2007	37	217	230	38	166	226	2,196217036
06.06.2007	37	217	230	38	166	226	18,76954022
06.06.2007	37	217	230	38	166	226	17,54658673
06.06.2007	37	217	230	38	166	226	10,28274078
07.06.2007	37	217	230	38	166	226	15,58794411
07.06.2007	37	217	230	38	166	226	1,360760073
07.06.2007	37	217	230	38	166	226	2,606234851
07.06.2007	37	217	230	38	166	226	7,47738861
11.06.2007	37	217	230	38	166	226	25,73345419
12.06.2007	37	217	230	38	166	226	112,4642656
11.06.2007	37	217	230	38	166	226	182,6529692
12.06.2007	37	217	230	38	166	226	9,509661275

11.06.2007	37	217	230	38	166	226	99,69976305
12.06.2007	37	217	230	38	166	226	7,509933214
11.06.2007	37	217	230	38	166	226	11,33524118
12.06.2007	37	217	230	38	166	226	10,44571313
11.06.2007	37	217	230	38	166	226	111,8910449
12.06.2007	37	217	230	38	166	226	35,2593096
12.06.2007	37	217	230	38	166	226	1,678734147
13.06.2007	37	217	230	38	166	226	40,51541863
12.06.2007	37	217	230	38	166	226	51,06466626
13.06.2007	37	217	230	38	166	226	5,669014914
12.06.2007	37	217	230	38	166	226	18,01721338
13.06.2007	37	217	230	38	166	226	12,16506958
19.06.2007	37	217	230	38	166	226	15,08587231
18.06.2007	37	217	230	38	166	226	559,2408074
18.06.2007	37	217	230	38	166	226	26,76238357
19.06.2007	37	217	230	38	166	226	2,220756536
18.06.2007	37	217	230	38	166	226	39,32733847
19.06.2007	37	217	230	38	166	226	67,2437284
18.06.2007	37	217	230	38	166	226	15,77084829
19.06.2007	37	217	230	38	166	226	427,2845507
18.06.2007	37	217	230	38	166	226	15,95476945
19.06.2007	37	217	230	38	166	226	609,6716879
18.06.2007	37	217	230	38	166	226	39,35121748
19.06.2007	37	217	230	38	166	226	6,043801624
19.06.2007	37	217	230	38	166	226	3,993925662
20.06.2007	37	217	230	38	166	226	2,105936994
19.06.2007	37	217	230	38	166	226	31,97231794
20.06.2007	37	217	230	38	166	226	553,7290803
25.06.2007	37	217	230	38	166	226	44,51791498
25.06.2007	37	217	230	38	166	226	55,66157438
26.06.2007	37	217	230	38	166	226	43,16786373
25.06.2007	37	217	230	38	166	226	38,18522956
26.06.2007	37	217	230	38	166	226	38,5169623

25.06.2007	37	217	230	38	166	226	45,46629594
26.06.2007	37	217	230	38	166	226	32,38996626
25.06.2007	37	217	230	38	166	226	42,15389564
26.06.2007	37	217	230	38	166	226	47,16990962
25.06.2007	37	217	230	38	166	226	50,48956152
26.06.2007	37	217	230	38	166	226	38,02201951
26.06.2007	37	217	230	38	166	226	38,76148127
27.06.2007	37	217	230	38	166	226	46,11644482
26.06.2007	37	217	230	38	166	226	47,19938974
27.06.2007	37	217	230	38	166	226	32,30060521
26.06.2007	37	217	230	38	166	226	42,11391439
27.06.2007	37	217	230	38	166	226	40,87045632
30.05.2007	36	128	206	38	166	226	958,9153117
30.05.2007	29	240	260	39	83	181	104,7655509
30.05.2007	29	240	260	39	83	181	122,8809918
30.05.2007	29	240	260	39	83	181	11,49782854
30.05.2007	29	240	260	39	83	181	118,9465379
31.05.2007	29	240	260	39	83	181	159,7431455
31.05.2007	29	240	260	39	83	181	71,44766343
31.05.2007	29	240	260	39	83	181	89,18534056
06.06.2007	29	240	260	39	83	181	62,90731761
06.06.2007	29	240	260	39	83	181	22,5037251
06.06.2007	29	240	260	39	83	181	19,72753017
06.06.2007	29	240	260	39	83	181	56,59207543
06.06.2007	29	240	260	39	83	181	39,36039785
07.06.2007	29	240	260	39	83	181	5,709499887
07.06.2007	29	240	260	39	83	181	42,43388037
07.06.2007	29	240	260	39	83	181	24,90001405
07.06.2007	29	240	260	39	83	181	21,73666121
11.06.2007	29	240	260	39	83	181	19,83005424
12.06.2007	29	240	260	39	83	181	10,0261154
11.06.2007	29	240	260	39	83	181	24,95291962
12.06.2007	29	240	260	39	83	181	37,52175203

11.06.2007	29	240	260	39	83	181	10,04411723
12.06.2007	29	240	260	39	83	181	47,97758967
11.06.2007	29	240	260	39	83	181	9,946974301
12.06.2007	29	240	260	39	83	181	84,89330023
11.06.2007	29	240	260	39	83	181	68,2557089
12.06.2007	29	240	260	39	83	181	10,81991585
12.06.2007	29	240	260	39	83	181	13,54105951
13.06.2007	29	240	260	39	83	181	13,43458034
12.06.2007	29	240	260	39	83	181	28,15707728
13.06.2007	29	240	260	39	83	181	3,353430448
12.06.2007	29	240	260	39	83	181	6,666335687
13.06.2007	29	240	260	39	83	181	107,2496584
19.06.2007	29	240	260	39	83	181	23,28683694
18.06.2007	29	240	260	39	83	181	71,95107062
18.06.2007	29	240	260	39	83	181	95,51721555
19.06.2007	29	240	260	39	83	181	26,19612921
18.06.2007	29	240	260	39	83	181	46,77609586
19.06.2007	29	240	260	39	83	181	15,39172216
18.06.2007	29	240	260	39	83	181	20,82517079
19.06.2007	29	240	260	39	83	181	14,88636996
18.06.2007	29	240	260	39	83	181	181,6591516
19.06.2007	29	240	260	39	83	181	56,16736192
18.06.2007	29	240	260	39	83	181	4,839560083
19.06.2007	29	240	260	39	83	181	98,0542551
19.06.2007	29	240	260	39	83	181	2,580110888
20.06.2007	29	240	260	39	83	181	109,9561831
19.06.2007	29	240	260	39	83	181	6,809108582
20.06.2007	29	240	260	39	83	181	98,65211829
25.06.2007	29	240	260	39	83	181	27,90395853
25.06.2007	29	240	260	39	83	181	27,58736372
26.06.2007	29	240	260	39	83	181	10,1572398
25.06.2007	29	240	260	39	83	181	27,83151742
26.06.2007	29	240	260	39	83	181	8,477724644

25.06.2007	29	240	260	39	83	181	41,33600145
26.06.2007	29	240	260	39	83	181	11,81323571
25.06.2007	29	240	260	39	83	181	49,7916841
26.06.2007	29	240	260	39	83	181	3,460799103
25.06.2007	29	240	260	39	83	181	46,20899537
26.06.2007	29	240	260	39	83	181	22,51175125
26.06.2007	29	240	260	39	83	181	8,071095511
27.06.2007	29	240	260	39	83	181	9,646402974
26.06.2007	29	240	260	39	83	181	4,427254566
27.06.2007	29	240	260	39	83	181	14,52645481
26.06.2007	29	240	260	39	83	181	9,197261157
27.06.2007	29	240	260	39	83	181	8,786410298
30.05.2007	32	194	230	37	217	230	996,4477401
30.05.2007	30	122	198	39	83	181	4,501106247
30.05.2007	30	122	198	39	83	181	13,51501963
30.05.2007	30	122	198	39	83	181	99,83520457
30.05.2007	30	122	198	39	83	181	6,084019603
31.05.2007	30	122	198	39	83	181	11,34838165
31.05.2007	30	122	198	39	83	181	60,08803465
31.05.2007	30	122	198	39	83	181	83,38126396
06.06.2007	30	122	198	39	83	181	142,0776949
06.06.2007	30	122	198	39	83	181	133,7954464
06.06.2007	30	122	198	39	83	181	172,0330022
06.06.2007	30	122	198	39	83	181	126,1565483
06.06.2007	30	122	198	39	83	181	184,1941125
07.06.2007	30	122	198	39	83	181	63,70475801
07.06.2007	30	122	198	39	83	181	112,4034912
07.06.2007	30	122	198	39	83	181	143,2556482
11.06.2007	30	122	198	39	83	181	27,65897474
12.06.2007	30	122	198	39	83	181	4,257731923
11.06.2007	30	122	198	39	83	181	38,50453138
12.06.2007	30	122	198	39	83	181	47,35910447
11.06.2007	30	122	198	39	83	181	3,447724637

12.06.2007	30	122	198	39	83	181	56,82931373
11.06.2007	30	122	198	39	83	181	6,749819613
12.06.2007	30	122	198	39	83	181	98,96575994
11.06.2007	30	122	198	39	83	181	6,229264387
12.06.2007	30	122	198	39	83	181	13,86373457
12.06.2007	30	122	198	39	83	181	2,492865497
13.06.2007	30	122	198	39	83	181	5,461263887
12.06.2007	30	122	198	39	83	181	8,584133795
13.06.2007	30	122	198	39	83	181	7,424655589
12.06.2007	30	122	198	39	83	181	5,416423802
13.06.2007	30	122	198	39	83	181	98,50872364
19.06.2007	30	122	198	39	83	181	14,17142192
18.06.2007	30	122	198	39	83	181	15,7800094
18.06.2007	30	122	198	39	83	181	8,183897951
19.06.2007	30	122	198	39	83	181	18,6781239
18.06.2007	30	122	198	39	83	181	8,266806954
19.06.2007	30	122	198	39	83	181	26,22955386
18.06.2007	30	122	198	39	83	181	19,1334343
19.06.2007	30	122	198	39	83	181	21,75431464
18.06.2007	30	122	198	39	83	181	130,775465
19.06.2007	30	122	198	39	83	181	33,55894113
18.06.2007	30	122	198	39	83	181	13,29637716
19.06.2007	30	122	198	39	83	181	9,321636504
19.06.2007	30	122	198	39	83	181	3,957575184
20.06.2007	30	122	198	39	83	181	2,265055227
19.06.2007	30	122	198	39	83	181	62,5951077
20.06.2007	30	122	198	39	83	181	5,592012833
25.06.2007	30	122	198	39	83	181	27,86881488
25.06.2007	30	122	198	39	83	181	43,65743384
26.06.2007	30	122	198	39	83	181	25,62564586
25.06.2007	30	122	198	39	83	181	29,7572634
26.06.2007	30	122	198	39	83	181	27,19063033
25.06.2007	30	122	198	39	83	181	14,39402994

26.06.2007	30	122	198	39	83	181	22,88422676
26.06.2007	30	122	198	39	83	181	21,01622051
25.06.2007	30	122	198	39	83	181	2,507317013
26.06.2007	30	122	198	39	83	181	13,82197427
26.06.2007	30	122	198	39	83	181	19,27172628
27.06.2007	30	122	198	39	83	181	27,86897694
26.06.2007	30	122	198	39	83	181	15,27951957
27.06.2007	30	122	198	39	83	181	32,52549147
26.06.2007	30	122	198	39	83	181	22,74645458
30.05.2007	31	191	228	32	194	230	997,7679905
30.05.2007	31	191	228	39	83	181	741,2349274
30.05.2007	31	191	228	39	83	181	714,5078159
30.05.2007	31	191	228	39	83	181	842,2983312
30.05.2007	31	191	228	39	83	181	800,6867463
31.05.2007	31	191	228	39	83	181	728,5740422
31.05.2007	31	191	228	39	83	181	793,7317246
31.05.2007	31	191	228	39	83	181	787,2959363
06.06.2007	31	191	228	39	83	181	897,0899032
06.06.2007	31	191	228	39	83	181	842,6493831
06.06.2007	31	191	228	39	83	181	921,463519
06.06.2007	31	191	228	39	83	181	861,377313
06.06.2007	31	191	228	39	83	181	919,4970649
07.06.2007	31	191	228	39	83	181	894,0492442
07.06.2007	31	191	228	39	83	181	960,5195278
07.06.2007	31	191	228	39	83	181	860,8321389
07.06.2007	31	191	228	39	83	181	885,7039047
12.06.2007	31	191	228	39	83	181	863,5209078
11.06.2007	31	191	228	39	83	181	890,6508999
11.06.2007	31	191	228	39	83	181	843,2745521
12.06.2007	31	191	228	39	83	181	872,7208473
11.06.2007	31	191	228	39	83	181	865,1495493
12.06.2007	31	191	228	39	83	181	879,2755571
11.06.2007	31	191	228	39	83	181	838,9601895

12.06.2007	31	191	228	39	83	181	1429,757403
11.06.2007	31	191	228	39	83	181	885,528051
12.06.2007	31	191	228	39	83	181	1359,984953
12.06.2007	31	191	228	39	83	181	870,104936
13.06.2007	31	191	228	39	83	181	797,7901423
12.06.2007	31	191	228	39	83	181	884,3164224
13.06.2007	31	191	228	39	83	181	796,0382499
12.06.2007	31	191	228	39	83	181	854,6651696
13.06.2007	31	191	228	39	83	181	943,4597072
19.06.2007	31	191	228	39	83	181	828,9552165
18.06.2007	31	191	228	39	83	181	864,3506761
18.06.2007	31	191	228	39	83	181	781,5649028
19.06.2007	31	191	228	39	83	181	882,5405053
18.06.2007	31	191	228	39	83	181	702,8109988
19.06.2007	31	191	228	39	83	181	864,5225522
18.06.2007	31	191	228	39	83	181	784,3907679
19.06.2007	31	191	228	39	83	181	750,6876785
18.06.2007	31	191	228	39	83	181	736,5430227
19.06.2007	31	191	228	39	83	181	159,0520391
19.06.2007	31	191	228	39	83	181	943,6157573
19.06.2007	31	191	228	39	83	181	843,346518
20.06.2007	31	191	228	39	83	181	934,1948921
19.06.2007	31	191	228	39	83	181	820,3222087
20.06.2007	31	191	228	39	83	181	133,9415932
25.06.2007	31	191	228	39	83	181	686,8370899
25.06.2007	31	191	228	39	83	181	703,2669427
26.06.2007	31	191	228	39	83	181	675,9629835
25.06.2007	31	191	228	39	83	181	689,9194517
26.06.2007	31	191	228	39	83	181	675,7161873
25.06.2007	31	191	228	39	83	181	672,9729076
26.06.2007	31	191	228	39	83	181	685,1305737
25.06.2007	31	191	228	39	83	181	674,8037718
26.06.2007	31	191	228	39	83	181	675,726714

25.06.2007	31	191	228	39	83	181	670,7464895
26.06.2007	31	191	228	39	83	181	671,5260227
27.06.2007	31	191	228	39	83	181	674,6317473
26.06.2007	31	191	228	39	83	181	670,4776063
27.06.2007	31	191	228	39	83	181	680,5346817
26.06.2007	31	191	228	39	83	181	671,8317961
27.06.2007	31	191	228	39	83	181	30,68425473
30.05.2007	33	162	220	38	166	226	1007,561833
30.05.2007	32	194	230	39	83	181	202,1050464
30.05.2007	32	194	230	39	83	181	227,6688791
30.05.2007	32	194	230	39	83	181	98,58640254
30.05.2007	32	194	230	39	83	181	245,1131324
31.05.2007	32	194	230	39	83	181	190,6617676
31.05.2007	32	194	230	39	83	181	86,26162401
31.05.2007	32	194	230	39	83	181	198,5333923
06.06.2007	32	194	230	39	83	181	96,23932201
06.06.2007	32	194	230	39	83	181	82,16711241
06.06.2007	32	194	230	39	83	181	55,45607843
06.06.2007	32	194	230	39	83	181	96,09509983
06.06.2007	32	194	230	39	83	181	23,33875514
07.06.2007	32	194	230	39	83	181	67,41645026
07.06.2007	32	194	230	39	83	181	175,0834884
07.06.2007	32	194	230	39	83	181	98,73851251
07.06.2007	32	194	230	39	83	181	67,68901962
11.06.2007	32	194	230	39	83	181	45,21808278
12.06.2007	32	194	230	39	83	181	92,11858144
11.06.2007	32	194	230	39	83	181	78,04250619
12.06.2007	32	194	230	39	83	181	103,2103868
11.06.2007	32	194	230	39	83	181	86,15422855
12.06.2007	32	194	230	39	83	181	60,27804133
11.06.2007	32	194	230	39	83	181	102,4182179
12.06.2007	32	194	230	39	83	181	21,73354441
11.06.2007	32	194	230	39	83	181	120,4732633

12.06.2007	32	194	230	39	83	181	116,2808707
12.06.2007	32	194	230	39	83	181	117,935765
13.06.2007	32	194	230	39	83	181	117,9218251
12.06.2007	32	194	230	39	83	181	94,80585384
13.06.2007	32	194	230	39	83	181	106,816907
12.06.2007	32	194	230	39	83	181	125,2936364
13.06.2007	32	194	230	39	83	181	14,97710816
19.06.2007	32	194	230	39	83	181	99,0569871
18.06.2007	32	194	230	39	83	181	150,144892
18.06.2007	32	194	230	39	83	181	112,4349871
19.06.2007	32	194	230	39	83	181	101,9204399
18.06.2007	32	194	230	39	83	181	120,6163813
19.06.2007	32	194	230	39	83	181	58,27648545
18.06.2007	32	194	230	39	83	181	115,62653
19.06.2007	32	194	230	39	83	181	99,81353361
18.06.2007	32	194	230	39	83	181	249,4650084
19.06.2007	32	194	230	39	83	181	103,1924883
18.06.2007	32	194	230	39	83	181	85,27102606
19.06.2007	32	194	230	39	83	181	41,30627275
19.06.2007	32	194	230	39	83	181	102,3908304
20.06.2007	32	194	230	39	83	181	84,51580997
19.06.2007	32	194	230	39	83	181	121,6414142
20.06.2007	32	194	230	39	83	181	24,25577582
25.06.2007	32	194	230	39	83	181	120,4523059
25.06.2007	32	194	230	39	83	181	66,01757797
26.06.2007	32	194	230	39	83	181	98,74555616
25.06.2007	32	194	230	39	83	181	112,8704863
26.06.2007	32	194	230	39	83	181	90,71897559
25.06.2007	32	194	230	39	83	181	126,3769842
26.06.2007	32	194	230	39	83	181	77,56617728
25.06.2007	32	194	230	39	83	181	125,3004848
26.06.2007	32	194	230	39	83	181	92,32059477
25.06.2007	32	194	230	39	83	181	128,162521

26.06.2007	32	194	230	39	83	181	119,1470722
26.06.2007	32	194	230	39	83	181	99,1965844
27.06.2007	32	194	230	39	83	181	89,17035013
26.06.2007	32	194	230	39	83	181	113,9332641
27.06.2007	32	194	230	39	83	181	82,20030419
26.06.2007	32	194	230	39	83	181	101,8388295
27.06.2007	32	194	230	39	83	181	109,5035684
30.05.2007	38	166	226	39	83	181	1266,202241
30.05.2007	33	162	220	39	83	181	330,237373
30.05.2007	33	162	220	39	83	181	258,9807119
30.05.2007	33	162	220	39	83	181	383,1205193
30.05.2007	33	162	220	39	83	181	273,8396342
31.05.2007	33	162	220	39	83	181	263,5527276
31.05.2007	33	162	220	39	83	181	325,3210021
31.05.2007	33	162	220	39	83	181	293,0900647
06.06.2007	33	162	220	39	83	181	153,4985264
06.06.2007	33	162	220	39	83	181	171,9075491
06.06.2007	33	162	220	39	83	181	173,4116832
06.06.2007	33	162	220	39	83	181	133,8686747
06.06.2007	33	162	220	39	83	181	235,946594
07.06.2007	33	162	220	39	83	181	203,84006
07.06.2007	33	162	220	39	83	181	232,865693
07.06.2007	33	162	220	39	83	181	160,3563763
07.06.2007	33	162	220	39	83	181	184,0714274
11.06.2007	33	162	220	39	83	181	11,19532674
12.06.2007	33	162	220	39	83	181	149,2673791
11.06.2007	33	162	220	39	83	181	181,6278487
12.06.2007	33	162	220	39	83	181	185,2014445
11.06.2007	33	162	220	39	83	181	147,4918542
12.06.2007	33	162	220	39	83	181	197,1467145
11.06.2007	33	162	220	39	83	181	164,6963159
12.06.2007	33	162	220	39	83	181	224,7823111
11.06.2007	33	162	220	39	83	181	140,620596

12.06.2007	33	162	220	39	83	181	136,0268784
12.06.2007	33	162	220	39	83	181	158,5391959
13.06.2007	33	162	220	39	83	181	136,8178563
12.06.2007	33	162	220	39	83	181	179,333869
13.06.2007	33	162	220	39	83	181	163,0961331
12.06.2007	33	162	220	39	83	181	154,8666809
13.06.2007	33	162	220	39	83	181	252,3376534
19.06.2007	33	162	220	39	83	181	161,0673753
18.06.2007	33	162	220	39	83	181	147,7667226
18.06.2007	33	162	220	39	83	181	150,2878814
19.06.2007	33	162	220	39	83	181	158,0726088
18.06.2007	33	162	220	39	83	181	143,297603
19.06.2007	33	162	220	39	83	181	174,3941294
18.06.2007	33	162	220	39	83	181	178,1504678
19.06.2007	33	162	220	39	83	181	129,6113408
18.06.2007	33	162	220	39	83	181	53,00535292
19.06.2007	33	162	220	39	83	181	139,5729018
18.06.2007	33	162	220	39	83	181	154,1479714
19.06.2007	33	162	220	39	83	181	141,2732975
19.06.2007	33	162	220	39	83	181	143,8290915
20.06.2007	33	162	220	39	83	181	113,4031999
19.06.2007	33	162	220	39	83	181	134,8089828
20.06.2007	33	162	220	39	83	181	9,754416258
25.06.2007	33	162	220	39	83	181	140,0832856
25.06.2007	33	162	220	39	83	181	162,2508406
26.06.2007	33	162	220	39	83	181	145,4247594
25.06.2007	33	162	220	39	83	181	138,2820717
26.06.2007	33	162	220	39	83	181	147,5849721
25.06.2007	33	162	220	39	83	181	143,8125426
26.06.2007	33	162	220	39	83	181	151,1523891
25.06.2007	33	162	220	39	83	181	134,7396845
26.06.2007	33	162	220	39	83	181	149,3628371
25.06.2007	33	162	220	39	83	181	128,8258968

26.06.2007	33	162	220	39	83	181	139,3373004
26.06.2007	33	162	220	39	83	181	149,0690995
27.06.2007	33	162	220	39	83	181	142,3952763
26.06.2007	33	162	220	39	83	181	131,5851062
27.06.2007	33	162	220	39	83	181	145,0459458
26.06.2007	33	162	220	39	83	181	147,258955
27.06.2007	33	162	220	39	83	181	144,9897642
30.05.2007	34	173	226	38	166	226	1351,351682
30.05.2007	34	173	226	39	83	181	67,56320076
30.05.2007	34	173	226	39	83	181	91,27504019
30.05.2007	34	173	226	39	83	181	43,6988967
30.05.2007	34	173	226	39	83	181	7,876828428
31.05.2007	34	173	226	39	83	181	7,494281232
31.05.2007	34	173	226	39	83	181	19,0695969
31.05.2007	34	173	226	39	83	181	16,16567547
06.06.2007	34	173	226	39	83	181	74,05921286
06.06.2007	34	173	226	39	83	181	123,279375
06.06.2007	34	173	226	39	83	181	152,6513893
06.06.2007	34	173	226	39	83	181	122,1308861
06.06.2007	34	173	226	39	83	181	162,0111428
07.06.2007	34	173	226	39	83	181	158,6678183
07.06.2007	34	173	226	39	83	181	173,5997722
07.06.2007	34	173	226	39	83	181	122,8761771
07.06.2007	34	173	226	39	83	181	129,4507468
11.06.2007	34	173	226	39	83	181	136,884156
12.06.2007	34	173	226	39	83	181	117,4793332
11.06.2007	34	173	226	39	83	181	142,08081
12.06.2007	34	173	226	39	83	181	109,5901034
11.06.2007	34	173	226	39	83	181	91,76141643
12.06.2007	34	173	226	39	83	181	131,9918795
11.06.2007	34	173	226	39	83	181	81,32880671
12.06.2007	34	173	226	39	83	181	86,63182015
11.06.2007	34	173	226	39	83	181	73,26055771

12.06.2007	34	173	226	39	83	181	5,871725003
12.06.2007	34	173	226	39	83	181	94,03216898
13.06.2007	34	173	226	39	83	181	24,62479679
12.06.2007	34	173	226	39	83	181	102,7453309
13.06.2007	34	173	226	39	83	181	84,01259276
12.06.2007	34	173	226	39	83	181	112,2047507
13.06.2007	34	173	226	39	83	181	178,370833
19.06.2007	34	173	226	39	83	181	116,3898802
18.06.2007	34	173	226	39	83	181	49,53633485
18.06.2007	34	173	226	39	83	181	86,68466557
19.06.2007	34	173	226	39	83	181	112,6764575
18.06.2007	34	173	226	39	83	181	97,02692019
19.06.2007	34	173	226	39	83	181	65,67711274
18.06.2007	34	173	226	39	83	181	141,6079925
19.06.2007	34	173	226	39	83	181	43,57459705
18.06.2007	34	173	226	39	83	181	14,57859231
19.06.2007	34	173	226	39	83	181	55,69572296
18.06.2007	34	173	226	39	83	181	117,3125257
19.06.2007	34	173	226	39	83	181	125,1653932
19.06.2007	34	173	226	39	83	181	122,5725398
20.06.2007	34	173	226	39	83	181	11,54721436
19.06.2007	34	173	226	39	83	181	95,11252102
20.06.2007	34	173	226	39	83	181	155,8840766
25.06.2007	34	173	226	39	83	181	88,2548549
25.06.2007	34	173	226	39	83	181	129,2595246
26.06.2007	34	173	226	39	83	181	104,6123614
25.06.2007	34	173	226	39	83	181	120,0855792
26.06.2007	34	173	226	39	83	181	115,7563675
25.06.2007	34	173	226	39	83	181	103,4633472
26.06.2007	34	173	226	39	83	181	114,2953905
25.06.2007	34	173	226	39	83	181	96,95460653
26.06.2007	34	173	226	39	83	181	112,8028444
25.06.2007	34	173	226	39	83	181	101,4976718

26.06.2007	34	173	226	39	83	181	107,2587958
26.06.2007	34	173	226	39	83	181	119,8289641
27.06.2007	34	173	226	39	83	181	106,6320663
26.06.2007	34	173	226	39	83	181	105,8567584
27.06.2007	34	173	226	39	83	181	109,4066456
26.06.2007	34	173	226	39	83	181	110,4191723
27.06.2007	34	173	226	39	83	181	109,5247241
30.05.2007	30	122	198	38	166	226	1351,999036
30.05.2007	36	128	206	39	83	181	282,2789578
30.05.2007	36	128	206	39	83	181	260,3576487
30.05.2007	36	128	206	39	83	181	412,0117648
30.05.2007	36	128	206	39	83	181	294,8455472
31.05.2007	36	128	206	39	83	181	295,3501297
31.05.2007	36	128	206	39	83	181	372,9870964
31.05.2007	36	128	206	39	83	181	338,4545724
06.06.2007	36	128	206	39	83	181	990,5139375
06.06.2007	36	128	206	39	83	181	960,4346831
06.06.2007	36	128	206	39	83	181	992,9397205
06.06.2007	36	128	206	39	83	181	943,0351749
06.06.2007	36	128	206	39	83	181	1001,186345
07.06.2007	36	128	206	39	83	181	960,096866
07.06.2007	36	128	206	39	83	181	991,8485249
07.06.2007	36	128	206	39	83	181	953,6638835
07.06.2007	36	128	206	39	83	181	966,4741434
11.06.2007	36	128	206	39	83	181	971,0518086
11.06.2007	36	128	206	39	83	181	921,5443213
12.06.2007	36	128	206	39	83	181	991,8321326
11.06.2007	36	128	206	39	83	181	947,2336369
12.06.2007	36	128	206	39	83	181	929,7838577
11.06.2007	36	128	206	39	83	181	947,6664727
12.06.2007	36	128	206	39	83	181	940,4329575
11.06.2007	36	128	206	39	83	181	985,1225109
12.06.2007	36	128	206	39	83	181	929,9521052

12.06.2007	36	128	206	39	83	181	948,4598339
13.06.2007	36	128	206	39	83	181	935,0310029
12.06.2007	36	128	206	39	83	181	932,3826936
13.06.2007	36	128	206	39	83	181	948,492508
12.06.2007	36	128	206	39	83	181	900,3935319
13.06.2007	36	128	206	39	83	181	1035,429236
12.06.2007	36	128	206	39	83	181	927,2687424
19.06.2007	36	128	206	39	83	181	956,6033563
18.06.2007	36	128	206	39	83	181	858,8664952
18.06.2007	36	128	206	39	83	181	931,6409989
19.06.2007	36	128	206	39	83	181	949,1716857
18.06.2007	36	128	206	39	83	181	932,1456861
19.06.2007	36	128	206	39	83	181	975,6968111
18.06.2007	36	128	206	39	83	181	934,3578742
19.06.2007	36	128	206	39	83	181	876,8703478
18.06.2007	36	128	206	39	83	181	837,2587398
19.06.2007	36	128	206	39	83	181	816,4516252
18.06.2007	36	128	206	39	83	181	954,0832793
19.06.2007	36	128	206	39	83	181	1358,192259
19.06.2007	36	128	206	39	83	181	930,0630878
20.06.2007	36	128	206	39	83	181	1330,445758
19.06.2007	36	128	206	39	83	181	937,7027344
20.06.2007	36	128	206	39	83	181	889,6309898
25.06.2007	36	128	206	39	83	181	934,4858046
25.06.2007	36	128	206	39	83	181	970,9353896
26.06.2007	36	128	206	39	83	181	942,9703669
25.06.2007	36	128	206	39	83	181	951,2824061
26.06.2007	36	128	206	39	83	181	928,0707716
25.06.2007	36	128	206	39	83	181	944,1548991
26.06.2007	36	128	206	39	83	181	942,6160044
25.06.2007	36	128	206	39	83	181	939,6927289
26.06.2007	36	128	206	39	83	181	937,9133567
25.06.2007	36	128	206	39	83	181	925,1234253

26.06.2007	36	128	206	39	83	181	924,7797075
26.06.2007	36	128	206	39	83	181	929,1507812
27.06.2007	36	128	206	39	83	181	932,7824819
26.06.2007	36	128	206	39	83	181	943,2083605
27.06.2007	36	128	206	39	83	181	941,7126792
26.06.2007	36	128	206	39	83	181	942,7891856
27.06.2007	36	128	206	39	83	181	932,2737796
30.05.2007	29	240	260	38	166	226	1388,431767
30.05.2007	37	217	230	39	83	181	808,7167373
30.05.2007	37	217	230	39	83	181	781,0026386
30.05.2007	37	217	230	39	83	181	897,0251088
30.05.2007	37	217	230	39	83	181	821,4145109
31.05.2007	37	217	230	39	83	181	1231,429465
31.05.2007	37	217	230	39	83	181	1326,094625
31.05.2007	37	217	230	39	83	181	1248,408777
06.06.2007	37	217	230	39	83	181	1412,209046
06.06.2007	37	217	230	39	83	181	1372,993671
06.06.2007	37	217	230	39	83	181	1440,121014
06.06.2007	37	217	230	39	83	181	1368,548775
06.06.2007	37	217	230	39	83	181	1438,881316
07.06.2007	37	217	230	39	83	181	1384,69558
07.06.2007	37	217	230	39	83	181	1423,105928
07.06.2007	37	217	230	39	83	181	1371,924688
07.06.2007	37	217	230	39	83	181	1380,93863
12.06.2007	37	217	230	39	83	181	1338,398501
11.06.2007	37	217	230	39	83	181	1364,258696
11.06.2007	37	217	230	39	83	181	1391,714349
12.06.2007	37	217	230	39	83	181	1396,004374
11.06.2007	37	217	230	39	83	181	1410,221723
12.06.2007	37	217	230	39	83	181	1413,488634
11.06.2007	37	217	230	39	83	181	1365,486603
12.06.2007	37	217	230	39	83	181	1433,311366
11.06.2007	37	217	230	39	83	181	1393,548418

12.06.2007	37	217	230	39	83	181	1357,722443
12.06.2007	37	217	230	39	83	181	1348,228719
13.06.2007	37	217	230	39	83	181	1363,163883
12.06.2007	37	217	230	39	83	181	1344,614478
13.06.2007	37	217	230	39	83	181	1354,936059
12.06.2007	37	217	230	39	83	181	1325,14192
13.06.2007	37	217	230	39	83	181	1451,027018
19.06.2007	37	217	230	39	83	181	1353,729753
18.06.2007	37	217	230	39	83	181	1396,25981
18.06.2007	37	217	230	39	83	181	1351,221517
19.06.2007	37	217	230	39	83	181	1361,059593
18.06.2007	37	217	230	39	83	181	1349,901966
19.06.2007	37	217	230	39	83	181	1348,698129
18.06.2007	37	217	230	39	83	181	1385,539798
19.06.2007	37	217	230	39	83	181	1363,958697
18.06.2007	37	217	230	39	83	181	1226,030658
19.06.2007	37	217	230	39	83	181	1398,806156
18.06.2007	37	217	230	39	83	181	1364,770043
19.06.2007	37	217	230	39	83	181	1363,712253
19.06.2007	37	217	230	39	83	181	1342,893988
20.06.2007	37	217	230	39	83	181	1359,988426
19.06.2007	37	217	230	39	83	181	1357,408945
20.06.2007	37	217	230	39	83	181	1363,556936
25.06.2007	37	217	230	39	83	181	1358,823678
25.06.2007	37	217	230	39	83	181	1373,472873
26.06.2007	37	217	230	39	83	181	1345,986102
25.06.2007	37	217	230	39	83	181	1334,748743
26.06.2007	37	217	230	39	83	181	1356,886273
25.06.2007	37	217	230	39	83	181	1361,41341
26.06.2007	37	217	230	39	83	181	1359,100687
25.06.2007	37	217	230	39	83	181	1343,589062
26.06.2007	37	217	230	39	83	181	1353,295482
25.06.2007	37	217	230	39	83	181	1346,013938

26.06.2007	37	217	230	39	83	181	1340,197293
26.06.2007	37	217	230	39	83	181	1358,398569
27.06.2007	37	217	230	39	83	181	1350,320331
26.06.2007	37	217	230	39	83	181	1342,326839
27.06.2007	37	217	230	39	83	181	1347,392637
26.06.2007	37	217	230	39	83	181	1341,298485
27.06.2007	37	217	230	39	83	181	1344,232598
30.05.2007	32	194	230	38	166	226	1473,747878
30.05.2007	38	166	226	39	83	181	1256,630048
30.05.2007	38	166	226	39	83	181	1233,798608
30.05.2007	38	166	226	39	83	181	1307,839535
30.05.2007	38	166	226	39	83	181	1260,347467
31.05.2007	38	166	226	39	83	181	1267,427575
31.05.2007	38	166	226	39	83	181	1318,673506
31.05.2007	38	166	226	39	83	181	1253,987082
06.06.2007	38	166	226	39	83	181	1404,500797
06.06.2007	38	166	226	39	83	181	1374,216625
06.06.2007	38	166	226	39	83	181	1433,22591
06.06.2007	38	166	226	39	83	181	1353,658244
06.06.2007	38	166	226	39	83	181	1440,999756
07.06.2007	38	166	226	39	83	181	1381,075625
07.06.2007	38	166	226	39	83	181	1422,119648
07.06.2007	38	166	226	39	83	181	1374,196584
07.06.2007	38	166	226	39	83	181	1375,832949
12.06.2007	38	166	226	39	83	181	1359,053523
11.06.2007	38	166	226	39	83	181	1374,966124
11.06.2007	38	166	226	39	83	181	1371,282308
12.06.2007	38	166	226	39	83	181	1391,440026
11.06.2007	38	166	226	39	83	181	1321,192012
12.06.2007	38	166	226	39	83	181	1414,493263
11.06.2007	38	166	226	39	83	181	1363,666725
12.06.2007	38	166	226	39	83	181	1435,053974
11.06.2007	38	166	226	39	83	181	1353,296496

12.06.2007	38	166	226	39	83	181	1364,99349
12.06.2007	38	166	226	39	83	181	1346,845943
13.06.2007	38	166	226	39	83	181	1390,964937
12.06.2007	38	166	226	39	83	181	1366,450811
13.06.2007	38	166	226	39	83	181	1358,53296
12.06.2007	38	166	226	39	83	181	1341,009767
13.06.2007	38	166	226	39	83	181	1453,578082
19.06.2007	38	166	226	39	83	181	1359,179874
18.06.2007	38	166	226	39	83	181	983,0595864
18.06.2007	38	166	226	39	83	181	1358,565365
19.06.2007	38	166	226	39	83	181	1358,990854
18.06.2007	38	166	226	39	83	181	1346,55006
19.06.2007	38	166	226	39	83	181	1415,607164
18.06.2007	38	166	226	39	83	181	1382,768443
19.06.2007	38	166	226	39	83	181	1194,094436
18.06.2007	38	166	226	39	83	181	1234,28162
19.06.2007	38	166	226	39	83	181	962,9208972
18.06.2007	38	166	226	39	83	181	1357,056045
19.06.2007	38	166	226	39	83	181	1367,242907
19.06.2007	38	166	226	39	83	181	1345,407246
20.06.2007	38	166	226	39	83	181	1360,841328
19.06.2007	38	166	226	39	83	181	1348,486403
20.06.2007	38	166	226	39	83	181	944,2434117
25.06.2007	38	166	226	39	83	181	1335,504268
25.06.2007	38	166	226	39	83	181	1346,49501
26.06.2007	38	166	226	39	83	181	1324,206435
25.06.2007	38	166	226	39	83	181	1310,357864
26.06.2007	38	166	226	39	83	181	1331,437459
25.06.2007	38	166	226	39	83	181	1327,336996
26.06.2007	38	166	226	39	83	181	1342,276721
25.06.2007	38	166	226	39	83	181	1328,725449
26.06.2007	38	166	226	39	83	181	1328,986891
25.06.2007	38	166	226	39	83	181	1317,857844

26.06.2007	38	166	226	39	83	181	1322,511005
26.06.2007	38	166	226	39	83	181	1330,676092
27.06.2007	38	166	226	39	83	181	1326,414391
26.06.2007	38	166	226	39	83	181	1321,151651
27.06.2007	38	166	226	39	83	181	1328,988943
26.06.2007	38	166	226	39	83	181	1324,306694
27.06.2007	38	166	226	39	83	181	1324,684104
07.06.2006	119	94	190	120	82	186	78,04
07.06.2006	119	94	190	121	68	173	97,65
07.06.2006	119	94	190	122	62	165	137,25
07.06.2006	119	94	190	123	68	176	73,98
07.06.2006	119	94	190	124	128	214	546,76
07.06.2006	119	94	190	125	64	170	51,39
07.06.2006	119	94	190	127	136	218	66,04
07.06.2006	119	94	190	129	115	216	815,88
07.06.2006	119	94	190	131	74	182	105,62
07.06.2006	120	82	186	121	68	173	20,08
07.06.2006	120	82	186	122	62	165	64,94
07.06.2006	120	82	186	123	68	176	4,14
07.06.2006	120	82	186	124	128	214	470,12
07.06.2006	120	82	186	125	64	170	27,07
07.06.2006	120	82	186	127	136	218	12,02
07.06.2006	120	82	186	129	115	216	739,36
07.06.2006	120	82	186	131	74	182	30,20
07.06.2006	121	68	173	122	62	165	53,22
07.06.2006	121	68	173	123	68	176	24,22
07.06.2006	121	68	173	124	128	214	450,05
07.06.2006	121	68	173	125	64	170	46,29
07.06.2006	121	68	173	127	136	218	31,78
07.06.2006	121	68	173	129	115	216	719,29
07.06.2006	121	68	173	131	74	182	20,42
07.06.2006	122	62	165	123	68	176	68,00
07.06.2006	122	62	165	124	128	214	426,16

07.06.2006	122	62	165	125	64	170	90,27
07.06.2006	122	62	165	127	136	218	75,60
07.06.2006	122	62	165	129	115	216	694,64
07.06.2006	122	62	165	131	74	182	34,91
07.06.2006	123	68	176	124	128	214	474,26
07.06.2006	123	68	176	125	64	170	23,19
07.06.2006	123	68	176	127	136	218	8,05
07.06.2006	123	68	176	129	115	216	743,51
07.06.2006	123	68	176	131	74	182	33,52
07.06.2006	124	128	214	125	64	170	495,66
07.06.2006	124	128	214	127	136	218	481,76
07.06.2006	124	128	214	129	115	216	269,25
07.06.2006	124	128	214	131	74	182	447,37
07.06.2006	125	64	170	127	136	218	15,15
07.06.2006	125	64	170	129	115	216	764,85
07.06.2006	125	64	170	131	74	182	56,40
07.06.2006	127	136	218	129	115	216	751,00
07.06.2006	127	136	218	131	74	182	41,38
07.06.2006	129	115	216	131	74	182	716,54
07.06.2006	119	94	190	120	82	186	117,43
07.06.2006	119	94	190	121	68	173	122,54
07.06.2006	119	94	190	122	62	165	135,85
07.06.2006	119	94	190	123	68	176	76,08
07.06.2006	119	94	190	124	128	214	547,19
07.06.2006	119	94	190	125	64	170	104,41
07.06.2006	119	94	190	127	136	218	105,24
07.06.2006	119	94	190	129	115	216	801,05
07.06.2006	119	94	190	131	74	182	119,73
07.06.2006	120	82	186	121	68	173	18,94
07.06.2006	120	82	186	122	62	165	33,16
07.06.2006	120	82	186	123	68	176	43,58
07.06.2006	120	82	186	124	128	214	429,77
07.06.2006	120	82	186	125	64	170	13,57

07.06.2006	120	82	186	127	136	218	16,67
07.06.2006	120	82	186	129	115	216	683,97
07.06.2006	120	82	186	131	74	182	9,47
07.06.2006	121	68	173	122	62	165	15,84
07.06.2006	121	68	173	123	68	176	54,57
07.06.2006	121	68	173	124	128	214	426,82
07.06.2006	121	68	173	125	64	170	27,79
07.06.2006	121	68	173	127	136	218	33,61
07.06.2006	121	68	173	129	115	216	682,12
07.06.2006	121	68	173	131	74	182	9,47
07.06.2006	122	62	165	123	68	176	70,20
07.06.2006	122	62	165	124	128	214	416,18
07.06.2006	122	62	165	125	64	170	43,50
07.06.2006	122	62	165	127	136	218	48,97
07.06.2006	122	62	165	129	115	216	672,02
07.06.2006	122	62	165	131	74	182	24,16
07.06.2006	123	68	176	124	128	214	471,87
07.06.2006	123	68	176	125	64	170	30,01
07.06.2006	123	68	176	127	136	218	29,42
07.06.2006	123	68	176	129	115	216	725,19
07.06.2006	123	68	176	131	74	182	48,54
07.06.2006	124	128	214	125	64	170	442,81
07.06.2006	124	128	214	127	136	218	442,46
07.06.2006	124	128	214	129	115	216	256,74
07.06.2006	124	128	214	131	74	182	428,10
07.06.2006	125	64	170	127	136	218	7,17
07.06.2006	125	64	170	129	115	216	696,72
07.06.2006	125	64	170	131	74	182	19,77
07.06.2006	127	136	218	129	115	216	695,86
07.06.2006	127	136	218	131	74	182	24,82
07.06.2006	129	115	216	131	74	182	682,88
07.06.2006	119	94	190	120	82	186	37,89
07.06.2006	119	94	190	121	68	173	34,16

07.06.2006	119	94	190	122	62	165	68,41
07.06.2006	119	94	190	123	68	176	33,65
07.06.2006	119	94	190	124	128	214	486,92
07.06.2006	119	94	190	127	136	218	17,33
07.06.2006	119	94	190	129	115	216	730,19
07.06.2006	119	94	190	131	74	182	53,90
07.06.2006	120	82	186	121	68	173	3,76
07.06.2006	120	82	186	122	62	165	30,57
07.06.2006	120	82	186	123	68	176	9,50
07.06.2006	120	82	186	124	128	214	463,13
07.06.2006	120	82	186	127	136	218	30,44
07.06.2006	120	82	186	129	115	216	707,82
07.06.2006	120	82	186	131	74	182	17,01
07.06.2006	121	68	173	122	62	165	34,32
07.06.2006	121	68	173	123	68	176	8,55
07.06.2006	121	68	173	124	128	214	465,77
07.06.2006	121	68	173	127	136	218	26,93
07.06.2006	121	68	173	129	115	216	710,34
07.06.2006	121	68	173	131	74	182	20,64
07.06.2006	122	62	165	123	68	176	36,14
07.06.2006	122	62	165	124	128	214	443,58
07.06.2006	122	62	165	127	136	218	59,84
07.06.2006	122	62	165	129	115	216	688,94
07.06.2006	122	62	165	131	74	182	15,60
07.06.2006	123	68	176	124	128	214	460,00
07.06.2006	123	68	176	127	136	218	30,85
07.06.2006	123	68	176	129	115	216	704,33
07.06.2006	123	68	176	131	74	182	20,80
07.06.2006	124	128	214	127	136	218	490,62
07.06.2006	124	128	214	129	115	216	245,59
07.06.2006	124	128	214	131	74	182	448,38
07.06.2006	127	136	218	129	115	216	734,74
07.06.2006	127	136	218	131	74	182	47,44

07.06.2006	129	115	216	131	74	182	693,36
07.06.2006	119	94	190	120	82	186	103,79
07.06.2006	119	94	190	121	68	173	109,26
07.06.2006	119	94	190	122	62	165	141,15
07.06.2006	119	94	190	123	68	176	104,30
07.06.2006	119	94	190	124	128	214	614,18
07.06.2006	119	94	190	125	64	170	50,60
07.06.2006	119	94	190	127	136	218	86,97
07.06.2006	119	94	190	129	115	216	815,64
07.06.2006	119	94	190	131	74	182	123,13
07.06.2006	120	82	186	121	68	173	14,02
07.06.2006	120	82	186	122	62	165	47,90
07.06.2006	120	82	186	123	68	176	15,97
07.06.2006	120	82	186	124	128	214	511,50
07.06.2006	120	82	186	125	64	170	53,31
07.06.2006	120	82	186	127	136	218	23,36
07.06.2006	120	82	186	129	115	216	714,43
07.06.2006	120	82	186	131	74	182	22,57
07.06.2006	121	68	173	122	62	165	55,68
07.06.2006	121	68	173	123	68	176	6,04
07.06.2006	121	68	173	124	128	214	504,93
07.06.2006	121	68	173	125	64	170	60,02
07.06.2006	121	68	173	127	136	218	22,80
07.06.2006	121	68	173	129	115	216	706,87
07.06.2006	121	68	173	131	74	182	29,43
07.06.2006	122	62	165	123	68	176	60,86
07.06.2006	122	62	165	124	128	214	486,04
07.06.2006	122	62	165	125	64	170	92,09
07.06.2006	122	62	165	127	136	218	71,14
07.06.2006	122	62	165	129	115	216	691,80
07.06.2006	122	62	165	131	74	182	26,29
07.06.2006	123	68	176	124	128	214	509,91
07.06.2006	123	68	176	125	64	170	55,69

07.06.2006	123	68	176	127	136	218	17,40
07.06.2006	123	68	176	129	115	216	711,53
07.06.2006	123	68	176	131	74	182	34,56
07.06.2006	124	128	214	125	64	170	564,50
07.06.2006	124	128	214	127	136	218	527,28
07.06.2006	124	128	214	129	115	216	208,41
07.06.2006	124	128	214	131	74	182	495,31
07.06.2006	125	64	170	127	136	218	39,20
07.06.2006	125	64	170	129	115	216	766,84
07.06.2006	125	64	170	131	74	182	72,65
07.06.2006	127	136	218	129	115	216	728,74
07.06.2006	127	136	218	131	74	182	45,88
07.06.2006	129	115	216	131	74	182	699,47
07.06.2006	119	94	190	120	82	186	83,80
07.06.2006	119	94	190	121	68	173	87,30
07.06.2006	119	94	190	122	62	165	103,52
07.06.2006	119	94	190	123	68	176	76,61
07.06.2006	119	94	190	124	128	214	523,13
07.06.2006	119	94	190	125	64	170	62,45
07.06.2006	119	94	190	127	136	218	75,99
07.06.2006	119	94	190	129	115	216	736,17
07.06.2006	119	94	190	131	74	182	88,06
07.06.2006	120	82	186	121	68	173	3,66
07.06.2006	120	82	186	122	62	165	24,35
07.06.2006	120	82	186	123	68	176	7,87
07.06.2006	120	82	186	124	128	214	457,35
07.06.2006	120	82	186	125	64	170	83,76
07.06.2006	120	82	186	127	136	218	30,00
07.06.2006	120	82	186	129	115	216	673,28
07.06.2006	120	82	186	131	74	182	4,79
07.06.2006	121	68	173	122	62	165	21,02
07.06.2006	121	68	173	123	68	176	10,91
07.06.2006	121	68	173	124	128	214	455,48

07.06.2006	121	68	173	125	64	170	87,07
07.06.2006	121	68	173	127	136	218	30,69
07.06.2006	121	68	173	129	115	216	671,54
07.06.2006	121	68	173	131	74	182	1,35
07.06.2006	122	62	165	123	68	176	28,75
07.06.2006	122	62	165	124	128	214	454,60
07.06.2006	122	62	165	125	64	170	108,08
07.06.2006	122	62	165	127	136	218	33,28
07.06.2006	122	62	165	129	115	216	671,28
07.06.2006	122	62	165	131	74	182	19,71
07.06.2006	123	68	176	124	128	214	464,81
07.06.2006	123	68	176	125	64	170	80,96
07.06.2006	123	68	176	127	136	218	24,70
07.06.2006	123	68	176	129	115	216	680,64
07.06.2006	123	68	176	131	74	182	11,51
07.06.2006	124	128	214	125	64	170	471,81
07.06.2006	124	128	214	127	136	218	485,04
07.06.2006	124	128	214	129	115	216	217,03
07.06.2006	124	128	214	131	74	182	455,69
07.06.2006	125	64	170	127	136	218	97,51
07.06.2006	125	64	170	129	115	216	682,18
07.06.2006	125	64	170	131	74	182	88,38
07.06.2006	127	136	218	129	115	216	701,36
07.06.2006	127	136	218	131	74	182	30,15
07.06.2006	129	115	216	131	74	182	671,79
08.06.2006	119	94	190	120	82	186	108,01
08.06.2006	119	94	190	122	62	165	131,64
08.06.2006	119	94	190	123	68	176	85,72
08.06.2006	119	94	190	124	128	214	594,71
08.06.2006	119	94	190	125	64	170	81,29
08.06.2006	119	94	190	127	136	218	95,91
08.06.2006	119	94	190	129	115	216	842,72
08.06.2006	119	94	190	131	74	182	121,40

08.06.2006	120	82	186	121	68	173	11,08
08.06.2006	120	82	186	123	68	176	36,87
08.06.2006	120	82	186	124	128	214	475,59
08.06.2006	120	82	186	125	64	170	42,58
08.06.2006	120	82	186	127	136	218	26,15
08.06.2006	120	82	186	129	115	216	726,42
08.06.2006	120	82	186	131	74	182	7,93
08.06.2006	121	68	173	122	62	165	10,81
08.06.2006	121	68	173	123	68	176	36,05
08.06.2006	121	68	173	125	64	170	39,30
08.06.2006	121	68	173	127	136	218	25,87
08.06.2006	121	68	173	129	115	216	726,78
08.06.2006	121	68	173	131	74	182	12,92
08.06.2006	122	62	165	123	68	176	48,12
08.06.2006	122	62	165	124	128	214	466,87
08.06.2006	122	62	165	125	64	170	35,16
08.06.2006	122	62	165	127	136	218	40,29
08.06.2006	122	62	165	131	74	182	3,67
08.06.2006	123	68	176	124	128	214	509,29
08.06.2006	123	68	176	125	64	170	6,22
08.06.2006	123	68	176	127	136	218	17,01
08.06.2006	123	68	176	129	115	216	758,44
08.06.2006	123	68	176	131	74	182	44,59
08.06.2006	124	128	214	125	64	170	436,73
08.06.2006	124	128	214	127	136	218	449,64
08.06.2006	124	128	214	131	74	182	469,50
08.06.2006	125	64	170	127	136	218	23,08
08.06.2006	125	64	170	129	115	216	762,10
08.06.2006	125	64	170	131	74	182	50,38
08.06.2006	127	136	218	129	115	216	752,44
08.06.2006	127	136	218	131	74	182	32,68
08.06.2006	129	115	216	131	74	182	716,49
08.06.2006	119	94	190	120	82	186	98,99

08.06.2006	119	94	190	122	62	165	105,31
08.06.2006	119	94	190	123	68	176	31,18
08.06.2006	119	94	190	124	128	214	542,54
08.06.2006	119	94	190	125	64	170	11,62
08.06.2006	119	94	190	127	136	218	83,27
08.06.2006	119	94	190	129	115	216	810,26
08.06.2006	120	82	186	121	68	173	29,50
08.06.2006	120	82	186	122	62	165	47,93
08.06.2006	120	82	186	123	68	176	44,53
08.06.2006	120	82	186	124	128	214	501,41
08.06.2006	120	82	186	127	136	218	34,09
08.06.2006	120	82	186	129	115	216	734,18
08.06.2006	120	82	186	131	74	182	35,67
08.06.2006	121	68	173	122	62	165	18,90
08.06.2006	121	68	173	123	68	176	53,10
08.06.2006	121	68	173	124	128	214	492,83
08.06.2006	121	68	173	127	136	218	26,21
08.06.2006	121	68	173	129	115	216	707,63
08.06.2006	121	68	173	131	74	182	33,12
08.06.2006	122	62	165	123	68	176	94,47
08.06.2006	122	62	165	124	128	214	465,56
08.06.2006	122	62	165	127	136	218	77,41
08.06.2006	122	62	165	129	115	216	692,24
08.06.2006	122	62	165	131	74	182	18,73
08.06.2006	123	68	176	125	64	170	20,16
08.06.2006	123	68	176	127	136	218	29,03
08.06.2006	123	68	176	129	115	216	757,56
08.06.2006	123	68	176	131	74	182	76,28
08.06.2006	124	128	214	127	136	218	516,14
08.06.2006	124	128	214	129	115	216	251,84
08.06.2006	124	128	214	131	74	182	478,48
08.06.2006	125	64	170	129	115	216	784,17
08.06.2006	127	136	218	129	115	216	728,60

08.06.2006	127	136	218	131	74	182	55,74
08.06.2006	129	115	216	131	74	182	701,42
08.06.2006	119	94	190	120	82	186	109,85
08.06.2006	119	94	190	121	68	173	100,22
08.06.2006	119	94	190	122	62	165	117,01
08.06.2006	119	94	190	123	68	176	5,70
08.06.2006	119	94	190	124	128	214	578,16
08.06.2006	119	94	190	125	64	170	72,40
08.06.2006	119	94	190	127	136	218	84,21
08.06.2006	119	94	190	129	115	216	822,91
08.06.2006	119	94	190	131	74	182	104,25
08.06.2006	120	82	186	121	68	173	10,47
08.06.2006	120	82	186	122	62	165	36,14
08.06.2006	120	82	186	123	68	176	105,47
08.06.2006	120	82	186	124	128	214	468,70
08.06.2006	120	82	186	125	64	170	38,64
08.06.2006	120	82	186	127	136	218	25,74
08.06.2006	120	82	186	129	115	216	713,48
08.06.2006	120	82	186	131	74	182	22,71
08.06.2006	121	68	173	122	62	165	34,08
08.06.2006	121	68	173	123	68	176	95,99
08.06.2006	121	68	173	124	128	214	478,02
08.06.2006	121	68	173	125	64	170	28,41
08.06.2006	121	68	173	127	136	218	16,08
08.06.2006	121	68	173	129	115	216	723,53
08.06.2006	121	68	173	131	74	182	17,52
08.06.2006	122	62	165	123	68	176	113,98
08.06.2006	122	62	165	124	128	214	465,23
08.06.2006	122	62	165	125	64	170	48,51
08.06.2006	122	62	165	127	136	218	43,63
08.06.2006	122	62	165	129	115	216	716,37
08.06.2006	122	62	165	131	74	182	17,44
08.06.2006	123	68	176	124	128	214	574,01

08.06.2006	123	68	176	125	64	170	68,48
08.06.2006	123	68	176	127	136	218	79,93
08.06.2006	123	68	176	129	115	216	818,19
08.06.2006	123	68	176	131	74	182	100,72
08.06.2006	124	128	214	125	64	170	505,79
08.06.2006	124	128	214	127	136	218	494,09
08.06.2006	124	128	214	129	115	216	268,09
08.06.2006	124	128	214	131	74	182	474,97
08.06.2006	125	64	170	127	136	218	13,49
08.06.2006	125	64	170	129	115	216	751,92
08.06.2006	125	64	170	131	74	182	33,00
08.06.2006	127	136	218	129	115	216	739,22
08.06.2006	127	136	218	131	74	182	26,37
08.06.2006	129	115	216	131	74	182	723,57
08.06.2006	120	82	186	121	68	173	24,83
08.06.2006	120	82	186	122	62	165	59,94
08.06.2006	120	82	186	123	68	176	20,73
08.06.2006	120	82	186	124	128	214	477,68
08.06.2006	120	82	186	125	64	170	12,74
08.06.2006	120	82	186	127	136	218	70,44
08.06.2006	120	82	186	129	115	216	737,00
08.06.2006	120	82	186	131	74	182	31,42
08.06.2006	121	68	173	122	62	165	37,74
08.06.2006	121	68	173	123	68	176	4,47
08.06.2006	121	68	173	124	128	214	455,00
08.06.2006	121	68	173	125	64	170	37,33
08.06.2006	121	68	173	127	136	218	92,39
08.06.2006	121	68	173	129	115	216	715,13
08.06.2006	121	68	173	131	74	182	6,77
08.06.2006	122	62	165	123	68	176	42,11
08.06.2006	122	62	165	124	128	214	436,60
08.06.2006	122	62	165	125	64	170	70,83
08.06.2006	122	62	165	127	136	218	116,79

08.06.2006	122	62	165	129	115	216	698,69
08.06.2006	122	62	165	131	74	182	31,43
08.06.2006	123	68	176	124	128	214	458,13
08.06.2006	123	68	176	125	64	170	33,36
08.06.2006	123	68	176	127	136	218	89,30
08.06.2006	123	68	176	129	115	216	718,02
08.06.2006	123	68	176	131	74	182	11,22
08.06.2006	124	128	214	125	64	170	490,25
08.06.2006	124	128	214	127	136	218	547,38
08.06.2006	124	128	214	129	115	216	263,30
08.06.2006	124	128	214	131	74	182	449,81
08.06.2006	125	64	170	127	136	218	58,51
08.06.2006	125	64	170	129	115	216	749,37
08.06.2006	125	64	170	131	74	182	43,77
08.06.2006	127	136	218	129	115	216	807,25
08.06.2006	127	136	218	131	74	182	97,74
08.06.2006	129	115	216	131	74	182	710,25
08.06.2006	119	94	190	120	82	186	107,75
08.06.2006	119	94	190	121	68	173	99,43
08.06.2006	119	94	190	122	62	165	119,97
08.06.2006	119	94	190	123	68	176	80,16
08.06.2006	119	94	190	124	128	214	561,09
08.06.2006	119	94	190	125	64	170	35,98
08.06.2006	119	94	190	127	136	218	86,15
08.06.2006	119	94	190	129	115	216	822,42
08.06.2006	119	94	190	131	74	182	104,18
08.06.2006	120	82	186	121	68	173	15,55
08.06.2006	120	82	186	122	62	165	28,00
08.06.2006	120	82	186	123	68	176	27,82
08.06.2006	120	82	186	124	128	214	456,26
08.06.2006	120	82	186	125	64	170	71,79
08.06.2006	120	82	186	127	136	218	24,22
08.06.2006	120	82	186	129	115	216	719,41

08.06.2006	120	82	186	131	74	182	8,76
08.06.2006	121	68	173	122	62	165	43,14
08.06.2006	121	68	173	123	68	176	20,82
08.06.2006	121	68	173	124	128	214	462,07
08.06.2006	121	68	173	125	64	170	63,73
08.06.2006	121	68	173	127	136	218	13,34
08.06.2006	121	68	173	129	115	216	724,27
08.06.2006	121	68	173	131	74	182	21,13
08.06.2006	122	62	165	123	68	176	47,28
08.06.2006	122	62	165	124	128	214	454,92
08.06.2006	122	62	165	125	64	170	85,58
08.06.2006	122	62	165	127	136	218	47,95
08.06.2006	122	62	165	129	115	216	719,44
08.06.2006	122	62	165	131	74	182	22,74
08.06.2006	123	68	176	124	128	214	482,29
08.06.2006	123	68	176	125	64	170	44,18
08.06.2006	123	68	176	127	136	218	8,64
08.06.2006	123	68	176	129	115	216	744,78
08.06.2006	123	68	176	131	74	182	26,19
08.06.2006	124	128	214	125	64	170	525,74
08.06.2006	124	128	214	127	136	218	475,41
08.06.2006	124	128	214	129	115	216	265,38
08.06.2006	124	128	214	131	74	182	462,06
08.06.2006	125	64	170	127	136	218	50,39
08.06.2006	125	64	170	129	115	216	787,61
08.06.2006	125	64	170	131	74	182	68,50
08.06.2006	127	136	218	129	115	216	737,57
08.06.2006	127	136	218	131	74	182	25,42
08.06.2006	129	115	216	131	74	182	725,58
08.06.2006	119	94	190	120	82	186	107,39
08.06.2006	119	94	190	121	68	173	107,45
08.06.2006	119	94	190	122	62	165	127,78
08.06.2006	119	94	190	123	68	176	79,75

08.06.2006	119	94	190	124	128	214	563,36
08.06.2006	119	94	190	125	64	170	46,01
08.06.2006	119	94	190	127	136	218	99,66
08.06.2006	119	94	190	129	115	216	817,50
08.06.2006	119	94	190	131	74	182	113,36
08.06.2006	120	82	186	121	68	173	14,05
08.06.2006	120	82	186	122	62	165	20,89
08.06.2006	120	82	186	123	68	176	31,81
08.06.2006	120	82	186	124	128	214	459,69
08.06.2006	120	82	186	125	64	170	61,73
08.06.2006	120	82	186	127	136	218	15,09
08.06.2006	120	82	186	129	115	216	722,07
08.06.2006	120	82	186	131	74	182	6,82
08.06.2006	121	68	173	122	62	165	28,44
08.06.2006	121	68	173	123	68	176	27,94
08.06.2006	121	68	173	124	128	214	456,67
08.06.2006	121	68	173	125	64	170	61,50
08.06.2006	121	68	173	127	136	218	7,81
08.06.2006	121	68	173	129	115	216	716,10
08.06.2006	121	68	173	131	74	182	18,69
08.06.2006	122	62	165	123	68	176	52,46
08.06.2006	122	62	165	124	128	214	441,84
08.06.2006	122	62	165	125	64	170	82,39
08.06.2006	122	62	165	127	136	218	33,68
08.06.2006	122	62	165	129	115	216	706,94
08.06.2006	122	62	165	131	74	182	14,46
08.06.2006	123	68	176	124	128	214	483,82
08.06.2006	123	68	176	125	64	170	34,07
08.06.2006	123	68	176	127	136	218	20,31
08.06.2006	123	68	176	129	115	216	741,06
08.06.2006	123	68	176	131	74	182	38,62
08.06.2006	124	128	214	125	64	170	517,88
08.06.2006	124	128	214	127	136	218	464,47

08.06.2006	124	128	214	129	115	216	290,45
08.06.2006	124	128	214	131	74	182	455,03
08.06.2006	125	64	170	127	136	218	53,69
08.06.2006	125	64	170	129	115	216	774,48
08.06.2006	125	64	170	131	74	182	67,93
08.06.2006	127	136	218	129	115	216	723,58
08.06.2006	127	136	218	131	74	182	21,44
08.06.2006	129	115	216	131	74	182	718,58
08.06.2006	119	94	190	120	82	186	95,38
08.06.2006	119	94	190	121	68	173	117,98
08.06.2006	119	94	190	122	62	165	112,87
08.06.2006	119	94	190	123	68	176	19,55
08.06.2006	119	94	190	124	128	214	570,34
08.06.2006	119	94	190	125	64	170	72,80
08.06.2006	119	94	190	127	136	218	90,02
08.06.2006	119	94	190	129	115	216	828,36
08.06.2006	119	94	190	131	74	182	121,82
08.06.2006	120	82	186	121	68	173	24,38
08.06.2006	120	82	186	122	62	165	18,97
08.06.2006	120	82	186	123	68	176	82,20
08.06.2006	120	82	186	124	128	214	475,48
08.06.2006	120	82	186	125	64	170	28,47
08.06.2006	120	82	186	127	136	218	11,37
08.06.2006	120	82	186	129	115	216	736,69
08.06.2006	120	82	186	131	74	182	27,84
08.06.2006	121	68	173	122	62	165	5,41
08.06.2006	121	68	173	123	68	176	105,91
08.06.2006	121	68	173	124	128	214	454,80
08.06.2006	121	68	173	125	64	170	52,75
08.06.2006	121	68	173	127	136	218	34,38
08.06.2006	121	68	173	129	115	216	718,66
08.06.2006	121	68	173	131	74	182	3,90
08.06.2006	122	62	165	123	68	176	100,61

08.06.2006	122	62	165	124	128	214	459,41
08.06.2006	122	62	165	125	64	170	47,36
08.06.2006	122	62	165	127	136	218	29,11
08.06.2006	122	62	165	129	115	216	722,69
08.06.2006	122	62	165	131	74	182	9,03
08.06.2006	123	68	176	124	128	214	554,98
08.06.2006	123	68	176	125	64	170	56,97
08.06.2006	123	68	176	127	136	218	75,31
08.06.2006	123	68	176	129	115	216	811,15
08.06.2006	123	68	176	131	74	182	109,63
08.06.2006	124	128	214	125	64	170	498,07
08.06.2006	124	128	214	127	136	218	480,32
08.06.2006	124	128	214	129	115	216	282,56
08.06.2006	124	128	214	131	74	182	450,91
08.06.2006	125	64	170	127	136	218	19,04
08.06.2006	125	64	170	129	115	216	755,68
08.06.2006	125	64	170	131	74	182	56,04
08.06.2006	127	136	218	129	115	216	739,55
08.06.2006	127	136	218	131	74	182	37,44
08.06.2006	129	115	216	131	74	182	714,86
08.06.2006	119	94	190	120	82	186	156,88
08.06.2006	119	94	190	121	68	173	144,63
08.06.2006	119	94	190	122	62	165	170,94
08.06.2006	119	94	190	123	68	176	93,73
08.06.2006	119	94	190	124	128	214	607,24
08.06.2006	119	94	190	125	64	170	73,69
08.06.2006	119	94	190	127	136	218	179,95
08.06.2006	119	94	190	129	115	216	859,34
08.06.2006	119	94	190	131	74	182	148,21
08.06.2006	120	82	186	121	68	173	48,37
08.06.2006	120	82	186	122	62	165	94,34
08.06.2006	120	82	186	123	68	176	80,34
08.06.2006	120	82	186	124	128	214	484,17

08.06.2006	120	82	186	125	64	170	128,05
08.06.2006	120	82	186	127	136	218	122,08
08.06.2006	120	82	186	129	115	216	752,58
08.06.2006	120	82	186	131	74	182	25,94
08.06.2006	121	68	173	122	62	165	49,27
08.06.2006	121	68	173	123	68	176	96,95
08.06.2006	121	68	173	124	128	214	469,55
08.06.2006	121	68	173	125	64	170	140,20
08.06.2006	121	68	173	127	136	218	75,64
08.06.2006	121	68	173	129	115	216	731,17
08.06.2006	121	68	173	131	74	182	22,43
08.06.2006	122	62	165	123	68	176	142,92
08.06.2006	122	62	165	124	128	214	436,30
08.06.2006	122	62	165	125	64	170	182,48
08.06.2006	122	62	165	127	136	218	28,10
08.06.2006	122	62	165	129	115	216	691,67
08.06.2006	122	62	165	131	74	182	69,72
08.06.2006	123	68	176	124	128	214	561,87
08.06.2006	123	68	176	125	64	170	47,71
08.06.2006	123	68	176	127	136	218	164,75
08.06.2006	123	68	176	129	115	216	826,89
08.06.2006	123	68	176	131	74	182	86,05
08.06.2006	124	128	214	125	64	170	608,24
08.06.2006	124	128	214	127	136	218	429,97
08.06.2006	124	128	214	129	115	216	280,02
08.06.2006	124	128	214	131	74	182	476,05
08.06.2006	125	64	170	127	136	218	201,03
08.06.2006	125	64	170	129	115	216	871,37
08.06.2006	125	64	170	131	74	182	132,24
08.06.2006	127	136	218	129	115	216	679,45
08.06.2006	127	136	218	131	74	182	97,02
08.06.2006	129	115	216	131	74	182	741,04
09.06.2006	119	94	190	120	82	186	121,33

09.06.2006	119	94	190	121	68	173	119,34
09.06.2006	119	94	190	121	68	173	111,35
09.06.2006	119	94	190	122	62	165	121,86
09.06.2006	119	94	190	123	68	176	90,83
09.06.2006	119	94	190	124	128	214	546,17
09.06.2006	119	94	190	125	64	170	110,22
09.06.2006	119	94	190	127	136	218	97,28
09.06.2006	119	94	190	129	115	216	823,22
09.06.2006	119	94	190	131	74	182	128,43
09.06.2006	120	82	186	121	68	173	5,86
09.06.2006	120	82	186	122	62	165	11,59
09.06.2006	120	82	186	122	62	165	19,36
09.06.2006	120	82	186	123	68	176	26,15
09.06.2006	120	82	186	124	128	214	438,42
09.06.2006	120	82	186	125	64	170	18,58
09.06.2006	120	82	186	127	136	218	21,00
09.06.2006	120	82	186	129	115	216	720,16
09.06.2006	120	82	186	131	74	182	24,22
09.06.2006	121	68	173	122	62	165	16,51
09.06.2006	121	68	173	123	68	176	33,97
09.06.2006	121	68	173	124	128	214	476,53
09.06.2006	121	68	173	124	128	214	436,59
09.06.2006	121	68	173	125	64	170	29,37
09.06.2006	121	68	173	127	136	218	31,60
09.06.2006	121	68	173	129	115	216	721,01
09.06.2006	121	68	173	131	74	182	13,70
09.06.2006	122	62	165	123	68	176	45,48
09.06.2006	122	62	165	124	128	214	426,86
09.06.2006	122	62	165	125	64	170	53,96
09.06.2006	122	62	165	127	136	218	35,77
09.06.2006	122	62	165	129	115	216	718,48
09.06.2006	122	62	165	129	115	216	712,66
09.06.2006	122	62	165	131	74	182	7,14

09.06.2006	123	68	176	124	128	214	456,35
09.06.2006	123	68	176	125	64	170	19,62
09.06.2006	123	68	176	127	136	218	6,81
09.06.2006	123	68	176	129	115	216	732,75
09.06.2006	123	68	176	131	74	182	49,74
09.06.2006	124	128	214	125	64	170	513,45
09.06.2006	124	128	214	127	136	218	501,74
09.06.2006	124	128	214	129	115	216	257,35
09.06.2006	124	128	214	129	115	216	313,97
09.06.2006	124	128	214	131	74	182	429,20
09.06.2006	125	64	170	127	136	218	12,95
09.06.2006	125	64	170	129	115	216	713,84
09.06.2006	125	64	170	131	74	182	41,34
09.06.2006	127	136	218	129	115	216	726,57
09.06.2006	127	136	218	131	74	182	45,13
09.06.2006	129	115	216	131	74	182	720,85
09.06.2006	119	94	190	120	82	186	61,90
09.06.2006	119	94	190	121	68	173	89,22
09.06.2006	119	94	190	121	68	173	107,69
09.06.2006	119	94	190	122	62	165	145,08
09.06.2006	119	94	190	123	68	176	54,64
09.06.2006	119	94	190	124	128	214	600,34
09.06.2006	119	94	190	127	136	218	28,21
09.06.2006	119	94	190	129	115	216	794,09
09.06.2006	119	94	190	131	74	182	95,44
09.06.2006	119	94	190	131	74	182	127,96
09.06.2006	120	82	186	121	68	173	12,18
09.06.2006	120	82	186	122	62	165	63,13
09.06.2006	120	82	186	123	68	176	30,78
09.06.2006	120	82	186	124	128	214	482,41
09.06.2006	120	82	186	125	64	170	50,91
09.06.2006	120	82	186	127	136	218	15,81
09.06.2006	120	82	186	129	115	216	713,62

09.06.2006	120	82	186	131	74	182	44,92
09.06.2006	121	68	173	122	62	165	51,08
09.06.2006	121	68	173	123	68	176	58,32
09.06.2006	121	68	173	124	128	214	460,72
09.06.2006	121	68	173	125	64	170	77,74
09.06.2006	121	68	173	127	136	218	61,01
09.06.2006	121	68	173	129	115	216	712,55
09.06.2006	121	68	173	131	74	182	6,27
09.06.2006	122	62	165	123	68	176	75,11
09.06.2006	122	62	165	124	128	214	451,64
09.06.2006	122	62	165	125	64	170	93,70
09.06.2006	122	62	165	127	136	218	74,45
09.06.2006	122	62	165	129	115	216	703,28
09.06.2006	122	62	165	131	74	182	13,63
09.06.2006	123	68	176	124	128	214	512,54
09.06.2006	123	68	176	124	128	214	545,72
09.06.2006	123	68	176	127	136	218	3,87
09.06.2006	123	68	176	129	115	216	764,23
09.06.2006	123	68	176	131	74	182	64,58
09.06.2006	124	128	214	125	64	170	532,52
09.06.2006	124	128	214	127	136	218	517,19
09.06.2006	124	128	214	129	115	216	266,08
09.06.2006	124	128	214	131	74	182	455,47
09.06.2006	125	64	170	127	136	218	16,89
09.06.2006	125	64	170	131	74	182	83,95
09.06.2006	127	136	218	129	115	216	767,85
09.06.2006	127	136	218	131	74	182	67,24
09.06.2006	129	115	216	131	74	182	707,28
12.06.2006	119	94	190	120	82	186	535,79
12.06.2006	119	94	190	121	68	173	34,61
12.06.2006	120	82	186	121	68	173	566,65
12.06.2006	119	94	190	122	62	165	136,58
12.06.2006	120	82	186	122	62	165	407,98

12.06.2006	121	68	173	122	62	165	170,78
12.06.2006	119	94	190	123	68	176	48,67
12.06.2006	120	82	186	123	68	176	487,92
12.06.2006	121	68	173	123	68	176	82,31
12.06.2006	122	62	165	123	68	176	88,62
12.06.2006	119	94	190	124	128	214	575,78
12.06.2006	120	82	186	124	128	214	49,79
12.06.2006	121	68	173	124	128	214	605,76
12.06.2006	122	62	165	124	128	214	450,89
12.06.2006	123	68	176	124	128	214	528,47
12.06.2006	119	94	190	125	64	170	89,55
12.06.2006	120	82	186	125	64	170	448,92
12.06.2006	121	68	173	125	64	170	123,31
12.06.2006	122	62	165	125	64	170	48,04
12.06.2006	123	68	176	125	64	170	41,00
12.06.2006	124	128	214	125	64	170	490,26
12.06.2006	119	94	190	127	136	218	113,79
12.06.2006	120	82	186	127	136	218	422,86
12.06.2006	121	68	173	127	136	218	146,53
12.06.2006	122	62	165	127	136	218	34,05
12.06.2006	123	68	176	127	136	218	65,29
12.06.2006	124	128	214	127	136	218	463,86
12.06.2006	125	64	170	127	136	218	26,86
12.06.2006	119	94	190	129	115	216	841,54
12.06.2006	120	82	186	129	115	216	356,32
12.06.2006	121	68	173	129	115	216	867,00
12.06.2006	122	62	165	129	115	216	731,63
12.06.2006	123	68	176	129	115	216	797,93
12.06.2006	124	128	214	129	115	216	306,55
12.06.2006	125	64	170	129	115	216	764,02
12.06.2006	127	136	218	129	115	216	737,19
12.06.2006	119	94	190	131	74	182	117,75
12.06.2006	120	82	186	131	74	182	419,44

12.06.2006	121	68	173	131	74	182	150,74
12.06.2006	122	62	165	131	74	182	29,21
12.06.2006	123	68	176	131	74	182	69,13
12.06.2006	124	128	214	131	74	182	460,67
12.06.2006	125	64	170	131	74	182	29,60
12.06.2006	127	136	218	131	74	182	5,04
12.06.2006	129	115	216	131	74	182	735,02
12.06.2006	119	94	190	120	82	186	515,96
12.06.2006	119	94	190	122	62	165	150,60
12.06.2006	120	82	186	122	62	165	376,75
12.06.2006	119	94	190	123	68	176	56,34
12.06.2006	120	82	186	123	68	176	460,98
12.06.2006	122	62	165	123	68	176	94,94
12.06.2006	119	94	190	124	128	214	569,55
12.06.2006	120	82	186	124	128	214	54,76
12.06.2006	122	62	165	124	128	214	431,33
12.06.2006	123	68	176	124	128	214	514,83
12.06.2006	119	94	190	125	64	170	109,56
12.06.2006	120	82	186	125	64	170	408,34
12.06.2006	122	62	165	125	64	170	46,11
12.06.2006	123	68	176	125	64	170	53,31
12.06.2006	124	128	214	125	64	170	462,38
12.06.2006	119	94	190	127	136	218	119,93
12.06.2006	120	82	186	127	136	218	398,66
12.06.2006	122	62	165	127	136	218	36,48
12.06.2006	123	68	176	127	136	218	63,62
12.06.2006	124	128	214	127	136	218	452,79
12.06.2006	125	64	170	127	136	218	10,55
12.06.2006	119	94	190	129	115	216	825,88
12.06.2006	120	82	186	129	115	216	366,16
12.06.2006	122	62	165	129	115	216	709,89
12.06.2006	123	68	176	129	115	216	777,13
12.06.2006	124	128	214	129	115	216	319,56

12.06.2006	125	64	170	129	115	216	729,74
12.06.2006	127	136	218	129	115	216	721,89
12.06.2006	119	94	190	131	74	182	126,32
12.06.2006	120	82	186	131	74	182	394,99
12.06.2006	122	62	165	131	74	182	26,48
12.06.2006	123	68	176	131	74	182	70,09
12.06.2006	124	128	214	131	74	182	449,30
12.06.2006	125	64	170	131	74	182	19,65
12.06.2006	127	136	218	131	74	182	10,77
12.06.2006	129	115	216	131	74	182	721,55
12.06.2006	119	94	190	120	82	186	546,37
12.06.2006	119	94	190	121	68	173	23,81
12.06.2006	120	82	186	121	68	173	522,66
12.06.2006	119	94	190	122	62	165	135,07
12.06.2006	120	82	186	122	62	165	416,53
12.06.2006	121	68	173	122	62	165	112,80
12.06.2006	119	94	190	123	68	176	82,89
12.06.2006	120	82	186	123	68	176	463,57
12.06.2006	121	68	173	123	68	176	59,35
12.06.2006	122	62	165	123	68	176	56,35
12.06.2006	119	94	190	124	128	214	579,09
12.06.2006	120	82	186	124	128	214	44,85
12.06.2006	121	68	173	124	128	214	555,29
12.06.2006	122	62	165	124	128	214	451,67
12.06.2006	123	68	176	124	128	214	496,65
12.06.2006	119	94	190	125	64	170	91,12
12.06.2006	120	82	186	125	64	170	455,67
12.06.2006	121	68	173	125	64	170	67,79
12.06.2006	122	62	165	125	64	170	47,21
12.06.2006	123	68	176	125	64	170	9,14
12.06.2006	124	128	214	125	64	170	489,05
12.06.2006	119	94	190	127	136	218	100,88
12.06.2006	120	82	186	127	136	218	445,76

12.06.2006	121	68	173	127	136	218	77,42
12.06.2006	122	62	165	127	136	218	39,99
12.06.2006	123	68	176	127	136	218	18,12
12.06.2006	124	128	214	127	136	218	479,08
12.06.2006	125	64	170	127	136	218	9,99
12.06.2006	119	94	190	129	115	216	825,87
12.06.2006	120	82	186	129	115	216	335,90
12.06.2006	121	68	173	129	115	216	802,51
12.06.2006	122	62	165	129	115	216	711,89
12.06.2006	123	68	176	129	115	216	747,63
12.06.2006	124	128	214	129	115	216	291,09
12.06.2006	125	64	170	129	115	216	741,47
12.06.2006	127	136	218	129	115	216	731,65
12.06.2006	119	94	190	131	74	182	116,48
12.06.2006	120	82	186	131	74	182	430,20
12.06.2006	121	68	173	131	74	182	93,02
12.06.2006	122	62	165	131	74	182	28,97
12.06.2006	123	68	176	131	74	182	33,68
12.06.2006	124	128	214	131	74	182	463,65
12.06.2006	125	64	170	131	74	182	25,46
12.06.2006	127	136	218	131	74	182	15,61
12.06.2006	129	115	216	131	74	182	717,26
12.06.2006	119	94	190	120	82	186	530,29
12.06.2006	119	94	190	121	68	173	7,03
12.06.2006	120	82	186	121	68	173	523,28
12.06.2006	119	94	190	122	62	165	105,29
12.06.2006	120	82	186	122	62	165	425,11
12.06.2006	121	68	173	122	62	165	98,26
12.06.2006	119	94	190	123	68	176	69,58
12.06.2006	120	82	186	123	68	176	462,32
12.06.2006	121	68	173	123	68	176	62,86
12.06.2006	122	62	165	123	68	176	41,19
12.06.2006	119	94	190	124	128	214	570,90

12.06.2006	120	82	186	124	128	214	44,04
12.06.2006	121	68	173	124	128	214	563,87
12.06.2006	122	62	165	124	128	214	465,62
12.06.2006	123	68	176	124	128	214	503,44
12.06.2006	119	94	190	125	64	170	91,15
12.06.2006	120	82	186	125	64	170	439,17
12.06.2006	121	68	173	125	64	170	84,14
12.06.2006	122	62	165	125	64	170	14,22
12.06.2006	123	68	176	125	64	170	28,00
12.06.2006	124	128	214	125	64	170	479,75
12.06.2006	119	94	190	127	136	218	82,10
12.06.2006	120	82	186	127	136	218	448,54
12.06.2006	121	68	173	127	136	218	75,17
12.06.2006	122	62	165	127	136	218	25,91
12.06.2006	123	68	176	127	136	218	15,28
12.06.2006	124	128	214	127	136	218	489,45
12.06.2006	125	64	170	127	136	218	13,04
12.06.2006	119	94	190	129	115	216	821,14
12.06.2006	120	82	186	129	115	216	338,18
12.06.2006	121	68	173	129	115	216	814,23
12.06.2006	122	62	165	129	115	216	718,84
12.06.2006	123	68	176	129	115	216	759,54
12.06.2006	124	128	214	129	115	216	295,93
12.06.2006	125	64	170	129	115	216	732,89
12.06.2006	127	136	218	129	115	216	744,47
12.06.2006	119	94	190	131	74	182	122,12
12.06.2006	120	82	186	131	74	182	413,12
12.06.2006	121	68	173	131	74	182	115,49
12.06.2006	122	62	165	131	74	182	36,88
12.06.2006	123	68	176	131	74	182	52,74
12.06.2006	124	128	214	131	74	182	454,88
12.06.2006	125	64	170	131	74	182	42,34
12.06.2006	127	136	218	131	74	182	43,33

12.06.2006	129	115	216	131	74	182	717,28
13.06.2006	119	94	190	120	82	186	544,58
13.06.2006	119	94	190	121	68	173	19,37
13.06.2006	120	82	186	121	68	173	561,24
13.06.2006	119	94	190	122	62	165	79,47
13.06.2006	120	82	186	122	62	165	469,79
13.06.2006	121	68	173	122	62	165	98,45
13.06.2006	119	94	190	123	68	176	73,63
13.06.2006	120	82	186	123	68	176	475,58
13.06.2006	121	68	173	123	68	176	92,65
13.06.2006	122	62	165	123	68	176	5,92
13.06.2006	119	94	190	124	128	214	560,12
13.06.2006	120	82	186	124	128	214	31,75
13.06.2006	121	68	173	124	128	214	576,27
13.06.2006	122	62	165	124	128	214	486,86
13.06.2006	123	68	176	124	128	214	492,56
13.06.2006	119	94	190	125	64	170	128,09
13.06.2006	120	82	186	125	64	170	416,52
13.06.2006	121	68	173	125	64	170	144,85
13.06.2006	122	62	165	125	64	170	59,25
13.06.2006	123	68	176	125	64	170	64,09
13.06.2006	124	128	214	125	64	170	432,11
13.06.2006	119	94	190	127	136	218	118,40
13.06.2006	120	82	186	127	136	218	428,02
13.06.2006	121	68	173	127	136	218	136,53
13.06.2006	122	62	165	127	136	218	42,00
13.06.2006	123	68	176	127	136	218	47,67
13.06.2006	124	128	214	127	136	218	444,90
13.06.2006	125	64	170	127	136	218	23,54
13.06.2006	119	94	190	129	115	216	817,28
13.06.2006	120	82	186	129	115	216	332,18
13.06.2006	121	68	173	129	115	216	830,48
13.06.2006	122	62	165	129	115	216	753,25

13.06.2006	123	68	176	129	115	216	758,43
13.06.2006	124	128	214	129	115	216	302,20
13.06.2006	125	64	170	129	115	216	694,57
13.06.2006	127	136	218	129	115	216	712,05
13.06.2006	119	94	190	131	74	182	121,54
13.06.2006	120	82	186	131	74	182	423,97
13.06.2006	121	68	173	131	74	182	139,34
13.06.2006	122	62	165	131	74	182	46,85
13.06.2006	123	68	176	131	74	182	52,32
13.06.2006	124	128	214	131	74	182	440,54
13.06.2006	125	64	170	131	74	182	17,13
13.06.2006	127	136	218	131	74	182	6,50
13.06.2006	129	115	216	131	74	182	706,62
13.06.2006	119	94	190	120	82	186	552,27
13.06.2006	119	94	190	121	68	173	5,55
13.06.2006	120	82	186	121	68	173	557,72
13.06.2006	119	94	190	122	62	165	101,53
13.06.2006	120	82	186	122	62	165	452,47
13.06.2006	121	68	173	122	62	165	106,74
13.06.2006	119	94	190	123	68	176	56,06
13.06.2006	120	82	186	123	68	176	499,72
13.06.2006	121	68	173	123	68	176	60,92
13.06.2006	122	62	165	123	68	176	47,25
13.06.2006	119	94	190	124	128	214	617,17
13.06.2006	120	82	186	124	128	214	78,55
13.06.2006	121	68	173	124	128	214	622,68
13.06.2006	122	62	165	124	128	214	519,19
13.06.2006	123	68	176	124	128	214	566,27
13.06.2006	119	94	190	125	64	170	123,42
13.06.2006	120	82	186	125	64	170	429,83
13.06.2006	121	68	173	125	64	170	128,73
13.06.2006	122	62	165	125	64	170	22,77
13.06.2006	123	68	176	125	64	170	69,93

13.06.2006	124	128	214	125	64	170	496,42
13.06.2006	119	94	190	127	136	218	155,47
13.06.2006	120	82	186	127	136	218	400,67
13.06.2006	121	68	173	127	136	218	160,64
13.06.2006	122	62	165	127	136	218	54,03
13.06.2006	123	68	176	127	136	218	100,36
13.06.2006	124	128	214	127	136	218	468,94
13.06.2006	125	64	170	127	136	218	33,88
13.06.2006	119	94	190	129	115	216	839,25
13.06.2006	120	82	186	129	115	216	339,48
13.06.2006	121	68	173	129	115	216	844,78
13.06.2006	122	62	165	129	115	216	748,34
13.06.2006	123	68	176	129	115	216	793,76
13.06.2006	124	128	214	129	115	216	261,20
13.06.2006	125	64	170	129	115	216	725,98
13.06.2006	127	136	218	129	115	216	703,10
13.06.2006	119	94	190	131	74	182	156,70
13.06.2006	120	82	186	131	74	182	399,41
13.06.2006	121	68	173	131	74	182	161,87
13.06.2006	122	62	165	131	74	182	55,24
13.06.2006	123	68	176	131	74	182	101,61
13.06.2006	124	128	214	131	74	182	467,68
13.06.2006	125	64	170	131	74	182	34,97
13.06.2006	127	136	218	131	74	182	1,26
13.06.2006	129	115	216	131	74	182	701,90
13.06.2006	119	94	190	120	82	186	554,84
13.06.2006	119	94	190	121	68	173	6,48
13.06.2006	120	82	186	121	68	173	555,74
13.06.2006	119	94	190	122	62	165	106,01
13.06.2006	120	82	186	122	62	165	449,92
13.06.2006	121	68	173	122	62	165	106,24
13.06.2006	119	94	190	123	68	176	107,30
13.06.2006	120	82	186	123	68	176	453,67

13.06.2006	121	68	173	123	68	176	106,39
13.06.2006	122	62	165	123	68	176	18,92
13.06.2006	119	94	190	124	128	214	610,70
13.06.2006	120	82	186	124	128	214	57,75
13.06.2006	121	68	173	124	128	214	611,43
13.06.2006	122	62	165	124	128	214	505,38
13.06.2006	123	68	176	124	128	214	508,47
13.06.2006	119	94	190	125	64	170	117,23
13.06.2006	120	82	186	125	64	170	438,69
13.06.2006	121	68	173	125	64	170	117,50
13.06.2006	122	62	165	125	64	170	11,26
13.06.2006	123	68	176	125	64	170	22,98
13.06.2006	124	128	214	125	64	170	494,13
13.06.2006	119	94	190	127	136	218	126,91
13.06.2006	120	82	186	127	136	218	431,31
13.06.2006	121	68	173	127	136	218	126,62
13.06.2006	122	62	165	127	136	218	22,62
13.06.2006	123	68	176	127	136	218	22,97
13.06.2006	124	128	214	127	136	218	486,32
13.06.2006	125	64	170	127	136	218	13,96
13.06.2006	119	94	190	129	115	216	836,18
13.06.2006	120	82	186	129	115	216	310,63
13.06.2006	121	68	173	129	115	216	838,29
13.06.2006	122	62	165	129	115	216	736,40
13.06.2006	123	68	176	129	115	216	743,77
13.06.2006	124	128	214	129	115	216	274,20
13.06.2006	125	64	170	129	115	216	725,61
13.06.2006	127	136	218	129	115	216	720,82
13.06.2006	119	94	190	131	74	182	134,72
13.06.2006	120	82	186	131	74	182	425,73
13.06.2006	121	68	173	131	74	182	134,11
13.06.2006	122	62	165	131	74	182	32,30
13.06.2006	123	68	176	131	74	182	28,16

13.06.2006	124	128	214	131	74	182	480,41
13.06.2006	125	64	170	131	74	182	24,07
13.06.2006	127	136	218	131	74	182	10,12
13.06.2006	129	115	216	131	74	182	717,01
13.06.2006	119	94	190	120	82	186	530,53
13.06.2006	119	94	190	121	68	173	37,02
13.06.2006	120	82	186	121	68	173	566,09
13.06.2006	119	94	190	122	62	165	42,52
13.06.2006	120	82	186	122	62	165	493,58
13.06.2006	121	68	173	122	62	165	79,13
13.06.2006	119	94	190	123	68	176	64,09
13.06.2006	120	82	186	123	68	176	469,02
13.06.2006	121	68	173	123	68	176	101,11
13.06.2006	122	62	165	123	68	176	24,68
13.06.2006	119	94	190	124	128	214	571,26
13.06.2006	120	82	186	124	128	214	52,66
13.06.2006	121	68	173	124	128	214	606,14
13.06.2006	122	62	165	124	128	214	535,65
13.06.2006	123	68	176	124	128	214	510,99
13.06.2006	119	94	190	125	64	170	98,18
13.06.2006	120	82	186	125	64	170	432,52
13.06.2006	121	68	173	125	64	170	134,44
13.06.2006	122	62	165	125	64	170	62,52
13.06.2006	123	68	176	125	64	170	38,07
13.06.2006	124	128	214	125	64	170	473,84
13.06.2006	119	94	190	127	136	218	87,47
13.06.2006	120	82	186	127	136	218	443,26
13.06.2006	121	68	173	127	136	218	123,82
13.06.2006	122	62	165	127	136	218	52,09
13.06.2006	123	68	176	127	136	218	27,93
13.06.2006	124	128	214	127	136	218	484,56
13.06.2006	125	64	170	127	136	218	10,74
13.06.2006	119	94	190	129	115	216	795,45

13.06.2006	120	82	186	129	115	216	321,06
13.06.2006	121	68	173	129	115	216	826,83
13.06.2006	122	62	165	129	115	216	765,73
13.06.2006	123	68	176	129	115	216	741,46
13.06.2006	124	128	214	129	115	216	268,44
13.06.2006	125	64	170	129	115	216	703,39
13.06.2006	127	136	218	129	115	216	713,66
13.06.2006	119	94	190	131	74	182	102,85
13.06.2006	120	82	186	131	74	182	428,56
13.06.2006	121	68	173	131	74	182	139,47
13.06.2006	122	62	165	131	74	182	65,28
13.06.2006	123	68	176	131	74	182	40,60
13.06.2006	124	128	214	131	74	182	470,39
13.06.2006	125	64	170	131	74	182	8,05
13.06.2006	127	136	218	131	74	182	16,25
13.06.2006	129	115	216	131	74	182	701,77
13.06.2006	119	94	190	120	82	186	547,87
13.06.2006	119	94	190	121	68	173	24,43
13.06.2006	120	82	186	121	68	173	564,73
13.06.2006	119	94	190	122	62	165	76,21
13.06.2006	120	82	186	122	62	165	471,66
13.06.2006	121	68	173	122	62	165	94,52
13.06.2006	119	94	190	123	68	176	69,99
13.06.2006	120	82	186	123	68	176	478,99
13.06.2006	121	68	173	123	68	176	90,55
13.06.2006	122	62	165	123	68	176	13,58
13.06.2006	119	94	190	124	128	214	589,73
13.06.2006	120	82	186	124	128	214	44,82
13.06.2006	121	68	173	124	128	214	606,07
13.06.2006	122	62	165	124	128	214	513,55
13.06.2006	123	68	176	124	128	214	521,24
13.06.2006	119	94	190	125	64	170	116,60
13.06.2006	120	82	186	125	64	170	431,72

13.06.2006	121	68	173	125	64	170	135,52
13.06.2006	122	62	165	125	64	170	41,02
13.06.2006	123	68	176	125	64	170	47,30
13.06.2006	124	128	214	125	64	170	473,94
13.06.2006	119	94	190	127	136	218	124,68
13.06.2006	120	82	186	127	136	218	423,30
13.06.2006	121	68	173	127	136	218	142,92
13.06.2006	122	62	165	127	136	218	48,57
13.06.2006	123	68	176	127	136	218	56,05
13.06.2006	124	128	214	127	136	218	465,37
13.06.2006	125	64	170	127	136	218	9,56
13.06.2006	119	94	190	129	115	216	851,99
13.06.2006	120	82	186	129	115	216	377,30
13.06.2006	121	68	173	129	115	216	862,18
13.06.2006	122	62	165	129	115	216	780,27
13.06.2006	123	68	176	129	115	216	791,10
13.06.2006	124	128	214	129	115	216	337,19
13.06.2006	125	64	170	129	115	216	746,27
13.06.2006	127	136	218	129	115	216	736,77
13.06.2006	119	94	190	131	74	182	130,68
13.06.2006	120	82	186	131	74	182	418,92
13.06.2006	121	68	173	131	74	182	150,41
13.06.2006	122	62	165	131	74	182	56,24
13.06.2006	123	68	176	131	74	182	60,73
13.06.2006	124	128	214	131	74	182	461,47
13.06.2006	125	64	170	131	74	182	16,19
13.06.2006	127	136	218	131	74	182	14,93
13.06.2006	129	115	216	131	74	182	737,88
13.06.2006	119	94	190	120	82	186	542,23
13.06.2006	119	94	190	121	68	173	13,58
13.06.2006	120	82	186	121	68	173	553,19
13.06.2006	119	94	190	122	62	165	77,12
13.06.2006	120	82	186	122	62	165	467,15

13.06.2006	121	68	173	122	62	165	89,66
13.06.2006	119	94	190	123	68	176	78,37
13.06.2006	120	82	186	123	68	176	466,35
13.06.2006	121	68	173	123	68	176	91,00
13.06.2006	122	62	165	123	68	176	1,99
13.06.2006	119	94	190	124	128	214	556,48
13.06.2006	120	82	186	124	128	214	29,49
13.06.2006	121	68	173	124	128	214	567,05
13.06.2006	122	62	165	124	128	214	482,38
13.06.2006	123	68	176	124	128	214	481,67
13.06.2006	119	94	190	125	64	170	117,33
13.06.2006	120	82	186	125	64	170	425,21
13.06.2006	121	68	173	125	64	170	128,94
13.06.2006	122	62	165	125	64	170	42,62
13.06.2006	123	68	176	125	64	170	42,19
13.06.2006	124	128	214	125	64	170	440,08
13.06.2006	119	94	190	127	136	218	104,13
13.06.2006	120	82	186	127	136	218	442,16
13.06.2006	121	68	173	127	136	218	116,82
13.06.2006	122	62	165	127	136	218	27,23
13.06.2006	123	68	176	127	136	218	25,82
13.06.2006	124	128	214	127	136	218	458,01
13.06.2006	125	64	170	127	136	218	24,53
13.06.2006	119	94	190	129	115	216	795,84
13.06.2006	120	82	186	129	115	216	284,48
13.06.2006	121	68	173	129	115	216	805,00
13.06.2006	122	62	165	129	115	216	725,77
13.06.2006	123	68	176	129	115	216	725,35
13.06.2006	124	128	214	129	115	216	259,18
13.06.2006	125	64	170	129	115	216	683,16
13.06.2006	127	136	218	129	115	216	703,47
13.06.2006	119	94	190	131	74	182	122,42
13.06.2006	120	82	186	131	74	182	420,63

13.06.2006	121	68	173	131	74	182	134,26
13.06.2006	122	62	165	131	74	182	46,58
13.06.2006	123	68	176	131	74	182	45,90
13.06.2006	124	128	214	131	74	182	435,80
13.06.2006	125	64	170	131	74	182	6,75
13.06.2006	127	136	218	131	74	182	24,93
13.06.2006	129	115	216	131	74	182	679,79
13.06.2006	119	94	190	120	82	186	616,80
13.06.2006	119	94	190	122	62	165	58,09
13.06.2006	120	82	186	122	62	165	558,74
13.06.2006	119	94	190	123	68	176	53,37
13.06.2006	120	82	186	123	68	176	563,74
13.06.2006	122	62	165	123	68	176	6,12
13.06.2006	119	94	190	124	128	214	561,13
13.06.2006	120	82	186	124	128	214	80,99
13.06.2006	122	62	165	124	128	214	503,61
13.06.2006	123	68	176	124	128	214	508,97
13.06.2006	119	94	190	125	64	170	100,20
13.06.2006	120	82	186	125	64	170	516,69
13.06.2006	122	62	165	125	64	170	42,48
13.06.2006	123	68	176	125	64	170	47,98
13.06.2006	124	128	214	125	64	170	461,15
13.06.2006	119	94	190	127	136	218	96,76
13.06.2006	120	82	186	127	136	218	521,20
13.06.2006	122	62	165	127	136	218	39,53
13.06.2006	123	68	176	127	136	218	43,48
13.06.2006	124	128	214	127	136	218	467,78
13.06.2006	125	64	170	127	136	218	18,25
13.06.2006	119	94	190	129	115	216	812,26
13.06.2006	120	82	186	129	115	216	308,52
13.06.2006	122	62	165	129	115	216	758,43
13.06.2006	123	68	176	129	115	216	764,35
13.06.2006	124	128	214	129	115	216	298,22

13.06.2006	125	64	170	129	115	216	717,10
13.06.2006	127	136	218	129	115	216	727,86
13.06.2006	119	94	190	131	74	182	106,95
13.06.2006	120	82	186	131	74	182	510,15
13.06.2006	122	62	165	131	74	182	48,95
13.06.2006	123	68	176	131	74	182	53,64
13.06.2006	124	128	214	131	74	182	456,06
13.06.2006	125	64	170	131	74	182	13,16
13.06.2006	127	136	218	131	74	182	12,61
13.06.2006	129	115	216	131	74	182	715,35
13.06.2006	119	94	190	120	82	186	524,91
13.06.2006	119	94	190	121	68	173	21,56
13.06.2006	120	82	186	121	68	173	537,58
13.06.2006	119	94	190	122	62	165	71,37
13.06.2006	120	82	186	122	62	165	454,21
13.06.2006	121	68	173	122	62	165	87,37
13.06.2006	119	94	190	123	68	176	70,35
13.06.2006	120	82	186	123	68	176	454,87
13.06.2006	121	68	173	123	68	176	85,83
13.06.2006	122	62	165	123	68	176	3,02
13.06.2006	119	94	190	124	128	214	567,01
13.06.2006	120	82	186	124	128	214	54,95
13.06.2006	121	68	173	124	128	214	578,52
13.06.2006	122	62	165	124	128	214	497,14
13.06.2006	123	68	176	124	128	214	497,58
13.06.2006	119	94	190	125	64	170	105,99
13.06.2006	120	82	186	125	64	170	418,97
13.06.2006	121	68	173	125	64	170	119,27
13.06.2006	122	62	165	125	64	170	37,09
13.06.2006	123	68	176	125	64	170	36,95
13.06.2006	124	128	214	125	64	170	461,12
13.06.2006	119	94	190	127	136	218	82,67
13.06.2006	120	82	186	127	136	218	442,85

13.06.2006	121	68	173	127	136	218	94,91
13.06.2006	122	62	165	127	136	218	21,55
13.06.2006	123	68	176	127	136	218	19,55
13.06.2006	124	128	214	127	136	218	484,45
13.06.2006	125	64	170	127	136	218	24,67
13.06.2006	119	94	190	129	115	216	801,01
13.06.2006	120	82	186	129	115	216	318,51
13.06.2006	121	68	173	129	115	216	808,99
13.06.2006	122	62	165	129	115	216	735,01
13.06.2006	123	68	176	129	115	216	734,88
13.06.2006	124	128	214	129	115	216	263,74
13.06.2006	125	64	170	129	115	216	697,96
13.06.2006	127	136	218	129	115	216	719,16
13.06.2006	119	94	190	131	74	182	121,91
13.06.2006	120	82	186	131	74	182	403,45
13.06.2006	121	68	173	131	74	182	136,60
13.06.2006	122	62	165	131	74	182	50,77
13.06.2006	123	68	176	131	74	182	51,57
13.06.2006	124	128	214	131	74	182	446,62
13.06.2006	125	64	170	131	74	182	19,63
13.06.2006	127	136	218	131	74	182	43,49
13.06.2006	129	115	216	131	74	182	686,40
14.06.2006	119	94	190	120	82	186	538,70
14.06.2006	119	94	190	121	68	173	28,48
14.06.2006	120	82	186	121	68	173	550,13
14.06.2006	119	94	190	122	62	165	96,00
14.06.2006	120	82	186	122	62	165	443,19
14.06.2006	121	68	173	122	62	165	112,05
14.06.2006	119	94	190	123	68	176	66,75
14.06.2006	120	82	186	123	68	176	471,96
14.06.2006	121	68	173	123	68	176	81,59
14.06.2006	122	62	165	123	68	176	30,46
14.06.2006	119	94	190	124	128	214	593,68

14.06.2006	120	82	186	124	128	214	56,00
14.06.2006	121	68	173	124	128	214	604,57
14.06.2006	122	62	165	124	128	214	498,38
14.06.2006	123	68	176	124	128	214	526,93
14.06.2006	119	94	190	125	64	170	102,55
14.06.2006	120	82	186	125	64	170	436,16
14.06.2006	121	68	173	125	64	170	116,69
14.06.2006	122	62	165	125	64	170	10,18
14.06.2006	123	68	176	125	64	170	35,88
14.06.2006	124	128	214	125	64	170	491,19
14.06.2006	119	94	190	127	136	218	101,29
14.06.2006	120	82	186	127	136	218	437,89
14.06.2006	121	68	173	127	136	218	117,13
14.06.2006	122	62	165	127	136	218	5,31
14.06.2006	123	68	176	127	136	218	35,55
14.06.2006	124	128	214	127	136	218	493,08
14.06.2006	125	64	170	127	136	218	7,71
14.06.2006	119	94	190	129	115	216	831,26
14.06.2006	120	82	186	129	115	216	337,63
14.06.2006	121	68	173	129	115	216	835,61
14.06.2006	122	62	165	129	115	216	741,60
14.06.2006	123	68	176	129	115	216	766,55
14.06.2006	124	128	214	129	115	216	288,58
14.06.2006	125	64	170	129	115	216	732,80
14.06.2006	127	136	218	129	115	216	736,56
14.06.2006	119	94	190	131	74	182	125,21
14.06.2006	120	82	186	131	74	182	414,56
14.06.2006	121	68	173	131	74	182	141,17
14.06.2006	122	62	165	131	74	182	29,32
14.06.2006	123	68	176	131	74	182	59,63
14.06.2006	124	128	214	131	74	182	469,87
14.06.2006	125	64	170	131	74	182	25,39
14.06.2006	127	136	218	131	74	182	24,10

14.06.2006	129	115	216	131	74	182	715,79
14.06.2006	119	94	190	120	82	186	499,67
14.06.2006	119	94	190	121	68	173	30,04
14.06.2006	120	82	186	121	68	173	526,41
14.06.2006	119	94	190	122	62	165	57,03
14.06.2006	120	82	186	122	62	165	446,51
14.06.2006	121	68	173	122	62	165	86,89
14.06.2006	119	94	190	123	68	176	45,27
14.06.2006	120	82	186	123	68	176	465,68
14.06.2006	121	68	173	123	68	176	74,93
14.06.2006	122	62	165	123	68	176	20,89
14.06.2006	119	94	190	124	128	214	541,83
14.06.2006	120	82	186	124	128	214	42,58
14.06.2006	121	68	173	124	128	214	568,41
14.06.2006	122	62	165	124	128	214	488,89
14.06.2006	123	68	176	124	128	214	508,12
14.06.2006	119	94	190	125	64	170	72,54
14.06.2006	120	82	186	125	64	170	436,73
14.06.2006	121	68	173	125	64	170	102,58
14.06.2006	122	62	165	125	64	170	17,72
14.06.2006	123	68	176	125	64	170	29,75
14.06.2006	124	128	214	125	64	170	479,22
14.06.2006	119	94	190	127	136	218	69,40
14.06.2006	120	82	186	127	136	218	437,97
14.06.2006	121	68	173	127	136	218	99,42
14.06.2006	122	62	165	127	136	218	13,69
14.06.2006	123	68	176	127	136	218	27,84
14.06.2006	124	128	214	127	136	218	480,43
14.06.2006	125	64	170	127	136	218	4,25
14.06.2006	119	94	190	129	115	216	786,17
14.06.2006	120	82	186	129	115	216	335,53
14.06.2006	121	68	173	129	115	216	808,10
14.06.2006	122	62	165	129	115	216	740,82

14.06.2006	123	68	176	129	115	216	761,52
14.06.2006	124	128	214	129	115	216	300,20
14.06.2006	125	64	170	129	115	216	735,43
14.06.2006	127	136	218	129	115	216	735,54
14.06.2006	119	94	190	131	74	182	79,45
14.06.2006	120	82	186	131	74	182	430,23
14.06.2006	121	68	173	131	74	182	109,48
14.06.2006	122	62	165	131	74	182	23,77
14.06.2006	123	68	176	131	74	182	36,71
14.06.2006	124	128	214	131	74	182	472,74
14.06.2006	125	64	170	131	74	182	7,01
14.06.2006	127	136	218	131	74	182	10,21
14.06.2006	129	115	216	131	74	182	729,87
14.06.2006	119	94	190	120	82	186	513,19
14.06.2006	119	94	190	121	68	173	68,28
14.06.2006	120	82	186	121	68	173	562,15
14.06.2006	119	94	190	122	62	165	40,57
14.06.2006	120	82	186	122	62	165	482,79
14.06.2006	121	68	173	122	62	165	81,38
14.06.2006	119	94	190	123	68	176	57,89
14.06.2006	120	82	186	123	68	176	509,85
14.06.2006	121	68	173	123	68	176	53,91
14.06.2006	122	62	165	123	68	176	39,88
14.06.2006	119	94	190	124	128	214	553,47
14.06.2006	120	82	186	124	128	214	50,52
14.06.2006	121	68	173	124	128	214	599,59
14.06.2006	122	62	165	124	128	214	521,49
14.06.2006	123	68	176	124	128	214	546,58
14.06.2006	119	94	190	125	64	170	84,91
14.06.2006	120	82	186	125	64	170	428,59
14.06.2006	121	68	173	125	64	170	138,34
14.06.2006	122	62	165	125	64	170	57,01
14.06.2006	123	68	176	125	64	170	93,24

14.06.2006	124	128	214	125	64	170	468,58
14.06.2006	119	94	190	127	136	218	83,17
14.06.2006	120	82	186	127	136	218	430,43
14.06.2006	121	68	173	127	136	218	136,27
14.06.2006	122	62	165	127	136	218	54,92
14.06.2006	123	68	176	127	136	218	91,13
14.06.2006	124	128	214	127	136	218	470,35
14.06.2006	125	64	170	127	136	218	2,12
14.06.2006	119	94	190	129	115	216	836,04
14.06.2006	120	82	186	129	115	216	374,02
14.06.2006	121	68	173	129	115	216	868,79
14.06.2006	122	62	165	129	115	216	798,96
14.06.2006	123	68	176	129	115	216	815,05
14.06.2006	124	128	214	129	115	216	323,50
14.06.2006	125	64	170	129	115	216	753,25
14.06.2006	127	136	218	129	115	216	754,67
14.06.2006	119	94	190	131	74	182	96,29
14.06.2006	120	82	186	131	74	182	417,79
14.06.2006	121	68	173	131	74	182	147,31
14.06.2006	122	62	165	131	74	182	66,03
14.06.2006	123	68	176	131	74	182	100,05
14.06.2006	124	128	214	131	74	182	457,42
14.06.2006	125	64	170	131	74	182	12,11
14.06.2006	127	136	218	131	74	182	13,41
14.06.2006	129	115	216	131	74	182	741,26
19.06.2006	119	94	190	120	82	186	537,66
19.06.2006	119	94	190	121	68	173	32,95
19.06.2006	120	82	186	121	68	173	563,88
19.06.2006	119	94	190	122	62	165	119,31
19.06.2006	120	82	186	122	62	165	420,72
19.06.2006	121	68	173	122	62	165	149,16
19.06.2006	119	94	190	123	68	176	53,41
19.06.2006	120	82	186	123	68	176	484,28

19.06.2006	121	68	173	123	68	176	81,39
19.06.2006	122	62	165	123	68	176	67,99
19.06.2006	119	94	190	124	128	214	569,39
19.06.2006	120	82	186	124	128	214	46,62
19.06.2006	121	68	173	124	128	214	594,34
19.06.2006	122	62	165	124	128	214	454,31
19.06.2006	123	68	176	124	128	214	516,00
19.06.2006	119	94	190	125	64	170	100,85
19.06.2006	120	82	186	125	64	170	438,53
19.06.2006	121	68	173	125	64	170	130,70
19.06.2006	122	62	165	125	64	170	18,49
19.06.2006	123	68	176	125	64	170	49,66
19.06.2006	124	128	214	125	64	170	471,72
19.06.2006	119	94	190	127	136	218	100,00
19.06.2006	120	82	186	127	136	218	438,90
19.06.2006	121	68	173	127	136	218	129,54
19.06.2006	122	62	165	127	136	218	19,67
19.06.2006	123	68	176	127	136	218	48,31
19.06.2006	124	128	214	127	136	218	471,90
19.06.2006	125	64	170	127	136	218	2,64
19.06.2006	119	94	190	129	115	216	774,24
19.06.2006	120	82	186	129	115	216	293,99
19.06.2006	121	68	173	129	115	216	794,11
19.06.2006	122	62	165	129	115	216	668,68
19.06.2006	123	68	176	129	115	216	722,44
19.06.2006	124	128	214	129	115	216	247,51
19.06.2006	125	64	170	129	115	216	684,26
19.06.2006	127	136	218	129	115	216	683,84
19.06.2006	119	94	190	131	74	182	119,01
19.06.2006	120	82	186	131	74	182	421,57
19.06.2006	121	68	173	131	74	182	149,10
19.06.2006	122	62	165	131	74	182	2,38
19.06.2006	123	68	176	131	74	182	68,09

19.06.2006	124	128	214	131	74	182	455,33
19.06.2006	125	64	170	131	74	182	18,44
19.06.2006	127	136	218	131	74	182	19,91
19.06.2006	129	115	216	131	74	182	670,14
19.06.2006	119	94	190	120	82	186	532,38
19.06.2006	119	94	190	121	68	173	14,81
19.06.2006	120	82	186	121	68	173	534,84
19.06.2006	119	94	190	122	62	165	127,55
19.06.2006	120	82	186	122	62	165	407,12
19.06.2006	121	68	173	122	62	165	132,94
19.06.2006	119	94	190	123	68	176	72,56
19.06.2006	120	82	186	123	68	176	461,27
19.06.2006	121	68	173	123	68	176	78,74
19.06.2006	122	62	165	123	68	176	55,02
19.06.2006	119	94	190	124	128	214	585,59
19.06.2006	120	82	186	124	128	214	56,95
19.06.2006	121	68	173	124	128	214	587,51
19.06.2006	122	62	165	124	128	214	461,35
19.06.2006	123	68	176	124	128	214	515,07
19.06.2006	119	94	190	125	64	170	118,12
19.06.2006	120	82	186	125	64	170	416,24
19.06.2006	121	68	173	125	64	170	123,52
19.06.2006	122	62	165	125	64	170	9,44
19.06.2006	123	68	176	125	64	170	45,61
19.06.2006	124	128	214	125	64	170	470,37
19.06.2006	119	94	190	127	136	218	119,59
19.06.2006	120	82	186	127	136	218	414,64
19.06.2006	121	68	173	127	136	218	124,89
19.06.2006	122	62	165	127	136	218	8,06
19.06.2006	123	68	176	127	136	218	47,12
19.06.2006	124	128	214	127	136	218	468,75
19.06.2006	125	64	170	127	136	218	1,66
19.06.2006	119	94	190	129	115	216	824,22

19.06.2006	120	82	186	129	115	216	342,98
19.06.2006	121	68	173	129	115	216	822,59
19.06.2006	122	62	165	129	115	216	710,82
19.06.2006	123	68	176	129	115	216	759,86
19.06.2006	124	128	214	129	115	216	289,51
19.06.2006	125	64	170	129	115	216	718,82
19.06.2006	127	136	218	129	115	216	717,16
19.06.2006	119	94	190	131	74	182	133,27
19.06.2006	120	82	186	131	74	182	404,11
19.06.2006	121	68	173	131	74	182	139,60
19.06.2006	122	62	165	131	74	182	11,08
19.06.2006	123	68	176	131	74	182	60,92
19.06.2006	124	128	214	131	74	182	458,76
19.06.2006	125	64	170	131	74	182	17,94
19.06.2006	127	136	218	131	74	182	17,24
19.06.2006	129	115	216	131	74	182	711,00
19.06.2006	119	94	190	120	82	186	532,35
19.06.2006	119	94	190	121	68	173	38,02
19.06.2006	120	82	186	121	68	173	564,29
19.06.2006	119	94	190	122	62	165	113,03
19.06.2006	120	82	186	122	62	165	430,29
19.06.2006	121	68	173	122	62	165	150,51
19.06.2006	119	94	190	123	68	176	68,89
19.06.2006	120	82	186	123	68	176	477,17
19.06.2006	121	68	173	123	68	176	106,91
19.06.2006	122	62	165	123	68	176	47,72
19.06.2006	119	94	190	124	128	214	549,14
19.06.2006	120	82	186	124	128	214	31,48
19.06.2006	121	68	173	124	128	214	580,05
19.06.2006	122	62	165	124	128	214	449,79
19.06.2006	123	68	176	124	128	214	496,11
19.06.2006	119	94	190	125	64	170	97,48
19.06.2006	120	82	186	125	64	170	447,72

19.06.2006	121	68	173	125	64	170	135,30
19.06.2006	122	62	165	125	64	170	17,46
19.06.2006	123	68	176	125	64	170	30,51
19.06.2006	124	128	214	125	64	170	467,13
19.06.2006	119	94	190	127	136	218	101,14
19.06.2006	120	82	186	127	136	218	441,21
19.06.2006	121	68	173	127	136	218	138,68
19.06.2006	122	62	165	127	136	218	11,93
19.06.2006	123	68	176	127	136	218	36,17
19.06.2006	124	128	214	127	136	218	460,42
19.06.2006	125	64	170	127	136	218	7,54
19.06.2006	119	94	190	129	115	216	795,39
19.06.2006	120	82	186	129	115	216	339,50
19.06.2006	121	68	173	129	115	216	818,87
19.06.2006	122	62	165	129	115	216	714,95
19.06.2006	123	68	176	129	115	216	756,42
19.06.2006	124	128	214	129	115	216	308,50
19.06.2006	125	64	170	129	115	216	731,07
19.06.2006	127	136	218	129	115	216	723,68
19.06.2006	119	94	190	131	74	182	112,37
19.06.2006	120	82	186	131	74	182	430,64
19.06.2006	121	68	173	131	74	182	149,82
19.06.2006	122	62	165	131	74	182	0,84
19.06.2006	123	68	176	131	74	182	47,24
19.06.2006	124	128	214	131	74	182	450,09
19.06.2006	125	64	170	131	74	182	17,08
19.06.2006	127	136	218	131	74	182	11,32
19.06.2006	129	115	216	131	74	182	715,02
19.06.2006	119	94	190	120	82	186	540,17
19.06.2006	119	94	190	121	68	173	37,92
19.06.2006	120	82	186	121	68	173	570,31
19.06.2006	119	94	190	122	62	165	121,11
19.06.2006	120	82	186	122	62	165	420,17

19.06.2006	121	68	173	122	62	165	154,65
19.06.2006	119	94	190	123	68	176	71,61
19.06.2006	120	82	186	123	68	176	468,85
19.06.2006	121	68	173	123	68	176	105,24
19.06.2006	122	62	165	123	68	176	49,62
19.06.2006	119	94	190	124	128	214	597,01
19.06.2006	120	82	186	124	128	214	61,08
19.06.2006	121	68	173	124	128	214	626,20
19.06.2006	122	62	165	124	128	214	477,80
19.06.2006	123	68	176	124	128	214	526,01
19.06.2006	119	94	190	125	64	170	122,09
19.06.2006	120	82	186	125	64	170	418,09
19.06.2006	121	68	173	125	64	170	153,47
19.06.2006	122	62	165	125	64	170	15,10
19.06.2006	123	68	176	125	64	170	51,13
19.06.2006	124	128	214	125	64	170	475,02
19.06.2006	119	94	190	127	136	218	117,48
19.06.2006	120	82	186	127	136	218	423,25
19.06.2006	121	68	173	127	136	218	150,52
19.06.2006	122	62	165	127	136	218	5,35
19.06.2006	123	68	176	127	136	218	45,87
19.06.2006	124	128	214	127	136	218	480,69
19.06.2006	125	64	170	127	136	218	11,83
19.06.2006	119	94	190	129	115	216	832,23
19.06.2006	120	82	186	129	115	216	357,02
19.06.2006	121	68	173	129	115	216	853,91
19.06.2006	122	62	165	129	115	216	723,72
19.06.2006	123	68	176	129	115	216	766,48
19.06.2006	124	128	214	129	115	216	301,37
19.06.2006	125	64	170	129	115	216	716,62
19.06.2006	127	136	218	129	115	216	725,15
19.06.2006	119	94	190	131	74	182	136,42
19.06.2006	120	82	186	131	74	182	410,31

19.06.2006	121	68	173	131	74	182	171,95
19.06.2006	122	62	165	131	74	182	24,69
19.06.2006	123	68	176	131	74	182	67,41
19.06.2006	124	128	214	131	74	182	468,91
19.06.2006	125	64	170	131	74	182	38,15
19.06.2006	127	136	218	131	74	182	29,98
19.06.2006	129	115	216	131	74	182	721,79
19.06.2006	118	96	195	119	96	195	233,09
19.06.2006	118	96	195	120	82	186	409,33
19.06.2006	119	94	190	120	82	186	630,76
19.06.2006	118	96	195	121	68	173	241,25
19.06.2006	119	94	190	121	68	173	8,18
19.06.2006	120	82	186	121	68	173	638,79
19.06.2006	118	96	195	122	62	165	7,60
19.06.2006	119	94	190	122	62	165	228,42
19.06.2006	120	82	186	122	62	165	411,48
19.06.2006	121	68	173	122	62	165	236,59
19.06.2006	118	96	195	123	68	176	79,85
19.06.2006	119	94	190	123	68	176	154,36
19.06.2006	120	82	186	123	68	176	486,93
19.06.2006	121	68	173	123	68	176	162,48
19.06.2006	122	62	165	123	68	176	76,24
19.06.2006	118	96	195	124	128	214	459,70
19.06.2006	119	94	190	124	128	214	686,55
19.06.2006	120	82	186	124	128	214	70,17
19.06.2006	121	68	173	124	128	214	694,66
19.06.2006	122	62	165	124	128	214	462,64
19.06.2006	123	68	176	124	128	214	538,81
19.06.2006	118	96	195	125	64	170	4,01
19.06.2006	119	94	190	125	64	170	233,60
19.06.2006	120	82	186	125	64	170	410,44
19.06.2006	121	68	173	125	64	170	241,76
19.06.2006	122	62	165	125	64	170	11,21

19.06.2006	123	68	176	125	64	170	79,89
19.06.2006	124	128	214	125	64	170	460,37
19.06.2006	118	96	195	127	136	218	7,63
19.06.2006	119	94	190	127	136	218	226,01
19.06.2006	120	82	186	127	136	218	414,82
19.06.2006	121	68	173	127	136	218	234,18
19.06.2006	122	62	165	127	136	218	3,91
19.06.2006	123	68	176	127	136	218	73,30
19.06.2006	124	128	214	127	136	218	465,75
19.06.2006	125	64	170	127	136	218	10,18
19.06.2006	118	96	195	129	115	216	703,08
19.06.2006	119	94	190	129	115	216	936,02
19.06.2006	120	82	186	129	115	216	349,81
19.06.2006	121	68	173	129	115	216	944,20
19.06.2006	122	62	165	129	115	216	707,61
19.06.2006	123	68	176	129	115	216	782,61
19.06.2006	124	128	214	129	115	216	279,76
19.06.2006	125	64	170	129	115	216	702,78
19.06.2006	127	136	218	129	115	216	710,06
19.06.2006	118	96	195	131	74	182	47,43
19.06.2006	119	94	190	131	74	182	225,92
19.06.2006	120	82	186	131	74	182	405,26
19.06.2006	121	68	173	131	74	182	234,02
19.06.2006	122	62	165	131	74	182	40,61
19.06.2006	123	68	176	131	74	182	88,01
19.06.2006	124	128	214	131	74	182	460,67
19.06.2006	125	64	170	131	74	182	51,40
19.06.2006	127	136	218	131	74	182	43,37
19.06.2006	129	115	216	131	74	182	714,57
19.06.2006	119	94	190	120	82	186	522,98
19.06.2006	119	94	190	121	68	173	29,35
19.06.2006	120	82	186	121	68	173	543,68
19.06.2006	119	94	190	122	62	165	132,73

19.06.2006	120	82	186	122	62	165	390,44
19.06.2006	121	68	173	122	62	165	155,29
19.06.2006	119	94	190	123	68	176	54,46
19.06.2006	120	82	186	123	68	176	470,62
19.06.2006	121	68	173	123	68	176	81,01
19.06.2006	122	62	165	123	68	176	80,43
19.06.2006	119	94	190	124	128	214	630,17
19.06.2006	120	82	186	124	128	214	127,90
19.06.2006	121	68	173	124	128	214	648,17
19.06.2006	122	62	165	124	128	214	499,79
19.06.2006	123	68	176	124	128	214	580,07
19.06.2006	119	94	190	125	64	170	115,07
19.06.2006	120	82	186	125	64	170	408,66
19.06.2006	121	68	173	125	64	170	138,71
19.06.2006	122	62	165	125	64	170	18,94
19.06.2006	123	68	176	125	64	170	61,96
19.06.2006	124	128	214	125	64	170	518,57
19.06.2006	119	94	190	129	115	216	831,02
19.06.2006	120	82	186	129	115	216	363,11
19.06.2006	121	68	173	129	115	216	844,73
19.06.2006	122	62	165	129	115	216	707,19
19.06.2006	123	68	176	129	115	216	785,30
19.06.2006	124	128	214	129	115	216	235,86
19.06.2006	125	64	170	129	115	216	726,10
19.06.2006	119	94	190	131	74	182	123,46
19.06.2006	120	82	186	131	74	182	405,76
19.06.2006	121	68	173	131	74	182	149,55
19.06.2006	122	62	165	131	74	182	31,30
19.06.2006	123	68	176	131	74	182	69,01
19.06.2006	124	128	214	131	74	182	518,77
19.06.2006	125	64	170	131	74	182	22,86
19.06.2006	129	115	216	131	74	182	730,31
20.06.2006	119	94	190	121	68	173	18,37

20.06.2006	119	94	190	122	62	165	150,26
20.06.2006	121	68	173	122	62	165	166,25
20.06.2006	119	94	190	123	68	176	81,78
20.06.2006	121	68	173	123	68	176	96,70
20.06.2006	122	62	165	123	68	176	70,28
20.06.2006	119	94	190	125	64	170	145,01
20.06.2006	121	68	173	125	64	170	160,99
20.06.2006	122	62	165	125	64	170	5,26
20.06.2006	123	68	176	125	64	170	65,05
20.06.2006	119	94	190	127	136	218	144,32
20.06.2006	121	68	173	127	136	218	160,10
20.06.2006	122	62	165	127	136	218	7,08
20.06.2006	123	68	176	127	136	218	63,81
20.06.2006	125	64	170	127	136	218	3,51
20.06.2006	119	94	190	131	74	182	147,81
20.06.2006	121	68	173	131	74	182	163,69
20.06.2006	122	62	165	131	74	182	3,16
20.06.2006	123	68	176	131	74	182	67,54
20.06.2006	125	64	170	131	74	182	3,22
20.06.2006	127	136	218	131	74	182	3,96
20.06.2006	119	94	190	120	82	186	534,13
20.06.2006	119	94	190	121	68	173	48,43
20.06.2006	120	82	186	121	68	173	581,56
20.06.2006	119	94	190	122	62	165	111,19
20.06.2006	120	82	186	122	62	165	427,92
20.06.2006	121	68	173	122	62	165	159,57
20.06.2006	119	94	190	123	68	176	63,00
20.06.2006	120	82	186	123	68	176	473,38
20.06.2006	121	68	173	123	68	176	111,41
20.06.2006	122	62	165	123	68	176	48,21
20.06.2006	119	94	190	124	128	214	562,72
20.06.2006	120	82	186	124	128	214	32,67
20.06.2006	121	68	173	124	128	214	609,85

20.06.2006	122	62	165	124	128	214	457,54
20.06.2006	123	68	176	124	128	214	502,48
20.06.2006	119	94	190	125	64	170	125,09
20.06.2006	120	82	186	125	64	170	413,07
20.06.2006	121	68	173	125	64	170	173,52
20.06.2006	122	62	165	125	64	170	14,86
20.06.2006	123	68	176	125	64	170	62,14
20.06.2006	124	128	214	125	64	170	442,67
20.06.2006	119	94	190	129	115	216	828,64
20.06.2006	120	82	186	129	115	216	342,21
20.06.2006	121	68	173	129	115	216	871,98
20.06.2006	122	62	165	129	115	216	734,88
20.06.2006	123	68	176	129	115	216	774,41
20.06.2006	124	128	214	129	115	216	310,00
20.06.2006	125	64	170	129	115	216	720,39
20.06.2006	119	94	190	131	74	182	137,62
20.06.2006	120	82	186	131	74	182	405,54
20.06.2006	121	68	173	131	74	182	185,86
20.06.2006	122	62	165	131	74	182	27,08
20.06.2006	123	68	176	131	74	182	74,86
20.06.2006	124	128	214	131	74	182	435,64
20.06.2006	125	64	170	131	74	182	17,40
20.06.2006	129	115	216	131	74	182	717,32
20.06.2006	119	94	190	120	82	186	589,24
20.06.2006	119	94	190	121	68	173	13,72
20.06.2006	120	82	186	121	68	173	600,96
20.06.2006	119	94	190	122	62	165	135,42
20.06.2006	120	82	186	122	62	165	454,57
20.06.2006	121	68	173	122	62	165	146,61
20.06.2006	119	94	190	123	68	176	58,75
20.06.2006	120	82	186	123	68	176	531,34
20.06.2006	121	68	173	123	68	176	69,70
20.06.2006	122	62	165	123	68	176	76,92

20.06.2006	119	94	190	124	128	214	631,87
20.06.2006	120	82	186	124	128	214	47,27
20.06.2006	121	68	173	124	128	214	643,82
20.06.2006	122	62	165	124	128	214	497,82
20.06.2006	123	68	176	124	128	214	574,35
20.06.2006	119	94	190	125	64	170	127,55
20.06.2006	120	82	186	125	64	170	461,74
20.06.2006	121	68	173	125	64	170	139,58
20.06.2006	122	62	165	125	64	170	17,46
20.06.2006	123	68	176	125	64	170	70,69
20.06.2006	124	128	214	125	64	170	504,32
20.06.2006	119	94	190	127	136	218	127,91
20.06.2006	120	82	186	127	136	218	461,33
20.06.2006	121	68	173	127	136	218	139,78
20.06.2006	122	62	165	127	136	218	14,41
20.06.2006	123	68	176	127	136	218	70,58
20.06.2006	124	128	214	127	136	218	504,04
20.06.2006	125	64	170	127	136	218	3,23
20.06.2006	119	94	190	129	115	216	922,13
20.06.2006	120	82	186	129	115	216	379,75
20.06.2006	121	68	173	129	115	216	935,23
20.06.2006	122	62	165	129	115	216	795,80
20.06.2006	123	68	176	129	115	216	868,56
20.06.2006	124	128	214	129	115	216	333,31
20.06.2006	125	64	170	129	115	216	798,21
20.06.2006	127	136	218	129	115	216	798,73
20.06.2006	119	94	190	131	74	182	150,13
20.06.2006	120	82	186	131	74	182	439,95
20.06.2006	121	68	173	131	74	182	161,31
20.06.2006	122	62	165	131	74	182	14,71
20.06.2006	123	68	176	131	74	182	91,62
20.06.2006	124	128	214	131	74	182	483,27
20.06.2006	125	64	170	131	74	182	27,79

20.06.2006	127	136	218	131	74	182	25,62
20.06.2006	129	115	216	131	74	182	782,18
20.06.2006	119	94	190	120	82	186	538,36
20.06.2006	119	94	190	121	68	173	26,97
20.06.2006	120	82	186	121	68	173	550,50
20.06.2006	119	94	190	122	62	165	108,88
20.06.2006	120	82	186	122	62	165	429,71
20.06.2006	121	68	173	122	62	165	124,12
20.06.2006	119	94	190	123	68	176	48,64
20.06.2006	120	82	186	123	68	176	490,84
20.06.2006	121	68	173	123	68	176	68,40
20.06.2006	122	62	165	123	68	176	61,18
20.06.2006	119	94	190	124	128	214	583,57
20.06.2006	120	82	186	124	128	214	52,66
20.06.2006	121	68	173	124	128	214	594,50
20.06.2006	122	62	165	124	128	214	475,43
20.06.2006	123	68	176	124	128	214	536,61
20.06.2006	119	94	190	125	64	170	105,89
20.06.2006	120	82	186	125	64	170	433,82
20.06.2006	121	68	173	125	64	170	122,90
20.06.2006	122	62	165	125	64	170	9,67
20.06.2006	123	68	176	125	64	170	57,42
20.06.2006	124	128	214	125	64	170	480,03
20.06.2006	119	94	190	127	136	218	107,20
20.06.2006	120	82	186	127	136	218	431,44
20.06.2006	121	68	173	127	136	218	122,61
20.06.2006	122	62	165	127	136	218	1,82
20.06.2006	123	68	176	127	136	218	59,43
20.06.2006	124	128	214	127	136	218	477,19
20.06.2006	125	64	170	127	136	218	8,54
20.06.2006	119	94	190	129	115	216	818,92
20.06.2006	120	82	186	129	115	216	344,84
20.06.2006	121	68	173	129	115	216	823,15

20.06.2006	122	62	165	129	115	216	718,06
20.06.2006	123	68	176	129	115	216	776,77
20.06.2006	124	128	214	129	115	216	293,74
20.06.2006	125	64	170	129	115	216	724,83
20.06.2006	127	136	218	129	115	216	719,88
20.06.2006	119	94	190	131	74	182	131,92
20.06.2006	120	82	186	131	74	182	407,95
20.06.2006	121	68	173	131	74	182	148,34
20.06.2006	122	62	165	131	74	182	24,70
20.06.2006	123	68	176	131	74	182	83,49
20.06.2006	124	128	214	131	74	182	454,38
20.06.2006	125	64	170	131	74	182	26,07
20.06.2006	127	136	218	131	74	182	26,00
20.06.2006	129	115	216	131	74	182	701,46
20.06.2006	119	94	190	120	82	186	559,71
20.06.2006	119	94	190	121	68	173	19,23
20.06.2006	120	82	186	121	68	173	564,64
20.06.2006	119	94	190	122	62	165	130,72
20.06.2006	120	82	186	122	62	165	429,66
20.06.2006	121	68	173	122	62	165	138,17
20.06.2006	119	94	190	123	68	176	56,67
20.06.2006	120	82	186	123	68	176	503,35
20.06.2006	121	68	173	123	68	176	62,39
20.06.2006	122	62	165	123	68	176	75,79
20.06.2006	119	94	190	124	128	214	571,55
20.06.2006	120	82	186	124	128	214	49,94
20.06.2006	121	68	173	124	128	214	574,87
20.06.2006	122	62	165	124	128	214	443,39
20.06.2006	123	68	176	124	128	214	514,88
20.06.2006	119	94	190	125	64	170	127,19
20.06.2006	120	82	186	125	64	170	432,58
20.06.2006	121	68	173	125	64	170	133,57
20.06.2006	122	62	165	125	64	170	8,89

20.06.2006	123	68	176	125	64	170	71,30
20.06.2006	124	128	214	125	64	170	445,37
20.06.2006	119	94	190	127	136	218	125,61
20.06.2006	120	82	186	127	136	218	434,10
20.06.2006	121	68	173	127	136	218	131,66
20.06.2006	122	62	165	127	136	218	11,78
20.06.2006	123	68	176	127	136	218	69,51
20.06.2006	124	128	214	127	136	218	446,61
20.06.2006	125	64	170	127	136	218	2,93
20.06.2006	119	94	190	129	115	216	844,55
20.06.2006	120	82	186	129	115	216	343,87
20.06.2006	121	68	173	129	115	216	844,02
20.06.2006	122	62	165	129	115	216	724,20
20.06.2006	123	68	176	129	115	216	788,88
20.06.2006	124	128	214	129	115	216	304,77
20.06.2006	125	64	170	129	115	216	724,29
20.06.2006	127	136	218	129	115	216	724,93
20.06.2006	119	94	190	131	74	182	128,48
20.06.2006	120	82	186	131	74	182	431,25
20.06.2006	121	68	173	131	74	182	134,62
20.06.2006	122	62	165	131	74	182	10,13
20.06.2006	123	68	176	131	74	182	72,43
20.06.2006	124	128	214	131	74	182	443,87
20.06.2006	125	64	170	131	74	182	2,10
20.06.2006	127	136	218	131	74	182	2,98
20.06.2006	129	115	216	131	74	182	722,51
20.06.2006	119	94	190	120	82	186	532,78
20.06.2006	119	94	190	121	68	173	9,88
20.06.2006	120	82	186	121	68	173	542,60
20.06.2006	119	94	190	122	62	165	113,66
20.06.2006	120	82	186	122	62	165	422,05
20.06.2006	121	68	173	122	62	165	123,50
20.06.2006	119	94	190	123	68	176	48,54

20.06.2006	120	82	186	123	68	176	491,05
20.06.2006	121	68	173	123	68	176	57,74
20.06.2006	122	62	165	123	68	176	69,05
20.06.2006	119	94	190	124	128	214	572,57
20.06.2006	120	82	186	124	128	214	64,46
20.06.2006	121	68	173	124	128	214	582,27
20.06.2006	122	62	165	124	128	214	464,96
20.06.2006	123	68	176	124	128	214	533,34
20.06.2006	119	94	190	127	136	218	102,19
20.06.2006	120	82	186	127	136	218	433,19
20.06.2006	121	68	173	127	136	218	112,03
20.06.2006	122	62	165	127	136	218	11,47
20.06.2006	123	68	176	127	136	218	57,86
20.06.2006	124	128	214	127	136	218	475,74
20.06.2006	119	94	190	129	115	216	826,97
20.06.2006	120	82	186	129	115	216	353,41
20.06.2006	121	68	173	129	115	216	836,08
20.06.2006	122	62	165	129	115	216	729,15
20.06.2006	123	68	176	129	115	216	794,23
20.06.2006	124	128	214	129	115	216	290,53
20.06.2006	127	136	218	129	115	216	738,90
20.06.2006	119	94	190	131	74	182	128,52
20.06.2006	120	82	186	131	74	182	410,45
20.06.2006	121	68	173	131	74	182	138,27
20.06.2006	122	62	165	131	74	182	17,12
20.06.2006	123	68	176	131	74	182	82,19
20.06.2006	124	128	214	131	74	182	454,79
20.06.2006	127	136	218	131	74	182	27,48
20.06.2006	129	115	216	131	74	182	721,99
20.06.2006	119	94	190	120	82	186	656,39
20.06.2006	119	94	190	121	68	173	221,66
20.06.2006	120	82	186	121	68	173	457,44
20.06.2006	119	94	190	122	62	165	246,24

20.06.2006	120	82	186	122	62	165	411,95
20.06.2006	121	68	173	122	62	165	69,62
20.06.2006	119	94	190	123	68	176	82,66
20.06.2006	120	82	186	123	68	176	635,09
20.06.2006	121	68	173	123	68	176	240,66
20.06.2006	122	62	165	123	68	176	241,51
20.06.2006	119	94	190	124	128	214	683,77
20.06.2006	120	82	186	124	128	214	35,19
20.06.2006	121	68	173	124	128	214	480,77
20.06.2006	122	62	165	124	128	214	438,29
20.06.2006	123	68	176	124	128	214	664,95
20.06.2006	119	94	190	125	64	170	225,88
20.06.2006	120	82	186	125	64	170	430,95
20.06.2006	121	68	173	125	64	170	72,54
20.06.2006	122	62	165	125	64	170	23,00
20.06.2006	123	68	176	125	64	170	218,70
20.06.2006	124	128	214	125	64	170	457,94
20.06.2006	119	94	190	127	136	218	236,88
20.06.2006	120	82	186	127	136	218	420,61
20.06.2006	121	68	173	127	136	218	69,74
20.06.2006	122	62	165	127	136	218	10,35
20.06.2006	123	68	176	127	136	218	231,21
20.06.2006	124	128	214	127	136	218	447,24
20.06.2006	125	64	170	127	136	218	12,65
20.06.2006	119	94	190	129	115	216	938,50
20.06.2006	120	82	186	129	115	216	324,30
20.06.2006	121	68	173	129	115	216	721,07
20.06.2006	122	62	165	129	115	216	694,22
20.06.2006	123	68	176	129	115	216	932,40
20.06.2006	124	128	214	129	115	216	289,20
20.06.2006	125	64	170	129	115	216	716,12
20.06.2006	127	136	218	129	115	216	704,16
20.06.2006	119	94	190	131	74	182	230,01

20.06.2006	120	82	186	131	74	182	426,55
20.06.2006	121	68	173	131	74	182	77,90
20.06.2006	122	62	165	131	74	182	22,42
20.06.2006	123	68	176	131	74	182	220,94
20.06.2006	124	128	214	131	74	182	453,76
20.06.2006	125	64	170	131	74	182	6,09
20.06.2006	127	136	218	131	74	182	12,87
20.06.2006	129	115	216	131	74	182	713,05
20.06.2006	119	94	190	120	82	186	547,70
20.06.2006	119	94	190	121	68	173	22,16
20.06.2006	120	82	186	121	68	173	569,64
20.06.2006	119	94	190	122	62	165	100,35
20.06.2006	120	82	186	122	62	165	453,76
20.06.2006	121	68	173	122	62	165	122,21
20.06.2006	119	94	190	123	68	176	55,81
20.06.2006	120	82	186	123	68	176	503,63
20.06.2006	121	68	173	123	68	176	76,12
20.06.2006	122	62	165	123	68	176	49,95
20.06.2006	119	94	190	124	128	214	557,27
20.06.2006	120	82	186	124	128	214	13,06
20.06.2006	121	68	173	124	128	214	579,16
20.06.2006	122	62	165	124	128	214	463,94
20.06.2006	123	68	176	124	128	214	513,77
20.06.2006	119	94	190	125	64	170	87,09
20.06.2006	120	82	186	125	64	170	468,53
20.06.2006	121	68	173	125	64	170	108,69
20.06.2006	122	62	165	125	64	170	14,78
20.06.2006	123	68	176	125	64	170	35,24
20.06.2006	124	128	214	125	64	170	478,73
20.06.2006	119	94	190	127	136	218	78,90
20.06.2006	120	82	186	127	136	218	477,72
20.06.2006	121	68	173	127	136	218	100,27
20.06.2006	122	62	165	127	136	218	23,96

20.06.2006	123	68	176	127	136	218	26,08
20.06.2006	124	128	214	127	136	218	487,91
20.06.2006	125	64	170	127	136	218	9,19
20.06.2006	119	94	190	129	115	216	854,77
20.06.2006	120	82	186	129	115	216	371,74
20.06.2006	121	68	173	129	115	216	874,77
20.06.2006	122	62	165	129	115	216	776,14
20.06.2006	123	68	176	129	115	216	822,91
20.06.2006	124	128	214	129	115	216	358,68
20.06.2006	125	64	170	129	115	216	790,33
20.06.2006	127	136	218	129	115	216	799,04
20.06.2006	119	94	190	131	74	182	116,09
20.06.2006	120	82	186	131	74	182	441,60
20.06.2006	121	68	173	131	74	182	137,76
20.06.2006	122	62	165	131	74	182	16,54
20.06.2006	123	68	176	131	74	182	63,69
20.06.2006	124	128	214	131	74	182	451,99
20.06.2006	125	64	170	131	74	182	29,09
20.06.2006	127	136	218	131	74	182	37,84
20.06.2006	129	115	216	131	74	182	767,98
21.06.2006	119	94	190	120	82	186	540,18
21.06.2006	119	94	190	121	68	173	11,62
21.06.2006	120	82	186	121	68	173	551,75
21.06.2006	119	94	190	122	62	165	123,13
21.06.2006	120	82	186	122	62	165	418,06
21.06.2006	121	68	173	122	62	165	134,75
21.06.2006	119	94	190	123	68	176	51,06
21.06.2006	120	82	186	123	68	176	489,12
21.06.2006	121	68	173	123	68	176	62,63
21.06.2006	122	62	165	123	68	176	72,70
21.06.2006	119	94	190	124	128	214	567,78
21.06.2006	120	82	186	124	128	214	28,37
21.06.2006	121	68	173	124	128	214	579,34

21.06.2006	122	62	165	124	128	214	445,87
21.06.2006	123	68	176	124	128	214	516,72
21.06.2006	119	94	190	125	64	170	123,23
21.06.2006	120	82	186	125	64	170	418,87
21.06.2006	121	68	173	125	64	170	134,83
21.06.2006	122	62	165	125	64	170	5,22
21.06.2006	123	68	176	125	64	170	73,31
21.06.2006	124	128	214	125	64	170	446,74
21.06.2006	119	94	190	127	136	218	125,44
21.06.2006	120	82	186	127	136	218	417,18
21.06.2006	121	68	173	127	136	218	137,02
21.06.2006	122	62	165	127	136	218	7,83
21.06.2006	123	68	176	127	136	218	75,76
21.06.2006	124	128	214	127	136	218	445,09
21.06.2006	125	64	170	127	136	218	3,13
21.06.2006	119	94	190	129	115	216	836,42
21.06.2006	120	82	186	129	115	216	347,43
21.06.2006	121	68	173	129	115	216	847,30
21.06.2006	122	62	165	129	115	216	723,73
21.06.2006	123	68	176	129	115	216	787,25
21.06.2006	124	128	214	129	115	216	322,27
21.06.2006	125	64	170	129	115	216	726,02
21.06.2006	127	136	218	129	115	216	725,17
21.06.2006	119	94	190	131	74	182	142,51
21.06.2006	120	82	186	131	74	182	402,84
21.06.2006	121	68	173	131	74	182	154,01
21.06.2006	122	62	165	131	74	182	24,98
21.06.2006	123	68	176	131	74	182	93,68
21.06.2006	124	128	214	131	74	182	430,88
21.06.2006	125	64	170	131	74	182	21,79
21.06.2006	127	136	218	131	74	182	18,80
21.06.2006	129	115	216	131	74	182	714,90
21.06.2006	119	94	190	120	82	186	526,62

21.06.2006	119	94	190	121	68	173	34,10
21.06.2006	120	82	186	121	68	173	555,53
21.06.2006	119	94	190	122	62	165	106,64
21.06.2006	120	82	186	122	62	165	420,46
21.06.2006	121	68	173	122	62	165	137,65
21.06.2006	119	94	190	123	68	176	90,89
21.06.2006	120	82	186	123	68	176	438,28
21.06.2006	121	68	173	123	68	176	123,39
21.06.2006	122	62	165	123	68	176	20,37
21.06.2006	119	94	190	124	128	214	564,28
21.06.2006	120	82	186	124	128	214	37,71
21.06.2006	121	68	173	124	128	214	593,11
21.06.2006	122	62	165	124	128	214	458,15
21.06.2006	123	68	176	124	128	214	475,99
21.06.2006	119	94	190	125	64	170	106,69
21.06.2006	120	82	186	125	64	170	419,99
21.06.2006	121	68	173	125	64	170	136,97
21.06.2006	122	62	165	125	64	170	5,90
21.06.2006	123	68	176	125	64	170	24,21
21.06.2006	124	128	214	125	64	170	457,67
21.06.2006	119	94	190	127	136	218	99,29
21.06.2006	120	82	186	127	136	218	427,62
21.06.2006	121	68	173	127	136	218	128,18
21.06.2006	122	62	165	127	136	218	17,48
21.06.2006	123	68	176	127	136	218	28,31
21.06.2006	124	128	214	127	136	218	465,25
21.06.2006	125	64	170	127	136	218	12,58
21.06.2006	119	94	190	129	115	216	836,62
21.06.2006	120	82	186	129	115	216	359,31
21.06.2006	121	68	173	129	115	216	859,49
21.06.2006	122	62	165	129	115	216	737,75
21.06.2006	123	68	176	129	115	216	757,67
21.06.2006	124	128	214	129	115	216	329,65

21.06.2006	125	64	170	129	115	216	735,52
21.06.2006	127	136	218	129	115	216	739,68
21.06.2006	119	94	190	131	74	182	122,62
21.06.2006	120	82	186	131	74	182	405,10
21.06.2006	121	68	173	131	74	182	153,94
21.06.2006	122	62	165	131	74	182	16,41
21.06.2006	123	68	176	131	74	182	33,40
21.06.2006	124	128	214	131	74	182	442,80
21.06.2006	125	64	170	131	74	182	18,85
21.06.2006	127	136	218	131	74	182	31,12
21.06.2006	129	115	216	131	74	182	724,82
21.06.2006	119	94	190	120	82	186	530,88
21.06.2006	119	94	190	121	68	173	15,61
21.06.2006	120	82	186	121	68	173	545,54
21.06.2006	119	94	190	122	62	165	95,19
21.06.2006	120	82	186	122	62	165	439,74
21.06.2006	121	68	173	122	62	165	108,26
21.06.2006	119	94	190	123	68	176	48,74
21.06.2006	120	82	186	123	68	176	489,08
21.06.2006	121	68	173	123	68	176	59,98
21.06.2006	122	62	165	123	68	176	49,46
21.06.2006	119	94	190	124	128	214	551,38
21.06.2006	120	82	186	124	128	214	34,69
21.06.2006	121	68	173	124	128	214	566,29
21.06.2006	122	62	165	124	128	214	461,90
21.06.2006	123	68	176	124	128	214	510,96
21.06.2006	119	94	190	125	64	170	95,83
21.06.2006	120	82	186	125	64	170	438,97
21.06.2006	121	68	173	125	64	170	108,94
21.06.2006	122	62	165	125	64	170	0,83
21.06.2006	123	68	176	125	64	170	50,21
21.06.2006	124	128	214	125	64	170	461,11
21.06.2006	119	94	190	129	115	216	799,06

21.06.2006	120	82	186	129	115	216	312,89
21.06.2006	121	68	173	129	115	216	814,58
21.06.2006	122	62	165	129	115	216	717,68
21.06.2006	123	68	176	129	115	216	764,61
21.06.2006	124	128	214	129	115	216	279,79
21.06.2006	125	64	170	129	115	216	716,86
21.06.2006	119	94	190	131	74	182	111,83
21.06.2006	120	82	186	131	74	182	426,58
21.06.2006	121	68	173	131	74	182	124,32
21.06.2006	122	62	165	131	74	182	17,89
21.06.2006	123	68	176	131	74	182	64,58
21.06.2006	124	128	214	131	74	182	449,48
21.06.2006	125	64	170	131	74	182	17,52
21.06.2006	129	115	216	131	74	182	707,97
26.06.2006	120	82	186	121	68	173	550,33
26.06.2006	120	82	186	122	62	165	411,20
26.06.2006	121	68	173	122	62	165	143,15
26.06.2006	120	82	186	123	68	176	474,66
26.06.2006	121	68	173	123	68	176	84,48
26.06.2006	122	62	165	123	68	176	63,47
26.06.2006	120	82	186	124	128	214	60,05
26.06.2006	121	68	173	124	128	214	603,48
26.06.2006	122	62	165	124	128	214	466,28
26.06.2006	123	68	176	124	128	214	529,71
26.06.2006	120	82	186	125	64	170	410,24
26.06.2006	121	68	173	125	64	170	143,66
26.06.2006	122	62	165	125	64	170	1,85
26.06.2006	123	68	176	125	64	170	64,48
26.06.2006	124	128	214	125	64	170	465,23
26.06.2006	120	82	186	127	136	218	410,21
26.06.2006	121	68	173	127	136	218	143,64
26.06.2006	122	62	165	127	136	218	2,07
26.06.2006	123	68	176	127	136	218	64,53

26.06.2006	124	128	214	127	136	218	465,18
26.06.2006	125	64	170	127	136	218	0,23
26.06.2006	120	82	186	129	115	216	343,53
26.06.2006	121	68	173	129	115	216	833,91
26.06.2006	122	62	165	129	115	216	710,97
26.06.2006	123	68	176	129	115	216	771,96
26.06.2006	124	128	214	129	115	216	285,72
26.06.2006	125	64	170	129	115	216	709,56
26.06.2006	127	136	218	129	115	216	709,46
26.06.2006	120	82	186	130	61	172	431,18
26.06.2006	121	68	173	130	61	172	127,32
26.06.2006	122	62	165	130	61	172	21,90
26.06.2006	123	68	176	130	61	172	44,29
26.06.2006	124	128	214	130	61	172	486,70
26.06.2006	125	64	170	130	61	172	23,46
26.06.2006	127	136	218	130	61	172	23,60
26.06.2006	129	115	216	130	61	172	732,71
26.06.2006	120	82	186	131	74	182	406,38
26.06.2006	121	68	173	131	74	182	150,13
26.06.2006	122	62	165	131	74	182	9,24
26.06.2006	123	68	176	131	74	182	68,67
26.06.2006	124	128	214	131	74	182	461,89
26.06.2006	125	64	170	131	74	182	10,21
26.06.2006	127	136	218	131	74	182	10,41
26.06.2006	129	115	216	131	74	182	708,77
26.06.2006	130	61	172	131	74	182	24,82
26.06.2006	120	82	186	121	68	173	559,68
26.06.2006	120	82	186	122	62	165	421,45
26.06.2006	121	68	173	122	62	165	144,04
26.06.2006	120	82	186	123	68	176	479,67
26.06.2006	121	68	173	123	68	176	89,31
26.06.2006	122	62	165	123	68	176	58,30
26.06.2006	120	82	186	124	128	214	50,62

26.06.2006	121	68	173	124	128	214	599,45
26.06.2006	122	62	165	124	128	214	463,81
26.06.2006	123	68	176	124	128	214	521,75
26.06.2006	120	82	186	125	64	170	422,23
26.06.2006	121	68	173	125	64	170	144,41
26.06.2006	122	62	165	125	64	170	3,36
26.06.2006	123	68	176	125	64	170	57,81
26.06.2006	124	128	214	125	64	170	464,80
26.06.2006	120	82	186	127	136	218	424,73
26.06.2006	121	68	173	127	136	218	141,30
26.06.2006	122	62	165	127	136	218	3,45
26.06.2006	123	68	176	127	136	218	55,10
26.06.2006	124	128	214	127	136	218	467,15
26.06.2006	125	64	170	127	136	218	3,34
26.06.2006	120	82	186	129	115	216	335,58
26.06.2006	121	68	173	129	115	216	833,05
26.06.2006	122	62	165	129	115	216	712,69
26.06.2006	123	68	176	129	115	216	767,51
26.06.2006	124	128	214	129	115	216	285,16
26.06.2006	125	64	170	129	115	216	714,41
26.06.2006	127	136	218	129	115	216	716,14
26.06.2006	120	82	186	131	74	182	411,66
26.06.2006	121	68	173	131	74	182	156,09
26.06.2006	122	62	165	131	74	182	12,82
26.06.2006	123	68	176	131	74	182	69,04
26.06.2006	124	128	214	131	74	182	454,56
26.06.2006	125	64	170	131	74	182	11,72
26.06.2006	127	136	218	131	74	182	14,96
26.06.2006	129	115	216	131	74	182	705,82
26.06.2006	120	82	186	121	68	173	557,06
26.06.2006	120	82	186	122	62	165	426,22
26.06.2006	121	68	173	122	62	165	131,85
26.06.2006	120	82	186	123	68	176	488,92

26.06.2006	121	68	173	123	68	176	68,82
26.06.2006	122	62	165	123	68	176	63,07
26.06.2006	120	82	186	124	128	214	57,36
26.06.2006	121	68	173	124	128	214	602,16
26.06.2006	122	62	165	124	128	214	472,72
26.06.2006	123	68	176	124	128	214	534,79
26.06.2006	120	82	186	125	64	170	429,84
26.06.2006	121	68	173	125	64	170	131,00
26.06.2006	122	62	165	125	64	170	13,54
26.06.2006	123	68	176	125	64	170	62,63
26.06.2006	124	128	214	125	64	170	477,29
26.06.2006	120	82	186	127	136	218	421,77
26.06.2006	121	68	173	127	136	218	136,68
26.06.2006	122	62	165	127	136	218	5,29
26.06.2006	123	68	176	127	136	218	67,87
26.06.2006	124	128	214	127	136	218	468,50
26.06.2006	125	64	170	127	136	218	12,94
26.06.2006	120	82	186	129	115	216	351,10
26.06.2006	121	68	173	129	115	216	845,86
26.06.2006	122	62	165	129	115	216	726,24
26.06.2006	123	68	176	129	115	216	783,65
26.06.2006	124	128	214	129	115	216	293,90
26.06.2006	125	64	170	129	115	216	733,81
26.06.2006	127	136	218	129	115	216	722,96
26.06.2006	120	82	186	131	74	182	409,21
26.06.2006	121	68	173	131	74	182	149,51
26.06.2006	122	62	165	131	74	182	17,87
26.06.2006	123	68	176	131	74	182	80,69
26.06.2006	124	128	214	131	74	182	456,18
26.06.2006	125	64	170	131	74	182	21,88
26.06.2006	127	136	218	131	74	182	12,83
26.06.2006	129	115	216	131	74	182	711,93
26.06.2006	120	82	186	121	68	173	568,40

26.06.2006	120	82	186	122	62	165	439,97
26.06.2006	121	68	173	122	62	165	140,62
26.06.2006	120	82	186	123	68	176	496,28
26.06.2006	121	68	173	123	68	176	79,12
26.06.2006	122	62	165	123	68	176	61,57
26.06.2006	120	82	186	124	128	214	49,76
26.06.2006	121	68	173	124	128	214	614,66
26.06.2006	122	62	165	124	128	214	488,01
26.06.2006	123	68	176	124	128	214	543,57
26.06.2006	120	82	186	125	64	170	447,20
26.06.2006	121	68	173	125	64	170	135,87
26.06.2006	122	62	165	125	64	170	7,92
26.06.2006	123	68	176	125	64	170	56,78
26.06.2006	124	128	214	125	64	170	495,32
26.06.2006	120	82	186	127	136	218	431,93
26.06.2006	121	68	173	127	136	218	143,64
26.06.2006	122	62	165	127	136	218	13,23
26.06.2006	123	68	176	127	136	218	65,75
26.06.2006	124	128	214	127	136	218	479,66
26.06.2006	125	64	170	127	136	218	20,58
26.06.2006	120	82	186	129	115	216	342,19
26.06.2006	121	68	173	129	115	216	848,24
26.06.2006	122	62	165	129	115	216	741,24
26.06.2006	123	68	176	129	115	216	788,23
26.06.2006	124	128	214	129	115	216	296,49
26.06.2006	125	64	170	129	115	216	749,08
26.06.2006	127	136	218	129	115	216	730,51
26.06.2006	120	82	186	131	74	182	424,45
26.06.2006	121	68	173	131	74	182	150,76
26.06.2006	122	62	165	131	74	182	18,52
26.06.2006	123	68	176	131	74	182	73,13
26.06.2006	124	128	214	131	74	182	472,19
26.06.2006	125	64	170	131	74	182	26,39

26.06.2006	127	136	218	131	74	182	7,49
26.06.2006	129	115	216	131	74	182	723,46
26.06.2006	120	82	186	121	68	173	566,83
26.06.2006	120	82	186	122	62	165	404,45
26.06.2006	121	68	173	122	62	165	162,59
26.06.2006	120	82	186	123	68	176	494,05
26.06.2006	121	68	173	123	68	176	122,52
26.06.2006	122	62	165	123	68	176	127,56
26.06.2006	120	82	186	124	128	214	63,14
26.06.2006	121	68	173	124	128	214	611,88
26.06.2006	122	62	165	124	128	214	450,82
26.06.2006	123	68	176	124	128	214	530,78
26.06.2006	120	82	186	125	64	170	408,52
26.06.2006	121	68	173	125	64	170	159,88
26.06.2006	122	62	165	125	64	170	12,67
26.06.2006	123	68	176	125	64	170	134,99
26.06.2006	124	128	214	125	64	170	456,04
26.06.2006	120	82	186	127	136	218	435,77
26.06.2006	121	68	173	127	136	218	159,31
26.06.2006	122	62	165	127	136	218	89,27
26.06.2006	123	68	176	127	136	218	58,31
26.06.2006	124	128	214	127	136	218	473,02
26.06.2006	125	64	170	127	136	218	100,17
26.06.2006	120	82	186	129	115	216	375,43
26.06.2006	121	68	173	129	115	216	881,41
26.06.2006	122	62	165	129	115	216	729,70
26.06.2006	123	68	176	129	115	216	779,86
26.06.2006	124	128	214	129	115	216	312,51
26.06.2006	125	64	170	129	115	216	737,52
26.06.2006	127	136	218	129	115	216	727,13
26.06.2006	120	82	186	131	74	182	402,35
26.06.2006	121	68	173	131	74	182	164,58
26.06.2006	122	62	165	131	74	182	11,77

26.06.2006	123	68	176	131	74	182	120,27
26.06.2006	124	128	214	131	74	182	447,55
26.06.2006	125	64	170	131	74	182	24,40
26.06.2006	127	136	218	131	74	182	78,84
26.06.2006	129	115	216	131	74	182	723,71
26.06.2006	120	82	186	121	68	173	549,72
26.06.2006	120	82	186	122	62	165	449,52
26.06.2006	121	68	173	122	62	165	114,94
26.06.2006	120	82	186	123	68	176	481,17
26.06.2006	121	68	173	123	68	176	72,33
26.06.2006	122	62	165	123	68	176	44,88
26.06.2006	120	82	186	124	128	214	41,29
26.06.2006	121	68	173	124	128	214	579,55
26.06.2006	122	62	165	124	128	214	482,43
26.06.2006	123	68	176	124	128	214	512,31
26.06.2006	120	82	186	125	64	170	445,71
26.06.2006	121	68	173	125	64	170	105,47
26.06.2006	122	62	165	125	64	170	35,11
26.06.2006	123	68	176	125	64	170	35,74
26.06.2006	124	128	214	125	64	170	476,65
26.06.2006	120	82	186	127	136	218	436,49
26.06.2006	121	68	173	127	136	218	119,53
26.06.2006	122	62	165	127	136	218	20,30
26.06.2006	123	68	176	127	136	218	47,22
26.06.2006	124	128	214	127	136	218	468,55
26.06.2006	125	64	170	127	136	218	21,03
26.06.2006	120	82	186	129	115	216	318,49
26.06.2006	121	68	173	129	115	216	815,47
26.06.2006	122	62	165	129	115	216	733,08
26.06.2006	123	68	176	129	115	216	755,43
26.06.2006	124	128	214	129	115	216	277,29
26.06.2006	125	64	170	129	115	216	720,10
26.06.2006	127	136	218	129	115	216	716,45

26.06.2006	120	82	186	131	74	182	427,32
26.06.2006	121	68	173	131	74	182	125,10
26.06.2006	122	62	165	131	74	182	34,38
26.06.2006	123	68	176	131	74	182	53,99
26.06.2006	124	128	214	131	74	182	458,77
26.06.2006	125	64	170	131	74	182	20,00
26.06.2006	127	136	218	131	74	182	14,09
26.06.2006	129	115	216	131	74	182	704,69
27.06.2006	120	82	186	121	68	173	532,61
27.06.2006	120	82	186	122	62	165	418,33
27.06.2006	121	68	173	122	62	165	125,10
27.06.2006	120	82	186	123	68	176	472,66
27.06.2006	121	68	173	123	68	176	70,89
27.06.2006	122	62	165	123	68	176	56,21
27.06.2006	120	82	186	124	128	214	61,87
27.06.2006	121	68	173	124	128	214	592,05
27.06.2006	122	62	165	124	128	214	479,24
27.06.2006	123	68	176	124	128	214	533,19
27.06.2006	120	82	186	125	64	170	413,81
27.06.2006	121	68	173	125	64	170	125,43
27.06.2006	122	62	165	125	64	170	10,25
27.06.2006	123	68	176	125	64	170	59,02
27.06.2006	124	128	214	125	64	170	474,49
27.06.2006	120	82	186	127	136	218	398,75
27.06.2006	121	68	173	127	136	218	136,49
27.06.2006	122	62	165	127	136	218	28,32
27.06.2006	123	68	176	127	136	218	74,31
27.06.2006	124	128	214	127	136	218	459,09
27.06.2006	125	64	170	127	136	218	18,87
27.06.2006	120	82	186	129	115	216	330,45
27.06.2006	121	68	173	129	115	216	824,73
27.06.2006	122	62	165	129	115	216	725,09
27.06.2006	123	68	176	129	115	216	774,89

27.06.2006	124	128	214	129	115	216	272,38
27.06.2006	125	64	170	129	115	216	718,60
27.06.2006	127	136	218	129	115	216	701,24
27.06.2006	120	82	186	131	74	182	394,83
27.06.2006	121	68	173	131	74	182	140,21
27.06.2006	122	62	165	131	74	182	31,37
27.06.2006	123	68	176	131	74	182	78,25
27.06.2006	124	128	214	131	74	182	455,17
27.06.2006	125	64	170	131	74	182	22,32
27.06.2006	127	136	218	131	74	182	3,94
27.06.2006	129	115	216	131	74	182	697,34
27.06.2006	120	82	186	121	68	173	548,58
27.06.2006	120	82	186	122	62	165	419,87
27.06.2006	121	68	173	122	62	165	136,72
27.06.2006	120	82	186	123	68	176	472,48
27.06.2006	121	68	173	123	68	176	76,66
27.06.2006	122	62	165	123	68	176	63,04
27.06.2006	120	82	186	124	128	214	72,65
27.06.2006	121	68	173	124	128	214	618,58
27.06.2006	122	62	165	124	128	214	487,99
27.06.2006	123	68	176	124	128	214	542,19
27.06.2006	120	82	186	125	64	170	415,10
27.06.2006	121	68	173	125	64	170	136,93
27.06.2006	122	62	165	125	64	170	14,22
27.06.2006	123	68	176	125	64	170	60,87
27.06.2006	124	128	214	125	64	170	483,94
27.06.2006	120	82	186	127	136	218	419,00
27.06.2006	121	68	173	127	136	218	134,26
27.06.2006	122	62	165	127	136	218	9,62
27.06.2006	123	68	176	127	136	218	58,84
27.06.2006	124	128	214	127	136	218	487,63
27.06.2006	125	64	170	127	136	218	5,48
27.06.2006	120	82	186	129	115	216	330,61

27.06.2006	121	68	173	129	115	216	829,28
27.06.2006	122	62	165	129	115	216	717,04
27.06.2006	123	68	176	129	115	216	758,37
27.06.2006	124	128	214	129	115	216	296,88
27.06.2006	125	64	170	129	115	216	708,84
27.06.2006	127	136	218	129	115	216	713,65
27.06.2006	120	82	186	131	74	182	418,88
27.06.2006	121	68	173	131	74	182	134,47
27.06.2006	122	62	165	131	74	182	9,29
27.06.2006	123	68	176	131	74	182	59,10
27.06.2006	124	128	214	131	74	182	487,50
27.06.2006	125	64	170	131	74	182	5,65
27.06.2006	127	136	218	131	74	182	0,36
27.06.2006	129	115	216	131	74	182	713,63
27.06.2006	120	82	186	121	68	173	561,24
27.06.2006	120	82	186	122	62	165	422,91
27.06.2006	121	68	173	122	62	165	145,50
27.06.2006	120	82	186	123	68	176	513,96
27.06.2006	121	68	173	123	68	176	50,02
27.06.2006	122	62	165	123	68	176	95,56
27.06.2006	120	82	186	124	128	214	49,24
27.06.2006	121	68	173	124	128	214	609,93
27.06.2006	122	62	165	124	128	214	470,79
27.06.2006	123	68	176	124	128	214	562,41
27.06.2006	120	82	186	125	64	170	423,43
27.06.2006	121	68	173	125	64	170	143,12
27.06.2006	122	62	165	125	64	170	5,67
27.06.2006	123	68	176	125	64	170	93,36
27.06.2006	124	128	214	125	64	170	471,45
27.06.2006	120	82	186	127	136	218	424,08
27.06.2006	121	68	173	127	136	218	143,83
27.06.2006	122	62	165	127	136	218	2,00
27.06.2006	123	68	176	127	136	218	93,92

27.06.2006	124	128	214	127	136	218	472,00
27.06.2006	125	64	170	127	136	218	4,08
27.06.2006	120	82	186	129	115	216	335,53
27.06.2006	121	68	173	129	115	216	846,11
27.06.2006	122	62	165	129	115	216	724,34
27.06.2006	123	68	176	129	115	216	804,61
27.06.2006	124	128	214	129	115	216	305,48
27.06.2006	125	64	170	129	115	216	723,33
27.06.2006	127	136	218	129	115	216	725,03
27.06.2006	120	82	186	131	74	182	425,13
27.06.2006	121	68	173	131	74	182	149,17
27.06.2006	122	62	165	131	74	182	13,93
27.06.2006	123	68	176	131	74	182	99,24
27.06.2006	124	128	214	131	74	182	472,64
27.06.2006	125	64	170	131	74	182	19,50
27.06.2006	127	136	218	131	74	182	15,44
27.06.2006	129	115	216	131	74	182	730,01
27.06.2006	120	82	186	121	68	173	531,53
27.06.2006	120	82	186	122	62	165	399,93
27.06.2006	121	68	173	122	62	165	136,52
27.06.2006	120	82	186	123	68	176	456,75
27.06.2006	121	68	173	123	68	176	80,84
27.06.2006	122	62	165	123	68	176	57,25
27.06.2006	120	82	186	124	128	214	60,48
27.06.2006	121	68	173	124	128	214	585,20
27.06.2006	122	62	165	124	128	214	455,69
27.06.2006	123	68	176	124	128	214	512,06
27.06.2006	120	82	186	125	64	170	395,42
27.06.2006	121	68	173	125	64	170	141,10
27.06.2006	122	62	165	125	64	170	4,61
27.06.2006	123	68	176	125	64	170	61,85
27.06.2006	124	128	214	125	64	170	451,23
27.06.2006	120	82	186	127	136	218	405,89

27.06.2006	121	68	173	127	136	218	132,29
27.06.2006	122	62	165	127	136	218	7,28
27.06.2006	123	68	176	127	136	218	52,16
27.06.2006	124	128	214	127	136	218	461,86
27.06.2006	125	64	170	127	136	218	10,96
27.06.2006	120	82	186	129	115	216	355,33
27.06.2006	121	68	173	129	115	216	833,55
27.06.2006	122	62	165	129	115	216	717,88
27.06.2006	123	68	176	129	115	216	770,32
27.06.2006	124	128	214	129	115	216	296,40
27.06.2006	125	64	170	129	115	216	713,83
27.06.2006	127	136	218	129	115	216	724,79
27.06.2006	120	82	186	131	74	182	390,70
27.06.2006	121	68	173	131	74	182	146,75
27.06.2006	122	62	165	131	74	182	10,33
27.06.2006	123	68	176	131	74	182	67,12
27.06.2006	124	128	214	131	74	182	446,72
27.06.2006	125	64	170	131	74	182	6,00
27.06.2006	127	136	218	131	74	182	15,20
27.06.2006	129	115	216	131	74	182	710,40
27.06.2006	120	82	186	121	68	173	532,62
27.06.2006	120	82	186	122	62	165	412,05
27.06.2006	121	68	173	122	62	165	128,91
27.06.2006	120	82	186	123	68	176	474,55
27.06.2006	121	68	173	123	68	176	69,61
27.06.2006	122	62	165	123	68	176	63,19
27.06.2006	120	82	186	124	128	214	38,31
27.06.2006	121	68	173	124	128	214	558,37
27.06.2006	122	62	165	124	128	214	440,54
27.06.2006	123	68	176	124	128	214	502,42
27.06.2006	120	82	186	125	64	170	423,34
27.06.2006	121	68	173	125	64	170	117,15
27.06.2006	122	62	165	125	64	170	11,83

27.06.2006	123	68	176	125	64	170	51,52
27.06.2006	124	128	214	125	64	170	451,60
27.06.2006	120	82	186	127	136	218	415,55
27.06.2006	121	68	173	127	136	218	122,74
27.06.2006	122	62	165	127	136	218	8,60
27.06.2006	123	68	176	127	136	218	59,01
27.06.2006	124	128	214	127	136	218	443,57
27.06.2006	125	64	170	127	136	218	8,95
27.06.2006	120	82	186	129	115	216	340,04
27.06.2006	121	68	173	129	115	216	812,22
27.06.2006	122	62	165	129	115	216	710,52
27.06.2006	123	68	176	129	115	216	767,63
27.06.2006	124	128	214	129	115	216	301,91
27.06.2006	125	64	170	129	115	216	720,25
27.06.2006	127	136	218	129	115	216	711,49
27.06.2006	120	82	186	131	74	182	411,43
27.06.2006	121	68	173	131	74	182	129,84
27.06.2006	122	62	165	131	74	182	1,10
27.06.2006	123	68	176	131	74	182	63,95
27.06.2006	124	128	214	131	74	182	439,98
27.06.2006	125	64	170	131	74	182	12,71
27.06.2006	127	136	218	131	74	182	9,69
27.06.2006	129	115	216	131	74	182	710,20
27.06.2006	120	82	186	121	68	173	554,78
27.06.2006	120	82	186	122	62	165	433,69
27.06.2006	121	68	173	122	62	165	125,51
27.06.2006	120	82	186	123	68	176	493,58
27.06.2006	121	68	173	123	68	176	66,54
27.06.2006	122	62	165	123	68	176	60,43
27.06.2006	120	82	186	124	128	214	59,48
27.06.2006	121	68	173	124	128	214	599,63
27.06.2006	122	62	165	124	128	214	481,26
27.06.2006	123	68	176	124	128	214	540,38

27.06.2006	120	82	186	125	64	170	439,72
27.06.2006	121	68	173	125	64	170	117,56
27.06.2006	122	62	165	125	64	170	10,10
27.06.2006	123	68	176	125	64	170	53,86
27.06.2006	124	128	214	125	64	170	486,63
27.06.2006	120	82	186	127	136	218	436,39
27.06.2006	121	68	173	127	136	218	122,14
27.06.2006	122	62	165	127	136	218	3,83
27.06.2006	123	68	176	127	136	218	57,42
27.06.2006	124	128	214	127	136	218	483,73
27.06.2006	125	64	170	127	136	218	6,33
27.06.2006	120	82	186	129	115	216	377,08
27.06.2006	121	68	173	129	115	216	858,21
27.06.2006	122	62	165	129	115	216	754,85
27.06.2006	123	68	176	129	115	216	808,68
27.06.2006	124	128	214	129	115	216	317,75
27.06.2006	125	64	170	129	115	216	757,74
27.06.2006	127	136	218	129	115	216	756,46
27.06.2006	120	82	186	131	74	182	431,20
27.06.2006	121	68	173	131	74	182	127,30
27.06.2006	122	62	165	131	74	182	3,25
27.06.2006	123	68	176	131	74	182	62,64
27.06.2006	124	128	214	131	74	182	478,61
27.06.2006	125	64	170	131	74	182	10,40
27.06.2006	127	136	218	131	74	182	5,22
27.06.2006	129	115	216	131	74	182	751,80
27.06.2006	120	82	186	121	68	173	527,20
27.06.2006	120	82	186	122	62	165	391,28
27.06.2006	121	68	173	122	62	165	135,98
27.06.2006	120	82	186	123	68	176	431,97
27.06.2006	121	68	173	123	68	176	95,90
27.06.2006	122	62	165	123	68	176	41,16
27.06.2006	120	82	186	124	128	214	51,13

27.06.2006	121	68	173	124	128	214	552,07
27.06.2006	122	62	165	124	128	214	417,14
27.06.2006	123	68	176	124	128	214	458,25
27.06.2006	120	82	186	125	64	170	392,17
27.06.2006	121	68	173	125	64	170	136,31
27.06.2006	122	62	165	125	64	170	12,73
27.06.2006	123	68	176	125	64	170	40,43
27.06.2006	124	128	214	125	64	170	419,40
27.06.2006	120	82	186	127	136	218	393,22
27.06.2006	121	68	173	127	136	218	134,00
27.06.2006	122	62	165	127	136	218	2,29
27.06.2006	123	68	176	127	136	218	39,46
27.06.2006	124	128	214	127	136	218	418,94
27.06.2006	125	64	170	127	136	218	13,99
27.06.2006	120	82	186	129	115	216	311,41
27.06.2006	121	68	173	129	115	216	808,41
27.06.2006	122	62	165	129	115	216	675,07
27.06.2006	123	68	176	129	115	216	713,03
27.06.2006	124	128	214	129	115	216	320,16
27.06.2006	125	64	170	129	115	216	672,71
27.06.2006	127	136	218	129	115	216	677,26
27.06.2006	120	82	186	131	74	182	393,20
27.06.2006	121	68	173	131	74	182	134,02
27.06.2006	122	62	165	131	74	182	5,79
27.06.2006	123	68	176	131	74	182	40,57
27.06.2006	124	128	214	131	74	182	418,45
27.06.2006	125	64	170	131	74	182	18,23
27.06.2006	127	136	218	131	74	182	4,25
27.06.2006	129	115	216	131	74	182	678,27
27.06.2006	120	82	186	121	68	173	554,80
27.06.2006	120	82	186	122	62	165	406,77
27.06.2006	121	68	173	122	62	165	150,05
27.06.2006	120	82	186	123	68	176	455,13

27.06.2006	121	68	173	123	68	176	106,87
27.06.2006	122	62	165	123	68	176	49,54
27.06.2006	120	82	186	124	128	214	42,15
27.06.2006	121	68	173	124	128	214	593,51
27.06.2006	122	62	165	124	128	214	444,71
27.06.2006	123	68	176	124	128	214	492,57
27.06.2006	120	82	186	125	64	170	404,68
27.06.2006	121	68	173	125	64	170	154,44
27.06.2006	122	62	165	125	64	170	10,28
27.06.2006	123	68	176	125	64	170	50,46
27.06.2006	124	128	214	125	64	170	442,17
27.06.2006	120	82	186	127	136	218	402,24
27.06.2006	121	68	173	127	136	218	158,11
27.06.2006	122	62	165	127	136	218	15,31
27.06.2006	123	68	176	127	136	218	53,11
27.06.2006	124	128	214	127	136	218	439,52
27.06.2006	125	64	170	127	136	218	5,18
27.06.2006	120	82	186	129	115	216	317,53
27.06.2006	121	68	173	129	115	216	816,94
27.06.2006	122	62	165	129	115	216	668,35
27.06.2006	123	68	176	129	115	216	710,34
27.06.2006	124	128	214	129	115	216	277,90
27.06.2006	125	64	170	129	115	216	662,90
27.06.2006	127	136	218	129	115	216	659,00
27.06.2006	120	82	186	131	74	182	406,77
27.06.2006	121	68	173	131	74	182	150,05
27.06.2006	122	62	165	131	74	182	0,10
27.06.2006	123	68	176	131	74	182	49,54
27.06.2006	124	128	214	131	74	182	444,71
27.06.2006	125	64	170	131	74	182	10,28
27.06.2006	127	136	218	131	74	182	15,31
27.06.2006	129	115	216	131	74	182	668,35
28.06.2006	127	136	218	120	82	186	482,12

28.06.2006	120	82	186	121	68	173	557,17
28.06.2006	127	136	218	121	68	173	80,37
28.06.2006	120	82	186	122	62	165	421,72
28.06.2006	121	68	173	122	62	165	140,21
28.06.2006	127	136	218	122	62	165	61,03
28.06.2006	120	82	186	123	68	176	487,72
28.06.2006	121	68	173	123	68	176	74,83
28.06.2006	122	62	165	123	68	176	66,70
28.06.2006	127	136	218	123	68	176	5,68
28.06.2006	120	82	186	124	128	214	31,16
28.06.2006	121	68	173	124	128	214	587,07
28.06.2006	122	62	165	124	128	214	452,19
28.06.2006	123	68	176	124	128	214	518,04
28.06.2006	127	136	218	124	128	214	512,46
28.06.2006	120	82	186	125	64	170	426,27
28.06.2006	121	68	173	125	64	170	135,85
28.06.2006	122	62	165	125	64	170	4,55
28.06.2006	123	68	176	125	64	170	62,20
28.06.2006	124	128	214	125	64	170	456,73
28.06.2006	127	136	218	125	64	170	56,52
28.06.2006	120	82	186	129	115	216	314,32
28.06.2006	121	68	173	129	115	216	826,81
28.06.2006	122	62	165	129	115	216	704,30
28.06.2006	123	68	176	129	115	216	765,95
28.06.2006	124	128	214	129	115	216	285,58
28.06.2006	125	64	170	129	115	216	708,69
28.06.2006	127	136	218	129	115	216	760,75
28.06.2006	120	82	186	131	74	182	420,04
28.06.2006	121	68	173	131	74	182	142,38
28.06.2006	122	62	165	131	74	182	2,51
28.06.2006	123	68	176	131	74	182	68,68
28.06.2006	124	128	214	131	74	182	450,53
28.06.2006	125	64	170	131	74	182	6,53

28.06.2006	127	136	218	131	74	182	62,99
28.06.2006	129	115	216	131	74	182	703,14
28.06.2006	120	82	186	121	68	173	545,75
28.06.2006	120	82	186	122	62	165	424,67
28.06.2006	121	68	173	122	62	165	135,85
28.06.2006	120	82	186	123	68	176	506,14
28.06.2006	121	68	173	123	68	176	47,60
28.06.2006	122	62	165	123	68	176	115,97
28.06.2006	120	82	186	124	128	214	59,00
28.06.2006	121	68	173	124	128	214	588,92
28.06.2006	122	62	165	124	128	214	472,73
28.06.2006	123	68	176	124	128	214	547,43
28.06.2006	120	82	186	125	64	170	429,31
28.06.2006	121	68	173	125	64	170	134,25
28.06.2006	122	62	165	125	64	170	6,33
28.06.2006	123	68	176	125	64	170	116,57
28.06.2006	124	128	214	125	64	170	477,67
28.06.2006	120	82	186	127	136	218	426,83
28.06.2006	121	68	173	127	136	218	144,16
28.06.2006	122	62	165	127	136	218	17,59
28.06.2006	123	68	176	127	136	218	128,97
28.06.2006	124	128	214	127	136	218	476,18
28.06.2006	125	64	170	127	136	218	13,46
28.06.2006	120	82	186	129	115	216	332,97
28.06.2006	121	68	173	129	115	216	816,81
28.06.2006	122	62	165	129	115	216	718,39
28.06.2006	123	68	176	129	115	216	771,07
28.06.2006	124	128	214	129	115	216	273,97
28.06.2006	125	64	170	129	115	216	724,04
28.06.2006	127	136	218	129	115	216	725,22
28.06.2006	120	82	186	131	74	182	414,89
28.06.2006	121	68	173	131	74	182	135,72
28.06.2006	122	62	165	131	74	182	24,07

28.06.2006	123	68	176	131	74	182	107,92
28.06.2006	124	128	214	131	74	182	461,22
28.06.2006	125	64	170	131	74	182	30,05
28.06.2006	127	136	218	131	74	182	41,07
28.06.2006	129	115	216	131	74	182	702,58
28.06.2006	120	82	186	121	68	173	558,64
28.06.2006	120	82	186	122	62	165	418,96
28.06.2006	121	68	173	122	62	165	140,50
28.06.2006	120	82	186	123	68	176	482,74
28.06.2006	121	68	173	123	68	176	77,41
28.06.2006	122	62	165	123	68	176	63,78
28.06.2006	120	82	186	124	128	214	34,69
28.06.2006	121	68	173	124	128	214	584,55
28.06.2006	122	62	165	124	128	214	445,70
28.06.2006	123	68	176	124	128	214	509,36
28.06.2006	120	82	186	125	64	170	421,69
28.06.2006	121	68	173	125	64	170	137,30
28.06.2006	122	62	165	125	64	170	5,39
28.06.2006	123	68	176	125	64	170	61,19
28.06.2006	124	128	214	125	64	170	448,19
28.06.2006	120	82	186	127	136	218	428,46
28.06.2006	121	68	173	127	136	218	131,34
28.06.2006	122	62	165	127	136	218	9,67
28.06.2006	123	68	176	127	136	218	54,36
28.06.2006	124	128	214	127	136	218	455,28
28.06.2006	125	64	170	127	136	218	9,40
28.06.2006	120	82	186	129	115	216	331,14
28.06.2006	121	68	173	129	115	216	825,25
28.06.2006	122	62	165	129	115	216	696,96
28.06.2006	123	68	176	129	115	216	757,20
28.06.2006	124	128	214	129	115	216	296,49
28.06.2006	125	64	170	129	115	216	698,00
28.06.2006	127	136	218	129	115	216	706,53

28.06.2006	120	82	186	131	74	182	416,62
28.06.2006	121	68	173	131	74	182	144,26
28.06.2006	122	62	165	131	74	182	9,18
28.06.2006	123	68	176	131	74	182	66,93
28.06.2006	124	128	214	131	74	182	443,81
28.06.2006	125	64	170	131	74	182	14,45
28.06.2006	127	136	218	131	74	182	13,84
28.06.2006	129	115	216	131	74	182	697,63
