# Supplementary material for: Diel movement of brown trout, Salmo trutta, is reduced in dense populations with high site fidelity
Source: Ecol Evol. 2018 Apr 6;8(9):4495–507. doi: 10.1002/ece3.3981 (PMC5938464; doi:10.1002/ece3.3981)
Supplement: Supplementary file 4 [file ECE3-8-4495-s004.pdf]

site	site_fidelity_ratio	sand_proportion	gravel_proportion	pebble_proportion
Modravsky_I	1	0	5	57
Hamersky_II	1	1	30	38
Vydra	0	0	22	53
Filipohutsky	1	1	45	48
Svelsky	0	2	22	25
Javori	0	0	23	64
Tmavy	0	0	40	57
Hammersky_I	0	3	57	39
Rokytka	0	4	83	11
Roklansky_II	0	6	29	52
Roklansky_III	1	4	29	55
Kremelna_II	1	7	45	39
Kremelna_III	1	1	48	39
Luzensky_I	0	0	75	22
Luzensky_II	1	1	51	43
Breznicky	1	2	30	59
Modravsky_II	1	0	18	40
Kremelna_I	1	6	64	28

boulder_proportion	slope_log
38	0.41581
31	0.77851
25	0.77851
6	0.55145
51	1.54407
13	0.25042
3	0.57749
1	0.36922
2	-0.16749
13	0.25042
12	0.25042
9	0.09342
12	0.02531
3	0.45637
5	0.45637
9	0.45637
42	0.41581
2	-0.04576
